# Repurposing hyperpolarization‐activated cyclic nucleotide‐gated channels as a novel therapy for breast cancer

**DOI:** 10.1002/ctm2.578

**Published:** 2021-11-04

**Authors:** Ka‐Chun Mok, Ho Tsoi, Ellen PS Man, Man‐Hong Leung, Ka Man Chau, Lai‐San Wong, Wing‐Lok Chan, Sum‐Yin Chan, Mai‐Yee Luk, Jessie Y.W. Chan, Jackie K.M. Leung, Yolanda H.Y. Chan, Sellma Batalha, Virginia Lau, David C.W. Siu, Terence K.W. Lee, Chun Gong, Ui‐Soon Khoo

**Affiliations:** ^1^ Department of Pathology Li Ka Shing Faculty of Medicine The University of Hong Kong Hong Kong Hong Kong; ^2^ Department of Clinical Oncology Queen Mary Hospital Hong Kong Hong Kong; ^3^ Department of Clinical Oncology Li Ka Shing Faculty of Medicine The University of Hong Kong Hong Kong Hong Kong; ^4^ Department of Surgery Pamela Youde Nethersole Eastern Hospital Hong Kong Hong Kong; ^5^ Department of Surgery Kwong Wah Hospital Hong Kong Hong Kong; ^6^ Department of Medicine The University of Hong Kong Hong Kong Hong Kong; ^7^ Department of Applied Biology & Chemical Technology The Hong Kong Polytechnic University Hong Kong Hong Kong

**Keywords:** ER‐stress, HCN, Ivabradine, targeted therapy, triple‐negative breast cancer

## Abstract

Hyperpolarization‐activated cyclic nucleotide‐gated (HCN) channels are members of the voltage‐gated cation channel family known to be expressed in the heart and central nervous system. Ivabradine, a small molecule HCN channel‐blocker, is FDA‐approved for clinical use as a heart rate‐reducing agent. We found that HCN2 and HCN3 are overexpressed in breast cancer cells compared with normal breast epithelia, and the high expression of HCN2 and HCN3 is associated with poorer survival in breast cancer patients. Inhibition of HCN by Ivabradine or by RNAi, aborted breast cancer cell proliferation in vitro and suppressed tumour growth in patient‐derived tumour xenograft models established from triple‐negative breast cancer (TNBC) tissues, with no evident side‐effects on the mice. Transcriptome‐wide analysis showed enrichment for cholesterol metabolism and biosynthesis as well as lipid metabolism pathways associated with ER‐stress following Ivabradine treatment. Mechanistic studies confirmed that HCN inhibition leads to ER‐stress, in part due to disturbed Ca^2+^ homeostasis, which subsequently triggered the apoptosis cascade. More importantly, we investigated the synergistic effect of Ivabradine and paclitaxel on TNBC and confirmed that both drugs acted synergistically in vitro through ER‐stress to amplify signals for caspase activation. Combination therapy could suppress tumour growth of xenografts at much lower doses for both drugs. In summary, our study identified a new molecular target with potential for being developed into targeted therapy, providing scientific grounds for initiating clinical trials for a new treatment regimen of combining HCN inhibition with chemotherapy.

## Graphical abstract

Overexpression of HCN provides advantages for the survival and proliferation of cancer cells. The addition of Ivabradine blocks HCN channels and affects intracellular homeostasis of Ca^2+^. Subsequently, this induces ER‐stress and triggers apoptosis. Also, it makes the cancer cells become more sensitive to other chemotherapeutic agents.

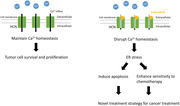

**Abbreviations**
4‐PBA: 4‐Phenylbutyric acid
CI: Combination Index
EC_50_
: half‐maximal effective concentration
ER: endoplasmic reticulum
FDA: U.S. Food and Drug Administration
GO: gene ontology
HCN: hyperpolarization‐activated cyclic nucleotide–gated channels
IP3: inositol 1 4 5‐trisphosphate
PDTX: patient‐derived tumour xenograft
TNBC: triple‐negative breast cancer

## 1 INTRODUCTION

Hyperpolarization‐activated cyclic nucleotide‐gated channels (HCN channels; HCN1‐4) are members of the voltage‐gated cation channel family. They are mainly located on the cellular membrane, with the exception that HCN3 tends to accumulate in intracellular compartments.^1^ HCN channels are highly selective cation channels^1^ and are one of the few that can be activated by hyperpolarization.^2^ Attention had been focused on HCNs mainly expressed in the heart and nervous system.^1^, ^3^ HCN4 is predominantly expressed in the cardiac conduction system and controls heart rate.^4^ Ivabradine is a U.S. Food and Drug Administration (FDA)‐approved therapeutic HCN channel‐blocker used clinically as a heart rate‐reducing agent.^5^ The drug selectively blocks the HCN channels,^6^, ^7^ reducing the spontaneous diastolic depolarization in the sinoatrial node, thereby regulating heart rate.^8^ HCN activation in hyperpolarized cells allows entry of cations, such as Na^+^ and Ca^2+^, into the cells and the efflux of K^+^ leading to depolarization of the membrane potential.^9^, ^10^ A recent report found HCN2 expressed in non‐small cell lung carcinoma cell lines which are hyperpolarized on exposure to proapoptotic triggers.^11^ From the Human Protein Atlas (https://www.proteinatlas.org/), it was noted that HCN2 and HCN3 were found to be unfavourable prognostic markers of renal cancer, and strong cytoplasmic and membranous expression of HCN3 protein expression was observed in colorectal cancers and several other cases including breast cancer.

In the current study, we found HCN2 and HCN3 are overexpressed in breast cancer cells. We hypothesized that interference of HCN function may affect cancer cell viability. We elucidated the underlying molecular mechanisms and successfully illustrated the efficacy of inhibiting HCNs to suppress breast cancer in vitro and in vivo.

## 2 MATERIALS AND METHODS

### 2.1 Cell culture and transfection

Human breast cell lines MCF‐10A, MCF‐7, MDA‐MB‐231, MDA‐MB‐453, BT‐474 and ZR‐75 were purchased from American Type Culture Collection. All cell lines were authenticated as mentioned previously.^12^ All cell lines were free from mycoplasma, confirmed by PCR. Cell lines were not passaged more than 50 times. Lipofectamine 2000 (Invitrogen) was employed for the transfection of plasmids. Oligofectamine (Invitrogen) was used for siRNA transfection.

### 2.2 Chemicals

A total of 100 mM of Ivabradine (Sigma), 100 mg/mL of puromycin (Sigma), 50 mM of sodium phenylbutyrate (4‐PBA; Santa Cruz Biotechnology) and 50 mg/mL of doxycycline hyclate (Santa Cruz Biotechnology) were dissolved in double‐distilled water. A total of 50 μM of Paclitaxel (Selleckchem) was dissolved in DMSO. Water and/or saline were used as solvent control in in vitro and in vivo experiments. Also, 10 mM of Inositol 1,4,5‐Trisphosphate (407137; Sigma) in water was used. A sum of 1 mM of Fura‐2‐AM (Invitrogen) stock solution in DMSO was prepared and 10 mg/mL of Hoechst33342 was purchased from ThermoFisher.

### 2.3 RNA sequencing

The sequencing was performed by Centre for Genomic Sciences (The University of Hong Kong). Illumina NovaSeq 6000 was used for Pair‐End 151bp sequencing. Using software from Illumina (bcl2fastq), sequencing reads were assigned. An average of 95% of the bases achieved an accuracy of 99.9%.

### 2.4 Orthotopic xenograft

Female nude mice aged between 5 and 6 weeks were used. A total of 1 × 10^7^ of MDA‐MB‐231 or MDA‐MB‐453 cells were inoculated onto the mammary fat pad of the mice. Also, 15 mg/Kg of Ivabradine or water was injected subcutaneously daily. For the combined treatment of Ivabradine and Paclitaxel experiment, both drugs were injected subcutaneously, twice a week. All the procedures were reviewed and approved by HKU Committee on the Use of Live Animals in Teaching and Research (CULATR No. 4204–16) for xenograft model.

### 2.5 Patient‐derived tumour xenografts (PDTX)

Triple‐negative breast cancer (TNBC) patients were recruited from Pamela Youde Nethersole Eastern Hospital and Kwong Wah Hospital with approval by Hong Kong East Cluster Research Ethics Committee (REC Reference No. HKEC‐2016‐063) and Kowloon West Cluster Research Ethics Committee (REC Reference No. KW/EX‐16‐158[103‐11]). All the procedures were reviewed and approved by HKU Committee on the Use of Live Animals in Teaching and Research (CULATR No. 4218‐16) for patient‐derived tumour xenograft (PDTX) model, and the Institutional Review Board of the University of Hong Kong/Hospital Authority Hong Kong West Cluster (HKU/HA HKW IRB No. UW 16–391) for collection of human tissue. A total of 1 × 10^7^ of the human TNBC cells were inoculated onto the mammary fat pad of the mice and 15 mg/kg of Ivabradine or water was injected subcutaneously twice a week for 4 weeks.

### 2.6 Fluorescent microscopy and live‐cell imaging

Carl Zeiss LSM 700/710 confocal laser scanning microscope system with Diode laser (405 nm), Argon laser (458/488/514 nm), SPSS laser (561 nm) and HeNe laser (633 nm). The filter cube contained filters for DAPI, DsRed, EGFP and Cy5. Cells were seeded on a coverslip and treated with 50 μM of Ivabradine for 72 h. On the day of imaging, 4 μM of Fura‐2‐AM was used to treat the cells for 30 min. The cells were then washed with 1× PBS three times. The coverslip was mounted on a coverslip chamber. The cells were excited by laser with 405 nm and signals were collected from EGFP filter at room temperature. Microplate reader Infinite F200 (Tecan) was employed to record the fluorescent signal. A total of 10 000 cells were seeded on 96‐well plate and treated with 50 μM of Ivabradine for 72 h. A sum of 4 μM of Fura‐2‐AM was used to treat the cells for 30 min. The cells were then washed with 1× PBS three times. The cells were excited by UV Xenon flashlamp. The signal was recorded with a 570 nm filter at room temperature. Nikon H600L fluorescent microscope was used for fluorescence analysis.

### 2.7 Subcellular fractionation

Subcellular fractionation was performed by using NE‐PER Nuclear and Cytoplasmic Extraction Reagents (Thermo Scientific), Subcellular Protein Fractionation Kit (Thermo Scientific) and Mitochondria Isolation Kit (Thermo Scientific).

### 2.8 Cell viability

MTT assay was performed (USB, Affymetrix). Clonogenic assay was performed. Also, 0.01% of crystal violate was employed to stain the cell colonies.

### 2.9 Flow cytometry

TUNEL assay was performed using APO‐BrdU TUNEL Assay Kit (Invitrogen). Annexin V/PI staining (Dead Cell Apoptosis Kit with Annexin V FITC and PI; Invitrogen) was performed. BD LSR Fortessa analyser (BD Biosciences) was used for analysing the cells. The cells were gated by (1) SSC‐A versus FSC‐A, (2) FSC‐W versus FSC‐H and (3) SSC‐W versus SSC‐H for singlets. The results were analysed and processed by the software FlowJo V10.

### 2.10 Luciferase reporters and caspase 3/7 activity assays

The luciferase reporter assay was performed using Dual‐Luciferase Reporter assay system (Promega). Cignal ERSE Reporter Kit (ATF4 response element; CCS‐2032L; Qiagen) was purchased for determining ER‐stress. Caspase‐Glo 3/7 Assay Systems (Promega) and Cell Event Caspase 3/7 Green Detection Reagent (Invitrogen) were employed to determine the activity of caspases. The chemiluminescence and fluorescent signals generated were recorded by Infinite F200 (Tecan).

### 2.11 Tissue microarray

A cohort totalling 316 breast cancer patients diagnosed between 1993 and 2003 with pathological and clinical follow‐up data of over 20 years were used for this study. All cases were retrieved from the records of the Department of Pathology, Queen Mary Hospital of Hong Kong, with approval by the Institutional Review Board of The University of Hong Kong (UW 08–147). Clinical pathological characteristics of the cases are summarized in Supporting information Table S1.

### 2.12 Immunohistochemistry

Antibodies used were anti‐Ki‐67 (M7240; 1:150 Dako), anti‐HCN2 (APC‐030; 1:300; Alomone Labs) and anti‐HCN3 (APC‐057; 1:500; Alomone Labs). Aperio ScanScope system (Aperio technology) was used for assessing the protein expression of HCN2 and HCN3.

### 2.13 Statistical and pathway enrichment analysis

METABRIC dataset from TCGA was used. The effect of Ivabradine on different molecular functions was determined using databases from Gene ontology (GO),^13^ Reactome,^14^ and gene set enrichment analysis (GSEA).^15^ All numerical data were processed in Excel (Microsoft), Prism5 (GraphPad), or SPSS25 (IBM). All data were expressed as mean ± SD from at least three independent experiments. G*Power software was used to determine the number of samples required to achieve 80% power.^16^ We assumed there was 50% difference between normal and tumour tissues and the ratio of normal to tumour samples was 1:7. A sum of 30 normal samples and 210 tumour samples will be the minimal number of samples from each group to achieve effective analysis. Mann–Whitney U test was used to determine the statistical difference between the expression of HCN2 and HCN3 in normal and tumour tissues because the data did not pass Shapiro–Wilk normality test which was a test to determine if the distribution of data was normally distributed. A two‐tailed Students’ *t*‐test was performed to compare the means of the two sample groups. Comparison between multiple groups was determined by one‐way analysis of variance (ANOVA) and two‐way ANOVA. Bonferroni test was used as a post‐test after one‐way ANOVA to compare means between two groups. Correlation with survival study of Tissue Microarray data was analysed by Kaplan–Meier estimates followed by Log‐rank test carried out by SPSS. Cox proportional hazards regression was used to estimate the association between clinical‐pathological parameters, or HCN scores with survival. Relative risk (RR) and 95% confidence interval (CI) were reported. The proportional‐hazards assumption was tested using the Omnibus test, and no major model violation was observed. Combination Index (CI) of drug‐drug interaction was calculated by using the formula: CI = [*a*/*A*] + [*b*/*B*] where *a* is the half‐maximal effective concentration (EC_50_) of drug 1 in the presence of drug 2; *A* is the EC_50_ of drug 1 without drug 2; *b* is the EC_50_ of drug 2 in the presence of drug 1; *B* is the EC_50_ of drug 2 without drug 1. EC_50_ is half‐maximal effective concentration. CI less than 1 suggested the presence of the synergistic effect.^17^ EC_50_ was calculated using Prism5 (GraphPad) by non‐linear regression (log_10_[drug] vs. normalized response). *P*‐value of less than .05 was considered statistically significant.

Detailed procedures, sequences and antibodies are listed in Supporting information.

## 3 RESULTS

### 3.1 HCN2 and HCN3 are overexpressed in breast cancer cells and associated with poor prognosis

**FIGURE 1:**
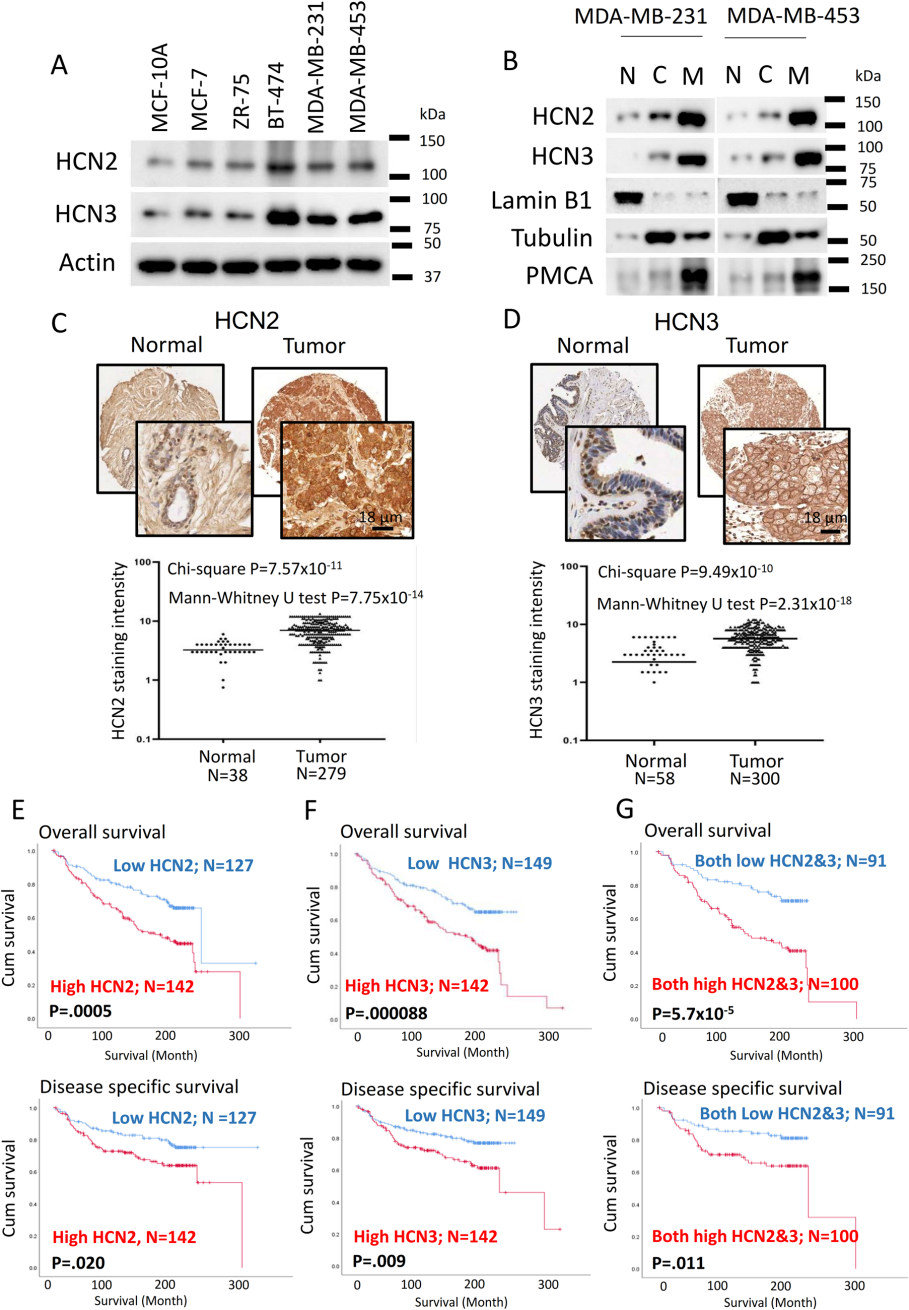
HCN 2 and HCN3 were overexpressed in breast cancer and associated with poor survival outcomes. A, Protein expressions of HCN2 and HCN3 were determined in breast cancer cell lines compared with non‐neoplastic breast epithelial cell line MCF‐10A by western blot. Actin was used as the loading control. Results were repeated three times and representative images are shown. B, HCN2 and HCN3 were predominantly localized to the cell membrane. MDA‐MB‐231 and MDA‐MB‐453 cells were used, and fractionation was performed to isolate different cellular fractions. Western blot was employed to determine the expression of the proteins. Lamin B1, PMCA and tubulin were used as nuclear (N), cell membranous (M) and cytoplasmic (C) markers, respectively. Results were repeated three times and representative images are shown. C, HCN2 was overexpressed in breast cancer. IHC was employed to determine the expression of HCN2 in normal (N = 38) and tumor tissues (N = 279). Mann−Whitney U test (*P* = 7.75 × 10^−14^) and chi‐square test (*P* = 7.57 × 10^−11^) were employed to determine the statistical significance. The scale bar represents 18 μm. D, HCN3 was overexpressed in breast cancer. IHC was employed to determine the expression of HCN3 in normal (N = 58) and tumor tissues (N = 300). Mann−Whitney U test (*P* = 2.31 × 10^−18^) and chi‐square test (*P* = 9.49 × 10^−10^) were employed to determine the statistical significance. The scale bar represents 18 μm. E, Breast cancer patients with high expression of HCN2 were associated with poorer survival outcomes. The patients with high expression of HCN2 (N = 142) in their breast cancer showed poorer overall survival (*P* = .0005, log‐rank test) and poorer disease‐specific survival (*P* = .020, log‐rank test). Red line represents the patient group with high HCN2 expression while blue line represents the patient group with low HCN2 expression. F, Breast cancer patients with high expression of HCN3 were associated with poorer survival outcomes. The patients with high expression of HCN3 (N = 142) in their breast cancer showed poorer overall survival (*P* = .000088, log‐rank test) and poorer disease‐specific survival (*P* = .009, log‐rank test). Red line represents the patient group with high HCN3 expression while blue line represents the patient group with low HCN3 expression. G, The patients with high expression of both HCN2 and HCN3 (N = 100) would have the poorest survival outcome when compared with low expressers of both (N = 91), for both overall survival (*P* = 5.7 × 10^−5^, log‐rank test) and disease‐specific survival (*P* = .011, log‐rank test). Red line represents the patient group with both high HCN2 and HCN3 expression while blue line represents the patient group with both low HCN2 and HCN3 expression. High expression of HCN2 and HCN3 was defined that the staining score is more than the median score.

Using the METABRIC database from TCGA, we found a significant difference in expression between breast tumour tissues compared with normal breast tissue in HCN2 (*P* < .001) and HCN3 (*P* < .001) but not in HCN1 and HCN4 (Supporting information Figure S1A‐D). We also examined the mRNA levels of HCN1‐4 in various breast cancer cell lines, including the estrogen‐receptor positive (MCF‐7, ZR‐75), HER2‐positive (BT‐474) and TNBC (MDA‐MB‐231, MDA‐MB‐453) cells compared with non‐neoplastic MCF‐10A breast epithelial cells. We confirmed that only HCN2 and HCN3 (Supporting information Figure S2A‐D) were upregulated in breast cancer cells. Overexpression of HCN2 and HCN3 proteins was consistently found in breast cancer cells (Figure 1A), with BT474 and TNBC cells MDA‐MB‐231, MDA‐MB‐453 showing the most significant upregulation. We found both HCN2 and HCN3 were predominantly localized to the cell membrane (Figure 1B), suggesting that HCN2 and HCN3 could be functionally important in breast cancer cells.

Using immunohistochemistry to examine HCN2 and HCN3 protein expression of primary breast cancer in tissue microarray, we confirmed that HCN2 (Figure 1C) and HCN3 (Figure 1D) were significantly upregulated in tumour compared with normal breast epithelia. From the analysis of 316 breast cancer cases, those cases which showed both high HCN2 and HCN3 expression were most frequently of the TNBC subtype (68.4%) (Table S2), prompting us to focus our study on TNBC. Patients with high HCN2 or HCN3 expression showed poorer overall, disease‐specific survival (Figure 1E and F) and patients expressing both high HCN2 and HCN3 showed the poorest survival when compared with both low expressers (Figure 1G). Cox‐regression analysis (Table 1A) showed cases with both high HCN2 and HCN3 was statistically significantly associated with poorer overall survival (*P* = .000104; Table 1A) and disease‐free survival (*P* = .013; Table 1B), which remained significant after multivariate cox‐regression analyses (*P* = .003; Table 1 and *P* = .017; Table 1B) for overall survival and disease‐free survival, respectively. These findings suggest that HCN2 and HCN3 may be oncogenic in breast cancer.

**TABLE 1A:** Cox regression analyses of overall survival

	Univariate analysis	
Clinical‐pathological parameters	RR (95% CI)	*P* value
Age (n = 306)	1.785 (1.256, 2.535)	**.001**
T‐stage (n = 199)	2.079 (1.095, 3.947)	**.025**
Lymph‐node involvement (n = 274)	1.421 (0.979, 2.063)	.064
Tumor‐grade (n = 274)	1.099 (0.757, 1.595)	.62
Histological type (n = 296)	0.885 (0.553, 1.415)	.610
Estrogen receptor status (n = 225)	0.74 (0.463, 1.182)	.207
HER2 status (n = 186)	1.228 (0.669, 2.255)	.507
Triple‐negative (n = 202)	1.219 (0.626, 2.375)	.56
Tumor size (n = 115)	1.7 (0.882, 3,277)	.113
Cases with Hi‐HCN2 cytoplasm score (n = 269)	1.952 (1.331, 2.861)	**.001**
Cases with Hi‐HCN3 cytoplasm score (n = 291)	2.029 (1.413, 2.913)	**.000125**
Both Hi‐HCN2 & Hi‐HCN3 cytoplasm score (n = 189)	2.567 (1.594, 4.132)	**.000104**

	Multivariate analysis	
Clinical‐pathological parameters	RR (95% CI)	*P* value
Age (n = 190)	1.869 (1.171, 2.984)	**.009**
T‐stage (n = 190)	1.565 (0.811, 3.021)	.182
Cases with Hi‐HCN2 cytoplasm score (n = 190)	2.01 (1.231, 3.284)	**.005**

	Multivariate analysis	
Clinical‐pathological parameters	RR (95% CI)	*P* value
Age (n = 189)	1.786 (1.115, 2.859)	**.016**
T‐stage (n = 189)	2.279 (1.198, 4.334)	**.012**
Cases with Hi‐HCN3 cytoplasm score (n = 189)	1.983 (1.232, 3.19)	**.005**

	Multivariate analysis	
Clinical‐pathological parameters	RR (95% CI)	*P* value
Age (n = 133)	1.809 (1.015, 3.225)	**.044**
T‐stage (n = 133)	2.75 (1.25, 6.051)	**.012**
Both Hi‐HCN2 & Hi‐HCN3 cytoplasm score (n = 133)	2.611 (1.394, 4.888)	**.003**

**TABLE 1B:** Cox regression analyses of disease‐specific survival

	Univariate analysis	
Clinical‐pathological parameters	RR (95% CI)	*P* value
Age (n = 306)	0.837 (0.541, 1.296)	.426
T‐stage (n = 199)	1.649 (0.729, 3.731)	.23
Lymph‐node involvement (n = 274)	1.967 (1.208, 3.201)	**.007**
Tumor‐grade (n = 274)	1.807 (1.123, 2.907)	**.015**
Histological type (n = 296)	0.89 (0.49, 1.615)	.701
Estrogen receptor status (n = 225)	0.57 (0.338, 0.962)	**.035**
HER2 status (n = 186)	1.333 (0.66, 2.691)	.423
Triple‐negative (n = 202)	1.651 (0.803, 3.392)	.173
Tumor size (n = 115)	2.419 (0.969, 6.036)	.058
Cases with Hi‐HCN2 cytoplasm score (n = 269)	1.731 (1.082, 2.769)	**.022**
Cases with Hi‐HCN3 cytoplasm score (n = 291)	1.822 (1.156, 2.871)	**.01**
Both Hi‐HCN2 & Hi‐HCN3 cytoplasm score (n = 189)	2.154 (1.178, 3.937)	**.013**

	Multivariate analysis	
Clinical‐pathological parameters	RR (95% CI)	*P* value
Lymph‐node involvement (n = 177)	1.473 (0.82, 2.647)	.195
Tumor‐grade (n = 177)	1.581 (0.819, 3.05)	.172
Estrogen receptor status (n = 177)	0.71 (0.373, 1.352)	.297
Cases with Hi‐HCN2 cytoplasm score (n = 177)	1.911 (1.031, 3.544)	**.04**

	Multivariate analysis	
Clinical‐pathological parameters	RR (95% CI)	*P* value
Lymph‐node involvement (n = 186)	1.42 (0.801, 2.518)	.23
Tumor‐Grade (n = 186)	1.687 (0.88, 3.235)	.115
Estrogen receptor status (n = 186)	0.715 (0.38, 1.343)	.297
Cases with Hi‐HCN3 cytoplasm score (n = 186)	2.337 (1.296, 4.215)	**.005**

	Multivariate analysis	
Clinical‐pathological parameters	RR (95% CI)	*P* value
Lymph‐node involvement (n = 125)	1.748 (0.853, 3.586)	.127
Tumor‐grade (n = 125)	2.563 (1.003, 6.548)	**.049**
Estrogen receptor status (n = 125)	0.747 (0.353, 1.582)	.447
Both Hi‐HCN2 & Hi‐HCN3 cytoplasm score (n = 125)	2.724 (1.197, 6.196)	**.017**

### 3.2 HCN suppression inhibits TNBC growth in vitro and in vivo

**FIGURE 2:**
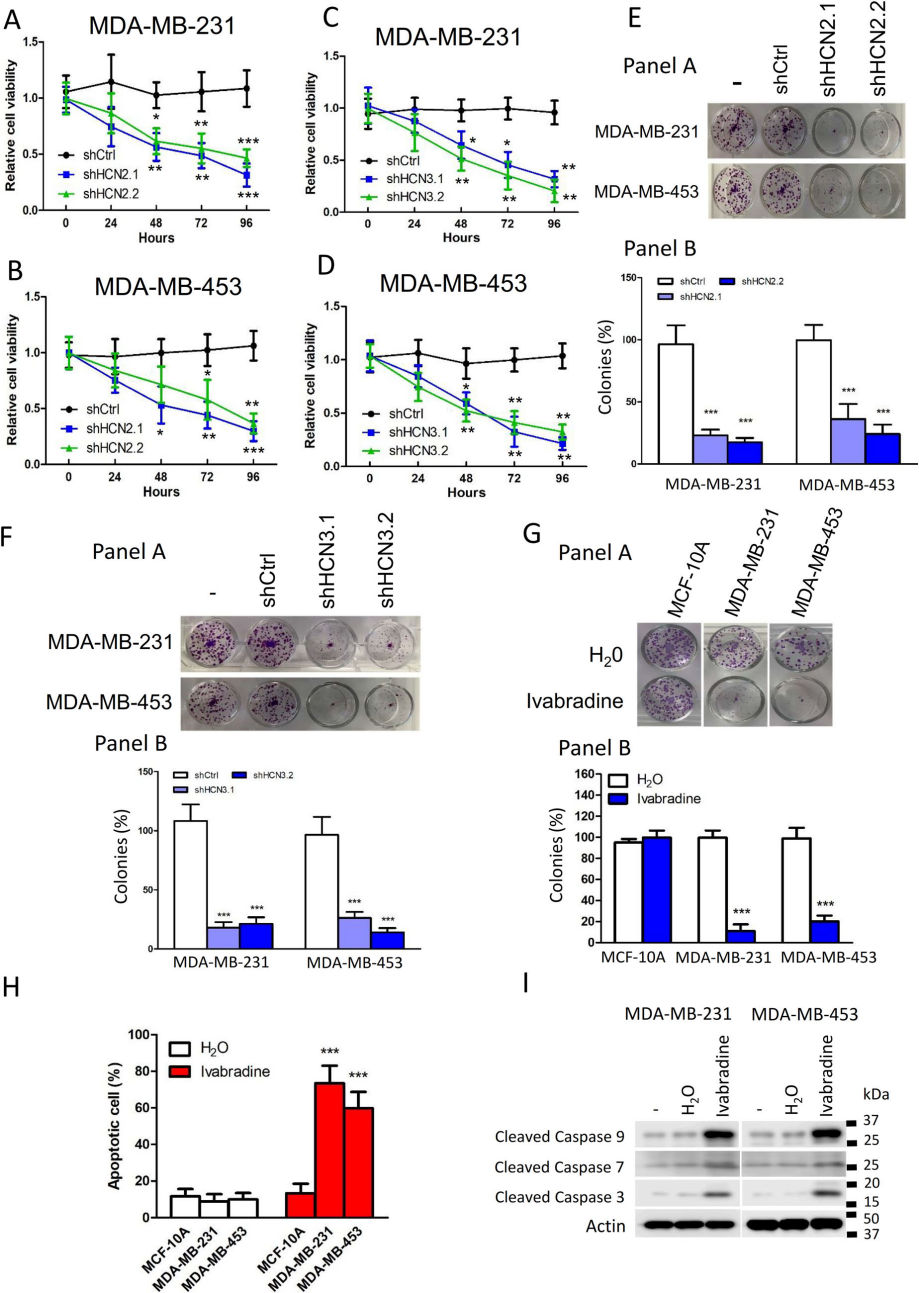
Suppression of HCN2 and HCN3 inhibited breast cancer cell growth in vitro. The knockdown of HCN2 could suppress the cell proliferation of TNBC cells A, MDA‐MB‐231 and B, MDA‐MB‐453 in the short term as assessed by MTT assay. Two independent shRNAs against HCN2, shHCN2.1 and shHCN2.2 were used. Results were shown as mean ± SD from three independent experiments. The knockdown of HCN3 could suppress the cell proliferation of TNBC cells C, MDA‐MB‐231 and D, MDA‐MB‐453 in the short term as assessed by MTT assay. Two independent shRNAs against HCN3, shHCN3.1 and shHCN3.2 were used. Results were shown as mean ± SD from three independent experiments. Two‐way ANOVA revealed there was a statistically significant interaction between the HCN2/3 knockdown and time (A: F = 2.71, *P* < .05; B: F = 4.71, *P* < .001; C: F = 6.00, *P* < .001; D: F = 7.43, *P* < .001). Statistical significance between shCtrl and shHCN2.1 or shHCN2.2 or between shCtrl and shHCN3.1 or shHCN3.2 at each time point was determined by Bonferroni test. *, ** and *** represent *P* < .05, *P* < .01 and *P* < .001 respectively. The knockdown of either HCN2 or HCN3 could suppress the cell proliferation of TNBC cells E, MDA‐MB‐231 and F, MDA‐MB‐453 in the long term as determined by clonogenic assays. Results were shown as mean ± SD from three independent experiments. One‐way ANOVA was used. Statistical significance between shCtrl and shHCN2.1/shHCN3.1 or shHCN2.2/shHCN3.2 was determined by Bonferroni test. *** represents *P* < .001. G, Inhibition of HCNs by Ivabradine could suppress the cell proliferation of TNBC cells MDA‐MB‐231 and MDA‐MB‐453 but not non‐neoplastic breast epithelial cells MCF‐10A in a clonogenic assay. The cells were incubated with 5 μM of Ivabradine for 2 weeks. Results were shown as mean ± SD from three independent experiments. Students’ *t*‐test was used. *** represents *P* < .001. H, Ivabradine significantly induced apoptosis in MDA‐MB‐231 and MDA‐MB‐453 but not MCF‐10A. A total of 50 μM of Ivabradine was used to treat cells for 48 h. FITC‐Annexin V/PI staining was employed. Cells were analysed by flow cytometry. Results were shown as mean ± SD from three independent experiments. Students’ *t*‐test was used to determine the statistical significance between H_2_O and Ivabradine treated groups. *** represent *P* < .001. I, Ivabradine treatment led to the activation of caspases 3, 7 and 9, as determined by western blot. Actin was used as the loading control. Representative images shown from three independent experiments.

Knockdown of HCN2 and HCN3 using two independent shRNAs significantly suppressed proliferation of TNBC cell lines as revealed by MTT assay (Figure 2A‐D and Supporting information Figure S3A and B) and clonogenic assay (Figure 2E and F). We confirmed that the shRNAs only hit the designated target but not the other members of the HCN family (Supporting information Figure S3A and B). Additionally, we employed Ivabradine, which is an FDA‐approved HCN channel blocker used clinically to treat chronic angina,^5^ to target HCN. The effect on cancer cell viability was dose‐dependent, with more than 50% reduction in both cell lines at Ivabradine concentrations greater than 5 μM (Supporting information Figure S4A and B). Results from clonogenic assay suggested that 5 μM Ivabradine could effectively suppress cell proliferation of MDA‐MB‐231 and MDA‐MB‐453 but not the non‐neoplastic cells MCF‐10A (Figure 2G). Furthermore, Ivabradine treatment could induce apoptosis in breast cancer cells but not MCF‐10A as revealed by Annexin V/PI staining (Figure 2H and Supporting information Figure S4C), consistent with increased levels of active caspase 3, 7 and 9 (Figure 2I). Ivabradine treatment, however, did not affect the expression of HCN2 and HCN3 in TNBC cells (Supporting information Figure S4D).

**FIGURE 3:**
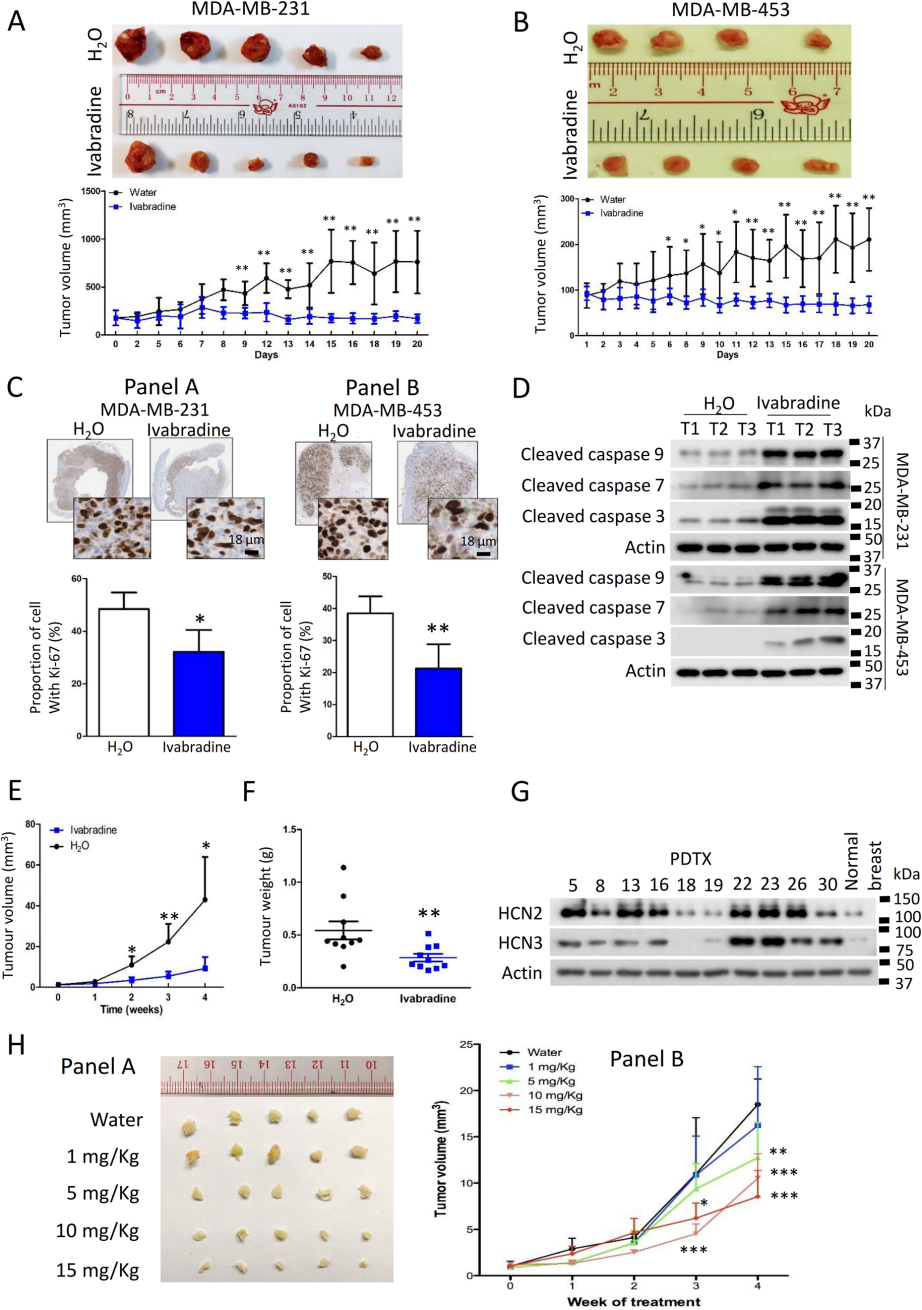
Ivabradine suppressed TNBC tumor growth in vivo. A, A comparison of tumours isolated from nude mice treated with 15 mg/kg of Ivabradine (N = 5) or water (N = 5) administered subcutaneously daily for 4 weeks. MDA‐MB‐231 was used to establish the xenografts. The graph shows the change in tumor volume with the time between Ivabradine and water‐treated groups. Results were shown as mean ± SD from five tumours. Students’ *t*‐test was used. ** represents *P* < .01. B, A comparison of tumours isolated from nude mice treated with 15 mg/Kg of Ivabradine (N = 4) or water (N = 4) administered subcutaneously daily for 4 weeks. MDA‐MB‐453 was used to establish the xenografts. The graph showed the change in tumor volume with the time between Ivabradine and water‐treated groups. Students’ *t*‐test was used to compare tumor sizes at the various time points between the Ivabradine and water‐treated groups. Results were shown as mean ± SD from four tumours. * and ** represent *P* < .05 and *P* < .01, respectively. C, Treatment with Ivabradine could suppress cell proliferation in tumours developed from MDA‐MB‐231 (Panel A) and MDA‐MB‐453 (Panel B). IHC was employed to determine the expression of cell proliferation marker Ki‐67. Results were shown as mean ± SD from five MDA‐MB‐231 tumours and four MDA‐MB‐453 tumours. Students’ *t*‐test was used. * and ** represent *P* < .05 and *P* < .01, respectively. The scale bar represents 18 μm. D, Ivabradine could activate caspases 9, 7 and 3. Protein lysates from three individual tumor samples were collected. Western blot was performed to determine the expression of cleaved caspase 3. Actin was used as the loading control. E, Ivabradine treatment could significantly reduce the volume of tumours in PDTX models. Ten PDTX models were tested. A total of 15 mg/kg Ivabradine was administered subcutaneously twice a week for 4 weeks. Each spot represented the average tumor volume of each of the PDTX models. Results were shown as mean ± SD from ten PDTX models. Students’ *t*‐test was used. * and ** represent *P* < .05 and *P* < .01, respectively. F, Ivabradine treatment could significantly reduce the weight of tumours in PDTX models. Ten PDTX models were tested. A total of 15 mg/kg Ivabradine was administered subcutaneously twice a week for 4 weeks. Each spot represented the average tumor weight of each of the PDTX models. Results were shown as mean ± SD from ten PDTX models. Mann–Whitney U test was used. ** represents *P* < .01. G, Expression of HCN2 and HCN3 in tumours from PDTX samples compared with normal breast lysate (1346‐N). Western blot was employed to detect the expression of HCN2 and HCN3. Actin was used as the loading control. H, The change of tumor volume in response to different dosages of Ivabradine given twice weekly for 4 weeks. PDTX5 was used. Tumor images were shown in Panel A and the graph showing the change in tumor volume was shown in Panel B. Two‐way ANOVA revealed there was no statistically significant interaction between the effects of the dosage and the treatment length (F = 3.54, *P* < .001). Bonferroni was used as a post‐test after two‐way ANOVA to determine the statistical significance between water control and different dosages of Ivabradine at each time point. *, ** and *** represent *P* < .05, *P* < .01 and *P* < .001.

We validated the tumour‐suppressive effect of Ivabradine in xenograft models. The nude mice were treated with either Ivabradine or water subcutaneously (15 mg/kg daily). The results showed a significant reduction in tumour volume for both MDA‐MB‐231 (Figure 3A) and MDA‐MB‐453 (Figure 3B) xenografts. Consistently, Ivabradine could significantly reduce the percentage of Ki‐67 positive cells (Figure 3C) and enhance the level of cleaved caspases 3, 7 and 9 in tumour samples shown by western blot (Figure 3D). Also, we confirmed that Ivabradine treatment did not affect the expression of HCN2 and HCN3 in vivo (Supporting information Figure S5). Similarly, depletion of HCN2 or HCN3 by shRNA could significantly reduce the growth rate of the tumours in vivo (Supporting information Figure S6A and B).

We tested the effect of Ivabradine on ten PDTX models generated from TNBC cases. The mice were treated with either Ivabradine (15 mg/Kg twice weekly) or water. The results showed that Ivabradine could significantly reduce tumour volume (Figure 3E) and tumour weight (Figure 3F) in 8 out of 10 PDTX models (Supporting information Figure S7). Western blot showed the two cases, which are non‐responsive to Ivabradine (PDTX18 and PDTX19) and had low HCN2 and HCN3 expression (Figure 3G). Ivabradine also exerted a dose‐dependent effect in the PDTX model, the minimal effective dosage being 5 mg/kg twice weekly (Figure 3H) but had no effect on HCN2 and HCN3 expression (Supporting information Figure S8).

Ivabradine could reduce the heart rate 20 min immediately after the injection, consistent with the known effect of the drug (Supporting information Figure S9A). However, its cumulative effect of 4 weeks treatment did not induce abnormal heart rhythm in the mice after 4 weeks of treatment (Supporting information Figure S9B). Cage‐side observation of mice treated with Ivabradine showed no observable adverse effect (>10% weight loss in body weight, signs of illness, or abnormal behaviour). Also, there was no sign of liver toxicity as the serum aspartate transaminase and alanine transaminase levels remained the same as the control group (Supporting information Figure S9C). These findings suggest that Ivabradine was relatively free of obvious side‐effects.

### 3.3 Transcriptome‐wide analysis showed HCN inhibition activates ER‐stress response leading to apoptosis

**FIGURE 4:**
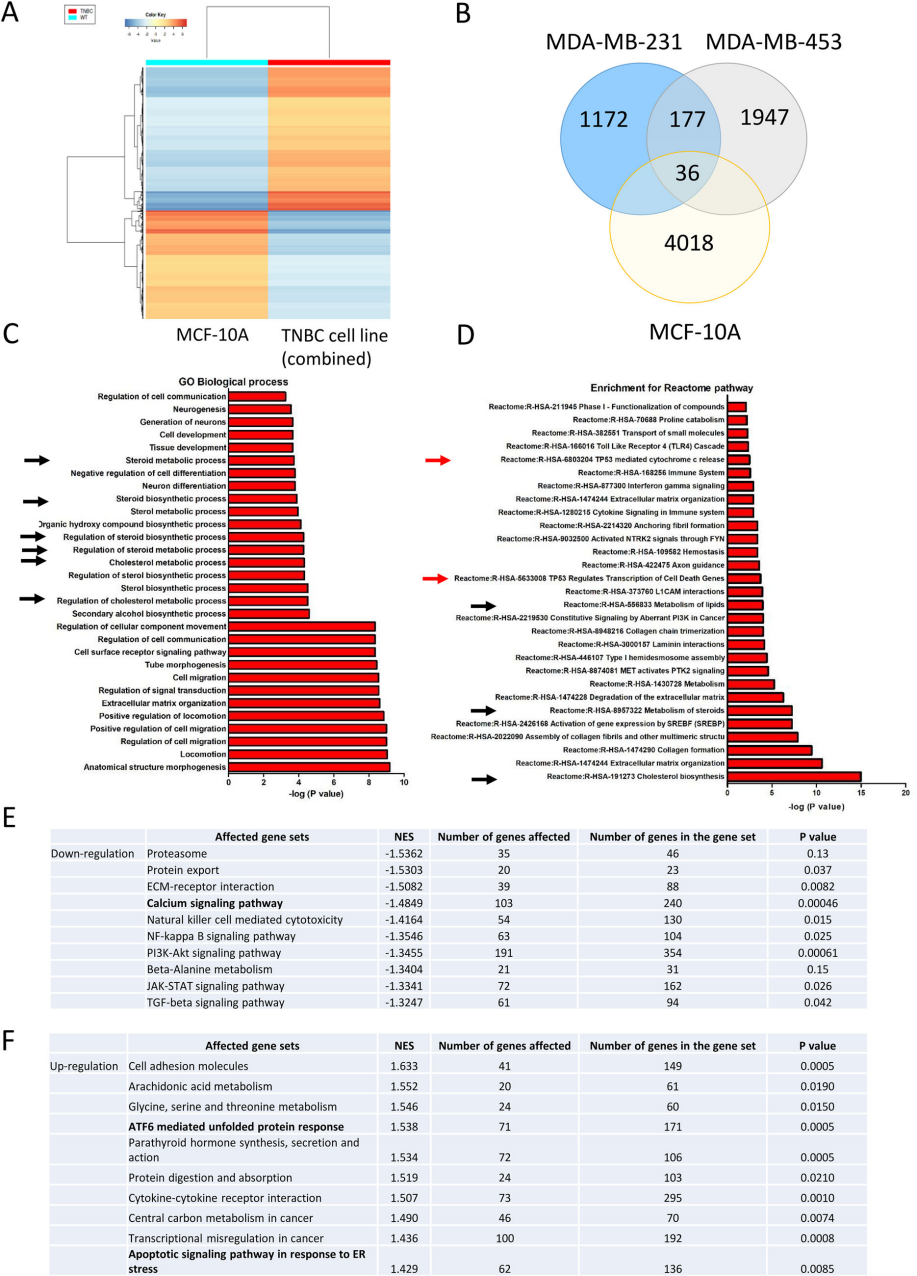
RNA‐seq analysis revealed transcriptome changes following inhibition of HCN. A, Ivabradine exerted distinctively different effects on TNBC cells compared with non‐cancerous breast cells. MDA‐MB‐231, MDA‐MB‐453 and MCF‐10A were used. The cells were either treated with 100 μM of Ivabradine or water for 72 h. RNA sequencing was performed to determine the gene expression profile. Gene expression levels were determined by transcript per millions (TPM). The differential expressions of genes in each of the cell lines between 72 h Ivabradine‐treated and water control were determined. The results from MDA‐MB‐231 and MDA‐MB‐453 were grouped as TNBC. The mean value of the differential expression level of each of the genes from MDA‐MB‐231 and MDA‐MB‐453 was calculated. A heatmap was plotted to show the distinctive effect of Ivabradine on differential gene expression profiles between normal breast and TNBC cells. B, Venn diagram was plotted to show the number of genes commonly and upregulated explicitly in MDA‐MB‐231 and MDA‐MB‐453 compared with MCF‐10A. A total of 177 genes were found. C, Gene ontology enrichment analysis was performed to identify the biological processes that were significantly affected based on these 177 genes. The top 30 biological processes were shown. Black arrows highlight steroid and cholesterol metabolic/biosynthetic processes. **D**, Pathway enrichment analysis was performed to identify the cellular pathways that were significantly affected by Ivabradine treatment. Information from Reactome was retrieved to perform the analysis. All potentially affected pathways were shown. Black arrows highlight steroid/lipid metabolic and cholesterol biosynthesis pathways. Red arrows highlight apoptosis‐related pathways. E, The top 10 gene sets being downregulated in Ivabradine‐treated TNBC cells. F, The top 10 gene sets being upregulated in Ivabradine‐treated TNBC cells. Gene expression profiles of MDA‐MB‐231 and MDA‐MB453 treated with H_2_O and Ivabradine were compared. Commonly affected pathways were retained. NES is normalized enrichment score which is the primary statistic for examining gene set enrichment results.

We performed RNA sequencing to reveal the effect of Ivabradine on global cellular activities in non‐cancerous epithelial breast cells MCF‐10A and TNBC cells MDA‐MB‐231 and MDA‐MB‐453. The findings showed Ivabradine treatment resulted in a distinct expression profile in MCF‐10A cells compared with that in TNBC cells (Figure 4A; Supporting information Table S3). We found 177 genes commonly and specifically upregulated by Ivabradine in TNBC cells (Figure 4B). GO enrichment analysis identified the biological processes that were significantly affected by Ivabradine in TNBC, the top 30 biological processes as shown (Figure 4C). Further analysis using data retrieved from Reactome,^14^ identified the pathways that were significantly affected by Ivabradine in TNBC (Figure 4D). The results from GO and Reactome analysis suggested that Ivabradine could alter lipid and cholesterol metabolism. As activation of ER‐stress pathways is known to alter lipid and steroid metabolism,^18^ these findings suggest that Ivabradine may induce ER‐stress. In addition, we employed GSEA to determine the effect of Ivabradine on different molecular mechanisms. Top 10 of the most significantly affected mechanisms in downregulation (Figure 4E; Supporting information Tables S4 and S5) and upregulation (Figure 4F; Supporting information Tables S6 and S7) are shown. From the analysis, Ca^2+^ signalling pathway showed significant downregulation (Figure 4E) while ATF6‐mediated unfolded protein response and apoptotic signalling pathway in response to ER‐stress were found to be enriched (Figure 4F). HCN channels are responsible for the entry of cations into cells^3^, ^19^ including the import of Ca^2+^.^9^, ^10^, ^19^ Since Ivabradine targets HCN channels in affecting ion homeostasis, including that of Ca^2+^, it could induce ER‐stress.

### 3.4 Targeting HCN channels causes ER‐stress and triggers apoptosis in breast cancer

**FIGURE 5:**
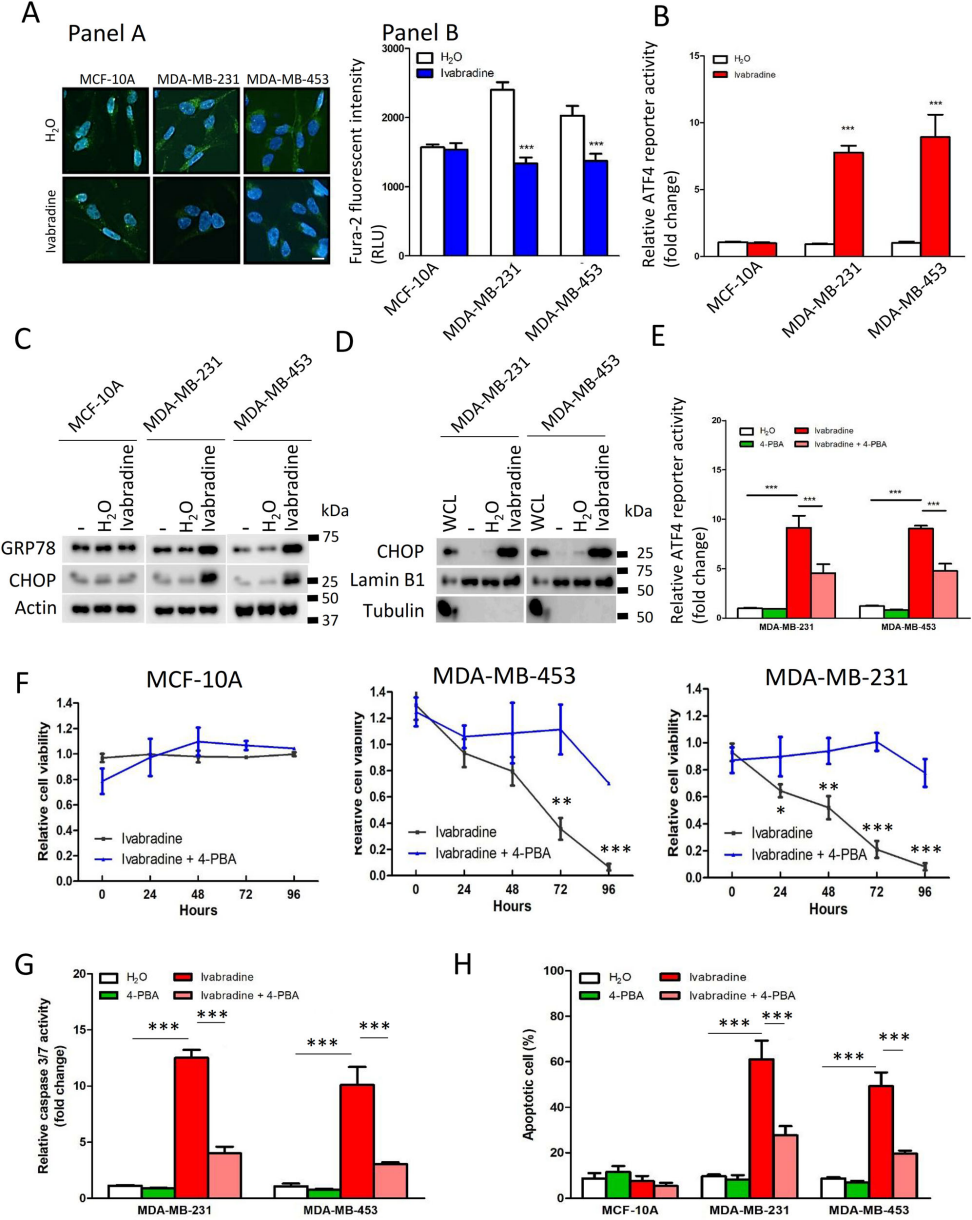
Inhibition of HCN channels reduced the level of intracellular Ca^2+^ and triggered ER‐stress. A, Ivabradine depleted intracellular Ca^2+^ levels in breast cancer cell lines but not in normal epithelial cell line MCF‐10A. A total of 4 μM of Fura‐2 was used to stain intracellular calcium ions for 30 min. Live cell imaging was performed to capture the image (Panel A). Infinite F200 microplate reader was employed to record the fluorescent signal from 10 000 cells (Panel B). Results were shown as mean ± SD from three independent experiments. Students’ *t*‐test was employed to determine statistical significance between H_2_O and Ivabradine‐treated groups. *** represents *P* < .001. The scale bar represents 5 μm. B, HCN inhibition by Ivabradine significantly induced ER‐stress response as measured by ATF4 luciferase reporter activities. Renilla luciferase was used as the internal control. A total of 10 μM of Ivabradine was used to treat the cells for 72 h. Results were shown as mean ± SD from three independent experiments. Students’ *t*‐test was employed to determine statistical significance between H_2_O and Ivabradine‐treated groups. *** represents *P* < .001. C, Ivabradine could enhance the protein expression of GRP78 and CHOP in MDA‐MB‐231 and MDA‐MB‐453 but not in MCF‐10A. Also, 10 μM of Ivabradine was used to treat the cells for 72 h. Western blot was used to determine protein expression. Actin was the loading control. Representative image from three independent experiments. D, Ivabradine enriched the nuclear localization of CHOP. Ten micromolar of Ivabradine was used to treat the cells for 48 h. Nucleocytoplasmic fractionation was performed to isolate the nuclear fraction. Western blot was used to determine protein expression. Tubulin was a cytoplasmic marker, and Lamin B1 was a nuclear marker. Representative images from three independent experiments. E, 4‐PBA could relieve ER‐stress induced by Ivabradine. A total of 10 μM of Ivabradine and 10 μM of 4‐PBA was used to treat the cells for 72 h. Luciferase reporter assay was used to determine the activity of ATF4. Results were shown as mean ± SD from three independent experiments. One‐way ANOVA was used. Bonferroni was used as post‐test of one‐way ANOVA to determine the statistical significance between two groups. *** represents *P* < .001. F, 4‐PBA could compromise the growth inhibition effect of Ivabradine on breast cancer cell lines by cell viability assay. MTT assay was performed after 72 h of treatment. Ten micromolar of Ivabradine and 10 μM of 4‐PBA was used. Results were shown as mean ± SD from three independent experiments. Students’ *t*‐test was employed. *, ** and *** represent *P* < .05, *P* < .01 and *P* < .001, respectively. G, 4‐PBA could compromise the effect of Ivabradine on caspase activation in breast cancer cell lines. Caspase 3/7 Glo assay was performed after 72 h of treatment. A total of 10 μM of Ivabradine and 10 μM of 4‐PBA was used. Results were shown as mean ± SD from three independent experiments. One‐way ANOVA was used. Bonferroni was used as post‐test of one‐way ANOVA to determine the statistical significance between two groups. *** represents *P *< .001. H, 4‐PBA could compromise the effect of Ivabradine on apoptosis in breast cancer cell lines. Annexin V/PI staining was performed. Flow cytometry was employed for cell analysis. Ten micromolar of Ivabradine and 10 μM of 4‐PBA was used. Results were shown as mean ± SD from three independent experiments. One‐way ANOVA was used. Bonferroni was used as post‐test of one‐way ANOVA to determine the statistical significance between two groups. *** represents *P *< .001

To examine whether induction of ER‐stress was through perturbation of Ca^2+^ homeostasis, we employed live‐cell imaging to examine Ca^2+^ levels in Ivabradine‐treated cells. We found that Ivabradine could reduce cytoplasmic Ca^2+^ levels in breast cancer cell lines but not in MCF‐10A cells that do not overexpress HCN (Figure 5A). As a positive control, when MCF‐10A was further treated with Inositol 1 4 5‐trisphosphate (IP3), a chemical that binds to IP3 receptors on the ER membrane to trigger Ca^2+^ release, this resulted in increased cytoplasmic Ca^2+^ level (Supporting information Figure S10), confirming that Ivabradine was ineffective to alter cytoplasmic Ca^2+^ levels in MCF‐10A.

Interference in Ca^2+^ homeostasis can induce ER‐stress (unfolded protein response),^20^ which will eventually induce apoptosis.^21^ We employed luciferase reporter containing a response element of ATF4, a transcriptional factor activated under ER‐stress to determine the effect of Ivabradine on ER‐stress.^22^ The results showed that 10 μM Ivabradine significantly promoted the luciferase activity in breast cancer cells but not in MCF‐10A (Figure 5B). Consistently, the levels of GRP78 and CHOP, which are two protein markers of ER‐stress, were significantly increased after Ivabradine treatment in MDA‐MB‐231 and MDA‐MB‐453 but not in MCF‐10A cells (Figure 5C). Furthermore, CHOP was enriched in the nucleus after Ivabradine treatment (Figure 5D), suggesting that ER‐stress induced by Ivabradine could not be resolved and cells were committed to apoptosis. These effects were validated in xenograft tumours (Supporting information Figure S11).

To further establish whether ER‐stress is the central mediator for Ivabradine‐induced apoptosis,4‐PBA commonly used to alleviate ER‐stress was employed.^23^ We confirmed that 4‐PBA could reduce the degree of ER‐stress induced by Ivabradine (Figure 5E) and that 4‐PBA could abolish the suppressive effect of Ivabradine on cell viability of breast cancer cells but not of MCF‐10A (Figure 5F). Subsequently, we showed that while 10 μM Ivabradine significantly increased the activities of the caspases, the addition of 4‐PBA compromised the effect of Ivabradine on caspase activities (Figure 5G). Flow cytometry confirmed that 4‐PBA could significantly reduce the proportion of apoptotic cells in Ivabradine‐treated breast cancer cells but not in MCF‐10A (Figure 5H). These results support our conclusion that Ivabradine induces cell death via ER‐stress.

**FIGURE 6:**
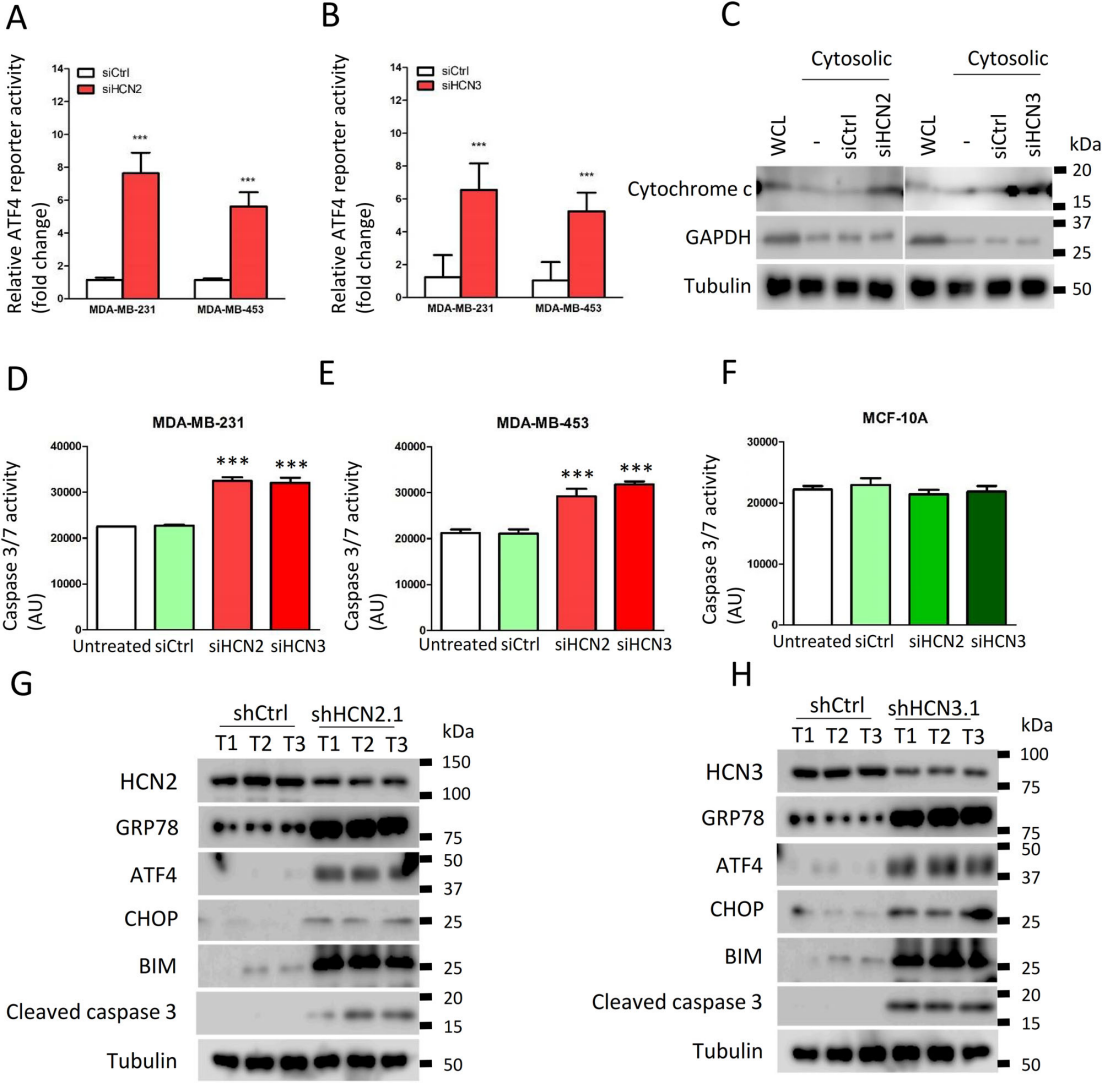
Knockdown of HNC2‐ and HCN3‐induced ER‐stress and apoptosis in breast cancer cells. Knockdown of HCN2 and HCN3 in A, MDA‐MB‐231 and B, MDA‐MB‐453 could enhance the activity of ATF4. The cells were treated with 50 pmol of siHCN2, siHCN3, or non‐targeting siRNA (siCtrl) for 72 h. ER‐stress response was determined by ATF4 luciferase reporter assay. Results were shown as mean ± SD from three independent experiments. Students’ *t*‐test was employed to determine statistical significance between two groups. * and *** represents *P* < .05 and *P* < .001, respectively. C, Knockdown of HCN2 and HCN3 promoted cytochrome c release to cytoplasm. The cells were treated with 50 pmol of siHCN2, siHCN3 and non‐targeting siRNA (siCtrl) for 72 h. Mitochondria Isolation Kit was performed to isolate cytoplasmic fraction which was free of mitochondria. Western blot was employed to determine the expression of cytochrome c in the fraction. Tubulin served as loading control and cytoplasmic marker. GAPDH served as mitochondrial marker. Representative image shown from three independent experiments. Knockdown of HCN2 and HCN3 enhanced caspase activity in D, MDA‐MB‐231 and E, MDA‐MB‐453 but not in F, MCF‐10A. The cells were treated with 50 pmol of siHCN2, siHCN3 and non‐targeting siRNA (siCtrl) for 72 h. Cell Event Caspase 3/7 Green Detection Reagent was employed to determine the caspase activity. Infinite F200 microplate reader was employed to record the fluorescent signal. Results were shown as mean ± SD from three independent experiments. One‐way ANOVA was used. Bonferroni was used as post‐test of one‐way ANOVA to determine the statistical significance between untreated and siHCN2 or siHCN3 groups. *** represents *P* < .001. Knockdown of G, HCN2 and H, HCN3 enhanced ER stress and apoptosis in breast tumor. The tumours were isolated from xenografts established from MDA‐MB‐231. Western blot was employed to determine the expression of indicated candidate proteins from three independent tumours. Tubulin was used as the loading control. Representative image shown from three independent experiments.

Since breast cancer cells showed increased expression of both HCN2 and HCN3, we investigated the significance of each to ER‐stress. Knockdown of either HCN2 or HCN3 by siRNA (Supporting information Figure S12A‐D) could induce ER‐stress as revealed by the luciferase assay (Figure 6A and B). Moreover, an enhanced level of free cytochrome c in the cytoplasm was demonstrated by western blot in either HCN2 or HCN3 knockdown cells (Figure 6C). Caspase activities were significantly enhanced in the breast cancer cells but not in MCF‐10A (Figure 6D‐F), indicating the apoptosis cascade had been activated, leading to reduction of viable cells (Supporting information Figure S12E and F). We also validated significantly upregulated expression of ER‐stress markers GRP78, ATF4, CHOP and apoptosis marker BIP, BIM, cleaved caspase 3 in the tumour xenografts with HCN2 or HCN3 knockdown (Figure 6G and H). These data suggest that Ivabradine induced apoptosis of breast cancer cells acts through inhibition of overexpressed HCN2 and HCN3, and not a result of non‐specific cytotoxicity.

To further demonstrate Ivabradine treatment does indeed depend on HCN2 and HCN3, knockdown experiments were performed (Supporting information Figure S12A‐F). While knockdown of HCN2 and HCN3 individually each compromised the effect of Ivabradine (Supporting information Figure S13A and B), double knockdown of HCN2 and HCN3 could further compromise its effect on cell viability in both cell lines (Supporting information Figure S13C and D). To assess the off‐target effects on other HCN family members, HCN1 and HCN4 were silenced in turn in cancer cells (Supporting information Figure S14A and B). Knockdown of HCN1 and HCN4 did not affect Ivabradine efficacy (Supporting information Figure S14C and D).

### 3.5 The synergistic effect of Ivabradine with paclitaxel on TNBC

We compared the effect of Ivabradine, doxorubicin and paclitaxel on cell viability and apoptosis in MDA‐MB‐231, MDA‐MB‐453 and MCF‐10A. Ivabradine could suppress cell proliferation and induce apoptosis of breast cancer cells as effectively as Paclitaxel and Doxorubicin but is much less toxic to non‐neoplastic MCF‐10A cells (Supporting information Figure S15A‐F). Although Ivabradine alone could suppress the proliferation of tumour cells in vitro (5 μM) and in vivo (5 mg/kg), it could not induce tumour regression. Therefore, we explored the possibility of combining paclitaxel with Ivabradine for enhancing the therapeutic effect in TNBC.

**FIGURE 7:**
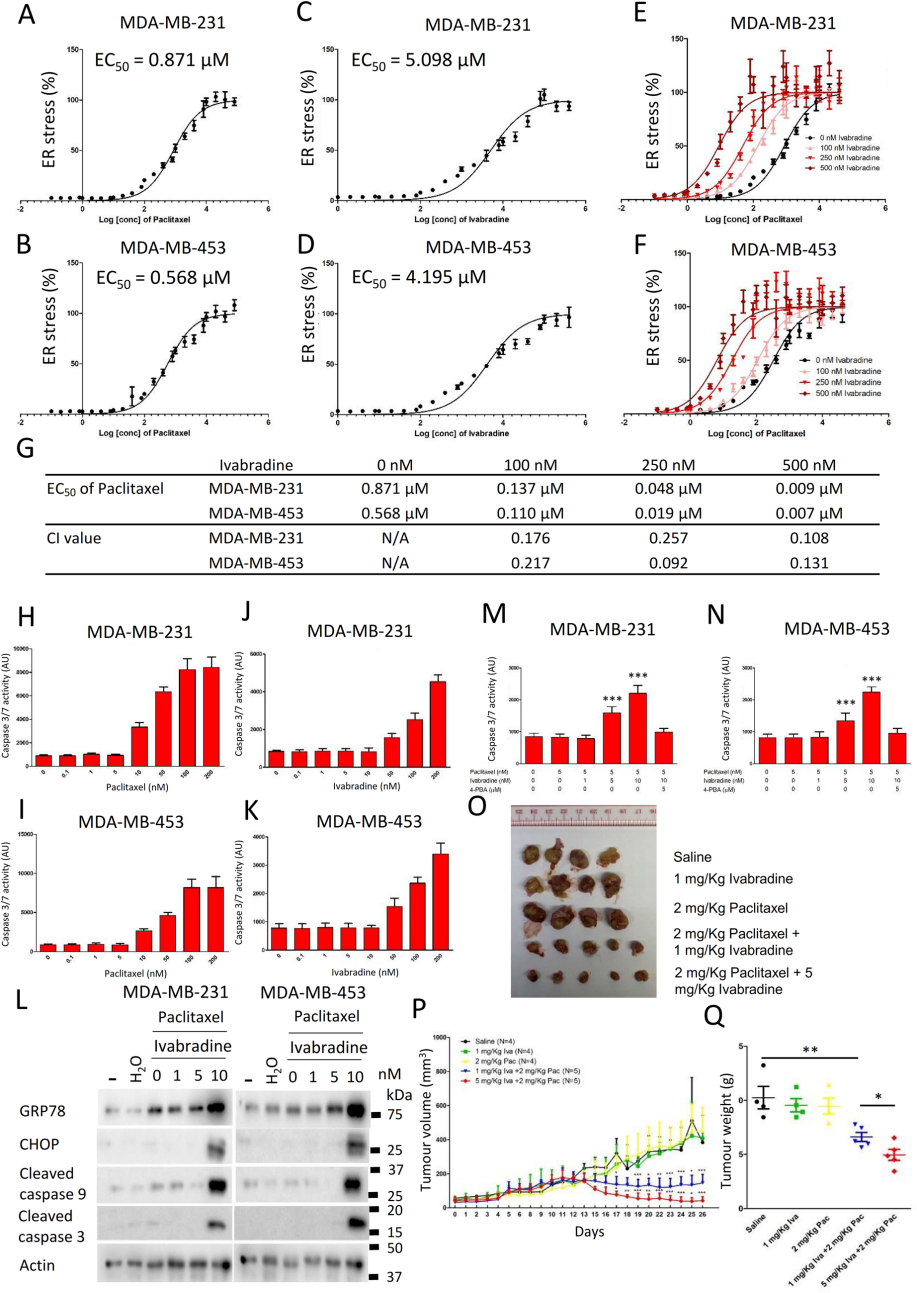
The synergistic effect of Ivabradine with paclitaxel. The curve showed the effect of paclitaxel on ER‐stress in A, MDA‐MB‐231 and B, MDA‐MB‐453. The curve showed the effect of Ivabradine on ER stress in C, MDA‐MB‐231 and D, MDA‐MB‐453. The effect of the addition of 100, 250 and 500 nM of Ivabradine on ER‐stress in E, MDA‐MB‐231 and F, MDA‐MB‐453 treated with paclitaxel. ER‐stress was determined using luciferase reporter with ATF4 response element. The assay was performed 24 h post drug treatment. Results were shown as mean ± SD from six independent experiments. G, A table showed the EC_50_ of paclitaxel with or without Ivabradine. Combination Index (CI) was calculated. The dose‐dependent effect of paclitaxel on caspase activities in H, MDA‐MB‐231 and I, MDA‐MB‐453. The dose‐dependent effect of Ivabradine on caspase activities in J, MDA‐MB‐231 and K, MDA‐MB‐453. Results were shown as mean ± SD from six independent experiments. One‐way ANOVA was used. Bonferroni was used as post‐test of one‐way ANOVA to determine the statistical significance between the control (0 nM) and the treatment groups. *** represents *P* < .001. L, Cotreatment of Ivabradine and paclitaxel could enhance ER‐stress and activate caspases in MDA‐MB‐231 and MDA‐MB‐453. Western blot was employed to determine the expression of the indicated candidate proteins. The addition of 5 μM of 4‐PBA could abolish the effect of Ivabradine and paclitaxel cotreatment on caspases activation in M, MDA‐MB‐231 and N, MDA‐MB‐453. The assay was performed after 48 h of the drug treatment. Results were shown as mean ± SD from three independent experiments. One‐way ANOVA was used. Bonferroni was used as post‐test of one‐way ANOVA to determine the statistical significance between untreated and the treatment groups. *** represents *P* < .001. O, The tumor‐suppressive effect of Ivabradine and paclitaxel cotreatment. MDA‐MB‐231 was employed to establish the xenograft on nude mice. Ivabradine and paclitaxel were administered subcutaneously twice a week for 4 weeks. The images showed the tumours on the mice and isolated tumours. P, Ivabradine and paclitaxel cotreatment reduced tumor weight. Each dot represents one tumor in the group. Results were shown as mean ± SD. Two‐way ANOVA revealed there was a statistically significant interaction between the effects of treatment methods and length (F = 7.45, *P* < .001). Bonferroni test was used as post‐test of two‐way ANOVA to determine the statistical significance between saline and treatment group at each of the time point. *, ** and *** represent *P* < .05, *P* < .01 and *P* < .001 respectively. Q, The effect of Ivabradine and paclitaxel co‐treatment on tumor growth. Results were shown as mean ± SD. One‐way ANOVA was used. Bonferroni test was used as post‐test of one‐way ANOVA to determine the statistical significance between saline and treatment group at each of the time point. * and **represent *P* < .05 and *P* < .01, respectively

We determined the value of EC_50_ of paclitaxel (Figure 7A and B) and Ivabradine (Figure 7C and D) with the use of ER‐stress luciferase reporter assay. In addition, we evaluated the effect of the addition of Ivabradine on ER‐stress mediated by paclitaxel. The results showed that the addition of Ivabradine made a left shift to the response curve (Figure 7E and F). EC_50_ of paclitaxel at different concentrations of Ivabradine was determined (Figure 7G), with values decreasing with increasing Ivabradine concentrations. To determine whether or not there was synergistic effect, the concentrations of combined treatment that result in 50% reduction in ER‐stress response were next calculated to give corresponding CI values. The CI values of Ivabradine and Paclitaxel were less than 1 (CI < 1), supportive that combining Ivabradine and Paclitaxel has indeed a synergistic effect on inducing ER‐stress.

We determined the dose‐dependent effect of paclitaxel and Ivabradine on caspase activities. The results show that 5 nM of paclitaxel (Figure 7H and I) and 10 nM of Ivabradine (Figure 7J and K) were the highest concentration of the drugs that would not activate caspases. While 5 nM of paclitaxel alone could not activate caspase 9 and caspase 3, 5 nM of paclitaxel together with 10 nM of Ivabradine could significantly induce ER‐stress and activate caspases (Figure 7L). The addition of 4‐PBA could abolish the effect of paclitaxel and Ivabradine on the activities of caspases (Figure 7M and N), suggesting that the activation of caspases in the cancer cells treated with paclitaxel and Ivabradine was mediated by ER‐stress.

Consistently in the xenograft model, a low dose of Ivabradine (1 mg/kg) or paclitaxel (2 mg/kg) alone, given twice weekly, did not affect tumour volume (Figure 7P) or did not affect the expression of HCN2 and HCN3 (Supporting information Figure S16). However, synergistically, they could abort tumour growth (Figure 7O‐Q). Our findings, therefore, highlight that cotreatment of Ivabradine and paclitaxel could be a possible treatment strategy for TNBC.

## 4 DISCUSSION

Breast cancer is the most common female cancer worldwide.^24^ Up to 10–15% of breast cancers are triple‐negative, lacking targetable proteins for treatment.^25^ TNBC have aggressive clinical behavior^26^ with cytotoxic chemotherapy being the mainstay of treatment for these patients. In this study, we present an attractive novel molecular target in breast cancer, with a focus on TNBC, and demonstrate that targeting HCN channels could significantly suppress breast cancer growth. More importantly, we found a significant synergistic effect of Ivabradine with paclitaxel in vitro and in vivo, providing preclinical evidence for the combination treatment.

We examined the expression levels of the four HCN isoforms (HCN1‐4) using cell lines from publicly available TCGA database, and on breast cancer patient biopsies by IHC and found that only HCN2 and HCN3 are overexpressed in breast cancer cells (Figure 1A‐D and Supporting information Figure S1 and S2). We inferred that HCN overexpression could be oncogenic, hence, tested the effects of HCN inhibition by shRNA (Figure 2A‐F) or by chemical inhibitor, Ivabradine (Figure 2G‐I and 3). We showed that Ivabradine could reduce cell proliferation and induce cell apoptosis in cancer cells, (Figure 2G‐I, 3A‐D and Supporting information Figure S4). This effect of Ivabradine could be replicated with the use of shHCN cell lines both in vitro (Figure 2E and F; 6D‐H) and in vivo (Supporting information Figure S6), which excludes the possibility of non‐specific Ivabradine cytotoxicity.

Comparing the efficacy of Ivabradine and paclitaxel in inhibiting cancer growth in the cell lines, we confirmed that, while as effective as paclitaxel in the TNBC cell lines, Ivabradine does not affect MCF‐10A, demonstrating its superiority over conventional chemotherapy (Supporting information Figure S15). Using TNBC PDTX models to demonstrate the efficacy of Ivabradine, significant tumour suppression was demonstrated in PDTX models that expressed high HCN2 and HCN3 (Figure 3E‐G). PDTX models, being constructed from patients’ tumour tissues, more faithfully resemble the original tumors,^27^ suggesting a similar response can be expected in breast cancer patients. HCN4 is the major isoform that controls heart rate, as it is the dominant isoform in the sinoatrial node and other parts of the cardiac conducting system.^1^, ^28^ Based on our results, only HCN2 and HCN3 are overexpressed in breast cancer. Therefore, the future effort can be made to develop a drug that recognizes explicitly HCN2 and HCN3 only, which would be expected to be more cancer‐specific than Ivabradine.

Our transcriptome‐wide analysis showed that among the pathways found to be enriched in Ivabradine‐treated TNBC cells, were the cholesterol metabolism, lipid metabolism and cholesterol biosynthesis pathways associated with ER‐stress and two other pathways associated with apoptosis signature (Figure 4C and D). Luciferase reporter assay confirmed that ATF4 activity was enhanced by Ivabradine (Figure 5B). ATF4 is a transcription factor that regulates lipid metabolism, including sterol biosynthesis, and its activity is enhanced under ER‐stress.^29^ ER‐stress is reported to modulate lipid metabolism by altering gene expression.^18^ Through GSEA, we found that Ivabradine could downregulate Ca^2+^ signalling pathway (Figure 4E, Supporting information Table S4) and upregulate ATF6‐mediated unfold protein response and apoptotic signalling pathway in response to ER‐stress (Figure 4F, Supporting information Table S6). Thus, enrichment for the pathways observed in Ivabradine‐treated cells reflects the induction of ER‐stress (Figure 5C). Since HCN channels are ion channels that are permeable to cations, it is possible that the induction of ER‐stress may be due in part to disturbance of intracellular Ca^2+^ levels. Ca^2+^ is responsible for regulating cellular activities, such as endoplasmic reticulum (ER)‐stress regulation,^30^ and ER‐stress induced cell death has been reported when Ca^2+^ homeostasis is perturbed.^31^ Our results demonstrated that blocking HCNs impaired the Ca^2+^ homeostasis in cancer cells that overexpress HCN2 and HCN3, resulting in lower levels of intracellular Ca^2+^ (Figure 5A) in the cells, which could trigger ER‐stress response (Figure 5B‐D) and subsequently commit cells to apoptosis. HCN2 has been shown to be expressed in non‐small‐cell lung carcinoma, and patch‐clamp experiments showed hyperpolarization of the cancer cells on exposure to proapoptotic trigger.^11^ Moreover, it demonstrated that HCN2 was necessary for influx of Ca^2+^ into cells. This supports our hypothesis that HCN channels when overexpressed in breast cancer cells may be functional and with an oncogenic effect. Further in vivo support of this oncogenic effect is reflected in our HCN2 and HCN3 expression in over 200 primary breast cancers which showed a significant correlation with poorer survival. (Figure 1E‐G; Table 1).

Apoptotic stimulus can alter ionic gradients across the plasma membrane leading to depolarization of the plasma membrane potential.^32^ It is not surprising that cancer cells have developed strategies such as overexpression of ion‐channels to counteract apoptotic stimulus by inducing hyperpolarization.^33^ Ion‐channels are located on the plasma membrane, some overexpressed in cancer cells, and were once considered ideal drug targets since drugs, exerting their effect in the extracellular space.^34^ Targeting various ion‐channels overexpressed in different cancer types have been reported to exert antitumor effect.^35^ Inhibition of voltage‐gated sodium (Na^+^) channels have been shown to suppress tumour metastasis,^36^ while inhibition of voltage‐gated calcium ion (Ca^2+^) and potassium ion (K^+^) channels have been reported to cause reduction of breast tumour size.^37^, ^38^ However, these drugs were toxic to non‐malignant tissues. Cardiac glycosides which inhibit the Na^+^/K^+^‐ATPase pump while exerting antitumor effects,^39^, ^40^, ^41^ demonstrate higher toxicity to normal breast epithelial cells than to breast cancer cells.^42^ In contrast, targeting HCN channels appeared to have minimal effect on normal breast epithelia (Figure 2G, H; 5A‐C; 5F; 6F; Supporting information Figure S15A‐D).

Apart from HCN channels, the transient receptor potential (TRP) channels superfamily, such as TRPC1^43^ and TRPC5,^44^ have been shown to be involved in the pathogenesis of breast cancer. Overexpression of various TRP channels has been suggested to associate with poorer outcome of breast cancer.^45^ TRP channels are responsible for mediating the import of calcium ions. Therefore, targeting TRP channels could be an alternative for targeting calcium signalling to compromise the survival advantage of breast cancer cells. Similarly, cyclic nucleotide‐gated (CNG) channels mediate the influx of calcium and sodium ions through messengers cAMP or cGMP.^46^ Activation of CNG channels can trigger various downstream effectors, such as PKA and PKC, in addition to increasing the level of calcium which could activate CaMKII.^47^ Hyperactivation of PKA can promote tumorigenesis in mammary tissue.^48^ Activation of CaMKII plays essential roles in the proliferation, differentiation and survival of various cancer cells.^49^ Therefore, targeting CNG channels should be able to suppress the growth of breast tumours.

We established the minimal subcutaneous dosage of 5 mg/kg Ivabradine twice weekly (human‐equivalent dosage [HED] 48.78 mg/week (Supporting information Table S8),^50^ sufficient to inhibit tumour growth (Figure 3H), which is below the maximum daily dosage of 15 mg allowed for humans. More importantly, we found a profound synergistic effect of Ivabradine with paclitaxel, the combination of both drugs at low doses resulting in tumour regression in vivo (Figure 7O‐Q). Paclitaxel inhibits cancer cell proliferation by disturbing the formation of microtubules. Such an effect can act on both normal and cancerous cells, with side‐effects being a common problem. While low‐dose paclitaxel might not effectively eliminate cancer cells, the addition of Ivabradine, can enable both drugs to act synergistically. We believe this synergistic effect is due to ER‐stress which serves as a common hub for Ivabradine and paclitaxel to amplify signals to activate caspase, thus, initiating cell death at much lower dosages when using both drugs together.

When combined with Ivabradine, the effective dose of paclitaxel is 2 mg/kg in mice (HED 19.5 mg/week),^50^ 27‐fold less than the recommended dose (Supporting information Table S8) used clinically to treat TNBC. Likewise, the effective dose of Ivabradine is reduced to 1 mg/kg in mice, given twice weekly (HED 9.76 mg/week) (Supporting information Table S8), which more than 10‐fold less the maximum allowed human dosage of 105 mg per week. Thus, combination treatment regimen besides being effective, could minimize systemic toxicity to patients. Promising future work remains to determine whether this combination therapy may also be effective for other cancer types.

Recent evidence has suggested HCN channels as pharmacological targets for analgesia.^51^ Indeed HCN channel inhibitors, Ivabradine and Gabapentin, both demonstrate the anti‐nociceptive effect. Given the known side‐effect of paclitaxel of chemotherapy‐induced peripheral neuropathy,^52^ Ivabradine given in combination with paclitaxel might help counteract the neuropathic pain induced by paclitaxel.

In summary, our findings provide new insight into the treatment of cancer, particularly TNBC. Given that Ivabradine is the only FDA‐approved inhibitor of HCN channels, its repurposing provides a fast‐track drug development route.

## CONFLICT OF INTEREST

The authors declare no conflict of interest. Patent Cooperation Treaty (PTC/CN2018/084417) “Use of HCN Inhibitors for Treatment of Cancer” Filed April 2018.

## AUTHORS’ CONTRIBUTIONS

Conception and design: C. Gong, U. S. Khoo.

Development of methodology: K. C. Mok, H. Tsoi, C. Gong, E. P. S. Man, T. K. W. Lee, U. S. Khoo Acquisition of data (provided animals, acquired and managed patients, provided facilities, etc.). K. C. Mok, H. Tsoi, E. P. S. Man, M. H. Leung, K. M. Chau, J. Chan, J. Leung, Y. H. Y. Chan, L. S. Wong, W. L. Chan, S. Y. Chan, M. Y. Luk, S. Batalha, V. Lau, D. C. W. Siu, U. S. Khoo.

Analysis and interpretation of data (e.g., statistical analysis, biostatistics, computational analysis): K. C. Mok, H. Tsoi, C. Gong, E. P. S. Man, M. H. Leung, T. K. W. Lee, U. S. Khoo.

Writing, review and/or revision of the manuscript: K.C. Mok, H. Tsoi, C. Gong, T. K. W.Lee, U. S. Khoo.

Administrative, technical, or material support (i.e.,reporting or organising data, constructing databases): H. Tsoi, E. P. S. Man, U. S. Khoo.

Study supervision: H. Tsoi, C. Gong, U .S. Khoo.

## AVAILABILITY OF DATA AND MATERIAL

The datasets and materials generated in this study are available from the corresponding author on reasonable request.

## Supporting information



Figures

Supplementry information

TableS3

TableS5

TableS3
