# Supplementary material for: Repurposing hyperpolarization‐activated cyclic nucleotide‐gated channels as a novel therapy for breast cancer
Source: Clin Transl Med. 2021 Nov 4;11(11):e578. doi: 10.1002/ctm2.578 (PMC8567035; doi:10.1002/ctm2.578)

FIGURE S1

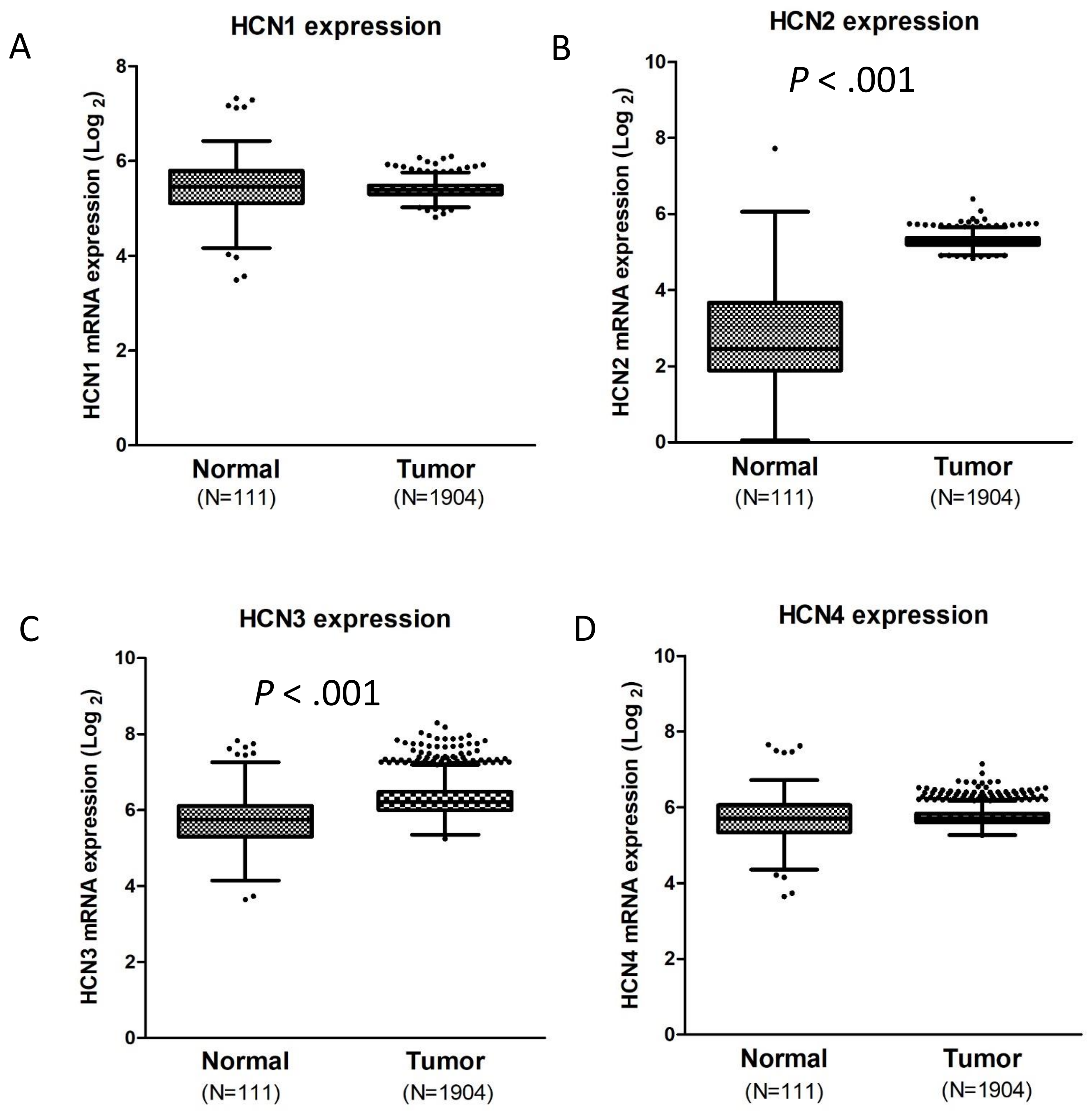
A
HCN1 expression
HCN1 mRNA expression (Log 2)
0
2
4
6
8
Normal
(N=111)
Tumor
(N=1904)
B
HCN2 expression
P < .001
HCN2 mRNA expression (Log 2)
0
2
4
6
8
10
Normal
(N=111)
Tumor
(N=1904)
C
HCN3 expression
P < .001
HCN3 mRNA expression (Log 2)
0
2
4
6
8
10
Normal
(N=111)
Tumor
(N=1904)
D
HCN4 expression
HCN4 mRNA expression (Log 2)
0
2
4
6
8
10
Normal
(N=111)
Tumor
(N=1904)

FIGURE S2

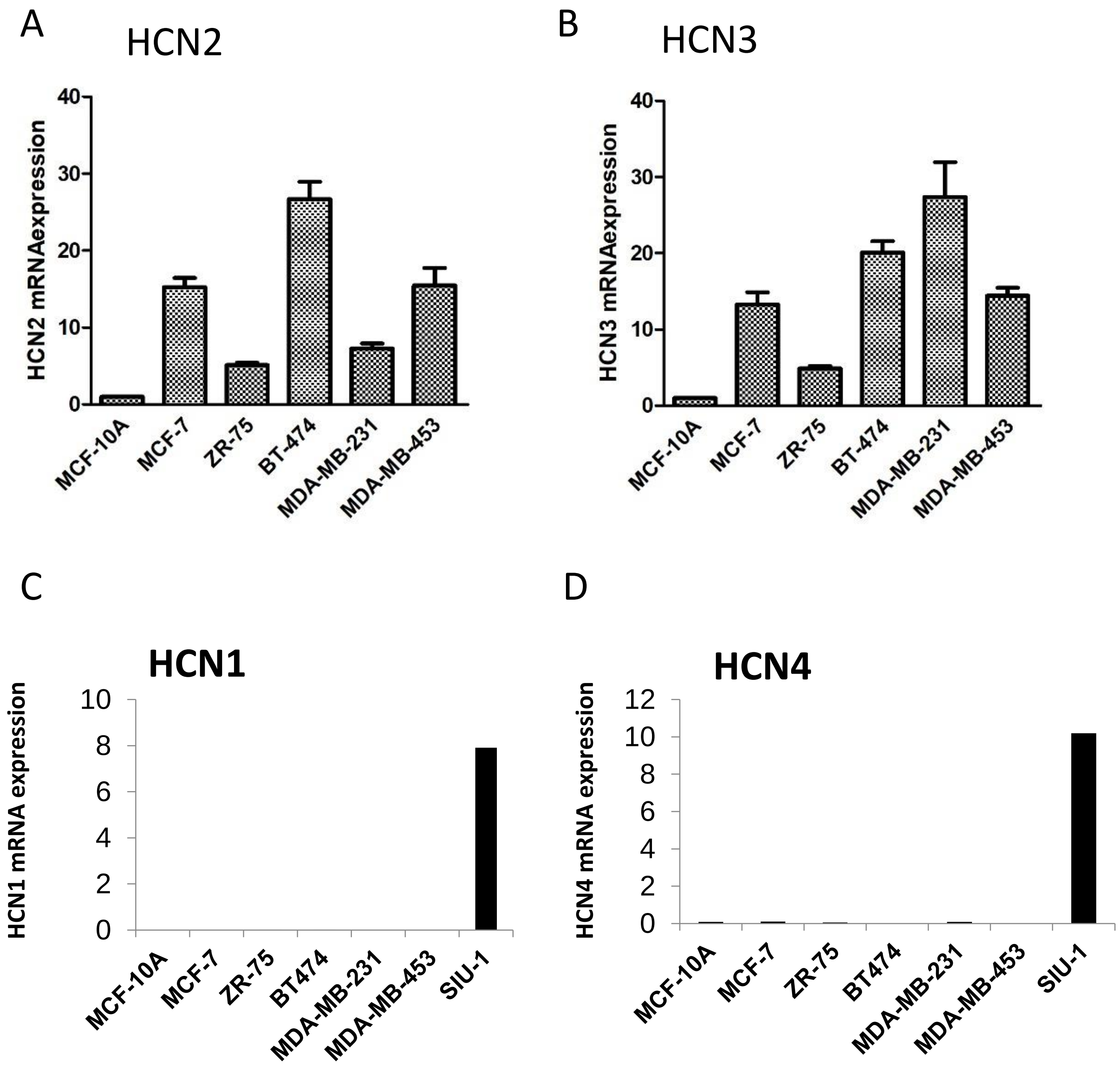
A
HCN2
HCN2 mRNAexpression
0
10
20
30
40
MCF-10A
MCF-7
ZR-75
BT-474
MDA-MB-231
MDA-MB-453
B
HCN3
HCN3 mRNAexpression
0
10
20
30
40
MCF-10A
MCF-7
ZR-75
BT-474
MDA-MB-231
MDA-MB-453
C
HCN1
HCN1 mRNA expression
0
2
4
6
8
10
MCF-10A
MCF-7
ZR-75
BT474
MDA-MB-231
MDA-MB-453
SIU-1
D
HCN4
HCN4 mRNA expression
0
2
4
6
8
10
12
MCF-10A
MCF-7
ZR-75
BT474
MDA-MB-231
MDA-MB-453
SIU-1

FIGURE S3

A

Panel A

MDA-MB-231

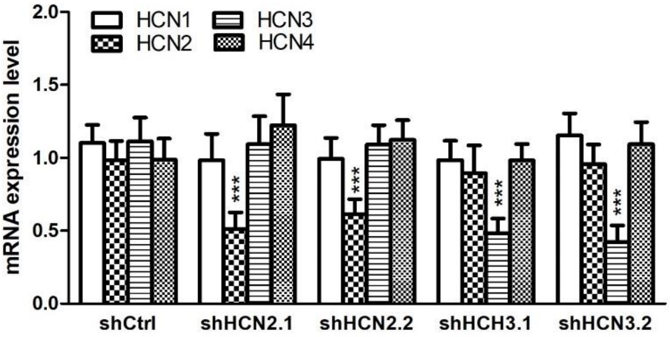

MDA-MB-231

Panel B

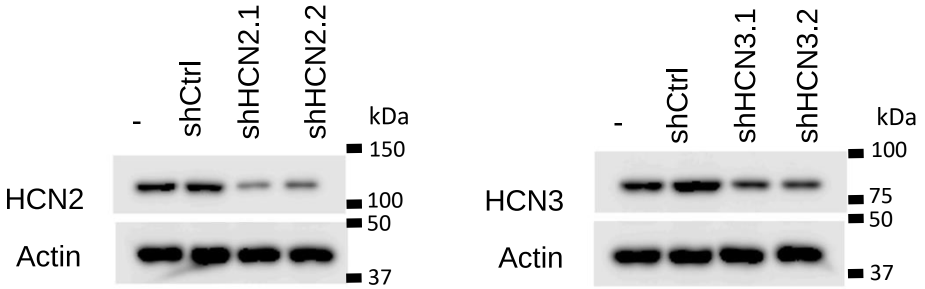

B

Panel A

MDA-MB-453

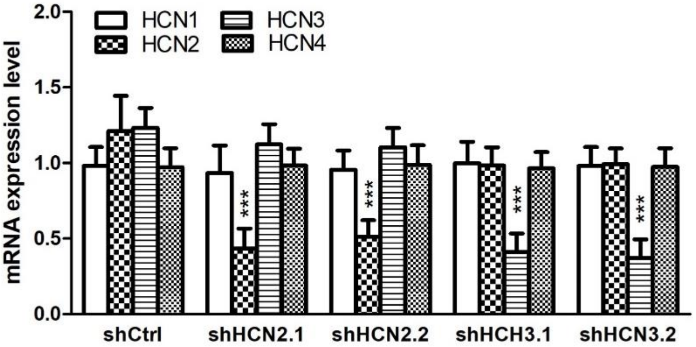

Panel B

MDA-MB-453

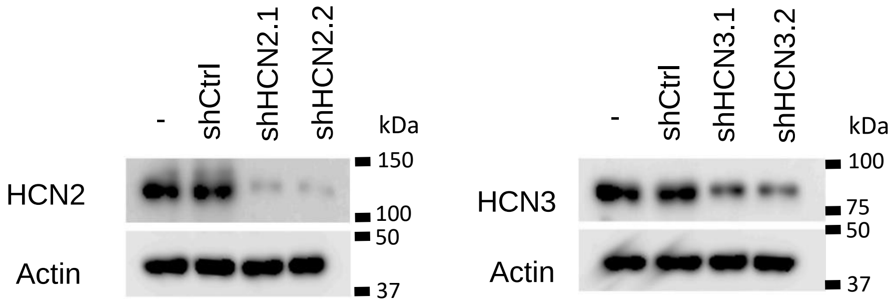

FIGURE S4

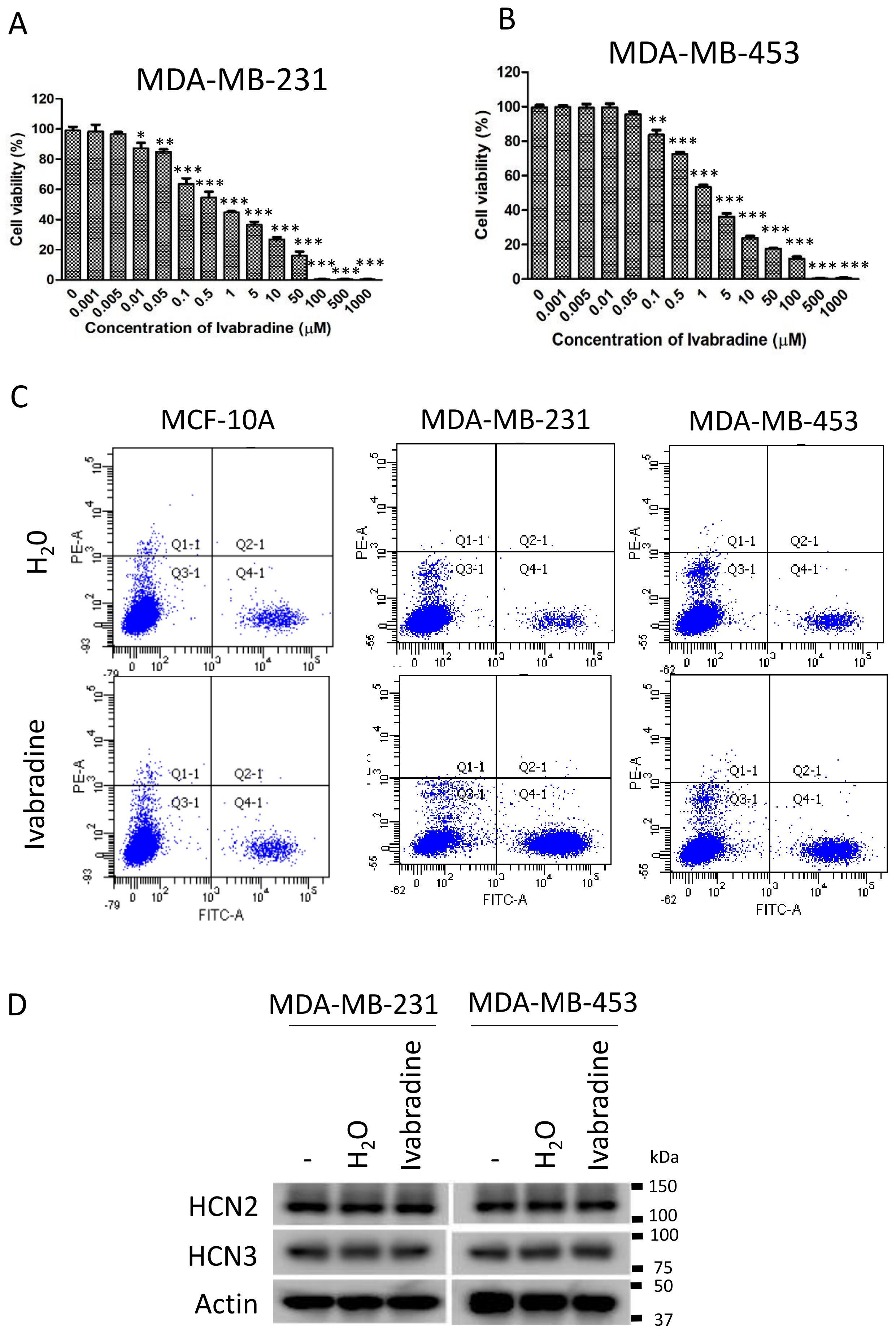

A
MDA-MB-231
Cell viability (%)
Concentration of Ivabradine (μM)
B
MDA-MB-453
Cell viability (%)
Concentration of Ivabradine (μM)
C
MCF-10A
MDA-MB-231
MDA-MB-453
H2O
Ivabradine
PE-A
FITC-A
Q1-1
Q2-1
Q3-1
Q4-1
D
MDA-MB-231
MDA-MB-453
-
H2O
Ivabradine
kDa
HCN2
HCN3
Actin
150
100
100
75
50
37

FIGURE S5

Panel A

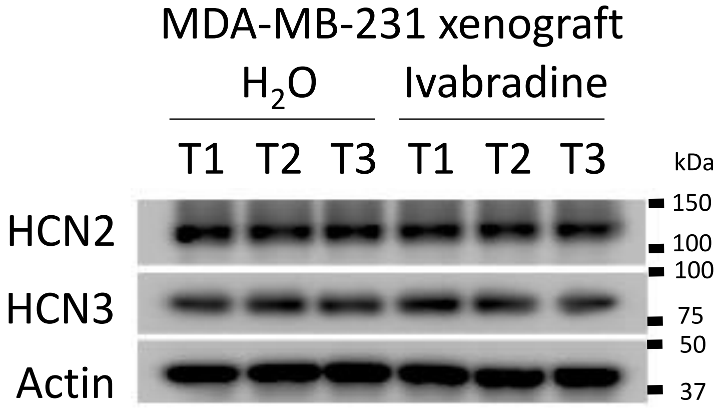

Panel B

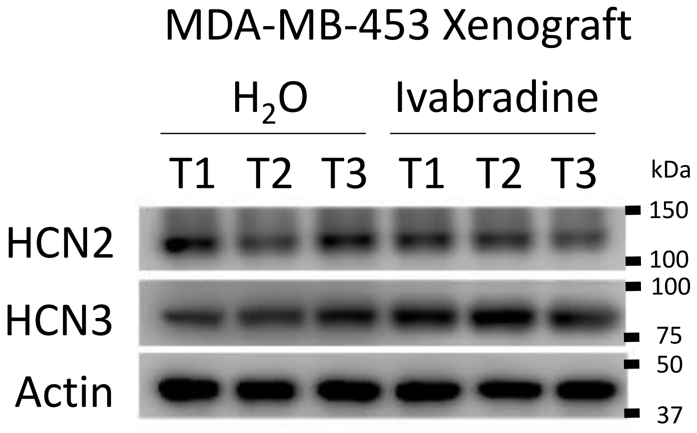

FIGURE S6

A

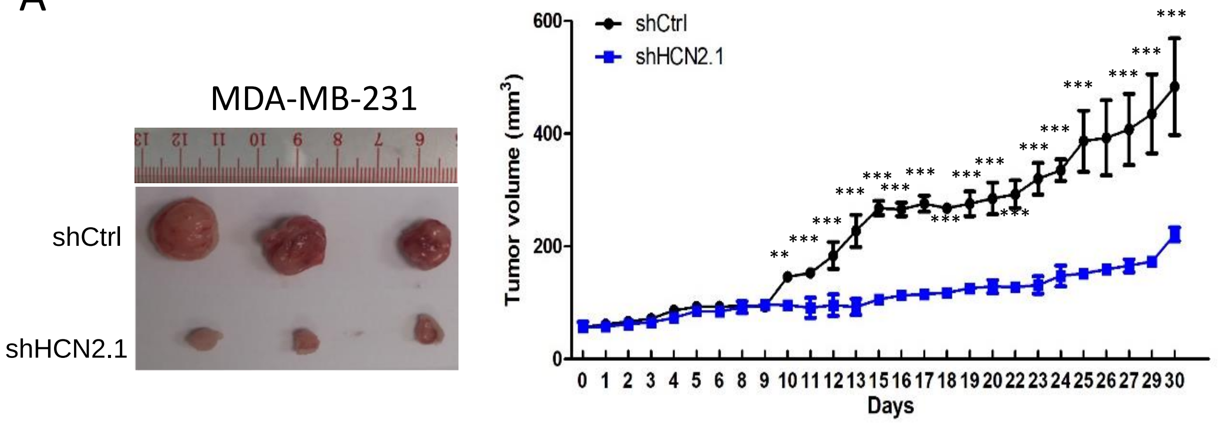
MDA-MB-231
shCtrl
shHCN2.1
shCtrl
shHCN2.1
Tumor volume (mm^3)
Days
0
200
400
600
0 1 2 3 4 5 6 8 9 10 11 12 13 15 16 17 18 19 20 22 23 24 25 26 27 29 30
**
***

B

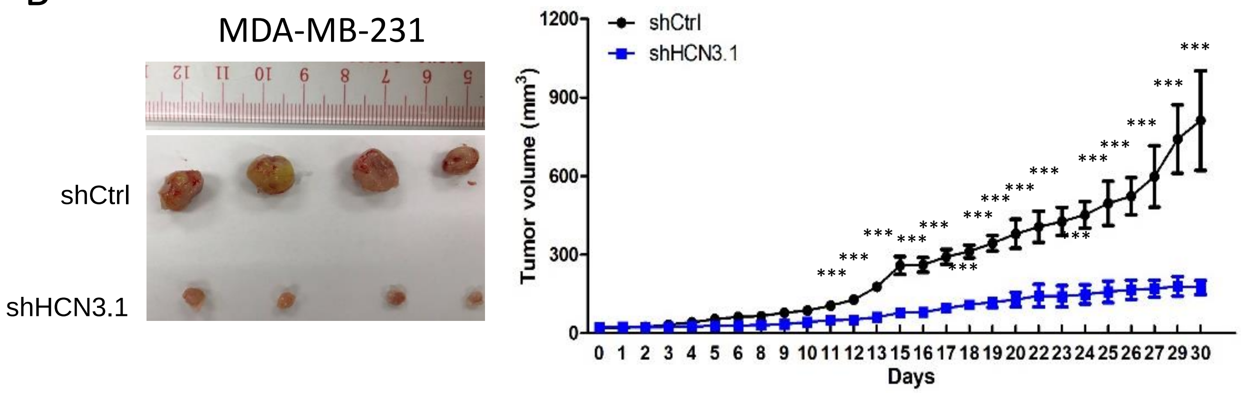
MDA-MB-231
shCtrl
shHCN3.1
shCtrl
shHCN3.1
Tumor volume (mm^3)
Days
0
300
600
900
1200
0 1 2 3 4 5 6 8 9 10 11 12 13 15 16 17 18 19 20 22 23 24 25 26 27 29 30
***

FIGURE S7

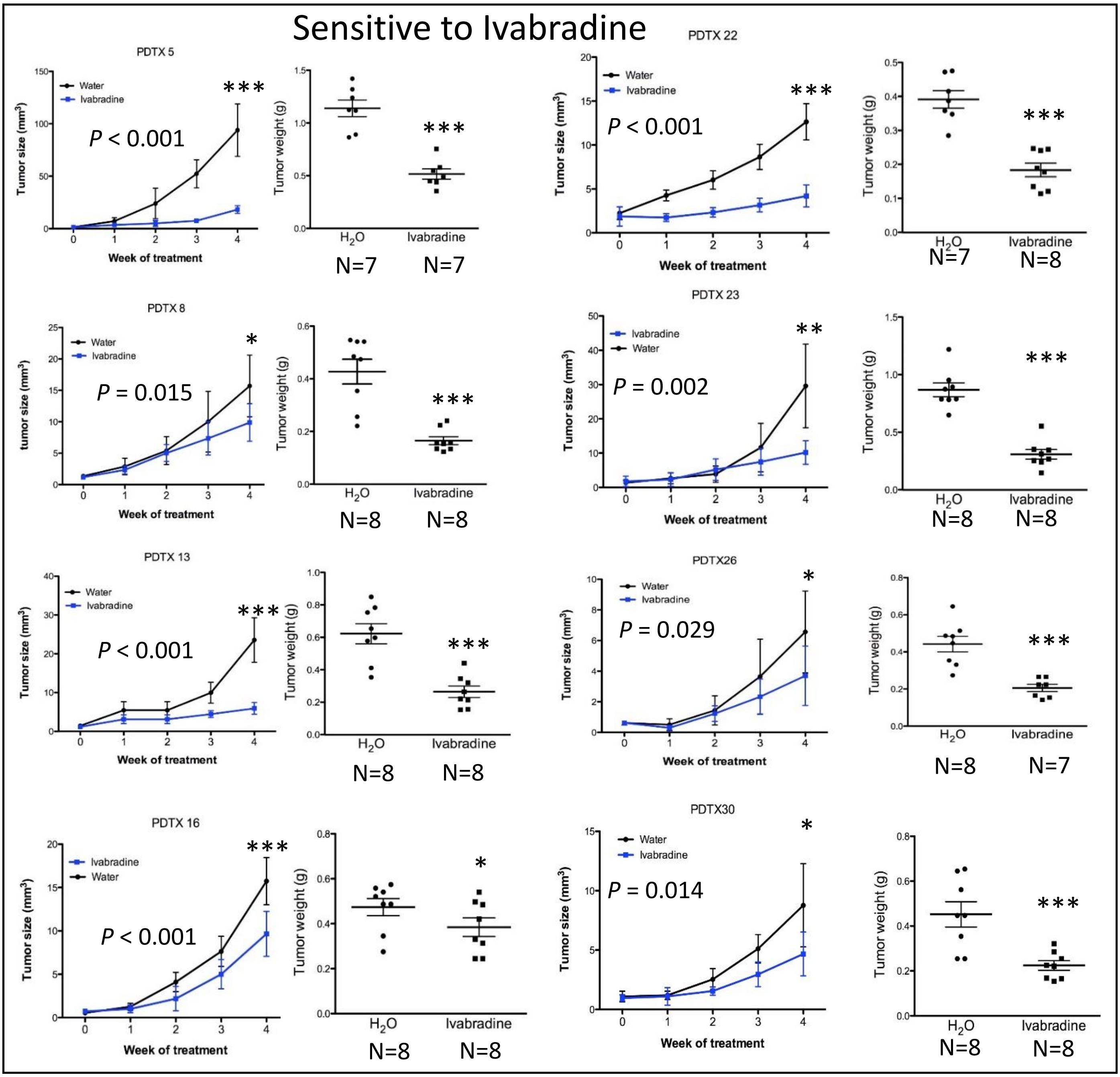
Sensitive to Ivabradine
PDTX 5
Water
Ivabradine
***
P < 0.001
Tumor size (mm3)
Week of treatment
Tumor weight (g)
H2O
Ivabradine
N=7
N=7
PDTX 22
P < 0.001
N=7
N=8
PDTX 8
*
P = 0.015
tumor size (mm3)
N=8
N=8
PDTX 23
**
P = 0.002
N=8
N=8
PDTX 13
P < 0.001
N=8
N=8
PDTX26
P = 0.029
N=8
N=7
PDTX 16
P < 0.001
N=8
N=8
PDTX30
P = 0.014
N=8
N=8

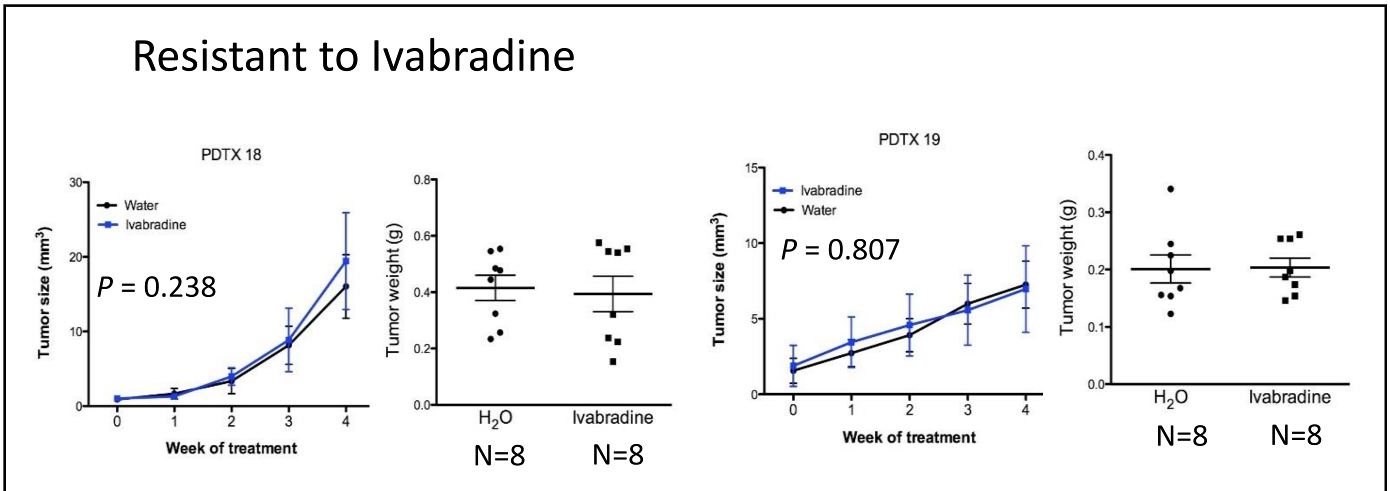
Resistant to Ivabradine
PDTX 18
Water
Ivabradine
P = 0.238
Tumor size (mm3)
Week of treatment
Tumor weight (g)
H2O
Ivabradine
N=8
N=8
PDTX 19
P = 0.807
N=8
N=8

FIGURE S8

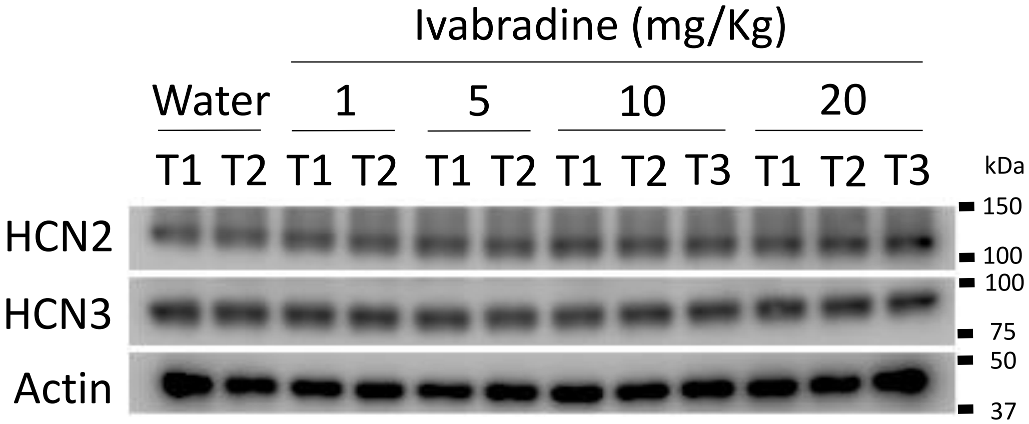

Ivabradine (mg/Kg)
Water
1
5
10
20
T1 T2 T1 T2 T1 T2 T1 T2 T3 T1 T2 T3
kDa
HCN2
HCN3
Actin
150
100
100
75
50
37

FIGURE S9

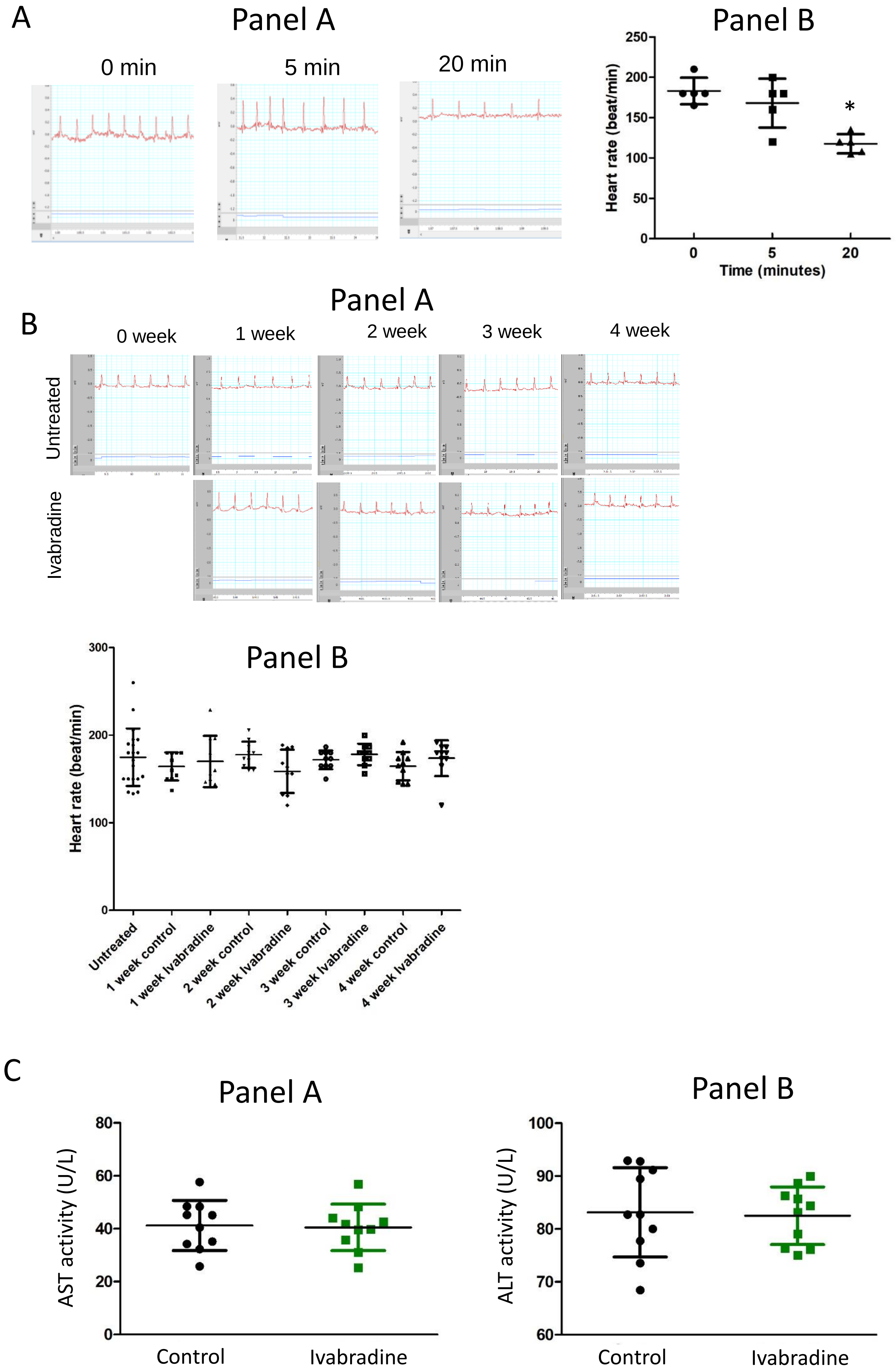

A
Panel A
0 min
5 min
20 min
Panel B
Heart rate (beat/min)
250
200
150
100
50
0
0
5
20
*
Time (minutes)
B
Panel A
0 week
1 week
2 week
3 week
4 week
Untreated
Ivabradine
Panel B
Heart rate (beat/min)
300
200
100
0
Untreated
1 week control
1 week Ivabradine
2 week control
2 week Ivabradine
3 week control
3 week Ivabradine
4 week control
4 week Ivabradine
C
Panel A
AST activity (U/L)
80
60
40
20
0
Control
Ivabradine
Panel B
ALT activity (U/L)
100
90
80
70
60
Control
Ivabradine

FIGURE S10

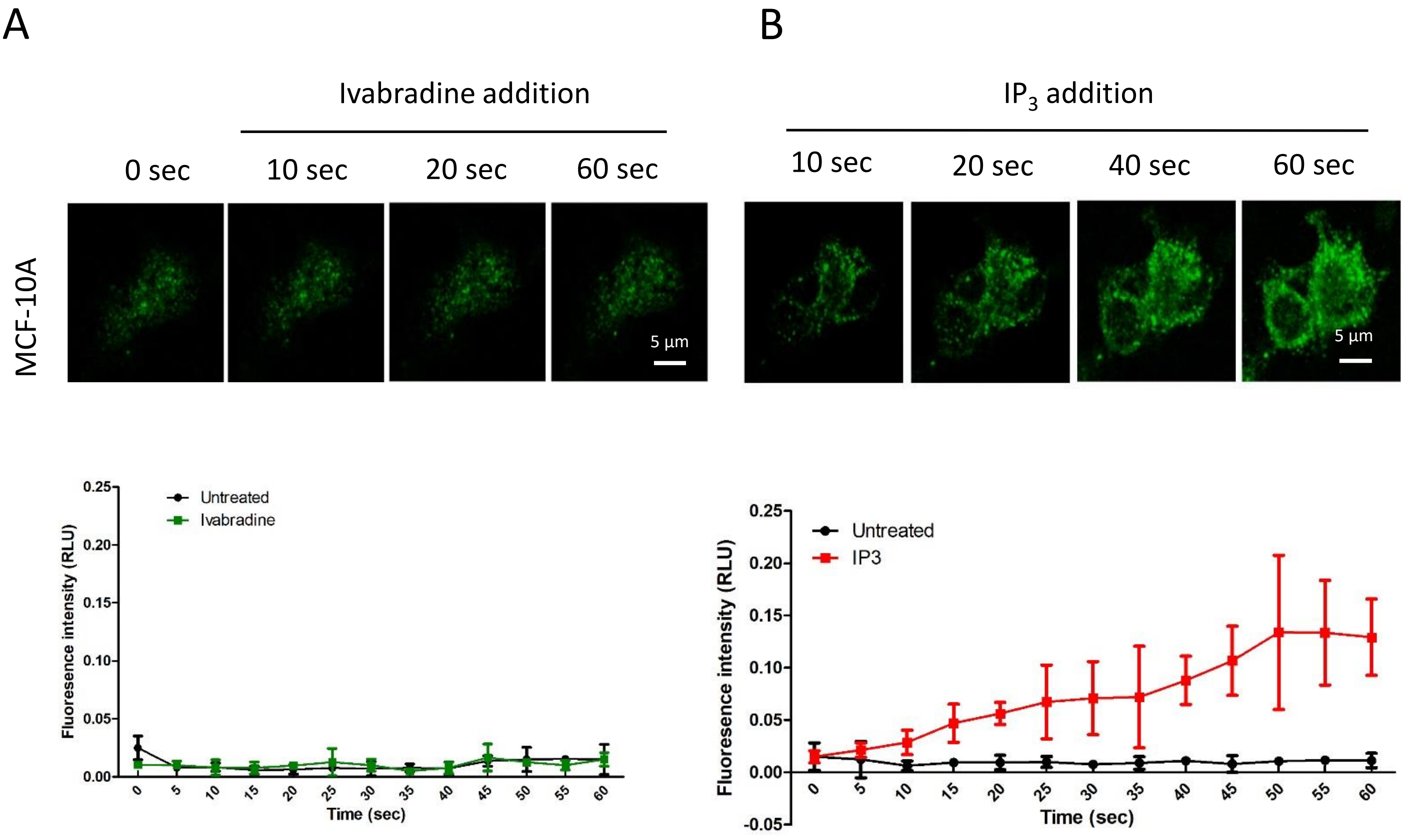
A
B
Ivabradine addition
0 sec
10 sec
20 sec
60 sec
MCF-10A
5 µm
IP3 addition
10 sec
20 sec
40 sec
60 sec
5 µm
Untreated
Ivabradine
Fluoresence intensity (RLU)
Time (sec)
Untreated
IP3
Fluoresence intensity (RLU)
Time (sec)

FIGURE S11

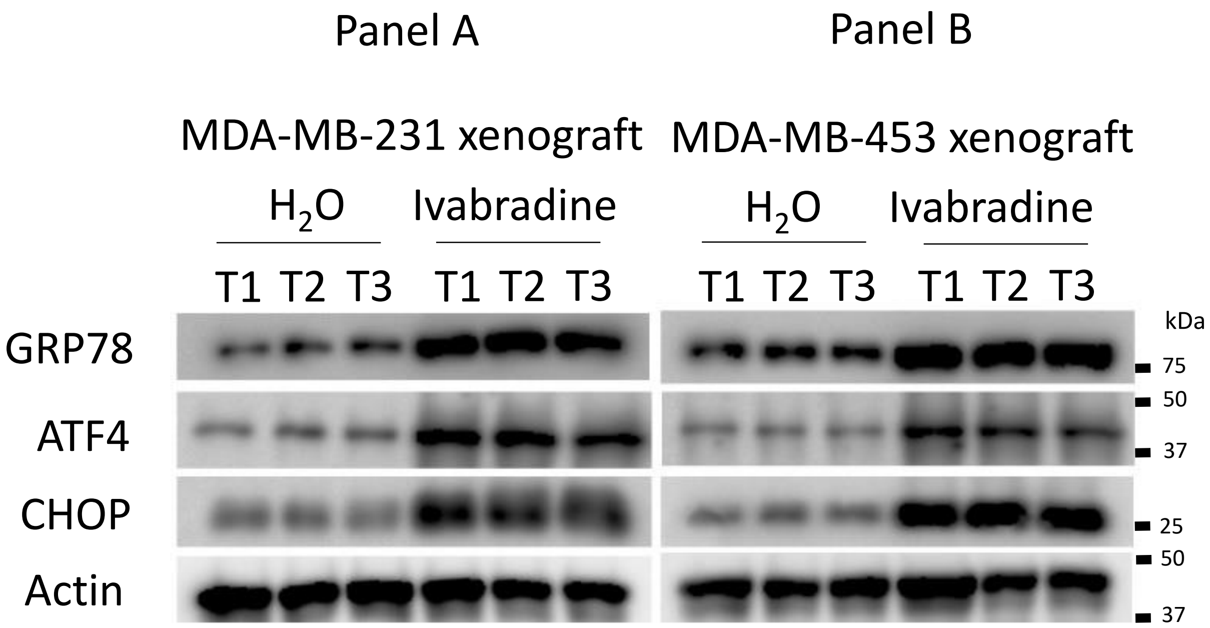

Panel A
Panel B
MDA-MB-231 xenograft
MDA-MB-453 xenograft
H2O
Ivabradine
H2O
Ivabradine
T1 T2 T3 T1 T2 T3
T1 T2 T3 T1 T2 T3
kDa
GRP78
ATF4
CHOP
Actin
75
50
37
25
50
37

FIGURE S12

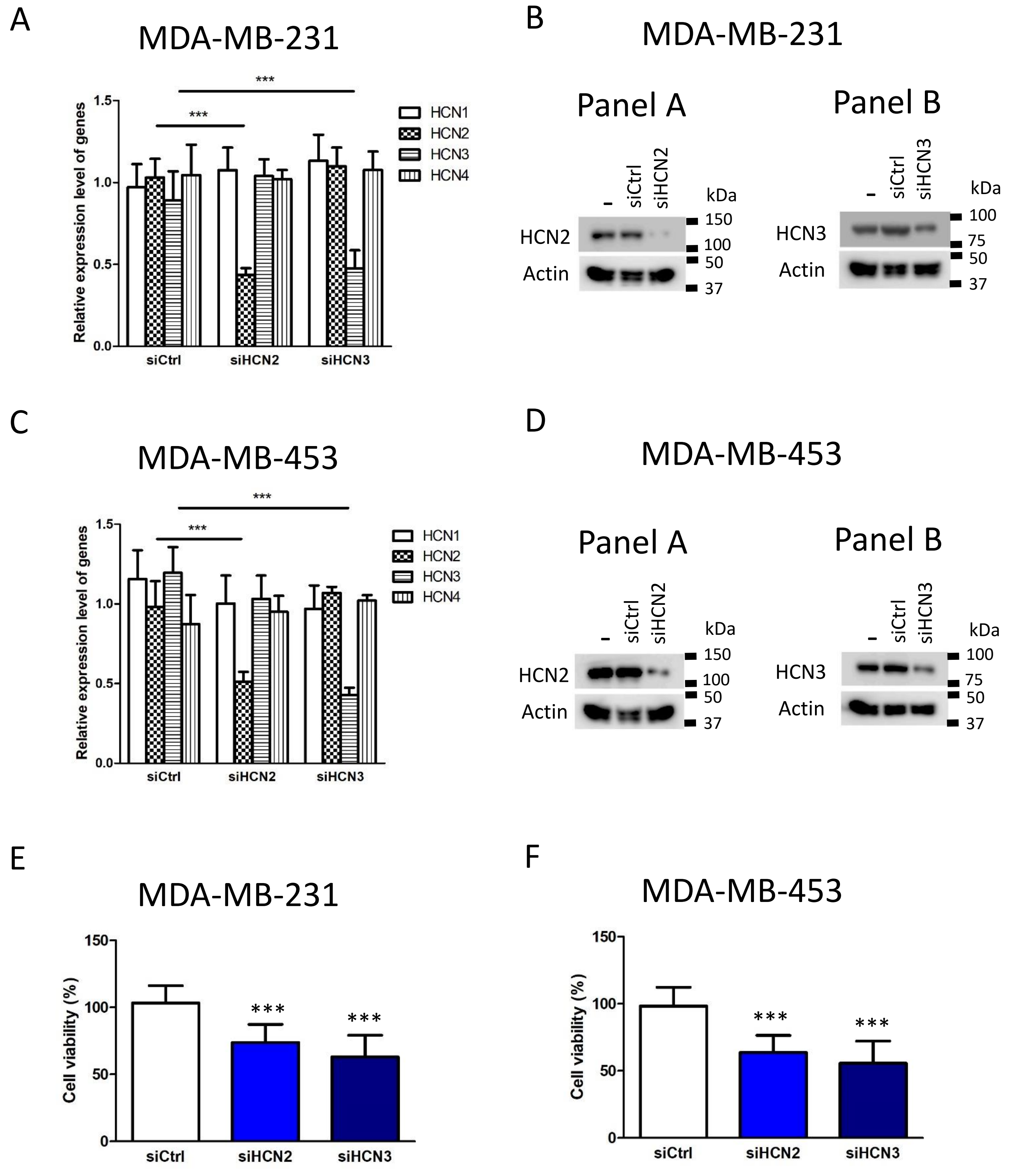
A
MDA-MB-231
***
***
Relative expression level of genes
1.5
1.0
0.5
0.0
HCN1
HCN2
HCN3
HCN4
siCtrl
siHCN2
siHCN3
B
MDA-MB-231
Panel A
Panel B
-
siCtrl
siHCN2
kDa
150
100
50
37
HCN2
Actin
siHCN3
HCN3
75
C
MDA-MB-453
D
MDA-MB-453
E
MDA-MB-231
Cell viability (%)
150
100
50
0
***
F
MDA-MB-453

FIGURE S13

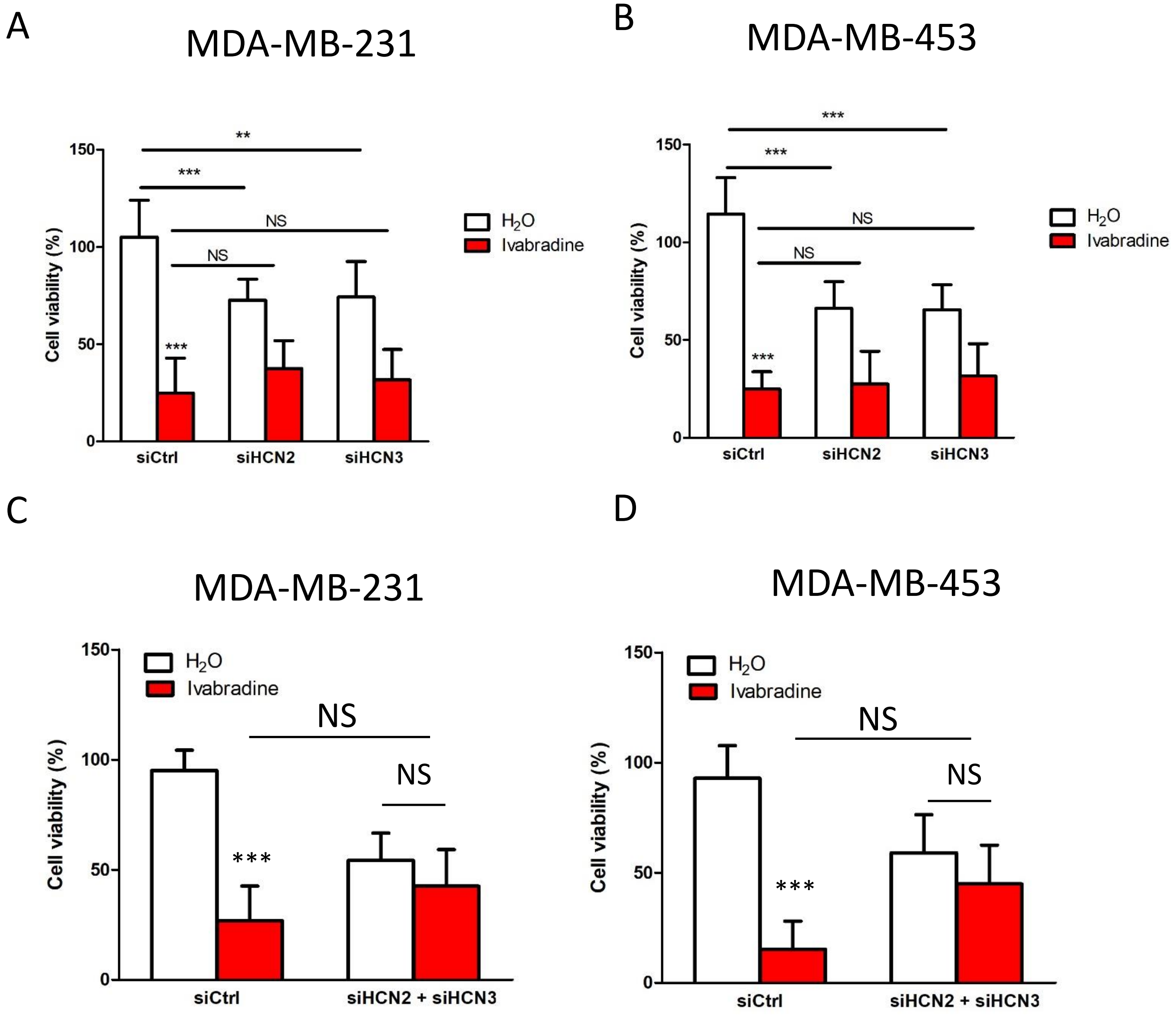
A
MDA-MB-231
150
100
50
0
Cell viability (%)
**
***
NS
NS
***
siCtrl
siHCN2
siHCN3
H2O
Ivabradine
B
MDA-MB-453
150
100
50
0
Cell viability (%)
***
***
NS
NS
***
siCtrl
siHCN2
siHCN3
H2O
Ivabradine
C
MDA-MB-231
150
100
50
0
Cell viability (%)
H2O
Ivabradine
NS
NS
***
siCtrl
siHCN2 + siHCN3
D
MDA-MB-453
150
100
50
0
Cell viability (%)
H2O
Ivabradine
NS
NS
***
siCtrl
siHCN2 + siHCN3

FIGURE S14

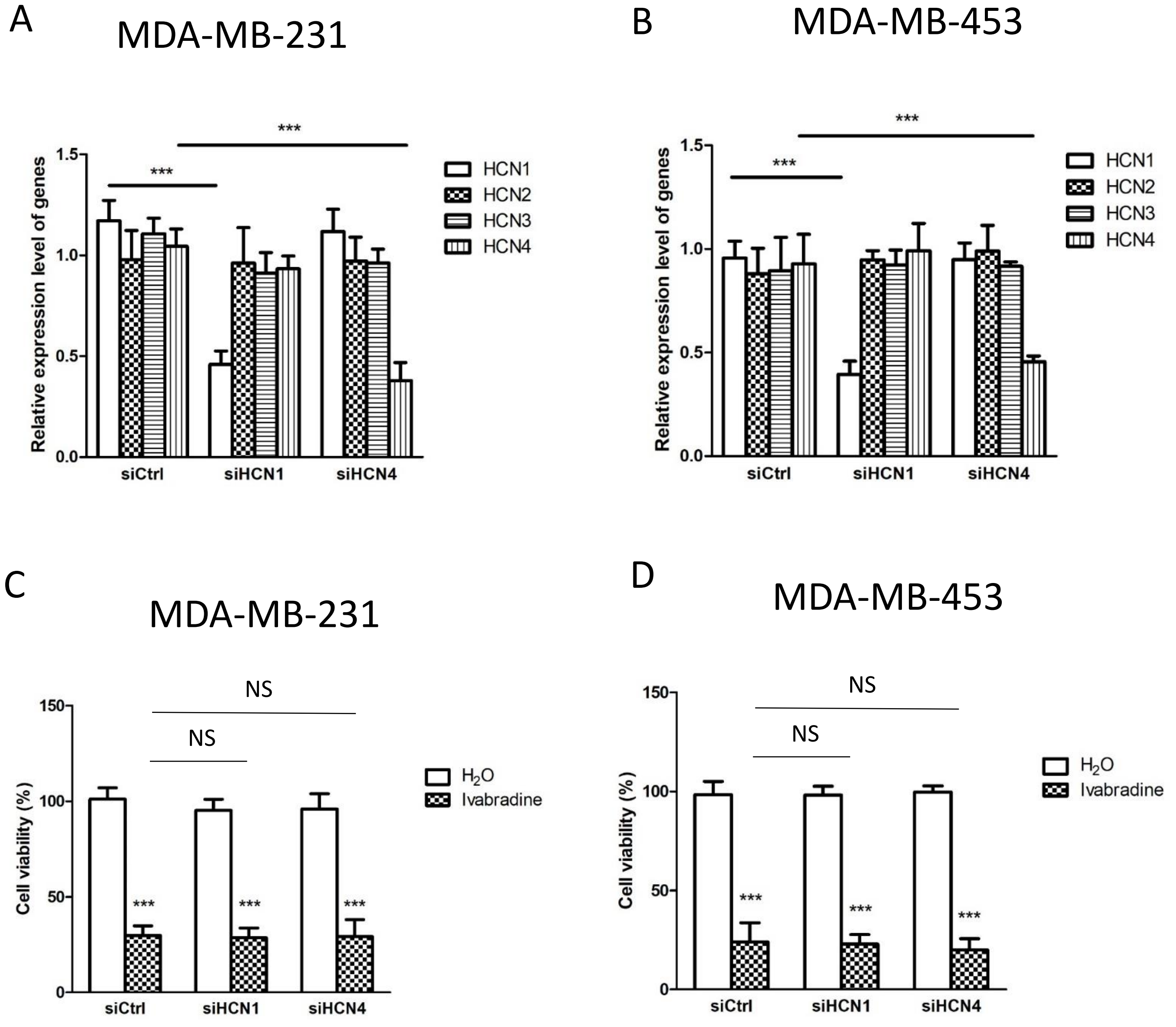

A
MDA-MB-231
***
***
Relative expression level of genes
1.5
1.0
0.5
0.0
siCtrl
siHCN1
siHCN4
HCN1
HCN2
HCN3
HCN4
B
MDA-MB-453
***
***
Relative expression level of genes
1.5
1.0
0.5
0.0
siCtrl
siHCN1
siHCN4
HCN1
HCN2
HCN3
HCN4
C
MDA-MB-231
NS
NS
Cell viability (%)
150
100
50
0
***
***
***
siCtrl
siHCN1
siHCN4
H_2O
Ivabradine
D
MDA-MB-453
NS
NS
Cell viability (%)
150
100
50
0
***
***
***
siCtrl
siHCN1
siHCN4
H_2O
Ivabradine

FIGURE S15

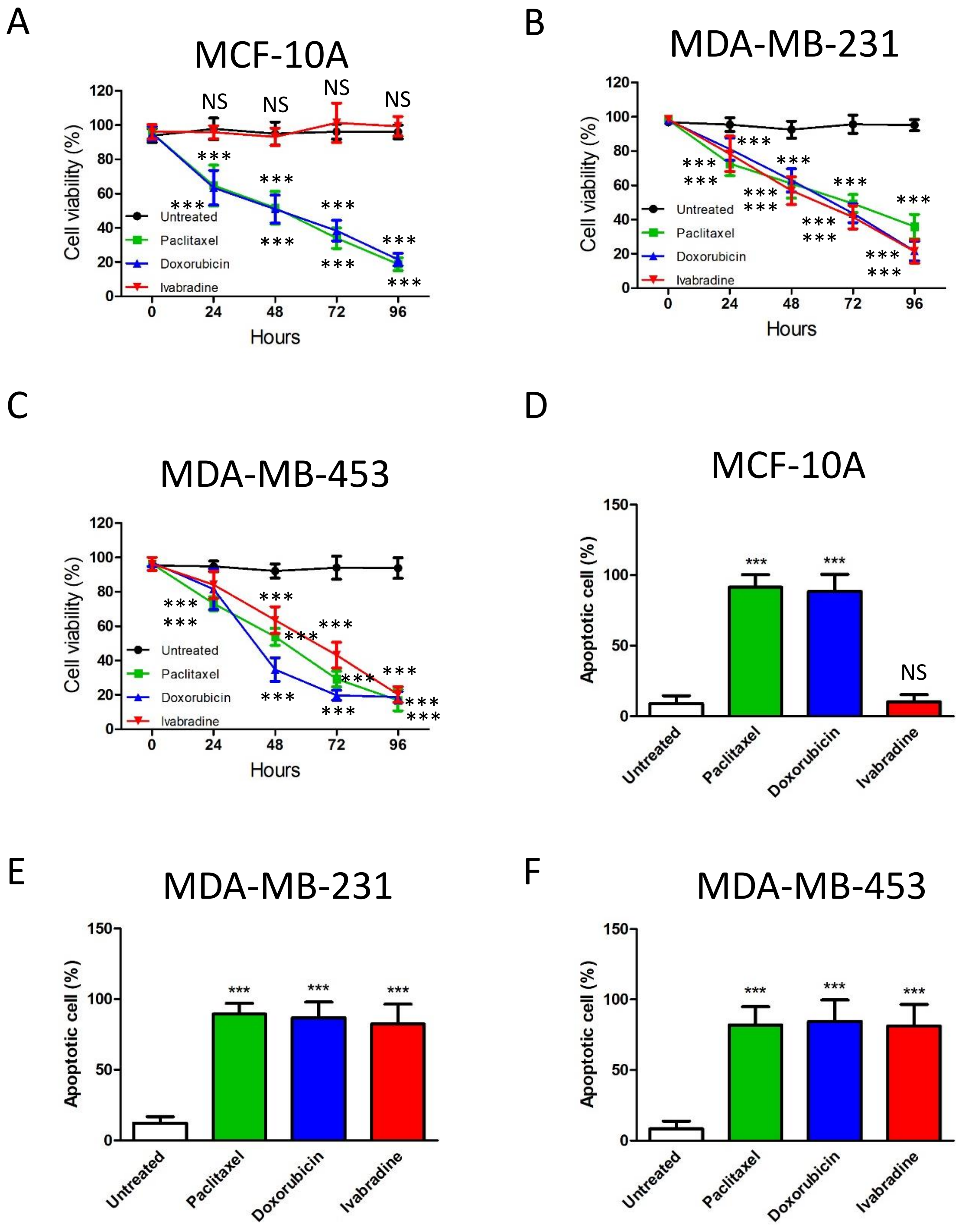
A
MCF-10A
Cell viability (%)
Hours
Untreated
Paclitaxel
Doxorubicin
Ivabradine
NS
B
MDA-MB-231
Cell viability (%)
Hours
Untreated
Paclitaxel
Doxorubicin
Ivabradine
C
MDA-MB-453
Cell viability (%)
Hours
Untreated
Paclitaxel
Doxorubicin
Ivabradine
D
MCF-10A
Apoptotic cell (%)
Untreated
Paclitaxel
Doxorubicin
Ivabradine
NS
E
MDA-MB-231
Apoptotic cell (%)
Untreated
Paclitaxel
Doxorubicin
Ivabradine
F
MDA-MB-453
Apoptotic cell (%)
Untreated
Paclitaxel
Doxorubicin
Ivabradine

FIGURE S16

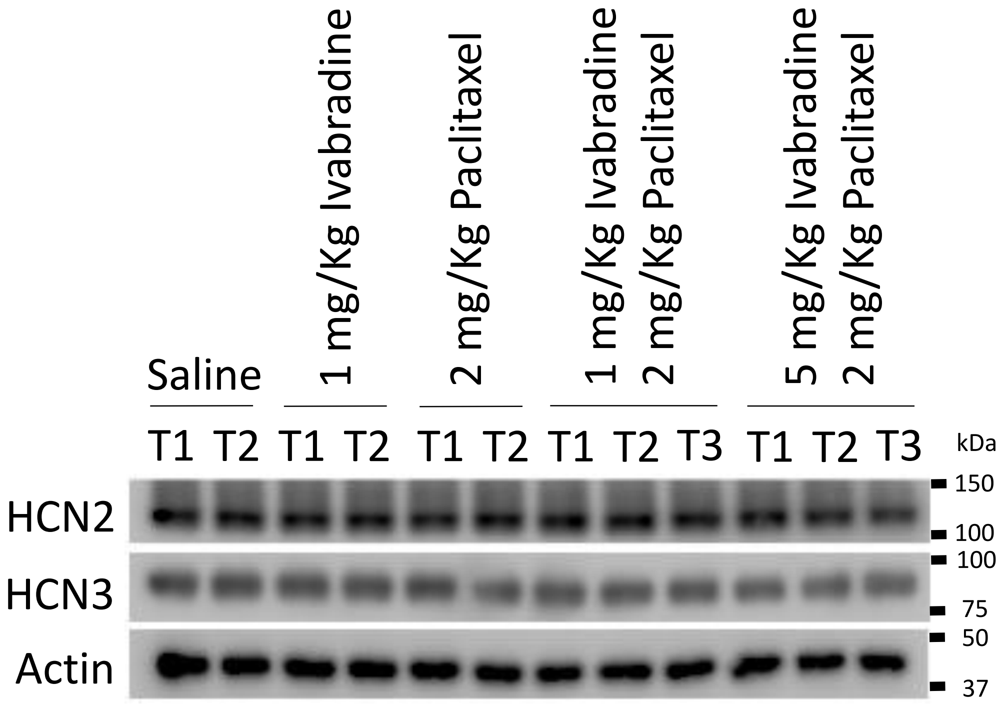
Saline
1 mg/Kg Ivabradine
2 mg/Kg Paclitaxel
1 mg/Kg Ivabradine 2 mg/Kg Paclitaxel
5 mg/Kg Ivabradine 2 mg/Kg Paclitaxel
T1 T2 T1 T2 T1 T2 T1 T2 T3 T1 T2 T3
kDa
HCN2
HCN3
Actin
150
100
100
75
50
37

FIGURE S17 Uncropped blots used in main figures

Figure 1A

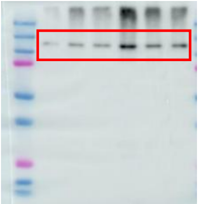

HCN2
anti-HCN2 (APC-030; 1:1,000; Alomone Labs)

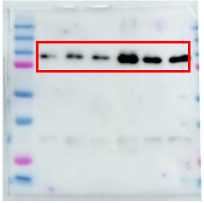

HCN3
anti-HCN3 (APC-057; 1:1,000; Alomone Labs)

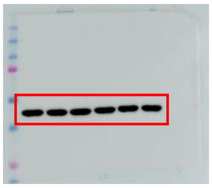

Actin
anti-actin (sc-47778; 1:5,000; Santa Cruz Biotechnology)

Figure 1B

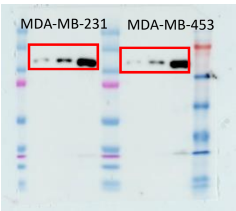

HCN2
anti-HCN2 (APC-030; 1:1,000; Alomone Labs)

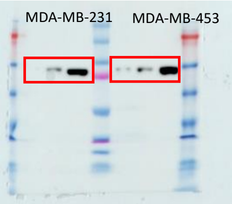

HCN3
anti-HCN3 (APC-057; 1:1,000; Alomone Labs)

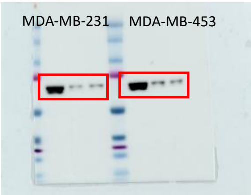

Lamin B1
anti-Lamin B1 (#12586; 1:5,000; Cell Signaling Technology)

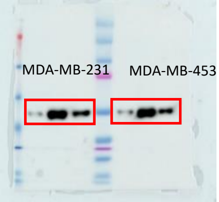

Tubulin
anti-tubulin (sc-9104; 1:10000; Santa Cruz Biotechnology)

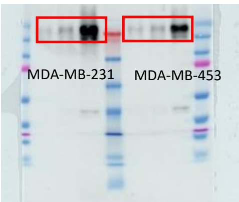

PMCA
anti-PMCA ATPase (MA3-914; 1:2,000; Invitrogen)

Figure 2I

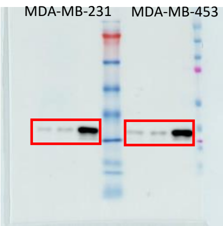

Cleaved caspase 9
cleaved caspase-9 (7237; 1:1000; Cell Signaling Technology)

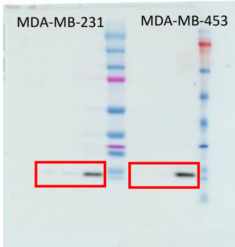

Cleaved caspase 3
anti-cleaved caspase-3 (9664; 1:1000; Cell Signaling Technology)

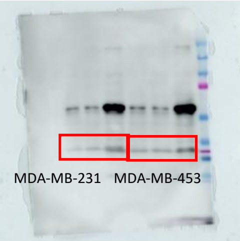

Cleaved caspase 7
anti-cleaved caspase-7 (9491; 1:1000; Cell Signaling Technology)

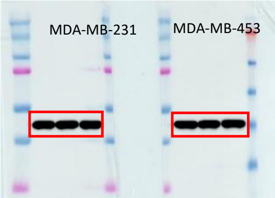

Actin
anti-actin (sc-47778; 1:5,000; Santa Cruz Biotechnology)

FIGURE S17 (continued)

Figure 3D

MDA-MB-231 xenograft

MDA-MB-453 xenograft

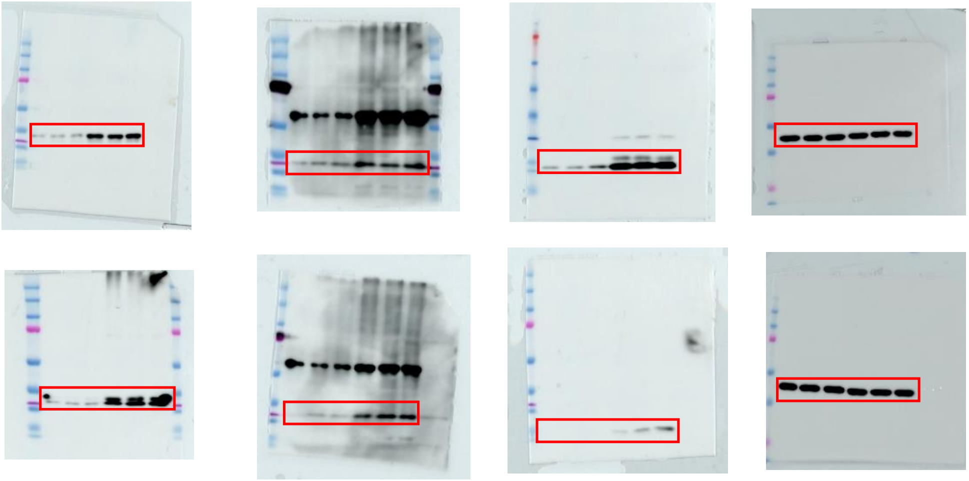

Cleaved caspase 9
cleaved caspase-9 (7237; 1:1000; Cell Signaling Technology)

Cleaved caspase 7
anti-cleaved caspase-7 (9491; 1:1000; Cell Signaling Technology)

Cleaved caspase 3
anti-cleaved caspase-3 (9664; 1:1000; Cell Signaling Technology)

Actin
anti-actin (sc-47778; 1:5,000; Santa Cruz Biotechnology)

Figure 3G

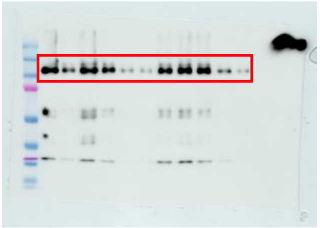

HCN2
anti-HCN2 (APC-030; 1:1,000; Alomone Labs)

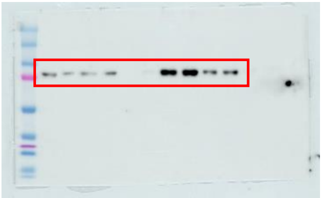

HCN3
anti-HCN3 (APC-057; 1:1,000; Alomone Labs)

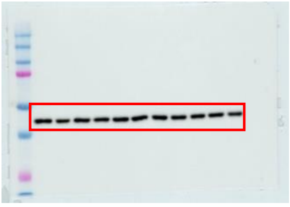

Actin
anti-actin (sc-47778; 1:5,000; Santa Cruz Biotechnology)

FIGURE S17 (continued)

Figure 5C

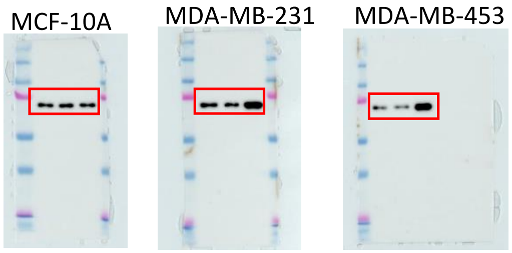

GRP78
Anti-GRP78 BiP antibody (ab21685; 1:4,000; Abcam)

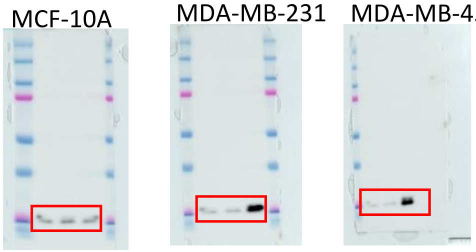

CHOP
anti-CHOP (MA1-250; 1:2,000; Invitrogen)

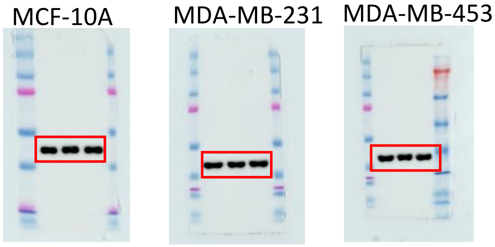

Actin
anti-actin (sc-47778; 1:5,000; Santa Cruz Biotechnology)

Figure 5D

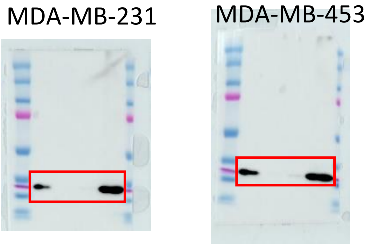

CHOP
anti-CHOP (MA1-250; 1:2,000; Invitrogen)

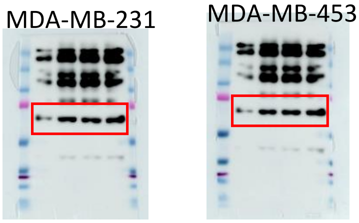

Lamin B1
anti-Lamin B1 (#12586;
1:5,000; Cell Signaling
Technology)

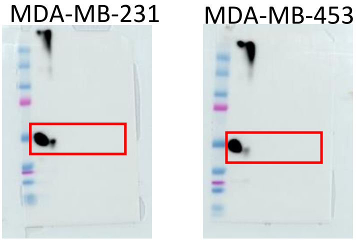

Tubulin
anti-tubulin (sc-9104; 1:10000;
Santa Cruz Biotechnology)

FIGURE S17 (continued)

Figure 6C

siHCN2

siHCN3

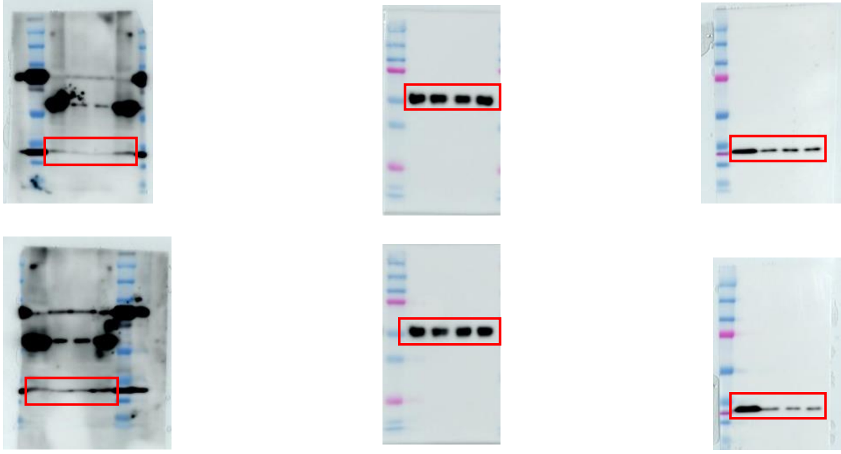

Cytochrome C
anti-cytochrome C antibody (ab13575; 1:1,000; Abcam)

Tubulin
anti-tubulin (sc-9104; 1:10000; Santa Cruz Biotechnology)

GAPDH
anti-GAPDH (sc-32233; 1:10,000; Santa Cruz Biotechnology)

Figure 6G

shHCN2.1

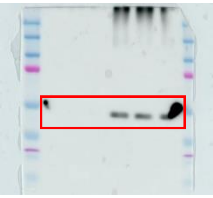

anti-ATF4 (11815; 1:1000; Cell Signaling Technology)

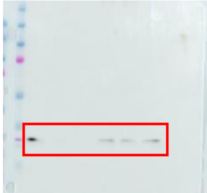

anti-CHOP (MA1-250; 1:2,000; Invitrogen)

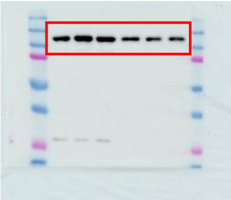

HCN2
anti-HCN2 (APC-030; 1:1,000; Alomone Labs)

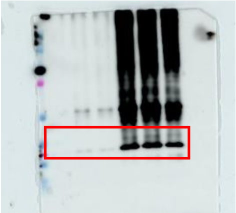

anti-Bim (2933; 1:1000; Cell Signaling Technology)

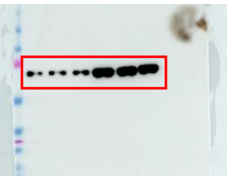

Anti-GRP78 BiP antibody (ab21685; 1:4,000; Abcam)

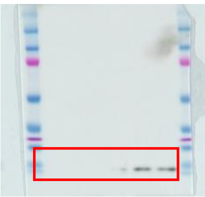

Cleaved caspase 3
anti-cleaved caspase-3 (9664; 1:1000; Cell Signaling Technology)

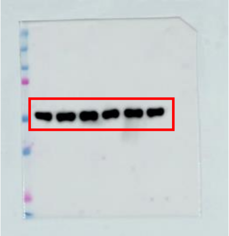

anti-tubulin (sc-9104; 1:10000; Santa Cruz Biotechnology)

FIGURE S17 (continued)

Figure 6H

shHCN3.1

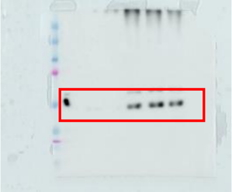

anti-ATF4 (11815; 1:1000; Cell Signaling Technology)

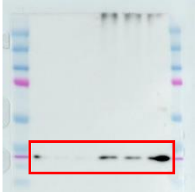

anti-CHOP (MA1-250; 1:2,000; Invitrogen)

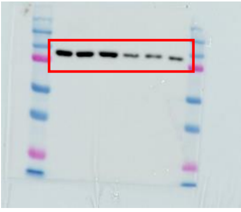

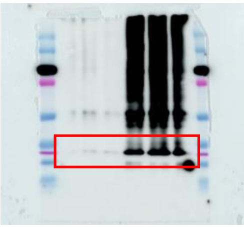

anti-Bim (2933; 1:1000; Cell Signaling Technology)

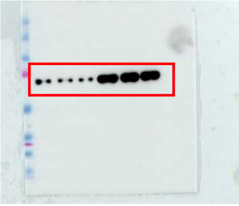

Anti-GRP78 BiP antibody (ab21685; 1:4,000; Abcam)

HCN3
anti-HCN3 (APC-057; 1:1,000; Alomone Labs)

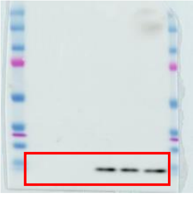

Cleaved caspase 3
anti-cleaved caspase-3 (9664; 1:1000; Cell Signaling Technology)

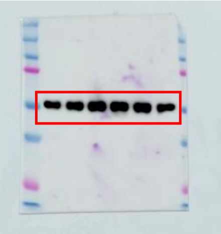

anti-tubulin (sc-9104; 1:10000; Santa Cruz Biotechnology)

FIGURE S17 (continued)

Figure 7L

GRP78

MDA-MB-231

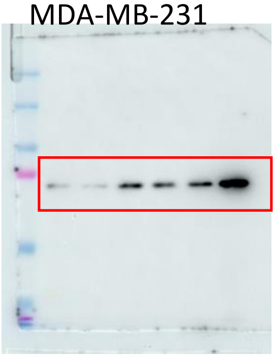

MDA-MB-453

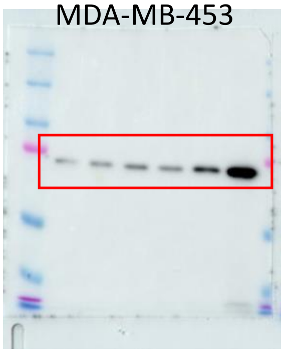

Anti-GRP78 BiP antibody (ab21685; 1:4,000; Abcam)

CHOP

MDA-MB-231

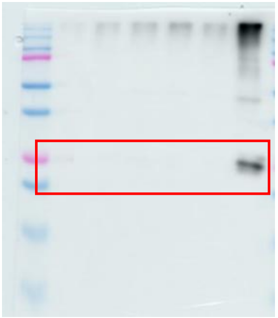

MDA-MB-453

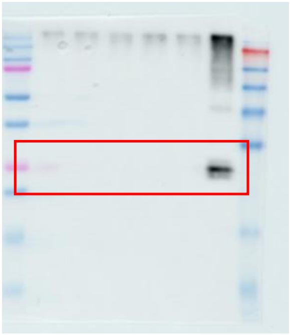

anti-CHOP (MA1-250; 1:2,000; Invitrogen)

Cleaved caspase 9

MDA-MB-231

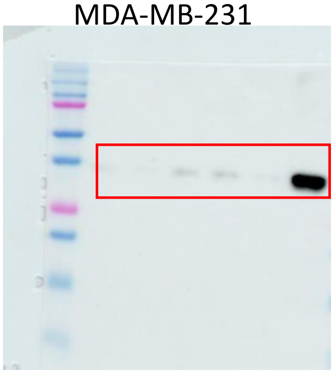

MDA-MB-453

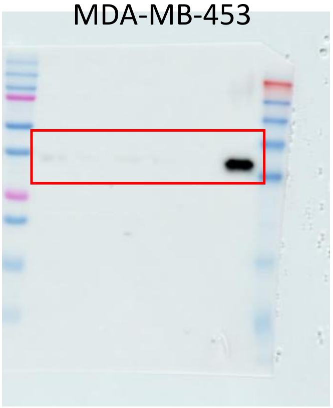

Cleaved caspase 9
Anti-cleaved caspase-9 (7237; 1:1000; Cell Signaling Technology)

Cleaved caspase 3

MDA-MB-231

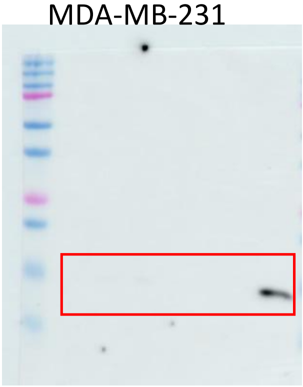

MDA-MB-453

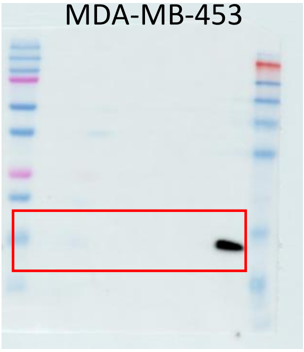

Cleaved caspase 3
anti-cleaved caspase-3 (9664; 1:1000; Cell Signaling Technology)

Actin

MDA-MB-231

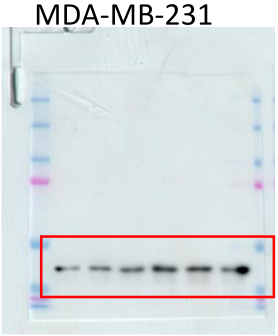

MDA-MB-453

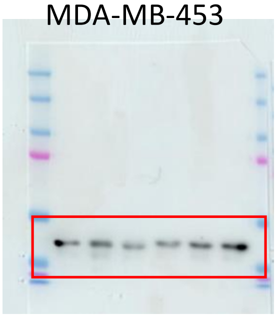

Actin
anti-actin (sc-47778; 1:5,000; Santa Cruz Biotechnology)

FIGURE S18

Uncropped blots used in supplemental figures

Figure S3A

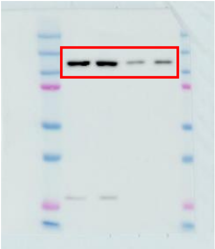

HCN2
anti-HCN2 (APC-030; 1:1,000; Alomone Labs)

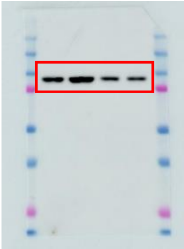

HCN3
anti-HCN3 (APC-057; 1:1,000; Alomone Labs)

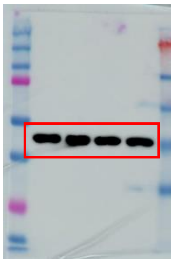

Actin
anti-actin (sc-47778; 1:5,000; Santa Cruz Biotechnology)

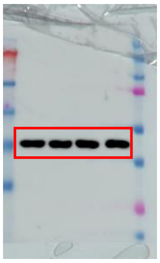

Actin
anti-actin (sc-47778; 1:5,000; Santa Cruz Biotechnology)

Figure S3B

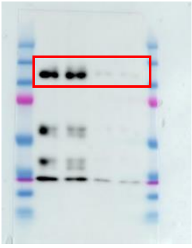

HCN2
anti-HCN2 (APC-030; 1:1,000; Alomone Labs)

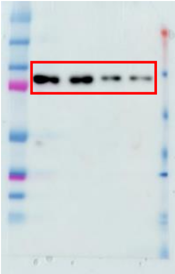

HCN3
anti-HCN3 (APC-057; 1:1,000; Alomone Labs)

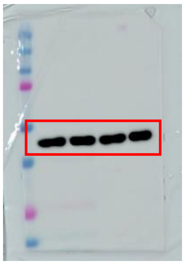

Actin
anti-actin (sc-47778; 1:5,000; Santa Cruz Biotechnology)

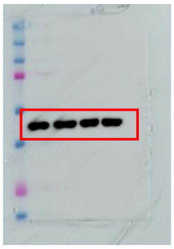

Actin
anti-actin (sc-47778; 1:5,000; Santa Cruz Biotechnology)

FIGURE S18 (continued)

Figure S4C

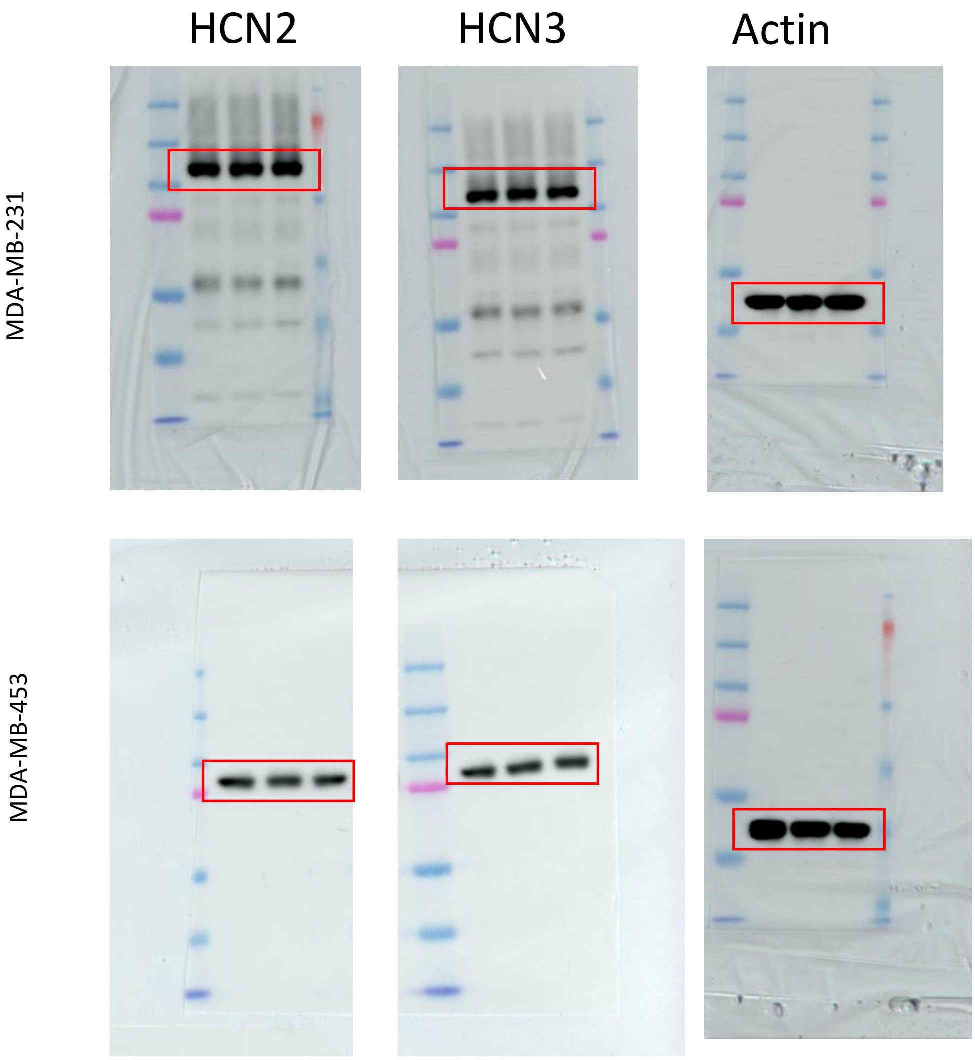

HCN2
anti-HCN2 (APC-030; 1:1,000; Alomone Labs)

HCN3
anti-HCN3 (APC-057; 1:1,000; Alomone Labs)

Actin
anti-actin (sc-47778; 1:5,000; Santa Cruz Biotechnology)

FIGURE S18 (continued)

Figure S5

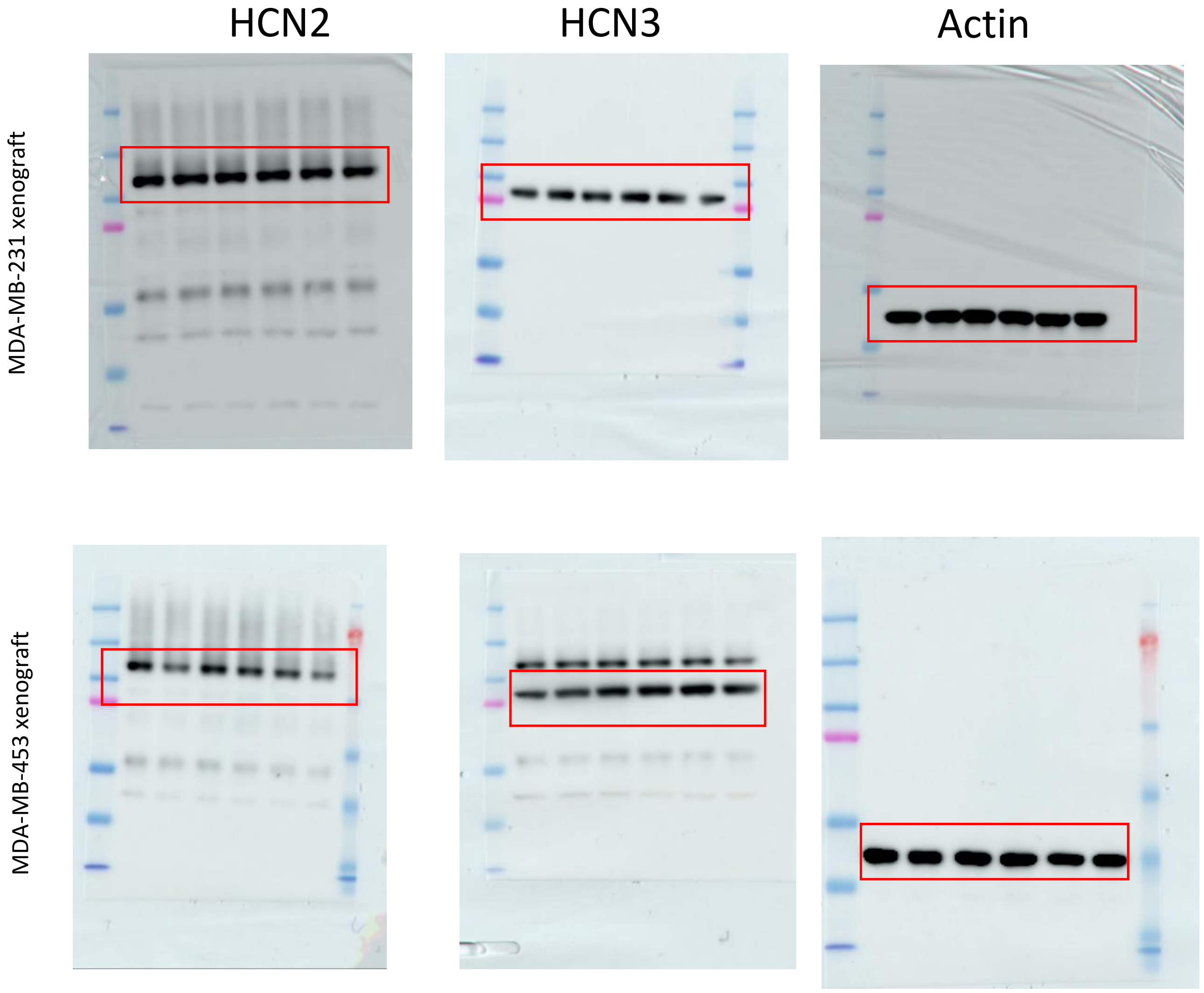

HCN2
anti-HCN2 (APC-030;
1:1,000; Alomone Labs)

HCN3
anti-HCN3 (APC-057; 1:1,000;
Alomone Labs)

Actin
anti-actin (sc-47778; 1:5,000;
Santa Cruz Biotechnology)

FIGURE S18 (continued)

Figure S8

HCN2

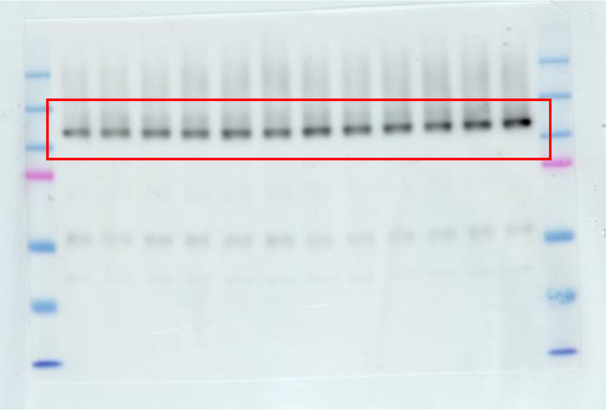

HCN2
anti-HCN2 (APC-030;
1:1,000; Alomone Labs)

HCN3

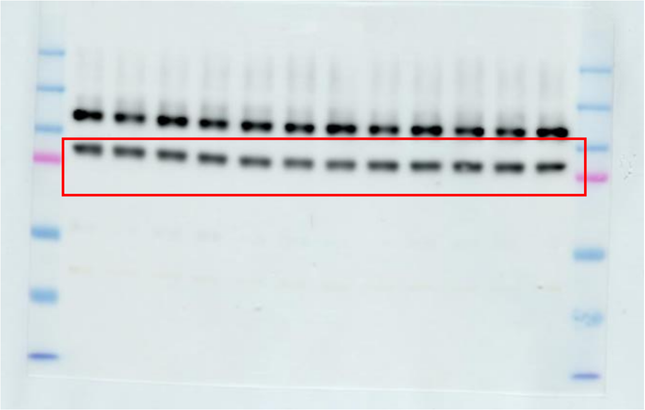

HCN3
anti-HCN3 (APC-057; 1:1,000;
Alomone Labs)

Actin

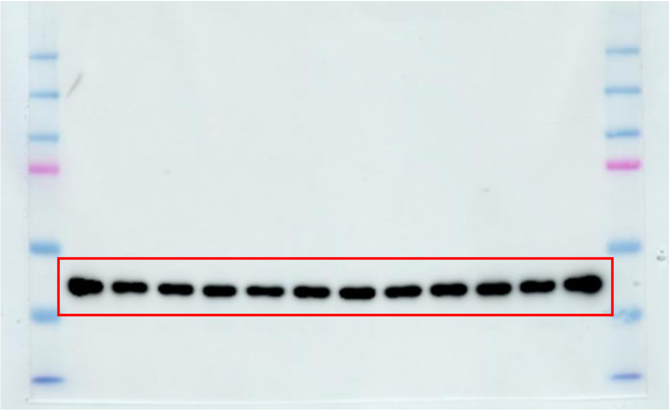

Actin
anti-actin (sc-47778; 1:5,000;
Santa Cruz Biotechnology)

FIGURE S18 (continued)

Figure S11

MDA-MB-231 xenograft MDA-MB-453 xenograft

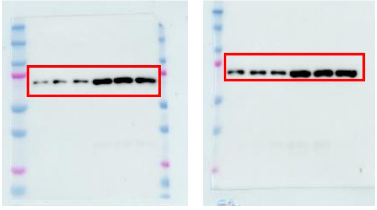

GRP78
Anti-GRP78 BiP antibody (ab21685; 1:4,000; Abcam)

MDA-MB-231 xenograft MDA-MB-453 xenograft

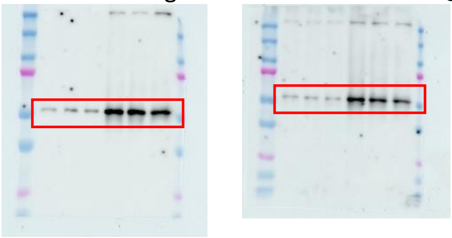

anti-ATF4 (11815; 1:1000; Cell Signaling Technology)

MDA-MB-231 xenograft MDA-MB-453 xenograft

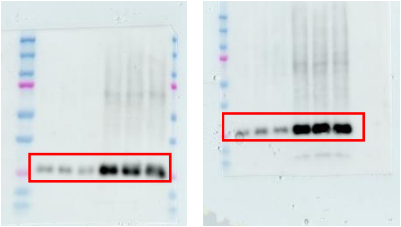

CHOP
anti-CHOP (MA1-250; 1:2,000; Invitrogen)

MDA-MB-231 xenograft MDA-MB-453 xenograft

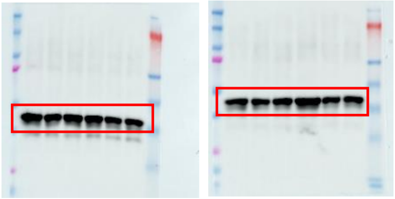

Actin
anti-actin (sc-47778; 1:5,000; Santa Cruz Biotechnology)

Figure S12B

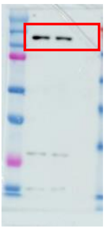

HCN2
anti-HCN2 (APC-030; 1:1,000; Alomone Labs)

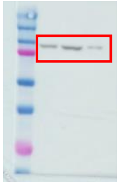

HCN3
anti-HCN3 (APC-057; 1:1,000; Alomone Labs)

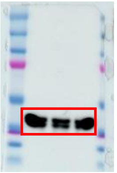

Actin
anti-actin (sc-47778; 1:5,000; Santa Cruz Biotechnology)

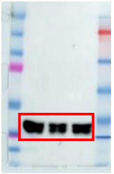

Actin
anti-actin (sc-47778; 1:5,000; Santa Cruz Biotechnology)

Figure S12D

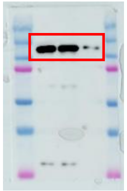

HCN2
anti-HCN2 (APC-030; 1:1,000; Alomone Labs)

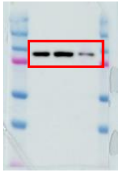

HCN3
anti-HCN3 (APC-057; 1:1,000; Alomone Labs)

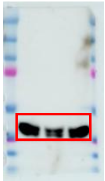

Actin
anti-actin (sc-47778; 1:5,000; Santa Cruz Biotechnology)

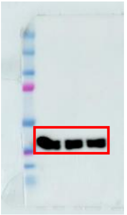

Actin
anti-actin (sc-47778; 1:5,000; Santa Cruz Biotechnology)

FIGURE S18 (continued)

Figure S16

HCN2

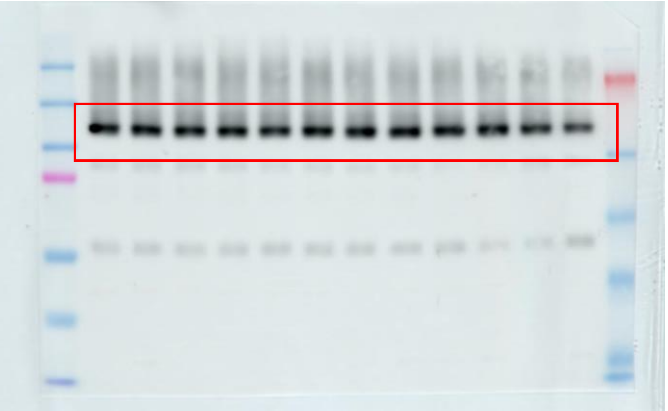

HCN2
anti-HCN2 (APC-030; 1:1,000; Alomone Labs)

HCN3

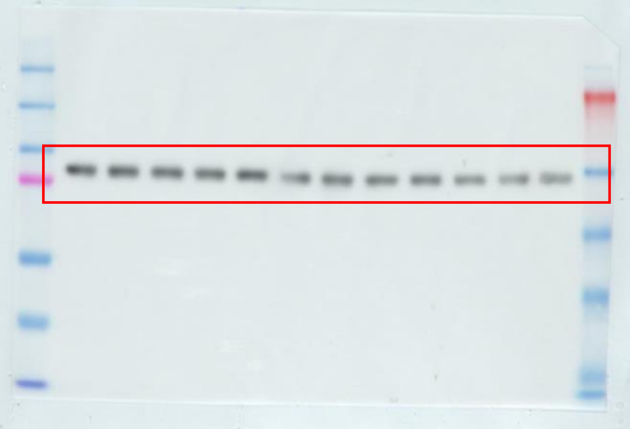

HCN3
anti-HCN3 (APC-057; 1:1,000; Alomone Labs)

Actin

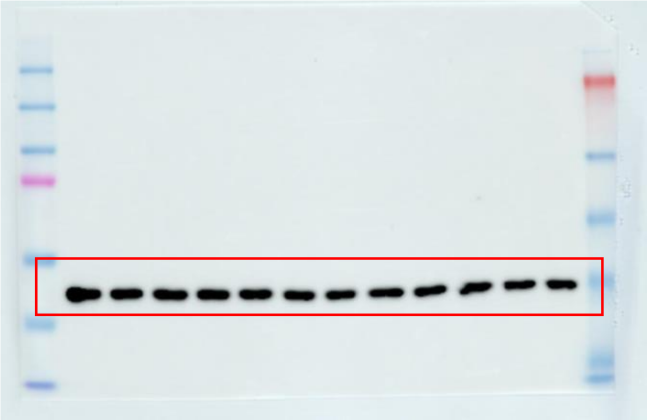

Actin
anti-actin (sc-47778; 1:5,000; Santa Cruz Biotechnology)

FIGURE S19

Spread sheets of protein ladder and antibodies used

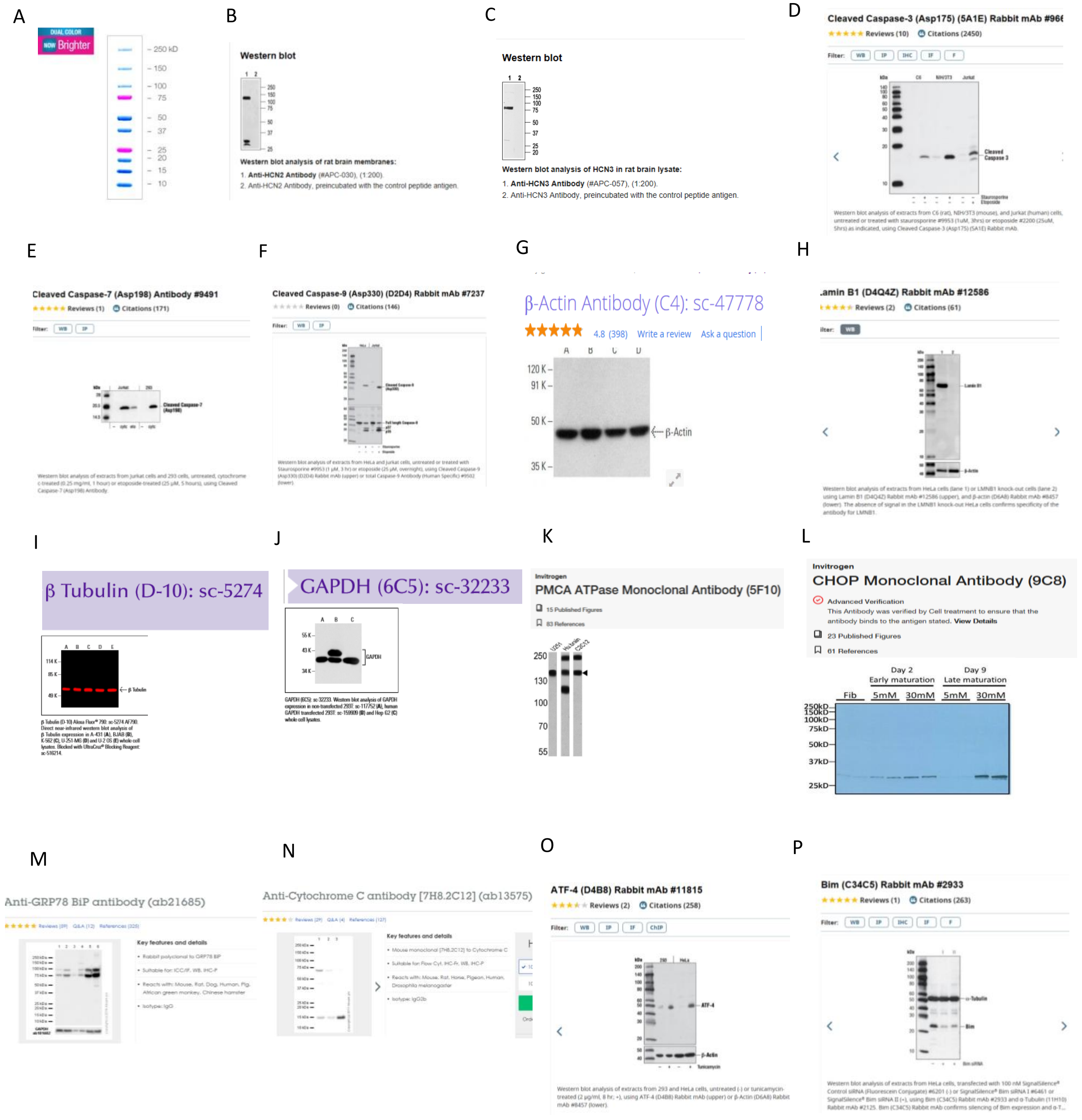

Supplement: Supplementary file 1 — Figures [file CTM2-11-e578-s003.pdf]
