# Supplementary material for: Repurposing hyperpolarization‐activated cyclic nucleotide‐gated channels as a novel therapy for breast cancer
Source: Clin Transl Med. 2021 Nov 4;11(11):e578. doi: 10.1002/ctm2.578 (PMC8567035; doi:10.1002/ctm2.578)
Supplement: Supplementary file 2 — Supplementry information [file CTM2-11-e578-s004.docx]

**Supplementary information**

**Repurposing hyperpolarization-activated cyclic nucleotide-gated channels as a novel therapy for breast cancer**

**Authors:** Ka-Chun Mok^1†^, Ho Tsoi^1†^, Ellen PS Man^1^, Man-Hong Leung^1^, Ka Man Chau^1^, Lai-San Wong^2^, Wing-Lok Chan^3^, Sum-Yin Chan^2^, Mai-Yee Luk^2^, Jessie YW Chan^4^, Jackie KM Leung^4^, Yolanda HY Chan^5^, Sellma Batalha^1^, Virginia Lau^6^, David CW Siu^6^, Terence KW Lee^7^, Chun Gong^1*^, Ui-Soon Khoo^1*^

^†^These authors contributed equally.

**Affiliations:**

^1^Department of Pathology, Li Ka Shing Faculty of Medicine, The University of Hong Kong, Hong Kong; ^2^Department of Clinical Oncology, Queen Mary Hospital, Hong Kong; ^3^Department of Clinical Oncology, Li Ka Shing Faculty of Medicine, The University of Hong Kong; ^4^Department of Surgery Pamela Youde Nethersole Eastern Hospital, Hong Kong; ^5^Department of Surgery, Kwong Wah Hospital, Hong Kong; ^6^Department of Medicine, The University of Hong Kong; ^7^Department of Applied Biology & Chemical Technology, The Hong Kong Polytechnic University, Hong Kong

**Running title**

Targeting HCN as a novel therapy for TNBC

**Key words**

1. Triple-negative breast cancer; 2. HCN; 3. Ivabradine; 4. Targeted therapy; 5. ER stress

**^*^Corresponding Authors:**

Ui-Soon Khoo, E-mail: [uskhoo@pathology.hku.hk](mailto:uskhoo@pathology.hku.hk)

Rm 014, 7/F, Block T, Queen Mary Hospital, Pokfulam Road, Hong Kong SAR, China.

Chun Gong, E-mail: [cg602@cam.ac.uk](mailto:cg602@cam.ac.uk)

Room 505, The Clifford Allbutt Building, Hills Road, Department of Haematology, University of Cambridge, Cambridge CB2 0AH, UK

**Supplementary Materials and Methods**

**Cell culture and stable cell line establishment**

MCF-7, MDA-MB-231 and BT-474 cells were cultured and maintained in Dulbecco's Modified Eagle Medium (DMEM, Gibco) supplemented with 10% fetal bovine serum (Gibco) and 1% penicillin/streptomycin (Gibco). MDA-MB-453 and ZR-75 cells were grown in Improved Minimum Essential Medium (IMEM, GIbco) with the addition of 10% FBS and 1% P/S. MCF-10A was cultured and maintained in Mammary Epithelial Cell Growth Medium (Lonza) supplemented by Bovine Pituitary Extract (Lonza), Human Epidermal Growth Factor (Lonza), Hydrocortisone and 100 ng/ml cholera toxin. All the cell lines were cultured in tissue culture incubator with 5% CO_2_ at 37°C. MBA-MB-231 and MDA-MB-453 were transfected with shCtrl, shHCN2.1, shHCN2.2, shHCN3.1 and shHCN3.2. After 72 hours post-transfection, 1 µg/mL of puromycin (Gibco) was employed for the selection of transfected cells. Fresh DMEM or IMEM with 10% FBS, 1% P/S and 1 µg/mL of puromycin was replaced every 72 hours. The selection was performed for six weeks. The cell lines were maintained in DMEM or IMEM with 10% FBS, 1% P/S and 1 µg/mL of puromycin.

**Knockdown of HCN2 and HCN3**

For HCN2 knockdown constructs, human TRIPZ lentiviral inducible shRNAmir individual clones V2THS_94714 (Dharmacon; shHCN2.1; mature sense: 5’-CTG ACC ATG CTC AGC ATG A-3’) and V2THS_385742 (Dharmacon; shHCN2.2; mature sense: 5’-CGC CAG AAG ATC CAC GAC T-3’) and the non-silencing-TRIPZ lentiviral inducible non-targeting shRNAmir control (shCtrl) were purchased from Dharmacon. 50 ng/mL of doxycycline hyclate was used to induce the expression. For HCN3 knockdown constructs, two unique 29-mer shRNA constructs in lentiviral GFP vectors TL304147A (OriGene; shHCN3.1; mature sense: 5’-TGG AGC AGT ACA TGT CCT TCC ACA AGC TG-3’), TL304147B (OriGene; shHCN3.2; mature sense: 5’-TAC GCA TCG TTC GCT TCA CCA AGA TCC TA-3’) with TR30021 scrambled negative control non-effective shRNA cassette (mature sense: 5’-GCA CTA CCA GAG CTA ACT CAG ATA GTA CT-3’) in pGFP-C-shLenti plasmid were purchased from OriGene. The SMARTpool ON-TARGETplus Human siRNAs, siHCN1 (Dharmacon; L-006200; target sequences: 5’- GAG GAG AGC CUU UGA GAC A-3’; 5’-ACA ACA ACA CCA UGG AUU A-3’; 5’-CCG ACU AGA UCG AAU AGG A-3’; 5’-CAU CAU ACC AGU UGG AAU C-3’), siHCN2 (Dharmacon; L-006201; target sequences; 5’-CCA GCG CGG UGA UGA GGA U-3’; 5’-CAG CUG ACU UCC GCC AGA A-3’; 5’-GUA GGU AGC CGU AGU UGG A-3’; 5’- CGC AAU AAA CGA CAG CAU U-3’) and siHCN3 (Dharmacon; L-022353; target sequences: 5’-GAU CGU CUU CAA CGU AUU G-3’; 5’-GCG AGG AGA UCA UUA ACU U-3’; 5’-GGC CAA ACC UCC AAG GAC A-3’; 5’-AAU CUG GGC CUG AGC CUA A-3’), siHCN4 (Dharmacon; L-006203; target sequences: 5’-GCA CAG GGA UCG UGG UGG A-3’; 5’-CGG AAG ACA UCC UCA GGU U-3’; 5’-UCA ACU ACC AGG AGA AUG A-3’; 5’-GGA CAC CGC UAU CAA AGU G-3’) and non-targeting siRNA control (Dharmacon; siCtrl; D-001810) were purchased.

**Orthotopic Xenograft**

4 x 10^6^ cells of MDA-MB-231, MDA-MB-453, MDA-MB-231-shCtrl, MDA-MB-231-shHCN2.1 and MDA-MB-231 shHCN3.1 were trypsinized and mixed with Matrigel (Corning) at 1:1 ratio in volume. The cell mixture was implanted into the mammary fat pad. Calliper was used to measure the sizes of the tumor. The volume of subcutaneously xenografted tumors was determined by current standard technique i.e. the use of the modified ellipsoid formula. The volume of the tumor was calculated by the longer diameter of the protrusion x (the shorter diameter)^2^/2 (http://tumor.informatics.jax.org/mtbwi/live/www/html/SOCHelp.html#volume). At the endpoint of the experiment, mice were euthanized, and tumours were harvested.

**Patient-derived tumor xenografts (PDTX)**

PDTX models were established as previously described with some modifications as detailed below.^1^ Immediately after surgical excision, the surgeon obtained 5 core biopsies from the tumor which was immediately washed with serum-free DMEN/F-12 medium and freshly minced into small pieces of around 1 mm x 1 mm in size. The tissue was then be transferred into a gentle MACS C tube (Miltenyi Biotec GmbH) containing 20 µg/ml DNaseI (Roche) and 4 µg/ml liberase (Roche) in DMEM/F-12 medium. The tissues were then homogenized with gentle MACS dissociator (Miltenyi Biotec GmbH) using the built-in protocol for human tumor homogenization. The homogenized tissue was then passed through a 100 µm cell stainer (BD Biosciences), washed twice with DMEM/F-12 medium by centrifugation at 700 rpm by Eppendorf 5810R for 5 minutes at 4ºC. The cell pellet was resuspended with 2 ml of DMEM/F-12 medium supplemented by 10% of FBS and 1% of Penicillin/Streptomycin. The viability of live cells was evaluated by trypan blue staining (Sigma). 1 x 10^6^ number of cells were resuspended in 50 µl of DMEM/F-12 medium supplemented by 10% of FBS and 1% of Penicillin/Streptomycin and mixed with 50 µl of Matrigel TM Matrix (BD Bioscience) at 1:1 ratio. The mixture was then injected into the mammary fat pad of NOD/SCID mice of between 4-6 weeks of age, using 27 gauge insulin needles (Terumo).

**RNA extraction, reverse transcription and qPCR**

TRIzol reagent was used for the extraction of total RNA from the cells. Complementary DNA (cDNA) was synthesized by reverse transcription using PrimeScript RT Master Mix (TaKaRa Biotechnology). 0.5 µg of RNA was used for reverse transcription. The synthesized cDNAs were further diluted 10-fold using double–distilled water. SYBR Premix Ex Taq II (Tli RNaseH Plus) from TaKaRa was used for qPCR. ABI 7900HT Fast Real-time PCR system (Applied Biosystem) was used for qPCR. ∆∆CT method was used to determine the relative expression of target genes. The following primers (5′🡪3′) were used: HCN1-F: TCG AAC ACT GGC AGT ACG AC; HCN1-R: GCC ATG CTG AGC AAG TTC AG; HCN2-F: CTG AGC GTG GAC AAC TTC AA; HCN2-R: GTT GTT GAA TAA CGC CGA GT; HCN3-F: GAT TCC GAG CCT ACG ACG C; HCN3-R: GAA GAT GCG AAC CAC AGC AC; HCN4-F:CCC GTG GAC TAC ATC TTC CT; HCN4-R: TAA CAG GCG TAA GAG GCT GA; ActinF, ATG TGC AAG GCC GGT TTC GC; ActinR, CGA CAC GCA GCT CAT TGT AG.

**Library preparation for NGS sequencing and analysis**

cDNA libraries were prepared by KAPA Stranded mRNA-Seq Kit (KR0960-v3.15). One microgram of total RNA was used for polyA tail RNA isolation. The purified mRNA was fragmented to 200–300 bp by incubating at 94^o^C for 6 minutes in the presence of magnesium ions. The fragmented mRNA was then applied as a template to synthesize the first-strand cDNA by using random hexamer-primer and reverse transcriptase. In the second strand cDNA synthesis, the mRNA template was removed, and a replacement strand was generated to form the blunt-end double-stranded (ds) cDNA. The ds cDNA underwent 3' adenylation and indexed adaptor ligation (xGen® Dual Index UMI Adapters). The adaptor-ligated libraries were enriched by 10 cycles of polymerase chain reaction (PCR). The libraries were denatured and diluted to optimal concentration. Reads were mapped to the reference genome (Human Genome GRCh38) using STAR (Version 2.5.2) with default parameters.^2^ Expression analysis was done using EBSeq (Version 1.18).^3^ The expression was quantified by RSEM (Version 1.2.31).^4^ Heatmap showing the differentially expressed genes was created by CIMminer (National Cancer Institute). Gene ontology (GO) enrichment analysis was performed. Pathway enrichment analysis was performed using Reactome. The analysis was performed on Consensus PathDB-human performed.^5^ A pathway contained at least three genes.

**Western blot**

Cell pellets were lysed using cell lysis buffer prepared by mixing 1x Cell Signaling buffer (Cell Signaling Technology), 10% glycerol, cOmplete Mini, EDTA-free protease inhibitor cocktail (Roche), phenylmethanesulfonyl fluoride and PhosSTOP EASYPack tablets (Roche). The protein concentration of the cell lysates was determined by DC protein assay (BioRad). SDS-polyacrylamide gels were made following the protocol for Western blot analysis. 20 µg of total proteins were loaded in each of the wells of the gel. The proteins were transferred onto PVDF membrane. Normal breast lysate (1346-N) was purchased from ProSci. The signal was captured Amersham Imager 680 (GE Healthcare). All experiments were repeated at least three times and representative images were shown. Uncropped blots were shown in supplementary figures. Colored images were converted to black-and-white for figure preparation.

The following primary antibodies were used: anti-HCN2 (APC-030; 1:1,000; Alomone Labs); anti-HCN3 (APC-057; 1:1,000; Alomone Labs); anti-cleaved caspase-3 (9664; 1:1000; Cell Signaling Technology); anti-cleaved caspase-7 (9491; 1:1000; Cell Signaling Technology); anti-cleaved caspase-9 (7237; 1:1000; Cell Signaling Technology); anti-actin (sc-47778; 1:5,000; Santa Cruz Biotechnology); anti-Lamin B1 (12586; 1:5,000; Cell Signaling Technology); anti-tubulin (sc-5274; 1:10000; Santa Cruz Biotechnology); anti-GAPDH (sc-32233; 1:10,000; Santa Cruz Biotechnology); anti-PMCA ATPase (MA3-914; 1:2,000; Invitrogen); anti-CHOP (MA1-250; 1:2,000; Invitrogen); Anti-GRP78 BiP antibody (ab21685; 1:4,000; Abcam); anti-cytochrome C antibody (ab13575; 1:1,000; Abcam); anti-ATF4 (11815; 1:1000; Cell Signaling Technology); anti-BIM (2933; 1:1000; Cell Signaling Technology).

**Electrocardiography**

The Electrocardiography signals were amplified with the amplifier (Aminal Bio Amp) and were recorded using PowerLab systems and Chart 5 software (AD Instruments, Inc, Colorado Springs, Co).

**Live-cell imaging and calcium measurement**

10 mM of Inositol 1,4,5-Trisphosphate (IP_3_; 407137; Sigma) was prepared. 1 µM of IP_3_ was used to treat MCF-10A permeabilized in 0.1% Saponin (Sigma). 10,000 MCF-10A cells were seeded on a coverslip. After 24 hours, the cells were treated with 4 µM of Fura-2-AM for 30 minutes at room temperature. The cells were washed three times with 1x PBS. The coverslip was mounted on coverslip chamber. The cells were excited by laser with 405 nm and signal passing through EGFP filter was recorded. The cells were excited every 5 seconds for 60 seconds. Then, 1 µM of IP_3_ was used to treat MCF-10A and the cells were excited and the signals were collected by the same methods. The fluorescent signals in 10 cells were randomly selected for data analysis.

**ELISA assay**

Alanine Transaminase Activity Assay Kit (ab105134, Abcam) and AST ELISA Kit (ab263882, Abcam) were employed to determine the amount of ALT and ASL in serum from mice.

**Tissue Microarray**

All patients had early operable primary breast cancer undergoing surgery as their primary treatment. TMA sections were obtained from the surgical resection samples. Histological sections of all cases were reviewed by the pathologist, the representative paraffin tumor blocks were chosen as donor block for each case, and the selected areas were marked for construction of TMA blocks. Each case was constructed as duplicate in the TMA, and the average score of the duplicate was taken as the score.

**Immunohistochemistry**

The sections from human and mouse tissues were deparaffinized and rehydrated by incubation with xylene and decreasing concentrations of ethanol. Antigen retrieval was performed by heating using a microwave for HCN staining, and pressure cooker where the slides were incubated in 3% H_2_O_2_/H_2_O for 10 minutes under room temperature. This process used for the stopping of the endogenous peroxidase. The slides were later washed by 1x PBS, which were later incubated with primary antibodies at 4°C overnight. After the incubation, all the slides were rinsed against 1x PBS with 0.05% Tween (PBST) twice and incubated with HRP-anti-mouse (for Ki-67 antibody) and HRP-anti-Rabbit (for HCN antibodies) respectively (DAKO) at room temperature for 30 minutes. After washing by PBST to remove excessive reagents, Chromogen DAB/substrate reagent was added onto the slides for a further few minutes. Finally, the slides were dehydrated and mounted for visualization. Scoring was performed by two independent individuals. The intensities and percentages of staining were determined by the semi-quantitative manner, and the means were taken. Cytoplasmic expressions of HCN2 and HCN3 were scored as follows. The intensity was scored as 0 = none, 1 = weak, 2 = moderate and 3 = strong. The percentage of cells stained was scored as 1 = less than 25%, 2 = 25% to 50%, 3 = 50% to 75% and 4 = more than 75%. The cytoplasmic score was calculated as the product of the percentage score and the intensity score. The median value was used to dichotomize the cutoff between low and high expression.

**Supplementary figure legends**

FIGURE S1

Evaluating the expression of HCN1, HCN2, HCN3 and HCN4 in breast cancer. Expression levels of **A**, HCN1, **B**, HCN2, **C**, HCN3 and **D**, HCN4 were retrieved from TCGA METABRIC database. The expression was compared between invasive breast carcinoma (N = 1904) and normal breast (N = 111).

FIGURE S2

Evaluating the expression level of HCN2 and HCN3 in cell lines. **A**, Expression of HCN2 in normal breast epithelial cell line MCF-10A compared with that in different breast cancer cell lines MCF-7, ZR-75, BT-474, MDA-MB-231 and MDA-MB-453. qPCR was employed to determine the expression of HCN2. Actin was used as the internal control. The results were shown as mean ± SD from three independent experiments. **B**, Expression of HCN3 in normal breast epithelial cell line MCF-10A compared with that in different breast cancer cell lines MCF-7, ZR-75, BT-474, MDA-MB-231 and MDA-MB-453. qPCR was employed to determine the expression of HCN3. Actin was used as the internal control. Expressions of **C**, HCN1 and **D**, HCN4 was undetectable in breast cancer cell lines. As the results were negative, cDNA of SIU-1 (an iPS-derived cardiac myocyte cell line) was used as positive control. qPCR was employed to determine the expression of HCN1 and HCN4. Actin was used as the internal control. Results were shown as mean ± SD from three independent experiments.

FIGURE S3

Knockdown efficiency of shRNA targeting HCN2 and HCN3. **A**, Expressions of HCN1-4 in the stable cell lines were examined. shCtrl represents MDA-MB-231 stable expression of non-targeting shRNA. shHCN2.1 represents MDA-MB-231 stable expression of shRNA (vector 1) against HCN2. shHCN2.2 represents MDA-MB-231 stable expression of shRNA (vector 2) against HCN2. shHCN3.1 represents MDA-MB-231 stable expression of shRNA (vector 1) against HCN3. shHCN3.2 represents MDA-MB-231 stable expression of shRNA (vector 2) against HCN3. qPCR was employed (Panel A). Actin was used as the internal control. Western blot was employed to determine the expression of HCN2 and HCN3 (Panel B). Tubulin was used as the loading control. **B**, Expressions of HCN1-4 in the stable cell lines were examined. shCtrl represents MDA-MB-453 stable expression of non-targeting shRNA. shHCN2.1 represents MDA-MB-453 stable expression of shRNA (vector 1) against HCN2. shHCN2.2 represents MDA-MB-453 stable expression of shRNA (vector 2) against HCN2. shHCN3.1 represents MDA-MB-453 stable expression of shRNA (vector 1) against HCN3. shHCN3.2 represents MDA-MB-453 stable expression of shRNA (vector 2) against HCN3. qPCR was employed (Panel A). Actin was used as the internal control. Western blot was employed to determine the expression of HCN2 and HCN3 (Panel B). Tubulin was used as the loading control. Results was shown as mean ± SD from three independent experiments. One-way ANOVA was used. Statistical significance between shCtrl and shHCN2.1/shHCN3.1 or shHCN2.2/shHCN3.2 was determined by Bonferroni test. *** represents *P* < .001.

FIGURE S4

The effect of Ivabradine on cell viability and apoptosis. **A**, The dose dependent effect of Ivabradine on cell viability. MDA-MB-231 was treated with different concentrations of Ivabradine for 96 hours. MTT assay was employed to determine the cell viability. Results was shown as mean ± SD from three independent experiments. One-way ANOVA was used. Statistical significance between untreated (0 nM) and treatment group was determined by Bonferroni test. *, ** and *** represent *P* < .05, *P* <.01 and *P* < .001 respectively. **B**, The dose dependent effect of Ivabradine on cell viability. MDA-MB-453 was treated with different concentrations of Ivabradine for 96 hours. MTT assay was employed to determine the cell viability. Results was shown as mean ± SD from three independent experiments. One-way ANOVA was used. Statistical significance between untreated (0 nM) and treatment group was determined by Bonferroni test. ** and *** represent *P* <.01 and *P* < .001 respectively. **C**, The cell lines were treated with 50 µM of Ivabradine for 72 hours. FITC-Annexin V/PI staining was employed. Cells were analyzed by flow cytometry. Representative scatter plots were shown. **D**, The effect of Ivabradine on the expression of HCN2 and HCN3. MDA-MB-231 and MDA-MB-453 cell lines were treated with 5 μM of Ivabradine for 72 hours. Western blot was employed. Actin was used as the loading control.

FIGURE S5

The effect of Ivabradine on the expression of HCN2 and HCN3 *in vivo*. Xenografts were established from MDA-MB-231 (Panel A) and MDA-MB-453 (Panel B). The tumors were isolated and western blot was employed to determine the expression of HCN2 and HCN3 in the tumor tissues. Three independent tumours from water control and Ivabradine treated groups were analysis. Actin was used as the loading control.

FIGURE S6

HCN2 and HCN3 knockdown showed tumor suppressive effect *in vivo*. A, Knockdown of HCN2 repressed the tumor growth. Xenograft was established from MDA-MB-231 shCtrl and MDA-MB-231 shHCN2.1. The tumors isolated from the nude mice were shown. Graph showing change in tumor volume was plotted. Results were shown as mean ± SD from three tumors. Students’ t test was used. ** and *** represent *P* < .01 and *P* < .001 respectively. B, Knockdown of HCN3 repressed the tumor growth. Xenograft was established from MDA-MB-231 shCtrl and MDA-MB-231 shHCN3.1. The tumors isolated from the nude mice were shown. Graph showing change in tumor volume was plotted. Results were shown as mean ± SD from three tumors. Students’ t test was used. ** and *** represent *P* < .01 and *P* < .001 respectively.

FIGURE S7

The effect of Ivabradine on tumor volume and weight on individual PDTX models. 10 PDTX models were established. The mice were treated with 15 mg/kg of Ivabradine twice a week for 4 weeks through subcutaneous injection. Water was used as negative control. Results were expressed as mean ± SD. Students’ t test was used to determine statistical significance. *, ** and *** represent *P* < .05, *P* < .01 and *P* < .001 respectively.

FIGURE S8

The dosage dependent effect of Ivabradine on HCN2 and HCN3 expression in PDTX5. Two or three independent tumors from each of the treatment groups were analyzed by western blot to determine the expression of HCN2 and HCN3. Actin was used as the loading control.

FIGURE S9

Four weeks treatment of Ivabradine did not exert observable effect on heart rate nor cause liver damage. **A**, The effect of Ivabradine on the heart rate of nude mice. The mice were treated with 15 mg/Kg of Ivabradine and electrocardiogram was employed to record the heart activity at time points 0, 5, and 20 minutes immediately after the treatment (Panel A). The graph showed the effect of Ivabradine on the heart rate (Panel B). Ivabradine could significantly reduce the heart rate after 20 minutes of the injection (N = 5). Results were shown as mean ± SD from five mice. One-way ANOVA was used for statistical analysis. Statistical significance between 0 minute and 20 minutes was determined by Bonferroni test. * represents *P* < .05. Representative traces were shown. **B**, No obvious alterations in the cardiac rate or rhythm of the mice following long term treatment with Ivabradine (N = 10). The mice were treated with 15 mg/Kg of Ivabradine daily for 4 weeks through subcutaneous injection. Electrocardiogram was employed at least 6 hours after Ivabradine administration to record the heart activity at the 4^th^ week (Panel A). Results were shown as mean ± SD from five mice (Panel B). One-way ANOVA was used for statistical analysis. Statistical significance between two groups was determined by Bonferroni test. The representative trace was shown. **C**, Treatment of Ivabradine did not alter serum AST (Panel A) and ALT (Panel B) activities in PDTX mice. The serum samples were collected from mice bearing PDTXs after 4-week treatment of either 15 mg/Kg of Ivabradine (N = 10) or water (N = 10). ELISA was employed to determine the activities of the enzyme. Each spot represents the average activity of the corresponding enzyme in each of the PDTXs. Results were shown as mean ± SD from ten mice.

FIGURE S10

Ivabradine did not affect the level of intracellular Ca^2+^ in MCF-10A. A, The cells were treated with 200 µM of Ivabradine for the indicated time. 4 µM of Fura-2 was used to visualize cytoplasmic calcium ions. Live cell imaging was performed to record the fluorescence change (N = 10). Result were expressed as mean ± SD. B, 10 µM of inositol 1,4,5-trisphosphate (IP_3_) was applied to MCF-10A as the positive control and fluorescence signal was recorded continuously. 4 µM of Fura-2 was used to visualize cytoplasmic calcium ions. Untreated (N = 5) represents the cells did not receive Ivabradine nor IP_3_. Result were expressed as mean ± SD.

FIGURE S11

Ivabradine treatment could enhance the expression of GRP78, ATF4 and CHOP in xenograft tumors. Xenograft tumors were established from MDA-MB-231 (Panel A) and MDA-MB-453 (Panel B) implanted onto the mammary fat-pad of nude mice. The nude mice treated with 15 mg/Kg of Ivabradine or water administered subcutaneously daily for 4 weeks. Western blot was employed to determine the expression of the candidate proteins. Actin was used as loading control.

FIGURE S12

Knockdown efficiency of HCN2 and HCN3 in MDA-MB-231 and MDA-MB-453. The cells were transfected with 50 pmol of the siRNA for 72 hours. **A**, qPCR was performed to determine the expression of HCN1, HCN2, HCN3 and HCN4. One-way ANOVA was used. Statistical significance between siCtrl and siHCN2 or siHCN3 was determined by Bonferroni test. *** represents *P* < .001. and **B**, western blot was employed to determine the expression of HCN2 (Panel A) and HCN3 (Panel B) in MDA-MB-231. Tubulin was used as the loading control. **C**, qPCR was performed to determine the expression of HCN1, HCN2, HCN3 and HCN4. One-way ANOVA was used. Statistical significance between siCtrl and siHCN2 or siHCN3 was determined by Bonferroni test. *** represents *P* < .001. **D**, western blot was employed to determine the expression of HCN2 (Panel A) and HCN3 (Panel B) in MDA-MB-453. Tubulin was used as the loading control. Knockdown of HCN2 and HCN3 could reduce cell viability in **E**, MDA-MB-231 and F, MDA-MB-453. MTT assay was performed 72 hours post-transfection. Results were shown as mean ± SD from three independent experiments. One-way ANOVA was used. Statistical significance between siCtrl and siHCN2 or siHCN3 was determined by Bonferroni test. *** represents *P* < .001.

FIGURE S13

Knockdown of HCN2 and HCN3 compromised the efficacy of Ivabradine. The effect of knockdown of either HCN2 or HCN3 on the efficacy of Ivabradine in **A**, MDA-MB-231 and **B**, MDA-MB-453. The cells were treated with 200 μM of Ivabradine and 50 pmol of the corresponding siRNA for 72 hours. MTT assay was performed to determine the cell viability. Results were shown as mean ± SD from three independent experiments. Students’ t test was employed to determine statistical significance between H_2_O and Ivabradine treated cells in siCtrl group. One-way ANOVA was used. Statistical significance between siCtrl and siHCN2 or siHCN3 was determined by Bonferroni test. ** and *** represent *P* < .01 and *P* < .001 respectively. The effect of double knockdown of HCN2 and HCN3 on the efficacy of Ivabradine in **C**, MDA-MB-231 and **D**, MDA-MB-453. MDA-MB-231 and MDA-MB-453 were treated with 200 μM of Ivabradine and 50 pmol of siHCN2 and siHCN3 or 100 pmol of siCtrl for 72 hours. MTT assay was performed to determine the cell viability. Results were shown as mean ± SD from three independent experiments. Students’ t test was employed to determine statistical significance between two groups. *** represents *P* < .001. NS represents non-significant.

FIGURE S14

Knockdown of HCN1 and HCN4 did not affect the efficacy of Ivabradine. Knockdown efficiency of siRNA against HCN1 and HCN4 in **A**, MDA-MB-231 and **B**, MDA-MD-453. The cells were treated with 50 pmol of the corresponding siRNAs. qPCR was performed to determine the expression of HCN1, HCN2, HCN3 and HCN4. One-way ANOVA was used. Statistical significance between siCtrl and siHCN1 or siHCN4 was determined by Bonferroni test. *** represents *P* < .001. **C**, The effect of HCN1 and HCN4 knockdown on the efficacy of Ivabradine. MDA-MB-231 cells were treated with 200 µM of Ivabradine for 72 hours. MTT assay was employed to determine the cell viability. **D**, The effect of HCN1 and HCN4 knockdown on the efficacy of Ivabradine. MDA-MB-453 cells were treated with 200 µM of Ivabradine for 72 hours. MTT assay was employed to determine the cell viability. Results was shown as mean ± SD from three independent experiments. Students’ t test was used to determine the statistical significance between H2O and Ivabradine treated groups. *** represents *P* < .001. One-way ANOVA was used. Statistical significance between siCtrl and siHCN1 or siHCN4 was determined by Bonferroni test. NS represents non-significant.

FIGURE S15

The effect of chemodrugs and Ivabradine on cell viability and apoptosis. **A**, MCF-10A, **B**, MDA-MB-231 and **C**, MDA-MB-453 were used. 40 nM of Paclitaxel, 625 nM of Doxorubicin and 200 µM of Ivabradine were used to treat the cells. MTT assay was performed. Untreated control at each time point was used as a reference for the comparison. Results were presented as mean ± SD from three independent experiments. Two-way ANOVA was used. There was a statistically significant interaction between the effects of drugs and treatment time (MCF-10A, F = 20.13, *P* < .001; MDA-MB-231, F = 17.31, *P* < .001; MDA-MB-453, F = 27.67, *P* < .001). Statistical significance between untreated and Paclitaxel, Doxorubicin or Ivabradine at each of the time points was determined by Bonferroni test. *** represents *P* < .001. NS represents non-significant. **D**, MCF-10A, **E**, MDA-MB-231 and F, MDA-MB-453 were used. 40 nM of Paclitaxel, 625 nM of Doxorubicin and 200 µM of Ivabradine were used to treat the cells for 24 hours. TUNEL assay was used to stain apoptotic cells. Flow cytometry was employed to analyze the cells. Results were presented as mean ± SD from three independent experiments. One-way ANOVA was used. Statistical significance between untreated and Paclitaxel, Doxorubicin or Ivabradine was determined by Bonferroni test. *** represents *P* < .001. NS represents non-significant.

FIGURE S16

The effect of Ivabradine and Pacitaxel co-treatment on expression of HCN2 and HCN3 in MDA-MB-231 xenograft tissues were examined. Two or three independent tumors from different treatment groups were analyzed by western blot. Actin was used as the loading control.

FIGURE S17

Uncropped blots used to prepare main figures.

FIGURE S18

Uncropped blots used to prepare supplementary figures.

FIGURE S19

Spread sheets of **A**, protein ladder and **B-P**, antibodies used.

**Supplementary Tables**

**Table S1. Clinical pathological characteristics of the breast cancer patients**

| Clinical pathological characteristics |  | Number of Cases | Percentage (%) |
| --- | --- | --- | --- |
| Breast cancer patients |  | 316 | 100 |
| Age | <54 | 162 | 51.3 |
|  | >=54 | 151 | 47.8 |
| T stage | I, II | 183 | 57.9 |
|  | III, IV | 19 | 6 |
| Lymph Node status | Positive | 147 | 46.5 |
|  | Negative | 136 | 43 |
| Tumor Grade | 1, 2 | 132 | 41.8 |
|  | 3 | 147 | 46.5 |
| Tumor Size | <2 cm | 44 | 13.9 |
|  | >=2 cm | 74 | 23.4 |
| Estrogen Receptor status | Positive | 170 | 53.8 |
|  | Negative | 57 | 18 |
| Progesterone receptor status | Positive | 133 | 42.1 |
|  | Negative | 83 | 26.3 |
| HER2 receptor status | Positive | 39 | 12.3 |
|  | Negative | 150 | 47.5 |
| Triple Negative status | Positive | 27 | 8.5 |
|  | Negative | 177 | 56 |

**Table S2. Frequency of cases with both high HCN2 and HCN3 expression**

|  | Total no. of cases expressing both HCN2 and HCN3 | Cases with both high HCN2 and HCN3 expression (%) |
| --- | --- | --- |
| All cases | 198 | 105 (53.0) |
| ER+ cases | 109 | 52 (47.7) |
| ER-/HER2+ cases | 6 | 4 (66.7) |
| TNBC cases | 19 | 13 (68.4) |

**Table S3. Gene expression levels in Ivabradine treated and untreated cells**

**(see excel file Table S3)**

**Table S4. Down-regulated molecular mechanisms mediated by Ivabradine revealed by GESA**

|  | **Affected gene-set** | **NES** | **Number of genes affected** | **Number of genes in the gene set** | **P value** |
| --- | --- | --- | --- | --- | --- |
| Down-regulation | Proteasome | -1.5362 | 35 | 46 | 0.0130 |
|  | Protein export | -1.5303 | 20 | 23 | 0.0370 |
|  | ECM-receptor interaction | -1.5082 | 39 | 88 | 0.0082 |
|  | Calcium signaling pathway | -1.4849 | 103 | 240 | 0.0005 |
|  | Natural killer cell mediated cytotoxicity | -1.4164 | 54 | 130 | 0.0152 |
|  | NF-kappa B signaling pathway | -1.3546 | 63 | 104 | 0.0250 |
|  | PI3K-Akt signaling pathway | -1.3455 | 191 | 354 | 0.0006 |
|  | Beta-Alanine metabolism | -1.3404 | 21 | 31 | 0.0150 |
|  | JAK-STAT signaling pathway | -1.3341 | 72 | 162 | 0.0260 |
|  | TGF-beta signaling pathway | -1.3247 | 61 | 94 | 0.0420 |
|  | Pathways in cancer | -1.2875 | 326 | 531 | 0.0006 |
|  | RAS signaling pathway | -1.2805 | 129 | 232 | 0.0180 |
|  | MAPK signaling pathway | -1.2797 | 186 | 294 | 0.0068 |
|  | Breast cancer | -1.2624 | 95 | 147 | 0.0500 |

**Table S5. Gene and gene-sets in down-regulated pathways**

**(see excel file Table S5)**

**Table S6. Up-regulated molecular mechanisms mediated by Ivabradine revealed by GESA**

|  | Affected gene-set | NES | Number of genes affected | **Number of genes in the gene set** | P value |
| --- | --- | --- | --- | --- | --- |
| Up-regulation | Cell adhesion molecules | 1.633 | 41 | 149 | 0.0005 |
|  | Arachidonic acid metabolism | 1.552 | 20 | 61 | 0.0190 |
|  | Glycine, serine and threonine metabolism | 1.546 | 24 | 40 | 0.0150 |
|  | ATF6 mediated unfolded protein response | 1.538 | 71 | 171 | 0.0005 |
|  | Parathyroid hormone synthesis, secretion and action | 1.534 | 72 | 106 | 0.0005 |
|  | Protein digestion and absorption | 1.519 | 24 | 103 | 0.0210 |
|  | Cytokine-cytokine receptor interaction | 1.507 | 73 | 295 | 0.0010 |
|  | Central carbon metabolism in cancer | 1.490 | 46 | 70 | 0.0074 |
|  | Transcriptional misregulation in cancer | 1.436 | 100 | 192 | 0.0008 |
|  | Apoptotic signaling pathway in response to ER stress | 1.429 | 62 | 136 | 0.0085 |
|  | Hypertrophic cardiomyopathy | 1.410 | 40 | 90 | 0.0290 |
|  | Proteoglycans in cancer | 1.402 | 138 | 205 | 0.0006 |
|  | Gap junction | 1.393 | 48 | 88 | 0.0250 |
|  | Adherents junction | 1.374 | 55 | 71 | 0.0250 |
|  | Rap1 signaling pathway | 1.358 | 130 | 210 | 0.0026 |
|  | CAMP signaling pathway | 1.356 | 110 | 219 | 0.0060 |
|  | TNF signaling pathway | 1.328 | 81 | 112 | 0.0220 |
|  | Focal adhesion | 1.315 | 127 | 201 | 0.0082 |
|  | Regulation of actin cytoskeleton | 1.307 | 145 | 218 | 0.0076 |
|  | Endocrine resistance | 1.307 | 71 | 98 | 0.0420 |

Table S7. Gene and gene-sets in up-regulated pathways

(see excel file Table S7)

**Table S8. Conversion of Ivabradine and Paclitaxel dosage between human and mouse**

**^#^**Guidance for Industry Estimating the Maximum Safe Starting Dose in Initial Clinical Trials for Therapeutics in Adult Healthy Volunteers. U.S. Department of Health and Human Services Food and Drug Administration. Center for Drug Evaluation and Research (CDER). July 2005. Pharmacology and Toxicology

|  | Mouse dosage (mg/Kg) | Conversion factor^#^ | Human equivalent dose (HED; mg/Kg) | Frequency | Dose/week (mg/Kg/week) | Total intake in 60 Kg subject (mg)/week |
| --- | --- | --- | --- | --- | --- | --- |
| Ivabradine | 5 | 12.3 | 0.406504065 | Twice per week | 0.81300813 | 48.7804878 |
| Ivabradine | 1 | 12.3 | 0.081300813 | Twice per week | 0.162601626 | 9.75609756 |
| Paclitaxel | 2 | 12.3 | 0.162601626 | Twice per week | 0.325203252 | 19.51219512 |
|  |  |  |  |  |  |  |
|  | Recommended human dosage | Body surface area (For 183 cm, 80 Kg person) | Intake amount (mg) | Dosage (mg/Kg) | Fold change compared to Paclitaxel HED |  |
| Paclitaxel | 175 mg/m^2^ | 2.02 | 353.5 | 4.41875 | 27.1753125 |  |

**References**

1. Zhang, X.M., Claerhout, S., Prat, A., et al. A Renewable Tissue Resource of Phenotypically Stable, Biologically and Ethnically Diverse, Patient-Derived Human Breast Cancer Xenograft Models. *Cancer Res*. 2013;73:4885-4897.

2. Dobin, A., Davis, C.A., Schlesinger, F., et al. STAR: ultrafast universal RNA-seq aligner. *Bioinformatics*. 2013;29:15-21.

3. Leng, N., Dawson, J.A., Thomson, J.A., et al. EBSeq: an empirical Bayes hierarchical model for inference in RNA-seq experiments. *Bioinformatics*. 2013;29:1035-1043.

4. Li, B. & Dewey, C.N. RSEM: accurate transcript quantification from RNA-Seq data with or without a reference genome. *Bmc Bioinformatics*. 2011;12.

5. Kamburov, A., Pentchev, K., Galicka, H., et al. ConsensusPathDB: toward a more complete picture of cell biology. *Nucleic Acids Res*. 2011;39:D712-717.
