# Supplementary material for: Repurposing hyperpolarization‐activated cyclic nucleotide‐gated channels as a novel therapy for breast cancer
Source: Clin Transl Med. 2021 Nov 4;11(11):e578. doi: 10.1002/ctm2.578 (PMC8567035; doi:10.1002/ctm2.578)
Supplement: Supplementary file 3 — TableS3 [file CTM2-11-e578-s005.pdf]

gene_name	231U Normalised Read Counts	231 Iva24hr Normalised Read Counts	453U Normalised Read Counts	453 Iva24hr Normalised Read Counts	MCF10A Normalised Read Counts	MCF10A Iva24hr Normalised Read Counts
TSPAN6	639.750738	Figure	25.07335827	20.1140043	690.2103232	134.0970097
TNMD	0	0	0	0	0	0
DPM1	1206.24757	1365.14646	885.591014	764.332164	2228.825161	899.71845
SCYL3	195.343737	208.225464	1415.140341	1112.30444	335.5329017	1270.297348
C1orf112	647.564487	672.41589	373.091571	491.787405	721.4461189	786.4608405
FGR	0	0	0	0	0	0
CFH	174.832644	169.627573	0	0	1.007606311	3.624243505
FUCA2	2381.24015	2806.47297	912.6702409	1651.35975	3460.120073	2118.370329
GCLC	480.545592	551.543547	997.9196591	934.2955	1440.877025	1383.554958
NFYA	2340.21797	2489.56397	1204.524131	1008.71732	1076.12354	1264.860983
STPG1	281.294981	269.169503	248.727714	184.043139	106.806269	129.5667053
NIPAL3	1747.34973	1452.49958	1387.058179	1395.9119	349.63939	926.9002763
LAS1L	1751.2566	1818.16381	2033.950823	1455.24821	1883.216196	1434.294367
ENPP4	1033.36837	1182.31434	208.6103408	240.362352	495.7423052	374.2031419
SEMA3F	345.758414	366.679964	4975.557215	6719.08314	2029.319111	7070.899078
CFTR	0	0	0	0	4.030425245	0.906060876
ANKIB1	2499.42311	2323.99933	1933.65739	2182.36947	3048.009092	2671.067463
CYP51A1	2026.6815	5187.32292	1476.319335	19038.418	3650.54759	1989.700623
KRIT1	808.977017	841.423865	873.4855966	1036.95738	944.6712211	982.3240201
RAD52	203.450502	170.033867	379.109177	308.096261	170.2854666	273.1411117
MYH16	55.672965	101.573397	2.005868661	0	13.09888205	6.342426133
BAD	2005.20346	1996.93299	1468.29586	1485.41922	805.0774427	1388.085262
LAP3	1475.82193	1732.84216	626.8339567	814.617175	1438.861813	1883.700562
CD99	2530.67811	2342.28254	1848.909439	1513.07597	788.4519386	751.5774968
HS3ST1	167.018895	182.832115	12.03521197	20.1140043	18.1369136	31.71213067
AOC1	0	0	0	0	3.022818934	1.812121752
WNT16	0	0	0	0	0	0
HECW1	37.11531	54.8496345	0	0	21.15973254	22.6515219
MAD1L1	1065.60008	1334.67444	853.0558243	482.736103	822.20675	631.5244307

LASP1	25672.0739	26619.3402	6451.876549	7066.04971	8796.403098	8950.975396
SNX11	509.847153	638.896669	499.4612967	610.460031	763.765584	660.5183787
TMEM176A	0	0	0	0	0	0
M6PR	2656.67482	3540.84863	3369.859351	4124.37658	3141.716479	3010.840292
KLHL13	9.76718684	7.11013781	2.005868661	1.00570022	147.1105214	17.21515665
CYP26B1	503.986841	360.58556	24.07042394	18.1026039	69.52483548	51.64546994
ICA1	36.1385913	34.5349551	1586.642111	1635.26855	733.5373946	1520.37015
DBNDD1	168.972332	120.872343	1288.770615	1246.06257	3174.967487	2074.879406
ALS2	1121.27305	1152.85806	726.1244554	1017.76862	885.6859476	858.9457106
CASP10	211.947954	291.51565	3.008802992	1.00570022	41.31185876	39.86667855
CFLAR	2333.99627	1946.76589	1634.782959	2030.53891	1145.40655	1900.915718
TFPI	2742.62606	2112.72666	1.002934331	0	686.179898	93.32427025
NDUFAF7	440.500126	406.293589	363.0622277	340.932373	428.2326823	425.8486118
RBM5	2466.51746	2368.69162	5111.956284	2794.36822	1887.246621	3215.963413
MTMR7	7.81374947	11.1730737	0	0	123.9355763	7.248487009
SLC7A2	847.791818	928.380851	0	0	14187.09686	1186.033687
ARF5	1458.241	1527.66389	3000.779517	3854.84893	4149.32279	2777.529616
SARM1	50.5842606	69.425417	271.1132083	87.1841517	240.8179084	133.834252
POLDIP2	3292.51868	3178.2316	2474.238994	2252.76848	3823.865951	3534.543478
PLXND1	1792.27878	1756.20404	566.6578969	997.654614	6217.938547	1662.621708
AK2	3024.30197	2716.57035	3507.853086	2403.8146	2062.832097	3655.502605
CD38	6.83703079	0	0	0	2.015212623	0
FKBP4	3203.63728	3237.14417	6437.835469	7278.25246	13024.31918	10310.06671
KDM1A	2879.36668	2518.00452	2611.640997	2299.03069	2647.989386	3729.346566
RBM6	2081.08473	2215.31579	4307.60295	3633.06186	1187.967841	2924.411145
CAMKK1	182.646394	227.52441	109.319842	95.5415205	171.2930729	152.2182272
RECQL	1594.00489	1735.88936	734.1479301	1135.43554	1123.481037	1011.163938
VPS50	505.940278	542.401941	637.8662343	578.277624	528.9933134	577.1607781
HSPB6	4.88359342	4.06293589	3.008802992	6.03420129	1.007606311	2.718182629
ARHGAP33	313.526698	311.830329	430.2588279	855.850883	177.3387108	317.1213067
NDUFAB1	1005.04353	1003.54516	1612.718404	1591.01774	2262.076169	1747.79143
PDK4	19.5343737	18.2832115	0	0	65.49441023	14.49697402
SLC22A16	0	0	0	0	1.007606311	0

ZMYND10	14.6507803	13.2045416	56.16432252	53.3021114	10.07606311	79.7333571
ABCB5	0	0	0	0	0	0
ARX	0.97671868	0	15.04401496	13.0741028	51.38792188	11.77879139
SLC25A13	1221.87507	1546.96284	618.810482	1300.37038	1391.504316	997.5730247
ST7	545.546221	625.692127	555.6256192	690.916048	480.6282105	501.9577254
CDC27	6242.20911	6203.08737	2572.526558	2972.84984	3304.948701	3313.464624
SLC4A1	0	0	0	0	2.015212623	0
CALCR	1.95343737	4.06293589	0	0	86.65414277	3.624243505
HCCS	1136.90055	1201.61329	871.5499334	914.181496	872.5870656	821.7972147
DVL2	1780.55816	2045.68822	712.0833748	1936.97862	1110.382155	714.8820313
PRSS22	35.1618726	29.4562852	380.1121113	620.517033	487.6814547	371.4849592
UPF1	5555.57587	4522.04764	4538.277846	3075.43126	6950.468335	5476.231936
SKAP2	617.286208	782.115159	0	0	865.5338214	92.41820937
SLC25A5	8150.71742	8822.66528	9174.843257	15849.8354	18844.25323	10763.09715
CCDC109B	771.60776	793.288232	826.4178885	902.113093	520.9324629	606.1547262
HOXA11	0	0	0	2.01140043	2.015212623	4.530304381
POLR2J	834.508443	890.036893	472.8033022	393.168442	1755.219966	471.8855649
DHX33	2195.6636	1973.57111	321.9419202	350.989375	2118.996073	702.197179
MEOX1	3.90687474	4.06293589	0	0	1.007606311	0
THSD7A	0	1.01573397	0	0	0	0.906060876
LIG3	1427.96272	1241.22691	2823.260141	2436.81162	1127.511462	3160.340336
RPAP3	1024.5779	997.450761	1147.356874	978.54631	1452.968301	1118.985182
ACSM3	0.97671868	5.07866986	34.09976724	3.01710065	7.053244179	38.96061768
AC004381.6	122.089835	84.3059197	377.1033083	357.023577	402.0349182	279.0667499
CIAPIN1	1000.15993	917.207777	1284.758878	1204.82886	1451.960695	1678.930804
SPPL2B	976.718684	912.129107	1641.803499	1134.54047	751.6743082	1585.606533
FAM214B	620.216364	819.697316	733.1449957	898.090292	241.8255147	634.2426133
COPZ2	17.5809363	19.2989455	6.017605984	7.03990151	55.41834712	20.83940015
PRKAR2B	286.178574	360.58556	114.3345137	49.2793106	373.8219415	87.88790499
MSL3	1012.85728	1080.74095	766.2418287	878.981988	419.1642255	829.0457017
CREBBP	3516.18726	3490.06193	2736.004854	4054.98327	6734.840585	4734.168078
BZRAP1	60.5565584	102.589131	0	0	1.007606311	0.906060876
MPO	0	0	0	0	0	0

PON1	0	0	0	1.00570022	0	0.906060876
GCFC2	864.396035	851.185069	741.1684704	708.012952	392.9664614	808.2063016
WDR54	791.142134	818.681582	352.0299501	498.827307	761.7503713	518.2668212
CROT	179.716238	276.27964	652.9102493	440.496694	766.7884029	2345.791608
ABCB4	0	0	4.011737323	4.02280086	17.58273013	0
KMT2E	1800.09253	1561.18312	1118.271779	1439.15701	2650.004599	1392.615567
RHBDD2	636.820582	648.038274	1586.642111	1670.46806	1401.580379	1744.167187
SOX8	0	0	0	0	11.08366942	1.812121752
IBTK	2360.72906	2112.72666	2576.538296	2075.76524	2455.536581	2173.640042
ZNF195	625.099958	553.575015	906.652635	772.377766	569.2975659	831.7638843
MYCBP2	3835.57427	3618.04441	1682.923807	929.266999	3750.310691	2379.315861
FBXL3	1269.73429	1227.00664	1154.377415	713.041453	1161.770077	1053.748799
ITGAL	0	0	126.3697257	80.4560172	7.053244179	192.9909666
PDK2	756.95698	716.09245	1050.072244	1293.33048	580.3812353	1277.545835
ITGA3	34467.4256	36065.6662	2917.535968	5318.14274	3725.120533	4073.649699
ZFX	2058.92299	2040.60955	1067.122128	1465.30521	1377.397828	1302.91554
LAMP2	4212.58768	4594.16476	6654.469284	9090.52425	5483.393546	6185.677602
ITGA2B	9.76718684	11.1730737	0	1.00570022	69.52483548	0
ASB4	0	0	0	0	0	0
GDE1	1828.41738	2238.67767	3516.287763	3525.98496	2787.039057	3615.182896
C19orf60	623.14652	670.384422	756.2124854	771.372065	636.8071887	691.3244485
CRLF1	0.97671868	4.06293589	0	4.02280086	337.5481143	30.80606979
OSBPL7	247.109827	275.263906	949.7788112	902.113093	393.9740677	885.221476
TMEM98	34.1851539	25.3933493	0	0	6.045637868	0.906060876
YBX2	0.97671868	3.04720192	2444.150964	1705.66757	795.0013796	1432.482245
KRT33A	0	0	0	0	0	0
MAP3K14	1174.99258	981.199017	96.28169575	213.208446	428.2326823	249.1667409
ABCC8	0	0	61.17899417	215.219846	28.21297672	17.21515665
CACNG3	0	0	0	0	0	0
TMEM132A	3046.38557	3588.58812	6186.098952	7798.19947	4694.437804	5167.265177
AP2B1	16196.9259	15211.632	6465.91763	6561.18821	4229.931295	7603.662873
TAC1	0	0	0	0	0	0
ZNF263	706.167608	667.33722	1567.586359	1247.06827	1242.378582	1407.112541

CX3CL1	31.2549979	57.8968364	0	0	12.09127574	0.906060876
SPATA20	1879.20675	2016.23193	1772.184962	2003.35483	1973.900764	1919.743663
CACNA1G	1.95343737	6.09440383	3.008802992	2.01140043	6.045637868	3.624243505
TNFRSF12A	2625.41982	2976.10054	769.2506317	1136.44124	2986.545107	1537.585307
DLX6	0	0	0	0	0	0
MAP3K9	1299.03585	1416.94889	1637.791762	2031.51444	1565.820208	2520.661358
RALA	1469.96162	1569.30899	1513.427905	1686.55926	1343.139213	1341.876158
BAIAP2L1	1048.99587	854.232271	291.8538902	292.658763	985.4389724	644.209283
KDM7A	320.363728	338.239413	943.7612052	874.959188	454.4304464	891.5639022
ETV1	955.230873	1392.57128	27.07922693	53.3021114	68.51722917	52.55153082
AGK	995.276339	969.01021	768.2476973	548.106617	1219.203637	847.1669192
ALDH3B1	1014.81071	724.218322	103.4827642	133.013911	464.2143037	132.4661001
TTC22	11.7206242	14.2202756	1.002934331	0	40.30425245	5.436365257
PHTF2	1622.32973	1633.30023	633.854497	1115.32154	1593.025578	1159.757922
CCL26	32.2317166	40.6293589	0	0	35.2662209	9.966669638
FARP2	1168.15555	1248.33705	799.3386616	1054.97953	2142.171018	1376.306471
USH1C	0	0	0	0	1.007606311	0.906060876
GGCT	988.439308	1147.77939	7689.487484	6410.67511	2887.739232	4458.70745
DBF4	1272.61561	1417.86305	816.3383985	1435.11409	1825.520658	1289.270263
TBXA2R	102.555462	161.501702	5.014671654	4.02280086	21.15973254	8.154547886
IFRD1	1402.56803	1558.13591	1156.383283	661.750742	2312.456484	1361.809497
LGALS14	0	0	0	0	0	0
COX10	477.615436	519.04006	136.399069	164.934835	571.3127785	230.1394625
GTF2IRD1	948.393842	965.963008	909.661438	780.423367	947.1499326	847.1669192
PAF1	2218.12813	2188.90671	1707.997165	2090.85075	2015.212623	2193.573381
VPS41	790.165415	1017.76544	1164.406758	1256.11957	1349.184851	1001.197268
ARHGAP44	0.97671868	4.06293589	97.28463008	38.2166082	704.3168116	140.4394358
ELAC2	3328.65727	3237.14417	1886.519476	1618.17165	3320.062796	2186.324894
SCIN	0	1.01573397	0	0	1809.660935	35.33637417
ARSD	126.973429	178.769179	0	0	1947.703	163.9970186
PNPLA4	241.249515	243.776153	682.9982792	297.687264	38.28903983	247.3546192
MYH13	0	0	0	0	0	0
ADIPOR2	2618.58279	2875.54288	1251.662045	1783.10648	4409.285218	1483.221654

CDKL3	27.6899747	22.1531579	28.65383383	29.7787834	15.20477924	17.32388395
UPP2	0	0	0	0	0	0.906060876
PRSS21	0	0	0	0	0	0
MARK4	723.748545	704.919377	1485.345744	1222.93146	866.5414277	1336.439792
PROM1	0	0	14.04108063	4.02280086	1.007606311	5.436365257
CCDC124	2470.12155	2572.85415	1234.612161	1249.07967	2002.113741	1462.382254
CEACAM21	0	0	1.002934331	0	1.007606311	0
PAFAH1B1	4833.76123	4508.03052	1325.076838	1737.84997	3545.998359	2043.167276
NOS2	0	0	0	0	0	0.906060876
DNAH9	0	0	0	0	0	0
KIAA0100	13975.8676	14662.1199	7622.300913	9529.00954	12032.83457	14791.4438
SLC13A2	0	0	0	0	0	1.812121752
GAS7	3.90687474	14.2202756	0	0	0	0
TRAPPC6A	20.5110924	35.550689	563.6490939	893.061791	379.8675794	348.8334373
MATK	0.97671868	3.04720192	0	0	507.8335809	6.342426133
CEACAM7	0	0	0	0	1.007606311	4.530304381
CD79B	0	0	0	0	0	0
SCN4A	0	2.03146794	0	0	0	2.718182629
ST7L	337.944665	356.522624	402.1766666	322.829769	219.6581759	388.7001159
TKTL1	0	0	0	0	0	0
PAX6	0	0	5.014671654	0	0	0
RPUSD1	774.537916	913.144841	901.6379633	1207.84596	2144.18623	1404.394358
RHBDF1	718.864951	663.274284	1488.354547	1816.29459	1208.119967	1259.424618
LUC7L	1186.7132	1138.63778	1417.146209	1385.8549	1464.05197	1591.948959
CACNA2D2	0.97671868	0	150.4401496	84.4788181	42.31946507	128.6606444
BAIAP3	62.5099958	114.777939	159.4665586	160.912034	280.1145545	252.7909845
TSR3	1112.48258	1093.94549	1375.022967	1337.58129	1713.938336	1268.485227
PIGQ	660.545079	751.663454	2988.744306	2499.15498	1107.953824	2628.845026
CRAMP1	1047.95078	978.375277	1690.305404	1996.38533	1772.520566	2351.191731
TEAD3	2069.66689	1855.74597	1721.035311	2519.27904	808.1002617	2146.458216
SELE	0	0	0	0	0	4.530304381
DNAJC11	1869.43956	1849.65156	1767.170291	1551.79543	1678.672115	2334.012817
FMO3	0	0	0	0	1.007606311	0

MYLIP	529.381527	121.888077	1157.386218	365.069178	606.5789994	1258.518557
NOX1	0	0	0	0	1.007606311	3.624243505
E2F2	271.527794	272.216705	430.2588279	1014.75152	597.5105426	1967.964223
PSMB1	4978.33513	4828.7993	3321.718503	2693.26518	4777.061522	3351.519181
SYN1	1.95343737	2.03146794	0	1.00570022	13.09888205	0.906060876
JARID2	1490.47271	1739.95229	1053.081047	1191.75476	1827.797849	1312.88221
CDKL5	251.016702	277.295374	273.8010723	310.761367	380.8751857	474.7758991
CAMK1G	2.93015605	5.07866986	0	0	10.07606311	0
CDK11A	725.91686	614.549525	996.1745533	993.390445	603.5662565	1088.532476
NADK	2001.29658	1900.43826	2410.051197	2378.48101	1585.972334	3050.70697
TFAP2B	0	0	11026.26003	10623.2114	0	18355.88729
TFAP2D	0	0	0	0	0	0
DLEC1	1.95343737	5.07866986	13.0381463	15.0855032	7.053244179	9.966669638
CYTH3	2692.81341	1915.67427	80.23474646	40.2280086	547.130227	137.7212532
ADAM22	196.320455	231.587346	570.6696342	490.781705	284.1449798	432.1910379
SYPL1	2603.93201	2724.19851	834.4413631	1196.78326	3512.515601	1430.670123
CYB561	1522.70443	1473.82999	7440.7698	6134.77131	10283.63001	6363.265533
SPAG9	4476.30173	4783.09128	1616.730141	2716.39628	4042.516521	2135.585485
CELSR3	596.775116	666.321486	3317.706766	3670.80579	1180.914597	3318.900989
AASS	785.281822	678.510293	142.416675	81.4617175	77.58568597	86.98184411
PLEKHG6	0	0	103.3022361	84.4788181	721.4461189	187.5546014
SS18L2	410.221847	442.860012	479.4026101	456.587898	286.1601924	427.6607336
MPND	214.87811	199.083859	317.9301828	488.770305	474.5825726	315.3091849
MGST1	1058.76305	1633.30023	4916.384089	6148.85112	3074.206856	3125.003962
CRY1	1838.18456	1578.45059	952.7876142	708.012952	1041.864926	937.7730068
PGLYRP1	0	0	0	0	0	0
NFIX	3006.34011	2827.80338	3576.463823	2552.46715	464.5065095	4945.280262
ST3GAL1	924.952594	1144.73219	3199.360515	1342.60979	4686.376954	1578.358046
MMP25	3.90687474	3.04720192	26.0762926	30.1710065	19.14451991	23.55758278
IL32	1080.25086	1048.23746	1.002934331	1.00570022	125.9507889	37.14849592
PKD1	2176.93014	2026.67368	7151.172512	3069.78928	2496.546157	3451.910726
MAPK8IP2	32.2317166	18.2832115	490.4348877	289.641662	356.6926342	403.1970899
MED24	2220.08157	2330.09373	2566.508952	2577.60965	1508.386648	3387.761616

RHOBTB2	324.270603	473.332031	195.5721945	277.573259	1117.435399	400.4789073
HEATR5B	906.394939	964.947274	1480.331072	2376.46961	1128.519069	2096.624867
SEC62	3548.41898	3703.36606	8542.994629	5629.90981	3158.845786	5756.204746
RPS20	6501.02979	6736.3477	11859.6784	9792.21135	9888.456894	7835.614457
CSDE1	18887.7859	18347.2027	12358.15682	11931.6274	82183.39357	18932.14201
UBE3C	6549.87549	6160.42654	3773.038952	3974.52725	6308.623115	4947.998445
REV3L	1386.94053	1180.28288	1507.410299	2026.48593	2609.700346	2935.637239
TENM1	1.95343737	5.07866986	68.19953449	101.575722	0	35.33637417
PAX7	0	0	90.26408976	171.974737	0	26.27576541
MASP2	0	2.03146794	2.005868661	2.01140043	2.015212623	17.21515665
IYD	0	0	11.03227764	4.02280086	1.007606311	2.718182629
FAM76A	210.971236	171.659041	263.771729	441.502395	131.9964268	296.2819065
TRAF3IP3	0	1.01573397	1.002934331	0	0	0.906060876
POMT2	1084.15774	1241.22691	604.7694014	1099.23034	1105.344123	1007.539694
VTA1	1181.82961	1323.50137	1141.339268	1032.85412	1877.170558	1289.324627
MLXIPL	86.9279629	100.557663	0	0	83.63132384	12.68485227
BAZ1B	5801.70898	5604.82006	4264.476774	4794.17293	5925.732717	6201.986697
RANBP9	2554.11936	2721.15131	2114.185569	3452.56884	1437.854206	3322.525233
ETV7	17.5809363	10.1573397	3.008802992	5.02850108	7.053244179	17.21515665
SPRTN	685.656516	699.840707	735.1508644	709.018652	524.9628882	828.1396408
METTL13	1137.87727	1207.70769	1370.008296	1933.96151	2166.353569	1962.527858
DYRK4	183.212891	186.570016	510.8446013	326.85257	90.93646959	261.4348052
ZNF207	5612.22556	5139.6139	4438.987348	3967.48735	3900.444031	5167.265177
UQCRC1	3635.34694	3834.39575	4300.58241	4179.6901	2759.833687	5007.798463
STARD3NL	858.535723	1023.85984	498.4583624	594.368827	552.1682586	448.5001337
CD9	3255.40337	2724.19851	14921.65697	10473.362	5819.934054	7517.58709
HHATL	0	0	0	0	1.007606311	0
NCAPD2	7646.73058	7554.01355	1592.659717	4021.79516	6581.684425	4716.952921
IFFO1	55.672965	22.3461474	6.017605984	5.02850108	2.015212623	12.68485227
GIPR	36.1385913	43.6765608	99.29049874	62.3534134	8.06085049	69.76668747
PHF7	278.364825	407.309323	87.25528677	134.763829	59.44877237	112.3515486
SEMA3G	34.1851539	18.2832115	26.0762926	13.0741028	4.030425245	70.67274834
NISCH	2015.94736	1800.89633	4075.92512	2559.50705	1045.895351	3158.528214

STAB1	0	0	1.002934331	2.01140043	0	4.530304381
FUZ	104.508899	109.699269	418.2236159	422.394091	155.1713719	460.2789251
SLC6A13	2.93015605	2.03146794	0	0	0	0
IDS	4103.21473	4536.59295	3187.184892	3383.12524	3534.310126	3165.704216
PRSS3	83.9978068	106.652067	2.005868661	0	1.007606311	3.624243505
ZNF200	295.945761	286.43698	310.9096425	255.447855	483.6510294	368.7667766
CD4	0	0	0	0	0	0
LRRC23	38.3752771	49.9030101	280.570879	202.306655	84.4877892	135.5195252
BTK	0	0	1.002934331	0	1.007606311	1.812121752
HFE	383.850443	496.693912	693.0276225	878.981988	88.66935539	542.7304648
SCMH1	1166.20211	1013.7025	1290.776484	1328.52998	704.3168116	1259.424618
FYN	1344.94163	1049.25319	9.026408976	4.02280086	100.7606311	30.80606979
HIVEP2	1502.19334	1603.84394	1313.843973	950.386704	1076.12354	1549.364098
FMO1	0	0	0	0	0	1.812121752
TCEB3	1924.13581	1896.37533	2099.141554	2417.70332	2019.243048	3758.340514
LYPLA2	1174.99258	1132.54338	1092.195486	1134.42984	951.1803579	1619.121725
CLCN6	622.892573	966.470875	595.4822295	2994.37182	237.7950895	616.7828202
MRC2	1826.46394	2198.04832	111.3257107	62.3534134	601.5409678	459.3728642
NME1-NME2	244.238274	214.594116	163.2676797	341.807332	231.5882346	530.2087035
SLC6A7	1.95343737	6.09440383	0	0	1.007606311	0
TSPAN9	1889.95065	1485.00307	1265.703125	1370.76939	375.8371541	1358.185253
BTBD7	2158.54829	2118.82107	1533.486592	2042.57714	2759.712774	2506.164384
APBA3	693.470266	644.991072	428.2529592	413.342789	265.0004599	496.5213601
MKS1	245.15639	211.272666	307.9008395	226.282548	691.2179295	314.403124
ABHD5	1060.71649	916.192043	490.4348877	550.118018	547.130227	540.9183431
ANOS1	509.847153	533.260335	58.17019118	82.4674177	44.3346777	18.12121752
AKAP8L	1275.5946	1404.76008	2906.50369	3334.90191	1206.104755	3527.294991
MBTD1	515.707465	479.426435	1034.025295	703.990151	1057.986627	1089.991234
UTP18	2391.98406	2585.04296	1092.195486	1162.58945	2723.559859	1226.806426
RNF216	1922.15307	1960.29547	1617.20152	1427.27969	2435.706888	1898.387808
TTC19	1648.70114	1553.21976	2130.633692	1336.57559	1358.253308	2080.74162
PTBP1	14779.7071	14644.8524	7546.077904	9131.75796	15340.80609	11745.26714
DPF1	224.645297	238.697483	69.20246882	115.655525	218.6505695	183.024297

SYT7	22.4645297	22.3461474	244.7159767	375.12618	1228.272093	753.842649
LARS2	1374.24319	1322.48563	870.5469991	751.258061	640.837614	1394.427688
PIK3C2A	2586.35107	2400.17938	862.5235244	1596.04624	2413.217116	1373.588288
PLAUR	2106.7822	3170.10573	38.11150457	22.1254047	150.1333404	102.384879
ANLN	12668.0413	10679.427	760.2242227	2287.96799	5886.436071	2809.694777
WIZ	2388.07718	2302.66892	2132.238387	2295.00789	3433.922309	3366.922216
RABGAP1	1655.53817	1659.70931	2663.793582	2212.54047	2848.503042	1965.24604
DCN	0	0	0	0	0	0.906060876
QPCTL	501.056685	534.276069	375.0974397	632.585436	292.2058303	305.3425153
PPP5C	2887.18043	2692.71076	2278.666799	2078.78235	2272.152232	2401.061322
CEP68	383.850443	318.940467	421.2324189	409.319988	584.4116605	463.9031686
MAP4K3	1093.92493	1055.3476	894.617423	1194.77186	1695.801422	1410.736784
ZBTB32	2.93015605	6.09440383	2.005868661	2.01140043	1.007606311	2.718182629
TYROBP	0	0	0	0	0	0
TMEM159	636.820582	681.557495	1006.946068	1039.89402	583.4040542	1143.448826
GABRA3	0	0	3.008802992	1.00570022	1.007606311	8.154547886
BRCA1	1643.81754	1599.78101	404.1825353	449.547996	2325.555366	928.7123981
ERCC1	2112.64251	2154.37176	1703.985428	1479.38502	689.2027169	1292.042809
CD22	171.902488	368.711432	0	0	73.55526072	33.52425242
SEMA3B	1265.82741	546.464877	245.718911	210.191345	734.5450009	262.7576541
MBTPS2	1238.7137	1177.23567	1282.913478	1225.59657	1126.675149	1094.612145
PRICKLE3	328.177478	377.853038	535.5669326	695.944549	543.0998018	405.9152725
LTF	2.93015605	3.04720192	1.002934331	1.00570022	0	1.812121752
EXTL3	1532.47161	1556.10445	329.9653948	343.949474	1206.104755	411.3516378
NR1H4	0	0	0	0	0	0.906060876
ELOVL5	7358.59856	8017.18824	2712.937365	4747.91072	1820.744604	3940.45875
ALOX5	42.9756221	36.566423	0	0	24.18255147	28.08788716
KDM5D	0	0	0	0	0	0
CALCOCO1	607.519021	636.865201	1193.491854	1273.21647	267.0156725	558.1334997
UBR7	2554.11936	2524.09892	2767.06573	2679.13509	3084.696037	3600.522831
MAP4K5	3522.04757	3025.8715	1179.450773	985.586211	1193.005873	1284.794322
EHD3	58.603121	104.620599	224.6572901	276.567559	404.0501308	291.7516021
PSMC4	2972.15495	3000.47815	3787.080033	2616.83196	4085.843592	3346.988877

MAN2B2	1091.97149	1285.91921	1255.673782	1334.56419	929.013019	1378.118593
SLC7A14	0	1.01573397	0	0	0	0.906060876
CLDN11	2.93015605	0	4.011737323	2.01140043	5.038031556	15.4030349
SLC25A39	3297.40228	3558.1161	1977.7865	1875.6309	7509.689838	2541.500758
MVP	2890.11059	3083.69724	535.5669326	226.282548	545.1150144	791.8972058
NUB1	2238.63922	2046.70395	1303.81463	1057.99663	1146.655982	1391.709506
PGM3	2161.47845	2431.66713	2954.644538	1385.8549	1233.310125	1424.327697
RWDD2A	167.018895	194.005189	531.5551953	301.710065	195.4756244	357.8940461
CLK1	1316.61679	1056.36333	1717.023574	2916.53063	809.107868	1425.233758
POLR3B	648.541206	622.644925	445.3028428	483.741804	700.2863863	628.8062481
ANGEL1	380.695641	340.687332	1275.852821	1295.47262	1516.689324	1759.651767
RNF14	1350.80194	1529.69536	1783.21724	1498.49332	1243.386188	1360.903436
DNASE1L1	519.61434	510.914188	3516.287763	4067.05167	1056.979021	2798.822047
DDX11	1450.74956	1502.25023	780.0221464	1054.37611	1562.263357	1461.748012
HEBP1	1024.5779	1100.03989	989.8961844	1372.78079	672.0734096	756.5608316
GPRC5A	10335.6371	4963.89192	215.6308811	325.84687	6752.977498	2848.655395
MAMLD1	2118.50283	2113.7424	544.5933416	478.713303	354.6774216	432.1910379
CD6	0	0	0	0	0	0
TACC3	4183.28612	3766.34157	1168.418495	1803.22049	3488.33305	3059.767579
UFL1	416.082159	440.828544	1298.799958	1337.58129	1232.302519	1309.257966
POLA2	924.952594	797.351168	893.6144887	929.266999	1600.078822	1592.85502
ZC3H3	658.308393	678.510293	1109.24537	1282.26777	912.891318	1125.327608
CAPN1	5724.54821	5522.54561	2732.996051	4446.20065	7156.020023	4398.925554
ACPP	0	0	63.18486283	38.2166082	29.22058303	65.23638308
MDH1	3617.76601	3931.90621	3260.539509	3084.48256	3031.887391	3008.122109
SLC30A9	1494.37959	1381.3982	2060.027115	1546.76693	1308.880598	2291.427956
MTMR11	362.362632	335.192211	173.5076392	383.171782	315.3807754	214.7364277
COX15	742.3062	642.959604	1163.403824	902.113093	1292.758897	1351.842827
CCDC88C	1638.93395	1589.62367	461.3497921	500.838707	1222.226456	1110.830634
YAF2	293.015605	290.499916	440.2881712	457.593598	311.3503502	420.4122465
ZMYND11	3086.43104	2802.41003	3327.736109	3227.29199	5193.202928	3334.304024
WAS	11.7206242	16.2517436	18.05281795	16.0912034	27.2053704	13.59091314
DPEP1	0.97671868	3.04720192	0	0	0	0

BID	841.931505	809.539976	189.5545885	159.906334	1385.458678	660.5183787
MATR3	9211.21903	7808.10956	7274.122231	6916.74346	8149.751595	8351.027187
NPC1L1	0	3.04720192	0	0	14.10648836	3.624243505
XYLT2	875.139941	932.443787	2093.123948	1980.22372	1491.257341	2107.497598
RGPD5	636.566635	389.879328	33.87912169	70.006792	746.847874	121.1131573
STMN4	0.97671868	0	0	0	0	0.906060876
NUDCD3	2405.65812	2341.26681	1779.205503	1488.43632	1896.315078	1668.964134
ISL1	0	1.01573397	3.008802992	4.02280086	0	0.906060876
CHDH	2.93015605	1.01573397	1744.102801	1387.8663	610.6094246	1822.088422
IL20RA	3.90687474	4.06293589	27.07922693	7.03990151	4.030425245	51.64546994
CLCA1	0	0	0	0	0	0
CLCA4	0	0	0	0	0	0
GLT8D1	1289.26866	1221.92797	996.9167247	716.058553	522.9476756	736.6274923
ATP2C1	2894.99418	2958.83306	1809.293533	2233.66018	2553.274393	2279.649164
IGF1	0	0	0	0	0	0
SLC38A5	0.97671868	1.01573397	2.005868661	1.00570022	2.015212623	0.906060876
RALBP1	4992.98591	4631.73676	2064.038853	2424.74322	3271.697693	2262.434008
RUFY3	476.638718	381.915974	699.0452285	814.617175	382.8903983	809.1123624
CNTN1	25.3946858	39.6136249	0	0	5.038031556	0
SLC11A1	0.97671868	3.04720192	36.10563591	50.2850108	4.030425245	18.12121752
WWTR1	2509.1903	2691.69503	509.49064	561.18072	554.1834712	813.6426668
AGPS	3240.75259	2950.70719	316.9272485	391.217384	91.69217433	350.6455591
ZNF806	0	0	0	0	0	0
CXorf56	504.96356	483.489371	633.854497	698.921422	961.256421	560.8154399
ATP1A2	0	0	0	0	1.007606311	2.718182629
TTC27	1073.41383	1100.03989	460.3468578	353.000776	825.2295689	621.5577611
ZNF582	0	0	53.15551953	49.2793106	0	46.20910469
VSIG2	7.81374947	3.04720192	0	1.00570022	1.007606311	0.906060876
PHLDB1	2552.16592	2596.21603	1826.343416	3452.56884	701.2939927	3072.452431
MARCO	0	0	0	0	0	0
CYP24A1	38.0920287	33.5192211	0	0	93.70738695	48.92728731
PRDM11	385.80388	369.727166	48.14084787	69.3933149	234.7722705	160.3727751
SYT13	0.97671868	5.07866986	0	0	13.09888205	1.812121752

SNAI2	1027.50806	1082.77241	0	0	46.34989032	9.966669638
CD74	5416.88182	4828.7993	1.002934331	0	200.5136559	135.9091314
HGF	0	0	0	0	4.030425245	0
ZRANB1	1741.77266	1368.28508	1114.380394	1281.7951	1219.606679	1203.593147
NCDN	777.468072	828.838921	2666.802385	2840.09741	1611.162492	3954.049664
ADGRA2	45.9057781	34.5349551	1.002934331	1.00570022	73.55526072	15.4030349
CCT8L1P	0	0	0	1.00570022	0	0
ZFP64	540.125432	583.0313	423.2382876	707.007252	906.8456802	639.6789786
MNAT1	1207.22429	1046.20599	909.661438	839.75968	742.6058514	749.3123446
SAMD4A	4511.4636	3260.50605	2.005868661	1.00570022	558.2138965	201.1455145
RUNX3	0	2.03146794	0	0	4.030425245	0.906060876
MRE11A	2211.2911	1885.20225	1792.243649	1564.86954	746.6362767	2159.143068
PLEKHB1	10.7439055	30.4720192	169.4959019	171.974737	352.662209	242.8243148
SERPINB1	2643.97748	1816.13234	952.7876142	876.970588	539.0693765	675.0153528
CYP3A43	0	0	12.03521197	14.079803	0	19.0272784
SLC7A9	1.95343737	5.07866986	1.002934331	0	3.022818934	0.906060876
SPAST	704.214171	784.146627	517.5141146	565.203521	863.5186088	736.6274923
NRXN3	0	0	33.09683291	261.482056	1.007606311	19.0272784
OSBPL5	418.035597	464.190425	5915.306683	5672.14922	421.1794381	4521.243772
AQR	2098.96845	2077.17597	2204.449659	1983.24083	2672.171938	2515.224992
CPS1	108.415774	94.4632594	740.1655361	627.556935	33.25100827	947.7396765
C8B	0	0	0	0	0	0
FHL1	736.445888	457.080288	0	0	954.2031768	141.3454967
RTFDC1	3246.61291	3281.64347	1934.660324	1911.94674	4230.777684	1869.203588
GABRA1	0	0	0	0	1.007606311	0
NLRP2	415.105441	483.489371	0	0	0	0
SLC45A4	251.99342	392.073313	548.6050789	519.947011	468.5369347	693.1365703
RNF10	5646.41071	5830.313	4418.928661	4731.81951	4458.657927	4883.668123
ZNF839	498.126529	417.466663	708.0716375	549.112318	560.2291091	755.6547707
ZDHHC6	1455.31084	1248.33705	927.7142559	863.896485	884.6783413	858.0396497
GRAMD1B	44.9290595	63.9912403	104.3051704	48.2736103	59.44877237	86.07578324
RNH1	5333.86073	5512.38827	5785.928154	5346.30235	3143.731691	4890.010549
NDUFS1	3306.19274	3017.90815	4066.898711	3337.91902	3651.625728	4089.052734

RB1CC1	2455.47077	2032.48368	2094.126883	2178.34667	1799.584872	1508.591359
ERP44	1240.43273	1548.99431	1178.447839	998.660314	1913.444385	769.2456839
ALAS1	1815.72003	1808.00647	1239.626833	1446.19691	837.3208447	1709.736873
BIRC3	2476.95858	2813.5831	47.13791354	34.1938073	336.540508	279.0667499
AKAP11	2658.62826	2332.1252	2426.098146	2799.8694	3608.238201	3788.240523
GLRX2	287.155293	307.767394	361.0563591	434.462493	446.3695959	416.788003
SNAPC1	2780.32741	2374.83681	309.7963854	269.598057	366.5167957	318.3535495
DERA	763.794011	753.674607	493.4436907	613.477131	538.0617702	426.7546727
STRAP	2722.11497	2853.19673	2815.236666	2577.60965	3688.846706	3329.77372
ABCC2	52.7428089	66.0227082	18.05281795	57.3249123	92.69978064	33.52425242
DEF6	660.26183	601.314512	331.9712635	274.556159	106.806269	449.4061946
PLEKHO1	379.943568	372.774368	43.12617622	23.131105	71.5400481	59.80001783
GCLM	2079.43408	2238.67767	1113.257107	1145.49255	1395.534741	1321.036757
UBR2	3760.36693	3434.19656	1515.433774	1810.26039	1477.150852	1894.573292
EHD2	5996.076	5190.4006	63.18486283	122.695426	637.814795	167.6212621
DEPDC1	2219.08532	2397.12202	896.6232917	1362.69362	1182.919733	1516.745907
CCDC28A	193.390299	174.706243	108.3169077	155.883533	102.7758438	128.6606444
RRAGD	804.816196	1090.89829	262.7687946	188.06594	234.7722705	135.9091314
HSF2	669.052298	600.298778	486.4231504	429.433992	385.9132172	486.5546905
PHF20	2720.16153	2534.25626	4306.600016	5010.39847	1141.617951	3555.382878
HSD17B6	19.5343737	30.4720192	39.1144389	56.3192121	39.29664614	48.92728731
NR1H3	294.969043	346.365285	404.1825353	453.570797	208.5745064	430.3789162
TYMP	180.028788	203.786707	1746.028435	1298.76126	54.15883923	1179.854352
NCAPH2	1281.45491	1295.06081	2221.499543	3036.20895	1066.047477	2738.115968
TOMM34	2809.04293	3150.80678	1057.092785	1024.80852	1216.180818	1277.545835
SEC63	2417.31037	2376.80734	5023.667974	2875.22652	2767.864309	4143.407326
KPNA6	3461.49102	3428.10216	2629.693815	2592.69516	2889.361478	3219.234293
VIM	53207.727	53008.1088	0	1.00570022	1754.242588	902.4366327
RTEL1-TNFRSF6B	1286.47525	762.623224	493.0023996	756.52793	358.254424	537.4028269
FAS	48.8359342	59.9283044	3.008802992	6.03420129	158.1941909	36.24243505
RNASET2	736.084502	929.396585	1275.732469	830.829062	1102.321305	1255.673526
CD44	17307.4551	23466.502	0	0	1855.003219	522.7971256
KCNG1	0	0	0	0	110.8366942	9.060608762

AGPAT4	249.063264	272.216705	0	1.00570022	8.06085049	9.966669638
SLAMF7	308.643104	285.421246	0	0	3.022818934	1.812121752
BTN3A1	648.384931	656.306349	261.0136596	139.359879	11.43633163	284.6752667
MIPEP	283.248418	294.562852	1163.403824	953.403804	329.4872638	1137.1064
PRKCH	244.179671	264.090833	1299.802893	4360.71613	668.0429844	791.8972058
INSRR	0	0	0	0	0	0
IFNGR1	767.700886	929.396585	578.6931088	610.460031	697.2635674	1226.806426
B4GALT7	516.684184	524.11873	456.3351205	350.989375	545.1150144	475.68196
SH2D2A	1.95343737	6.09440383	14.04108063	20.1140043	9.068456802	37.14849592
VRK2	479.568874	577.95263	357.0446217	570.232022	755.7047335	477.4940817
TNFRSF1B	366.269506	389.026111	0	0	17.12930729	7.248487009
VEZT	2452.54062	2330.09373	3023.847007	2339.2587	1755.250194	2493.479531
POU2F2	2522.86436	3858.77336	4.011737323	8.04560172	163.2322224	85.16972236
BRD9	2410.54171	2018.2634	816.6793962	675.971343	1196.39143	1154.049738
SNX1	2089.20126	1976.61831	3555.402202	3140.80177	2498.863652	3815.42235
TBPL1	305.712948	416.450929	275.8069409	263.493456	350.6469963	262.7576541
ARNTL2	2503.32999	2562.18894	0	2.01140043	711.3700558	67.04850484
BCLAF1	6031.64809	4404.30376	4332.245047	3481.31175	6556.403583	5612.249794
SLC39A9	2816.85668	3011.65123	4254.447431	4218.9124	1995.060496	5185.386394
ANK1	983.555715	685.620431	0	1.00570022	114.8671195	16.30909577
IBSP	0	0	0	0	6.045637868	2.718182629
TFB1M	167.995614	164.548904	302.8861679	239.356651	122.92797	316.2152458
RABEP1	2723.09169	2786.15829	4005.719717	4598.06139	2796.107514	4043.74969
HMGB3	1999.34315	1892.31239	1984.80704	3723.1022	5983.166276	3041.646361
NUP160	2770.95091	2593.16883	796.3298586	918.204297	4846.586357	1892.76117
BAK1	1759.05082	2124.885	779.279975	752.23359	1417.681928	1014.779121
MUSK	91.8115563	95.4789934	0	0	0	0
IKZF2	156.274989	106.652067	151.4430839	210.191345	469.5445411	298.0940283
GRN	5982.40194	6627.66417	1648.82404	3958.43605	4137.231514	3308.028259
FAM13B	1601.81864	1754.17257	807.3621362	814.617175	1229.2797	1081.836686
ARHGAP31	787.235259	789.225296	0	0	82.62371753	23.55758278
CENPQ	836.071193	726.24979	114.3345137	138.78663	246.8635463	183.024297
SARS	4347.37486	3414.89761	12691.13102	3083.47686	4273.258366	3671.35867

RANBP3	2821.74028	2605.35764	1568.589293	1374.79219	985.4389724	1957.091493
ARID4A	1224.80523	1134.57485	1959.733682	2455.91993	923.9749875	1793.094474
TSSC1	1019.69431	978.151815	919.6907813	828.696978	986.4465788	919.6517893
PNPLA6	4284.24953	4673.89987	879.4129385	1778.79203	1123.481037	1065.880954
IFT88	333.061071	330.113541	427.2500249	325.84687	240.8179084	286.3152369
ALG1	670.029017	742.501534	815.2251414	902.153321	809.3698456	1199.642721
ZCCHC8	1312.70991	1194.50315	1277.738337	993.631813	974.355303	968.5790766
ABCF2	2979.9687	3113.22462	1200.512394	1220.92006	3470.196136	2130.14912
CHPF2	2026.69127	2190.37952	1542.513001	914.161382	1521.48553	1400.770115
LRRC7	1.95343737	3.04720192	3.008802992	5.02850108	0	5.436365257
FUT8	2703.55732	3080.72114	2072.062327	3122.69917	2389.034564	2874.93116
UBA6	2643.00076	2420.49406	2904.497822	2590.68376	1862.056463	2935.637239
GAB2	337.944665	338.239413	118.346251	222.259748	566.2747469	144.9697402
ATP6V0A1	1234.57242	1692.2128	1104.230698	1634.26285	1073.100722	1833.867213
PIAS1	876.116659	789.225296	2059.024181	2464.97123	1176.884172	2929.294813
SLC4A7	1803.02269	1486.0188	475.3908728	581.294725	1685.725359	968.5790766
APBA2	894.674314	740.470066	0	0	181.369136	19.0272784
MAP2K3	2711.37107	3103.06729	1443.222502	1620.18305	1260.515495	1739.636882
EFCAB1	0	0	0	0	0	0
TMSB10	19519.7229	18602.152	7423.719916	10101.253	6416.43699	7202.277905
ASTE1	110.369211	137.124086	185.5428512	214.214146	243.8407273	177.5879317
RNF19A	1305.87288	1163.0154	3359.830008	3391.22113	1606.12446	3763.77688
PEX3	355.525601	403.246387	499.4612967	376.131881	493.6767122	601.5881793
GABARAPL2	658.308393	721.17112	1389.064048	1095.20753	1984.984433	1089.085173
MYOC	0	0	0	0	0	0
SH3YL1	196.320455	187.910785	2650.755436	2395.57791	1147.663589	1987.897562
FAM136A	2684.99966	2831.86631	1264.700191	1257.12527	3499.416719	1737.824761
VCL	13253.0958	12046.6049	2130.232518	3448.54604	2398.103021	4594.634703
DEPDC1B	1001.13665	953.7742	115.337448	331.881071	813.1382932	339.7728286
DAPK2	163.11202	140.171288	257.754123	208.179945	104.7910564	331.6182807
NSMAF	1506.10021	1805.975	519.5199833	732.149757	1350.192457	622.4638219
ADSS	2655.6981	2588.09016	1707.997165	1993.29783	1257.492676	2131.04612
STAP1	0	0	0	0	0	0

TIMP2	14925.0038	12739.0206	0	0	1829.813061	626.9941263
RFC1	1892.88081	1797.84913	1397.087523	1260.14237	1926.543267	2036.82485
TBC1D23	1785.44175	1567.27752	622.8222194	712.035753	995.5150356	775.58811
CUL3	2518.95749	2447.91887	2950.632801	3187.06398	3077.229675	3215.61005
MYOM2	15.6274989	18.2832115	0	0	0	0
OTC	0	0	0	0	0	0
CYP46A1	0.97671868	0	25.07335827	10.0570022	54.41074081	54.36365257
ZZZ3	1757.11691	1724.71628	994.9108561	1044.92252	1617.20813	1123.515486
SLC18A1	0	0	0	0	0	0
USP2	11.7206242	16.2517436	0	2.01140043	88.66935539	28.08788716
CASR	0	0	0	0	1.007606311	0
TUBG2	532.194476	469.309725	230.2436343	254.039874	315.2094824	556.2670143
RPL26L1	902.46853	875.34938	728.9828183	608.398345	1172.470856	645.0247378
FLT4	0	1.01573397	307.9008395	150.855032	47.35749663	223.7970364
NSUN2	4670.66875	4052.77855	2367.927955	1972.17812	3766.432392	3108.694866
FBXO42	624.123239	608.424649	900.635029	746.22956	650.9136771	917.8396676
MFAP3	814.583382	859.310941	456.3351205	518.941311	670.058197	665.954744
MRI1	738.399325	731.32846	2141.796351	1341.60409	871.5794593	1638.312094
METTL1	294.969043	288.448133	196.5751288	161.917735	648.8984645	426.7546727
HOXC8	3.90687474	6.09440383	224.6572901	134.763829	32.24340196	235.5758278
AGA	252.970139	297.610054	1534.489526	583.306125	299.2590745	440.3455858
PI4K2B	1341.3766	1038.08012	751.5889581	897.42653	1035.486778	1013.936484
BOD1L1	3859.99224	3491.07766	2396.010116	2033.52584	1765.326257	2154.612764
MAT2B	2087.24783	2169.60776	881.5792767	970.500708	1006.598705	987.606355
TLL1	0.97671868	0	0	1.00570022	0	0
EDC4	1908.71342	1858.88459	2783.072562	2963.37614	3269.40035	4094.715615
TRIO	17176.5748	12567.6764	2452.174439	3214.21789	4617.859725	3767.401123
VCAN	16.6042176	12.1888077	431.2617622	173.986137	1.007606311	77.92123535
CLEC16A	889.790721	953.7742	1486.348678	1372.78079	1502.34101	1784.939926
MSR1	0	0	0	0	0	0
CDH1	21.487811	7.11013781	1169.42143	1347.63829	24013.27361	4205.934587
SKIV2L2	1262.89726	1127.46471	2014.89507	1660.41106	2365.859619	2300.488565
DNAH5	184.599831	144.234224	208.6103408	109.621323	107.8138753	144.0636793

ZFYVE16	1879.20675	1707.44881	2154.302942	1973.18382	1843.91955	2240.688547
FAM65A	3479.07195	3406.77174	195.5721945	211.197045	2651.012205	518.2668212
C6	0	0	0	0	0	0
RAI14	6383.83332	5690.14171	466.3644638	1120.35004	767.7960092	726.6608227
SOX30	0	0	0	0	1.007606311	0
PNKP	993.322901	1165.04687	1217.562277	2108.95335	704.3168116	1145.260947
BEST2	0	0	2.005868661	2.01140043	0	0.906060876
PHLPP2	1050.9493	1109.1815	652.9102493	1149.51535	1812.683754	1091.803356
SPDL1	2462.3078	2229.53607	575.6843058	828.696978	1130.534281	926.9002763
STAU2	687.805297	742.471062	1312.459924	1576.78708	1055.558296	1359.335951
PQLC2	385.80388	539.354739	954.7934828	947.369603	258.954822	652.3638308
CTNS	489.336061	753.674607	170.4988362	334.898172	353.6698153	216.5485494
RTN4R	51.7660902	114.777939	275.8069409	731.144057	166.2550414	244.6364366
PHF23	1171.0857	1154.88953	727.1273898	956.420905	1125.49625	941.3972503
CDH10	0	0	0	0	0	1.812121752
INPP4A	1324.43054	1365.14646	757.2154197	672.813444	1137.587525	897.0002674
RAB27B	269.574357	473.332031	3299.653948	4036.88067	1076.12354	2325.858269
PSMA4	3788.69177	3866.89923	1771.182028	1645.32555	5532.766255	2125.618816
MYO16	2.93015605	16.2517436	0	0	32.24340196	28.99394804
LSG1	1337.12788	1455.54678	1699.973691	1564.86954	2305.40324	2312.267356
PARP3	680.772923	612.487585	480.4055444	498.827307	73.55526072	504.675908
TNC	1711.21113	2133.04134	3525.314172	3724.1079	224.6962074	8427.272209
THAP3	273.481231	237.68175	507.4847713	355.012176	206.5592938	308.0606979
FAM65C	67.3935892	79.2272498	541.5845386	1474.35652	36.27382721	342.4910112
TDP1	2108.73564	1880.12358	793.3210556	920.215697	1264.545921	1205.967026
AIFM2	1330.29085	1282.87201	86.25235244	58.3306125	918.9369559	147.6879228
C2orf83	0	0	0	0	0	0
SPATA7	68.3703079	103.604865	213.6250124	174.991838	559.2215028	189.3667231
MED17	1621.35302	1804.95927	1188.477182	1099.23034	642.8528266	1192.376113
RETSAT	1215.03804	1431.16917	1311.838105	2329.2017	593.4801173	1861.04904
CAPG	6001.93631	6705.87568	679.9894762	634.596836	441.3315643	610.6850305
AP2S1	3524.00101	3985.74011	3027.858744	2943.68453	2542.190723	3172.119127
USH2A	0	3.04720192	0	1.00570022	0	12.68485227

ZPBP	0	0	0	0	0	0
TG	0.97671868	1.01573397	11.03227764	10.0570022	0	21.74546103
ADAM28	58.603121	57.8968364	1.002934331	0	2.015212623	2.718182629
BARX2	0	0	2.005868661	1.00570022	119.905151	13.59091314
DCUN1D1	1206.24757	1320.45416	2077.076999	2099.90205	1853.995613	2402.873444
JADE2	3667.57866	3468.73152	164.4812302	230.305349	2538.160298	762.9032577
ZIC2	705.19089	652.10121	18.05281795	12.0684026	250.8939715	51.64546994
LCP2	3.90687474	7.11013781	0	0	0	0
TRIT1	426.826065	431.686938	204.5986035	180.020339	456.445659	338.8667677
ADRB1	9.76718684	10.1573397	0	0	3.022818934	0
GUCA2B	0	0	0	0	0	0
CUL7	2147.80439	2187.89098	1775.193765	1837.41429	1458.006332	1707.018691
CTNNA1	17451.0327	16371.6002	12791.42445	9233.33368	11607.62471	9597.902861
PHKA2	807.746352	650.069742	1952.713142	2053.63984	780.8948912	1720.609604
CNTLN	0	0	221.6484871	209.185645	36.27382721	116.881853
EPHA3	0	0	0	2.01140043	71.5400481	0.906060876
HSPA5	3008.5425	32907.8178	2588.568185	37847.4687	14191.08698	16655.83639
DSG2	5217.63121	5006.55275	899.6320946	1282.26777	2775.955388	1445.167098
GEMIN8	108.415774	120.872343	505.4789027	393.228784	179.3539234	300.8122109
OFD1	822.397132	929.396585	1094.201355	1176.66925	902.8152549	954.9881635
GPM6B	4.88359342	2.03146794	1.002934331	0	4.030425245	0.906060876
MAGEC2	0.97671868	0	0	0	0	0
PREX2	0	0	0	0	0	0.906060876
WDR37	753.050105	789.225296	980.8697754	917.198597	977.3781219	1114.454878
YTHDC2	1643.81754	1419.99609	1567.586359	1423.06581	899.792436	1753.227795
CTPS2	499.103247	492.630977	1066.119194	752.263761	1355.230489	849.8851019
ATP6V1H	958.161029	1037.06439	1447.234239	1773.04948	979.3933346	1106.30033
POLR2B	3801.38912	3802.90799	2323.798844	2963.79854	3704.968407	3554.476817
FAM214A	166.042176	187.910785	3121.131637	3798.52971	1863.06407	3942.270872
ARAP2	388.734036	410.356525	266.780532	359.034977	404.0501308	454.8425598
TPR	7244.32248	6542.34252	5330.595968	5209.52712	8222.0675	6247.289741
CP	5.8603121	8.12587178	0	1.00570022	42.31946507	358.800107
KIAA0556	416.082159	517.008592	1544.518869	1293.33048	1190.99066	1588.324716

DTNBP1	563.566681	607.408915	447.3087115	516.929911	432.2631075	588.0335086
XK	16.6042176	11.1730737	7.020540315	12.0684026	122.92797	19.0272784
ANO2	31.2549979	11.1730737	0	0	0	0.906060876
C12orf4	571.38043	618.581989	775.2682376	683.876147	353.6698153	660.5183787
SCML1	1181.82961	1277.79334	127.37266	513.91281	494.7346988	115.9757922
WWC3	5156.09793	4330.07392	9539.911354	6932.29159	2827.343309	7578.293168
ARHGAP6	0	1.01573397	119.3491854	17.0969037	2.015212623	42.58486118
FAM184B	1.95343737	3.04720192	1.002934331	4.02280086	4.030425245	4.530304381
MAP4	13402.5338	12027.306	5633.482136	4129.40509	5908.603409	4533.928624
GOPC	1427.96272	1369.20939	1119.274713	1053.97383	1255.477464	1224.088244
ROS1	1.95343737	3.04720192	0	0	1.007606311	0
USP28	1918.2755	2113.7424	751.1978137	947.369603	629.7539446	998.4790855
HDAC9	1403.54475	1647.5205	1.002934331	0	111.8443006	40.77273943
TSPAN17	2312.86984	2460.10768	1312.841039	1713.71317	2266.106594	1610.070177
NOP16	1244.3396	1174.18847	281.8245469	220.248347	1380.420646	566.2880476
CC2D2A	385.80388	481.457903	214.6279468	233.32245	232.7570579	205.6758189
RRM2B	977.695403	1076.67801	889.6027513	1468.32231	3569.949161	1603.727751
ZNF800	664.168705	590.141438	704.0599002	829.702678	1063.024658	1113.548817
TNFRSF17	0.97671868	0	1.002934331	0	0	0
SNX29	335.991227	395.120515	405.1854696	412.337088	653.936496	571.7244129
LMO3	0	0	5.014671654	0	4.030425245	2.718182629
MRPS10	4176.44909	4128.94844	984.8614541	728.126956	1477.070244	1218.642818
GUCA1A	0	0	0	0	0	0
RSF1	2628.34998	2364.62869	4469.336141	4390.26361	2687.88052	4938.910654
VPS13D	1421.12569	1262.55733	5332.601836	5950.72818	2696.354489	6569.847413
CELF2	939.603374	686.636165	0	0	33.25100827	9.966669638
FAM120A	7861.218	7139.46205	4427.95507	4181.37967	16243.06716	5022.78471
R3HDM1	1933.90299	2300.63745	1292.782352	1620.18305	3164.891424	2424.618905
COL9A2	452.220751	347.381019	40.11737323	252.430754	253.9167904	66.14244396
KITLG	134.787178	214.319868	1202.518263	2069.73104	2884.776869	959.5184679
ERCC8	322.199959	338.960584	202.6629402	163.929135	241.4829286	213.8303668
ADAMTS6	419.989034	425.592534	2.005868661	10.0570022	104.7910564	54.36365257
H6PD	3213.40447	2412.36818	1932.654455	1523.63583	1053.956202	1478.69135

VAMP3	2365.61265	2249.85075	1163.403824	1344.62119	1438.861813	1498.624689
PER3	390.687474	461.143223	3141.190324	5617.8414	500.7803367	2369.349191
UTS2	0.97671868	0	0	2.01140043	1.007606311	1.812121752
TNFRSF9	103.53218	63.9912403	2.005868661	0	3.022818934	4.530304381
EPN3	3.90687474	3.04720192	1642.806434	3391.22113	2528.084235	3414.236715
LTBP1	351.618726	537.323271	825.4149542	975.529209	1299.812142	1135.294278
RCN1	11377.6201	9841.87307	2567.662327	1040.75893	595.9286007	1425.822698
ELN	0	0	3.008802992	3.01710065	0	0
RFC2	1506.10021	1282.87201	1239.626833	1160.57805	1412.664048	1941.688458
ARID1B	1062.66993	1187.39301	2378.960232	2816.9663	1799.584872	3248.228241
CLPTM1L	5156.09793	5110.15761	2702.908021	2567.55265	4108.010931	3526.38893
NEDD4L	10602.2813	8696.71427	2562.497215	2889.37672	1292.758897	2312.267356
FOXP3	0.97671868	1.01573397	71.20833748	80.4560172	25.19015778	74.29699185
PPP1R3F	283.248418	242.760419	301.8832335	226.282548	254.9243968	169.4333838
HEXB	3657.81147	4863.33426	2407.042394	3011.06645	2464.605037	2015.079389
PTCD2	477.615436	511.929922	457.3380548	377.137581	457.4532653	480.2122644
KIAA2022	0	1.01573397	409.1972069	679.853346	28.21297672	617.9335176
JKAMP	1792.27878	2006.0746	2161.323483	2131.07876	1006.598705	1894.573292
DKK3	43.9523408	51.8024326	0	0	2.015212623	2.718182629
ARHGEF5	613.096085	617.28185	1996.090052	1938.61791	1127.471158	1747.628339
NFE2L3	5426.64901	3781.57758	15.04401496	9.05130194	785.9329228	190.272784
MCUR1	3157.7315	3192.45187	884.5880797	993.631813	1089.222423	810.9244842
LIMA1	6373.08941	4309.75924	1195.497722	1910.83041	1277.644803	1187.845809
LETMD1	576.264023	562.716621	1106.236567	981.56341	1150.686407	1016.600303
SLC4A8	78.1374947	86.3373876	490.4348877	1119.34434	238.8026958	1270.297348
LAMC3	0	0	1.002934331	0	21.15973254	2.718182629
PTGER3	0	1.01573397	4.011737323	11.0627024	8.06085049	2.718182629
TNIP3	49.8126529	127.982481	0	0	14.10648836	10.87273051
MAPK9	2175.15251	2166.56056	3210.392793	2502.18214	3040.955847	2998.155439
COL23A1	0	0	0	0	15.11409467	3.624243505
BCAR1	3569.90679	4002.36767	1298.799958	1547.77263	4683.656417	2219.849147
FAM160A2	595.798397	621.629191	999.9255277	802.548772	410.0957687	801.8638754
HERPUD1	1034.34509	873.531216	6604.322568	3248.4117	837.3208447	1832.961153

HOMER3	1202.02815	1342.80031	717.0980465	751.258061	1902.048358	761.091136
RAD51	546.962463	518.024326	313.9184455	672.813444	644.8680392	935.9608851
POLQ	716.911514	519.04006	621.819285	740.195359	1703.862272	1690.709595
PIK3CB	1253.13007	1250.36852	966.8286948	1260.14237	2225.802342	1336.439792
CYBA	1451.40396	1452.49958	1831.358088	1001.67741	5018.887037	2855.903882
THOC3	2145.11841	1779.45419	2152.246927	2105.45352	2656.785789	2443.075365
HEBP2	1092.94821	1000.49796	2278.666799	2236.67728	1554.736538	2124.712755
MPHOSPH9	2039.38861	1701.3544	660.4824035	1077.10493	1581.941909	1138.021521
PLEKHA5	1293.17554	1138.63778	719.1039151	785.451868	704.3168116	714.8820313
PRSS8	0	0	6447.864812	7674.49834	1585.972334	3230.107024
SIKE1	1728.79207	1715.57468	1223.579883	1016.76292	4738.772482	1411.642845
RRP12	1724.8852	1948.17776	991.9020531	734.161157	1800.592478	1700.676265
FNIP2	480.545592	603.34598	2280.672668	2266.84829	333.517689	1203.248844
MSMO1	789.188697	3457.55844	823.4090855	32214.5893	1122.473431	821.7972147
TTC17	1775.67457	1615.01702	2824.263075	1715.72457	1380.420646	2215.318842
ALX4	0.97671868	0	0	0	0	0.906060876
FSTL4	0	0	69.20246882	226.282548	81.61611121	133.1909488
FOXN3	3862.92239	2467.21782	2283.681471	3014.08355	1075.115934	2002.394536
METTL24	0	0	0	0	0	0
AKR7A2	1239.41694	1300.13948	1967.436218	1593.11965	1074.088176	1739.247276
MRTO4	1728.79207	1635.3317	941.7553365	677.841945	1930.573692	1662.621708
NNAT	0	0	0	0	0	0.906060876
USE1	220.738423	259.012163	362.0592934	371.10338	184.391955	316.2152458
MCF2L2	22.4645297	37.582157	16.04694929	7.03990151	22.16733885	11.77879139
NRIP2	0	0	0	0	3.022818934	1.812121752
LAMA3	2428.12265	1988.80712	2.005868661	0	468.5369347	173.0576274
AP5M1	1384.01038	1410.85449	1791.240715	2040.56574	749.6590956	1866.485405
ANAPC4	566.496837	558.653685	476.3938071	447.536596	625.7235193	596.1880565
KCNQ1	0	0	26.0762926	22.1254047	11.08366942	19.93333928
TRAPPC3	1106.62227	1250.36852	1686.935544	1372.78079	1266.561133	1163.382165
THRAP3	6270.53395	5704.36199	6723.671753	8250.76457	6018.432497	5993.592696
PHPT1	989.416027	955.805668	1747.111604	1340.59839	1430.800962	1113.548817
ENTPD2	0	1.01573397	258.7570573	423.399791	309.3351376	503.7698472

LY75	36.9688022	39.9386598	0	0	0	0
ARID4B	1782.53113	1676.33688	4761.330442	4624.13919	1525.022228	3977.344489
OPN3	159.205145	90.4003235	356.0416874	548.106617	618.6702751	573.5365346
SDCCAG8	308.643104	361.601294	1473.310532	1166.61225	203.5364749	1030.191216
PTPRN	1.95343737	0	0	0	8.06085049	0
HHAT	191.436862	187.910785	149.4372153	280.59036	65.49441023	229.2334017
KIF1B	2274.20155	2273.1009	2525.910171	2834.63646	2786.031451	4155.485118
FOXC1	442.453564	413.403727	6.017605984	10.0570022	697.2635674	125.9424618
TBC1D22A	593.84496	584.047034	932.7289276	989.609012	479.6206042	1032.909399
SYNE2	6665.1283	5417.92501	5591.358894	7969.16851	6799.327389	7316.441575
PLEKHH1	467.84825	438.797076	493.4436907	347.972275	862.5110025	701.2911182
ATP9A	410.221847	374.805836	82.24061512	113.644124	7750.507746	887.9396587
SPO11	0	0	0	0	0	0
CBLN4	0	0	0	0	5.038031556	0
CHRDL2	0	0	0	0	2.015212623	0
FAM168A	2891.0873	2703.88383	2487.27714	2331.2131	3494.378688	3335.210085
RELT	733.515732	711.013781	542.5874729	592.357427	448.3848085	747.5002228
GALC	864.396035	852.200803	567.6608312	579.283324	4576.547866	1100.863965
NOP58	2597.09498	2386.97483	1533.486592	976.534909	2973.446225	2024.139997
SZRD1	4257.51674	4279.28723	2827.271878	2984.91824	4151.338003	3699.446557
KCNH2	0	0	4.011737323	4.02280086	200.5136559	21.74546103
CUL1	2758.25356	2825.77191	1928.642718	1879.6537	3344.245347	2079.409711
FAM114A2	323.293884	375.82157	556.6285535	466.6449	322.4340196	448.5001337
CYFIP2	310.596541	431.686938	757.2154197	1327.52428	1353.215276	960.4245287
TAB2	2768.99747	2571.83842	2189.405644	2577.60965	3020.803721	2840.500847
GINM1	1328.33741	1200.59756	1372.014164	1426.08291	1377.397828	1490.470141
EIF2AK2	3839.48115	3593.66679	1815.311139	1632.25145	2998.636382	3710.319288
USP36	1797.16238	1745.03096	1156.383283	1019.78002	2349.737918	1631.815638
KMT2C	3345.26149	3207.66757	6409.72322	8214.52919	6505.993039	8380.755044
MCOLN3	191.436862	236.666016	3.008802992	8.04560172	159.2017972	22.6515219
CCDC85A	5.8603121	2.28540144	8.023474646	8.8803329	32.05195676	5.436365257
PUM2	5110.19215	4848.09825	4584.412826	4572.91888	5208.317023	5073.034846
MRPL43	1311.73319	1351.94192	1192.488919	1313.44448	1270.591559	1343.688279

ITIH4	28.2369372	35.6014757	74.62834355	22.2762598	1.007606311	35.75316217
ITIH1	0	0	0	0	0	0
C4orf27	394.594348	449.97015	530.5522609	501.844408	307.3199249	422.2243683
ZFR	6478.57503	5952.20108	2985.735503	2735.50459	3545.766609	3793.676889
ZNF280C	251.99342	276.27964	132.3873317	99.5643213	395.9892803	207.4879406
NPFFR2	0	0	0	0	0	0
PHF21B	0	0	64.18779717	46.2622099	9.068456802	70.67274834
TRAF1	685.656516	801.414104	113.3315794	58.3306125	44.3346777	98.7606355
RC3H2	3094.24479	2802.41003	2833.289484	2908.48502	3295.880244	2842.312969
IL17RB	2.93015605	0	336.9859351	430.439692	115.8747258	593.4698739
TRAF3IP2	410.221847	497.709646	1057.092785	953.403804	998.5378545	983.0760507
GYG2	80.0909321	68.0541761	0	0	464.5065095	45.30304381
DCBLD2	8825.63003	9268.5725	343.0035411	1082.13343	1809.660935	774.6820491
SERPINB3	0	1.01573397	0	0	0	0
SOAT1	3275.91447	3448.41684	283.8304156	769.360665	1007.606311	636.960796
PKP2	1270.71101	823.750094	37.10857024	28.159606	726.4841504	103.2546974
MSH4	1.95343737	1.01573397	3.008802992	1.00570022	0	2.718182629
F7	0.97671868	0	1326.88212	766.343564	9.068456802	249.1667409
GDI2	7887.75545	8233.3669	5504.103607	5580.6305	9795.807493	5827.70201
PRDM1	41.0221847	51.8024326	0	4.02280086	3.022818934	87.88790499
ATG5	933.743062	936.506722	952.7876142	859.873684	817.1687185	926.9002763
TMCC3	1333.221	978.151815	1.002934331	0	58.44116605	42.58486118
PITHD1	880.023534	793.288232	921.6966499	625.545534	814.1458995	1139.824582
MTA3	1062.66993	868.452546	977.8609724	898.090292	1462.036758	882.5032934
USP13	1697.53707	1727.76349	1042.04877	1340.59839	2040.40278	1532.148942
ATP11B	3230.98541	2899.92049	340.9976724	331.881071	1758.273013	485.6486296
LAMC2	2265.01063	2806.47297	12.03521197	37.210908	730.5145757	138.6273141
CDK14	136.740616	141.187022	121.355054	109.621323	101.7682374	84.26366148
SEC61A1	8148.76398	9234.03754	10206.86268	6071.4122	11736.59831	9419.408869
PPP1R12A	3457.58414	3006.57256	2622.673275	2774.72689	3160.860999	2888.522073
RASGRF1	661.238549	833.917591	0	0	33.25100827	17.21515665
CAMK2B	1.95343737	3.04720192	5.014671654	1.00570022	180.3615297	25.36970453
CROCC	553.662753	489.837708	723.5469142	699.172847	326.1520869	631.8234308

POLR3E	927.88275	998.466495	2491.288877	1428.09431	1488.234522	2589.521984
ATP2B4	2200.54719	2013.18473	1226.588686	2669.12837	1101.313698	2714.558385
ZC3H11A	6634.16632	6046.72528	13554.8681	9386.30068	7228.487069	11641.84935
RIOK2	846.815099	945.648328	588.7224521	674.824845	682.1494727	712.1638487
YIPF1	427.802784	491.615243	885.591014	1026.81992	359.7154531	781.9305361
NDC1	3976.22176	3631.24895	1286.764746	1707.67897	2231.84798	2728.149298
DGKG	0.97671868	2.03146794	0	0	11.08366942	3.624243505
FLYWCH1	691.516828	778.052223	2320.790041	1920.88741	1512.417073	1907.258144
UNKL	778.444791	754.690341	991.9020531	837.74828	1541.637656	1221.370061
TBXAS1	26.3714045	71.1013781	1.002934331	1.00570022	9.068456802	20.83940015
PARP12	1220.89835	1072.61507	93.27289276	181.026039	289.1830113	704.0093008
ALDH18A1	3540.60523	3547.95877	5082.871188	2346.2986	3068.161218	4697.019582
TARBP1	905.41822	875.562684	919.6907813	1124.37284	922.9673811	2125.618816
GATB	423.895909	430.671204	725.1215211	631.579735	323.4416259	709.445666
MXD1	309.688193	332.145009	311.9125769	744.21816	975.6148109	461.3843194
CDK17	1362.52256	1454.53105	844.4707065	928.261299	830.2676005	1022.942729
DNAJC25	369.531747	328.620412	185.2820883	114.237487	248.3648797	111.8260333
SLC2A3	1031.97166	1671.35978	2.005868661	0	25.19015778	14.49697402
PSD	3.90687474	3.04720192	182.5340482	191.083041	59.44877237	185.7424796
CTDP1	967.928216	998.466495	951.7846798	944.352502	722.4537252	935.9608851
YBX3	4844.52467	4079.18763	0	0	4730.711631	473.8698382
STYK1	178.739519	158.4545	14.04108063	8.04560172	95.72259957	33.52425242
WNK1	5670.82868	5894.30424	7957.28098	9209.19687	7676.952486	9930.427203
RPS17P5	0	0	0	0	1.017682374	0
CCAR1	3260.28697	3464.66858	3599.531313	3345.96462	2951.278886	3613.370774
OGFR	3271.03087	3493.10913	1373.017099	1504.52752	2726.582678	1856.518735
GNA15	1092.94821	1382.41394	3.008802992	2.01140043	29.22058303	16.30909577
CREB3L3	4.88359342	15.2360096	0	0	0	0.906060876
PIGV	335.991227	383.947442	305.8949709	281.59606	306.3123186	210.2061233
PTPRU	786.258541	777.036489	1500.389759	1752.93548	1089.222423	1461.476193
SNRNP40	1445.54365	1376.31953	677.9836076	773.383466	845.3816952	1173.348835
RIMBP2	9.76718684	6.09440383	0	0	0	0.906060876
COL11A1	2.93015605	3.04720192	0	0	0	1.812121752

QSER1	4413.79173	3904.48139	1665.873923	2118.00465	3473.218955	2958.288761
MPC1	389.710755	545.449143	702.0540315	1022.79712	223.6886011	577.1607781
ACAA1	914.208688	952.758466	1694.959019	1483.40782	1365.306552	1990.615745
BCAT1	6379.92644	4925.29403	0	0	274.0689167	102.384879
HDAC7	1742.46613	1666.81945	1213.55054	1101.24174	1538.614837	1549.364098
LZTS1	3.90687474	0	0	0	0	0
PRDM6	17.5809363	26.4090833	114.3345137	73.4161157	165.2474351	93.32427025
WNT8A	0	0	0	0	0	0
SPAG4	177.7628	143.21849	12.03521197	25.1425054	99.75302482	50.73940907
NCKAP1	8084.30055	7962.33861	9364.397846	7351.66858	8459.86259	7114.39
MRPS35	1174.99258	1142.70072	2937.594655	2234.66588	1565.820208	2126.524876
GUCY1B3	18.557655	36.566423	327.9595261	419.37699	4.030425245	400.4789073
SFSWAP	1318.57022	1239.19545	1856.431446	1609.12034	1694.793816	1881.88844
TNK2	1693.6302	1569.30899	8884.995236	10000.6829	1389.489103	8339.384304
MON2	1126.15664	990.340623	2329.81645	2237.68298	2913.997452	2332.200695
CDH3	22.4645297	24.3776153	206.6044721	567.214922	2824.320491	652.3638308
ARSF	0	0	0	0	0	0
GPBP1	2706.48747	2760.76494	3142.193258	2807.915	2206.657822	2886.709952
DGAT2	296.92248	318.940467	256.7511887	133.758129	228.7266327	374.2031419
ZNF112	152.368115	150.328628	177.5193765	189.071641	104.7910564	192.0849057
CS	4138.47427	4076.42484	6124.929987	6555.506	9633.965768	7734.190003
LTK	75.2073387	61.9597723	8.023474646	13.0741028	6.045637868	11.77879139
MRPS24	1679.878	1775.32015	925.7485046	1016.37069	1502.461923	994.8820239
ELMO2	1675.07254	1825.27395	1533.486592	1451.22541	1135.572313	1478.69135
WAPL	3565.0232	3252.38018	2090.115145	2283.94519	2634.890504	3243.697937
VMP1	4088.54441	4112.70685	7316.405943	5283.94893	16380.6558	8652.881368
APPBP2	1255.08351	1241.22691	2004.865727	2237.68298	17657.293	4019.286047
POLD1	2817.8334	2586.99317	1229.597489	1352.66679	1793.539234	1987.897562
SEZ6	0	0	2.005868661	0	0	0
EIF4B	7950.40218	7438.21988	10874.1851	13689.1388	21287.26527	9183.21692
SLC6A16	0.97671868	1.01573397	57.16725685	74.421816	14.10648836	46.20910469
GLTSCR1	659.285112	641.94387	827.4208228	1126.38424	819.1839311	963.1427114
SPHK2	686.633235	691.714835	1035.028229	1106.27024	384.9056109	1104.488208

RPL18	11691.3226	11527.9204	13484.45208	9939.33523	12799.6129	9067.857249
CA11	14.6507803	60.9440383	25.07335827	84.4788181	40.30425245	137.7212532
ISOC2	810.676508	855.248005	834.4413631	1147.50395	1171.84614	932.3366416
U2AF2	6434.62269	6094.40383	6003.564904	5762.66224	8034.652726	6422.15949
EPN1	5877.89304	5614.9774	3924.482036	4001.68116	4219.855232	4969.96136
MED29	1214.06132	1426.0905	1920.619243	1553.80683	1878.178164	1868.297527
AHRR	34.1851539	44.6922948	62.1819285	45.2565097	219.6581759	107.8212443
GSC2	0	0	0	0	2.015212623	0
ZNF275	388.734036	353.475422	1228.594555	762.320763	1351.200063	1154.321556
MTMR1	1952.46065	1469.76706	2710.931496	2913.51352	2186.505695	2845.937212
GPC1	853.65213	732.344194	613.7958104	1429.10001	2456.544187	860.7578324
ADCK1	26.3714045	37.582157	309.9067082	335.903872	268.0232788	248.2606801
HAGH	859.512442	724.218322	1791.240715	1872.6138	986.4465788	1152.509434
RNF4	1749.30316	1788.70753	1459.269451	1281.26207	1758.273013	1795.812657
CASP8	1183.78304	1349.91045	625.8310224	560.17502	461.4836906	749.3123446
LIMCH1	5197.12012	3266.60045	3013.817664	2760.64709	630.7615509	2604.018958
ASUN	838.024631	959.868604	510.4935743	631.579735	1292.758897	907.8729979
TM7SF3	1080.25086	1254.43146	1806.28473	2505.19924	3142.724085	2627.576541
DLX3	14.6507803	22.3461474	88.2582211	148.843632	328.4796575	173.0576274
SPA17	480.545592	478.410701	17.04988362	21.1197045	131.9964268	40.77273943
TSPAN32	0	0	0	0	0	0
WISP2	6.83703079	2.03146794	0	0	767.7960092	106.0091225
DMRT3	0	0	0	0	0	0
ST3GAL6	258.830451	405.277855	183.376513	195.739433	10.07606311	99.66669638
ATP2C2	3.90687474	3.04720192	4294.564804	4056.99467	892.7391918	1607.351994
NGFR	946.440405	1023.85984	0	0	32.24340196	10.87273051
CDON	800.909321	669.368688	134.3932003	103.587122	162.2246161	159.4667142
TAF2	1984.69237	1809.0222	1151.368612	1308.41598	1789.508809	1600.103507
HIPK2	5117.02918	6110.65558	1461.27532	1246.06257	3321.070402	2411.934052
TNPO3	2415.42531	2389.0063	2008.877464	1953.06982	3963.923229	2217.130964
BORCS8-MEF2B	164.801744	152.400725	170.1076918	125.350475	92.58894394	190.4539962
RFXANK	766.724167	814.618646	1432.190224	1172.64645	811.1230806	1139.824582
TMEM161A	756.95698	778.052223	469.3732668	393.228784	591.4649047	607.060787

LPAR2	164.088739	142.202756	170.4988362	353.000776	739.5830325	347.9273765
CTSA	3371.6329	3908.54433	1559.562884	3297.69101	2562.34285	2375.691617
SUGP2	4610.11219	4398.1281	5743.804912	4207.8497	2802.153152	5477.137996
SLC12A2	1471.91506	1642.44183	762.2300913	2103.92485	7671.914454	2418.276479
SNX24	1052.90274	822.744518	165.4841646	124.706827	612.6246373	128.6606444
EYA2	0.97671868	0	0	0	369.7915162	38.0545568
CNN2	6633.8733	5728.7396	3057.946774	2854.17721	1262.530708	5077.56515
ABCA7	444.407001	474.347765	935.7377306	1922.89881	237.7950895	1535.773185
SNCAIP	8.79046815	13.2045416	1.002934331	0	13.09888205	6.342426133
DDX20	1118.34289	1025.89131	694.0305569	695.944549	757.7199461	1012.069999
BTBD1	2744.5795	2733.34012	643.8838403	773.383466	2125.041711	1038.345764
FAR2	0	0	983.8785784	1036.87692	72.54765441	231.0455234
BCAS1	0	0	27.07922693	19.1083041	719.4309063	935.9608851
POU1F1	0	0	0	0	0	0
CHI3L2	2.93015605	2.03146794	0	1.00570022	3.022818934	26.27576541
SBNO2	4581.78735	4627.68398	3287.618736	3555.15026	2839.434585	2868.588734
PMS1	834.117756	816.650114	648.898512	465.6392	511.8640061	666.8608049
HMG20B	3627.53319	3686.09859	7184.018611	11591.7007	2572.418913	7020.159669
CALCRL	3.90687474	9.14160575	0	0	1.007606311	0.906060876
TAF11	711.051202	788.209562	753.2036824	818.639975	1383.443465	1102.676086
ANKS1A	1426.00928	1649.55197	741.1684704	854.845183	1271.599165	1355.467071
AP3D1	6302.76567	6810.49628	6994.464022	6039.22979	5579.116146	6779.147476
ZNF76	1810.83644	1582.51353	839.4560348	946.363903	737.5678199	814.5487277
SLC9A3R2	2295.28891	2018.2634	5129.006167	4146.50199	1222.226456	4080.898186
NTHL1	374.083256	371.758634	657.9249209	411.331388	548.1378333	571.7244129
UHRF1BP1	2421.28562	1903.48546	2053.08681	1553.80683	1792.531628	2164.579433
GNAI3	4276.0744	4193.96557	2289.699077	2752.60149	3597.154531	2765.297794
IPO5	16459.6633	14913.0062	1448.237174	1877.6423	6191.740783	2594.958349
OAT	4771.27077	4978.1122	2070.056459	3025.14625	1507.379042	2249.749156
WDR3	2140.96736	2214.30006	747.1860764	757.292262	1598.06361	2020.515754
PKN2	2288.45188	2108.66373	1982.801172	1974.18952	1549.698507	2584.085619
WDR18	1753.21004	1618.06422	2015.898005	1036.87692	824.2219626	1336.439792
TRAM2	19744.3682	15460.4868	723.1156524	1042.91112	1969.870339	1579.264107

NTN1	17.5809363	16.2517436	0	1.00570022	255.9320031	23.55758278
GLP2R	2.93015605	3.04720192	0	0	7.053244179	2.718182629
MCM10	1968.08815	1931.92602	301.8832335	538.049615	2024.281079	1101.770025
DGKA	334.03779	313.861797	94.27582709	84.4788181	573.3279911	195.7091493
ERBB3	260.783889	263.075099	10954.04876	13276.2485	5442.081687	8980.875405
ROPN1	0	0	0	0	0	0
ANKRD44	331.107634	396.136249	19.05575228	30.1710065	159.2017972	26.27576541
KARS	3547.44226	3518.50248	2360.907414	1857.5283	9915.853709	2964.631187
ADAT1	591.891522	554.590749	343.93627	401.053132	2378.938349	628.8062481
PDIA5	573.333867	525.134464	1071.133865	625.545534	652.9288897	1239.491279
TBC1D22B	1305.87288	1215.83356	488.4290191	483.741804	551.1606523	588.9395695
NDUFB4	1312.52433	1458.93933	3194.315755	2910.43608	1985.971887	2457.853218
SPEN	3898.08427	3562.17904	4149.139326	3913.17954	4320.615863	6193.83215
MYLK	3370.65618	2574.88562	31.09096425	40.2280086	94.71499326	61.61213958
ZC3H15	2597.09498	2748.57613	2309.757764	1945.02422	2682.248001	2372.973435
MAP2K4	1659.44504	1723.70055	714.0892435	681.864746	1294.753958	809.1123624
TMEM206	496.173091	567.79529	624.828088	476.701902	207.5669001	371.4849592
SNAP91	34.1851539	58.9125704	2.005868661	0	4.030425245	0
SLK	3940.08317	4135.053	2419.077606	3039.22605	1679.679721	2090.282441
CYB5R4	856.582286	964.947274	190.5575228	334.898172	616.6550625	296.2819065
COL17A1	228.552172	303.704458	2.005868661	0	11.08366942	2.718182629
GSTO2	18.557655	33.5192211	1314.846908	786.457569	613.6322436	762.9032577
SEC61A2	392.640911	431.686938	323.9477888	540.061016	303.2894997	453.0304381
PRKCQ	2.93015605	2.03146794	0	0	2.015212623	1.812121752
TLE2	44.9290595	28.4405512	455.3321861	502.850108	330.4948701	932.3366416
ASB1	1739.53598	1666.81945	540.5816043	611.465731	824.2219626	858.9457106
FAM107B	3145.03416	2371.73883	174.5105735	214.214146	580.3812353	286.3152369
ME1	821.420413	1067.5364	0	0	1956.771457	174.8697491
TBC1D1	1941.71674	1930.91028	48.14084787	107.609923	1024.735619	343.3970721
CDK13	1865.53269	1805.975	1650.829908	1519.61303	2201.61979	1948.030884
MTHFD2	1707.30426	1308.26536	12785.40685	5213.54992	5152.898676	5864.932052
SLC9A7	2476.95858	2482.45383	436.2764339	638.619637	2338.654249	1301.103418
FOXJ2	719.84167	773.989287	914.6761096	1386.8606	1050.933383	1369.964045

YBX1	14912.0916	15407.1811	9687.623522	11086.1151	20489.45267	13859.79577
PDE4A	1529.54146	1757.21977	5.014671654	3.01710065	105.7986627	86.07578324
PPP2R5A	732.539013	786.178095	3922.476167	4773.05322	1151.694014	4410.704345
CTNNA2	0	0	0	0	3.022818934	0.906060876
ELAVL1	4601.32172	4611.43223	3119.125769	2802.8865	3242.47711	3672.264731
TIE1	25.3946858	17.2674775	0	0	13.09888205	8.154547886
DIP2B	1903.62471	1686.11839	1923.628046	3496.81965	4021.356788	3206.549441
SMARCD1	1930.97284	1887.23372	1024.998886	1150.52105	2025.288686	1504.967115
KDM4A	1039.22868	1028.93851	2703.910956	3324.84491	1801.600085	2427.337087
NFYC	1080.25086	976.120347	1563.574622	1743.88417	856.4653646	1174.254896
ZMYND12	8.79046815	10.1573397	7.020540315	5.02850108	3.022818934	3.624243505
SLC9A3	0	0	6.017605984	9.05130194	0	6.342426133
NGEF	130.880304	148.29716	28.08216126	41.2337088	131.9964268	41.6788003
ASPM	5597.57478	4759.72939	2046.988969	7010.7362	3719.074895	6825.35658
CD84	0	0	0	0	0	0
ELOVL1	1678.97942	1787.69179	1318.858645	2534.36454	1859.033644	2164.579433
SPI1	0	0	0	0	0	0
ZNRD1	453.197469	408.325057	267.7834663	241.368052	275.076523	259.1334106
MPPED2	0	0	0	0	128.9736078	5.436365257
CLDN18	0	0	0	0	1.007606311	0
ZBTB11	1210.15445	1009.63957	985.8844471	912.170096	1843.91955	1394.427688
ATXN3	972.216011	974.099037	1104.581725	816.397264	458.2190461	1008.953149
GOLGA5	1184.75976	1274.74614	2935.588786	2542.41014	1806.638116	2110.215781
FGFR2	0	0	43.12617622	175.997538	603.5561805	160.3727751
LRRC40	640.727457	499.741114	672.9689359	600.403029	514.8868251	675.9214136
ISOC1	557.706368	573.889694	1665.873923	1754.94688	3812.782282	1793.094474
EML1	96.6951497	108.683535	0	0	1385.458678	75.20305272
TRMT11	503.010122	436.765608	351.0270157	284.613161	306.3123186	310.7788805
THUMPD1	1116.38946	1279.82481	2064.038853	1549.78403	1867.094495	2156.424885
MSANTD3	2076.50392	2135.94634	165.4841646	232.31675	1561.76963	238.2940104
KIF26A	0.97671868	0	659.9307896	895.073192	1185.952628	1022.942729
ATG2B	1645.77098	1585.56073	2818.245469	3038.22035	2254.015318	2698.249289
ARFGEF1	1499.26318	1610.95408	3627.613474	5195.44731	1624.261374	4309.225527

ACSM2B	0	0	0	0	0	0
ZFAT	439.523408	450.985884	280.8216126	358.029277	299.2590745	277.2546281
MTFR1	830.210881	918.223511	4174.212684	3230.30909	1099.298486	3174.83731
STAG3	2.98875917	3.15893265	253.7423857	243.731447	10.35819288	875.4088367
FECH	883.930409	803.445572	1390.066982	1108.28164	1325.002299	1439.730732
MYO9A	1550.05255	1417.96463	665.9483956	987.597612	1456.998726	923.2760328
DDX3Y	0	0	0	0	0	0
PFKP	10702.8833	9397.56055	2938.597589	2129.06736	9991.404031	3020.806961
IDI1	1246.29304	3670.86258	935.7377306	22270.2256	2145.193837	1232.242792
SP100	1477.81444	1421.86504	127.37266	165.940536	349.63939	326.1819154
KLF6	5968.72788	4946.62444	1349.949609	3092.52816	658.9745276	919.6517893
PLPP1	516.684184	575.921162	40.11737323	80.4560172	333.517689	138.6273141
NEO1	713.981358	766.879149	106.3110391	106.604223	2102.874372	252.7909845
TRAM1	3678.32256	3678.98845	3835.220881	4475.36596	2592.571039	4103.549708
PHKA1	720.623045	703.852856	1125.703522	1105.19414	1221.672272	832.8511574
TNFRSF1A	2461.33108	2362.59722	2386.983707	1896.75061	3280.76615	2166.391555
CACNB1	341.851539	323.003403	47.13791354	93.53012	87.66174908	93.32427025
EVI5	1684.83973	1756.20404	491.437822	464.6335	376.8447604	434.9092206
STOML1	382.873724	412.387993	179.5252452	156.889234	183.3843487	172.1515665
PKM	64285.6703	63824.6599	10365.32631	12407.3236	47376.64115	21234.44269
DHX29	2406.63484	2404.24231	1256.676716	1322.49578	1783.463171	1629.097455
DNTTIP2	3318.89009	3525.61262	1406.113932	1361.71809	1472.112821	1905.446023
METTL22	220.738423	185.879317	549.6080132	455.582198	447.3772022	544.5425866
TP53BP1	1652.60801	2000.99593	2891.459675	2769.69839	1573.881058	4039.219386
TRO	0	0	3.008802992	1.00570022	0	0
RRP15	1373.26647	1404.76008	774.2653033	637.613937	1718.976367	980.357868
RHOA	14418.3212	12750.5086	14590.68864	11168.3009	7580.22228	10362.61824
DHX8	2511.14374	2371.73883	758.218354	821.657076	1813.69136	1084.554869
PMS2P4	32.9056525	17.5823551	46.18512593	30.7844836	42.23885657	38.0545568
PRKCZ	599.705272	408.325057	1366.999493	1469.32801	712.3776621	925.0881546
ZFY	0	0	0	1.00570022	0	0
IARS2	6527.41096	5885.16264	8153.856109	7882.67829	4827.441837	10513.02435
SYT1	12.6973429	14.2202756	0	1.00570022	757.7199461	92.41820937

NAV3	1700.46723	1947.16202	1.002934331	6.03420129	190.4375928	106.9151834
IDH3G	782.351666	699.840707	2827.271878	2917.53633	2640.936142	2237.064303
ROGDI	171.902488	171.659041	1146.35394	1454.24251	675.0962286	1097.239721
PDZD4	0	0	0	1.00570022	874.6022782	86.07578324
ATP2B3	0	0	1.002934331	0	0	1.812121752
ROCK1	4339.81506	3925.10079	2262.589762	2758.3541	3473.07789	2938.291997
CBFB	1582.28427	1466.71986	1441.216633	1652.36545	1618.215736	2237.064303
PDK3	277.388106	300.657256	485.4202161	691.921748	758.7275524	666.8608049
HYAL2	1000.15993	1072.61507	421.2324189	606.43723	784.9253165	745.6881011
HDAC4	726.678701	629.755063	856.5059184	637.613937	594.4877237	976.7336245
RASSF1	738.399325	624.676393	428.2529592	368.086279	338.5557206	482.930447
FGFR3	58.603121	71.1013781	15.04401496	17.0969037	686.179898	53.45759169
IFI35	431.709658	443.875746	0	0	41.31185876	52.55153082
HEATR6	888.814002	834.933325	489.4319534	652.69944	7089.518006	1181.503383
COASY	2151.71126	2251.88222	1971.768894	1886.6936	1379.41304	1706.11263
PLEKHH3	440.500126	369.727166	1878.496001	1713.71317	1190.99066	1723.327786
MEF2A	1567.63349	1588.60793	628.8398254	676.836245	924.9825938	922.3699719
OTUD5	1499.26318	1254.43146	2206.455528	1779.08368	778.8796786	1439.730732
TFE3	3430.23602	3268.63192	2856.356974	2611.80346	1710.915517	2180.888529
TBC1D25	954.254154	901.971767	484.4172817	636.608236	401.0273119	399.5728464
ACSL4	4345.42142	4504.78017	4.011737323	423.399791	142.0724899	196.6152101
INPP5A	452.220751	484.505105	626.8339567	574.254823	968.3096651	663.2365614
GPKOW	903.464783	819.697316	1157.386218	914.181496	790.9709544	693.1365703
GRIPAP1	1292.19882	1261.54159	2291.704946	1812.27179	1331.047937	1583.794412
FTSJ1	2210.31438	2019.27914	1257.679651	1009.72302	1030.781256	983.9821115
PRR11	4088.54441	3699.30313	221.6484871	593.363127	4996.719698	634.2426133
REEP1	123.066554	133.06115	100.2934331	266.510557	191.4451991	116.881853
ATP11A	2223.01172	2139.13575	359.0504904	258.464955	2660.080662	585.315326
POLR1A	6586.9908	6806.43335	1672.894464	1470.33372	4910.065555	3594.343496
LAPTM4A	3862.92239	3991.83451	5351.657589	5439.83247	3710.006438	5252.434899
TTC7A	1039.22868	863.373876	374.0945054	598.391628	1700.839453	594.3759348
IP6K2	1049.97259	1078.70948	2747.037132	3271.5428	877.6250971	2286.897651
STON1-GTF2A1L	0	0	0	0	8.917315855	2.92657663

SRBD1	698.353859	653.116944	709.0745718	869.930686	861.5033962	775.58811
KIF2A	2343.14812	1988.80712	861.5205901	1052.96813	1636.35265	1688.897473
RASGRP2	11.7206242	5.07866986	4.011737323	1.00570022	12.09127574	2.718182629
PSME4	4601.32172	4684.56508	1712.008903	1591.01774	4524.152338	3034.397874
IFT80	699.936143	675.463092	943.7612052	741.201059	638.5503477	722.1305183
SIRT2	850.721974	949.711264	1435.199027	1670.46806	513.8792188	938.6790677
ERLEC1	1748.32644	1962.39803	2286.690274	1683.54216	1203.081936	1515.839846
PPP2R5B	753.050105	726.24979	428.2529592	445.525195	144.0877025	431.2849771
PYGM	2.93015605	5.07866986	11.03227764	13.0741028	16.12170098	9.966669638
PAGE1	0	0	2.005868661	0	0	0
PITX1	1963.20455	1710.49601	789.3093183	988.603312	595.49533	1044.68819
TRPC7	0	0	0	0	0	0
MAST4	495.196373	503.80405	1140.336334	1403.9575	796.0089859	2321.327965
ADGRF5	11046.6297	11241.0974	1.002934331	6.03420129	5.038031556	56.17577432
SDK2	8.79046815	8.12587178	1.002934331	0	572.3203848	77.92123535
ADAM7	0	0	0	0	0	0
NUP133	2370.49625	2301.65318	2411.054131	2870.26842	1714.945942	3558.101061
NUCKS1	15376.4822	14548.3577	17980.60668	25405.9988	29746.55352	21988.28534
VPS35	3880.50333	4203.10718	6360.589467	6673.82663	4973.5246	6160.307897
DNAJA2	2258.1736	2191.95391	4335.685112	3837.75202	3407.724545	5077.56515
BCL3	969.881653	660.227082	1138.330465	1383.8435	1203.081936	1194.188235
KCNAB2	395.571067	478.410701	402.1766666	788.468969	459.4684779	585.315326
ABCC9	4.88359342	2.03146794	0	3.01710065	4.030425245	0.906060876
GAL	106.462337	114.777939	0	0	691.2179295	9.060608762
CLEC2D	37.0176381	35.8249372	389.0181682	339.162341	87.67182515	192.3839058
FUNDC1	440.500126	473.332031	677.9836076	819.645676	428.2326823	600.7183609
MAOB	6.83703079	10.1573397	0	0	55.41834712	2.718182629
RORA	16.6042176	5.07866986	485.4202161	328.86397	23.17494516	426.7546727
DRD4	13.6740616	3.04720192	15.04401496	17.0969037	5.038031556	11.77879139
TGFBR3	961.091185	863.373876	82.24061512	102.581422	75.57047335	57.0818352
KIAA1107	172.879207	121.888077	192.5633915	161.917735	86.65414277	141.3454967
PLA2G10	0	0	0	1.00570022	58.0179714	13.98957993
HES2	83.0210881	86.3373876	16.04694929	0	83.63132384	30.80606979

ATP1B3	2807.0895	2900.93622	1802.272992	2227.62598	2575.441732	1827.524787
NEDD4	3613.85913	3649.53216	2362.913283	1980.22372	1309.888205	2034.106667
PIGB	204.134205	273.232439	423.2382876	455.582198	249.8863652	347.9273765
MAPK6	1870.41628	2101.55359	2893.465544	2051.60833	5635.542099	2667.443219
GNB5	1264.8507	1303.18669	439.2852369	459.604999	893.7467981	565.3819867
RAB27A	817.513538	786.178095	874.5587364	349.048374	297.2438618	309.8728197
CECR5	1170.10898	1073.63081	1681.920873	1620.18305	1438.861813	1920.849057
UFD1L	1217.9682	1208.72343	2364.919152	2575.59825	2215.726279	2878.555404
LRP6	2091.1547	1812.06941	1166.412627	1559.84103	1178.899384	1977.024832
GUCY2C	0	0	1.002934331	0	0	0
SCT	0	0	0	1.00570022	0	0
PHRF1	2601.00186	2642.9398	3278.592327	3255.4516	1926.543267	4147.04063
IKBKAP	2199.57048	2124.91547	1306.823433	1267.18227	3096.374195	1259.424618
NUCB2	1717.07145	2507.84718	5067.827173	3409.32373	1096.275667	1224.994305
PFN2	1324.43054	1436.24784	1537.498329	842.776781	5776.606983	1470.536802
PTPN3	979.64884	940.569658	739.1626017	803.554472	1469.090002	636.0547351
SPTB	30.2782792	50.7866986	4.011737323	4.02280086	21.15973254	8.154547886
DAPP1	1.95343737	3.04720192	0	0	10.07606311	20.83940015
FGF10	0	0	0	0	0	0
SLC44A1	1755.16347	1903.48546	2432.115752	3187.06398	4702.498655	2102.967294
TMEM260	413.152003	365.66423	846.4765751	401.274386	454.4304464	545.4486475
SMG6	1960.2744	2084.28611	484.4172817	456.587898	1062.017052	640.5850395
EXOC5	4652.11109	4442.80008	2736.004854	3154.88158	2611.715559	4134.355778
CLTCL1	334.03779	366.679964	68.19953449	46.2622099	206.5592938	125.9424618
FGF22	3.90687474	2.03146794	0	12.0684026	4.030425245	2.718182629
FSTL3	828.257444	633.817999	165.4841646	277.573259	722.4537252	202.0515754
DGCR2	2002.2733	2063.97143	5513.130016	5001.34717	2704.415339	6246.38368
RNF126	1982.73893	1703.38587	1213.55054	1052.96813	1438.861813	1414.361028
MNT	804.816196	939.553924	608.7811387	874.959188	739.5830325	862.5699541
ZXDC	758.910417	672.41589	1257.679651	1303.38748	1290.743685	1428.858002
JMJD6	940.580093	1000.49796	694.7626989	672.813444	627.7387319	647.8335265
POLB	276.411388	265.106567	686.0070822	555.146519	222.6809948	555.4153171
ST6GALNAC1	0	0	1.002934331	0	0	0.906060876

WIPI1	676.866048	1094.96122	191.5604572	856.856584	527.9857071	341.5849503
FRMPD1	0	0	144.4225436	29.1653063	5.038031556	26.27576541
GBA2	1161.31852	1239.19545	2866.386317	3579.28707	827.2447816	2396.531017
NDST1	8573.63661	7074.58712	8004.418893	9313.7897	3446.013585	7959.744797
ASNS	1488.51927	981.199017	5543.218046	588.334626	2404.148659	679.5456571
AP3M2	714.958077	714.060982	726.1244554	522.964112	484.6586357	585.315326
CNGB1	2.93015605	15.2360096	0	0	12.09127574	1.812121752
ST6GALNAC2	68.3703079	67.0384422	15371.97449	15726.1343	448.3848085	1914.506631
CHAT	0	0	0	0	0	0
PABPC1	37415.1236	35685.741	38712.20205	36213.193	94346.07836	47335.27493
TESK2	69.3470266	108.683535	1900.560557	3470.67144	158.1941909	1419.797393
CFAP20	928.859468	1002.52943	920.6937156	840.76538	860.4957898	784.6487188
CSNK2A2	2217.15141	2235.63047	2038.965494	2161.24976	2202.627396	2241.594608
PTPN21	1495.3563	1427.10623	118.346251	178.008938	1017.682374	257.3212888
EIF2B3	573.333867	571.858226	555.6256192	395.240185	640.837614	549.072891
CAMK2A	0.97671868	3.04720192	0	0	0	0.906060876
TCOF1	5591.71447	5442.30262	1875.487198	1680.52506	5394.724191	3356.955546
CDC42	6627.74927	5913.5524	6264.32783	7086.05309	4080.493203	7124.175457
OSBPL3	4734.15546	3136.58651	451.3204488	639.625337	60.45637868	448.5001337
EPHA8	0	0	0	0	1.007606311	2.718182629
SLC12A3	0	0	0	0	2.015212623	0.906060876
RAD18	1811.81316	1550.01004	675.9777389	639.625337	1247.416613	1123.515486
ATP2B1	6614.33893	8890.71946	1010.957805	1965.13822	3911.5277	2714.558385
TRPM5	0	0	0	0	5.038031556	0
NCK2	1326.38397	1213.8021	890.6056857	1023.80282	837.3208447	636.0547351
MAP4K4	8441.77958	10533.1613	49.1437822	46.2622099	3670.709792	405.0092117
MGAT4A	359.432476	382.931708	1411.128603	1404.9632	1080.153966	1561.14289
RPL31	8187.73505	8141.03669	11877.64096	7770.86454	7408.898979	6041.57768
WDR1	14726.9643	13291.8948	5223.281994	5942.68257	9475.529751	7507.62042
SNX13	917.138844	934.475254	1794.249518	1778.07798	1246.409007	1734.200517
MS4A12	0	0	0	0	0	0
ARHGAP10	673.935892	609.440383	157.4606899	209.185645	726.4841504	257.3212888
RPS6KA2	457.104344	608.424649	4.011737323	1.00570022	265.0004599	69.76668747

ING3	208.04108	245.807621	145.425478	225.276848	330.4948701	212.9243059
VASH1	115.702095	183.522814	3.369859351	3.01710065	322.4340196	19.14506631
LMCD1	128.926866	155.407298	409.1972069	903.118794	176.3311045	435.8152814
WBSCR22	897.60447	892.830162	1389.064048	989.609012	2402.133446	1621.848968
SEL1L	3933.24614	4299.6019	9212.954762	7159.57983	4214.8172	6440.280708
TRIP13	1718.04816	1534.77403	487.4260847	528.998313	1673.634083	1212.309452
ATP6AP1	1944.6469	2420.49406	15686.89587	21839.7859	7965.127891	16458.59582
TCF3	3041.50198	2865.38554	3267.560049	3464.63724	4327.669107	5888.489634
TRIB2	2082.36423	2179.7651	388.135586	583.306125	214.6201443	727.5668836
DAZAP1	4472.39485	3569.28918	4021.766666	3114.65357	4639.019457	4242.177022
MBD3	4809.88046	5463.2369	1889.809101	2420.67013	2233.077259	2230.749059
PRLH	0	0	1.002934331	0	1.007606311	0
HLTF	2109.71236	1978.64978	4880.278453	4233.99791	2337.646642	6759.214136
FAM50A	1582.28427	1841.52569	6341.553773	6138.79412	1668.596051	5907.516913
FAM3A	717.888233	843.059197	3859.291305	3730.1421	1791.524021	3203.831258
CPSF1	3566.97663	3207.68788	4274.506117	4258.13471	3160.860999	4706.080191
MYO3B	0	0	0	0	3.022818934	0.906060876
CYBRD1	3746.69287	4080.20337	1755.135079	2424.74322	39.29664614	1101.770025
CDH19	0	0	0	1.00570022	0	0
PDCD2	2055.01611	2190.93818	1596.671454	1413.0088	1957.779063	1851.988431
SLC6A15	0	0	0	0	0	0
RDH11	3016.1073	4550.80307	3814.15926	7525.33289	1710.915517	2879.09904
PRKACA	2130.22345	2087.33331	2972.697356	3077.44266	3326.108434	3173.025188
ADGRL1	934.71978	830.596141	5328.590099	4273.23027	3366.412686	7500.308509
SPP2	0	0	0	0	0	0
ACTN1	29243.0941	27791.7715	6272.361334	6579.13996	9394.669345	8977.930707
ZFYVE26	1266.80413	1474.84573	1793.246583	3462.62584	1198.043904	2748.082637
RPS6KA6	0	0	375.0974397	270.533358	41.31185876	247.3546192
EPN2	1290.24538	1224.97517	1973.774763	1932.95581	1427.778143	2456.331035
PTPN18	806.769633	940.569658	776.271172	1115.32154	1822.759817	965.860894
LIMS2	757.933699	496.693912	0	0	12.09127574	5.436365257
ASIC4	0.97671868	1.01573397	4.011737323	2.01140043	8.06085049	1.812121752
SPEG	520.591058	477.394967	15.04401496	21.1197045	283.1373735	117.7879139

LNX1	20.5110924	19.2989455	549.6080132	454.576497	126.9583952	454.8425598
ALDH3A2	1340.05803	1738.93656	3908.435087	1942.00712	1454.983514	2173.640042
TFRC	21269.0261	22140.9691	13533.59586	16800.2221	11238.8408	22215.70662
SREBF1	4354.21189	1636.34743	12570.7789	11329.2129	3460.120073	6317.056429
TRPC5	0	0	0	0	0	0
AFF4	6590.89768	5982.6731	3892.388137	4961.11916	4859.685239	5545.092562
UBE2D1	711.051202	731.32846	284.8333499	304.727165	363.7458784	322.5576719
MPP5	3769.1574	2926.32957	1392.072851	2093.86785	1489.242128	2129.243059
RHOBTB1	160.181864	155.407298	31.09096425	19.1083041	388.9360362	54.36365257
SMC1A	7356.64513	7127.40528	4043.831221	5751.59953	6618.965859	5245.186412
HSD17B10	1017.74087	1173.17274	2741.019526	2967.82134	2530.099448	2594.052288
MARK2	1935.85643	2169.60776	3064.967315	3384.18123	2482.741951	3235.543389
HMMR	561.613243	520.055794	386.1297173	855.850883	1125.49625	585.315326
CHFR	768.677604	746.56447	1140.336334	1249.10984	1464.05197	1210.533573
TRHDE	0	0	0	0	1.007606311	0.906060876
P4HA2	1689.72332	2025.37354	629.8427597	726.115556	875.6098845	729.3790053
FCGR2B	0	0	0	0	3.022818934	1.812121752
NFATC3	1112.8928	1226.83396	1265.903712	1723.74	2066.519936	2264.508887
TRNT1	440.500126	477.394967	382.11798	333.892472	1233.310125	566.2880476
ACADVL	4966.61451	5005.53702	3878.347057	1946.02992	3559.873098	3068.828188
STK10	4619.87937	4618.54237	168.4929676	378.143281	1676.656902	633.3365524
FBXW11	2573.65373	2365.64442	1342.929069	1674.49086	2425.308391	1962.527858
ACAP1	15.6274989	12.1888077	5.014671654	5.02850108	4.030425245	3.624243505
CRMP1	2.93015605	10.1573397	1.002934331	2.01140043	2.015212623	0
EVC	1570.56364	1592.67087	467.3673981	346.966574	41.31185876	490.178934
DERL2	1350.80194	1421.01183	485.4202161	374.12048	1139.602738	484.7425688
SIDT1	1.95343737	6.09440383	614.7987447	860.879385	636.8071887	1408.924662
NDE1	1051.92602	974.088879	1731.064655	1894.73921	808.1002617	1513.121663
MRVI1	1.95343737	5.07866986	0	0	1.007606311	9.060608762
TMEM38A	273.481231	306.75166	113.3315794	57.3249123	140.0572773	148.5939837
AP1M1	3986.96567	3843.53735	932.7289276	1237.01127	1376.390221	1218.651878
PVR	4350.21711	3504.22126	452.3233831	483.741804	1273.584149	692.1761457
XRCC1	679.796204	603.34598	1370.008296	1294.33618	1241.370976	1261.23674

SCARB1	1671.16567	1969.50817	477.3967414	500.838707	5240.560425	1528.524698
CYP2W1	0	1.01573397	0	0	1.007606311	0
MCM2	4460.67423	3999.96038	1817.317007	2916.53063	5699.021297	5744.425955
MOV10L1	0	0	4.011737323	0	1.007606311	5.436365257
PANX2	104.508899	120.872343	8.023474646	5.02850108	655.9517086	102.384879
SELO	713.981358	693.746303	1750.120407	1288.30198	565.2671406	1405.300419
TP63	13.6740616	13.2045416	51.14965087	36.2052078	60.45637868	175.77581
ALPK1	58.603121	61.9597723	1139.3334	566.209221	218.6505695	525.5153082
LLGL2	316.456854	360.58556	4710.782551	5631.92121	12309.92631	6732.938371
PDE8A	3708.60084	3156.90119	214.6279468	310.761367	1644.4135	461.184986
CLCN4	2779.74137	2284.3857	25.07335827	20.1140043	122.92797	26.27576541
NLE1	1102.71539	1012.68677	404.1825353	347.972275	617.6626688	747.5002228
SDHA	5633.7329	4672.15281	3453.694632	2794.47885	5074.446449	4616.380164
SMARCE1	2852.99528	2798.34709	1677.909135	1412.0031	2319.882543	1473.254985
FNDC8	0.97671868	0	0	0	0	0.906060876
GSDMB	30.2782792	23.3618814	970.8404321	641.636738	58.44116605	413.1637595
KDM5A	1378.15006	1347.87898	2048.994838	2001.34343	1698.824241	2327.670391
ADAM11	69.3470266	67.0384422	34.09976724	18.1026039	125.9507889	28.08788716
PPP2R3A	1165.22539	955.805668	256.7511887	411.331388	501.787943	532.7637952
FERMT2	2881.32012	2906.01489	877.5675394	782.434768	1090.230029	782.836597
ABCB11	0	0	0	0	0	0.906060876
DHRS9	0	2.03146794	0	0	0	0
CD5L	0	0	0	0	0	0
PTGS2	63.4867145	256.980695	0	0	9.068456802	4.530304381
IGF2BP2	4163.75175	3271.67912	0	0	177.3387108	75.20305272
MAP3K13	588.961366	646.006806	1577.615702	1291.31908	508.8411872	1176.973078
ST6GAL1	383.850443	313.861797	1339.920266	324.84117	16.12170098	298.0940283
TBX21	0	0	0	0	0	0.906060876
VDAC1P1	3.91664192	5.0888272	5.024700997	11.0828164	8.070926553	4.865546905
FRY	63.4867145	68.0541761	297.8714962	347.972275	34.25861458	70.67274834
PICALM	6630.94314	6123.86012	3321.718503	4017.77236	5941.854418	4178.752761
NSF	1805.95285	1800.31736	2022.84834	1943.2039	2622.799228	3105.206532
GLI2	117.206242	148.29716	0	0	19.14451991	9.966669638

CLASP1	2556.0728	2477.37516	1470.301729	1482.40212	2761.848899	1956.185432
MRPS34	1673.11911	1636.34743	3190.334106	2645.99727	4052.592584	2763.485672
NOTCH3	101.578743	71.1013781	4288.547198	4129.40509	9708.286809	8529.657088
CLNS1A	2076.50392	1817.14808	1647.821105	1415.0202	2561.335243	1992.427867
PPP2R2C	348.68857	291.51565	1575.609834	1404.9632	2159.300325	1590.136838
TEAD2	691.516828	654.132678	1007.949002	1363.72949	1088.214816	1263.954922
EED	1094.90164	1103.08709	821.4032169	817.634275	1253.462251	1068.245773
CDHR2	0	0	2.005868661	0	2.015212623	1.812121752
SNCB	0	0	0	0	0	0
TSG101	1845.02159	1695.26	2689.869875	3018.10635	1566.827814	2395.624957
NCBP3	2484.77233	2107.64799	1180.453707	964.466507	2159.300325	1318.318575
ATP2A3	54.6962463	31.4877531	25.07335827	137.78093	9651.860856	1126.233669
CA12	294.969043	324.019137	5975.482742	10218.9199	5368.526427	13919.81324
MGLL	2505.28342	2495.65837	0	3.01710065	1596.048397	528.2334908
NTN4	297.899199	186.895051	1623.750681	1848.477	576.3508101	2719.088689
BCS1L	485.429186	442.860012	550.6109476	392.223084	800.0394112	715.7880922
NUAK1	2182.96626	1629.23729	51.14965087	55.3135119	650.9136771	219.266732
DPP8	1359.59241	1360.06779	1673.897398	1533.69283	1730.060036	1762.288404
SLC24A1	251.016702	227.52441	632.8515627	591.351727	570.3051722	604.3426044
ZNF532	1149.59789	942.590969	775.2682376	956.420905	323.4416259	699.2706024
SCARF1	98.6485871	134.076884	18.05281795	28.159606	90.68456802	56.17577432
LMAN1	6504.94643	6567.73586	4092.975004	3197.12099	2672.171938	4290.198249
HACD3	3729.11193	3617.02868	4110.024887	3752.2675	4877.822153	4749.571113
IPCEF1	1.95343737	1.01573397	0	0	13.0283496	9.912305985
ZZEF1	1936.83315	1865.90331	1417.146209	1744.88987	4361.927722	2074.879406
NOX3	0	0	0	0	0	0.906060876
ENO1	62363.5173	51352.4624	21357.48657	30301.7374	35208.78734	27747.19921
SLC12A1	0	0	0	1.00570022	0	7.248487009
MYDGF	2661.55841	2906.01489	3243.489626	1529.67003	1306.865386	1321.036757
ANO8	741.329481	489.583775	1078.154406	1210.86306	387.9284298	848.979041
TUBE1	240.272796	217.36707	700.0481628	245.390853	158.0632021	357.8940461
ARHGEF10L	409.245129	375.82157	323.9477888	162.923435	779.8872849	360.6122287
TXK	85.9512442	53.8339005	6.017605984	21.1197045	1.007606311	27.18182629

WSCD2	0	0	0	0	0	0
KCNQ2	0	0	3.008802992	0	33.25100827	7.248487009
TACR2	6.83703079	0	270.7922693	181.026039	1.007606311	175.77581
ACTR6	564.543399	617.566255	756.2124854	568.220622	657.9669213	572.6304737
TIPIN	422.91919	485.520839	213.2940441	301.710065	472.1743935	448.5001337
SRI	1513.91396	1381.3982	396.1590606	305.732866	1176.884172	509.2062124
EIF4G3	3320.84353	3303.16688	6386.685818	3843.78622	2845.480223	4896.352975
NUP37	996.253058	983.230485	627.836891	592.357427	859.4881835	639.6789786
SEMA3A	83.9978068	103.604865	0	0	10.07606311	1.812121752
GTSE1	2457.42421	2662.23874	925.7083872	2240.70008	1261.523102	3105.976684
SEMA3C	3008.29355	1962.39803	963.8198918	986.591911	11539.10748	1351.842827
TTC38	1343.96491	1464.68839	1504.401496	1042.91112	399.0120993	1473.254985
ACAT1	3150.89447	2938.51838	1831.358088	1609.12034	696.2559611	1281.170079
GRAMD4	657.331674	797.351168	7276.288569	6533.0286	780.8948912	6302.559455
CELSR1	1450.42725	1271.69893	160.4694929	149.849332	5562.994445	910.5911806
WNT8B	0.97671868	0	8.023474646	4.02280086	9.068456802	15.4030349
ZNF638	1999.34315	1928.87881	4038.81655	3571.24147	3811.774676	3606.122287
SLC25A40	459.057781	452.001618	599.7547298	922.227098	713.3852684	548.1668301
TIMM21	516.684184	613.503319	498.4583624	480.724703	442.3391707	629.7123089
ADD2	450.267313	389.026111	0	0	39.29664614	25.36970453
FGF4	0	0	0	0	0	0
RASAL2	2880.3434	2337.20387	861.5205901	1183.70915	855.4577583	1419.797393
VPS9D1	165.065458	190.957987	391.144389	496.815906	318.4035944	318.9334284
ZNF37A	1304.46641	1336.28946	1198.376144	1079.96112	1017.682374	1106.807724
MARK3	2305.05609	2173.6707	3994.687439	4013.74956	4204.741137	3738.407175
SLC25A3	14934.0287	15172.0183	7366.552659	10283.2847	11594.52582	9317.930051
FNDC3B	6221.69802	6085.26223	1079.15734	1189.74335	1397.549954	1850.176309
FOSL2	9727.14137	8182.75288	1045.057573	1717.73597	6951.475942	1591.948959
CACNG5	0	0	0	0	0	0
CACNG4	10.7439055	0	80.23474646	513.91281	9218.590142	944.115433
FRYL	2375.37984	2076.16024	1260.688454	1507.54462	2588.540614	2257.903703
TMEM131	4288.77174	4862.31853	1612.718404	2297.01929	2794.092301	2638.449271
FSCN1	1144.7143	1273.7304	1.002934331	0	621.6930941	87.88790499

ACTB	121697.966	109093.201	45235.91879	62003.5604	139122.4907	89632.86951
MOCOS	1457.26428	1528.67963	195.5721945	81.4617175	604.5637868	208.3940015
PLD1	465.894812	527.165932	473.3850041	366.074878	113.8595132	131.378827
ATP12A	1.95343737	5.07866986	0	0	2.015212623	3.624243505
WDR62	2094.08486	2250.86648	754.2066167	1517.60163	1309.888205	1749.603552
DLG1	7209.16061	4973.03353	3585.490232	3972.51585	1934.604118	3687.667766
RAB7A	5176.60902	5811.01406	8144.8297	7892.73529	7482.484468	6352.392803
BCAP29	1382.32065	1315.46691	433.5985992	336.356437	1077.131147	505.2195446
SEC31B	293.494197	374.775364	484.0762841	401.978376	17.68349076	202.9213938
SART3	2869.59949	2525.11465	1695.961953	1276.23357	2819.282459	2097.530928
ARHGAP15	0	0	2.005868661	1.00570022	0	8.154547886
TUBA3D	0	0	0	0	4.030425245	0
PAX2	0	0	82.24061512	158.900634	0	86.98184411
EXOSC7	876.94687	808.524242	724.1185868	576.266224	514.8868251	691.3244485
KIFAP3	288.132012	317.924733	857.5088528	630.574035	367.7763036	596.1880565
MKRN2	1379.12678	1284.90347	548.6050789	528.998313	1314.926236	710.3517269
MCM6	3917.61864	3416.92908	1898.554688	2897.42232	4407.270006	4279.325518
REXO2	2923.99296	2718.00254	1170.243836	870.936387	488.689061	834.1649457
RBM7	1475.36287	1256.13789	350.8665463	489.776005	327.4720512	509.8223338
RBMS2	4327.02981	3208.17544	413.2089443	364.988722	340.1779668	625.9612169
BAZ2A	3722.2749	3992.85024	5798.9663	8948.72052	5250.636488	7973.33571
PTPN23	2793.41544	3194.48334	2214.479002	2943.68453	1633.329831	2487.137105
MLH1	1408.42834	1402.72862	1590.653849	1620.18305	1416.694474	1551.17622
UNG	3363.81915	2594.18457	905.6497006	810.594374	3382.534387	2373.879496
FMO4	2.93015605	9.14160575	51.14965087	90.5130194	31.23579565	61.61213958
KLHL20	419.989034	437.781342	662.9395926	596.380228	544.1074081	584.4092651
RGS11	0	0	0	1.00570022	52.39552819	15.4030349
SLC46A1	699.330578	923.302181	532.5581296	396.245885	665.0201655	930.5245198
PLXNA2	828.257444	979.167549	29.08509559	18.1026039	386.9208235	85.16972236
SPAG5	4173.51894	4154.35195	1078.154406	2527.32464	2445.460517	3114.131231
ANKRD13A	3273.96103	2379.8647	1303.81463	1090.17903	798.0241985	1318.318575
TPD52	1376.19663	1428.12196	7797.473424	4596.92494	3197.134826	4789.945186
ACACB	376.036693	436.765608	94.27582709	184.043139	386.9208235	267.2879585

TRAF4	1195.50367	1324.5171	2663.793582	3862.89453	2692.324064	3319.80705
PAG1	695.423703	937.522456	98.28756441	152.866433	50.38031556	328.9000981
GPATCH1	464.918094	512.945656	464.3585951	449.547996	480.6282105	499.2395428
ICAM3	100.602024	153.37583	225.6602244	296.681564	243.8407273	289.0334195
NT5C2	3235.869	2713.02544	702.0540315	583.306125	5504.553279	1284.794322
MCAM	2268.9175	1625.17436	1.002934331	7.03990151	131.9964268	61.61213958
GPC4	0	0	169.4959019	297.687264	268.0232788	366.9546549
MBNL3	56.6496837	58.9125704	947.7729425	969.495008	1196.028691	1162.476104
CAMSAP3	43.9523408	47.7394967	2384.977838	2570.56975	1310.895811	2235.252182
RAP1GAP	55.672965	120.872343	4414.916924	3498.83105	472.56736	2071.255163
XAB2	1775.67457	1694.24427	2597.599917	2015.42323	1247.416613	2099.34305
ARHGEF1	3662.69506	3581.47799	1096.207223	1086.15623	1365.306552	1195.094296
STXBP2	1448.47381	1558.13591	3191.33704	2944.69023	1327.017512	2380.221922
MAP2K7	1965.15799	1893.32812	1419.152078	1639.29135	876.6174908	1540.30349
NMRK2	0	0	0	0	0	0
DGKD	1682.88629	1796.8334	3265.554181	2378.48101	1431.808568	2787.043255
CTTNBP2	0	0	3.008802992	4.02280086	0	3.624243505
ACTL6B	0	0	0	0	0	0
RARB	5.8603121	0	22.06455528	2.01140043	1.007606311	2.718182629
TOP2B	5375.85964	5286.89533	4886.296059	7751.93726	7371.647773	8363.847948
TM9SF3	7318.5531	7269.60804	7814.864305	6891.05788	5442.081687	7686.114413
NFKB2	2329.47406	2684.58489	649.9014463	641.636738	1426.770537	954.0821026
UBE2T	831.1876	884.70429	1545.521804	1718.74167	1488.234522	1655.373221
PPP1R12B	370.176381	325.034871	1485.345744	1349.64969	1481.181278	1641.782308
DNAJC10	5034.0081	25618.0246	5940.156624	24227.9637	2665.118693	2860.725393
GTF3C1	3613.85913	3609.91854	5453.95689	5320.15414	7688.036155	8275.053982
IL4R	1139.8307	1173.17274	30.08802992	67.3819144	746.6362767	141.3454967
USP33	1857.71894	1921.76868	1824.337548	2151.19276	1357.245701	1885.512683
PAK3	0	0	7.020540315	19.1083041	0	17.21515665
CAPN6	0	0	0	0	0	0
DCX	0	0	0	0	0	0
SNRPA	2172.22235	1998.96446	1586.642111	1529.67003	2247.96968	1615.506542
SPAG6	0	0	1.002934331	0	0	0

EXOSC5	813.606664	680.541761	227.6660931	191.083041	719.4309063	391.4182985
DYNC1I2	3169.98932	3516.94841	1804.118391	1942.81168	1980.772639	1512.088754
APBB1IP	990.392745	1044.17452	0	0	15.11409467	9.060608762
LRCH4	1002.23058	1078.9431	607.7481164	1027.75522	1402.910419	709.1194841
FAM76B	729.608857	542.401941	581.7019118	669.796344	261.9776409	681.3577789
SIRT6	600.681991	778.052223	781.2858436	555.146519	366.7686973	594.3759348
TYR	0	0	0	0	0	0
POLD3	1406.4749	1365.14646	1384.049376	936.306901	1384.451072	1473.254985
ACTN2	0	1.01573397	605.7723357	566.209221	0	514.6425777
CAPZB	3492.74601	3307.22981	5849.113017	6300.71185	3235.423866	6647.768649
GPR137B	247.109827	342.302349	285.8362843	266.510557	316.3883817	411.3516378
NAALAD2	0	0	0	0	0	0
JADE1	1367.40616	1199.58182	1449.240108	1332.55279	697.2635674	1309.257966
SLC25A43	839.978068	854.232271	314.9213798	348.977975	1912.436779	209.3000624
UBE2A	2060.87642	2242.74061	3137.178586	2728.46468	2896.868145	2318.609782
FGFR1	4878.70983	5413.86207	302.8861679	451.559397	493.7270925	451.2183163
FKBP6	0.97671868	0	0	1.00570022	0	0
SMC1B	0	0	1555.551147	1061.01373	9.068456802	1699.770204
FBLN1	202.180768	253.933493	123.3609227	300.704364	460.4760843	580.7850216
ITGA8	0	0	0	0	0	0
CST7	213.901392	274.248173	0	1.00570022	0	1.812121752
MAP2	4.88359342	15.2360096	43.12617622	75.4275162	177.3387108	58.89395695
PIAS2	595.798397	582.015566	806.3592019	738.183958	571.3127785	1001.197268
AMPH	53.7195276	59.9283044	0	0	6.045637868	0.906060876
ARAF	1849.90519	1748.07817	1330.893857	1994.30353	1930.573692	1449.697402
MCCC1	1018.71759	956.821402	2301.734289	1828.36299	1045.895351	2203.540051
LAMP3	194.367018	208.225464	0	0	35.2662209	9.966669638
FAP	2.93015605	1.01573397	0	0	0	1.812121752
NEBL	4.88359342	12.1888077	2847.330565	2205.50057	3284.796575	1986.085441
ACER3	1659.44504	1687.13413	1931.651521	1158.56665	1181.922203	1686.179291
UBE2K	2330.45078	22429.6357	2281.675602	22161.2498	2225.802342	2565.05834
PIK3C3	1931.94956	1883.17078	901.6379633	943.346802	356.6926342	902.4366327
N4BP2	415.105441	358.554092	373.091571	463.627799	715.400481	506.4880298

TIGAR	295.945761	345.349551	211.6191438	368.086279	820.1915374	385.0758724
TULP3	909.325095	936.506722	905.6497006	1323.50148	721.4461189	806.3941798
SYNJ2	5011.54357	4586.03888	420.2294846	379.148981	1229.2797	1032.909399
ADCY2	0	0	1567.586359	1134.42984	0	1303.821601
PPP2R5C	3151.87119	3641.40629	3608.557722	4084.14858	3674.740217	4679.804425
PMS2P1	76.0180152	59.9283044	44.12911055	59.8793908	100.3978929	47.31449895
RBFOX1	0	0	0	0	0	0
GNB1	10190.106	9063.39423	10544.85155	10274.2334	7489.537712	10216.74244
HOXA9	0	0	0	0	5.038031556	0.906060876
EDN1	765.747448	431.686938	490.4348877	456.587898	36.27382721	496.5213601
MLLT10	1214.06132	1117.30737	753.2036824	826.685577	1020.705193	701.2911182
ZCWPW1	43.9523408	68.0541761	187.5487198	128.729628	123.9355763	144.9697402
ADCYAP1R1	0.97671868	0	0	0	0	0
FGF20	0	1.01573397	0	0	0	0
P2RY10	0	0	0	0	1.007606311	0
ITM2A	0	0	0	0	1.007606311	0
NRD1	4731.2253	4723.16297	4532.26024	5811.94155	4667.232434	5153.674264
VDAC3	2228.87204	2096.47492	2383.974904	2723.43618	1894.299865	2449.082548
PCM1	2391.00734	2422.52552	1853.422643	2099.90205	3377.496355	2375.691617
TNRC6C	771.60776	857.279473	765.2388943	982.569111	769.8112218	848.979041
CBFA2T2	1235.54914	1174.18847	1320.864514	1582.97214	915.914137	1311.976149
BRINP1	0	0	0	0	0	0
ITCH	3588.46444	3075.64247	2080.085802	1899.76771	2024.281079	2382.034043
PKD2L2	0	0	0	0	1.007606311	0
TP53INP2	1900.69456	1865.90331	2031.944954	5362.39355	155.1713719	2367.537069
SDF4	3102.05854	2956.80159	4278.517855	4649.3521	3675.747824	4784.001426
MYH7B	0.97671868	0	13.0381463	18.1026039	4.030425245	11.77879139
BPIFB2	0	0	909.661438	168.957636	1.007606311	113.2576095
TP73	269.574357	242.760419	439.2852369	468.6563	378.859973	748.4062837
TOLLIP	1743.44285	2213.28433	2105.15916	2066.71394	773.8416471	1428.858002
UBE2D4	479.568874	471.300563	398.1649293	360.040677	358.7078468	400.4789073
CLUL1	0	0	1.002934331	3.01710065	0	0
RUNX1T1	0	0	0	0	11.08366942	0

CDH17	0	0	0	0	0	0
THOC1	1450.42725	1432.1849	474.3879384	405.297187	626.7311256	536.3880387
FKBP7	259.80717	352.459688	1.002934331	3.01710065	12.09127574	7.248487009
OSBPL6	588.961366	700.856441	860.5176557	472.679101	172.3006792	499.2395428
SLC1A3	263.714045	238.697483	88.2582211	71.4047153	3.022818934	51.64546994
XRCC5	10225.2679	9543.8364	7273.279766	7412.01059	12787.5317	9507.296774
LXN	268.597638	203.146794	0	0	4136.223908	808.2063016
SP140	341.861307	522.320881	9.026408976	1.00570022	7.103624495	3.624243505
MKNK1	720.818389	781.099425	430.2588279	618.505633	866.5414277	590.7516913
TNS1	733.515732	1139.65352	92.26995843	85.4845183	318.4035944	251.8849236
REXO1	1873.34644	1791.75473	1030.013558	989.609012	1072.093115	1336.439792
SAR1A	3861.94568	4209.20158	2316.778304	1920.88741	1931.581299	2421.900722
CDC14A	294.969043	311.830329	42.12324189	87.4959188	236.7874832	125.0364009
RAPGEF3	122.089835	96.4947274	3060.955577	1856.5226	54.41074081	1513.121663
CEACAM1	47.8592155	53.8339005	4.011737323	0	2.015212623	45.30304381
SENP1	976.718684	955.805668	630.845694	773.383466	1379.41304	1009.351816
DUSP13	0	4.06293589	0	0	10.07606311	1.812121752
CIC	3378.46993	3862.8363	3010.808861	6821.66456	2644.966567	3727.534445
LIPE	118.182961	150.328628	239.701305	730.138356	15.11409467	182.1182361
FDFT1	1278.52476	3475.84165	1828.349285	20859.2282	2643.958961	1803.061144
PAFAH1B3	1320.52366	1336.70591	1879.498936	2639.96307	839.3360573	1925.379362
OPHN1	1369.35959	1018.78117	835.4442975	769.360665	448.3848085	664.1426222
AFM	0	0	0	0	0	0
KIF22	2078.45736	2110.69519	3879.349991	4128.39938	4120.102207	4987.865123
SCGN	0	0	0	0	0	0.906060876
LRRC16A	482.49903	479.426435	1012.963674	914.181496	930.0206253	1514.027724
PGM1	3527.90789	3947.14222	2108.167963	1494.47052	363.7458784	2079.409711
DDX1	3571.86023	3668.83111	3052.932103	2698.29368	4075.767529	3223.764597
DNM2	6279.32442	5865.86369	6434.826666	5689.24612	7124.784227	6905.089937
EPB41L2	2975.08511	2552.53947	1248.653242	1404.9632	1219.203637	1825.712665
RIMS1	28.3248418	14.2202756	0	1.00570022	0	2.718182629
MOXD1	136.740616	142.202756	0	0	5.038031556	2.718182629
STX7	1431.86959	1471.79853	1230.600424	1363.72949	735.5526072	727.5668836

RABL2B	254.80637	297.366278	869.8549744	807.416361	388.7949713	653.1339826
KEAP1	2634.21029	2571.83842	1584.636243	2256.79128	2640.936142	2068.53698
DDX43	0	0	0	0	78.59329228	3.624243505
PTPRH	113.299367	71.1013781	5.014671654	0	53.4031345	39.86667855
DCT	0.97671868	1.01573397	0	0	0	0
SLC35C2	1427.96272	1609.93835	1463.281189	1410.9974	1299.812142	1172.442774
CRYBG3	1505.12349	1502.27054	234.6866334	419.37699	542.0921955	589.8456304
EPHA6	1.95343737	3.04720192	5.014671654	19.1083041	169.2778603	16.30909577
SCTR	0	0	0	0	0	0
RFX3	158.228427	131.029682	25.07335827	21.1197045	201.5212623	38.96061768
RIF1	6361.36879	5140.62963	1426.172618	1894.73921	4856.66242	2988.18877
RAB21	1881.16019	1884.18652	1844.396234	2196.44927	3367.420292	2116.558207
SLC4A4	41.9989034	46.7237627	0	0	2.015212623	0
SMARCA2	1147.64445	1284.90347	3033.87635	2939.66173	1479.166065	2511.600749
RDH8	0	0	0	0	0	0
SESN1	165.065458	223.461474	388.135586	666.779243	553.1758649	697.6668747
MID2	135.763897	127.982481	487.4260847	715.052853	1125.49625	453.936499
PIH1D3	0	0	0	0	0	0.906060876
COL5A3	0	0	0	0	5.038031556	0
SRCAP	3591.57041	3456.09578	4508.179787	5984.63033	6418.411899	8632.458755
PUM3	2223.98844	2087.33331	434.2705652	394.234484	1525.515955	595.2819956
CPB2	0	0	0	1.00570022	0	0.906060876
CHRNA3	0	0	0	0	12.09127574	0.906060876
KCNN2	0.97671868	0	4.011737323	1.00570022	1.007606311	5.436365257
CNOT4	754.026824	801.414104	561.6432252	581.294725	1096.275667	702.197179
PSEN1	2223.98844	2139.13575	2604.620457	2362.38981	1704.869879	2771.64022
CPOX	1614.51598	1833.39982	378.1062427	426.416891	630.7615509	420.4122465
CLDND1	3566.97663	3388.48853	892.6115543	797.520271	885.6859476	487.4607514
MOK	611.425896	498.72538	335.9830008	169.963336	230.7418453	428.5667944
HSP90AA1	51963.0358	55245.9739	40063.81654	37730.503	84268.24186	63721.05063
RBL1	2213.24454	2106.63226	447.3087115	554.140819	1081.161572	851.6972236
DLGAP4	3597.25491	3451.46404	1724.044114	1836.40859	1659.527595	2470.828009
IGSF9B	5.8603121	4.06293589	552.6168162	341.938073	87.66174908	395.042542

CFHR2	0	0	0	0	0	0
CROCCP3	79.250954	79.9890503	77.80764538	64.5257258	101.0729891	74.29699185
NDC80	1969.06487	2048.73542	161.4724272	358.029277	866.5414277	394.1364811
AP4E1	1036.29852	874.54695	615.8016791	788.468969	2508.939715	1157.9458
RSBN1	1030.43821	869.46828	560.6402909	553.135119	1307.872992	684.9820224
MAGI3	797.002446	727.265524	995.9137904	2097.89065	898.7848297	1416.173149
CXCL2	236.737075	455.04882	0	0	12.09127574	9.966669638
AFP	0	0	31.09096425	12.0684026	1.007606311	10.87273051
COL4A4	61.5332771	74.14858	0	0	14.10648836	0.906060876
TCF7	135.763897	126.966747	0	0	36.27382721	4.530304381
OSTM1	620.216364	1030.96998	596.7459268	864.902185	683.1570791	481.1183253
CDH7	0	0	60.17605984	77.4389166	0	145.8758011
IMPG2	8.79046815	3.04720192	4.011737323	18.1026039	4.030425245	13.59091314
PCNP	3680.276	3820.17547	2212.473134	2530.34174	2535.137479	2719.99475
EXD2	468.824968	538.339005	1399.093391	1363.72949	769.8112218	1249.457948
ARG2	438.546689	443.875746	287.8421529	454.576497	31.23579565	579.8789608
MEF2C	19.5343737	24.3776153	159.4665586	69.3933149	19.14451991	206.5818798
PTPRC	2.93015605	0	0	0	0	0
CACNA1S	0	0	0	0	0	0
PKP1	0	0	0	1.00570022	648.8984645	9.060608762
UBA5	844.861662	887.751492	1557.557016	1360.71239	2420.27036	1411.642845
STK17B	1199.41054	1373.27233	442.2940398	523.969812	420.1718318	681.3577789
CDC14B	1257.03695	1214.81783	25.07335827	13.0741028	464.5065095	57.98789608
ZNF510	595.798397	525.134464	532.5581296	462.622099	683.1570791	426.7546727
LRP2	1.95343737	2.03146794	20.05868661	115.655525	11.08366942	33.52425242
ZNF506	302.782792	319.803841	531.5551953	376.131881	184.391955	415.8819422
JMJD4	691.516828	584.047034	1276.735403	1198.79466	768.8036155	1525.806515
DUSP12	671.005736	608.424649	713.0863091	598.391628	494.7346988	559.0395606
AACS	1028.48477	1419.99609	2214.479002	3808.58672	2242.931649	2554.18561
KIAA0141	1136.90055	1191.45595	1145.351006	1217.90296	870.571853	1119.891243
SLC13A1	0	0	0	0	0	0
CADPS2	139.670772	206.193996	843.4677721	1103.25314	287.1677987	1325.567062
PCDHB4	1.16229523	9.14160575	34.09976724	35.3101346	135.4827446	35.19140443

PCDHA6	0	0	202.7130869	273.168293	351.715059	221.1603993
PCDHGA2	2.93015605	5.52559281	6.087811387	1.00570022	370.7386662	20.06924841
HSPB11	771.60776	718.123918	694.0305569	687.898947	369.7915162	680.451718
PHLPP1	1049.97259	685.620431	397.161995	480.724703	585.4192669	360.6122287
ATP8B1	1978.83205	1340.76884	4250.435694	4955.08496	2273.159838	3364.204033
IL12RB2	1.95343737	2.03146794	0	0	0	0
SMARCD3	251.99342	220.414272	1552.542344	923.232798	165.2474351	1290.230688
WDR70	823.37385	685.620431	681.9953449	529.470992	420.1718318	660.998591
FYB	0	0	0	0	1.007606311	5.436365257
MPP4	85.9512442	171.659041	0	3.01710065	13.09888205	3.624243505
STRADB	1221.33788	1276.27989	198.5007627	229.299649	396.9465063	157.61835
BZW1	14604.3471	13664.3847	3954.168892	5852.57183	12949.64548	8805.507322
PGR	0.97671868	1.01573397	18.05281795	11.0627024	1219.203637	67.04850484
C1QTNF3	25.3946858	16.2517436	24.63206716	71.1331762	23.17494516	28.08788716
ME2	1191.59679	1148.79512	424.2412219	641.636738	651.9212834	803.6759972
C5orf22	1163.27195	1085.81962	1051.075179	866.913586	923.9749875	1320.130697
CCNT2	1218.94492	1095.97696	1327.885054	1154.54385	1352.20767	1395.333749
FAM135A	458.081063	460.127489	0	0	74.56286704	16.30909577
COL19A1	0	3.04720192	0	0	0	0
EPB41L3	0	0	0	0	0	0
COBLL1	246.133108	222.44574	1667.879792	1751.92978	2161.315538	1451.509524
DLG3	688.586672	784.146627	3964.599409	3473.68854	1917.47481	2393.812835
KCNK2	6.83703079	7.11013781	158.4636243	115.655525	15.11409467	683.1699006
SERTAD4	853.65213	389.026111	2964.673882	3518.94505	217.6429632	3369.640399
TRAF5	769.654323	659.211348	925.7083872	493.798806	557.2062901	1466.006498
MRPL22	689.563391	730.312726	600.7576641	564.197821	744.621064	596.1880565
GEMIN5	2157.57157	2057.87703	438.2823025	532.015414	2512.97014	1279.357957
OPRK1	0	0	0	0	0	0
NFE2L1	9178.22547	9175.12497	10544.85155	6011.07019	8100.147136	6342.426133
SEMA5B	0	0	2.005868661	0	2.015212623	7.248487009
GSK3B	2054.03939	1995.91726	5122.988561	6424.41298	3363.389867	3935.022385
ITGB5	4246.77284	3623.12308	943.7612052	1283.27348	2161.315538	1156.133678
ERC1	1676.04926	1747.06243	877.5675394	1163.59515	1719.983973	1507.685298

XPO1	10768.3235	10342.2033	7291.332584	7283.28096	10127.45103	10249.36063
LINC01587	0	0	0	0	5.038031556	0
RNF13	791.142134	885.720024	835.4442975	808.582973	1025.743225	752.0305272
TRPM3	0.97671868	2.03146794	0	0	1.007606311	0
PALB2	591.891522	569.826758	914.6761096	1157.56095	749.6590956	1309.257966
DOPEY1	408.26841	366.679964	1094.201355	1170.63505	868.5566403	1147.97913
LYRM2	806.769633	1059.41053	1107.239501	1335.56989	1389.479027	1016.600303
BCKDHB	378.966849	340.270881	497.455428	466.6449	340.5709332	612.4971523
KAT6A	2868.62277	2518.00452	2771.107556	2919.54773	2061.562513	3322.525233
ZCCHC6	887.837284	893.845896	902.6408976	976.534909	1028.766044	769.2456839
ULK2	703.237452	588.10997	523.5317206	579.283324	586.4268732	423.1304292
GRHL2	0	0	4078.933923	2994.97524	4488.886117	3723.00414
TNPO1	7852.81822	8273.1532	2407.042394	2932.62183	5113.60203	4370.837667
PLOD1	2659.60498	3072.59527	2086.103408	1952.06412	1754.242588	3173.025188
P2RX5	542.362118	488.090646	11.60395021	3.31881071	135.5835052	27.41740211
ITGAE	439.523408	426.608268	220.6455528	268.521958	349.63939	295.3758456
DIS3	2094.08486	1949.19349	897.626226	726.115556	1461.029151	915.1214849
PIBF1	499.103247	448.954416	483.4143474	270.533358	215.6277506	281.7849325
TDRD3	439.523408	465.206159	248.727714	320.818369	422.1870444	260.9455323
AC000111.6	0	0	0	0	0	0
NUFIP1	422.91919	406.283432	495.43953	392.223084	456.435583	501.9577254
PDS5B	1819.62691	1476.8772	1727.052917	2455.91993	2206.657822	2983.658465
OXCT1	477.615436	489.583775	1035.028229	977.54061	257.9472157	1458.758011
RRAGB	309.619823	325.034871	436.2764339	437.479594	108.8214816	311.6849414
EPYC	0	0	0	0	0	0.906060876
CYLD	1828.41738	1527.66389	1107.239501	1442.17411	626.7311256	1356.373132
SLC27A5	52.7428089	56.8811024	159.4665586	255.447855	204.5440812	173.0576274
ZNF324	415.964953	413.342783	322.0422136	434.27141	439.356656	446.2440421
ZNF671	83.0210881	89.3845896	2.005868661	0	3.022818934	4.530304381
ZNF416	164.088739	159.470234	171.5017706	141.80373	115.8747258	123.2242792
ZNF586	209.994517	172.04502	190.7781684	147.837932	221.0990529	164.0151398
ZNF446	234.412484	261.795274	427.1898488	452.524869	327.1999975	310.0812137
ZNF264	724.725263	666.321486	819.3973482	782.434768	51.38792188	901.5305718

RPS5	9703.70012	10111.6317	9387.465335	9596.39146	13643.99706	8813.254143
FAT1	12343.7707	10867.3378	1933.65739	3375.12992	4160.406459	3907.840559
YTHDC1	2119.47954	1894.34386	2347.869268	2253.77418	2134.110167	2483.512862
CHMP2B	958.161029	1208.72343	1644.812302	2094.87355	2378.958501	1756.852039
SMAP2	621.193083	679.526027	1347.94374	1126.38424	1135.572313	1757.7581
PPIE	796.025727	732.344194	909.9823769	782.09283	1164.379777	953.8737086
ZMPSTE24	1984.69237	2125.9312	2070.056459	2221.59178	2709.453371	3555.382878
STARD7	5250.83964	5351.9023	3132.163915	3261.4858	7187.255818	5314.047039
NOA1	685.656516	615.534787	819.3973482	1034.86552	1205.097148	840.8244931
REST	1655.53817	1518.52229	1403.105129	1649.34835	2461.582218	1893.667231
HAL	7.81374947	9.14160575	12.03521197	14.079803	2.015212623	6.342426133
SSH1	6382.8566	5585.52111	896.6232917	846.799581	2293.311964	1353.654949
GSTP1	6077.14365	6524.0593	0	0	574.3355974	305.3425153
APLP2	28640.322	27324.2596	8015.451171	9558.17485	9968.249238	10493.99707
FAM234B	420.965753	368.711432	633.854497	600.403029	1387.473891	636.960796
SLCO1A2	3.90687474	1.01573397	0	0	3.022818934	0
WBP11	2477.9353	2371.73883	2631.699684	2652.03147	3520.576452	3589.813191
EIF3I	4271.1908	4205.13865	4511.19862	3361.05012	3972.991685	5383.813726
NKAIN1	5.8603121	5.07866986	3.008802992	2.01140043	178.3463171	15.4030349
COL16A1	137.717334	251.902025	418.2236159	151.860733	13.09888205	291.7516021
TXLNA	3263.21712	3373.25252	2494.29768	2628.90036	1687.740571	3096.916075
APOB	0	0	0	0	1.007606311	0.906060876
NCOA1	1171.0857	1388.50834	975.8551038	2001.34343	2427.323604	1539.397429
AGBL5	773.561198	702.887909	559.6373565	863.896485	947.1499326	800.9578145
EFR3B	127.950148	277.295374	172.5047049	407.308587	576.3508101	575.3486564
KIF3C	1675.07254	1984.74418	61.17899417	454.576497	1234.317731	297.1879674
RAB10	5028.14778	5510.3568	5807.992709	4662.4262	4607.783662	4400.737676
GCKR	6.83703079	6.09440383	1.002934331	0	0	0.906060876
HADHA	5096.51809	5335.65056	3915.455627	4219.9181	5791.721077	4542.989233
MAPRE3	180.692957	174.706243	551.6138819	462.622099	602.5485742	441.2516467
CAD	7279.48435	6821.66936	1899.557622	2365.40691	4887.898216	4018.379986
CD59	6814.69323	6532.72351	2453.869398	3652.70318	4261.16709	2586.803801
CD82	2696.72029	3876.04084	1244.641504	1697.62196	116.8823321	1992.427867

BCORL1	935.696499	913.144841	420.2294846	514.91851	484.6586357	538.2001604
ATRX	4098.3116	3405.75601	4202.294846	4835.40664	3670.709792	3409.507077
AK6	729.149799	893.348186	895.5000052	822.803574	490.7042736	654.2303163
FCN1	0	0	0	0	0	0
MYNN	664.168705	512.945656	969.8374978	915.187196	939.0890821	983.9821115
MECOM	1652.60801	1313.34403	137.4020033	157.894934	319.4112007	183.9303579
SCAMP1	1598.88849	1595.71807	1686.935544	1607.10894	1655.497169	1658.997464
PREP	1406.4749	1416.94889	892.6115543	950.386704	1445.915057	1188.75187
HACE1	567.473555	446.922948	449.3145802	494.804506	232.7570579	345.2091938
SEH1L	4143.24066	3682.03565	936.7406649	1293.33048	1746.181737	1554.800464
WDR47	689.563391	715.076716	270.7922693	363.057778	568.2899596	299.90615
WDFY1	2304.07938	1715.57468	1212.547606	1246.06257	1748.19695	1274.827653
OVGP1	4.88359342	5.07866986	126.3697257	65.370514	9.068456802	130.4727662
SLC25A24	3808.22615	3290.97807	802.3474646	1205.83456	5202.271385	1348.218584
MAP3K4	886.860565	859.310941	567.6608312	820.651376	634.7919761	951.36392
PILRA	4.88359342	8.17665848	32.23430939	8.06571573	3.032894997	12.71203409
IGSF9	1.95343737	2.03146794	681.9953449	1099.23034	432.2631075	1013.88212
ABCB1	0	1.01573397	0	0	465.060693	183.9303579
ZNF213	174.832644	176.737711	1315.849842	954.409505	865.5338214	899.71845
AKR1B1	7073.39671	7689.10617	0	0	343.5937521	180.3061144
CPNE3	4271.1908	4615.49517	4230.377007	6305.74035	11728.53746	6137.656375
RRN3	1144.685	1039.56309	1883.450497	1539.08338	3228.511686	2599.497714
CTTN	7734.63526	7307.1902	21149.87917	14729.4854	11785.97102	16677.86255
WNT11	0	4.06293589	14.04108063	7.03990151	14.10648836	10.87273051
MTIF2	1761.02379	1820.19528	1095.204289	1052.96813	1572.873452	1269.391288
DDHD2	1143.73758	825.791719	774.2653033	648.676639	714.3928747	627.9001872
TTC39A	30.2782792	47.7394967	5496.080132	5177.34471	2508.939715	2709.12202
EPS15	1993.48283	1994.90152	1637.791762	1548.77833	2090.783096	1291.136749
ORC1	561.613243	576.936896	206.6044721	424.405491	842.3588762	752.0305272
MGST2	241.249515	287.452714	495.4495594	460.610699	205.5516875	360.6122287
CHERP	3041.50198	3081.73687	2229.523017	2077.77665	3160.860999	3297.155528
ATG16L1	607.519021	671.400156	1366.999493	1098.22464	1019.697587	1224.088244
USP40	1943.67018	1694.24427	987.8903157	775.394866	1079.146359	848.0729801

POMGNT1	1696.56035	1915.67427	1518.442577	1726.78727	1629.299405	1973.400588
RAD54L	630.96027	565.763823	340.9976724	514.91851	739.5830325	1154.321556
MAST2	2256.22016	2236.64621	3877.344123	3207.17799	2581.48737	4017.473925
DNAJA1	7274.60076	7693.16911	7567.139525	6469.66949	7505.659413	9274.439129
B4GALT1	4314.16643	3645.46923	8190.964679	5830.04415	6892.027169	6390.44736
CHMP5	1570.56364	1666.81945	2780.133965	2443.85152	1282.682834	1943.500579
NFX1	1473.86849	1343.81605	2873.406857	3271.5428	2124.034104	2886.709952
AQP6	0.97671868	0	6.017605984	6.03420129	1.007606311	1.812121752
DIMT1	1294.15226	1424.05903	300.8802992	394.234484	805.0774427	603.4365435
IPO11	2060.87642	2253.15188	778.2770406	824.674177	1182.929809	1341.477491
FOLH1	0	0	4.011737323	5.02850108	0	13.59091314
EIF2AK1	2903.78465	2777.01668	5043.756749	5482.07187	6008.356434	5466.265266
NME8	0	0	0	0	0	0
EPDR1	1032.39165	1068.55214	0	0	130.9888205	45.30304381
SNX10	98.6485871	85.3216537	2.005868661	3.01710065	500.7803367	31.71213067
SEPHS1	2720.16153	2470.26502	1689.944347	1552.80113	4260.159484	1747.79143
MRPL28	1282.43163	1129.49618	2090.115145	1772.04378	2493.82562	2150.082459
HBQ1	0	0	0	0	0	0
ITPKC	298.875917	318.940467	1050.072244	844.788181	804.0698364	702.197179
CEACAM6	0	0	0	0	265.0004599	717.6002139
FAT2	0	0	0	0	0	5.436365257
RBM22	1393.77756	1528.67963	924.7054529	1011.73442	1413.671655	1215.027635
TMED2	7812.77275	8123.84031	10359.3087	7414.02199	8268.41739	9122.220901
ERO1B	409.245129	360.58556	826.4178885	437.479594	80.6085049	468.433473
ZFAND6	1458.241	1455.53662	1364.993624	1357.69529	1403.595592	1089.085173
HSD17B2	3.90687474	1.01573397	10.02934331	3.01710065	1.007606311	9.966669638
TXLNG	2114.59595	2139.13575	960.8110888	991.620413	1980.954008	1435.200428
PPEF1	0	2.03146794	11.03227764	23.131105	2.015212623	23.55758278
LAT2	93.7649937	198.068125	1.002934331	7.03990151	35.2662209	22.6515219
HUWE1	22085.5629	20782.9328	12438.39157	14287.983	22409.16436	17879.29927
ZW10	1655.53817	1495.16041	436.2764339	632.585436	570.3051722	806.3941798
ALG9	1315.64007	1238.17971	685.0041479	682.156399	471.5597537	656.5770139
MYBPC2	0	0	0	0	0	0

NOX4	0	0	0	0	0	0
ACOX3	220.738423	233.618814	478.3996757	440.496694	525.9704945	640.5850395
MTMR2	2726.02185	2862.33833	876.564605	915.187196	953.1955705	1188.75187
PPP1R15A	3385.30696	4844.03531	445.3028428	471.673401	459.4684779	451.2183163
HSD17B14	1.95343737	7.11013781	14.04108063	9.05130194	15.11409467	26.27576541
TRIP6	1786.41847	1432.1849	2313.769501	2280.92809	4053.60019	2686.470498
ACHE	7.81374947	17.2674775	2.005868661	4.02280086	21.15973254	20.83940015
FTL	33397.8796	58995.7793	5983.496188	6786.455	6301.559795	6007.174548
SRRT	3828.73724	3971.51983	2481.259534	2417.70332	3759.379147	3651.425331
BAX	1626.23661	19770.4243	1597.748861	18791.4861	1674.641689	1795.521449
NLK	864.396035	922.286447	571.6725685	629.568335	629.7539446	566.2880476
PIGS	2003.25002	1917.70574	1366.999493	1550.78973	2053.501662	2189.043077
ADAMTS2	0	0	0	0	0	0.906060876
TMPRSS11E	0	0	1.002934331	0	9.068456802	3.624243505
ATXN7L3	2131.20017	2232.58327	1043.051704	1021.79142	1814.698967	1022.036668
PGS1	501.056685	496.693912	560.6402909	454.576497	660.9897402	530.9516734
PSMC5	4207.70409	4419.45851	9110.65546	8281.94128	8866.935539	10812.02444
UIMC1	967.928216	981.199017	589.7253865	644.653838	1013.651949	515.5486385
CETP	0	0	1.002934331	0	0	0
MMP2	6.83703079	5.07866986	2.005868661	1.00570022	1.007606311	0
MT3	0	0	0	0	0	0
LPCAT2	495.108468	473.332031	124.363857	119.678326	341.5785395	98.7606355
GNAO1	790.165415	609.440383	64.18779717	70.3990151	54.41074081	235.5758278
OGFOD1	1344.94163	1196.53462	1178.447839	997.654614	1368.329371	1618.224725
SH3BP2	2076.50392	1593.6866	853.4971154	613.477131	2088.767883	962.2366505
NOP14	2513.09717	2511.91011	909.661438	931.2784	2165.345963	1726.95203
ADD1	5048.65888	4502.7487	2422.086409	2597.72366	3478.256987	3081.51304
L2HGDH	970.858372	884.70429	637.8662343	661.750742	1163.78529	1125.327608
TXNDC16	365.292788	429.65547	774.2653033	1632.25145	337.5481143	978.5457463
C14orf166	3762.32037	4003.00758	3355.818271	2819.9834	2508.939715	2808.788716
NID2	0	1.01573397	0	0	0	0
GMCL1	1459.21771	1478.90866	1158.389152	1126.38424	1148.671195	1272.10947
SF3B2	8977.99814	9041.04809	9356.374371	8461.96161	10102.26088	10163.28485

KLHL42	824.350569	930.412319	1648.82404	1055.98523	1652.474351	1329.191305
GNAS	19219.8703	17451.3254	32724.74428	30492.8305	42745.68254	31924.14891
DNM1L	2628.34998	2551.52374	2148.285336	2038.55434	2311.448878	2123.806694
PTHLH	9.76718684	20.3146794	1.002934331	2.01140043	20.15212623	8.154547886
PHACTR3	0.97671868	0	0	0	0	0
ERGIC2	1229.68882	1249.35279	1825.340482	1427.08861	1274.621984	1547.551977
TFAP2C	924.952594	1166.0626	840.4589691	990.614712	4455.635109	1009.351816
AURKA	4840.5885	5068.48205	1021.990083	1706.60287	3790.584715	1581.891684
CASS4	0.97671868	1.01573397	2.005868661	5.02850108	39.29664614	10.87273051
PIR	169.949051	265.106567	904.6467663	782.434768	685.1722917	438.5334641
AAMDC	201.204049	206.193996	225.6602244	261.482056	83.63132384	181.2121752
RFX2	413.152003	373.790102	293.8597589	296.681564	194.4680181	341.5849503
METTL2A	887.202416	1002.88494	1521.561702	1558.74482	2272.807176	1930.308333
SULT2B1	2.93015605	6.09440383	32.09389858	145.826531	1913.444385	212.9243059
ALG6	420.965753	495.678178	149.4372153	237.345251	386.9208235	210.2061233
CNOT3	2253.29	1977.63404	1991.827581	1911.83611	2201.61979	2142.833972
GP6	0.97671868	0	8.023474646	0	4.030425245	31.71213067
PTPN4	543.055588	566.779557	896.6232917	963.460806	781.9024976	1023.84879
DDX18	4050.45238	3891.27685	1777.199634	1750.92408	4185.596617	2478.982557
KHSRP	11785.0876	11023.7608	6025.629459	5816.97005	6245.143917	7215.868818
GNA11	5318.23323	5360.02817	3461.126375	3303.72521	3022.818934	3323.431294
ASAP3	807.746352	924.317915	2647.746633	2198.46067	322.4340196	1631.815638
EDEM2	490.312779	493.646711	714.0892435	503.855808	371.8067289	418.6001248
DNMT3B	653.4248	549.512079	493.4436907	431.445392	1272.606771	1113.548817
REM1	0	0	0	0	0	0
TPX2	12316.4226	10825.6927	2125.217847	4181.7015	5747.3864	5196.259125
FER1L4	91.8115563	70.0856441	97.28463008	144.820831	524.9628882	212.018245
PDRG1	646.587769	501.772582	913.6731753	624.539834	913.8989243	1430.670123
EPB41L1	1706.32754	1853.7145	770.253566	803.554472	4194.665074	2095.718807
SLC15A1	0	4.06293589	0	0	0	0
DOCK9	7970.02446	9600.71751	559.6373565	1080.12203	1251.447039	949.5517982
ANKRD10	809.699789	863.373876	805.3562676	723.098455	467.5293284	725.7547618
TGDS	183.623113	233.618814	155.4548213	113.644124	219.6581759	132.2848879

DOCK3	273.481231	255.964961	1.002934331	0	67.50962286	30.80606979
C3orf18	399.477942	541.386207	5.014671654	15.0855032	41.31185876	14.49697402
COQ9	513.754028	640.928136	1261.691388	1434.12851	923.9749875	1767.724769
TMEM40	40.045466	59.9283044	2.005868661	1.00570022	38.28903983	21.74546103
KIF9	83.9978068	90.4003235	84.24648378	67.3819144	77.58568597	96.94851375
ARHGAP28	6.83703079	4.06293589	3.008802992	9.05130194	0	6.342426133
CRLS1	1057.78633	1073.63081	1177.444904	946.363903	603.5561805	966.7669549
DEFB127	0	0	0	0	0	0
PPP1R13B	516.684184	556.622217	796.3298586	1030.84272	2296.334783	1310.164027
ATRN	2369.51953	2502.76851	1511.422036	1795.17488	1671.61887	2126.524876
SMOX	349.665289	314.877531	5.014671654	2.01140043	195.4756244	37.14849592
SIGLEC1	0	0	0	0	1.007606311	0
FKBP1A	11010.8427	11349.4254	2340.196821	2439.05433	3520.415235	3902.63977
NSFL1C	1977.85533	2011.15327	1830.355154	1777.07228	1337.093575	1906.352083
SLC4A11	394.594348	521.071528	419.2265502	498.827307	174.3158919	561.7577432
C20orf194	700.307296	712.029515	517.5141146	486.758904	206.5592938	402.291029
ZNF343	436.925336	373.079088	291.8538902	296.309455	185.3995613	280.0634168
EBF4	503.010122	446.922948	215.6308811	310.761367	22.16733885	144.0636793
CPXM1	0	0	0	0	0	2.718182629
MAVS	7009.90999	7970.46448	5340.625311	4268.19171	7157.027629	5054.007567
RP5-1187M17.10	352.595445	363.632762	510.4935743	289.641662	50.38031556	375.1092027
F11	0	0	0	0	0	0
XRN2	5145.35403	4836.92518	2977.712028	3809.59242	3516.546026	3635.116235
KIZ	252.970139	233.618814	298.8744306	234.32815	131.9964268	274.5364455
DYNLL1	4259.47018	4589.08609	4543.292518	5955.75668	3735.196596	4722.389287
TESC	1.95343737	4.06293589	0	0	50.38031556	18.12121752
SNX5	3245.63619	2674.42755	2915.530099	2613.81486	4259.151878	3952.237542
RPL6	24226.7256	24041.0824	17811.26122	13959.7023	25271.40108	15683.94095
SIRPG	0	0	0	0	0	0
MAPKAPK5	1258.99038	1151.84232	1628.835558	922.227098	1751.219769	1455.133767
P2RX7	0	0	0	0	2.015212623	0.906060876
ESF1	1478.75209	1638.3789	1130.306991	805.565873	942.1119011	962.2366505
RBBP9	1246.29304	1279.82481	616.8046134	737.178258	789.9633481	1005.727573

ANAPC5	3889.2938	3699.30313	2925.559443	3102.58516	4943.316563	3848.040541
SLC23A2	1642.84083	1832.38409	932.7289276	475.696202	783.9177102	856.227528
SLC8B1	2504.30671	2359.55002	153.4489526	227.288249	400.0197056	264.5697758
TMEM230	2203.47735	2286.40701	1820.32581	1479.38502	799.0318049	1233.148852
DZANK1	78.1374947	73.132846	186.5457855	160.912034	79.60089859	206.5818798
KDM2B	1681.90957	1556.10445	970.8404321	1253.10247	1824.77503	1315.600392
CFAP61	0	0	0	1.00570022	0	0.906060876
LHX5	0	0	0	0	0	0
TASP1	134.787178	118.840875	311.9125769	224.271148	456.445659	322.5576719
OAS1	22.4645297	19.2989455	35.10270157	40.2280086	49.37270925	987.606355
GCN1	9199.71328	8789.14606	4905.351811	4624.20959	9410.035341	9703.911984
RPLP0	40336.6259	41772.1206	26829.74701	31062.9922	49079.71749	29749.60281
PXN	10019.1803	10012.0898	2960.662144	3636.61198	9767.735582	5013.234828
SIRT4	7.81374947	19.2989455	15.04401496	13.0741028	23.17494516	19.0272784
RPH3A	0	0	0	0	0	1.812121752
KIF16B	120.136398	189.942253	557.6314879	939.324001	677.1114412	575.3486564
TRMT6	1076.34399	1188.40875	712.0833748	671.807744	674.0886223	1063.715469
CHGB	0	0	0	0	0	0
PEBP1	4782.99139	4833.87797	6980.422942	8448.88751	6882.958712	6830.792946
TBX5	0	0	0	0	4.030425245	0
BRAP	921.045719	849.153601	777.2741063	645.659538	1010.62913	841.730554
ERP29	3362.84243	3952.22089	3913.449758	4637.28369	3273.712905	3588.00107
NOS1	0	1.01573397	0	0	2.015212623	0
FUS	11340.6806	10279.2278	9140.74349	8911.50961	8902.20176	11113.74271
IGBP1	875.139941	859.310941	1692.95315	1783.10648	758.7275524	700.3850573
FXYD5	2348.03172	2680.52195	925.7083872	1264.16517	137.0344583	992.1366594
ZNF302	681.358954	709.998047	1231.703652	823.628249	434.2783202	935.0095212
GRAMD1A	1267.78085	1164.03113	1304.817564	1243.04547	1239.355763	1205.967026
FXYD3	0	0	5969.465136	8832.05929	3681.793461	6114.098792
HEPH	0	0	0	0	0	4.530304381
CDIP1	545.985744	465.206159	575.6843058	569.226322	35.2662209	531.8577343
CMTM1	19.5343737	13.2045416	69.20246882	27.1539058	13.09888205	61.61213958
KCNH4	0	0	0	0	0	0

GANAB	16504.5923	16760.6263	7639.350797	8860.2189	10223.17363	11416.36704
GMIP	465.894812	512.945656	456.3351205	621.522733	820.1915374	864.3820759
RBM41	580.170898	549.512079	663.9425269	586.323226	468.5369347	530.0456126
BIRC5	3822.87693	3536.78569	549.6080132	1377.8093	2826.335703	1853.800553
LAG3	8.79046815	12.1888077	8.023474646	30.1710065	7.053244179	4.530304381
MLF2	2982.89886	3022.8243	3406.967921	3337.91902	4053.60019	3300.779772
OTUB2	791.142134	1240.21118	253.7423857	105.598523	293.2134366	379.6395071
DDX24	6442.43644	6341.22719	4716.800157	4327.52803	6348.927367	5892.113878
ZBTB25	474.68528	430.671204	1477.322269	1316.46158	565.2671406	1288.418566
NECAP1	513.754028	626.707861	902.6408976	757.292262	1143.633163	814.5487277
ARHGAP4	21.487811	18.2832115	199.473609	181.036096	310.3427439	94.23033112
ANKRD24	15.6274989	34.5349551	136.399069	132.752428	27.2053704	106.9151834
DHX32	1245.31632	1146.76365	1308.829302	1608.11464	1239.355763	1045.594251
RCOR1	3032.71151	2862.33833	3185.319434	3322.83351	3026.849359	4590.104399
GPATCH2L	1887.0205	1830.35262	1949.704339	1625.21155	1608.139673	1774.973256
LTBP4	3295.44884	2970.00613	441.2911055	855.850883	789.9633481	638.7729177
BLVRB	846.815099	935.490988	8768.654853	6170.97652	1954.756244	4224.055805
SLC9A1	812.629945	985.261953	2607.62926	3024.14055	2465.612644	3565.349548
SPTLC1	1527.53919	1445.3285	683.9811549	712.035753	1959.119179	605.2486653
PAPOLA	8303.08553	8907.98694	8699.452385	8524.31503	11747.68198	10361.71218
CCNK	2022.78439	2075.14451	2093.123948	2036.54294	3150.784935	2411.027992
PCBP4	1066.5768	962.915806	1448.237174	1023.80282	314.3731691	1155.227617
RGS1	0	0	0	0	0	0
YPEL3	237.34264	272.216705	1133.315794	2509.22204	966.2944525	1262.142801
MRPS33	669.052298	648.038274	744.1772734	504.861508	845.3816952	449.4061946
NDUFB2	1160.3418	1191.45595	1143.345137	1295.34188	1765.326257	1110.830634
NUDC	3610.92897	3689.14579	3689.795403	2282.93949	2941.202823	4103.549708
MAEA	1971.0183	1749.0939	1314.846908	1180.69205	1715.953548	1702.488386
ICAM1	3979.15192	4324.99525	334.9800665	383.171782	441.3315643	596.1880565
STRN4	4818.15327	4552.51966	3313.695029	2474.02253	3404.701726	3365.110094
IRAK3	146.507803	143.21849	7.020540315	3.01710065	12.09127574	12.68485227
LYZ	0	2.03146794	6.017605984	1.00570022	4.030425245	7.248487009
SI	0	0	0	0	0	0.906060876

MUL1	821.420413	930.412319	767.244763	735.166858	564.2595343	1017.506364
TFAP4	642.680894	564.748089	428.2529592	440.496694	651.9212834	543.6365257
PDCD7	769.654323	980.183283	1222.576949	1244.05117	997.5302482	1350.030706
SPG21	1809.85972	2214.30006	5170.126475	5015.42697	2826.335703	3796.395071
FETUB	0	0	0	0	0	0.906060876
DNAJB11	2478.91202	2773.96948	3294.639276	2519.27904	2538.160298	2248.843095
P3H2	2810.01965	1608.92261	0	0	74.56286704	31.71213067
THPO	0	0	0	2.01140043	1.007606311	0.906060876
CHRD	3.90687474	10.1573397	7.020540315	24.1368052	45.34228401	24.46364366
FLT3LG	163.365967	152.786704	80.64594953	160.881863	17.12930729	110.2313662
RAB11FIP3	1100.76196	1063.47347	1568.589293	1617.16595	1750.212163	2056.758189
GNPTG	490.312779	487.552307	912.6702409	1135.43554	498.7651241	974.9215028
ZNF268	553.799494	604.361713	946.7700082	893.061791	911.8837117	934.1487633
GOLGA3	3691.50827	3897.37125	4894.891206	3881.97266	5003.772942	5049.441021
PABPC4	4822.06014	5016.71009	2794.175045	3894.07123	6285.44817	4639.937747
CD209	0	1.01573397	0	0	0	0
CERS4	257.853733	272.216705	33.09683291	52.2964112	331.5024764	225.6091582
MCOLN1	599.29505	688.494958	487.406026	1158.48619	222.6809948	695.7550862
USP48	1924.13581	1547.97857	2534.415054	2180.35807	1493.272553	2230.721877
EFNB1	182.646394	207.20973	300.8802992	220.248347	299.2590745	202.0515754
PDPR	2095.18855	1831.20583	1734.334221	1922.99938	3127.539458	2979.381858
AARS	3350.14509	2693.72649	15058.05604	7875.63839	8022.56145	5551.434988
GLG1	4671.64546	4905.99509	2186.396841	2826.01761	6791.266538	3568.973791
KIF4A	2593.18811	2528.16186	786.2704272	1927.91726	1865.079282	1764.100526
TNRC6A	1939.76331	1966.46097	2477.247797	2150.18706	3655.595697	2793.385681
PLEKHG2	964.021341	1057.37907	46.13497921	95.5415205	977.3781219	270.912202
DLL3	0.97671868	0	0	0	1.007606311	0.906060876
NAT14	676.866048	622.644925	614.7987447	519.947011	229.734239	491.9910558
PITPNM2	1026.53134	867.436812	15.04401496	51.290711	348.6317837	139.5333749
EXOC1	780.398228	769.926351	769.2506317	802.548772	911.8837117	705.8214225
RBM27	1910.45198	1840.66232	1333.822425	1297.39351	1692.768527	1815.175178
POU4F3	0	0	0	0	4.030425245	2.718182629
OSBPL8	5369.99932	4874.50733	945.7670739	1404.9632	2920.04309	1388.991323

DTX2	1311.30344	1201.5625	563.6089765	608.428516	527.7237295	717.6092745
NLRC4	0	0	0	0	0	0
PUS7	991.369464	973.073145	211.6191438	169.963336	1635.345043	566.2880476
LAMB4	0	0	0	0	0	0
NRCAM	36.1385913	28.4405512	0	0	5554.933594	402.291029
LAMB1	6036.12147	5238.14009	22.06455528	87.4959188	1815.706573	248.2606801
SLC26A4	15.6274989	14.2202756	1.002934331	3.01710065	4.030425245	6.342426133
SLC26A3	0	0	3.008802992	0	0	11.77879139
DLD	2261.10375	2100.53785	1494.372153	1114.31584	4098.942474	1900.009657
WDR7	430.73294	473.332031	723.1156524	906.135894	571.393387	931.4305807
TXNL1	1583.26099	1481.95587	2176.367498	2382.50381	1420.724899	1855.612674
IL5RA	0	0	0	0	0	0
ABCC6	24.7402843	23.3720387	1133.857378	850.822382	3.022818934	729.5239751
CMTM6	3521.07086	4004.02332	1018.98128	1350.65539	2352.760737	1612.78836
ITGA6	12429.722	12012.07	4372.793682	3002.01514	460.4760843	3716.661714
RAPGEF4	15.6274989	19.2989455	70.20540315	92.5244198	20.15212623	30.80606979
AC013461.1	4904.10451	3809.0024	465.3615294	975.529209	2068.615757	867.1002585
SMPX	1.95343737	0	0	0	0	0
FH	2529.70139	2265.08676	4703.762011	3544.08756	1721.999186	5083.001515
SEL1L3	217.808267	257.996429	465.3615294	328.86397	554.1834712	250.9788627
TF	0	0	2.005868661	6.03420129	0	2.718182629
CDV3	6536.20143	5696.23612	5218.267323	4377.81304	5630.504067	5420.962222
MYO15A	9.76718684	7.11013781	7.020540315	34.1938073	4.030425245	4.530304381
ALKBH5	3612.88241	3881.11951	4732.847107	4166.61599	3688.846706	4934.407532
APOH	0.97671868	0	0	0	0	0
NLRP1	1269.73429	931.428053	0	0	38.28903983	11.77879139
PITPNM3	1034.34509	698.824973	157.4606899	57.3249123	128.9736078	130.4727662
SPAG7	788.211978	689.683367	249.7306483	423.399791	709.3548431	281.7849325
ORC6	834.117756	775.005021	635.8603657	630.574035	1155.724439	1371.776167
ZFHX4	387.757317	370.7429	12.03521197	5.02850108	25.19015778	36.24243505
SLC17A6	0	0	0	0	0	0
CPA1	0	0	0	0	0	0
ZC3HC1	430.73294	364.648496	304.8920365	336.909572	433.2707139	380.545568

ESR1	2.93015605	1.01573397	0	2.01140043	8159.595909	216.5485494
RGS17	56.6496837	89.3845896	0	0	187.4147739	26.27576541
ANGPT2	0	0	0	0	0	1.812121752
TMEM101	676.866048	640.928136	0	1.00570022	444.3543833	60.7060787
CD200	0	1.01573397	2.005868661	3.01710065	0	5.436365257
CCDC80	2356.82218	1979.66551	0	1.00570022	121.9203637	53.45759169
CMA1	0	0	0	0	0	0
PSME1	3440.0032	3340.74903	1904.572294	2143.14716	2446.468124	2990.906952
PPP2R3C	891.744158	816.650114	406.1884039	461.616399	463.4989032	500.1456037
HAUS4	1224.08246	1020.81264	1289.593021	1636.87767	1007.243573	906.4414217
JPH4	0	7.11013781	0	0	0	0
MYH7	0	0	0	0	1.007606311	0
CEBPE	0.97671868	0	0	0	0	0.906060876
SLC7A8	3.90687474	5.07866986	2159.317614	6093.53761	38.28903983	1316.506453
OSGEP	1206.24757	1165.04687	685.0041479	527.992613	514.8868251	643.3032221
SLC22A17	0	0	524.534655	686.893247	169.2778603	600.7183609
RNF31	932.912851	995.246618	1289.91396	1067.2893	1505.001091	1803.00678
SCFD1	2026.69127	1986.77565	2228.520083	1655.38255	1335.078362	1685.27323
G2E3	1156.43492	1165.04687	634.8574313	1140.46404	974.355303	956.8002852
HECTD1	6159.18802	6122.84438	5566.285535	5993.97328	7446.21064	6789.114145
HNRNPC	15609.205	14378.7606	13196.00816	10993.9426	15277.85085	13449.98443
RPGRIP1	14.6507803	31.4877531	23.06748961	73.4161157	22.16733885	10.87273051
SUPT16H	9663.43001	8576.71546	2826.188709	3636.47118	8474.664326	6079.478206
TOX4	1872.36972	1913.6428	1833.353927	1884.6822	2347.722705	2157.321886
GEMIN2	349.665289	341.286615	464.3585951	303.721465	392.9664614	420.4122465
TGM1	34.1851539	24.3776153	82.24061512	17.0969037	110.8366942	19.0272784
TINF2	878.070097	1022.84411	830.4296258	1031.84842	783.9177102	829.0457017
DAZL	0	0	0	0	0	0
TBL1Y	0	0	0	0	0	0
SEMA6A	4.88359342	9.14160575	36.10563591	29.1653063	169.2778603	121.4121574
TRPM7	1559.81974	1602.82821	3125.143374	3301.71381	4564.45659	3270.879763
TYRO3	842.88869	964.947274	291.8538902	490.781705	881.6555224	635.1486742
WDR76	1183.78304	1033.00145	561.6432252	871.942087	646.8832518	1177.879139

CAPN3	2.36365921	2.03146794	26.18661537	58.7328926	0	26.04925019
SNAP23	1046.06571	1100.03989	2482.262469	1785.11788	582.3964479	2167.297616
TBX15	4.88359342	2.03146794	0	1.00570022	2.015212623	0
PHGDH	30.2782792	34.5349551	19575.27227	4480.39446	1709.90791	3857.10115
COL9A3	5.8603121	8.12587178	147.4313466	107.609923	21.15973254	80.63941798
EZR	9137.20329	7342.74089	9385.459467	9871.95332	4925.17965	9341.487633
MYL6	10972.4577	9613.92205	11562.8299	13534.7135	17025.52384	11520.56404
AGO1	1384.01038	1334.67444	1923.628046	1901.77911	2256.030531	2788.855377
TEKT2	0	3.04720192	1.002934331	0	1.007606311	1.812121752
CLSPN	1945.62362	1931.92602	223.6543557	409.319988	1449.945482	880.6911716
RFFL	1171.0857	1088.86682	1651.832843	1738.85567	634.7919761	1813.027813
UNC13D	7043.11843	6525.07504	22.06455528	68.3876147	4183.581404	592.563813
MFSD11	300.829355	317.924733	636.8633	949.381003	648.8984645	936.866946
DPYSL2	2130.22345	2116.7896	219.6426184	1430.10571	972.3400904	349.7394982
TGFB2	6505.92315	3654.33658	8.023474646	12.0684026	543.0998018	91.73866371
GPATCH2	448.313876	410.356525	1255.673782	964.466507	1258.500283	1104.488208
NUP50	3200.29691	2957.64465	3281.169868	3349.27337	2589.276166	4668.542089
CDC45	1104.66883	1057.37907	479.4026101	869.930686	1326.009906	1400.770115
COMT	1513.91396	1365.14646	6206.157638	8173.32565	2411.201903	5238.843986
CECR1	0	0	1.002934331	23.131105	34.25861458	4.530304381
XXbac-B461K10.4	133.195127	59.5931122	513.171409	125.008537	23.19509729	155.0270159
VNN3	2.93015605	4.06293589	0	0	2.015212623	0
ECHDC1	1461.17115	1602.82821	430.2588279	509.890009	902.8152549	522.7971256
LRRFIP2	1881.16019	1737.92083	954.7934828	901.107393	968.3096651	970.3911984
SEC22C	2450.58718	2322.98359	2709.928562	3214.21789	754.6971272	2806.976594
XYLB	315.480135	332.145009	331.9712635	248.407953	239.8103021	416.788003
HDAC6	1787.39519	1744.01523	1679.915004	2239.69438	654.9441023	845.3547975
OR1I1	0	0	0	0	0	0
GABRP	0	0	0	0	9.068456802	0.906060876
KRT31	0	0	0	0	0	0
CDC6	3376.51649	3106.11449	420.2294846	577.271924	1593.025578	911.4972414
UPRT	229.528891	325.034871	541.5845386	586.323226	170.2854666	338.8667677
CDC23	1834.27769	1834.41555	694.0305569	751.258061	1663.55802	1053.748799

AAAS	807.746352	801.414104	961.8140231	879.987689	1173.861353	846.2608584
CBX5	4710.71421	3508.34514	8393.557414	12247.4172	14099.43511	12690.06212
FMO2	0	0	0	0	0	0
SUCO	2781.69481	2366.66016	1746.10867	2885.35392	2463.597431	2450.89467
MSH2	3099.12838	2692.71076	1140.336334	1298.35898	2276.182657	1917.224814
MAP3K1	1695.58364	1196.53462	1169.42143	2223.60318	1058.994233	1814.839935
DHPS	1744.41957	1626.19009	2722.966708	2805.9036	1114.41258	1866.485405
HOOK2	435.616533	437.781342	3227.442676	4427.09235	1478.158459	2808.788716
NXPE1	0	0	0	0	0	0
ARCN1	9104.97157	10071.0023	5788.936957	5078.78609	6748.947073	4710.610495
EPB41L4B	873.186503	648.038274	603.7664671	512.90711	1087.20721	528.2334908
TMEM38B	504.96356	661.242816	198.5809975	204.157144	1095.26806	178.4939926
PSMD5	1913.3919	1779.56592	3447.085295	3076.43696	2479.719132	3279.034311
PTGS1	73.2539013	72.117112	5.014671654	1.00570022	23.17494516	10.87273051
NUP188	5625.89962	5496.13652	1610.712535	1994.30353	5146.853038	2967.349369
CRAT	1197.45711	1233.10104	8635.264587	9323.8467	143.0800962	11508.78525
SH2D3C	1.95343737	4.06293589	0	7.03990151	2.015212623	7.248487009
NANS	1858.69566	2142.18295	2092.121014	1398.929	2656.050237	1088.179112
TBC1D2	1934.87971	1442.34224	245.718911	271.539058	523.9552819	295.3758456
DFNB31	187.529987	213.304134	106.3110391	95.5415205	304.297106	231.9515843
PDE6C	0	0	1.002934331	0	0	0
CWF19L1	863.419317	676.478826	647.8955776	627.556935	915.914137	794.6153884
SEMA4G	123.066554	119.856609	0	0	58.44116605	17.21515665
BTAF1	2619.55951	2434.71433	1235.615095	1025.81422	1856.010825	1408.018602
IKZF5	617.286208	608.424649	474.3879384	457.593598	408.0805561	566.2880476
BLNK	0	0	564.6520282	973.517809	15.11409467	303.5303935
TLL2	8.79046815	8.12587178	0	6.03420129	3.022818934	9.060608762
CYP26A1	0.97671868	3.04720192	0	6.03420129	41.31185876	38.96061768
TDRD1	0	0	0	0	0	0
SORBS1	38.0920287	66.0227082	27.07922693	11.0627024	27.2053704	61.61213958
CRTAC1	0	0	0	0	0	0
BAMBI	131.857022	150.328628	912.6702409	1197.78896	3383.541993	1191.470052
IL11	3968.40801	3773.45171	27.07922693	26.1482056	98.74541851	42.58486118

MYO3A	0	0	0	0	0	0
WAC	5000.79966	4617.52664	4104.007281	4268.19171	4620.882544	4803.934765
CREM	335.014509	360.58556	199.5839318	258.464955	470.5521474	236.4818887
NUBP2	1013.83399	958.85287	2233.534754	1835.40289	1764.318651	2193.573381
TPSD1	0	0	0	0	0	0
SMIM24	0.97671868	0	0	0	0	0
HIVEP1	1204.11833	1309.28109	1408.1198	2082.80515	916.9217433	1901.821779
TREM2	0	0	0	0	0	0
KCNK16	0	0	0	0	0	0
CRISP3	0	0	91.26702409	53.3021114	1.007606311	104.1970008
FKBP5	3302.28587	3501.235	1492.366284	1760.98108	1321.97948	3107.788805
SRPK1	12106.4281	11121.2713	2080.085802	1969.16102	2726.582678	3281.752494
BRPF3	5545.80869	6363.57334	1526.466051	2155.21556	2373.920469	2543.312879
MRPS18A	1006.02024	1017.76544	852.4941811	660.745042	814.1458995	739.345675
PGC	0	0	0	0	2.015212623	11.77879139
TMEM14A	547.939182	785.162361	533.5610639	631.579735	513.8792188	630.6183698
EFHC1	426.826065	386.994643	643.8838403	621.522733	274.0689167	609.7789697
NCR2	0	0	0	0	0	0
HSP90AB1	50418.5505	50497.8747	21858.68295	24944.2316	49093.97512	46495.02126
MLN	0	0	0	0	0	0
CDC5L	4272.16752	4265.06695	1546.524738	1232.98846	2169.376388	1440.636793
ITPR3	16848.3973	17336.5474	4043.831221	5362.39355	4530.197976	4480.471033
ZNF184	247.109827	283.389778	502.4700997	399.262986	158.1941909	450.3122555
DSP	7025.53749	5678.96864	1755.135079	2668.12267	19441.76378	2114.746085
SIRT1	1084.15774	1132.54338	495.4495594	915.187196	1023.728012	740.2517358
HNRNPH3	4147.14753	3894.32405	2812.227863	2432.78882	4622.897756	2835.064482
IFT74	496.173091	523.102996	693.0276225	557.157919	317.3959881	452.1243772
JAK2	164.088739	143.21849	191.5604572	287.630262	723.4613315	212.018245
IL12RB1	0.97671868	0	0	0	0	0
ABL1	3188.00978	2789.20549	1774.190831	1806.23759	2809.206396	2687.376559
ACOT7	1795.20894	2031.46794	1277.738337	1560.84673	1154.716833	1183.315504
SH3GLB1	2739.69591	3010.63549	2153.300008	2138.11866	908.8608928	1698.864143
CDC7	1011.88056	1152.85806	513.5023773	714.047153	598.5181489	957.7063461

SYDE2	256.877014	241.744685	21.06162094	20.1140043	913.8989243	82.45153973
PCSK5	0	0	18.05281795	37.210908	5.038031556	20.83940015
SCD	13169.098	17452.3411	15432.15055	165095.697	13169.41449	2831.440238
TMED1	780.398228	681.557495	327.9595261	531.009714	553.1758649	390.5122376
ABLIM1	2849.0884	3026.88724	3072.990789	3689.91409	1072.093115	4102.643647
ERMP1	353.572164	435.749874	6053.71162	8263.83867	5765.523313	6134.938193
RAB18	1609.63239	1677.99252	1900.560557	1768.02098	2073.653789	1558.424707
NRP1	9241.71219	9279.74557	6.017605984	2.01140043	4618.867331	312.5910023
HSD17B7P2	16.2819005	29.3445545	27.16949102	208.270458	40.87858805	33.65110094
PRTFDC1	533.288401	430.671204	0	0	223.6886011	33.52425242
PALMD	0	0	157.4606899	263.493456	30.22818934	292.657663
TSPAN15	479.568874	594.204374	3484.193865	3106.60797	2748.750017	3935.022385
H2AFY2	0	0	142.416675	98.5586211	750.6667019	262.7576541
FAM21A	1853.96834	2377.40662	1316.371368	2516.69439	1319.792975	1441.769369
MAST3	432.686377	502.788316	662.9395926	1077.10493	501.787943	1311.070088
MZF1	618.262927	743.517268	1081.163209	1088.16763	530.0009197	773.7759883
OCEL1	377.990131	367.695698	355.0387531	341.938073	155.1713719	300.8122109
MYO9B	4253.60987	4258.97255	1983.804106	2770.70409	2136.12538	3300.779772
KCNK6	209.994517	246.823355	98.28756441	109.621323	1334.070756	376.9213245
CATSPERG	5.8603121	21.3304134	54.15845386	119.678326	45.34228401	71.57880922
PSMD8	4894.33732	5176.18032	4491.139933	3500.84245	4329.68432	3840.792054
FBXL19	841.931505	978.151815	1828.349285	1586.99494	2540.175511	1959.809675
STX1B	15.6274989	24.3776153	3.008802992	15.0855032	31.23579565	19.0272784
HSD3B7	434.639814	496.693912	606.7752701	599.397328	968.3096651	691.3244485
SETD1A	1510.9838	1462.65692	1203.521197	1762.99248	2178.444845	2345.791608
BCL7C	1021.64774	926.349383	993.9079217	899.095993	983.4237598	1090.897295
MAGEB2	2.93015605	0	0	0	0	0
EFNA2	2.93015605	1.01573397	0	1.00570022	19.14451991	0.906060876
CIRBP	2398.82109	1980.68125	5774.895876	3502.85385	2594.586252	4504.028615
ATP5D	1661.39848	1736.90509	1949.704339	1385.8549	2551.25918	1748.697491
CBARP	167.995614	141.187022	96.28169575	57.3249123	160.2094035	153.1242881
PCDH11Y	0	0	1.313843973	1.17666925	0	0
AMELY	0	0	0	0	0	0

PRKY	0	0	0	0	0	0
IGFALS	0	0	32.09389858	15.0855032	8.06085049	12.68485227
HNRNPM	8736.74863	8494.58321	5411.833649	6093.53761	8449.786526	7966.087223
MARCH2	262.737326	281.35831	442.2940398	580.289024	186.4071676	417.6940639
NDUFB7	1026.53134	1040.11159	1386.055245	1125.37854	1360.26852	1053.748799
TECR	3142.05517	3039.86832	3688.130532	5622.4274	2225.772113	3870.510851
TIMM13	1706.1322	1640.71509	1986.903173	1647.12576	455.8713234	1907.665872
CDC34	1581.30755	1506.33348	1372.014164	1138.45264	1198.043904	1289.324627
MTAP	0	0	533.0896848	505.867208	21.03881978	446.688012
MISP	128.926866	60.9440383	17.04988362	73.4161157	3252.553173	322.5576719
CEP170B	2297.24234	2366.66016	4781.990889	4511.57117	5776.606983	6573.471657
POLR2E	4267.28393	4038.55827	3623.601737	2953.74153	2610.707953	3544.510148
POLRMT	1595.04998	1803.93338	1535.612812	1227.55768	1629.15834	2039.533972
HCN2	8.79046815	2.03146794	163.3479144	132.742371	1344.136743	399.7268767
CDHR5	0	0	0	1.00570022	1.007606311	0
IZUMO4	39.0687474	62.9755063	16.04694929	53.3021114	11.08366942	28.99394804
RASSF7	995.276339	802.429838	1239.626833	1253.10247	666.0277718	1367.245862
GADD45B	819.466976	510.914188	403.1796009	320.818369	553.1758649	329.8061589
PALM	259.80717	190.957987	20.05868661	3.01710065	837.3208447	178.4939926
MADCAM1	2.93015605	3.04720192	36.10563591	39.2223084	9.068456802	27.18182629
IGF2-AS	19.5343737	10.1573397	0	0	0	0
MKNK2	2056.96955	2126.94694	10470.63441	8079.79553	1331.047937	7615.441664
ARVCF	152.368115	176.737711	2558.485478	2213.54617	440.323958	1949.843006
TRMT2A	977.695403	951.742732	1552.542344	1255.11387	980.4009409	1462.382254
RANBP1	3810.09168	3431.03763	3086.931576	2650.97548	4879.686225	5179.750696
ZDHHC8	1465.07803	1374.28806	1359.978952	1452.23111	487.6814547	1733.294456
KLHL22	443.430282	443.875746	1596.420721	1271.20507	701.2939927	1427.625759
MED15	2587.32779	2386.97483	2981.723765	2711.36778	2112.950435	3598.8738
SERPIND1	0.97671868	1.01573397	1.002934331	0	0	0.906060876
SNAP29	1025.55462	1117.30737	2946.621064	3113.64787	1449.945482	3294.437346
CRKL	2747.50966	2546.44507	3853.273699	3675.83429	3337.192103	4714.234739
LZTR1	1383.03366	1415.93316	2115.188503	2071.74244	680.1342601	2190.855199
MMP11	126.973429	140.171288	1.002934331	0	21.15973254	3.624243505

CECR2	19.5343737	11.1730737	1.002934331	0	13.09888205	6.342426133
SMARCB1	2084.31767	2117.80533	3292.633408	3094.53956	2692.324064	3033.491813
P2RX6	98.6485871	112.746471	4.011737323	0	2.015212623	0.906060876
DERL3	13.6740616	11.1730737	3.008802992	102.581422	0	29.90000891
SLC7A4	6.83703079	2.03146794	0	0	0	0
BCL2L13	995.276339	1030.96998	2227.517149	2121.02175	1536.599625	2121.088511
DDTL	19.5929768	33.6106371	1.193491854	4.74690502	13.0283496	5.372940996
DDT	1177.86413	1133.4677	1796.064829	1618.08113	1341.194533	1515.90327
GSTT2	2.83248418	0	2.808216126	8.23668477	0	4.412516467
OSM	0	1.01573397	0	0	0	1.812121752
CABIN1	1543.21552	1694.24427	4196.27724	5922.56857	2065.592938	5590.395606
TBC1D10A	443.430282	505.784731	1112.75564	1598.38952	804.5736396	1285.147686
SUSD2	60.5565584	16.2517436	2126.220781	2957.76433	172.3006792	1826.618726
SF3A1	3672.46225	3760.24717	3801.121113	3308.75371	3664.664154	4122.576987
GGT5	1238.47929	926.329068	1.002934331	0	4.030425245	0
RNF215	288.132012	313.861797	517.5141146	538.049615	219.6581759	537.2940996
SEC14L2	397.797986	437.649297	456.0242108	271.539058	165.2474351	999.3851464
SEC14L3	0	0	0	0	0	0
SPECC1L	2223.01172	1824.25821	4178.735918	3676.28685	2375.663628	4128.647594
PPIL2	845.83838	830.870389	2708.925627	2346.2986	1736.105674	2266.964312
UPB1	5.8603121	3.04720192	5.014671654	15.0855032	1.007606311	4.530304381
YPEL1	51.7660902	29.4562852	287.8421529	182.031739	21.15973254	160.3727751
SNRPD3	2156.59485	2206.20466	3801.121113	3293.76878	2908.959421	3895.590616
PES1	2386.12374	2411.35245	2825.26601	2465.97693	3518.561239	3418.567686
MAPK1	4403.04783	4239.6736	5719.734488	5638.96111	3822.858345	5908.422974
GGT1	183.623113	255.934489	2972.887914	1171.17813	21.15973254	2738.206574
PRODH	2.93015605	1.01573397	13207.4717	9732.28167	12.65553527	3359.157274
PPM1F	2949.69043	2824.75618	931.7259932	827.691277	1048.91817	1125.327608
SLC35E4	271.527794	349.412486	226.6631587	237.345251	134.0116394	201.1455145
TOP3B	612.402615	557.637951	1904.070827	2346.2986	764.1081701	1614.8723
CRYBB3	1.95343737	0	7.020540315	12.0684026	6.045637868	13.59091314
CYTH4	53.7195276	103.604865	0	0	5.038031556	3.624243505
DGCR14	388.734036	480.442169	1214.553474	1050.95673	585.4192669	1094.521538

CRYBB2P1	266.644201	225.492942	583.7077805	447.496368	191.4451991	480.9642949
MFNG	39.0687474	27.4248173	0	0	7.053244179	0.906060876
CARD10	1660.42176	1415.93316	25.07335827	55.3135119	479.6206042	154.9364098
LRP5L	142.600928	105.636333	483.4143474	547.100917	109.8290879	610.6850305
SLC25A1	1788.37191	2122.884	5094.9064	11835.0801	3738.219415	5794.259303
ADRBK2	274.45795	303.704458	747.1860764	851.828083	422.1870444	577.1607781
PLA2G3	0	0	2.005868661	0	1.007606311	0
LGALS2	0	0	0	0	0	0
GGA1	926.906031	909.081905	3675.754322	2854.17721	1200.059117	3514.610139
HIRA	1209.08006	1274.74614	1848.508265	1698.20527	1505.051471	2467.049735
SH3BP1	532.77074	703.233258	700.7803049	761.063638	623.8594476	1446.344977
SEZ6L	0	0	0	0	0	1.812121752
LGALS1	9511.28654	9383.35044	3109.096425	4749.92212	1739.128493	3659.579879
HPS4	1032.39165	959.868604	1827.346351	2030.50874	847.3969078	1776.785378
PIK3IP1	37.11531	70.0856441	274.8040066	1027.82562	70.53244179	287.2212977
RP1-37E16.12	2.88132012	0	0	11.5353815	1.712930729	0
SRRD	688.586672	700.856441	974.8521694	903.118794	516.9020377	1100.863965
PATZ1	865.372754	716.09245	4273.503183	6124.71431	2019.243048	5343.947048
TRIOBP	1693.0637	1708.46454	2906.50369	2754.47209	1207.606088	2323.140087
TFIP11	988.439308	971.041677	1397.087523	1566.88094	1140.610344	1821.182361
GCAT	207.064361	229.555878	1481.334006	1166.61225	229.734239	1543.021672
GGTLC2	0	0	0	0	0	0
CRYBB1	0	0	0	0	0	0
ANKRD54	424.872627	428.639736	1892.537082	1625.21155	670.8642821	1796.329111
EIF3L	5158.42252	4776.54995	11034.34368	8890.54076	3734.984999	7351.651101
SNU13	2556.0728	2574.88562	3161.24901	2668.12267	1960.801882	3511.891956
MICALL1	1817.18511	1802.9278	1960.164944	2154.20986	1028.766044	2411.934052
POLR2F	640.54188	697.636564	1006.755511	973.04513	685.8070837	919.5974257
SOX10	0	0	0	0	0	0
CCDC134	116.229523	103.604865	160.4694929	251.425054	106.806269	251.8849236
DEPDC5	247.109827	250.886291	550.6109476	806.571573	451.4076275	761.091136
PICK1	202.180768	234.634548	1313.843973	1105.26454	396.9968866	1113.548817
TTC28	634.867145	571.858226	708.0716375	706.001551	377.8523667	712.1638487

SLC16A8	3.90687474	0	14.04108063	8.04560172	2.015212623	20.83940015
CENPM	563.566681	515.992858	445.3028428	783.440468	428.2326823	743.8759793
SEPT3	24.4179671	34.5349551	988.8932501	962.455106	157.1865846	895.1881457
SLC5A1	0	0	0	0	0	0
TPTEP1	0	0	0	0	3.022818934	0
SLC5A4	0	0	0	1.00570022	2.015212623	0
KDELR3	508.870434	580.999832	100.2934331	76.4332164	288.175405	135.9091314
CYP2D6	4.3073294	5.96235842	25.69517755	40.2280086	11.80914597	29.18422082
DDX17	5370.97604	5803.90392	33834.99258	22523.662	9569.237138	22321.71575
DMC1	2.93015605	6.09440383	12.03521197	17.0969037	79.60089859	26.27576541
TCF20	2294.31219	2161.48189	2465.212585	3224.27489	1680.687327	3265.443398
HSCB	95.718431	110.715003	297.8714962	383.171782	399.0120993	339.7728286
CBY1	327.200759	304.720192	1032.019426	423.399791	315.3807754	747.5002228
TOMM22	1446.52037	1418.98036	1381.040573	1624.20585	1679.679721	2072.161224
RSPH14	0	0	1.002934331	4.02280086	0	1.812121752
XBP1	1826.46394	21801.9121	1721.025546	15983.3045	12028.64519	12693.78238
RTCB	2073.57377	2149.29309	2685.858138	2910.49642	2681.240394	2983.658465
JOSD1	2255.24344	2351.42415	2232.53182	2573.58685	1252.454645	2363.006765
FBXO7	1692.65348	1635.3317	2005.868661	2289.97939	2113.958041	1655.373221
GTPBP1	1565.68005	1439.29504	2918.538902	2360.37841	816.1611121	2556.903793
POLDIP3	2103.85205	1992.87005	2972.697356	2763.66419	1591.010366	3070.640309
RAB36	198.273893	246.823355	10.02934331	194.100142	219.6581759	60.7060787
TIMP3	19345.867	14453.8944	1.002934331	8.04560172	919.9445622	404.1031508
PPP6R2	1022.62446	956.821402	3438.058886	2885.35392	1229.2797	2849.561456
SBF1	4197.9369	4200.05998	5900.262668	5512.24288	3060.100367	5435.459196
SUN2	2565.83998	2195.00111	4435.978545	5397.59306	1154.716833	4328.252806
CYB5R3	4896.29076	4422.50572	3040.896891	3038.22035	2158.292719	3115.037292
DNAL4	125.019992	128.998214	671.9660016	488.770305	193.4604118	462.0910469
C22orf31	0	0	0	0	0	0
MIOX	1.95343737	0	5.014671654	1.00570022	0	2.718182629
LMF2	1971.99502	1990.83859	2486.274206	3531.01346	971.3324841	3205.64338
RHBDD3	262.737326	352.459688	1302.811696	816.628575	597.5105426	1192.376113
PACSIN2	2119.47954	2358.53428	7652.388943	8316.13508	1029.77365	6931.365703

TTLL1	34.1851539	37.582157	511.4965087	204.157144	49.37270925	260.9455323
RASL10A	4.88359342	4.06293589	14.04108063	60.3420129	18.1369136	45.30304381
AP1B1	2704.53404	2735.48332	11090.53809	10137.4582	7069.134131	13332.74016
HMGXB4	1205.27086	1033.00145	2616.655669	2503.18784	899.792436	2448.176487
TOM1	434.639814	665.305752	2608.632194	2191.42077	470.5521474	1615.506542
NEFH	0	0	0	0	0	1.812121752
CHKB	431.016188	366.883111	939.7494679	1118.35875	245.6241905	818.5353955
BIK	24.4179671	39.6136249	379.109177	505.867208	163.2322224	327.0879763
HMOX1	285.201856	621.629191	599.7547298	1242.03977	446.3695959	610.6850305
MCAT	395.571067	341.286615	640.8750373	699.96735	438.3087454	1009.351816
THOC5	749.143231	682.593544	937.7435992	937.312601	1233.310125	1233.148852
MCM5	3381.40008	2795.29989	1919.616309	2811.9378	3132.648022	4673.461999
APOBEC3H	0	3.04720192	0	0	16.12170098	9.966669638
ARSA	205.110924	209.241198	1126.295253	1019.78002	146.1029151	774.6820491
TSPO	1839.16128	1687.13413	2754.057672	2097.89065	448.3848085	1842.021761
RASD2	37.11531	45.7080288	6.017605984	8.04560172	12.09127574	8.154547886
TTLL12	2139.01392	2092.41198	3603.54305	4307.41402	2876.716019	7297.414297
CBX7	316.456854	296.59432	551.6138819	583.306125	353.6698153	507.3940907
PDGFB	87.9046815	80.2429838	482.4114131	531.009714	1121.465824	727.5668836
ACR	0	0	0	0	3.022818934	0
CABP7	0	1.01573397	4.011737323	4.02280086	0	0
RPL3	19733.6048	18933.8704	52470.22453	32424.3281	21851.7767	23137.43329
ZMAT5	181.669675	245.807621	523.5317206	354.006476	192.4528055	260.9455323
RBFOX2	4934.38279	5211.73101	1791.240715	1733.82717	1308.880598	2146.458216
SYNGR1	140.64749	92.4317915	710.0775061	542.072416	369.7915162	782.836597
TAB1	681.749641	607.408915	1453.251845	1426.08291	593.4801173	1564.767133
ASCC2	987.462589	967.994476	3060.955577	2752.60149	1958.786669	3260.913093
MTMR3	1310.75647	1143.71645	2356.895677	2497.15364	1489.242128	2371.161313
MIEF1	1631.1202	1718.62188	1698.970756	1806.23759	1288.728472	2951.040274
APOL4	0	0	0	0	0	1.812121752
PNPLA5	0	0	0	0	0	0
APOL1	503.010122	532.244601	3.008802992	6.03420129	20.15212623	115.0697313
PNPLA3	109.392493	171.679356	529.5994733	3093.9462	17.12930729	735.8392194

MYH9	54659.131	48017.8078	21435.71545	27087.5296	29272.97856	35484.96815
CACNA1I	13.6740616	28.4405512	35.10270157	45.2565097	163.2322224	68.86062659
SAMM50	1109.55242	1096.97238	2730.940036	2320.74376	1079.146359	2755.213337
TXN2	915.185407	1041.12732	2203.446725	2344.2872	1136.579919	2305.018869
FOXRED2	1684.83973	2345.32974	7744.658902	9189.08287	1600.078822	10861.85778
GRAP2	1.95343737	3.04720192	0	0	1.007606311	0
EIF3D	4912.89498	4427.58438	7725.603149	5492.12888	3833.942014	6212.859428
TNRC6B	1775.67457	2114.75813	2420.08054	3268.5257	1241.370976	3368.734338
SGSM3	895.651033	972.057411	5752.520411	6862.85804	545.1150144	4157.569058
IFT27	152.368115	202.13106	2147.282402	1495.47622	395.9892803	1392.615567
PVALB	0	0	4.011737323	17.0969037	1.007606311	0.906060876
KIAA0930	1741.48941	1942.08335	1768.173225	2360.37841	1427.778143	2717.276568
NCF4	0.97671868	1.01573397	3.008802992	4.02280086	1.007606311	8.154547886
CSF2RB	2.93015605	2.03146794	0	0	0	0
SLC25A17	613.379333	666.321486	1203.521197	1477.37362	602.5485742	1376.306471
UPK3A	0	0	0	0	30.22818934	81.54547886
FAM118A	440.500126	441.844278	1152.371546	1106.27024	614.6398499	1188.75187
KCTD17	272.504513	301.67299	202.5927348	288.635962	225.7038137	242.8243148
ST13	4132.47722	4438.71683	13990.53274	12324.9869	4604.74069	10959.59457
IL2RB	4.88359342	1.01573397	0	0	19.14451991	7.248487009
RBX1	881.000253	733.359928	1743.099867	1427.08861	653.936496	1216.839757
EP300	3789.66849	3921.74887	4893.3166	6941.34289	4127.155451	7815.681118
L3MBTL2	678.819485	729.296992	1837.375694	1430.10571	970.3248778	2136.491546
CHADL	2.93015605	8.12587178	35.10270157	23.131105	15.11409467	27.18182629
RANGAP1	7195.48654	6580.94041	4585.41576	6112.64591	3867.193023	9485.551313
ZC3H7B	4159.84487	3743.99542	6579.249209	6025.14999	3442.990766	7523.929516
PHF5A	802.862758	785.162361	1044.054638	1012.74012	1098.290879	1041.063947
ACO2	2336.31109	2563.71255	7368.558528	6546.1027	3265.652055	7885.447805
POLR3H	1180.85289	1160.98393	2575.535361	2433.79452	2315.479303	2909.361473
TRMU	1035.3218	1107.15003	1925.633915	1727.79297	647.8908582	2324.046147
PMM1	594.821678	574.905428	797.3327929	1006.70592	314.3731691	786.4608405
DESI1	1267.78085	1332.64297	1472.307597	1376.8036	822.20675	1909.976327
CERK	1500.2399	1542.8999	513.5023773	526.986913	731.522182	1020.224547

BRD1	962.067904	745.548736	2572.526558	2033.52584	1163.78529	2542.406819
ZBED4	1468.9849	1471.79853	2578.544164	2499.16504	979.3933346	2883.991769
MLC1	2.93015605	0	0	0	0	0.906060876
HDAC10	466.627351	483.540157	768.237668	765.368035	289.6465102	985.0512634
KCNK10	0	0	0	0	18.1369136	1.812121752
ABHD4	1349.82522	1488.05027	1148.359809	1117.33294	1147.663589	836.2941887
KHNYN	2283.56828	2739.43452	1900.560557	2703.32218	2162.323144	1408.018602
FKBP3	2004.22674	2102.56932	2813.230798	2067.71964	1588.995153	2440.928
SDR39U1	585.054492	629.755063	1042.04877	1177.67495	503.8031556	756.5608316
CTSG	0	0	0	0	0	0
GZMH	0	0	0	0	0	0
GZMB	0	0	0	0	0	0
RBM23	1726.83863	2009.1218	1317.855711	1988.26933	2111.942828	1663.527769
PRMT5	4726.34171	4307.72778	1553.545278	1340.59839	3889.360362	2649.322002
COCH	162.135302	117.825141	0	0	268.0232788	12.68485227
AP4S1	296.92248	214.319868	296.8685619	175.997538	238.8026958	265.4758367
POLE2	1055.8329	900.956033	164.4812302	316.795568	358.7078468	369.6728375
VCPKMT	187.529987	169.627573	255.7482543	198.122942	244.8483336	284.5031151
SOS2	1708.28098	1700.33867	2018.906808	1927.92731	900.8000423	1555.706524
CDKL1	269.574357	257.996429	2.005868661	3.01710065	28.21297672	11.77879139
NIN	6920.05188	7139.59409	1114.260041	1892.72781	1877.170558	2046.791519
PYGL	5720.64133	5341.74496	4.011737323	4.02280086	1444.90745	232.8576452
TRIM9	41.0221847	70.0856441	4.011737323	6.03420129	2.015212623	7.248487009
PSMC6	2878.38019	2844.04496	3447.055207	2735.49453	3300.898124	3090.383376
GNPNAT1	3305.21603	3127.4449	7114.816142	5992.96758	3487.325443	6775.523232
DDHD1	1366.42944	1552.04151	1589.650914	1438.15131	1571.865846	1486.845898
CDKN3	1388.89397	1514.45935	563.6490939	1230.97706	814.1458995	926.9002763
CNIH1	3223.17166	3508.34514	3074.996658	2687.23098	2315.479303	2525.191662
CGRRF1	450.267313	474.347765	314.9213798	340.932373	229.734239	302.6243326
ATP6V1D	1718.04816	1804.95927	2170.349892	1994.30353	903.8228612	1403.488297
C14orf105	0	0	0	0	0	0
PLEK2	516.684184	586.078502	6.017605984	2.01140043	109.8290879	51.64546994
PIGH	372.129819	370.7429	418.2236159	326.85257	230.7418453	417.6940639

LRRC74A	0	0	0	0	0	0
PSMA3	2982.89886	2641.92406	2932.579983	2356.35561	2937.172397	3038.022118
VTI1B	2614.67592	2435.66912	3048.920365	2104.15616	3517.553633	2691.807196
TIMM9	868.30291	886.735758	1047.063441	916.192896	739.5830325	839.0123713
GSTZ1	782.351666	907.050437	595.7429924	1207.84596	1087.20721	579.8789608
KIAA0586	1242.38617	1475.86146	1179.450773	1479.38502	500.7803367	1369.057984
TMED8	1475.82193	1361.08352	936.7406649	1240.02837	2928.103941	1628.191394
SAMD15	10.7439055	10.1573397	39.1144389	22.1254047	185.3995613	86.07578324
AHSA1	3410.70164	3758.2157	3683.777797	2938.65603	5615.067539	4704.268069
DAAM1	809.699789	908.066171	1604.694929	1779.08368	1555.744145	1668.964134
ISM2	5.8603121	5.07866986	0	0	12.09127574	0.906060876
SPTLC2	3826.7838	3409.81894	12785.40685	6882.00658	2632.875291	8035.853911
RIN3	1617.44614	1301.15522	0	0	383.8980046	40.77273943
LGMN	2066.73674	2538.3192	558.6344222	790.480369	1070.077903	948.6457374
ALKBH1	344.781695	327.066339	667.9542643	558.16362	538.0617702	747.5002228
SNW1	3018.06073	2951.72292	3681.771928	2927.59333	2494.833227	3019.9009
CHGA	0	0	0	0	1.007606311	0
ITPK1	4339.56111	2963.91173	5567.28847	3947.37335	4314.570225	5719.05625
DHRS7	535.241839	827.823187	2230.525951	2300.03639	788.9557417	1198.718539
PPM1A	2375.37984	2087.33331	3660.710307	4140.46779	1904.375928	3266.349459
SIX4	964.99806	721.17112	718.1009808	588.334626	974.355303	1079.118504
GALNT16	4.88359342	17.2674775	0	2.01140043	104.7910564	9.060608762
ASB2	870.256347	2406.27378	0	0	5.038031556	4.530304381
CEP128	498.126529	489.583775	353.0328844	382.166082	373.8219415	615.2153349
ERH	3666.60194	3426.07069	3862.300108	2419.71472	3314.017158	2990.000891
HIF1A	10111.3532	9654.49046	1633.308646	2961.15354	6430.745	2843.363999
SUSD6	543.055588	553.575015	2553.470806	3622.53218	682.1494727	3176.649432
SRSF5	4601.32172	3968.47263	6574.234538	4919.88545	3390.595237	6125.877584
SLC10A1	0	0	0	0	0	0
EIF5	8188.80945	8206.11476	4027.784272	7137.45443	12294.81221	8902.954169
SERPINA4	0	0	0	0	0	0
SLC8A3	0	0	0	0	2.015212623	0
DICER1	3806.27271	3269.64766	3373.871089	3165.94428	6462.786881	4368.119484

ZFYVE21	1016.76415	955.805668	1480.331072	1551.79543	2635.89811	1513.121663
MTHFD1	6960.09734	6311.7709	2895.471413	3455.58594	6646.171229	5831.407799
TCL1A	0	0	0	0	0	0
ZC3H14	3944.96676	3499.20353	3691.801271	2829.03471	4312.555012	3390.479799
TELO2	1099.78524	983.230485	1603.691995	1477.37362	2629.852472	2016.89151
PCNX	3324.7504	2681.53769	1734.073458	2057.66264	2474.681101	2365.724948
BDKRB1	0	0	2.005868661	0	3.022818934	4.97427421
GSKIP	615.332771	757.737543	1034.025295	1062.01943	1525.515955	993.0427203
VRK1	1603.77208	1389.52407	852.4941811	816.628575	1173.861353	1148.885191
PSMC1	5303.33827	5237.99789	5018.171894	3854.85898	7615.236599	4627.071682
PAPLN	58.603121	18.2832115	43.12617622	31.1767067	15.11409467	19.0272784
RPS6KA5	579.19418	521.071528	1157.386218	1090.17903	272.053704	1451.056493
PPP4R3A	3398.0043	3317.38715	3382.897497	3634.60058	4206.75635	4096.301221
C14orf93	149.437959	164.548904	374.0945054	318.806968	206.5592938	183.024297
PSMB5	3803.48906	3615.50507	2388.397844	2604.0998	5523.969852	2943.583393
YY1	5089.68106	5117.26775	4003.713848	4077.10867	6131.284404	4418.858893
ACIN1	7035.30468	6612.42816	5531.182834	4843.45224	5720.181029	6749.247467
CCNB1IP1	879.046815	776.020755	822.4061512	752.263761	775.8568597	618.8395784
TRIP11	1941.33582	2043.64659	3861.949081	3271.77411	2004.370779	3633.70278
APEX1	4164.72847	4099.50231	1949.704339	3264.5029	3983.067749	3397.728286
PABPN1	4079.53906	4029.75186	3288.220497	3044.97866	3273.108342	2727.27948
EFS	1.95343737	0	1002.934331	1323.50148	19.14451991	805.4881189
ARHGAP5	6732.51212	6039.5542	2397.01305	3157.89868	2751.772836	3658.664757
CINP	796.025727	835.949059	841.4619035	866.913586	887.7011602	1006.633633
DHRS2	9.76718684	47.7394967	5.014671654	40.2280086	141.0648836	530.0456126
SRP54	1998.36643	2238.67767	3464.135178	2705.33358	2345.707493	2735.397785
CPNE6	0	0	0	0	0	0
CHD8	3837.52771	3802.90799	2792.169177	3420.38643	5259.704945	5078.471211
PCK2	952.300717	742.501534	9687.3427	1744.88987	1780.440352	1953.467249
KIAA0391	1203.95229	1206.17393	1437.345307	1513.10615	1207.192969	1825.024059
DCAF11	1293.17554	1485.00307	2363.966364	3730.9366	2028.905992	2976.409978
PSMA6	4191.79334	4129.20237	5088.256945	4171.53387	4747.367364	4853.450992
NFKBIA	3296.42556	3381.37839	2821.254272	3740.1991	3212.24892	3008.122109

EMC9	381.897005	399.183451	425.2441562	431.445392	565.2671406	444.8758902
PSME2	2557.04951	2758.73347	1991.827581	1772.04378	2047.355264	3375.076764
BRMS1L	611.425896	682.573229	265.7775976	421.38839	482.6434231	350.6455591
REC8	69.3470266	53.8339005	72.21127181	76.4332164	30.22818934	205.6758189
TM9SF1	2377.67513	2608.04933	1835.660676	1686.87103	2397.095415	2061.587493
SEC23A	4840.6178	4603.30636	1312.841039	1540.73273	2621.791622	1419.797393
GMPR2	1676.04926	1617.04848	1275.732469	1254.10817	1264.545921	1131.670034
PNN	4988.10232	4306.71204	4361.761404	3611.46947	4195.67268	5552.341049
RABGGTA	603.612147	597.251576	613.7958104	677.841945	691.2179295	502.8637863
NFATC4	44.9290595	39.6136249	188.5516542	366.074878	56.42595343	54.36365257
PLTP	64.4634331	54.8496345	0	2.01140043	31.23579565	3.624243505
PCIF1	1750.27988	1208.72343	1195.497722	1321.49008	984.4313661	1043.782129
GSS	2881.32012	2807.4887	3078.005461	2286.96229	1601.086429	2306.830991
MMP9	24.4179671	69.0699101	0	0	118.8975447	46.20910469
VSX1	1.95343737	0	0	0	0	0.906060876
TRPC4AP	4094.40472	3934.95341	3498.234946	2994.97524	1511.409467	3200.207015
PYGB	5928.68241	5150.78697	5368.707472	2495.14223	3891.375574	3233.731267
ABHD12	2100.92189	1593.6866	1653.838711	1638.28565	1771.371895	1505.873176
PROCR	771.60776	1018.78117	4.011737323	60.3420129	92.69978064	67.04850484
GINS1	1364.476	1300.13948	519.5199833	918.204297	1679.679721	1478.69135
NINL	619.239646	547.480611	38.11150457	59.3363127	399.0120993	96.94851375
CD40	610.449177	726.24979	0	0	41.31185876	25.36970453
UQCC1	1831.34753	2028.42074	1313.843973	894.067492	843.3664825	1461.476193
ZMYND8	4351.28174	4353.43581	4042.828287	3538.05336	5505.560885	4435.167989
SGK2	10.7439055	5.07866986	28.08216126	11.0627024	6.045637868	22.6515219
IFT52	903.464783	934.475254	750.1948794	723.098455	432.2631075	644.209283
MYBL2	1732.69895	1565.24605	2261.616916	5436.81537	11980.43904	6856.16265
R3HDML	0	0	0	0	5.038031556	0
HNF4A	0	0	0	0	7.053244179	3.624243505
NDRG3	1904.60143	2332.1252	1073.139734	1533.69283	888.7087666	1343.688279
SLA2	0	1.01573397	3.008802992	2.01140043	0	0
C20orf24	3317.80593	3834.53795	2073.065262	1968.54754	3323.65995	2112.698387
NFATC2	1296.10569	1215.83356	0	3.01710065	765.7807966	74.29699185

RIMS4	0	1.01573397	0	0	825.2295689	28.08788716
PABPC1L	1690.70004	1660.72504	3162.251945	1189.74335	840.3436636	1591.042899
STK4	3589.44116	3693.20872	1235.615095	1282.26777	2055.516875	1683.461108
SALL4	2.93015605	1.01573397	1.002934331	0	683.1570791	52.55153082
ADNP	3686.13631	3668.83111	5010.659916	5702.32022	7324.290277	6723.877762
PFDN4	873.186503	828.838921	274.8040066	299.698664	4778.069128	516.4546994
DOK5	0	0	0	0	0	0
CSTF1	1521.72771	1587.5922	1253.667913	1177.67495	1820.744604	1243.115522
BMP7	0	0	0	0	4997.727304	251.8849236
RAE1	1793.2555	1673.92959	1063.110391	959.438006	4408.277612	1452.415585
TPD52L2	6954.23703	6169.56815	3903.420415	2724.44188	8188.816492	3777.367793
DNAJC5	6342.81113	5863.83222	4671.668112	3518.94505	6043.622655	4227.680048
NELFCD	4093.428	3387.4728	2928.568246	2614.82056	7380.71623	3392.29192
CTSZ	5636.64352	5404.72047	668.9571986	472.679101	181.369136	444.8758902
PRPF6	3417.53867	3423.02349	5087.88586	3663.76589	7168.111299	3874.316307
TUBB1	0	0	0	0	1.007606311	0
PRELID3B	3788.69177	3821.1912	2583.558836	2396.58361	6752.977498	2492.57347
HRH3	0	0	0	2.01140043	2.015212623	0.906060876
MTG2	1245.31632	1136.60631	980.8697754	668.790643	1571.865846	1171.536713
PSMA7	7069.48983	7275.70244	4619.515527	4166.61599	10599.01079	4610.943799
SLCO4A1	625.099958	970.025943	280.8216126	421.38839	1006.598705	540.0122822
NTSR1	8.79046815	17.2674775	0	0	64.48680392	3.624243505
MRGBP	2396.86765	2448.93461	740.1655361	674.824845	2436.392061	909.6851197
TCFL5	1273.64116	1183.33008	531.5551953	391.217384	1253.462251	530.9516734
DIDO1	3753.5299	3596.714	3522.305369	2985.92394	6180.657113	3602.498044
GID8	2998.52636	2864.3698	2033.950823	2276.90529	6055.713931	2412.840113
SLC17A9	2098.96845	2299.62171	0	0	98.74541851	45.30304381
BIRC7	0	0	0	0	1.007606311	0
NKAIN4	0	1.01573397	0	0	0	0
ARFGAP1	2667.41873	2847.10232	2858.362843	2020.45173	2962.362555	2055.852128
AVP	0	0	0	0	0	0
COL20A1	0.97671868	8.12587178	0	0	4.030425245	4.530304381
CHRNA4	0	0	0	0	0	0.906060876

EEF1A2	636.820582	483.489371	14945.7274	15264.5179	36060.21467	17099.18086
PTK6	406.314972	402.230653	689.0158852	490.781705	790.9709544	477.4940817
GMEB2	1299.03585	1173.17274	822.4061512	815.622875	1503.348616	1063.715469
C20orf27	1992.50612	2182.81231	309.9067082	419.37699	2491.810408	722.1305183
SPEF1	0	1.01573397	12.03521197	4.02280086	15.11409467	13.59091314
CDC25B	3666.60194	4413.36411	1989.821712	3969.49875	4303.486556	2527.003784
ISM1	226.598735	213.304134	0	2.01140043	34.25861458	5.436365257
RNF24	1879.20675	1844.57289	1308.829302	814.617175	915.914137	1511.309541
ARFRP1	1814.74331	1417.96463	1024.998886	762.320763	1152.70162	977.6396854
NDUFAF5	312.549979	323.003403	440.2881712	386.188883	267.0156725	471.1516556
SEL1L2	0	0	0	0	0	0
TRIB3	863.419317	943.61686	18080.90011	11015.4345	1610.154885	3667.734427
RASSF2	218.784985	237.68175	0	0	128.9736078	42.58486118
CSNK2A1	5442.11046	4766.41292	3378.314088	2589.96971	2797.991738	3860.390151
SLC52A3	7.81374947	5.07866986	319.9360515	457.593598	130.9888205	234.6697669
RPS10L	0	3.02688724	6.027635328	10.0570022	8.111230806	0.906060876
ANGPT4	1.95343737	0	0	0	7.053244179	1.812121752
RSPO4	0	0	0	0	0	0
CDS2	2541.42202	2496.6741	1124.289385	1335.56989	849.4121204	1163.382165
PROKR2	0	0	0	0	0	0
HM13	6110.02	7111.61062	8354.583386	5537.70721	3928.183433	6627.1014
SNPH	348.6202	673.431624	385.126783	967.47355	27.2053704	3900.592072
MYLK2	13.6740616	20.3146794	2.005868661	2.01140043	6.045637868	10.87273051
SIRPB1	8.52675411	11.1730737	3.119125769	1.13644124	1.007606311	0
SEC23B	2116.54939	2571.83842	4350.729127	3468.66004	2836.411766	4238.552779
FERMT1	609.472459	588.10997	0	0	159.2017972	42.58486118
HAO1	0	0	0	0	1.007606311	0
PDYN	0	0	0	0	0	0
CCM2L	0	2.03146794	5.014671654	18.1026039	52.39552819	45.30304381
PLCB4	0.97671868	1.01573397	3211.395727	3127.72767	622.7007004	2963.725126
MYL9	5251.81636	4258.97255	0	0	88.66935539	43.49092206
HCK	0	0	0	0	11.08366942	2.718182629
TM9SF4	3232.93884	3132.52357	2217.487805	2123.03316	1990.022465	3040.7403

TLDC2	1.95343737	0	0	0	0	2.718182629
CRNKL1	1246.29304	1368.19366	1620.741878	1493.46482	1110.382155	1684.367169
POFUT1	6445.36659	6486.47715	1527.468986	1455.24821	1783.463171	2551.467427
SAMHD1	2735.78903	3152.83825	566.6578969	518.941311	956.2183894	1197.812478
PAK7	0	0	0	0	0	0
KIF3B	3138.19713	3825.25414	1438.20783	2437.81732	2190.536121	2452.706792
MROH8	31.2549979	36.566423	17.04988362	27.1539058	10.07606311	13.59091314
NOP56	5850.53515	5542.86029	2818.225411	2635.92015	3751.318297	5300.438004
MANBAL	1338.1046	1350.92618	705.0628345	555.146519	674.0886223	517.3607603
IDH3B	1765.90738	1659.70931	1575.609834	1634.26285	885.6859476	1996.958171
MAPRE1	8403.68756	8112.66724	3002.785386	3764.33591	4678.316103	4486.813459
JAG1	1288.29194	1633.30023	671.9660016	682.870446	659.9821339	694.0426312
CDK5RAP1	1194.52695	1015.73397	1538.501263	926.249899	714.3928747	1282.982201
SNTA1	909.325095	916.192043	113.3315794	119.678326	254.9243968	96.94851375
OXT	0	0	0	1.00570022	0	0
TTI1	1580.33083	1558.13591	755.209551	646.665239	1284.698047	1105.394269
E2F1	2950.66714	2485.50103	1336.911463	2134.09586	1608.139673	3240.073693
RPRD1B	2002.2733	1832.38409	1246.647373	1213.88016	1760.288226	1593.761081
PXMP4	200.22733	251.902025	2780.133965	5567.55639	599.5257552	3284.470676
CHMP4B	3904.9213	3848.61602	3873.332385	3710.0281	2719.529434	3753.81021
BPI	0	0	0	0	0	0
CST9L	0	0	0	0	0	0
SLC32A1	0	0	0	0	0	0
CST3	3774.04099	3854.71042	1213.55054	1768.02098	2543.19833	1255.800374
ASIP	0	0	0	0	0	0
CST4	117.518792	94.3820007	6.017605984	37.210908	1.007606311	50.54913628
ACTR5	416.082159	448.954416	315.9243142	423.399791	308.3275313	458.4668033
WFDC2	2.93015605	3.04720192	1.002934331	3.01710065	10.07606311	8.154547886
AHCY	7689.60853	7482.84107	6094.6514	7378.68168	5996.18455	7873.487802
PPP1R16B	0	0	0	0	0	0
SPINT3	0	0	0	0	0	0
FAM83D	2692.81341	2608.40484	732.1420614	1460.27671	1651.466744	2016.89151
EPPIN	0	0	0	0	0	0.906060876

DHX35	749.143231	730.312726	397.161995	334.898172	296.2362555	400.4789073
DNTTIP1	1121.27305	1080.74095	670.9630672	620.517033	789.9633481	624.2759437
MAP1LC3A	101.578743	86.3373876	0	0	2.015212623	1.812121752
SYNDIG1	3.90687474	1.01573397	0	0	11.08366942	0
PIGU	755.003543	724.218322	665.9283369	679.853346	575.3432037	743.8759793
TNNC2	0	0	2.005868661	7.03990151	3.022818934	3.624243505
ACOT8	448.313876	477.394967	691.0217539	636.608236	359.7154531	490.178934
APMAP	4208.68081	4012.14919	9257.083872	4505.53697	2983.522288	5449.95617
CELF4	9.76718684	13.2045416	5.014671654	6.03420129	1.007606311	10.87273051
ZNF516	554.776212	522.087262	177.5193765	179.014638	732.5297883	251.8849236
CDH20	0	0	0	0	0	0
ADNP2	1422.1024	1540.86844	1489.357481	1839.42569	1346.162032	2044.073337
RBFA	613.340265	474.286821	487.0951164	476.520876	553.0045718	728.0017928
USP14	4504.62657	4469.22948	1026.00182	1194.77186	3465.158105	1569.297438
VAPA	4603.27516	4285.38163	4168.195078	3324.84491	2961.354949	3610.652592
METTL4	634.867145	656.164146	133.390266	147.837932	420.1718318	188.4606622
LPIN2	2990.71261	3333.6389	488.4290191	634.596836	1174.868959	657.8001961
SMCHD1	5270.37402	4403.20677	950.7817455	800.537372	2839.434585	1472.348924
MYOM1	4.88359342	1.01573397	9.026408976	5.02850108	6.045637868	3.624243505
MYL12A	5934.49389	4341.03369	1904.572294	1999.73431	5926.579106	2145.905519
CEP76	441.476845	391.057579	250.7335827	308.749966	282.1297672	391.4182985
ST8SIA5	4.88359342	3.04720192	2.005868661	3.01710065	1.007606311	16.30909577
CEP192	2225.94188	1721.66908	924.7054529	1180.69205	1126.503856	1677.118682
RNMT	2639.09388	2640.90833	990.8991187	1104.25884	1184.945022	1296.573114
SMAD7	142.600928	305.735926	351.0270157	497.821607	305.3047123	436.7213423
LIPG	123.066554	417.466663	0	437.479594	34.25861458	22.6515219
LAMA1	58.603121	207.20973	0	0	5.038031556	5.436365257
RNF125	83.9978068	117.530578	35.10270157	36.2052078	53.4031345	32.67255519
ANKRD12	3163.55275	3565.11451	2779.110972	3326.64512	1367.140395	2339.431061
NOL4	0	0	1.002934331	0	0	9.060608762
POLI	404.361535	308.783128	805.3562676	653.70514	508.8411872	676.8274745
MIB1	3838.50443	3537.80143	1529.474854	1686.55926	2398.103021	2064.006676
RBBP8	2501.37655	2513.94158	833.4384288	754.275162	1586.97994	1096.33366

RIOK3	2322.63703	1810.03794	1123.28645	1022.79712	1450.953088	1111.736695
CSTF2	1073.41383	1098.00842	694.0305569	547.100917	1358.253308	667.7668657
H2BFM	0	0	0	0	3.022818934	0
MXRA5	0	0	0	0	0	0.906060876
VSIG1	11.7206242	9.14160575	10.02934331	4.02280086	1.007606311	9.060608762
PSMD10	848.768536	929.396585	2299.72842	1463.29381	1568.843027	1197.812478
ATG4A	224.645297	236.666016	894.617423	907.141594	523.9552819	642.3971612
STS	2456.44749	2700.83663	5218.267323	8323.17498	708.3472368	3909.652681
TBL1X	2273.8011	1958.3351	3087.03187	2165.27256	3618.314264	1654.46716
GPR143	2.93015605	1.01573397	74.21714047	63.3591136	57.43355974	59.80001783
PGRMC1	1999.34315	2245.78781	2938.597589	2726.45328	3723.10532	2892.146317
POLA1	1976.87862	1894.34386	665.9483956	775.394866	1593.025578	1041.063947
MID1	1669.21223	1600.79674	31.09096425	31.1767067	102.7758438	66.14244396
NKAP	460.0345	441.844278	895.6203573	647.670939	754.6971272	480.2122644
RHOXF1	0	0	0	0	0	0
NXT2	312.549979	331.129275	440.2881712	392.223084	462.4912969	367.8607157
GUCY2F	0	0	0	0	0	0.906060876
ATP1B4	0	0	0	0	0	0
MCTS2P	70.8218718	87.9117753	38.97402809	36.8890839	44.08277612	98.58848394
ALG13	821.420413	809.539976	969.8374978	828.696978	1028.766044	601.6244218
PRPS2	2795.36887	2634.81392	275.8069409	371.10338	1558.766964	272.7243237
TLR8	0	0	0	0	0	0
MOSPD1	520.591058	567.79529	580.6989775	538.049615	563.251928	526.4213691
AMMECR1	313.526698	340.270881	2136.250124	1693.59916	2328.578185	1664.43383
CHRDL1	21.487811	28.4405512	1.002934331	3.01710065	7.053244179	13.59091314
WDR13	1097.8318	1296.07655	1694.959019	1489.44202	801.0470175	768.339623
SUV39H1	1060.71649	1115.2759	509.49064	616.494232	864.5262151	755.6547707
PAGE4	0	0	1.002934331	0	0	0.906060876
SRPX	832.164319	1075.66228	0	0	46.34989032	28.08788716
GLRA2	0	0	0	0	0	0
XIAP	1842.09144	1930.91028	3244.49256	2489.10803	4120.102207	2005.112719
STAG2	3941.05989	3298.08821	4011.737323	4548.78207	4394.171124	2744.458394
ATP11C	1236.52585	1102.07136	574.6813715	743.212459	1883.216196	808.2063016

MCF2	0	1.01573397	0	2.01140043	4.030425245	4.530304381
F9	0	0	0	0	0	0.906060876
ABCD1	426.826065	280.342576	2890.456741	1936.97862	860.4957898	3628.773809
CCDC22	515.707465	524.11873	1170.424364	887.02759	814.1458995	577.1607781
CACNA1F	1.95343737	4.06293589	5.014671654	2.01140043	0	3.624243505
SYP	34.1851539	46.7237627	29.08509559	46.2622099	23.17494516	15.4030349
PLP2	7175.95217	7832.32466	750.1948794	2050.62274	2030.326717	882.5032934
BMX	0	0	0	0	0	0
LUZP4	0	0	0	0	0	0
PLS3	7657.47448	6687.59247	2711.93443	3380.15842	2206.657822	2067.630919
NAA10	1093.92493	1056.36333	2187.510098	2104.92049	3905.482063	2725.431116
RENBP	0	0	156.4577556	90.5130194	6.045637868	30.80606979
ELF4	1576.42396	1660.72504	410.2001413	536.038215	2930.119153	618.8395784
SMARCA1	2414.44859	1992.87005	0	0	115.8747258	50.73940907
MTMR8	51.7660902	42.6608268	16.04694929	36.2052078	5.038031556	16.30909577
ASB9	131.857022	149.312894	6.017605984	33.1881071	160.2094035	26.27576541
ZC3H12B	3.90687474	2.03146794	0	0	0	0
RBBP7	8000.30274	8250.80706	3509.267223	4378.81874	6829.555578	3822.670837
PPP1R2P9	0	0	0	0	0	0
KCND1	101.578743	156.423032	79.23181213	89.5073192	42.31946507	99.66669638
OPN1LW	0	0	0	0	0	0
SLC25A14	119.159679	113.762205	280.8216126	293.664463	296.2362555	212.9243059
FMR1	1174.99258	1093.94549	2883.436201	3337.91902	2172.399207	3059.767579
PIM2	419.989034	707.966579	211.6191438	550.118018	338.5557206	201.1455145
SCML2	546.962463	457.080288	187.5487198	159.906334	122.92797	188.4606622
SLC35A2	769.654323	885.720024	1146.35394	1126.38424	920.9521685	1061.903347
PQBP1	1451.40396	1592.67087	2195.42325	2929.60473	1414.679261	1236.773096
RS1	0	0	0	0	0	0
PCSK1N	125.019992	133.06115	0	0	697.2635674	154.9364098
EMD	1430.89287	1348.89472	5490.062526	6203.15893	3655.595697	6600.653483
TAZ	398.501223	443.875746	2826.268944	2485.08523	478.6129979	1784.033865
RAB40AL	9.76718684	2.8643698	6.709630672	2.01140043	10.64032265	9.060608762
PGK1	18540.0741	16956.6629	14661.89698	14006.3869	14794.68347	12868.78262

GATA1	0	0	0	0	0	0
MAGT1	3485.90898	3431.14936	3341.77719	2763.66419	2822.305278	2694.625046
SMS	7590.9404	6894.62953	2300.691237	2482.01785	3155.732282	2217.103782
PHEX	8.79046815	11.1730737	359.0504904	342.943774	44.3346777	236.4818887
UBL4A	1527.58802	1316.39123	5645.517348	6732.15724	2720.53704	7251.205192
CD99L2	1956.36752	1791.75473	727.1273898	621.522733	969.3172715	822.7032756
EEA1	2805.13606	2652.0814	1293.785287	1925.91591	1879.185771	2103.873354
GPR50	0	0	0	0	0	0.906060876
RP2	1266.80413	1205.67623	479.4026101	1035.87122	260.9700346	353.3637417
JADE3	736.445888	754.690341	1151.368612	1851.4941	867.549034	1749.603552
CDK16	6086.91084	5930.87066	4265.479709	5441.84387	3573.979586	4975.180271
USP11	2155.61814	2253.91368	1206.53	1665.43956	1789.508809	1590.136838
PCYT1B	0	0	0	0	2.015212623	0
BRS3	0	0	0	0	0	0
HTATSF1	2007.1569	1933.95748	3453.102901	4946.03366	3982.060142	4385.334641
VGLL1	0	0	0	0	8.06085049	6.342426133
CD40LG	0	0	0	0	0	4.530304381
TIMP1	2777.78794	3371.22105	7.020540315	118.672625	1616.200523	422.2243683
KLHL4	385.80388	309.798862	0	0	32.24340196	2.718182629
GABRE	110.369211	61.9597723	2.005868661	0	13.09888205	10.87273051
PCDH11X	0	0	21.75364563	74.2508469	0	59.80001783
FGD1	827.280725	1004.5609	695.0334912	880.993389	159.2017972	578.9728999
PIN4	566.448001	532.234444	500.464231	254.442155	421.1794381	211.1121841
PORCN	616.30949	698.824973	244.7159767	307.744266	101.7682374	157.6545925
ITIH6	0	0	10.02934331	41.2337088	3.022818934	11.77879139
MAGED2	2387.10046	2617.54645	9633.184246	10533.7041	1895.307472	4518.52559
RBM3	8173.18195	7097.949	8236.096724	3552.13316	7239.651347	4351.810388
KLF8	0	0	18.05281795	25.1425054	69.52483548	31.71213067
SRPX2	82.0443694	100.557663	0	0	10.07606311	6.342426133
SYTL4	512.777309	522.087262	110.3227764	328.86397	563.251928	165.8091403
ZDHHC15	0	0	0	0	0	0
CENPI	986.485871	1031.98572	143.4196093	739.189658	853.4425457	395.9486029
DRP2	35.1618726	51.8024326	27.07922693	56.3192121	21.15973254	41.6788003

TAF7L	0.97671868	3.04720192	2.005868661	11.0627024	20.15212623	27.18182629
PBDC1	777.468072	906.034703	606.7752701	476.701902	735.5526072	491.9910558
GLA	632.913707	825.791719	888.599817	1511.56742	1779.432746	1271.203409
ARMCX3	964.99806	1227.00664	2549.459069	2451.89713	1749.204556	1732.388395
BEX4	0	0	0	0	39.29664614	14.49697402
KIAA0226L	0	0	0	0	28.21297672	28.08788716
NALCN	156.274989	132.045416	0	0	0	0
FGF14	0	0	0	0	0	0
HTR2A	0	0	0	0	0	0
NDFIP2	884.907128	873.531216	543.5904072	621.522733	950.1727515	647.8335265
TNFSF13B	0	0	3.008802992	5.02850108	12.09127574	11.77879139
FNDC3A	1728.79207	1805.975	1580.624505	1562.85813	1568.843027	1972.494527
MLNR	0	0	0	0	0	0.906060876
CDADC1	173.855926	198.068125	260.762926	294.670163	82.62371753	297.1879674
CAB39L	357.479038	365.66423	1478.325203	2000.33773	201.5212623	2025.952119
KLF5	837.047912	735.391396	164.4812302	64.3648138	1300.819748	250.9788627
STK24	5270.37402	5085.78	1295.791155	1403.9575	3681.793461	1409.830723
ACP5	0	0	0	11.0627024	0	1.812121752
DNAJC3	2599.04842	2800.37856	2244.567032	1693.59916	2260.060956	1729.670213
UGGT2	595.798397	512.945656	377.1033083	370.097679	542.0921955	375.1092027
ARHGEF7	2578.53733	2180.78084	815.3856109	749.246661	1518.462711	833.5760061
FGF9	0	0	2.005868661	0	0	0.906060876
SGCG	0	0	0	0	9.068456802	1.812121752
PARP4	5168.53156	5127.06958	135.3961346	162.923435	1862.671103	351.5425593
SUPT20H	1041.18212	1239.19545	1949.704339	1544.75553	1312.911024	1867.391466
MRPS31	372.129819	363.632762	538.5757356	439.490994	340.5709332	434.0031597
SLC25A15	371.1531	353.475422	169.4959019	254.442155	824.2219626	443.9698293
KPNA3	2495.51624	2493.6269	1492.366284	1824.34019	2413.217116	2271.494617
FLT1	20.5110924	15.2360096	0	0	2.015212623	0
RGCC	26.3714045	28.4405512	0	0	5.038031556	10.87273051
VWA8	594.821678	579.984098	3541.361122	2073.75384	811.1230806	3272.691885
DGKH	958.161029	669.368688	3500.240814	3376.13562	1925.535661	5926.544191
KATNAL1	1001.13665	930.412319	143.4196093	355.012176	418.1566192	232.8576452

INTS6	905.41822	854.232271	6040.643386	3175.98117	1443.899844	4350.904327
IRG1	0.97671868	0	0	0	0	0
DHRS12	83.9978068	73.132846	271.7952036	299.698664	53.4031345	212.018245
MEDAG	0	1.01573397	0	0	0	0
TSC22D1	2043.29549	2911.09356	7832.917123	11902.4621	2267.1142	5146.425777
CLN5	293.992324	339.255147	190.5575228	174.991838	341.5785395	227.4212799
OLFM4	0	0	0	0	0	0.906060876
MSLN	925.929312	665.305752	0	2.01140043	4.030425245	9.060608762
MGRN1	1346.89507	1247.32132	2137.253059	2163.26116	2886.792082	3143.125179
ZNF629	857.559004	684.604697	1901.563491	1607.10894	2048.463631	2390.188591
TRADD	249.063264	226.508676	802.3474646	723.098455	615.6474562	762.9032577
HSF4	163.473406	205.909591	541.7951548	746.199389	191.2839821	493.6129047
CORO1A	96.3337638	144.457686	4.011737323	2.01140043	1281.675228	83.89217653
MAPK3	902.488064	908.066171	3095.055345	4886.69735	4874.799334	3147.655484
GDPD3	22.4645297	15.2360096	47.13791354	76.4332164	804.0698364	114.1636704
ELMO3	24.4179671	25.3933493	1554.548213	1872.6138	714.3928747	1692.521717
MT4	0	0	0	0	0	0
PHKB	1215.03804	1086.83535	5980.497414	4495.47996	2133.102561	5301.362187
LYRM1	419.989034	355.50689	320.9389858	256.453555	531.008526	258.2273497
NUTF2	2962.38777	2814.59884	2184.390972	1978.21232	3275.728118	2701.873533
NUP93	1808.883	1726.74775	1388.061114	1423.06581	1918.482417	2452.706792
CENPT	856.582286	859.310941	1081.163209	1077.10493	858.4805772	1206.873087
TSNAXIP1	16.6042176	23.3618814	22.06455528	35.1995075	9.068456802	39.86667855
NFAT5	3867.51297	3081.38136	1765.184481	1731.39338	3860.562973	2851.989699
LONP2	1587.16786	1735.88936	10952.04289	8534.37203	3785.576912	14358.3467
N4BP1	1117.36617	1082.77241	1614.724272	1757.96398	1239.355763	2115.652146
CBLN1	0	0	37.10857024	38.2166082	0	28.99394804
ARL2BP	1423.07912	1494.14467	1117.268844	1506.53892	1394.527135	1337.345853
PLLP	201.204049	134.076884	11.03227764	18.1026039	180.3615297	90.60608762
ZNF423	0	0	558.6344222	165.940536	48.36510294	175.77581
CCL22	0	0	6.017605984	0	108.8214816	34.43031329
DHODH	323.293884	243.776153	240.7042394	140.79803	575.3432037	391.4182985
CCL17	0	0	7.020540315	0	0	1.812121752

CTCF	1867.48612	1911.61134	2477.247797	3107.61367	3875.253873	3141.313058
ACD	632.913707	680.541761	866.5352617	909.152995	724.4689378	1292.042809
POLR2C	1554.93614	1541.88417	1614.724272	1568.89234	2183.482877	2302.300686
PARD6A	133.81046	192.989455	403.1796009	432.451093	143.0800962	539.1062213
ZNF821	59.5798397	62.9755063	387.1326517	340.932373	194.4680181	289.0334195
MMP15	399.477942	655.148412	3270.568852	3249.4174	1303.842567	3732.064749
USB1	2865.69262	3087.83128	808.3650706	931.2784	1238.348157	1481.409533
CYB5B	1980.78549	2293.52731	1107.239501	3716.0623	3415.785395	1639.064125
CCDC113	31.2549979	51.8024326	407.1913383	178.008938	174.3158919	413.1637595
PRSS54	0	0	0	0	0	0
NME3	294.969043	301.67299	2283.681471	1497.48762	790.9709544	1551.17622
NDRG4	23.4412484	41.6450929	4.011737323	0	508.8411872	78.82729623
PSMD7	2408.58827	2290.48011	1956.724879	1677.50796	5213.355055	2282.367347
SETD6	271.527794	260.027897	632.8515627	512.90711	536.0465576	985.7942333
SLC38A7	434.639814	681.557495	822.4061512	1256.11957	510.8563998	834.482067
VAC14	1896.78768	2423.54126	972.8463008	1189.74335	1732.075249	1123.515486
HAS3	43.9523408	34.5349551	15.04401496	14.079803	40.30425245	41.6788003
TANGO6	670.029017	636.865201	218.6396841	205.162844	322.4340196	326.1819154
COG4	941.556811	863.373876	1428.178487	1344.62119	2479.719132	1687.085351
SMPD3	31.2549979	22.3461474	23.06748961	19.1083041	13.09888205	37.0216474
SLC7A6OS	710.074483	683.588963	506.481837	483.741804	846.3893015	684.9820224
SLC7A6	2025.71455	1606.89114	3154.22847	2780.7611	1830.820668	3538.167721
PLA2G15	544.032307	668.352954	594.7400581	919.209997	579.373629	1110.830634
ESRP2	319.38701	205.178262	4797.034904	5435.80967	3804.721431	6167.556384
FA2H	0	0	210.6162094	466.6449	275.076523	291.7516021
WDR59	472.731843	496.693912	723.1156524	665.773543	1664.565626	900.6245109
MON1B	821.29344	963.921382	1132.312859	1133.42414	2382.948622	1040.157886
CMC2	604.207945	702.512087	415.8366322	640.631037	1626.145598	545.4486475
AXIN1	1250.19992	1108.16576	1370.008296	1221.92576	1581.941909	1593.761081
HCFC1R1	1075.36727	915.176309	817.3914795	909.152995	370.7991226	625.1820046
NPRL3	1475.82193	1423.0433	1930.648587	1660.41106	1653.481957	2295.052199
MLYCD	156.421497	164.376229	202.8133804	204.358284	906.5232461	348.9965283
MPG	640.727457	655.148412	1339.920266	1546.76693	1610.154885	1311.976149

NECAB2	0	0	0	5.02850108	62.4715913	11.77879139
HSDL1	743.282918	779.067957	545.5962759	681.864746	1654.489563	848.979041
TAF1C	660.26183	690.699101	882.582211	955.415205	1706.885091	1158.851861
NAGPA	272.504513	285.421246	239.701305	341.938073	308.3275313	373.297081
WFDC1	0	0	1.002934331	0	0	1.812121752
SEC14L5	0	1.01573397	103.3022361	130.741028	10.07606311	163.0909577
COTL1	10566.1427	9424.99553	319.9360515	564.197821	5739.325549	1360.903436
USP10	2643.00076	2403.22658	2111.176766	1717.73597	7662.835921	3288.09492
CRISPLD2	456.127625	306.75166	15.04401496	69.3933149	546.1226207	135.9091314
TSC2	1761.02379	1517.50655	2425.095212	2370.43541	2486.772376	2751.706881
ZNF500	277.388106	213.304134	560.6402909	513.91281	388.9360362	661.4244396
RP11-723C11.2	0	0	0	0	0	0
NME4	1022.97608	846.309546	4476.888236	2643.62381	2216.713733	4400.329948
ABCC1	4058.26613	3013.6827	2104.156226	1578.94934	3113.503502	3472.931338
NOMO3	1921.77215	1695.27016	1878.716647	2582.90969	4324.33393	3603.802771
LMF1	57.6264023	45.7080288	698.0422942	448.542296	378.859973	534.5759169
FOXF1	0	0	0	0	10.07606311	0.906060876
NARFL	518.637621	482.473637	1730.06172	1595.04054	983.4237598	1397.145871
MTHFSD	433.663096	416.450929	474.3879384	356.017876	729.5069694	480.2122644
CLCN7	2122.4097	2564.72828	2515.359301	3674.82859	1404.603198	2323.140087
HAGHL	293.992324	286.43698	1859.440249	1679.51936	795.0013796	1514.027724
FAM173A	313.526698	353.475422	949.7788112	984.580511	490.7042736	1482.315593
SLC7A5	17431.8793	13890.5887	9515.730607	593.433526	34526.55725	4418.614257
METRN	590.914804	635.849467	2571.523624	2300.03639	2932.134366	2928.388752
FBXO31	676.748842	664.462693	921.6966499	704.070607	1250.08677	841.9570692
STUB1	1855.7655	1824.25821	3519.296566	3502.85385	4360.920115	4768.598391
RHBDL1	63.4867145	60.9440383	76.22300913	34.1938073	31.23579565	61.61213958
NUBP1	604.588865	620.613457	483.4143474	670.802044	681.1418664	495.6152993
UBE2I	2889.13387	3009.61976	3031.870482	3050.28875	4209.779169	3321.619172
ZP2	0	0	5.014671654	20.1140043	0	9.060608762
MEFV	0	1.01573397	1.002934331	0	4.030425245	3.624243505
CRYM	0.97671868	4.06293589	0	0	0	3.624243505
EEF2K	994.29962	1075.66228	2242.561163	2382.50381	1565.820208	2402.873444

CAPN15	2022.78439	1822.22675	1663.868055	1391.8891	3169.929455	2289.615834
PIEZO1	6372.11269	5091.8744	2499.312352	2944.69023	20935.03633	7328.220367
GSPT1	5709.89743	5424.01941	5360.683998	6789.48216	8848.949767	8478.917679
ZNF174	227.575453	231.587346	356.8941816	289.641662	437.3011391	406.8213334
CLUAP1	311.57326	287.452714	571.6725685	364.063478	575.3432037	479.3062035
UBFD1	1447.49709	1327.5643	2239.55236	2052.63414	4050.577371	3019.9009
PRSS33	0	2.03146794	0	0	0	0.906060876
EARS2	681.749641	657.17988	996.9167247	711.030052	1355.230489	1423.421636
TCEB2	2049.1558	2142.18295	2339.845794	1728.79867	2462.589825	2103.873354
GGA2	2137.06048	2075.14451	2482.262469	2287.96799	2657.057843	3177.555493
AQP8	0	0	0	0	1.007606311	7.248487009
CPPED1	1186.7132	1189.42448	1.002934331	0	764.7731903	102.384879
USP31	766.724167	789.225296	600.7576641	794.50317	1231.294912	865.2881368
HMOX2	1475.82193	1383.42967	1237.620964	1156.55525	1471.105214	1268.485227
DNAJA3	1565.68005	1551.02578	3129.155112	2037.54864	3695.89995	3637.834418
CORO7-PAM16	0	18.2121101	0	0	17.99584872	0
BFAR	1575.44724	1657.67784	1338.917331	1398.929	1411.656442	1618.224725
SALL1	0	0	0	0	0	0
TOX3	0	0	1363.99069	722.092755	6.045637868	873.4426846
RRN3P2	1.24043273	1.08683535	0	2.38350951	6.640125591	4.992395428
RBL2	934.71978	844.074931	4893.3166	4288.30572	2881.75405	3130.440327
QPRT	0.97671868	0	704.0799588	2679.53737	1117.022281	939.2227042
XYLT1	0	0	8.023474646	6.03420129	178.3463171	27.18182629
PYCARD	87.9046815	104.620599	74.21714047	75.4275162	620.6854878	204.769758
RPGRIP1L	306.689667	353.475422	495.4495594	340.932373	312.3579565	508.3001515
MAZ	4287.79502	4647.99866	11775.45198	18180.9077	14019.70323	15670.32285
STX4	716.911514	905.018969	1194.494788	1359.70669	1035.819288	1172.442774
CDIPT	935.696499	990.340623	1620.741878	1651.35975	2566.373275	1660.809586
BCKDK	1523.68115	1233.10104	1693.956085	1641.30275	1862.056463	2200.821868
KAT8	457.104344	481.457903	1203.521197	1092.19043	910.8761054	1060.091225
NOMO1	3706.73531	3912.36348	4519.613239	5143.59341	8155.24305	6988.103235
IL21R	0.97671868	1.01573397	0	27.1539058	7.053244179	0
SYT17	76.897062	53.8339005	441.2911055	288.635962	84.63893015	327.9940372

TMC5	108.415774	68.0541761	465.3615294	432.451093	41.31185876	320.2109743
CCP110	1240.43273	1229.03811	886.5939483	984.580511	1479.166065	1452.415585
C16orf62	1057.78633	1077.69374	1219.568146	1241.03407	669.0505907	927.4348522
SLC6A2	0	0	0	0	0	0
RNF40	2467.1914	2637.86113	3986.663965	4146.50199	5546.872744	5631.168345
KNOP1	852.675411	910.097639	2065.041787	1783.10648	2035.364749	2420.0886
AQP9	0	0	0	0	0	1.812121752
AAGAB	958.161029	987.293421	1850.41384	2120.01605	2578.464551	2539.688636
IQCH	16.6042176	29.4562852	107.3139734	71.4047153	119.905151	161.278836
LACTB	1520.75099	1238.17971	597.7488611	607.44293	1392.511922	807.3002407
CORO2B	337.944665	431.686938	0	0	34.25861458	20.83940015
CSK	3089.3612	3094.94141	753.2036824	1061.01373	3780.53888	1640.876247
HERC1	1970.04159	1856.7617	3281.60113	3960.44745	3901.451637	4934.407532
TRIP4	426.826065	376.837304	987.8903157	901.107393	813.1382932	974.9215028
MTFMT	284.225137	290.499916	396.1590606	306.738566	445.3619896	462.0910469
RASL12	0	0	0	0	1.007606311	0
AP3B2	2.93015605	0	0	0	90.68456802	12.68485227
ACSBG1	0.97671868	3.04720192	0	1.00570022	4.030425245	8.154547886
IGDCC4	375.059975	527.165932	1.002934331	1.00570022	27.2053704	7.248487009
RAB11A	2521.88764	2637.81034	8769.657788	7221.68182	3711.658913	6654.111075
CTSH	744.259637	853.216537	178.5223109	375.12618	1740.1361	569.9122911
GOLGA8UP	2.93015605	0	1.002934331	10.0570022	0	6.342426133
TTC23	283.248418	317.924733	367.073965	285.618861	218.6505695	352.4576808
CD276	2132.17689	2753.6548	16.04694929	20.1140043	4003.219875	405.9152725
FAH	899.557908	1119.33884	2101.147423	1611.13175	527.9857071	1313.78827
CEMIP	7392.78372	3074.62673	1.002934331	9.05130194	124.9431826	116.881853
RPAP1	969.881653	949.711264	984.8815128	1019.78002	672.0734096	1311.070088
HOMER2	737.422606	669.368688	47.13791354	68.3876147	1303.842567	168.527323
EHD4	3682.22944	3414.89761	1366.999493	2104.93055	2217.741491	2250.655216
TMEM87A	899.557908	998.466495	2904.497822	3277.577	907.8532865	2717.276568
ZNF106	4307.3294	3672.89404	3748.968528	3751.2618	2743.711986	5817.816886
CEP152	796.025727	748.595938	362.0592934	413.342789	433.2707139	569.9122911
ATP8B4	0.97671868	0	9.026408976	6.03420129	8.06085049	3.624243505

OCA2	0	0	0	0	0	0
DTWD1	262.737326	353.455108	849.3850847	600.292402	564.2595343	656.8397716
TGM5	12.6973429	17.2674775	0	0	0	0
FAM189A1	4.88359342	5.07866986	0	0	2.015212623	4.530304381
GABPB1	948.393842	854.232271	1018.98128	1000.67171	2165.345963	1109.924573
TJP1	4002.59317	3345.8277	2369.933823	2895.41092	4434.475376	3505.54953
BMF	190.460143	299.641522	291.8538902	274.556159	162.2246161	302.6243326
DMXL2	1275.5946	1145.74792	2008.877464	1572.91514	3489.340656	2268.776434
SCG3	0	0	0	0	0	0
DNAJC17	264.690763	227.52441	983.8785784	538.049615	173.3082855	644.209283
EIF3J	2070.64361	2124.91547	2465.212585	1663.42816	2413.217116	2685.564437
SPG11	1408.42834	1485.00307	4148.136392	4080.12577	1360.26852	4205.028526
RHOV	23.4412484	38.5978909	854.5000498	682.870446	543.0998018	865.2881368
VPS18	644.634331	944.632594	1812.302336	2580.62675	1100.306092	2195.385503
OIP5	388.734036	402.230653	210.6162094	349.983675	403.0425245	459.3728642
SLC30A4	275.434669	241.744685	250.7335827	579.283324	148.1181278	371.4849592
BLOC1S6	1385.94428	1441.32651	1738.997865	2054.64554	854.9035748	1766.818709
MYEF2	1145.69102	1015.73397	2300.731355	1879.6537	1146.655982	2024.139997
SGK3	337.944665	336.207945	48.14084787	109.621323	331.3009552	104.1970008
PDGFRL	59.5798397	119.856609	1.002934331	0	367.7763036	41.6788003
CSPP1	543.055588	603.34598	843.4677721	785.451868	432.2631075	874.3487455
ZDHHC2	911.278532	962.915806	7.020540315	3.01710065	190.4375928	27.18182629
BRF2	169.949051	113.762205	316.9272485	255.447855	347.6241774	314.403124
TRIM35	344.781695	331.129275	192.5633915	206.168544	660.9897402	290.8455413
ZFAND1	1081.22758	1069.56787	2107.165029	1052.96813	978.3857283	1108.112452
RP1	0	1.01573397	0	0	0	0
CA2	0.97671868	3.04720192	47.13791354	22.1254047	2723.559859	54.36365257
FZD3	133.81046	145.249958	6.017605984	4.02280086	868.5566403	57.98789608
INTS9	240.272796	277.295374	169.4959019	166.946236	464.5065095	244.6364366
RIPK2	691.516828	772.973553	773.262369	1069.05933	2050.478843	1315.600392
EYA1	0	0	0	0	1.007606311	0
NBN	2547.28233	2427.60419	1643.809368	2112.97615	6689.498301	2805.164473
TRPA1	0	0	0	0	0	0

CPQ	178.739519	154.391564	0	0	35.2662209	6.342426133
DECR1	861.465879	888.767226	1422.160881	1401.9461	2533.122267	1205.967026
CALB1	0	0	0	0	22.16733885	0
IMPAD1	3429.2593	3672.89404	3929.496708	3260.4801	2640.936142	3206.549441
SFRP1	0.97671868	1.01573397	11.03227764	4.02280086	0	16.30909577
LAPTM4B	3524.97773	3966.44116	820.4002825	667.784943	10487.16649	1515.839846
UBE2W	733.291086	772.465686	965.053501	924.731291	641.2809608	1081.972595
POP1	738.399325	750.627405	912.6702409	1083.13913	2188.520908	1412.548906
NIPAL2	252.970139	350.42822	527.543458	572.243423	1809.298197	578.066839
IKBKB	1242.38617	1071.59934	3007.800058	2233.66018	543.0998018	1828.430848
PLAT	9430.21889	5686.07878	0	0	71.5400481	57.98789608
JPH1	118.182961	101.573397	188.5516542	399.262986	308.3275313	404.1031508
DKK4	0	0	0	0	0	0
STK3	882.95369	816.650114	764.23596	760.309363	1032.796469	565.3819867
GDAP1	181.669675	180.800647	78.2288778	69.3933149	327.4720512	263.663715
RAB2A	1629.16676	1923.80014	3225.436808	2859.20571	1875.155345	2424.618905
EIF3E	9119.47584	8726.12993	8639.978379	7058.80867	11468.16192	6075.980811
EMC2	906.394939	957.837136	1140.336334	974.523509	1238.348157	805.4881189
ESRP1	0.97671868	8.12587178	9644.216524	5503.19158	5820.94166	6381.386751
WISP1	0	0	0	0	1.007606311	0
NDRG1	9778.90746	7421.96814	2449.165636	425.411191	1522.493136	1157.9458
ZC2HC1A	154.321552	161.501702	125.3667913	196.111542	198.4984433	113.2576095
IL7	1.95343737	11.1730737	0	0	7.053244179	5.436365257
STMN2	0	0	0	0	0	0.906060876
ARMC1	1101.73868	1017.76544	1738.085195	1810.26039	1303.842567	1949.843006
TRPS1	178.739519	161.501702	6760.780323	8875.3044	3295.880244	10970.58509
SPAG1	937.649937	982.214751	434.2705652	641.636738	919.9445622	610.6850305
CHRAC1	1834.27769	1701.3544	766.2418287	1044.92252	1006.598705	1096.33366
NCALD	3.90687474	4.06293589	3.008802992	8.04560172	5.038031556	9.060608762
SNX16	394.594348	479.426435	348.0182128	416.359889	237.7950895	285.409176
GML	0	0	0	0	0	0
UBR5	5922.8221	5276.73799	4302.588279	5410.66716	15299.49423	9359.608851
GSDMD	1152.52805	1367.17793	1496.378021	2072.74814	39.29664614	1631.815638

TSTA3	1468.00818	1410.85449	5789.939891	3112.64217	1331.047937	5830.501738
PYCRL	572.357149	559.669419	635.8603657	596.380228	915.914137	1322.848879
EEF1D	9093.25095	9157.85749	14113.28187	14569.569	11561.25466	10012.87874
ANXA13	0	0	0	0	0	0
SQLE	431.709658	1498.20761	824.4120198	23235.6978	6268.318863	1127.13973
SH2D4A	680.772923	568.811024	434.2705652	372.10908	759.7351587	651.45777
INTS10	985.509152	923.302181	952.7876142	803.554472	1551.713719	1056.466982
ERI1	1181.82961	931.428053	209.6132751	204.157144	1009.621524	417.6940639
SLC39A14	2498.44639	2786.15829	536.5698669	308.749966	1830.820668	640.5850395
MTMR9	322.317166	388.010377	207.6074065	257.459255	644.8680392	330.7122198
LEPROTL1	505.940278	515.992858	360.0534247	336.909572	650.9136771	420.4122465
DCTN6	383.850443	312.846063	431.2617622	401.274386	442.3391707	386.8879941
R3HCC1	339.898102	275.263906	205.6015378	277.573259	682.1494727	363.3304113
GSR	1302.94272	1451.48385	5269.416974	6794.51066	3467.173317	3715.755653
TNFRSF10A	619.239646	652.10121	335.9830008	445.525195	601.5409678	459.3728642
UBXN8	189.483425	203.146794	64.18779717	48.2736103	185.3995613	116.881853
PPP2CB	1964.18127	1758.23551	749.191945	637.613937	1054.963808	605.2486653
ERICH1	179.716238	187.910785	310.9096425	332.886771	400.0197056	319.8394893
NEFM	0	0	0	0	0	0
TUSC3	1038.25196	1170.12554	540.5816043	682.870446	970.3248778	722.1305183
ARHGEF10	1713.16457	1362.09926	472.3820698	301.710065	1150.686407	572.6304737
KLHDC4	800.909321	686.636165	756.2124854	649.682339	2022.265867	1154.321556
MCM4	6436.57613	5624.119	3172.281288	4442.17785	11181.40724	8664.660159
ADAM2	0	0	0	0	59.44877237	13.59091314
KCTD9	1176.54556	1033.88514	609.753985	700.97305	1482.14858	783.62487
FGL1	0	0	0	0	0	0
ASAH1	740.352762	1223.95944	1947.69847	1774.05518	1797.569659	1649.030795
BNIP3L	2845.18153	2444.87167	1627.762419	1862.5568	1635.345043	1893.667231
MAN2B1	1990.29873	2050.03556	1565.771048	2475.72216	1579.120611	3357.444819
KCNN4	756.95698	1204.66049	0	0	891.7315855	52.55153082
TULP2	0	0	0	0	0	0
NUCB1	3345.26149	3466.70005	2784.145702	3665.77729	1932.588905	2952.852395
DHDH	4.88359342	5.07866986	3.008802992	2.01140043	5.038031556	3.624243505

GYS1	1904.60143	1844.57289	2390.995444	2453.90853	2536.145086	3202.925197
MAP4K1	1.95343737	6.09440383	10.02934331	25.1425054	15.11409467	28.08788716
CGB2	0	0	0	0	0	0
ECH1	1543.21552	1608.92261	730.1361928	637.613937	1787.493596	432.1910379
HNRNPL	8723.97315	7734.60089	5480.935824	6987.51458	9007.87951	7856.381372
NFKBIB	694.446984	813.602912	537.5728013	422.394091	809.107868	499.2395428
LHB	1.95343737	5.07866986	12.03521197	4.02280086	0	6.342426133
CGB	0	0	0	0	0	0
TUBB4A	4.88359342	8.12587178	2.005868661	1.00570022	35.27629696	4.530304381
SARS2	792.118853	736.40713	213.6250124	312.772767	598.5181489	352.0952565
KCNA7	0	0	0	0	0	0
SNRNP70	6452.20363	6361.54187	6978.417073	4228.96941	4488.886117	5460.828901
CLPTM1	4319.05002	4790.20141	4092.975004	4844.45794	4868.753696	5750.768381
RELB	788.211978	859.310941	20.05868661	78.4446168	301.2742871	115.9757922
CLASRP	1125.17992	949.711264	976.8580381	985.586211	684.1646854	882.5032934
LIN7B	71.3004639	62.9755063	156.4577556	144.820831	79.60089859	127.7545835
PPP1R37	1301.96601	1389.52407	3413.988462	4559.84478	2247.96968	2757.143246
FCGRT	343.804977	332.145009	268.7864006	99.5643213	11.08366942	299.90615
PIH1D1	2601.97857	2205.15845	1853.422643	1222.93146	891.7315855	1783.127804
CKM	0	0	0	0	0	0
ARHGEF18	4371.79283	4553.5354	1558.55995	1632.25145	932.0358379	1568.391377
PPP1R13L	2193.71016	1713.54321	2223.505411	2074.75954	896.769617	2253.373399
PEX11G	22.4645297	20.3146794	139.407872	90.5130194	25.19015778	32.61819154
ERCC2	3955.71067	2977.11627	953.7905485	694.938849	1109.374549	1214.121574
DOT1L	3405.81805	3413.88188	2283.681471	2567.55265	2552.266786	2896.676621
PLEKHJ1	1282.43163	1186.37728	858.5117871	831.714078	1032.796469	846.2608584
SLC17A7	8.79046815	9.14160575	22.06455528	32.1824069	5.038031556	19.0272784
RNASEH2A	1627.21333	1792.77046	1168.418495	1612.13745	1486.219309	2238.876425
KLC3	140.64749	124.935279	6.017605984	18.1026039	142.0724899	12.68485227
CD37	0	0	2.005868661	1.00570022	0	5.436365257
SF3A2	2689.88326	2348.37694	2073.065262	1826.35159	1344.146819	1759.570222
AMH	301.806073	302.688724	378.1062427	410.325688	350.6469963	318.0273675
DKKL1	6.83703079	2.03146794	6.017605984	15.0855032	7.053244179	14.49697402

LYL1	147.484521	140.171288	0	0	0	0.906060876
OAZ1	11391.47	10856.1647	12475.50014	11994.5741	10352.14724	12079.6036
TRMT1	1171.0857	1176.21994	560.6402909	499.833007	1020.705193	831.7638843
STX10	958.161029	962.915806	892.6115543	923.232798	657.9669213	916.9336067
RETN	0	0	0	0	0	0
FCER2	0	0	0	0	0	0
DMPK	754.026824	966.978742	809.1172713	1689.57636	1419.717293	1010.90118
CLEC4M	0	0	0	0	0	0
RSPH6A	1.95343737	0	3.008802992	0	0	1.812121752
TBC1D17	934.71978	1256.46292	1047.063441	2413.68052	514.8868251	973.109381
IL4I1	27.4262606	17.3487362	1.022993017	2.02145743	12.09127574	18.17558118
TLE6	4.88359342	12.1888077	243.7130424	112.638424	12.09127574	114.1636704
CCDC130	497.14981	498.72538	941.7553365	704.995851	325.4568385	650.5517091
PTOV1	2346.78152	1949.93498	4905.863308	3831.71782	1750.212163	3989.938735
AES	4176.44909	3531.70702	10121.61327	10301.3873	3068.161218	7718.732604
NOVA2	0	1.01573397	0	0	19.14451991	8.154547886
SGTA	3239.77587	3065.48513	2973.700291	2083.81085	1845.934762	3010.840292
KIR3DX1	0	0	0	0	0	0
LILRB1	0	0	0	0	0	0
MED25	1372.56323	1124.69176	1463.772626	1197.78896	943.1195074	1432.835609
LILRA1	0	0	0	0	0	0
SNAPC2	720.818389	853.216537	509.49064	604.42583	255.9320031	584.4092651
C19orf53	1457.26428	1497.19188	1034.496674	1255.11387	1490.249734	1309.103936
TIMM44	1767.86082	1686.11839	1807.287664	1054.97953	1128.519069	1451.509524
CCDC61	238.319359	178.769179	112.328645	154.877833	164.2398287	135.9091314
IL27RA	392.640911	524.11873	22.06455528	37.210908	237.7950895	104.1970008
ASF1B	1902.648	1771.44005	790.3122526	1044.92252	2400.118233	1713.361117
TNNT1	1790.32535	1717.60615	1117.268844	1091.18473	805.0774427	1072.776077
VRK3	604.588865	712.029515	890.6056857	975.529209	511.8640061	1031.097277
FAM32A	1989.57596	1964.4295	1454.25478	1224.94286	1746.181737	1399.864054
PPP6R1	4660.04205	4772.26355	2869.134357	2644.70997	4726.721511	3929.006141
C19orf44	220.738423	241.744685	150.4401496	121.689726	115.8747258	121.4121574
MED26	434.30773	470.284829	423.2382876	358.029277	483.6510294	537.2940996

OLFM2	6.83703079	14.2202756	211.6191438	84.4788181	122.92797	243.7303757
RASAL3	0.97671868	0	1.002934331	0	1.007606311	0
AKAP8	1149.59789	1107.15003	886.5939483	903.118794	849.4121204	1254.894314
EPHX3	7.81374947	10.1573397	0	3.01710065	2.015212623	2.718182629
ILVBL	1325.40725	1683.07119	2578.544164	4295.34562	1477.150852	1829.336909
ZNF419	185.57655	197.204751	478.8209082	189.906372	204.1208865	259.5683198
SYDE1	3346.23821	2819.67751	2.005868661	0	235.7798768	59.80001783
CASP14	0	0	760.2242227	31.1767067	11.08366942	28.08788716
SLC1A6	0	0	1.002934331	1.00570022	1.007606311	0.906060876
AURKC	11.7206242	10.1573397	1.002934331	2.01140043	0	16.30909577
POP4	818.490257	984.246219	920.6937156	929.266999	1042.872532	930.5245198
CCNE1	505.940278	565.763823	120.3521197	116.661225	471.5597537	326.1819154
URI1	2112.64251	2112.72666	1402.102194	1335.56989	1815.706573	1991.521806
PDCD5	2071.62033	2193.98538	902.6408976	892.056091	1365.306552	853.5093454
ANKRD27	1907.53159	1903.48546	969.8374978	988.603312	1826.790242	1162.476104
RPS16	11544.8148	11585.4617	10040.36556	8356.36309	11078.63139	6346.929256
TIMM50	2498.44639	2458.07621	1429.181421	1103.25314	2209.680641	1545.739855
LGALS13	0	0	0	0	0	0
FBL	4374.72299	4184.82397	1549.533541	1635.26855	4187.61183	2031.388484
DYRK1B	290.085449	376.837304	970.8404321	1924.91021	318.4035944	693.1365703
CLC	0	0	0	0	0	0
CNTD2	1.95343737	3.04720192	92.26995843	47.2679101	25.19015778	51.64546994
GPI	12349.631	12448.6629	13108.17117	11158.2137	13623.84493	18670.29041
AKT2	3901.01442	3584.52519	4131.086508	3610.46377	3968.96126	4032.87696
PLD3	2796.34559	3090.87848	4908.360614	4011.73816	1334.070756	3433.06466
PRX	183.623113	185.879317	143.4196093	241.368052	127.9660015	176.6818709
PIAS4	1155.4582	1227.00664	892.6115543	1030.84272	937.0738695	897.0002674
NUMBL	558.683087	495.678178	171.5017706	120.684026	608.594212	317.1213067
EBI3	210.971236	319.956201	0	0	9.068456802	0.906060876
CCDC94	527.428089	608.424649	521.525852	738.183958	420.1718318	393.2304203
SHD	0	0	0	1.00570022	1.007606311	0
TBCB	1860.56119	1922.69299	838.4531005	1503.44137	1478.067774	931.2584291
FSD1	0	0	0	0	306.3123186	19.93333928

POLR2I	723.748545	677.494559	561.6432252	540.061016	766.7884029	780.1184144
OVOL3	0	0	5.014671654	0	0	1.812121752
CLIP3	54.6962463	30.4720192	182.5340482	193.094441	30.22818934	60.7060787
ZFR2	0	0	0	0	0	0
SLC1A5	8301.13209	7647.46108	18845.13607	8118.01214	8914.293036	9114.972414
PRKD2	879.046815	937.522456	1500.389759	1469.32801	1391.504316	2121.994572
TJP3	11.7206242	12.1888077	908.6585036	2072.74814	1605.116854	1754.133856
APLP1	183.623113	253.933493	332.9741978	499.833007	246.8635463	496.5213601
CACTIN	1409.40506	1251.38425	801.3445302	737.178258	909.8684991	1066.433651
CCDC9	657.331674	590.141438	590.7283208	578.277624	498.7651241	543.6365257
HNRNPUL1	15211.4168	13609.8195	10568.92198	10791.1633	12325.0404	10885.41537
FZR1	2091.1547	2159.45042	1606.700798	2243.71718	1048.91817	1865.579344
BBC3	197.297174	262.059365	267.7834663	345.960874	226.71142	173.0576274
TGFB1	2352.91531	3150.80678	9.026408976	6.03420129	901.8076486	204.769758
DENND3	948.393842	602.330246	13.0381463	36.2052078	166.2550414	93.32427025
ATP5SL	1318.57022	1233.10104	827.4208228	830.708378	814.1458995	847.1669192
CEACAM4	0	0	0	0	0	0
PLIN3	5242.04918	5446.36556	1991.827581	2362.38981	1690.76339	2315.8916
MYH14	1.95343737	2.03146794	2956.650407	3064.36856	5018.887037	3708.507166
MRPL4	2264.03391	2407.28951	1307.826367	1276.23357	2617.761197	1983.367258
SIGLEC8	0	0	0	0	0	0
CD79A	0	0	2.005868661	2.01140043	8.06085049	0.906060876
LIM2	0	0	0	0	0	0
ICAM4	27.3481231	37.582157	0	0	2.015212623	0
RPS19	11808.5289	11701.2554	12358.15682	11750.6013	13243.97736	9417.596747
GLTSCR2	2655.6981	2301.65318	7577.168869	4479.38876	3293.865032	2496.197714
NKG7	0	0	0	0	0	0
ICAM5	166.042176	169.627573	29.08509559	108.615623	29.22058303	44.39698293
ETFB	203.499338	206.193996	2862.364551	2419.93597	1109.807819	2125.999361
CD33	4.88359342	3.04720192	1.002934331	0	0	0
CEACAM5	0	0	0	0	293.9389131	311.3134564
CRX	0	0	0	0	0	0
BABAM1	2282.60133	2498.70557	1625.977196	1585.98924	1191.847125	1394.427688

TYK2	2921.36558	2982.19494	2217.487805	3670.80579	1933.596511	2652.946245
SULT2A1	0	0	0	0	0	0
CDC37	7601.80152	7233.04162	5712.713948	3605.43527	5597.253059	5650.195624
NAPA	1828.12436	1883.16063	7125.417158	8182.26633	2248.886602	4507.290435
RABAC1	919.092282	1181.29861	2103.153291	2197.45497	394.981674	1333.72161
ATP1A3	307.666385	355.50689	0	0	185.3995613	15.4030349
MEIS3	60.5565584	78.2115159	25.07335827	9.05130194	428.8775503	112.3515486
PTPRS	4731.2253	3665.78391	4081.942726	5310.09714	3687.839099	7235.802157
CNFN	36.1385913	52.8181666	73.21420614	115.655525	28.21297672	65.23638308
ZNRF4	0	0	0	0	0	0
MEGF8	1336.15116	1036.04865	2374.948495	2719.41338	805.0774427	2811.506899
KDELR1	5250.83964	5850.62768	5022.695128	5638.96111	4940.293744	5338.510682
CYTH2	2743.60278	2875.54288	2371.939692	3590.34977	1475.13564	2123.806694
GRWD1	2582.3856	2686.35227	1184.465445	1136.44124	2471.658282	2409.21587
GRIN2D	56.6496837	58.9125704	0	1.00570022	122.92797	36.24243505
SYNGR4	0	1.01573397	11.03227764	8.04560172	3.022818934	4.530304381
CLEC11A	0	0	225.6602244	307.744266	93.70738695	203.8636971
CCDC114	44.9290595	82.2744518	35.10270157	33.1881071	15.11409467	28.08788716
CARD8	530.358245	622.644925	866.5352617	851.828083	227.7190264	667.7668657
LIG1	2445.70358	1970.52391	1368.002427	2104.93055	2637.913323	3025.337266
SIGLEC6	0	0	0	0	0	0
ZNF175	385.80388	334.176477	547.6021446	676.836245	6.045637868	623.3698828
PLA2G4C	33.2084353	52.8181666	92.26995843	135.769529	35.2662209	116.881853
SIGLEC5	0	0	0	0	0	0
CABP5	0	0	0	0	0	0
HAS1	0	0	0	0	0	1.812121752
RAB3D	361.385913	453.088453	3238.474954	4190.7528	2556.287136	4037.389143
DBP	150.414677	116.809407	2629.693815	2413.68052	262.9852472	1004.821512
TMEM205	582.124336	713.989881	1274.729534	1396.9176	1133.567176	1590.154959
CAPS	79.1142134	84.9559894	545.5962759	522.60206	155.0303071	404.7826964
PLPPR2	1140.80742	1187.39301	1167.415561	1562.85813	1441.884631	1945.312701
FAM83E	3.90687474	1.01573397	9.026408976	7.03990151	13.09888205	24.46364366
RASIP1	5.8603121	13.2045416	5.014671654	6.03420129	53.4031345	4.530304381

THEG	0	0	0	0	4.030425245	0
FGF21	0	0	19.05575228	0	1.007606311	0
BCAT2	1061.69321	1132.54338	1216.559343	1040.89972	1102.321305	973.109381
MIER2	915.185407	969.01021	579.6960431	449.547996	440.323958	513.7365168
PLEKHA4	237.34264	275.263906	0	0	36.27382721	18.12121752
PPP2R1A	6646.57064	6701.81275	7442.775668	6828.70446	7559.062547	7415.202211
TNPO2	3880.50333	3481.93606	3656.69857	3141.80747	3452.059222	4764.974148
WDR83OS	1550.05255	1258.49439	2462.203782	1961.11542	1414.679261	2195.385503
CACNG7	3.90687474	2.03146794	0	0	0	0
GCDH	430.73294	447.938682	561.6432252	437.479594	411.103375	897.0002674
LILRB5	0	0	0	0	0	0
KLF1	0.97671868	2.03146794	0	4.02280086	4.030425245	4.530304381
DNASE2	802.862758	1044.17452	2131.235453	2415.69192	1276.637196	2047.69758
MAST1	228.552172	192.989455	418.2236159	461.616399	68.51722917	655.9880744
LENG1	219.761704	259.012163	277.8128096	539.055316	176.3311045	201.1455145
PRPF31	2844.20481	2512.92585	1736.079326	1393.9005	1718.976367	1880.076318
TFPT	542.07887	595.220108	1153.37448	1105.26454	541.0845892	934.1487633
JAK3	0	4.06293589	0	0	19.14451991	0.906060876
RPL18A	14423.3904	14501.5527	15234.52234	13371.6895	16283.24042	11417.67177
SLC5A5	0	0	1.002934331	0	27.2053704	28.99394804
KCNN1	0	3.04720192	3.008802992	0	4.030425245	6.342426133
ARRDC2	522.544496	482.473637	344.0064754	424.405491	504.810762	453.0304381
PIK3R2	3549.3957	3720.63354	7698.152837	15100.6089	3807.038926	10995.04873
RAB3A	31.2549979	61.9597723	316.9272485	262.487756	146.1029151	363.3304113
PDE4C	1.95343737	7.11013781	8.023474646	31.1767067	27.2053704	28.08788716
ISYNA1	83.9978068	74.14858	4794.026101	4812.27553	1537.607231	5374.753117
ELL	835.094475	832.901857	803.3503989	717.064254	636.8071887	968.5790766
CRTC1	689.563391	834.933325	677.9836076	720.081354	721.4461189	858.0396497
COMP	0	0	0	0	0	0
UPK1A	0	0	5.014671654	11.0627024	10.07606311	9.966669638
COPE	2619.3544	2613.48351	4177.221487	3454.58024	2979.481786	2816.037203
DDX49	978.672121	1047.22173	1635.785893	1260.14237	1746.181737	1419.797393
ETV2	9.76718684	15.2360096	26.0762926	50.2850108	8.06085049	20.83940015

ATP4A	0	0	0	0	0	0
ARMC6	2148.7811	2239.69341	874.5587364	1151.52675	1153.709226	1412.548906
TMEM147	1788.37191	1875.04491	1323.873317	1471.33942	1400.572773	1531.242881
GAPDHS	0	0	0	1.00570022	0	0
TCEB1P28	0	0	0	0	0	0
MAG	0	0	0	0	12.09127574	0.906060876
TMEM59L	0	0	14.04108063	7.03990151	11.08366942	5.436365257
HAMP	0	1.01573397	0	0	0	0
USF2	2202.50063	2497.68984	3187.325303	3841.77482	2387.019351	2777.982646
LSR	1370.33631	1187.39301	1694.959019	2463.96553	3534.68294	3916.901168
KXD1	1727.81535	1772.45578	1065.116259	1017.76862	1793.539234	1314.694331
FKBP8	5340.69776	5027.88316	6087.811387	4895.74865	4856.66242	5191.728821
SUGP1	831.1876	853.216537	1074.142668	822.662776	701.2939927	968.5790766
HPN	0	0	0	0	70.53244179	20.83940015
ZNF14	134.787178	142.202756	328.9624605	224.271148	250.8939715	312.5910023
SCN1B	44.9290595	67.0384422	27.07922693	10.0570022	493.7270925	47.11516556
PBX4	136.740616	132.045416	0	1.00570022	42.31946507	10.87273051
ERF	1823.53378	1661.74078	1591.385991	2240.70008	1612.492532	2254.27946
GSK3A	2841.27465	2922.26664	1928.92354	2147.16996	2106.340537	2369.349191
ATP13A1	2203.47735	2317.90492	2249.581704	2371.44111	2645.974173	3542.698026
ZNF574	806.769633	805.47704	666.9513299	940.329702	810.1154743	843.5426757
GRIK5	0	1.01573397	5.014671654	11.0627024	10.07606311	24.46364366
SIPA1L3	5196.1434	4862.31853	5738.79024	5766.68504	1779.432746	7664.368952
ZNF85	205.110924	210.256932	183.5369825	169.269403	286.1601924	287.2212977
ETHE1	420.965753	522.087262	497.455428	583.306125	129.9812142	337.0546459
CADM4	231.482328	214.319868	263.771729	189.071641	345.6089648	281.7849325
SMG9	1435.77647	1527.66389	722.1127181	948.375303	1345.154426	931.4305807
AVL9	1442.6135	1400.69715	2205.452593	2338.253	4025.387214	2177.264285
RUNDC3B	14.6507803	11.1730737	25.07335827	30.1710065	83.63132384	12.68485227
CFAP69	58.603121	28.4405512	18.05281795	0	39.29664614	17.21515665
GTPBP10	655.378237	594.204374	556.6285535	434.462493	763.765584	575.982899
RASA4	61.3379333	83.3511298	22.04449659	10.7509353	188.7750424	99.05057498
CDK6	4745.87608	4041.60548	71.20833748	128.729628	1484.204097	331.6182807

PMPCB	1512.93724	1468.75132	873.555802	713.041453	1630.307012	677.7335354
DNAJC2	595.798397	563.732355	374.0945054	324.84117	2084.737458	693.1365703
TFPI2	604.588865	1343.81605	0	0	338.5557206	85.16972236
BET1	375.059975	370.7429	349.0211471	218.236947	284.1449798	217.4546103
NAMPT	8448.08919	9801.27418	904.7871771	1418.47981	3384.972794	1596.062476
TWISTNB	1060.71649	936.506722	603.7664671	444.519495	525.9704945	569.9122911
PIK3CG	12.6973429	19.2989455	0	0	13.09888205	4.530304381
PON3	13.6740616	11.1730737	1.002934331	0	5.038031556	4.530304381
PON2	361.385913	511.929922	1156.383283	1105.26454	712.3776621	1536.679246
ITGB8	58.603121	65.0069742	3.008802992	2.01140043	20.15212623	9.966669638
HBP1	685.656516	637.880935	821.4032169	922.227098	725.4765441	603.4365435
DUS4L	168.972332	177.753445	122.3579883	64.3648138	404.0501308	120.5060965
SP4	275.434669	273.232439	205.6015378	290.647362	202.5288686	331.6182807
WDR91	241.249515	262.059365	271.7952036	329.869671	342.5861458	478.4001426
DNAH11	167.995614	117.825141	13.0381463	19.1083041	26.19776409	49.83334819
CBLL1	868.30291	836.964793	496.4524937	499.833007	1612.170098	607.060787
DLX5	0	0	0	0	0	0
MTPN	7361.32361	7303.26946	3180.174381	3185.66606	8249.575152	4166.38503
STEAP1B	0.99625306	0	0	0	0	0
PTN	39.0687474	51.8024326	0	0	9.068456802	0.906060876
MPP6	905.41822	901.971767	517.5141146	723.098455	444.3543833	462.0910469
DFNA5	1238.47929	1237.16398	2.005868661	4.02280086	82.62371753	57.98789608
ATP6V0A4	292.038886	317.924733	0	0	83.63132384	14.49697402
ZC3HAV1	2185.89641	2618.56218	2630.696749	2845.12591	3514.530814	4821.149922
TTC26	565.520118	621.629191	253.7423857	140.79803	461.4836906	278.160689
OGDH	3795.52881	3928.859	2052.003641	3232.32049	3428.884277	3459.340425
NPVF	0	0	0	0	0	0
ADAP1	53.5534854	57.8968364	2.417071737	3.01710065	1008.795287	103.2909399
TFEC	0	0	0	0	1.007606311	2.718182629
H2AFV	5404.18448	5126.40936	3129.155112	3847.80902	3420.823427	3101.446379
CAV2	2612.72248	1876.06065	0	0	838.328451	123.2242792
CAV1	15220.2073	10107.5688	0	0	1950.725819	434.0031597
MET	6852.65829	5764.29029	0	0	2020.250654	380.545568

RNF32	52.7428089	65.0069742	57.16725685	42.2394091	343.5937521	28.99394804
LMBR1	2173.19907	1991.85432	1685.93261	1631.24575	9245.795512	2575.02501
NHP2P1	0.97671868	2.03146794	2.005868661	0	4.030425245	3.624243505
WNT2	0	1.01573397	0	0	0	0
HOXA1	0	0	0	0	26.19776409	5.436365257
DNAJB6	7634.55089	7056.95397	2365.791705	3745.94165	5687.504357	2863.877217
HOXA2	0	0	0	0	1.007606311	0.906060876
HOXA3	0	0	0	0	11.08366942	2.718182629
LFNG	327.200759	445.907214	333.9771321	405.297187	1090.230029	429.4728553
HOXA5	0	0	0	0	88.66935539	8.154547886
HOXA6	0	0	0	0	24.18255147	0.906060876
BRAT1	1300.98929	1244.27412	1340.9232	1288.30198	1991.030071	1768.63083
IQCE	655.378237	719.139652	1513.427905	1481.39642	2477.703919	2047.69758
ANKRD7	5.8603121	6.09440383	0	0	3.022818934	0.906060876
VIPR2	0	0	0	0	5.038031556	1.812121752
TSPAN12	116.229523	185.879317	6.017605984	11.0627024	28.21297672	9.966669638
SSBP1	2256.22016	2277.27557	1630.771222	1236.00556	2625.822047	1422.515576
HOXA13	21.487811	24.3776153	0	1.00570022	21.15973254	8.154547886
CPED1	222.69186	210.256932	0	0	25.19015778	9.966669638
EVX1	0	0	0	7.03990151	4.030425245	4.530304381
HIBADH	1426.00928	1214.81783	1038.037032	716.058553	867.549034	845.3547975
TAX1BP1	2980.94542	2887.73168	2877.418595	2785.7896	2543.19833	2459.049218
CPVL	171.902488	292.531384	3.008802992	4.02280086	1.007606311	9.966669638
CHN2	22.4645297	30.4720192	283.8304156	142.809431	4.030425245	173.9636882
GRB10	1589.1213	1693.22853	986.8873814	431.445392	367.7763036	845.3547975
ABHD11	407.291691	420.513865	2351.881006	1955.08122	1153.709226	2070.349102
COBL	202.180768	137.124086	1005.943134	881.999089	807.0926553	725.7547618
FKBP14	807.746352	1186.37728	742.1714047	569.226322	252.9091841	1009.351816
PLEKHA8	1038.97473	1094.4432	1200.121249	1091.46633	797.6916885	1380.093805
STX1A	512.777309	577.95263	297.8714962	249.413653	271.0460977	399.5728464
NOD1	217.808267	206.193996	526.5405236	206.168544	159.2017972	270.0061411
GARS	5695.24665	4579.94448	10447.56692	2857.19431	4532.213188	4722.389287
CRHR2	0	0	0	0	0	0

EPHB6	0.97671868	2.03146794	2.005868661	0	6.045637868	11.77879139
FAM188B	160.181864	150.328628	63.18486283	47.1472261	83.63132384	111.4454878
GHRHR	0	0	0	0	0	0
NSUN5P2	141.331194	141.085449	292.8267365	256.674809	176.2908002	286.2065096
CASP2	1341.03475	1260.52586	1271.720731	1661.41676	1633.329831	2112.933963
CHCHD2	4280.95799	4259.98828	11699.18885	9728.12813	5838.070968	7106.860634
CCL24	0	1.01573397	0	0	3.022818934	0.906060876
HSPB1	3583.58085	3570.30491	3064.967315	6001.01319	35585.51118	8402.808566
NPTX2	0	0	0	0	1.007606311	0
PDAP1	3060.04987	2836.93483	1340.913171	909.152995	4847.281606	1649.891552
BUD31	1058.76305	1064.4892	932.7289276	571.237722	1830.820668	769.2456839
PTCD1	242.509482	308.376834	212.8427237	219.564471	595.0922874	311.4312444
CYP3A5	10.7439055	10.1573397	0	0	6.045637868	0.906060876
ZKSCAN1	1579.35411	1401.71288	11712.26711	7882.67829	3926.641795	8210.72366
EIF3B	9459.52045	8629.67583	4619.515527	4096.21698	15511.09156	7642.623491
SNX8	1857.71894	1752.1411	630.845694	822.662776	1321.97948	566.2880476
NUDT1	282.2717	261.043631	226.6631587	362.052078	536.0465576	355.1758635
PTPRZ1	0	2.03146794	102.2993017	27.1539058	0	4.530304381
TAF6	2158.54829	1948.17776	1665.873923	1758.96968	2843.46501	2081.221833
WASL	1585.21442	1485.00307	1914.601637	1891.72211	2872.685593	1784.033865
HYAL4	2.93015605	9.14160575	0	0	0	0
SPAM1	0	0	0	0	0	0.906060876
AIMP2	1161.31852	1234.11678	771.2565003	616.494232	1730.060036	834.482067
TFR2	5.8603121	4.06293589	53.15551953	86.4902185	12.09127574	122.3182183
FSCN3	1.95343737	0	1.002934331	1.00570022	1.007606311	4.077273943
MOSPD3	344.781695	320.971935	111.3257107	171.974737	237.7950895	195.7091493
PAX4	0	0	0	0	0	0
PCOLCE	59.5798397	75.1643139	84.24648378	141.80373	93.70738695	146.7818619
FBXO24	3.90687474	1.01573397	0	8.04560172	17.12930729	9.966669638
PPP1R17	0	0	0	0	0	0
RBM28	1321.50038	1441.32651	1967.757157	1699.63336	2450.498549	1900.915718
USP42	576.264023	583.0313	609.7840731	626.551234	1086.199604	772.8699274
IMPDH1	1615.4927	1640.41037	2821.254272	3941.32909	3648.542453	2579.555314

AGFG2	740.352762	622.644925	215.6308811	264.499157	603.5561805	410.4455769
LSM5	948.393842	844.074931	1008.951937	955.415205	1476.143246	1047.406373
SERPINE1	14050.0983	13766.2425	0	0	1621.238555	800.9578145
AP1S1	1065.60008	1167.07833	774.2653033	678.847645	2658.065449	934.1487633
MOGAT3	0	0	0	0	0	0
C1GALT1	1706.32754	1481.95587	103.3022361	187.06024	517.909644	222.8909755
PLOD3	2078.45736	2117.80533	723.1156524	747.23526	4491.908936	1227.712487
RPA3	610.449177	696.793505	727.1273898	928.261299	806.085049	687.700205
ZNHIT1	704.214171	649.054008	846.4765751	609.454331	1394.527135	790.085084
CLDN15	158.228427	138.13982	67.19660016	80.4560172	121.9203637	105.1030616
NOBOX	0	0	0	2.01140043	0	0
GLCCI1	133.156058	149.069118	194.5692602	201.522209	840.8474668	329.5796437
MYL10	0.97671868	0	0	0	0	0
PHF14	965.974778	1070.58361	823.4090855	1010.72872	1045.895351	1073.682138
NRF1	546.962463	510.914188	536.4394855	450.553697	519.1489998	476.5880209
TMEM106B	811.653226	819.697316	805.3562676	976.534909	720.4385126	684.0759615
EZH2	2182.96626	1859.8089	1060.101588	1044.92252	2111.942828	1339.157975
CEP41	641.704175	607.408915	374.0945054	255.447855	608.594212	337.0546459
ZNF862	319.38701	295.578586	1007.949002	730.138356	458.4608716	597.0941174
SFRP4	0	0	0	0	0	0
MEST	519.61434	426.608268	1678.91207	1408.986	2975.461437	1811.215691
MEOX2	0	0	0	0	0	0
ANKMY2	456.127625	439.81281	627.836891	693.933149	233.7646642	580.7850216
ACTR3C	42.0965753	47.2214724	112.1079995	137.650189	146.1734476	124.6830372
POU6F2	0	0	0	0	0	1.812121752
TSPAN13	39.0687474	76.1800479	5187.176358	5308.08574	2706.430552	4404.361919
RARRES2	65.4401518	48.7552307	5.014671654	0	0	0.906060876
AC004837.3	0	1.01573397	0	0	1.007606311	2.718182629
AGR2	0	0	5482.039052	5459.94647	2381.98132	821.7972147
AHR	3175.31244	3398.64587	111.3257107	113.644124	1161.770077	501.0516645
CHCHD3	2417.64246	2726.49407	1425.269977	1519.83428	3061.541244	1598.463537
GIMAP2	0.97671868	0	7.020540315	4.02280086	4.030425245	9.966669638
TMEM176B	0	0	0	0	3.022818934	0.906060876

GLI3	795.049009	902.987501	311.9125769	731.144057	437.3011391	1344.59434
PSMA2	2065.14468	2125.96168	2176.036529	2201.63868	2713.75585	2054.937007
MRPL32	693.470266	593.18864	703.0569658	618.505633	974.355303	720.3183966
COA1	1508.05365	1443.35797	1093.19842	863.896485	1084.184391	1079.118504
BLVRA	718.864951	672.41589	1018.98128	943.346802	622.7007004	568.1001694
URGCP	1147.3026	1105.74832	1186.441225	1133.94711	1214.770169	1200.403812
TMEM248	3276.89118	3435.21229	2471.230191	2233.66018	3052.039517	2792.47962
STAG3L4	240.272796	256.980695	421.2324189	373.11478	374.8295478	329.8061589
RHEB	4394.18899	4081.15816	1922.574965	2287.96799	2096.828734	1601.888447
PRKAG2	1644.79426	1427.10623	318.9331172	378.143281	480.6282105	314.403124
AEBP1	1.95343737	2.03146794	1.002934331	31.1767067	31.23579565	9.060608762
POLD2	3167.49869	3205.65642	1707.997165	2527.32464	3142.724085	3625.149566
MYL7	0	0	0	0	11.08366942	0.906060876
GCK	0	0	0	0	6.045637868	0.906060876
BCL7B	1111.50586	1077.69374	771.2565003	1008.71732	1270.591559	1009.351816
YKT6	2253.29	2494.64264	2391.998379	1981.22942	3545.766609	2673.785646
TBL2	1121.27305	1220.91223	1043.051704	728.126956	1013.651949	1111.736695
GALNTL5	0	0	0	0	0	0
CLIP2	1137.87727	1385.46114	61.17899417	76.4332164	77.58568597	70.67274834
EIF4H	14907.335	12738.1166	10077.32369	7923.61029	12570.41269	10674.00418
LIMK1	3273.96103	3609.91854	832.4354945	695.944549	1721.999186	1497.718628
SPATA6L	0.97671868	0	64.18779717	30.1710065	10.07606311	38.96061768
SLC1A1	41.0221847	51.8024326	16.04694929	11.0627024	55.41834712	54.36365257
LHX2	0	0	38.11150457	45.2565097	137.0344583	114.1636704
FKTN	1253.13007	1275.76187	684.0012135	545.089517	1674.641689	641.4911003
FSD1L	239.296078	202.13106	59.17312551	60.3420129	141.0648836	43.49092206
CNTNAP3	313.077407	234.563446	146.2980308	105.145958	22.16733885	84.74387375
SPIN1	2987.78245	2598.2475	1560.565819	1665.43956	3744.265053	1247.645826
NMRK1	42.9756221	50.7866986	525.5375893	429.433992	206.5592938	218.3606712
TMEM245	5207.86402	4567.75567	1413.134472	1921.89311	4331.699532	2099.34305
PRUNE2	0	0	0	2.01140043	3.022818934	3.624243505
MEGF9	941.556811	1182.31434	1488.354547	2343.2815	3168.921849	2270.588556
TRIM14	1389.87069	1328.58004	814.3826765	463.627799	1933.596511	1131.670034

CORO2A	875.139941	1018.78117	1016.975411	473.684802	854.450152	615.2153349
TGFBR1	2659.60498	3078.68967	399.1678636	794.50317	1332.055544	394.1364811
SEC61B	1283.40835	1480.94013	2254.596375	1028.83132	2143.178624	755.6547707
C5	155.298271	120.872343	160.4694929	209.185645	219.6581759	223.7970364
OGN	0	0	0	0	0	0
ASPN	0	0	0	0	0	0
ECM2	0.97671868	2.03146794	1.002934331	1.00570022	0	0
TLE4	0	0	213.6149831	322.548173	371.8067289	562.989986
LHX6	32.2317166	32.5034871	0	0	69.52483548	4.530304381
PTGR1	1133.97039	1069.56787	672.9689359	609.454331	434.2783202	657.8001961
SUSD1	621.193083	471.300563	7.020540315	7.03990151	575.3432037	54.36365257
AMBP	0	0	177.5193765	355.012176	3.022818934	192.9909666
AKNA	377.990131	483.489371	259.7599917	299.698664	500.7803367	242.8243148
TNFSF8	0	0	0	0	0	0
DNM1	501.056685	469.269095	44.12911055	44.2508095	812.1306869	111.4454878
ENG	5.8603121	11.1730737	9.026408976	0	83.63132384	57.98789608
AK1	848.465753	821.728784	698.1526169	645.941134	548.7625493	839.8278261
CDC37L1	139.670772	189.942253	302.8861679	300.704364	431.2555012	265.4758367
RLN2	2.93015605	0	28.04204389	68.3876147	28.21297672	14.49697402
RLN1	0.97671868	1.01573397	4.051854696	17.0969037	5.038031556	6.342426133
PLGRKT	193.390299	204.162528	481.4084787	565.203521	91.69217433	230.1394625
TBC1D13	1172.06242	1281.85627	705.0628345	693.933149	1191.998266	905.1548153
RIC1	847.791818	590.141438	854.5000498	955.415205	1053.956202	903.3426935
KDM4C	141.624209	189.942253	842.4648378	817.443192	655.9517086	539.1062213
DOCK8	0	2.03146794	0	0	943.1195074	187.5546014
KANK1	128.926866	167.596105	293.8697882	420.38269	519.9248566	306.2485761
ELAVL2	0	0	131.3843973	193.094441	0	99.66669638
NCS1	1668.23551	1547.97857	353.0328844	314.784167	2109.927616	564.4759259
TESK1	683.703079	782.115159	2541.435594	3213.21219	1058.994233	1872.827831
KCNT1	0	0	0	0	2.015212623	0.906060876
CA9	41.9989034	27.4248173	150.4401496	0	18.1369136	4.530304381
FUBP3	2436.91312	2310.79479	1996.842252	1451.22541	3398.656088	2038.636971
TYRP1	0	1.01573397	0	0	40.30425245	8.154547886

CREB3	1010.90384	8148.79512	1176.367498	9380.49241	1549.165145	1687.085351
RGP1	2124.36314	2251.88222	3664.722044	4342.61353	1869.109707	4282.949762
MPDZ	1204.29414	1148.79512	451.3204488	302.715765	836.3132384	479.3062035
LHX3	8.79046815	3.04720192	0	2.01140043	1.007606311	6.342426133
DDX58	2043.29549	2343.29827	310.9096425	611.465731	211.5973254	2502.54014
EDF1	2778.76466	2708.9625	7342.482235	4341.60783	4887.898216	4805.746887
PIP5K1B	0.97671868	1.01573397	150.4401496	89.5073192	38.28903983	91.51214849
GLIS3	518.637621	498.72538	0	0	51.38792188	12.68485227
BAG1	1066.5768	866.421078	1672.894464	2532.35314	2073.653789	2142.833972
RAPGEF1	3881.48005	3589.60386	1432.190224	1552.80113	3279.758543	2295.95826
NPDC1	489.336061	517.008592	6375.65354	6979.5595	2757.788246	6584.344387
APBA1	59.9119241	85.240395	17.78202568	14.079803	105.7785106	9.060608762
SETX	3508.37351	3358.01651	1443.222502	2067.71964	3626.375114	3090.573649
SH3GL2	0	0	0	0	0	0
PTGDS	0	0	0	0	0	0
ABCA2	2055.01611	1457.57825	4507.186882	4268.19171	7104.632101	7298.320358
SHB	1882.1369	1639.39463	2806.210257	3477.42975	1013.651949	2516.620326
UBE2R2	2047.20236	1957.31936	3972.622884	3110.63077	1902.360716	2967.349369
ABHD17B	467.84825	487.552307	233.6836991	313.778467	392.9664614	209.3000624
EXOSC3	1159.35531	1191.45595	743.1743391	779.387496	978.3857283	1001.142905
ZFAND5	3807.89406	3631.34037	3419.283955	3483.15218	2567.884684	2434.81209
DVL1	2894.01746	2725.21425	2186.396841	1853.5055	1961.809488	2185.418833
PDLIM1	2575.60717	1810.03794	0	1.00570022	2326.562973	328.9000981
CCNJ	437.56997	395.120515	265.7775976	174.991838	513.8792188	341.5849503
DNTT	0	0	0	0	0	0
GATA3	269.574357	200.099593	15414.09773	18961.4719	26958.50686	19816.45742
ATRNL1	228.552172	218.382804	0	0	150.1333404	22.6515219
HPS1	861.465879	1153.87379	1206.53	1548.77833	682.1494727	595.2819956
PHYH	508.870434	619.597723	249.7306483	137.78093	415.1338003	197.521271
RASSF4	499.103247	409.340791	64.18779717	34.1938073	22.16733885	58.89395695
DNMBP	6874.1461	6536.24811	273.8010723	487.764605	1361.276127	660.5183787
RAB11FIP2	879.046815	863.373876	374.0945054	415.354189	341.5785395	334.3364633
CXCL12	0	0	0	0	327.4720512	7.248487009

ERLIN1	3589.44116	3826.26987	789.3093183	888.03329	1245.401401	1324.661001
EIF3A	16058.2319	14633.6793	9583.03753	10427.0998	9040.243825	9730.187749
PKD2L1	0	0	0	1.00570022	0	2.718182629
CUBN	50.7893716	59.9283044	3.008802992	0	2.015212623	1.812121752
TRDMT1	444.407001	406.293589	59.17312551	24.1368052	65.49441023	38.96061768
DDX50	1732.64034	1621.10126	757.0950676	844.727839	949.0543085	889.6792955
MAPK8	1803.99941	1471.79853	622.8222194	568.220622	626.7311256	607.060787
SEC23IP	2349.00843	2437.76153	2999.776583	1763.99818	2691.316457	3648.707148
ATE1	1277.54804	1209.73916	817.3914795	926.249899	742.6058514	834.482067
NSMCE4A	1157.41164	970.025943	946.7700082	924.238498	671.0658033	915.1214849
PLEKHA1	639.750738	577.95263	629.8427597	750.252361	896.769617	715.7880922
PALD1	0	0	4.011737323	0	18.1369136	1.812121752
UNC5B	12.6973429	28.4405512	6774.821404	1761.98678	210.5897191	879.7851108
CDH23	0.97671868	0	2.005868661	0	0	0.906060876
C10orf54	481.522311	276.27964	28.08216126	7.03990151	1.007606311	28.99394804
SPOCK2	1.95343737	8.12587178	16.04694929	27.1539058	17.12930729	25.36970453
MICU1	1483.63568	1529.69536	1271.720731	1491.45342	978.3857283	993.0427203
PPP3CB	2108.73564	1931.92602	1674.900332	1510.56172	1265.553527	1382.648897
CCSER2	2551.1892	2225.47313	1006.946068	2058.66834	1094.260454	1145.260947
BMPR1A	1559.80997	1385.64397	419.8784576	402.280086	1214.951538	482.97575
MINPP1	1021.64774	1177.23567	647.8955776	676.836245	568.2899596	825.4214582
ACTA2	30.2782792	30.4720192	0	0	273.0613104	20.83940015
LIPA	2133.15361	2692.71076	601.7605984	1581.96644	1535.592018	1061.903347
TLX1	0	0	22.06455528	7.03990151	1.007606311	9.060608762
C10orf2	833.141037	673.431624	226.6631587	132.752428	862.5110025	540.0122822
LZTS2	1772.74441	1715.57468	2008.877464	1533.69283	1354.222882	1840.20964
SFXN3	1781.53488	2272.1969	1870.472527	2902.45082	267.0156725	1233.148852
KAZALD1	202.180768	250.886291	780.2829093	1212.87446	163.2322224	777.4002318
FBXW4	697.37714	717.108184	1421.157947	1279.25067	1017.682374	901.5305718
FGF8	2.93015605	0	0	0	0	0
NPM3	920.069	906.034703	288.8450872	285.618861	804.0698364	598.0001783
TNKS2	2546.30561	2426.58846	2773.113424	3516.93365	2058.539694	3519.140443
PITX3	6.83703079	2.03146794	0	0	13.09888205	3.624243505

GBF1	4307.3294	4436.72599	4168.195078	3737.182	3338.199709	4678.898365
ARHGAP21	6016.80197	5249.81088	1567.365713	2563.12757	2273.925619	1281.63217
CPEB3	81.0676508	72.117112	412.2060099	289.641662	102.7758438	306.2485761
FBXL15	203.157486	260.027897	440.2881712	453.570797	430.2478949	396.8546638
CUEDC2	799.932602	737.422864	1059.098653	968.489308	486.6738484	927.8063372
SUFU	780.398228	913.144841	1244.641504	965.472207	611.617031	1635.439882
ANKRD26	827.280725	895.877364	760.2242227	635.602536	714.3928747	600.7183609
ACBD5	1256.06023	1379.36673	1070.130931	1365.74089	1794.54684	1083.648808
LHPP	132.833741	115.793673	392.1473233	363.057778	196.4832307	338.8667677
LARP4B	5044.752	4003.00758	3225.436808	3021.12345	5835.048149	3780.992036
GTPBP4	4279.98127	3946.12648	1645.815237	1181.69775	5026.947887	2551.467427
EDRF1	769.654323	897.908831	565.6549625	568.220622	823.2143563	819.0790321
BCCIP	2696.72029	2434.71433	1714.014771	1375.79789	1887.246621	1957.091493
MTPAP	1000.15993	993.387825	541.5845386	467.6506	1002.56828	738.4396141
NEURL1	4.88359342	15.2360096	7.020540315	6.03420129	57.43355974	40.77273943
SH3PXD2A	3167.49869	3470.76298	4.011737323	3.01710065	150.1333404	52.55153082
PITRM1	3650.97444	3668.83111	1829.352219	2485.08523	5400.769829	2488.043166
OBFC1	419.989034	462.158957	567.6608312	981.56341	400.0197056	436.7213423
MAP3K8	187.529987	245.807621	29.08509559	27.1539058	228.7266327	77.01517448
DKK1	8737.72535	4567.75567	0	0	1712.930729	43.49092206
EBF3	0	2.03146794	0	0	5.038031556	0
GLRX3	2643.97748	2878.59008	1231.603358	1306.40458	2275.175051	1454.227706
SORCS1	0	0	0	0	4.030425245	0.906060876
FAM208B	4832.80405	4434.69452	2239.55236	1921.89311	8135.413357	3893.343585
XPNPEP1	1669.21223	1557.12018	1159.392086	918.204297	1112.397368	1171.536713
SMC3	6960.91778	6326.92565	2885.401952	2892.15245	2923.065909	3038.891936
SHOC2	1351.77866	1324.5171	1347.94374	1311.43308	1137.587525	2364.818887
TFAM	3760.36693	3338.71757	2166.338154	2192.42647	1570.858239	2642.979576
CCDC6	2559.00295	2669.34888	3208.386924	7691.59525	4214.8172	6497.362543
CUL2	2045.24892	2119.8368	1181.456642	1232.98846	1998.083315	1468.72468
CCNY	2546.30561	2357.51855	1151.368612	1209.85736	2055.516875	1321.942818
UBE2S	9032.2451	9251.94493	2032.636979	3324.59349	4776.910381	2627.984268
RPL28	12937.6157	12660.1082	8570.073856	9704.00138	9303.229072	8560.463158

ZMIZ1	5921.84538	5702.33052	3849.261961	5550.45949	7242.674166	6759.214136
DNAJC12	0	1.01573397	101.2963674	106.604223	250.8939715	124.13034
PPIF	5513.57697	6303.64503	1693.956085	1430.10571	3020.803721	2948.322091
PBLD	29.3015605	35.550689	385.126783	338.920973	232.7570579	316.2152458
TSPAN14	6213.88427	5162.97578	2484.268337	2808.9207	2383.996533	2519.755297
LGI1	0	0	0	0	2.015212623	0.906060876
TBC1D12	1007.97368	785.162361	484.4172817	583.306125	530.0009197	520.9850038
CYP2C18	0.97671868	0	0	1.00570022	0	1.812121752
KRT23	0	0	168.4929676	205.162844	0	43.49092206
CRYBA1	0	0	0	0	0	0
NUFIP2	4938.28967	4679.48641	4161.174538	3634.60058	4923.164437	5921.107826
GIT1	2390.03062	2472.29649	1604.694929	1857.5283	2550.251574	2068.53698
RPL19	19721.8939	20076.9875	16332.78558	14607.7956	11552.20636	12974.78269
FBXL20	652.448081	580.999832	618.810482	622.528433	303.2894997	710.3517269
RUNDC3A	1.95343737	3.04720192	0	1.00570022	20.15212623	3.624243505
UBTF	3146.9876	2940.54985	2319.787107	2196.44927	4909.057949	2717.276568
CSF3	1392.80084	4492.59136	0	0	69.52483548	54.36365257
PSMD3	4617.92594	4721.1315	7517.995743	6562.19391	3840.995259	7664.368952
CASC3	3801.38912	3501.235	1103.227764	934.2955	1860.041251	1215.933696
RAPGEFL1	185.57655	148.29716	74.21714047	87.4959188	1032.796469	137.7212532
RGS9	60.5565584	61.9597723	0	0	3.022818934	3.624243505
RNF43	33.2084353	16.2517436	493.4436907	756.286562	175.3234982	863.476015
WNT3	351.618726	318.940467	10.02934331	15.0855032	52.39552819	24.46364366
ASPA	0	0	0	0	0	0
RAD51C	466.871531	465.206159	217.6367498	182.031739	4387.117879	367.8607157
SEPT4	52.7428089	40.6293589	0	1.00570022	21.15973254	4.530304381
MTMR4	2443.75015	2473.31222	761.227157	896.078892	4132.193483	1186.939748
TRIM37	2644.9542	2438.77727	1702.982494	2106.94195	28403.41431	3170.307006
P2RX1	1.95343737	0	0	0	0	0
DHX40	1359.89519	1465.41972	2087.407223	2011.00821	4295.667531	2671.882918
KRT37	0	0	0	0	1.007606311	0
TUBD1	256.877014	275.263906	549.6080132	503.855808	3478.256987	816.3608494
KPNB1	21582.5528	20068.8718	9170.83152	10108.2929	16386.70144	15606.89859

GOSR2	770.631042	718.123918	647.3339344	445.967704	1261.523102	774.2652611
PNPO	797.979165	762.816213	1664.870989	1938.99002	2438.407273	2516.131053
TVP23BP2	0	0	0	0	0	0
RPS6KB1	1411.3585	1309.18967	3197.3045	2098.77567	22863.52428	4364.449938
TRIM16L	934.71978	1581.77204	2725.273457	1647.74929	1443.62779	1724.614393
ZNF29P	0	0	0	0	0	0
CDK5RAP3	2137.06048	2073.11304	4300.58241	3412.34083	2317.494516	3238.252511
CBX1	3775.01771	3375.28399	4190.259634	4006.70966	6396.284864	5352.101596
RECQL5	556.72965	607.408915	969.8374978	707.007252	1249.431826	880.6911716
PIGL	154.321552	149.312894	209.6132751	172.980437	239.8103021	208.3940015
GALK1	629.006832	650.069742	395.1561263	437.479594	1224.241668	603.4365435
INTS2	1199.41054	1235.13251	761.227157	961.449406	3141.716479	1308.351905
CAMTA2	1431.86959	1454.53105	1082.166143	1319.47868	1179.906991	1297.479175
MED13	3815.06318	3333.6389	4091.972069	4965.14196	13274.20554	5706.371398
HOXB6	6.83703079	7.11013781	0	0	17.12930729	2.718182629
ENO3	227.575453	228.540144	90.26408976	137.78093	91.69217433	152.2182272
AC003958.6	0	0	0	0	0	0
PFN1	29725.4564	28008.8643	5245.34655	6980.5652	22861.5796	9426.648295
RNF167	1487.54256	1804.95927	891.60862	1075.09353	2051.48645	1488.65802
SLC25A11	1234.57242	1157.93673	900.635029	933.2898	1876.162952	1060.091225
RASD1	39.0687474	26.4090833	40.11737323	9.05130194	53.4031345	113.2576095
CHRNE	1.95343737	2.03146794	4.011737323	4.02280086	5.038031556	8.154547886
RAI1	2628.34998	2251.88222	4328.664571	5040.56948	4540.274039	6414.911003
NUP88	2550.21248	2325.01506	760.2242227	585.317525	2395.080202	929.618459
C1QBP	5744.08258	5571.30084	1314.846908	1181.69775	7506.667019	2460.86134
SLC6A4	0.97671868	1.01573397	0	0	0	1.812121752
BLMH	1624.28317	1766.36138	157.4606899	195.105842	601.5409678	370.5788984
CPD	10090.4807	10411.2732	8552.021038	6443.52128	1717.968761	6500.986787
GOSR1	1356.66225	1326.54857	2121.206109	1515.59022	987.4541851	1740.542943
CCDC47	4124.683	3998.94465	8655.323274	7406.98209	9766.727975	11143.64272
MED31	303.759511	360.58556	92.26995843	102.581422	124.9431826	90.60608762
DRG2	1081.22758	1025.89131	1019.984214	1017.76862	1227.264487	1003.915451
FTSJ3	3096.19823	3203.62495	3850.264896	3994.64126	6452.710817	6471.086778

AKAP10	1172.06242	1168.09407	863.5264587	969.495008	781.9024976	893.3760239
ALDH3A1	1.95343737	7.11013781	2.005868661	0	75.57047335	25.36970453
SMARCD2	2094.08486	2080.22318	14345.97267	14317.1483	14050.0624	16234.79878
ICAM2	222.69186	245.807621	14.04108063	2.01140043	24.18255147	13.59091314
SYNGR2	3845.34146	3683.05138	7062.663557	6920.22318	5723.203848	6054.298775
B9D1	159.205145	183.847849	333.9771321	225.276848	198.4984433	220.1727929
UTP6	1721.95504	1734.87362	1173.433167	892.056091	904.8304675	1354.56101
DDX5	16802.4915	16926.1909	38490.61374	31278.2824	26467.80258	36059.41075
C17orf75	483.475749	474.347765	618.810482	494.804506	249.8863652	436.7213423
CYTH1	1177.92273	1262.55733	695.0334912	894.067492	949.1651452	877.972989
PSMD11	5446.18338	5129.45656	2423.089343	2638.95737	3682.801068	3855.289028
LGALS3BP	12359.3982	12771.839	116.3403824	27.1539058	929.013019	1777.691439
ASIC2	0	0	0	0	0	0
CCL7	0	0	0	0	0	0
CCL2	36.1385913	30.4720192	2.005868661	2.01140043	18.1369136	4.530304381
CCL8	0	0	0	0	0	0
CCL1	0	0	0	0	0	0
PEX12	212.924673	267.138035	174.5105735	186.05454	91.69217433	162.1848968
KRT32	0.97671868	1.01573397	2.005868661	8.04560172	1.007606311	4.530304381
DHX58	110.369211	114.777939	161.4724272	119.678326	4.030425245	312.5910023
KAT2A	2360.72906	2447.91887	626.8339567	421.38839	1935.611724	1164.288226
RAB5C	4580.81063	4662.21893	3203.372252	3427.42633	4617.859725	3795.48901
NAGLU	847.791818	761.191039	453.3263175	908.147295	837.3208447	563.569865
HSD17B1P1	0	1.01573397	0	1.00570022	0	2.718182629
HSD17B1	20.5110924	27.4248173	45.13204488	52.2964112	24.18255147	14.49697402
MLX	2036.45846	2147.26162	1014.969543	1088.16763	1795.554447	1126.233669
CNTNAP1	1395.731	1460.62545	23.06748961	13.0741028	84.63893015	28.08788716
ABI3	2.93015605	0	0	3.01710065	1.007606311	2.718182629
EZH1	924.952594	772.973553	464.3585951	427.422592	479.6206042	385.0758724
DLX4	58.603121	56.8811024	79.23181213	161.917735	66.50201655	198.4273319
PPP1R9B	3344.28477	3667.81537	5741.799043	5869.26646	3386.564812	6840.759615
COL1A1	406.314972	424.5768	1985.809975	3030.17475	146.1029151	2874.025099
SGCA	0	0	0	0	0	0

PTGES3L-AARSD1	32.5931025	35.0529794	23.40848728	21.1800465	0	31.34064571
MRPL27	897.60447	901.971767	857.5088528	1118.33864	1300.819748	974.0154419
VAT1	5869.10257	7217.80561	2604.620457	2439.82872	3244.492322	1837.491457
LRRC59	7893.8404	8475.28426	8446.712933	6111.64021	10391.44389	7690.644717
RND2	0.97671868	0	0	0	39.29664614	2.718182629
ALOX12	0	0	0	0	1.007606311	0
HDAC5	723.748545	861.494769	425.2441562	493.798806	235.8302572	555.4153171
ABCC3	1154.48148	993.387825	1001.931396	1248.07397	1111.389761	1080.024564
LUC7L3	3838.50443	3484.98326	10372.72796	6894.6784	3452.059222	7752.256857
PPY	0	0	0	0	0	0
MPP2	201.204049	235.650282	37.10857024	37.210908	236.7874832	118.6939748
SMURF2	9472.2178	7728.71979	1372.014164	1437.14561	1750.212163	1359.091314
DUSP3	2590.25795	2823.74044	893.6144887	1845.4599	2138.140593	1088.179112
CACNG1	0	0	0	5.02850108	15.11409467	6.342426133
EFTUD2	5691.33977	5660.68543	1853.422643	2249.75138	4170.482522	3178.461554
HLF	0.97671868	0	1001.931396	689.910348	37.28143352	864.3820759
SLC16A6	13.6740616	25.3933493	4987.452016	2815.9606	701.2939927	7123.450609
PRKAR1A	6412.15816	6646.96311	13743.20913	15034.2125	11217.68106	14144.50728
EFNB3	71.3004639	59.9283044	21.06162094	24.1368052	139.049671	26.27576541
FAM20A	0.97671868	0	1.002934331	0	0	1.812121752
YWHAE	18394.543	17472.6558	7938.225228	6995.6507	13408.21718	8370.190374
AC016292.3	0.98648587	1.02589131	4.051854696	3.01710065	1.007606311	0.906060876
MMD	839.001349	826.807453	159.4665586	159.906334	292.2058303	219.266732
RANGRF	454.174188	499.741114	164.4812302	275.561859	47.35749663	183.9303579
DPH1	847.606241	777.026331	665.8180141	586.172371	554.31446	820.863972
MAP2K6	14.6507803	15.2360096	7.020540315	5.02850108	139.049671	50.73940907
DHRS7B	527.428089	495.678178	534.5639983	297.687264	342.5861458	457.5607425
WSB1	2220.7555	2027.40501	1247.650307	1184.71485	1736.105674	1913.60057
RCVRN	0.97671868	1.01573397	0	0	0	0
MYH1	0	0	0	0	0	0
SLC9A3R1	1560.79646	1815.11661	30345.78404	28041.9391	24493.90182	34404.03753
MYH3	251.016702	224.477208	60.17605984	40.2280086	30.22818934	71.57880922
NAT9	684.679797	686.636165	1655.84458	1566.88094	464.5065095	1750.509613

TMEM104	1031.41493	1237.16398	1490.360415	1686.55926	827.2447816	2023.233937
VTN	5.8603121	1.01573397	0	0	20.15212623	0.906060876
TNFAIP1	2163.43188	1684.08693	1197.503591	1116.32724	1132.549494	1227.712487
IFT20	379.943568	330.113541	611.7899417	413.342789	271.0460977	443.9698293
TMEM97	1269.73429	2081.23891	742.1714047	12130.756	1754.242588	1374.494349
CDR2L	1213.08461	1492.11321	12663.04886	11716.4075	1274.621984	13634.40406
PMP22	1306.8496	1410.85449	14.04108063	72.4104155	1047.910564	140.4394358
FOXN1	0	0	0	2.01140043	15.11409467	2.718182629
UNC119	689.563391	865.405344	697.0393598	590.346027	796.0089859	732.097188
ALDOC	209.994517	358.554092	9.026408976	16.0912034	315.3807754	38.96061768
SUPT6H	6369.18254	6416.3915	4771.961546	4767.01902	5474.325089	7108.953634
RAB34	3926.40911	3966.44116	0	2.01140043	101.7682374	50.73940907
PHF12	1686.79317	1341.78458	2045.986035	1833.39149	1751.219769	2980.034222
PHOX2B	0	0	0	0	0	0
TMEM33	2331.4275	2529.17759	2175.364563	1679.51936	1637.360256	2268.776434
GABRA4	0	0	0	0	1.007606311	0
GNRHR	0	0	0	1.00570022	0	0
SLAIN2	1884.09034	1836.44702	1669.885661	2642.98017	1617.20813	2034.106667
OCIAD1	1865.53269	1962.39803	2636.714355	2106.94195	1818.729392	2326.76433
UGT2B10	0	0	0	0	0	0
CWH43	0	0	0	1.00570022	0	0
DCUN1D4	996.253058	969.01021	1176.44197	751.258061	1128.519069	974.9215028
USP46	590.914804	695.777771	636.8633	737.178258	962.2640273	764.7153795
SULT1E1	0	0	1045.057573	131.746728	0	401.3849681
ODAM	0	0	3.008802992	1.00570022	0	7.248487009
SMR3A	0	0	0	0	0	0
CHIC2	633.890426	517.008592	169.4959019	152.866433	177.3387108	125.9424618
NMU	89.8581189	100.557663	60.17605984	153.872133	26.19776409	81.54547886
KIAA1211	148.46124	172.674775	135.3961346	255.447855	127.9660015	139.5333749
LAMTOR3	383.850443	353.475422	1520.448445	1254.10817	566.2747469	771.0578056
PF4V1	0	0	0	0	0	0
NFKB1	1907.53159	1927.86308	917.6849126	1342.60979	1544.660475	1304.727662
AREG	65.4401518	106.652067	69.20246882	30.1710065	85.64653646	48.92728731

MANBA	228.552172	284.405512	898.6291603	1613.14315	476.5977852	1418.891332
UBE2D3	5099.86824	5354.43148	5902.66971	5397.92494	4494.569016	4954.938871
MAPK10	0	2.03146794	12.03521197	5.02850108	17.12930729	11.77879139
ELF2	682.72636	611.471851	1166.412627	1242.03977	926.9776543	1446.979219
NDUFC1	503.986841	458.096021	1335.908529	1260.95699	968.3096651	1108.873543
UCP1	0	0	0	0	0	0
TBC1D9	1518.79755	1231.06957	988.8932501	1519.61303	4567.479409	1770.442952
ZNF330	633.890426	574.905428	1023.995952	999.666014	611.617031	665.0486831
INPP4B	1749.30316	1413.90169	765.2388943	233.32245	2923.065909	483.8365079
GAB1	259.80717	318.940467	838.4531005	1360.71239	354.6774216	1262.142801
KLHL2	585.454946	642.959604	148.3239582	383.030984	345.3973674	248.8858621
IL2	0	0	0	0	0	0
CPE	91.8115563	42.6608268	84.24648378	79.450317	188.4223802	93.32427025
RPL34	4425.51236	4307.4129	6665.501562	4622.09762	4283.868461	3564.443487
WFS1	779.42151	970.025943	1286.764746	1108.28164	1686.732965	2235.252182
ANXA10	6.83703079	4.06293589	0	0	0	0
GRPEL1	1666.28207	1446.40518	602.7635328	569.226322	1237.34055	934.1487633
GAR1	596.775116	622.644925	410.2001413	274.556159	569.2975659	484.7425688
FRG1	458.198269	465.896858	819.0864385	685.233842	141.0145033	593.9863286
CLCN3	1849.90519	2297.59025	3817.168063	3559.17306	1328.025118	3598.8738
AADAT	178.739519	156.423032	0	0	139.049671	9.966669638
GALNT7	1909.48503	1926.84735	4000.705045	2612.80916	2033.349536	4243.083083
DHX15	5311.3962	5341.74496	5132.01497	5209.52712	4665.217221	6664.077744
SOD3	140.64749	191.973721	3.008802992	0	4.030425245	1.812121752
SEPSECS	155.298271	138.13982	365.0680964	384.177482	198.4984433	384.1698115
CPZ	0	0	0	0	4.050577371	0.244636437
TRIM2	1012.85728	1145.74792	223.6543557	279.58466	322.4340196	163.0909577
SLC2A9	0	0	14.04108063	9.05130194	1.007606311	9.966669638
FBXW7	573.333867	493.646711	2205.452593	1658.39966	681.1418664	1885.512683
NEIL3	356.50232	376.837304	163.4782959	334.898172	249.8863652	429.4728553
TBC1D19	43.9523408	62.9755063	111.3257107	89.5073192	28.21297672	61.61213958
CLNK	0	0	0	0	0	0
WHSC1	9787.69793	8863.29464	3991.678636	4301.37982	6168.565838	5737.177468

SH3D19	1549.07583	1457.57825	538.5757356	510.895709	1351.200063	646.0214047
STIM2	451.244032	503.80405	421.2324189	577.271924	656.959315	675.0153528
NKX3-2	92.788275	100.557663	26.0762926	33.1881071	21.15973254	19.93333928
MFSD10	1688.7466	1442.34224	1350.952543	1039.89402	970.3248778	1187.845809
GLRB	542.07887	462.158957	283.8304156	203.151444	12.09127574	235.5758278
BST1	7.81374947	5.07866986	8.023474646	4.02280086	0	4.530304381
RAPGEF2	1166.20211	800.39837	734.1479301	913.175796	829.2599942	963.1427114
HGFAC	12.6973429	9.14160575	2.005868661	2.01140043	5.038031556	2.718182629
SNX25	554.737144	650.069742	262.7687946	496.815906	122.6458402	198.4273319
LRP2BP	35.1618726	37.582157	18.05281795	34.1938073	1.007606311	22.6515219
UFSP2	478.592155	494.662444	198.5809975	395.240185	179.3539234	337.0546459
KLF3	934.71978	818.681582	1810.296467	1737.84997	1026.750831	2031.388484
KLHL5	2280.63813	2094.44345	4.011737323	13.0741028	1353.215276	145.8758011
FAM149A	64.8931894	49.5068738	0	0	21.37132986	5.436365257
NCAPG	3409.72493	3271.67912	267.7834663	543.078116	1409.641229	742.0638576
UGDH	1073.41383	1205.67623	844.4707065	721.087055	6211.892909	1121.703365
PPARGC1A	53.7195276	137.124086	321.9419202	256.453555	5.038031556	198.4273319
DDX25	1.95343737	5.07866986	4.011737323	2.01140043	2.015212623	3.624243505
CRYAB	0	0	0	1.00570022	0	9.060608762
DBX1	0	0	0	0	0	0
HTATIP2	914.208688	1119.33884	1379.034705	1278.24497	1873.140133	954.9881635
CTSC	3280.79806	3561.16331	148.4342809	316.795568	691.2179295	599.8123
CCDC34	503.986841	483.489371	191.5604572	315.789868	498.7651241	325.2758545
ZBTB16	0	0	25.07335827	1.00570022	0	38.0545568
ELP4	496.173091	479.426435	328.9624605	409.319988	349.63939	372.3910201
ZPR1	1995.43627	2214.30006	650.9043806	725.109855	1030.781256	1151.603374
MTCH2	2887.18043	3190.42041	2814.233732	3291.65681	3537.705759	3053.425153
FNBP4	1868.46284	1651.58344	2667.80532	2385.52091	2709.453371	3290.813102
TECTA	6.83703079	5.07866986	3.008802992	20.1140043	2.015212623	18.12121752
SC5D	680.772923	1707.44881	498.4583624	4306.40832	715.400481	759.2790142
CRTAM	3.90687474	1.01573397	0	0	0	0.906060876
C11orf63	199.250612	213.304134	1.002934331	7.03990151	84.63893015	14.49697402
B3GAT1	0	0	0	0	0	0

HSPA8	44764.9707	52753.1596	31406.08622	31932.9932	52143.62661	41879.03976
P2RX3	0	1.01573397	0	0	0	0
VWA5A	0	1.01573397	14.04108063	6.03420129	2.015212623	9.966669638
DNAJC4	353.435423	324.852039	1103.338087	1114.74829	231.1247357	824.5063367
SIAE	502.033404	517.008592	35.10270157	29.1653063	416.1414066	144.9697402
SNX15	947.417123	1115.2759	584.7107148	533.021114	494.7346988	451.2183163
LPXN	329.154196	811.571444	93.27289276	125.712527	22.16733885	79.7333571
DTX4	12.6973429	13.2045416	295.8656276	243.379452	194.4680181	342.4910112
ATG2A	2873.50637	3248.31724	1539.504198	1908.81901	1270.591559	2224.379451
EHD1	9919.55495	9070.50437	1488.354547	2183.37517	2429.338817	2728.149298
OSBP	2321.66031	2433.6986	3560.416874	3371.10712	1735.098068	3462.058608
UNC93B1	1692.65348	1422.02756	809.3579755	982.569111	1317.928903	1220.418697
PUS3	195.343737	183.847849	204.5986035	167.951936	163.2322224	192.9909666
DCPS	846.815099	689.683367	400.170798	288.635962	434.2783202	361.5182896
SUV420H1	961.091185	945.648328	1517.439642	2177.34097	1933.596511	1769.536891
FOXRED1	1246.29304	1222.9437	990.8991187	795.508871	680.1342601	1213.215513
PPP6R3	5946.26335	5604.82006	5064.81837	5251.76653	5617.405185	7070.899078
NRXN2	0.97671868	0	0	0	3.022818934	0.906060876
MS4A6A	0	0	0	0	0	0
MS4A4A	1.95343737	0	0	0	0	0
ST3GAL4	1439.68334	1316.39123	302.8861679	254.442155	1758.273013	526.4213691
CPT1A	1745.39629	2235.63047	2917.535968	3710.0281	5826.987298	3234.637328
CCND1	27556.1642	23731.6085	14405.14579	12575.2755	21347.14731	20540.40006
CCDC86	2063.80658	2186.87524	500.464231	628.562635	1602.094035	1338.251914
PRPF19	4256.54002	4218.34319	3765.015477	4193.7699	4492.916542	4890.91661
TMEM109	4029.94129	4358.51447	1171.427298	1390.8834	2298.349996	2064.912737
CCKBR	0	0	2.005868661	0	0	0
HPX	1.95343737	0	246.7218454	241.368052	10.07606311	342.4910112
TRIM3	402.408098	556.622217	4438.987348	5229.64112	268.0232788	4234.928535
CHORDC1	1676.01019	1906.49204	1534.489526	1328.51993	1103.318835	1473.254985
FOLR1	30.2782792	22.3461474	0	0	27.2053704	4.530304381
ANAPC15	519.61434	521.071528	190.5575228	337.915272	789.9633481	224.7030973
FOLR3	0.97671868	0	0	0	0	0

PANX1	1636.98051	1809.0222	401.1737323	711.030052	427.225076	894.2820848
ARHGEF17	2256.22016	1937.00469	2149.288271	2062.69114	1253.462251	2564.15228
APOA5	0	0	0	0	0	0
APOA4	0	0	0	0	0	0
APOC3	0	0	0	0	0	0
CEP164	1664.32864	1941.06762	672.9689359	828.696978	705.3244179	914.2154241
RNF141	587.984648	564.748089	535.5669326	470.667701	573.3279911	524.6092473
CEP126	187.529987	199.083859	110.3227764	135.769529	107.8138753	172.1515665
EIF4G2	25011.8121	24349.6623	10114.80334	9728.13819	29117.80718	14276.23947
IL10RA	17.5809363	20.3146794	8.023474646	0	4.030425245	2.718182629
GALNT18	730.585576	709.998047	164.4812302	151.860733	314.3731691	163.9970186
BIRC2	3823.85365	3619.06014	1497.380956	1279.25067	811.1230806	1232.242792
UBE4A	3324.7504	3187.3732	2342.854597	2568.55835	3764.417179	3391.38586
DDX6	5297.72214	5807.96685	4459.046034	3742.2105	7972.181135	4504.934676
UPK2	2.93015605	0	2.005868661	0	99.75302482	57.98789608
CBL	5388.55698	5068.51252	951.7846798	1425.07721	2388.026958	1948.936945
PVRL1	668.07558	940.569658	4311.614688	4244.05491	2438.407273	5939.229043
HIPK3	1530.54748	1312.32829	1842.390366	2772.71549	2290.289146	2386.564348
KIAA1549L	4.88359342	3.04720192	0	0	10.07606311	4.530304381
FBXO3	1196.48039	1137.62205	978.8639068	957.426605	753.6895208	1006.633633
PDHX	1132.01695	1228.02237	1229.597489	1026.81992	1026.750831	1363.621619
SLC1A2	0.97671868	1.01573397	27.07922693	24.1368052	161.2170098	51.64546994
COMMD9	569.426993	596.235842	645.889709	876.970588	506.8259746	751.1244664
SLC15A3	136.740616	142.202756	2.005868661	7.03990151	8.06085049	16.30909577
CD5	0	0	0	0	0	0
ACCS	179.716238	117.825141	378.1062427	277.573259	137.0344583	183.024297
SCGB2A2	0	0	1.002934331	0	0	9.060608762
MDK	649.517925	492.630977	11.03227764	10.0570022	3798.675794	714.8820313
AMBRA1	971.83509	970.025943	1135.321662	1044.92252	1606.12446	1366.339801
MADD	3033.68823	3327.54449	2345.8634	2829.03471	3142.724085	2842.312969
PTPMT1	1127.49475	1104.23487	1085.054594	858.455647	1912.819669	1138.991006
NAA40	1131.04024	1151.84232	1081.163209	806.571573	1031.788863	1152.509434
CARS	2971.17824	2036.54661	3878.347057	1272.21077	1419.717293	1808.497509

SLC22A18	88.8814002	195.020923	1212.547606	1432.11711	297.2438618	981.2639289
CD81	5034.0081	4698.78536	4906.354746	3806.57532	5104.533573	4570.171059
SLC35F2	2557.04951	2590.12163	255.7482543	282.601761	691.2179295	546.3547083
C11orf21	0	0	0	0	0	0
ELMOD1	17.5809363	24.3776153	0	0	0	0
CALCA	0	0	0	0	0	0
SOX6	3.90687474	1.01573397	6.017605984	2.01140043	3.022818934	4.530304381
C11orf58	4036.77832	3901.43419	1660.859252	1236.00556	3237.439078	1764.100526
PITPNM1	2426.16921	2237.66194	1908.584031	1800.20339	684.1646854	2057.66425
RPS13	6432.61065	6784.93026	3301.639758	2667.11697	7039.097386	2370.237131
AIP	1622.32973	1611.96981	1413.134472	1850.4884	985.4389724	1123.515486
NUP98	6024.40084	5987.75177	2821.254272	3586.32697	2093.805915	5126.492437
NDUFS8	2629.3267	2626.68805	4258.459168	3256.4573	1912.436779	3555.382878
TCIRG1	3164.16808	3245.02627	820.4002825	1316.25039	1103.328911	1132.576095
CHKA	2233.75563	3073.611	1149.362743	1758.96968	1092.245241	1043.782129
EXPH5	320.363728	313.861797	491.437822	1288.30198	195.4756244	813.6426668
HPS5	1110.52914	963.93154	998.9225934	1041.90542	816.1611121	1361.809497
GTF2H1	1987.62252	2291.49584	2546.450266	2866.24561	1596.048397	2324.952208
POU2AF1	0	0	0	0	0	0
PTPN5	0	0	0	0	0	0
VWF	3.90687474	0	7.020540315	7.03990151	23.47722705	17.21515665
PSMD9	1560.73785	1558.3289	1199.228638	1180.56131	1733.546354	1024.573639
P3H3	125.99671	94.4632594	691.0217539	521.958412	21.15973254	547.2607692
PPFIBP1	1650.65458	1822.22675	408.1742139	632.585436	1280.667622	716.6669712
PRPF40B	244.179671	328.082073	868.5411304	715.052853	402.0349182	675.9214136
CD69	0	5.07866986	0	0	0	0
PRDM4	1853.81206	1971.53964	1171.427298	1268.18797	2193.55894	1130.763973
CLEC2B	12.6973429	65.0069742	0	0	5.038031556	0
COQ5	265.667482	250.886291	950.7817455	834.731179	609.6018183	703.1032399
SELPLG	39.0687474	62.9755063	1.002934331	1.00570022	3.022818934	1.812121752
CORO1C	10224.2912	9960.28733	1188.477182	1863.5625	3163.883817	2072.161224
ASIC1	155.298271	154.391564	253.7423857	134.763829	221.6733885	256.415228
DAO	0	0	0	0	0	0

CAPRIN2	1135.92383	941.585392	377.1033083	459.604999	218.6505695	334.3364633
TSPAN11	0	0	0	1.00570022	0	0
KCTD10	2665.46529	2524.09892	939.7494679	1306.40458	1756.257801	1478.69135
SLC11A2	1432.84631	1353.97338	1725.047049	1633.25715	1422.740112	1276.639775
MLEC	11490.1186	12962.797	9302.215917	7491.46091	8567.676465	8523.314662
MVK	460.0345	1191.45595	845.4736408	5141.1395	608.594212	715.7880922
CSRNP2	602.635428	607.408915	965.8257605	1014.75152	836.3132384	839.0123713
CAMKK2	1601.81864	1454.53105	1165.409692	1050.95673	2082.722245	1574.733803
BIN2	0	1.01573397	1.002934331	0	9.068456802	3.624243505
IL23A	2.93015605	15.2360096	3.008802992	4.02280086	6.045637868	1.812121752
ATP5B	13215.0038	12766.7603	12593.84639	14360.3934	20117.86761	20136.29691
PTGES3	8598.02527	8148.16714	6761.713052	8266.80549	12229.23719	9126.705903
SYT10	0	0	0	0	182.3767423	49.83334819
BCL7A	678.819485	691.714835	491.437822	412.337088	825.2295689	525.5153082
RSRC2	2404.6814	2133.04134	2782.139833	2206.50627	1851.9804	2465.391644
CYP27B1	30.2782792	33.5395358	313.9184455	273.550459	93.70738695	393.2304203
MYF6	0	0	0	0	0	0
MYF5	0	0	0	0	0	0
LIN7A	2.93015605	3.04720192	2008.877464	1544.75553	350.6469963	671.3911092
KRT18	8746.51581	6744.47358	16439.09661	15000.0891	153827.9304	16368.89579
ACSS3	178.739519	225.492942	76.22300913	88.501619	633.7843698	77.01517448
TNS2	1329.31413	1425.07476	1398.090457	1214.88586	252.9091841	1090.897295
GLI1	0.97671868	1.01573397	11.03227764	9.05130194	3.022818934	17.21515665
PPM1H	72.2771826	135.092618	1764.161488	2597.72366	2167.330947	1780.518349
METAP2	4282.91143	4200.05998	1736.079326	2053.63984	3366.412686	2271.494617
LTA4H	3075.68714	3114.24036	1726.049983	2140.13006	1555.744145	1904.539962
ELK3	5063.30966	4316.86938	164.4812302	160.912034	668.0429844	488.3668123
SLC6A12	3.90687474	2.03146794	0	0	1.007606311	0.906060876
WNT5B	629.006832	662.25855	5.014671654	1.00570022	14.10648836	5.436365257
MAGOHB	281.294981	255.964961	525.5375893	282.601761	457.4532653	410.4455769
TRPV4	0	6.09440383	7.020540315	2.01140043	142.0724899	31.71213067
ITFG2	237.34264	220.414272	394.153192	365.069178	472.56736	356.0819243
FOXM1	5526.27431	5873.98956	1445.228371	4563.86758	5331.244993	5109.277281

PRR4	86.4591379	81.3501338	91.88884338	74.431873	50.02765336	82.18878208
PRMT8	0	0	0	0	0	0
PARP11	189.483425	182.832115	116.3403824	142.809431	143.0800962	117.7879139
ARPC3	5241.9222	5321.1154	5052.743041	5426.55722	5374.491456	4430.483654
GPN3	1109.55242	1114.26017	410.2001413	443.513795	671.0658033	529.1395517
VPS29	1786.41847	1532.74256	1580.624505	1517.60163	1355.230489	1520.37015
FGF6	0	0	0	0	0	0
MYL2	0	0	0	0	1.007606311	0
RAD51AP1	1002.11337	700.856441	622.8222194	752.263761	558.2138965	964.9548331
CUX2	0	0	0	0	3.022818934	0.906060876
SH2B3	4489.97579	6782.05573	81.23768079	137.78093	534.031345	348.8334373
AKAP3	2.93015605	1.01573397	6.017605984	8.04560172	4.030425245	1.812121752
MANSC1	56.6496837	85.3216537	1760.14975	2125.04456	674.0886223	2052.227885
KCNA1	0	0	0	0	0	0
DUSP16	599.705272	526.150198	3568.440349	5783.78194	1674.641689	4504.934676
CREBL2	1185.73648	1184.34581	1278.741272	1362.72379	1188.975447	1070.963956
ACAD10	371.944242	406.293589	744.1170973	621.522733	587.4344795	723.0365792
ALDH2	1448.8645	1662.44164	94.83747031	49.7721037	60.45637868	59.81813905
CDKN1B	777.468072	747.580204	3992.681571	4539.73077	1940.649756	3048.894848
GPRC5D	0.97671868	0	0	1.00570022	0	0
NAA25	2551.1892	2328.06226	1058.095719	748.24096	2583.502582	1728.764152
GSG1	3.90687474	4.06293589	0	0	1.007606311	0.906060876
SCNN1A	180.692957	141.187022	7.020540315	43.2451093	1022.720406	377.8273854
LTBR	2393.93749	2619.57791	3005.794189	2586.66095	4256.129059	2735.397785
OGFOD2	385.540166	349.585161	429.7974781	351.552567	576.6329398	365.2784422
CDK2AP1	2378.63231	2153.82326	1438.578916	1410.00176	4044.199223	1994.013473
OAS3	1273.64116	1377.33527	611.7899417	1221.92576	1342.131607	5645.665319
OAS2	257.853733	475.363499	4.011737323	20.1140043	29.22058303	323.4637328
ART4	0	0	0	0	4.030425245	0
MGP	0	0	1084.172011	1220.92006	151.1409467	2667.443219
RASAL1	1.95343737	0	110.3227764	56.3192121	752.6819145	227.4212799
ARHGDIB	2181.98954	2293.52731	588.7224521	749.246661	131.9964268	711.2577878
GTF2H3	2222.03501	1917.70574	979.8668411	755.280862	1419.717293	1155.227617

EIF2B1	1529.54146	1472.81426	1678.91207	1315.45588	2129.072136	2015.079389
DDX55	1373.26647	1372.2566	1094.201355	895.073192	1533.576806	1451.509524
SLC38A1	9531.79764	9487.97103	19309.49467	22644.3461	18720.31766	19607.15736
RERGL	0.97671868	1.01573397	0	0	0	0.906060876
ENDOU	0	0	13.0381463	2.01140043	4.030425245	3.624243505
C12orf49	2122.4097	2693.72649	2435.124555	4531.68517	3113.503502	2253.373399
VDR	501.056685	748.595938	3094.05241	2204.49487	732.5297883	3317.994929
FZD10	0	0	0	0	0	0
RFC5	1512.93724	1397.64995	700.0481628	897.084592	1891.277046	1308.351905
STX2	1128.11008	1110.19723	556.6285535	454.576497	416.1414066	295.3758456
ADGRD1	168.972332	208.225464	0	1.00570022	35.2662209	4.530304381
COPZ1	2115.57267	2199.06405	3934.511379	3383.17553	3155.822967	2684.658376
TBC1D30	39.0687474	42.6608268	627.836891	923.232798	1437.854206	1200.530661
CAND1	4620.85609	4202.09144	3545.372859	4483.41156	9145.034881	5508.850127
IL26	0	0	0	0	0	0.906060876
IFNG	0	0	0	0	0	0
RAB5B	2304.07938	2349.39268	4273.503183	5527.32838	3349.283379	3457.528303
MDM1	260.783889	265.106567	331.9712635	260.476356	512.8716124	298.0940283
NUP107	2240.59266	2186.87524	1141.339268	1064.03083	3041.963454	1746.885369
CNOT2	1342.98819	1297.09228	2113.182635	1643.31415	1902.360716	1825.712665
TIMELESS	1982.73893	1907.5484	1755.135079	3240.36609	4997.727304	4058.246664
CPSF6	2382.21687	2156.40322	2618.661537	2457.93133	3525.614483	2719.088689
KRR1	2277.70797	2107.64799	1771.182028	1312.43878	1918.482417	1518.558028
MRPL51	1696.56035	1837.46276	1465.287057	1923.90451	2650.004599	1665.33989
GAPDH	65413.7706	69164.3734	42810.25191	62126.1251	112096.182	66153.30763
NOP2	1741.48941	1763.31418	800.3415959	686.893247	2566.373275	1484.127715
CHD4	7481.66512	7637.30374	7011.513906	7910.8379	12284.73615	10603.63043
ACRBP	4.88359342	2.03146794	1.002934331	9.05130194	7.053244179	3.624243505
UHRF1BP1L	1604.7488	1493.12894	1340.9232	2118.00465	1171.84614	1774.973256
COPS7A	972.811809	1096.99269	645.889709	634.596836	779.8872849	691.3244485
ING4	203.157486	173.690509	232.6807647	228.293949	88.66935539	189.3667231
GNB3	7.81374947	1.01573397	53.15551953	46.2622099	2.015212623	46.20910469
CDCA3	1396.70772	1298.10802	65.1907315	388.200283	1148.671195	403.1970899

CHPT1	1636.0038	1809.0222	2270.643325	1215.89156	2326.562973	2150.98852
USP5	2870.57621	2955.78586	1537.498329	1493.46482	3778.523667	2073.973346
TPI1	12944.1499	12302.4683	3931.472488	4128.36921	17228.90918	6920.438609
GNPTAB	1116.38946	1244.27412	1305.820499	2084.81655	2465.612644	1628.191394
SPSB2	105.485618	125.951013	197.5780631	205.162844	205.5516875	221.9849147
ENO2	2332.40422	2606.37337	1515.433774	1971.17242	764.7731903	1482.315593
ATN1	3235.869	2971.02187	5673.599509	6408.32177	5280.673232	6645.956527
C12orf57	737.422606	775.005021	2596.596982	1708.68467	833.2904194	1128.951852
PTPN6	58.603121	39.6136249	1142.342203	1076.09923	951.1803579	1060.997286
LPCAT3	600.681991	384.963175	479.4026101	949.381003	622.7007004	555.4153171
NT5DC3	2038.41189	1495.16041	88.2582211	199.128643	577.3584164	250.0728018
SLCO1B3	418.758369	447.938682	0	0	33.25100827	14.49697402
APOBEC1	0	0	0	0	0	0
NANOG	0	0	1.002934331	0	0	0
SUDS3	1581.30755	1516.48066	2782.139833	2726.45328	2484.716859	2234.346121
GOLT1B	1175.9693	1217.86503	700.0481628	531.009714	790.9709544	572.6304737
GYS2	5.8603121	4.06293589	0	0	0	0
LDHB	9942.9962	10525.0354	0	0	729.5069694	354.2698026
PRKAB1	621.193083	663.274284	433.2676309	444.519495	1143.633163	588.0335086
CMAS	943.510249	1030.96998	1186.471313	1199.80036	1388.481497	998.4790855
HCFC2	328.177478	323.003403	345.0094098	452.565097	458.4608716	398.6667855
ST8SIA1	3.90687474	9.14160575	0	0	0	0
CLEC4A	0.97671868	2.03146794	4.011737323	5.02850108	3.022818934	10.87273051
C2CD5	1020.67102	1057.37907	977.8609724	1184.71485	986.4465788	983.9821115
AICDA	0	0	0	0	0	0
RAB35	2731.88216	2786.15829	1471.304663	1601.07474	2900.89857	1909.976327
PHC1	622.736298	697.504519	896.6834677	886.695709	607.8284312	704.3354827
COX6A1	3369.49388	3729.61263	4357.769726	4676.66691	4903.012311	4012.182529
AL021546.6	8.07746352	0	0	3.58029277	26.9333167	6.44209283
RFX4	0	0	0	0	1.007606311	0
RIC8B	758.910417	872.515482	248.727714	296.681564	409.0881624	461.184986
SRSF9	4825.96702	4958.81325	3977.637556	5622.86991	7031.07684	4930.783288
RP11-22B23.1	206.566234	180.049004	124.5644439	119.326331	181.4598206	173.8277791

FGFR1OP2	924.952594	799.382636	941.7553365	747.23526	912.891318	821.7972147
KLRB1	0	0	0	0	0	0
COL12A1	6287.13817	4245.768	49.1437822	49.2793106	1214.165605	530.0456126
BTN3A3	388.362883	468.18226	292.5459149	115.514727	17.18976367	251.0151051
TDP2	1401.59131	1563.21458	861.5205901	875.964888	1244.393794	877.972989
FRK	19.5343737	24.3776153	24.07042394	23.131105	610.6094246	136.8151923
DSE	1338.1046	1625.17436	97.12416059	132.752428	121.9203637	121.4121574
RWDD1	1034.34509	1033.00145	1065.116259	1018.77432	790.9709544	987.606355
RSPH4A	8.79046815	2.03146794	17.04988362	12.0684026	0	19.93333928
MAK	8.79046815	18.2832115	0	1.00570022	8.06085049	7.248487009
TMEM14C	2899.87777	2743.49746	1954.719011	1746.90127	1737.113281	1597.385325
PAK1IP1	1311.73319	1310.29682	440.2881712	349.983675	680.1342601	635.1486742
GCNT2	3137.22041	3112.20889	848.4824438	923.232798	129.9812142	572.6304737
SMIM8	89.8581189	122.903811	122.3579883	106.604223	96.73020588	163.0909577
NEDD9	755.003543	489.583775	88.2582211	79.450317	1463.044364	641.4911003
CEP85L	392.640911	318.940467	69.20246882	89.5073192	141.0648836	94.23033112
ADTRP	18.557655	24.3776153	0	0	0	0.906060876
ASF1A	554.776212	535.291803	1276.735403	854.845183	860.4957898	1153.415495
MCM9	329.154196	373.790102	466.3644638	679.853346	553.1758649	622.4638219
FAM184A	41.9989034	32.5034871	312.9155112	252.430754	68.51722917	348.8334373
RNGTT	853.65213	935.490988	707.0687031	683.876147	1072.093115	788.2729623
MAN1A1	254.923576	333.160743	411.2030756	152.866433	5968.052182	1157.039739
GABRR2	7.81374947	10.1573397	3.008802992	11.0627024	1.007606311	9.966669638
SERINC1	3756.46006	3553.03743	3397.941512	2973.85554	2430.346423	4265.734605
HDDC2	570.403711	562.716621	1075.145603	963.460806	1319.964268	1218.651878
TPD52L1	20.5110924	26.4090833	2338.842859	836.742579	6521.228047	1721.515665
HINT3	526.451371	640.928136	1385.052311	1208.85166	1091.237635	795.5214493
NCOA7	1027.50806	1137.62205	647.8955776	413.342789	621.6930941	633.3365524
FAM65B	0.97671868	1.01573397	0	0	0	3.624243505
SASH1	1359.59241	1307.24962	4753.908728	4013.74956	864.5262151	6324.304916
UST	337.944665	448.954416	76.22300913	169.963336	144.0877025	75.20305272
ULBP1	0	1.01573397	509.4104053	58.3306125	97.73781219	138.3645564
FBXO5	1027.50806	990.340623	330.9683291	570.232022	892.7391918	896.0942065

MTRF1L	921.905231	1014.26116	738.5106944	757.03078	837.4014532	837.2183708
PPARD	2606.86217	2251.88222	2079.082868	3455.58594	668.0429844	2990.000891
OPRM1	0	0	0	0	0	0
FANCE	1189.64336	1104.10283	215.6308811	365.069178	196.4832307	325.2758545
TULP1	0	0	1.002934331	13.0741028	0	8.154547886
SLC26A8	0	0	0	1.00570022	3.022818934	2.718182629
MAPK14	6217.79114	6519.99637	1907.581097	1759.97538	2171.391601	2940.167543
RHAG	0	0	0	0	0	0
KCTD20	10135.4098	8842.97996	2396.010116	2457.93133	2775.955388	3145.843362
STK38	5150.23762	5157.89711	982.8756441	1031.84842	2848.503042	1563.861072
SRSF3	9686.11919	9610.87485	6306.451071	5853.17525	7247.712197	6796.362632
SOD2	4488.02235	5492.07359	851.4912468	803.554472	2191.543727	1357.279193
MRPL18	844.861662	870.484014	939.7494679	853.839483	870.571853	1053.748799
IL17A	0	0	0	0	0	0
IL17F	0	0	0	0	0	0
MCM3	10017.2268	8700.77721	2334.831122	3550.12176	4604.760843	7128.886974
RNF8	1881.16019	1808.00647	303.8891022	316.795568	436.2935328	385.9819333
PHACTR1	196.320455	304.720192	0	0	23.17494516	9.966669638
MDGA1	396.547786	545.449143	0	5.02850108	16.12170098	26.27576541
ICK	3252.47322	2345.32974	1283.755943	1589.00634	1207.112361	1865.579344
FBXO9	1669.21223	1507.34921	1927.639784	1956.08692	1181.922203	2361.194643
CD83	649.517925	799.382636	5.014671654	9.05130194	72.54765441	35.33637417
MDN1	3795.52881	3004.54109	1906.578163	1714.71887	4535.236007	3248.228241
GLP1R	0	0	0	0	0	0
SAYSD1	344.781695	398.167717	359.0504904	388.200283	436.2935328	294.4697848
BMP5	2.93015605	0	0	0	75.57047335	0
BACH2	160.181864	101.573397	0	0	10.07606311	0.906060876
RBM24	391.664192	315.893265	0	1.00570022	187.4147739	12.68485227
CAP2	1986.6458	1729.79495	583.7077805	421.38839	668.0429844	464.8092295
TREML2	1.95343737	0	0	0	0	0
ZNF451	2627.05094	2501.64104	1118.271779	1109.05603	1299.530012	1706.11263
BAG2	2151.71126	2137.10428	44.12911055	40.2280086	306.3123186	148.5939837
RAB23	1437.7299	1537.82123	388.135586	351.995075	513.8792188	566.2880476

TSPO2	0	1.01573397	1.002934331	0	0	0
FHL5	0	0	0	0	0	0
GPR63	68.3703079	121.888077	44.12911055	43.2451093	12.09127574	35.33637417
KHDRBS2	0.97671868	1.01573397	0	0	0	0
FBXL4	641.704175	573.889694	311.9125769	404.291487	367.7763036	338.8667677
CCNC	1314.66335	1260.52586	2429.106949	1677.50796	1650.459138	2083.033954
PRDM13	0	0	0	0	0	0
E2F3	2227.89532	2245.78781	512.499443	455.582198	1219.203637	1257.612496
PTP4A1	3784.7849	3961.36249	5255.375893	6081.4692	7488.530105	5670.128963
SIM1	0	0	1.002934331	0	5.038031556	0.906060876
ASCC3	1662.3752	1618.06422	1617.733075	1816.29459	3637.458784	3451.185877
HDGFL1	0	0	0	0	0	0
BVES	0	0	0	0	4.030425245	3.624243505
COL9A1	0	0	0	0	0	0
MED23	598.728553	537.323271	1603.691995	1306.40458	897.7772234	1658.091403
WASF1	634.867145	699.840707	265.7775976	437.479594	398.004493	451.2183163
GPLD1	28.3248418	18.2832115	5.014671654	10.0570022	51.38792188	14.49697402
ALDH5A1	282.2717	211.272666	345.0094098	217.231247	1546.675688	632.4304916
AIM1	534.26512	569.826758	2664.796517	4162.59319	2426.315998	6428.501916
VNN1	194.367018	297.610054	0	0	1.007606311	0
VNN2	0	3.04720192	0	0	0	1.812121752
ACOT13	399.477942	453.017352	775.2682376	564.197821	543.0998018	497.427421
SMAP1	1374.24319	1657.67784	1434.196093	1723.77017	1224.241668	1368.151923
RPS12	8673.25215	9662.63665	13064.15239	12320.7629	11814.1437	11972.61593
C6orf62	6871.21594	7079.66579	3490.211471	4411.00115	5095.465116	6925.929338
B3GAT2	2.93015605	8.12587178	3.008802992	13.0741028	5.038031556	2.718182629
GMNN	1406.4749	1552.04151	286.8392186	584.311825	851.427333	458.4668033
EYA4	184.599831	255.964961	0	0	34.25861458	8.154547886
SOBP	0.97671868	3.04720192	0	0	195.4756244	7.248487009
NR2E1	0	0	0	0	0	0
SNX3	2590.25795	2705.9153	2891.459675	2929.60473	2356.791162	2676.503828
SLC17A2	0	0	0	0	0	0
HBS1L	1487.54256	1356.00485	1705.991297	1524.64153	1542.645263	1903.633901

TRIM38	1134.94711	1396.63421	3.008802992	0	181.369136	61.61213958
PEX7	90.8348376	72.117112	152.4460183	160.912034	194.4680181	202.0515754
ZBTB24	455.736938	470.579392	377.1033083	445.525195	778.8796786	739.345675
FIG4	359.432476	376.837304	549.6080132	848.810982	310.3427439	710.3517269
PERP	2012.04049	2209.22139	2479.253666	3698.96539	4631.966213	2467.203766
ARFGEF3	1468.9849	1182.31434	33712.63459	26101.9434	4708.544293	30698.24855
SLC16A10	48.8359342	68.0541761	23.06748961	25.1425054	76.57807966	63.42426133
HECA	653.4248	594.204374	668.9571986	944.352502	583.4040542	904.2487544
ADGRG6	2116.54939	1778.55019	498.4583624	767.349264	6041.607442	782.836597
PHACTR2	1671.16567	1425.542	149.4372153	447.536596	2399.110627	502.8637863
EPM2A	103.53218	104.620599	147.4313466	272.544758	120.9127574	154.030349
OR12D3	0	0	0	0	0	0
SLC39A7	6290.06832	7010.59588	5293.487397	4554.81628	2940.195216	5333.980378
CCR6	0	0	0	0	0	0
UNC93A	0	0	0	0	0	0
SLC22A2	0	0	0	0	0	0
PHF1	1761.02379	1951.22496	752.200748	857.862284	602.5485742	624.2759437
CUTA	3393.12071	3722.66501	1552.542344	2298.02499	1013.651949	1146.167008
PACRG	0	0	1.002934331	1.00570022	1.007606311	0
QKI	3759.39021	3235.1127	611.7899417	752.263761	1879.185771	789.1790232
C6orf118	0.97671868	3.04720192	0	0	0	0
PDE10A	1040.2054	936.506722	35.10270157	18.1026039	31.23579565	52.55153082
MDFI	15.6274989	19.2989455	2.005868661	0	5.038031556	1.812121752
TFEB	119.159679	115.793673	272.798138	328.86397	50.38031556	256.415228
SMOC2	0	0	0	1.00570022	37.28143352	5.436365257
CCND3	3337.44774	3562.17904	1002.934331	1405.9689	1779.432746	2189.043077
BYSL	2639.09388	2402.21084	357.0446217	308.749966	948.1575389	668.6729266
FAM120B	1322.4771	1255.44719	1236.61803	860.879385	1307.872992	1277.545835
TBP	601.658709	598.26731	419.2265502	329.869671	443.5281461	437.6274032
GUCA1B	13.6740616	14.2202756	25.07335827	21.1197045	8.06085049	29.90000891
PRPH2	0	1.01573397	0	0	0	0
GLTSCR1L	1061.69321	995.419293	894.617423	1207.84596	711.3700558	1011.163938
PPP2R5D	5117.02918	5275.72225	1215.556409	1552.80113	1607.132067	1653.561099

MRPL2	1687.76989	1622.12715	605.7723357	473.684802	740.5906388	754.7487099
PTK7	3392.14399	3445.36963	969.8374978	1228.96566	969.3172715	1796.718717
SRF	4513.41704	4591.11755	973.8492351	1827.35729	1819.736998	1863.767222
CUL9	1451.40396	1697.29147	1390.066982	1483.40782	765.7807966	1839.303579
DNPH1	1034.34509	958.85287	927.7142559	648.676639	974.355303	673.203231
DUSP22	624.123239	728.281258	591.7312551	586.323226	378.859973	468.433473
EXOC2	2848.11168	2713.02544	852.4941811	1038.88832	1180.914597	1001.197268
COX7A2	2060.87642	2274.22836	2373.945561	2279.92239	1853.995613	1783.127804
TMEM30A	6941.53969	6876.51899	2101.147423	2114.98755	3322.078008	2961.006943
GMDS	656.354956	678.510293	610.7870074	528.998313	687.1875043	622.4638219
SENP6	2473.05171	2358.53428	2223.505411	2155.21556	2411.201903	2341.261304
IMPG1	0	0	0	0	0	0
VEGFA	9127.4361	13052.1815	9837.78285	4704.66561	1587.987547	4476.846789
PRPF4B	5238.1423	4596.19622	3002.775357	2771.70979	3097.381801	3261.819154
TTK	1624.28317	1828.32115	452.3233831	968.489308	1006.598705	1118.985182
SLC29A1	4980.28857	5118.28349	5626.461595	6800.54486	1658.519988	4135.261839
WISP3	0	0	0	0	0	0
BTN2A1	1262.85819	1349.64636	368.6184839	409.933465	426.6910446	508.0192727
LAMA4	2.93015605	3.04720192	0	0	0	1.812121752
FAM46A	173.855926	255.964961	364.065162	851.828083	1553.728932	1013.88212
CLIC5	315.480135	186.895051	0	0	5.038031556	10.87273051
FBRSL1	1312.70991	1146.76365	1763.158553	1929.93871	3213.256527	3038.022118
ENPP5	14.6507803	14.2202756	218.6396841	192.088741	217.6429632	328.9000981
LY86	0	0	0	0	0	0
PRSS16	58.603121	94.4632594	232.6807647	210.191345	98.74541851	125.9424618
MEP1A	0.97671868	0	0	0	0	0
TBX18	0	0	0	0	0	0.906060876
ERBB2IP	7293.15841	5936.96507	4180.23029	4565.87898	3359.359442	4943.46814
PCDHB2	65.9871143	88.5923171	153.3586885	169.963336	302.1710567	224.639673
HARS2	658.69908	581.649902	888.1685552	906.196236	1265.291549	1193.599295
NUDT12	261.760607	278.311108	874.5587364	821.657076	519.9248566	572.6304737
CEP72	571.38043	592.172906	214.6279468	224.271148	345.6089648	365.1425331
MAN2A1	3781.85474	4113.72259	1031.016492	1830.37439	2159.300325	1861.04904

SEMA5A	64.4634331	19.2989455	0	2.01140043	673.0810159	93.32427025
C7	0	0	0	0	0	0
PAPD7	2746.53294	2351.42415	1342.929069	1365.74089	1508.386648	1568.391377
GHR	0	0	3857.285436	2619.84906	42.31946507	6208.329124
HMGCS1	1044.11227	4737.38325	919.6907813	74881.4209	1778.425139	1408.018602
DAP	10556.3755	8330.03431	3486.199734	2932.62183	3825.881164	3972.170881
NME5	2.93015605	1.01573397	0	0	1.007606311	2.718182629
BRD8	2428.12265	2504.79998	1315.849842	1741.87277	1219.203637	2197.197625
KIF20A	3449.77039	3343.79624	606.7752701	1686.55926	1824.77503	1636.345942
NNT	3572.83695	3688.13005	209.6132751	204.157144	1107.359336	314.403124
MRPS30	1421.12569	1492.11321	1647.821105	1380.8264	1091.237635	1466.912559
HSPA9	12140.6132	10785.0633	11752.38449	6194.10763	13587.57111	13361.67974
MRPS27	2931.13277	2991.33655	1389.064048	1824.34019	1784.470777	1954.37331
PFDN1	2365.61265	2078.19171	605.7723357	478.713303	937.0738695	562.6638041
HBEGF	3629.48663	3713.5234	4.011737323	3.01710065	274.0689167	120.5060965
SLC4A9	0	0	2.005868661	0	1.007606311	0
LOX	1355.68553	1243.25838	1.002934331	3.01710065	49.37270925	19.93333928
GZMK	0.97671868	0	0	0	0	0
CDH9	0	0	0	0	0	0
APBB3	186.553269	217.36707	428.2529592	356.017876	117.8899384	317.1213067
TMCO6	488.359342	388.010377	118.346251	98.5586211	188.4223802	141.3454967
SPARC	10.7439055	16.2517436	0	0	0	5.436365257
IK	2758.25356	2600.27897	3916.448532	2714.37482	2077.674138	2827.390146
HMGCR	1587.16786	4707.92696	2048.994838	32048.6488	1917.47481	2423.712844
COL4A3BP	1356.66225	1650.5677	2469.224322	3607.44667	1252.454645	2604.925019
FAF2	2963.36449	3021.80857	2082.091671	1820.31739	4646.072701	2875.837221
HAND1	16.6042176	27.4248173	0	0	2.015212623	0
PCDHB3	4.06314972	4.18482397	11.12254173	8.57862284	345.3469871	28.77649343
PCDHB5	3.90687474	10.2385984	5.135023773	5.02850108	20.15212623	9.060608762
PCDHB6	11.7206242	9.14160575	6.017605984	0	21.37132986	1.812121752
PCDHB7	1.95343737	11.1730737	4.011737323	0	19.14451991	1.812121752
PDE8B	52.7428089	52.8181666	21.06162094	10.0570022	12.09127574	15.4030349
CLK4	447.337157	344.333817	806.1084683	667.784943	376.2301206	580.7850216

PCDHB15	11.2713336	16.3025303	11.03227764	5.03855808	19.73900764	0.906060876
HAVCR1	0	0	0	0	0	0
GRM6	0	0	0	0	0	0
ITK	4.88359342	9.14160575	0	2.01140043	0	0
RNF130	1364.476	1368.19366	1845.399169	1522.63013	1874.147739	1633.62776
THG1L	355.525601	401.214919	80.23474646	150.855032	434.2783202	162.1848968
ARSB	915.185407	967.994476	68.19953449	219.242647	429.2402886	159.4667142
CLINT1	2874.48309	2684.58489	2097.135686	2039.56004	2828.350916	2410.121931
THBS4	6.83703079	2.03146794	1.002934331	0	2.015212623	5.436365257
CNOT6	2275.75453	2395.10071	2559.488412	2385.52091	2991.583138	2957.3827
IL12B	1.95343737	2.03146794	0	0	0	0
BTNL8	0	0	0	0	1.007606311	0
TTC1	1787.39519	1702.37014	966.8286948	701.97875	988.4617914	827.23358
MSH3	808.72307	907.050437	545.5962759	824.674177	864.5262151	755.6547707
RASGRF2	0.97671868	2.03146794	3.008802992	8.04560172	2.015212623	14.49697402
GABRG2	0	0	0	0	0	0
CCNG1	2251.33657	2336.18814	3412.985527	5143.1509	2062.570119	1996.958171
POLR3G	805.792914	847.122133	64.18779717	49.2793106	281.1221608	215.6424885
DROSHA	2940.89996	2889.76315	1393.075785	1677.50796	2267.1142	2321.327965
CDH6	0	0	0	0	0	0
LMNB1	4655.04125	4171.61942	2070.056459	4031.85216	5234.514787	4692.489278
ARRDC3	810.676508	885.720024	759.2212883	1079.11633	477.6053916	1100.863965
GOLPH3	3609.95226	3724.69648	3139.184455	3270.5371	2745.727198	2966.443309
SUB1	3587.48773	3674.92551	2986.738437	2723.43618	2514.985353	3120.473658
NPR3	16.6042176	18.2832115	272.798138	499.833007	52.39552819	539.1062213
FAM172A	426.826065	432.702672	616.8046134	660.745042	581.3888416	498.3334819
SLC27A6	0	0	0	0	148.1181278	7.248487009
TARS	14901.797	12650.9666	9635.190115	4828.36673	5025.940281	5033.168167
IRX4	0	0	95.28879076	112.658538	203.5364749	98.7606355
LNPEP	2953.5973	3077.67394	2685.858138	2931.61613	2573.426519	5149.143959
PDE4D	1044.11227	1053.31613	21.06162094	23.131105	45.34228401	31.71213067
RAD1	1551.02927	1736.90509	855.5029841	1306.40458	994.5074292	1282.07614
BRIX1	2160.50173	2077.17597	914.6761096	749.246661	1172.853746	1089.991234

AGXT2	0	0	0	0	0	0
PRLR	71.3004639	153.37583	3130.158046	4336.57933	1650.459138	6772.805049
SLC12A7	710.074483	560.685153	2027.933217	1969.16102	914.9065306	2449.082548
IL4	0	0	0	0	0	0
RAD50	2720.16153	2622.62512	3200.363449	3172.98418	2267.1142	3168.494884
IL5	0	0	0	3.01710065	0	0.906060876
ST8SIA4	42.9756221	123.919545	0	0	105.7986627	11.77879139
GNPDA1	1770.79097	2671.38035	1213.55054	2058.66834	729.5069694	949.5517982
PCDH12	0	3.04720192	0	1.00570022	1.007606311	0.906060876
SKP1	5474.27381	5727.0027	4969.308934	4699.56671	5652.107147	5675.239146
NUP155	5320.18667	4538.29939	1555.551147	1649.34835	3607.230594	3095.103953
PPP2CA	6573.65859	6089.05092	4951.717466	4228.91912	5491.000974	4916.97492
FGF1	135.763897	132.045416	1.002934331	0	4.030425245	0.906060876
NR3C1	3993.8027	3370.20532	1572.601031	2348.31	1152.70162	3473.837399
C5orf15	2556.0728	2617.54645	1644.812302	1566.88094	1315.933843	1764.100526
PPWD1	1020.67102	1039.09585	537.5728013	494.804506	685.1722917	567.1941085
LIFR	2451.5639	1788.70753	266.780532	160.912034	216.6353569	511.0183342
TRIM23	594.821678	626.707861	551.6138819	538.049615	362.7382721	495.6152993
TRAPPC13	490.478821	468.426036	915.9899536	843.762367	583.8574771	932.5450356
C9	0	0	0	0	0	0
SEC24A	1759.07035	1861.84037	1442.219568	1519.61303	1832.83588	1600.103507
TXNDC15	1342.01147	1677.99252	1146.35394	983.574811	763.765584	753.842649
TTC33	429.756221	388.010377	234.6866334	245.390853	136.026852	164.9030795
RARS	3276.89118	3372.23679	1287.767681	1443.17981	2696.354489	1941.688458
WWC1	4982.24201	3941.04781	4755.914596	2811.9378	3003.674414	5253.34096
H2AFY	9187.01594	8919.16001	10938.00181	10913.8587	7925.831245	10847.36081
TCERG1	3446.84024	2931.40824	2495.300615	1809.25469	2743.711986	2856.809943
DPYSL3	1.95343737	5.07866986	1.002934331	0	10.07606311	1.812121752
SMAD5	3014.15386	2674.42755	1688.941413	1571.90944	2163.33075	1987.897562
CSNK1A1	7393.54556	7413.18204	7916.461553	6405.50581	5953.955769	9527.547234
HMGXB3	2964.34121	3323.48156	2478.250731	2319.1447	1810.668541	2577.743193
ERGIC1	3141.12729	2847.10232	22686.37456	18800.5598	3846.03329	15837.03805
PDGFRB	611.425896	613.503319	3.008802992	0	6.045637868	4.530304381

CDX1	0	0	0	0	0	0
ATP6V0E1	2269.89422	2861.3226	1866.460789	1918.87601	2663.103481	1813.933874
BNIP1	357.479038	357.538358	262.7687946	292.658763	381.882792	299.0000891
STC2	21.487811	18.2832115	2505.329958	261.482056	14281.81186	1513.121663
CPEB4	804.816196	959.868604	1546.524738	1837.41429	485.666242	2083.033954
HRH2	0	0	0	0	32.24340196	0.906060876
DBN1	2562.90983	1915.67427	1063.110391	913.175796	2647.989386	2737.209907
ZNF346	429.756221	443.875746	528.5463923	486.758904	720.4385126	664.1426222
UNC5A	4.88359342	2.03146794	177.5193765	137.78093	265.0004599	84.26366148
EHHADH	125.99671	134.076884	68.19953449	33.1881071	231.7494516	57.0818352
CNTN3	0	0	17.04988362	28.159606	0	16.30909577
SMC4	9048.32189	8838.91703	5431.892335	6716.06604	7648.739509	8439.957062
SELK	550.869338	668.352954	1125.292319	745.22386	406.0653434	590.7516913
ACTR8	908.348376	937.522456	438.2823025	580.289024	627.7387319	646.0214047
TBCCD1	326.22404	340.270881	366.3618817	404.291487	340.5709332	472.5469894
TIMMDC1	799.932602	822.744518	1132.312859	941.335402	1373.367402	1002.103329
CRBN	662.215268	675.463092	639.872103	619.511333	548.1378333	504.675908
KNG1	0	0	0	0	2.015212623	0
HRG	0	0	0	0	1.007606311	0.906060876
BCL6	707.144327	591.157172	1072.1368	422.394091	213.612538	633.3365524
HGD	1.95343737	0	1067.122128	372.10908	4.030425245	821.7972147
CLDN16	0	3.04720192	0	0	0	0.906060876
ARL6	181.669675	286.43698	117.3433167	113.644124	103.7834501	139.5333749
NPHP3	508.372308	519.639343	316.9272485	354.338357	512.80108	372.300414
CD86	0	0	0	1.00570022	0	0
AMOTL2	6183.44971	4772.93394	2497.306483	1432.11711	2576.157132	1323.392516
NIT2	1120.29633	1109.1815	1108.242435	913.175796	1421.732505	989.4184768
FAM162A	657.331674	551.543547	441.2911055	399.262986	796.0089859	737.5335532
OGG1	662.215268	673.431624	455.3321861	606.43723	702.301599	504.675908
KPNA1	2745.55622	2716.07264	1597.674389	1983.24083	3535.690546	2529.721966
PCCB	835.094475	793.288232	1075.145603	940.329702	2193.55894	1783.127804
UBE3A	2815.87997	2670.36461	4191.262568	4266.18031	3336.184497	4364.495241
ARMC8	918.115563	915.176309	653.9131836	676.836245	1185.952628	855.3214671

CEP70	327.200759	337.223679	462.3527265	433.456793	350.6469963	472.9637774
RBP2	0	0	0	0	0	0
RBP1	0	1.01573397	0	0	0	0.906060876
SLC25A36	1871.393	1594.70234	6344.562576	3405.30093	2234.870798	1989.709684
GRK7	0	0	0	0	3.022818934	1.812121752
RNF7	868.30291	981.199017	1081.163209	1198.79466	1657.512382	906.9669371
TFDP2	1577.40067	1405.77582	717.0980465	797.520271	899.792436	683.1699006
XRN1	1292.19882	1036.04865	1254.670848	1314.45018	1556.751751	1533.961063
KAT2B	929.836187	908.066171	532.5581296	768.354965	1044.887745	536.3880387
BCHE	64.4634331	103.604865	0	0	4.030425245	2.718182629
SERPINI2	0	1.01573397	0	0	0	1.812121752
PDCD10	1216.01476	1174.18847	1430.184356	1459.27101	1394.527135	1158.851861
LRRC31	0	0	2617.658603	1056.99093	3.022818934	1431.576184
WNT5A	7.81374947	8.12587178	500.464231	253.436454	12.09127574	591.6577521
PFKFB4	925.528858	1056.49538	341.4489929	616.805999	421.1794381	429.9258857
COL7A1	3675.39241	6282.31462	925.7083872	634.596836	266.0080662	393.2304203
FGF12	1.95343737	1.01573397	50.14671654	51.290711	143.0800962	14.49697402
PRKAR2A	4198.91362	4607.3693	2405.036525	2882.33682	3092.343769	3132.252449
HES1	198.273893	251.902025	2485.271271	2472.01113	1121.465824	915.1214849
USP4	1278.52476	1265.60453	1149.362743	1424.07151	792.986167	1783.127804
ACAP2	2223.98844	2145.23015	1583.633308	1900.77341	2127.056923	2639.355332
ECT2	6245.13926	5102.03174	3092.046542	5618.8471	3577.002405	5815.098703
GNAT1	0	0	0	2.01140043	1.007606311	1.812121752
GNAI2	8265.97022	8209.16196	3866.311845	4831.38384	3094.358982	4648.092295
TFG	3855.10865	3959.33102	3354.815336	2333.2245	3837.97244	3142.219119
USP9Y	0	0	0	0	0	0
HYAL1	12.6973429	13.2045416	9.026408976	7.03990151	5.038031556	3.624243505
TUSC2	643.657613	698.824973	575.6843058	544.083817	596.5029363	863.476015
NPRL2	532.311683	500.756848	737.1567331	524.975513	180.3615297	560.8516824
RPL24	8921.28986	9139.50318	9175.765957	7358.66825	10251.28585	6893.247722
CYB561D2	337.944665	415.435195	456.3351205	534.258126	257.9472157	477.6028091
C3orf14	345.758414	427.624002	869.5440647	773.383466	2160.307931	661.4244396
FXR1	4807.40936	4576.89728	3593.513707	4130.41079	3143.731691	3337.928268

CBLB	254.923576	358.554092	539.5786699	513.91281	386.9208235	419.5061857
BBX	5334.83745	5219.85688	1855.428512	1735.83857	2140.155805	2889.428134
IFT57	834.117756	943.61686	0	0	965.2868462	90.60608762
GNB4	2620.53623	2391.03777	0	0	152.148553	52.55153082
HHLA2	0	0	0	0	0	0
IQCG	83.0210881	126.966747	400.170798	404.291487	55.41834712	268.1940193
GBE1	2911.5984	2914.14077	419.2265502	809.588674	932.0358379	653.2698917
MORC1	0	0	0	0	0	0
UMPS	940.580093	904.003235	814.3826765	698.96165	1697.816635	1233.148852
NCBP2	2481.84218	2563.71255	3438.058886	3155.88728	2177.437239	3702.16474
SNX4	538.171995	576.936896	1308.829302	996.648914	1336.085969	1033.81546
C3orf52	219.761704	209.241198	944.7641395	1168.62365	35.2662209	711.2577878
FRMD4B	89.8581189	123.919545	192.5633915	157.894934	111.8443006	222.8909755
SLC41A3	979.64884	813.602912	870.5469991	1022.79712	675.0962286	819.9850929
ROPN1B	0.97671868	2.03146794	2.005868661	0	3.022818934	0
PLXNA1	11589.7439	9525.55319	2262.61985	2354.3442	5676.853958	2778.888707
ATP6V1A	1550.05255	1525.63243	3022.844073	4348.64773	3722.097714	4194.155796
ABTB1	692.493547	676.478826	71.20833748	94.5358203	83.63132384	82.45153973
PODXL2	68.3703079	77.1957819	0	0	1001.560673	112.3515486
UPK1B	0	1.01573397	0	0	5.038031556	3.624243505
CSPG5	167.995614	236.666016	158.4636243	181.026039	149.1257341	514.6425777
KLHL18	843.884943	906.034703	451.3806249	530.004014	737.5678199	855.3214671
SCAP	3170.42885	3414.89761	3699.824746	2944.69023	2011.182197	3673.170792
EFCC1	0.97671868	1.01573397	310.9096425	146.832231	13.09888205	184.8364187
KIAA1257	2.93015605	3.04720192	177.9807263	105.739321	55.41834712	199.1249988
NEK11	120.136398	90.4003235	180.5281795	124.706827	93.70738695	135.9091314
MRPL3	2439.84327	2245.78781	2331.822319	1722.76447	4063.676253	2518.849236
PLSCR4	394.594348	234.634548	5.014671654	9.05130194	12.09127574	4.530304381
HEMK1	461.011219	483.489371	1215.556409	884.010489	335.5329017	685.8880833
CISH	14.6507803	18.2832115	1056.08985	1093.19613	244.8483336	870.724502
MAPKAPK3	2445.70358	2942.58132	976.8580381	1194.77186	999.5454608	1471.442863
ACVR2B	787.235259	569.826758	496.4524937	382.166082	596.5029363	372.3910201
WDR48	1120.29633	1064.4892	634.8574313	659.739341	688.1951106	857.1335889

COMMD2	802.862758	884.70429	866.5352617	708.012952	891.7315855	617.0274567
GORASP1	907.371657	916.192043	1366.999493	1371.77509	1159.754864	1496.812567
PEX5L	0	0	0	1.00570022	0	0.906060876
RRP9	781.374947	956.821402	492.4407564	432.451093	650.9136771	540.9183431
ABCC5	1402.56803	1659.70931	8642.285128	15032.2011	2294.319571	5401.028883
AADAC	0	0	0	0	0	4.530304381
ABHD14B	1118.34289	1203.64476	1363.99069	1567.88664	505.8183683	997.5730247
EIF1B	612.402615	613.503319	695.0334912	1252.09677	526.9781008	751.1244664
ABHD14A-ACY1	43.805833	7.414858	12.04524131	15.2363583	2.972438618	17.44167187
ARHGEF26	988.439308	752.658873	824.4120198	1099.23034	867.549034	1064.62153
KLHL24	565.520118	603.34598	1151.368612	2465.97693	210.5897191	562.6638041
PLCH1	51.7660902	88.3688556	490.4348877	733.155457	702.301599	738.4396141
VIPR1	663.191986	1205.67623	872.5528677	741.201059	167.2626477	1020.224547
DNAH1	74.23062	93.4475254	339.9947381	212.202745	79.60089859	131.378827
SSR3	7233.57857	6892.77074	5717.728619	3337.91902	4923.164437	3742.937479
ZBTB47	486.405905	513.96139	358.0475561	296.681564	53.4031345	500.1456037
TNNC1	3.90687474	1.01573397	3.008802992	7.03990151	2.015212623	6.342426133
NKTR	4598.39156	3320.43436	5659.558428	3552.13316	2256.030531	5033.168167
CLCN2	285.201856	365.66423	351.0270157	383.171782	251.9015778	695.8547529
FOXP1	2415.42531	2556.60241	1846.402103	1475.36222	1451.960695	1642.688369
EIF4G1	29851.4531	28548.219	23176.80945	20772.738	33216.74966	35079.05288
SPCS1	1468.00818	1569.30899	1964.748354	1445.19121	1366.314158	1798.530839
NEK4	1226.75867	1311.31256	1287.767681	1180.69205	933.0434443	1577.451985
SLC4A3	181.669675	176.737711	96.28169575	72.4104155	194.4680181	91.51214849
INO80D	703.237452	632.802265	598.7517954	704.995851	1204.089542	910.5911806
EEF1B2	6698.19023	6626.08978	7280.581128	5754.93846	8083.048057	5204.549582
ADAM23	0.97671868	1.01573397	0	0	49.37270925	6.342426133
DGUOK	1168.15555	1165.04687	744.1772734	643.648138	972.3400904	783.7426579
MOB1A	6373.08941	6112.68704	3131.16098	2797.858	5661.739863	3790.052645
KANSL3	2755.32341	2333.14093	2344.860465	1457.25961	1684.717752	1774.973256
LMAN2L	710.074483	844.074931	867.5381961	455.582198	609.6018183	750.2184055
RTKN	540.125432	552.559281	1118.271779	1351.66109	251.9015778	1137.1064
TTL	5034.0081	5241.1873	1105.233632	1485.41922	1445.915057	1506.779237

IL1A	1039.22868	1089.88255	0	0	134.0116394	85.16972236
CCL20	5.8603121	18.2832115	0	2.01140043	4.030425245	2.718182629
PIKFYVE	1877.25331	1793.78619	1361.984821	1943.01282	995.5150356	1752.321735
KCNIP3	636.820582	470.284829	41.12030756	16.0912034	93.70738695	19.0272784
FAHD2A	595.896069	683.324872	480.8167475	652.789953	557.5891805	410.9439104
NCL	21576.6924	20877.3961	8273.205294	9078.45585	24067.68435	15657.638
ACTR1B	2567.79342	2231.56754	2347.869268	2474.02253	1669.603658	1689.803534
SLC35F5	1351.77866	1359.05205	1252.664979	1551.79543	1699.831847	1786.752048
ZAP70	1.95343737	4.06293589	8.023474646	5.02850108	1.007606311	11.77879139
ACTR3	9019.02033	8670.30519	7466.846092	5535.37399	6021.455316	6315.244307
STEAP3	2748.48638	2964.92746	148.4342809	138.78663	1166.808108	706.7274834
EPB41L5	796.025727	798.366902	1852.419709	1320.48438	4824.419018	1756.852039
TFCP2L1	109.392493	125.951013	0	0	13.09888205	2.718182629
SF3B6	2122.4097	2053.81409	1892.537082	1560.84673	2017.227835	1577.451985
TP53I3	334.03779	457.080288	334.9800665	754.275162	278.0993419	311.6849414
DNAJC27	198.273893	192.989455	261.7658603	239.356651	215.6277506	200.2394536
POMC	0	0	0	0	0	0
STAM2	1059.73977	1158.95246	1166.412627	1219.91436	1129.526675	1070.963956
OTOF	0	0	0	0	3.022818934	0
GPD2	2767.04403	2405.25805	1351.955478	1599.06334	3369.435505	2037.730911
CENPA	1041.18212	957.837136	275.8069409	569.226322	753.6895208	482.930447
CYTIP	0	0	0	0	0	0
ACVR1	840.954787	998.466495	296.8685619	364.063478	521.9400692	520.9850038
TANC1	2137.06048	1865.90331	2255.59931	2440.83442	1313.91863	2819.661447
SLC30A3	61.5332771	87.3531216	0	8.04560172	101.7682374	19.0272784
MPV17	340.874821	401.214919	845.4736408	876.970588	922.9673811	841.730554
GTF3C2	1772.74441	1779.56592	1913.598703	1802.21479	3007.704839	2594.958349
EIF2B4	1145.69102	1152.85806	1541.510066	1292.32478	1134.564707	1626.379273
NRBP1	4043.61535	4671.36054	2089.112211	2583.64385	6927.29339	2719.99475
ITGB6	8.79046815	22.3461474	1.002934331	6.03420129	124.9431826	112.3515486
FNDC4	116.229523	139.155554	0	0	1.007606311	2.718182629
ITGA4	0	0	0	0	0	0
PSMD14	3717.39131	3722.66501	2681.8464	1869.5967	3457.097254	2154.612764

SNX17	3189.96322	3219.87669	3637.642817	2627.89466	3027.856965	3004.497865
ASB3	233.494369	247.910191	275.2653564	237.274852	341.0948885	252.6369541
PPM1G	7058.74593	6363.57334	2346.866334	2763.66419	8562.638433	4682.522608
PDE1A	0	0	2.005868661	3.01710065	6.045637868	1.812121752
REEP6	153.344833	160.485968	2510.34463	3012.07215	428.2326823	4683.428669
PCSK4	53.7195276	77.1957819	259.7599917	282.601761	27.2053704	190.272784
GCG	0	0	0	0	0	0
APC2	77.160776	103.604865	65.1907315	87.4959188	74.56286704	270.0061411
IFIH1	203.157486	354.491156	505.4789027	551.123718	165.2474351	1022.036668
RPS15	9823.31886	10278.2629	9672.168304	10318.8563	7639.509835	9109.073958
GCA	306.689667	277.295374	419.2265502	411.331388	439.3163517	461.184986
INO80B	252.892002	241.094616	577.178678	611.143907	397.9238845	550.1782852
MOGS	2488.67921	2782.09535	1652.835777	1879.6537	2225.802342	1977.024832
TTC31	840.954787	772.973553	597.7488611	653.70514	780.8948912	634.2426133
NDUFS7	878.070097	954.789934	1877.493067	1755.95258	680.1342601	1623.66109
PCGF1	344.781695	354.491156	476.3938071	403.285786	396.9968866	270.0061411
GRB14	6.83703079	11.1730737	4.011737323	10.0570022	238.8026958	24.46364366
CLIP4	2684.02294	2608.40484	0	0	198.4984433	117.7879139
TLX2	1.95343737	0	28.08216126	6.03420129	0	12.68485227
SPTBN1	26814.8347	21394.4047	3426.023674	4774.05892	6984.72695	6894.217207
AUP1	2958.48089	3243.23857	1334.905594	1903.79051	3310.994339	2190.855199
RTN4	15499.5488	14764.709	3636.639883	4055.98897	4777.061522	4048.279995
HTRA2	818.490257	912.129107	647.8955776	673.819144	659.9821339	707.6335443
LOXL3	105.485618	85.3216537	11.03227764	33.1881071	118.8975447	53.45759169
DOK1	575.287305	652.10121	28.08216126	47.2679101	205.5516875	78.82729623
GALNT3	371.1531	588.10997	1564.577556	1720.75307	1745.174131	1600.103507
POLE4	676.866048	691.714835	423.2382876	464.6335	911.8837117	443.9698293
TACR1	0	0	0	0	2.015212623	0.906060876
CCDC88A	4904.10451	4876.5388	1.002934331	3.01710065	293.2134366	142.2515576
ACADL	9.76718684	11.1730737	0	0	10.07606311	0
EVA1A	711.051202	797.351168	0	1.00570022	20.15212623	19.93333928
MRPL19	1748.32644	1639.39463	1199.50946	1283.27348	2496.848439	1970.682406
LANCL1	1224.80523	1201.61329	1778.202568	1570.90374	1223.234062	1604.633812

WDR75	1633.07364	1849.65156	709.0745718	837.74828	1495.287766	1126.233669
EFEMP1	1752.23332	1115.2759	0	0	690.2103232	27.18182629
REG1A	0	0	0	0	0	0
FANCL	414.128722	323.003403	563.6490939	324.84117	487.6814547	442.1577076
FN1	13445.5094	13969.3893	83.24354945	105.598523	3495.386294	1008.445755
STAT1	3727.1585	3731.80661	1953.716076	1801.20909	3623.352295	7614.535603
GLS	3975.24504	4574.86581	217.6367498	233.32245	1445.915057	405.0092117
PAPOLG	594.821678	679.526027	366.0710307	457.593598	495.7423052	387.794055
DNAH6	0	5.07866986	3.008802992	28.159606	4.030425245	16.30909577
PECR	195.373038	198.068125	1469.389059	310.761367	279.2177849	288.1273586
UNC50	573.333867	778.052223	795.3269243	657.727941	513.8792188	701.2911182
IGFBP2	0.97671868	1.01573397	1417.146209	2259.80838	3711.014044	2362.100704
ELMOD3	441.476845	410.356525	1271.720731	1081.12773	472.56736	1031.097277
IGFBP5	2.93015605	2.03146794	34973.32305	21482.7623	763.765584	79108.1751
USP34	6180.67583	5379.32712	3765.015477	4723.77391	6787.236113	5806.926034
EFHD1	284.225137	235.650282	2002.859858	1142.47544	322.4340196	1896.385414
KCNJ13	0	0	6.017605984	189.071641	0	22.6515219
CCT4	9131.34298	9458.51475	5585.341288	4746.90502	9703.248778	6681.292901
GGCX	1615.4927	1822.22675	1890.531213	2277.91099	1388.481497	2843.219029
NEU2	0	0	0	0	0	0
EHBP1	2287.47516	2081.23891	566.6578969	635.602536	1273.614377	736.6274923
OTX1	77.160776	78.2115159	85.24941811	93.53012	162.2246161	85.16972236
TXNDC9	1560.79646	1603.84394	701.0510972	624.539834	1060.99937	713.0699096
COQ10B	847.791818	1072.61507	711.0804405	818.639975	1054.963808	641.4911003
GNLY	0	0	0	0	0	0.906060876
SF3B1	10446.0063	9255.36796	7348.499841	6273.55794	9662.944525	7918.065997
ST3GAL5	251.016702	380.90024	1221.574015	1076.09923	65.49441023	792.8032667
CHST10	706.167608	711.013781	0	0	236.7874832	45.30304381
PDCL3	867.277355	787.132884	780.8646112	776.169255	1215.142983	703.8371492
MOB4	1585.58558	1501.71189	1093.519359	1027.24231	1416.08991	1090.172446
HSPE1	2813.9949	3028.96949	3275.162292	1972.23846	3427.372868	3240.644512
KDM3A	2709.41763	2564.72828	1627.762419	2159.23836	1157.739652	2025.046058
PLCD4	22.4645297	20.3146794	247.7247797	745.22386	5.038031556	428.5667944

CHMP3	4387.33242	4227.01755	1994.836384	2363.91847	3594.423918	1987.951926
ZNF142	1113.4593	1195.51889	935.7377306	1137.44694	1765.326257	1643.594429
IL1R2	1.95343737	1.01573397	25.07335827	42.2394091	1.007606311	30.80606979
PRKAG3	1.95343737	0	0	0	0	1.812121752
SMYD1	0	0	0	0	0	0
IL1R1	385.80388	367.695698	27302.88128	31315.4933	278.0993419	23794.97073
WNT6	0	0	0	0	2.015212623	7.248487009
IL1RL2	0.97671868	0	118.346251	226.282548	0	231.9515843
IL1RL1	0	0	0	0	8.06085049	4.530304381
IL18R1	1.95343737	2.03146794	0	0	3.022818934	0
IL18RAP	0	0	0	0	0	0
SLC9A2	2.93015605	8.12587178	232.6807647	222.259748	1460.021545	462.0910469
FHL2	2373.4264	1634.31596	0	2.01140043	460.4760843	96.04245287
MLPH	1185.73648	1310.29682	16042.93755	11292.002	7063.320242	6755.589893
CNPPD1	829.234163	796.335434	1174.436101	1375.79789	1495.287766	1407.112541
UXS1	2617.60607	2409.32098	475.3908728	693.933149	1329.032725	627.9001872
ABCB6	271.95755	311.759228	1132.894561	928.261299	1203.484978	1528.25288
STK16	536.218557	518.024326	646.8926433	694.938849	405.0577371	656.8941352
SLC5A7	0	0	0	0	0	0
HDLBP	15336.4368	15944.9919	18741.83384	14155.2305	22959.31741	17521.40522
PPP1R7	575.287305	783.130893	1499.386824	1717.73597	1922.512842	1349.124645
PASK	430.73294	312.846063	381.1150457	756.286562	1135.572313	798.2396319
STK25	2442.77343	2094.44345	1429.181421	1544.75553	2889.814901	1794.000535
TPO	0	1.01573397	0	0	0	0.906060876
PROC	0	0	0	0	0	0
ID2	0.97671868	2.03146794	3922.476167	12299.7136	565.2671406	5040.416654
TAF1B	1192.57351	1196.53462	227.6660931	462.622099	575.3432037	421.3183074
HPCAL1	8070.62648	9451.40461	700.0481628	904.124494	1646.428713	1149.791252
ODC1	4098.3116	4437.74172	1184.465445	642.642438	3803.713825	1954.37331
BIRC6	5235.21215	4719.10004	3960.587672	4013.74956	8517.296149	6213.765489
NOL10	1560.79646	1588.60793	834.4413631	735.166858	1827.797849	1226.806426
PLEKHB2	6771.59064	7120.29515	2915.530099	2782.7725	5917.671866	3565.349548
GORASP2	3447.81695	3386.45706	3396.938578	2660.07707	3595.139319	3328.867659

STRN	2063.80658	2081.23891	1709.0001	1670.46806	2011.182197	1889.136927
CEBPZ	2821.74028	2694.74223	1226.588686	1313.44448	2076.676608	1775.879317
PRKD3	3607.0221	3467.71578	0	0	722.4537252	86.98184411
DCAF17	1060.71649	967.994476	542.5874729	579.283324	661.9973465	652.3638308
QPCT	140.64749	302.688724	0	0	70.53244179	13.59091314
RAB3GAP1	2772.90434	2565.74401	5285.463923	2452.90283	2049.471237	2752.612942
SLC25A12	772.584479	962.915806	378.1062427	397.251585	387.9284298	579.8789608
RMDN2	66.4168705	100.557663	0	0	23.17494516	2.718182629
DLX2	6.83703079	16.2517436	5.014671654	7.03990151	9.068456802	7.248487009
LCT	0	1.01573397	1.002934331	8.04560172	8.06085049	23.55758278
DARS	4217.47128	3817.12827	1573.603965	1257.12527	2493.82562	1537.585307
SRSF7	3704.69397	3150.80678	2751.048869	2080.79375	3497.401506	3135.876692
SDC1	4279.98127	4484.46549	2572.526558	3566.21296	7614.480894	4599.165007
PLCL1	8.79046815	6.09440383	0	0	9.068456802	1.812121752
SLC1A4	434.639814	343.318083	3818.170997	2636.94597	350.6469963	1218.651878
SOS1	2128.27001	1893.32812	1702.982494	1613.14315	3115.518715	1890.042988
KYNU	1179.87617	2025.37354	50.14671654	29.1653063	3326.108434	366.048594
RP11-153K16.2	0	0	0	0	0	0
WIPF1	1247.26976	1106.1343	0	1.00570022	533.0237387	76.1091136
ORC2	1217.9682	1251.38425	1033.022361	887.02759	1823.767423	1244.927644
COX7A2L	2018.87752	2245.78781	3929.496708	2491.11943	2743.711986	1512.215602
PNO1	1187.68992	1324.5171	497.455428	457.593598	1406.618411	801.8638754
ORC4	1283.40835	1388.50834	805.3562676	781.429067	952.1879642	789.1790232
PLEK	0	0	1.002934331	0	0	0.906060876
RND3	2819.78684	1894.34386	788.3063839	323.835469	1000.553067	1119.891243
ATF2	2241.56938	2148.27735	1058.095719	1564.86954	888.7087666	880.6911716
THADA	1491.44943	1436.24784	798.3357272	701.97875	1845.934762	1114.454878
AAK1	3559.16288	3525.61262	971.8433665	1458.26531	2140.155805	1680.742925
TRAK2	1756.14019	1910.5956	1890.531213	1941.00142	1963.824701	1591.948959
C2orf42	256.877014	261.043631	131.3843973	159.906334	233.7646642	167.6212621
TIA1	1362.52256	1304.20242	2347.869268	1718.74167	1529.546381	1833.867213
PCYOX1	3600.18507	3998.94465	2273.591952	3452.56884	3440.975553	3497.394982
KISS1R	0.97671868	0	4.011737323	3.01710065	1.007606311	1.812121752

EPAS1	4120.77613	3324.49729	2464.209651	3785.45561	2798.122726	3763.77688
ARID3A	242.226234	237.68175	651.907315	531.009714	921.9597748	814.5487277
SUMO1	3673.43897	3359.03225	2638.720224	2200.47207	2681.240394	2508.882566
CD207	6.83703079	5.07866986	0	0	2.015212623	0
GRIN3B	5.8603121	1.01573397	1.002934331	2.01140043	32.24340196	8.154547886
VAX2	31.2549979	24.3776153	0	0	55.41834712	2.718182629
ATP6V1B1	25.3946858	30.4720192	0	0	57.43355974	19.93333928
NFE2L2	3231.47377	3331.13003	1672.824258	1349.64969	2722.552253	2776.170525
MSH6	5362.18557	4803.40595	2944.615195	3530.00776	4317.593044	4805.746887
PLEKHA3	718.864951	803.445572	359.0504904	420.38269	608.594212	352.3670747
SPR	1079.27415	1308.26536	661.9366583	967.483607	1530.553987	1050.124555
EPHA4	154.321552	170.643307	596.7459268	215.219846	568.2899596	523.7031864
PARD3B	299.852636	271.200971	472.3820698	482.736103	173.3082855	537.2940996
FARSB	1437.7299	1321.4699	1368.002427	1003.68882	1970.877945	2008.736962
ALMS1	1954.41409	1681.03972	771.2565003	1070.01474	2355.783556	1475.067106
BCL9	644.634331	723.202588	803.3503989	1478.37932	1174.868959	1182.409443
PRRX1	0	1.01573397	0	0	0	0
DHCR24	6050.77225	12746.4456	15114.22036	53569.6277	10661.48238	24007.89504
DNAJC16	642.680894	773.989287	692.0246882	795.508871	502.7955493	922.3699719
MARK1	131.857022	113.762205	139.407872	25.1425054	35.2662209	23.55758278
TNR	0	0	5.014671654	2.01140043	0	5.436365257
MORN1	180.692957	164.548904	168.4929676	125.712527	170.2854666	191.1788449
GPX7	32.2317166	53.8339005	0	0	0	0.906060876
CACYBP	3231.74725	3148.53154	3294.488836	2878.27379	4169.978719	3455.679939
SCP2	2141.94407	1964.4295	7755.079389	6226.29003	1624.261374	5042.781473
TPSG1	0	0	0	0	4.030425245	0
PAPPA2	0	0	0	2.01140043	3.022818934	0.906060876
RALGPS2	1668.23551	1395.61848	1239.626833	1656.38826	1409.641229	1608.258055
ANGPTL1	0	0	0	0	0	0
CEP104	705.19089	711.013781	1595.66852	1611.13175	1149.678801	1672.588377
FAM20B	2403.70468	2255.94515	2943.612261	2882.33682	2959.339736	2661.100793
TCEANC2	314.503416	399.183451	485.4202161	325.84687	372.8143352	439.4395249
TMEM59	1912.41518	1907.5484	4330.67044	4743.88792	1742.151312	4477.75285

LRRC42	1480.70552	1315.37549	949.7788112	1107.27594	779.8872849	1302.009479
WRAP73	548.9159	532.244601	921.6966499	832.719778	574.3355974	1013.88212
NPHS2	0	0	0	0	0	0
MRPL37	2470.12155	2384.94337	1901.563491	2286.96229	3066.146005	2788.855377
ICMT	4662.855	4425.55292	2886.445004	2731.48179	3152.800148	3580.752583
RPL22	6354.32665	5912.12021	12294.5407	9408.74791	6617.343613	7007.492938
CHD5	3.90687474	7.11013781	4.011737323	20.1140043	81.61611121	39.86667855
QSOX1	10756.6029	10928.2818	1936.666193	1087.16193	6590.752882	3617.901079
STXBP3	808.72307	771.957819	802.3474646	1046.93392	1175.876565	925.0881546
PHF13	928.859468	785.162361	1382.043508	1343.61549	909.8684991	1533.055002
ERRFI1	9231.945	6329.03838	1718.026509	1186.72625	850.4197267	1712.455056
PARK7	3046.38557	22964.9275	4031.796009	23362.0558	3173.959881	3653.237453
KIAA1324	57.6264023	50.7866986	5821.030855	7454.25	1081.161572	6010.807853
OPRD1	0	0	3.008802992	0	5.038031556	0
AMPD2	2070.64361	2237.66194	906.652635	721.087055	1462.036758	1766.818709
SRSF4	2683.11459	2508.86291	2291.614682	2410.66342	2152.247081	3038.928179
MECR	285.201856	290.499916	489.4319534	312.772767	247.8711526	547.2607692
KCNC4	140.64749	214.319868	36.10563591	33.1881071	40.30425245	159.4667142
EDEM3	1540.28536	1900.43826	2001.856924	1667.45096	1932.588905	2254.27946
WDR77	1416.61324	1485.74455	635.8001896	761.003296	1757.648297	1162.983498
ATP5F1	3914.52244	3925.68991	4075.714504	3988.48637	4124.938717	4431.380654
RAP1A	1433.82303	1531.72683	889.6027513	987.597612	989.4693977	1043.782129
HDAC1	2139.99064	2348.37694	2036.959626	2270.87109	2759.833687	2449.988609
CAPZA1	6976.70156	6689.62394	4947.475053	3973.52155	4996.719698	5285.959152
S100PBP	706.167608	611.471851	943.7612052	889.038991	586.4268732	790.9911449
RNF19B	472.731843	494.662444	324.9507231	784.446168	389.9436425	517.3607603
SCAMP3	1181.82961	1404.76008	2015.898005	2058.66834	1934.604118	2307.737052
TRIM62	143.577647	206.193996	371.0857024	757.292262	472.56736	541.824404
ASH1L	2339.24125	2190.93818	3796.106442	3780.42711	4881.852578	5254.247021
DLGAP3	0	0	12.03521197	21.1197045	117.8899384	33.52425242
SFPQ	10871.8557	10332.046	9798.668411	9513.92404	11770.85693	12316.92813
RHOU	68.3703079	57.8968364	667.9542643	564.197821	310.3427439	509.2062124
GON4L	1412.93102	1359.13331	2036.939567	1939.42247	1960.993327	2801.241229

ARHGEF2	2663.51185	2908.04636	4716.800157	1877.6423	678.1190475	2540.594697
LAMTOR2	487.382623	441.844278	1253.667913	1197.78896	760.742765	974.9215028
MEF2D	1389.87069	1690.18133	2391.998379	2608.78636	718.4232999	1839.303579
DOCK7	2783.64825	2558.63388	937.7435992	1310.42738	1615.192917	1060.997286
SRM	2459.37765	2659.19154	1373.017099	1387.8663	3339.207316	2985.470587
DLEU2L	13.6740616	8.12587178	48.69246176	36.2052078	6.055713931	30.80606979
FBXO2	45.9057781	38.5978909	24.07042394	36.2052078	82.62371753	86.98184411
FBXO6	106.462337	100.974114	60.17605984	24.1368052	212.6956162	39.86667855
C1orf21	966.951497	833.917591	164.4812302	151.860733	1107.359336	269.1000802
SWT1	93.7649937	171.659041	136.399069	221.254047	45.34228401	99.66669638
MAD2L2	956.207592	913.144841	429.2558935	502.850108	450.4000211	573.5365346
DNAJC6	709.097764	418.482397	166.4870989	356.017876	104.7910564	347.0213156
LEPR	157.466586	102.771963	23.13769501	39.2223084	45.78563078	64.98268604
IVNS1ABP	2056.96955	1974.58684	2060.027115	2969.83274	2789.05427	3408.601016
KIAA2013	1558.5207	1695.10764	1642.886668	1514.35321	1928.487947	1466.831013
MFN2	3838.50443	3597.72973	3902.417481	3775.39861	2856.563893	5617.577432
PRG4	0	0	0	2.01140043	53.4031345	0.906060876
MIIP	355.525601	400.199185	452.3233831	462.622099	339.5633269	392.3243594
SMG7	2230.82547	2214.30006	2884.439135	3163.93288	5756.454856	4664.401391
NCF2	67.3935892	169.627573	0	1.00570022	4.030425245	4.530304381
PDC	0	0	0	0	0	0
SLC35D1	607.519021	740.470066	1148.359809	1672.47946	898.7848297	1769.536891
PLA2G4A	17.5809363	26.4090833	0	0	3.022818934	0.906060876
GADD45A	825.327288	677.494559	1259.685519	541.066716	352.662209	528.2334908
PRAMEF1	0	0	0	0	0	0
PRAMEF12	0	0	0	0	0	0
WLS	1368.38288	1808.00647	1196.500657	1198.79466	77.58568597	1794.000535
PRDM2	563.566681	623.660659	1008.951937	986.591911	1130.534281	1009.351816
RGS2	41.0221847	57.8968364	4.011737323	10.0570022	12.09127574	23.55758278
RPE65	0	0	0	0	0	0
TROVE2	1653.58473	1612.98555	2827.271878	2742.54449	1762.303438	2970.973613
AMPD1	0	0	0	1.00570022	4.030425245	0.906060876
UCHL5	1417.21881	1240.21118	2664.796517	1684.54786	1687.740571	2480.794679

BCAS2	1154.48148	1131.52765	782.288778	649.682339	6806.380633	1166.100348
SRSF11	4419.65204	3861.82056	5547.229783	3832.72352	2936.164791	4810.277192
CTH	285.201856	207.20973	120.3521197	70.3990151	38.28903983	45.30304381
AGMAT	178.739519	155.407298	66.19366583	69.3933149	338.5557206	198.4273319
OLFML3	16.6042176	33.5192211	1.002934331	0	26.19776409	4.530304381
TNNI3K	0	0	25.49459069	6.46665239	0	7.221305183
CFHR3	0	0	0	0	0	0
PLEKHM2	2240.59266	2174.68643	1638.794696	1719.74737	1730.060036	1736.9187
CRYZ	543.055588	497.709646	510.4935743	462.622099	457.4532653	499.2395428
PHTF1	1134.94711	1483.98733	89.26115543	196.111542	1309.888205	243.7303757
ZBTB17	572.357149	585.062768	902.6408976	937.312601	431.2555012	887.0335978
CD58	396.547786	572.87396	51.14965087	61.3477131	377.8523667	96.94851375
TFAP2E	136.740616	122.903811	102.2993017	21.1197045	7.053244179	31.71213067
CD2	0	0	0	0	0	0
TTF2	1259.9671	1237.16398	383.1209143	790.480369	2835.40416	783.7426579
NR5A2	0	1.01573397	6.017605984	11.0627024	218.6505695	20.83940015
KIF21B	224.645297	257.996429	23.06748961	4.02280086	93.70738695	30.80606979
TMEM9	741.329481	808.524242	6149.993316	3815.62662	1975.915976	4205.934587
ADPRHL2	793.095571	796.335434	643.8838403	684.881847	657.9669213	810.9244842
MAP7D1	5245.95605	5153.83418	3148.210864	3040.23175	2691.316457	3704.882923
WARS2	613.379333	490.599509	230.6748961	247.402253	429.2402886	368.7667766
HAO2	0	0	0	0	0	4.530304381
RP11-268J15.5	146.507803	155.844063	28.81430332	35.5213316	84.86060354	54.89822849
OSCP1	36.1385913	39.6136249	127.37266	73.4161157	73.55526072	69.76668747
MRPS15	1255.08351	1342.80031	1666.876858	1542.74413	1448.937876	1387.179201
EXOC8	749.143231	861.342408	1450.243042	1348.64399	642.8528266	1519.464089
GNPAT	1819.62691	1682.05546	2518.368104	2258.80268	838.328451	1694.333838
TSNAX	1243.96845	1255.83317	1781.211371	1545.60032	831.0333813	1778.5975
C1orf109	506.916997	439.81281	366.0710307	357.023577	656.959315	718.5062748
RRAGC	914.208688	1462.65692	1250.65911	2072.74814	742.0516679	795.5214493
TBCE	956.207592	1085.81962	1250.65911	1395.9119	610.6094246	1106.30033
NID1	0.97671868	0	0	0	0	2.718182629
LGALS8	4582.76406	4101.53378	23935.0278	8982.91433	1284.698047	12562.53405

NT5C1A	0.97671868	0	0	0	0	0
HPCAL4	0.97671868	12.1888077	0	4.02280086	3.022818934	0
MTR	2705.51075	2014.20047	2721.963774	2571.57545	2129.072136	3309.840381
BMP8B	213.901392	204.162528	0	0	205.5214593	28.4412509
MYCL	12.6973429	17.2674775	55.16138819	81.4617175	89.6769617	131.378827
SIPA1L2	69.3470266	92.4317915	2269.64039	2266.84829	475.5901789	4277.513396
ZP4	0	0	0	0	0	0
RLF	1221.87507	1203.64476	2324.801779	2626.88896	1203.081936	1614.600481
KMO	0	0	2679.840532	1808.24899	34.25861458	1036.533642
ZNF684	50.7893716	72.117112	119.3491854	109.621323	66.50201655	132.2848879
KCNQ4	162.135302	118.840875	56.16432252	49.2793106	140.0572773	108.7273051
RIMS3	166.042176	204.162528	232.6807647	332.886771	336.540508	507.3940907
AKT3	3040.52526	2444.87167	1.002934331	0	78.59329228	35.33637417
ETV3	564.543399	621.629191	1734.073458	1788.13498	1122.473431	2064.912737
ACADM	749.143231	739.464489	401.1737323	282.601761	491.7118799	372.3910201
ST6GALNAC5	2.93015605	6.09440383	0	0	10.07606311	3.624243505
SLAMF1	0	0	0	0	0	0
CD48	0	0	0	0	0	0
ADGRL2	102.555462	96.4947274	47.13791354	99.5643213	242.833121	163.9970186
PADI2	2.93015605	2.03146794	32.09389858	44.2508095	136.026852	58.89395695
SDHB	1454.33412	1661.74078	2030.94202	1732.82147	1520.477924	1955.279371
MFAP2	259.80717	261.043631	48.14084787	18.1026039	115.8747258	65.23638308
RPF1	888.814002	852.200803	1073.139734	845.793881	742.6058514	1085.46093
KDM5B	1891.90409	1953.25643	3242.486691	3658.73738	5885.428464	5280.522786
UAP1	3559.16288	2814.59884	3841.238487	3013.07785	818.1763248	2635.731089
ACTL8	0	1.01573397	1.002934331	3.01710065	36.27382721	15.4030349
CTBS	624.123239	647.02254	868.5411304	566.209221	245.85594	578.9728999
RGS4	84.9745255	117.825141	0	0	9.068456802	6.342426133
KLHL12	666.122142	647.02254	1894.542951	1664.43386	1299.812142	1687.076291
IGSF21	0	0	1.002934331	0	0	0
SSX2IP	927.88275	894.86163	808.3650706	1061.01373	1260.515495	1192.376113
ZNHIT6	1128.11008	1113.24443	861.5205901	609.454331	460.4760843	661.4244396
PLA2G2D	0	0	0	0	0	0

RBBP5	937.649937	866.421078	1808.290598	1749.91838	1869.109707	2279.649164
GBP3	1045.63595	961.21953	1.002934331	11.0627024	84.63893015	120.5060965
GBP1	866.779229	791.937306	0	1.00570022	49.37270925	56.17577432
PINK1-AS	183.398467	223.319271	144.1918687	85.8063424	272.2249971	161.2335329
KIF17	34.1851539	16.2517436	0	2.01140043	8.06085049	1.812121752
GPR89A	522.847279	520.624605	824.3618731	777.738148	1132.458809	855.9738309
CDK18	21.487811	12.1888077	258.7570573	198.122942	381.882792	675.0153528
RAB29	841.931505	1036.04865	1109.24537	1472.34512	664.0125591	1160.663982
CD160	0	0	0	0	1.007606311	0
ECE1	7355.66841	7137.56262	2013.892136	2788.8067	3811.774676	2848.655395
HMGCL	200.22733	185.879317	375.0974397	285.618861	357.7002405	230.1394625
GALE	1421.12569	1461.64119	1312.841039	883.004789	842.3588762	1442.448915
ID3	98.6485871	62.9755063	329.9653948	2785.7896	582.3964479	2000.582415
CR2	0.97671868	3.04720192	0	0	1.007606311	0
CD46	3839.48115	4241.70507	20106.82746	18709.0411	2830.366128	14820.43775
PRPF3	1243.36288	1234.11678	2421.083474	1585.98924	4055.615403	1726.95203
APH1A	2819.78684	2853.19673	4824.114131	5495.14598	5588.184602	5206.225795
P3H1	1885.06706	2236.64621	580.6989775	798.525971	488.689061	536.3880387
SLC2A1	11118.9655	10547.3816	1362.987755	1179.68635	4223.885657	1500.436811
EBNA1BP2	2995.5962	3027.90297	998.9225934	953.403804	2801.145545	1727.858091
CDC20	4377.65314	4300.61764	885.591014	2376.41932	3121.564352	3172.979885
MPL	0.97671868	0	0	0	0	0.906060876
ARTN	235.389203	207.20973	13.0381463	19.1083041	44.3346777	28.08788716
IPO13	759.887136	881.657088	1299.802893	1402.9518	1145.648376	1682.555047
ATP6V0B	1149.59789	1514.45935	3490.211471	4432.12085	1788.501203	3269.973702
B4GALT2	1593.02817	1488.05027	1079.15734	689.910348	1506.371435	1240.397339
ERI3	1058.76305	1036.04865	1123.28645	844.788181	1036.826894	1364.52768
PTCH2	5.87007929	21.3304134	63.18486283	33.1881071	7.053244179	82.48778217
AKR1A1	764.770729	935.490988	4954.495594	9619.52256	1022.720406	3045.270605
PRDX1	5329.95386	5469.72744	9238.02812	11005.3775	12240.40147	10919.84568
PIK3R3	151.391396	292.531384	4669.662244	16373.8052	2912.989846	7797.5599
TSPAN1	26.3714045	43.6765608	3388.915103	4359.71043	145.0953088	3963.110272
BLZF1	670.029017	601.314512	1252.664979	983.574811	847.3969078	1119.891243

CCDC181	0.97671868	1.01573397	1.002934331	0	2.015212623	9.060608762
SLC19A2	446.360439	490.599509	1452.248911	1003.68882	1120.458218	1207.779148
FAAH	8.79046815	5.07866986	1313.843973	1131.41274	390.9512488	816.3608494
NSUN4	327.200759	370.7429	982.8756441	1040.89972	851.427333	1032.909399
TMED5	3777.94787	3849.63175	2309.757764	1854.5112	1299.812142	1875.546014
MROH9	0	0	0	0	0	0
DR1	3077.64057	3242.22284	1846.402103	2221.59178	1221.218849	2411.027992
FMO6P	0	0	0	0	0	0
CNN3	9204.59688	7938.97673	6545.149442	10343.6267	392.9664614	8442.675244
PRRC2C	10352.2413	10563.6333	10935.99594	10220.9313	14698.96087	14380.99823
F3	10799.5785	7150.76716	39.1144389	39.2223084	290.1906177	191.1788449
ABCD3	2199.57048	2251.88222	1175.439036	1707.67897	1128.519069	2013.267267
VAMP4	461.011219	474.347765	510.4935743	437.479594	195.4756244	336.1485851
DPH5	489.336061	509.898454	693.0276225	555.146519	316.3883817	507.3940907
FASLG	0	0	0	0	0	0
PTBP2	483.475749	384.963175	350.0240814	271.539058	333.517689	385.9819333
TNFSF4	16.6042176	20.3146794	1.002934331	0	0	1.812121752
PRDX6	3958.64083	4091.37644	4728.835369	4128.39938	5085.389053	5111.995463
DARS2	1092.94821	1038.08012	1208.535869	2055.65124	3366.412686	1981.555136
HSD11B1	1.95343737	6.09440383	0	0	0	0.906060876
IRF6	2.93015605	2.03146794	2196.426184	2998.99804	439.3163517	1996.958171
DIEXF	1165.22539	1285.91921	943.7612052	1019.78002	655.9517086	1196.906417
PLPPR5	0	0	0	0	90.68456802	7.248487009
PLPPR4	0	0	0	0	1.007606311	1.812121752
SERPINC1	0	0	0	1.00570022	0	1.812121752
RCAN3	834.117756	847.122133	612.7928761	1166.61225	207.5669001	797.333571
SYF2	661.238549	672.41589	1115.262976	1140.46404	395.9892803	703.1032399
RSRP1	425.80051	405.90761	1965.470467	1976.20092	564.1386216	1321.181727
SLC35A3	1183.78304	1183.33008	1762.155619	2323.1675	933.0434443	1850.176309
RCOR3	823.37385	698.824973	2877.418595	2075.76524	596.5029363	1993.333928
STMN1	10398.1471	9393.50778	3913.449758	7884.68969	2485.76477	5834.125982
MTFR1L	595.798397	584.047034	827.541175	759.303663	426.2174697	868.0063194
MAN1C1	82.0443694	89.3845896	54.15845386	88.501619	135.0192457	60.7060787

NEK2	1022.62446	1031.98572	967.8316291	1577.94364	444.3543833	1600.103507
RPS6KA1	1035.3218	1191.45595	392.1473233	510.895709	768.8036155	406.8213334
DHDDS	700.307296	682.573229	1019.984214	887.02759	605.5713931	1111.736695
NENF	952.300717	1005.57663	1135.321662	925.244198	532.0161324	764.7153795
NSL1	509.847153	434.73414	1194.494788	1343.61549	339.5633269	945.9275547
PROX1	0	0	8.023474646	10.0570022	0	10.87273051
ARID1A	3711.531	3057.35926	5032.724471	6178.01642	4241.014964	7432.417367
CENPF	14878.3557	13376.2007	4548.30719	12070.414	3514.530814	12127.62483
RPA2	1239.45601	1141.68498	1177.444904	1154.54385	914.9065306	1957.997553
PPP1R8	1054.85618	1088.86682	985.8844471	1103.25314	853.4425457	1353.654949
STX12	494.219654	486.536573	1258.682585	1488.43632	751.6743082	1396.23981
MARC2	234.412484	208.225464	1416.143275	822.662776	129.9812142	717.6002139
SLC5A9	0	0	0	1.00570022	0	0
OSBPL9	2285.52172	2132.02561	631.8486283	795.508871	2148.216656	744.7820402
TXNDC12	1152.34247	1165.69694	1421.669443	960.081654	1132.89208	1324.996243
ESYT2	6806.75251	5938.99654	2078.079933	2318.139	4660.17919	3188.428223
CD3EAP	1151.55133	1159.9682	359.0504904	334.898172	1039.849713	713.0699096
MESDC2	2013.99393	2073.11304	812.3768079	531.009714	2273.159838	800.0517537
RCN2	3120.61619	3215.81376	763.2330257	643.648138	2385.004139	964.9548331
CHRNB4	0	0	0	0	33.25100827	1.812121752
MUC5B	5.8603121	0	574.6813715	431.445392	1890.26944	2263.340069
CTSD	26199.6485	29071.4033	9783.87513	13456.9125	45023.76958	17138.68511
COLEC11	0.97671868	0	3.008802992	1.00570022	1.007606311	2.718182629
STAG1	1738.55926	1663.77225	919.6907813	1201.81176	2275.175051	1110.830634
A4GNT	0	0	0	0	0	0
STK11	2535.5617	2549.49227	2058.021247	1462.28811	2243.939255	1943.500579
KMT2A	10421.5884	8889.70373	3546.375793	3883.00853	6954.498761	5310.422795
TREH	1.95343737	5.07866986	2.005868661	2.01140043	0	0
IFT46	389.710755	483.489371	885.591014	1093.19613	661.9973465	1102.676086
MMP8	2.93015605	2.03146794	0	0	0	0
APOA1	6.83703079	1.01573397	1.002934331	1.00570022	4.030425245	3.624243505
ZNF541	0	2.03146794	0	2.01140043	31.23579565	2.718182629
SLC8A2	1.95343737	0	1.002934331	34.1938073	2.015212623	15.4030349

KPTN	292.038886	275.263906	153.4489526	239.356651	202.5288686	211.1121841
RPS25	10491.1698	11378.902	9666.973104	8105.13918	11337.19325	7054.952406
KIF14	2594.16482	2294.54304	425.2441562	1388.872	1876.162952	1168.81853
TNNT2	0	0	16.04694929	4.02280086	6.045637868	14.49697402
DDX59	329.154196	299.641522	1343.932003	1281.26207	491.7118799	776.4941709
CAMSAP2	2150.73454	2414.39965	2774.116359	4225.95231	4219.855232	5575.898632
ATF6	1982.73893	8925.83161	2653.764239	8582.97214	1183.937416	1178.358046
CRYGD	0	0	0	0	0	0
MREG	312.520677	327.066339	708.9843077	921.221397	2292.193521	1025.660912
TNP1	0	0	0	0	0	0
FASTKD2	861.465879	895.877364	801.3445302	656.722241	1431.808568	1001.197268
NRP2	692.493547	512.945656	0	3.01710065	65.49441023	13.59091314
CREB1	1909.48503	1535.78977	1689.944347	1525.64723	1398.55756	1591.948959
KLF7	259.80717	239.713217	3.008802992	0	395.9892803	68.86062659
TTR	0	0	0	1.00570022	0	0
B4GALT6	871.233066	1017.76544	90.26408976	110.627024	167.2626477	132.2848879
C1orf54	0.97671868	3.04720192	8.023474646	11.0627024	15.11409467	16.30909577
CA14	0	0	1.002934331	1.00570022	0	0.906060876
CASC1	0	0	16.04694929	10.0570022	4.030425245	14.49697402
LRMP	0.97671868	0	0	0	0	0
ATP10B	0	0	29.08509559	8.04560172	0	18.12121752
SPCS2	2233.824	2403.42973	5151.963334	3119.27979	2384.298814	3058.553457
USP35	353.572164	389.026111	350.0240814	507.878609	381.882792	526.4213691
ELOVL4	0	0	0	0	100.7606311	1.812121752
FILIP1	6.83703079	8.12587178	3.008802992	6.03420129	2.015212623	2.718182629
CASP8AP2	1098.80852	1041.12732	1017.978346	980.55771	1146.655982	1412.548906
HMGN3	440.500126	510.914188	955.7964172	1245.05687	651.9212834	1225.900365
UBE3D	118.182961	156.423032	81.23768079	104.592822	131.9964268	115.9757922
CNR1	0	0	0	0	0	1.812121752
SPACA1	0	0	0	0	0	0
ANKRD13C	1260.94382	1351.94192	759.2212883	823.668477	698.2711737	846.2608584
SGIP1	0	0	0	0	1.007606311	0
PHF3	4909.96482	4687.61228	3433.044214	3814.62092	2332.608611	3320.713111

ZC2HC1B	0	0	0	0	0	0.906060876
ADGB	0	0	0	0	0	0
PLAGL1	0	0	0	0	186.4071676	9.966669638
FBXO30	775.514635	731.32846	677.9836076	596.380228	536.0465576	830.8578235
TNFAIP3	845.83838	752.658873	7.020540315	13.0741028	367.7763036	83.35760061
AKAP7	115.252805	78.2115159	51.14965087	68.3876147	34.25861458	37.14849592
RAB32	1204.29414	1172.157	1.002934331	3.01710065	627.7387319	44.39698293
MYB	29.3015605	20.3146794	774.2653033	761.315063	588.4420858	1246.739766
ALDH8A1	4.88359342	3.04720192	34.09976724	26.1482056	0	43.49092206
SGK1	935.696499	855.248005	8.023474646	6.03420129	171.2930729	40.77273943
RNF146	477.615436	490.599509	919.6907813	1005.70022	481.6358168	684.0759615
ARG1	0	0	0	0	0	0
CTGF	8688.88941	8011.09384	9.026408976	44.2508095	621.6930941	356.0819243
TCF21	4.88359342	0	0	0	0	0
PMFBP1	2.93015605	5.07866986	6.017605984	4.02280086	10.07606311	16.30909577
FBXL5	1079.27415	1101.05563	1669.885661	1382.8378	706.3320242	1369.057984
MED28	1028.48477	985.261953	1073.139734	1063.02513	967.3020588	1206.873087
SLC16A7	770.631042	696.793505	0	0	1505.363829	80.63941798
TMEM5	461.987937	582.015566	396.1590606	441.502395	734.5450009	494.7092384
ZNF430	226.598735	273.232439	246.7218454	446.420269	329.8802303	385.0758724
VAMP8	1360.56913	1253.41572	2109.170897	2685.21958	1778.425139	2226.191573
DCLRE1B	1022.62446	948.69553	352.0299501	456.587898	3519.568845	725.7547618
MYL12B	10085.6362	8534.41014	3028.861679	3271.14052	11560.42843	3620.265897
FOXO3	1958.9949	2001.06703	1693.885879	4952.0578	2836.673744	2279.712589
ARMC2	28.3248418	25.3933493	48.14084787	50.2850108	34.25861458	42.58486118
GHRH	0	0	0	0	0	0
RPN2	10168.6182	11081.6576	10736.41201	7858.54148	9059.388345	10754.03654
TGIF2	1129.96585	1037.66367	483.4143474	391.328011	1527.470712	941.0620078
CASQ2	0	0	0	0	6.045637868	0
OLFM3	0.97671868	0	0	0	0	0
PKD2	1032.39165	930.412319	179.5252452	201.140043	661.9973465	258.2273497
ABCG2	36.1385913	65.0069742	1.002934331	0	716.4080873	39.86667855
SPP1	0	2.03146794	0	0	0	0

STBD1	279.810369	351.210336	18.85516542	39.7553295	334.5655996	22.71494617
CCNI	5718.68789	5797.80951	11080.41849	12881.0084	6641.133198	10903.53658
RARRES1	8.79046815	3.04720192	5.014671654	25.1425054	3.022818934	38.96061768
MFSD1	1102.71539	1236.14824	826.4178885	1398.929	1789.508809	1032.909399
RAB3GAP2	2443.78922	2376.65498	3705.200474	3488.9249	1386.496513	3466.298973
EEF2KMT	385.315521	405.60289	443.2568568	498.666395	755.5233643	737.5969775
PPL	852.675411	327.066339	695.0334912	912.170096	3095.366588	1522.182272
UBN1	2167.33876	2312.82625	3125.143374	4103.25688	3237.439078	4623.628651
BTF3P11	0	0	0	0	0	0
KLF12	684.679797	607.408915	86.25235244	108.615623	177.3387108	76.1091136
UCHL3	1138.21912	1156.52486	231.7781238	256.483726	800.1200197	430.4514011
PCDH17	0.97671868	0	0	1.00570022	12.09127574	0.906060876
HS1BP3	597.751835	976.120347	1259.685519	1583.97784	809.107868	628.8062481
LDAH	258.830451	305.735926	275.8069409	271.539058	547.130227	244.6364366
WDR35	1812.78988	1526.64816	257.754123	252.430754	292.2058303	443.9698293
CCND2	193.390299	145.249958	1.002934331	0	7.053244179	3.624243505
FGF23	0	0	0	0	0	0
OTUD4P1	0	0	1.012963674	3.06738566	4.060653434	0.924182094
ELL2	2803.18262	2936.4666	853.4971154	959.427949	423.1946507	1123.488305
GLRXP3	0	0	0	0	0	0
DNAH7	4.88359342	10.1573397	14.04108063	17.0969037	30.22818934	15.4030349
CYP20A1	1815.72003	1922.78441	1203.521197	1232.98846	561.2367154	761.091136
NDUFB3	830.210881	835.949059	974.8521694	921.221397	832.2828131	842.6366148
GTF3C3	881.976972	1020.81264	1351.955478	1014.75152	1446.922663	1165.194287
SATB2	1265.82741	1330.6115	251.736517	367.080579	281.1221608	316.2152458
UBE2B	1183.78304	1293.02935	936.7406649	1175.66355	916.9217433	936.866946
TRPM6	1.95343737	2.03146794	1.002934331	1.00570022	14.10648836	5.436365257
GDA	0	0	0	0	0	0
KLF9	203.157486	122.903811	1139.3334	1057.99663	656.959315	521.8910647
TJP2	4614.99578	3901.43419	1841.387431	2879.31972	3186.051156	2068.53698
C2orf40	0	0	0	0	1.007606311	0
ITGB1BP1	2469.14483	2513.94158	796.3298586	989.609012	1708.900304	1282.982201
CPSF3	1762.00051	1748.07817	1253.667913	1227.95996	2074.661395	1617.318664

PIGZ	38.0920287	29.4562852	543.5904072	282.601761	114.8671195	330.7122198
SENP5	1736.60582	1745.03096	2870.398054	2382.50381	1498.310585	2777.982646
CCDC92	929.836187	744.533002	870.5469991	738.183958	744.621064	715.7880922
C1orf198	1172.06242	1011.67104	2108.167963	3294.67391	1182.929809	2710.028081
TRIM67	0	0	0	1.00570022	2.015212623	5.436365257
HEATR1	2314.82328	2089.36478	1883.510673	1821.29292	2966.39298	3509.110349
PTBP3	3211.45103	2880.62155	1613.721338	2427.76032	7616.496107	1926.285423
RAD23B	7002.09624	6489.52435	5482.039052	5195.44731	13900.93667	5323.107648
FKBP15	1013.83399	1034.01718	927.7142559	824.674177	1808.653329	765.6214404
CTNNAL1	9795.51168	7368.13423	359.0504904	788.468969	1361.276127	484.7425688
FAM206A	284.225137	365.66423	275.8069409	219.242647	1044.887745	237.3879496
WDR34	3146.9876	2971.02187	1635.785893	3820.65512	6613.927827	3401.352529
SET	14712.0596	13144.4407	7771.637835	8596.60476	21907.95076	11174.15885
PPP2R4	3349.16837	3446.38537	4562.34827	4445.19495	6471.855337	6678.574718
GLE1	1816.69675	1856.7617	1579.621571	1806.23759	2827.343309	2164.579433
RAB14	2891.0873	2952.73866	4648.600623	4746.90502	4372.003785	4436.980111
CNTRL	1006.02024	974.088879	275.8069409	461.616399	380.8751857	459.3728642
TRIM32	659.285112	689.683367	1297.797024	1310.42738	680.1342601	1194.188235
FBXW2	2517.00405	2505.81571	4416.922792	3774.39291	3212.24892	3779.179915
PHF19	4194.03003	3342.7805	467.3673981	854.845183	1921.505236	874.3487455
NEK6	2883.27355	2476.35942	1245.644439	1151.52675	1410.648836	1873.733892
BSPRY	0.97671868	0	794.3239899	980.55771	1057.986627	530.9516734
PPP6C	2075.5272	2086.31758	1755.135079	1853.5055	2978.484256	1953.467249
NDUFA8	1046.06571	1119.33884	1749.117473	1153.53815	1229.2797	819.0790321
HDHD3	265.667482	314.877531	561.6432252	511.90141	897.7772234	360.6122287
LCN1P1	0	0	0	0	0	0
RBM18	788.211978	862.358142	1305.820499	958.432305	768.8036155	775.58811
SLC46A2	0	0	0	0	3.022818934	0
HSDL2	1971.99502	1871.99771	749.191945	659.739341	1421.732505	548.1668301
MAPKAP1	2530.67811	2766.85934	1459.269451	1312.43878	3522.591664	1517.651968
NR4A3	7.81374947	18.2832115	9.026408976	17.0969037	149.1257341	42.58486118
INVS	492.266217	522.087262	256.7511887	327.85827	625.7235193	261.8515932
GALNT12	30.2782792	45.7080288	14.04108063	7.03990151	197.490837	22.6515219

DENND1A	1188.66664	1085.81962	1098.213092	1302.38178	1153.709226	1168.81853
ALG2	1192.57351	1397.64995	1273.7266	467.6506	1526.523562	594.3759348
CSF3R	0	0	123.3609227	10.0570022	0	25.36970453
KDSR	2285.52172	2408.30525	720.1068494	1205.83456	568.2899596	670.4850484
VPS4B	1564.70333	1489.066	1652.835777	1735.83857	1045.895351	1579.264107
ONECUT2	352.595445	309.798862	751.1978137	537.043915	729.5069694	1285.700383
C19orf25	565.520118	598.26731	916.6819783	759.303663	458.4608716	891.5639022
ZBTB45	446.360439	443.875746	530.5522609	670.802044	500.7803367	513.7365168
YLPM1	4188.16972	4205.13865	3461.126375	3897.08834	3846.03329	4767.69233
DCAF4	859.512442	836.964793	1065.116259	1672.47946	684.1646854	1273.015531
PROX2	0	0	2.005868661	0	0	0
VSX2	0	0	0	0	0	1.812121752
FCF1	1629.16676	1757.21977	1383.046442	1134.42984	1541.637656	1354.56101
PGF	530.358245	673.431624	0	3.01710065	21.15973254	5.436365257
IFI27L2	497.14981	536.307537	546.5992102	575.260523	86.65414277	290.8455413
BBOF1	86.9279629	73.132846	154.4518869	105.598523	83.63132384	160.3727751
NEK9	1704.3741	1445.38944	3407.970856	2322.1618	86.65414277	2752.612942
ACYP1	283.248418	295.578586	103.3022361	193.094441	98.74541851	148.5939837
IFT43	138.694053	211.272666	635.8603657	369.091979	292.2058303	446.688012
NPC2	4299.51565	4792.23288	2603.617523	5752.60523	632.7767635	1256.706435
DPPA5P4	0	0	0	0	0	0
DNAL1	441.476845	446.922948	461.3497921	453.570797	488.689061	569.0062302
IRF2BPL	1906.55487	2562.69681	5582.332485	5070.74049	3456.089648	4590.104399
ACOT2	1078.04348	796.57921	1061.555842	1137.06478	435.2556983	1033.951369
LTBP2	1726.83863	1577.43486	61.17899417	22.1254047	301.2742871	202.0515754
AREL1	2861.78574	2748.57613	1764.161488	1805.23189	2160.307931	2305.92493
MLH3	663.191986	598.26731	997.9196591	817.634275	735.5526072	1041.970008
TTLL5	1772.74441	1834.41555	1526.466051	1844.4542	1492.264947	1455.133767
FLVCR2	55.672965	53.8339005	52.1525852	48.2736103	640.837614	101.4788181
ABCD4	1821.96127	1759.71848	1128.271034	1338.79818	430.3889598	1279.747563
DLST	3521.06109	3061.42219	3448.088229	3236.34329	2909.967027	4118.046682
PPP4R4	179.716238	165.564637	0	0	18.1369136	6.342426133
TGFB3	32.2317166	52.8181666	5312.54315	4302.38552	123.9355763	3686.761705

ZC2HC1C	62.5099958	63.9912403	124.363857	76.4332164	21.15973254	100.5727573
SLIRP	1113.4593	1220.91223	1171.427298	823.054999	1654.489563	1329.191305
RBM25	5009.59013	4677.45494	5795.957497	4121.35948	4512.061062	6250.007924
ALDH6A1	614.356052	504.819784	3382.897497	2691.25378	978.3857283	3636.928357
GPR68	881.000253	1269.66747	2.005868661	1.00570022	224.6962074	33.52425242
ESRRB	0	0	3.008802992	1.00570022	2.015212623	9.060608762
EIF2B2	1548.09911	1692.2128	976.8580381	1039.89402	1232.302519	1252.176131
NRDE2	1035.3218	990.340623	732.1420614	777.406267	1138.595132	729.3790053
COQ6	444.407001	439.81281	1215.556409	906.135894	561.2367154	1073.682138
ZNF410	1035.73203	997.156198	2128.106298	1987.17311	1413.560818	2216.487661
RHOQ	1175.80325	1272.61309	934.815031	647.489913	1113.203453	664.1426222
GPR75	85.9512442	103.330617	35.10270157	15.0855032	90.68456802	35.33637417
SUPT7L	1558.84302	1614.00128	2081.088736	1503.52182	1167.815715	1660.809586
KLHL29	1624.28317	1393.58701	15.04401496	3.01710065	272.053704	92.41820937
DNMT3A	574.310586	495.678178	1796.255386	1100.23604	1762.303438	1820.2763
TMEM214	2242.5461	2639.89259	2148.285336	1706.67327	2151.239475	1936.252092
ATAD2B	497.14981	361.601294	1152.371546	1073.08213	1630.307012	1108.112452
FKBP1B	29.3015605	19.2989455	0	0	8.06085049	0
ATL2	1788.37191	2003.02739	953.7905485	1196.78326	2071.638576	1381.742836
YPEL5	1338.1046	1160.98393	1580.624505	1460.27671	966.2944525	1196.000357
FAM98A	1878.23003	2154.37176	1245.644439	1213.88016	2042.417993	1774.067196
YIPF4	1591.07474	1709.48028	1743.099867	1092.19043	1315.933843	1043.782129
AFTPH	1484.6124	1561.18312	2433.118686	2307.07629	1469.090002	2106.591537
LGALSL	302.782792	261.043631	515.508246	1201.81176	273.0613104	531.8577343
CNRIP1	0	0	0	0	0	0
BCL11A	0	0	5.014671654	2.01140043	0	0
CRIPT	353.572164	372.774368	226.6631587	305.732866	238.8026958	266.3818976
EPCAM	226.598735	154.391564	7808.846699	7612.14493	6544.402992	6830.792946
SLC17A5	1220.89835	1975.60258	475.3908728	783.440468	273.0613104	506.4880298
OGFRL1	10168.6182	9238.10048	2662.790648	1190.74906	1153.709226	1929.909666
SLF2	2497.46967	2777.01668	1224.582818	1761.98678	1908.406354	1820.2763
IDE	2145.85095	2076.16024	1797.258321	2102.91915	2729.605497	2685.564437
TECTB	0	0	0	0	0	0

ELOVL3	31.2549979	29.4562852	0	0	17.12930729	12.68485227
IFIT3	1808.883	2824.75618	9.026408976	59.3363127	40.30425245	413.1637595
NKX2-3	0	0	0	0	5.038031556	0
IFIT2	832.164319	1139.65352	48.14084787	146.832231	37.28143352	1275.733714
GPAM	566.496837	657.17988	470.3762011	465.6392	703.3092053	632.4304916
CUTC	357.479038	372.774368	270.7922693	253.436454	349.63939	260.0394715
PPP1R3C	189.483425	170.643307	0	0	344.6013585	68.86062659
PYROXD2	220.738423	204.162528	100.2934331	97.5529209	227.7190264	117.7879139
CNNM1	0	1.01573397	0	0	341.5785395	48.92728731
MXI1	689.563391	611.471851	339.9947381	550.118018	338.5557206	328.9000981
SMNDC1	1518.79755	1235.13251	774.2653033	808.582973	860.4957898	933.2427025
C10orf88	405.83638	392.550708	256.2497215	282.350336	213.4311689	266.2278672
HELLS	2412.1826	2006.63325	840.6796147	966.477907	1962.464432	1576.545925
PRLHR	0	0	0	0	0	0
TCTN3	1023.60118	1254.43146	752.200748	649.682339	1063.024658	1101.770025
FAM45A	1711.44555	1628.04888	1461.656435	1962.88545	928.1162494	912.2130295
AVPI1	437.56997	367.695698	113.3315794	190.077341	311.3503502	198.4273319
WDR11	1704.3741	1473.82999	2015.898005	1887.6993	940.0966884	2213.506721
C10orf76	994.29962	955.805668	779.279975	754.275162	846.3893015	848.979041
KCNIP2	38.0920287	37.582157	20.05868661	37.210908	17.12930729	16.30909577
CFAP58	15.6274989	15.2360096	4.011737323	3.01710065	0	4.530304381
GOT1	1649.67786	1746.0467	1907.581097	2412.67482	1099.298486	1310.164027
CPN1	0	0	0	0	0	0
C10orf95	4.88359342	2.03146794	16.04694929	19.1083041	45.34228401	18.12121752
SFRP5	0	0	0	0	0	0.906060876
GNA13	3323.77368	2910.07783	8252.143673	9600.41426	5026.947887	9447.496756
HOXB8	8.79046815	5.07866986	0	0	1.007606311	0.906060876
KANSL1	1376.28453	1330.22552	808.5556281	832.719778	1071.085509	1009.533028
HOXB5	3.90687474	2.03146794	0	0	0	0
CRHR1	0	0	5.014671654	1.00570022	0	6.342426133
HOXB3	113.299367	126.966747	94.27582709	116.661225	54.41074081	139.5333749
HOXB1	0	0	0	0	0	0
DUSP1	1024.5779	635.849467	384.1238487	1955.08122	176.3311045	207.4879406

PANK3	5565.4798	6851.73508	5836.07487	15228.3026	5074.305384	6226.44128
MSX2	77.160776	116.809407	724.1185868	710.024352	597.5105426	915.1214849
TEK	0	1.01573397	0	0	0	0.906060876
RCL1	233.435765	200.099593	291.8538902	349.983675	961.256421	320.7455502
CAAP1	698.353859	749.611672	1128.301122	1468.32231	612.6246373	955.8942244
EQTN	0	0	0	0	0	0
MOB3B	251.99342	261.043631	15.04401496	8.04560172	19.14451991	16.30909577
INSL6	0	0	0	0	0	0
INSL4	1.95343737	2.03146794	0	0	0	0
MLANA	0	0	0	0	0	0
CD274	891.744158	675.463092	2.005868661	16.0912034	30.22818934	26.27576541
IFNA6	0	0	0	0	0	0
IFNA8	0	0	0	0	0	0
GRIA2	0	0	0	1.00570022	1.007606311	0.906060876
NUP43	2034.50502	1976.61831	1429.181421	1051.96243	1250.439432	1673.494438
MTHFD1L	1931.94956	1582.51353	426.2470906	130.741028	2100.859159	435.8152814
LRP11	1223.82851	1242.24265	1521.45138	1037.88262	2011.182197	1248.551887
CCDC170	0	0	2.005868661	1.00570022	1250.439432	103.2909399
PCMT1	1947.57706	2013.18473	2317.781238	2649.01437	2217.741491	2900.300865
PLEKHG1	0.97671868	5.07866986	0	0	53.4031345	16.30909577
MYCT1	7.81374947	19.2989455	0	0	0	0.906060876
CXorf21	0	0	0	0	0	0
MAGEB4	0	0	0	0	0	0
CYSTM1	331.107634	395.120515	957.8022858	782.434768	285.1525861	414.9758813
WDR55	1954.41409	1972.55537	1925.633915	1710.69607	2006.144166	2234.346121
ARAP3	1643.81754	1533.7583	2.005868661	5.02850108	117.8899384	50.73940907
PCDHB8	26.8011607	31.2541343	1.15337448	0	236.1627672	11.39824582
PCDHB10	48.0740936	72.2085281	25.57482543	11.3442984	74.39157396	17.02488386
PCDHB14	194.025167	189.586746	59.17312551	31.4583027	138.7876933	51.02028794
PCDHB12	26.5374466	24.7839089	4.132089443	2.01140043	26.19776409	4.530304381
SLC25A2	1.95343737	0	1.002934331	0	4.030425245	0
TNN	1.95343737	0	0	0	0	0.906060876
MRPS14	371.1531	397.151983	923.7025186	822.662776	751.6743082	958.612407

CENPL	506.916997	514.977124	231.6778304	467.6506	748.6514893	550.8850127
TNFSF18	33.2084353	10.1573397	0	0	1.007606311	0
SEC16B	21.8784985	11.7114127	14.32190224	5.02850108	0	8.136426668
GORAB	235.389203	221.430006	237.6954364	203.151444	218.6505695	235.5758278
GPR31	0	0	0	0	0	0
ACAT2	2285.52172	3526.62835	397.161995	6054.3153	1466.067183	559.9456215
TCP1	6832.14719	6093.57093	4232.382876	4589.01008	6260.258012	7467.753741
TTLL2	0	0	0	0	0	0
SNX19	3908.82817	3981.67717	2728.984314	2150.18706	1569.850633	2312.267356
KCNJ5	0	0	0	0	0	0
MSANTD2	713.981358	612.487585	384.1238487	622.528433	187.4147739	312.5910023
TP53AIP1	1.95343737	2.03146794	6.017605984	16.0912034	2.015212623	19.0272784
TEX11	0	0	0	0	0	0
ARR3	0	0	0	0	0	0
PDZD11	917.138844	959.868604	1325.879185	1316.46158	774.8492534	785.5547796
SLC10A7	115.252805	176.737711	375.0974397	287.630262	157.1865846	304.4364544
NUDCD1	4085.61425	3325.51303	585.7136491	666.779243	2353.768343	922.3699719
ENY2	2660.58169	2510.89438	881.5792767	989.609012	1830.820668	870.724502
MASTL	1926.08924	1651.58344	306.8979052	833.725479	1433.823781	890.6578413
KIAA1217	1546.14568	1265.60453	3027.858744	3862.89453	880.6479161	5184.480333
SEPT7P9	0.97671868	0	3.028861679	2.01140043	4.080805561	0.95136392
LYZL1	0	1.01573397	0	0	0	0
PLXDC2	0	1.01573397	13.0381463	32.1824069	1066.047477	77.01517448
EPC1	592.868241	684.604697	658.9278553	986.591911	881.6555224	696.7608138
IQSEC3	2.93015605	0	1.002934331	0	1.007606311	4.530304381
CCDC77	292.038886	318.940467	146.4284123	208.179945	88.66935539	247.3546192
TAF12	52.7428089	43.6765608	494.446625	381.160382	491.7118799	382.3576897
ENOX1	207.064361	181.816381	28.08216126	58.3306125	12.09127574	30.80606979
TNFSF11	0	0	1.002934331	0	0	0
MTRF1	141.624209	175.721977	302.8861679	314.784167	110.8366942	269.1000802
SPG20-AS1	3.90687474	3.04720192	0	0	1.007606311	0
SOHLH2	1.95343737	0	1.002934331	0	0	0
DNAJC15	944.486967	933.45952	596.7459268	526.986913	1190.99066	551.7910736

PROSER1	1173.03914	1204.66049	2197.429119	2442.84582	2773.940175	2604.925019
UFM1	1280.47819	1323.50137	2884.439135	1409.9917	1294.77411	1477.785289
WBP4	577.240742	543.417675	690.0188195	537.043915	498.7651241	514.6425777
ELF1	882.95369	960.884338	1445.228371	2192.42647	2709.453371	1929.909666
SMAD9	30.2782792	35.550689	2.005868661	3.01710065	26.19776409	7.248487009
HSPH1	5245.95605	5775.46337	5494.074264	5516.26568	7691.058974	7638.093186
KBTBD7	204.690935	233.242992	835.3740921	682.89056	649.3619634	591.2137823
ALG5	530.358245	550.527813	945.7670739	654.71084	679.1266538	847.1669192
EXOSC8	1169.13226	1027.92278	1684.929676	1162.58945	961.256421	1112.642756
ETF1	6905.40109	6795.26027	3901.414546	3230.30909	6320.714391	5694.592607
TGFBI	2477.9353	2415.41539	4.011737323	1.00570022	1657.512382	863.476015
FAM53C	1891.90409	2007.09033	1529.474854	1377.8093	1646.428713	2288.709773
SIL1	944.486967	929.396585	1103.227764	961.449406	828.2523879	959.5184679
PAIP2	1969.06487	1980.68125	1850.41384	2083.81085	1553.728932	2380.221922
MYOT	0	0	0	0	0	0
KDM3B	3685.15959	3419.97628	2769.101687	3243.3832	4017.326363	3489.240434
EGR1	1277.54804	2143.19868	921.6966499	4174.66159	256.9396094	1035.627581
SERP1	2162.45517	2251.88222	7579.174737	4244.05491	4127.155451	4918.098436
PLS1	962.067904	845.090665	1013.966608	1096.21323	925.9902001	913.3093632
ZFP30	421.942471	458.096021	721.1097838	677.841945	349.63939	760.1850751
NR2C1	977.695403	713.045249	790.3122526	639.625337	449.3924148	641.4911003
UTP20	3197.77697	3192.45187	547.6021446	646.665239	2831.373735	1455.133767
TMPO	11878.8526	10567.6962	4264.476774	5846.13535	10978.87837	7382.584019
ARL1	2055.99283	2274.22836	2767.095818	1820.31739	1783.463171	1739.636882
GLT8D2	8.79046815	3.04720192	0	0	0	1.812121752
MTERF2	220.738423	232.60308	799.3386616	570.232022	279.1069482	620.6517002
SOCS2	496.173091	318.940467	909.661438	1396.9176	732.5297883	717.6002139
NFYB	971.83509	829.854655	999.9255277	957.426605	909.8684991	980.357868
CCDC53	654.401518	630.770797	465.3615294	568.220622	253.9167904	282.6909934
APAF1	1288.29194	11371.2409	1574.681371	17784.1448	521.9400692	671.6305629
DUSP4	7071.44327	7471.7391	4663.644638	5669.13212	811.1230806	3322.525233
CLU	273.481231	303.704458	228.6690274	590.346027	6174.480487	5233.407621
TNFRSF10B	3365.77258	3504.2822	1586.642111	631.579735	1822.759817	1378.118593

SORBS3	1169.13226	832.901857	3.008802992	3.01710065	742.6058514	82.45153973
PTK2B	98.6485871	157.438766	113.3315794	187.06024	249.8863652	314.403124
CHRNA2	0	0	2.005868661	0	0	0
ADRA1A	0	0	0	0	0	0
PPP3CC	179.716238	216.351336	0	0	219.6581759	19.93333928
PDLIM2	488.486315	362.413881	33.08680357	20.1140043	774.3252981	100.2556359
EPHX2	1.95343737	2.03146794	239.701305	150.855032	123.9355763	235.5758278
RNF170	428.779502	439.81281	462.3527265	408.314288	211.5973254	340.6788894
NPPB	4.88359342	0	0	0	1.007606311	0.906060876
UBIAD1	964.021341	985.261953	428.2529592	424.405491	583.4040542	761.9971969
TARDBP	5107.262	5056.54718	4633.556608	4454.24625	5723.949477	5279.698271
TNFRSF8	1.95343737	2.03146794	0	1.00570022	0	0
PRAMEF2	0	0	0	0	0	0
ZNF706	1701.44395	1918.72147	1804.278861	1611.10157	2377.930743	1399.864054
LYPLA1	1197.45711	1198.55593	956.7993515	1081.12773	1926.513039	1690.709595
CRISPLD1	24.4179671	33.5192211	0	0	2.015212623	0
COPS5	1348.8485	1274.74614	2297.722552	2330.2074	1551.713719	2313.06469
RDH10	127.950148	238.697483	801.3445302	628.562635	451.4076275	1508.591359
EPX	0	0	0	0	0	0
AKAP1	1742.46613	1724.71628	820.4002825	695.944549	4194.665074	1649.936856
COIL	661.238549	787.193828	314.9213798	260.476356	1541.637656	396.8546638
TRIM25	5155.26772	5443.31836	1709.0001	1378.815	6361.018643	2593.146228
SCPEP1	786.258541	1042.14306	798.3357272	1014.75152	778.8796786	965.860894
SPOP	1521.72771	1647.5205	2255.59931	2660.07707	1759.28062	2220.755208
TBX2	0.97671868	2.03146794	0	2.01140043	11923.00548	868.0063194
SLC35B1	1006.02024	1020.81264	2824.263075	2061.68544	2247.96968	2153.706703
TBX4	0	2.03146794	0	0	35.2662209	5.436365257
NACA3P	2.93015605	2.03146794	0	5.02850108	3.022818934	1.812121752
TEX14	11.7206242	39.6136249	0	3.01710065	227.7190264	4.530304381
FAM117A	83.9978068	104.620599	657.9249209	1404.9632	245.85594	910.5911806
NCAPH	2669.37216	2556.60241	857.5088528	1167.61795	1543.652869	1849.270248
LRAT	15.6274989	13.2045416	4.011737323	0	2.015212623	4.530304381
KIAA0922	515.707465	474.347765	463.3556608	793.49747	993.4998229	799.1456928

MND1	397.524504	404.262121	164.4812302	268.521958	189.4299865	218.3606712
TRIM6	363.33935	375.82157	0	0	62.4715913	14.49697402
ABCC11	0.97671868	1.01573397	25040.09094	5756.54758	15.11409467	15072.26831
PAPD5	523.521215	576.936896	631.8486283	862.890785	1105.344123	981.2639289
ADCY7	1075.36727	1206.69196	42.12324189	19.1083041	365.761091	158.5606533
CEP89	904.441501	1023.85984	563.6490939	507.878609	843.3664825	609.7789697
TSHZ3	0	0	57.16725685	40.2280086	9.068456802	25.36970453
ECHDC2	844.861662	601.314512	1207.532934	578.277624	1008.613918	853.5093454
TAS2R8	0	0	3.008802992	0	0	0
PLBD1	256.877014	279.326842	5.014671654	21.1197045	1236.332944	105.1030616
TAS2R10	2.93015605	12.1888077	6.017605984	3.01710065	0	8.154547886
PRB2	0	0	0	0	0	0
PYROXD1	177.7628	180.800647	874.548707	475.63586	353.6698153	395.0244208
IAPP	0	0	0	0	0	0.906060876
KCNJ8	0.97671868	1.01573397	0	0	266.0080662	3.624243505
TAS2R7	0	0	0	0	0	0
BCL2L14	0	0	0	4.02280086	0	5.436365257
TAS2R9	0	0	0	0	0	1.812121752
RP11-408E5.4	0	0	0	1.00570022	0	0
PSPC1	1900.69456	1713.54321	2715.434671	3218.87428	1376.390221	2811.343808
ZNF549	277.388106	287.452714	284.8333499	241.368052	201.5212623	274.5364455
A1BG	45.9057781	63.9912403	213.0533399	283.758316	42.97440918	179.3728717
ZSCAN18	950.347279	1029.95425	0	0	303.2894997	22.6515219
ZNF211	416.082159	443.875746	329.9653948	353.000776	216.6353569	279.9728107
PDZRN3	0	0	210.6162094	466.6449	504.810762	485.6486296
RGSL1	0	0	0	0	1.007606311	0.906060876
LHX4	94.7417123	117.825141	81.23768079	47.2679101	101.7682374	109.633366
RNF2	545.009026	551.543547	124.363857	193.094441	403.0425245	250.9788627
TRMT1L	545.985744	664.290018	642.880906	985.586211	767.7960092	888.8457195
SEC22A	309.619823	315.893265	442.2940398	427.422592	536.0465576	456.6546816
CSTA	0	0	1.002934331	0	227.7190264	90.60608762
DPPA4	0	0	0	0	0	0
POPDC2	2.93015605	1.01573397	5.014671654	6.03420129	5.038031556	14.49697402

B4GALT4	305.712948	360.58556	498.4583624	521.958412	733.5373946	578.066839
NAA50	4965.63779	4153.33621	4895.322468	4312.44252	9411.042947	6849.820224
CD80	0	0	0	0	0	0
KIF18A	1396.70772	1450.46811	429.2558935	786.457569	590.4572984	999.3851464
GJA8	0	0	0	0	0	0
DESI2	2401.75124	2445.88741	2269.64039	2483.07383	1341.124	2098.436989
MAPK8IP1	642.680894	789.225296	214.6279468	297.647036	629.7237164	457.5607425
CRY2	252.970139	236.666016	749.191945	578.277624	769.8112218	983.0760507
PEX16	372.129819	374.805836	516.5111803	632.585436	688.1951106	650.5517091
DEPDC7	26.3714045	27.4248173	0	0	9.068456802	1.812121752
CAT	1233.5957	1462.65692	1986.812909	2505.19924	875.6098845	1726.045969
PILRB	505.823072	492.844281	1097.912212	1537.17255	670.8844342	1020.260789
ZMYM2	1318.57022	1061.442	3302.662751	3101.57946	2898.883358	3966.734516
GJB6	0	0	0	0	0	0
GJA3	375.059975	416.450929	0	0	15.11409467	6.342426133
TBC1D15	832.164319	826.807453	1059.098653	1107.27594	609.6018183	893.3760239
ADGRB2	224.645297	185.879317	1643.809368	1138.45264	90.68456802	4747.758991
HCRTR1	0	0	0	0	0	0
ZCCHC17	556.72965	601.314512	700.0481628	576.266224	491.7118799	647.8335265
FABP3	3.90687474	3.04720192	1.002934331	0	0	0.906060876
KHDRBS1	5914.03163	5221.88835	5207.235045	4551.79918	5217.38548	5555.965293
TMEM39B	517.660902	532.244601	100.2934331	170.969037	232.7570579	239.2000713
CCRL2	761.840573	970.025943	0	0	13.09888205	10.87273051
CCR2	0	0	17.04988362	1.00570022	0	6.342426133
POLR3GL	354.548882	340.270881	150.4401496	175.997538	201.5212623	174.8697491
GHSR	0	0	0	0	0	0.906060876
TNFSF10	422.91919	224.477208	2032.947888	763.326464	121.9203637	1186.033687
ZNF639	1527.58802	1366.16219	1224.582818	994.637513	1199.05151	1050.124555
SLITRK3	0	0	0	0	0	0
PIK3CA	1309.77976	1188.40875	1233.609227	1576.93794	949.1651452	1386.273141
PDS5A	5610.27212	4744.49338	4242.412219	4269.19742	5241.568031	5931.980556
TMEM156	184.599831	217.36707	2.005868661	0	13.09888205	3.624243505
LIAS	64.4634331	55.8653685	110.3227764	147.837932	156.1789782	196.6152101

CPXM2	0	0	3.008802992	3.01710065	2.015212623	9.060608762
TMEM54	750.119949	702.887909	994.9108561	1036.87692	924.9825938	1047.406373
ZSCAN20	111.34593	80.2429838	258.7570573	308.749966	99.75302482	218.3606712
CSMD2	0	0	0	0	0	2.718182629
HPCA	0	2.03146794	11.03227764	7.03990151	4.030425245	9.060608762
LRIF1	570.403711	659.211348	1551.53941	1605.09754	672.0734096	1494.094385
TMIGD3	0.97671868	1.01573397	0	0	0	0
CLCC1	1380.1035	1234.11678	1020.957061	1049.95103	1394.527135	1630.909577
GPSM2	2243.52282	2090.38051	798.3357272	1763.99818	1885.231408	1456.945889
GTDC1	397.524504	391.057579	94.27582709	146.832231	295.2286492	145.8758011
CXCR4	234.412484	184.863583	346.0123441	140.79803	54.41074081	231.0455234
ZRANB3	149.437959	153.37583	125.3667913	153.872133	362.7382721	228.3273408
ACVR2A	364.316069	385.978909	215.6308811	198.122942	352.662209	271.8182629
POLK	1085.13446	957.837136	827.4208228	832.719778	412.1109813	722.1305183
SV2C	0.97671868	0	0	2.01140043	0	6.342426133
FLT3	0	0	60.17605984	45.2565097	17.12930729	14.49697402
RPL21	6650.77053	6932.69924	11898.99343	10544.0225	11569.08376	9197.333348
MTIF3	382.873724	430.671204	1003.937265	842.776781	541.0845892	637.8668568
GTF3A	2369.51953	2445.88741	3475.167456	2872.27982	3849.056109	4226.773987
RASL11A	87.9046815	74.14858	169.4959019	168.957636	33.25100827	173.0576274
UBL3	741.329481	727.265524	4701.756142	3733.1592	2228.825161	5004.174219
LINC00544	0	0	0	0	0	0
FYTTD1	1757.11691	1775.50298	2891.459675	2913.51352	2676.202363	3541.791965
MTERF4	425.849346	377.853038	1028.007689	1232.98846	850.4197267	1100.863965
XPNPEP2	0	0	0	0	0	0
SASH3	0	0	0	0	0	0
OCRL	1743.44285	1662.75651	1969.763026	1289.30768	2361.829194	1641.782308
PAEP	2.93015605	0	0	0	24.18255147	9.966669638
OBP2A	0	0	0	0	0	0
MRPS2	1823.53378	1696.27573	829.4266915	667.784943	2964.377768	1370.870106
TBX22	0	0	0	0	0	0
FMOD	9.76718684	13.2045416	5144.050182	3996.65266	7.053244179	1514.027724
MYOG	0	0	0	0	2.015212623	0

LAX1	0	0	0	1.00570022	0	0
PLG	1.95343737	3.04720192	0	1.00570022	2.015212623	0
KIAA1191	4106.12535	3850.64749	2083.094605	2075.76524	3282.781362	2170.015798
COPA	4902.15107	5017.72582	10450.57573	8548.45183	4773.031097	8482.541923
CD244	0	0	0	0	0	0
LY9	0	0	0	0	0	0
HS3ST2	1.95343737	3.04720192	0	0	0	0
RBBP6	2042.31877	2128.97841	3535.343516	2907.47932	3176.982699	3407.694955
ZC3H7A	1442.6135	1258.49439	1574.606899	1435.13421	1735.098068	1938.064214
PRM2	0	0	0	0	0	0
SERAC1	299.852636	288.468448	339.9947381	386.188883	184.391955	348.8334373
ANXA11	4723.41156	3945.11075	813.3797422	761.315063	2939.18761	873.4426846
LDB3	1.95343737	3.04720192	2.005868661	0	18.1369136	7.248487009
OPN4	2.93015605	0	0	0	0	0
FAM35A	1502.90634	1461.98654	1511.432066	2007.42792	1422.175852	1773.84068
FAM213A	1574.47052	2208.20566	2834.292419	7460.2842	2354.775949	1725.139908
ZNF205	317.433572	280.342576	665.9483956	707.007252	472.56736	666.8608049
NAA60	754.026824	728.281258	2030.701315	2129.06736	1947.703	2403.779505
RPL5	18778.2762	17722.5162	17275.51376	12573.254	11734.5831	10727.75171
ODF2L	903.464783	931.428053	6721.665884	3560.17876	194.4680181	4273.889153
PTGFR	0	4.06293589	0	0	0	0
SPATA1	1.95343737	1.01573397	10.02934331	11.0627024	5.038031556	3.624243505
TRMT13	327.200759	363.632762	338.9918038	260.476356	252.9091841	391.4182985
LRRC39	3.90687474	9.14160575	5.014671654	14.079803	2.015212623	9.060608762
RWDD3	238.631909	215.76221	223.1428592	205.223186	111.2095086	202.6767574
ZNF644	1989.57596	1980.68125	1557.557016	1554.81253	859.4881835	1972.494527
CCDC18	398.501223	394.104781	498.4583624	401.274386	178.3463171	520.0789429
RPAP2	1020.67102	1033.00145	1163.403824	1123.36714	592.472511	1402.582236
PQLC1	915.185407	836.964793	795.3269243	736.172558	402.0349182	770.1517448
BBS9	135.763897	224.477208	99.29049874	171.974737	199.5060496	134.0970097
PMS2	390.091675	420.097414	847.8305365	715.575817	838.016093	881.4341416
ZMIZ2	2562.90983	2342.28254	1659.856317	1525.64723	2897.875751	1971.588467
OCM	0	0	2.005868661	0	0	0.906060876

SEPT7	5061.35622	4598.18706	2780.97643	2659.07137	4313.562619	2998.989015
EEPD1	143.577647	139.155554	105.3081047	129.735328	125.9507889	198.4273319
KIAA0087	0	0	0	0	0	0
KLHL7	648.541206	717.108184	539.5786699	608.44863	392.9664614	549.072891
HERPUD2	676.866048	641.94387	394.153192	417.365589	498.7651241	373.297081
CBX3	4707.77429	4587.04446	3392.916811	4275.2115	9180.170113	5323.089526
HNRNPA2B1	24967.8597	23437.0457	25055.30545	22652.3917	30757.18265	27167.32931
WIPF3	20.5110924	28.4405512	0	0	14.10648836	5.436365257
NXPH1	0	0	38.11150457	136.775229	4.030425245	5.436365257
NPY	0	0	0	0	0	0
FAM126A	1657.49161	2023.34207	24.07042394	62.3534134	359.7154531	77.92123535
HOXA7	0.97671868	0	0	0	45.34228401	0.906060876
INHBA	363.33935	372.774368	2.005868661	0	45.34228401	74.29699185
FKBP9	2936.33868	2913.20629	625.5100834	785.592666	3345.797061	817.0403951
NT5C3A	612.334244	566.586567	445.3028428	394.204313	1126.483704	539.9760398
ARL4A	652.223436	693.472055	238.0564927	369.836197	453.1507864	244.5186487
CCZ1	1559.86857	1535.86087	1592.248514	1596.93126	2235.485438	1514.10927
POLM	436.593252	421.529598	630.845694	840.76538	443.346777	876.1608673
RAMP3	0	0	0	0	115.8747258	3.624243505
FTSJ2	947.417123	819.697316	665.9483956	632.585436	1793.539234	1118.079121
TWIST1	0	0	0	0	2.015212623	0
SMU1	2229.82922	2128.91746	2778.087979	2680.1609	2013.167182	3020.734476
GLIPR2	241.249515	297.610054	0	0	112.8519069	9.060608762
SLC25A51	451.244032	448.954416	657.9249209	630.372895	493.7270925	642.37904
CLTA	4216.49456	5189.38486	5904.274405	7325.52037	2819.282459	3466.588912
RECK	374.083256	309.798862	0	0	71.5400481	11.77879139
SPINK4	5.8603121	12.1888077	0	0	2.015212623	0
OR2S2	0	1.01573397	0	0	0	0
TAF1L	0	1.01573397	0	0	1.007606311	0
ACO1	6694.42986	6407.2499	1077.151471	1950.05272	902.8152549	951.36392
PHF24	0	0	0	0	2.015212623	4.530304381
DNAI1	0	0	7.020540315	6.03420129	0	12.68485227
DCAF10	1616.46942	1698.3072	2622.673275	2785.7896	1551.713719	2761.673551

CNTFR	0	1.01573397	1.002934331	1.00570022	4.030425245	4.530304381
KIAA1549	1086.11118	1150.82659	961.8140231	1245.05687	1148.671195	781.9305361
TRIM24	1325.40725	1426.0905	1084.172011	1449.21401	2344.699886	1113.548817
C7orf49	1050.9493	1093.94549	602.7635328	677.841945	1353.215276	858.0396497
CALD1	5358.2787	4650.03012	1.002934331	0	107.8138753	46.20910469
AKR1D1	0	0	0	0	1.007606311	0
NUDT10	0	0	0	0	3.022818934	0
SFTPA1	0	0	0	0	1.007606311	0
NEUROG3	0	0	0	0	0	0
PLAU	9560.12248	10555.5074	0	1.00570022	608.594212	290.8455413
SRGN	3768.18068	3296.05674	0	0	62.4715913	30.80606979
CHST3	1656.51489	1546.96284	0	0	161.2170098	41.6788003
BICC1	342.828258	295.578586	0	0	38.28903983	19.0272784
ARL4AP1	0	0	0	0	0	0
CISD1	733.515732	776.020755	450.3175145	480.724703	363.7458784	586.2213869
EGR2	2.93015605	14.2202756	6.017605984	7.03990151	1.007606311	8.154547886
ECD	1179.87617	1178.25141	842.4648378	603.420129	1141.617951	695.8547529
P4HA1	2774.85778	2686.61636	2489.283009	1991.28643	1077.131147	1818.464178
SLC25A16	473.708562	513.96139	664.9454613	606.43723	488.689061	634.2426133
ZWINT	3439.02649	2897.88902	1570.595162	2725.44758	2809.206396	3695.822314
VPS26A	3283.72822	3332.62316	1917.61044	1920.88741	1603.101641	1642.688369
RBM19	1165.22539	1371.24086	1094.201355	1234.99986	1275.62959	1244.021583
CIT	4336.63096	3336.6861	714.0892435	1541.73843	2388.026958	1970.682406
IFT81	541.102151	602.330246	204.5986035	184.043139	254.9243968	247.3546192
ACADS	500.079966	441.844278	27.07922693	10.0570022	256.9396094	50.73940907
HVCN1	117.206242	142.202756	0	0	15.11409467	2.718182629
NME2P1	0	2.03146794	0	0	3.022818934	2.718182629
DDX54	4652.11109	4469.22948	2043.980166	2788.8067	5380.617702	3404.976773
MED13L	4735.13218	4058.87295	1313.843973	1698.62766	7477.446436	2072.161224
CDKN2C	1497.30974	1822.22675	554.6226849	1278.24497	585.4192669	894.2820848
RNF11	2471.09827	2537.30346	2725.975511	2511.23344	2986.545107	3410.413138
RASSF8	610.449177	651.085476	70.20540315	187.06024	751.6743082	147.6879228
BHLHE41	168.972332	246.823355	70.20540315	168.957636	52.39552819	73.39093097

SSPN	48.8359342	44.6922948	59.17312551	98.5586211	60.45637868	44.39698293
ITPR2	1066.5768	1149.81086	1788.231912	2381.49811	1103.328911	2813.319021
CCDC91	445.38372	461.143223	657.9249209	521.958412	309.3351376	444.8758902
NECAB1	0.97671868	1.01573397	0	0	6671.361387	144.0636793
WWP1	1481.68224	1658.69358	5132.01497	5660.08081	5499.515247	4019.286047
ACOT9	2780.71809	2098.50639	514.5053117	827.691277	673.0810159	473.8698382
PRDX4	2082.36423	2244.77208	2741.019526	2136.10726	2037.379961	1446.979219
DDX39A	3831.6674	4390.00223	3468.146916	4289.31142	3422.838639	3034.397874
PKN1	12126.9392	11648.4372	4937.44571	4828.36673	3946.793921	4193.249735
C19orf43	3568.93007	3960.34676	4987.592427	5598.7331	4244.037783	4376.274032
ADGRE5	5355.30947	5738.89694	1.002934331	14.079803	632.7767635	153.9669247
WDR83	275.434669	335.192211	553.6197506	353.000776	244.8483336	442.1577076
GIPC1	4167.65862	4110.67539	3506.25842	6474.69799	5004.780548	4247.613388
ACTRT1	0	0	0	0	0	0
CCDC70	0	0	0	0	0	0
SPRYD7	437.56997	492.630977	471.3791354	552.129418	294.2210429	376.9213245
EBPL	394.594348	409.340791	1083.169077	902.113093	939.0890821	1471.442863
ATP7B	271.527794	278.311108	449.3145802	487.764605	385.9132172	621.5577611
ZC3H13	2813.92653	2649.0342	4229.374073	3456.59164	2522.038597	3615.182896
GUCY1B2	1.95343737	0	132.3873317	58.3306125	173.3082855	60.7060787
NLN	1709.2577	1536.8055	793.3210556	525.981213	1694.793816	1282.07614
CENPK	1142.76086	997.450761	204.5986035	268.521958	418.1566192	335.2425242
OPTN	2986.80574	3340.74903	152.4460183	303.721465	760.742765	430.3789162
ITIH5	0	0	0	0	0	0
ATF1	660.26183	629.755063	590.7283208	675.830545	852.4349394	598.9062392
TSFM	496.173091	460.899447	825.0739565	698.730339	1476.314539	1050.351071
NEUROD4	0	0	0	0	0	0
ARHGAP9	9.76718684	20.3146794	1.002934331	1.00570022	5.038031556	3.624243505
NCKAP1L	0.97671868	2.03146794	0	0	0	0.906060876
MMP19	148.46124	140.171288	0	1.00570022	90.68456802	12.68485227
PFDN5	1728.79207	1838.47849	2735.00192	2444.85722	3123.579565	1964.33998
SPATS2	828.257444	847.122133	1370.008296	1526.65293	1838.881518	1818.464178
ORMDL2	322.317166	354.491156	545.5962759	713.041453	571.3127785	526.4213691

NR4A1	47.8592155	43.6765608	449.3145802	1405.9689	158.1941909	559.9456215
PDE1B	0	0	0	0	0	0.906060876
HOXC13	0	0	533.5610639	807.577273	685.1722917	823.6093364
CDK2	2448.63374	2029.43648	780.2829093	1178.68065	3065.138399	1781.315683
LRP1	3401.91118	3360.04798	1058.095719	1152.53245	763.765584	2372.067374
HOXC11	0	0	1233.609227	1224.94286	116.8823321	1414.361028
ATG101	714.958077	856.263739	1015.972477	930.272699	1578.91909	935.9608851
NFE2	5.8603121	3.04720192	0	0	7.053244179	2.718182629
HOXC12	0	0	0	0	8.06085049	0
IKZF4	198.273893	202.13106	211.6191438	235.33385	208.5745064	300.8122109
SMUG1	249.063264	273.232439	684.0012135	682.870446	770.8188281	665.0486831
TUBA1B	45301.8241	40758.4688	11434.2136	22546.3707	56488.4351	31534.00816
METTL21B	145.531084	109.699269	160.4694929	143.815131	203.5364749	241.9182539
KBTBD4	492.266217	564.748089	516.5111803	837.74828	796.0089859	652.3638308
TYRL	0	0	0	0	0	0
SARDH	400.45466	388.010377	1.002934331	1.00570022	28.21297672	17.21515665
DBH	1.95343737	3.04720192	0	0	0	0
ATPAF1	690.540109	687.651899	1473.310532	1273.21647	1654.489563	1811.215691
STIL	1637.95723	1645.48904	510.4935743	1044.92252	1483.19649	1366.339801
HJURP	2715.27794	2708.9625	390.1414546	923.232798	1992.037677	1151.603374
IL13RA2	0	0	1.002934331	0	0	0
COL10A1	0	0	0	0	0	0.906060876
AMD1	2979.95894	2863.34391	1431.18729	1629.22429	3714.026787	2986.376648
NDUFAF4	356.50232	394.104781	286.8392186	256.453555	342.5861458	334.3364633
USP45	432.686377	378.868772	296.8685619	230.305349	349.63939	296.2819065
PLP1	1.95343737	1.01573397	0	0	0	0
SERPINA7	0	0	0	0	0	0
MORF4L2	5463.76432	5438.23969	8327.363748	6963.46829	7124.784227	5044.946959
H2BFWT	0	0	0	0	6.045637868	0.906060876
RAB9B	120.136398	81.2587178	136.399069	178.008938	116.8823321	102.384879
NRK	0	0	1374.020033	3343.95322	118.8975447	1849.270248
FAM199X	1788.37191	1478.90866	2865.383383	2838.08601	3386.564812	2850.467516
ESX1	0	0	0	0	0	0

MAGEA9	0	0	3.008802992	0	0	0
ATXN3L	0	0	0	0	0	0
RAB9A	684.679797	803.445572	1377.028836	1308.41598	571.3127785	784.6487188
METTL8	918.115563	938.53819	457.3380548	535.032515	578.3458706	895.1881457
TTC21B	746.213074	702.887909	289.8480216	326.85257	449.3924148	443.9698293
NMI	462.964656	455.04882	32.09389858	137.78093	194.4680181	125.9424618
TNFAIP6	0	4.06293589	0	0	0	0
ACVR1C	11.7206242	9.14160575	14.04108063	23.131105	5.038031556	16.30909577
BAZ2B	589.703673	543.011382	1089.477534	1097.87264	967.8864705	1381.344169
SLC36A1	655.378237	1025.89131	219.6426184	549.112318	614.6398499	361.5182896
LPGAT1	2740.67263	2663.25448	2214.479002	2640.96877	1692.637538	2279.649164
BATF3	125.99671	115.793673	0	0	21.15973254	5.436365257
G0S2	2740.67263	3340.74903	0	0	167.2626477	97.85457463
KCNJ2	0	0	0	0	0	0
RAP2C	816.53682	889.78296	1999.851055	2151.19276	2046.448418	2025.952119
EXOSC9	1534.42505	1181.29861	712.0833748	585.317525	691.2179295	790.9911449
PLA2G12A	356.50232	450.985884	1995.839318	1468.32231	1511.369163	1569.297438
B9D2	92.788275	115.793673	24.07042394	66.3762142	76.57807966	23.55758278
ADCK4	342.828258	428.639736	1048.066376	1146.49825	461.4836906	919.6517893
PFKFB2	509.847153	613.503319	1087.180814	2282.93949	656.959315	1266.673105
C4BPA	0	0	0	0	0	1.812121752
C4BPB	365.292788	199.083859	0	0	1.007606311	5.436365257
ZNF137P	25.3946858	39.6136249	38.11150457	25.2531324	81.61611121	69.06902059
RAB38	113.299367	130.013948	0	0	7.053244179	5.436365257
GPR83	20.5110924	34.5349551	4.011737323	4.02280086	2.015212623	15.4030349
AGO2	5728.45508	5486.99492	4850.190423	5544.42529	6724.764522	6890.592963
MXD4	920.069	978.151815	2962.668013	4026.82366	1090.230029	1535.773185
PMS2P5	3.99477942	2.66122301	44.60048969	41.877357	3.798675794	49.07225705
CKS2	1351.77866	1781.59739	780.2829093	1499.49902	1305.857779	466.6213512
DAW1	58.603121	71.1013781	0	0	1.007606311	4.530304381
ACSL3	2689.88326	2809.52017	2738.010723	4299.36842	4793.183223	2640.261393
CHPF	1828.41738	2299.62171	3371.86522	2469.99973	3713.029257	3070.640309
DNPEP	1170.10898	1112.2287	1613.721338	2109.95905	2268.121807	1958.903614

INHA	3.90687474	2.03146794	33.09683291	59.3363127	44.3346777	35.33637417
MOGAT1	0	0	0	1.00570022	0	0
OBSL1	1534.42505	1319.43843	2184.390972	1787.12928	3421.831033	1813.933874
FAM124B	1.95343737	0	0	0	0	0
SLC12A4	1854.66181	1617.48525	74.21714047	201.04953	185.3995613	324.3697937
ENKD1	214.87811	180.800647	695.0334912	391.217384	220.6657822	590.7516913
MC3R	0	1.01573397	0	0	0	0
GCNT7	0	0	0	0	0	0
CTCFL	0	0	0	0	0	0
HMGB1P1	10.8025086	11.2340177	7.080716375	19.1988171	50.76320596	30.12652413
FAM210B	1278.52476	1509.38068	5124.99443	5401.61586	1055.971414	4425.201319
PI3	5.8603121	5.07866986	0	1.00570022	2.015212623	2.718182629
FAM209A	2.93015605	1.20872343	2.005868661	4.91787405	1.168823321	3.624243505
SNX21	578.217461	608.424649	350.0240814	481.730403	308.3275313	440.3455858
SLPI	0	7.11013781	926.7113216	195.105842	9.068456802	705.8214225
WFDC3	17.5809363	27.4248173	1.002934331	9.05130194	1.007606311	2.718182629
TTPAL	1660.42176	1697.29147	508.4877057	554.140819	1592.017972	876.1608673
PREX1	5.8603121	13.2045416	507.4847713	582.300425	25629.47413	1657.185343
KCNS1	23.4412484	29.4562852	10.02934331	1.00570022	0	73.39093097
SLC12A5	19.5343737	20.3146794	0	0	175.3234982	19.0272784
ARHGAP40	261.760607	217.36707	16.04694929	12.0684026	2.015212623	2.718182629
SDC4	8767.02691	7466.66043	2728.984314	3643.65188	4347.821233	2270.588556
NCOA3	1997.38971	2060.92423	2760.145484	3506.87665	42334.57917	6999.320268
PIGT	3691.01991	3915.65446	1631.774156	2156.22126	2704.415339	2018.703632
SEMG2	0	0	0	0	0	2.718182629
MATN4	3.90687474	3.04720192	1.002934331	4.02280086	0	4.530304381
NCOA5	1823.53378	2092.41198	1061.104522	1046.93392	2047.456025	1019.318486
VAPB	2207.38423	2011.15327	2157.311745	1770.03238	6519.212834	2301.394625
PARD6B	1783.48832	1478.4008	229.6719617	255.447855	5147.860644	853.5093454
ATP5E	3612.88241	4050.74708	3811.150457	4102.25118	7101.609282	3626.961687
CHD6	2463.28452	2264.07102	3226.439742	3616.49798	4345.806021	3601.591983
PLCG1	4272.16752	4029.41667	3393.929775	3563.19586	4807.289711	3642.364722
TOX2	922.022438	908.066171	0	0	108.8214816	12.68485227

SRSF6	6497.74802	5910.84039	6354.973034	4296.85417	4737.301377	5676.054601
GDAP1L1	0.97671868	1.01573397	1.002934331	0	0	0
GTSF1L	0	0	0	0	0	0
ARFGEF2	4987.1256	5100.00027	3504.252551	3908.15104	4471.756809	3717.567775
ZNFX1	4064.12644	3905.49712	1537.498329	1447.20261	3185.04355	3840.792054
ZNF831	0	0	0	0	0	0
EDN3	0	0	0	0	0	0
CSE1L	14696.686	14080.1043	5450.948087	5048.61508	10828.74503	7532.990125
TMEM189-UBE2V1	57.1087414	51.3758243	0	59.6681938	87.47030388	14.27951941
RAB22A	2678.16263	2653.09714	2263.622784	2592.69516	3998.181843	2174.546103
PTGIS	0	0	2.005868661	2.01140043	0	3.624243505
STAU1	6624.10611	6198.0087	4069.907514	4181.7015	7511.705051	5284.14703
CDH26	0	3.04720192	5.014671654	16.0912034	401.0273119	41.6788003
SNAI1	30.2782792	28.4405512	11.03227764	6.03420129	40.30425245	27.18182629
MOCS3	417.058878	453.017352	353.0328844	348.977975	913.8989243	440.3455858
STX16	2633.45822	2445.88741	4434.97561	2494.13653	8285.637382	3847.505965
PPP4R1L	537.195276	411.372259	79.23181213	50.2850108	487.6814547	94.23033112
PMEPA1	1024.5779	1347.87898	1951.710208	2797.858	2073.653789	1832.055092
RNF114	2427.14593	2389.0063	3086.028936	2405.63492	5134.761762	3609.746531
ANKRD60	0	0	0	0	0	0
DDX27	3156.75479	2963.91173	1853.422643	1640.29705	3943.771102	2437.303757
RBPJL	0	0	0	0	0	0.906060876
SEMG1	0	3.04720192	0	0	0	0.906060876
C20orf85	0	0	0	0	0	0
BCAS4	887.837284	866.421078	22.06455528	13.0741028	1180.914597	115.9757922
KCNK15	0	1.01573397	0	0	584.4116605	131.378827
TP53TG5	0	4.06293589	0	0	0	0
PCK1	0	0	0	0	0	0
ZBP1	0	0	0	0	0	0.906060876
NEURL2	28.3248418	24.3776153	43.12617622	67.3819144	20.15212623	48.02122644
MAGEA10	0	0	0	0	0	0
MTRR	1678.0027	1457.57825	655.9190523	588.334626	631.7691572	810.9244842
FASTKD3	512.777309	481.457903	271.7952036	285.618861	296.2362555	321.651611

PEPD	1690.70004	2013.18473	1480.331072	1805.23189	647.8908582	1248.551887
CHST8	0	0	23.06748961	15.0855032	72.54765441	28.08788716
IQSEC2	661.238549	778.052223	746.183142	662.756442	496.7499115	539.1062213
VAMP7	741.81784	713.553115	1300.30436	1266.67942	1153.205423	1164.741256
IL9R	0	3.5550689	14.50243042	16.7951936	11.65800502	13.3372161
XG	0	0	0	0	8.06085049	0
STAMBP	1555.91286	1669.86665	1159.392086	1282.26777	1470.097608	1597.385325
NAGK	981.602277	1173.17274	1959.733682	1761.98678	1216.180818	1289.324627
MCEE	76.1840573	78.2115159	237.6954364	240.362352	94.71499326	174.8697491
PAIP2B	133.81046	134.076884	136.399069	150.855032	368.7839099	158.5606533
SNRNP27	753.958454	706.950845	626.8339567	648.676639	741.3463435	559.7462881
MPHOSPH10	961.091185	1002.52943	1094.201355	836.742579	1405.610804	1394.427688
IL17C	0	1.01573397	0	0	76.57807966	18.12121752
RP11-663P9.2	0.18557655	1.68611839	5.044759683	5.08884309	1.027758438	0.28993948
ATP8A1	20.5110924	29.4562852	269.789335	336.909572	88.66935539	462.0910469
USP22	10429.4021	10667.2382	6514.058478	7825.35338	7902.656299	9799.048376
POF1B	0	0	49.1437822	47.2679101	750.6667019	96.94851375
HIF3A	0	0	0	0	0	0
ZNF576	270.355732	298.625788	373.091571	381.160382	253.4029112	302.3978174
IRGC	0	0	0	0	0	0
ZNF45	587.007929	601.314512	452.3233831	584.311825	343.5937521	716.6941531
LYPD3	16.6042176	20.3146794	564.6520282	834.731179	1464.05197	503.7698472
PSG8	0.97671868	0	0	0	7.506667019	4.530304381
CEACAM8	0	0	0	0	0	0
NDP	0.97671868	10.1573397	0	2.01140043	0	0
USP9X	11545.7916	10374.7068	6428.80906	7404.97069	6483.946613	7137.041522
CRISP2	0	0	1.002934331	0	0	1.812121752
F13A1	0.97671868	6.09440383	0	0	0	0
GRM4	0	0	306.8979052	333.892472	129.9812142	476.5880209
TRERF1	3835.57427	4008.08625	21.06162094	24.1368052	1049.925776	66.14244396
PACSIN1	9.76718684	19.2989455	8.023474646	18.1026039	648.8984645	48.92728731
BTN2A2	801.925108	756.9859	362.5206432	270.925581	115.4011508	286.5961157
SIRT5	263.714045	330.113541	363.0622277	256.453555	413.1185876	252.7909845

MRS2	1257.03695	1321.4699	1166.412627	884.010489	1220.211243	1424.327697
WRNIP1	3196.80025	3176.20013	1401.09926	1526.65293	2220.76431	1822.088422
RRP36	3010.24698	3266.60045	969.8374978	982.569111	1721.999186	1443.354976
BTN2A3P	167.995614	153.37583	26.0762926	26.1482056	28.21297672	47.11516556
BTN1A1	0	0	0	0	2.015212623	0
SNRPC	3291.54196	3379.34693	1516.436708	1080.12203	2081.714639	1423.421636
SLC17A3	0	0	0	0	0	0
SLC17A1	0	0	0	0	0	0
SERPINB6	1955.39081	1850.6673	1748.114538	2002.34913	1157.739652	1623.66109
XPO5	5955.05382	5871.95809	1416.143275	1171.64075	3782.554093	2752.612942
ABCC10	984.532433	960.884338	354.0358187	452.565097	791.9785607	636.0547351
HIST1H1D	0	0	80.23474646	55.3135119	13.09888205	73.39093097
PEX6	1584.23771	1912.62707	629.8427597	701.97875	260.9700346	655.9880744
NQO2	756.95698	801.414104	1299.802893	762.320763	574.3355974	648.7395873
PRICKLE4	338.706505	410.823762	210.7766789	182.172537	129.6083998	372.717202
OARD1	1190.13172	1134.40217	594.4792952	430.439692	594.4877237	478.4545063
UNC5CL	30.7666385	30.6446939	28.34292419	20.1140043	7.053244179	38.00019315
AARS2	641.704175	621.629191	632.8515627	558.16362	667.0353781	769.2456839
HIST1H1A	0	0	0	4.02280086	0	0
ZNF391	174.832644	191.973721	216.6338154	166.946236	117.8899384	203.8636971
RPS10	17704.0517	18846.1719	9948.366389	9378.62719	13169.68654	10083.83237
MOCS1	361.385913	380.90024	42.12324189	46.2622099	307.3199249	86.98184411
HIST1H2BJ	21.487811	33.5598504	44.12911055	124.706827	50.41054375	192.0849057
MED20	1714.14129	1358.03632	398.1649293	336.909572	533.0237387	542.7304648
OR2B6	3.90687474	7.11013781	17.04988362	20.1140043	2.015212623	27.18182629
TBCC	1355.68553	1323.50137	440.2881712	392.223084	691.2179295	439.4395249
SPDEF	79.1142134	171.659041	12677.08994	15453.5895	2566.373275	8575.866193
TCP11	0.97671868	8.12587178	1.002934331	1.00570022	0	0
MAD2L1BP	887.837284	910.097639	532.5581296	510.895709	506.8259746	585.315326
APOBEC2	0	0	0	0	0	0
KLHDC3	5968.72788	5803.90392	2000.85399	1883.6765	1890.26944	2540.594697
GNMT	11.7206242	11.1730737	94.27582709	137.78093	12.09127574	154.9364098
DNAH8	0	0	3.008802992	0	0	1.812121752

TREM1	0	0	0	0	0	0
MEA1	4207.70409	4555.56687	994.9108561	1206.84026	1516.447498	1275.733714
KLHL31	6.83703079	1.01573397	15.04401496	24.1368052	29.22058303	38.96061768
COL21A1	1.95343737	0	3.008802992	0	31.23579565	18.12121752
CDKN1A	1814.74331	3109.16169	616.8046134	1213.88016	5080.351022	2402.873444
SOX4	144.554365	92.4317915	3916.458561	3190.08108	2935.157185	4512.183163
GLO1	9214.36406	9348.81548	3028.861679	3632.58918	5203.278991	3202.925197
CPNE5	53.7195276	95.4789934	0	0	3.022818934	3.624243505
KCNK17	0	0	0	0	0	0
RREB1	1706.32754	1834.41555	2540.43266	3056.32296	2361.829194	3400.446468
SSR1	6731.54517	7692.15337	5550.238586	3412.34083	4431.452557	5161.828812
RIOK1	916.162125	958.85287	346.0123441	408.314288	544.1074081	557.2274388
NRN1	0.97671868	2.03146794	0	0	0	0
SLC35B3	430.73294	454.033086	527.543458	584.311825	342.5861458	488.3668123
RPP40	232.459047	257.996429	174.5105735	152.866433	440.323958	347.9273765
ATXN1	980.625559	1122.38604	1794.961601	1669.46236	2180.460058	1900.009657
NUP153	7977.83821	6750.56798	1602.68906	2006.37193	3696.907556	3673.170792
DEK	18077.1094	14883.5499	2911.518362	3372.11282	6477.900975	4372.649788
EEF1E1	1233.58593	1178.25141	825.1140739	621.522733	708.3472368	723.0365792
CRISP1	0	0	0	0	0	0
RUNX2	1818.65019	1721.66908	7.020540315	12.0684026	178.3463171	97.85457463
OPN5	0	0	0	0	0	0
GCM2	2.93015605	2.03146794	0	0	0	0
LRRFIP1	3854.8254	3694.46823	4223.577112	7024.81601	3764.820221	4357.781329
AC105760.3	0	0	1.002934331	0	1.007606311	0
RAB17	1.95343737	7.11013781	1522.454314	3383.17553	785.9329228	983.0760507
CXCL6	0	0	0	0	0	0
EREG	87.9046815	125.951013	0	0	14.10648836	7.248487009
TRIM51	0	0	0	0	0	0
DKFZP434K028	0	0	0	0	0	0
MYRF	1094.90164	775.005021	1.002934331	5.02850108	73.55526072	37.14849592
SCGB1D2	0	0	0	0	1.007606311	0
SCGB2A1	0	0	1.002934331	2.01140043	0	1.812121752

AHNAK	63589.2699	55879.5887	29051.99876	55849.5501	26502.0612	33420.05542
EMC3	930.812906	1025.89131	1781.211371	1755.95258	2091.790702	1770.442952
SSUH2	0.97671868	1.01573397	0	4.02280086	2.015212623	5.436365257
WNT1	0	0	0	0	0	0
SH3TC1	67.3935892	88.3688556	14.04108063	8.04560172	157.1865846	40.77273943
CNOT1	7612.54542	7333.59928	10533.81928	12268.5369	11135.05735	14437.174
LRRC29	15.6274989	11.1730737	89.26115543	59.3363127	14.10648836	48.02122644
BBS2	573.333867	514.977124	1284.758878	871.942087	640.837614	1011.163938
MT1G	0	0	0	0	0	0
MT2A	11830.0167	14362.3768	2367.927955	2219.58038	2000.098528	1813.933874
C16orf70	470.778406	474.347765	908.6585036	839.75968	525.9704945	1393.521628
GOT2	3457.58414	3401.69307	4660.635835	3863.90023	4987.651241	6453.871621
DOK4	117.206242	130.013948	102.2993017	194.100142	613.6322436	270.0061411
PIWIL1	0	0	0	0	0	0
GPR18	0	0	0	0	0	0
CLYBL	19.5343737	11.1730737	10.02934331	8.04560172	18.1369136	20.83940015
TMTC4	489.336061	416.450929	319.9360515	363.057778	391.9588551	362.4243505
RAP2A	1613.53927	1679.00826	422.2353532	1280.25637	1071.085509	615.2153349
SLC10A2	0	0	0	0	0	0
ABCC4	1404.52147	1244.27412	38.11150457	143.815131	764.7731903	208.3940015
EFNB2	1723.90848	1858.79317	1392.072851	1166.61225	1346.162032	1485.033776
SOX21	0	3.04720192	0	0	0	0
TM9SF2	5287.95495	5970.48429	5137.029642	4324.51093	4661.186796	4684.33473
C17orf53	366.269506	362.617028	92.26995843	97.5529209	345.6089648	215.6424885
KIF25	8.79046815	5.07866986	0	1.00570022	0	2.718182629
IRF1	1493.40287	1246.30558	91.26702409	142.809431	364.7534847	352.4576808
UPF3B	539.148713	496.693912	614.7987447	564.197821	929.013019	620.6517002
RNF113A	388.734036	362.617028	472.3820698	533.021114	556.1986838	351.55162
SEPT6	727.655419	603.34598	1096.207223	968.489308	45.34228401	790.9911449
TMEM255A	0	0	0	0	0	0
NDUFA1	1016.76415	1096.99269	2087.106342	1947.03562	1473.120427	993.9487812
AMELX	0	0	2.005868661	2.01140043	0	3.624243505
ATP5S	652.448081	558.653685	679.9894762	586.323226	672.0734096	637.8668568

BMP4	2678.16263	1864.88757	2116.191438	1567.88664	170.2854666	1255.800374
PTGER2	47.8592155	28.4405512	0	0	46.34989032	4.530304381
RP11-297B17.2	0	0	0	0	0	0
FAM193A	937.649937	935.490988	871.5499334	831.714078	926.9978064	839.0123713
GRK4	130.880304	115.793673	41.12030756	63.3591136	69.52483548	67.04850484
SOX9	2687.92982	2292.51158	248.727714	368.086279	640.837614	389.6061768
TEKT3	1.95343737	0	2.005868661	0	2.015212623	7.248487009
MYH2	0	0	0	0	0	0
HS3ST3B1	83.9978068	80.2429838	30.08802992	39.9665266	85.92866623	69.12338424
SLC25A35	153.344833	154.391564	233.6836991	551.123718	71.5400481	172.1515665
MRPS7	1702.42067	1764.32991	4587.421629	4695.61431	2528.084235	5742.613833
GGA3	1095.87836	1151.84232	4326.658703	4721.76251	1358.253308	5449.050109
ARMC7	353.572164	366.679964	1671.891529	4063.02887	437.3011391	3811.798106
NUP85	2310.91641	2087.33331	3851.26783	3756.29031	2040.40278	4406.174041
SLC25A19	367.246225	374.805836	1135.321662	1130.40704	514.8868251	1561.14289
MIF4GD	393.61763	452.001618	2220.496608	2294.00219	399.0120993	1710.642934
NT5C	919.092282	844.074931	1861.446118	1297.35328	1021.7128	1511.309541
MSTO1	998.724156	995.073943	1399.785416	876.417453	839.2957531	1395.850204
C1orf61	0	0	2.005868661	5.02850108	8.06085049	2.718182629
TTF1	537.195276	549.512079	497.455428	367.080579	878.6327034	490.178934
GTF3C4	2634.21029	2432.68286	1493.369218	1838.41999	3993.143812	2831.440238
DDX31	678.819485	779.067957	480.4055444	445.525195	950.1727515	663.2365614
BARHL1	0.97671868	0	0	0	0	0
KIR2DL1	0	0	0	0	0	0
PPP1R12C	2573.65373	2755.68627	1404.108063	1557.82963	1636.35265	1521.276211
MBOAT7	7866.49228	8542.32271	4550.313058	6615.49602	5214.362661	8326.699452
SRMS	0	0	462.3527265	312.772767	1.007606311	245.5424974
OPRL1	45.9057781	44.6922948	13.0381463	11.0627024	52.39552819	25.36970453
LINC00029	0	0	0	0	0	0
SLC2A4RG	3176.28916	3089.86274	5037.739143	5142.1452	4347.821233	4668.931695
NPBWR2	0	0	0	0	0	0
C20orf195	0	1.01573397	11.03227764	16.0912034	5.038031556	0.906060876
BHLHE23	0	0	0	0	0	0

PPDPF	2654.72138	2527.14612	7863.005153	5459.94647	11379.90568	6783.67778
IL1B	1650.65458	1886.21799	0	0	105.7986627	39.86667855
PLGLB2	28.803434	31.1119316	8.595147214	38.2367222	33.25100827	33.46082816
IL37	4.88359342	0	0	1.00570022	1.007606311	0.906060876
CHCHD5	172.879207	158.4545	293.8597589	403.285786	402.0349182	265.3036852
PAX8	338.921383	371.758634	0	0	54.41074081	24.46364366
INSIG2	1059.73977	957.837136	5160.097131	5475.03197	335.5329017	3474.74346
POLR1B	1997.38971	1971.53964	719.1039151	786.457569	2241.924043	1547.551977
HTR5BP	0	0	0	0	0	0
CCDC93	2202.50063	2198.04832	2269.64039	2158.23266	1265.553527	2060.382432
PSD4	243.202952	215.335602	3297.648079	3559.17306	2098.843946	2740.83415
SLC25A23	2473.05171	2899.92049	1779.205503	3632.58918	1210.13518	1907.258144
PSPN	96.6951497	175.721977	148.4342809	128.729628	40.30425245	114.1636704
GTF2F1	3006.34011	3054.31205	3319.712635	3008.04934	2124.034104	3230.107024
ALKBH7	578.217461	609.440383	836.4472318	1057.99663	268.0232788	504.675908
CLPP	1833.30097	1745.03096	1304.817564	1322.49578	942.1119011	1233.148852
TNFSF9	156.274989	231.587346	0	0	27.2053704	3.624243505
GRIA3	0	0	0	0	0	0.906060876
THOC2	3797.48224	3265.58472	4987.592427	5496.15168	4393.163517	4390.771006
MED1	3830.69068	3596.714	1644.812302	1797.18629	1612.170098	2740.83415
RPL23	11541.0935	11589.5246	13269.82413	9005.03973	6708.018105	7520.839848
RP11-51F16.8	0	0	0	0	0	0
ATG4C	315.480135	351.443954	177.5193765	131.746728	245.85594	162.1848968
CD70	0	0	0	0	0	0
C3	7967.0943	8784.06739	2.005868661	2.01140043	200.5136559	269.1000802
SH2D3A	604.588865	463.174691	715.0921778	874.959188	343.5937521	638.7729177
TRIP10	4867.96592	4303.66484	853.4971154	708.012952	917.9293496	1033.81546
GPR108	1107.59899	1246.30558	1616.730141	1564.86954	585.4192669	1236.773096
TNFSF14	1.95343737	0	0	0	0	0
FOSB	44.9290595	72.117112	27.07922693	355.012176	24.18255147	93.32427025
OPA3	913.231969	991.356357	615.8016791	813.611474	1215.173211	704.9153617
SNRPD2	3255.40337	3271.67912	2385.980773	2549.45005	3076.222068	2339.449182
RTN2	461.011219	465.206159	432.2646965	636.608236	144.0877025	519.1728821

EML2	1250.19992	1218.88077	974.8521694	789.474669	510.8563998	790.9911449
VASP	3636.32366	3359.03225	2548.456134	2272.88249	2661.088268	3184.80398
SYMPK	4388.39705	4226.46906	3012.814729	3149.85308	2368.882438	4139.792143
GPCPD1	426.826065	313.861797	922.6995843	1351.66109	214.6201443	761.091136
SDCBP2	9.76718684	11.1730737	3.008802992	7.03990151	66.50201655	33.45176755
PANK2	1270.71101	1380.38247	816.3885452	668.790643	568.2899596	949.5517982
TGM3	0	0	0	0	0	1.812121752
GNRH2	2.93015605	5.07866986	0	1.00570022	0	4.530304381
DEFB126	0	0	0	0	0	1.812121752
FOXA2	854.628848	799.382636	0	0	0	0
FAM182A	0	3.04720192	44.12911055	20.5464554	0	45.30304381
CD93	0	0	0	0	1.007606311	0
GZF1	688.586672	690.699101	899.6320946	932.2841	977.3781219	876.1608673
PAX1	0	0	0	0	0	0
NAPB	326.22404	286.43698	311.9125769	295.675863	285.1525861	282.6909934
CST8	0	0	0	0	0	0
NKX2-4	0	0	0	0	0	0
CENPB	2695.74357	2632.78246	2528.397448	2630.91176	5585.161783	3128.628205
PSMF1	2792.43872	2837.96072	3103.078819	3065.37426	2361.829194	3757.434454
NKX2-2	0	0	152.4460183	121.689726	5.038031556	223.7970364
DTD1	1539.51376	1608.92261	220.6455528	168.957636	1408.825068	233.1657059
CSTL1	0	0	0	0	5.038031556	0
RBCK1	2205.43079	2278.2913	8618.214704	2233.66018	1561.789782	2781.60689
TMX4	790.165415	804.461306	1407.116866	1937.98432	1687.740571	1429.764063
CST11	0	0	0	0	0	0
STK35	1542.2388	1594.70234	1868.466658	2098.89635	1266.561133	2217.130964
SNRPB	6992.91509	6710.83247	4634.559542	4252.72405	4190.634649	4286.900187
NRSN2	1089.04133	937.522456	1161.397955	1076.09923	654.9441023	1302.009479
AP5S1	588.961366	582.015566	255.2668459	388.200283	770.5770026	376.9213245
RRBP1	13338.0703	13570.2059	9424.573906	7542.75162	5511.606523	8458.98434
BMP2	0.97671868	0	0	0	0	0
ZNF133	377.013412	289.484182	590.7283208	461.616399	227.7190264	453.0304381
FLRT3	0	0	5.014671654	2.01140043	286.1601924	48.02122644

OVOL2	0	0	600.7576641	547.100917	90.68456802	498.3334819
PCSK2	0	0	0	0	4.030425245	0
GFRA4	0	0	0	0	0	0
MKKS	1474.84521	1524.61669	1027.004755	934.2955	1322.987087	1165.194287
BFSP1	74.23062	83.2901857	111.3257107	205.162844	88.66935539	154.030349
DSTN	6016.56756	6272.10649	9588.964872	14528.1844	8844.566679	8841.296727
LAMP5	0	0	0	5.02850108	0	6.342426133
SNRPB2	1783.48832	1951.22496	1575.609834	1590.01204	2012.189804	1629.097455
MGME1	1211.13117	1238.17971	1204.524131	1025.81422	1116.427793	1113.548817
LRRN4	209.017798	226.508676	0	0	5.038031556	0
TBC1D20	1433.82303	1629.23729	3509.267223	2171.30677	886.6935539	1666.245951
ITPA	783.328384	811.571444	1113.257107	728.126956	629.7539446	1031.097277
TCF15	0	0	0	0	0	0
OTOR	0	0	15.04401496	21.1197045	0	0.906060876
MCM8	2522.86436	2426.58846	858.5117871	1101.24174	1171.84614	2131.961242
BANF2	0	0	0	0	0	0
TMEM74B	1.95343737	2.03146794	11.03227764	9.05130194	0	46.20910469
FAM110A	325.247322	307.767394	1906.578163	1820.31739	419.1642255	2178.170346
C20orf187	0	0	0	0	0	0
SIRPD	0	0	0	0	0	0
MRPS26	1275.5946	1248.33705	2268.637456	1307.41028	1000.553067	1437.01255
DEFB129	0	0	0	0	0	0
S1PR4	0	3.04720192	0	0	1.007606311	0.906060876
NCLN	3850.22505	4571.81861	1874.484264	1623.20015	2477.703919	2430.05527
CITED1	12.6973429	9.14160575	25.07335827	19.1083041	13.09888205	16.30909577
HNRNPR	6181.65255	6078.15209	4544.295452	4359.71043	5558.964019	5582.241058
ZNF436	332.084353	348.396752	689.0158852	828.696978	386.9208235	967.6730158
MAX	1579.35411	1605.87541	1857.43438	1468.32231	1050.933383	1446.073158
CHURC1-FNTB	31.6359182	0	13.57973084	48.6457194	15.57759357	9.966669638
ARMCX5	500.265543	499.710642	567.861418	416.742055	442.9739626	516.6812146
GDF5	25.3946858	33.5192211	0	0	0	0
MMP24	141.624209	45.7080288	2.005868661	11.0627024	23.17494516	9.060608762
NECAB3	537.195276	560.685153	2047.991903	1784.11218	553.1758649	1822.088422

ID1	703.237452	456.064554	277.8128096	3758.30171	1548.6909	4694.301399
RALY	4867.96592	5289.94253	4483.116458	4368.76174	3049.016698	4035.595142
DYNLRB1	2522.86436	2455.02901	2627.687946	2530.34174	1239.355763	1549.364098
C20orf173	0	0	0	0	0	0
EIF2S2	8104.0791	6866.4226	6513.928096	3291.64675	3870.57858	4480.679427
ERGIC3	4034.82488	4314.83791	3856.282502	5136.111	3650.557666	4423.389198
ROMO1	1389.87069	1441.32651	1069.127997	785.451868	1235.325338	1083.648808
BPIFB9P	0	0	0	0	0	0
FAM83C	0	0	0	0	0	3.624243505
BPIFB1	0	0	2.005868661	3.01710065	18.1369136	157.6545925
CEP250	6069.3299	5258.45477	2529.400382	2283.94519	1561.789782	2400.155261
PLAGL2	1926.08924	1998.96446	624.828088	707.007252	1224.201364	1187.845809
MMP24-AS1	2888.74318	2617.28236	1212.878574	2269.86539	560.9646617	1440.636793
GRPR	24.4179671	20.3146794	1.002934331	4.02280086	2.015212623	0.906060876
KDM5C	4188.16972	3986.75584	7654.394812	6981.5709	3736.204202	6463.838291
AMOT	51.7660902	38.5978909	37.10857024	136.775229	401.0273119	209.3000624
TMEM115	1262.89726	1559.15165	1230.600424	1030.84272	787.9481354	983.0760507
PSMB2	3185.07963	3105.09875	2211.2997	2132.08446	4009.265513	2474.452253
AGO3	689.563391	651.085476	637.8662343	677.841945	720.4385126	976.7336245
UROD	1333.221	1383.42967	1451.245977	1439.15701	556.1986838	1275.733714
ST3GAL3	377.013412	306.75166	104.3051704	87.4959188	62.4715913	97.85457463
TMEM53	82.0443694	157.438766	79.23181213	244.385152	103.7834501	67.95456571
HECTD3	1035.3218	1043.15879	1179.450773	1616.16025	1168.823321	1656.279282
KLC1	3523.68846	3053.81434	3554.409297	3001.45195	4332.183183	3216.905717
XRCC3	1284.38507	1282.87201	724.1185868	673.819144	1517.455105	1340.064036
TUBGCP3	2238.63922	1885.20225	506.2712208	469.22955	1333.06315	545.9107385
MCF2L	2.93015605	7.11013781	405.1854696	1166.61225	1087.20721	600.7183609
F10	0	0	0	0	0	0.906060876
PCID2	1753.21004	1574.38766	775.2682376	507.878609	1188.975447	782.836597
PROZ	0	0	0	0	2.015212623	0
SLURP1	0	0	0	0	3.022818934	0
LRFN3	638.774019	717.108184	600.7576641	623.534134	461.4836906	666.8608049
IGFLR1	97.808609	105.057365	48.07064247	92.0617977	72.15468795	121.6114908

CAPNS1	9224.13125	7951.16553	6527.096624	7171.64824	6262.273225	6568.941352
PDCD2L	347.711851	359.925333	184.7605624	90.5130194	371.8067289	276.3485672
GPR42	0	0	0	0	0	0
RBM42	2014.97064	1952.24069	2870.398054	1950.05272	2343.69228	2425.524966
KIRREL2	0	0	0	0	0	0
UBA2	3297.40228	3492.0934	2853.348171	2715.39058	3925.634189	3164.87064
FFAR2	0	0	7.020540315	27.1539058	10.07606311	3.624243505
HCST	0.97671868	0	4.011737323	5.02850108	2.015212623	1.812121752
FFAR1	0	7.11013781	0	0	0	0.906060876
COX6B1	1943.67018	2150.30882	2781.136899	2911.50212	2099.851553	2407.403748
KRT36	0	0	0	0	0	0
THRA	942.53353	1133.55911	447.3087115	442.508095	341.5785395	471.1516556
CCR7	0	0	1.002934331	0	4.030425245	2.718182629
NR1D1	258.830451	264.090833	251.736517	259.470656	116.8823321	157.6545925
FRMD8	911.278532	991.356357	607.7782044	816.628575	1074.108328	1360.903436
PRDX5	2526.77124	2676.45902	4880.278453	4419.04675	2161.315538	2854.997821
BCL2L12	961.091185	972.057411	302.8861679	338.920973	741.5982451	657.8001961
IRF3	1374.24319	1633.30023	1668.882726	1354.67819	1732.075249	1710.642934
PRMT1	5151.72223	4436.61426	2459.194979	1832.39585	5686.063479	3135.006874
RRAS	952.300717	955.805668	315.9243142	375.12618	306.3123186	278.160689
PRRG2	5.8603121	2.03146794	406.1884039	526.986913	58.44116605	444.8758902
SCAF1	4100.26503	3960.34676	2704.91389	2666.11127	3255.575992	3450.279816
PRR12	1765.90738	1964.4295	2069.053524	2899.43372	1998.083315	3296.249468
TSKS	0	0	0	0	0	0
FLRT1	1.95343737	0	0	1.00570022	4.030425245	0
ASL	782.351666	633.08667	671.1235367	861.050354	1082.169178	899.4738136
SBDS	2740.48705	2483.47972	2387.204353	2090.05624	2865.078166	2080.207044
CSN1S1	0	0	0	0	0	0.906060876
STATH	0	0	0	0	0	0
HTN1	0	0	0	0	0	0
STAT5A	605.594886	489.279054	0	1.00570022	46.22897756	14.87751959
WNK4	171.902488	171.659041	30.08802992	53.3021114	20.15212623	51.64546994
BECN1	2391.98406	2263.05529	964.8228261	931.2784	1475.13564	1267.579166

PRKCG	15.6274989	8.12587178	0	1.00570022	9.068456802	0.906060876
TRAP1	2890.11059	2660.20727	2254.596375	1713.71317	7033.092053	5217.098525
GLIS2	810.676508	863.373876	30.08802992	31.1767067	1099.298486	147.6879228
NSRP1	1204.29414	1399.68141	1084.172011	1181.69775	513.8792188	1125.318548
DNAJC8	2205.43079	2071.08157	2778.128096	2120.01605	2048.463631	2046.791519
AHDC1	1035.3218	1064.4892	868.5411304	2405.63492	905.8380738	1487.751959
IFI6	145.531084	190.957987	206.6044721	248.407953	194.4680181	7465.94162
DACH2	0	0	0	0	0	0
ZNF384	1015.78743	1052.3004	909.661438	990.614712	1335.078362	1032.003338
EMG1	559.601203	517.069536	363.6339003	327.868327	1006.256119	623.0980645
SSX1	0	0	0	0	0	0
UXT	643.657613	721.17112	905.6497006	899.095993	601.5409678	495.6152993
CFP	276.411388	285.421246	31.09096425	21.1197045	6.045637868	17.21515665
ELK1	2105.80548	2400.17938	1862.449052	2224.60888	1366.314158	1660.809586
TIMM17B	718.864951	807.508508	1544.518869	1399.9347	1028.766044	711.2577878
PCNXL4	7146.65061	5406.75193	2024.924414	2305.06489	1646.428713	2613.985628
ATG14	872.209785	770.942085	757.2154197	781.429067	531.008526	908.7790588
KTN1	14128.2358	14015.0973	11064.37154	13005.7152	4492.916542	14910.13778
SIX1	861.465879	741.4858	1262.694322	1368.75799	177.3387108	1735.106578
RHOJ	127.950148	154.391564	0	0	3.022818934	0
DLGAP5	5000.79966	4676.43921	599.7547298	1506.53892	1472.112821	1335.533731
L3HYPDH	688.586672	783.384826	279.4375632	154.103444	232.7570579	246.9378312
HSPA2	106.462337	86.3373876	260.762926	217.231247	209.5821127	190.272784
ZBTB1	1080.25086	1084.80388	1698.970756	2351.3271	1231.294912	2228.003695
TRMT5	1020.48545	965.119948	772.3998455	610.460031	591.4649047	886.1365975
SGPP1	1675.07254	1774.48725	62.1819285	57.3249123	1099.298486	148.5939837
PLEKHG3	1138.85399	1079.72521	1326.88212	1893.73351	1216.180818	1720.609604
PZP	0	0	0	0	7.053244179	1.812121752
PRDM7	0	0	0	0	2.015212623	0
RHOT1	672.959173	740.470066	2080.085802	2362.38981	527.9857071	1974.306649
EVI2A	1049.97259	1229.35298	0	0	6.781190475	2.464485583
OMG	2.93015605	1.01573397	0	0	0	0
WDR60	855.605567	756.721809	878.5704737	479.719003	533.0237387	839.0123713

AIF1L	285.201856	261.043631	121.355054	397.251585	3154.815361	581.6910825
FAM78A	37.11531	87.3531216	150.4401496	138.78663	124.9431826	150.4061054
NUP214	2437.88983	2473.31222	1562.571687	1497.48762	3104.435045	2397.437078
CTAG2	0	0	0	0	0	0
AVPR2	0.97671868	0	0	0	0	0
SLC10A3	928.859468	971.041677	1301.808761	1850.4884	1932.588905	2364.818887
MAP2K2	5933.52694	6247.75935	3781.052397	3206.16223	3932.667281	3510.985895
HNRNPH2	2400.77452	2541.3664	3289.624605	4249.08341	3505.462357	2805.164473
ARMCX1	931.789624	1267.636	8.023474646	1.00570022	494.7346988	44.39698293
TMEM35	0	0	0	0	0	0
NXF5	0	0	2.005868661	4.02280086	0	0.906060876
TIMM8A	415.105441	507.866986	210.6162094	181.026039	824.2219626	359.7061678
ZC4H2	81.0676508	102.589131	64.18779717	29.1653063	9.068456802	54.36365257
CANX	33243.5971	39019.4205	21461.79174	21098.5848	24722.62845	19788.36954
CPSF3L	2206.40751	1957.31936	3020.838204	2169.29536	1322.987087	2367.537069
RGS13	0	0	0	0	0	0
IPPK	454.174188	456.064554	531.5551953	412.337088	911.8837117	489.2728731
ZNF484	195.343737	139.155554	115.337448	128.729628	273.0613104	96.94851375
OMD	0	0	0	0	0	0
FGD3	8.79046815	15.2360096	0	0	1343.139213	361.5182896
HIVEP3	1407.45162	1160.98393	752.200748	897.084592	629.7539446	865.2881368
PPCS	454.017913	623.193421	2030.560905	1694.03161	1044.887745	1656.523918
EDN2	84.9745255	24.3776153	123.3609227	193.094441	42.31946507	144.0636793
BCL11B	0	0	0	0	142.0724899	8.154547886
COX7C	3309.11313	3760.23701	5085.859932	4543.75357	3001.659201	3241.867694
TRAF2	1091.97149	13024.8756	1000.928462	12875.9649	1070.077903	1286.606444
ABHD8	798.955883	741.4858	809.3680049	517.935611	215.6277506	657.8001961
MASP1	0	0	0	0	1.007606311	1.812121752
ATP13A4	1.95343737	0	0	0	17.12930729	6.342426133
HRASLS	8.79046815	0	48.14084787	30.1710065	3.022818934	41.6788003
HELB	176.786082	186.895051	144.4225436	175.997538	357.7002405	164.9030795
RAP1B	3154.5767	3055.81534	1599.740434	1646.3212	3903.487002	1931.613061
IL22	0	0	0	0	0	0

TSPAN8	3.90687474	4.06293589	0	0	0	0
BEST3	39.0687474	34.5349551	0	3.01710065	5.038031556	2.718182629
RAB3IP	748.166512	845.090665	768.2476973	849.816682	1176.884172	830.8578235
PTPRB	307.666385	230.571612	4.011737323	1.00570022	7.053244179	10.87273051
DYRK2	579.19418	548.496345	1130.306991	1355.68389	1932.588905	1700.676265
YEATS4	837.047912	765.863415	576.6872402	650.688039	540.0769829	528.2334908
TAS2R3	5.8603121	0	10.02934331	6.03420129	1.007606311	13.59091314
TAS2R4	33.2084353	29.4562852	50.14671654	39.2223084	14.10648836	45.30304381
TAS2R5	13.6740616	11.1730737	40.11737323	23.131105	12.09127574	23.55758278
CRYGN	0	1.01573397	0	0	1.007606311	0.906060876
LRRC61	1050.9493	767.894883	331.9712635	373.11478	537.0541639	349.7394982
TRPV5	0	0	3.008802992	1.00570022	0	1.812121752
IDUA	177.7628	183.847849	822.4061512	553.135119	154.1637656	520.9850038
FGFRL1	1343.96491	1239.19545	508.4877057	380.154681	1161.770077	677.7335354
TMEM175	216.831548	194.005189	1075.145603	775.394866	363.7458784	818.1729712
AUNIP	265.442837	203.146794	222.8921257	201.230556	304.297106	361.5182896
PIN1	1588.14458	1516.49082	1212.547606	1353.67249	740.5906388	1244.021583
FBXL12	521.567777	527.165932	386.1297173	464.6335	250.8939715	451.2183163
EMC1	2640.0706	2431.66713	2143.270665	1944.01852	1939.642149	3325.243416
PLA2G5	0	0	16.04694929	4.02280086	0	5.436365257
UBR4	14372.2689	13553.2634	7591.470712	8708.58948	10126.8767	12860.67338
HP1BP3	6232.44192	5630.21341	3831.209143	5238.69242	3934.702646	5613.047128
ADGRE2	1.01578743	0	0	0	32.24340196	1.875546014
SIN3B	2581.46748	2357.51855	1508.413233	1505.53322	1846.942369	2165.485494
OR7A10	0	0	0	0	0	0
SLC35E1	3208.52088	3232.0655	2453.177373	2233.66018	3485.310231	2921.140265
EPS15L1	1380.1035	1440.31077	1179.450773	916.192896	2890.822507	1294.760992
KLF2	483.475749	465.206159	1401.09926	1034.86552	43.32707139	607.9668479
OR7C2	0	0	0	0	0	0
OR7C1	0	0	0	0	0	0
F2RL3	21.487811	49.7709646	1.002934331	0	4.030425245	0.906060876
UQCR11	1491.36153	1346.84293	2200.949418	1905.5706	882.2701622	1857.017069
GFER	475.661999	456.064554	917.6849126	894.067492	1005.591099	1006.633633

SYNGR3	33.2084353	40.6293589	8.023474646	9.05130194	295.2286492	127.7545835
PKMYT1	1504.14677	1301.15522	1659.856317	2206.50627	2659.073055	2918.422082
WFIKKN1	11.7206242	19.2989455	59.17312551	150.855032	44.3346777	62.51820046
WDR24	351.618726	398.167717	788.3063839	947.369603	787.9481354	1010.257877
FBXL16	3.90687474	2.03146794	2144.273599	1836.40859	1204.089542	2243.406729
CHTF18	1339.08132	1282.87201	1595.66852	1862.5568	1377.397828	2883.991769
GNG13	0	0	1.002934331	2.01140043	5.038031556	3.624243505
TUBBP1	101.373632	71.6295597	15.16436708	40.6302887	38.63162597	34.67494973
MACF1	17124.8087	15114.1215	829.4266915	968.489308	8169.671972	1545.739855
SMARCA4	6613.36221	6444.83205	5583.335419	5082.80889	9486.613421	7349.965828
KDM4B	2449.61046	2360.56575	3719.883433	3231.31479	1304.850173	4630.877138
TICAM1	585.054492	538.339005	437.2793682	440.496694	324.4492322	688.6062659
METTL25	81.0676508	74.14858	74.21714047	119.678326	121.9203637	90.60608762
IL17B	0	0	0	0	0	0.906060876
EMC6	1132.01695	1254.43146	461.3497921	387.194583	1210.13518	433.0970988
OR1E2	0	0	0	0	0	0
METTL16	1096.85508	1017.76544	495.4495594	413.342789	1816.714179	666.8608049
TUBA4A	874.163222	1024.87558	1107.239501	860.879385	269.0308851	2177.264285
VIL1	0.97671868	1.01573397	2.005868661	1.00570022	24.18255147	11.77879139
AAMP	2833.4609	2857.25966	3042.902759	2735.50459	4852.631995	3367.828277
PNKD	945.463686	1477.89293	1742.096932	1452.23111	2679.225182	1429.764063
TNFRSF19	30.2782792	58.9125704	23.06748961	7.03990151	9.068456802	8.154547886
RNF6	1586.19114	1751.12537	2095.129817	2341.2701	1903.368322	2341.261304
ECHS1	2323.61375	2399.16364	2823.260141	2765.67559	4623.905363	3954.955725
ZNF835	0	0	0	0	0	0
AKAP9	2598.0717	2486.51676	5286.466857	7096.22072	3691.869525	4948.904506
GNG11	556.72965	952.758466	0	0	133.0040331	73.39093097
SHFM1	1168.15555	1205.67623	433.2676309	414.348489	1593.025578	472.0577165
GNGT1	0	0	0	0	10.07606311	0
HIP1	601.658709	616.550521	119.3491854	136.775229	4068.714285	484.7425688
PTPN12	4393.28064	4398.1281	3766.018412	3612.47517	2138.140593	2788.855377
POR	1032.39165	1414.91742	1266.70606	1874.6252	3297.895457	1794.906596
FGL2	0	0	0	0	0	0

STYXL1	587.007929	572.87396	545.5962759	360.040677	678.1190475	615.2153349
STEAP4	0.97671868	0	2.005868661	0	60.45637868	53.45759169
GNAI1	567.473555	526.150198	200.5868661	182.031739	29.22058303	482.0243861
PMS2P3	144.39809	133.071308	147.5817868	118.210003	113.9098935	146.8815286
PEX1	490.312779	527.165932	1217.562277	1248.07397	704.3168116	1080.024564
MTERF1	198.273893	198.068125	276.8098753	207.174244	415.1338003	236.4818887
SGCE	671.005736	720.155386	1092.195486	920.215697	196.4832307	811.8305451
RBM48	265.667482	279.326842	297.8714962	273.550459	359.7154531	320.7455502
CASD1	366.269506	381.915974	3304.66862	1819.31169	512.8716124	2284.179469
ZNF780B	518.100426	478.329442	608.2696422	410.939165	441.432325	646.5650412
LRFN1	21.4487423	17.1252748	15.04401496	4.02280086	332.7317561	52.12568221
ZFP36	283.248418	319.956201	1281.750075	2153.20416	617.6626688	1110.830634
SRD5A3	165.065458	169.627573	1365.996558	847.805282	579.373629	990.3245377
SPINK2	0	1.01573397	0	1.00570022	4.030425245	0
RASL11B	1.95343737	3.04720192	45.13204488	31.1767067	16.12170098	59.80001783
PAICS	7738.50306	7365.05656	5501.064716	4511.56111	8641.22165	6246.365559
KDR	170.92577	214.319868	0	0	18.1369136	7.248487009
PPAT	982.578996	919.21893	447.2886528	602.414429	1468.072319	877.9639284
TUBGCP6	886.860565	801.414104	2330.819385	2306.07059	704.3168116	2749.894759
ADM2	147.484521	97.5104613	2396.010116	477.707602	135.0192457	608.8729088
DGCR6L	506.9756	551.391187	1970.053876	2259.67764	894.2606773	1990.525139
DGCR8	1236.52585	1248.33705	1830.355154	1570.5618	1387.473891	2264.24613
ASPHD2	121.113117	140.171288	2.005868661	0	16.12170098	9.060608762
VPREB3	0	0	2.005868661	0	1.007606311	3.624243505
SDF2L1	460.0345	533.260335	1324.876251	1162.58945	775.8568597	1162.476104
GAL3ST1	0	0	2.005868661	0	4.030425245	1.812121752
YWHAH	3997.70957	3377.31546	3271.571787	4454.24625	2934.149578	3082.419101
RFPL1	0	0	1.002934331	0	1.007606311	0
RFPL2	0	0	11.03227764	23.131105	0	24.46364366
C22orf24	0.97671868	0	1.002934331	2.01140043	6.045637868	0
POM121L9P	9.76718684	0	94.14544562	35.5917306	2.015212623	19.47124823
GNAZ	21.487811	28.4405512	92.26995843	118.672625	372.8143352	106.9151834
MGAT3	0	0	0	0	87.66174908	2.718182629

ADORA2A	35.6209304	28.1663031	19.6374542	30.7744266	10.9526806	7.928032667
ATF4	4833.55612	63933.6837	4682.849097	61506.8498	4347.730549	4167.848859
A4GALT	394.594348	456.064554	0	0	348.6317837	102.384879
RFPL3	0	0	1.002934331	0	0	0
CDC42EP1	2602.95529	2094.44345	1127.298188	769.360665	656.959315	755.6547707
APOL3	138.694053	103.604865	3.008802992	5.02850108	6.045637868	78.82729623
MCHR1	0	0	0	0	0	2.718182629
TPST2	867.326191	700.856441	891.60862	1065.03653	214.6201443	1033.81546
BAIAP2L2	15.6274989	10.1573397	12.03521197	9.05130194	0	6.342426133
MPST	1416.24209	1249.35279	2866.386317	2796.8523	873.5946719	1758.664161
GALR3	0	1.01573397	10.02934331	10.0570022	0	5.436365257
TST	439.523408	497.709646	658.9278553	995.643213	716.4080873	772.8699274
APOL5	0	0	0	0	0	0
IGLL1	0	0	0	0	0	0
APOL2	574.310586	524.11873	1011.96074	875.964888	358.7078468	993.0427203
RAC2	3901.01442	4727.22591	0	0	183.3843487	105.1030616
LIF	1805.95285	2536.28773	0	77.4389166	245.85594	139.5333749
C22orf23	10.7439055	11.1730737	11.03227764	14.079803	4.030425245	15.4030349
APOBEC3A	1.01578743	0	3.119125769	6.22528433	0	2.78160689
APOBEC3F	162.447851	212.064939	19.51710208	15.1458452	5.995257552	56.06704702
RIBC2	39.0687474	45.7080288	147.4313466	339.926673	77.58568597	529.1395517
KRT17	0	4.06293589	0	0	575.3432037	7.248487009
TBC1D27	0	0	0	0	0	0
EMC4	991.369464	1082.77241	2094.126883	1703.65617	1762.303438	1722.421726
RNF112	30.2782792	27.4248173	12.03521197	0	2.015212623	3.624243505
SPECC1	1739.53598	1660.72504	128.3755943	415.354189	2109.927616	448.5001337
CPA4	413.152003	250.886291	24.07042394	10.0570022	7.053244179	38.96061768
DOCK4	2108.73564	2191.95391	262.7687946	451.559397	232.7570579	499.2395428
POT1	610.449177	668.352954	489.4319534	625.545534	691.2179295	434.9092206
TAS2R16	0	0	0	0	0	0
ATP6V1F	792.118853	1064.4892	1722.038246	1546.76693	2090.783096	1170.630652
LSM8	566.496837	560.685153	968.8345635	905.130194	971.3324841	779.2123535
CDHR3	32.2317166	39.6136249	13.0381463	7.03990151	11.08366942	8.154547886

PRKRIP1	614.922549	589.846875	494.8778868	322.347033	1273.372552	609.3531211
VGF	90.8348376	118.840875	57.16725685	144.820831	1204.089542	173.9636882
PODXL	9191.89953	11244.1751	102.2993017	177.003238	3582.040437	797.333571
FOXP2	27.3481231	8.12587178	15.04401496	3.01710065	0	0
STRIP2	830.210881	1067.5364	128.3755943	511.90141	303.2894997	365.1425331
IFT22	220.738423	285.421246	278.8157439	240.362352	933.0434443	271.8182629
MKLN1	2348.03172	2082.25464	1994.836384	1925.91591	2125.041711	2546.031062
DNAJB9	339.898102	382.931708	617.8075477	290.647362	370.7991226	501.9577254
FLNC	12366.4404	10975.0056	0	3.01710065	1060.001839	440.3455858
LRRC4	4.88359342	0	1.002934331	9.05130194	6.045637868	19.93333928
CALU	10350.2879	11187.294	2970.691488	3082.47116	5937.823992	3214.703989
CCDC136	89.8581189	114.777939	8.023474646	12.0684026	222.6809948	35.33637417
SMO	0	0	5.014671654	0	3.022818934	6.342426133
IRF5	61.5332771	74.14858	6.017605984	2.01140043	477.6053916	52.55153082
LRRC17	0.97671868	2.03146794	2.005868661	2.01140043	5.038031556	7.248487009
KLHDC10	1030.43821	1049.25319	1782.214306	1698.62766	2493.82562	1722.421726
NDUFA5	869.279629	827.752086	1855.428512	1362.72379	1294.602817	1364.52768
FEZF1	33.2084353	20.3146794	0	0	3.022818934	0
OPN1SW	0	0	0	0	0	0.906060876
MRPS12	1132.99367	1106.1343	675.9777389	783.440468	1561.789782	974.9215028
MYO1B	5461.81088	4693.70669	26.0762926	68.3876147	6111.132278	895.1881457
HOXD1	111.34593	112.746471	0	0	2.015212623	1.812121752
HOXD3	48.4257123	51.0711041	0	0	0	0
MTX2	1136.90055	1212.78636	676.9806732	673.819144	687.1875043	581.6910825
PDE11A	172.879207	246.823355	37.10857024	95.5415205	4.030425245	73.39093097
CHN1	571.38043	585.062768	1.002934331	0	422.1870444	31.71213067
GAD1	248.086546	233.618814	5.014671654	6.03420129	99.75302482	27.18182629
EIF2S2P4	52.4986293	48.6739719	42.18341795	15.0955602	24.78711526	16.10070177
OSGEPL1	264.690763	359.569826	233.6836991	215.219846	130.9888205	216.5485494
ORMDL1	1476.79865	1424.05903	1259.685519	897.084592	897.7772234	1027.473034
HAT1	2377.33328	2435.73007	1932.654455	1875.6309	2244.946862	2403.779505
HOXD9	0	0	0	0	0	0
HOXD10	0	0	0	0	1.007606311	0

HOXD11	0	1.01573397	0	0	0	0
HOXD13	0	0	0	0	0	0
HERC2	4484.35966	4101.02591	4874.130466	5629.95003	4984.305988	7871.22265
SNRPN	0	0	1086.659289	583.698348	1.007606311	747.3190107
PSMG2	1683.86301	1730.81069	969.8374978	1137.44694	1048.91817	1108.112452
TWSG1	4179.37925	3676.95698	327.9595261	527.992613	1084.184391	373.297081
ARHGAP22	1133.97039	974.088879	0	0	80.6085049	13.59091314
WDFY4	0	0	1.002934331	2.01140043	5.038031556	2.718182629
EIF2AK4	3971.33817	3739.93249	3167.266616	2429.77172	1739.128493	4349.998267
MYO5C	335.014509	288.468448	2184.390972	3307.74801	5784.667833	2759.861429
CGNL1	35.1618726	39.6136249	170.4988362	237.345251	100.7606311	230.1394625
TMOD2	148.46124	200.099593	0	0	291.198224	50.73940907
TTBK2	1120.29633	1091.91402	624.828088	857.862284	373.8219415	749.3123446
ELL3	43.9523408	60.9440383	797.9245242	702.632456	176.9356683	840.0452807
C15orf57	282.2717	362.617028	565.6549625	815.622875	514.8868251	620.6517002
INO80	1839.16128	1541.88417	1175.439036	1221.92576	1739.128493	1894.573292
ICE2	1290.24538	1170.12554	1664.870989	1657.39396	725.4765441	1978.836954
DLL4	10.7439055	6.09440383	31.09096425	11.0627024	23.17494516	32.61819154
ALDH1A2	2.93015605	2.03146794	0	3.01710065	6.045637868	4.530304381
FAM63B	1050.9493	978.151815	1162.400889	1857.5283	357.7002405	1350.936766
IVD	1401.59131	1349.91045	2836.298287	4559.84478	2339.661855	3857.10115
KNSTRN	1405.49819	1450.46811	761.227157	1159.57235	1131.541888	1244.021583
DUT	2142.92079	1989.82285	2565.506018	2929.60473	1368.329371	3286.282798
CHAC1	226.598735	163.53317	1814.308204	66.3762142	146.1029151	202.9576363
CLN6	1153.50477	1304.20242	1355.395543	1898.80223	2466.62025	2184.530894
ARPP19	5592.69118	5578.41098	5701.68167	6817.64176	10018.62955	6482.865569
VPS13C	1522.70443	1457.57825	4260.465037	6157.90242	1613.177704	4960.683297
CALML4	106.462337	92.4317915	202.7331456	181.518832	401.2993656	232.051251
ISLR	0	1.01573397	0	0	0	0
THAP10	96.6951497	95.4789934	23.06748961	36.2052078	220.6657822	54.36365257
LOXL1	938.626655	851.185069	12.03521197	15.0855032	1438.861813	131.378827
ACKR4	0	3.04720192	2.005868661	0	2.015212623	3.624243505
ANAPC13	724.725263	754.690341	854.5000498	688.904648	1195.021085	604.3426044

MBD4	571.38043	642.959604	1106.236567	909.152995	1673.634083	1156.133678
COPB1	7658.82235	7866.85962	3323.082494	2745.56159	3934.702646	2540.957121
PSMA1	3984.86572	3908.54433	1944.238347	1525.64723	3385.557206	1696.780203
SUMF2	3334.51759	3225.9711	2315.77537	2590.68376	2719.529434	2446.364366
PALLD	3255.40337	2721.15131	597.7488611	615.488532	1897.322684	2119.276389
SPCS3	3262.2404	3426.07069	2979.717897	1910.83041	2196.581759	2237.970364
BBOX1	0	0	35.10270157	19.1083041	0	99.66669638
MYOD1	0	0	0	0	0	0
SERGEF	336.880041	327.066339	62.1819285	141.80373	159.2017972	51.64546994
KCNC1	0	0	0	0	16.12170098	0
TPH1	35.0544336	46.7237627	7.020540315	2.01140043	4.030425245	16.30909577
CSRP3	0	0	1.002934331	0	0	0
E2F8	805.792914	705.935111	322.9448545	490.781705	1025.743225	790.9911449
DCTD	2162.45517	2269.14969	989.8961844	872.947787	619.6778814	907.8729979
SOX15	51.7660902	47.7394967	0	2.01140043	3.022818934	2.718182629
FAM64A	950.347279	888.767226	84.24648378	261.482056	565.2671406	224.7030973
RPAIN	1117.36617	953.7742	555.6256192	447.536596	916.9217433	648.7395873
USP6	1.98273893	6.58195614	8.79573408	11.1532154	33.5432141	1.821182361
SHBG	3.90687474	8.12587178	3.008802992	12.0684026	6.045637868	6.342426133
PLD2	613.379333	489.583775	273.8010723	280.59036	411.103375	424.9425509
AIPL1	0	1.01573397	0	0	0	0
CD68	1850.41308	2106.72367	0.381115046	0	125.4469858	43.73555849
TXNDC17	1106.62227	1223.95944	642.880906	465.6392	898.7848297	753.842649
ATP1B2	0	0	0	0	6.045637868	0
FXR2	2536.53842	2692.71076	1256.676716	1046.93392	1662.550414	1254.894314
KIF1C	7585.1973	7317.34754	2903.494887	3792.49551	7213.453583	5246.998534
MPDU1	1278.52476	1260.52586	612.7928761	713.041453	1312.911024	678.6395963
PHF20L1	2414.44859	2611.45204	3108.093491	2621.86046	1583.957121	2357.5704
LRRC6	7.81374947	19.2989455	48.14084787	28.159606	4.030425245	45.30304381
CCNT1	1153.50477	1090.89829	1520.448445	1478.37932	1822.759817	1802.155083
PUS7L	1307.82632	1214.81783	754.2066167	675.830545	1044.887745	1135.294278
KRI1	2287.47516	2493.6269	819.3973482	900.101693	1086.199604	1611.882299
ILF3	18303.7081	17441.168	6476.949908	5969.83648	13596.63956	10551.0789

SLC44A2	4818.15327	4331.08966	4436.981479	7600.07653	6087.957333	6805.423241
AP1M2	730.585576	695.777771	4734.852975	4500.50846	3105.442651	4311.94371
CDKN2D	781.374947	827.823187	492.4407564	701.97875	172.3006792	286.3152369
MTUS1	1001.13665	817.665848	40.11737323	66.3762142	2153.254687	363.3304113
KLK14	0	1.01573397	38.11150457	18.1026039	8.06085049	40.77273943
SIGLEC9	0	0	0	0	0	0
KLK10	0	0	0	0	8.06085049	1.812121752
KLK8	0	0	0	0	19.01353109	5.436365257
NGDN	794.07229	738.438598	767.244763	650.688039	777.8720723	654.1759526
RIPK3	0	1.01573397	0	0	0	0
ADCY4	0	2.03146794	2.005868661	2.01140043	1.007606311	2.718182629
RAB2B	482.49903	478.410701	426.2470906	410.325688	343.5937521	402.291029
BCL2L2	1398.8858	1506.60773	1325.04675	1347.15555	1171.13074	1026.005215
AJUBA	8394.89709	6691.65541	1818.319942	730.138356	1546.675688	870.724502
DTD2	537.732471	545.926538	369.7919171	358.129847	638.600728	407.4465154
PARP2	1364.476	1220.91223	1210.541737	1040.89972	959.2412083	1155.227617
HEATR5A	1226.75867	1348.89472	1196.049336	1787.41088	1233.310125	2172.036314
FOXA1	2.93015605	2.03146794	17461.0867	15763.3452	9714.332447	14735.26803
SNX6	3551.18309	3266.58014	1992.228755	1846.48571	2075.477556	1979.96953
EAPP	659.285112	550.527813	871.5499334	953.403804	758.7275524	1144.354887
EGLN3	449.290595	374.805836	9062.514612	7680.53255	593.4801173	16435.03823
MIS18BP1	3190.93994	2761.78067	1063.110391	1492.45912	1453.975907	1627.285334
NRL	23.4412484	24.3776153	32.09389858	48.2736103	39.29664614	29.90000891
RNASE1	7.81374947	7.11013781	0	0	4.030425245	3.624243505
NEDD8	2705.01263	2729.38891	1764.051165	1664.52437	2346.765479	1806.332023
DAD1	1868.46284	2063.97143	2302.737223	1483.40782	1800.592478	1385.36708
TEP1	682.72636	930.412319	922.6995843	1509.55602	634.7919761	1352.748888
EPB41L4A	8.79046815	21.3304134	2363.916217	3493.80255	40.30425245	2546.031062
CDO1	0.97671868	0	3.008802992	1.00570022	0	2.718182629
REEP5	3324.7504	3266.60045	9519.852667	9038.22784	4079.797954	10079.02119
ITFG1	883.930409	885.720024	2166.338154	2381.49811	1471.105214	2535.158332
QRICH2	86.9279629	128.998214	217.6367498	231.31105	49.37270925	257.3212888
FOXJ1	0	0	1.002934331	0	0	9.966669638

SEC14L1	6130.65807	5165.81984	1001.891279	1685.57368	2573.174617	1626.578606
RHBDF2	1432.84631	1521.56949	242.710108	343.949474	1050.933383	851.6972236
AANAT	1.95343737	1.01573397	2.005868661	1.00570022	3.022818934	1.812121752
ARHGEF6	16.6042176	21.3304134	161.4724272	124.706827	29.22058303	79.7333571
MAP7D3	2306.03281	2092.41198	0	0	1038.842107	107.8212443
FGF13	0.97671868	0	1398.090457	1119.34434	180.3615297	488.3668123
ASH2L	1338.1046	1012.68677	1575.609834	1627.22295	1028.766044	1514.933785
TTI2	229.528891	269.169503	213.6250124	264.499157	321.4264133	226.515219
ART1	0	0	0	0	1.007606311	0.906060876
CHRNA10	28.3248418	23.3618814	11.03227764	16.0912034	11.08366942	23.55758278
CDKN1C	180.692957	110.715003	17.04988362	28.159606	14.10648836	17.21515665
SGOL1	729.608857	779.067957	251.736517	513.91281	843.3664825	538.2001604
TTTY1B	0	0	0	0	0	0
RPS4Y1	0	0	0	0	0	0
TTTY1	0	0	0	0	0	0
VCY1B	0	0	0	0	0.503803156	0
VCY	0	0	0	0	0.503803156	0
CDY2B	0	0	0	0	0	0
CDH15	3.90687474	6.09440383	7.020540315	9.05130194	21.15973254	13.59091314
KLF16	1617.44614	1475.86146	740.1655361	996.648914	881.6555224	1242.209461
TMEM8A	1388.89397	1635.3317	1264.700191	1183.70915	2689.301245	1665.33989
DOHH	792.118853	828.838921	506.481837	471.673401	510.8563998	540.9183431
MAU2	1625.25989	1522.58522	1468.29586	1319.47868	1515.439892	1763.194465
SHC2	22.4645297	20.3146794	113.3315794	29.1653063	47.35749663	76.1091136
PLPPR3	1.95343737	1.01573397	0	0	51.38792188	20.83940015
INS-IGF2	0	0	0	0	0	0
ABHD17A	2135.82005	2063.6464	2163.840848	2316.35891	1341.990542	2168.828859
LBP	0	0	3.008802992	2.01140043	0	0.906060876
SYT5	1.95343737	0	11.03227764	0	3.022818934	7.248487009
TNNI3	2.93015605	4.06293589	4.011737323	5.02850108	1.007606311	0
CBFA2T3	0.97671868	0	0	0	4168.46731	439.4395249
GAMT	876.116659	832.901857	1204.524131	1199.80036	103.7834501	1205.060965
PUDP	838.024631	736.40713	537.5728013	498.827307	1232.302519	419.5061857

ERMARD	699.330578	594.204374	570.6696342	464.6335	236.7874832	442.1577076
PHF10	3554.26952	2792.25269	1526.466051	1207.84596	1343.139213	1619.067361
PRRG3	0	0	0	0	0	0
GALNT8	10.7439055	36.566423	3.008802992	5.02850108	1.007606311	0
KCNA5	0	0	0	0	0	0
CRACR2A	669.052298	591.157172	7.020540315	6.03420129	370.7991226	36.24243505
NXNL2	1.95343737	0	1.002934331	0	66.50201655	5.436365257
STARD8	659.285112	656.164146	11.03227764	40.2280086	40.30425245	41.6788003
FAM155B	0	0	427.2500249	343.949474	338.5557206	339.7728286
GDPD2	0.97671868	0	2.005868661	0	6.045637868	3.624243505
SAT1	1071.4604	800.39837	1614.724272	1797.18629	1219.203637	1466.006498
GNL3L	1070.48368	980.183283	784.2946466	741.201059	1607.132067	934.1487633
SH3BP4	3012.20042	2320.95213	5163.105934	4281.26582	1658.519988	4571.07712
MOSPD2	535.241839	685.620431	539.5786699	1005.70022	415.1338003	298.0940283
DOCK6	673.935892	844.074931	670.9630672	742.206759	2151.239475	1321.036757
ECSIT	1095.87836	980.183283	746.183142	830.708378	680.1342601	737.5335532
LDLR	9858.99839	14897.7702	662.9395926	12627.5719	2063.577726	933.2427025
ELOF1	647.564487	820.71305	807.3621362	891.050391	1180.914597	949.5517982
TSPAN16	0	0	0	0	0	0
C19orf80	0	0	0	0	0	0
PRKCSH	8408.57115	8434.65491	5085.879991	5563.53359	6797.312176	5748.956259
CNN1	3.90687474	4.06293589	0	0	0	1.812121752
CDC16	2369.51953	2061.93996	872.5528677	758.297963	1325.002299	885.221476
ZSCAN10	0	0	1.002934331	1.00570022	1.007606311	0.906060876
THEM6	847.791818	1028.93851	3834.217946	4879.65745	2720.53704	3012.652413
EXOC3L2	0	0	0	0	0	0
PVRL2	4254.58659	3646.48496	4006.722651	4618.17539	3552.819854	4579.231668
APOE	26.3714045	23.3618814	4.011737323	2.01140043	28.21297672	16.30909577
TOMM40	5686.44641	5311.27294	2254.586346	2085.82225	5474.315013	4223.140683
APOC1	70.3237452	69.0699101	0	13.0741028	23.17494516	2.718182629
GADD45G	1.95343737	3.04720192	515.508246	129.735328	86.65414277	80.63941798
LRCH2	0	0	0	0	0	0
DPP6	0	0	0	0	1.007606311	0.906060876

XPO7	1912.41518	1842.54143	1447.234239	1405.9689	4069.721891	2197.197625
ACE2	0	2.03146794	0	0	11.08366942	3.624243505
FAM98C	284.225137	243.776153	686.0070822	546.095217	643.8604329	616.1213958
SAFB2	2928.4761	3078.16149	1771.071705	1918.76538	2397.427925	2564.088855
RPL36	11115.42	11883.0717	10189.8128	8475.66931	7041.152903	8418.682752
ATP8B3	419.989034	434.73414	34.09976724	186.05454	39.29664614	43.49092206
GDF1	2.04134205	2.68153769	11.34318728	0	71.12692951	0
NCAN	0	0	0	1.00570022	1.007606311	1.812121752
KIF1A	0	0	1.002934331	0	812.1306869	144.9697402
GTPBP3	1023.60118	961.900072	539.5786699	701.97875	746.6362767	754.7487099
PLVAP	0.97671868	2.03146794	16.04694929	3.01710065	0	10.87273051
BST2	2182.96626	2583.01149	3980.646359	4340.60213	17.12930729	7092.644539
SLC27A1	701.284015	721.17112	501.4671654	605.43153	152.148553	546.3547083
NSUN5	776.579258	770.31233	1134.078024	824.835089	1181.247107	1039.206522
USHBP1	0.97671868	1.01573397	0	0	0	0
COLGALT1	6772.56735	6931.36863	1173.433167	1594.03484	2430.346423	2192.66732
DDA1	2355.84547	2077.17597	1132.312859	945.358203	853.4425457	781.9305361
MRPL34	828.257444	958.85287	1299.802893	1401.9461	697.2635674	958.612407
PGLS	1260.94382	1155.90526	1782.214306	1687.56496	936.0662632	1245.833705
LSM7	1090.99477	1014.71824	795.3269243	672.813444	700.2863863	874.3487455
TULP4	797.002446	815.63438	1946.695536	2275.89959	1424.755324	2699.15535
SNX9	1724.8852	1716.59041	1413.134472	1384.8492	864.5262151	1166.100348
RTN4IP1	256.877014	299.641522	168.4929676	171.974737	150.1333404	221.9849147
QRSL1	403.384816	470.284829	537.5728013	545.089517	557.2062901	744.7820402
C6orf203	199.250612	166.580371	302.8861679	284.613161	220.6657822	242.8243148
RSPH3	193.390299	241.744685	625.8310224	658.733641	274.0689167	527.3274299
MAS1	0	0	0	0	0	0
ACSBG2	0	0	0	0	0	0
MLLT1	4452.86048	4652.06159	5827.048461	6335.91136	2195.574152	7234.896096
FUT5	0	0	0	0	0	0
BMP15	0	0	0	0	0	0
MLLT4	5159.02809	4959.82899	4434.97561	4178.6844	3032.894997	4885.480244
ACTN4	34017.3244	27388.8907	11054.34219	10935.9841	17775.18294	11628.23125

STK33	279.341544	315.893265	0	0	9.068456802	8.154547886
NDUFA10	1536.37849	1510.39642	2220.496608	2035.53724	2852.533467	2478.982557
EPO	1.95343737	0	10.02934331	0	1.007606311	0
ARPC1B	3461.42265	3510.79306	507.6151528	493.074702	6949.057686	998.5515704
CACNG6	1.95343737	2.03146794	0	0	8.06085049	1.812121752
ZSWIM6	1516.84412	1443.35797	287.8421529	326.85257	589.4496921	418.6001248
FCHO1	123.066554	155.407298	6.017605984	2.01140043	743.6134577	90.60608762
UNC13A	60.5565584	179.784913	0	0	15.11409467	4.530304381
MAP1S	1841.11472	1982.71271	1314.846908	1526.65293	696.2559611	1506.779237
KLHDC7B	0.97671868	0	23.06748961	0	17.12930729	14.49697402
SCO2	274.1454	332.25674	559.0255666	507.647298	273.9983842	614.6988802
PXDN	16923.6046	13283.7689	1.002934331	0	2381.98132	671.3911092
SSBP4	1176.94601	1069.56787	967.8316291	878.981988	1313.91863	1203.248844
GDF15	202.180768	428.639736	5434.901138	1915.85891	2219.756704	3315.276746
PGPEP1	854.628848	1255.44719	1975.780631	1689.57636	842.3588762	1908.164205
KIAA1683	16.6042176	30.4720192	56.16432252	221.254047	138.0420646	90.60608762
LSM4	3423.39899	3156.90119	2812.227863	2746.56729	4006.242694	3009.934231
JUND	2529.70139	2628.71952	2710.931496	5058.67208	2318.502122	1800.342961
HRC	0	0	5.014671654	10.0570022	2.015212623	9.966669638
TRPM4	334.03779	328.082073	2779.13103	4230.98081	1247.416613	3127.722145
OR11H1	0	0	0	0	0	0
SULT4A1	0	0	0	0	0	0.906060876
ZNF557	236.365921	229.555878	337.9888694	475.696202	261.9776409	554.5092562
CRB3	2.93015605	3.04720192	734.1479301	924.238498	338.5557206	594.3759348
OLFM1	0	0	1.002934331	1.00570022	1103.328911	36.24243505
CAMSAP1	2188.82657	1959.35083	1388.061114	1395.9119	3122.571959	1954.37331
UBAC1	907.371657	934.475254	802.3474646	722.092755	1822.759817	834.482067
SAG	0	0	1.002934331	0	0	3.624243505
ZBTB46	65.4401518	63.9912403	64.18779717	61.3477131	420.1718318	140.4394358
HELZ2	2663.51185	2134.05708	762.2300913	703.990151	2137.132986	2561.434097
SAMD10	363.33935	347.381019	392.1473233	282.601761	191.4451991	347.9273765
LSP1	3.02782792	4.3473414	0	0	2.408179084	0
TNNT3	0	0	0	0	0	0

TNNI2	0	0	0	0	8.06085049	1.812121752
H19	4.88359342	0	10.02934331	4.02280086	1277.57427	148.5939837
CYP2G1P	0	0	13.0381463	2.01140043	4.030425245	5.844092651
COL5A1	3418.51539	2859.29113	0	0	677.1114412	91.51214849
ATXN10	2680.98535	2223.81749	4186.568835	4135.51974	1764.933291	4628.086471
TUBGCP2	2977.03855	2674.42755	3235.466151	2773.72119	2622.799228	3370.546459
CALY	4.88359342	0	2.005868661	0	1.007606311	0
CYP2E1	3.90687474	4.06293589	207.6074065	87.4959188	2.015212623	144.9697402
PNPLA7	14.6507803	44.6922948	3436.053017	2588.67235	85.64653646	759.2790142
HBZ	0	1.01573397	0	0	0	0
PAK4	2660.58169	2620.59365	5321.569559	6248.41544	4205.748743	5285.053091
MNX1	135.763897	146.265692	2078.079933	659.739341	765.7807966	765.6214404
ZNF337	490.312779	347.381019	632.8515627	413.342789	603.5561805	525.5153082
CEP85	1183.78304	1221.92797	631.8486283	775.394866	695.2483548	918.7457284
TAF4	1385.96381	1300.13948	992.9049874	1018.77432	1985.99204	1231.354852
GATA5	0	0	0	0	10.07606311	2.718182629
RBBP8NL	0	0	321.9419202	509.890009	477.6053916	393.2304203
LAMA5	14363.625	10636.7662	5584.338353	6608.45612	13098.88205	9273.533068
OSBPL2	1440.66006	1425.07476	3276.586458	2881.33112	3176.982699	3183.897919
ADRM1	4806.43264	4846.06678	2170.349892	2080.79375	9020.091699	2796.103864
ASS1	331.107634	419.498131	6082.796716	1317.46728	3748.295478	1448.791341
PRDM12	5.8603121	4.06293589	11.03227764	12.0684026	21.15973254	12.68485227
EXOSC2	1523.68115	1299.12375	633.854497	500.838707	1859.033644	1166.100348
POMT1	780.398228	796.335434	1056.08985	1229.97136	1019.697587	1214.121574
UCK1	850.721974	820.71305	923.7025186	934.2955	1229.2797	1109.924573
FIBCD1	40.045466	53.8339005	36.10563591	380.154681	707.3396305	126.8485227
PRRC2B	8233.7385	7354.92969	7894.096117	8196.45676	15792.21372	12673.97954
CHMP2A	1238.47929	1161.99966	2909.512493	2095.87925	1436.8466	2017.797571
UBE2M	2990.71261	2965.9432	2143.270665	1865.5739	3601.164804	2150.082459
TRIM28	12549.8584	13275.643	6333.530298	7803.22797	22338.63192	10633.53044
C16orf13	572.357149	547.480611	1249.656176	724.104155	1172.853746	1268.485227
YIPF2	702.260734	803.445572	1691.950216	1312.43878	1337.093575	1280.264018
ATG4D	913.231969	916.192043	670.9630672	867.919286	1000.553067	704.9153617

EIF2S3	7714.06556	6254.84917	7329.414001	6377.0445	6152.38368	5602.120034
TMEM160	566.496837	578.968364	390.1414546	560.17502	482.6434231	401.3849681
ZC3H4	2008.13361	2088.34905	1255.673782	2177.34097	2393.064989	2134.679424
NPAS1	58.603121	82.2744518	70.20540315	80.4560172	9.068456802	38.96061768
GMFG	4.88359342	4.06293589	0	1.00570022	8.06085049	2.718182629
MAP3K10	459.057781	520.055794	555.6256192	490.781705	267.0156725	440.3455858
ARHGEF16	26.3714045	23.3618814	2115.188503	2230.64308	1118.443006	2024.139997
LRRC47	1218.94492	1280.84054	1576.612768	1329.53568	1509.394254	1836.585396
SESN2	334.03779	259.012163	2407.042394	1430.10571	813.1382932	1032.003338
SMPDL3B	43.9523408	58.9125704	173.5076392	350.989375	183.3843487	416.788003
ATPIF1	1216.01476	1352.95765	3975.631687	4841.44084	1394.527135	3505.54953
MED18	302.782792	299.641522	484.4172817	548.106617	352.662209	373.297081
THEMIS2	125.99671	186.895051	3.008802992	3.01710065	11.08366942	15.4030349
CLIP1	4063.14972	3858.77336	2730.990183	2754.61289	3792.630156	2602.206836
CCDC62	8.79046815	12.1888077	7.020540315	16.0912034	3.022818934	19.93333928
HIP1R	1925.11253	2051.78262	3129.155112	3198.12669	2209.680641	3031.679692
ZNF317	1284.38507	1312.32829	1230.600424	1185.72055	774.8492534	1310.164027
PPAN	1465.37104	1317.8742	663.9425269	538.049615	1358.253308	1375.55444
EIF3G	3325.72712	3412.86615	2549.459069	2008.38333	2758.82608	2543.312879
ANGPTL6	9.76718684	7.11013781	17.04988362	29.1653063	3.022818934	26.27576541
C19orf66	418.035597	417.466663	457.3380548	488.770305	30.22818934	671.3911092
DNMT1	12686.599	11534.675	4788.008495	5319.14844	7433.111758	8208.005477
ZNF426	689.563391	661.242816	460.3468578	507.878609	33.25100827	584.4092651
SLC6A8	251.016702	262.059365	3898.385685	3321.75741	853.4425457	3994.804282
PNCK	0	1.01573397	30.08802992	20.1140043	0	9.060608762
DKC1	3244.65947	3107.13022	4307.60295	3301.71381	4764.970246	8984.499648
PLXNA3	1298.05913	1329.59577	6966.381861	8487.10412	2185.498089	7340.905219
DUSP9	14.6507803	27.4248173	1.002934331	1.00570022	318.4035944	17.21515665
MPP1	728.632138	806.492774	58.17019118	99.5643213	20.15212623	144.0636793
ZNF331	508.870434	520.055794	569.6666998	376.131881	918.9369559	648.7395873
ZNF236	785.281822	606.393181	479.4026101	623.534134	634.7919761	561.7577432
SLC7A10	0	0	0	0	0	0
LRP3	546.454569	627.591549	2718.463533	2096.02005	1556.499849	2223.092845

C12orf65	942.53353	755.706075	551.6138819	427.422592	579.373629	388.7001159
NOL11	2541.42202	2589.1059	3615.568233	3333.89621	5403.792647	5127.398498
UBE4B	1737.58254	1838.47849	1842.410424	3224.27489	2033.359612	1993.333928
CASZ1	303.759511	218.382804	2199.434987	2925.58193	848.4045141	3657.767757
PKDREJ	0	0	10.02934331	6.03420129	0	10.87273051
HSD17B3	0	0	0	0	0	1.812121752
NUTM2F	0	0	0	2.01140043	0.503803156	0.906060876
HABP4	732.539013	689.683367	135.3961346	330.875371	551.1606523	124.13034
FBP2	0	0	0	0	2.015212623	2.718182629
SLC35D2	348.68857	408.325057	1.002934331	3.01710065	521.9400692	73.39093097
PRRG1	372.276326	425.308129	284.3820295	313.778467	220.6657822	196.6152101
UBA1	12330.0967	12497.5908	11532.74187	14736.5253	11726.52225	11983.56115
RGN	3.90687474	7.11013781	0	2.01140043	0	0.906060876
POLN	9.04441501	18.6184037	211.6392025	162.410528	9.441271137	207.0349102
TXLNGY	0	0	0	0	0	0
TTTY9B	0	0	0	0	0	0
PPIL4	757.933699	651.085476	661.9366583	655.716541	780.8948912	859.8517715
ULBP2	218.443134	246.406904	112.328645	86.4902185	173.2780574	103.6533642
AKAP12	2780.71809	2364.62869	0	0	294.2210429	80.63941798
SYNE1	667.098861	735.391396	40.11737323	173.986137	144.0877025	66.14244396
ULBP3	59.5798397	96.9518077	1.002934331	13.0741028	57.39325549	21.74546103
LATS1	1340.05803	1191.45595	924.7054529	897.084592	1574.888665	1243.115522
EPS8L1	208.04108	277.295374	1631.774156	1443.17981	718.4232999	1294.760992
LILRB2	0	0	0	0	0	0
AAR2	1638.93395	1885.20225	1759.38752	1368.75799	1029.77365	1494.094385
TTLL9	0	0	2.005868661	6.03420129	5.038031556	13.59091314
BPIFA2	0	0	3.008802992	0	1.007606311	0
RBM39	9048.32189	7372.19717	8752.607904	6290.65485	4604.760843	6166.650323
COX4I2	0	0	0	0	0	0
BPIFA3	0	0	0	0	0	0
ZNF341	197.297174	213.304134	303.8891022	307.744266	183.3843487	370.5788984
GGT7	57.6264023	41.6450929	326.9565918	895.073192	118.8975447	428.5667944
DEFB118	0	0	0	0	0	0

ACSS2	1407.45162	2452.99754	1008.951937	21353.027	554.1834712	885.221476
EDA2R	0	0	6.017605984	0	249.8863652	42.58486118
ARHGEF9	402.408098	388.010377	887.5968827	1447.20261	506.8259746	511.924395
C1QL1	137.717334	108.683535	1.002934331	4.02280086	54.41074081	9.966669638
GFAP	166.042176	170.643307	0	0	2.015212623	0.906060876
PYY	1.95343737	0	0	0	0	0
HIGD1B	0	0	0	0	0	0
ATP6V1E1	1544.19224	1863.87184	3952.564197	3682.87419	2210.688247	2454.518914
ZNF227	362.362632	439.81281	506.481837	466.6449	270.0384914	484.7425688
ZNF428	513.754028	532.244601	445.3028428	394.234484	473.5749663	381.4516289
TEX101	0.97671868	0	0	0	0	0
ZNF141	281.294981	262.059365	303.8891022	276.567559	335.5329017	340.6788894
CCL25	0	0	0	0	2.015212623	0
COX4I1	3181.17275	3319.41862	4375.802485	4243.04921	8660.376246	4263.016422
EMC8	1054.85618	936.506722	676.9806732	652.69944	2286.25872	884.3154151
GSE1	1130.06352	949.711264	9088.590905	6506.88039	17200.84734	9147.590606
RP11-178L8.4	10.0113665	9.99482229	0	23.7345251	30.06697233	26.24858358
GINS2	739.376044	570.842492	255.7482543	508.884309	2414.224722	734.8153706
CHMP1A	2167.33876	2108.66373	1778.202568	1675.49656	5779.629802	2259.715825
SH3BGRL	501.056685	535.291803	2497.306483	2405.63492	1551.713719	3005.403926
COX7B	1575.44724	1824.25821	3435.050083	3082.47116	1582.949515	1969.776345
SLC34A1	0	1.01573397	0	0	4.030425245	1.812121752
F12	74.23062	119.856609	544.5933416	600.403029	487.6814547	580.7850216
PRR7	365.292788	298.625788	327.9595261	307.744266	1074.108328	700.3850573
NFATC1	199.250612	173.690509	5.014671654	7.03990151	330.4948701	35.33637417
IDO1	8.79046815	15.2360096	0	0	0	0
GJA9	0	6.09440383	0	1.00570022	0	0
CAP1	8295.27178	8058.83334	7243.181707	6155.89102	5653.679013	6529.074674
PPT1	2921.36558	3162.99559	6868.094297	6750.25985	4403.23958	6986.635416
RAB11FIP4	8.79046815	12.1888077	784.2946466	1075.09353	1799.584872	1042.876068
RLIM	3179.21932	3140.61897	2509.341695	2420.72042	2388.026958	2430.05527
CDX4	0	0	0	0	0	0
ABCB7	611.425896	569.826758	837.4501661	1017.76862	321.4264133	654.1759526

TRAF3	3139.17385	2867.417	742.1714047	642.642438	2219.756704	1322.848879
HAUS8	540.125432	512.945656	383.1209143	458.599298	516.9020377	770.1517448
ADGRE3	0	0	0	0	0	0.906060876
MRPS25	1290.24538	1250.36852	1890.531213	1083.13913	2857.571499	2349.415852
SH3BP5	377.990131	565.763823	190.5575228	382.166082	2945.233248	1089.991234
HACL1	414.128722	395.120515	443.2969742	284.613161	674.0886223	443.9698293
TBC1D5	1186.7132	1477.89293	1131.309925	1515.59022	2784.016238	1553.894403
CAPN7	1101.73868	1309.28109	1665.873923	1563.86384	2132.094955	2001.488475
RFTN1	2424.21577	2362.59722	28.08216126	6.03420129	232.7570579	128.6606444
C3orf20	0.97671868	0	0	0	1.007606311	1.812121752
RBSN	1367.40616	1301.15522	1360.981887	1393.9005	2156.277506	1842.927822
GALNT15	0	0	0	4.02280086	0	0
SLC6A6	5517.48385	7053.2567	262.7687946	1043.91682	3937.725465	1229.524609
KCNC3	26.3714045	36.566423	460.3468578	397.251585	385.9132172	493.8031775
NAPSA	14.6507803	17.2674775	52.1525852	93.53012	19.14451991	46.20910469
NAPSB	0	0	2.005868661	3.01710065	0	0
NR1H2	1428.93943	1515.47509	1412.131538	1609.12034	1110.382155	1321.942818
LRRC4B	0	0	8.023474646	6.03420129	9.068456802	9.060608762
PDLIM4	960.114466	938.53819	0	0	65.49441023	35.33637417
KIF3A	778.444791	914.160575	1009.954871	810.594374	745.6286704	828.1396408
MGAT1	3914.68848	4416.41131	1789.234846	1493.46482	2123.026498	1820.2763
GFPT2	2335.33437	3688.13005	0	0	392.9664614	136.8151923
TUBG1	3665.74243	3525.57199	781.7171054	887.42987	3195.290906	1510.457844
PSME3	6231.4652	5887.1941	1299.802893	1265.17087	4429.437344	2219.849147
RPL27	9734.95512	10139.0565	4724.823632	3288.6397	8232.143563	4002.976951
PSMC3IP	576.264023	627.723595	116.3403824	90.5130194	383.8980046	194.8030884
AOC3	24.4179671	45.7080288	15.04401496	8.04560172	6.045637868	38.0545568
ACLY	10117.8288	13041.0085	3967.608212	33525.0167	8337.942226	6186.583663
VPS25	1480.70552	1364.13072	458.3409891	451.559397	1555.744145	604.3426044
RAMP2	0	0	0	0	2.015212623	0
AOC2	51.7660902	78.2115159	35.10270157	27.1539058	28.21297672	64.33032221
G6PC	0	0	0	0	0	0
RP11-798G7.5	1.1134593	0	11.04230698	11.1632724	3.707991226	7.311911271

NDUFA2	972.811809	956.821402	1275.732469	1258.13097	792.986167	1001.197268
ANKHD1	3043.73867	3877.14799	2492.492399	3085.84026	3546.290565	3248.590665
DIAPH1	9197.75985	8848.05863	3545.372859	4385.85864	9013.038454	5919.295704
NDFIP1	2314.82328	2511.91011	3467.143981	2970.83844	1760.288226	2677.409889
UBE2D2	2920.94559	2975.50126	2104.757986	2497.82745	2445.823256	2314.387538
TTTY6	0	0	0	0	0	0
TTTY6B	0	0	0	0	0	0
EXOC4	1573.4938	1581.49779	1216.559343	1400.9404	1626.276586	1236.773096
ACAP3	811.653226	917.207777	3061.958512	3671.81149	1210.13518	3462.964669
C1orf159	512.777309	518.024326	555.6256192	750.252361	447.3772022	663.2365614
ANO1	1.95343737	11.1730737	2186.396841	3330.87911	26.19776409	1707.018691
PPFIA1	3649.99772	3689.14579	5188.179293	5409.66146	3529.644908	5747.144138
TMEM204	0	0	1.002934331	0	0	0
KREMEN2	67.3935892	49.7709646	87.25528677	91.5187196	304.297106	446.688012
THOC6	1342.01147	1170.12554	452.3233831	625.545534	707.3396305	520.0789429
TRAF7	2508.21358	2553.55521	2713.940299	3458.60304	5545.865137	4427.013441
BARX1	0	0	3.008802992	0	0	0
NINJ1	709.097764	763.831947	1922.625112	2256.79128	3309.986733	1456.039828
CA6	0	0	0	0	0	0
NPHP4	415.105441	390.041845	835.4442975	782.434768	481.6358168	960.4245287
MAP1B	16243.8084	14475.2248	29.08509559	6.03420129	791.9785607	369.6728375
RHOXF2	0	0	0	0	0	0
IL13RA1	1918.2755	1974.58684	1005.943134	1248.07397	3447.021191	1023.84879
WDR44	863.419317	880.641354	669.9601329	897.084592	749.6590956	668.6729266
CKMT2	0	0	0	0	1.007606311	0
ZCCHC9	820.443694	769.926351	435.2734995	349.983675	552.1682586	418.6001248
KRT34	21.487811	21.3304134	1.002934331	0	0	0
KRT33B	0	2.03146794	0	0	0	0
TNS4	25.3946858	11.1730737	21.06162094	3.01710065	80.6085049	42.58486118
TOP2A	19016.7128	18045.5298	1706.994231	3901.11114	9267.962851	4474.128607
STARD3	1289.26866	1395.61848	2368.930889	2647.00297	515.8944314	2781.60689
RARA	926.906031	1020.81264	697.0393598	460.610699	2934.149578	878.8790499
PPP1R1B	1.95343737	0	0	1.00570022	0	0.906060876

KHDRBS3	600.681991	536.307537	60.17605984	36.2052078	52.39552819	48.02122644
CHD1L	1330.29085	1123.40177	1281.750075	901.107393	2300.365209	1672.588377
PEX11B	261.584798	186.895051	624.8080293	683.876147	802.8506328	605.2486653
FMO5	10.7439055	20.3146794	141.4137406	72.4104155	27.2053704	144.9697402
PIAS3	1182.80633	1098.00842	1307.826367	2322.1618	3530.652515	1882.794501
PRKAB2	440.500126	418.482397	814.3826765	490.781705	1512.417073	929.618459
CLUHP3	134.787178	124.935279	1232.606292	925.244198	572.3203848	1203.248844
FSHB	0	0	0	0	0	0
PDHA1	2472.07499	2686.61636	2857.359908	2480.05673	2997.628776	2374.785556
RAI2	0	0	0	0	19.14451991	9.966669638
MCCC2	2056.96955	1928.87881	4030.793075	3417.36933	4738.772482	6888.780842
ZNF304	392.640911	365.66423	445.3028428	393.228784	321.4264133	482.0243861
ZSCAN5A	27.3481231	57.8968364	88.2582211	101.575722	108.8214816	137.7212532
ZNF132	29.3015605	21.3304134	2.005868661	0	1.007606311	3.624243505
USP29	0	0	0	0	0	0
VIMP	1062.66993	1366.16219	889.6027513	710.024352	1334.070756	635.1486742
CHSY1	2266.96407	2139.13575	587.7195178	528.998313	1303.842567	1009.351816
SNRPA1	1560.79646	1449.38128	885.5809847	740.195359	3724.777947	1214.076271
KRT17P1	0	0	0	0	2.015212623	0
LLGL1	2460.35436	2266.10249	767.244763	994.637513	1263.538314	1293.854931
NR0B2	0	0	0	1.00570022	0	0
LIN28A	0	0	0	0	0	0
THAP1	285.201856	317.924733	225.6602244	365.069178	300.2666808	277.2546281
RHPN2	830.201114	953.7742	1129.304056	1226.95426	1718.936063	1210.497331
C19orf12	722.771826	799.382636	770.253566	740.195359	403.0425245	578.066839
FAAP24	112.322649	149.312894	132.3873317	161.917735	170.2854666	206.5818798
LRRC9	0	0	1.002934331	2.01140043	0	1.812121752
ACTR10	1660.42176	1698.3072	1961.739551	1611.13175	1117.435399	1248.551887
ABHD12B	0.97671868	0	0	0	0	0.906060876
GCH1	379.943568	443.875746	1239.626833	1374.79219	945.13472	1252.176131
LGALS3	2132.17689	2100.53785	4846.178686	2711.36778	4787.137585	2348.509791
UBE2L2	0	0	0	0	0	0
PODNL1	15.6274989	30.4720192	7.020540315	18.1026039	81.61611121	24.46364366

DNAJB1	4207.70409	4127.94286	3804.129916	4634.26659	4436.490589	4166.97397
ZSWIM4	320.363728	311.830329	299.8773649	206.168544	254.9243968	318.0273675
FBXW9	212.924673	239.713217	580.6989775	457.593598	376.8447604	635.1486742
RFX1	674.912611	623.660659	740.1655361	704.995851	668.0429844	617.9335176
ZNF20	71.4176702	58.0288818	0	0	22.24794735	2.083940015
C19orf57	61.5332771	69.0699101	696.0364255	681.864746	71.5400481	1356.373132
DCAF15	1173.03914	1257.47866	492.4407564	734.161157	1133.5571	786.4608405
CC2D1A	1218.94492	1295.06081	3327.736109	2594.70656	2705.422946	3196.582771
RTBDN	4.88359342	9.14160575	191.5604572	276.567559	4.030425245	419.5061857
MATN3	308.643104	275.263906	1.002934331	0	514.8868251	70.67274834
TRIM21	333.061071	350.42822	177.5193765	212.202745	65.49441023	231.9515843
SPATA6	0.97671868	0	173.5076392	153.872133	19.14451991	221.9849147
LRRC41	1470.93834	1391.55554	2738.010723	2200.47207	2318.502122	2575.02501
CCT6B	19.5343737	11.1730737	91.26702409	63.3591136	26.19776409	79.7333571
DHX30	4441.13986	4565.72421	2936.59172	3000.00374	2476.696313	3510.940592
RAF1	3313.02978	3368.17385	2617.658603	2525.31324	4727.688813	3346.988877
SLC6A11	4.88359342	7.11013781	0	0	0	0
PPARG	1063.64665	1132.54338	0	0	641.8452203	70.67274834
NUP210	4678.4825	3900.41845	3306.674488	3575.26427	9818.115897	5971.847235
FCRLA	0	3.04720192	0	0	1.007606311	0
HSD17B7	172.224806	449.066146	261.6755962	3047.18114	219.0838403	236.3550402
ENOSF1	1386.94053	1057.37907	1057.092785	698.96165	1446.922663	790.085084
LINC00470	1.95343737	1.01573397	0	0	0	0.906060876
EMILIN2	303.759511	281.35831	0	0	16.12170098	7.248487009
SLX1A	113.875631	292.155562	159.6169987	165.417571	405.8638222	288.7978437
ARFIP2	1506.10021	1615.01702	6306.451071	5323.17124	1105.344123	3186.616102
TRIM5	346.735133	481.457903	355.0387531	183.037439	189.4299865	324.3697937
CNGA4	0	2.03146794	31.09096425	11.0627024	2.015212623	5.436365257
TRIM22	41.9989034	39.6136249	4.011737323	7.03990151	2.015212623	36.24243505
RRP8	524.497933	498.72538	514.5053117	448.542296	382.8903983	603.4365435
TIMM10B	563.98667	575.169519	667.5129731	1060.00803	641.7444597	909.2502105
EFR3A	3212.42775	3299.10394	2000.85399	2334.2302	2354.775949	3502.831347
HHLA1	0.97671868	0	1.002934331	0	0	0

PTCD3	3782.83146	3509.36087	1332.899726	986.591911	2061.562513	1714.267178
IMMT	5228.37511	4890.75908	3838.229684	3300.70811	3582.040437	4417.046771
MRPL35	1170.06991	1281.83596	833.3882821	761.294949	1417.671852	856.2184674
IQCA1	0.97671868	3.04720192	0	0	0	0
ILKAP	488.359342	557.637951	428.2529592	510.895709	576.3508101	573.5365346
PER2	136.740616	125.951013	1313.843973	970.500708	1039.849713	1454.227706
RAMP1	403.384816	349.412486	0	0	245.85594	24.46364366
SCLY	741.329481	910.097639	387.9650871	493.637894	373.7614851	559.0395606
PTPRE	782.351666	804.461306	0	0	323.4416259	62.51820046
RAN	9803.97006	9457.2654	5515.868027	6299.57541	15313.13722	9108.330988
PRKAA1	3644.13741	3010.63549	2083.094605	1360.71239	1601.086429	1790.376291
CARD6	268.597638	307.767394	1.002934331	2.01140043	34.25861458	11.77879139
RAP1GAP2	460.0345	349.412486	362.0592934	515.924211	865.5338214	498.3334819
CLUH	4798.61889	4522.04764	2018.906808	1908.81901	6393.262045	4600.977129
INPP5K	637.797301	675.463092	355.0387531	388.200283	500.7803367	468.433473
MYBBP1A	3268.10072	3200.57775	714.0892435	491.787405	2812.229215	1693.427778
RPA1	5310.41948	5228.99849	966.8286948	1165.60655	3884.32233	2217.130964
SERPINF1	0	1.01573397	0	0	28.21297672	10.87273051
UBE2G1	1988.59924	2006.0746	1129.304056	1326.51858	1895.307472	1512.215602
EEFSEC	357.479038	366.679964	892.6115543	783.440468	647.8908582	819.9850929
TBC1D14	2144.87423	2261.02382	1653.838711	2131.07876	1215.173211	1803.061144
TMEM128	239.296078	248.854823	469.3732668	276.567559	255.9320031	337.0546459
COQ3	177.7628	183.847849	239.701305	171.974737	217.6429632	224.7030973
PNISR	1994.45955	1933.95748	5170.126475	2937.65033	1889.261834	3541.791965
POPDC3	268.597638	351.443954	0	0	11.08366942	4.530304381
SEC61G	853.65213	969.01021	535.5669326	315.789868	1276.637196	321.651611
LANCL2	543.055588	598.26731	165.4841646	238.350951	943.1195074	318.0273675
FIGNL1	814.583382	691.714835	721.1097838	805.565873	1154.716833	1352.748888
DDC	0	0	169.4959019	265.504857	3.022818934	85.16972236
FTHL17	0	0	0	0	0	0
GRSF1	2914.52855	2782.09535	2831.283616	3784.44991	3787.592124	3954.955725
ENAM	0.97671868	2.03146794	0	0	0	0
JCHAIN	0	0	0	0	0	1.812121752

ANKRD17	4062.17301	4058.87295	3951.561263	4766.01332	4209.779169	6997.508147
UTP3	964.99806	1055.3476	843.4677721	854.845183	1198.043904	953.1760417
ITGB4	5295.7687	3482.95179	1303.81463	1229.97136	9602.488147	2256.997643
WBP2	2834.43762	3306.21408	3711.859958	6303.72895	2363.844406	2404.685565
H3F3B	10164.7113	10802.3308	13382.14275	15513.9114	25767.46582	13792.03148
UNK	1048.99587	950.726998	1933.65739	2107.94765	1882.208589	1909.070266
TRIM47	830.210881	705.935111	53.15551953	84.4788181	670.058197	126.8485227
ZRANB2	1527.58802	1778.55019	2772.11049	1503.52182	1399.565166	2034.106667
EIF5A	16098.7559	14632.5316	4164.183341	3709.01234	14586.99566	7272.01741
KDM6B	533.288401	568.811024	1874.484264	1828.36299	1658.519988	1314.694331
CLEC10A	0	0	0	0	0	0
SLC52A1	0	0	0	0	28.21297672	0
GUCY2D	0	0	3.008802992	6.03420129	0	1.812121752
GPS2	1582.48938	1696.79376	510.2929875	615.548874	1003.575886	623.3698828
XAF1	288.132012	169.627573	0	1.00570022	5.038031556	31.71213067
DLG4	448.313876	442.860012	176.5164422	189.071641	120.9127574	370.5788984
HRSP12	259.80717	319.956201	424.2412219	345.960874	1092.245241	414.9758813
VPS13B	2881.32012	2446.90314	1582.630374	2185.38657	3931.679827	2813.319021
RGS22	0	0	0	0	14.10648836	25.36970453
MATN2	1306.8496	692.730569	0	0	808.1002617	42.58486118
REEP2	277.388106	436.765608	0	3.01710065	26.19776409	22.6515219
PCBD2	223.668579	178.525403	154.6123564	162.883207	306.9773388	183.8759942
SDF2	931.789624	1000.49796	724.1185868	631.579735	633.7843698	741.1577967
FLOT2	2240.59266	2297.59025	2399.018919	2234.66588	1734.090462	2601.300776
ERAL1	1827.44066	1704.40161	1362.987755	1122.36144	1413.671655	1644.50049
PRMT7	766.724167	790.24103	1233.609227	934.2955	756.7123398	1443.354976
NIP7	1455.31084	1412.88596	402.1766666	400.268686	1824.77503	829.9517626
TERF2	795.049009	730.312726	542.5874729	503.855808	980.4009409	839.9184322
VPS4A	1611.17561	1715.9505	1356.398477	1164.35948	2587.240802	1424.391122
MTSS1L	3564.04648	3220.89243	836.4472318	741.201059	5591.207421	1676.212621
HSPA12B	0	1.01573397	0	0	1.007606311	0
ANKEF1	280.318262	271.200971	500.464231	599.397328	726.4841504	780.1184144
SCP2D1	0	0	0	0	0	0

PCED1A	675.889329	691.714835	576.6872402	723.098455	253.9167904	708.5396052
SNAP25	13.6740616	15.2360096	0	0	0	0
BTBD3	922.999156	934.475254	1038.037032	1167.61795	869.5642466	1046.500312
PCNA	4824.01358	4532.20498	2029.939085	3667.78869	3517.553633	4466.88012
NXT1	789.188697	716.09245	309.9067082	341.938073	670.058197	529.1395517
POLR3F	434.639814	417.466663	355.0387531	346.966574	584.4116605	384.1698115
RIN2	1844.04488	1659.70931	33.09683291	49.2793106	242.833121	118.6939748
PTPRA	2495.51624	2395.10071	1680.948026	1765.00388	1127.511462	2073.067285
SSTR4	0	0	0	0	0	0
DAP3	2150.73454	2035.53088	5361.807284	5431.74664	3434.113754	4274.151911
RHBG	0.97671868	0	0	0	95.72259957	10.87273051
KIAA0907	1466.05474	1450.46811	2456.186176	1595.04054	1567.83542	2604.925019
ATP1A4	0	0	3.008802992	7.03990151	0	1.812121752
NES	411.198566	341.286615	1085.174946	1619.17735	508.8411872	3376.888886
BCAN	1.95343737	0	2.005868661	4.02280086	2.015212623	7.248487009
CRP	0	0	0	0	0	0
ARHGEF11	1942.69346	2244.77208	1687.938479	1957.09262	2386.011745	1888.230866
RAB25	0	0	2583.558836	2198.46067	1248.42422	2394.718896
HAPLN2	0.97671868	0	8.023474646	2.01140043	0	0.906060876
APCS	0	0	0	0	0	0
FCRL2	0	0	0	0	0	0
DCAF8	2162.45517	2138.12001	4118.048362	3090.63745	1739.874122	3769.630033
SYT11	32.2317166	51.8024326	1.002934331	1.00570022	0	0.906060876
IGHMBP2	985.509152	851.185069	1209.538803	1133.42414	674.0886223	1468.72468
ACY3	0	0	6.017605984	105.598523	1.007606311	13.59091314
ALDH3B2	0	2.03146794	11738.16288	17218.3319	183.6765545	7348.878555
MTL5	147.484521	143.21849	750.1948794	690.916048	603.5561805	781.9305361
MMACHC	374.083256	379.884506	472.3820698	348.977975	364.7534847	521.8910647
DPH2	794.07229	798.366902	586.7165835	496.815906	1243.386188	999.3851464
TOE1	385.80388	395.120515	166.4870989	210.191345	419.1642255	405.0092117
NASP	4249.70299	4069.02014	3497.232011	3235.33759	3535.690546	5062.162115
MUTYH	262.737326	262.059365	339.9947381	282.601761	395.9892803	360.6122287
CTNNBL1	1995.43627	2011.15327	804.3533332	875.964888	1665.573233	1192.376113

LPIN3	592.868241	570.842492	927.7142559	773.383466	1024.735619	611.5910914
ZSWIM3	139.670772	130.013948	176.5164422	234.32815	178.3463171	239.2000713
RBM38	936.673218	969.01021	425.2441562	471.673401	3083.275313	728.4729444
VSTM2L	317.433572	456.064554	2.005868661	0	2.015212623	1.812121752
OSER1	1321.50038	1363.11499	639.872103	1001.67741	639.8300077	491.0849949
SERINC3	6513.7369	6069.01048	2897.477281	3487.76835	3343.237741	3304.404015
PPP1R3D	262.737326	306.75166	2023.921479	1703.65617	1134.564707	1610.976238
LINC01260	0	0	0	0	0	2.718182629
DMGDH	0	0	66.19366583	5.02850108	2.015212623	6.342426133
BHMT2	0	0	0	0	0	0
AP3B1	2664.48857	2603.32617	1433.193159	1571.18533	1820.744604	1491.376202
ZBED3	800.909321	633.817999	1102.224829	475.696202	199.5060496	658.706257
INADL	414.128722	363.632762	2142.26773	2275.89959	2200.612184	3155.810032
KANK4	0	0	11.03227764	1.00570022	1.007606311	2.718182629
ANGPTL3	0	0	0	0	0	0
SYT4	0	0	0	0	0	0
SLC14A2	0	0	1.002934331	0	3.022818934	0
FBXO44	194.367018	209.840481	479.4026101	270.533358	195.3849398	353.3637417
RSG1	23.4412484	30.4720192	151.4430839	153.872133	90.68456802	130.4727662
CASP9	251.016702	297.610054	227.6660931	236.339551	122.92797	224.7030973
NMUR2	0	0	0	0	1.007606311	0
DCTN4	2083.34095	2113.7424	2082.091671	2248.74568	2054.509269	2143.740033
PDE6A	4.88359342	1.01573397	1.002934331	0	4.030425245	0
ATP8A2	45.9057781	80.2429838	488.4290191	201.140043	10.07606311	1242.209461
MTUS2	0	0	148.4342809	161.917735	7.053244179	228.3273408
ZMYM5	314.503416	265.106567	557.6314879	384.177482	380.8751857	526.4213691
USPL1	445.38372	401.214919	1002.934331	739.189658	770.8188281	1238.585218
XPO4	1823.53378	1662.75651	1039.039967	1050.95673	1881.200983	1804.873265
TPTE2	0	0	3.008802992	3.01710065	1.007606311	0.906060876
POMP	2031.57486	2290.48011	2867.389251	2373.45251	2431.354029	2426.431026
CDK8	515.707465	629.755063	490.4348877	647.670939	880.6479161	866.1941976
ALOX5AP	12.6973429	15.2360096	0	0	6.045637868	1.812121752
HMGB1P5	374.981837	410.874549	321.410365	629.699076	702.6643372	597.9911177

WASF3	551.846056	562.716621	122.3579883	109.621323	299.2590745	245.5424974
RNF17	0	0	0	0	0	0
GPR12	0	0	0	0	0	0.906060876
CHRM3	11.7206242	8.12587178	8.023474646	0	0	4.530304381
MYH8	0	0	0	0	0	0
MYH10	5319.20995	4364.60888	301.8832335	406.302887	3236.431472	697.6668747
PEMT	377.990131	357.538358	369.0798337	275.561859	371.8067289	304.4364544
SCO1	1725.86191	1406.79155	516.5111803	497.821607	1475.13564	729.3790053
MPRIP	11588.7672	10627.6246	4903.345943	4834.40094	4617.849649	6748.332345
CHI3L1	0	0	0	0	0	0.906060876
MYBPH	0	0	0	0	5.038031556	0
PIK3C2B	192.413581	294.562852	1823.334613	2673.15117	2163.33075	3670.452609
DSTYK	750.119949	783.130893	1976.783566	1752.93548	1819.736998	1875.546014
CHIT1	0	0	1.002934331	4.02280086	0	0
SLC41A1	2092.13142	1979.66551	1437.204896	1780.08938	1455.99112	2463.579522
LGR6	0	0	0	0	3.022818934	15.4030349
TMCC2	178.739519	263.075099	3.008802992	14.079803	37.28143352	29.90000891
DCLK1	44.9290595	67.0384422	218.6396841	393.228784	149.1257341	90.60608762
CCNA1	73.2539013	183.847849	0	0	8.06085049	0
COG6	506.916997	532.244601	1490.360415	1622.19445	624.715913	1359.091314
SPG20	1597.91177	1879.10785	33.09683291	8.04560172	313.3655628	83.35760061
RXFP2	0	1.01573397	0	0	0	0
EPSTI1	82.0443694	55.8653685	1.002934331	0	9.068456802	1.812121752
TRPC4	1.95343737	3.04720192	0	0	0	0
POSTN	0	0	0	2.01140043	0	0
RFXAP	140.64749	147.281426	426.2470906	252.430754	129.9812142	244.6364366
TPT1	17023.2299	16755.5476	98543.29261	62759.7062	33827.34901	32548.41579
GPALPP1	770.631042	663.274284	1139.3334	842.776781	658.9745276	1006.633633
STOML3	0	0	0	0	0	0
KL	0	0	0	0	0	0
RFC3	1048.01915	911.113373	1286.764746	1052.96813	2132.094955	2104.779415
STARD13	1103.69211	716.09245	359.0504904	429.433992	384.9056109	499.2395428
IRS4	0	0	530.5522609	643.497283	1.007606311	389.6061768

MORC4	1891.90409	1789.72326	2448.162701	2099.90205	842.3588762	1615.506542
BEX2	46.8824968	68.0541761	0	0	90.68456802	15.4030349
RNF128	0	0	1919.616309	1648.34265	1.007606311	1066.433651
GNG5P2	0	0	0	0	1.007606311	2.727243237
TBC1D8B	60.5565584	107.667801	219.6426184	219.242647	172.3006792	136.8151923
TCEAL4	1348.8485	1511.41215	3393.929775	2671.13977	3077.229675	1836.585396
BEX1	0	0	0	0	0	0
FAM104A	1424.05584	1211.77063	518.517049	451.559397	1307.872992	677.7335354
SLC39A11	191.436862	292.531384	365.0680964	493.798806	719.4309063	549.072891
EPHB2	1782.5116	1929.89455	24.07042394	24.1368052	332.5100827	180.3061144
SRRM1	3838.35792	3119.15651	3504.904459	2866.98983	3412.571131	4222.751077
BTBD2	1892.88081	1708.46454	2446.718476	1953.78387	2533.122267	2593.499591
PRAM1	1.95343737	3.04720192	12.03521197	7.03990151	7.053244179	6.342426133
SUV420H2	224.645297	211.272666	514.5053117	613.477131	458.4608716	644.209283
ZNF414	230.505609	237.68175	295.8656276	307.744266	197.490837	253.6970453
PDE6B	0	1.01573397	0	0	23.17494516	4.530304381
HSPBP1	1157.41164	1136.60631	1070.130931	827.691277	1016.674768	962.2366505
CSNK1G2	3004.38667	2868.43274	2544.444397	2521.29044	1862.056463	2999.967561
SLF1	637.797301	701.872175	372.0886367	405.297187	582.3964479	494.7092384
CNDP2	3715.43787	4187.87117	1483.339875	2299.03069	3348.275772	1969.776345
MACROD1	331.107634	252.917759	1294.788221	1000.67171	630.7615509	837.2002496
WDR74	1004.06681	939.553924	793.5015838	550.118018	1231.325141	898.9211165
LGALS12	21.487811	3.04720192	5.014671654	0	0	3.624243505
RTN3	5748.69269	6102.15388	12175.92366	6647.36666	3540.587513	7615.459785
RARRES3	454.174188	246.823355	21.06162094	19.1083041	15.11409467	170.3394447
HRASLS2	2.93015605	5.07866986	32.09389858	21.1197045	2.015212623	32.61819154
MYH11	0.97671868	0	5.014671654	0	4.030425245	1.812121752
FOPNL	1492.42615	1277.79334	1222.576949	1437.14561	1500.325798	1427.951941
MED10	733.515732	653.116944	503.473034	366.074878	760.742765	402.291029
PDZD2	628.030114	526.150198	808.3650706	1082.13343	14.10648836	955.8942244
MORC2	1491.44943	1463.67265	2671.817057	2268.85969	2366.867225	2525.191662
LARGE	6.83703079	5.07866986	1274.729534	1482.40212	1486.219309	2018.703632
GSTT2B	230.603281	243.776153	206.805059	163.738052	18.1369136	158.6784412

MYO18B	0	1.01573397	4.011737323	0	0	0
SLC2A11	76.1840573	117.825141	1050.242743	943.346802	363.7458784	1021.130607
C1QTNF6	217.808267	303.704458	327.9595261	407.308587	1908.406354	666.8608049
GGT2	0	0	1.193491854	3.88200283	0	1.522182272
FAM83F	0	1.01573397	259.7599917	274.556159	349.63939	342.4910112
SEC14L4	30.2782792	28.552282	0	0	26.19776409	2.718182629
ZDHHC8P1	0	2.03146794	368.0768994	1628.22865	33.25100827	256.415228
GIMAP6	0	0	0	0	0	2.718182629
GIMAP4	0	0	0	0	0	0
ADCK2	952.300717	898.924565	693.0276225	936.306901	1334.070756	896.0942065
MKRN1	2113.61923	2197.03258	3114.111097	3012.07215	3253.560779	2787.043255
AGAP3	2297.24234	2045.68822	2083.094605	1986.25793	2728.597891	2246.124912
KRBA1	304.736229	289.484182	452.3233831	571.237722	227.7190264	490.178934
ZNF767P	243.310391	218.951615	609.5634275	453.952963	340.4500205	524.9988535
ACTR3B	223.570907	173.1928	135.2858119	182.7659	263.9223211	231.7431903
NTS	0	0	0	0	0	0
BTG1	1381.08022	1750.10963	2568.514821	3084.48256	936.0662632	2142.833972
LRRIQ1	28.3248418	44.6922948	5.014671654	0	1.007606311	0
C12orf29	910.301813	979.167549	499.4612967	481.730403	481.6358168	603.4365435
ATP13A3	8352.89818	7642.38241	3524.311238	4745.89932	3120.556746	4756.8196
SFTPD	0	0	0	0	9.068456802	0
DYDC2	0	0	0	0	0	0
TMEM254	251.99342	332.145009	785.2975809	804.560172	329.4368835	604.3426044
TMTC1	6.83703079	9.14160575	2396.010116	2882.33682	92.69978064	2511.600749
KRAS	1165.22539	1119.33884	1808.290598	1488.43632	1670.611264	1585.606533
IPO8	2122.4097	1976.61831	1256.676716	1916.86461	1801.600085	1845.646005
LARS	5656.1779	5290.95826	4382.823025	2314.1162	3136.678447	3323.431294
SPINK5	0	0	0	0	41.31185876	9.966669638
IMPA1	1102.71539	1241.22691	302.8861679	435.468193	325.4568385	290.8455413
LRRCC1	159.205145	218.382804	1308.829302	1761.98678	213.612538	1244.021583
E2F5	284.225137	337.223679	167.4900332	151.860733	260.9700346	288.1273586
CA1	0	0	0	0	0	0.906060876
CCDC59	886.860565	946.664062	1284.758878	849.816682	1022.720406	897.0002674

SWAP70	4527.0911	4078.1719	1155.380349	1857.5283	768.8036155	1422.515576
ARNTL	243.202952	260.027897	333.9771321	533.021114	227.7190264	360.6122287
LYVE1	0	0	0	0	0	0
AMPD3	787.235259	1055.3476	5.014671654	0	98.74541851	20.83940015
MICALCL	57.6264023	51.8024326	1.002934331	3.01710065	37.28143352	11.77879139
SBF2	2197.61704	1670.51672	723.0554764	698.96165	734.5450009	782.836597
MICAL2	19685.7651	18529.0191	15.04401496	44.2508095	2633.882898	527.3274299
RRAS2	2660.58169	2061.87902	2.005868661	1.00570022	789.953272	93.32427025
HSD17B4	1621.35302	2052.79836	4341.702718	4011.73816	2835.40416	3327.961598
ZFC3H1	1891.90409	2046.70395	1841.387431	1790.14638	1652.474351	1666.245951
TEX15	0	0	0	0	9.068456802	1.812121752
SARAF	1550.05255	1831.36835	2305.746026	2608.78636	2162.323144	2588.615923
RNF122	41.0221847	34.5349551	28.08216126	31.1767067	30.22818934	27.18182629
DUSP26	0	0	0	0	0	0
DPF2	1778.60472	1663.77225	1160.395021	1336.57559	1562.797389	1705.206569
MEN1	2538.49186	2677.47475	1611.715469	1679.51936	1489.242128	2067.630919
C14orf1	429.756221	953.7742	1043.051704	7524.64901	1042.872532	1116.266999
GSC	0	0	0	0	153.1561593	5.436365257
C14orf159	533.288401	512.945656	671.9660016	345.960874	59.44877237	743.8759793
UNC79	5.8603121	3.04720192	0	15.0855032	4.030425245	5.436365257
NUMB	2362.6825	2431.66713	2522.379842	2525.31324	1754.242588	2968.25543
CATSPERB	0	0	223.6944731	155.984103	7.053244179	306.3663641
VRTN	0	0	0	0	0	0.906060876
COX16	849.842927	845.050036	1489.899066	1089.51527	1006.518096	965.0454392
TTC9	182.646394	141.187022	4150.14226	4879.65745	632.7767635	3860.725393
MED6	800.909321	758.753277	776.271172	578.277624	867.549034	663.7620767
EIF2S1	5303.58245	5149.77124	3047.917431	2615.82626	4739.780088	3950.42542
ADAM20	0.97671868	1.01573397	1.002934331	5.02850108	1.007606311	3.624243505
LOXL2	12815.5259	10156.324	6.017605984	3.01710065	2091.790702	236.4818887
ELP3	404.361535	432.702672	571.6725685	372.10908	904.8304675	461.184986
PEBP4	0	0	0	0	1.007606311	0.906060876
ADAMDEC1	0	0	0	0	0	0
CTIF	1335.17444	1080.74095	980.8697754	983.574811	420.1718318	787.3669014

MRO	0	0	0	2.01140043	0	1.812121752
MBD2	1669.21223	1561.18312	1736.079326	1892.72781	1509.394254	2078.50365
IER3IP1	1159.63856	1326.89392	1906.738632	1403.49488	674.4110563	1297.55166
MRPS36	605.54605	684.604697	423.2382876	516.929911	442.3391707	352.4576808
CCNB1	6476.62159	7142.64129	1749.117473	3143.81887	3897.421212	3434.876782
CDK7	1075.36727	1147.77939	698.0422942	660.745042	785.9329228	665.954744
CD180	0	0	0	0	0	0
IRAK2	745.236356	987.293421	0	1.00570022	50.38031556	18.12121752
CAMK1	860.48916	850.169335	55.16138819	51.290711	167.2626477	102.384879
THUMPD3	1222.85179	1213.8021	1190.483051	1152.53245	2010.174591	1437.91861
VHL	1702.42067	1809.0222	2853.348171	1644.31985	3344.245347	2280.555225
BHLHE40	1371.31303	2415.41539	6073.770307	8127.06344	370.7991226	3771.931428
ARL8B	2437.88983	2794.28416	2963.670947	2125.04456	3110.480683	2089.37638
EDEM1	3999.66301	4722.14724	3990.675702	2323.1675	1812.683754	2914.797839
CNTN6	0	0	1.002934331	0	0	0.906060876
CHL1	0	0	1.002934331	0	0	0
MEIS2	222.69186	247.839089	203.5956691	340.932373	408.0805561	402.291029
DPH6	86.9279629	98.5261953	251.736517	242.373752	228.7266327	173.9636882
KATNBL1	708.121046	657.17988	714.0892435	616.494232	645.8756455	598.0001783
EMC7	730.585576	859.310941	1526.466051	1200.80606	1188.975447	1141.636704
TRPM1	0	0	1.002934331	0	0	0.906060876
GNAT2	1.95343737	3.04720192	1.002934331	1.00570022	1.007606311	4.530304381
GSTM1	550.605624	617.556098	0	2.28293949	23.1648691	17.42355065
PRPF38B	2463.28452	1981.69698	2073.065262	1679.51936	1976.923583	2236.158242
REG4	0	0	3.008802992	1.00570022	19.14451991	2.718182629
TSPAN2	143.577647	73.132846	0	0	7.053244179	0
TSHB	0	0	0	0	0	0
GSTM5	0	0	1.484342809	6.0744293	0	2.129243059
GSTM3	975.741965	1110.19723	0	0	1765.326257	206.5818798
SYT6	0	0	0	0	0	0
VAV3	0	0	3086.028936	3597.38967	442.3391707	5039.510593
CHIA	0	0	0	0	0	0
PSRC1	1222.85179	1172.157	381.1150457	833.725479	872.5870656	630.6183698

HMGCS2	0	0	188.5516542	41.2337088	0	15.4030349
PTPN22	115.252805	206.193996	1.002934331	1.00570022	9.068456802	1.812121752
SORT1	6273.46411	6032.44406	3999.702111	5065.71199	4984.628422	5334.886439
WNT2B	68.3703079	69.0699101	9.026408976	20.1140043	37.28143352	19.0272784
PTGFRN	705.19089	657.17988	867.5381961	1297.35328	3828.903983	1701.582325
LAMTOR5	1218.94492	1253.41572	1397.087523	1272.21077	1186.960235	938.6790677
ADAM30	0	0	0	0	0	0.906060876
NOTCH2	13939.6607	12358.5571	4468.443529	7705.92648	7336.774519	8673.403646
TRIM45	124.043273	159.470234	239.701305	112.638424	392.9664614	167.6212621
CEPT1	964.99806	980.183283	718.1009808	703.990151	638.8224014	823.6093364
CD101	4.88359342	1.01573397	17.04988362	24.1368052	12.09127574	25.36970453
VTCN1	0	0	171.5017706	47.2679101	37.28143352	270.912202
NGF	144.554365	73.132846	0	1.00570022	0	0
AP4B1	516.684184	549.512079	302.8861679	362.052078	1573.881058	444.8758902
NAPG	1120.29633	1011.67104	1485.345744	1156.55525	1267.56874	1176.973078
SPIRE1	2639.09388	2465.18635	4420.93453	2869.26271	1741.143706	1917.224814
PPHLN1	1196.48039	1248.64177	1462.278254	1147.50395	1371.35219	1461.476193
FKBP11	306.689667	296.59432	436.2764339	264.499157	416.1414066	352.4576808
ARF3	4342.49127	4530.17352	5438.311115	6244.39264	10188.91502	7331.735883
TMEM106C	1648.70114	1742.9995	2911.518362	4207.8497	3731.166171	3853.476906
SLC38A2	8518.94036	7379.30731	6944.317306	12180.0353	5947.900056	6714.817153
PLEKHA8P1	58.8570679	65.5249985	81.62882518	79.168721	65.82692032	70.50965738
YWHAQ	14625.3856	13855.6271	6425.800257	9568.23185	11866.57953	10269.29397
KIDINS220	2542.39873	2653.09714	1920.619243	2487.09663	2786.031451	2265.15219
GRHL1	48.8359342	46.7237627	1321.867448	1318.47298	1119.450612	1939.876336
ROCK2	5686.45618	4698.78536	103.3022361	180.020339	3044.986273	548.1668301
RSAD2	5.8603121	7.11013781	2.005868661	14.079803	2.015212623	82.45153973
MYCN	0	0	24.07042394	50.2850108	11.08366942	17.21515665
LPIN1	1988.59924	3365.12665	888.599817	8521.29793	603.5561805	913.3093632
CMPK2	0	0	0	12.0684026	11.08366942	65.23638308
IAH1	932.766343	800.39837	978.8639068	1062.01943	1092.245241	719.4123357
LDHA	53569.9822	47921.8108	21528.61726	18496.6875	26572.15029	24735.3985
SAA2	4885.19524	2882.09436	0	0	43.93163517	21.6095519

ANO3	0	0	0	0	0	0.906060876
IL6ST	16612.7834	14101.9731	115.4176828	188.297251	2019.243048	817.2669103
FST	862.442598	452.001618	1.002934331	0	9.068456802	7.248487009
CFHR4	0	0	0	0	0	0
NAV1	2154.64142	1879.10785	518.517049	498.827307	223.6886011	737.5335532
CDC73	1608.65567	1541.88417	3209.389858	3453.57454	2086.752671	3951.331481
TIMM17A	1587.16786	1649.55197	3012.814729	2895.41092	1154.716833	3226.48278
CRB1	2.93015605	1.01573397	6.017605984	0	0	0.906060876
CFHR5	0	0	0	0	1.007606311	0
ERN2	0	0	102.2993017	161.917735	1.007606311	176.6818709
RPS15A	6855.85216	6546.16167	15194.45511	11073.7047	14551.1954	9142.462301
RAX	0	0	0	0	0	0
NARS	4539.78844	4685.58081	8815.792767	4185.7243	3668.694579	4956.152993
GRP	0	0	0	0	0	0
KIAA1468	678.819485	714.060982	1283.755943	1236.00556	826.2371753	1599.197446
FBXO18	1988.59924	2148.27735	1499.386824	1422.0601	2539.167904	1885.512683
RBM17	2894.99418	2673.41181	1696.964888	1485.41922	3618.314264	1848.364187
IL2RA	0	0	0	0	0	0
ANKRD16	294.021625	340.971737	324.8905471	178.27042	339.5633269	280.8788716
ECHDC3	768.677604	622.644925	231.6778304	162.923435	213.612538	103.2909399
IL15RA	560.636525	547.480611	3.008802992	2.01140043	79.60089859	17.21515665
CCNH	1278.52476	1005.57663	712.0833748	589.340326	588.4420858	756.5608316
HRH4	0	0	1.002934331	3.01710065	0	0.906060876
TMEM241	382.873724	358.554092	647.8955776	868.924986	453.4228401	743.8759793
KCTD1	219.761704	215.335602	491.437822	575.260523	325.4568385	733.9093097
CABLES1	1633.07364	1625.17436	1331.896791	1330.54139	733.5373946	1262.142801
DOCK2	624.123239	553.575015	23.06748961	21.1197045	15.11409467	67.04850484
EMP1	3732.04209	5354.9495	39.1144389	9.05130194	267.0156725	123.2242792
SOX5	1.95343737	0	4.011737323	0	0	0
RERG	0	0	159.4665586	98.5586211	141.0648836	206.5818798
SLCO1B1	4.88359342	2.03146794	0	0	1.007606311	0
KLRD1	0	0	0	0	0	0
KLRC1	1.00602024	0	1.002934331	0	0	0

SPX	4.88359342	1.01573397	26.0762926	0	0	4.530304381
PRH2	0	0	4.553321861	0	0	3.986667855
LRP4	330.130915	377.853038	2.005868661	1.00570022	105.7986627	26.27576541
MYBPC3	0	0	0	0	0	0
DDB2	403.384816	506.851252	344.0064754	674.824845	2002.113741	505.5819689
ACP2	948.393842	1089.88255	1421.157947	1386.8606	822.20675	1649.030795
USP26	0	0	0	0	0	0
FAM127A	1451.19885	1502.0674	2006.871596	2312.1048	1589.720629	1456.945889
RAB33A	0	1.01573397	0	1.00570022	1.007606311	3.624243505
SOX3	0	0	0	0	141.0648836	22.6515219
RBMX2	520.591058	484.505105	803.3503989	676.836245	974.355303	592.563813
STK26	520.591058	631.786531	883.5851454	1203.82316	1898.33029	1075.49426
FOLH1B	0	0	0	0	0	0
PIWIL4	25.5314264	18.7402918	0	0	14.00572773	1.812121752
MTNR1B	0	0	0	1.00570022	0	0
PUM1	2826.62387	2593.16883	3090.040673	2924.57623	2169.376388	3807.267802
SPOCD1	337.944665	299.641522	90.26408976	74.421816	74.56286704	591.6577521
YARS	3314.98321	2665.28594	9087.587971	2985.92394	3131.640415	5000.549976
PHC2	3963.52442	3781.57758	1430.184356	1478.37932	979.3933346	1731.482334
CDCA8	2033.5283	1958.3351	400.170798	843.782481	1543.652869	1128.951852
GNL2	1651.63129	1455.54678	1804.278861	1164.60085	1519.470317	1658.997464
AGO4	499.103247	536.307537	575.6843058	463.627799	410.0957687	410.4455769
HOOK1	10.7439055	5.07866986	939.7494679	1181.69775	1426.770537	1325.567062
CYP2J2	6.83703079	15.2360096	21.06162094	15.0855032	130.9888205	105.1030616
BTF3L4	1496.98743	1512.87481	2200.257393	1555.70761	1969.144862	2154.168794
ZCCHC11	1820.60363	1675.96105	1123.28645	934.2955	320.418807	1224.994305
PRPF38A	1244.3396	1334.67444	1320.864514	1382.8378	1459.013939	1562.048951
DSC2	400.45466	277.295374	654.916118	999.666014	359.7154531	847.1669192
DSG3	0	0	0	0	0	0
RNF138	1467.03146	1500.23908	594.7400581	622.528433	727.4917568	885.221476
ELP2	1518.79755	1444.37371	1226.588686	938.318301	2535.137479	1391.709506
DSG1	0	0	0	0	0	0
DSC3	0	1.01573397	4.011737323	1.00570022	0	0

DSC1	0	0	0	2.01140043	2.015212623	0.906060876
DTNA	4.88359342	0	511.4965087	915.187196	1197.036298	608.8729088
FHOD3	1632.09692	1368.19366	0	0	80.6085049	11.77879139
TPGS2	2483.79561	2671.38035	1677.909135	2244.72288	1690.76339	2005.112719
DAGLA	520.591058	440.828544	108.3169077	92.5244198	502.7955493	231.0455234
SLC43A3	10764.0845	12674.1254	1767.79211	1496.41152	403.0425245	3973.892397
TIMM10	573.333867	679.526027	961.8140231	927.255599	674.0886223	858.9457106
GIF	0	0	0	0	0	0
DHX34	1135.92383	1348.89472	993.9079217	874.959188	989.4693977	1108.112452
APLNR	0	0	3.008802992	0	0	0
FADS2	4691.17984	7665.75445	302.8861679	1302.38178	476.5977852	367.8607157
TMEM258	770.631042	845.090665	801.3445302	676.836245	699.27878	523.7031864
TCN1	2.93015605	6.09440383	0	0	0	20.83940015
C5AR2	0	1.01573397	0	2.01140043	553.1758649	43.49092206
TMEM165	2436.91312	2365.64442	1767.170291	1499.49902	1758.273013	1842.927822
CLOCK	2181.01282	2140.15148	2141.264796	2268.85969	2935.157185	2480.794679
PDGFRA	0	5.07866986	0	0	0	0.906060876
GGACT	15.6274989	10.1573397	95.27876142	32.1824069	55.41834712	48.02122644
COL4A2	11588.7672	9817.06884	20.05868661	9.05130194	463.4989032	198.4273319
CLDN10	0	0	0	0	0	0
DZIP1	0	0	0	0	1.007606311	1.812121752
UBAC2	1330.29085	1461.64119	991.9020531	1059.00233	1109.374549	919.6517893
ARGLU1	2116.54939	1951.22496	1434.196093	1009.72302	1556.751751	1213.215513
BIVM	1009.86852	967.354563	643.4525786	398.659565	401.1683768	391.1827227
ERCC5	678.311592	889.559498	701.2015373	814.647346	747.9058606	618.9573663
TPP2	2524.8178	2268.13396	742.1714047	765.337864	1406.618411	794.6153884
KDELC1	181.669675	235.650282	67.19660016	59.3363127	246.8635463	64.33032221
CARS2	1884.09034	2209.22139	839.4560348	487.764605	813.1382932	642.3971612
ARHGAP32	2313.84656	2045.68822	3766.018412	4633.26089	1698.824241	4852.862053
STT3A	7616.4523	8417.38743	6795.883025	3977.54435	3220.309771	4550.23772
ADAMTS8	4.88359342	0	0	0	0	0
ACRV1	0	1.01573397	0	3.01710065	6.045637868	1.812121752
ETS1	9390.17343	7031.92629	0	0	444.3543833	189.3667231

SLC37A2	3005.36339	4871.46013	31.09096425	22.1254047	21.15973254	61.61213958
KLB	0	0	12.03521197	17.0969037	30.22818934	19.0272784
TMED7	5046.54916	5157.29783	3061.005724	2344.51851	2532.830061	2469.477979
APC	1712.18785	1998.96446	1017.978346	2216.56328	1749.204556	2061.288493
NREP	241.249515	292.531384	622.8222194	513.91281	195.4756244	827.23358
WDR36	2638.11717	2572.85415	771.2565003	722.092755	1336.085969	1299.291296
OSTF1	406.314972	461.143223	764.23596	798.525971	786.9405291	499.2395428
RFK	1015.78743	1114.26017	232.6807647	287.630262	925.9902001	263.663715
UBQLN1	4988.10232	4854.19265	3661.713241	2897.42232	6634.079954	2725.431116
NAA35	852.675411	952.758466	504.4759683	508.884309	1119.450612	511.924395
C9orf40	1112.48258	1208.72343	175.5135079	273.550459	713.3852684	215.6424885
ANXA1	8343.131	6747.52078	0	2.01140043	326.4644449	137.7212532
CTSL	1237.50257	1498.20761	457.3380548	919.209997	1912.436779	380.545568
TMEM2	1524.65787	1353.97338	44.12911055	113.644124	1420.724899	253.6970453
AGTPBP1	1768.83754	1527.66389	376.100374	445.525195	492.7194862	351.55162
GOLM1	4379.60658	4149.27328	0	2.01140043	361.7306658	192.0849057
FAM189A2	42.9756221	43.6765608	0	1.00570022	169.2778603	9.060608762
PSAT1	3206.56744	2861.3226	3.008802992	2.01140043	181.369136	89.70002674
ISCA1	650.279765	704.614657	520.5229176	544.083817	1026.49893	460.2155008
ADAM19	2291.38203	3198.54628	0	0	202.5288686	84.26366148
HAVCR2	2.93015605	0	3.008802992	3.01710065	2.015212623	5.436365257
CCNJL	1139.8307	1315.37549	1.002934331	0	153.1561593	62.51820046
TAOK3	3126.47651	3123.38196	1754.132144	1806.23759	2165.345963	1599.197446
USP30	212.924673	181.816381	685.0041479	606.43723	437.3011391	598.9062392
SDS	0	0	0	1.00570022	1.007606311	0
MSI1	5.8603121	4.06293589	0	0	526.9781008	52.55153082
HNF1A	0.97671868	2.03146794	0	0	2.015212623	0.906060876
FBXO21	1673.11911	1659.70931	1316.852776	1261.14807	2429.338817	1492.282263
TBX3	0.97671868	1.01573397	4359.755536	4127.39368	222.6809948	3269.067641
OASL	192.413581	277.295374	21.06162094	121.689726	45.34228401	669.5789875
HRK	0.97671868	0	13.0381463	0	13.09888205	5.436365257
RNFT2	147.484521	244.791887	210.6162094	309.755666	158.1941909	243.7303757
P2RX4	523.521215	446.922948	665.9483956	785.451868	705.3244179	578.9728999

CCDC64	4.88359342	3.04720192	578.6931088	1063.02513	1004.583492	1350.030706
DTX1	0	0	1.002934331	0	3.022818934	0
TRAFD1	671.005736	765.863415	816.3885452	864.902185	2220.76431	1051.030616
DMTF1	1354.70881	1184.34581	1496.378021	1301.37608	1377.397828	1279.357957
OCM2	0	0	0	0	0	0
TMEM243	420.965753	468.253361	357.0446217	378.143281	457.4532653	299.90615
CCDC146	161.158583	103.604865	143.4196093	122.695426	15.11409467	200.855575
TMEM60	194.367018	296.59432	330.9683291	401.274386	329.4872638	163.9970186
CD36	1.95343737	2.03146794	6.017605984	1.00570022	4.030425245	35.33637417
UGT2A3	0	0	0	0	0	0
CSN2	0	0	0	0	0	0
UGT2B28	0	0	91.28708278	7.88468969	0	111.092124
PNPLA8	680.421304	791.34818	466.7957255	571.036582	724.660383	440.3455858
HILPDA	255.763555	242.557273	1998.266419	1912.42947	948.5404293	1133.636186
FAM71F1	0.97671868	0	4.011737323	0	0	9.060608762
RINT1	622.169802	598.26731	337.9888694	369.091979	1509.394254	569.0062302
SRPK2	1197.45711	1217.86503	856.5059184	913.175796	2902.913783	1166.100348
KCP	1.95343737	31.4877531	5.014671654	8.04560172	72.54765441	32.61819154
TES	1794.23222	1428.12196	2045.986035	1307.41028	736.5602136	956.8002852
MDFIC	851.698692	830.870389	200.5868661	362.052078	58.44116605	201.1455145
MTO1	1120.29633	1101.05563	1037.034098	895.073192	1022.720406	1113.548817
ADGRB3	4.88359342	3.04720192	0	1.00570022	2.015212623	0
ANKRD6	283.248418	237.68175	10.02934331	0	120.9127574	14.49697402
HTR1B	2.93015605	0	0	0	0	0.906060876
KHDC1	448.313876	562.716621	176.5164422	237.345251	161.2170098	212.018245
CEP162	163.11202	216.351336	152.4460183	256.453555	125.9507889	180.3061144
SYNCRIP	8462.53486	7981.70866	5333.494448	5338.36737	11072.79735	8637.559878
SNX14	1782.98042	1917.93936	2762.733054	3094.40882	1881.120375	3001.254167
NT5E	16936.302	16968.8517	0	0	1064.032265	434.0031597
MRAP2	1.95343737	4.06293589	0	0	2.015212623	0.906060876
EPHA7	0	0	39.1144389	58.3306125	77.58568597	81.54547886
AKIRIN2	724.725263	667.33722	565.6549625	610.460031	905.8380738	802.7699363
ORC3	864.396035	820.71305	786.3005153	670.802044	925.9902001	844.4487366

LCA5	42.9756221	83.2901857	56.16432252	111.632724	37.28143352	49.83334819
MAP3K7	1002.11337	982.214751	1008.951937	827.691277	1598.06361	1058.279103
CGA	0	0	0	1.00570022	13.09888205	17.21515665
GJA10	0	0	0	0	0	0
PRR5L	324.270603	249.870557	6.017605984	10.0570022	25.19015778	19.0272784
LMO2	40.045466	48.7552307	0	0	26.19776409	5.436365257
PHF21A	683.703079	750.627405	1579.621571	1270.19937	738.5754262	1298.385236
NAT10	2847.13496	2653.09714	1532.483657	1072.07643	2926.088728	2516.131053
EHF	2237.6625	2125.9312	5238.326009	3329.87341	57.43355974	10988.70631
ELF5	0	0	0	0	25.19015778	1.812121752
PRRG4	87.9046815	62.9755063	931.7259932	836.742579	400.0197056	945.9275547
CAPRIN1	11642.4867	10969.9269	7007.502169	7553.81432	9814.085472	9609.681653
ATP5G2	1846.97503	1924.81588	3745.959725	3124.71057	3714.036863	2423.712844
DNAJC14	1041.78768	1045.02774	1552.030847	1535.97577	1973.779851	1419.60712
CD63	4147.14753	5127.42509	10437.53758	9672.82467	9176.270677	7124.356669
PRPH	0.97671868	2.03146794	0	0	23.17494516	2.718182629
AVIL	23.4412484	16.2517436	110.3227764	178.008938	17.12930729	160.3727751
AMHR2	0	0	0	0	2.015212623	0.906060876
LACRT	0	0	0	0	0	0
GDF11	438.546689	694.762037	217.6367498	567.214922	1697.816635	704.9153617
GLS2	1.95343737	7.11013781	221.6484871	594.368827	85.64653646	503.7698472
ITGA7	39.0687474	26.4090833	39.1144389	48.2736103	33.25100827	63.42426133
TESPA1	0	0	0	0	0	2.718182629
FAM186B	2.93015605	4.06293589	13.0381463	11.0627024	20.15212623	19.0272784
RDH5	13.381046	25.8199576	38.10147522	11.5655525	19.27550873	18.43833883
AGAP2	7.81374947	10.1573397	169.4959019	238.350951	394.981674	368.7667766
BLOC1S1	335.434498	360.514459	449.866194	673.688403	651.064818	427.6426123
KRT85	0	0	0	0	0	0
CDK4	3542.55867	3849.63175	2475.241928	3700.97679	6466.817306	3253.664606
PPP1R1A	0	0	0	0	0	0.906060876
TROAP	1179.87617	1191.45595	523.5317206	1173.65215	1237.34055	1158.851861
TSPAN31	297.899199	302.688724	1086.17788	730.138356	693.2331422	647.8335265
B4GALNT1	182.646394	231.587346	0	0	1692.778603	38.96061768

TFCP2	504.96356	552.559281	773.262369	754.275162	21.15973254	723.0365792
COQ10A	311.57326	312.846063	371.0857024	267.516257	123.9355763	218.3606712
FAIM2	0	0	66.19366583	26.1482056	0	8.154547886
PAN2	469.801687	483.489371	1080.160274	1349.65975	865.5338214	668.6729266
ESPL1	2170.26892	1738.93656	484.4172817	1333.55849	2846.487829	1755.039917
KRT87P	0	3.23003403	0	0	0	0
KRT7	4672.62218	2715.05691	17364.805	9890.05592	597.5105426	5990.874513
ZC3H10	134.787178	119.237011	230.6748961	323.714785	244.8483336	242.8243148
HNRNPA1	28966.4093	25493.4702	17630.73304	21958.4585	30875.34464	20931.54654
SLC26A10	4.88359342	11.1730737	4.011737323	12.0684026	17.12930729	10.87273051
ACVR1B	481.522311	556.622217	2345.8634	1691.58776	1240.363369	2358.476461
OS9	2232.77891	2540.35066	4779.98502	6101.58321	5674.838745	5048.571202
MIP	0	1.01573397	0	8.04560172	5.038031556	6.342426133
KCNH3	6.83703079	11.1730737	4.011737323	3.01710065	33.25100827	8.154547886
LTV1	1000.15993	1106.11398	573.6784372	409.319988	1024.735619	842.6366148
MAP7	30.2782792	26.4090833	1877.493067	1902.78481	1838.881518	2009.643023
CD164	3429.2593	3621.09161	13773.29716	17797.8767	5688.945234	10629.9062
LACE1	230.505609	268.153769	110.3227764	145.826531	102.7758438	143.1576184
NHSL1	198.273893	233.618814	252.7394513	331.881071	723.4613315	459.3728642
AHI1	577.240742	528.181666	660.9337239	560.17502	436.2935328	593.4698739
HEY2	0	0	3.008802992	5.02850108	253.9167904	10.87273051
PKIB	15.6274989	13.2045416	494.2861555	564.097251	2004.018116	659.2227117
TAAR5	0	0	0	0	0	0
NMBR	0	1.01573397	0	0	0	0
SMPD2	184.599831	197.052391	384.1238487	413.342789	393.9740677	458.4668033
MICAL1	726.09267	810.261147	36.10563591	97.5529209	333.517689	280.8788716
REPS1	1186.7132	1201.61329	1040.042901	1108.28164	1356.238095	1252.176131
STX11	48.8359342	42.6608268	0	0	8.06085049	9.966669638
TEC	176.786082	203.146794	64.18779717	130.741028	86.65414277	76.1091136
PRADC1	202.180768	296.59432	474.3879384	891.050391	473.5749663	594.3759348
SEMA4F	653.4248	771.957819	179.5252452	163.929135	257.9472157	274.5364455
CCT7	11208.8236	10839.913	4706.770814	4690.58581	12536.63773	6813.577789
EGR4	0.97671868	0	0	1.00570022	3.022818934	3.624243505

RAB11FIP5	3977.19848	3905.49712	1132.312859	1007.71162	533.0237387	1210.497331
SMYD5	1440.66006	1423.0433	628.8398254	474.690502	1090.230029	806.3941798
DYSF	3772.08756	3239.17564	2.005868661	1.00570022	447.3772022	243.7303757
CCDC142	293.992324	323.765204	357.5260302	376.564332	451.4076275	435.8152814
EMX1	43.9523408	46.7237627	0	0	6.045637868	0.906060876
KCNMB4	44.9290595	33.5192211	52.1525852	65.370514	38.28903983	42.58486118
USP15	1321.50038	1346.86325	1067.122128	1173.65215	2203.635003	1403.488297
GNS	4605.22859	5437.22395	3126.146309	4713.71691	4843.563538	4131.637595
CPM	87.9046815	105.636333	0	0	44.3346777	21.74546103
MDM2	2237.80901	2228.34766	2105.760921	2945.6557	6854.997637	3767.609517
KLHL36	1350.80194	1165.04687	1349.949609	1257.12527	4308.524587	1589.230777
BCO1	0.97671868	3.04720192	0	0	39.29664614	1.812121752
MPHOSPH6	482.49903	526.150198	3020.838204	1903.79051	597.5105426	2446.364366
CHST5	0	2.03146794	0.842464838	5.42072416	2.408179084	6.342426133
KIAA0513	282.2717	306.75166	620.8163507	1024.80852	1427.778143	1066.433651
DYNC1LI2	2786.57841	2602.31044	5379.73975	4073.08587	4707.536686	6103.226062
FBXL8	165.045923	162.263502	496.2017601	589.169357	148.3498772	425.5133693
FHOD1	1129.0868	1191.45595	3227.442676	4279.25442	1105.344123	5490.72891
CCDC102A	412.175285	376.837304	12.03521197	31.1767067	204.5440812	80.63941798
SLC9A5	125.019992	97.5104613	612.7928761	794.664082	23.17494516	1002.103329
AGT	0	3.04720192	1.002934331	0	0	2.718182629
ZNF670-ZNF695	8.01886039	5.37323271	40.67901645	25.2732464	13.59260914	49.47998445
PCNXL2	614.512327	894.302976	1000.928462	1024.80852	446.3695959	1295.667053
KCNK1	15.6274989	32.5034871	1126.295253	861.885085	550.153046	1089.991234
URB2	1307.82632	1311.31256	578.6931088	669.796344	1217.188424	1687.991412
EGLN1	3534.74492	3327.54449	2466.215519	3186.05828	1474.128033	2595.86441
CAPN9	0	0	56.16432252	49.2793106	102.7758438	44.39698293
COG2	831.1876	884.70429	2174.361629	1669.46236	893.7467981	2025.952119
ABCB10	1345.91835	1182.31434	913.6731753	1099.23034	860.4957898	1101.770025
NTPCR	712.027921	654.132678	2265.628653	1905.80191	699.27878	2034.106667
TAF5L	1263.87398	1272.71467	1080.160274	1222.93146	993.4998229	1426.139819
GLUL	1413.31194	1211.77063	4083.948595	6982.5766	3136.678447	5111.089403
STX6	1389.01117	1288.57027	2408.787499	2054.62543	2432.512776	2996.714802

RGS8	0	0	0	0	8.06085049	0
RNASEL	21.487811	28.4405512	292.8568246	299.698664	452.4152338	327.9940372
DHX9	7524.64074	7993.82636	5334.607705	6772.38525	11954.24128	8303.141869
KIAA1614	1.95343737	2.03146794	7.020540315	14.079803	7.053244179	17.21515665
CEP350	2811.97309	2631.76672	2417.071737	3473.68854	4300.463737	4303.789162
NPL	1.95343737	2.03146794	77.22594346	24.1368052	54.41074081	48.02122644
FAM129A	3996.73285	3985.74011	786.3005153	22.1254047	170.2854666	129.5667053
PIGC	615.332771	615.534787	642.880906	557.157919	775.8568597	438.5334641
LAMC1	9822.8598	8278.23187	1269.714863	2587.66665	6013.394466	3115.943353
RC3H1	973.788528	878.609886	1199.50946	1521.62443	1768.349076	1909.070266
GPR55	10.7439055	6.09440383	0	0	0	0.906060876
SP110	279.820136	307.259527	370.1529734	395.551952	189.4299865	790.5381145
MRPL44	581.147617	574.905428	585.7136491	664.767842	780.8948912	718.5062748
CHRND	0	0	0	0	0	0
PAX3	0	0	0	0	0	0
DOCK10	185.57655	214.319868	0	0	129.9812142	14.49697402
TTLL4	847.791818	916.192043	556.6285535	719.075654	951.1803579	769.2456839
USP37	1025.55462	920.254979	1040.042901	1216.89726	1533.576806	1768.63083
HTR2B	0	1.01573397	0	0	1.007606311	0
ITM2C	1799.11582	1751.12537	152.4460183	129.735328	342.5861458	301.7182718
SLC19A3	5.8603121	15.2360096	0	0	2.015212623	2.718182629
SERPINE2	589.938085	683.588963	4.011737323	4.02280086	77.58568597	19.0272784
DNAJB2	527.428089	644.991072	1739.088129	1599.06334	1023.728012	1856.518735
WNT10A	39.0687474	54.8496345	2.005868661	0	7.053244179	2.718182629
TMBIM1	2492.58608	2922.26664	427.2500249	333.892472	122.92797	468.433473
CYP27A1	187.529987	274.248173	8.023474646	6.03420129	2.015212623	6.342426133
EIF4E2	1490.47271	1681.03972	844.4707065	916.192896	2176.429632	951.36392
ARMC9	473.708562	647.02254	29.08509559	50.2850108	589.4496921	101.4788181
CAB39	3333.54087	2971.02187	1794.249518	1658.39966	2236.886011	1594.667142
COX5B	1539.30865	1621.11142	2351.881006	1880.6594	2157.285112	2070.349102
REV1	1136.90055	1062.45773	1132.312859	1117.33294	1043.880138	1254.894314
TSGA10	69.3470266	96.4947274	185.5428512	191.083041	161.2170098	191.1788449
MFSD9	246.133108	269.169503	274.8040066	275.561859	669.0505907	370.5788984

TMEM127	2497.46967	2808.50443	2662.790648	3274.5599	3071.184037	3030.773631
EDAR	2.93015605	0	0	0	1.007606311	0
TGFBRAP1	2066.73674	1942.08335	1107.239501	1438.15131	1703.862272	1727.858091
GCC2	1712.51994	1532.43784	1538.451117	1289.54904	1079.388185	1746.45046
MRPS9	879.046815	894.86163	721.1097838	667.784943	910.8761054	931.4305807
GPR45	0	3.04720192	0	0	2.015212623	0
C2orf49	667.098861	741.4858	605.7723357	461.616399	617.6626688	615.2153349
ANKRD36	523.960738	400.351545	521.6261454	514.496116	458.3399589	433.7857051
EPC2	812.629945	857.279473	264.7746633	414.348489	776.864466	446.688012
ARHGEF4	972.811809	820.71305	0	0	502.7955493	48.92728731
ISCU	1372.28975	1351.94192	1945.692602	1198.79466	1262.530708	1486.845898
ALDH1L2	11.7206242	4.06293589	5.014671654	2.01140043	54.41074081	11.77879139
STAB2	0	0	0	0	0	0.906060876
USP44	58.603121	50.7866986	0	0	5.038031556	0.906060876
SCYL2	1856.73245	2102.56932	2183.388038	2093.86785	3776.508455	2954.664517
CKAP4	8243.50569	8175.64274	1097.210158	1095.20753	4799.228861	1602.82169
PLXNC1	96.6951497	110.715003	10.02934331	4.02280086	4.030425245	17.21515665
APPL2	1177.92273	1206.69196	788.3063839	850.822382	993.4998229	783.7426579
PWP1	2151.71126	1909.57987	1079.15734	1280.25637	1873.140133	1251.27007
DRAM1	866.349473	898.924565	539.5786699	1426.08291	1146.655982	342.4910112
KIAA1033	3289.58853	3428.10216	1310.83517	1617.16595	2135.117774	1512.215602
SLC41A2	692.493547	946.664062	95.27876142	217.231247	531.008526	231.9515843
VILL	1.95343737	3.04720192	76.22300913	104.592822	9.068456802	89.70002674
FLNB	42109.2726	32714.7598	8280.225834	7141.47723	12676.695	7391.644628
NEK3	54.6962463	57.8968364	307.9008395	295.675863	146.1029151	274.5364455
PCDH8	0	0	2.005868661	2.01140043	19.14451991	4.530304381
VPS36	832.164319	825.791719	1942.683799	1819.31169	936.0662632	1724.233847
RNASEH2B	613.379333	711.013781	349.0211471	484.747504	509.8487935	616.1213958
CKAP2	3422.42227	3390.52	2083.094605	3673.82289	2432.361635	3256.382789
LECT1	0	0	0	0	0	0
TBC1D4	2433.00624	1912.62707	960.8110888	809.588674	816.1611121	715.7880922
THSD1	55.51669	84.8442587	34.77173325	50.8783739	8.655338214	59.09329034
BORA	459.057781	491.615243	127.37266	179.014638	428.2326823	248.2606801

LRCH1	911.278532	762.816213	649.9014463	912.170096	904.8304675	1040.157886
SUCLA2	802.862758	696.793505	1555.551147	1481.39642	1174.868959	1743.261126
RCBTB1	718.864951	832.901857	1229.597489	1845.4599	518.9172503	1018.412425
MED4	712.027921	692.730569	1329.890923	1117.33294	884.6783413	1261.23674
PHF11	340.874821	304.720192	333.9771321	452.565097	348.6317837	330.7122198
RPL13AP25	61.3574677	47.7394967	85.39985826	77.8713677	65.04098739	46.5352866
COG3	677.842767	706.950845	2812.227863	1724.77587	1199.05151	1947.124823
LMO7	2125.55473	2057.39963	122.9597489	271.710027	444.9387949	231.634463
SCEL	0	1.01573397	0	0	0	2.718182629
ITM2B	2999.50308	2856.24393	9180.860863	7652.37294	3002.666808	9024.366327
SPRY2	797.002446	957.837136	38.11150457	32.1824069	51.38792188	48.02122644
NUDT15	1193.55023	1244.27412	386.1297173	516.929911	1169.830927	902.4366327
EDNRB	157.251708	179.784913	0	0	6.045637868	5.436365257
RCBTB2	88.8814002	99.5419293	44.12911055	76.4332164	29.22058303	64.33032221
LCP1	8697.67988	10406.1945	549.6080132	519.947011	1077.131147	810.9244842
SETDB2	185.57655	168.611839	492.4407564	760.309363	210.5897191	701.2911182
SCRN1	7125.1628	6281.29888	4011.737323	4956.09066	6442.634754	5500.695579
C7orf25	87.6116659	91.4668442	46.50606491	81.2605774	80.6085049	63.66889777
TNS3	4307.3294	3129.47637	961.8140231	1182.70345	2497.856046	906.0608762
SPDYE1	0	0	2.51736517	1.00570022	1.007606311	0
CHST12	827.280725	791.256764	629.8427597	619.511333	699.27878	614.309274
IGF2BP3	731.562294	937.522456	1.002934331	2.01140043	67.50962286	19.93333928
GPNMB	14.6507803	31.4877531	12.03521197	9.05130194	100.7606311	162.1848968
RAPGEF5	26.3714045	41.6450929	490.4348877	429.433992	66.50201655	470.2455947
RAC1	8203.59697	6960.99759	8160.134478	6722.65338	9619.39578	6557.950834
KDELR2	5053.62061	5871.95809	6324.503889	4292.32852	7319.363082	4790.343852
NUPL2	385.80388	437.781342	325.9536575	304.727165	354.6774216	337.9607068
IL6	1073.41383	1804.95927	0	0	16.12170098	2.718182629
ZDHHC4	854.628848	906.034703	851.4912468	893.061791	1031.788863	978.5457463
AOAH	0	1.01573397	0	0	1.007606311	0
BZW2	1557.8663	1473.82999	2641.729027	3988.60705	2293.311964	4249.425509
DGKB	0	0	0	0	0	0
TBRG4	2101.89861	1991.85432	1440.213699	1326.51858	2443.445305	2112.933963

DDX56	1803.02269	1842.54143	1763.158553	1284.27918	3187.058763	2285.08553
HUS1	321.340447	355.50689	370.082768	313.778467	358.7078468	327.0879763
NACAD	3.90687474	2.03146794	0	0	2.015212623	3.624243505
C7orf69	0.97671868	0	0	0	0	0
DBNL	2061.85314	2123.89974	3578.921012	2943.98624	2808.19879	3724.471959
CCM2	1030.43821	970.025943	879.573408	1059.00233	1142.625557	1114.454878
MYO1G	1.95343737	0	7.020540315	2.01140043	1.007606311	9.966669638
TTYH3	3782.83146	4596.19622	3225.436808	3204.16089	3087.305738	4042.84363
MMD2	0	0	0	0	0	0.906060876
CIDEB	3.90687474	5.07866986	6.017605984	3.01710065	6.045637868	5.436365257
RP11-84C10.2	0	0	0	0	0	0
TTC5	376.007392	432.702672	456.3351205	362.41413	608.594212	359.7061678
NKX2-8	0.97671868	0	0	0	10.07606311	2.718182629
NKX2-1	0	2.03146794	0	0	4.030425245	0
ZFHX2	110.369211	131.029682	426.2470906	392.223084	129.9812142	453.0304381
MTHFS	214.80974	247.016345	196.9662732	239.406936	476.2048188	184.8454793
ADAMTS7	157.251708	178.769179	0	0	94.43286349	39.86667855
ABHD17C	1198.43383	984.246219	233.6836991	279.58466	919.9445622	368.7667766
IREB2	2291.38203	2149.29309	1032.019426	1081.12773	4359.912509	1552.082281
ALPK3	9.76718684	13.2045416	5.014671654	13.0741028	116.8823321	57.0818352
TM6SF1	0.97671868	0	0	0	2.015212623	0
CIB2	6.83703079	7.11013781	1.002934331	0	6.045637868	0
CALCOCO2	2134.13032	2173.6707	3891.385203	2975.86694	1547.683294	3497.394982
RSAD1	940.580093	1164.03113	1729.058786	1161.58375	870.571853	1189.65793
NMT1	4654.06453	4630.73118	2646.743699	2452.90283	3262.629236	3064.297883
MYCBPAP	1.95343737	8.12587178	15.04401496	8.04560172	1.007606311	7.248487009
SRSF1	9777.93074	8808.37391	4589.437527	4726.82118	10605.94312	7286.759021
VEZF1	3511.30367	2939.53412	795.3269243	834.731179	2790.061876	1172.442774
CHAD	0	1.01573397	2.005868661	0	1.007606311	0.906060876
TACO1	774.537916	783.130893	609.7840731	654.71084	2601.639496	945.9275547
TEX2	1884.09034	1925.83161	5656.549625	7005.7077	3291.849819	11292.2367
DCAF7	6705.17376	6836.90537	3416.997265	3855.85463	9665.967344	4464.161937
GH2	0	0	0	0	0	0

CSH1	0	0	0	0	0	0
LIMD2	580.170898	559.669419	47.13791354	30.1710065	118.8975447	67.95456571
BRIP1	1879.20675	1400.69715	385.126783	665.773543	10666.52041	1464.194376
KAT7	1834.27769	1700.33867	4058.81506	4269.13707	2265.098988	4102.598344
RTP4	4.88359342	4.06293589	0	0	0	0
ACTL6A	2483.79561	2378.84896	1742.096932	1833.39149	2332.608611	2163.673372
NDUFB5	1147.64445	1249.35279	1931.651521	2123.03316	1346.162032	1966.152101
MRPL47	1158.38836	1230.05384	1269.714863	973.517809	868.5566403	1272.10947
TRA2B	5605.38853	5070.54399	5047.768486	4110.29678	5681.891989	5339.416743
SCN2A	0.97671868	6.09440383	0	1.00570022	0	0
TBR1	1.95343737	5.07866986	3.008802992	2.01140043	3.022818934	2.718182629
MARCH7	2770.95091	2717.08838	3082.017198	2612.80916	2375.935682	3175.743371
ERMN	0	0	0	0	0	0.906060876
GALNT5	427.802784	584.047034	0	0	7.053244179	3.624243505
SCN7A	0	0	0	0	0	0.906060876
TANK	808.72307	850.169335	906.652635	817.634275	585.4192669	679.5456571
BLK	1.95343737	0	0	0	0	0
GATA4	0	0	98.28756441	127.723927	32.24340196	147.6879228
SKIL	1138.85399	1133.55911	1280.74714	1313.44448	3695.89995	2147.364277
EPRS	6146.49068	6026.34966	18765.90426	9943.35803	7404.898782	11969.06417
HLX	118.182961	111.730737	18.05281795	31.1767067	6.045637868	52.55153082
VPS45	550.869338	592.172906	1528.47192	1394.9062	1636.35265	1735.106578
IL10	0	0	0	0	0	0
KCTD3	2079.43408	2078.19171	9692.357372	8924.58371	3970.976473	7706.047752
RPS6KC1	799.932602	1000.49796	1212.547606	1386.8606	459.4684779	1581.076229
CBWD2	562.648565	465.581981	743.6657769	932.394727	987.3735766	842.7090997
IL36G	0	1.01573397	0	2.01140043	0	0
IL1RN	0	0	2.005868661	4.02280086	15.11409467	16.30909577
IL36A	0.97671868	0	0	0	0	0
IL36RN	0	2.03146794	0	0	1.007606311	0
IL36B	0	0	0	0	0	0
IL1F10	0	0	0	0	0	0
CFC1	0	0	0	0	1.007606311	0

SMPD4	4664.01729	4203.43221	2565.084786	2632.52088	5476.904561	3753.194089
WDR33	2450.58718	2458.07621	1600.683192	1917.87031	2682.248001	2487.137105
CCDC115	400.45466	457.080288	727.1273898	784.446168	740.5906388	689.5123268
SAP130	1906.55487	1966.46097	1675.903267	1875.6309	2124.034104	1896.385414
BIN1	1527.58802	1633.30023	1.002934331	7.03990151	196.4832307	64.33032221
IMP4	1758.09363	1750.10963	1281.750075	1026.81992	3500.424325	1921.755118
HS6ST1	1076.99839	978.009613	1631.874449	1669.49253	3369.788167	2060.753917
UGGT1	6016.58709	5690.14171	4508.189817	3006.00777	5494.477215	4179.658822
GYPC	0	0	0	0	0	0
STAM	1410.38178	1625.17436	540.5816043	581.294725	949.1651452	620.6517002
GAD2	0	0	0	0	0	0
ABI1	1693.6302	1641.4261	990.8991187	1183.70915	1415.686867	1012.97606
YME1L1	6474.66816	6232.54365	3811.150457	3486.76265	7905.679118	4646.280173
DNAJC1	666.122142	702.887909	2167.341089	2449.88573	1661.542807	2558.715914
NIPSNAP3A	438.546689	392.073313	281.8245469	238.32078	452.4152338	116.881853
LRRC8A	3698.83366	3913.623	678.9865419	716.058553	1590.002759	888.8457195
CDK9	1557.8663	1545.94711	1083.169077	1194.77186	1648.443925	1649.030795
TXN	4956.84732	5780.54204	3291.630473	2726.45328	3695.89995	2147.355216
ODF2	1632.09692	1810.03794	1101.221895	1707.67897	2392.057383	2117.464268
KIAA0368	5772.40742	5138.59817	3150.216733	3247.406	5097.480329	2551.467427
TOR1B	1324.43054	1466.71986	306.8979052	422.394091	871.5794593	582.5971434
C9orf78	1167.17883	1245.28985	990.8991187	1165.60655	1983.976827	1519.464089
SMC2	4340.53783	3876.04084	1213.55054	1784.11218	3030.879784	1489.56408
KLF4	721.795107	596.235842	431.2617622	487.764605	1446.922663	437.6274032
TOR1A	1276.57132	1333.65871	808.3650706	755.280862	1879.185771	1024.754851
RALGPS1	123.066554	197.052391	697.0393598	649.682339	686.179898	559.9456215
FAM129B	21407.7201	23879.9057	4994.612967	5610.8015	8865.927933	6051.580592
OR1J1	0	0	0	0	0	0
OR13C9	0	0	0	0	0	0
ST6GALNAC4	573.333867	666.321486	0	2.01140043	313.3655628	61.61213958
TMOD1	1.95343737	9.14160575	7.020540315	16.0912034	107.8138753	11.77879139
DAB2IP	1697.53707	1797.84913	625.8310224	1318.47298	1896.315078	1223.182183
STXBP1	1189.64336	1568.29325	235.6895677	1348.64399	497.7575178	337.0546459

SLC2A8	290.085449	209.241198	228.6690274	349.983675	542.0921955	362.4243505
ANGPTL2	606.542303	309.798862	11.03227764	1.00570022	8.06085049	1.812121752
CDK5RAP2	2689.88326	2223.44167	2775.119293	2889.37672	2542.190723	3057.955457
ZFP37	129.903585	147.281426	157.4606899	175.997538	4.030425245	87.88790499
SLC31A2	295.945761	362.617028	39.1144389	68.3876147	193.4604118	74.29699185
SLC31A1	1760.04707	2077.17597	2788.157439	1175.66355	2156.277506	1203.248844
TLR4	1231.64226	1156.92099	0	0	34.25861458	20.83940015
ZNF189	321.340447	338.239413	516.5111803	487.764605	533.0237387	355.1758635
ALDOB	0	0	0	0	0	0
STX17	629.983551	597.251576	449.3145802	506.872909	1148.671195	512.8304559
PRPF4	2060.87642	2057.87703	447.3087115	478.713303	2619.776409	848.0729801
FPGS	1495.3563	1492.11321	846.4765751	993.631813	1548.6909	1253.082192
USP20	487.382623	465.206159	708.0716375	601.408729	542.0921955	790.085084
BAAT	0	1.01573397	2.005868661	3.01710065	0	2.718182629
KIF12	0.97671868	1.01573397	0	0	102.7758438	23.55758278
ATP6V1G1	1177.92273	1353.97338	3283.606999	3030.17475	2621.791622	1669.870195
TEX10	1597.91177	1717.60615	359.0504904	423.399791	1598.06361	458.4668033
GARNL3	136.740616	104.620599	160.4694929	262.487756	123.9355763	253.6970453
MRPL50	586.03121	627.723595	274.8040066	359.034977	886.6935539	320.7455502
DPM2	962.067904	1007.6081	498.4583624	576.266224	1675.649296	941.3972503
WDR38	0	1.01573397	0	0	2.015212623	0
TSTD2	768.677604	805.47704	702.0540315	488.770305	1169.830927	469.3395339
GABBR2	48.8359342	36.566423	2.005868661	7.03990151	145.0953088	12.68485227
HEMGN	0	0	0	0	0	0
PSMB7	3243.68275	3493.10913	2340.848728	2404.62922	3769.455211	2197.197625
NR5A1	0	0	0	0	0	0
TRMO	252.970139	294.562852	93.27289276	133.758129	323.4416259	89.70002674
RABEPK	603.612147	714.060982	368.0768994	363.057778	547.130227	458.4668033
GOLGA1	612.842138	696.976337	1564.005883	1488.41621	1305.172607	1226.199366
XPA	386.780599	378.868772	165.4841646	161.917735	271.0460977	150.4061054
NCBP1	2061.85314	2198.04832	1034.025295	966.477907	4325.653894	1230.43067
ANP32B	6293.9752	5542.86029	2791.166242	3023.13485	9559.150999	2531.534088
OR1L4	0	0	0	0	0	0

PDCL	697.37714	705.935111	747.1860764	959.438006	755.7047335	555.4153171
RPL35	9705.60473	10397.0022	9603.056099	8246.7116	12420.70254	6706.644484
CTSV	198.273893	163.53317	54.15845386	48.2736103	395.9892803	96.04245287
LMX1B	30.2782792	52.8181666	619.8134164	717.064254	2220.76431	626.9941263
ARPC5L	1796.18566	1771.44005	1707.997165	1349.64969	2486.772376	1867.391466
ENPP2	0	2.03146794	2.005868661	0	2.015212623	1.812121752
DSCC1	701.284015	791.256764	170.4988362	279.58466	905.8380738	491.9910558
DERL1	3056.15276	3566.24198	3073.993724	3088.50536	5942.862024	3003.591805
MYC	5225.44496	4431.64732	1060.101588	298.692964	2226.809948	1731.482334
NOV	32.2317166	93.4475254	11.03227764	99.5643213	17.12930729	50.73940907
IL33	0	0	0	1.00570022	0	0
TMEM261	182.646394	245.807621	334.9800665	471.673401	675.0962286	286.3152369
RANBP6	530.358245	566.779557	844.4707065	690.916048	900.8000423	626.0880654
POLR1E	1325.40725	1199.58182	592.7341894	516.929911	910.8761054	935.9608851
PLAA	1443.59021	1599.78101	1565.58049	1851.4941	1328.025118	1785.845987
IL11RA	75.2073387	102.690705	628.6292091	487.61375	8.06085049	279.3566893
UBAP2	2252.31328	2575.90135	2426.098146	4085.15428	1947.703	3131.346388
APTX	672.959173	745.548736	1316.852776	1468.32231	712.3776621	1343.688279
RNF38	928.859468	955.805668	884.5880797	1251.09107	613.6322436	1080.930625
TLN1	26745.4877	22122.6859	7483.895976	9024.14803	6156.474562	9157.557276
CCL21	0	0	0	0	0	0
SIT1	0	0	0	0	0	0
IFNA21	0	0	0	0	0	0
DMRT1	0	0	0	0	0	1.812121752
DNAJB5	351.618726	401.214919	254.74532	455.582198	134.0116394	241.0121931
SPAG8	6.83703079	10.1573397	23.06748961	26.1482056	3.022818934	18.12121752
DCTN3	830.210881	860.326674	2243.564098	2382.50381	974.355303	1640.876247
CD72	3.90687474	2.03146794	23.06748961	32.1824069	6.045637868	17.21515665
TMEM8B	126.973429	147.281426	1083.169077	1323.50148	94.71499326	622.4638219
GRHPR	1852.0735	1734.06104	5671.59364	4242.27482	1799.594948	4027.558383
ALDH1B1	2104.82876	1738.93656	351.0270157	311.767067	1223.234062	822.7032756
HINT2	496.173091	482.473637	997.9196591	1499.49902	499.7727304	523.7031864
ARHGEF39	318.410291	328.082073	239.701305	632.585436	386.9208235	777.4002318

IGFBPL1	363.33935	350.42822	0	0	24.18255147	7.248487009
DENND4C	883.930409	828.838921	1451.245977	1652.36545	1518.462711	1420.703454
RPS6	14265.9531	13873.8392	18362.72466	13612.1524	20918.87432	10097.93974
CNPY3	2376.35656	2229.53607	1154.377415	1422.0601	824.2219626	914.2154241
FOXP4	4714.62109	3943.07928	2331.822319	2604.76356	2459.567006	3143.125179
PPIL1	3355.02868	3451.46404	456.3351205	453.570797	1528.538774	757.4668925
KLC4	680.772923	773.989287	484.4172817	540.061016	272.053704	475.68196
KIF13A	2293.33547	2416.43112	1290.776484	1887.6993	1342.131607	2057.66425
ZSCAN9	390.687474	403.246387	324.9507231	302.715765	238.8026958	289.9394804
PIM1	2028.64471	1492.11321	18.05281795	49.2793106	384.9056109	66.14244396
GMPR	362.362632	477.394967	8.023474646	19.1083041	46.34989032	36.24243505
CMTR1	3642.18397	3586.55666	973.8492351	1067.04793	1711.923123	1803.967204
TFAP2A	264.690763	228.540144	5535.194571	4347.64203	425.2098634	5962.786626
SLC22A7	0	0	0	1.00570022	0	0
YIPF3	2849.0884	3010.63549	1869.469592	1975.19522	1307.872992	2089.37638
TMEM14B	2565.83998	2627.70379	1167.415561	938.318301	1715.893092	1128.906549
TMEM63B	1512.93724	1749.0939	1703.985428	2491.11943	1322.987087	2547.843184
FRS3	289.10873	287.452714	102.2993017	90.5130194	56.42595343	82.45153973
TJAP1	1682.88629	1655.64637	521.525852	534.026814	519.9248566	559.0395606
CAPN11	26.3714045	14.2202756	0	3.01710065	2.015212623	0.906060876
TINAG	0	0	0	0	0	0
HCRTR2	0	0	0	0	0	0.906060876
KIAA0319	2.93015605	6.09440383	0	2.01140043	129.9812142	12.68485227
IRF4	0	5.07866986	0	1.00570022	2.015212623	0.906060876
SLC22A23	38.0920287	76.1800479	2366.92502	2735.50459	500.7803367	1223.182183
TUBB2A	1524.55043	1796.60994	147.3912292	136.926084	585.3789626	235.1499792
LRRC1	629.006832	584.047034	1642.806434	1897.75631	817.1687185	1293.854931
GCM1	0	2.03146794	0	1.00570022	1.007606311	0
FOXF2	218.784985	177.753445	0	0	8.06085049	0.906060876
BPHL	464.918094	424.5768	424.2412219	289.641662	326.4644449	357.8940461
RIPK1	1393.77756	1626.19009	748.1890107	1114.31584	940.0966884	1302.009479
TUBB2B	95.9626107	71.4975143	1.945692602	3.40932373	38.80291905	5.943759348
UQCC2	975.741965	922.286447	1794.249518	878.981988	800.0394112	1192.376113

HMGA1	48056.4834	44869.0223	2689.869875	3351.98876	5808.820156	5576.777511
TCF19	3996.73285	3659.6895	724.1185868	1181.69775	1665.573233	1758.664161
FLOT1	5951.14694	4462.11934	7539.057364	5769.70214	1736.105674	5331.262195
IER3	15063.9323	22996.2171	3906.429218	2777.744	2817.267246	2493.479531
MDC1	2894.99418	2860.30687	1616.730141	1874.6252	2937.172397	2713.652324
PGBD1	585.054492	683.588963	173.5076392	196.111542	46.34989032	158.5606533
ATAT1	530.358245	563.732355	432.2646965	305.732866	143.0800962	463.9031686
TPMT	413.152003	565.763823	579.6960431	467.6506	487.6814547	634.2426133
CLPS	0	0	0	0	0	0
RNF144B	515.707465	401.214919	1128.301122	520.952712	249.8863652	826.3275191
NRM	1138.85399	1168.09407	155.4548213	414.348489	472.56736	296.2819065
MTCH1	14961.3768	14806.3541	4632.553674	4958.10206	4210.786775	5553.24711
VARS2	1321.36364	1325.4211	984.4001043	914.965942	823.2345084	1248.388796
TAF8	2900.85449	2835.92925	353.0328844	539.055316	548.1378333	602.5304827
FAM8A1	959.137748	1026.90705	1433.193159	1294.33618	1128.519069	1593.761081
C6orf52	137.717334	120.872343	1.002934331	3.01710065	17.12930729	7.248487009
FGFBP1	3.90687474	2.03146794	0	0	0	0
FGFBP2	0	0	0	0	0	0
CPEB2	341.851539	400.199185	524.534655	758.297963	454.4304464	659.6123179
FHDC1	128.926866	143.21849	433.2676309	488.770305	849.4121204	687.700205
TLR2	120.136398	137.124086	8.023474646	19.1083041	88.66935539	31.71213067
MGARP	0	1.01573397	4.011737323	1.19678326	1.007606311	1.957091493
TTC29	0	0	0	0	0	1.812121752
MYO7A	20.5110924	33.5192211	38.11150457	134.763829	30.22818934	54.36365257
FCHSD2	1237.50257	1242.24265	657.9249209	863.896485	908.8608928	995.7609029
ARRB1	780.398228	1046.20599	688.0129509	1324.50718	1066.047477	1085.46093
SLCO2B1	3.90687474	40.6293589	0	0	3.022818934	4.530304381
PRKRIR	2833.50974	2852.38414	3368.766153	3133.21879	3027.23225	3596.499921
ANKRD42	348.317417	430.061764	167.7006494	140.737688	451.014661	291.244208
IL18BP	159.205145	148.29716	121.355054	105.598523	306.3123186	184.8364187
NUMA1	11407.0975	9631.18952	15713.97509	15857.881	9610.548997	14683.62256
CCDC90B	1189.64336	1085.81962	759.2212883	704.995851	1104.336517	569.0062302
SYTL2	130.880304	145.249958	232.6807647	746.22956	12028.80414	1544.833794

RAB30	429.756221	343.318083	134.3932003	239.356651	1419.717293	330.7122198
CREBZF	2524.8178	1967.4767	2254.596375	2061.68544	4034.45567	2454.518914
LRRC32	0	0	1.002934331	0	4.030425245	3.624243505
PRCP	2907.69152	2917.18797	360.0534247	424.405491	1040.85732	721.2244574
NARS2	493.242935	449.97015	553.6197506	485.753204	780.8948912	602.5304827
RNF121	607.519021	545.449143	528.5463923	388.200283	962.2640273	636.0547351
MRPL15	992.346183	1026.90705	855.5029841	959.438006	1207.112361	933.2427025
PI15	0	0	0	0	0	1.812121752
TTPA	14.6507803	20.3146794	2.005868661	1.00570022	8.06085049	7.248487009
GGH	845.83838	1016.74971	795.3269243	1009.72302	814.1458995	964.9548331
SLCO5A1	0	0	2.005868661	0	9.068456802	1.812121752
SULF1	0	0	1.002934331	2.01140043	493.7270925	19.0272784
TGS1	722.771826	661.242816	717.0980465	602.414429	765.7807966	852.6032845
SDCBP	4751.7364	5488.01065	1563.574622	1555.81823	2080.948858	1529.430759
NEK1	411.198566	401.214919	415.2148129	441.502395	244.8483336	586.2213869
DDX60	401.431379	386.994643	34.09976724	161.917735	44.3346777	1160.663982
NXPE4	0	0	0	0	0	0
SORL1	275.434669	389.026111	1997.845187	3362.05582	459.4684779	2420.994661
TMPRSS4	0	0	6.017605984	0	138.0420646	109.633366
BUD13	1082.2043	1089.88255	446.3057772	470.667701	423.1946507	481.1183253
TRPC6	1.95343737	3.04720192	0	0	23.17494516	2.718182629
MMP7	0	0	0	0	0	0
MMP20	0	0	0	0	0	0
MMP27	0	0	0	0	0	0
C11orf70	12.6973429	11.1730737	0	0	12.09127574	3.624243505
DCUN1D5	1887.99722	2141.16721	1393.075785	1376.8036	757.7199461	1890.042988
YAP1	8138.99679	6990.2812	3281.60113	4370.77314	1944.680181	4822.055983
TRIM29	9.76718684	2.03146794	44.12911055	166.946236	216.6353569	108.7273051
SLC37A4	2010.08705	1969.50817	1110.248304	1009.72302	1023.728012	1258.518557
BTG4	2.93015605	5.07866986	0	0	0	0
POU2F3	6.83703079	5.07866986	1090.189617	1252.09677	32.24340196	704.0093008
RDX	4948.05685	4516.96897	907.6555693	757.292262	10952.6806	1099.051843
PPP2R1B	1674.09582	1738.93656	533.5610639	857.862284	891.7315855	976.7336245

FDX1	1050.9493	1033.00145	478.3996757	512.90711	782.9101039	471.1516556
C11orf1	191.436862	206.193996	423.2382876	238.350951	78.59329228	241.9182539
FXYD6	3.90687474	3.04720192	0	0	13.09888205	3.624243505
ARHGAP20	0.97671868	5.07866986	0	0	3.022818934	1.812121752
FXYD2	0	2.03146794	0	0	1.007606311	0
MMP13	0	4.06293589	2.005868661	24.1368052	30.22818934	38.96061768
TMPRSS13	0.97671868	0	819.3973482	892.056091	380.8751857	638.7729177
CASP1	44.0304783	110.715003	9.026408976	5.02850108	5.038031556	14.49697402
CASP5	0	0	1.002934331	0	0	0
ALKBH8	621.193083	716.09245	257.754123	215.219846	244.8483336	319.8394893
MAP2K5	308.643104	337.223679	637.8662343	664.767842	304.297106	682.2638398
UNC13C	0	0	0	0	1.007606311	2.718182629
SQRDL	886.674988	994.403559	3140.297712	3742.2105	273.8976236	2871.306917
CTDSPL2	1433.82303	1304.20242	971.8433665	1133.42414	1011.636737	1305.633723
SLTM	2864.7159	3167.05853	5504.103607	5862.22656	3259.606417	4867.359027
THBS1	18985.4578	16216.1929	4595.445103	7825.35338	6310.638328	21224.47602
MAPKBP1	1346.89507	1401.71288	569.6666998	929.266999	425.2098634	612.4971523
NUSAP1	4262.40034	3774.46744	1434.196093	4507.54837	2491.810408	4371.743728
NDUFAF1	256.877014	296.59432	453.3263175	446.530896	263.9928536	519.1728821
KIF23	4237.00565	3702.35033	737.1567331	1531.68143	3039.948241	2696.437168
RP11-809H16.2	0	0	0	0	4.765977852	1.938970275
ITGA11	0	2.03146794	0	0	1.007606311	0
CASC5	3017.08401	2562.69681	609.7840731	1532.68713	2368.882438	2159.143068
HAUS2	1210.15445	1123.40177	424.2412219	625.545534	792.986167	801.8638754
RTF1	2289.42859	2448.93461	3801.121113	3409.32373	2417.247541	4242.177022
PARP6	1306.8496	1293.02935	1042.04877	904.124494	1210.13518	1055.560921
RPLP1	12288.0978	12634.7149	23507.77778	25598.0775	17410.42945	19254.69062
PAQR5	1172.06242	1091.91402	605.7723357	209.185645	90.68456802	262.7576541
LRRC49	149.437959	189.942253	18.05281795	19.1083041	70.53244179	49.83334819
TUBGCP4	1552.98271	1354.98912	2660.784779	2332.2188	939.0890821	2990.000891
RMDN3	693.470266	755.706075	1333.90266	1044.92252	1543.652869	1463.288315
ITPKA	108.415774	119.856609	49.1437822	64.3648138	107.8138753	60.7060787
UACA	3988.9191	2722.16705	75.2200748	113.644124	1088.214816	205.6758189

SMAD6	102.555462	77.1957819	1119.274713	1777.07228	681.1418664	1616.412603
PLCB2	14.6507803	18.2832115	4.011737323	4.02280086	19.14451991	10.87273051
TMEM62	284.225137	355.50689	1101.221895	1302.38178	267.0156725	1400.770115
PAK6	208.275492	160.282821	59.09289077	144.951572	132.3087847	323.0378842
ADAM10	7160.32467	5693.18891	4130.083574	4896.75435	2548.236361	4837.459018
DUOX1	97.5839637	95.4485214	3.008802992	0	0	1.812121752
SLC28A2	0.97671868	1.01573397	1.002934331	0	4.030425245	0
STRA6	3.90687474	4.06293589	0	0	202.5288686	124.13034
CYP19A1	0	0	0	0	1.007606311	1.812121752
ZNF280D	426.826065	414.419461	2095.129817	1630.24005	536.0465576	1405.300419
SEMA6D	16.6042176	16.2517436	5.014671654	25.1425054	11.08366942	22.6515219
BCL2L10	0	0	0	0	6.045637868	0
RSL24D1	1951.48393	1806.95011	6387.658664	3128.73337	1590.002759	2238.876425
SPTBN5	188.506706	185.879317	263.771729	288.635962	48.36510294	453.0304381
GCOM1	52.0200371	72.3710455	0	2.3332245	79.43968158	7.348153706
GCHFR	34.1851539	34.5349551	916.6819783	759.303663	100.7606311	519.1728821
BCAR3	4140.3105	4405.23824	2118.197306	2629.90606	498.7651241	1796.718717
TTLL7	362.362632	367.695698	526.5405236	829.702678	123.9355763	574.4425955
FNBP1L	1718.04816	1373.27233	3742.950922	4279.25442	1592.017972	4634.501382
CCBL2	616.661108	592.274479	267.512674	518.076409	281.938322	422.69552
GTF2B	401.431379	402.230653	701.0510972	692.927449	263.9928536	546.3547083
BRDT	0	0	1.002934331	4.02280086	3.022818934	1.812121752
RABGGTB	1756.14019	1764.31975	1641.803499	1359.70669	1202.074329	1607.351994
IFI44L	2.93015605	3.04720192	0	6.03420129	5.038031556	70.67274834
GIPC2	0	0	0	0	0	0
ARHGAP29	14280.6039	10346.2662	697.0393598	1068.05363	700.2863863	1469.630741
IFI44	82.0443694	179.784913	12.03521197	24.1368052	15.11409467	514.6425777
SLC44A5	3.90687474	8.12587178	13.0381463	21.1197045	1.007606311	26.27576541
RPL7P9	110.505952	128.02311	291.1919536	236.892686	158.4863967	222.8275513
CLCA2	1.95343737	4.06293589	45489.0895	21268.5482	7.053244179	30513.41213
DNASE2B	0	0	77.22594346	91.5187196	3.022818934	87.88790499
DBT	905.41822	851.185069	749.191945	932.2841	460.4760843	972.2033201
RTCA	1304.89616	1448.43664	815.3856109	785.451868	523.9552819	744.7820402

IFT172	625.099958	679.526027	855.5029841	643.648138	370.7991226	760.1850751
EPT1	2537.51514	2897.88902	1629.768287	2594.70656	3656.603304	2827.815995
CGREF1	0	3.04720192	603.7664671	292.658763	584.4116605	537.2940996
HADHB	2296.26563	2196.01685	1144.348071	1271.20507	2168.368782	2020.515754
KHK	225.622016	314.877531	403.1796009	518.941311	392.9664614	579.8789608
ADCY3	2923.31902	2913.12503	704.0599002	576.266224	1934.604118	967.6730158
PPM1B	1373.26647	1488.05027	1250.65911	1401.9461	1463.044364	1284.794322
PNPT1	1697.53707	1657.67784	790.3122526	691.921748	1865.079282	1419.797393
DYNC2LI1	139.670772	191.973721	441.2911055	336.909572	201.5212623	333.4304024
LHCGR	0	0	0	0	0	0.906060876
THUMPD2	527.428089	509.898454	273.8010723	301.710065	266.0080662	356.9879852
CYP1B1	2035.48174	5248.29743	3006.797123	1216.89726	3721.090108	2995.437257
SULT6B1	0	0	0	0	0	0.906060876
RAB1A	2962.38777	3358.01651	4346.717389	3456.59164	4553.372921	3445.749512
ACTR2	10559.2959	9665.71432	7272.266803	6936.29427	12577.90928	9664.027184
PREB	1733.67566	1950.20923	2825.26601	1878.648	2354.775949	2223.47339
SLC5A6	1624.28317	1806.99074	1556.554081	2864.23421	3320.062796	2511.600749
ABCG5	0	0	0	0	0	0
PREPL	796.025727	1158.95246	2705.916824	1936.97862	2361.829194	2256.997643
SLC3A1	7.81374947	9.14160575	16.04694929	2.01140043	0	9.966669638
EMILIN1	7.81374947	14.2202756	33.09683291	36.2052078	14.10648836	44.39698293
FBXO11	1882.1369	1907.5484	2163.329351	1812.27179	1792.531628	1839.303579
SIX3	10.7439055	10.1573397	5.014671654	1.00570022	0	4.530304381
ATRAID	1584.23771	1635.3317	2248.578769	2167.28396	2059.5473	1678.930804
CENPO	2380.26343	2012.169	537.5728013	944.352502	1726.029611	1614.600481
LRPPRC	8631.26301	8178.68994	4269.491446	3687.90269	8786.327034	6067.889688
TRIM54	0.97671868	5.07866986	6.017605984	1.00570022	3.022818934	9.060608762
DTNB	276.411388	292.531384	1082.166143	943.346802	403.0425245	1091.803356
ACTR1A	4266.30721	4609.40077	2937.594655	3679.85709	2721.544647	3021.713022
CYP2C9	0	0	0	0	0	0.906060876
TMEM180	163.11202	180.800647	72.21127181	77.4389166	103.7834501	113.2576095
CYP2C8	0	0	6.017605984	1.00570022	14.10648836	5.436365257
MYOF	23139.4423	19267.4577	556.6285535	2529.33604	17319.74488	2050.415763

LOXL4	4166.68191	1950.20923	0	0	65.49441023	35.33637417
STAMBPL1	1229.68882	1181.29861	66.19366583	108.615623	64.48680392	107.8212443
CH25H	4.88359342	32.5034871	0	0	0	0
LBX1	0	0	0	0	0	0
ATAD1	1739.53598	1718.62188	1801.270058	1104.25884	1082.169178	1328.285244
BTBD16	0	0	0	0	1.007606311	0
KIF11	5270.37402	5003.50555	1159.392086	2353.3385	2779.985813	3005.403926
CUZD1	31.2940666	34.6365285	41.53151063	34.5558594	28.22305278	41.153285
TACC2	954.254154	966.978742	1014.969543	1426.08291	789.9633481	1004.821512
DUSP5	2290.40531	3319.41862	2846.327631	2534.36454	173.3082855	4011.131499
CALHM2	456.127625	491.615243	0	0	385.9132172	27.18182629
ARL3	708.121046	769.926351	651.907315	724.104155	851.427333	728.4729444
CEP55	3813.10974	3778.53038	406.1884039	1190.74906	2078.69182	1130.763973
KIF20B	4426.48908	4546.42526	1388.061114	2025.48023	1403.595592	2373.879496
ENTPD1	0.97671868	0	2.005868661	5.02850108	10.07606311	12.07779148
EXOC6	557.706368	535.291803	557.6314879	830.708378	612.6246373	607.060787
PLCE1	587.007929	511.929922	3.008802992	3.01710065	10.07606311	11.77879139
RBP4	0	0	0	0	0	0
DBR1	462.964656	443.875746	462.3527265	566.209221	592.472511	594.3759348
DNAJC13	2628.34998	2267.11823	1857.43438	2806.9093	3225.347802	2660.194732
GPR87	2.93015605	9.14160575	0	0	144.0877025	31.71213067
ANXA7	2999.50308	3080.72114	3133.166849	2793.8352	3014.758083	2715.464446
FAM149B1	153.344833	104.620599	556.6285535	496.815906	271.0460977	340.6788894
ASCC1	774.537916	933.45952	491.437822	660.745042	623.7083067	533.6698561
PLA2G12B	0.97671868	0	0	0	0	0
ZNF365	2.93015605	13.2045416	3.008802992	15.0855032	69.52483548	26.27576541
OIT3	0.97671868	1.01573397	0	0	2.015212623	0
ADAMTS14	44.9290595	31.4877531	0	0	11.08366942	5.436365257
RPS24	14406.0634	14419.8369	12882.53101	11699.7632	14820.35728	11725.82307
TET1	81.0676508	56.8811024	47.13791354	44.2508095	219.6581759	72.48487009
DNA2	941.556811	802.429838	372.0886367	490.781705	688.1951106	941.3972503
MYPN	37.11531	38.5978909	0	0	228.7266327	29.90000891
AOX1	4371.79283	4247.79947	0	0	886.6935539	147.6879228

ATIC	2778.76466	2710.99397	3552.393399	3139.79607	5839.078574	3829.013263
SMARCAL1	656.354956	691.714835	551.6138819	788.468969	657.9669213	694.0426312
BARD1	882.95369	865.405344	399.1678636	749.246661	881.6555224	1003.915451
STAT4	23.4412484	22.3461474	0	0	11.08366942	12.68485227
MSTN	0	0	6.017605984	2.01140043	0	7.248487009
CARF	83.9978068	105.636333	210.6162094	185.04884	146.1029151	154.9364098
ASNSD1	1784.46504	1776.51872	888.599817	983.574811	1734.090462	1143.448826
METTL5	1254.10679	1256.46292	818.3944139	652.69944	672.0734096	635.1486742
SSB	5022.28747	4651.04586	2839.30709	2251.76278	3368.427899	3068.828188
NAB1	2085.29439	2051.78262	16.04694929	13.0741028	279.1069482	126.8485227
CDK15	45.9057781	76.1800479	0	0	0	0
PPIG	2194.68688	2119.8368	2466.215519	1828.36299	2038.387568	2171.82792
FASTKD1	400.45466	404.262121	393.1502576	438.485294	422.1870444	499.2395428
MDH1B	11.7206242	8.12587178	84.24648378	35.1995075	25.19015778	44.39698293
HECW2	48.8359342	47.7394967	60.17605984	5.02850108	243.8407273	34.43031329
IDH1	1114.43602	2344.31401	8414.619035	7156.56273	2736.658741	3497.394982
OLA1	4508.50414	5133.5195	2709.928562	3059.33	3372.458324	2928.379691
CIR1	774.537916	708.982313	1174.436101	778.411967	514.8868251	653.2698917
SSFA2	5487.20557	4941.54577	1262.694322	2004.36053	3128.617597	1894.573292
CHRNA1	0	0	0	0	0	0
FAM117B	237.34264	276.27964	295.8656276	503.855808	467.5293284	533.6698561
WDR12	1817.67347	1917.70574	695.0334912	663.762142	1068.06269	953.1760417
ABI2	3027.82792	2918.2037	1640.800565	1513.57882	2413.217116	1861.955101
ITGAV	3465.39789	3994.88171	2084.097539	3378.14702	5254.666913	3302.591894
SLC40A1	136.740616	166.580371	4993.610033	7195.78504	6.045637868	7172.377896
SLC35A5	802.862758	994.403559	510.4935743	738.183958	475.5901789	430.3789162
DIRC2	451.244032	548.496345	300.8802992	235.33385	504.810762	310.7788805
SENP7	450.267313	363.632762	402.1766666	591.351727	437.3011391	418.6001248
GUCA1C	0	0	0	0	0	0
CCDC54	0	0	0	0	0	0
COX17	373.106537	400.199185	1046.060507	1055.98523	1036.826894	578.9728999
PARP9	324.270603	291.51565	499.4612967	592.357427	497.7575178	1975.21271
MNS1	110.369211	180.800647	75.2200748	58.3306125	58.44116605	85.16972236

USP8	1280.47819	1290.99788	1681.920873	1870.6024	4060.653434	2324.952208
SECISBP2L	1418.19553	1408.82302	1203.521197	2078.78235	939.0890821	1556.612585
TMOD3	4724.38827	3733.83808	3467.143981	3963.46455	5368.526427	3644.176844
SPPL2A	1743.44285	1776.51872	2834.292419	2901.44512	3881.299511	3225.576719
GLCE	2097.01501	1758.23551	301.8832335	284.613161	934.0510506	420.4122465
SHF	8.79046815	7.11013781	20.05868661	54.0463296	22.16733885	12.68485227
APH1B	419.012315	401.214919	481.4084787	531.009714	307.3199249	510.1122733
VWA9	733.515732	693.746303	1449.240108	1262.15377	1364.298945	1508.591359
CILP	0	0	7.020540315	4.02280086	8.06085049	8.154547886
PARP16	186.553269	198.068125	523.5317206	499.833007	396.9968866	562.6638041
PPCDC	258.830451	229.555878	127.37266	112.638424	376.8447604	154.030349
HCN4	8.79046815	14.2202756	0	0	14.10648836	5.436365257
SEMA7A	2464.26124	2676.45902	4.011737323	3.01710065	351.6546026	163.0909577
UBL7	956.207592	967.994476	221.6484871	376.131881	692.2255359	421.3183074
ARHGAP24	82.0443694	108.683535	3.008802992	2.01140043	85.64653646	12.68485227
FAM13A	360.409194	468.253361	164.4812302	57.3249123	63.47919761	168.527323
HERC3	557.706368	472.316297	1121.280582	1196.78326	851.427333	1187.845809
HERC6	241.249515	312.846063	22.06455528	42.2394091	99.75302482	692.2305094
HERC5	659.285112	835.949059	12.03521197	63.3591136	79.60089859	205.6758189
PCDH10	7.81374947	19.2989455	0	0	3582.040437	96.94851375
NDST4	0	0	0	2.01140043	0	1.812121752
ZGRF1	442.453564	439.81281	567.6608312	553.135119	524.9628882	1054.65486
AP1AR	669.052298	717.108184	1449.240108	1407.9803	774.8492534	922.3699719
COPS4	956.207592	993.387825	1045.057573	1146.49825	1125.49625	1185.127626
HNRNPD	8889.11674	7826.23026	5693.658195	5562.52789	6501.07592	7206.364239
PRKG2	11.7206242	7.11013781	32.09389858	19.1083041	9.068456802	14.49697402
RASGEF1B	0	0	42.12324189	28.159606	1.007606311	20.83940015
SEC31A	4801.9202	4246.35713	4429.32909	3560.7319	4545.362451	3999.289283
FGF5	597.751835	664.290018	0	0	25.19015778	2.718182629
GPAT3	961.091185	1163.0154	15.04401496	10.0570022	331.5024764	36.24243505
IL21	0	0	0	0	0	0
FGF2	0.97671868	0	1.002934331	1.00570022	0	0
BBS7	498.126529	542.401941	305.8949709	338.920973	193.4604118	259.1334106

KIAA1109	2484.77233	2074.12877	2877.418595	2839.09171	2512.97014	3428.534355
BMPR1B	30.2782792	31.4877531	0	0	539.0693765	13.59091314
RAP1GDS1	1428.93943	1262.59796	851.3307773	1225.11383	979.2724218	1253.480859
LARP1B	547.939182	569.826758	1045.057573	731.144057	629.7539446	698.5729355
MMRN1	0	0	0	0	0	0
PDE5A	41.0221847	55.8653685	219.6426184	267.516257	372.8143352	196.6152101
PRDM5	427.802784	369.727166	145.425478	149.849332	91.69217433	153.1242881
TRPC3	20.5110924	55.8653685	0	0	3.022818934	2.718182629
NAAA	354.548882	487.552307	332.9741978	356.017876	677.1114412	563.569865
NUP54	1392.80084	1082.77241	995.9137904	753.269461	1214.165605	1147.97913
CXCL9	0	0	0	0	0	0
BMP2K	2144.87423	1991.85432	759.2212883	1294.33618	790.9709544	1026.566973
G3BP2	4756.61999	4573.85008	5987.517954	5539.39679	3400.671301	6451.425257
SEPT11	6789.17157	6078.15209	2738.010723	5756.62803	2935.157185	3741.125358
FRAS1	1470.93834	1069.56787	796.3298586	651.69374	471.5597537	955.8942244
SCARB2	2547.28233	2835.92925	12354.14509	9310.7726	3814.797495	10546.5486
CCNG2	723.748545	889.78296	3755.989069	8747.58047	610.6094246	3074.264553
CNOT6L	855.605567	828.777977	1144.317983	1300.35026	924.9624416	1542.088429
USO1	2407.61156	2496.6741	3663.71911	3092.52816	1709.90791	2987.282709
CDKL2	59.5798397	82.2744518	1.002934331	0	3.022818934	6.342426133
SHROOM3	1344.94163	1063.47347	3412.985527	3321.82781	1317.949055	3135.876692
ANXA3	2890.11059	1941.06762	75.2200748	146.832231	1594.033184	202.9576363
PPA2	735.469169	760.784745	1980.795303	1990.28073	1432.816175	2194.479442
CENPE	3313.02978	3220.89243	1121.280582	1816.29459	1237.34055	1569.297438
GSTCD	461.987937	423.561066	1171.427298	1239.02267	903.8228612	2554.18561
INTS12	294.969043	339.255147	525.5375893	452.565097	433.2707139	683.1699006
ENPEP	0	1.01573397	0	0	156.1789782	9.966669638
CASP6	356.50232	368.711432	527.543458	687.898947	527.9857071	614.309274
LEF1	119.159679	121.888077	6.017605984	13.0741028	50.38031556	43.49092206
HADH	1020.67102	926.349383	2028.936151	2030.50874	1204.089542	1842.927822
EGF	15.6274989	26.4090833	113.3315794	39.2223084	14.10648836	260.9455323
PAPSS1	1000.15993	1219.8965	1699.973691	1793.16348	1553.728932	1580.170168
SEC24B	1420.14897	1470.78279	1337.914397	1659.40536	1526.523562	1522.182272

C4orf17	0	0	0	0	0	0
PPP3CA	2426.16921	2354.47135	1193.491854	1818.30599	5394.724191	1760.476282
SLC39A8	829.234163	1089.88255	789.3093183	1313.44448	2071.638576	1003.915451
MTTP	8.79046815	6.09440383	9.026408976	6.03420129	1.007606311	2.718182629
FBN2	0.97671868	0	11.03227764	12.0684026	232.7570579	54.36365257
MAPK8IP3	1986.6458	1555.08871	4914.37822	3855.85463	1379.41304	4301.97704
RGS3	964.021341	1040.11159	8166.894255	4997.32437	973.3476967	4398.019493
GUCD1	2132.17689	2134.05708	5523.159359	7785.12537	2168.368782	4683.428669
TTLL8	0	0	0	0	0	0
RNF185	894.674314	1015.73397	5518.144688	3814.61086	770.8188281	1969.776345
KIAA1644	0.97671868	0	0	0	0	0
PARVG	0	0	0	0	0	0
B4GALNT3	33.2084353	23.3618814	133.390266	145.826531	18.1369136	115.9757922
PDE6H	0	0	0	0	0	0
ERP27	2.93015605	0	94.27582709	177.003238	32.24340196	96.94851375
ETV6	1071.4604	1003.54516	651.907315	847.805282	327.4720512	1195.094296
GABARAPL1	341.851539	313.861797	45.13204488	16.0912034	64.48680392	58.89395695
KIF21A	668.07558	703.903643	1312.841039	1508.55032	2173.406813	1572.01562
CPNE8	605.565584	758.753277	0	0	35.2662209	22.6515219
YARS2	558.683087	565.763823	451.3204488	536.038215	613.6322436	523.7031864
FGD4	21.487811	24.3776153	413.2089443	630.574035	576.3508101	472.9637774
ALG10	133.283032	129.424823	184.5298875	207.697209	492.679182	343.5511024
PIK3C2G	0.97671868	0	102.2993017	90.5130194	26.19776409	66.14244396
FAM60A	1459.13958	1243.80688	1148.359809	965.371637	1220.211243	1207.733845
PLCZ1	0	11.1730737	0	0	0	0
AEBP2	815.560101	794.303966	745.1802077	623.534134	940.0966884	742.9699185
SLCO1C1	0	0	0	0	0	0
METTL20	46.8824968	46.7237627	290.8509559	232.31675	17.12930729	165.8091403
ETNK1	686.633235	975.104613	1524.460183	1613.14315	934.0510506	1668.964134
ZCRB1	683.703079	799.382636	1197.503591	1056.99093	717.4156936	745.6881011
TMEM117	30.2782792	31.4877531	38.11150457	50.2850108	193.4604118	58.89395695
PRICKLE1	630.96027	292.531384	135.3961346	155.883533	51.38792188	344.3031329
C1RL	152.368115	137.124086	6.017605984	5.02850108	14.10648836	15.4030349

NDUFA9	983.438508	1058.85188	1281.629723	1309.73345	1412.845418	1283.389928
CLSTN3	334.03779	416.450929	1280.74714	1821.32309	331.5024764	2459.955279
KLRG1	2.93015605	3.04720192	3.008802992	3.01710065	4.030425245	6.342426133
VAMP1	129.903585	125.951013	216.6338154	290.647362	170.2854666	307.154637
TAPBPL	42.9756221	41.6450929	151.4430839	134.763829	70.53244179	150.4061054
CD27	0.97671868	0	0	1.00570022	0	2.718182629
RBP5	0.97671868	0	50.14671654	37.210908	1.007606311	43.49092206
PEX5	967.928216	1060.42627	763.2330257	801.543072	949.1651452	1235.867035
PIANP	0	0	0	0	7.053244179	0.906060876
SLC38A4	235.389203	181.816381	0	0	0	0
AMIGO2	1524.65787	2429.63566	2171.352826	2385.52091	692.2255359	582.5971434
SCAF11	3395.07415	3070.5638	3202.369318	3283.6112	4853.639601	3873.410246
COL2A1	0	0	3.008802992	9.05130194	6.045637868	2.718182629
PPFIA2	23.4412484	57.8968364	0	1.00570022	46.34989032	2.718182629
ANP32D	0	0	2.015898005	0	0	0
LLPH	894.615711	974.088879	601.7605984	604.42583	1743.07831	801.872936
RPL14P1	132.687233	93.2443787	126.3396376	122.423887	114.6655982	71.73283957
LRIG3	266.644201	247.839089	157.4606899	263.493456	306.3123186	287.2212977
MARCH9	342.828258	389.026111	1162.400889	1343.61549	1013.651949	1083.648808
INHBE	1.95343737	1.01573397	14.04108063	0	5.038031556	0
GLIPR1	1147.64445	1135.59058	5.014671654	4.02280086	43.32707139	25.36970453
TPH2	0	0	0	0	0	0.906060876
PHLDA1	15051.2349	15453.3767	16.04694929	17.0969037	1008.613918	538.2001604
TMEM19	1088.06461	1171.85228	281.8245469	874.265254	631.1242891	661.4244396
LGR5	7.81374947	7.11013781	0	0	0	0
PTPRQ	1.95343737	0	0	0	13.09888205	0
DUSP6	3797.48224	4033.4796	1798.261255	1916.86461	108.8214816	1841.1157
POC1B	1023.61095	884.653503	579.194576	491.63655	814.5187139	508.4632425
TMTC3	2084.31767	2142.18295	853.4971154	1252.09677	2491.810408	1521.276211
LUM	0	0	0	0	0	22.6515219
KERA	0	0	0	0	0	0
SNRPF	1703.39738	1612.98555	898.6291603	1000.67171	1939.642149	1240.397339
AMDHD1	25.3946858	29.4562852	4.011737323	3.01710065	3.022818934	10.87273051

NEDD1	1579.35411	1217.86503	845.4736408	733.155457	1352.20767	1260.330679
SYCP3	0	0	4.011737323	7.03990151	3.022818934	6.342426133
ASCL1	0	0	0	0	493.7270925	68.86062659
GAS2L3	1233.5957	1361.08352	238.6983707	760.309363	798.0241985	505.5819689
TMEM132B	0	0	2.005868661	2.01140043	4.030425245	9.966669638
SLC15A4	892.720877	864.38961	471.3791354	627.556935	623.7083067	588.9395695
TDG	1144.70453	1310.28667	1562.551629	1400.93034	3410.707059	1655.3551
RITA1	1561.77318	1609.93835	828.4237572	1278.24497	1553.728932	1318.318575
SDSL	202.180768	218.382804	307.9008395	239.356651	487.6814547	346.1152547
MMAB	418.035597	794.303966	724.1185868	1886.6936	791.9785607	354.2698026
GLTP	2406.63484	2254.92942	1151.368612	838.75398	1704.869879	916.0275458
GIT2	2332.40422	2329.078	1203.521197	1135.43554	1229.2797	1335.533731
TCHP	1192.57351	1160.98393	684.0012135	501.844408	853.4425457	753.842649
FAM222A	161.158583	171.659041	490.4348877	626.551234	640.837614	875.2548064
FOXN4	0	1.01573397	4.011737323	1.00570022	0	5.436365257
NUP58	3569.90679	3425.05495	3072.990789	2985.92394	2376.943288	4248.519448
MTMR6	1426.986	1333.65871	1476.319335	1455.24821	1045.895351	1270.297348
SLC46A3	183.623113	307.767394	1002.934331	940.329702	228.7266327	675.9214136
SLC7A1	10087.5506	8486.45734	7053.637148	2588.67235	6490.999857	4194.155796
PDX1	0	0	0	0	0	0
LNX2	342.828258	346.365285	1275.732469	2277.91099	1138.595132	1946.218762
SUOX	202.180768	231.587346	654.916118	762.320763	515.8944314	815.4547886
CCDC65	3.90687474	1.01573397	4.011737323	4.02280086	2.015212623	12.68485227
SLC39A5	0.97671868	4.06293589	6.017605984	6.03420129	3.022818934	0.906060876
TARBP2	461.011219	609.440383	319.9360515	384.177482	1032.796469	426.7546727
RDH16	3.90687474	5.07866986	20.05868661	30.1710065	10.07606311	65.23638308
DHH	4.88359342	8.12587178	0	0	1.007606311	0
ACVRL1	4.88359342	16.2517436	0	0	1.007606311	1.812121752
GPR84	0	0	0	3.01710065	0	3.624243505
NPFF	0	0	0	0	0	0
NABP2	976.718684	1074.64654	1098.213092	1333.55849	1706.885091	1426.139819
N4BP2L1	73.0390232	24.3776153	52.08237979	54.3078116	14.10648836	55.26971345
CELA1	0	3.04720192	1.002934331	0	0	0

SMARCC2	2642.02404	2442.8402	3433.044214	3261.4858	3979.037323	3694.010192
BRCA2	1806.92957	1478.90866	484.4172817	853.839483	1950.725819	1958.903614
KANSL2	473.708562	509.898454	572.6755028	622.528433	955.2107831	845.3547975
CERS5	566.496837	602.330246	550.6109476	550.118018	738.5754262	528.2334908
MAP3K12	288.132012	375.82157	297.8714962	525.981213	200.5136559	341.5849503
ITGB7	2.93015605	5.07866986	0	3.01710065	25.19015778	5.436365257
GALNT6	1030.43821	1001.5137	7614.277439	8015.43072	2344.699886	6920.492972
CSAD	106.462337	114.777939	732.1420614	525.981213	417.1490129	707.6335443
LMBR1L	175.809363	195.020923	269.789335	192.088741	261.9776409	243.7303757
C12orf10	695.423703	709.998047	771.2565003	662.756442	1239.355763	752.9365881
ESYT1	4823.03686	4735.97138	2383.974904	3822.7872	5448.127325	2866.776612
TMBIM6	11719.6475	12390.9387	23415.50782	27680.8927	19773.26625	19041.77537
ANKRD52	4336.63096	5087.81147	2309.757764	2361.38411	9635.739155	3572.598035
KRT71	0	0	0	0	0	0
ZNF740	891.744158	766.879149	954.7934828	959.438006	1303.842567	1131.670034
SMIM2	0	0	0	0	0	0
WDFY2	1125.17992	927.365117	4129.08064	1757.96398	508.8411872	2129.243059
HNRNPA1L2	219.996116	203.167109	248.085836	243.087799	240.4047898	257.5387434
LPAR6	0	0	5.014671654	0	17.12930729	9.060608762
ESD	1051.92602	985.261953	2391.998379	2864.23421	1433.823781	1655.373221
RB1	2258.1736	2271.18116	2647.746633	3103.59087	2315.479303	3099.634257
SBNO1	4877.73311	4861.30279	2877.418595	2982.90684	4473.772022	4050.092117
MORN3	1.95343737	2.03146794	31.09096425	20.1140043	44.3346777	37.14849592
SETD1B	1598.88849	1427.10623	1466.289991	1997.32063	3476.241774	2758.049307
VPS33A	1096.85508	1219.72383	666.9513299	993.752497	1288.09368	873.1980482
VPS37B	1805.36682	1829.73302	944.9948144	884.845221	744.8225853	1228.35579
RHOF	1883.3871	1499.46712	168.7236425	219.594642	1851.265	419.2706098
DENR	4691.17007	4694.68179	2333.818158	1893.72345	3869.188083	3375.076764
DIAPH3	3441.95664	2586.05869	157.4606899	378.143281	791.9785607	460.2789251
SLAIN1	246.133108	264.090833	0	0	118.8975447	18.12121752
RBM26	2039.38861	1760.26697	1045.057573	855.850883	1643.405894	1234.960974
SRRM4	0	0	0	0	0	0
METTL21C	0	0	0	0	0	0

MBNL2	2720.16153	1698.3072	299.8773649	463.627799	1991.030071	566.2880476
RNF113B	0	0	0	0	0	1.812121752
ZIC5	1.95343737	0	0	0	160.2094035	3.624243505
ABHD13	611.425896	703.903643	280.8216126	260.476356	540.0769829	297.1879674
RAB20	355.525601	326.050605	386.1297173	310.761367	357.7002405	319.8394893
GRTP1	4.88359342	9.14160575	360.0534247	345.960874	758.7275524	413.1637595
CUL4A	4274.12096	3759.23143	2004.865727	1450.21971	2826.335703	2207.164294
TTC6	0	1.01573397	1634.782959	867.919286	71.5400481	603.4365435
SSTR1	0	0	4.011737323	5.02850108	6.045637868	9.060608762
CDH24	878.070097	842.043463	154.4518869	253.436454	631.7691572	530.9516734
REM2	10.7439055	11.1730737	35.10270157	28.159606	16.12170098	28.99394804
CBLN3	33.2084353	22.3461474	7.020540315	60.3420129	46.34989032	15.4030349
TSSK4	0.97671868	0	10.02934331	3.01710065	5.038031556	1.812121752
NOVA1	0	0	366.0710307	323.835469	17.12930729	462.0910469
FITM1	5.8603121	5.07866986	3.008802992	11.0828164	4.030425245	6.342426133
MDGA2	0	0	0	0	2.015212623	0
TMX1	3817.99334	3869.94643	1410.125669	1760.98108	2246.962074	2146.458216
FRMD6	9960.57714	8688.5884	316.9272485	598.391628	1129.526675	511.924395
PELI2	2.93015605	7.11013781	1753.12921	413.342789	2.015212623	3241.885815
RTN1	77.160776	140.171288	18.05281795	167.951936	12.09127574	70.67274834
C14orf37	81.0676508	110.887678	4.352734995	9.69495008	10.07606311	11.43448826
SYT16	231.482328	161.501702	0	0	1.007606311	3.624243505
SLC38A6	235.389203	270.185237	389.1385203	372.10908	228.7266327	271.8182629
NAA30	1180.85289	1347.87898	1384.049376	1462.28811	1503.348616	1437.91861
ADAM21	0.97671868	1.01573397	4.011737323	0	0	4.530304381
RDH12	0	2.03146794	0	9.05130194	6.045637868	13.59091314
DCAF5	1618.42286	1730.81069	3370.862286	3062.35716	1863.06407	3460.246486
RAB15	885.883846	912.129107	254.74532	715.052853	2665.118693	738.4396141
WDR89	571.38043	632.802265	425.2441562	475.696202	659.9821339	453.0304381
ESR2	11.7206242	9.14160575	1.002934331	2.08179945	2.277190264	7.121638487
KCNH5	6.83703079	5.07866986	0	0	0	0
STON2	26.3714045	1.01573397	135.3961346	246.396553	462.4912969	476.5880209
EFCAB11	293.992324	342.302349	401.1737323	412.337088	336.540508	340.6788894

GPR65	1.95343737	0	0	0	72.54765441	22.6515219
PTGR2	580.844834	503.164138	1106.818269	814.174667	461.9774177	691.5419031
JDP2	324.270603	167.596105	2023.921479	1341.60409	311.3503502	1325.567062
AK7	0.97671868	5.07866986	5.014671654	6.03420129	907.8532865	77.01517448
FAM181A	0	0	0	0	0	0
SLC24A4	0	1.01573397	0	0	0	0
FBLN5	81.0676508	90.4003235	40.11737323	22.1254047	8.06085049	48.92728731
SERPINA10	0	0	0	0	0	0
C14orf79	149.437959	141.187022	420.2294846	248.407953	376.8447604	546.3547083
WARS	3828.73724	3332.62316	7093.754521	2124.03886	4465.711172	3752.904149
SLC25A47	0.97671868	0	1.002934331	1.00570022	0	0.906060876
WDR20	904.441501	869.46828	1075.145603	1124.37284	1140.610344	1167.006409
NIPA2	2295.28891	2375.80176	1078.154406	1181.69775	2190.536121	1674.400499
SLC12A6	596.775116	636.865201	122.3579883	274.556159	460.4760843	347.0213156
DUOXA1	4.88359342	12.1888077	1.002934331	0	0	1.812121752
MFAP1	836.071193	970.025943	1220.57108	1139.45834	600.5333615	1635.439882
TCF12	1991.5294	1802.9278	2848.333499	2908.48502	938.0814758	3252.758545
SORD	1894.16031	1979.21859	10036.13317	7184.54131	1442.176837	9019.364871
SERF2	3955.37859	4011.44833	16413.33123	14502.9212	5620.448156	11683.77279
ZSCAN29	750.119949	668.352954	1289.773549	1227.95996	762.7579776	1469.630741
DUOXA2	0	0	0	0	0	0
DUOX2	5.94821678	3.07767394	1.002934331	2.01140043	0	1.812121752
LYSMD2	199.250612	285.421246	124.363857	107.609923	313.3655628	175.77581
SLC27A2	13.6740616	8.12587178	71.20833748	52.2964112	437.3011391	444.8758902
FGF7	0	0	2.025927348	3.2584687	0	0
HDC	0	0	0	0	0	0
GCNT3	0	0	0	0	8.06085049	8.154547886
BNIP2	1878.23003	1732.84216	2022.918545	1671.47376	972.3400904	2087.564259
GTF2A2	927.88275	1072.61507	1298.799958	1265.17087	757.7199461	1088.179112
SRP14	2710.39435	2992.35228	4440.993216	3968.49305	3618.314264	4406.174041
BAHD1	872.209785	912.129107	1948.701405	2508.21634	1413.671655	2488.949227
DISP2	445.38372	484.505105	85.24941811	185.04884	81.61611121	273.6303846
CDAN1	551.846056	490.599509	631.8486283	639.625337	464.5065095	790.085084

TLE3	2131.20017	2059.9085	4044.834156	2790.8181	2892.83772	4826.586287
ANP32A	2793.41544	2549.49227	9285.156004	4956.09066	4089.874018	8672.814707
COMMD4	1040.2054	1064.4892	313.9184455	498.827307	1260.515495	484.7425688
UBE2Q2	1204.29414	1132.54338	508.4877057	608.44863	698.2711737	679.5456571
PSTPIP1	5.8603121	2.03146794	0	0	0	0.906060876
ETFA	2828.57731	2745.52893	2519.371039	3098.56236	4226.908476	2663.818976
BCL2A1	7.81374947	17.2674775	0	0	1.007606311	0
HMG20A	1015.78743	1040.11159	240.7042394	281.59606	830.2676005	442.1577076
SCAPER	541.102151	453.017352	366.0710307	319.812669	339.5633269	324.3697937
TSPAN3	3417.53867	3213.78229	321.9419202	235.33385	1874.147739	722.1305183
WDR61	662.215268	607.408915	602.7635328	490.781705	2031.334324	676.8274745
NCOA2	748.166512	806.492774	1887.52241	2829.03471	2157.285112	3112.31911
NEIL1	84.9745255	49.7709646	609.7840731	416.359889	40.30425245	448.5001337
MAN2C1	979.64884	1079.72521	782.288778	660.745042	1090.230029	825.4214582
DNAJA4	0	0	1324.876251	1111.29874	1101.313698	1429.764063
MESDC1	1092.94821	1073.63081	412.2060099	501.844408	1488.234522	598.9062392
TPM1	2397.84437	2148.27735	2363.916217	1750.92408	4765.977852	3609.746531
IGF1R	2468.16811	1989.82285	1260.688454	1104.25884	8733.931506	2040.449093
ARRDC4	70.3237452	76.1800479	417.2206816	303.721465	720.4385126	725.7547618
PIF1	415.105441	366.679964	97.28463008	239.356651	256.9396094	270.912202
USP3	811.653226	821.728784	3123.137506	2503.18784	1608.139673	2623.046237
CYP11A1	15.6274989	20.3146794	0	0	4.030425245	0
BBS4	168.972332	184.863583	288.8450872	189.071641	464.5065095	252.7909845
PML	2952.62058	2824.75618	245.718911	237.345251	700.2863863	508.3001515
CYP1A1	66.4168705	287.452714	146.4284123	12.0684026	276.0841293	165.8091403
ADAMTS17	40.045466	37.582157	4.011737323	0	98.74541851	21.74546103
LINS1	258.830451	206.193996	291.8538902	196.111542	565.2671406	285.409176
ULK3	633.890426	652.10121	548.6050789	1025.81422	957.2259957	848.979041
GOLGA6D	0	0	0	0	1.219203637	0.054363653
PCSK6	119.22805	67.0384422	212.6120488	323.815355	761.7906756	396.4106939
CCDC33	1.95343737	0	0	3.01710065	0	0
CELF6	1.66042176	20.9647492	38.10147522	54.3882677	0	28.69494795
SCAMP2	2002.2733	2479.40663	884.5880797	1163.59515	2898.883358	1304.727662

CYP1A2	0	0	29.08509559	6.03420129	1.007606311	9.060608762
LMAN1L	0	0	3.008802992	4.52565097	0	3.624243505
HAPLN3	273.481231	327.066339	0	0	21.15973254	16.30909577
RHCG	0	0	0	0	43.32707139	13.59091314
POLG	3196.80025	2758.73347	1929.645652	2090.85075	2293.311964	2966.443309
RLBP1	0	0	0	0	0	0
FANCI	3245.63619	2948.67572	1384.049376	2379.48671	4665.217221	3913.276924
ABHD2	4667.73859	5730.77107	519.5199833	749.246661	4854.647208	1831.149031
WDR93	0	0	0	1.00570022	6.045637868	2.718182629
TICRR	1354.70881	1351.94192	295.8656276	899.095993	1680.687327	1038.345764
NTRK3	0	0	0	0	8.06085049	0
DET1	129.903585	103.604865	48.14084787	10.0570022	108.8214816	16.30909577
MFGE8	1914.36862	2101.55359	20.05868661	76.4332164	211.5973254	94.23033112
ZNF710	675.889329	547.480611	270.7922693	321.824069	628.7463382	481.1183253
UNC45A	2695.74357	2713.02544	2172.35576	2171.30677	3370.443111	2519.755297
ST8SIA2	0	0	0	0	0	0.906060876
MCTP2	4.88359342	7.11013781	0	0	3.022818934	1.812121752
FURIN	4557.36938	4554.55113	1040.042901	1001.67741	1688.748178	1681.648986
IQGAP1	17056.4384	15543.777	2892.46261	4960.11346	9549.085012	4475.034667
CRTC3	1272.66445	1272.71467	533.5610639	584.311825	1270.591559	659.6123179
EFTUD1	997.229776	1164.02097	400.170798	541.066716	804.0698364	445.7819511
SH3GL3	0	0	0	4.02280086	11.08366942	2.718182629
SEC11A	2294.31219	2334.15667	1428.178487	985.586211	3710.006438	1073.682138
SEPT12	0	0	16.04694929	21.1197045	0	15.4030349
GLYR1	1655.53817	1521.56949	3593.513707	2939.66173	3250.53796	4388.052823
PMM2	873.450217	1018.87259	1371.402374	1022.39484	2053.562119	1720.219998
SLC5A2	4.88359342	6.09440383	25.07335827	14.079803	12.09127574	8.154547886
ITGAX	4.88359342	8.12587178	0	0	1.007606311	5.436365257
TGFB1I1	1445.54365	1254.43146	0	0	46.34989032	18.12121752
C16orf58	1030.43821	1300.13948	2966.67975	2038.55434	1788.501203	2283.273408
ARMC5	286.178574	342.302349	217.6367498	523.969812	468.5369347	512.8304559
PARN	876.116659	906.034703	1421.157947	1176.66925	1648.443925	1639.064125
FTO	736.445888	776.020755	1384.049376	1401.9461	962.2640273	1927.191484

UQCRC2	2482.81889	2412.36818	5556.256192	4220.9238	3961.908016	6149.435167
CDR2	956.813157	996.485813	481.4084787	585.317525	592.0694685	547.2607692
IGSF6	0	0	1.002934331	0	0	0.906060876
ARHGAP17	1664.32864	1428.12196	728.1303241	986.591911	1089.222423	956.8002852
MYLK3	3.90687474	1.01573397	13.0381463	22.1254047	4.030425245	10.87273051
ABCC12	0	0	124.363857	98.5586211	0	404.1031508
NKD1	21.487811	23.3618814	1372.014164	919.209997	46.34989032	1058.623407
DHX38	1473.86849	1482.9716	1352.958412	1503.52182	3509.492782	1943.500579
TXNL4B	263.714045	270.185237	128.3755943	174.991838	578.3660227	268.1940193
MARVELD3	0	0	1523.457248	1309.42168	872.5870656	1110.830634
CHST4	32.2317166	73.132846	0	0	6.045637868	7.248487009
ZFHX3	2431.0528	2044.67249	2176.367498	2019.44603	4879.837366	3444.843451
CLEC18B	1.17206242	0	0	0.60342013	2.42833121	5.536031953
CPNE2	988.439308	888.767226	154.4518869	307.744266	813.1382932	256.415228
NLRC5	656.354956	770.942085	13.0381463	32.1824069	35.2662209	163.0909577
KATNB1	875.139941	909.081905	955.7964172	815.622875	663.0049528	867.1002585
KIFC3	5985.33209	5494.10506	1.002934331	0	633.7843698	263.663715
ADAMTS18	0	0	0	0	0	0
NUDT7	0	6.09440383	0	0	93.70738695	5.436365257
GCSH	739.629991	785.182675	268.2147281	409.07862	2331.046821	538.6169485
CMTM3	832.164319	794.303966	4.011737323	0	80.6085049	24.46364366
CMTM2	0	0	4.011737323	1.00570022	0	3.624243505
CDH11	860.450092	657.078307	0	0	9.068456802	1.812121752
NOL3	484.452467	444.89148	768.2476973	981.56341	1081.161572	1054.65486
MAP1LC3B	1856.4199	1939.65575	2545.637889	1820.43807	2338.089989	1776.96659
MBTPS1	3433.16617	13330.5917	3166.263682	13180.0241	5514.629342	5351.810388
CDH13	0	0	2.005868661	0	3.022818934	0.906060876
ZCCHC14	1268.75757	1402.72862	869.5440647	1222.93146	2737.666348	1499.53075
TLDC1	1530.51818	1351.94192	328.9624605	411.331388	981.4085472	455.7486207
ADAD2	0	0	0	1.00570022	3.022818934	0
OSGIN1	158.228427	312.846063	518.517049	327.85827	1388.481497	538.2001604
IRF8	0	0	0	0	20.15212623	2.718182629
RHOT2	1464.10131	1195.51889	2681.8464	2437.81732	2898.883358	3547.22833

RPL3L	0	3.04720192	0	0	0	0.906060876
ZSCAN32	247.109827	223.461474	463.3556608	425.411191	438.3087454	545.4486475
RPS2	31784.5845	33018.4846	35064.64022	61616.9693	62200.81726	54648.18287
NDUFB10	1111.50586	1218.88077	2246.572901	2483.07383	2940.195216	2966.443309
PDPK1	1865.19083	2042.4074	2825.988122	3400.96636	3782.523864	3892.047918
TIGD7	239.296078	207.20973	299.8773649	255.447855	351.6546026	356.0819243
DEF8	874.163222	835.949059	770.253566	730.138356	1926.543267	860.7578324
TCF25	1491.44943	1363.11499	2487.27714	2499.16504	4069.701739	2546.937123
GALNS	560.636525	644.991072	316.9272485	528.998313	1628.291799	562.6638041
GAS8	495.196373	555.606483	859.5147214	706.001551	1191.998266	904.2487544
MED9	335.014509	355.527205	634.8574313	427.422592	314.3731691	598.0001783
NCOR1	6654.30626	5902.43011	6560.193457	5976.87638	5185.142078	6778.241415
CDRT15P1	0	0	0	0	0	0
COPS3	2968.24808	2881.63728	1372.014164	1455.24821	2291.296752	1909.070266
GID4	461.011219	504.819784	349.0211471	309.755666	281.1221608	287.2212977
ZNF287	31.2549979	37.582157	0	0	136.026852	14.49697402
MYOCD	0	0	0	0	0	0.906060876
KSR1	403.384816	414.419461	44.44002019	35.1995075	305.3047123	116.0210952
CIRH1A	1994.45955	1794.80193	726.1244554	672.813444	2425.308391	1305.633723
RANBP10	503.986841	590.141438	1419.152078	1252.09677	1275.62959	1680.742925
CTRL	2.75434669	6.74447358	4.172206816	5.73249123	7.738416471	12.91136749
DPEP3	0	0	0	0	0	0
GFOD2	677.842767	795.3197	1150.365677	944.352502	1061.009446	1211.403391
NOB1	1427.96272	1247.32132	490.4348877	571.237722	2417.247541	980.357868
PRPSAP2	784.305103	809.539976	633.854497	834.731179	850.4197267	668.6729266
UNC45B	0	0	0	0	0	0
PCTP	326.22404	459.111755	225.6602244	216.225546	698.2711737	200.2394536
OR4D1	0	0	0	0	0	0
TOM1L1	671.005736	668.352954	2756.063541	2559.50705	2067.608151	1833.867213
KIF2B	0	0	0	0	0	0
C17orf80	888.814002	854.232271	320.9389858	303.721465	773.8416471	475.68196
TOB1	426.826065	431.686938	7517.995743	11914.5305	4193.657468	6890.592963
VPS53	2338.26453	2468.95472	892.6115543	827.691277	1991.030071	1163.382165

SPATA22	0	0	0	0	0	0
SGSM2	2666.44201	2114.75813	1112.254173	1695.61056	2272.152232	1600.103507
NPEPPS	4742.59431	4313.83234	5210.745315	6117.71464	5018.413462	4762.120056
SKAP1	2.93015605	2.03146794	0	0	8.06085049	1.812121752
LRRC46	9.76718684	12.1888077	73.21420614	58.3306125	50.38031556	73.39093097
SCRN2	800.909321	760.784745	884.5880797	802.548772	461.4836906	949.5517982
SSH2	1280.47819	1224.97517	971.8433665	1307.41028	1019.697587	1955.279371
RHBDL3	0.97671868	3.04720192	1.002934331	3.01710065	7.053244179	6.342426133
SPACA3	0	0	0	0	0	0
ARSG	27.3481231	30.4720192	3351.896797	1439.47883	320.741241	1921.057451
ABCA8	1.95343737	2.03146794	0	1.00570022	0	0
G6PC3	1206.24757	1455.54678	782.288778	767.349264	1102.321305	850.7911627
CLTC	22278.9532	23604.6418	20631.36212	20912.5303	63067.08663	26828.46254
C17orf64	0	0	0	1.00570022	4.030425245	0
BCAS3	291.062168	296.59432	560.6402909	588.334626	16714.17349	1897.291475
PTRH2	813.606664	880.641354	856.5059184	675.830545	1620.230949	1075.49426
SS18	3233.91556	3573.35211	2464.209651	2754.61289	2510.954928	3105.976684
TAF4B	636.820582	526.150198	50.14671654	35.1995075	339.5633269	169.4333838
AFG3L2	3472.23492	3394.58294	2499.312352	2890.38242	3227.363015	3293.531285
PRELID3A	129.903585	92.4317915	47.13791354	92.5244198	113.8595132	57.98789608
IMPA2	632.913707	608.424649	244.7159767	398.257285	492.7194862	322.5576719
GNAL	99.6253058	159.470234	3.008802992	6.03420129	13.09888205	7.248487009
SLC39A6	2496.49296	2722.16705	2482.262469	2265.84259	16969.09789	4922.62874
RPRD1A	2596.11826	2516.98878	1839.381563	2245.72858	2315.479303	2827.815995
C18orf21	311.43652	345.461281	218.5293613	240.80486	298.5739022	295.2943002
GALNT1	2848.11168	3397.63014	1726.049983	1998.32633	2219.756704	1969.776345
ASXL3	0	0	0	0	0	0
ADCYAP1	0	0	0	0	0	0
MEP1B	0	1.01573397	0	0	0	0
SLC25A52	0	0	0	3.17801268	0	0
GAREM	15.6274989	22.3461474	791.3151869	469.662001	485.666242	573.5365346
ESCO1	1049.97259	1077.69374	822.4061512	1149.51535	849.4121204	1130.763973
OSBPL1A	619.239646	715.036087	322.8746491	308.749966	295.2286492	314.2853361

GATA6	611.425896	452.001618	335.9830008	433.456793	274.0689167	445.7819511
GREB1L	361.385913	260.027897	75.2200748	79.450317	963.2716336	102.384879
C18orf8	898.581189	1071.59934	368.0768994	613.477131	501.787943	453.0304381
PELP1	1451.40396	1299.12375	983.8785784	846.799581	2115.973254	1566.579255
NPC1	6050.77225	8313.78256	954.7934828	2166.27826	1709.90791	1201.436722
SLC14A1	0	6.09440383	71.20833748	76.4332164	7.053244179	124.13034
ARRB2	545.009026	371.758634	303.8891022	440.496694	625.7235193	519.1728821
SLC13A5	0	0	0	0	0	0
ZMYND15	0	0	15.04401496	13.0741028	20.15212623	29.90000891
WRAP53	931.789624	863.373876	229.6719617	222.259748	485.666242	243.7303757
MINK1	3098.15167	3258.47458	1460.272386	1516.59593	3487.325443	2203.540051
SAT2	603.612147	773.989287	482.4114131	581.294725	130.9888205	232.8576452
ASGR1	13.6740616	24.3776153	3.008802992	7.03990151	4.030425245	7.248487009
PIK3R5	0.97671868	0	0	0	57.43355974	9.060608762
TP53	4251.65643	3517.48675	396.1590606	302.715765	5286.910315	832.6699452
CCDC40	21.487811	33.5192211	461.3497921	214.214146	42.31946507	481.1183253
ARHGDIA	14272.7901	14151.2057	5848.110082	6177.01072	9336.48008	6194.73821
TMC6	1595.74345	1678.98794	639.7517509	708.726999	1397.247672	971.4694108
SLC16A3	12655.344	11107.051	46.13497921	102.581422	821.1991437	192.9909666
CARD14	11.7206242	6.09440383	150.4401496	185.04884	256.9396094	546.3547083
TTYH2	22.4645297	29.4562852	28.08216126	6.03420129	47.35749663	40.77273943
RAB40B	162.135302	128.998214	348.0182128	386.188883	203.5364749	274.5364455
EIF4A3	3420.46883	3198.54628	3333.753715	2869.26271	3577.002405	4061.870908
CSNK1D	4305.37596	3501.235	5057.79783	4571.91318	4522.137125	5528.783466
ANAPC11	1307.82632	1473.06819	1273.27528	1269.19367	1228.735592	1062.256711
TBCD	3103.03526	3199.56201	4062.886974	5058.67208	2662.095874	5454.486475
FN3KRP	950.347279	831.886123	1696.964888	1833.39149	979.3933346	2100.249111
NARF	1209.17773	1122.38604	635.8603657	814.617175	1283.690441	861.6638932
RPTOR	2978.99199	3233.08123	1513.427905	1233.99416	1725.022005	1970.682406
FOXK2	3870.73614	4331.08966	3065.970249	3511.90515	3793.637762	3320.713111
TRIM65	1553.95943	1394.60274	328.9624605	469.662001	1368.329371	454.8425598
CBX8	354.548882	440.828544	668.9571986	601.408729	611.617031	664.1426222
SECTM1	377.990131	281.35831	466.3644638	342.943774	85.64653646	655.9880744

RNF157	3.90687474	2.03146794	0	0	181.369136	23.55758278
CEP131	623.14652	497.709646	760.2242227	969.495008	1146.655982	1032.003338
ZNF750	0	0	38.11150457	39.2223084	32.24340196	103.2909399
WDR45B	4504.62657	4210.21731	3080.0013	2249.74133	3711.991423	2787.025134
CBX4	551.846056	709.998047	3197.354646	1551.79543	2308.426059	2355.758278
RNF165	16.6042176	9.14160575	18.05281795	5.02850108	24.18255147	40.77273943
DYM	719.84167	821.728784	1228.594555	1453.23681	1036.826894	1227.712487
MAPK4	0	0	0	1.00570022	17.12930729	13.59091314
ELAC1	66.4168705	47.2925737	60.17605984	82.0450236	48.77822153	60.82386662
MBD1	1411.3585	1496.17614	1566.583425	1589.00634	1347.169638	2210.788538
SMAD4	1689.72332	1511.25979	1189.480116	1168.64376	921.3955153	1263.501892
TNFRSF11A	333.061071	400.199185	33.09683291	30.1710065	94.71499326	75.20305272
ZCCHC2	406.314972	417.466663	954.7934828	1109.28734	712.3776621	1985.17938
FBXO15	0	0	33.09683291	37.210908	7.053244179	34.43031329
CBLN2	0	0	10.02934331	42.2394091	0	104.1970008
PMAIP1	1686.79317	1636.34743	5.014671654	3.01710065	185.3995613	77.92123535
P3H4	345.758414	475.363499	517.5141146	414.348489	1203.081936	703.1032399
NT5C3B	1504.14677	1493.12894	475.3908728	400.268686	1148.671195	515.5486385
FAM134C	1638.93395	1575.40339	812.3768079	856.856584	998.5378545	1137.1064
ERBB2	2737.74247	2436.7458	27324.94584	25313.4744	2786.031451	27073.09898
GRB7	48.8359342	41.6450929	1983.804106	2341.2701	177.3387108	1669.870195
MIEN1	616.30949	749.611672	2075.07113	2274.89389	452.4152338	1065.52759
PNMT	3.90687474	3.04720192	8.023474646	3.01710065	0	9.060608762
ARL5C	0	0	0	1.00570022	0	0
STAC2	0	0	2.005868661	0	0	0.906060876
IGFBP4	34569.9811	18408.1468	298.8744306	171.974737	6130.276798	1070.963956
FKBP10	22.4645297	37.582157	1121.280582	964.466507	633.7843698	1642.688369
TXNL4A	1635.02708	1707.44881	1916.607506	1761.98678	1278.652409	2031.388484
CACNA1A	1.95343737	0	0	0	0	4.530304381
CTB-55O6.8	21.487811	27.4248173	461.3497921	595.374528	910.8761054	395.9486029
SAMD1	1578.37739	1295.06081	2161.323483	2449.88573	2290.289146	3214.703989
BRD4	4427.46579	4170.60369	3005.794189	2510.22774	2869.662775	3557.195
SLC39A3	978.672121	1179.17573	913.6731753	804.560172	495.7423052	893.3760239

NFIC	5772.40742	5272.67505	6186.098952	6483.74929	3768.447604	8675.532889
TPGS1	318.410291	319.956201	453.3263175	623.534134	222.6809948	398.6667855
PLPP2	818.490257	920.254979	1138.330465	1736.84427	776.864466	1186.033687
ZIM3	0	0	0	0	0	0
PRDM15	554.776212	525.134464	340.9976724	424.405491	738.5754262	657.8001961
PFKL	5239.11902	4354.45154	6821.959317	6396.25337	7250.735016	7610.91136
FEM1A	1683.86301	1751.12537	1776.056289	1568.89234	1398.55756	1720.609604
VAV1	13.6740616	25.3933493	4.011737323	11.0627024	30.22818934	14.49697402
MVB12A	932.570999	980.193441	1140.195923	1349.76032	445.3619896	970.9982592
CIB3	0	0	4.011737323	1.00570022	7.053244179	7.248487009
CTD-3222D19.2	0	0	0	0	0	0
SH3GL1	4181.33269	3962.37823	2328.813516	2542.41014	3009.720052	3124.097901
DUS3L	895.651033	847.122133	475.3908728	570.232022	807.0624272	574.3519894
DPP9	7249.20607	6902.92808	2145.276533	2368.42401	2572.418913	2723.618994
DMRTC2	0	0	0	0	0	0
CCDC97	756.95698	875.562684	1384.049376	1506.53892	1061.009446	1533.055002
TMEM91	56.3957368	41.2286419	81.48841437	58.6122086	46.86376954	42.05934587
ZFP14	137.717334	124.935279	315.9443729	331.881071	119.905151	213.8303668
SIRT3	396.547786	300.657256	750.1948794	627.556935	252.9091841	796.4275102
IFITM3	3929.87646	3483.63233	1.002934331	0	1391.635305	718.0532444
ATHL1	395.571067	411.372259	103.3022361	115.655525	192.4528055	248.2606801
HUNK	0	0	55.16138819	80.4560172	110.8366942	63.42426133
COL6A1	3266.14728	3914.63873	0	0	349.63939	67.04850484
OR1E3	0	0	0	0	0	0
IFNAR1	2213.24454	2171.63923	1983.804106	1874.6252	2020.250654	2251.561277
SOD1	3801.38912	3984.72437	6194.122426	5049.62078	3966.946048	6066.077566
COL6A2	2706.48747	2691.69503	0	0	512.8716124	95.136392
SIK1	40.9147457	78.0896278	2.477247797	1.29735328	14.92264947	8.227032756
DNMT3L	0	0	0	0	0	0
TRPM2	41.9989034	72.117112	0	3.01710065	15.11409467	4.530304381
SCYL1	3516.18726	3746.02689	3106.087622	3174.99558	3514.530814	2914.797839
TMEM50B	703.237452	884.70429	1054.083982	1227.95996	625.7235193	1142.542765
APP	17373.8719	16077.0373	15115.2233	15389.2247	6702.597183	11370.15794

DOPEY2	1355.68553	1070.58361	4043.831221	4637.28369	2334.623823	3679.513218
URB1	2825.64715	2708.9625	1235.615095	1003.68882	2743.711986	2548.749245
AKT1	7644.77714	6724.1589	14900.59535	12167.9669	14637.49688	16085.29873
IL19	0	0	0	0	0	0
EMP3	3306.19274	3507.32941	28.08216126	72.4104155	112.8519069	78.82729623
SAE1	5057.44935	5127.42509	2333.828188	3279.5884	4361.927722	3467.494973
NTN5	2.93015605	4.06293589	29.08509559	20.1140043	18.1369136	20.83940015
LMTK3	60.5565584	76.1800479	626.8339567	844.788181	391.9588551	535.4819778
GEMIN7	553.799494	596.235842	548.6050789	437.479594	528.9933134	491.0849949
CBLC	0	0	631.8486283	432.451093	2.015212623	450.3122555
WTIP	1027.50806	828.838921	45.13204488	28.159606	135.0192457	46.20910469
ADAMTS10	0	0	1.002934331	0	1.007606311	0
SLC6A3	0	0	1.002934331	0	2.015212623	0.906060876
RNPEPL1	1190.62008	1191.45595	1870.472527	1985.25223	1459.013939	1996.958171
CAPN10	365.595571	366.679964	521.9270257	476.872871	336.8730181	395.414027
MYO1F	7.81374947	14.2202756	1.002934331	18.1026039	11.08366942	4.530304381
ERVK3-1	1117.20013	1182.45655	1233.749638	968.479251	1512.346541	1503.082509
NLRP12	0	0	0	0	0	0.906060876
CACNG8	44.9290595	70.0856441	0	2.01140043	67.50962286	23.55758278
ZNF787	1335.17444	1374.28806	941.7553365	1100.23604	1201.066723	1201.436722
C19orf52	552.822775	537.323271	471.3791354	471.673401	833.2904194	564.4759259
FBN3	0	0	0	0	3.022818934	0
CARM1	3615.81257	3415.91335	1473.310532	1218.90866	4466.718778	1704.300508
EVI5L	1580.33083	1491.09747	1923.628046	1389.8777	744.621064	1620.942907
TM4SF5	0	0	0	0	0	0
SLC47A1	119.159679	118.840875	0	0	80.6085049	3.624243505
PSMB6	2598.0717	2558.63388	1044.054638	816.628575	2982.514681	1173.348835
GPR32	0	0	0	0	0	0
SIGLEC10	0.97671868	0	0	0	0	0.906060876
ACPT	0	0	0	0	1.007606311	0
KLK3	0	0	0	0	0	0
ZNF473	697.37714	597.251576	349.0211471	365.069178	688.1951106	593.4698739
FAM71E1	3.90687474	5.07866986	44.12911055	85.4845183	110.8366942	56.17577432

RPS11	11990.1986	12218.264	12499.54048	11577.6209	11819.21196	7700.65669
PTH2	0	0	0	0	0	0
CTD-2545M3.6	0	0.41645093	0	0	0	0
RPL13A	26131.3758	26211.0761	39928.12955	30139.3169	30420.12826	21615.46847
CTU1	229.528891	236.666016	236.692502	235.33385	208.5745064	260.9455323
NOSIP	1157.41164	1184.34581	948.7758769	815.622875	961.256421	1043.782129
IGLON5	0	0	0	0	0	0
RCN3	71.3004639	93.4475254	5.014671654	4.02280086	14.10648836	16.30909577
ZNF614	458.081063	494.662444	473.3850041	381.160382	442.3391707	701.2911182
SLC2A5	3.90687474	2.03146794	0	3.01710065	0	1.812121752
RERE	1885.06706	1736.90509	7129.860157	7920.8949	1675.649296	8337.572183
MMEL1	1.95343737	1.01573397	2.005868661	4.02280086	42.31946507	16.30909577
CFAP74	0.97671868	0	2.005868661	0	4.030425245	6.342426133
PRDM16	0	0	0	23.131105	266.0080662	11.77879139
CELA2A	0	0	0	0	0	0
PADI3	17.5809363	48.7552307	0	0	27.2053704	9.060608762
FHAD1	2.93015605	5.07866986	3.008802992	2.01140043	3.022818934	5.436365257
PADI1	31.2549979	42.6608268	0	0	9.068456802	2.718182629
EPHA2	10176.432	10453.934	259.7599917	140.79803	1532.569199	627.9001872
ARHGEF19	160.181864	183.847849	351.0270157	477.707602	436.2935328	995.7609029
EFHD2	6841.91438	5515.43547	801.3445302	1301.37608	1561.789782	2308.643113
PEX14	528.404808	469.269095	754.2066167	603.420129	355.6850279	706.7274834
PGD	2729.92872	3412.86615	5981.500348	8999.00553	4647.080308	6114.098792
MYOM3	1.95343737	9.14160575	0	0	0	0
SH3BGRL3	4697.04015	4675.42347	1316.852776	1701.64476	1618.215736	1866.485405
CNKSR1	26.3714045	10.1573397	629.8427597	764.332164	278.0993419	654.1759526
RPL11	9714.44403	9434.13713	12595.85226	9701.98998	11202.56697	10519.36677
IL22RA1	8.79046815	24.3776153	0	2.01140043	3.022818934	4.530304381
ZNF593	373.106537	352.459688	446.3057772	420.38269	390.9512488	488.3668123
C1orf216	545.985744	541.386207	698.0422942	652.69944	564.2595343	818.1729712
KIAA0319L	1400.61459	1406.79155	4559.339467	4632.25519	1581.941909	4378.086154
EVA1B	1.95343737	2.03146794	39.1144389	63.3591136	139.049671	59.80001783
C1orf94	0	0	0	0	0	1.812121752

DMRTA2	0	0	1.002934331	1.00570022	1.007606311	0.906060876
PLK4	1283.40835	1099.02416	530.5522609	877.976288	1068.06269	1180.597322
MAP3K6	692.493547	723.202588	830.4296258	1409.9917	606.5789994	958.612407
FCN3	0	0	0	0	0	0.906060876
GPN2	718.864951	635.849467	881.5792767	824.674177	836.3132384	1018.412425
SYTL1	76.1840573	95.4789934	1217.562277	944.352502	569.2975659	606.1547262
WDTC1	1019.69431	1094.96122	2027.933217	2064.70254	996.5226419	1800.342961
CELA3A	0	0	0	0	0	0
NBPF3	490.009997	411.981699	393.9526051	397.40244	466.5922546	480.9733555
HSPG2	4860.15217	3002.50962	94.27582709	80.4560172	2429.338817	673.203231
ITGB3BP	333.061071	381.915974	331.9712635	310.761367	332.5100827	237.3879496
SERBP1	13194.1118	12332.788	6271.217988	7014.55786	14760.04196	11076.99288
BCL10	782.351666	795.3197	579.6960431	465.6392	492.7194862	490.178934
CYR61	29898.3356	21781.3993	700.0481628	1534.69853	1415.686867	1471.442863
PRKACB	584.077773	572.87396	927.7142559	726.115556	606.5789994	1327.379184
PIGK	1156.43492	1139.65352	984.8815128	811.600074	617.6626688	1014.788181
TINAGL1	3136.24369	2398.14791	77.22594346	49.2793106	72.54765441	88.79396587
AZIN2	184.599831	148.29716	18.05281795	11.0627024	10.07606311	12.68485227
RPS8	13465.0438	13101.9424	22399.52531	17235.9317	20100.72823	15296.11065
KIF2C	3333.54087	3316.37142	960.8110888	1561.85243	1877.170558	2048.603641
PTPRF	14604.8745	10735.2924	18033.7622	19871.6306	14095.40469	23325.6312
BEST4	1.95343737	2.03146794	2.005868661	7.03990151	6.045637868	2.718182629
MOB3C	250.039983	277.295374	158.4636243	181.026039	274.0689167	258.2273497
CYP4B1	0	0	33.09683291	389.205983	14.10648836	115.9757922
TMEM61	9.76718684	5.07866986	0	0	5.038031556	2.718182629
DMRTB1	0	0	0	0	0	0
LMO4	545.009026	627.723595	1615.727207	1029.83702	287.1677987	1115.360939
SYPL2	51.7660902	101.573397	0	0	14.10648836	0.906060876
BARHL2	1.95343737	0	0	0	0	0
MTF2	1207.22429	1005.57663	691.0217539	575.260523	629.7539446	851.6972236
SLC44A3	3.90687474	11.1730737	13.0381463	23.131105	23.17494516	22.6515219
IGSF3	1024.48023	1164.98592	1812.212072	2485.08523	4982.522525	3070.604067
ZNF697	794.07229	1005.57663	97.28463008	75.4275162	182.3767423	140.4394358

CTTNBP2NL	1629.16676	1373.27233	255.7482543	369.091979	622.7007004	480.2122644
STRIP1	1387.91725	1326.54857	972.8463008	900.101693	1088.214816	1315.600392
KCNA10	0	0	0	0	0	0.906060876
PSMA5	2822.717	2900.93622	2513.353433	1874.6252	3481.279806	2377.503739
FNDC7	0	0	1.002934331	1.00570022	0	0.906060876
C1orf162	1.95343737	2.03146794	27.07922693	18.1026039	5.038031556	21.74546103
CD53	0	0	0	2.01140043	1.007606311	0
PROK1	0	0	1.002934331	0	0	0
CELSR2	1359.59241	1333.65871	1733.070523	1987.26363	15516.12959	3223.764597
ITGA10	99.6253058	137.124086	37.10857024	137.78093	16.12170098	54.36365257
GPR161	855.605567	945.648328	9.026408976	34.1938073	51.38792188	28.08788716
ALDH9A1	2110.68908	1868.95051	3194.375931	2994.97524	1236.332944	2882.179647
ATP1B1	4237.98237	3583.50945	2532.409185	3287.634	2747.742411	2710.934142
TIPRL	1279.50148	1364.13072	2016.900939	1759.97538	1151.694014	1485.939837
NME7	822.397132	871.499748	349.0211471	393.228784	227.7190264	462.0910469
POGK	2096.0383	2021.3106	3440.064754	3539.05906	3673.732611	4494.968007
MPC2	815.560101	1028.93851	1939.674996	3541.07046	838.328451	4304.695223
CREG1	1533.44833	1274.74614	3060.955577	2341.2701	1693.786209	1792.188413
DCAF6	1593.02817	1587.5922	2166.338154	3461.62014	1345.154426	4938.937836
GPA33	0.97671868	0	0	0	0	1.812121752
RXRG	0	2.03146794	0	0	0	0.906060876
TBX19	30.2782792	17.2674775	4.011737323	19.1083041	18.1369136	36.24243505
UCK2	2567.79342	2337.20387	1561.568753	1663.42816	1582.949515	2559.621975
TMCO1	1307.82632	1458.59398	3304.66862	2538.38734	1868.102101	3078.794857
XCL1	0	2.11272666	0	0	0	0
XCL2	0	1.95020923	0	0	0	0
POU2F1	1319.54694	1125.43324	1654.841646	1744.88987	1828.805455	2037.730911
MAEL	25.3946858	10.1573397	0	0	0	0.906060876
ILDR2	732.539013	531.228867	0	4.02280086	32.24340196	25.36970453
DPT	0	0	0	0	0	0.906060876
MGST3	696.400422	946.664062	1205.527066	1810.26039	297.2438618	953.1760417
ADCY10	0	0	10.02934331	8.04560172	2.015212623	18.12121752
RFWD2	721.795107	750.627405	1671.891529	1536.70993	1553.728932	1677.118682

PVRL4	0	0	2404.033591	2584.64955	406.0653434	2193.573381
UFC1	846.815099	868.452546	1355.967215	2249.75138	1302.83496	1533.055002
PPOX	259.80717	238.697483	672.9689359	589.340326	131.9964268	570.818352
FCGR2A	1.95343737	11.8942448	5.014671654	15.0855032	2.015212623	3.624243505
NUF2	1403.54475	1344.83178	750.1948794	1200.80606	596.5029363	1066.433651
RGS5	18.4111472	38.628363	12.37620964	8.35736879	8.242219626	20.19609693
SDHC	2256.21039	2497.67968	3867.274662	4218.9124	2141.163411	4059.152725
PFDN2	1204.29414	1074.64654	1166.412627	823.668477	881.6555224	1013.88212
NR1I3	0	0	3.008802992	3.01710065	0	3.624243505
USP21	403.384816	434.73414	1156.383283	1000.67171	499.7727304	1170.630652
F13B	0	3.04720192	14.04108063	1.00570022	0	2.718182629
PRCC	1444.56693	1510.39642	2808.216126	3347.97602	2835.40416	3565.349548
FCRL5	0	0	0	0	0	0.906060876
RRNAD1	260.783889	249.870557	1237.620964	1305.39888	338.5557206	868.9123803
MRPL24	1049.97259	1090.89829	1595.66852	1053.97383	1325.002299	1482.315593
PIGM	232.459047	237.68175	705.0628345	787.463269	886.6935539	839.9184322
CASQ1	0	0	0	1.00570022	1.007606311	0.906060876
ISG20L2	807.746352	873.531216	1551.53941	1478.34915	2786.031451	2454.518914
CRABP2	65.4401518	37.582157	21376.54232	17905.4866	11290.22872	10225.80305
HDGF	10151.0373	9844.49366	13255.78305	13685.5685	16478.39361	15653.1077
ABL2	6213.88427	5740.92841	1398.090457	1057.99663	2158.292719	1768.63083
XPR1	1154.48148	1318.4227	988.8932501	1972.17812	1978.938795	2497.103775
RGS16	6.83703079	5.07866986	112.328645	55.3135119	719.4309063	251.8849236
TOR1AIP1	2073.57377	2010.13753	1303.81463	1770.03238	1944.680181	2074.879406
FAM163A	0	0	1.002934331	2.01140043	1.007606311	0.906060876
HMCN1	48.8359342	58.9125704	0	0	8891.118091	187.5546014
RGL1	189.483425	318.940467	17.04988362	18.1026039	34.25861458	64.33032221
LYPLAL1	271.527794	328.082073	604.7694014	487.764605	197.490837	491.9910558
LHX9	15.6274989	18.2832115	0	0	2.015212623	1.812121752
PRUNE	306.689667	273.232439	1065.116259	1191.75476	1381.428253	1427.951941
RORC	0	0	511.4965087	232.31675	29.22058303	182.1182361
TUFT1	800.909321	601.314512	972.8463008	1301.37608	1506.371435	3163.058519
SF3B4	2534.58498	2485.50103	2928.568246	2530.34174	6210.885303	3347.894937

ECM1	2008.13361	2142.18295	49.1437822	29.1653063	91.69217433	100.5727573
ZNF687	955.230873	894.86163	2139.258927	2411.66912	3202.172857	2978.2221
TARS2	779.42151	714.060982	1405.110997	1138.45264	1447.930269	1648.124734
CGN	164.088739	167.596105	1021.990083	1520.61873	2619.776409	1740.542943
SNX27	1079.27415	1060.42627	1409.122735	2054.64554	2848.503042	2174.546103
SETDB1	646.587769	685.620431	1639.797631	1651.35975	2256.030531	1949.843006
ADAMTSL4	1088.06461	493.646711	2.005868661	1.00570022	22.16733885	3.624243505
MCL1	6980.60843	7674.88589	5498.086001	8132.09194	9270.98567	6983.917234
CTSK	41.9989034	42.6608268	33.09683291	43.2451093	71.5400481	38.96061768
RFX5	1347.87178	1643.45757	2287.693208	6898.09778	988.4617914	2619.421993
PI4KB	1619.39958	1700.33867	3099.067082	2975.86694	5219.400692	3905.122376
PIP5K1A	2456.41819	1957.26858	2818.23544	2950.70432	4018.333969	3259.961729
ANP32E	4329.79393	3836.42721	1071.133865	1943.01282	2438.407273	2050.415763
FAM63A	252.970139	303.704458	1299.802893	1253.10247	659.9821339	1272.10947
ANXA9	2.93015605	4.06293589	1400.096326	1496.48192	812.1306869	1200.530661
SELENBP1	36.1385913	53.8339005	6857.062019	6646.67272	990.477004	5939.229043
CERS2	4631.6	4756.68219	10102.55751	15499.8517	14963.96133	10694.23652
ENSA	2512.11069	2928.33057	7388.486833	13465.0587	8656.154375	8516.772903
AC027612.6	323.606434	282.069324	0	0	250.490929	30.80606979
SEMA6C	144.554365	115.793673	949.7788112	551.123718	35.2662209	406.8213334
MRPL9	1109.53289	1160.96362	1727.9355	1982.1245	3102.419832	1753.218735
ARNT	963.044622	907.050437	1484.342809	2102.91915	2308.426059	1835.679335
POGZ	2451.5639	2250.86648	5080.865319	4773.05322	4628.943394	4398.925554
C1orf56	95.718431	82.2744518	82.24061512	82.4674177	434.2783202	121.4121574
OAZ3	9.76718684	4.06293589	266.7404146	319.420445	51.38792188	263.5549877
HORMAD1	3.90687474	0	0	0	0	0
GOLPH3L	402.408098	482.473637	1327.885054	1167.61795	968.3096651	1181.503383
GABPB2	314.503416	487.552307	803.3503989	716.058553	1200.059117	552.6971345
SYT14	1.95343737	2.03146794	0	0	0	1.812121752
KCNH1	47.8592155	58.9125704	0	0	19.14451991	4.530304381
DTL	1850.88191	1543.91564	999.9255277	1370.76939	824.2219626	2538.782575
DYRK3	295.945761	405.277855	18.05281795	12.0684026	42.31946507	45.30304381
EIF2D	1239.45601	1347.87898	3213.401596	2705.33358	1297.796929	2040.449093

INTS7	1173.03914	1302.17095	1857.43438	2020.45173	764.7731903	2231.627938
VASH2	30.2782792	45.7080288	108.3169077	617.499932	33.25100827	261.8515932
TAF1A	595.798397	509.898454	273.8010723	249.413653	175.3234982	393.2304203
SMYD2	1283.40835	1281.85627	515.508246	974.523509	706.3320242	1130.763973
SUSD4	35.1618726	26.4090833	447.3087115	633.591136	186.4071676	650.5517091
DUSP10	592.868241	804.461306	777.2741063	722.092755	72.54765441	1408.924662
HHIPL2	7.81374947	7.11013781	6.017605984	7.03990151	23.17494516	18.12121752
TP53BP2	2274.77781	2218.363	674.9748046	948.375303	935.0586569	1057.373042
ATP8B2	473.708562	439.81281	15.04401496	35.1995075	943.1195074	405.0092117
FLG2	0	0	0	0	0	0.906060876
CRNN	0	0	0	0	0	0
ADAM15	2827.60059	2860.30687	5817.019118	5544.42529	5211.339842	10260.97633
JTB	1692.65348	1813.08514	6418.779717	4635.27229	4304.494162	5261.495508
RAB13	1135.88476	1478.57347	2458.924187	3998.19138	1707.177297	3725.214929
S100A8	0	0	2.005868661	1.00570022	79.60089859	52.55153082
TPM3	10485.0751	9987.69183	6708.557533	7509.55345	11518.92512	9759.082031
NUP210L	0	0	7.020540315	0	301.2742871	32.61819154
SNAPIN	478.592155	556.622217	907.6555693	1073.08213	732.5297883	809.1123624
SLC27A3	68.897736	90.1057607	534.8147319	1147.61457	1200.210258	1266.618741
S100A7	0	0	0	0	27.2053704	9.060608762
UBAP2L	4749.78296	5206.65234	7567.139525	6032.18989	6180.657113	8457.172218
SLC39A1	3862.92239	3762.27863	5650.532019	9223.27668	4942.308957	5134.646985
HAX1	1221.87507	1153.87379	3927.490839	2466.98263	2513.977747	2220.755208
CREB3L4	118.182961	127.982481	5752.831321	5979.89348	451.4076275	3885.189037
EFNA3	78.1374947	74.14858	1102.806531	717.064254	259.2974081	1714.267178
AQP10	0	0	0	0	0	0
KCNN3	54.6962463	170.643307	2.005868661	0	0	2.718182629
C1orf43	3062.98979	3574.36785	7678.465236	8573.59434	6185.695145	6423.971612
GATAD2B	1461.17115	1316.39123	2679.840532	3086.49396	1428.785749	3619.7132
ILF2	5150.23762	4858.25559	5971.471005	5289.98313	7174.156936	7201.371844
RIT1	590.914804	661.242816	1100.218961	906.135894	891.7315855	914.2154241
INTS3	1929.46869	2385.23793	4250.18496	4037.77574	2777.819459	4360.0193
PKLR	0	0	2.005868661	0	0	0

HCN3	174.832644	166.580371	510.4935743	794.50317	182.3767423	482.0243861
FLG	248.086546	241.744685	0	1.00570022	5.038031556	2.718182629
ACTA1	4.88359342	3.04720192	13.0381463	6.03420129	1.007606311	18.12121752
C1orf131	179.716238	197.052391	741.1684704	775.394866	563.251928	702.197179
GALNT2	6355.50848	5641.38648	1586.642111	1978.21232	2467.627856	2333.106756
TTC13	953.277435	886.735758	1019.984214	773.383466	612.6246373	994.854842
SCCPDH	1703.39738	1650.5677	1478.325203	1806.23759	2393.064989	1628.191394
LYST	903.464783	790.24103	1326.88212	3246.4003	334.5252953	1209.59127
RP5-862P8.2	894.674314	752.658873	13.0381463	13.0741028	669.0505907	53.45759169
CEP170	5286.17733	4951.4695	1740.311709	1943.98835	833.8849072	1762.496798
ACP1	1855.7655	1819.17954	1812.302336	1571.90944	2605.669921	1734.200517
SNAP47	1014.81071	908.066171	1985.809975	2200.47207	754.6971272	2110.215781
SRP9	4853.8328	5249.12018	9537.163314	8234.28114	4761.755982	9283.400071
NVL	1093.92493	1047.22173	1942.683799	1925.91591	826.2371753	2805.164473
SDE2	990.392745	961.900072	940.7524022	1030.84272	627.7387319	994.854842
DEGS1	4907.03467	5205.63661	2005.868661	2170.30107	1131.541888	3073.358492
FBXO28	2915.50527	2773.96948	2589.576442	2363.39551	1381.428253	3120.473658
ARF1	11305.5188	11502.1715	27074.21226	19955.1037	10687.68014	16815.5838
LEFTY2	1.04508899	0	0	0	0	0
CNIH4	1468.9849	1520.55376	1531.480723	1546.76693	788.9557417	1167.912469
ITPKB	271.527794	336.207945	1987.815843	2369.42971	568.2899596	1799.4369
GUK1	2555.09608	2447.91887	6121.911155	5975.87068	2280.213082	6119.535158
CDC42BPA	6860.47204	5617.00887	5843.095411	5359.37645	2089.77549	5387.43797
CNIH3	284.225137	343.318083	43.12617622	46.2622099	23.17494516	98.7606355
C1orf35	588.961366	575.921162	1165.409692	1051.96243	880.6479161	1109.924573
MBOAT2	1588.14458	1463.67265	2617.658603	3090.51676	1154.716833	2138.303668
PARP1	10200.8499	9507.26998	10454.58746	10816.3058	7205.392732	17161.69906
PSEN2	779.42151	864.38961	725.1215211	676.836245	274.0689167	682.2638398
PYCR2	2350.96187	2159.45042	3091.043607	2484.07953	1519.470317	2498.915896
LBR	6050.77225	5140.62963	2871.400989	4909.82845	1322.987087	4225.867926
WNT9A	155.298271	161.501702	120.3521197	233.32245	92.69978064	65.23638308
EPHX1	1014.81071	1108.16576	6680.545577	4079.12007	697.2635674	11286.80033
REN	3.90687474	5.07866986	95.27876142	58.3306125	3.022818934	103.2909399

SOX13	811.653226	822.744518	6702.610132	6515.9317	1233.310125	5920.201765
ETNK2	637.797301	588.10997	1792.243649	1413.0088	334.5252953	2281.461286
PPFIA4	344.781695	248.854823	36.10563591	21.1197045	268.0232788	78.82729623
PLEKHA6	739.376044	753.674607	5901.265602	7750.93156	1015.667162	6286.250359
PTPN7	0	0	0	0	4.030425245	0.906060876
SYT2	25.3946858	29.4562852	2.005868661	3.01710065	1.007606311	1.812121752
ARL8A	543.055588	577.95263	3187.325303	2674.15687	1363.291339	1711.548995
OSR1	211.947954	122.903811	0	0	3.022818934	0
GDF7	10.7439055	17.2674775	0	0	7.053244179	0
PDIA6	9977.18136	10592.0739	5988.520889	5422.73556	9109.76866	5245.186412
RHOB	1008.9504	1345.84751	15497.34128	16032.8728	5551.910775	9731.09381
ATP6V1C2	10.7439055	13.2045416	6.017605984	1.00570022	30.22818934	10.87273051
HNRNPLL	1170.10898	1151.84232	642.880906	1021.79142	1422.740112	901.5305718
GALM	286.178574	415.435195	115.337448	192.088741	257.9472157	210.2061233
CAMKMT	113.299367	104.620599	122.3579883	123.701127	144.0877025	144.9697402
ABCG8	0	0	0	0	0	0.906060876
EML4	4055.33598	3672.89404	869.5440647	1182.70345	2911.98224	1721.515665
CALM2	17074.9863	15368.0448	10176.77465	9281.60729	9060.395951	9398.569469
CHAC2	333.061071	340.270881	89.26115543	110.627024	269.0308851	130.4727662
RPS27A	14809.6338	15418.4963	12799.25737	10604.9177	17300.13687	9356.646032
WDPCP	79.1142134	93.4475254	106.3110391	114.649825	157.1865846	93.32427025
VPS54	992.346183	1109.1815	657.9249209	499.833007	954.2031768	491.9910558
REG3G	0	0	0	0	0	0
ASXL2	3394.09743	3200.57775	503.473034	582.300425	2921.050696	868.9123803
ETAA1	641.704175	507.866986	483.4143474	498.827307	375.8371541	479.3062035
SNRPG	2025.74385	2006.13554	866.9865822	1332.92489	1838.659845	907.2568765
ABHD1	12.6973429	9.14160575	30.08802992	32.1824069	11.08366942	31.71213067
MEIS1	4.88359342	6.09440383	309.9067082	283.607461	164.2398287	239.2000713
TRIM43B	0	0	0	0	0	0
TRIM43	0	0	0	0	2.015212623	0
CIAO1	2451.5639	2506.83144	2925.559443	2602.75216	3257.591204	3536.3556
ZNF514	1093.92493	965.963008	895.6203573	656.722241	704.3168116	548.1668301
SNRNP200	13242.3519	12750.5086	13318.96791	13573.9358	19157.6188	15245.3803

MRPS5	1963.20455	1827.30542	1325.879185	1323.50148	1896.315078	1415.267089
ANKRD53	5.8603121	18.2832115	0	0	0	0.906060876
TPRKB	678.819485	802.429838	307.9008395	292.658763	637.814795	384.1698115
NAT8	0	0	0	0	0	0
EXOC6B	1093.92493	1299.12375	498.4583624	569.226322	1446.922663	824.5153973
SFXN5	415.105441	393.089047	508.4877057	665.773543	1193.005873	773.7759883
TEX261	3485.70387	3805.4778	1881.504804	1829.36869	4566.360966	2504.352262
DQX1	5.8603121	13.2045416	159.4665586	59.3363127	8.06085049	47.11516556
DUSP11	762.817292	791.256764	384.1238487	369.091979	600.5333615	406.8213334
ST6GAL2	0	0	0	0	0	0
NPHP1	70.3237452	102.589131	56.16432252	17.0969037	41.31185876	57.0818352
MALL	253.946858	328.082073	0	0	241.875895	135.9091314
THNSL2	0.97671868	3.04720192	393.1502576	260.476356	306.3123186	251.8849236
RALB	1510.9838	1583.52926	1124.289385	1370.76939	3058.085155	1383.554958
C1QL2	0	0	0	0	0	0
TMEM177	321.340447	342.302349	391.144389	284.613161	333.517689	418.6001248
NT5DC4	1.95343737	2.03146794	0	3.01710065	0	0.906060876
RABL2A	97.7890746	107.911577	281.5136373	142.970343	151.2820116	188.5965714
SLC20A1	7446.50325	10830.7713	1682.923807	3086.49396	2309.433665	2440.02194
FBLN7	162.135302	84.3059197	0	2.01140043	16.12170098	13.59091314
RP11-395L14.17	3.90687474	8.17665848	1.012963674	1.00570022	18.23767423	6.387729177
ZC3H8	323.293884	294.562852	405.1854696	304.727165	616.6550625	450.3122555
LIPT1	93.7649937	141.644102	82.91258112	77.4389166	91.69217433	75.20305272
TRIM43CP	0	0	0	0	0	0
CNGA3	0	0	0	0	2.015212623	0.906060876
FAHD2B	268.685543	305.624195	177.9305796	52.4372092	47.66985459	153.7766519
LYG1	11.7206242	4.06293589	47.13791354	77.4389166	5.038031556	45.30304381
AFF3	721.795107	760.784745	1000.928462	458.599298	296.2362555	610.6850305
UBXN4	4083.66082	3838.45868	5949.40645	4017.77236	4633.981426	4045.561812
NXPH2	0	0	0	0	0	0
SPOPL	1193.52093	1144.26495	1519.425452	1924.39731	2301.826238	1940.338427
THSD7B	3.90687474	2.03146794	0	0	4.030425245	1.812121752
GPR17	95.718431	147.281426	0	0	3.022818934	2.718182629

POLR2D	2050.99203	1951.8344	1437.465659	1101.14117	1968.570526	1777.673318
AMMECR1L	1513.53304	1365.30898	1151.368612	1014.12798	1208.119967	1142.089734
GALNT13	249.063264	313.861797	2.005868661	2.01140043	5.038031556	2.718182629
PKP4	5410.04479	4066.99882	3215.407464	3483.74555	4247.060602	5653.819867
SCN1A	0	0	4.011737323	0	0	13.59091314
SLC4A10	0	1.01573397	4.011737323	2.01140043	260.9700346	11.77879139
SCRN3	436.593252	456.064554	723.1156524	616.494232	500.7803367	601.6244218
KIAA1715	2333.38094	2256.96089	1029.010623	1032.85412	1811.676148	1311.976149
ZNF385B	28.3248418	29.4562852	1.002934331	0	23.17494516	0.906060876
TMEFF2	0	0	0	0	0	0
CDCA7	1429.91615	1257.47866	413.2089443	687.898947	480.6282105	1303.821601
DLX1	20.5110924	13.2045416	45.13204488	80.4560172	172.3006792	169.4333838
UBR3	1791.30207	1702.37014	1088.183749	1497.48762	3266.659661	1792.188413
PHOSPHO2	105.915374	106.804427	158.3432721	204.820906	86.39216513	169.4515051
GULP1	873.186503	820.71305	28.08216126	121.689726	476.5977852	101.4788181
FAM171B	641.704175	736.40713	53.15551953	110.627024	610.6094246	120.5060965
HSPD1	15640.0693	16571.5677	18775.60264	10319.3994	24992.40497	19153.87323
CCDC150	291.062168	320.971935	162.4753616	184.043139	125.9507889	304.4364544
METTL21A	298.875917	283.389778	374.5658845	342.088928	515.9649638	303.0501813
UNC80	0	0	0	0	0	0
PTH2R	0	0	0	0	0	0
CPO	0	0	1.002934331	4.02280086	0	3.624243505
NBEAL1	881.537448	688.403542	777.9761603	1048.09048	1216.755153	1303.903146
KANSL1L	174.832644	231.587346	99.29049874	146.832231	127.9660015	74.29699185
SPAG16	232.459047	211.272666	409.1972069	219.242647	9.068456802	195.7091493
ABCA12	0	0	9508.820389	6089.51481	366.7686973	12925.86446
SUMF1	422.91919	399.183451	577.6901745	759.303663	702.301599	618.8395784
NYAP2	3.90687474	9.14160575	0	0	9.068456802	0
RHBDD1	454.174188	474.347765	585.7136491	262.487756	533.0237387	456.6546816
ACKR3	0.97671868	1.01573397	6.017605984	15.0855032	1907.398747	121.4121574
TRPM8	5.8603121	0	0	0	1.007606311	0
HES6	219.761704	229.555878	707.0687031	2481.06243	476.5977852	791.8972058
ESPNL	0	0	0	0	2.015212623	4.530304381

ANKMY1	358.455757	254.949227	240.7042394	235.33385	339.5633269	324.3969755
COPS7B	766.724167	773.989287	807.3621362	731.144057	738.5754262	841.730554
DIS3L2	692.288436	713.715633	470.6971401	405.779923	555.3321424	432.1819773
CPNE9	2.93015605	3.04720192	2.005868661	5.02850108	0	0.906060876
FANCD2	1425.03256	1263.48165	771.2364416	1185.65016	2479.719132	2390.088925
TAMM41	230.505609	235.650282	238.6983707	185.04884	444.3543833	293.5637239
VGLL4	813.606664	953.7742	1781.211371	2256.79128	1918.482417	1877.358135
RAB5A	2275.75453	2128.97841	1429.181421	1119.34434	2269.129413	1345.500401
FAM134A	1590.09802	1831.36835	1894.542951	3463.63154	2084.737458	2510.694688
CTDSP1	1663.35192	1478.90866	3166.263682	3813.61522	2579.472157	3492.864678
RQCD1	1801.06925	1832.38409	1930.648587	1843.4485	2050.478843	2057.66425
MARCH4	945.463686	1046.20599	2.005868661	0	37.28143352	17.21515665
STK11IP	383.850443	337.223679	304.8920365	439.490994	660.9897402	392.3243594
GMPPA	713.981358	813.602912	1701.979559	1166.61225	847.3969078	952.2699809
GRIP2	0	0	0	0	0	8.154547886
EAF1	839.978068	890.798694	1478.325203	1376.8036	2747.742411	1635.439882
CNTN4	0	0	48.14084787	40.2280086	0	18.12121752
DYNC1LI1	2002.2733	2227.5046	1155.380349	1733.82717	1298.804535	1328.285244
RBMS3	774.537916	578.968364	0	0	18.1369136	8.154547886
GADL1	0	0	0	0	0	0.906060876
OSBPL10	1905.57815	2059.9085	382.11798	869.930686	1204.089542	897.0002674
POMGNT2	752.073387	637.880935	630.845694	458.599298	416.1414066	563.569865
ACKR2	1.95343737	0	1.002934331	2.13208446	17.12930729	38.96061768
FAM198A	7.81374947	4.06293589	0	0	6.045637868	0
CSRNP1	493.242935	622.644925	578.6931088	671.807744	379.8675794	681.3577789
SLC25A38	802.862758	921.270713	1210.541737	1423.06581	1013.651949	1012.060938
ITGA9	2.93015605	0	2.005868661	6.03420129	4.030425245	6.342426133
SLC22A14	0	1.01573397	1.002934331	0	0	0.906060876
GOLGA4	6334.02066	6588.05054	10221.9067	9781.4403	4206.75635	6703.944423
CTDSPL	2129.24673	2025.37354	2756.063541	3032.18615	295.2286492	2384.752226
STAC	377.990131	341.286615	0	0	62.4715913	30.80606979
IQSEC1	1797.16238	1806.99074	1195.497722	1410.9974	2836.411766	1704.300508
CAND2	8.79046815	12.1888077	0	1.00570022	4.030425245	0.906060876

RPL32	12012.6533	12059.779	11272.97185	9331.27882	19341.9503	8886.880649
PTPRG	5450.09026	4294.52323	482.4114131	477.707602	1624.261374	655.0820135
IL17RD	1381.08022	1279.82481	37.10857024	45.2565097	93.70738695	64.33032221
SHQ1	551.846056	594.204374	296.8685619	325.84687	698.2711737	520.0789429
SLC25A26	331.107634	325.034871	169.4959019	144.820831	254.9243968	147.6879228
UBA3	1935.85643	1803.94353	1180.453707	1275.22787	1101.313698	1271.203409
ARL6IP5	1554.93614	1519.53802	3157.237273	3235.33759	1921.505236	2648.415941
TMF1	1812.78988	1855.74597	2009.880399	1807.24329	2205.650215	1731.482334
LRIG1	2336.31109	2119.8368	5875.189309	5667.12071	516.9020377	13746.75561
LRTM1	0	0	0	0	0	0
RP11-977G19.10	235.46734	210.328034	104.265053	44.8341156	244.1631614	161.7046846
LIMD1	2782.67153	2640.90833	2572.526558	2098.89635	1371.35219	2479.888618
ZNF660	35.1618726	33.5192211	0	0	4.030425245	0
NFKBIZ	1582.28427	1753.15684	92.26995843	113.644124	737.5678199	407.7273943
COL8A1	4208.68081	3184.326	0	0	46.34989032	19.93333928
NXPE3	1929.0194	2487.5325	1690.947282	2131.07876	516.9020377	1591.948959
ADGRG7	0	0	0	0	0	0
MYH15	42.9756221	183.847849	6.017605984	6.03420129	75.57047335	43.49092206
PHLDB2	3375.53977	2059.9085	48.14084787	785.451868	290.1906177	274.5364455
ABHD10	1038.25196	1124.41751	784.2946466	665.773543	551.1606523	719.4123357
TAGLN3	24.4179671	45.7080288	0	0	5.038031556	0
PLA1A	0	0	0	0	0	0
RABL3	453.197469	439.81281	1849.410906	2355.3499	756.7123398	2884.89783
ADPRH	25.3946858	25.3933493	2.005868661	0	41.31185876	0.906060876
IGSF11	0	0	0	1.00570022	0	2.718182629
ATG3	1050.9493	1305.21815	1422.160881	1218.90866	1505.363829	1364.52768
NR1I2	0	0	0	0	1.007606311	3.624243505
BOC	96.6951497	73.132846	81.23768079	240.362352	22.16733885	151.3121663
SRPRB	774.537916	1015.73397	1319.861579	827.691277	1875.155345	1630.003516
TMEM108	0	0	4.011737323	1.00570022	3.022818934	11.77879139
AGTR1	0	0	0	0	3.022818934	3.624243505
MED12L	87.9046815	107.667801	137.4020033	184.043139	1083.176785	361.5182896
EIF2A	2221.05829	2084.28611	2725.975511	2391.55511	2560.327637	2063.100615

ALDH1L1	71.3004639	70.0856441	2.005868661	0	1.007606311	2.718182629
OSBPL11	632.913707	692.730569	478.3996757	673.819144	899.792436	655.0820135
TRPC1	262.737326	298.625788	0	1.00570022	193.4604118	12.68485227
NCEH1	5595.62134	6140.11186	1252.664979	1571.90944	924.9825938	1040.157886
SPATA16	0	0	0	0	0	0
FAM86B2	10.3922868	4.64190425	3.951561263	3.61046377	13.81428253	5.237031864
LPP	5874.96288	4604.3221	4239.403416	3104.59657	4602.74563	3606.122287
TMEM44	632.913707	611.471851	347.0152784	403.285786	157.1865846	283.5970542
RUBCN	1507.07693	1523.60096	1376.025902	1823.33449	1086.199604	1707.018691
AMT	152.368115	151.344362	300.8802992	174.991838	19.14451991	87.88790499
TCTA	779.42151	718.123918	1991.827581	1215.89156	577.3584164	965.860894
NICN1	341.851539	321.987669	472.3820698	418.37129	84.63893015	252.7909845
UCN2	164.489194	278.179063	5.566285535	20.8079375	23.17494516	15.85606533
VPRBP	2057.94627	2068.03437	921.6966499	1128.39564	1113.404974	1229.524609
MANF	1906.55487	2348.37694	2169.346957	1502.51612	935.0586569	1294.760992
AC062028.1	0.97671868	0	1.002934331	0	2.015212623	0
CCDC39	0	3.04720192	2.005868661	1.00570022	0	0
STXBP5L	0	1.01573397	12.03521197	38.2166082	1.007606311	30.80606979
EAF2	6.83703079	12.1888077	607.7782044	377.137581	43.32707139	414.9758813
ILDR1	0	0	148.4342809	189.071641	261.9776409	206.5818798
TM4SF19	162.135302	417.466663	0	0	15.11409467	1.132576095
MUC4	484.452467	239.713217	8.023474646	0	1.007606311	13.59091314
SLIT2	698.353859	802.429838	23.06748961	41.2337088	372.8143352	39.86667855
EIF2B5	2251.33657	2226.48887	3670.73965	4121.35948	1966.84752	4337.313414
AHSG	0.97671868	0	0	0	0	0
ECE2	223.668579	236.666016	530.5522609	324.84117	396.9968866	369.6728375
VWA5B2	10.7439055	17.2674775	3.008802992	4.02280086	21.15973254	18.12121752
DGKQ	359.432476	349.412486	722.1127181	678.847645	391.9588551	594.3759348
FIP1L1	1030.43821	946.664062	1293.785287	731.144057	1123.481037	1165.194287
SLC26A1	1.95343737	3.04720192	152.4460183	215.219846	106.806269	141.3454967
LYAR	1710.23442	1527.66389	288.8450872	222.259748	953.1955705	555.4153171
CENPC	710.074483	556.622217	714.0892435	730.138356	461.4836906	768.339623
EPHA5	9.76718684	13.2045416	35.10270157	65.370514	0	15.4030349

CORIN	0.97671868	0	3.008802992	1.00570022	1.007606311	7.248487009
ATP10D	795.049009	1023.85984	24.07042394	55.3135119	36.27382721	85.16972236
OCIAD2	839.001349	1016.74971	413.2089443	460.610699	639.8300077	479.3062035
SLC10A4	0	0	1.002934331	4.02280086	3.022818934	3.624243505
SLC10A6	0	0	1.002934331	1.00570022	0	0
SCD5	611.425896	593.18864	264.7746633	302.715765	252.9091841	549.072891
PLAC8	1546.14568	945.648328	0	0	89.6769617	28.99394804
ENOPH1	1280.47819	1368.19366	1669.885661	1748.91267	1923.520448	1602.82169
CABS1	0	0	0	0	0	0
GC	0	0	0	0	0	0
TRMT10A	113.299367	117.825141	186.5457855	177.003238	220.6657822	112.3515486
KLHL8	709.097764	742.501534	596.7459268	656.722241	989.4693977	745.6881011
SNCA	0	0	0	0	0	0
PYURF	2129.79369	2217.3371	1397.58899	1092.37146	1612.895575	1254.459404
TBCK	200.22733	176.737711	386.1297173	461.616399	491.7118799	509.2062124
CAMK2D	793.095571	757.737543	2214.479002	2480.05673	600.5333615	2773.452342
CISD2	810.139312	946.897681	979.2149338	772.629191	804.0698364	527.5086421
DDIT4L	13.6740616	23.3618814	3.008802992	0	8.06085049	0.906060876
ANK2	405.338254	546.464877	0	0	25.19015778	13.59091314
TIFA	408.26841	412.387993	277.8128096	319.812669	474.5825726	312.5910023
SPATA5	613.379333	600.298778	221.6484871	295.675863	361.7306658	366.9546549
FABP2	0	0	0	0	0	0.906060876
CCNA2	3132.33682	3220.89243	624.828088	1617.16595	2351.753131	1835.679335
METTL14	657.331674	693.746303	863.5264587	654.71084	683.1570791	666.8608049
USP53	772.584479	807.508508	1056.08985	1482.40212	744.621064	1485.939837
SETD7	4031.89473	4084.2663	7187.027414	5388.54175	6375.125132	7253.923375
NAF1	408.26841	358.554092	295.8656276	322.829769	495.7423052	413.1637595
MARCH1	0	3.04720192	1.002934331	0	26.19776409	1.812121752
SFRP2	0	0	0	0	0	0.906060876
RPS3A	15440.8675	15910.071	25738.90549	22970.4343	22145.89699	16462.72745
RNF175	0	0	0	0	0	0
PDGFC	2255.24344	1851.68303	184.5399169	134.763829	60.45637868	407.7273943
CBR4	277.388106	246.823355	978.8639068	530.004014	328.4796575	713.9759704

GLRA3	0	0	0	0	26.19776409	31.71213067
CYP4V2	765.747448	752.658873	466.3644638	217.231247	67.50962286	136.8151923
ROPN1L	30.2782792	36.566423	0	0	10.07606311	2.718182629
NDUFS6	1504.14677	1486.0188	1419.152078	1284.27918	1524.508349	1475.973167
MARCH6	5411.99823	4657.14026	7144.904172	5454.91797	4908.050342	7755.8811
NKD2	0	0	270.7922693	101.575722	118.8975447	227.4212799
CDH18	16.6042176	12.1888077	0	0	30.22818934	13.59091314
ADAMTS16	1190.62008	956.821402	0	0	18.1369136	1.812121752
SRD5A1	1165.22539	1366.16219	660.9337239	756.286562	771.8264344	518.2668212
MYO10	4311.23627	4862.31853	918.6878469	952.398104	968.3096651	1599.197446
FAM105A	317.433572	352.459688	87.25528677	110.627024	429.2402886	239.2000713
RPL37	12437.5357	11674.8463	13028.11696	9418.38252	10150.62598	7220.399122
SKP2	1815.72003	1796.8334	498.4583624	503.855808	632.7767635	808.2063016
OSMR	6689.54627	5703.34625	104.3051704	258.464955	1396.542347	408.6334552
UGT3A1	0	0	0	0	0	0
PLK2	9774.02387	6600.23935	3602.540116	6735.17434	3658.618516	7278.387018
FAM159B	0	0	0	0	0	0
GZMA	0	1.01573397	0	0	0	0
PIK3R1	504.96356	639.912403	2808.216126	4509.55977	2533.122267	3848.040541
HAPLN1	0	0	0	1.00570022	2.015212623	0
LHFPL2	1557.8663	1931.92602	357.0446217	137.78093	944.1271137	277.2546281
SSBP2	74.23062	66.0227082	1022.993017	943.346802	418.1566192	503.7698472
BHMT	0	0	0	0	0	0
ANKRD31	4.88359342	7.11013781	25.07335827	9.05130194	27.2053704	27.18182629
IQGAP2	25.3946858	19.2989455	2584.56177	1812.27179	6.045637868	2514.318931
CRHBP	0	0	0	0	2.015212623	0
RASA1	3132.33682	3463.65285	841.4619035	1317.46728	1120.458218	1299.291296
LIX1	0	0	0	0	0	0
GIN1	135.763897	103.604865	198.5809975	185.04884	162.2246161	154.9364098
PPIP5K2	2186.87313	2371.73883	1281.750075	1830.37439	1325.002299	1885.512683
PAM	5042.79856	4265.06695	350.0240814	545.089517	1608.139673	650.5517091
BDP1	2999.50308	2546.44507	1874.484264	2082.80515	2282.228295	2576.837132
GTF2H2	657.575854	629.480815	354.8682542	327.928669	607.4656929	451.7981953

SLC30A5	1565.68005	1762.29844	2386.983707	2092.86215	2272.152232	2295.052199
BTF3	11658.1142	10864.2906	9689.348569	7413.01629	8808.494373	7239.426401
FBXL17	522.544496	613.503319	575.6843058	672.813444	671.0658033	744.7820402
SPATA9	0.97671868	1.01573397	0	0	32.24340196	5.436365257
TSLP	0	0	0	0	0	0
TNFAIP8	560.636525	527.165932	61.17899417	41.2337088	232.7570579	98.7606355
FEM1C	1157.41164	1115.2759	1490.360415	1368.75799	538.0617702	1953.467249
COMMD10	580.170898	552.559281	496.4524937	368.086279	546.1226207	423.1304292
ATG12	1382.05694	1279.82481	1268.711928	1025.81422	1269.583952	1092.709417
MEGF10	2.93015605	5.07866986	21.06162094	18.1026039	0	17.21515665
ADAMTS19	0	0	0	5.02850108	1041.864926	77.01517448
YIPF5	1585.21442	1786.67606	1240.629767	1054.97953	790.9709544	1057.373042
ARHGAP26	1279.50148	1157.93673	1689.944347	290.647362	538.0617702	607.9668479
CXCL14	1.95343737	0	0	0	6.045637868	0.906060876
LECT2	0	0	0	0	0	0
SLC25A48	0	0	0	0	1.007606311	0.906060876
DDX46	6079.09709	5067.02955	3056.94384	3056.78558	3805.729038	3609.945864
CDKN2AIPNLP2	0	0	0	0	0	0
IL9	0	0	0	0	0	0
TIMD4	0	0	0	0	0	0
RNF145	3231.96212	3567.25771	436.2764339	240.362352	560.2291091	405.0092117
C1QTNF2	21.487811	31.4877531	0	1.00570022	2.015212623	6.342426133
GABRA6	0	0	1.002934331	0	0	0
GABRB2	0	0	0	0	0	0
FBXO38	1181.82961	1027.92278	861.5205901	837.74828	561.2367154	899.71845
SPINK7	0	0	0	0	0	0
PCYOX1L	206.087642	294.562852	182.5340482	232.31675	584.4116605	327.9940372
GLRA1	0	0	0	0	0	0
TNIP1	7448.45668	9053.23689	1115.262976	1378.815	2307.418453	1466.006498
G3BP1	11132.6396	10533.1613	4186.237867	4207.8497	6452.710817	5495.259214
ZNF300	0	0	385.126783	342.943774	106.806269	321.651611
N4BP3	1.95343737	6.09440383	465.3615294	468.6563	1692.778603	536.3880387
NHP2	2016.92408	2064.98717	1227.591621	971.506408	5134.761762	1958.903614

RMND5B	630.96027	760.784745	1837.375694	1427.08861	2982.514681	1775.879317
BOD1	2276.73125	2299.62171	809.3680049	758.297963	2662.095874	874.3487455
CPLX2	0	0	0	0	0	0
TENM2	407.291691	242.760419	1.002934331	1.00570022	4.030425245	0
KCNMB1	0.97671868	0	0	0	8.06085049	0
FAM50B	191.436862	166.580371	426.2470906	361.046377	450.4000211	391.4182985
MYLK4	6.83703079	11.1730737	14.04108063	11.0627024	6.045637868	48.02122644
FAM217A	0	0	0	0	0	0
TBC1D7	311.57326	399.183451	304.8920365	325.84687	489.6966673	384.1698115
FARS2	333.061071	314.877531	308.9037739	215.219846	217.6429632	235.5758278
GFOD1	488.359342	621.629191	128.3755943	232.31675	495.7423052	434.0031597
CDKAL1	831.1876	871.499748	268.7864006	256.453555	360.7230594	276.3485672
PCDHB18P	3.90687474	9.14160575	3.008802992	0	9.118837117	3.624243505
PSD2	0	0	3.008802992	5.02850108	15.11409467	11.77879139
LRRTM2	0.97671868	0	0	2.01140043	1.007606311	1.812121752
ZMAT2	1458.82703	1424.42469	1555.982409	1467.25627	1735.360046	1655.96216
GFRA3	3.90687474	1.01573397	6.017605984	3.01710065	21.15973254	6.342426133
KLHL3	16.6042176	17.2674775	1.002934331	2.01140043	1.007606311	1.812121752
DCDC2	0.97671868	5.07866986	0	0	1263.538314	280.8788716
SLC17A4	0	0	0	0	0	0
HIST1H2BA	0	0	0	0	0	0
KAAG1	0	0	0	0	2.015212623	0
TRIM7	24.4179671	12.1888077	4.011737323	4.02280086	80.6085049	0
TRIM41	1250.19992	1383.42967	851.4912468	1079.11633	1128.519069	1135.294278
HIGD2A	1596.93505	1656.66211	1057.092785	1429.10001	1582.949515	959.5184679
FAM193B	1397.68444	1274.74614	1431.18729	1334.56419	764.7731903	1430.670123
PLA2G7	0	0	0	0	0	0
TNFRSF21	6334.99738	5530.67148	43.12617622	43.2451093	829.2599942	330.7122198
RNF44	853.65213	817.665848	748.1890107	944.352502	1547.683294	1336.439792
MUT	1410.38178	1236.14824	1349.949609	1111.29874	631.7691572	1141.636704
RASGEF1C	0	0	0	0	7.053244179	0.906060876
DOK3	247.109827	239.713217	74.21714047	139.79233	352.662209	221.9849147
ABT1	1275.5946	1354.98912	520.5229176	505.867208	906.8456802	585.315326

PPP1R18	5235.21215	6008.06645	131.3843973	301.710065	833.2904194	569.0062302
DAAM2	98.6485871	86.3373876	0	0	0	1.812121752
PRIM2	1242.38617	1453.51531	299.8773649	373.11478	623.7083067	537.2940996
MLIP	0.97671868	0	0	0	0	0.906060876
HMGCLL1	1.95343737	3.04720192	0	0	74.56286704	1.812121752
LGSN	0	0	0	2.01140043	5.038031556	0.906060876
FGD2	9.76718684	8.12587178	0	2.01140043	4.030425245	0.906060876
SCUBE3	199.250612	121.888077	20.05868661	86.4902185	29.22058303	69.76668747
ANO7	149.437959	121.888077	25.07335827	37.210908	71.5400481	62.51820046
CRIP3	0	0	4.011737323	3.01710065	0	0.906060876
TTBK1	0.97671868	1.01573397	0	3.01710065	19.14451991	4.530304381
TCTE1	0	0	20.05868661	9.05130194	0	4.530304381
RPL7L1	22748.7842	21169.3993	4813.312528	3609.26699	8855.811566	7214.437242
NFKBIE	976.718684	1074.64654	344.0064754	311.767067	237.7950895	324.3697937
CYP39A1	41.9989034	65.0069742	15.04401496	10.0570022	0	1.812121752
TPBG	1217.9682	1294.04508	747.1860764	1067.04793	3238.446684	1167.006409
IRAK1BP1	71.3004639	88.3688556	276.8098753	237.345251	25.19015778	286.3152369
PHIP	3382.3768	2960.86453	4137.104114	3861.88883	3652.572878	5287.771273
PRSS35	15.6274989	31.4877531	0	0	6.045637868	2.718182629
MMS22L	1255.08351	1098.00842	424.2412219	535.032515	1302.83496	1052.842738
FAXC	48.8359342	80.2429838	150.4401496	169.963336	49.37270925	173.0576274
GABRR1	0	0	0	0	0	0
PNRC1	802.862758	807.508508	1995.839318	2859.20571	628.7463382	1571.109559
PM20D2	2110.68908	1742.9995	52.1525852	77.4389166	481.6358168	59.80001783
RARS2	400.45466	368.711432	476.3938071	497.821607	963.2716336	653.2698917
SCML4	0	0	0	0	0	0
TBC1D32	49.8126529	60.9440383	230.6748961	151.860733	102.7758438	175.77581
CLVS2	0	0	0	0	0	0
GPR6	0	0	0	0	0	0
RNF217	614.356052	577.95263	482.4114131	410.325688	41.31185876	595.2819956
RSPO3	0	0	0	0	0	0
ARHGAP18	2318.73016	2601.2947	255.7482543	420.38269	470.5521474	473.8698382
TAAR2	0	0	0	0	0	0

TAAR6	0	0	0	0	0	0
TAAR8	0	0	0	0	0	0
ABRACL	502.033404	909.081905	300.8802992	614.482832	808.1002617	453.0304381
TAAR1	0	0	0	0	0	0
SLC18B1	211.947954	298.625788	612.7928761	458.599298	47.35749663	530.9516734
MTFR2	381.897005	369.727166	123.3609227	215.219846	294.2210429	258.2273497
SLC2A12	202.180768	141.187022	2.005868661	12.0684026	17.12930729	12.68485227
SHPRH	557.706368	493.646711	970.8404321	922.227098	888.7087666	1050.124555
AIG1	719.84167	804.461306	1567.586359	831.714078	396.9968866	859.8517715
DYNLT1	839.001349	876.578418	1308.829302	1157.56095	950.1727515	982.1699898
TIAM2	381.828635	298.625788	37.81062427	62.3232424	77.91819605	62.89874602
TMEM181	1511.96052	1650.5677	1725.047049	1359.70669	1395.534741	1667.152012
PNLDC1	0.97671868	1.01573397	5.014671654	4.02280086	0	3.624243505
WTAP	1901.70058	1758.97699	1299.341543	1451.11478	1484.728052	1806.57666
ZMYM4	2064.7833	1978.64978	2308.754829	2156.22126	2223.787129	2625.764419
VIP	0	0	0	0	0	0
ARMT1	832.164319	803.445572	706.0657688	708.012952	5662.747469	840.8244931
SLC22A3	293.015605	317.924733	0	0	3.022818934	1.812121752
LINC01558	0	0	0	0	3.022818934	0
VWDE	135.763897	117.825141	141.4137406	111.632724	17.12930729	294.4697848
GNA12	3705.67069	3798.84506	3299.653948	3055.31725	2960.347343	4060.058786
C7orf50	1136.90055	1284.90347	2165.33522	1195.77756	3671.717398	1665.33989
SDK1	285.201856	200.099593	3.008802992	1.00570022	770.8188281	43.49092206
WASH2P	300.233556	236.757432	369.8420638	342.320239	241.2007988	397.914755
CCZ1B	998.157659	885.648922	1655.252849	1468.43294	1791.906912	1373.506743
C7orf26	530.358245	596.235842	540.5816043	658.733641	847.3969078	649.6456482
RBAK	738.643505	680.826167	1146.975759	1083.95375	1968.157408	1491.974202
CREB5	298.875917	263.075099	0	0	40.30425245	19.93333928
FERD3L	0	0	0	0	0	0
EGFR	11802.6686	10206.095	19.05575228	9.05130194	861.5033962	491.0849949
LINC00525	3.90687474	1.01573397	0	0	6.045637868	0
CDCA5	4232.12206	3898.38699	502.4700997	1039.89402	2960.347343	2637.543211
IGFBP3	783.328384	694.762037	687.0100165	1111.29874	209.5821127	2244.31279

PURB	3393.12071	3537.80143	3437.055951	3449.55174	4929.210075	4067.307273
AC004453.8	25.404453	32.5339591	33.10686226	30.1710065	43.3774517	15.43021672
IGFBP1	414.128722	239.713217	0	0	16.12170098	1.812121752
SSC4D	13.6740616	10.1573397	13.0381463	15.0855032	10.07606311	11.77879139
MDH2	4121.75285	4107.62818	4651.609426	4370.77314	7145.94396	6507.329213
POMZP3	304.579954	316.137042	170.4386602	118.732967	109.1036114	173.365688
AC006014.8	0	0	1.002934331	2.01140043	0	0
GBAS	996.253058	820.71305	1668.882726	1760.98108	744.621064	1952.561188
CCT6A	8240.556	8220.31472	3801.050908	3876.89387	7656.760055	7530.235699
PSPH	686.349986	637.880935	1742.096932	683.876147	594.4877237	822.7032756
TRIM50	0	1.04620599	0	3.15789868	3.022818934	0
ZNF92	229.528891	255.964961	661.9366583	861.885085	344.6114345	1043.283796
ATXN7L1	151.391396	137.124086	117.3433167	174.991838	306.3123186	199.3333928
TMEM168	534.26512	497.709646	577.6901745	561.18072	818.1763248	615.2153349
ASB15	0	0	0	0	0	0
C7orf43	365.292788	316.908999	404.1825353	392.223084	732.5297883	614.309274
SLC12A9	674.912611	675.463092	356.0416874	475.696202	1793.539234	789.1790232
GIGYF1	1655.53817	1443.35797	1263.697257	1021.79142	1747.189344	1292.94887
TRIM4	409.245129	478.410701	1451.245977	1772.04378	522.9476756	1590.136838
MEPCE	1399.63787	1696.27573	796.3298586	1007.71162	3467.173317	1388.085262
ZAN	0	0	0	0	0	0
TMEM209	803.839477	791.256764	850.4883124	434.462493	913.8989243	854.3882244
AGBL3	16.6042176	27.4248173	42.12324189	45.2565097	12.09127574	39.86667855
STRA8	0	0	0	0	0	0
ZC3HAV1L	302.782792	234.634548	124.363857	103.587122	66.50201655	197.521271
TMEM140	54.6962463	63.9912403	16.04694929	22.1254047	29.22058303	157.6545925
TLK2	1675.89299	1581.33528	1297.175205	1197.50736	3999.139069	1383.491534
EPHA1	0	0	548.6050789	552.129418	762.7579776	699.4789964
NOM1	2056.96955	1612.98555	751.1978137	605.43153	2036.372355	998.4790855
CNPY1	0	0	0	0	0	0
NCAPG2	5069.16997	3958.31529	686.0070822	1627.22295	6347.919761	2347.60373
ASB10	0	0	0	0	0	0
NLGN4X	0	0	0	1.00570022	0	0

SHROOM2	389.710755	476.379233	1431.18729	1505.53322	1000.553067	1167.006409
RAB19	0	0	33.09683291	34.1938073	27.2053704	35.33637417
LUC7L2	2953.24568	2592.52892	2370.89664	2998.64605	3181.758753	3005.521714
DENND2A	1023.60118	918.223511	0	1.00570022	1.007606311	0.906060876
TMEM27	7.81374947	6.09440383	9.026408976	2.01140043	5.038031556	3.624243505
SH3KBP1	3565.0232	4264.05122	1214.553474	517.935611	939.0890821	708.5396052
TMEM47	0	0	40.11737323	62.3534134	0	43.49092206
LANCL3	1.95343737	3.04720192	0	0	4.030425245	0.906060876
SYTL5	0	0	631.8486283	70.3990151	1657.512382	115.0697313
CASK	2181.98954	1905.51693	3272.574721	3326.85631	1364.298945	4158.819422
KDM6A	1140.80742	1084.80388	1145.351006	1454.24251	822.20675	1696.14596
SPIN2A	15.9302817	17.8972326	35.34340581	39.2625364	16.43405894	28.60434186
MSN	20161.4075	19415.7549	0	1.00570022	521.9400692	315.3091849
AKAP4	0	0	0	0	0	0
CCNB3	46.8824968	56.8811024	33.09683291	37.210908	5.038031556	21.74546103
HDAC8	545.009026	520.055794	591.7312551	574.254823	327.4720512	428.5667944
SLC16A2	198.273893	302.688724	2162.326417	4070.06877	1036.826894	1264.860983
CXorf36	0	0	0	0	0	0
ZNF157	0	0	0	0	9.068456802	2.718182629
ZNF182	415.105441	366.679964	522.5287863	537.043915	141.0648836	311.6849414
CHST7	569.426993	643.975338	21.06162094	12.0684026	129.9812142	24.46364366
KRBOX4	260.471339	230.023115	452.5139407	283.537062	213.9450481	230.8733719
NDUFB11	1280.47819	1173.17274	1880.50187	1821.32309	1528.538774	1349.124645
ZNF41	416.082159	382.931708	476.3938071	531.009714	489.6966673	418.6001248
RAB41	0	0	0	0	0	0
ZMYM3	2169.2922	2004.04313	2301.734289	3245.3946	1879.185771	2734.491724
TAF1	1628.19005	1488.05027	1715.017706	1683.54216	1727.037218	1516.745907
GPR174	0	0	0	0	0	0
NONO	11151.7539	11250.9094	12176.32483	12871.5246	12547.19744	10068.71927
CCDC120	398.501223	392.073313	1214.553474	1253.10247	805.0774427	1191.470052
LPAR4	1.95343737	1.01573397	0	0	1.007606311	0
EBP	1112.48258	2147.26162	1066.119194	8891.39561	1459.013939	1022.942729
AWAT2	0.97671868	6.09440383	2.005868661	9.05130194	0	5.436365257

OGT	7570.54652	5942.04374	15651.79317	7388.87948	4454.627502	6840.759615
SNX12	1576.42396	1752.1411	1327.885054	1976.20092	1578.91909	1216.839757
ITGB1BP2	14.6507803	7.11013781	9.026408976	19.1083041	10.07606311	5.436365257
IL2RG	3.90687474	5.07866986	5.014671654	4.02280086	0	0.906060876
ACRC	10.7439055	8.12587178	73.21420614	65.370514	23.17494516	60.7060787
ZNF711	0	0	1058.095719	877.976288	44.3346777	780.1184144
CPXCR1	0	0	0	0	0	0
DIAPH2	887.837284	902.987501	50.14671654	60.3420129	713.3852684	72.48487009
NXF3	21.487811	37.582157	0	0	0	0.906060876
RIPPLY1	0.97671868	0	0	0	0	0.906060876
PRPS1	1448.47381	1282.87201	1006.946068	1012.74012	1257.492676	1004.821512
CXorf57	12.6973429	24.3776153	400.170798	645.659538	6.045637868	231.9515843
FRMPD3	10.7439055	16.2517436	35.10270157	36.2052078	16.12170098	48.92728731
HTR2C	0	0	0	0	5.038031556	0
DOCK11	376.036693	521.071528	53.15551953	72.4104155	483.6510294	48.92728731
IGSF1	0	0	0	0	654.9441023	67.95456571
ARHGAP36	0	0	0	0	54.41074081	1.812121752
GPC3	0	0	0	0	10.07606311	0.906060876
GPR119	0	0	0	0	0	0
RBMX	5439.05333	4723.11218	6399.93458	6018.52243	7863.702239	6875.941959
MCPH1	306.689667	305.735926	283.8304156	266.510557	544.1074081	348.8334373
MFHAS1	713.981358	823.760251	306.8979052	341.938073	1609.147279	451.2183163
FBXO25	303.759511	240.728951	484.1464895	546.296357	480.6282105	306.1398488
FATE1	0	0	0	0	0	5.436365257
MAGEA4	0	0	22.06455528	1.00570022	0	13.59091314
NSDHL	621.193083	1021.82838	1116.26591	6904.13198	1768.349076	934.1487633
ZNF185	2213.24454	1977.63404	322.9448545	254.442155	4002.212268	476.5880209
CETN2	516.684184	615.534787	1981.798237	3315.79361	1630.307012	1265.767044
RPL10	15880.8402	15020.0341	95302.2702	69319.2551	36372.56255	50435.0904
CSGALNACT1	56.6496837	51.8024326	13.0381463	13.0741028	45.34228401	22.6515219
ATP6V1B2	1629.16676	1762.29844	1764.161488	1535.70423	1461.029151	1552.988342
CCDC25	618.262927	633.817999	498.4583624	493.798806	1321.97948	675.9214136
HMBOX1	167.995614	152.360096	187.5487198	198.122942	462.4912969	264.5697758

CHRNB3	0	0	0	0	0	0
CHRNA6	0	0	0	0	0	0
GNRH1	32.2317166	8.12587178	18.05281795	18.1026039	10.07606311	10.87273051
BIN3	288.132012	275.263906	195.5721945	179.014638	524.9628882	274.5364455
DOK2	0	0	0	0	0	0
SLC25A37	4779.08452	4398.1281	835.4442975	507.878609	1357.245701	964.0487723
CHMP7	926.906031	946.664062	1834.366891	1237.01127	1548.6909	1832.961153
DOCK5	2433.00624	2149.29309	1860.443183	2058.66834	2623.806835	2569.588645
STAR	0	0	0	0	0	1.812121752
PROSC	701.284015	777.036489	794.3239899	1247.06827	862.5110025	1143.448826
ERLIN2	1308.80304	1522.58522	1426.172618	1244.05117	1624.261374	1369.964045
SNTG1	0	0	0	0	0	0
PXDNL	0	0	0	0	0	0
ST18	2.93015605	5.07866986	0	0	2.015212623	0
RGS20	115.252805	99.5419293	0	0	22.16733885	6.342426133
TACC1	5468.64791	4797.31155	5979.49448	7109.29482	1886.239015	6258.162472
GOLGA7	957.18431	1022.84411	983.8785784	966.477907	793.9937733	872.5366238
PLPP5	311.57326	340.270881	955.7964172	673.819144	337.5481143	551.7910736
GINS4	1796.18566	1436.24784	456.3351205	441.502395	752.6819145	934.1487633
WHSC1L1	2715.27794	2366.66016	3377.882826	2793.8352	2401.12584	3352.425242
DNAJC5B	0	0	0	0	0	0.906060876
CRH	0	0	0	0	0	0
TRIM55	4.88359342	2.03146794	0	0	0	0.906060876
ADHFE1	30.2782792	7.24218322	779.279975	187.06024	5.038031556	146.7818619
MRPS28	361.385913	404.262121	356.0517167	391.49898	436.2935328	397.3439366
PMP2	0	0	0	0	0	0
LACTB2	314.503416	297.610054	775.2682376	1605.09754	298.2514681	484.7425688
PRDM14	0	0	0	0	0	1.812121752
TERF1	629.964017	714.995458	1247.640278	1205.77422	718.4232999	1485.033776
RPL7	12078.0642	11932.6802	29970.7869	22240.3462	16645.42451	20639.00667
SLC26A7	11.7206242	18.2832115	3.008802992	3.01710065	6.045637868	9.060608762
PSKH2	0	0	0	0	0	0
ATP6V0D2	3.90687474	21.3304134	0	0	3.022818934	9.060608762

SYBU	93.7649937	117.825141	257.754123	832.719778	88.66935539	341.5849503
DPYS	0	0	0	1.00570022	0	4.530304381
MTDH	9522.03045	10278.2121	10341.25588	8183.38265	11670.0963	9542.633148
LRP12	1655.53817	1944.11482	190.5575228	313.778467	285.1525861	331.6182807
EBAG9	372.129819	414.419461	776.271172	857.862284	867.549034	697.6668747
RSPO2	1.95343737	0	0	0	0	0
POLR2K	984.532433	995.419293	1621.744813	1265.17087	1453.975907	1250.364009
MAL2	1.95343737	3.04720192	13984.91631	8116.00074	8340.965045	13918.00112
EIF3H	4804.47921	4714.02137	6706.621869	5432.79256	4625.920575	4600.071068
UTP23	767.700886	857.279473	838.4531005	651.69374	1175.876565	852.6032845
NDUFB9	853.65213	964.947274	3276.586458	2289.97939	7624.556958	3091.47971
TATDN1	237.34264	194.005189	645.889709	528.998313	1944.680181	639.6789786
FAM83A	209.994517	280.342576	158.4636243	79.450317	914.9065306	569.9122911
GSDMC	21.487811	22.3461474	27.07922693	3.01710065	1.007606311	7.248487009
FAM135B	0	0	0	0	0	0
TTTY7	0	0	0	0	0	0
TTTY7B	0	0	0	0	0	0
ZNF7	627.053395	502.788316	1036.031164	764.332164	757.7199461	1102.676086
ARHGAP39	702.260734	664.290018	800.3415959	735.166858	741.5982451	1052.842738
SLC39A4	485.429186	434.73414	419.2265502	481.730403	432.2631075	578.9728999
NAPRT	614.356052	604.361713	1007.949002	862.890785	655.9517086	1047.406373
VLDLR	399.477942	330.113541	672.9689359	361.046377	408.0805561	299.0000891
AK3	990.392745	892.830162	1019.984214	894.067492	1897.322684	926.9002763
UHRF2	609.472459	623.660659	626.8339567	562.18642	808.1002617	513.7365168
NFIB	1315.64007	917.207777	5613.423449	5170.30481	1749.204556	6123.159401
CER1	0	0	0	0	0	0
PLIN2	1532.47161	1573.37192	0	1.00570022	415.1338003	168.527323
IFNA5	0	0	0	0	0	0
HAUS6	1581.95218	1457.74077	1124.720646	848.609842	1489.907148	1014.353272
CDKN2B	0	0	92.26995843	128.729628	330.4948701	147.6879228
IFNA16	0	0	0	0	0	0
CDKN2A	0	0	517.5141146	515.924211	23.17494516	314.403124
C9orf72	272.504513	291.51565	264.7746633	403.285786	65.49441023	173.9636882

IFNK	0	0	0	0	0	0
ZCCHC7	451.244032	468.253361	835.4442975	588.334626	389.9436425	741.1577967
FBXO10	313.468094	328.813401	184.2189779	179.457146	249.1205844	174.9241128
SIGMAR1	2954.57402	3181.2788	3206.381055	3166.94998	1459.013939	3838.073871
CBWD5	602.742867	666.99187	1276.424493	1068.52631	1253.149893	1067.01353
CEP78	1823.53378	1836.44702	778.2770406	780.423367	1899.337897	686.7941441
NTRK2	1.95343737	0	0	1.00570022	9.068456802	1.812121752
IDNK	65.4401518	68.0541761	182.5340482	87.4959188	64.48680392	56.17577432
SHC3	372.129819	459.111755	54.15845386	91.5187196	16.12170098	60.7060787
AUH	167.018895	140.171288	118.346251	92.5244198	122.92797	115.0697313
HIATL1	2688.61352	2898.85397	1338.957449	1418.84186	2805.528633	1275.026986
C9orf3	705.19089	623.660659	84.24648378	86.4902185	665.0201655	145.8758011
PLPPR1	0	0	10.02934331	14.079803	2.015212623	4.530304381
OR13C4	0	0	0	0	0	0
ZNF462	1412.33522	1472.81426	287.8421529	262.487756	1430.800962	397.7607246
INIP	712.027921	754.690341	806.3592019	728.126956	1583.957121	546.3547083
UGCG	6721.77798	5862.81649	357.0446217	391.217384	1662.550414	404.1031508
ACTL7B	0	0	0	0	1.007606311	0
SNX30	1243.36288	1338.73738	838.4531005	1249.07967	1389.489103	438.5334641
STOM	273.481231	305.735926	9608.110888	9457.60483	2189.528514	12214.60667
GSN	851.698692	1067.5364	435.2734995	613.477131	3518.561239	1551.17622
MRRF	784.305103	829.854655	878.5704737	1064.03083	1006.598705	719.4123357
NR6A1	146.507803	172.674775	29.08509559	19.1083041	247.8711526	70.67274834
CRB2	0	0	0	0	6.045637868	0
OR5C1	0	0	0	0	0	0
ALAD	765.747448	816.650114	909.661438	1043.91682	1152.70162	766.5275012
ASTN2	38.0920287	33.5192211	247.7247797	514.91851	258.954822	271.8182629
WDR31	80.0909321	73.132846	226.6631587	118.672625	114.8671195	119.6000357
POLE3	2396.86765	2421.50979	1186.471313	1024.80852	4043.524127	1481.409533
SURF4	8239.59882	9638.29966	4444.002019	3604.42957	8319.805312	4518.52559
GBGT1	13.6740616	22.3461474	1.002934331	0	0	5.436365257
SURF1	357.479038	407.309323	589.7253865	611.465731	714.3928747	766.5275012
SURF2	323.293884	374.805836	351.0270157	330.875371	888.7087666	487.4607514

SURF6	1102.71539	1203.64476	1224.582818	844.395958	1965.839913	1665.104315
MED22	1347.87178	1229.03811	773.262369	841.77108	1964.832307	1379.930714
REXO4	886.860565	950.726998	732.1420614	684.881847	1638.367862	1051.030616
RPL7A	23267.5195	23576.3637	18751.16113	16403.8455	34007.30749	17688.18385
GTF3C5	1861.62581	1705.41734	1046.060507	1104.25884	2640.936142	1386.273141
ASB6	838.024631	884.70429	612.7928761	622.528433	1102.321305	784.6487188
PTGES2	1573.4938	1533.7583	1376.025902	1109.28734	2865.632349	1644.50049
NTMT1	981.602277	865.405344	479.4026101	504.861508	1504.356223	634.2426133
CIZ1	3194.84681	3407.78748	1926.636849	2571.57545	5632.51928	2571.400767
SLC25A25	585.054492	677.494559	247.7247797	385.183183	1416.694474	473.8698382
SH3GLB2	2078.45736	2144.21442	3540.358187	3919.21374	3944.778709	3075.170614
FAM73B	636.820582	701.364308	632.8515627	643.426884	1088.214816	836.2941887
PTGES	1808.883	1679.00826	0	4.02280086	361.7306658	64.33032221
LCN2	314.503416	288.468448	1.002934331	0	452.4152338	1300.197357
LRSAM1	487.382623	426.608268	1359.978952	1073.08213	716.4080873	1381.742836
HMCN2	0	0	0	0	9.068456802	7.248487009
GPR107	2934.06293	2989.30508	3426.023674	2728.46468	3104.435045	3981.23149
C9orf142	751.096668	787.193828	1982.801172	1854.5112	1276.637196	2082.127893
IDI2	1.95343737	0	0	1.00570022	0	0
INPP5E	708.121046	623.660659	727.1273898	690.916048	679.1266538	779.2123535
LCN9	0	0	0	0	0	0
SEC16A	3709.57756	4005.03905	17315.66122	20303.076	11124.98128	18612.30252
DPH7	759.887136	706.950845	1081.163209	893.061791	885.6859476	1146.167008
NOTCH1	2398.82109	2639.89259	823.4090855	902.113093	1944.680181	2035.012728
CACNA1B	0	0	7.020540315	0	5.038031556	13.59091314
NACC2	1954.41409	1744.01523	652.9102493	964.466507	3459.112467	1118.079121
PROSER2	2313.84656	1501.25481	9.026408976	1.00570022	602.5485742	85.16972236
USP6NL	1115.41274	1028.93851	472.3820698	533.021114	1503.348616	628.8062481
COMMD3	437.872753	497.516657	1330.683241	940.017934	399.5058264	906.5139066
MSRB2	303.759511	296.59432	390.1414546	278.57896	548.1378333	349.7394982
PDSS1	375.059975	382.931708	129.3785287	106.604223	596.5029363	251.8849236
FAM171A1	2523.84108	1941.06762	0	1.00570022	199.5060496	48.02122644
FAM188A	638.774019	636.865201	568.6637655	607.44293	454.4304464	528.2334908

SLC39A12	0	0	0	0	0	0
TMEM236	83.9978068	72.117112	3.008802992	2.01140043	5.038031556	16.30909577
RSU1	1928.04268	2241.72488	661.9366583	713.041453	2073.653789	676.8274745
ST8SIA6	14.6507803	4.06293589	228.6690274	228.293949	95.72259957	306.2485761
PARD3	2123.38642	2034.51515	644.8867746	651.69374	2336.639036	920.5578502
ANKRD30A	0	0	9365.40078	5518.27708	1.007606311	4267.546727
ZEB1	2332.40422	2179.7651	6.017605984	9.05130194	87.66174908	36.24243505
FAM13C	20.5110924	18.2832115	0	2.01140043	1.007606311	19.0272784
NRBF2	518.637621	524.11873	682.9982792	522.964112	409.0881624	426.6459454
A1CF	0	0	0	0	0	0
CDHR1	0	0	0	0	0	0
LRIT1	0	0	0	0	2.015212623	0
RGR	0	0	6.017605984	0	0	0.906060876
POLR3A	1562.74989	1459.60972	1193.491854	1074.08783	1282.682834	1507.685298
HERC4	2269.89422	2221.4102	951.7846798	1057.99663	1863.06407	1243.115522
C10orf11	0.97671868	0	0	0	4.030425245	0.906060876
CAMK2G	1502.19334	1719.63762	1004.940199	988.603312	1372.359796	1527.618637
ADIRF	18.557655	48.7552307	450.2974558	699.96735	784.9253165	329.7789771
GLUD1	4794.2725	4178.435	5293.096253	4347.29004	3358.82541	4690.831186
ANKRD1	12296.8882	10202.032	0	0	242.833121	115.0697313
HTR7	280.318262	315.893265	0	0	18.1369136	7.248487009
RPP30	981.602277	1084.80388	496.4524937	484.747504	629.7539446	524.6092473
FRA10AC1	488.359342	480.442169	974.8521694	419.37699	268.0232788	474.7758991
ADD3	1536.37849	1243.25838	0	0	431.2555012	54.36365257
HABP2	0	0	0	35.1995075	2.015212623	6.342426133
VAX1	5.8603121	8.12587178	0	0	0	0
DNAJB12	1501.21662	1768.39285	1090.189617	1408.986	1231.294912	1000.291207
EIF4EBP2	4452.86048	5191.41633	3810.147522	5666.11501	3906.489669	5222.53489
NPFFR1	0	0	0	2.01140043	0	0
PLEKHS1	193.390299	90.4003235	57.16725685	756.286562	28.21297672	204.769758
TCF7L2	643.657613	592.172906	251.736517	291.653063	325.4568385	465.7152904
MKI67	32168.2299	30234.3374	3756.992003	12883.0198	20848.38219	14902.88929
CYP17A1	0	0	1.002934331	1.00570022	0	0

INA	0	0	0	0	0	0
FUOM	0	0	215.6308811	154.877833	45.34228401	141.3454967
LRRC27	111.34593	93.4475254	328.9624605	210.191345	107.8138753	197.521271
MTG1	766.675331	751.14543	592.9949524	1217.04812	988.4617914	1040.592795
NKX6-2	0.97671868	3.04720192	0	0	0	0
PAOX	263.684743	205.178262	166.4870989	199.128643	69.52483548	120.5060965
GSTO1	2823.69372	3347.85917	846.4765751	861.885085	734.5450009	730.2850662
TAF5	959.137748	810.55571	184.5399169	217.231247	382.8903983	290.8455413
PPRC1	2888.15715	2889.76315	923.7025186	830.708378	2110.935222	1986.991501
ITPRIP	1855.7655	1702.37014	68.19953449	85.4845183	285.1525861	196.6152101
CNNM2	408.26841	598.26731	278.8157439	439.490994	504.810762	238.2940104
PDCD11	2838.3445	2783.11108	1421.157947	1085.15053	2597.609071	2900.300865
ADAM12	11.7206242	41.6450929	0	0	10.07606311	2.718182629
RGS10	2198.59376	2081.23891	81.23768079	135.769529	502.7955493	118.6939748
BTBD10	1619.39958	1639.39463	310.9096425	423.399791	940.0966884	391.4182985
ADM	4560.29953	2931.40824	43.12617622	23.131105	146.1029151	99.66669638
GAS2	0	0	10.02934331	9.05130194	0	4.530304381
SLC5A12	0	0	0	0	17.12930729	1.812121752
LIN7C	1565.68005	1626.19009	931.7259932	1078.11063	965.2868462	1176.067017
LRRC4C	0.97671868	0	4.011737323	23.131105	0	10.87273051
IMMP1L	80.0909321	85.3216537	242.710108	152.866433	101.7682374	177.5879317
SAA4	21.8589641	5.14977124	0	0	0	0
PGAP2	325.247322	412.387993	663.9425269	634.596836	377.8523667	674.1092919
TUT1	307.178026	315.253353	597.2975407	454.06359	582.2856112	607.477575
SCGB1A1	0	0	0	0	0	0
SYT8	6.83703079	7.11013781	0	0	33.25100827	7.248487009
ZNF214	28.3248418	31.4877531	38.11150457	23.131105	3.022818934	19.93333928
ZNF215	313.526698	299.641522	0	0	19.14451991	10.87273051
HSD17B12	2886.20371	3214.79802	3495.226143	5308.08574	813.1382932	3742.031419
APIP	919.092282	877.594152	625.8310224	533.021114	475.5901789	477.4940817
PAMR1	0.97671868	0	1.002934331	1.00570022	0	2.718182629
DGKZ	2949.74903	3219.14536	2240.755882	2336.56342	5278.829313	3397.773589
EIF3M	5828.08039	5363.07537	3518.293632	2796.8523	2942.210429	2812.41296

TNKS1BP1	3183.12619	3792.75065	16596.5573	12751.273	3731.166171	12345.9855
GLYAT	0	0	0	0	0	0
SERPING1	9.76718684	2.03146794	1.002934331	0	3.022818934	4.530304381
OR5F1	0	0	0	0	0	0
SSRP1	10498.7491	9895.28036	4884.290191	5586.6647	5703.051722	8777.917768
SLC43A1	448.313876	439.81281	71.20833748	5.02850108	52.39552819	22.6515219
PTPRJ	7983.69852	5726.70814	2548.456134	2571.57545	1964.832307	2329.482513
C11orf49	488.359342	485.520839	684.0012135	475.696202	684.1646854	654.1759526
ARFGAP2	1438.87266	1159.03372	1707.696285	2100.94798	3212.752724	2245.925579
CELF1	5844.6846	5853.67488	4147.133457	3602.41817	6436.589117	4963.40148
C11orf73	485.429186	528.181666	718.1009808	653.70514	697.2635674	688.6062659
CCDC81	6.83703079	1.01573397	11.03227764	11.0627024	10.07606311	13.59091314
SESN3	10.7439055	9.14160575	149.4372153	767.349264	209.5821127	367.8607157
ENDOD1	1366.42944	1343.81605	249.7306483	100.570022	795.0013796	355.1758635
CCDC82	1665.30536	1689.1656	76.22300913	79.450317	78.59329228	94.23033112
KLHL35	8.79046815	13.2045416	1395.081654	1326.51858	174.3158919	1229.524609
TENM4	20.5110924	42.6608268	7.020540315	4.02280086	43.32707139	17.21515665
SERPINH1	6180.67583	6888.7078	2698.896284	2263.83119	5397.74701	3384.137373
CAPN5	453.197469	532.244601	77.22594346	37.210908	358.7078468	159.4667142
INTS4	972.811809	1090.89829	691.202282	730.037786	818.8614971	813.3346061
PAK1	849.745255	928.380851	201.5898005	189.071641	422.1870444	558.1334997
RPS3	20765.0392	21596.5357	25665.18982	28971.1961	46518.14042	23550.6786
ZC3H12C	4169.61206	4513.92177	27.07922693	31.1767067	380.8751857	77.92123535
TTC12	649.517925	631.786531	781.3359903	890.044691	141.0648836	720.3183966
NCAM1	0	0	11.03227764	9.05130194	1.007606311	71.57880922
DRD2	0	0	0	0	4.030425245	0
C11orf52	4.88359342	8.12587178	591.7312551	690.916048	192.4528055	405.0092117
HTR3B	0	0	0	0	0	0
NPAT	1801.06925	1626.19009	579.6960431	580.289024	525.9704945	827.23358
ATM	4944.14998	4189.90264	1956.724879	1898.76201	1133.5571	1995.146049
AASDHPPT	1826.46394	1818.16381	1041.045835	940.329702	722.4537252	1309.257966
GLB1L2	8.79046815	5.07866986	0	0	332.5100827	19.93333928
SLX4IP	291.062168	290.499916	692.0246882	676.836245	509.8487935	733.0032488

LAMTOR1	1762.00051	2067.01863	1855.428512	1979.21802	2568.388487	1542.115611
P4HA3	3.90687474	12.1888077	0	0	5.038031556	3.624243505
GRIK4	343.804977	217.36707	51.14965087	6.03420129	19.14451991	16.30909577
ST14	40.045466	46.7237627	4822.108262	5176.33901	4249.075815	6390.44736
HYOU1	11331.8902	13580.3632	9360.386109	6839.76717	6060.751962	7813.868996
GGTLC1	0	0	1.012963674	0	0	3.642364722
C20orf78	0	0	0	0	0	0
ADAM33	0	0	0	0	0	0
SLC22A8	0	0	0	0	0	0.906060876
CSRP2BP	646.734277	586.586369	544.3225493	636.336697	678.5623943	706.899635
TKFC	391.664192	396.136249	1262.413501	1838.95302	1217.43025	1641.084641
MTA2	4572.02016	4349.37287	4185.244962	3615.49227	4538.258826	5150.956081
TMEM138	766.724167	912.129107	549.6080132	502.850108	695.2483548	905.1548153
FADS1	5428.60244	7707.38938	589.7253865	2493.13083	3237.439078	579.8789608
TMC2	0	0	0	0	0	1.812121752
ROM1	31.2549979	38.5978909	34.09976724	61.3477131	58.44116605	58.89395695
EML3	1204.29414	1211.77063	969.8374978	1351.66109	1171.84614	1662.621708
INCENP	3103.03526	3093.92568	846.4765751	1639.29135	2290.289146	2015.079389
ZP1	0	0	2.005868661	0	0	1.812121752
OOSP2	0	0	0	0	0	0
MS4A3	0	0	0	0	0	0
PLCH2	3.90687474	4.06293589	3.008802992	13.0741028	21.15973254	3.624243505
FRG1BP	88.8911674	81.8478435	158.7945926	144.046442	85.43493913	150.2520751
CPSF7	3631.44007	3627.18601	2767.095818	2618.84336	2683.255607	3700.352618
MS4A2	0	0	0	0	0	0
B3GAT3	701.274248	809.539976	1681.920873	1756.93816	643.8604329	1007.539694
EI24	4281.93471	4775.98114	2886.445004	3533.02486	3010.727658	3651.425331
CCDC15	429.756221	477.394967	186.5457855	185.04884	137.0344583	237.3879496
CHEK1	2397.84437	2310.79479	467.3673981	846.799581	821.1991437	1228.618548
FEZ1	0	5.07866986	8.023474646	1.00570022	85.64653646	9.060608762
ESAM	2927.2259	2469.24929	28.08216126	283.607461	133.0040331	77.01517448
KIRREL3	3.90687474	0	0	0	0	0
MPZL2	166.042176	229.555878	76.22300913	101.575722	675.0962286	368.7667766

SCN2B	0.97671868	0	0	0	0	0.906060876
SIDT2	864.396035	920.254979	1383.046442	2431.78312	756.7123398	1458.758011
TMEM25	242.226234	236.198778	1454.25478	1786.727	87.66174908	1680.434865
TAGLN	1241.40945	730.312726	338.9918038	375.12618	1422.740112	249.1667409
JPH2	268.597638	211.272666	0	0	6.045637868	1.812121752
DUSP15	0.97671868	4.06293589	4.011737323	2.01140043	3.022818934	16.30909577
COMMD7	1569.58692	1497.19188	2470.227257	1719.74737	1079.146359	1530.33682
C20orf144	3.90687474	8.12587178	2.005868661	6.03420129	6.045637868	7.248487009
KIAA1755	31.2549979	30.4720192	0	0	3.022818934	0.906060876
SPATA25	4.88359342	5.07866986	15.04401496	18.1026039	4.030425245	7.248487009
OCSTAMP	0	0	0	0	0	0
DSN1	1667.25879	1599.78101	861.5205901	863.896485	885.6859476	1196.000357
SOGA1	6839.96094	6519.99637	8192.970548	9907.15282	5238.545212	9094.133014
CNBD2	46.8824968	39.6136249	81.23768079	98.5586211	39.29664614	58.89395695
SPINT4	0	0	0	0	0	0
CDH22	0	0	0	0	1.007606311	0
LINC00266-1	0	0	0	2.19242647	1.954756244	3.198394893
LSM14B	2182.96626	2322.98359	2081.088736	2487.09663	2670.156725	2752.612942
YTHDF1	2696.72029	2700.83663	2218.49074	1814.28319	4450.597077	2365.724948
CABLES2	881.976972	1001.5137	1134.318728	1071.07073	2418.255147	1323.75494
ORAOV1	940.580093	829.854655	413.2089443	368.086279	799.0318049	540.9183431
GPHA2	0	0	0	0	2.015212623	0.906060876
SLC22A9	0	0	1.002934331	0	0	2.718182629
TRPT1	381.897005	419.498131	585.7136491	618.505633	338.5557206	710.3517269
NUDT22	901.648086	971.22451	858.4014643	1047.50717	650.5307867	1014.797242
FERMT3	76.1840573	87.3531216	15.04401496	7.03990151	30.22818934	24.46364366
PLCB3	3640.23053	3598.74546	1715.017706	1801.20909	1942.664968	2734.491724
MRPL49	2415.42531	2401.19511	1989.821712	2614.82056	2634.890504	2823.28569
CDC42EP2	2091.1547	1906.53267	154.4518869	238.350951	362.7382721	361.5182896
FAU	5057.44935	5541.68204	4886.968025	4555.60072	5569.040082	4117.965137
TM7SF2	234.412484	436.765608	1347.94374	10092.2017	1512.417073	1763.194465
VPS51	2328.49734	2013.18473	2602.614588	3092.52816	1905.383535	2339.449182
TBX6	15.6274989	21.3304134	383.1209143	566.209221	84.63893015	347.0213156

PPP4C	1965.15799	1980.68125	4482.113524	4610.12979	6131.284404	5354.819778
ALDOA	22910.8902	23203.4269	41798.29117	71098.9824	66342.81475	54937.18911
FAM57B	3.90687474	8.12587178	86.25235244	150.855032	34.25861458	48.02122644
DOC2A	103.53218	127.982481	269.789335	229.299649	294.2210429	328.9000981
HIRIP3	637.797301	624.676393	1476.319335	1398.929	763.765584	1536.679246
TAOK2	1682.88629	1721.66908	6434.826666	6079.4578	4766.985459	7172.377896
TMEM219	677.842767	823.760251	1828.349285	2110.96475	854.450152	1687.991412
HMGA2	1313.68663	1615.01702	0	1.00570022	69.52483548	21.74546103
MMP3	19.5343737	46.7237627	0	0	12.09127574	8.154547886
CNKSR2	0	1.01573397	0	0	0	1.812121752
CNTN5	0	0	0	2.01140043	0	1.812121752
KLRF1	0	0	0	0	0	0
CLEC1A	0	0	0	0	0	0
MKX	68.3703079	35.550689	0	0	7.053244179	0.906060876
MPP7	69.3470266	117.825141	25.07335827	26.1482056	1158.747258	118.6939748
CCDC7	1.22089835	2.03146794	13.97087523	10.6000803	6.045637868	14.49697402
ITGB1	36892.0028	33324.515	3534.621403	5305.0083	13380.26619	5508.333672
FRMPD2B	0	0	0	0	0	0
FXYD4	0	0	0	0	6.045637868	0
TRIM48	0	0	0	0	0	0
OR8K1	0	0	0	0	0	0
OR5M9	0	0	0	0	0	0
PCDH15	0	0	0	0	0	0.906060876
PPIAP26	0	0	0	0	0	0
CTF1	183.623113	152.360096	2.005868661	0	11.08366942	5.436365257
CWC15	1329.31413	1186.37728	1641.803499	1163.59515	562.2443217	1157.039739
FCGR1A	0	0	0	0	0	1.812121752
ARID5B	671.005736	539.354739	1307.826367	1168.62365	1242.378582	1501.342872
KLHL1	1.95343737	0	0	0	0	0
CDH8	0	0	0	0	1.007606311	0.906060876
DCUN1D2	560.636525	430.671204	425.2441562	233.32245	444.3543833	364.2364722
TMCO3	2246.45297	1927.86308	2601.611654	1815.28889	1319.964268	1067.339712
TMEM218	286.178574	312.846063	378.1062427	284.613161	149.1257341	206.5818798

TIRAP	254.923576	279.326842	302.8861679	313.778467	227.7190264	365.1425331
N6AMT2	58.603121	49.7709646	132.3873317	80.4560172	173.3082855	119.6000357
LATS2	2134.13032	1625.17436	301.8832335	405.297187	738.5754262	409.539516
SAP18	1909.48503	1931.92602	3908.435087	2732.48749	2256.030531	2598.582593
ADGRL3	0	0	0	0	0	0
KIAA1328	183.623113	188.926519	105.3081047	155.883533	97.73781219	190.272784
FAM124A	91.8115563	88.3688556	3.008802992	0	30.22818934	9.060608762
MIA2	9.22022438	2.85421246	0	0	0	0
CTAGE5	823.784072	769.66226	2906.50369	2143.14716	618.6702751	1757.7581
HNMT	451.244032	512.945656	0	0	121.9203637	19.93333928
LYPD1	7.81374947	3.04720192	17.04988362	11.0627024	748.6514893	38.0545568
LYPD6B	20.5110924	14.2202756	113.3315794	119.678326	470.5521474	65.23638308
PDCD4	1196.48039	979.167549	3048.920365	4623.20389	1117.435399	4526.680137
ADRA2A	0	0	0	4.02280086	0	11.77879139
GPM6A	3.90687474	0	6.017605984	2.01140043	2.015212623	4.530304381
WDR17	129.903585	147.281426	0	0	136.026852	8.154547886
SPATA4	0	1.01573397	0	1.00570022	0	2.718182629
VEGFC	966.951497	1215.83356	161.4724272	280.59036	91.69217433	167.6212621
CCDC102B	2.93015605	8.12587178	33.09683291	13.0741028	4.030425245	28.08788716
CD226	17.5809363	11.1730737	12.03521197	1.00570022	0	20.83940015
CNDP1	0	0	1.002934331	0	2.015212623	3.624243505
FSIP1	9.76718684	14.2202756	449.3145802	34.1938073	20.15212623	71.57880922
DLG2	8.79046815	16.2517436	20.05868661	32.1824069	16.12170098	52.55153082
CCDC83	0	0	1.002934331	1.00570022	337.5481143	15.4030349
RGS18	0	0	0	0	0	0
PRSS23	10256.5229	9292.95011	2977.712028	3024.14055	5212.347448	3211.079745
MTMR12	2808.06622	2549.49227	1621.744813	2232.65448	1447.930269	2006.01878
PPP1R1C	4.88359342	11.1730737	0	0	27.2053704	3.624243505
RP11-134C1.1	0	0	0	0	0	0
C11orf53	0	0	0	0	0	0.906060876
CCT5	15004.3524	14285.2826	5552.244455	4935.97666	14511.5461	8222.502451
FAM173B	368.222944	362.617028	227.6660931	226.282548	598.5181489	314.403124
DOCK1	2720.16153	2565.74401	1580.624505	1420.0487	990.477004	1714.267178

DIXDC1	136.740616	157.438766	720.1068494	842.776781	105.7986627	886.1275369
DLAT	3683.20616	3439.27523	1426.172618	2268.85969	1626.276586	2041.355154
PIH1D2	39.0687474	55.8653685	40.11737323	39.2223084	12.09127574	65.23638308
C11orf57	1090.01805	1099.02416	825.4149542	779.417667	393.9740677	1036.533642
TIMM8B	845.828613	815.63438	948.7758769	693.933149	687.1875043	796.4275102
IL18	209.017798	218.382804	13.0381463	45.2565097	14.10648836	35.33637417
TEX12	0	1.01573397	0	1.00570022	2.015212623	1.105394269
PTS	598.728553	751.643139	447.3087115	426.416891	306.3123186	300.8122109
PIP4K2A	1453.3574	1580.48206	717.0980465	1148.50965	2031.334324	1264.860983
C2orf50	1.95343737	0	2.005868661	2.01140043	14.10648836	1.812121752
FREM2	186.553269	183.847849	147.4313466	257.459255	6407.368533	408.6334552
FOXO1	269.574357	242.760419	268.7864006	616.494232	205.5516875	263.663715
CRIM1	17652.2368	10098.4272	55.16138819	33.1881071	1516.447498	472.9637774
SEC24D	1770.79097	2398.14791	1978.789434	1172.64645	1344.146819	1244.021583
ABCB9	403.384816	482.473637	616.8046134	899.095993	710.3624495	1106.30033
RILPL2	679.796204	764.847681	393.1502576	620.517033	252.9091841	532.7637952
DHX37	1156.43492	1218.88077	572.6755028	714.047153	2153.254687	1539.397429
UBC	10373.7291	11357.9373	16087.06666	18169.9858	11712.41576	13034.59176
ITPR1	319.38701	561.700887	7579.174737	13836.4236	190.4375928	9296.18459
TKTL2	0	0	0	0	0	0
PRSS53	93.5598827	85.3216537	94.04515219	94.1938822	116.9427885	85.16972236
SLC7A11	2515.05061	2537.30346	5989.523823	494.804506	2178.444845	1128.045791
NOCT	423.895909	372.774368	143.4196093	161.917735	369.7915162	311.6849414
ENKUR	2.93015605	0	0	0	2.015212623	0.906060876
GPR158	0.97671868	2.03146794	0	0	62.4715913	18.12121752
LYZL2	0	0	0	0	0	0.906060876
CACNA2D4	0	0	8.023474646	12.0684026	3.022818934	9.966669638
DCP1B	88.8814002	73.132846	356.0416874	341.938073	244.8483336	372.3910201
CACNA1C	0.97671868	0	0	1.00570022	20.15212623	1.812121752
KCNA6	0	0	0	0	0	0
THRB	6.83703079	12.1888077	1439.210765	1297.35328	391.9588551	1528.524698
NGLY1	835.094475	1035.03292	935.7377306	1183.70915	682.1494727	1629.097455
OXSM	247.109827	242.760419	441.2911055	278.57896	332.5100827	431.2849771

UEVLD	687.609953	731.32846	1698.970756	1894.73921	961.256421	2074.879406
TMEM86A	41.0221847	69.0699101	2026.930282	6515.9317	29.22058303	1123.515486
C12orf45	195.343737	208.225464	144.4225436	125.712527	286.1601924	183.024297
TMEM263	2744.5795	3024.85577	1680.917938	1293.33048	1103.328911	1311.070088
BTBD11	0	1.01573397	2.005868661	0	113.8595132	25.36970453
UBE3B	2173.19907	1967.4767	1290.776484	1279.25067	1450.953088	1586.512594
ANK3	65.4401518	113.762205	1303.81463	1367.75229	1486.219309	1751.415674
IPMK	882.95369	691.714835	794.3239899	1264.16517	539.9560701	1726.95203
RAD9B	7.81374947	15.2360096	73.21420614	62.3534134	8.262371753	49.02695401
PLBD2	4181.33269	4554.55113	739.1626017	1026.81992	2803.160758	1274.827653
DLG5	3985.98895	4352.42007	2518.368104	4359.71043	6701.589576	4002.07089
MAT1A	0	1.01573397	0	0	5.038031556	0
SLC2A13	294.969043	359.569826	313.9184455	485.753204	192.4528055	256.415228
GXYLT1	1711.21113	1727.76349	811.3738735	955.415205	1410.648836	1087.273051
TWF1	3846.1619	3888.82893	2372.471247	2988.83041	5685.912339	2987.255527
DIP2C	1390.84741	1337.72164	990.8991187	1314.45018	2348.730312	859.8517715
EIF4E	1622.54461	1601.24366	1738.656868	1749.07359	2061.39122	2073.302861
MAGI1	1781.53488	1509.38068	1302.811696	1826.35159	1089.222423	1820.2763
TEX30	823.37385	757.737543	196.5751288	200.134343	339.5633269	183.024297
CSNK1G3	1555.91286	1444.37371	739.1626017	870.936387	954.2031768	945.0214939
RP11-96C23.13	11.603418	3.66679964	15.26466051	13.5266679	3.687839099	9.196517893
SRFBP1	520.591058	500.756848	391.144389	291.653063	378.859973	388.7001159
AKAP6	65.4401518	78.2115159	296.8685619	692.927449	15.11409467	318.0273675
NPAS3	0	0	2.005868661	18.1026039	61.46398499	14.49697402
FAM177A1	933.743062	1058.3948	2445.153898	2135.10156	1158.747258	1572.01562
MBIP	472.731843	353.475422	456.3351205	537.043915	304.297106	560.8516824
MIPOL1	144.554365	177.753445	259.7599917	232.31675	158.1941909	299.0000891
EXT2	3431.21274	3518.50248	1911.592834	1959.10402	1548.6909	2218.037025
TMEM18	883.930409	883.688556	609.7840731	679.853346	1200.059117	568.1001694
ALLC	0	0	0	0	0	0.906060876
KCTD14	1275.5946	1117.30737	1.002934331	2.10191345	41.31185876	18.12121752
THRSP	0	0	19.05575228	19.1083041	2.015212623	19.93333928
NDUFC2	1651.63129	1721.66908	2598.602851	2396.81492	2304.395634	2498.281654

ME3	670.029017	493.646711	418.2236159	238.350951	48.36510294	221.9849147
MSGN1	0	0	0	0	0	0
ADAMTS12	5151.21434	4441.80466	4.011737323	5.02850108	83.63132384	43.49092206
NUBPL	274.45795	302.688724	383.1209143	283.607461	282.1297672	453.0304381
NEK7	2648.86107	2302.66892	1973.774763	2686.22528	1704.869879	2101.155172
ATP6V1G3	0	0	0	0	0	0
FER	1827.44066	1715.57468	539.5786699	537.043915	514.8868251	494.7092384
VIPAS39	502.033404	492.630977	897.626226	777.406267	851.427333	970.3911984
ANKRD50	1513.91396	1659.70931	2274.655062	3417.36933	5095.465116	3267.255519
UPF2	1600.84192	1569.30899	1208.535869	1036.87692	2063.577726	1420.703454
CDC123	2751.41653	2824.75618	1373.017099	1432.11711	1977.931189	1875.546014
SCLT1	387.757317	427.624002	431.2617622	486.758904	283.1373735	503.7698472
CCDC3	152.368115	96.4947274	0	1.00570022	0	0
C4orf33	70.3237452	142.202756	410.2001413	331.881071	72.54765441	339.7728286
FRMD4A	1575.44724	1766.36138	220.6455528	381.160382	535.0389513	337.9607068
SLC25A31	0	0	0	0	0	0
PTPRO	0	0	0	0	1.007606311	0
EPS8	1119.31961	995.419293	1491.36335	2443.85152	1556.751751	2938.355421
ACAD8	1113.4593	1031.98572	720.1068494	683.876147	408.0805561	871.6305629
THYN1	637.797301	753.674607	600.7576641	501.844408	187.4147739	522.7971256
VPS26B	1876.27659	1886.21799	3541.361122	3396.24963	1481.181278	3339.74039
NCAPD3	5481.34525	5003.50555	1885.516542	2350.3214	1887.246621	4189.625491
VTI1A	746.213074	766.879149	648.898512	843.782481	878.6327034	1002.103329
QDPR	660.26183	632.802265	735.1508644	504.861508	221.6733885	679.5456571
FAM160B1	1305.87288	1012.68677	721.1097838	760.309363	1142.625557	876.1608673
ANO4	0	0	0	0	0	0
TEX9	56.6496837	75.1643139	7133.871894	6131.75421	58.44116605	4861.016601
QTRTD1	767.700886	783.130893	538.5757356	456.587898	1493.272553	830.8578235
DRD3	0	0	0	0	0	0
MMAA	82.0443694	37.582157	225.6602244	179.014638	63.47919761	175.77581
ZNF827	585.054492	608.424649	554.6226849	420.38269	381.882792	498.3334819
POU4F2	0	0	0	0	0	0
EDNRA	12.6973429	9.14160575	20.05868661	1.00570022	30.22818934	12.68485227

NR3C2	293.992324	339.255147	112.328645	206.168544	20.15212623	139.5333749
AKR1C6P	0	0	0	0	0	0
AKR1C2	3.17433572	4.28639736	4.011737323	1.00570022	719.3200696	320.6187016
DPYSL4	0	3.04720192	15.04401496	21.1197045	203.5364749	41.6788003
VENTX	0	0	0	0	0	0
ADAM8	3312.05306	3125.41343	155.4548213	141.80373	195.4756244	196.6152101
ITIH2	0	0	0	0	52.39552819	21.74546103
KIN	354.548882	412.387993	430.2588279	360.040677	463.4989032	424.0364901
PIGF	217.808267	239.713217	418.2236159	429.433992	332.5100827	340.6788894
ANKAR	60.5565584	46.7237627	46.13497921	40.2280086	43.32707139	57.0818352
INPP1	1030.43821	1125.43324	49.1437822	60.3420129	76.57807966	79.7333571
MFSD6	1279.50148	1231.06957	1122.283516	1221.92576	661.9973465	1362.715558
RNF144A	4.88359342	8.12587178	17.04988362	21.1197045	123.9355763	19.93333928
ASAP2	4461.65095	3891.27685	1155.380349	1252.09677	2621.791622	1608.258055
ADAM17	2254.26672	2483.46956	632.8515627	802.548772	1291.751291	1046.500312
FLI1	125.99671	82.2744518	0	0	0	0
KCNJ1	0	4.06293589	4.011737323	0	0	2.718182629
TMEM45B	14.6507803	19.2989455	3604.545985	2403.62352	238.8026958	2662.912915
WWC2	2227.89532	1896.37533	446.3057772	486.758904	623.7083067	727.5668836
CENPU	1700.46723	1601.81247	1010.957805	1545.76123	594.4877237	1883.700562
ACSL1	1151.55133	1219.8965	15801.23038	21103.6133	504.810762	11830.43686
SLC25A4	795.049009	764.847681	1464.284123	1471.33942	446.3695959	1037.439703
AMN1	75.2073387	53.8339005	188.5516542	208.179945	66.50201655	144.9697402
BICD1	416.082159	489.583775	29.08509559	57.3249123	1008.613918	120.5060965
SAV1	2602.04694	2251.88222	1279.674001	1349.36809	612.3022032	1136.109733
CCDC122	92.788275	98.5261953	146.4284123	124.706827	163.2322224	115.9757922
SERP2	0	0	0	0	1.007606311	0
NBAS	1608.65567	1664.78798	1261.691388	1488.43632	1250.439432	1469.630741
ZNF385D	0	0	0	2.01140043	0	0
TDO2	0	0	15.04401496	59.3363127	4.030425245	166.7152012
GUF1	955.230873	1028.93851	1072.1368	620.517033	979.3933346	1125.327608
SLC35F4	0	0	0	0	0	0.906060876
GABRA2	0	0	0	1.00570022	1.007606311	12.68485227

SACS	4817.17655	4378.82915	29.08509559	284.613161	1550.706113	354.2698026
CCDC175	0	0	2.005868661	0	0	0
PABPC3	24.4570358	29.4969146	29.14527165	26.2085476	73.67617348	39.93010281
CENPJ	867.326191	775.005021	779.279975	906.135894	579.373629	1157.039739
FBXO4	154.321552	128.998214	313.9184455	269.527658	146.1029151	212.018245
C5orf28	261.760607	314.877531	332.9741978	175.997538	216.6353569	195.7091493
CCL28	94.7417123	114.777939	464.3585951	610.460031	64.48680392	517.3607603
PARP8	584.077773	636.062771	26.0762926	36.2052078	143.0800962	86.98184411
GFRA1	10.7439055	4.06293589	0	0	9904.77004	292.657663
CACUL1	3262.2404	3329.57596	2138.255993	2739.52739	2194.566546	3196.582771
DST	33526.8455	24575.6835	2142.26773	3747.239	5207.309417	2392.906774
BEND6	102.555462	120.872343	36.10563591	34.1938073	110.8366942	13.59091314
TIAL1	3606.04538	3315.35569	3082.017198	2613.81486	2691.316457	2959.194822
BAG3	3967.43129	3965.42543	1755.135079	1565.87524	9331.442049	2027.764241
GLT1D1	0	0	0	0	0	0
TMEM132D	0	0	0	0	0	0
RBM46	0	0	0	0	1.007606311	0
ZNF37CP	0	0	0	1.00570022	0	0
SCHIP1	57.9584867	45.2814205	8.986291603	25.5648995	5.430998018	15.66579255
MCHR2	0	0	0	0	2.015212623	0
KCNE4	0	0	0	2.01140043	3.022818934	9.966669638
AP1S3	345.758414	290.499916	155.4548213	167.951936	459.4684779	265.4758367
RABGAP1L	514.730746	578.968364	259.7599917	413.342789	482.6434231	360.6122287
CCDC74B	29.4676027	33.2449729	2.005868661	9.05130194	299.9039425	45.13995285
TMEM56	549.257752	565.255956	507.7555636	771.82463	179.7166617	705.6220892
MZT2B	2662.74024	2414.93799	2560.772168	2234.1932	3301.915806	1773.043347
TUBA3E	0	0	0	0	4.030425245	0
ASTN1	0	1.01573397	0	0	1.007606311	0.906060876
CFC1B	0	0	0	0	0	0
FAM168B	6964.00422	6956.76198	3950.558329	3651.69748	6706.627608	4425.201319
PTPN14	10170.5717	8445.82798	1279.744206	2298.02499	961.256421	5198.062186
AC093838.4	764.585153	565.438788	888.0181151	709.420932	943.5628541	924.7982151
MGAT5	3331.58743	3425.05495	1277.738337	1218.90866	3524.606877	2065.818798

TMEM163	54.6962463	50.7866986	0	0	9.068456802	2.718182629
GPATCH11	695.062317	845.090665	469.3732668	436.473894	572.3203848	507.3940907
HSPB8	20.5110924	42.6608268	0	0	1428.785749	330.7122198
GEMIN6	377.990131	468.253361	283.8304156	244.385152	534.031345	308.0606979
TMEM178A	5.8603121	6.09440383	57.16725685	83.4731179	1.007606311	31.71213067
POU4F1	0	0	0	0	0	0
RNF219	617.286208	616.550521	290.8509559	278.57896	538.0617702	348.8334373
CYSLTR2	0	0	0	0	0	2.718182629
GRID2	0	0	0	0	0	0
ARL11	0	0	0	0	75.57047335	10.87273051
RIT2	0	0	5.014671654	0	0	0.906060876
SETBP1	1.95343737	3.04720192	999.9255277	1336.57559	214.6201443	1551.17622
ARL14EP	534.26512	521.071528	588.7224521	329.869671	319.4112007	340.6788894
EPG5	5112.14559	4593.14902	1390.066982	1768.02098	1134.564707	1798.530839
PSTPIP2	146.507803	123.919545	391.144389	453.570797	273.0613104	772.8699274
ATP5A1	13795.1747	13499.1045	10828.68197	11275.9108	7806.9337	14846.71352
HAUS1	1101.73868	1106.1343	322.9448545	445.525195	464.5065095	450.3122555
C18orf25	1626.23661	1485.00307	865.5323274	772.377766	963.2716336	1062.809408
SPC25	783.328384	776.020755	82.24061512	254.442155	477.6053916	270.0061411
G6PC2	0	0	0	0	0	0
PDK1	1709.2577	1276.7776	404.1825353	322.829769	553.1758649	435.8152814
PTH	0	0	0	0	0	0
PDE3B	77.160776	77.1957819	6.017605984	29.1653063	636.8071887	69.76668747
TCF7L1	112.322649	136.108352	2.005868661	3.01710065	331.5024764	18.12121752
TGOLN2	9651.93403	9134.49561	4230.377007	4178.6844	8292.599942	5735.365346
SH2D6	3.90687474	0	12.03521197	16.0912034	6.045637868	9.060608762
KCNK13	0	0	0	0	1.007606311	1.812121752
UHMK1	5266.46714	4937.48284	5579.323682	5829.03845	5369.534033	8244.247912
ATG10	88.8814002	114.777939	251.736517	227.288249	158.1941909	107.8212443
POC5	340.874821	369.727166	334.9800665	379.148981	383.8980046	389.6061768
SPOCK1	23.4412484	34.5349551	0	0	31.23579565	15.4030349
FAM151B	16.6042176	30.4720192	79.23181213	64.3648138	21.15973254	53.45759169
TADA1	301.806073	301.67299	382.11798	527.992613	552.1682586	545.4486475

GUCY1A2	0	1.01573397	1502.395627	2175.32957	0	3908.74662
CWF19L2	491.289498	482.473637	662.9395926	499.833007	249.8863652	482.0243861
JMY	856.582286	716.09245	1217.562277	766.343564	963.2716336	797.333571
HOMER1	642.680894	779.067957	676.9806732	629.568335	792.986167	742.9699185
XRCC4	470.778406	460.127489	204.5986035	221.254047	233.7646642	224.7030973
BOLL	0	0	0	0	0	0
ZNF547	46.8238937	29.3851838	85.68067987	41.907528	60.13394466	110.88373
ZNF773	19.5343737	15.0836495	305.1527995	176.168507	53.82632915	217.0197011
ZNF776	640.278166	597.942275	514.3147541	381.160382	466.0682993	494.5733293
ZNF256	64.4634331	37.582157	175.5135079	143.815131	146.1029151	209.3000624
SUV39H2	937.649937	985.261953	329.9653948	419.37699	903.8228612	657.8001961
DCLRE1C	460.0345	438.797076	182.5340482	297.687264	383.8980046	357.8940461
OLAH	2.93015605	2.03146794	0	0	0	0
RPP38	424.872627	397.151983	411.2030756	355.012176	573.3279911	410.4455769
NMT2	2116.54939	1672.91385	147.4313466	210.191345	566.2747469	173.0576274
ZSCAN1	0	0	0	0	0	0
ZNF837	47.8592155	47.7394967	55.16138819	67.3819144	147.1105214	76.1091136
USP12	1744.41957	1609.92819	1472.307597	1734.83287	1538.614837	1519.464089
CCDC50	4923.63889	3989.80304	1132.312859	1149.51535	1013.651949	1112.642756
CAMK4	408.26841	570.842492	0	0	44.3346777	8.154547886
TRIM36	235.389203	330.113541	63.18486283	105.598523	274.0689167	117.7879139
ZFP36L2	1618.42286	1879.10785	4131.086508	4790.15013	6425.505447	7732.323517
PAN3	1640.88739	1425.07476	1607.703732	1584.98354	1372.359796	1948.030884
PLEKHH2	302.782792	261.043631	0	1.00570022	218.6505695	45.30304381
PFKM	1437.7299	1907.5484	1336.911463	1763.99818	1558.766964	2075.785467
TMEM123	14911.5641	14279.1882	5552.244455	3991.62416	3355.329017	5212.568221
GRIA4	0	0	2.005868661	0	0	1.812121752
IGSF10	2.93015605	10.1573397	0	1.00570022	8.06085049	0.906060876
SPEF2	51.7660902	35.550689	246.7218454	99.5643213	46.34989032	119.6000357
SPARCL1	0	0	0	0	8.06085049	1.812121752
DSPP	0	0	0	0	0	0.906060876
DMP1	0	0	0	0	0	0
MEPE	0	0	0	0	0	0

MBNL1	5737.24555	5227.98276	1603.691995	2390.54941	3731.166171	2961.913004
CAPSL	0	1.01573397	4.011737323	4.02280086	0	6.342426133
NADK2	1079.27415	1008.62383	1298.799958	1184.71485	587.4344795	1432.482245
GPD1L	903.464783	827.823187	5243.340681	3718.0737	3331.146465	5208.943977
GJA1	9.76718684	11.1730737	0	0	139.049671	37.14849592
CCNO	208.04108	204.162528	512.499443	594.368827	178.3463171	633.3365524
DDX4	0	0	0	0	2.015212623	1.812121752
CLEC4F	4.88359342	6.09440383	0	0	0	0
SLC30A6	1336.15116	1331.62724	715.0921778	674.824845	1588.995153	1063.715469
PELO	1477.69723	1586.54599	727.1273898	769.360665	744.621064	754.7487099
RASGRP3	42.9756221	63.9912403	1.002934331	2.01140043	11.08366942	14.49697402
SAR1B	1287.31523	1514.45935	2955.135976	2017.43463	1837.390261	2572.306827
CATSPER3	0	6.33817999	15.8864798	14.120031	5.380617702	10.02103329
GPR180	533.288401	690.699101	349.0211471	454.576497	442.3391707	390.5122376
TCTEX1D1	0	0	0	0	0	0
WDR78	4.88359342	3.04720192	83.24354945	80.4560172	33.25100827	72.48487009
ANKRD22	0.97671868	7.11013781	155.4548213	276.567559	6.045637868	180.3061144
FARP1	3138.19713	3057.35926	784.2946466	848.810982	2318.502122	630.6183698
IFIT5	1251.17663	1076.67801	294.8626932	301.710065	227.7190264	553.6031953
SLC16A12	5.8603121	6.09440383	0	0	1.007606311	0
PANK1	206.771345	251.333214	220.3246138	794.171289	358.7481511	323.8895814
PRDM8	343.804977	361.601294	0	0	31.23579565	15.4030349
BMP3	0.97671868	0	0	0	0	1.812121752
HNRNPDL	6752.05626	5763.27456	5748.819584	5004.36427	4346.813627	5875.342691
HHEX	283.248418	289.484182	0	0	164.2398287	29.90000891
UTRN	4307.3294	3288.9466	2682.849335	4209.8611	971.3324841	5362.974326
GRM1	0.97671868	2.03146794	1.002934331	0	0	2.718182629
PTPRK	1958.32096	2444.87167	1215.556409	1513.57882	5022.917462	2339.449182
GGPS1	291.062168	541.386207	2330.819385	4034.86926	541.0845892	1746.885369
CNTNAP4	0	0	1.002934331	0	0	0
ZNF117	36.1385913	34.5349551	262.1168873	277.965483	642.3691756	548.7557697
PART1	0	0	69.00188195	43.8988144	1.007606311	9.060608762
RAB3C	0	0	0	0	1.007606311	0

LMNTD1	0	0	0	0	2.015212623	0
MARVELD2	99.6253058	94.4632594	992.9049874	1205.83456	1955.76385	1427.951941
RAD17	1316.61679	1300.13948	940.7524022	889.038991	782.9101039	928.7123981
MED21	637.797301	650.069742	428.2529592	586.323226	600.5333615	526.4213691
PLOD2	7611.5687	5926.80773	748.1890107	876.970588	3253.560779	1141.636704
STK32B	0	0	454.3292518	580.289024	0	263.663715
NRSN1	0	0	0	0	0	0
JAKMIP1	0	0	0	0	9.068456802	7.248487009
ZIC1	0	0	0	0	601.5409678	29.90000891
ADGRA3	1677.9441	1727.65176	371.0857024	474.690502	1041.824622	595.2819956
CPB1	0	0	0	0	0	1.812121752
SREK1IP1	823.37385	917.207777	758.218354	636.608236	400.0197056	613.4032132
LGI2	2.93015605	1.01573397	10.02934331	22.1254047	16.12170098	44.39698293
CWC27	874.163222	859.310941	886.5939483	557.157919	555.1910775	733.9093097
MR1	145.531084	142.202756	782.288778	547.100917	525.9704945	772.8699274
SRP19	1127.20173	1202.91343	1787.058478	1071.15119	1106.623783	1180.334564
CENPH	329.154196	403.246387	290.8509559	405.297187	652.9288897	493.8031775
CDYL	1704.3741	1796.8334	674.9748046	741.201059	1293.766504	1093.615478
CARHSP1	1669.21223	1236.14824	1070.130931	1695.61056	1411.656442	1441.542854
TEKT5	0	0	24.07042394	17.0969037	6.045637868	12.68485227
BANK1	23.4412484	23.3618814	25.07335827	14.079803	433.2707139	66.14244396
TXNDC11	578.217461	648.038274	1927.639784	1548.77833	1179.906991	1592.85502
DAB2	1053.87946	937.522456	5.014671654	25.1425054	66.50201655	57.98789608
ACMSD	0	0	0	1.00570022	0	0
ACOXL	3.90687474	13.2045416	5.014671654	2.01140043	31.23579565	5.436365257
BCL2L11	758.910417	638.896669	1810.296467	1331.54709	1345.154426	2306.830991
ANAPC1	3970.7619	3544.31228	1327.905113	1262.16383	4026.364592	2752.848518
CAST	9090.32079	8512.86642	2578.544164	2878.31402	2807.191183	2295.052199
SCOC	1585.21442	1595.71807	2206.455528	2114.98755	1260.515495	1895.479353
CLGN	612.402615	668.352954	5395.786699	1172.64645	83.63132384	727.5668836
CETN3	790.165415	750.627405	480.4055444	429.433992	328.4796575	404.1031508
SMARCA5	2838.3445	3196.51481	3373.871089	3756.29031	4726.681206	4686.146852
SYCP2L	118.182961	82.2744518	9.026408976	3.01710065	0	9.966669638

BMP6	5.8603121	6.09440383	0	0	25.19015778	0.906060876
RGPD3	18.6064909	19.136428	6.33854497	6.34596836	18.20744604	6.994789964
RASSF3	1578.37739	1773.47152	321.9419202	694.938849	936.0662632	713.9759704
HNRNPU	26009.7158	23340.2869	18104.85019	15645.0245	21136.28554	23393.13273
RANBP2	6967.76458	6751.45167	4318.434641	3658.44573	6617.998557	6141.806134
AHCTF1	5753.67396	5134.47429	4829.901062	5538.64251	3834.667491	7982.269471
MERTK	862.442598	953.7742	1.002934331	3.01710065	139.049671	17.21515665
TMEM87B	2232.77891	2558.63388	1793.246583	2395.57791	1516.447498	2470.828009
OR14K1	0	0	0	0	0	0
PTPRR	96.6951497	145.249958	0	0	4.030425245	0
NR4A2	49.8126529	39.6136249	50.14671654	81.4617175	93.70738695	76.1091136
CCDC148	25.3946858	29.4562852	19.05575228	19.1083041	12.09127574	52.55153082
PLA2R1	177.7628	191.973721	0	0	38.28903983	22.6515219
RBMS1	2477.8767	2182.79199	201.5898005	317.801268	1328.004966	611.5820308
SCN3A	8.79046815	18.2832115	0	0	0	0.906060876
FEZF2	0	0	0	0	0	0
CD96	0	0	0	0	2.015212623	0
SLC25A27	227.575453	212.2884	24.07042394	10.0570022	3.022818934	6.342426133
ADGRF1	1912.41518	2079.20744	5.014671654	0	5.038031556	7.248487009
ADGRF4	3.90687474	16.2517436	0	3.01710065	161.2170098	28.99394804
FRMD1	0.97671868	0	0	0	1.007606311	0
FAM49B	3302.28587	3386.45706	1661.862186	1659.40536	1924.528055	2023.233937
ASAP1	7239.43888	5188.36913	1097.210158	1801.20909	2258.045744	2210.788538
TRAPPC8	2069.66689	1928.87881	1388.061114	1591.01774	1278.652409	1428.858002
FAM81B	0	0	0	0	0	0
LINC00467	149.437959	145.249958	279.8186783	166.946236	150.1333404	289.9394804
INO80C	392.689747	490.254159	106.3110391	180.784671	311.3503502	170.3394447
LPCAT1	4033.84816	4439.77319	2590.579376	2326.1846	7371.647773	2582.273497
PLEKHG4B	2.93015605	4.06293589	0	0	20.15212623	3.624243505
NMRAL1	591.891522	566.779557	2011.886267	1691.58776	1262.530708	1774.973256
UBALD1	554.776212	510.914188	641.8779717	719.075654	866.5414277	1107.206391
C16orf89	0	0	3.008802992	6.03420129	0	0
TMEM251	535.241839	656.164146	922.7898483	982.569111	623.7083067	575.9738384

ING1	547.939182	603.34598	314.9213798	268.521958	560.2291091	309.8728197
TEX29	0.97671868	1.01573397	1.002934331	0	0	0
SPACA7	0	0	0	0	0	0
ADPRHL1	291.062168	353.475422	90.26408976	231.31105	442.3391707	125.0364009
CMTM7	1271.68773	1150.82659	2.005868661	4.02280086	72.54765441	29.90000891
FBXL2	414.128722	345.349551	271.7952036	207.174244	47.35749663	165.8091403
UBP1	3258.33353	3202.60921	3026.85581	2635.94026	3959.892803	3943.176933
RMND5A	2566.8167	2464.17062	4853.199226	4522.63387	2089.77549	3117.755475
CD8A	51.7660902	69.0699101	26.0762926	10.0570022	45.34228401	23.55758278
RPIA	1269.73429	1125.43324	328.9624605	304.727165	816.1611121	381.4516289
GOLGA8F	0	11.630154	0	0	0	0
PTPRD	0	0	0	1.00570022	1.007606311	0.906060876
LURAP1L	30.2782792	54.8496345	168.4929676	284.613161	506.8259746	128.6606444
CNKSR3	793.095571	497.709646	100.2934331	91.5187196	205.6222199	100.6271209
GTF2E1	251.016702	331.129275	537.5728013	560.17502	572.3203848	737.5335532
CFDP1	1090.80919	1165.86961	830.4296258	833.725479	1291.751291	772.8699274
TGIF2LX	53.7195276	65.0069742	0	0	7.053244179	2.718182629
ZDHHC7	2656.67482	2276.25983	1491.36335	1599.06334	3531.660121	1938.064214
FAM92B	0	0	0	0	0	0
C7orf31	110.369211	102.589131	0	0	6.045637868	4.530304381
TMPRSS11D	0	0	0	0	0	0
JAZF1	93.7649937	121.888077	2.005868661	1.00570022	115.8747258	31.71213067
CMIP	4938.28967	3629.21748	569.6666998	617.499932	2262.076169	1110.830634
SPHKAP	0	0	0	0	0	0.906060876
KCNJ16	0	3.04720192	0	0	2.015212623	0
PID1	216.831548	266.122301	0	0	13.09888205	9.966669638
TRIP12	6625.08283	6389.98242	4429.960939	5305.06864	7218.491614	7086.302113
FBXO36	74.23062	59.9283044	73.21420614	49.2793106	165.2474351	86.98184411
CEBPG	1439.68334	1213.8021	2927.565311	951.392404	1284.698047	1089.991234
KCTD15	0	0	1297.797024	619.511333	1394.527135	773.7759883
ZNF599	25.3946858	37.582157	142.416675	143.815131	57.43355974	122.3182183
MCOLN2	134.787178	242.760419	0	0	99.75302482	21.74546103
LGI4	0	0	5.014671654	0	0	0.906060876

DDAH1	3577.20288	2773.87806	2487.27714	2987.93534	778.8796786	3143.840968
SREK1	4021.15082	3120.33476	2771.107556	2462.95983	2521.030991	3257.28885
CHD1	3497.62961	3120.33476	1434.196093	1275.22787	2465.612644	1832.961153
CLCA3P	1.95343737	7.11013781	0	0	0	0
ANKFN1	6.83703079	7.11013781	5.014671654	0	0	0
DGKE	122.089835	127.982481	443.2969742	428.428292	1325.002299	552.6971345
HS2ST1	1845.94948	2056.07918	1207.532934	1338.58699	1345.063741	1552.408463
MSI2	265.667482	194.005189	1086.17788	982.569111	5199.248566	2011.455145
CACNA2D1	138.694053	150.328628	0	0	16.12170098	4.530304381
ZUFSP	232.459047	287.452714	430.2588279	467.6506	378.859973	484.7425688
HS3ST3A1	0	0	0	7.30138356	52.11339842	3.361485851
GDPD1	30.2782792	77.1957819	62.1819285	37.210908	49.37270925	26.27576541
NUS1	2510.73351	2410.7938	1982.209441	1503.37097	3024.904679	2055.815886
SEMA3D	80.0909321	75.1643139	0	0	326.4644449	21.74546103
PPP2R5E	2663.51185	2643.95553	2176.357468	2442.84582	3012.742871	2690.094741
ASB17	0	0	0	0	0	0
GRAP	0	0	0	0	1.007606311	0.906060876
SLC5A10	0	2.03146794	7.020540315	21.1197045	4.030425245	1.812121752
AK5	13.6740616	8.12587178	1.002934331	1.00570022	0	0
CABYR	29.3015605	19.2989455	112.328645	183.037439	98.74541851	144.0636793
IMPACT	576.264023	521.071528	1159.392086	726.115556	408.0805561	916.9336067
ANKRD29	455.150907	492.630977	0	0	10.07606311	13.59091314
SDHAF4	119.159679	114.777939	453.3263175	252.430754	106.806269	178.4939926
CHST9	1.95343737	1.01573397	3.008802992	2.01140043	0	0
THY1	1.95343737	1.01573397	0	0	0	0
DNAAF1	0	1.01573397	5.014671654	1.00570022	50.38031556	32.61819154
C16orf74	172.879207	160.485968	82.24061512	89.5073192	499.7727304	137.7212532
TBCEL	1724.8852	1672.91385	963.8198918	834.731179	413.1185876	822.7032756
JPH3	0	0	0	0	211.5973254	49.83334819
ANKH	1079.27415	1161.99966	222.6514214	256.453555	350.6469963	194.8030884
OTULIN	1653.58473	1648.53624	727.1273898	895.073192	1527.531168	1039.251825
UBASH3B	3624.60304	3527.64409	5.014671654	8.04560172	116.8823321	46.20910469
ROBO4	3148.94104	4094.42364	0	0	28.21297672	18.12121752

ROBO3	808.72307	782.115159	7.020540315	4.02280086	32.24340196	16.30909577
PANX3	0	0	0	0	1.007606311	0
TBRG1	1722.93176	1811.05367	697.0393598	590.346027	381.882792	562.6638041
NRGN	784.305103	880.641354	85.24941811	300.704364	153.1561593	164.9030795
FAM134B	17.5809363	26.4090833	387.1326517	329.869671	206.5592938	238.2940104
CDH12	0	2.03146794	0	0	2.015212623	0.906060876
GPR15	0	0	0	0	0	0
TOMM70A	3575.7671	3449.43257	3318.7097	2994.97524	3231.39344	4219.5255
ABI3BP	19.5343737	22.3461474	0	0	1.007606311	0
ANGPT1	23.4412484	18.2832115	0	0	4.030425245	1.812121752
CYP4Z2P	0	0	299.8773649	320.587058	2.015212623	560.3805307
PITPNC1	300.829355	332.145009	772.2594346	1425.07721	1215.173211	1224.088244
CC2D1B	1435.77647	1379.36673	2057.018312	1776.06658	975.3629093	1395.333749
CERS3	0	0	0	0	0	0.906060876
PRKCA	6432.66925	5692.17318	262.7687946	115.655525	882.6631287	247.3546192
LRRK1	1983.71565	1375.3038	63.18486283	52.2964112	250.8939715	107.8212443
CEP112	186.299322	188.926519	1.002934331	0	120.9127574	15.4030349
GAL3ST2	9.76718684	2.03146794	0	0	4.030425245	3.624243505
ABCA9	0	0	0	4.02280086	0	2.718182629
ABCA6	0.97671868	2.03146794	1.002934331	6.03420129	1.007606311	2.718182629
ABCA10	13.6740616	11.1730737	14.04108063	19.1083041	2.015212623	4.530304381
ABCA5	245.15639	256.980695	1005.943134	899.095993	942.1119011	428.5667944
ENPP3	0.97671868	2.03146794	132.3873317	42.2394091	1.007606311	49.83334819
C4orf19	25.3946858	28.4405512	209.6132751	340.932373	476.5977852	356.0819243
UCHL1	0	0	64.18779717	86.4902185	21.15973254	140.4394358
MIA3	2105.80548	2174.68643	9824.744704	5884.35196	1281.675228	4604.601373
DISP1	74.4161965	138.13982	100.2934331	209.185645	99.75302482	257.3212888
TNIK	93.7649937	175.721977	874.5587364	869.930686	51.38792188	1091.803356
TDH	0	0	4.011737323	9.05130194	31.23579565	24.46364366
FAM167A	173.855926	184.863583	49.1437822	18.1026039	45.34228401	35.33637417
NEIL2	222.69186	232.60308	47.13791354	57.3249123	452.4152338	183.9303579
PGM5	0.99625306	0	0	0	49.32232894	5.436365257
WNT3A	2.93015605	2.03146794	0	0	16.12170098	0.906060876

OBSCN	334.03779	214.319868	2304.743092	3960.44745	645.8756455	2970.973613
LONRF1	246.133108	297.610054	227.6660931	230.305349	735.5526072	187.5546014
TRIM11	1008.9504	1025.89131	3647.672161	4208.8554	1174.868959	3000.873622
ENAH	2529.70139	2106.63226	1686.935544	2284.95089	3873.238661	3698.540497
PPP1R3A	0	0	0	0	0	0
CCSAP	905.41822	948.69553	1130.306991	1520.61873	663.0049528	1574.733803
ASZ1	0	0	0	0	0	0.906060876
SH3RF1	1280.47819	1026.90705	1575.609834	1698.62766	542.0921955	1521.276211
GBP5	10.7439055	18.2832115	0	0	1.007606311	1.812121752
BUB3	7112.46546	6450.92646	8704.467056	7603.09363	3479.264593	6452.059499
GPR26	0	0	0	0	3.022818934	0
CCDC173	0.97671868	4.06293589	0	1.00570022	1.007606311	0
MMP21	0.97671868	1.01573397	4.011737323	3.01710065	0	0
C10orf90	4.88359342	9.14160575	0	0	0	0.906060876
FAM69A	885.883846	963.93154	72.21127181	78.4446168	161.2170098	144.0636793
ATP5G3	3458.56086	3923.78033	4110.024887	3345.96462	3438.96034	3748.373845
CNTNAP3B	65.137369	48.3286224	11.04230698	4.47536596	1.007606311	1.938970275
FAM27C	11.7206242	8.36964793	119.4494788	62.2930714	1.007606311	45.2939832
MAGED4	0.07813749	1.01573397	0	0	91.37981637	16.40876247
SRSF12	0	0	0	0	0	0
PDLIM3	126.973429	134.076884	0	0	10.07606311	0.906060876
SORBS2	108.415774	88.3688556	0	0	2.015212623	0
TCEB1	1141.78414	1152.85806	1540.507132	1382.8378	1090.230029	1318.318575
LY96	20.5110924	56.8811024	0	0	2.015212623	0.906060876
CEP170P1	56.4738743	56.0989873	16.82923807	16.1213745	10.4891817	12.47645826
PSMA8	0.97671868	0	0	0	0	0
TMSB4Y	0	0	0	0	0	0
CXADR	219.761704	325.034871	712.0833748	1260.14237	12.09127574	1492.282263
BTG3	1005.04353	871.499748	377.1033083	325.84687	44.3346777	359.7061678
C21orf91	450.267313	523.102996	354.0358187	475.696202	258.954822	516.4546994
CHODL	0.97671868	1.01573397	0	1.00570022	0	0
TMPRSS15	2.93015605	2.03146794	0	0	0	0.906060876
NCAM2	78.1374947	79.2272498	1554.548213	2800.8751	3568.941555	2234.346121

L3MBTL4	0	0	0	0	0	0
PDE1C	6.83703079	1.01573397	2.005868661	0	0	0
RABGEF1	1539.30865	1465.70412	750.1948794	748.24096	963.2716336	788.2729623
MRPL39	642.680894	684.604697	444.2999085	487.764605	562.2443217	470.2455947
JAM2	0	2.03146794	6.017605984	4.02280086	3.022818934	3.624243505
ATP5J	1553.95943	1583.52926	1592.659717	1565.87524	1331.047937	1504.967115
GABPA	1026.61924	1055.07335	721.7316031	808.774056	762.7579776	795.1227825
ADAMTS1	160.181864	110.715003	2003.862793	1179.68635	3.022818934	7204.090026
ADAMTS5	0	0	0	0	0	0
TSEN2	393.61763	465.206159	287.8421529	168.957636	596.5029363	345.2091938
SLFN13	545.614591	392.032684	2604.149078	2839.57444	27.3162071	3761.674818
WNT7A	0	0	0	0	0	0
XPC	547.939182	622.644925	1113.257107	916.192896	1887.246621	1004.821512
CCDC174	408.26841	382.931708	573.6784372	476.701902	682.1494727	472.0577165
FGD5	0	0	145.425478	324.84117	5.038031556	90.60608762
FLCN	1676.05903	2106.44943	1675.110948	2337.04616	804.9766821	826.445307
DPH3	1067.55352	1046.20599	544.5933416	339.926673	833.2904194	399.5728464
OXNAD1	460.0345	464.190425	421.2324189	248.407953	578.3660227	406.8213334
PLCL2	423.895909	406.293589	0	2.01140043	10.07606311	7.248487009
CXXC1	1038.25196	1213.8021	955.7964172	1087.16193	629.7539446	1157.039739
SKA1	816.53682	914.160575	284.8333499	451.559397	517.909644	589.8456304
PPP4R1	4966.61451	4333.12113	1321.867448	2012.40613	2021.25826	2202.63399
APCDD1	1.95343737	0	0	0	89.6769617	9.966669638
PIEZO2	0.97671868	7.11013781	0	0	0	1.812121752
CCDC144B	515.883274	363.348357	1.574606899	2.14214146	237.8253177	22.4431279
MPPE1	99.6253058	94.4632594	749.5028547	646.665239	346.6165711	612.1075461
CCDC144CP	39.4399005	33.1637142	2.527394513	8.25679877	42.16832413	6.306183698
USP43	263.714045	231.587346	84.24648378	125.712527	362.7382721	112.3515486
RAB6B	17.5809363	8.12587178	0	0	303.2894997	121.4121574
EME1	367.246225	347.381019	515.508246	382.166082	359.7154531	722.1305183
EPHB1	0	2.03146794	1.002934331	0	0	2.718182629
ACSS1	211.947954	216.351336	3.008802992	0	67.50962286	21.74546103
ANKRD40	2844.20481	2935.47118	3162.251945	2787.801	2238.901224	3025.337266

ZNF18	67.3935892	72.117112	90.26408976	93.53012	137.0344583	82.45153973
CA10	0	0	1.002934331	0	0	0.906060876
VOPP1	3522.87778	3242.04001	550.6109476	478.713303	2916.879206	862.5155905
SEPT14	0	1.01573397	0	0	0	0
APOOL	803.839477	716.09245	896.6232917	860.879385	850.4197267	713.0699096
DKK2	0	0	0	0	0	0.906060876
CYP2U1	599.705272	657.17988	97.28463008	88.501619	174.3158919	77.01517448
RSPH10B	0.97671868	0.32503487	4.152148129	17.6802098	0	6.433032221
FBXL18	676.866048	709.998047	392.1473233	584.311825	970.3248778	831.7638843
CNTNAP5	0	0	0	1.00570022	1.007606311	0
PROM2	89.8581189	60.9440383	3283.606999	5075.76899	1894.299865	4070.931517
UNC93B2	0	0	0	0	0	0
AK9	91.8115563	101.573397	179.5252452	262.487756	52.39552819	198.4273319
ODF1	0	0	0	0	0	0
KLF10	1256.06023	1286.93494	546.5992102	811.600074	2727.590285	935.0548242
PTPRN2	0	0	2.005868661	0	24.18255147	17.21515665
AZIN1	7389.85356	7288.90698	5698.672867	5695.28032	13034.39524	6108.662427
ATP6V1C1	2466.21468	3059.39072	1762.155619	2858.20001	3793.637762	2172.733981
TMEM55A	429.756221	650.069742	0	0	289.1830113	20.83940015
OTUD6B	614.356052	701.872175	383.1209143	358.029277	1532.569199	498.3334819
CDK19	725.701982	759.769011	470.3762011	716.058553	1184.945022	677.7335354
GTF3C6	743.282918	785.162361	724.1185868	888.03329	1143.613011	1022.036668
TTC39B	182.646394	187.910785	616.8046134	242.373752	355.6850279	206.5818798
AGPAT5	1267.78085	1259.51013	328.9624605	359.034977	1859.033644	569.0062302
MMS19	3327.68056	3069.54806	1865.457855	1580.96074	1501.333404	1889.136927
OR4K1	0	0	0	0	0	0
PI4K2A	1587.16786	1690.18133	650.9043806	995.643213	916.1257343	740.2517358
MARVELD1	996.253058	1027.92278	199.5839318	288.635962	844.3740889	588.0335086
ZFYVE27	584.077773	624.676393	644.8867746	674.824845	619.6778814	657.8001961
GOLGA7B	89.8581189	179.784913	1.002934331	5.02850108	118.8975447	16.30909577
GPR78	0.97671868	0	0	0	4.010273119	1.567485316
TRMT44	228.552172	237.68175	287.8421529	246.396553	201.5212623	393.2304203
SLC25A28	457.104344	414.419461	516.5111803	372.10908	441.3315643	552.6971345

HSPA13	2750.43981	3082.75261	1703.985428	798.525971	1014.659555	884.3154151
SAMSN1	2.93015605	1.01573397	0	0	0	0.906060876
USP25	1386.94053	1118.3231	608.7811387	805.565873	958.233602	781.9305361
GRAMD3	839.978068	1010.6553	340.9976724	695.944549	372.8143352	497.427421
ZCCHC10	342.828258	365.66423	286.8392186	279.58466	457.4532653	287.2212977
C16orf87	248.086546	229.555878	641.8779717	488.770305	486.6738484	531.8577343
MOV10	1533.44833	1210.75489	1209.538803	1351.66109	1398.55756	1739.636882
RHOC	4588.62438	4598.19722	5036.325006	6788.47645	3250.53796	5478.950118
PPM1J	47.8592155	46.1752664	132.6681533	96.5472207	58.44116605	131.378827
DBI	1893.85753	2490.5797	4239.403416	10151.538	3271.697693	6100.507879
SLC16A1	40.045466	39.6136249	216.6338154	116.661225	774.8492534	270.912202
HEATR3	607.519021	646.006806	1047.063441	857.862284	776.864466	1279.357957
TRIM74	0	7.72973553	25.38426791	23.473043	0	7.493123446
NIFK	1496.33302	1580.48206	606.7752701	624.539834	1692.778603	1012.069999
OXA1L	2590.25795	2431.66713	2345.8634	2217.56898	2527.076629	2395.624957
SLC7A7	38.0920287	40.6293589	0	0	4.030425245	0.906060876
MAGEC1	9.76718684	9.14160575	0	1.00570022	1.007606311	4.530304381
LARP1	13071.4261	13103.984	8472.789226	8269.87287	13680.27089	10258.42124
CNOT8	1716.09473	1644.4733	1572.601031	1960.10972	995.5150356	1302.009479
GRIA1	0	0	0	1.00570022	0	0
LRGUK	1.95343737	5.07866986	9.026408976	16.0912034	6.045637868	26.27576541
SETD9	120.136398	113.762205	41.12030756	117.666925	150.1333404	103.2909399
MIER3	1044.11227	949.711264	921.6966499	898.090292	1108.366942	1126.233669
NUP205	6853.635	5742.95988	1689.944347	2041.57144	6731.817766	3816.32841
ZKSCAN2	372.129819	369.727166	202.5927348	276.567559	450.4000211	458.4668033
C9orf85	181.669675	205.178262	147.4313466	129.735328	221.6733885	104.1970008
XAGE2	0	0	0	0	0	0.906060876
PIK3AP1	185.57655	168.611839	0	0	27.2053704	9.060608762
RBM45	227.575453	267.138035	294.8626932	311.767067	421.1794381	336.1485851
C10orf12	868.30291	815.63438	273.8010723	528.998313	555.1910775	749.3123446
TTN	4.88359342	10.1573397	2.005868661	0	0	5.436365257
VSIG4	0	0	0	0	0	0
PDIA4	9647.05044	10021.2314	6813.935843	5399.60446	7983.264804	5293.207639

KDM8	91.8115563	65.0069742	54.15845386	113.644124	67.50962286	98.7606355
PDZD9	0	0	0	0	1.007606311	0
OTOA	0.97671868	4.06293589	0	0	0.120912757	0
KCTD18	331.107634	342.302349	617.8075477	661.750742	533.0237387	495.6152993
FAM126B	692.493547	711.013781	1577.615702	1295.34188	1069.070296	1720.673028
ALS2CR12	1.95343737	0	5.014671654	3.01710065	5.038031556	8.154547886
ALS2CR11	57.6264023	80.2429838	0	0	4.030425245	0
TMEM237	922.129877	922.286447	414.2118786	483.741804	402.0349182	700.3850573
FZD7	1652.88149	2029.7412	103.3022361	187.06024	254.9243968	257.5568647
SPAG17	0	0	0	0	0	2.718182629
DEPTOR	4.88359342	7.11013781	558.6344222	291.653063	228.7266327	414.9758813
FMN2	2.93015605	0	0	0	0	0
RNF20	1135.92383	1164.03113	984.8815128	737.178258	1478.158459	626.9941263
CYLC2	0	0	0	0	0	0
PPARGC1B	367.246225	354.491156	379.109177	430.439692	486.6738484	639.6789786
ELMO1	0	0	1.002934331	0	17.12930729	12.68485227
SLC26A2	2063.80658	2143.19868	32.09389858	22.1254047	549.1454397	89.70002674
LSM11	416.082159	311.830329	209.6132751	243.379452	547.130227	279.9728107
MED7	285.201856	284.405512	298.8744306	287.630262	365.761091	283.5970542
SAXO1	0.97671868	0	3.008802992	13.0741028	0	5.436365257
RRAGA	1679.95614	1677.99252	1118.271779	1134.42984	1424.755324	716.6941531
SLC24A2	0.97671868	1.01573397	0	0	1.007606311	0
TRIM42	0	0	0	0	0	0
PXYLP1	166.042176	145.249958	349.0211471	254.442155	149.1257341	260.0394715
ADCY8	0	0	0	0	0	0.906060876
RASA2	964.021341	825.791719	518.517049	427.422592	375.8371541	501.9577254
RMND1	241.249515	236.666016	247.7247797	262.487756	1140.610344	354.2698026
RAET1L	9.36673218	3.19956201	0	0	15.84964728	5.04675908
SLA	0	0	0	0	0	0
TMBIM4	456.645286	423.957203	924.093663	1008.05355	729.6883385	580.939052
VBP1	1305.87288	1296.07655	4756.917531	5010.39847	2870.670381	4038.313325
RAB39B	0	0	2.005868661	6.03420129	278.0993419	16.30909577
CLIC2	2.93015605	3.04720192	133.390266	124.706827	5.038031556	112.3515486

AFF2	0	1.01573397	0	0	18.1369136	1.812121752
MICU3	101.578743	77.1957819	0	0	1.007606311	3.624243505
GRIP1	125.99671	104.620599	96.28169575	151.860733	427.225076	196.6152101
VPS37A	478.592155	525.134464	575.6843058	425.411191	1022.720406	570.818352
KIF5A	0.97671868	2.03146794	1.002934331	2.01140043	8.06085049	0.906060876
NAT2	0	1.01573397	0	0	0	0
MAGEA8	0	0	0	0	0	0
PSD3	1347.87178	1167.07833	1142.342203	1173.65215	1768.349076	1127.13973
CARNMT1	665.145424	772.973553	524.534655	553.135119	664.0125591	600.7183609
MCU	1170.10898	939.553924	237.6954364	361.046377	854.450152	462.0910469
ELMSAN1	3237.82244	2676.45902	1737.082261	1767.01528	4007.2503	2553.279549
CFAP70	24.4179671	25.3933493	76.22300913	39.2223084	5.038031556	64.33032221
GNA14	0.97671868	1.01573397	4.011737323	6.03420129	61.46398499	12.68485227
FAM161B	193.273093	190.957987	453.4366403	350.677608	118.8975447	438.2888276
GNAQ	1465.07803	1680.02399	0	0	2317.222462	178.4939926
WIF1	0	0	0	0	2.015212623	1.812121752
UGT2B4	0	0	0	0	0	0
GPR61	0.97671868	3.04720192	0	0	1.007606311	1.812121752
MMP16	33.2084353	73.132846	514.5053117	866.913586	685.1722917	1357.279193
ADK	2017.44174	2056.86129	810.3709392	1026.81992	1674.641689	910.5911806
KCNMA1	3.90687474	4.06293589	8626.238178	5946.70537	19.14451991	7432.417367
BATF	38.0920287	69.0699101	100.2934331	131.746728	461.4836906	154.9364098
DCK	985.509152	972.057411	831.4325602	1053.97383	921.9597748	964.0487723
ADAMTS3	63.4867145	89.3845896	1.002934331	0	18.1369136	11.77879139
ALX3	0.97671868	3.04720192	1.002934331	0	0	0.906060876
DPY19L4	1797.16238	1728.77922	532.5581296	602.414429	2581.48737	863.476015
NDUFAF6	397.524504	381.915974	525.5375893	596.380228	671.0658033	759.2790142
DRAM2	444.407001	439.81281	1100.218961	831.714078	350.6469963	1089.085173
C8orf37	89.8581189	86.3373876	237.6954364	245.390853	133.0040331	204.769758
PPEF2	0	0	1.002934331	1.00570022	0	0.906060876
CFAP161	0	0	0	7.03990151	1.007606311	2.718182629
ADAMTSL3	14.6507803	9.14160575	24.07042394	24.1368052	36.27382721	31.71213067
ART3	0	0	0	0	0	0

SLC28A1	0	0	0	0	19.14451991	15.4030349
WHAMM	559.659806	513.96139	285.8362843	336.909572	495.7423052	298.0940283
CXCL13	0	0	1.002934331	0	0	0.906060876
N6AMT1	194.367018	226.508676	448.3116458	369.091979	247.8711526	362.4243505
RWDD2B	425.849346	394.104781	431.2617622	403.285786	796.0089859	696.7608138
USP16	1688.7466	1737.92083	1299.802893	1398.929	1188.975447	1139.824582
CCT8	8119.46242	7970.46448	5427.85051	4644.3236	6142.368074	5913.850278
MAP3K7CL	83.0210881	111.730737	0	0	5.038031556	5.436365257
NAA11	0	0	0	0	0	0
BACH1	2572.67701	2377.83323	656.9219866	805.565873	571.3127785	717.6002139
CLDN17	0	0	0	0	0	0
CLDN8	0	0	303.8891022	137.78093	0	217.4546103
TSPAN7	0	5.07866986	11.03227764	5.02850108	4.030425245	15.4030349
TIAM1	839.001349	780.083691	1142.342203	1393.9005	503.8031556	1993.333928
SCAF4	1814.74331	1809.0222	1490.360415	1316.46158	1560.782176	1640.876247
RPGR	389.710755	375.82157	273.8010723	164.934835	117.8899384	174.8697491
CDK20	10.7439055	7.11013781	194.5692602	152.866433	228.7266327	122.3182183
PCGF6	972.811809	979.167549	225.6602244	175.997538	446.3695959	313.4970632
ANKRD9	1139.8307	1494.14467	1611.715469	2201.47777	1176.884172	1486.845898
SFR1	196.320455	267.138035	69.20246882	126.718227	143.0800962	100.5727573
SORCS3	0	0	0	0	0	0
SFXN2	708.121046	690.699101	205.6015378	237.345251	836.3132384	534.5759169
C14orf2	1406.4749	1485.00307	2141.264796	2075.76524	2780.993419	1889.136927
FUT6	0	0	9.176849126	1.00570022	1.007606311	9.568002852
TDRD9	0.97671868	2.03146794	0	0	2.015212623	0
FGF18	4.88359342	4.06293589	2.005868661	1.00570022	53.4031345	20.83940015
PCDH1	311.57326	228.540144	1539.504198	1823.33449	749.6590956	2067.630919
SH3RF2	877.093378	842.043463	1.002934331	0	68.51722917	15.4030349
GDF6	0	0	0	0	2.015212623	0
UQCRB	2985.82902	3093.92568	5065.821304	4675.5003	4263.182303	3702.16474
MTERF3	664.168705	638.896669	524.534655	330.875371	625.7235193	429.4728553
PTDSS1	3726.18178	4098.48658	1492.366284	2205.50057	4522.137125	3120.473658
PPP2R2B	0	0	0	1.00570022	22.16733885	3.624243505

RPL30	10187.1759	10900.857	17385.86662	13793.1785	25598.23834	11000.4851
KCNS2	0	0	0	0	1.007606311	0
FAM122C	51.7660902	48.7552307	76.22300913	54.3078116	119.905151	88.79396587
SUPV3L1	1147.64445	1019.79691	720.1068494	511.90141	745.6286704	989.4184768
FAM122B	1127.13336	1383.42967	1692.95315	2264.83689	2950.271279	2153.706703
EEF1A1	256452.21	251112.719	199309.2782	156741.677	185771.1866	128937.5638
FBXO43	159.205145	173.690509	41.12030756	99.5643213	24.18255147	81.54547886
HKDC1	80.0909321	180.800647	5.014671654	2.01140043	0	1.812121752
HK1	7565.66293	7675.90163	7798.817356	10020.7969	5613.37476	9292.560346
TYSND1	907.371657	991.356357	763.2330257	759.303663	1363.291339	762.9032577
PHF6	1695.58364	1460.62545	2719.957905	1941.00142	1338.101181	2532.440149
CD109	0.97671868	6.09440383	3.008802992	6.03420129	265.0004599	46.20910469
LRFN2	0	0	212.6220781	115.655525	19.14451991	60.7060787
NODAL	0	0	1.002934331	0	0	0.906060876
PRG3	0	0	0	0	0	0
UBE2L6	739.376044	878.609886	392.1473233	294.670163	84.63893015	1668.058073
ZDHHC5	4131.52003	4513.92177	5852.12182	5095.88299	2580.479763	6835.32325
MED19	443.430282	500.756848	984.8815128	660.745042	272.053704	584.4092651
ZFAND3	6256.85989	5202.58941	2357.898611	1704.66187	2164.338357	2441.834061
NPTN	2989.73589	3206.67215	1017.978346	1186.72625	2610.707953	1104.488208
KAT6B	992.346183	1023.85984	1767.170291	2144.15286	2153.254687	1424.327697
SAMD8	1855.7655	1820.19528	922.6995843	966.477907	789.9633481	920.5578502
RAB11FIP1	1979.80877	1731.82642	1337.914397	1593.02914	2448.483336	1979.743014
UNC5D	0	0	0	0	0	0
GLYATL2	0	0	1.002934331	0	0	0.906060876
UTP14A	1055.07106	990.594556	684.3422112	630.312553	1305.253216	732.1515516
AIFM1	966.951497	929.396585	2951.635735	1977.20662	2366.867225	2263.340069
MAPK13	3406.79477	4154.35195	2898.480216	3509.89375	648.8984645	2764.391733
BAG4	748.166512	699.840707	614.7987447	641.636738	577.3584164	655.0820135
MS4A1	0	0	0	0	0	0
IGKV1OR-2	0	0	0	0	0	0
TBC1D31	1058.76305	1035.03292	323.9477888	287.630262	694.2407485	332.5243416
WDYHV1	599.705272	585.062768	335.9830008	651.69374	741.5982451	362.4243505

ATAD2	8592.19426	7224.91574	2614.6498	4711.70551	11385.95132	7381.677958
FBXO32	1575.44724	2393.06924	1323.873317	1730.81007	268.0232788	2045.885458
NSMCE2	141.624209	157.438766	508.4877057	652.69944	887.7011602	405.0092117
ZNF689	409.245129	349.412486	327.9595261	487.764605	769.8112218	731.1911271
PRR14	742.3062	860.326674	2085.100474	2001.34343	1344.146819	2118.370329
FBRS	2091.1547	2231.56754	5033.727406	5590.6875	3837.97244	6463.629897
FRRS1	79.1142134	101.573397	184.5399169	196.111542	64.48680392	255.5091671
PHKG2	442.795415	481.285228	1002.603362	1417.63502	1186.133998	1402.609418
HIAT1	2189.80329	2642.9398	1049.06931	2104.93055	878.6327034	1390.803445
SASS6	834.117756	869.46828	378.1062427	425.411191	415.1338003	682.2638398
COX6A2	0	0	0	0	0	0
ITGAD	0	0	0	0	0	0
ADGRG4	0	0	0	0	0	0.906060876
ZIC3	0	0	92.26995843	59.3363127	26.19776409	67.04850484
MALSU1	630.96027	600.298778	299.8773649	299.698664	410.0957687	320.7455502
VPS8	798.955883	866.421078	1561.568753	1725.78157	872.5870656	1662.621708
GALK2	252.970139	288.468448	1203.521197	1307.41028	364.7534847	869.8184411
LHFPL4	387.757317	340.270881	0	2.01140043	6.045637868	0.906060876
B3GNT7	44.9290595	46.7237627	1.002934331	0	4.030425245	2.718182629
MPV17L	0	0	1226.588686	949.381003	0	1221.370061
BUB1B	2405.65812	2529.17759	723.1156524	1824.34019	1593.025578	2186.324894
PDE6D	503.986841	530.213134	274.8040066	306.738566	473.5749663	310.7788805
EIF4A2	8922.32518	7602.76878	10815.95473	10070.6998	4900.997098	10480.27931
BRPF1	1073.41383	1104.10283	708.0716375	676.836245	1641.390681	941.3972503
RPUSD3	847.791818	891.814428	703.0569658	683.876147	1066.047477	605.2486653
SST	0	0	0	0	0	0
TATDN2	1971.65317	1754.78201	1309.140211	1646.82405	3655.00121	1971.878406
GHRL	2.93015605	1.01573397	4.011737323	0	2.015212623	6.342426133
SEC13	2678.16263	2870.46421	2569.517755	2153.20416	4262.174697	2273.306738
FAM92A1P1	3.92640911	3.08783128	0	0	4.080805561	0
EXOG	401.431379	300.657256	145.425478	150.855032	307.3199249	195.7091493
NTAN1	1016.76415	970.025943	392.1473233	550.118018	663.0049528	525.5153082
SHCBP1L	0	0	0	0	0	0

NMNAT2	254.923576	355.50689	0	6.03420129	66.50201655	28.99394804
ZFYVE9	1035.3218	1077.69374	747.1860764	965.472207	500.7803367	1017.506364
ATP2B2	3.90687474	2.03146794	0	0	1.007606311	0.906060876
LYZL4	0.97671868	2.03146794	2.005868661	1.00570022	0	0.906060876
SLC6A1	0	1.01573397	0	0	0	0
SMG1	6749.12611	5747.77446	5940.821332	5896.7623	10860.04128	8790.348924
FCHO2	1083.18102	990.340623	723.1156524	855.850883	493.7270925	840.8244931
RBPMS	188.506706	251.902025	120.3521197	226.282548	1818.729392	123.2242792
TMEM171	452.220751	529.1974	0	1.00570022	11.08366942	0.906060876
KLHL40	0	0	0	0	0	0
C8A	0	0	0	0	0	0
TIMP4	24.4179671	78.2115159	0	0	23.17494516	3.624243505
SYN2	0	3.04720192	0	0	0	0.906060876
NRG1	2170.26892	1785.66032	0	0	46.34989032	15.4030349
C1orf27	501.056685	423.561066	1388.061114	1017.76862	882.6631287	1155.227617
CPT2	279.341544	280.342576	1323.873317	1073.08213	904.8304675	1514.027724
NECAP2	1347.87178	1326.54857	747.1860764	823.668477	636.8071887	895.1881457
LRP8	1468.9849	1848.63583	525.5375893	2420.72042	2002.113741	1185.127626
CDCP2	0	1.01573397	0	0	0	0.906060876
PAXIP1	1368.38288	1200.59756	660.9337239	859.873684	1327.017512	1101.770025
STEAP2	116.229523	177.753445	0	0	87.66174908	45.30304381
SSBP3	623.14652	784.146627	1543.455759	1454.24251	1087.197134	1565.673194
HTR5A	0	0	0	0	0	0
CLDN12	1371.31303	1784.64459	586.7165835	664.767842	603.5561805	585.315326
MMP14	12278.3306	14131.9068	19.05575228	29.1653063	523.9552819	333.4304024
FZD1	919.092282	1202.62902	646.8926433	733.155457	643.8604329	426.7546727
GATAD1	659.285112	736.40713	809.3680049	644.653838	1545.668082	683.1699006
SUSD3	216.831548	173.690509	0	0	137.0344583	33.52425242
RP11-66N24.4	73.185531	70.0856441	257.112245	138.605604	27.8905427	170.3938084
TMED6	1.95343737	3.38239413	0	5.02850108	5.038031556	1.812121752
CLEC18A	13.4787178	15.0734921	6.017605984	35.6017876	25.44205936	6.242759437
DHRS4	245.15639	337.365882	505.7095776	567.818342	645.4020706	393.2576021
C1orf158	0	0	5.014671654	0	0	0

CLEC18C	0	0.16251744	0	0	2.357798768	5.427304648
ARMC12	15.6274989	21.3304134	12.03521197	8.04560172	2.015212623	2.718182629
DDX19B	483.270638	449.32008	651.8772269	557.007064	817.5919131	763.129773
ST3GAL2	1561.77318	1754.17257	47.13791354	56.3192121	210.5897191	105.1030616
FUK	156.274989	139.155554	481.4084787	463.627799	573.3279911	607.9668479
IL34	2.93015605	2.03146794	1.002934331	0	1.007606311	0
DHRS1	512.777309	472.316297	685.0041479	478.713303	280.1145545	519.1728821
CACNA1D	0	0	2080.085802	3248.4117	105.7986627	1825.712665
ARSE	82.0443694	125.951013	0	0	1.007606311	0
KIT	0	0	0	0	4.030425245	0.906060876
HYDIN	81.0676508	91.639519	3.008802992	4.02280086	6.045637868	8.154547886
AASDH	263.714045	275.263906	365.0680964	317.801268	209.5821127	403.1970899
ZNF19	30.1220042	32.9097807	97.60556906	66.9092353	33.4626056	100.8445755
CACNA2D3	0	0	5.014671654	2.01140043	0	2.718182629
RNF111	822.397132	747.580204	1078.154406	1185.72055	643.8604329	1417.07921
CCNB2	2089.20126	1983.72845	892.6115543	2421.72612	1321.97948	2483.512862
FAM81A	92.788275	117.825141	1.002934331	1.00570022	63.47919761	13.59091314
MYO1E	1617.44614	1263.57306	434.2705652	570.232022	652.9288897	762.9032577
APPL1	2903.78465	2647.00273	1718.026509	2106.94195	1442.892238	2338.543121
MUM1L1	0	0	46.13497921	143.815131	1.007606311	43.49092206
AFAP1L1	715.934795	468.253361	0	0	42.31946507	12.68485227
TSC22D3	91.8115563	166.580371	9709.407256	8515.26373	1758.273013	3244.603998
DSCR3	1995.43627	2283.36997	803.3503989	1473.35082	750.6667019	1038.345764
DYRK1A	1974.92518	2026.38927	2226.514214	2880.32542	1835.858699	2399.2492
KCNJ6	0	1.01573397	0	0	1.007606311	0
KCNJ15	0.97671868	3.04720192	0	0	0	3.624243505
ERG	0	0	0	0	71.5400481	5.436365257
ETS2	2104.82876	1683.07119	213.6250124	162.923435	779.8872849	327.9940372
TSPAN18	0	0	0	0	0	0.906060876
LCA5L	5.8603121	10.1573397	37.10857024	19.1083041	135.0192457	31.71213067
SLC35B2	2338.26453	2869.44847	1463.281189	1793.16348	1808.461884	1746.223945
TMEM164	1548.09911	2158.43469	1130.306991	2030.50874	3577.002405	2176.358225
MX1	385.80388	395.120515	35.10270157	42.2394091	46.34989032	54.36365257

CREB3L1	1424.05584	1524.61669	2458.192045	1564.86954	54.41074081	871.6305629
C2CD2	1185.73648	1213.8021	14.04108063	9.05130194	681.1418664	86.07578324
TAB3	1366.42944	1290.99788	1896.548819	2291.99079	1496.295372	1812.121752
SLC38A10	1609.63239	2180.78084	3265.554181	2936.64463	3646.527241	2831.440238
C9orf43	8.79046815	11.1730737	3.008802992	15.0855032	42.31946507	8.154547886
PALM2-AKAP2	315.499669	318.056779	0	13.969176	54.68279451	19.57091493
ZNF618	1074.39055	1009.63957	0	0	1960.801882	106.9151834
DGKI	8.79046815	17.2674775	0	0	3.022818934	5.436365257
C9orf91	335.991227	352.459688	2793.172111	2179.35237	562.2443217	1609.164116
SVOPL	3.90687474	6.09440383	1.002934331	1.00570022	2.015212623	3.624243505
SNX22	63.4867145	70.0856441	117.3433167	97.5529209	92.69978064	68.86062659
UBN2	855.605567	733.359928	1079.15734	1020.78572	1793.539234	1384.461019
BRAF	1204.29414	1154.88953	1002.934331	956.420905	630.7615509	1181.503383
SLC34A2	0.97671868	2.03146794	0	0	2.015212623	0
ACAN	0	0	0	0	0	0
PSMG3	581.147617	646.006806	809.3680049	634.596836	1231.294912	1028.379094
CABP1	1.95343737	5.07866986	0	1.00570022	3.022818934	1.812121752
WDR19	607.519021	563.732355	335.9830008	329.869671	199.5060496	262.7576541
SLC37A3	1565.68005	1421.01183	477.3967414	545.089517	865.5338214	647.8335265
AP3S2	637.699629	598.917379	706.7176761	518.126694	1272.727684	720.7623664
FMNL2	3459.53758	3736.88528	126.3697257	150.855032	447.3772022	257.3212888
GAREML	86.9279629	75.1643139	1.002934331	2.01140043	46.34989032	5.436365257
SPPL3	1994.45955	2056.86129	898.6291603	1258.13097	1496.295372	1061.903347
DPYSL5	0	0	0	0	233.7646642	7.248487009
DRC1	0	2.03146794	0	2.01140043	0	0.906060876
RAB28	445.38372	485.520839	277.8128096	287.630262	249.8863652	262.7576541
FAM213B	1043.13555	953.7742	725.1215211	730.138356	1026.750831	920.5578502
TNFRSF14	257.853733	286.43698	52.1525852	165.940536	4.030425245	18.12121752
PANK4	563.566681	536.307537	1090.189617	813.611474	489.6966673	766.5275012
CIB4	0	0	0	0	0	0.906060876
MEGF11	7.81374947	10.1573397	1.002934331	0	17.12930729	3.624243505
C12orf43	788.211978	820.71305	561.6432252	407.308587	716.4080873	599.8123
PEX10	453.197469	423.561066	909.661438	816.628575	616.6550625	781.0244753

RER1	2327.52062	2292.51158	3168.269551	2595.71226	2169.376388	2859.528125
RADIL	96.6951497	73.132846	23.06748961	28.159606	105.7986627	9.060608762
SKI	3034.66495	3206.67215	2085.100474	2012.40613	1390.49671	2391.094652
WIPI2	2055.01611	1990.83859	1517.439642	1455.24821	1947.703	1674.400499
SSX8	0	0	0	0	0	0
LDLRAP1	920.069	935.490988	1717.023574	1666.44526	644.8680392	1371.776167
AGAP1	1907.53159	1754.17257	3045.911562	2933.62753	2038.387568	1875.536953
KRTCAP3	3.90687474	12.1888077	349.0211471	232.31675	285.1525861	354.2698026
ANKRD61	0.97671868	1.01573397	6.017605984	2.01140043	12.09127574	0.906060876
PAFAH2	106.462337	161.501702	712.0833748	597.385928	353.6698153	472.9637774
EXTL1	0	0	1.002934331	8.04560172	1.007606311	26.27576541
SLC30A2	0	0	0	0	0	0
BRE	571.976228	596.753866	847.4795094	685.887547	810.1154743	674.1092919
TRIM63	2.93015605	13.2045416	2.005868661	1.00570022	0	3.624243505
WDR66	615.332771	641.02971	78.2288778	63.3591136	73.55526072	93.32427025
MRPL17	1665.30536	1992.87005	1044.054638	964.466507	1258.500283	1180.597322
DUSP2	7.81374947	7.11013781	119.3491854	159.906334	65.49441023	480.2122644
GRHL3	0	0	302.8861679	547.100917	135.0192457	560.8516824
UBXN11	302.782792	287.452714	471.3791354	458.599298	230.7418453	550.8850127
NLRP14	3.90687474	4.06293589	0	0	2.015212623	0
PTPDC1	750.119949	716.09245	450.3175145	282.601761	503.8031556	214.7364277
GALNT14	46.8824968	36.566423	1735.076392	1276.23357	207.5669001	1243.115522
NCK1	502.033404	481.457903	417.2206816	374.12048	559.2215028	399.5728464
HPD	0	1.01573397	3.008802992	0	0	0
RHPN1	284.225137	376.837304	3437.055951	2943.68453	939.0890821	3174.83731
TPRG1L	518.637621	675.463092	923.7025186	1126.38424	590.4572984	1036.533642
LRRC43	1.95343737	1.01573397	0	0	1.007606311	0
AAED1	531.334964	588.10997	276.8098753	387.194583	399.0120993	167.6212621
XDH	25.3946858	25.3933493	653.9131836	74.421816	1.007606311	221.0788538
XKR8	420.965753	494.662444	164.4812302	133.758129	169.2778603	146.7818619
CNNM4	628.030114	663.700892	1086.448672	853.256177	2410.194297	1159.404558
EYA3	509.847153	493.646711	604.7694014	789.474669	648.8984645	835.3881278
DZIP1L	118.182961	97.5104613	7.020540315	25.1425054	15.11409467	10.87273051

TMSB15A	0	0	12.03521197	57.3249123	3.022818934	30.80606979
FANCC	789.188697	688.667633	314.9213798	295.675863	1010.62913	299.0000891
MRAS	156.274989	169.627573	75.2200748	67.3819144	56.42595343	125.9424618
WASF2	4630.59398	4337.16375	4071.883295	8227.61335	3342.230135	4147.03157
ABHD3	392.640911	537.323271	373.091571	379.148981	313.3655628	352.4576808
ESYT3	28.3248418	30.4720192	62.1819285	32.1824069	52.39552819	62.51820046
FAIM	319.38701	274.248173	294.8626932	401.274386	375.8371541	318.0273675
FAM46B	277.388106	301.67299	1058.095719	2781.7668	105.7986627	1147.97913
CLSTN2	0.97671868	1.01573397	2121.206109	3386.19263	178.3463171	2002.394536
COLEC12	35.1618726	6.09440383	105.3081047	119.678326	35.2662209	99.66669638
RNF207	541.102151	409.340791	161.4724272	169.963336	242.833121	245.5424974
CUL4B	4499.74298	3914.63873	2770.104621	4349.65343	1963.824701	2410.121931
GPR153	225.622016	237.68175	510.4935743	326.85257	322.4340196	878.8790499
SLC13A3	7.81374947	6.09440383	4.011737323	4.02280086	45.34228401	10.87273051
GPRASP2	407.291691	406.242802	199.2329048	223.335847	329.1749058	201.1455145
RHBDL2	56.6496837	55.8653685	1.002934331	0	6.045637868	3.624243505
AUTS2	0	3.04720192	2016.900939	1193.76616	15.11409467	2762.579611
SHROOM4	3.90687474	9.14160575	0	0	1.007606311	1.812121752
HIST1H2BD	251.99342	157.438766	907.6555693	1098.22464	384.9056109	1284.794322
CDC25C	667.098861	623.660659	160.4694929	374.12048	261.9776409	279.9728107
HIST1H4H	46.8824968	46.7237627	822.4061512	504.861508	160.2094035	903.3426935
MITD1	604.588865	619.597723	432.2646965	388.200283	290.1906177	384.1698115
EIF5B	7093.9078	6982.15533	4018.757863	2911.50212	5071.282565	4388.052823
RIBC1	14.6507803	15.2360096	74.21714047	120.684026	19.14451991	38.0545568
TMSB15B	147.806838	121.908391	4.011737323	20.1140043	50.38031556	18.46552066
CATIP	0	0	4.011737323	2.01140043	3.022818934	0
CNOT11	1565.68005	1626.19009	1429.181421	1274.22217	2457.551793	1558.424707
KCNB1	0	0	91.26702409	14.079803	4.030425245	160.3727751
TSPAN33	83.0210881	102.589131	245.718911	191.083041	262.9852472	202.9576363
NRG2	26.3714045	66.0227082	13.0381463	9.05130194	11.08366942	10.87273051
AHCYL2	437.56997	555.606483	460.3468578	599.397328	877.6250971	536.3880387
B4GALT5	3998.68629	5086.79573	871.5499334	946.363903	1429.793356	1352.748888
CD1D	1.95343737	0	0	0	0	0.906060876

CD1A	0	0	0	0	0	0
SPATA2	547.939182	551.543547	505.4789027	363.057778	1303.842567	650.5517091
CD1C	0	0	0	0	0	0
SNX29P1	3.83850443	4.4997015	0	7.52263761	1.36026852	2.908455413
FAM86C1	433.809603	366.456503	235.7196557	262.236331	545.0747102	582.0444462
CD1B	0	0	0	0	1.007606311	0
DNAH3	1.95343737	1.01573397	1.002934331	9.05130194	94.71499326	136.8151923
CD1E	0	0	0	0	0	0
HMHB1	0	0	0	0	0	0
CPA2	1.95343737	0	0	0	1.007606311	0
NCF1	0.97671868	0	0	2.01140043	3.022818934	1.812121752
CPA5	1.95343737	0	0	0	0	0
TSR2	1143.73758	1064.4892	1351.955478	1077.10493	1188.975447	1019.318486
PPP1R9A	245.15639	237.68175	10.02934331	5.02850108	373.8219415	80.63941798
ZC3H18	1748.32644	1782.61312	1395.081654	1345.62689	4282.326823	1802.155083
ZFAND2B	298.875917	316.908999	566.6578969	559.16932	504.810762	381.4516289
POM121L2	0	0	0	0	2.015212623	0
GDPD5	667.098861	912.129107	431.2617622	619.511333	252.9091841	440.3455858
DYNC1I1	1.95343737	8.12587178	0	0	8.06085049	1.812121752
PFKFB1	1.95343737	5.07866986	56.16432252	89.5073192	2.015212623	78.82729623
ALAS2	0	0	0	0	0	0
TMED4	1859.67237	1843.55716	2062.032984	1705.66757	1866.086889	2105.685476
PPP1R15B	1847.95175	1832.38409	7608.259833	5618.8471	5002.765336	6896.029329
COPG2	1016.76415	1048.23746	792.3181213	972.512108	1473.120427	1014.788181
C11orf30	1495.3563	1405.77582	1985.809975	1760.98108	1609.147279	2236.158242
PAGE5	0	0	0	0	0	0
GPAT4	2114.59595	2208.20566	2040.971363	1918.87601	1370.344583	2259.715825
PKD1L1	0	3.04720192	8.023474646	6.03420129	2.015212623	8.154547886
ZSCAN12	303.759511	334.176477	255.7482543	227.288249	61.46398499	212.9243059
TAGLN2	6638.61039	7281.50228	1888.525345	3355.01592	10693.50411	5070.316663
ELK4	2006.18018	1868.95051	2511.347564	2345.2929	3762.401966	3941.364811
SLAMF8	0	2.03146794	0	0	0	0.906060876
SLC45A3	327.200759	419.498131	172.5047049	249.413653	157.1865846	313.4970632

DUSP23	325.247322	328.082073	795.3269243	807.577273	363.7458784	481.1183253
RNF166	551.846056	530.213134	282.8274813	237.345251	964.2792399	332.5243416
OR10J6P	0	0	0	0	0	0
NBL1	1074.39055	1071.8228	3754.986134	1837.41429	491.7118799	3247.32218
HTR6	0	0	2.005868661	1.00570022	4.030425245	2.718182629
ITLN2	0	0	0	0	0	0
F11R	285.201856	308.46825	5178.691534	6214.92562	3685.632441	5831.154102
USF1	571.38043	609.440383	1421.157947	1439.15701	690.2103232	1771.349013
PLA2G2F	0	0	1.002934331	0	0	2.718182629
SPATA2L	132.833741	164.548904	439.2852369	458.599298	1078.138753	541.824404
NIT1	399.477942	448.954416	1447.234239	1073.08213	348.6317837	1213.215513
DEDD	424.872627	399.183451	1442.219568	1439.15701	880.6479161	1456.945889
ZNF276	587.007929	503.80405	477.3967414	558.16362	1194.013479	915.1214849
NPM2	0	0	39.1144389	43.2451093	0	28.08788716
EDA	1.95343737	6.09440383	22.06455528	55.3135119	146.1029151	48.02122644
FGF17	0	0	10.02934331	8.04560172	2.015212623	2.718182629
VWA5B1	0	0	0	0	0	0
CDA	1053.87946	1115.2759	0	0	19.14451991	5.436365257
PINK1	2912.57512	3043.13898	1744.102801	2592.69516	778.8796786	1500.436811
B4GALT3	1053.87946	1142.70072	1785.223109	2176.33527	914.9065306	2256.997643
DMTN	356.50232	261.043631	6.017605984	3.01710065	748.6514893	104.1970008
ADAMTS4	0.97671868	1.01573397	250.7335827	221.254047	2.015212623	431.2849771
FAM160B2	652.448081	579.984098	1018.98128	755.280862	1455.99112	1013.88212
NDUFS2	1467.03146	1356.00485	3983.655162	3372.11282	1936.61933	3984.855733
SLC5A11	1.95343737	6.09440383	3.008802992	16.0912034	3.022818934	15.4030349
FCER1G	0	1.01573397	4.011737323	7.03990151	0	1.812121752
APOA2	0	0	1.002934331	1.00570022	0	0
TOMM40L	447.337157	546.464877	1365.996558	1200.80606	367.7763036	1424.327697
MPZ	1.95343737	5.07866986	2.005868661	0	1.007606311	0.906060876
WFDC8	0	0	0	0	0	0
CCAR2	2271.84766	2346.34548	2071.059393	1936.97862	4433.46777	2929.294813
WNT9B	0	0	0	0	0	0
CACHD1	267.620919	361.601294	4.011737323	12.0684026	171.2930729	55.26971345

CDC42SE2	1233.5957	1204.66049	1183.46251	1089.17333	1221.218849	1143.448826
RAPGEF6	1858.69566	1594.70234	845.4736408	878.981988	1069.070296	1192.376113
EPB41	869.279629	956.821402	2049.997772	1857.5283	1411.656442	1919.942997
MIS18A	725.701982	643.975338	353.0328844	385.183183	858.4805772	617.0274567
ALG8	1515.8674	1687.13413	1328.887988	978.54631	1415.686867	1630.909577
FBXW5	2661.55841	2665.28594	3972.622884	5120.0198	3770.462817	4433.355867
C21orf59	899.763019	1021.00563	1491.774553	1016.05893	1400.572773	1388.184929
SYNJ1	679.796204	769.926351	468.3703324	750.252361	715.400481	929.618459
PAXBP1	1402.56803	1250.36852	2165.33522	1491.45342	1383.443465	1750.509613
IFNAR2	570.403711	702.512087	80.23474646	137.78093	537.0541639	133.1909488
MRPL10	881.000253	768.910617	1389.064048	1318.47298	1695.801422	1445.167098
IFNGR2	1595.95833	1954.27216	497.455428	334.898172	548.1378333	318.9334284
GART	5165.86512	4690.65948	1972.771829	1951.05842	2906.944208	3379.607068
SON	13296.0714	12153.257	8644.290996	10630.2513	12757.30351	14599.3589
DONSON	1202.3407	1204.66049	411.2030756	538.049615	836.5651399	841.730554
SV2A	0	1.01573397	4.011737323	1.00570022	8.06085049	6.342426133
LAD1	0	2.03146794	1614.724272	1585.98924	4931.225287	1367.245862
STC1	1637.95723	2770.92228	1013.966608	718.069954	1319.964268	1593.761081
TNNI1	0	0	1.002934331	0	0	0
CSRP1	4684.34281	3926.82754	13596.78072	20578.6378	5017.87943	12767.30381
PRAC1	0	0	0	0	0	0
HOXB13	0	0	0	0	3.022818934	0
AC007383.6	0	0	0	0	0	0
C1QC	0	0	0	0	0	0
KCNE2	0	0	2.005868661	0	0	0
ATP5G1	1019.69431	1203.64476	1007.949002	758.297963	1734.090462	809.1123624
RCAN1	1382.05694	1195.51889	480.4055444	542.072416	337.5481143	786.4608405
UBE2Z	4656.01797	4537.28365	4307.60295	4793.16723	6673.3766	6035.271496
CIART	22.4645297	36.566423	79.23181213	153.872133	152.148553	64.33032221
SNF8	1719.02488	1859.8089	2024.924414	2095.87925	1975.915976	2231.627938
CLIC6	939.603374	1082.77241	0	0	16.12170098	7.248487009
CCDC24	139.670772	160.485968	412.2060099	541.066716	121.9203637	555.4153171
RUNX1	4453.8372	3872.99364	2185.393907	1689.57636	2637.913323	2711.840202

IGF2BP1	2.93015605	2.03146794	0	7.03990151	44.3346777	11.77879139
GIP	0	0	0	0	0	0
CBR1	1941.71674	2365.64442	3.008802992	0	1845.934762	158.5606533
CBR3	372.129819	360.58556	573.6784372	670.802044	211.5973254	342.4910112
C2orf81	45.9057781	80.2531411	96.28169575	103.597179	225.7340419	111.4545484
TUBBP5	0	0	194.2282625	206.088088	88.73988784	149.7084386
GJD2	0	0	0	0	0	0
ACTC1	2.93015605	0	0	0	0	0
MORC3	1219.62862	1070.58361	645.6590341	861.191152	784.7338713	1051.447404
CHAF1B	1570.56364	1243.25838	402.1766666	547.100917	1068.06269	963.1427114
CLDN14	0	0	0	0	0	0
SIM2	312.549979	240.728951	0	0	125.9507889	25.36970453
HLCS	1035.3218	1184.34581	543.5904072	394.234484	934.0510506	480.2122644
GOLGA6A	0	0	0	1.00570022	1.571865846	1.7577581
SCUBE1	0	0	0	0	3.022818934	8.154547886
ARHGAP27	933.743062	973.073145	462.3527265	524.975513	801.0470175	729.3790053
ADPGK	1867.48612	1714.55895	547.6021446	456.587898	2061.562513	704.0093008
PTMS	1981.76221	2078.19171	4139.109983	6620.52452	2106.904797	2746.270516
PLA2G4D	7.81374947	20.3146794	1.002934331	1.00570022	7.053244179	5.436365257
PADI4	15.6274989	26.4090833	0	0	9.068456802	0
ADIPOR1	2078.45736	2214.30006	6249.283815	6248.41544	4930.217681	5804.225973
CYB5R1	714.958077	754.690341	1522.454314	1934.96721	1253.462251	1882.794501
PSMD4	2385.87957	2382.90174	2569.507726	3024.06009	6115.132475	3193.846467
ATP13A2	3056.15276	3066.50086	2259.611047	2898.42802	1669.603658	2956.476639
M1AP	0.97671868	0	0	0	0	0.906060876
PSMB4	3843.38802	3918.70166	4846.178686	5230.64682	10010.5687	5723.586555
IRX6	0	0	0	0	8.06085049	0
BTG2	56.6496837	44.6922948	1827.346351	5071.74619	984.4313661	2209.882477
CES5A	0	0	0	0	0	0
HK2	4034.82488	3855.716	8070.60253	5764.67364	2146.201443	5429.11677
C1R	324.270603	236.666016	16.04694929	34.1938073	82.62371753	104.1970008
CELF3	0	0	5.014671654	0	5.038031556	6.342426133
ALDH4A1	422.391762	346.771578	12379.91047	14941.7987	946.1423263	13348.59622

STARD9	1110.52914	870.484014	4.011737323	55.3135119	65.49441023	48.92728731
THEM4	293.015605	253.933493	122.3579883	48.2736103	305.3047123	96.04245287
TCHH	6.83703079	9.14160575	0	0	275.076523	25.36970453
LCE2B	0	0	0	0	0	0
UBR1	1225.78195	1353.97338	3193.342909	2160.24406	946.1423263	2970.973613
AMFR	3029.78136	2731.30865	3482.187996	3320.82211	2532.11466	3902.404194
MED8	609.472459	668.352954	785.2975809	727.121256	775.8568597	708.5396052
TGM7	0	0	0	0	0	0
RGL4	0	0	2.005868661	3.01710065	1.007606311	0
SPRR2G	0	0	0	0	0	0
PGLYRP3	0	0	0	0	0	0
ISL2	0	0	37.10857024	31.1767067	11.08366942	28.08788716
RSPRY1	841.931505	790.24103	1226.588686	1257.12527	1064.032265	1941.688458
CCDC17	67.3935892	78.2115159	26.0762926	34.1938073	27.2053704	41.6788003
GPBP1L1	2019.85424	2087.33331	3626.61054	3077.44266	2039.395174	3366.922216
NAE1	1573.4938	1493.12894	1585.639177	1409.9917	1372.359796	1979.743014
TMEM69	772.584479	786.178095	499.4612967	512.90711	550.153046	672.2971701
ADGRG5	0	1.01573397	0	0	35.2662209	2.718182629
DRC7	0	1.01573397	0	2.01140043	0	2.718182629
ACE	0	4.06293589	177.5193765	175.997538	55.41834712	260.0394715
TEPP	30.2782792	19.2989455	0	0	0	0
UROC1	1.95343737	0	0	0	0	0
EFCAB14	2112.64251	2285.40144	2798.186783	3353.00452	3133.655628	3252.758545
SPON2	28.3248418	12.1888077	1.002934331	5.02850108	11.08366942	5.436365257
CHCHD6	235.389203	231.587346	268.7864006	242.373752	230.7418453	236.4818887
CTBP1	2989.73589	2759.7492	2861.371646	2815.9606	3256.583598	3356.049485
LRRC36	1.95343737	5.07866986	1.002934331	0	0	0
ANKRD18CP	0.97671868	0	29.08509559	24.1368052	1.007606311	10.87273051
TPPP3	4.88359342	4.06293589	2.005868661	1.00570022	8.06085049	8.154547886
ZDHHC1	163.11202	171.659041	108.3169077	104.592822	30.22818934	113.2576095
ATP6V0D1	1424.05584	1786.67606	3431.038345	4295.34562	3132.648022	4271.17097
AGRP	0	0	0	0	0	0
ZFYVE28	477.615436	448.954416	197.5780631	184.043139	117.8899384	263.663715

RLTPR	5.8603121	5.07866986	11.03227764	11.0627024	81.61611121	14.49697402
C16orf86	0.97671868	5.07866986	55.16138819	75.4275162	2.015212623	41.6788003
PIP	0	0	2821.254272	40.2280086	0	2316.79766
FAM131B	7.81374947	1.01573397	0	0	9.068456802	7.248487009
RGS12	766.724167	656.164146	358.0475561	456.587898	575.3432037	433.0970988
PSKH1	673.935892	682.573229	1368.002427	1135.43554	1321.97948	1504.061054
ZYX	5930.63585	5467.69597	1128.301122	1478.37932	4043.524127	2381.127983
ABR	3743.76272	3962.37823	783.2917123	851.828083	3120.556746	1192.376113
TCAF2P1	15.1391396	1.97052391	28.28274813	32.584687	9.511803579	18.22994483
LYPD5	0	4.06293589	0	1.00570022	0	8.154547886
CCDC117	1159.36508	1144.73219	1409.122735	1807.24329	2230.840373	2050.415763
ZNF230	184.599831	157.438766	204.5986035	206.168544	86.65414277	211.1121841
CCDC107	590.914804	544.433409	475.3908728	520.952712	161.2170098	313.4970632
ZNF222	121.376831	106.723168	135.9978952	117.345101	94.44293956	87.99663229
NPR2	67.3935892	57.8968364	118.346251	58.3306125	81.61611121	67.95456571
ZNF890P	0	0	1.173433167	5.82300425	5.108563998	2.292334017
ZNF221	156.274989	115.793673	116.3403824	93.53012	17.12930729	131.378827
ZNF233	4.88359342	2.03146794	158.4636243	109.621323	31.23579565	124.13034
ZNF235	87.9046815	77.1957819	167.4900332	166.946236	118.8975447	144.9697402
GNE	1771.76769	1764.32991	851.4912468	4244.05491	321.4264133	664.1426222
TNFRSF13C	16.6042176	34.5349551	96.28169575	31.1767067	17.12930729	49.83334819
OR3A3	0	0	0	0	0	0
ARHGAP35	6787.21813	5570.2851	10060.43427	9662.76767	7021.000777	10196.8091
PTGIR	0	0	0	0	0	0
CALM3	10194.0129	9967.39747	3980.646359	5883.34626	5636.549705	5996.310879
DFFA	1952.46065	2132.02561	1690.947282	1311.43308	1727.037218	1957.091493
CCDC28B	75.2073387	90.4003235	148.4342809	453.570797	20.15212623	319.8394893
IQCC	137.717334	68.0541761	175.5135079	139.79233	116.8823321	257.3212888
TMEM234	86.9279629	84.3059197	295.8656276	218.236947	100.7606311	233.7637061
BSDC1	1180.85289	1194.50315	2507.335827	2276.90529	1010.62913	2044.979398
ZBTB8A	416.082159	328.082073	260.7228086	272.544758	292.9816871	239.2000713
ATAD3B	905.535426	1161.80667	783.8132381	806.068723	698.7044444	1370.109015
SSU72	2475.98186	2412.36818	3407.970856	2834.06321	2163.33075	3164.87064

UBE2J2	1454.33412	1404.76008	1496.378021	1426.08291	1308.880598	1575.639864
ZNF362	306.689667	298.625788	723.1156524	721.087055	508.8411872	666.8608049
FNDC5	0	1.01573397	0	0	14.10648836	2.718182629
CPAMD8	0.97671868	2.03146794	206.3938559	868.200882	81.61611121	379.4311131
NR2F6	1770.79097	1999.98019	3138.181521	2988.94104	3474.226561	3798.207193
ANKLE1	25.3946858	30.4720192	24.8727714	19.1083041	11.78899384	22.6515219
CCDC58	271.527794	292.531384	253.7423857	168.957636	363.7458784	241.0121931
VMA21	2137.06048	2063.97143	2626.685012	2015.42323	2451.506155	2296.864321
KALRN	31.2549979	27.4248173	3.008802992	18.1026039	310.3427439	54.36365257
CILP2	73.2539013	75.1643139	25.07335827	17.0969037	299.2590745	70.67274834
FAM86C2P	66.4168705	82.2744518	139.1571384	132.752428	158.1639627	179.1644777
ABCG1	12.6973429	0	5467.997971	459.604999	190.4375928	1484.127715
TFF3	0	2.03146794	16.04694929	32.1824069	182.3767423	68.86062659
TFF2	3.90687474	12.1888077	0	0	5.038031556	0.906060876
TFF1	0	0	0	4.02280086	22916.99794	2484.418922
TMPRSS3	0	1.01573397	0	0	37.28143352	6.342426133
UBASH3A	0	0	0	0	0	0
RSPH1	2.93015605	6.09440383	23.06748961	32.1824069	46.34989032	48.92728731
SLC37A1	372.129819	463.174691	662.9395926	815.622875	1701.84706	881.2710506
PDE9A	108.415774	78.2115159	0	0	246.8635463	41.6788003
WDR4	243.202952	224.477208	183.5369825	243.379452	987.4541851	465.7152904
NDUFV3	1189.64336	1186.37728	839.4560348	684.881847	1652.474351	874.3487455
PKNOX1	473.708562	495.678178	391.144389	506.872909	594.4877237	545.4486475
CBS	0	0	10160.29644	3615.24085	3008.873663	5848.822289
U2AF1	2682.00114	2146.64202	2097.36636	1808.57081	4313.48201	2403.607353
CRYAA	0	0	0	0	0	0
HSF2BP	39.3519958	34.0067734	58.16016184	22.7690529	28.66639956	67.04850484
RRP1B	2983.87558	2851.16526	909.661438	936.306901	3424.853852	1755.945978
PDXK	4032.87145	4753.63499	3786.077098	4016.76666	7042.16051	3924.149655
G6PD	2548.25905	2995.39948	48611.22408	44470.0521	34130.64858	44262.88592
CSTB	2721.13825	3502.25074	1985.809975	3506.87665	2549.243968	1983.367258
RRP1	1292.19882	1225.9909	621.819285	585.317525	2132.094955	1038.345764
AGPAT3	4355.18861	3930.89047	2576.538296	2181.36377	4190.634649	3312.558563

TRAPPC10	1840.92914	1842.05387	1096.71872	1521.17186	2665.632573	1924.283028
GAB3	21.487811	20.3146794	0	2.01140043	4.030425245	0.906060876
C21orf33	97.7793074	42.0412291	1323.191321	917.208654	105.214251	980.7474742
ICOSLG	719.450983	524.971946	662.1071571	518.891026	369.1869525	674.7163527
AIRE	0	0	0	0	0	0
C21orf2	276.743472	318.940467	452.2230897	494.251371	366.7686973	290.8455413
ZNF66	51.7660902	42.6608268	156.527961	154.877833	167.2626477	257.3212888
LRRC3	278.364825	291.51565	39.1144389	86.4902185	233.7646642	64.33032221
ITGB2	302.782792	338.239413	19.05575228	9.05130194	70.53244179	51.64546994
FAM207A	855.576266	831.886123	460.3468578	415.354189	1318.956661	580.7669004
RALGDS	1046.06571	977.136081	1440.213699	1138.45264	1238.348157	1543.021672
FTCD	3.90687474	5.07866986	2.005868661	3.01710065	19.14451991	2.718182629
SPATC1L	354.548882	461.143223	236.692502	153.872133	425.2098634	211.1121841
LSS	3630.46335	7118.26368	2089.112211	33671.8489	3940.748283	2110.215781
VAV2	1762.00051	1568.29325	996.9167247	912.170096	3863.162597	1627.285334
MCM3AP	3148.94104	3326.50844	4185.244962	4983.10377	4483.827933	5278.710665
C21orf58	367.246225	323.003403	375.0974397	379.148981	822.20675	407.7273943
PCNT	4669.69203	4656.12453	1652.835777	2731.48179	4013.295938	2920.234204
DIP2A	2044.27221	1713.54321	2545.447331	2957.76433	2539.167904	2343.979487
S100B	0	0	0	0	0	0
PRMT2	1802.04597	1618.06422	2135.24719	1923.90451	1992.037677	1633.62776
CLDND2	1.95343737	1.01573397	19.05575228	59.0547167	3.022818934	27.18182629
ZNF208	0	0	0	0	0	0
ADAMTS13	8.79046815	9.14160575	144.4225436	148.843632	66.50201655	101.4788181
CACFD1	197.297174	257.996429	1354.964281	1364.73519	424.2022571	900.6245109
SLC2A6	676.866048	1102.07136	0	0	447.3772022	85.16972236
ZNF761	556.387798	529.1974	662.307744	637.523424	836.2729341	788.3001441
FCN2	0	0	0	0	0	0
C9orf116	48.8359342	54.8496345	84.24648378	140.79803	279.1069482	131.378827
LCN1	0	0	0	0	0	0
ZNF714	779.42151	806.492774	1061.104522	1283.27348	901.8076486	2047.69758
GPSM1	740.352762	704.919377	76.22300913	65.370514	831.2752068	277.2546281
C19orf47	693.470266	672.41589	873.555802	1425.07721	506.8259746	1369.057984

HIPK4	0.97671868	6.09440383	12.03521197	5.02850108	10.07606311	3.624243505
CFAP157	0.97671868	3.04720192	4.011737323	12.0684026	2.015212623	4.530304381
TOR2A	428.779502	404.262121	347.0152784	396.245885	618.6702751	307.154637
ST6GALNAC6	983.819429	918.223511	427.2500249	432.219782	375.8371541	610.9477882
SHKBP1	1997.38971	1929.89455	952.7876142	1325.51288	1449.945482	1116.266999
RDH13	564.543399	558.653685	923.7025186	1080.12203	791.9785607	1236.773096
ZER1	938.626655	1044.17452	1362.987755	1389.8777	1761.295832	1676.212621
ZDHHC12	561.613243	566.779557	484.4172817	843.782481	2271.144626	680.451718
PKN3	1221.87507	848.137867	172.5047049	234.32815	482.6434231	389.6061768
SPTBN4	12.6973429	22.3461474	178.5223109	163.929135	28.21297672	141.3454967
BRSK1	151.391396	114.777939	180.5281795	132.752428	37.28143352	119.6000357
COX6B2	1.95343737	1.01573397	0	0	0	0
TMEM190	0	1.01573397	0	0	0	0
NLRP4	0	0	0	0	1.007606311	0
PLPP7	0	0	0	1.00570022	3.022818934	0
TAOK1	5837.84757	4935.45137	3770.030149	3826.68932	4802.25168	5785.198694
MED27	725.692215	722.16654	313.9184455	342.943774	714.3223423	430.2430071
DEDD2	861.465879	749.611672	735.1508644	686.893247	442.3391707	648.7395873
SIK3	1883.11362	1623.14289	490.4348877	515.924211	493.7270925	979.4518071
MPZL3	54.6962463	78.2115159	313.9184455	357.023577	752.6819145	364.2364722
AMICA1	0	0	0	0	0	0
NEK8	132.833741	281.35831	153.4489526	1384.8492	127.9660015	173.9636882
TLCD1	244.179671	330.113541	69.20246882	367.080579	202.5288686	162.1848968
PCSK7	1610.60911	1614.00128	1648.82404	1774.05518	1399.756612	2275.11886
SAFB	4236.73217	4570.31532	3155.341727	2931.72676	3591.78399	3619.776625
CD3G	0	0	0	0	0	0
S100A1	0	0	53.15551953	24.1368052	10.07606311	79.7333571
CHTOP	2013.01721	1877.07638	3622.598803	3139.79607	2782.001025	3134.064571
CXCR5	6.83703079	13.2045416	11.03227764	15.0855032	26.19776409	11.77879139
ZBTB7B	404.361535	414.419461	8036.512792	5662.09221	3015.76569	6072.419992
FLAD1	375.059975	445.907214	1086.17788	851.828083	1173.861353	1489.56408
SHC1	4444.07001	4634.07294	3001.782452	2663.09417	2547.228755	2342.167365
VPS11	1242.38617	1264.5888	1108.242435	1302.38178	913.8989243	1135.294278

NLRX1	419.012315	366.679964	54.15845386	39.2223084	194.4680181	71.57880922
ADAR	6186.53614	5811.01406	9345.342094	9564.20905	11438.34685	18935.76625
IL6R	39.0687474	47.7394967	163.4782959	179.014638	111.8443006	658.706257
UBE2Q1	1431.86959	1585.56073	2812.227863	3009.05504	2748.750017	2656.570489
CHRNB2	0	3.04720192	6.017605984	11.0627024	3.022818934	19.0272784
CRTC2	1105.64555	1111.21297	1056.08985	1348.64399	1128.519069	1384.461019
ANO10	1435.77647	1425.07476	862.5235244	1351.66109	536.0465576	837.2002496
FDPS	2678.16263	4332.10539	2062.032984	38752.6464	3263.636842	2498.915896
RUSC1	1040.2054	986.277687	1810.296467	2988.94104	1908.406354	2933.825117
GBAP1	343.775675	256.767391	270.9727975	510.915824	321.7286952	365.9761091
FAM189B	1440.66006	1510.39642	799.3386616	1570.90374	1232.302519	1689.803534
PAQR6	95.718431	111.730737	966.8286948	742.206759	115.8747258	482.0243861
PMF1	788.895681	606.088461	809.9998535	1170.53448	1280.728078	936.0424306
SLC25A44	944.486967	1157.93673	1361.984821	1839.42569	1740.1361	2070.349102
LMNA	11392.4467	12678.3914	15449.20043	13747.9219	8170.679578	10230.33335
CCR5	0	0	4.011737323	0	0	0
NBEAL2	1477.77537	1297.09228	6682.551446	8814.96239	2879.738838	8154.547886
CCDC12	614.356052	567.79529	659.9307896	609.454331	424.2022571	604.3426044
PTH1R	24.4179671	25.3933493	3.008802992	8.04560172	5.038031556	1.812121752
UBQLN4	1608.43103	1514.34762	2071.982092	2885.91711	3380.14636	4257.824694
MYL3	0	1.01573397	0	0	0	0
PPP1R35	676.866048	494.662444	327.9595261	221.254047	717.4156936	338.8667677
GPATCH4	948.393842	1168.09407	1144.348071	734.161157	1735.098068	1942.594519
LRRC71	0	6.09440383	0	0	1.007606311	0
FCRL3	0	0	0	0	0	0.906060876
AZGP1	5.8603121	4.06293589	28429.28686	8474.4323	3.022818934	15896.12228
FGFR4	186.553269	302.688724	33244.26426	67499.5814	417.1490129	22160.43691
CYP3A4	0	0	0	0	0	0
CYP3A7	0	0	0	0	0	0
NACC1	5157.07465	4961.86045	4706.770814	4747.91072	7261.818685	7545.674977
CYP11B1	0	0	0	0	1.007606311	0
HK3	0.97671868	0	2.005868661	0	0	3.624243505
LY6K	28.3248418	24.3776153	0	0	0	0

IER2	1939.76331	2559.64961	3240.480823	5418.71276	2227.817554	2965.537248
ZNF394	320.363728	295.578586	207.6074065	187.06024	557.2062901	243.7303757
CPSF4	1164.24867	1024.87558	358.0475561	367.080579	1204.089542	564.4759259
LY6E	3665.62522	3595.69826	5628.467464	6262.49524	3741.242234	7514.868907
VPS28	1109.55242	1096.99269	3025.852876	2364.40121	934.0510506	1811.215691
TONSL	2699.65044	2366.66016	2104.156226	2105.93625	2020.250654	5387.43797
PTGER1	1.95343737	1.01573397	0	3.01710065	1.007606311	4.530304381
MUM1	1216.99148	1190.44022	1786.226043	1447.20261	816.1611121	2351.227974
RECQL4	2202.50063	1820.19528	1231.603358	1856.5226	2416.239934	3484.71013
LRRC14	1026.53134	979.167549	1479.328138	1331.54709	649.9060708	1655.373221
ZNF333	246.133108	201.115327	224.6572901	162.923435	92.69978064	153.1242881
COL26A1	0.97671868	0	0	0	2.015212623	2.718182629
PPP1R16A	1360.56913	1419.99609	2014.89507	1564.86954	1863.06407	2401.061322
FOXH1	1.95343737	8.12587178	31.09096425	45.2565097	3.022818934	28.99394804
ORAI2	1106.62227	1209.73916	296.8685619	239.356651	1325.002299	424.0364901
ALKBH4	212.924673	235.650282	251.736517	226.282548	565.2671406	275.4425064
CCDC105	0	0	0	0	0	0
SH2B2	144.554365	121.888077	73.21420614	64.3648138	310.3427439	98.7606355
C5orf45	452.220751	416.999425	1028.328628	891.342044	676.446421	997.5730247
SQSTM1	10760.5097	22291.2978	17411.94292	15556.1709	10439.80899	12558.00374
MGAT4B	5425.67229	6366.62054	3554.399268	3774.39291	3855.101747	4315.567953
RPL8	23316.2284	23860.6067	32068.82522	28403.9912	34883.3305	30085.75139
MAML1	2777.98328	2853.39987	1445.519222	1559.9416	3488.494267	2319.733298
PGLYRP2	0	0	4.011737323	11.0627024	14.10648836	24.46364366
LRWD1	693.470266	714.060982	402.1766666	396.245885	1124.488643	512.8304559
FBXL13	81.0676508	72.117112	0	0	13.09888205	16.30909577
NAPEPLD	172.879207	206.193996	162.4753616	171.974737	456.445659	173.9636882
SCGB3A1	0	0	3.008802992	0	0	0.906060876
PSMC2	2377.33328	2475.34369	1758.123823	1086.15623	4549.342495	1436.967247
CELF5	0.97671868	4.06293589	0	0	37.28143352	2.718182629
MFSD12	3023.92105	3317.38715	1149.362743	1244.05117	1032.796469	1373.588288
AC008132.13	0	2.03146794	2.045986035	0	0	0
XXbac-B444P24.10	0	0	1.002934331	0	0	0

USP41	1.02555462	0	14.06113932	28.3808601	0	27.66203855
TUBA3FP	0	1.01573397	0	0	1.007606311	0
YDJC	820.443694	790.24103	1227.591621	1038.88832	1253.462251	1407.112541
CCDC116	5.8603121	3.04720192	2.005868661	3.01710065	5.038031556	7.248487009
DVL3	2668.39544	2645.987	5768.87827	6517.9431	4789.152798	7217.68094
AP2M1	7696.54323	6943.55743	11289.02883	11016.4402	14092.38187	13412.41915
ABCF3	1673.11911	1658.69358	2719.957905	2006.37193	2307.418453	2845.031151
PCYT1A	2415.42531	2503.78424	5174.138212	4679.12082	4069.721891	5305.421339
FBXO27	722.771826	810.55571	1.002934331	2.01140043	360.7230594	53.45759169
DMKN	161.158583	136.108352	213.6250124	141.80373	274.0689167	490.178934
U2AF1L4	114.432361	132.086046	203.3850529	229.098509	121.819603	235.2587065
BDH1	1000.15993	941.585392	3973.625818	2890.38242	589.4496921	3898.77995
NPHS1	1.95343737	8.12587178	0	0	3.022818934	4.530304381
THAP8	101.578743	144.234224	201.5898005	292.658763	102.7758438	113.2576095
COX7A1	0	0	0	0	0	0
ZNF382	17.5809363	12.1888077	507.4847713	347.972275	2.015212623	402.291029
LRRC56	12.6973429	6.09440383	205.6015378	210.191345	56.42595343	214.7364277
PLXDC1	3.90687474	13.2045416	24.07042394	22.1254047	1.007606311	5.436365257
PGAP3	224.645297	211.272666	947.7729425	914.181496	137.0344583	781.9305361
IKZF3	2.93015605	8.12587178	0	0	8.06085049	3.624243505
GRIN2C	0	3.04720192	30.08802992	23.131105	65.49441023	46.20910469
FDXR	253.946858	257.996429	642.880906	823.668477	905.8380738	987.606355
SAP30BP	1326.38397	1306.23389	1087.180814	1119.34434	2679.225182	1266.673105
ACOX1	1065.60008	1201.61329	1789.234846	1878.648	1825.782636	2155.518824
PRPSAP1	1250.19992	1114.26017	1871.475461	2168.28966	1221.218849	2170.015798
CYGB	43.9523408	42.6608268	9.919020531	6.63762142	14.69090002	8.154547886
SRSF2	8562.8927	7781.53796	5613.423449	4936.98236	7742.446896	7360.838558
ZNF577	120.136398	96.4947274	371.0857024	232.31675	7.053244179	329.3621891
TMEM143	161.158583	136.108352	266.780532	243.379452	143.0800962	212.9243059
KLHL10	0	2.03146794	3.008802992	1.00570022	4.030425245	0
CCDC155	0	0	162.4753616	86.4902185	0	84.26366148
HCRT	1.95343737	1.01573397	0	0	0	0
ALDH16A1	1589.91244	1699.32294	1474.313466	1887.6993	1071.085509	1686.179291

DCD	0	0	0	0	0	0
ITGA5	3899.06099	4530.17352	16.04694929	12.0684026	1159.754864	382.3576897
SIGLEC11	0	0	0	0	0	0
ZNF385A	179.716238	239.713217	4252.441562	3502.85385	1856.010825	4190.531552
SIGLEC16	0	0	0	0	2.015212623	0
MPP3	502.033404	438.797076	290.8509559	245.390853	250.8939715	343.3970721
CD300LG	0	0	0	0	0	0
IZUMO2	0	0	0	0	0	0
NAGS	500.079966	402.230653	6.017605984	10.0570022	45.34228401	32.61819154
LSM12	2491.66797	2441.05251	856.5560651	772.961072	2096.304779	1188.389445
ASB16	3.90687474	1.01573397	5.014671654	6.03420129	6.045637868	5.436365257
EMC10	1299.03585	1451.48385	2490.285943	3646.66898	3481.279806	2808.788716
JOSD2	278.364825	336.207945	326.9565918	464.6335	512.8716124	203.8636971
SHANK1	0	6.09440383	0	1.00570022	4.030425245	0.906060876
FAM171A2	228.552172	183.847849	54.15845386	30.1710065	675.0962286	135.9091314
DBF4B	1160.3418	1089.88255	530.5522609	525.981213	546.1226207	792.8032667
PLCD3	2851.04184	2593.16883	201.5898005	351.995075	5679.876777	1024.754851
FMNL3	1410.38178	1770.42431	39.1144389	113.644124	456.445659	251.8849236
AQP5	3.90687474	4.06293589	0	0	0	0.906060876
RACGAP1	3472.23492	3264.56899	1244.641504	2308.08199	4035.463277	2383.846165
OR7G1	0	0	0	0	0	0
LARP4	3464.42117	2834.91352	2258.608113	2403.62352	3568.941555	3044.364544
GRASP	3.90687474	4.06293589	21.06162094	13.0741028	12.09127574	47.11516556
RAVER1	4444.07001	4361.56168	1922.50476	2193.1908	3513.523207	3289.000981
KRT84	0	0	0	0	0	0
KRT82	0	0	0	0	0	0
SYCE2	4.88359342	4.06293589	110.3227764	153.872133	15.11409467	170.3394447
SPC24	1141.78414	844.074931	321.9419202	586.323226	1131.541888	664.1426222
IP6K3	0	0	0	0	4.030425245	0
LEMD2	2522.86436	2535.27199	2106.162094	1783.10648	1608.139673	1919.942997
ALOX15	0	0	221.6484871	161.917735	517.909644	339.7728286
TREML1	0	0	0	0	0	0
ADCY10P1	20.5110924	21.3304134	35.10270157	35.1995075	7.053244179	21.74546103

ZNF653	275.434669	296.59432	149.4372153	261.482056	178.4370017	199.0615745
MED11	361.385913	380.90024	105.3081047	131.746728	293.2134366	146.7818619
CXCL16	45.9057781	52.8181666	366.0710307	458.599298	721.4461189	718.5062748
SCIMP	0	0	0	0	0	0
RNASEK-C17orf49	10.2848477	43.7984489	8.755616707	19.4602992	32.53560779	21.4102185
BCL6B	0.97671868	1.01573397	0	0	2.015212623	0
ASGR2	0	3.04720192	0	0	0	0
TNFSF13	139.289852	226.356316	449.3145802	509.890009	173.3082855	618.8395784
SENP3	2856.90215	2886.21824	743.1743391	682.78999	2608.602055	1151.268131
FGF11	18.7041628	41.3099007	25.07335827	9.57426605	152.9546381	28.75837221
EIF4A1	24986.2416	24848.3674	5670.640853	5772.95055	26363.75716	11423.2078
RPL26	12441.5012	12317.9379	7606.996136	4831.43412	12518.38997	5150.675202
CCDC42	0	0	0	0	0	0
POLR3K	368.222944	414.419461	626.8339567	808.582973	1198.043904	837.2002496
SNRNP25	408.26841	425.592534	912.6702409	1223.93716	1447.930269	999.3851464
PRR35	0	0	0	0	5.038031556	0
WDR90	688.586672	520.055794	1294.788221	1198.79466	388.9360362	2546.031062
JMJD8	1027.50806	965.963008	1390.066982	1442.17411	1607.132067	1863.767222
CCDC78	27.3481231	28.4405512	338.9918038	473.684802	188.4223802	574.4425955
MSLNL	0.97671868	0	1.002934331	0	0	0.906060876
SSTR5	0	0	0	0	4.030425245	0
SPSB3	490.312779	398.167717	1384.049376	1291.31908	528.9933134	905.1548153
MEIOB	0	0	0	0	2.015212623	0
HS3ST6	0	0	0	1.00570022	0	4.530304381
C16orf59	339.898102	353.475422	345.0094098	322.829769	481.6358168	783.7426579
CCNF	1400.61459	1407.80729	837.4501661	2027.49163	1971.885551	2654.758367
TBC1D24	545.009026	562.716621	946.7700082	742.206759	1050.177678	1547.551977
AMDHD2	427.802784	505.835518	1042.04877	2048.96333	1014.659555	1307.445844
NTN3	0	1.01573397	10.02934331	8.04560172	2.015212623	31.71213067
CCDC64B	0	0	2228.520083	1229.97136	681.1418664	1684.367169
PAQR4	1169.13226	1128.48044	1484.342809	2023.46883	1731.067643	2573.212888
FLYWCH2	362.362632	286.43698	1275.732469	836.742579	991.4846103	799.1456928
ZG16B	2.93015605	2.03146794	685.0041479	475.696202	382.8903983	405.0092117

ZNF75A	766.724167	660.227082	884.5880797	692.575453	895.7620107	1007.539694
ADCY9	1692.65348	1528.67963	741.1684704	1175.66355	3134.663234	967.6730158
SHANK2	203.157486	123.919545	5261.393499	5040.56948	1841.904337	4498.59225
CLPB	1082.2043	1133.55911	301.8832335	548.106617	1175.876565	662.3305005
NEU3	718.864951	765.863415	733.1449957	544.083817	757.7199461	1257.612496
CYB561A3	872.209785	1146.76365	567.6608312	1188.73765	1007.606311	979.4518071
PPP1R32	0.97671868	5.07866986	45.28248503	16.0912034	2.015212623	15.4030349
ASRGL1	77.160776	68.0541761	158.4636243	83.4731179	248.8787589	164.9030795
GNG3	0	1.01573397	3.008802992	1.00570022	3.022818934	4.530304381
UBXN1	1451.40396	1586.57646	2184.390972	1817.30029	926.9978064	1252.176131
LBHD1	57.558032	91.8731378	86.09188295	5230.19426	126.4646681	170.9193237
TTC9C	578.217461	634.833733	479.4026101	501.844408	414.1261939	447.5940728
TAF6L	561.613243	588.10997	449.3145802	703.990151	445.3619896	508.3001515
NXF1	1721.95504	1744.01523	2744.028329	2190.41507	1522.493136	2526.097723
STX5	1101.73868	1203.64476	1467.292926	1041.90542	613.6322436	889.7517804
SLC25A45	136.740616	183.847849	233.6836991	410.325688	66.50201655	300.8122109
RPL29	14513.1411	13584.9035	9981.483281	7722.05791	8302.686081	7060.243802
ITIH3	0	0	0	0	0	0
SYVN1	2060.87642	2308.92584	1941.008898	1862.77805	1152.70162	1944.488186
ZFPL1	978.672121	1024.87558	889.6027513	818.639975	763.765584	713.0699096
RPS6KA4	4361.04892	4585.02315	782.288778	1215.89156	2801.145545	1577.451985
LRP5	6754.0097	5928.8392	1634.782959	1529.67003	2811.221608	2438.209818
TPCN2	599.705272	409.340791	831.4325602	603.420129	510.8563998	713.9759704
FGF19	2.93015605	0	0	0	0	0
CYP4A22	0	0	0	0	0	0
PDZK1IP1	302.782792	204.162528	0	0	1.007606311	7.248487009
TAL1	9.76718684	11.1730737	0	0	0	0
CMPK1	3834.59755	3778.53038	3621.595868	3263.4972	2933.141972	3134.064571
BEND5	0	0	2.005868661	3.01710065	1.007606311	1.812121752
ELAVL4	0	0	0	0	0	0
COA7	1098.80852	1112.2287	843.4677721	1505.53322	1430.800962	1443.354976
ZYG11B	1600.84192	1337.72164	1149.362743	1371.77509	1548.6909	1379.024654
SLC1A7	34.1851539	27.4248173	5672.596574	6950.39419	0	2021.421815

C1orf123	627.053395	600.298778	575.6843058	355.012176	606.5789994	347.0213156
MAGOH	799.932602	793.288232	589.7253865	412.337088	635.7995824	506.4880298
ACOT11	14.6507803	12.1888077	9.026408976	20.1140043	104.7910564	30.80606979
FAM151A	0	0	0	0	0	0
PARS2	199.250612	171.659041	164.4812302	148.843632	222.6809948	188.4606622
LEXM	0	3.04720192	0	0	0	0
BSND	0	0	0	0	0	1.812121752
USP24	3944.96676	3283.86793	1861.446118	2349.3157	2760.841293	3135.876692
PLPP3	495.196373	370.7429	223.6543557	160.912034	193.4604118	254.6031062
NOL9	1049.97259	1055.3476	674.9748046	648.676639	1066.047477	1080.024564
PRKAA2	627.053395	506.851252	2581.552967	1170.63505	69.52483548	1139.824582
KLHL21	1315.64007	1736.90509	690.0188195	1148.50965	892.7391918	955.8942244
ZSWIM5	25.3946858	27.4248173	7.020540315	51.290711	598.5181489	98.7606355
GMEB1	485.429186	525.134464	547.6021446	572.243423	379.8675794	540.0122822
SLC45A1	24.4179671	20.3146794	22.06455528	21.1197045	1.007606311	25.36970453
SEPN1	3111.82573	3198.54628	1239.456334	1055.98523	2059.5473	1805.779326
AK4	4037.78434	3095.35786	3839.563586	3481.58329	1672.112598	3630.404719
JAK1	4312.21299	3927.84327	1472.307597	1767.01528	6173.603869	2516.131053
RAVER2	491.289498	494.662444	83.24354945	71.4047153	507.8335809	134.0970097
CTRC	0.97671868	0	0	3.01710065	1.007606311	0
LZIC	691.516828	668.352954	731.1391271	733.155457	532.0161324	719.4123357
RBP7	0	2.03146794	0	0	46.34989032	1.812121752
KNCN	0	0	0	0	0	0.906060876
FBLIM1	805.792914	647.02254	0	1.00570022	572.3203848	126.8485227
TMEM82	0	0	1.002934331	0	0	0
SLC25A34	9.76718684	10.1573397	8.023474646	18.1026039	9.068456802	19.93333928
AKR7A3	11.3690055	7.11013781	1820.837307	801.754269	21.17988466	1082.208171
DRAXIN	0	9.14160575	0	0	0	0
PDPN	0	0	0	0	0	0
LRRC38	0	0	0	0	0	0
DHRS3	219.761704	88.3688556	1299.802893	358.029277	251.9015778	298.0940283
MATN1	0	0	1.002934331	1.00570022	1.007606311	2.718182629
LAPTM5	1548.09911	1606.89114	0	1.00570022	48.36510294	39.86667855

SDC3	2527.74795	2321.96786	543.5904072	461.616399	992.4922166	770.1517448
PEF1	980.625559	939.553924	1264.700191	1263.15947	1133.5571	1362.715558
SYNC	83.9978068	95.4789934	4.011737323	3.01710065	13.09888205	3.624243505
RBBP4	3192.89338	2967.94419	5364.675676	4482.40586	2713.463644	5471.692571
KIAA1522	1468.9849	1378.351	8709.481728	14831.0611	6724.764522	11632.00953
TSSK3	2.93015605	4.06293589	10.02934331	13.0741028	5.038031556	11.77879139
TMCO4	357.479038	310.814596	415.2148129	463.627799	238.8026958	472.9637774
UBXN10	0.97671868	2.03146794	0	0	0	0
CAMK2N1	352.595445	449.97015	5761.85773	8091.86393	701.2939927	4073.649699
ALPL	0	0	2.005868661	0	1.007606311	1.812121752
WNT4	1.95343737	0	16.04694929	16.0912034	57.43355974	25.36970453
TTLL10	0	0	2.005868661	3.01710065	2.015212623	4.530304381
SCNN1D	260.783889	217.36707	199.5839318	113.644124	46.34989032	93.32427025
MXRA8	10.7439055	6.09440383	76.22300913	37.210908	5.038031556	77.01517448
FAAP20	777.468072	758.753277	1578.618637	1490.44772	528.9933134	1483.221654
MEGF6	138.694053	82.2744518	1434.196093	1462.28811	246.8635463	1185.127626
CCDC27	0	0	0	0	0	0
IL23R	0	0	0	0	0	0
DIRAS3	0.97671868	4.06293589	0	3.01710065	0	1.812121752
C1orf87	0	0	0	0	0	0
NFIA	469.801687	535.291803	5071.83891	4553.81058	50.38031556	5502.507701
OMA1	235.389203	309.798862	484.4172817	494.804506	270.0384914	284.5031151
MYSM1	684.679797	565.763823	3338.768387	1956.08692	1034.811682	3274.504007
TM2D1	394.594348	365.66423	308.9037739	269.527658	281.1221608	272.7243237
USP1	4733.17874	3913.623	1514.430839	2086.82795	3212.24892	2652.040185
FUBP1	1918.2755	1925.83161	2220.496608	2574.59255	4128.163057	3112.31911
NEXN	815.560101	634.833733	2.005868661	3.01710065	6.045637868	19.93333928
DNAJB4	277.388106	276.27964	52.1525852	73.4161157	100.7606311	104.1970008
ADGRL4	0	0	0	0	0	0
LRRIQ3	8.79046815	21.3304134	35.10270157	34.1938073	16.12170098	35.33637417
LRRC53	0	0	0	0	0	0
TYW3	707.144327	831.886123	535.5669326	503.855808	495.7423052	504.675908
LHX8	0	0	1.002934331	0	0	0

SNX7	1126.15664	1215.83356	626.8339567	1153.53815	708.3472368	669.5789875
B3GALT2	0	0	0	0	0	0
NTNG1	0	0	3.008802992	0	9.068456802	0.906060876
FAM102B	2158.54829	2011.15327	312.9155112	599.397328	2136.12538	792.8032667
HENMT1	0	0	0	0	994.5074292	24.46364366
AKNAD1	7.81374947	23.3618814	0	4.02280086	5.038031556	3.624243505
C1orf52	464.918094	541.386207	459.3439235	344.955174	539.0693765	388.7001159
WDR63	1.95343737	2.03146794	0	0	6.045637868	0.906060876
GBP2	365.292788	305.735926	44.12911055	165.940536	118.8975447	498.3334819
ATXN7L2	189.483425	194.005189	99.29049874	175.997538	233.7646642	257.3212888
GBP4	84.9745255	78.2115159	0	0	6.045637868	8.154547886
ZNF326	847.791818	802.429838	1324.876251	1132.41844	817.1687185	1299.291296
HFM1	12.6973429	2.03146794	0	0	1.007606311	1.812121752
BRINP3	1.95343737	0	1693.956085	2439.82872	450.4000211	2449.082548
GFI1	13.6740616	8.12587178	1.002934331	1.00570022	27.2053704	9.966669638
LSP1P3	0	0	0	0	0	0
KCNT2	2.93015605	6.09440383	0	0	0	0.906060876
AGL	867.326191	913.144841	725.1215211	1230.97706	767.7960092	1499.53075
VCAM1	0	0	0	0	0	0
EXTL2	714.958077	843.059197	213.6250124	199.128643	99.75302482	458.4668033
SLC30A7	1576.42396	1619.07995	990.8991187	881.999089	520.9324629	1311.070088
DNAJA1P5	0	0	0	0	0	0
ZNF281	1284.38507	1403.74435	2961.665079	2024.47453	1517.455105	2801.540229
ARPC5	4566.15985	4561.66127	3379.888694	4536.71367	3914.550519	3010.840292
CADM3	0	0	0	0	0	0
NLRP3	272.504513	249.870557	2.005868661	0	12.09127574	4.530304381
ZNF496	1899.71784	1723.70055	2309.757764	2425.74892	1219.203637	2340.355243
TRIM58	0	0	0	0	0	0
SLAMF9	0	0	1.002934331	9.05130194	0	0.906060876
OR2M5	0	0	0	0	0	0
KCNJ9	0	0	1.002934331	2.01140043	1.007606311	0
IGSF8	595.798397	670.384422	1701.979559	2857.19431	550.153046	1819.370239
DDR2	52.7428089	44.6922948	130.381463	185.04884	11.08366942	168.527323

PEA15	7114.41889	6555.54706	1573.603965	1780.08938	2808.19879	1866.485405
PEX19	1569.58692	1612.98555	2233.534754	1987.05243	1092.013492	2794.40953
NCSTN	2677.18591	2818.66177	2725.975511	2284.95089	1517.455105	2886.709952
VANGL2	0	1.01573397	0	0	15.11409467	3.624243505
SLAMF6	0	0	0	0	0	0
OLFML2B	68.3703079	121.888077	1.002934331	3.01710065	4.030425245	1.812121752
FCRLB	21.487811	33.5192211	0	7.03990151	40.30425245	1.812121752
FCGR3B	0	0	0	0	0	0
SLC9C2	0	1.01573397	0	0	0	0
KLHDC9	21.487811	30.4720192	174.5105735	108.615623	69.52483548	89.70002674
C1orf74	83.0210881	101.573397	356.0416874	259.470656	131.9964268	409.539516
LMX1A	0	0	0	0	0	0
LRRC52	0	0	0	0	0	0
FLVCR1	327.200759	396.136249	873.555802	4673.4889	309.3351376	1117.17306
FAM71A	0	0	0	0	0	0
ATF3	118.182961	62.9755063	360.0534247	103.587122	86.65414277	123.2242792
RBM15	768.677604	771.957819	655.9190523	767.349264	825.2295689	1169.724591
DENND2D	30.2782792	24.3776153	1123.28645	885.01619	689.2027169	842.6366148
AXDND1	1.95343737	1.01573397	3.008802992	2.01140043	3.022818934	0
TDRD5	0	0	113.3315794	162.923435	177.3387108	163.9970186
IER5	2729.92872	2097.49065	450.3175145	497.821607	1463.044364	842.6366148
SNED1	61.5332771	53.8339005	1364.993624	1255.11387	133.0040331	1141.636704
BPNT1	953.277435	992.372091	3187.325303	3081.46546	755.7047335	2434.585574
SPATA17	2.93015605	12.1888077	63.18486283	17.0969037	38.28903983	48.02122644
C1orf115	243.202952	179.784913	11770.43731	6162.93092	217.6429632	4192.343674
BROX	2511.14374	2762.7964	3199.360515	4046.93767	1888.254227	3674.076853
NBPF20	296.131338	315.100993	119.3291267	148.793347	369.8418966	237.0255252
ACP6	460.0345	463.174691	784.2946466	547.100917	882.6631287	621.5577611
MT2P1	0	0	0	0	0	0
WDR64	0	0	0	0	1.007606311	2.718182629
KIF26B	15.6274989	26.4090833	16.04694929	8.04560172	5.038031556	3.624243505
TFB2M	550.869338	565.763823	759.2212883	615.488532	386.9208235	936.866946
CNST	888.814002	804.461306	1702.982494	1572.91514	533.0237387	1446.979219

PPP1R21	393.61763	352.459688	334.9800665	419.37699	451.4076275	414.0698204
KLHDC8A	0	0	0	2.01140043	0	0
PM20D1	0	0	0	0	0	0
PKDCC	58.603121	55.8653685	0	0	54.41074081	9.060608762
OXER1	0.97671868	2.03146794	6.017605984	1.00570022	0	4.530304381
HAAO	11.7206242	5.07866986	8.023474646	0	0	5.436365257
B3GALNT2	1132.99367	952.758466	800.3415959	870.936387	657.9669213	1490.470141
C1orf147	0	0	0	0	0	0
MAPKAPK2	3719.34475	3532.72276	3593.513707	4099.23408	3848.048503	4957.059054
IL20	0	0	0	0	0	3.624243505
IL24	262.737326	882.672822	0	0	0	1.812121752
FCMR	137.717334	169.627573	2.005868661	1.00570022	12.09127574	0.906060876
PIGR	0	1.01573397	0	0	0	2.718182629
FCAMR	1.95343737	0	0	0	0	0
CAPN2	20798.2477	19392.393	4245.421022	5844.12395	2115.973254	3798.207193
MRPL55	404.361535	400.199185	1372.014164	1070.06503	488.689061	1080.930625
C1orf145	6.83703079	3.04720192	70.20540315	161.917735	29.22058303	80.63941798
WDR26	4424.53564	4129.97433	6785.853682	5842.11255	3048.009092	7235.802157
REL	707.310369	540.644721	216.3730525	216.989879	1451.043773	498.1975728
PUS10	99.6253058	108.683535	218.6396841	233.32245	138.0420646	194.8030884
PEX13	891.744158	885.720024	1135.321662	995.643213	1391.504316	1246.739766
KIAA1841	450.238012	419.04105	559.8178847	587.75132	357.6397841	331.0837048
TRIM17	0	0	1.002934331	5.02850108	0	2.718182629
RFTN2	0	1.01573397	0	0	1.007606311	0
DISC1	211.820981	174.889075	47.13791354	46.1113549	51.3274655	422.2243683
AC007364.1	1.95343737	0	0	0	0	1.812121752
CAPN13	0	0	1009.954871	839.75968	235.7798768	1356.373132
LRRTM1	0	0	0	0	0	0
MEMO1	1093.73935	1172.05543	891.5283853	1000.56109	1218.054965	919.3256074
DPY30	769.654323	940.650917	814.2121777	834.499868	1063.024658	834.482067
TYW5	375.059975	336.207945	407.1913383	304.727165	519.9248566	443.9698293
C2orf47	489.336061	518.024326	498.4583624	517.995953	756.6216552	537.2940996
KCNF1	0	6.09440383	0	0	46.34989032	7.248487009

PQLC3	360.409194	426.608268	53.15551953	102.581422	586.4268732	110.5394269
ARL5A	1655.53817	1723.70055	660.9337239	753.269461	1720.99158	848.0729801
FAM84A	0.97671868	0	161.4724272	130.741028	383.8980046	281.7849325
KCNJ3	0	0	1.002934331	1.00570022	320.418807	24.46364366
NEUROD1	0	0	0	0	0	0
CLHC1	93.7649937	106.652067	94.27582709	50.2850108	32.24340196	72.48487009
PRORSD1P	12.6973429	10.1573397	15.04401496	6.03420129	53.4031345	8.154547886
FRZB	0.97671868	0	11.03227764	5.02850108	2.015212623	3.624243505
DUSP19	20.5110924	20.3146794	14.04108063	39.2223084	31.23579565	36.24243505
CFAP36	611.425896	840.011995	514.5053117	665.773543	667.0353781	416.788003
NUP35	782.351666	771.957819	361.0563591	356.017876	591.4649047	457.5607425
CCDC138	527.428089	565.763823	196.5751288	196.111542	361.7306658	374.2031419
C2orf48	73.2539013	46.7237627	76.22300913	50.2850108	23.17494516	70.67274834
ZSWIM2	0	0	0	0	0	0
FBXO41	1199.41054	1246.30558	1446.231305	1440.16271	924.9825938	2216.224903
ALMS1P	0	2.03146794	18.05281795	7.09018652	2.015212623	15.4030349
ACTG2	1.95343737	0	1.002934331	1.00570022	1974.374339	5.436365257
C2orf44	465.894812	548.496345	166.4870989	213.208446	405.0577371	265.4758367
SMC6	3102.05854	2577.93282	973.8492351	863.896485	1769.356683	1217.745818
VSNL1	2.93015605	4.06293589	0	0	3.022818934	0.906060876
CCDC74A	123.877231	229.830126	1.002934331	0	648.2535964	34.59340425
H3F3A	5364.99852	5200.61888	13857.88465	13074.5453	6411.247818	12850.45301
ANKRD30BL	0	0	0	0	0	0
ADCK3	1034.34509	797.351168	723.1156524	980.55771	429.2402886	1145.260947
SLC16A14	7.81374947	20.3146794	3220.422136	963.460806	354.6774216	1423.421636
TEKT4	0	0	0	0	0	0
EN1	0	0	0	0	0	0
SGCB	1731.72223	1718.62188	9.026408976	8.04560172	622.7007004	82.45153973
SPATA18	0	2.03146794	45.13204488	22.1254047	346.6165711	67.04850484
NOSTRIN	6.83703079	11.1730737	2.005868661	1.00570022	7.053244179	4.530304381
CFAP221	0	0	4.011737323	4.02280086	1.007606311	9.060608762
CCDC140	0	0	0	0	0	0
SGPP2	0	2.03146794	1.002934331	1.00570022	73.55526072	9.060608762

INHBB	570.403711	267.138035	9222.984105	6554.1483	7183.225393	5909.329034
XIRP2	4.88359342	19.2989455	0	0	0	0
BBS5	202.942608	226.508676	137.391974	77.4389166	223.3258628	188.7777836
BIRC8	0	0	0	0	0	0
SMARCAD1	1272.66445	1246.30558	916.6819783	988.603312	847.3969078	1191.470052
HPGDS	0	0	0	0	0	0
PDLIM5	3487.86242	2763.81214	748.1890107	1206.84026	1423.747718	1041.063947
PDHA2	0	0	0	0	0	0
STPG2	0	0	3.008802992	2.01140043	1.007606311	0.906060876
NEURL3	4.88359342	7.11013781	0	1.00570022	3.022818934	0.906060876
RPRD2	1536.37849	1466.71986	2301.734289	2547.43865	4121.109813	2758.955368
ANKRD23	66.3875689	35.6319477	184.8207385	135.578446	220.4642609	147.4614076
CTSS	357.479038	619.597723	2.005868661	0	20.15212623	42.58486118
MSX1	0	0	0	0	79.60089859	7.248487009
PACRGL	226.598735	241.744685	125.3667913	115.655525	188.4223802	198.4273319
BNIPL	6.83703079	8.12587178	66.19366583	56.3192121	24.18255147	96.04245287
C1QTNF7	0	0	0	0	0	0
TNFAIP8L2	0	0	0	2.01140043	0	0
LYSMD1	108.415774	82.2744518	433.2676309	392.223084	289.1830113	289.0334195
SCNM1	420.965753	335.192211	582.7048461	534.026814	865.5338214	655.9880744
TMOD4	0.16604218	2.99641522	2.39701305	3.93228784	5.491454397	1.658091403
VPS72	735.303127	673.48241	1883.119529	1254.19868	1735.652252	1227.866518
ERCC3	2117.52611	2326.0308	1445.228371	1573.92084	2261.068563	2025.952119
RNF149	1634.05036	1925.83161	1615.727207	1403.9575	1229.2797	1662.621708
IWS1	1777.628	1633.30023	1769.176159	1356.68959	1705.877485	1957.997553
BOLA3	590.914804	661.242816	545.5962759	422.394091	790.9709544	508.3001515
CDC42EP3	9048.32189	7776.45929	22.06455528	32.1824069	184.391955	109.633366
S100A11	3962.5477	4437.74172	4610.489118	5994.97898	9248.818331	4823.868105
LCE3D	0	0	0	0	0	0
SMCP	0	0	0	0	0	0
IVL	0	0	0	0	4.030425245	0
SPRR3	0	0	0	0	3.022818934	5.436365257
DHX57	1096.85508	1216.8493	538.5757356	562.18642	1301.827354	964.9548331

SPRR2D	0	0	0	0	1.007606311	0
BMP10	2.93015605	2.03146794	0	0	0	0
PGLYRP4	0	0	0	0	0	0.906060876
ARHGAP25	36.1385913	28.4405512	1.002934331	0	0	14.49697402
S100A9	0	0	2.005868661	2.01140043	613.6322436	333.4304024
S100A12	0	0	0	0	10.07606311	1.812121752
TGFA	2852.01856	2946.64425	9.026408976	17.0969037	217.6429632	37.14849592
TDRD10	0	0	0	0	0	0
CCNYL1	940.580093	1090.89829	371.0857024	442.508095	1056.979021	582.5971434
FZD5	474.68528	542.401941	106.3110391	311.767067	973.3476967	214.7364277
CRYGC	0	0	0	0	0	0
DCAF16	1398.66116	1505.31775	619.8134164	365.069178	913.8989243	682.2638398
C1orf189	0	0	1.002934331	0	0	2.718182629
NPPC	0	0	0	0	13.09888205	3.624243505
GNPDA2	222.69186	266.122301	311.9125769	326.85257	54.41074081	322.5576719
ALPP	788.211978	85.3216537	0	0	32.24340196	3.624243505
GABRG1	0	0	0	0	0	1.812121752
ALPPL2	0	0	0	0	0	0
GABRB1	0	0	0	0	0	0
PAQR3	1177.92273	1338.73738	167.4900332	154.877833	358.7078468	264.5697758
NIPAL1	536.218557	554.590749	56.16432252	65.370514	196.4832307	93.32427025
ALPI	0	0	0	0	0	0
ANTXR2	2057.94627	2750.6076	7.020540315	0	114.8671195	77.01517448
HELQ	237.34264	224.477208	254.74532	284.613161	308.3275313	328.9000981
MRPS18C	468.824968	430.671204	448.3116458	438.485294	715.390405	459.3728642
CGGBP1	2840.29793	2771.93801	2164.332286	2242.71148	2564.358062	1972.494527
FAM175A	569.426993	493.646711	140.4108063	239.356651	368.7839099	216.5485494
GPR155	97.6718684	109.699269	48.14084787	47.2679101	75.57047335	76.1091136
DAPL1	0	0	0	4.02280086	0	0
PMVK	462.964656	495.678178	1509.416168	1600.06904	666.0277718	1147.073069
PBXIP1	595.798397	637.880935	980.8697754	1785.11788	318.4035944	1263.048861
CLDN1	476.638718	276.27964	0	0	79.60089859	26.27576541
PYGO2	1279.50148	1437.98474	2237.546492	2153.20416	1588.995153	2504.352262

HIPK1	2658.62826	2651.06567	2090.115145	2369.42971	19587.86669	3888.813281
LENEP	0	0	1.002934331	0	0	0
DCST2	0	3.04720192	53.15551953	22.1254047	13.09888205	13.59091314
DCST1	0	0	2.005868661	0	1.007606311	8.317638843
COL6A3	86.9279629	220.414272	5.014671654	6.03420129	14.10648836	4.530304381
C1orf106	461.987937	520.055794	240.4334471	272.544758	379.8675794	414.0698204
LINC01116	450.267313	455.04882	1.002934331	1.00570022	77.58568597	22.6515219
YY1AP1	1526.59177	1483.12396	3276.375842	4124.35647	2125.857872	3673.651004
KBTBD8	6.83703079	3.04720192	45.13204488	47.2679101	129.9812142	123.2242792
FAM19A4	0	0	0	0	0	0
EOGT	778.444791	762.816213	122.3579883	216.225546	346.6165711	243.7303757
LMOD3	0	0	1.002934331	0	13.09888205	0.906060876
APOA1BP	1115.41274	1176.21994	2881.430332	2691.25378	1466.067183	3289.000981
POGLUT1	515.707465	519.04006	233.6836991	214.214146	508.8411872	424.0364901
SLC22A15	291.062168	294.562852	0	0	173.3082855	29.90000891
CCKAR	0	0	0	0	0	0
IGFN1	0	4.06293589	3.008802992	1.00570022	182.3767423	115.9757922
ATP1A1	20825.5958	21881.957	9093.605577	9646.67647	17852.76862	12230.00971
SLC15A2	1.95343737	2.03146794	577.6901745	342.943774	165.2474351	295.3758456
EIF4E3	51.7660902	40.6293589	2750.045935	2466.98263	598.5181489	2417.370418
PROK2	0	0	0	0	0	0
C3orf30	0	0	0	0	0	0
LRRC58	3193.8701	3985.74011	2511.327505	3514.92225	5568.0224	3144.03124
FSTL1	10937.2958	7406.73213	8.023474646	6.03420129	127.9660015	70.67274834
LMOD1	4.88359342	5.07866986	2.005868661	0	0	0.906060876
ELF3	149.437959	40.6293589	3646.809637	5108.42407	3599.784384	2136.147243
PDCL2	0	0	0	0	0	0
TMEM183A	1521.464	1566.70871	6432.209007	7068.48351	4285.99451	5988.971785
TMEM169	11.7206242	3.04720192	2.005868661	0	31.23579565	1.812121752
IGFBP7	4059.24285	3256.44312	0	0	118.8975447	49.83334819
TRIM46	5.8603121	9.14160575	52.1525852	92.5244198	29.22058303	110.5394269
KRTCAP2	643.657613	709.998047	1516.436708	1333.55849	928.0054127	1089.991234
CXCR1	0	0	1.002934331	0	0	4.530304381

ARPC2	6283.23129	6335.13278	3916.458561	4123.37088	6292.501414	5748.050198
TSACC	11.7206242	19.2989455	3.008802992	1.00570022	13.09888205	3.624243505
CCT3	7978.81493	8232.52385	12001.1122	10893.7447	14210.27181	16061.74115
TMEM79	111.34593	87.3531216	879.573408	922.227098	341.5785395	1322.848879
SSR2	2901.83121	3164.01132	6555.178786	4356.69333	3575.994799	3740.219297
RNF25	461.011219	455.04882	548.6050789	493.798806	553.1758649	571.7244129
STK36	474.68528	442.860012	1193.491854	1114.31584	708.3472368	1149.791252
ADORA1	95.718431	107.667801	77.22594346	212.202745	37.28143352	345.2091938
NEK10	39.0687474	46.7237627	1.002934331	0	11.08366942	10.87273051
CCDC141	0	1.01573397	0	0	0	0
FEV	0	0	0	1.00570022	0	0.906060876
CRYBA2	0	0	10.02934331	8.04560172	0	12.68485227
IHH	0	0	1.002934331	2.01140043	0	4.530304381
KIAA1524	2174.17579	1996.93299	388.135586	795.508871	1269.583952	949.5517982
EOMES	0	1.01573397	16.04694929	13.0741028	25.19015778	22.6515219
CWC22	1101.73868	1178.25141	516.5111803	473.684802	696.2559611	569.0062302
AZI2	1243.36288	1228.02237	1402.102194	1164.60085	869.5642466	1123.515486
TGFBR2	6189.4663	7623.08346	2.005868661	21.1197045	857.4729709	216.5485494
RETNLB	3.90687474	2.03146794	0	0	0	0
ANKZF1	1067.55352	795.3197	1730.06172	1083.13913	781.9024976	1037.439703
HDAC11	382.873724	311.830329	365.0680964	311.767067	453.4228401	347.9273765
FCRL4	0	0	0	0	0	0
TRAT1	0	1.01573397	0	0	0	0
FBLN2	0	0	3.008802992	2.01140043	4.030425245	1.812121752
GLB1L	120.136398	175.721977	246.7218454	242.373752	79.60089859	212.9243059
STT3B	7987.6054	8688.5884	6222.204588	6288.64345	6126.246373	8071.190285
CHCHD4	631.936988	601.314512	412.2060099	412.337088	615.6474562	540.9183431
DPPA2	0	0	0	0	0	0
NFASC	0	0	0	1.00570022	22.16733885	17.21515665
FCRL1	0	0	0	0	0	0.906060876
SGOL2	2521.88764	2469.24929	336.9859351	774.389166	1098.290879	770.1517448
SERPINI1	11.7206242	31.4877531	3.008802992	9.05130194	18.1369136	15.4030349
CLASP2	2205.43079	2166.56056	945.7670739	848.810982	1367.321764	1258.518557

SUCLG1	1446.52037	1572.35619	2409.048262	2334.2302	2113.958041	2170.015798
NUAK2	368.222944	259.012163	360.0534247	600.403029	238.8026958	546.3547083
SPTA1	0	0	0	0	0	0.906060876
PRKCI	2869.59949	2696.7737	1790.23778	2019.44603	2264.091381	2449.988609
MNDA	0	0	0	0	0	0
PYHIN1	0	0	0	1.00570022	0	0
IFI16	1749.30316	2174.68643	1.002934331	0	76.57807966	47.11516556
AIM2	39.0687474	23.3618814	0	0	1.007606311	0
EFHB	0.97671868	1.01573397	4.011737323	2.01140043	5.038031556	2.718182629
EIF5A2	676.866048	911.113373	0	0	340.5709332	31.71213067
SLC2A2	0	1.01573397	0	0	0	0
RPL22L1	798.955883	817.665848	527.543458	391.217384	1646.428713	612.4971523
FABP1	0	0	0	0	0	0
PPM1L	195.343737	246.823355	275.8069409	607.44293	747.643883	460.2789251
ICA1L	93.7649937	110.715003	16.04694929	34.1938073	162.2246161	32.08361563
SNHG16	2902.80793	2484.4853	4877.26965	4603.08989	2775.955388	3983.949673
CTLA4	6.83703079	2.03146794	0	0	0	0
ICOS	0	0	0	0	0	0.906060876
RYBP	1583.26099	1663.77225	2026.930282	2696.28228	1452.968301	2900.300865
PPP4R2	2909.53752	3015.69385	2358.881487	2669.0278	2588.490233	3288.910374
CD200R1	0	1.01573397	0	1.00570022	0	0
GTPBP8	321.340447	361.601294	92.26995843	171.974737	245.85594	179.4000535
C3orf17	772.584479	739.454332	895.6203573	828.696978	1332.055544	936.866946
SPICE1	261.760607	278.311108	687.0100165	435.468193	921.9597748	723.9426401
FAM86KP	0	0	0	0	0	0.915121485
CCDC191	28.3248418	12.1888077	174.5105735	125.712527	78.59329228	109.633366
CADPS	0	0	0	0	4.030425245	1.812121752
NKX6-1	57.6264023	57.8968364	0	0	1.007606311	0.906060876
CDS1	20.5110924	18.2832115	2423.089343	2187.39797	1686.732965	1599.197446
WDFY3	3247.58962	2595.2003	2252.590507	2937.65033	3098.389407	3632.398053
COX18	482.49903	482.473637	592.7341894	485.753204	393.9740677	581.6910825
PTPN13	459.057781	589.125704	1558.55995	1635.26855	1647.436319	1822.088422
SYNPR	0	0	0	0	0	0.906060876

ALB	0	1.01573397	440.2881712	587.328926	0	520.9850038
C3orf49	0	0	0	0	2.015212623	0.906060876
C4orf36	82.0443694	111.730737	110.3227764	121.689726	72.54765441	85.16972236
THOC7	1436.75318	1445.38944	1181.456642	855.850883	7221.514433	1369.964045
ATXN7	1515.8674	1304.20242	978.8639068	1168.62365	2435.384454	1562.955011
PSMD6	2554.11936	2483.46956	1972.771829	1480.39072	33729.62127	3677.701096
PRICKLE2	853.65213	884.70429	370.082768	914.181496	1729.05243	1231.336731
ADAMTS9	362.362632	704.919377	0	0	70.53244179	27.18182629
PPM1K	390.687474	536.307537	446.3057772	290.647362	407.0729498	537.2940996
ERICH6	0	1.01573397	0	2.01140043	0	0
CLRN1	0	0	2.005868661	0	0	0
GMPS	3300.3129	3047.0394	4278.427591	3644.52684	4750.783149	4339.03493
TIPARP	1258.99038	1517.50655	365.0680964	545.089517	565.2671406	694.948692
CCNL1	1155.4582	1174.18847	2217.487805	1506.53892	2046.448418	1833.867213
PTX3	4306.35268	3643.43776	1.002934331	0	170.2854666	71.57880922
HESX1	8.79046815	6.09440383	7.020540315	17.0969037	6.045637868	12.68485227
DCLK3	0	0	0	0	0	2.718182629
SLMAP	2928.20261	2676.45902	1682.923807	1855.5169	1392.511922	2097.530928
RPL9	7527.86391	7419.76399	11348.15181	9140.02481	8938.908858	7424.806456
SMIM14	128.926866	138.13982	6880.129509	5567.55639	3168.921849	3144.03124
RPP14	817.562374	692.03987	624.0457993	608.368174	708.528606	1009.079998
ABHD6	355.525601	518.024326	191.5604572	286.624561	60.45637868	212.9243059
DNASE1L3	0	2.03146794	0	0	0	0
C3orf67	67.3935892	60.9440383	17.04988362	23.131105	84.63893015	30.80606979
RBM47	276.411388	327.066339	4458.0431	6721.09454	2137.132986	4793.968096
APBB2	3542.55867	2940.54985	1155.380349	974.523509	1519.470317	1454.227706
IL17RE	247.109827	252.917759	316.9272485	731.144057	46.34989032	107.8212443
IL17RC	683.703079	768.910617	1666.876858	1978.21232	321.4264133	1289.324627
CRELD1	405.338254	470.284829	611.7899417	655.716541	204.5440812	594.3759348
PRRT3	77.160776	78.2115159	822.4061512	1215.89156	806.085049	1191.470052
FANCD2OS	0	0	0	0	0	0
PCOLCE2	75.2073387	134.076884	0	0	92.69978064	7.248487009
U2SURP	3362.84243	2930.39251	3588.499035	2496.14793	4062.668647	3779.179915

MTMR14	1212.10789	1255.44719	713.0863091	832.719778	1580.934302	803.6759972
TTC14	440.500126	360.58556	2542.438528	1521.62443	562.2443217	1463.288315
CXCL3	180.321803	294.562852	0	0	9.068456802	1.812121752
CXCL5	2.93015605	2.03146794	0	0	1.007606311	0
PPBP	0	0	0	0	0	0
PF4	0.97671868	1.01573397	0	0	0	0
MTHFD2L	261.760607	260.027897	227.6660931	271.539058	148.1181278	277.2546281
CXCL1	1544.19224	2417.44685	0	0	55.41834712	15.4030349
RCHY1	620.216364	559.669419	600.7576641	777.406267	496.7499115	728.4729444
PLSCR2	0	0	0	0	1.007606311	0
CCDC158	0	0	3.008802992	2.01140043	1.007606311	0
CPA3	3.90687474	2.03146794	0	0	0	0.906060876
GYG1	554.776212	636.865201	854.5000498	1110.29304	2232.855586	1205.060965
HPS3	1120.29633	1392.57128	861.5205901	1311.43308	1368.329371	926.9002763
TM4SF18	0.97671868	1.01573397	4.011737323	0	5.038031556	7.248487009
TOPBP1	2512.12045	2139.13575	1723.04118	2159.23836	3114.511108	3131.346388
RYK	1650.65458	1557.12018	1174.436101	1180.69205	1603.101641	1605.539873
SNRK	985.509152	876.578418	453.3263175	433.456793	436.2935328	495.6152993
TCF23	0	0	0	0	0	0
DNAJC5G	2.93015605	0	3.008802992	1.00570022	3.022818934	1.812121752
UCN	32.2317166	36.566423	57.16725685	40.2280086	13.09888205	28.08788716
ZNF513	104.508899	97.5104613	659.9307896	504.861508	75.57047335	472.0577165
SLC4A1AP	1266.73576	1213.78178	1181.356348	1124.37284	1191.98819	1099.930722
PLB1	161.158583	213.304134	3.008802992	3.01710065	3.022818934	15.4030349
SPDYA	10.3825196	17.6737711	16.09709601	12.3801697	1.319964268	14.86845898
KIAA1143	1856.06828	1748.71808	1870.472527	1910.00574	1052.636237	1436.070246
KIF15	2013.01721	1766.36138	480.4055444	676.836245	701.2939927	1111.736695
TGM4	1.95343737	6.09440383	7.020540315	2.01140043	0	9.966669638
WDR43	3332.56415	2954.77013	731.1391271	674.824845	3349.283379	1819.370239
ZDHHC3	2229.84876	2138.12001	3460.123441	3161.92148	1706.885091	2922.046326
CDCP1	15830.6564	18689.5051	2276.660931	2734.49889	449.3924148	2922.952387
CLEC3B	1.12322649	20.3146794	25.07335827	33.1881071	0	21.74546103
SLC6A20	0.97671868	0	1.002934331	1.00570022	0	0

LZTFL1	443.430282	428.639736	271.7952036	308.749966	122.92797	212.018245
FYCO1	1557.8663	1443.35797	3809.144588	3085.48826	1276.637196	3142.219119
CCR1	6.83703079	7.11013781	0	0	3.022818934	0.906060876
RTP3	0.97671868	5.07866986	0	0	0	0
LRRC2	6.83703079	3.04720192	0	0	0	0.906060876
ELP6	885.883846	874.54695	582.7048461	723.098455	499.7727304	636.0547351
FBXO40	0	0	0	0	0	0
DTX3L	1283.40835	1091.91402	346.0123441	637.613937	804.0698364	1687.991412
ZNF148	1305.87288	1214.81783	1249.656176	1341.60409	1986.999646	1606.445933
NMNAT3	2.93015605	3.04720192	104.3051704	126.748398	26.19776409	101.5331818
SMIM12	859.512442	829.854655	574.6813715	673.819144	704.3168116	761.9971969
ZMYM6	420.233214	408.61962	732.6736166	655.978023	405.0879653	619.9087303
TPRA1	911.278532	1307.24962	692.0246882	704.995851	737.5678199	707.6335443
YEATS2	2438.86655	2352.43988	1107.239501	1013.74582	1300.819748	1631.815638
GRIK3	0	0	0	0	65.49441023	5.436365257
ZC3H12A	947.417123	1307.24962	326.9565918	615.488532	884.6783413	600.7183609
MEAF6	738.399325	706.950845	837.4501661	1266.17657	1078.138753	1072.776077
SNIP1	518.022288	547.480611	506.481837	530.004014	544.1074081	544.5425866
DNALI1	1.95343737	0	0	0	3.022818934	4.530304381
POLR2H	1465.07803	1511.41215	1874.484264	1714.71887	1407.626017	1785.845987
KLF15	145.531084	144.234224	207.6074065	114.649825	8.06085049	68.86062659
CFAP100	0	0	0	0	0	0
CAMK2N2	539.148713	551.543547	660.9337239	830.708378	161.2170098	852.6032845
LIPH	318.410291	327.066339	44.12911055	34.1938073	109.8290879	111.4454878
TMEM41A	709.097764	703.903643	676.9806732	558.16362	757.7199461	649.6456482
RPN1	5235.21215	5636.30781	8006.424762	6777.41375	9339.502899	7664.368952
SENP2	1416.24209	1504.30201	1703.985428	1739.86137	1423.747718	1678.930804
HEYL	124.043273	108.683535	0	0	90.68456802	12.68485227
IFT122	399.477942	310.692707	563.6490939	705.941209	952.1879642	934.1306421
RHO	0	0	0	1.00570022	0	0
IGF2BP2-AS1	0	0	0	0	0	0
RFC4	1510.00709	1391.55554	846.4765751	795.508871	949.1651452	1306.539783
RPL39L	424.872627	434.73414	0	0	405.1081175	21.74546103

BAP1	3183.12619	3483.96752	2497.306483	2671.13977	1467.074789	2933.825117
TKT	12002.8959	15244.1355	15470.26205	19201.8342	16931.81645	13125.19785
PRKCD	1443.59021	1581.49779	4142.118786	2695.27658	2926.088728	2785.231133
RFT1	485.429186	426.608268	530.5522609	387.194583	668.0429844	737.098644
SFMBT1	896.422641	770.942085	694.0305569	912.109753	694.2407485	1119.84594
GNL3	3386.28368	3162.99559	4679.691587	1583.97784	1846.942369	3748.373845
PBRM1	4630.62328	4092.39217	6696.592526	2914.51922	1747.189344	2931.106934
UVSSA	383.850443	409.340791	690.0188195	693.933149	330.4948701	516.4546994
FAM208A	3097.17495	2759.7492	2783.142768	3612.47517	2645.974173	4124.389108
ARHGEF3	503.986841	538.339005	208.6103408	331.881071	163.2322224	394.1364811
SLBP	2038.41189	1865.90331	1303.81463	1744.88987	1528.538774	1643.594429
LRPAP1	2062.82986	2176.7179	1724.044114	1636.27425	2699.377308	2072.161224
ZDHHC19	0	0	0	0	0	0.906060876
SLC51A	0	0	73.21420614	103.587122	10.07606311	110.5394269
UBXN7	3234.89228	2693.72649	4951.486791	4646.335	1801.600085	5497.071336
RNF168	1468.9849	1581.49779	2099.141554	4904.79995	1087.20721	3005.403926
PIGX	801.143733	841.332449	2960.662144	2814.94485	1053.946126	2608.549263
MFI2	2390.03062	2650.04993	211.6191438	346.966574	353.6698153	279.9728107
OTOP1	0	0	0	0	0	0
S100P	1.95343737	0	645.889709	3.01710065	798.0241985	338.8667677
ABLIM2	6.83703079	7.11013781	21.06162094	29.1653063	81.61611121	46.20910469
EXO5	257.853733	251.902025	196.5751288	174.991838	229.734239	208.3940015
CLDN19	0	0	0	0	0	0
C1orf50	92.788275	98.5261953	141.4137406	212.202745	213.612538	201.1455145
ERMAP	62.5099958	68.0541761	146.4284123	381.160382	234.7722705	287.2212977
ZNF691	95.718431	112.746471	287.8421529	265.504857	156.1789782	269.1000802
AIMP1	1078.29743	1038.08012	1625.75655	807.577273	1429.793356	1012.97606
SGMS2	1547.1224	1395.61848	74.21714047	32.1824069	425.2098634	153.1242881
METAP1	1397.68444	1422.02756	898.6291603	1193.76616	2372.912863	1308.351905
DNAJB14	852.655877	908.066171	1501.372634	1300.33015	1140.600268	1330.097366
H2AFZ	7590.08089	7362.03983	13720.14164	11070.748	10059.94141	10629.00014
EMCN	0	0	0	0	37.28143352	8.154547886
SLC9B1	1.95343737	0	9.026408976	1.00570022	2.015212623	6.342426133

SLC9B2	0	0	0	0	120.9127574	9.060608762
BDH2	158.228427	165.564637	438.2722732	303.721465	54.41074081	289.9213592
PGRMC2	1926.08924	1847.6201	2097.135686	2377.47531	1294.77411	1794.906596
CDC25A	1216.01476	1263.57306	422.2353532	603.420129	710.3624495	884.3154151
CAMP	0	0	137.4020033	21.1197045	1.007606311	7.248487009
ZNF589	318.410291	350.42822	292.8568246	254.442155	211.5973254	265.4758367
FBXW12	0	0	1.002934331	0	0	0
PLXNB1	193.390299	194.005189	2586.567639	2917.53633	1150.686407	2927.482691
CCDC51	357.244626	336.207945	558.6344222	478.713303	369.8217444	615.2153349
ATRIP	315.187119	367.583967	227.8466213	341.093285	406.3474732	458.6208337
SHISA5	3372.60962	3591.63533	2822.257207	3164.93858	2565.365669	3207.455502
SPRY1	516.684184	363.632762	2810.221995	3526.99066	7.053244179	1559.330768
BSN	32.2317166	43.6765608	0	0	126.9583952	9.060608762
APEH	2679.13935	2625.67232	3367.853483	4010.73246	2483.749557	5319.483404
INTU	248.086546	231.587346	421.2324189	250.419354	153.1561593	374.2031419
RNF123	1293.17554	1274.74614	1612.718404	1550.78973	543.0998018	1550.270159
HSPA4L	1340.05803	1323.50137	807.3621362	895.073192	1188.975447	1302.91554
MFSD8	341.851539	443.875746	478.3996757	504.861508	454.4304464	584.4092651
ABHD18	277.388106	229.555878	551.6138819	650.688039	384.9056109	598.0001783
CAMKV	0.97671868	0	0	0	2.015212623	0.906060876
MON1A	544.032307	506.851252	422.2353532	427.422592	265.0004599	343.3970721
MST1R	516.684184	632.802265	3165.260748	4507.54837	226.71142	3554.476817
RAD54L2	1587.10926	1391.34224	1369.005361	1118.73086	1065.039871	1367.06465
TEX264	1194.58555	1298.32132	1546.524738	1560.45451	722.4537252	1269.5725
GRM2	2.93015605	5.07866986	3.008802992	1.00570022	9.068456802	0.906060876
DUSP7	1725.86191	1574.38766	516.5111803	463.627799	375.8371541	784.6487188
POC1A	1219.92164	1118.3231	539.5786699	591.351727	436.2935328	896.0942065
PPM1M	354.548882	380.90024	452.3233831	182.031739	76.57807966	215.6424885
ETNPPL	0	0	1.002934331	1.00570022	2.015212623	0
WDR82	4834.75749	4749.57205	2588.573508	2689.24238	2792.077089	4115.3285
PITX2	17.5809363	21.3304134	1.002934331	2.01140043	75.57047335	10.87273051
C4orf3	1775.67457	1651.58344	1662.86512	1859.5397	1744.166525	1647.218673
PRSS12	516.684184	570.842492	0	0	25.19015778	16.30909577

NDST3	10.7439055	14.2202756	1.002934331	17.0969037	2.015212623	1.812121752
HMGB2	5957.98397	4694.72242	2416.068803	4788.13873	6059.744356	4250.33157
SAP30	585.054492	628.739329	518.517049	805.565873	462.4912969	664.1426222
SCRG1	0	0	0	0	0	0.906060876
HAND2	0	0	0	0	0	0
MAD2L1	2297.16421	2104.57032	1198.506525	1284.20878	1836.856229	2220.755208
ANXA5	7901.65415	8382.85247	5364.695735	5065.71199	3656.603304	3499.207104
TMEM155	0	0	1.002934331	0	0	0
ADAD1	0	0	0	0	0	0.906060876
MAP9	467.84825	603.34598	1909.586966	1409.9917	228.7266327	1374.494349
GUCY1A3	0.97671868	1.01573397	577.6901745	1156.55525	0	1729.670213
FBXO8	344.781695	437.781342	459.3439235	572.243423	206.5592938	309.8728197
CEP44	335.014509	370.7429	462.3527265	411.331388	208.5745064	347.9273765
HPGD	5.8603121	2.03146794	7.020540315	4.02280086	15.11409467	36.24243505
ASB5	0	0	1.002934331	1.00570022	0	2.718182629
C4orf45	0	0	0	0	0	0
TMEM144	61.5332771	92.4317915	732.1420614	504.861508	163.2322224	503.7698472
FAM198B	0.97671868	9.14160575	3546.375793	1217.90296	154.1637656	2142.833972
NPY1R	6.83703079	14.2202756	50.14671654	65.370514	3019.796115	96.94851375
NPY5R	0	0	0	0	61.46398499	1.812121752
NAA15	3584.55757	3729.77515	1634.782959	1621.18875	3707.991226	2777.076585
IL15	59.5798397	52.8181666	12.03521197	27.1539058	8.06085049	30.80606979
FAM160A1	133.81046	166.580371	429.0954241	640.631037	237.4424273	431.2849771
ARFIP1	1066.5768	1171.14127	1544.518869	1477.37362	1072.093115	1188.75187
ICE1	3462.46773	3075.64247	1347.94374	1632.25145	2327.570579	2041.355154
HHIP	31.2549979	41.6450929	0	0	5.038031556	2.718182629
ANAPC10	222.69186	227.52441	236.692502	191.083041	181.369136	153.1242881
ABCE1	5292.75064	5233.03095	2182.385104	1458.26531	4675.242904	3528.201052
OTUD4	3188.9865	2721.15131	1242.625606	1293.28019	2535.107251	2234.327999
LSM6	538.171995	522.087262	169.4959019	189.071641	386.9208235	240.1061322
TMEM184C	946.440405	1002.52943	951.7846798	966.477907	1507.379042	1388.991323
PRMT9	187.529987	210.256932	303.8891022	307.744266	398.004493	368.7667766
ITGA2	3300.33243	3665.78391	734.1479301	1198.79466	606.5789994	1075.49426

MOCS2	1389.87069	1387.49261	1118.271779	940.329702	785.9329228	1060.997286
SLC45A2	2.93015605	2.03146794	0	1.00570022	0	7.248487009
EDIL3	2621.51295	1740.96803	0	1.00570022	6.045637868	4.530304381
TMEM161B	847.791818	885.720024	640.7145678	483.741804	619.6778814	714.8276677
ELOVL7	533.288401	422.545332	318.9331172	532.015414	1237.34055	316.2152458
NDUFAF2	780.241953	812.2723	304.5610682	194.914759	439.205515	291.7516021
ZNF474	0	2.03146794	2.005868661	0	0	1.812121752
LMBRD2	863.419317	860.326674	1068.125062	1264.16517	958.233602	1454.227706
RANBP3L	0	2.03146794	376.100374	125.712527	0	910.5911806
NIPBL	4105.14863	3746.02689	2856.356974	3623.53788	3094.358982	4008.413316
RNF180	46.8824968	37.582157	0	0	22.16733885	5.436365257
ADGRV1	313.526698	405.277855	0	2.01140043	648.8984645	28.08788716
SLC25A46	2013.99393	2206.17419	1335.908529	1427.08861	1340.116394	1505.873176
STARD4	1386.94053	2402.21084	8.023474646	1356.68959	116.8823321	37.14849592
PGGT1B	490.312779	517.008592	760.2242227	659.739341	795.0013796	904.2487544
F2RL2	43.9523408	137.124086	0	0	16.12170098	8.154547886
CCDC112	315.480135	363.632762	429.2558935	363.057778	242.833121	351.55162
ANKRD33B	1915.34534	1258.49439	2.005868661	1.00570022	104.7910564	28.99394804
CMBL	1160.3418	1376.31953	5242.337747	3896.08264	1596.048397	3484.71013
C5orf63	147.484521	104.620599	93.27289276	43.2451093	102.7758438	87.88790499
PRRC1	2356.82218	2482.45383	3247.501363	2601.74646	2241.924043	2719.088689
F2RL1	5276.23433	6502.72889	11.03227764	12.0684026	300.2666808	56.17577432
AGGF1	1242.38617	1249.35279	1189.480116	1382.8378	1227.264487	1508.591359
WDR41	921.045719	1056.36333	2024.924414	1249.07967	855.4577583	1327.379184
PRDM9	0	0	0	0	0	0.906060876
NDUFS4	1071.4604	1107.15003	520.5229176	584.311825	551.1606523	540.9183431
SCGB3A2	0	0	0	0	2.015212623	0
SPINK1	0	0	0	0	0	3.624243505
HTR4	0	0	0	0	1.007606311	0
ESM1	97.6718684	78.2115159	0	0	6.045637868	0.906060876
GRPEL2	1149.59789	965.963008	1006.946068	613.477131	846.3893015	781.9305361
CDC20B	0	1.01573397	0	1.00570022	1.007606311	8.154547886
ARSK	384.827161	425.592534	108.3169077	154.877833	78.59329228	136.8151923

RHOBTB3	9111.8086	8132.98192	48.14084787	92.5244198	17060.79006	941.3972503
GPX8	1630.14348	1795.81766	44.12911055	15.0855032	346.6165711	154.9364098
TIGD6	41.9989034	51.8024326	239.701305	261.482056	177.3387108	191.1788449
SPZ1	0	0	0	0	0	0
SERINC5	732.539013	771.957819	2191.411513	1771.03808	2024.281079	1382.648897
ENPP6	0	0	0	0	0	0
CAGE1	0	0	0	0	1.007606311	0.906060876
CASP3	1215.03804	1229.03811	1039.039967	1108.28164	185.3995613	1220.464
PRIMPOL	234.412484	214.319868	368.0768994	267.516257	75.57047335	318.0273675
ERAP1	2488.67921	2664.27021	287.8421529	340.932373	532.0161324	573.5365346
ERAP2	396.547786	318.940467	484.4172817	359.034977	16.12170098	888.8457195
CMYA5	15.6274989	13.2045416	425.2441562	1166.61225	488.689061	700.3850573
EGFLAM	0	0	100.2934331	111.632724	0	99.66669638
CFAP97	1649.67786	1350.92618	992.9049874	1207.84596	541.0845892	1568.391377
TMEM174	0	0	0	0	0	0
CARTPT	0	0	0	0	0	2.718182629
RICTOR	2930.15605	2208.20566	850.4883124	855.850883	959.2412083	1008.445755
PAPD4	1072.43711	1011.67104	1065.116259	824.674177	704.3168116	991.2305985
EBF1	0	0	0	0	0	0
ANKRA2	167.995614	205.178262	390.1414546	271.539058	180.3615297	253.6970453
UBLCP1	1329.31413	1460.62545	240.7042394	227.288249	442.3391707	365.1425331
FAM170A	0	0	1.002934331	0	1.007606311	0
UTP15	950.347279	1022.84411	482.4114131	447.536596	717.4156936	725.7547618
TLR3	91.8115563	130.013948	2.005868661	3.01710065	6.045637868	17.21515665
KLKB1	1.95343737	0	8.023474646	2.01140043	0	2.718182629
NSA2	2449.59092	2580.85814	2238.509309	1997.32063	2022.235639	1596.470203
GFM2	1125.17992	924.317915	1058.095719	766.343564	904.8304675	1058.279103
TERT	188.506706	133.06115	1.002934331	2.01140043	20.15212623	10.87273051
SLC6A18	0	0	0	0	0	0
CCDC127	1138.66841	1084.24523	935.3465862	571.237722	432.2631075	682.8980824
FOXQ1	127.950148	76.1800479	0	2.01140043	2.015212623	3.624243505
LINC01600	3.90687474	1.01573397	0	0	4.030425245	0
ADGRF2	0	0	0	0	0	0

ACSL6	6.83703079	11.1730737	0	1.00570022	2.015212623	1.812121752
IL3	0	0	0	0	0	0
CSF2	1183.78304	1670.88238	0	0	28.21297672	14.49697402
SEPT8	2588.30451	2298.60598	1383.046442	1254.10817	2675.194756	1581.98229
SHROOM1	741.329481	741.4858	78.2288778	231.31105	191.4451991	314.403124
GDF9	1.95343737	9.14160575	11.03227764	6.03420129	14.10648836	9.966669638
UQCRQ	2132.17689	2232.58327	1610.712535	1930.94441	2033.349536	2003.300597
LEAP2	8.79046815	6.09440383	109.319842	81.4617175	34.25861458	53.45759169
GJB7	0	0	0	0	64.48680392	2.718182629
SLC35A1	263.118246	222.44574	1573.463554	1103.59507	975.9271689	1371.033197
GRIK2	17.5809363	25.3933493	3.008802992	0	10.07606311	7.248487009
MB21D1	1779.58144	2043.65675	278.8157439	458.599298	513.8792188	299.0000891
FABP7	0	0	301.8832335	55.3135119	0	2018.703632
TLX3	0	0	0	0	0	0
TXLNB	0	0	4.011737323	0	27.14491403	18.53800553
CITED2	2523.84108	1745.03096	2789.160374	2157.22696	903.8228612	2382.034043
FAM26D	0	0	0	0	0	0
T	0	0	0	0	0	0
CREBRF	584.077773	677.494559	390.1414546	485.753204	143.0800962	235.5758278
DCBLD1	887.837284	746.56447	303.8891022	390.211684	523.9552819	428.5667944
SFXN1	3183.12619	3277.77353	1675.903267	1842.44279	4710.559505	2618.515932
SAMD3	0	0	0	0	0	0
TMEM200A	0	0	0	0	1.007606311	0
IL22RA2	0	0	0	0	0	1.812121752
DACT2	0	0	0	0	0	0
PDSS2	353.572164	388.010377	483.4143474	549.112318	572.3203848	999.3851464
C7orf72	0	0	0	0	0	0
STXBP5	926.906031	923.302181	352.0299501	563.192121	1267.56874	589.8456304
HIST1H2AA	0	0	0	0	0	0
IL31RA	349.665289	478.410701	0	0	12.09127574	5.436365257
ANKRD55	0	3.04720192	0	0	0	0
RAET1E	49.8126529	91.4160575	0	0	4.030425245	2.718182629
PI16	2.93015605	11.1730737	0	0	0	0.906060876

TBX20	0	3.04720192	0	0	1.007606311	0
DAGLB	953.199298	880.641354	486.4231504	426.416891	780.7840546	684.0759615
KIAA0895	203.157486	205.178262	8.023474646	4.02280086	252.9091841	20.83940015
STK17A	1692.65348	1597.74954	484.4172817	556.152219	404.0501308	765.6214404
TRA2A	1593.02817	1570.32472	1362.987755	991.620413	1392.511922	1228.618548
FAM183BP	0	0	1.002934331	1.00570022	0	2.718182629
GALNT10	4992.00919	5506.29386	10461.608	8505.20672	2040.40278	6611.526213
SAP30L	1018.71759	1268.65173	1350.952543	1352.66679	591.4649047	890.6578413
RPS14	7889.93353	8440.74931	9711.413124	7267.18976	10430.74053	6239.135193
HCN1	0	0	0	0	0	0
MYOZ3	0	0	0	0	1.007606311	1.812121752
COG5	1055.8329	920.254979	615.8016791	472.679101	1279.660015	584.4092651
NEUROD6	0	0	0	0	0	0
C7orf60	281.294981	274.248173	208.6103408	264.499157	275.076523	274.5364455
GPR85	5.8603121	30.4720192	0	0	0	1.812121752
SLU7	1418.19553	1304.20242	1331.896791	1179.68635	1036.826894	1060.091225
RP9	253.409663	270.926722	278.6552744	211.197045	539.9661462	186.3767222
PTTG1	2686.9531	2632.78246	520.5229176	1215.89156	828.2523879	892.469963
CAMLG	901.511345	920.254979	973.8492351	1257.12527	639.8300077	780.1184144
FBXL21	0	0	0	0	0	0
BMPER	137.717334	128.998214	4.011737323	0	35.2662209	3.624243505
RELL2	288.132012	288.468448	535.5669326	740.195359	220.6657822	667.7668657
SMAD5-AS1	0	0	3.008802992	2.01140043	3.022818934	2.718182629
KCNK5	14.6507803	7.11013781	0	0	280.1145545	9.966669638
KIF6	2.93015605	11.1730737	1.002934331	2.01140043	14.10648836	0.906060876
ZNF12	459.057781	374.805836	1034.025295	821.657076	1613.177704	1110.830634
SLC29A4	84.9745255	93.4475254	22.06455528	10.0570022	403.0425245	136.8151923
C7orf62	0	0	0	0	0	0
STEAP1	175.789829	242.760419	0	0	57.43355974	32.61819154
CDCA7L	4581.78735	2569.80695	485.4202161	327.85827	521.9400692	585.315326
SP8	0	0	0	0	0	0.906060876
MIOS	665.145424	657.17988	883.5851454	762.320763	884.6783413	1016.600303
KIAA1324L	327.200759	255.964961	53.15551953	200.134343	453.4228401	177.5879317

USP49	1419.17225	1084.80388	270.7922693	233.32245	223.6886011	457.5607425
INTS4P1	0	0	21.18197306	21.2202745	0	17.05206569
SYTL3	38.0920287	66.0227082	30.08802992	39.2223084	28.21297672	36.24243505
IQUB	6.83703079	9.14160575	3.008802992	1.00570022	5.038031556	2.718182629
HEY1	13.6740616	29.4562852	7.020540315	10.0570022	45.34228401	20.83940015
ZNF704	556.72965	497.709646	3990.675702	5439.83247	924.9825938	4883.668123
FABP5	621.13448	629.694119	2.005868661	1.00570022	291.8330159	23.55758278
SHH	129.903585	160.485968	12.03521197	6.03420129	6671.361387	116.881853
TAGAP	0	0	0	0	0	0
COL1A2	10.7439055	7.11013781	1.002934331	0	0	8.154547886
FNDC1	0	0	0	0	0	0
CHMP4C	48.8359342	62.9755063	1230.600424	1103.25314	480.6282105	778.3062926
SLC13A4	0	0	0	0	0	0
PGAM2	0	3.04720192	9.026408976	1.00570022	1.007606311	1.812121752
BRI3	1966.13471	2312.82625	712.0833748	880.993389	653.936496	387.794055
LMTK2	1485.58912	1608.92261	533.5610639	709.018652	2423.293179	856.227528
SLC35G3	0	0	0	0	0	0
CTSB	5053.54247	4722.14724	2299.72842	2955.75293	3791.622549	2326.76433
SOX17	0	0	0	0	0	0
SEPT7P5	0	0	0	0	0	0
DLC1	3244.65947	2588.09016	0	0	1158.747258	85.16972236
ADCY1	0	0	0	0	3557.857885	163.0909577
C8orf48	29.3015605	18.2832115	0	0	34.25861458	3.624243505
SUN3	21.487811	27.4248173	0	0	0	0
C7orf57	3.90687474	6.09440383	0	0	1.007606311	0.906060876
HNF4G	15.6274989	14.2202756	0	0	3.022818934	7.248487009
PEX2	735.469169	690.699101	1459.269451	1580.96074	715.400481	1271.203409
RAD21	12359.3982	10800.2993	7699.526857	10283.2847	7735.393652	10032.81208
SLC30A8	0	0	1.002934331	0	0	0
MED30	451.244032	411.372259	289.8480216	204.157144	159.2017972	216.5485494
TNFRSF11B	252.970139	245.807621	0	0	25.19015778	7.248487009
SBSPON	0	0	0	0	1.007606311	0
PHKG1	7.81374947	9.14160575	51.14965087	66.3762142	11.08366942	38.0545568

EN2	131.857022	126.966747	0	0	251.9015778	11.77879139
KCNV1	0	0	0	0	4.030425245	0.906060876
CSMD3	3.90687474	4.06293589	0	0	66.50201655	6.342426133
SPIDR	1036.29852	907.050437	1049.06931	1280.25637	1767.34147	1223.182183
ORC5	354.548882	422.545332	311.9125769	259.470656	1146.655982	338.8667677
DEFA5	0	0	0	0	0	0
DNAAF5	1971.99502	1813.08514	1227.591621	1045.92822	3132.648022	1786.752048
DEFA4	0	0	0	0	0	0
DEFA6	0	0	0	0	0	0
OSGIN2	787.235259	943.61686	5238.326009	6528.0001	3023.82654	4850.14387
DEFB1	0	0	0	0	0	4.530304381
SUN1	5334.83745	4949.67165	3618.587065	3481.73415	3748.295478	3319.80705
OXR1	1549.73023	1341.85568	1681.920873	1927.92731	2499.871258	2116.703176
TMEM74	15.6274989	16.2517436	1.002934331	1.00570022	1.007606311	2.718182629
FAM86FP	12.7852476	10.1573397	13.0381463	24.3580592	4.201718318	6.342426133
GPR146	37.11531	40.6293589	58.17019118	88.501619	28.21297672	16.30909577
GPER1	32.2317166	30.4720192	0	1.00570022	590.4572984	37.14849592
UNCX	0	0	0	0	0	0
TMEM184A	95.718431	87.3531216	1717.023574	1789.14068	2009.166985	2306.830991
NOS3	17.5809363	10.1573397	41.12030756	180.020339	9.068456802	33.52425242
SPAG11B	0	0	0	0	0	0
MICALL2	407.291691	455.04882	1082.166143	1258.13097	1625.26898	1260.330679
CA3	4.88359342	5.07866986	0	1.00570022	1.007606311	0.906060876
INTS1	3028.80464	2853.19673	4319.638162	4566.88468	7354.518466	5916.577521
CDK5	360.409194	477.394967	602.7635328	879.987689	545.1150144	701.2911182
SLC4A2	5682.5493	5339.71349	7131.866026	7674.49834	7106.647314	7406.141602
SLC7A13	0	0	14.04108063	13.0741028	3.022818934	9.966669638
FASTK	1528.56474	1539.8527	1621.744813	1317.46728	2081.714639	1571.109559
TMUB1	1533.44833	1482.9716	1216.559343	1072.07643	1547.683294	1016.600303
C7orf55	202.171	170.054182	168.3926741	192.953643	215.8998043	113.6562763
GBX1	0	0	0	0	0	0
PHAX	1954.41409	2008.10606	1397.087523	1239.02267	1669.603658	1529.430759
ALDH7A1	2831.47816	2797.3212	3246.46834	3737.17194	1124.458415	3607.898167

FOXK1	3353.07524	2830.85058	1481.334006	1450.21971	3237.439078	2156.424885
COX6C	1905.57815	1948.17776	3024.849941	2809.9264	5232.499575	2936.5433
OSR2	101.578743	86.3373876	40.11737323	17.0969037	1335.078362	118.6939748
YWHAZ	34477.1147	37014.0468	20194.53407	24138.0925	59964.67687	24886.51133
BAALC	0	0	0	0	1.007606311	8.154547886
FZD6	1892.88081	1893.32812	1434.196093	1406.9746	2445.460517	1451.509524
CTHRC1	0	0	7.020540315	6.03420129	10.07606311	19.93333928
SLC25A32	1795.20894	1985.75992	695.0334912	671.807744	2202.627396	1155.227617
DCAF13	1661.39848	1850.6673	1495.375087	1382.8378	4346.813627	1852.894492
DCSTAMP	0	0	0	0	0	0
TP53INP1	277.388106	314.877531	2013.892136	5955.75668	1203.081936	2102.967294
INTS8	1035.3218	1628.22156	1520.448445	2223.60318	3002.666808	2266.058251
KIAA1429	3023.92105	2868.43274	2320.790041	2955.75293	4608.791268	3308.93432
FREM1	0	0	0	2.01140043	53.4031345	12.68485227
GEM	497.14981	553.575015	0	1.00570022	42.31946507	20.83940015
PDP1	2717.23138	3398.64587	507.4847713	1173.65215	608.594212	726.6608227
TMEM67	287.155293	302.688724	224.6572901	170.969037	292.2058303	243.7303757
KIAA0196	758.910417	786.178095	2325.804713	3008.04934	4894.95146	2633.012906
RPP25L	335.991227	390.041845	879.573408	821.657076	460.4760843	519.1728821
FAM219A	964.99806	849.153601	719.1039151	851.828083	895.7620107	719.4123357
C9orf24	0	1.01573397	0	0	0	0
SNAPC3	473.708562	441.844278	1094.201355	695.944549	885.6859476	638.7729177
KIAA1161	347.711851	517.008592	1627.762419	2744.55589	1527.531168	1824.806605
NUDT2	263.714045	280.342576	697.0393598	616.494232	361.7306658	351.55162
TMEM65	1121.27305	1134.57485	857.5088528	897.084592	1291.751291	821.7972147
PSIP1	2206.40751	1854.73023	1278.741272	1036.87692	1874.147739	884.3154151
CCDC171	35.1618726	45.7080288	59.17312551	97.5529209	77.58568597	67.95456571
UBAP1	1238.47929	1287.95068	2527.394513	2684.21388	1162.777683	2131.961242
DIRAS2	0	0	0	0	39.29664614	1.812121752
SYK	0.97671868	1.01573397	0	0	893.7467981	30.80606979
NIPSNAP3B	4.88359342	1.01573397	4.011737323	5.05867208	16.12170098	1.812121752
ABCA1	928.859468	26.4090833	4.011737323	0	531.008526	204.769758
NFIL3	593.84496	637.880935	297.8714962	267.516257	494.7346988	325.2758545

LETM2	267.620919	218.382804	32.09389858	70.3990151	59.44877237	62.51820046
METTL2B	1034.97995	1082.76226	879.5633787	796.595027	1376.279385	1010.30318
PRKACG	0	0	0	0	0	0
FXN	388.734036	385.978909	89.26115543	92.5244198	319.4112007	158.5606533
ZMAT4	0	0	93.27289276	57.3249123	47.35749663	57.98789608
NKX6-3	0	0	0	0	0	0
TMEM71	365.292788	385.978909	0	0	20.15212623	14.49697402
MAMDC2	599.705272	487.552307	0	0	17.12930729	12.68485227
PRSS37	0	0	0	0	0	0
CPA6	0	0	0	0	1.007606311	1.812121752
C8orf34	0	0	25.07335827	31.1767067	1.007606311	29.90000891
TMC1	0	0	16.04694929	16.0912034	1.007606311	8.154547886
ALDH1A1	0	0	0	0	0	1.812121752
KDM1B	989.416027	1236.14824	862.5235244	864.902185	1115.420187	1046.500312
HGSNAT	1563.72661	1629.23729	1022.521638	969.344153	481.5652844	865.768349
RASEF	11.7206242	8.12587178	1377.028836	1945.02422	687.1875043	1195.094296
GKAP1	182.646394	187.910785	138.4049376	159.906334	124.9431826	110.5394269
KIF27	321.770203	256.279839	159.4665586	118.672625	187.263633	89.70002674
C9orf64	830.210881	834.933325	692.0246882	532.015414	693.2331422	374.2031419
HNRNPK	17226.3777	16480.2735	13893.64928	12756.2915	19334.93736	12429.52431
SSMEM1	0	0	0	0	0	0
RP11-213G2.3	23.3631109	9.5885287	7.020540315	23.131105	10.22720406	15.4030349
SVEP1	11.7206242	2.03146794	0	0	0	0.906060876
TRPV6	0.97671868	0	3185.319434	1183.70915	5.038031556	3402.25859
C7orf34	0	0	3.008802992	0	0	0
ANKS6	1552.98271	1460.62545	154.4518869	89.5073192	1053.956202	147.6879228
FBP1	598.728553	497.709646	1828.349285	1468.32231	4682.346529	847.1669192
TMEM246	0	0	0	0	1.007606311	0
ZHX1	1758.48432	1857.68602	3025.592113	3158.85409	4168.608375	3715.501956
CFAP47	0	0	10.02934331	23.131105	1.007606311	15.4030349
CYBB	3.90687474	1.01573397	1.002934331	1.00570022	9.068456802	0.906060876
DYNLT3	655.378237	745.548736	582.7048461	424.405491	921.9597748	407.7273943
WBSCR27	38.0920287	35.550689	137.4020033	226.282548	18.1369136	123.2242792

MID1IP1	1348.8485	1133.55911	3375.876957	2874.29122	1509.394254	785.5547796
NCF1C	0	0	0	0	0	0.906060876
C9orf84	25.3946858	8.12587178	0	0	2.015212623	0.906060876
CXorf58	1.95343737	1.01573397	0	1.00570022	0	2.718182629
KIAA1958	709.097764	612.487585	536.5698669	668.790643	694.2407485	590.7516913
PTCHD1	0	0	163.4782959	235.33385	0	89.70002674
RNF183	0	0	1.002934331	1.00570022	1.007606311	0.906060876
ASB11	0	0	0	0	2.015212623	0
PCDH19	0	0	0	2.01140043	427.225076	16.30909577
PIGA	693.470266	583.0313	433.2676309	325.84687	490.7042736	355.1758635
FIGF	0	1.01573397	3.008802992	1.00570022	0	3.624243505
OR1Q1	0	0	2.005868661	0	0	0
OR1K1	0	0	0	0	0	0
STRBP	780.398228	767.894883	1042.239327	985.586211	2632.099435	1182.726565
CLDN3	143.577647	117.825141	2095.129817	3212.20649	2737.666348	2284.179469
GAPVD1	2778.76466	2521.05172	1737.082261	1467.31661	4069.721891	2335.824939
CARD19	795.049009	860.326674	245.718911	221.254047	824.2219626	302.6243326
WNK2	20.5110924	21.3304134	222.6514214	159.906334	2674.18715	549.9789518
ATP7A	497.14981	590.141438	837.4501661	782.434768	486.6436202	781.9305361
ZNF367	1243.36288	1005.57663	266.780532	569.226322	1402.587985	770.1517448
NLGN4Y	0	0	0	0	0	0
HDX	2.93015605	1.01573397	135.3961346	121.689726	2.015212623	112.3515486
NDUFB6	1193.55023	1238.17971	1972.771829	2086.82795	793.9937733	1439.730732
AQP7	0	0	0	0	1.007606311	3.624243505
NOL6	2400.77452	2365.64442	1503.398562	1490.44772	2539.167904	3192.958528
AQP3	76.1840573	58.9125704	164.4812302	285.618861	2358.806375	3330.679781
TRMT10B	180.692957	148.29716	321.9419202	237.345251	182.3767423	230.1394625
VCP	13424.9983	13465.5853	24414.43041	27541.1004	14936.75596	19586.31796
PIGO	1159.36508	1242.24265	1395.081654	2266.84829	1473.120427	1637.252003
STOML2	3234.89228	3094.94141	3920.470299	3122.69917	2960.347343	3457.528303
BRWD3	1078.29743	895.877364	1589.650914	1687.56496	405.0577371	1234.960974
SLITRK5	62.5099958	68.0541761	3.008802992	0	12.09127574	6.342426133
MELK	3113.77916	2833.89778	1088.183749	2200.47207	1595.040791	2356.664339

ARMC3	0	0	3.008802992	1.00570022	2.015212623	9.060608762
OTUD1	228.552172	232.60308	200.5868661	345.960874	295.2286492	127.7545835
ARHGAP12	1821.58035	1977.63404	985.8844471	1339.59269	1812.683754	1226.806426
FAT3	590.914804	330.113541	0	0	2.015212623	3.624243505
CCDC67	0	0	0	0	0	0.906060876
HECTD2	322.317166	338.239413	38.11150457	48.2736103	356.6926342	45.30304381
SLC7A3	0	0	0	0	0	0
FBXO33	604.588865	657.17988	521.525852	707.007252	488.689061	596.1880565
DDX26B	129.903585	184.863583	907.6555693	1063.02513	632.7767635	1111.736695
GPR101	0	0	0	0	0	0
CLDN2	26.3714045	30.4720192	3.008802992	4.02280086	0	1.812121752
LRFN5	0	0	0	0	237.7950895	6.342426133
LRRC18	0	0	0	0	0	0
SPTSSA	1007.97368	1066.52067	2070.056459	2030.50874	1505.363829	1936.252092
WRN	652.448081	610.456117	243.7130424	279.58466	481.6358168	377.8273854
MARCH8	1048.99587	974.088879	197.5780631	369.091979	193.4604118	184.8364187
TSHR	0	0	0	0	1.007606311	0
CFL2	2538.49186	2659.19154	0	1.00570022	704.3168116	59.80001783
SUGT1	1182.80633	1300.13948	1305.820499	1242.03977	951.1803579	1312.88221
GTF2A1	3473.21164	3226.98683	1085.174946	1929.93871	2090.783096	2302.300686
ZCCHC24	1463.12459	1645.48904	364.065162	419.37699	181.369136	432.1910379
PGM2L1	557.706368	534.276069	15.04401496	31.1767067	263.9928536	126.8485227
PHYHIPL	0	0	0	0	0	0
SLC16A9	4.88359342	8.12587178	27.07922693	10.0570022	196.4832307	21.74546103
FOLR2	0	0	0	0	0	0
INPPL1	4143.24066	3885.18244	7528.025086	7073.08962	7795.85003	8818.690508
PHOX2A	0	0	1.002934331	0	0	0
MBL2	0	0	0	0	0	0
GJB2	150.414677	141.187022	0	0	70.53244179	2.718182629
CRYL1	172.879207	190.957987	226.6631587	149.849332	230.7418453	163.0909577
REEP3	3006.34011	2812.56737	1910.5899	1640.29705	1636.35265	2229.815816
HEPACAM	2.93015605	1.01573397	0	0	0	0.906060876
SKA3	1638.93395	1593.6866	316.9272485	760.309363	956.2183894	1080.930625

MICU2	1684.83973	1308.26536	1049.06931	1064.03083	1100.306092	939.5851286
DDIAS	770.631042	850.169335	193.5663258	229.299649	1249.431826	794.4069944
PCF11	1243.36288	1318.4227	1748.114538	2349.19502	2374.928076	2182.700651
PKNOX2	0	1.01573397	4.011737323	11.0627024	1.007606311	9.060608762
RPL10L	0	0	0	0	0	0
LRR1	1289.26866	1238.20003	576.6872402	681.945202	813.1584453	953.1760417
RPL36AL	3637.30038	3986.75584	3446.995031	3122.69917	2296.334783	2507.070444
DNAAF2	595.798397	670.384422	554.6226849	785.451868	528.9933134	838.1063105
C10orf10	98.6485871	67.0384422	50.14671654	25.1425054	21.15973254	129.5667053
MAGEC3	0	0	1.002934331	4.02280086	0	2.718182629
C10orf25	19.5343737	60.9440383	18.05281795	8.04560172	9.068456802	28.99394804
ZNF22	2047.20236	1700.33867	1267.708994	713.041453	448.3848085	798.2396319
KLHDC2	1308.80304	1254.43146	1806.28473	1845.4599	830.2676005	1533.055002
EML5	244.179671	185.879317	61.17899417	140.79803	401.0273119	124.13034
NEMF	2139.01392	2352.43988	2616.655669	2002.34913	1502.34101	2131.961242
RPUSD4	1175.9693	1090.89829	389.1385203	570.232022	463.4989032	604.3426044
ARF6	7309.76263	7197.49093	3321.718503	4027.82936	4277.288791	3888.813281
TTC8	652.448081	517.008592	936.7406649	1107.27594	905.8380738	886.1275369
TMEM63C	0	0	3382.566529	5538.58217	135.0192457	4891.82267
NGB	0	0	0	0	0	0
NOXRED1	1.95343737	6.09440383	15.04401496	37.210908	16.12170098	24.46364366
CDX2	0	0	0	0	0	0
AMER2	0	0	0	0	17.12930729	1.812121752
AKR1E2	23.4412484	29.4562852	0	0	137.0344583	13.59091314
KBTBD6	561.056514	519.415881	586.7867889	597.365814	645.4121466	805.9320888
SSX5	0	0	0	0	0	0
SSX3	0	0	0	0	0	0
OTX2	0	0	0	0	0	0
FAAH2	0	0	414.2118786	291.653063	40.30425245	180.3061144
DRGX	14.6507803	6.09440383	0	0	0	0
NUDT5	2503.32999	2334.15667	1951.710208	1267.18227	2049.471237	1579.264107
DACT1	0	3.04720192	0	0	0	0.906060876
OXGR1	0	0	1.002934331	4.02280086	0	9.060608762

UCMA	0	0	0	0	0	0
BEND7	1559.81974	1390.53981	0	0	612.6246373	69.76668747
ATP5C1	3954.72418	4041.60548	3790.088836	3693.92683	5088.411872	3884.282976
PRPF18	590.914804	504.819784	468.3703324	431.445392	731.522182	442.7375865
TAF3	822.397132	884.70429	486.4231504	580.289024	1473.120427	659.6123179
VSTM4	0	0	0	0	2.015212623	0
VDAC2	4493.40407	4431.57622	3369.398001	3479.05898	3838.859133	3778.799369
SOHLH1	0	0	0	0	2.015212623	0
COMTD1	276.411388	283.389778	1332.899726	903.118794	444.3543833	553.6031953
SLC18A2	1.95343737	0	2.005868661	1.00570022	1.007606311	2.718182629
PDZD8	3846.31818	3640.39056	1090.189617	1523.63583	1328.025118	1162.476104
ZNF503	47.8592155	61.9597723	771.2565003	643.648138	369.7915162	841.730554
FAM175B	777.468072	771.957819	665.9483956	654.71084	597.5105426	623.3698828
QSOX2	3188.00978	2941.56558	298.8744306	246.396553	1464.05197	609.7789697
FAM204A	889.790721	875.562684	986.8873814	852.833783	653.936496	812.7366059
NSD1	4040.6852	4088.32924	4584.412826	5422.73556	8037.675545	6643.238344
PRDX3	6092.77115	5674.9057	2029.939085	2165.27256	2922.058303	2151.894581
ENOX2	293.992324	346.365285	308.9037739	323.835469	982.4161535	270.0061411
GHITM	5503.80978	5607.86726	8800.748752	5209.52712	6697.559151	6145.810923
CLEC1B	0	0	0	0	0	0
SNAPC4	593.84496	544.433409	548.6050789	530.004014	1037.834501	857.1335889
TMEM52B	0	0	0	0	0	0
PMPCA	1106.62227	931.428053	1068.125062	785.451868	2313.464091	1919.036936
SDCCAG3	1885.06706	1927.86308	556.6285535	694.938849	1903.368322	1470.536802
FRMD7	0	1.01573397	0	0	1.007606311	1.812121752
AK8	3.90687474	1.01573397	21.06162094	2.01140043	13.09888205	8.154547886
C9orf9	70.3237452	69.0699101	67.19660016	74.421816	85.64653646	57.98789608
TSC1	1525.63458	1534.77403	1498.38389	1449.21401	1165.800502	1282.07614
GFI1B	0	0	0	0	0	0
HPRT1	1640.88739	1499.22334	906.652635	1086.15623	1969.870339	1344.59434
BORCS5	179.716238	154.391564	422.2353532	347.972275	230.7418453	372.3910201
FAM69B	79.1142134	47.7394967	11.03227764	13.0741028	35.2662209	15.4030349
ZMYND19	1025.55462	973.073145	1965.751288	1924.91021	1222.226456	2216.224903

STOX1	41.9989034	53.8339005	18.05281795	75.4275162	134.0116394	34.43031329
RET	0.97671868	1.01573397	467.3673981	660.745042	3853.086534	908.7790588
DDX21	10862.0885	9794.72269	3414.991396	2392.56081	8359.101958	5331.262195
BMS1	4194.39141	4204.91518	1537.377977	1568.45989	3634.808779	2611.983233
STK32C	525.474652	587.094236	303.8891022	271.539058	431.2555012	318.9334284
KIAA1462	542.07887	311.830329	0	0	124.9431826	17.21515665
OR4K2	0	0	0	0	0	0
FUNDC2	882.95369	901.971767	3133.909021	4335.51329	1848.70568	3095.375771
TMEM55B	766.724167	964.947274	866.5352617	2801.8808	925.9902001	737.5335532
METTL17	1531.4949	1290.99788	1007.949002	910.158695	875.6098845	935.0548242
SLC39A2	0	0	1.002934331	0	0	0.906060876
NDRG2	38.0920287	43.6765608	30.08802992	23.131105	222.6809948	48.02122644
RNASE7	0	3.04720192	0	0	0	0
ARHGEF40	1479.72881	1716.59041	122.3579883	135.769529	215.6277506	166.7152012
NSMF	2480.86546	2430.6514	2861.371646	2421.72612	2730.613104	4333.689171
ZNF219	1094.90164	986.277687	459.3439235	409.319988	1165.800502	745.6881011
C12orf50	1.95343737	0	1.002934331	1.00570022	3.022818934	0.906060876
CASP7	688.586672	825.791719	632.8515627	995.643213	780.8948912	760.1850751
PPP1R36	6.83703079	4.06293589	1.002934331	5.02850108	7.053244179	1.812121752
BTNL9	0.97671868	3.04720192	142.416675	193.094441	46.34989032	141.3454967
CCDC186	739.376044	668.352954	1115.262976	1662.42246	959.2412083	1315.600392
VWA2	2.93015605	0	2.005868661	2.01140043	1.007606311	0.906060876
METTL3	1292.19882	1323.50137	1139.3334	1014.75152	1236.332944	1060.091225
SALL2	228.552172	196.036657	209.6132751	215.219846	385.9132172	503.7698472
PRAP1	0	0	7.481890107	6.00403029	1.007606311	5.11924395
TRUB1	706.167608	647.02254	789.3093183	682.870446	1026.750831	958.612407
ERICH6B	0	0	1.002934331	0	0	2.718182629
CYP2C19	0	0	0	0	0	0.906060876
ZFYVE1	773.561198	887.751492	961.8140231	1370.76939	364.7534847	891.5639022
C10orf82	0	1.01573397	1.002934331	1.00570022	26.19776409	3.624243505
HSPA12A	494.219654	548.496345	130.381463	63.3591136	323.4416259	133.1909488
FAM35BP	15.5591286	23.5447135	13.64993624	15.0352182	18.1369136	16.1369442
FRAT1	84.9745255	68.0541761	302.8861679	299.849519	101.7682374	271.8182629

UBTD1	1089.04133	1201.61329	301.8832335	489.776005	356.6926342	423.1304292
ANKRD2	12.6973429	5.07866986	0	0	36.27382721	3.624243505
E2F7	2620.53623	2978.13201	138.4049376	279.58466	1478.158459	393.2304203
ARHGAP42	768.677604	814.618646	60.17605984	98.5586211	154.1637656	67.04850484
ISCA2	766.724167	824.775985	813.3797422	1146.49825	501.787943	664.1426222
OTOGL	0.97671868	1.01573397	3.008802992	6.03420129	5.038031556	3.624243505
GYLTL1B	43.9523408	22.3461474	51.14965087	58.3306125	272.053704	114.1636704
PACSIN3	1266.80413	1136.60631	1062.107456	948.375303	2139.148199	1203.248844
TTC7B	1482.65896	1429.1377	66.19366583	55.3135119	711.3700558	188.4606622
SLC39A13	2084.31767	2386.97483	916.6819783	883.004789	416.1414066	557.2274388
PSMC3	5130.70325	5217.82542	2405.036525	2225.61458	7089.518006	2958.288761
RAPSN	10.7439055	7.11013781	0	1.00570022	9.068456802	10.87273051
AGBL2	12.6973429	18.2832115	5.014671654	17.0969037	22.16733885	41.6788003
TC2N	48.8359342	40.6293589	15163.32403	9323.74613	1632.322224	8254.096794
CPSF2	4235.05221	4015.19639	2535.417988	2676.16827	3111.488289	3911.464802
SMCO2	13.6740616	8.12587178	0	0	2.015212623	0.906060876
MOAP1	867.326191	829.854655	1879.498936	1423.06581	957.2259957	1865.579344
IFI27L1	267.620919	342.302349	179.5252452	228.293949	70.53244179	97.85457463
IFI27	435.616533	450.985884	22.06455528	17.0969037	43.32707139	319.8394893
SERPINA12	0	0	0	0	0	0
CLMN	1472.89178	1234.11678	2045.986035	1078.11063	1052.948595	2320.421904
PDZRN4	0	0	0	0	0	0
SLC6A5	0	0	0	0	0	0
CCDC38	1.95343737	0	2.005868661	0	1.007606311	1.812121752
NELL1	0	0	0	0	0	0
PTER	645.61105	527.165932	790.3122526	459.604999	487.6814547	508.3001515
C1QL3	0	0	0	0	2.015212623	0
CACNB2	0.97671868	0	0	0	2.015212623	4.530304381
HACD1	827.280725	916.192043	53.15551953	35.1995075	404.0501308	57.98789608
ARL5B	1597.91177	1571.34046	946.7700082	814.617175	1743.158919	984.8881724
SMCO4	165.065458	259.012163	142.416675	101.575722	34.25861458	86.07578324
CEP295	1282.43163	1207.70769	1849.410906	1864.5682	768.8036155	2308.643113
KCNC2	0	0	0	0	0	0

TRIM51HP	0	0	0	0	0	0
TAF1D	3967.06991	3518.50248	2797.183848	2255.78558	1363.240959	3835.355689
TRIM53BP	0	0	0	0	0	0
ABTB2	862.442598	1052.3004	121.355054	175.997538	760.742765	255.5091671
R3HCC1L	436.593252	398.167717	251.736517	330.875371	504.810762	374.2031419
AMOTL1	4366.90924	4244.75227	1945.692602	1196.78326	1189.983054	1955.279371
HTRA1	477.615436	417.466663	2.005868661	0	439.3163517	27.18182629
LIPC	0	0	0	0	0	0
CEP57	2277.70797	2146.24588	2995.764846	2609.79206	714.3928747	2113.840024
TCP11L2	146.507803	107.667801	108.3169077	157.894934	109.8290879	115.9757922
PASD1	0	0	0	0	0	0
SPRED1	2055.01611	2029.43648	1655.84458	1478.37932	425.2098634	1964.33998
TMCO5A	0	0	0	0	0	0.906060876
GPR176	1167.17883	1047.22173	106.3110391	115.655525	176.3311045	251.8849236
JAM3	1.95343737	2.03146794	5.014671654	0	0	0.906060876
IL25	0	0	0	0	0	0
CMTM5	0	0	0	0	0	0
RP11-299H22.3	0	0	0	0	1.007606311	1.812121752
GLB1L3	0	0	0	0	0	0
ADAMTS15	1736.60582	1070.58361	34.09976724	44.2508095	28.21297672	50.73940907
SVOP	0	0	0	0	2.015212623	0.906060876
SPATA19	0	0	0	0	0	0
GPT2	965.974778	810.55571	4190.259634	842.776781	2115.973254	2083.033954
AMN	58.603121	19.2989455	14.04108063	7.03990151	10.07606311	19.93333928
RAB8B	1928.04268	1706.43307	468.3703324	421.38839	708.3472368	728.4729444
IKBIP	2977.03855	2874.52714	79.23181213	134.763829	532.0161324	229.2334017
RPUSD2	317.433572	340.270881	387.1326517	384.177482	301.2742871	480.2122644
HIF1AN	2182.96626	2554.57094	2346.866334	2407.64632	2094.813521	3009.934231
NDUFB8	1578.51413	1705.87442	2010.572423	1907.95411	1025.662616	1779.277046
ZFYVE19	790.165415	709.998047	2818.245469	2996.98664	593.4801173	2300.488565
PPP1R14D	0	0	13.0381463	5.02850108	1.007606311	3.624243505
SPINT1	248.086546	209.241198	7698.523923	9774.40039	3882.307117	8144.581216
FBN1	1153.50477	1353.97338	71.20833748	130.741028	226.71142	183.9303579

AVPR1A	0	0	1.002934331	0	0	0.906060876
C16orf78	0	0	0	0	0	0
DEPDC4	28.3248418	31.4877531	81.23768079	25.1425054	49.37270925	39.86667855
LRTM2	0	0	0	0	5.038031556	0
OPN1MW2	0	3.04720192	0	0	0	0
BRD7	1946.42453	2124.78342	2886.274505	2798.73296	2111.892448	2945.467999
CKB	1027.50806	804.461306	12694.13982	12690.931	385.9132172	16993.17173
TRMT61A	873.186503	908.066171	408.1942726	283.607461	1570.858239	756.5608316
BTRC	978.672121	1110.19723	596.7459268	628.562635	776.864466	614.309274
POLL	458.081063	421.529598	686.0070822	712.035753	423.1946507	704.0093008
BAG5	1845.99831	1779.56592	1989.821712	1963.12682	3489.340656	2781.60689
DPCD	86.9279629	88.3688556	206.6044721	152.866433	298.2514681	210.2061233
LARP6	858.535723	815.63438	6.017605984	0	159.2017972	48.92728731
API5	3883.41395	3794.77196	3068.969023	2672.12536	3837.942212	3622.395141
ASPG	0	0	1.002934331	3.01710065	1.007606311	0.906060876
ZNF319	360.409194	429.65547	504.4759683	455.582198	323.4416259	465.7152904
HPS6	872.209785	969.01021	524.534655	630.574035	871.5794593	617.9335176
SENP8	133.81046	107.667801	38.11150457	36.2052078	134.0116394	35.33637417
NOLC1	6992.32906	6578.90894	2445.153898	1941.00142	6654.23208	6303.465516
ALKBH3	578.217461	478.410701	581.7019118	471.673401	224.6962074	477.4940817
COPS2	1871.393	1802.9278	2346.866334	2248.74568	1786.48599	2318.609782
GABRB3	0	0	0	0	2.015212623	4.530304381
SPIC	0	0	0	0	0	0
TBATA	0	0	14.04108063	11.0627024	0	5.436365257
SGPL1	2480.86546	1979.66551	2043.980166	2410.66342	2223.787129	2133.773363
FRS2	910.301813	852.200803	988.8932501	1015.75722	1422.740112	1307.445844
CCT2	6404.34441	6404.20269	4417.925727	4250.08911	9502.735122	6171.180628
PCBD1	908.348376	1009.63957	1295.791155	1470.33372	1221.218849	1485.939837
ARIH1	2368.54281	2328.06226	1217.562277	979.55201	2700.384914	1401.676175
C16orf71	2.93015605	8.12587178	43.12617622	61.3477131	29.22058303	54.36365257
CLMP	517.660902	610.456117	0	1.00570022	60.45637868	26.27576541
SCN3B	0	0	0	0	0	0
COX11	1570.56364	1673.92959	1201.515328	1378.815	1766.333864	1678.024743

ZNF202	656.354956	668.352954	378.1062427	273.550459	262.9852472	368.7667766
FAM227B	7.81374947	6.09440383	101.2963674	91.5187196	39.29664614	57.0818352
STXBP4	328.177478	353.475422	547.6021446	826.685577	471.5597537	682.2638398
CYYR1	0	0	0	0	0	0
CUL5	2337.28781	2168.59203	1412.131538	1509.55602	1165.800502	1504.967115
MYRFL	0	0	122.3579883	137.78093	0	128.6606444
WBP1L	993.322901	1181.29861	1548.530607	1603.08614	1529.546381	1628.191394
BORCS7	293.992324	313.160941	297.8714962	359.034977	321.4264133	198.3820288
C2	72.5897326	46.7237627	27.07922693	18.1026039	19.14451991	33.52425242
PLEKHF1	154.321552	234.634548	999.9255277	924.238498	69.52483548	369.6728375
TMEM100	0	0	0	0	2.015212623	0
ANAPC16	1633.07364	1744.01523	2345.8634	2041.57144	1852.988006	1495.906507
SMPD1	911.278532	1249.35279	721.1097838	1102.24744	317.3959881	845.3547975
APBB1	544.032307	476.379233	26.0762926	31.1767067	24.18255147	48.92728731
SYNPO2L	1.95343737	2.03146794	0	1.00570022	7.053244179	0
NUDT13	68.3703079	52.8181666	42.12324189	55.3135119	70.53244179	44.39698293
C11orf65	14.6507803	5.07866986	5.014671654	8.04560172	11.08366942	8.154547886
TRIM44	6442.43644	6398.10829	3514.281895	3126.72197	3059.092761	3964.922394
CCDC182	0	0	0	0	0	0
ILK	5145.35403	4652.06159	1829.352219	2173.31817	1224.241668	1928.097545
TAF10	1367.40616	1286.8029	1154.377415	1151.52675	764.7731903	1338.251914
TPP1	2114.59595	3243.23857	1835.369825	3889.04273	694.2407485	1215.027635
DCHS1	30.2782792	29.4562852	1.002934331	11.0627024	6.045637868	17.21515665
NETO1	0	0	0	0	0	0
MSS51	71.3004639	35.550689	75.2200748	44.2508095	26.19776409	81.54547886
CYB5A	674.912611	518.024326	1407.116866	2049.61704	993.4998229	1891.855109
USP54	699.330578	881.657088	1052.078113	1218.90866	656.959315	888.8457195
RAG1	0	0	0	3.01710065	1.007606311	0.906060876
POTED	0	0	0	0	0	0
C11orf74	61.5332771	69.0699101	128.3755943	85.4845183	61.46398499	98.7606355
WDR88	0	1.01573397	0	0	0	0
OR10A5	0	0	0	0	0	0
OR2D2	1.95343737	0	0	0	0	0

ATP9B	570.403711	498.72538	578.6931088	481.730403	355.6850279	599.8123
PPFIBP2	50.7893716	38.5978909	2491.288877	1881.6651	77.58568597	1313.78827
MOGAT2	0	0	5.014671654	0	0	13.59091314
CYB5R2	7.81374947	16.2517436	0	0	0	0
SERPINB7	9.76718684	9.14160575	0	0	2.015212623	0.906060876
KIAA0355	1192.57351	901.971767	367.073965	337.915272	359.7154531	575.3486564
SERPINB8	548.437308	801.007811	20.05868661	67.9249926	75.77199461	81.54547886
TUB	153.344833	99.5419293	0	0	46.34989032	12.68485227
RIC3	0	0	0	0	1.007606311	0.906060876
LMO1	0	1.01573397	0	0	8.06085049	0
OR5P1P	0	0	0	0	0	0
IDH3A	1991.5294	2064.98717	726.1244554	774.389166	2332.608611	932.3366416
WDR72	55.672965	43.6765608	0	0	4.030425245	0.906060876
CRABP1	0	0	0	0	133.0040331	1.812121752
PLD4	0	2.03146794	5.014671654	0	5.038031556	2.718182629
ZMAT1	83.9978068	45.7080288	1631.774156	947.369603	177.3387108	575.3486564
XRRA1	330.130915	355.50689	522.5287863	357.023577	246.8635463	301.7182718
TRIM66	280.318262	232.60308	149.4372153	218.236947	570.3051722	287.2212977
RNF169	1673.11911	1495.16041	1365.996558	1664.43386	1674.641689	1905.446023
RPL27A	14310.4133	14760.7578	6503.437403	5649.97353	10996.99513	5349.999534
ST5	989.416027	961.900072	12.03521197	32.1824069	98.74541851	54.36365257
CDYL2	554.776212	617.566255	747.1860764	2094.87355	1648.443925	1021.130607
TMEM130	6.83703079	2.03146794	0	0	6.045637868	0
PRTG	817.513538	607.408915	0	0	430.2478949	36.24243505
CENPN	1117.36617	1260.52586	253.7423857	566.209221	2522.038597	761.091136
AKIP1	639.750738	605.377447	230.6748961	201.140043	408.0805561	238.2940104
ATMIN	1229.68882	1324.5171	1011.96074	1534.69853	5254.656837	2022.327876
C16orf46	25.4728233	35.550689	27.7913103	15.5481253	55.76093327	29.09361473
TMEM41B	1756.14019	1938.02042	523.5317206	735.166858	769.8112218	473.8698382
PKD1L2	14.4163678	28.1967751	4.011737323	14.5122541	123.9355763	88.79396587
LEO1	749.143231	798.366902	3434.047148	2895.41092	1954.756244	3118.661536
ZNF143	624.123239	606.393181	439.2852369	364.063478	408.0805561	437.6274032
TMX3	2046.22564	1765.34564	1656.847514	1163.59515	705.3244179	1318.318575

MFAP4	0	0	0	1.00570022	0	0
WEE1	1839.16128	1544.93137	557.6314879	922.227098	837.3208447	1019.354728
MAPK7	802.862758	805.47704	314.9213798	460.610699	433.2707139	440.3455858
FAM86GP	1.95343737	0	0	2.01140043	1.289736078	3.66048594
PRKCB	0	0	0	0	2.015212623	0
RP11-382A20.3	1991.5294	1905.51693	684.0012135	1028.83132	2903.921389	839.9184322
NDST2	566.408932	576.794693	500.6949059	535.454909	470.5722995	601.9053007
MCM7	7858.67853	6897.84941	2953.641604	3948.37905	11014.14459	7345.435523
CLEC3A	0	0	0	0	296.2362555	200.2394536
CCDC68	191.436862	213.304134	0	0	133.0040331	9.060608762
CLEC4E	0	0	0	0	0	0
ZNF3	528.404808	402.230653	973.8492351	733.155457	1255.477464	1248.551887
CLEC4D	0	0	0	0	0	0
ZSCAN21	187.529987	196.036657	323.9477888	251.425054	166.2550414	264.5697758
HSBP1P2	0	0	0	0	0	0
RIMKLB	931.789624	864.38961	8.023474646	4.02280086	735.5526072	99.66669638
A2ML1	0	0	1.002934331	0	11.08366942	0.906060876
BEAN1	14.6507803	12.1888077	0	0	10.07606311	1.812121752
TK2	782.351666	860.326674	581.2505914	288.635962	76.57807966	526.1948538
TMED3	1844.04488	2155.38749	3387.912169	3964.47025	3589.093681	2880.367525
SLC38A8	0	0	0	0	1.007606311	0
SEC11C	386.780599	588.10997	1245.644439	822.662776	689.2027169	628.8062481
CPLX4	0	1.01573397	0	0	0	0.906060876
GALR1	0	0	0	0	0	0
TMEM135	542.07887	374.805836	3270.568852	3484.75125	336.540508	1935.346032
IQCD	31.2549979	36.566423	69.20246882	18.1026039	100.7606311	52.55153082
NDEL1	1324.43054	1479.9244	485.4202161	487.764605	947.1499326	535.4819778
CENPV	733.515732	504.819784	46.13497921	34.1938073	243.8407273	57.98789608
CDH16	0	0	0	0	5.038031556	0
RRAD	370.176381	479.426435	9.026408976	1.00570022	11.08366942	9.966669638
FAM96B	861.465879	927.365117	958.8052202	811.600074	1034.811682	852.6032845
CFAP52	0	0	0	0	1.007606311	0
HSP90B1	6691.76819	24019.0612	6877.081028	23681.5457	13718.55993	13578.22829

MC4R	0.97671868	1.01573397	0	0	0	0
BLCAP	1697.53707	1701.3544	1738.085195	2051.62844	1130.534281	2323.140087
SERPINB12	0	0	0	0	0	0
CHRFAM7A	211.92842	139.978299	0	0	0.231749452	6.342426133
ATF7IP2	0	0	296.8685619	316.795568	190.4375928	279.0667499
MMP10	0	0	0	0	9.068456802	0.906060876
TVP23A	2.93015605	3.04720192	2.005868661	2.01140043	4.030425245	2.718182629
NGFRAP1	1801.06925	1720.65335	325.9536575	194.100142	1866.086889	307.154637
TMPRSS5	14.6507803	8.12587178	1.002934331	7.03990151	0	5.436365257
COG1	1290.24538	1083.78815	309.9067082	254.442155	1491.257341	431.2849771
PLEKHA7	127.950148	77.1957819	624.828088	684.881847	1143.633163	791.8972058
OR5M13P	0	0	0	0	0	0
ZNF606	207.064361	191.973721	525.5375893	355.012176	270.0384914	359.7061678
ZCCHC18	20.5110924	24.3776153	0	1.00570022	9.068456802	2.718182629
B2M	11236.1717	11689.0666	7460.828486	8120.02354	3056.069942	10854.6093
ZNF592	2406.63484	2231.56754	798.3357272	982.569111	2835.242943	1160.663982
CASC4	2611.74576	2579.96429	4544.295452	4705.67131	1759.28062	4231.304292
HTR3A	0	0	0	0	0	0
NNMT	1126.0199	590.141438	0	0	3.083275313	8.154547886
ACSM1	0	0	15.04401496	34.1938073	0	10.87273051
AP1G1	2532.63155	2633.79819	2916.533034	3235.33759	7203.377519	4086.334552
AGBL1	0	0	11.03227764	22.1254047	0	5.436365257
SLFN5	358.455757	369.727166	896.6232917	1055.98523	822.20675	1319.224636
CATSPER2	86.5275082	77.1653099	391.525504	319.631643	64.4666518	296.4903005
STRCP1	0	0	6.288398254	5.1391281	0	2.645697758
ZNF667-AS1	0	0	0	0	0	0
C16orf45	174.832644	243.776153	2.005868661	8.04560172	201.5212623	82.45153973
KIAA0430	1373.26647	1269.66747	2414.062934	2385.52091	1359.260914	2095.718807
SAA3P	0	0	0	0	0	0
SAAL1	1216.99148	1122.38604	566.6578969	605.43153	439.3163517	712.1638487
YPEL4	0.97671868	4.06293589	2.005868661	0	1.007606311	0
PPIB	6237.32552	6595.16068	18139.07031	10230.9883	7361.57171	10401.57886
LDHC	0.97671868	0	176.5164422	96.5472207	0	125.0364009

FAM96A	671.982454	773.989287	1549.533541	1771.03808	1106.35173	1473.254985
LDHAL6A	0	0	3.008802992	0	0	0
FAM111A	2804.15934	2770.92228	4851.193358	5093.87159	1005.591099	3760.152636
KIAA0101	1277.86059	1221.87718	661.4452204	1497.52785	1828.553553	1662.621708
KIF7	629.983551	681.557495	0	0	67.50962286	19.0272784
LDHD	21.487811	20.3146794	198.5809975	467.6506	115.8747258	211.1121841
PLIN1	7.81374947	11.1730737	3.008802992	4.02280086	8.06085049	5.436365257
PEX11A	120.136398	143.21849	53.15551953	45.2565097	570.3051722	104.1970008
TMEM170A	743.654072	814.019363	460.3468578	514.91851	1347.169638	471.1516556
MESP1	180.692957	204.162528	0	1.00570022	71.5400481	12.68485227
ANPEP	0.97671868	4.06293589	0	1.00570022	8.06085049	3.624243505
SCNN1G	0	0	0	0	48.36510294	2.718182629
RBPMS2	209.017798	231.587346	0	0	125.9507889	21.74546103
NAV2	4783.96811	4706.91123	64.18779717	360.040677	367.7763036	435.8152814
ANKDD1A	46.8824968	41.6450929	2.005868661	1.00570022	11.08366942	9.966669638
GLYATL1	0	0	8.023474646	2.01140043	0	1.812121752
C18orf54	696.400422	699.840707	405.1854696	458.599298	316.3883817	552.6971345
DCTN5	2567.16832	2678.49048	2736.536409	2503.60017	5151.004376	3706.695044
TERF2IP	817.513538	907.050437	1317.855711	1074.08783	3358.351836	1428.858002
PLK1	3480.04867	3371.22105	1062.107456	3144.82457	3734.18899	3269.973702
CLPX	1146.66773	1132.54338	2102.150357	1781.09508	1807.645722	2313.173417
GPR182	0	0	0	0	0	0
ZBTB39	314.503416	319.956201	467.3673981	458.599298	551.1606523	593.4698739
CACNG2	0	1.01573397	0	0	0	0
TAC3	0	0	4.011737323	0	0	2.718182629
MYO1A	0	1.01573397	2.005868661	12.0684026	0	12.68485227
CHP2	0	0	0	0	0	0
NEMP1	1754.18676	1974.58684	1642.806434	1975.19522	1620.230949	2409.21587
OR4D6	0	0	0	0	0	0
NAB2	449.290595	508.88272	703.0569658	839.75968	590.4572984	909.6851197
VPS39	951.323998	974.088879	2540.43266	2045.59424	951.1803579	2362.100704
STAT6	2829.55403	3229.0183	4129.08064	4130.41079	240.8179084	3005.403926
PATL1	4014.31379	3931.89605	1916.607506	2287.96799	1393.519529	1675.30656

XRCC6BP1	109.392493	137.124086	212.6220781	358.029277	239.8103021	190.272784
ELFN2	588.961366	441.844278	7.020540315	5.02850108	175.3234982	31.71213067
STX3	1642.84083	2545.16524	2527.394513	3251.4288	1006.598705	1593.761081
MRPL16	1090.01805	915.176309	804.3533332	555.146519	871.5794593	884.3154151
PIP4K2C	632.913707	736.40713	1948.701405	2804.8979	2636.905717	2440.928
MTMR10	684.679797	685.620431	709.0745718	985.586211	389.9436425	817.2669103
YWHAB	15014.1196	15588.4693	12273.91034	9660.75627	10423.68729	9953.078725
MIR202HG	0	0	0	0	0	0
C15orf48	1960.99717	1689.41953	0	0	181.369136	43.49092206
SCG5	97.6718684	287.452714	8.023474646	16.0912034	4.030425245	16.30909577
GREM1	0	0	0	0	0	0
NYAP1	37.11531	14.2202756	0	0	8.06085049	0.906060876
TSC22D4	1569.85064	1411.87022	892.6115543	1118.35875	1588.995153	1114.454878
MS4A6E	0	0	0	0	0	0
MS4A7	0	0	100.2934331	111.632724	0	33.52425242
MS4A14	0	0	35.10270157	39.2223084	0	22.6515219
MS4A5	0	0	0	0	0	0
DIS3L	1060.71649	959.868604	1039.039967	1372.78079	933.0434443	1264.860983
CCNDBP1	975.741965	896.893097	462.3527265	612.471431	361.7306658	414.0698204
EPB42	0	0	0	2.01140043	0	0
TGM6	0	0	0	0	0	0
SMAD3	6318.39317	5059.37092	934.7347962	2249.75138	3547.781822	1816.652057
MS4A8	0	0	0	0	0	0
CCDC178	0	0	0	2.01140043	0	0
MS4A15	0	0	0	0	0	0.906060876
MAP1A	20.5110924	89.3845896	24.07042394	73.4161157	98.74541851	128.6606444
RCCD1	1253.13007	1210.75489	500.464231	698.96165	590.4572984	719.4123357
AKTIP	381.770032	357.538358	1480.932833	1156.17308	1162.394793	1264.208619
MAPRE2	731.562294	1058.3948	37.10857024	89.5073192	177.3387108	86.98184411
EVA1C	167.995614	144.234224	2.005868661	1.00570022	23.17494516	21.74546103
TCP10L2	0	0	0	0	0	0
MARS	3984.03551	3369.18959	6922.252751	2517.26764	6296.531839	4604.601373
MBD6	887.837284	870.484014	3356.821205	2617.83766	1938.634543	2294.146138

CNPY4	206.087642	288.468448	354.0358187	400.268686	179.3539234	214.7364277
PDIA3	10377.1281	10557.8639	24043.07392	15764.4917	5456.349393	14885.36607
NUDT21	4585.69422	4127.94286	3668.733782	3196.11529	4990.67406	4322.81644
NAT16	4.88359342	3.04720192	0	0	0	1.812121752
C15orf43	0	0	0	0	0	0
NKX3-1	512.777309	508.88272	13.0381463	9.05130194	73.55526072	60.7060787
SGSM1	0	1.01573397	12.03521197	2.01140043	50.38031556	14.49697402
RP11-93B14.6	33.2084353	17.2674775	2.005868661	7.03990151	1.007606311	1.812121752
DUSP18	109.392493	130.013948	103.3022361	81.7734845	191.4653513	93.32427025
TEF	156.274989	160.485968	1493.369218	1838.41999	674.0886223	1127.13973
MEI1	8.79046815	16.2517436	0	0	3.022818934	2.718182629
B4GALNT2	4.88359342	1.01573397	484.4172817	697.95595	3.022818934	132.2848879
PBX3	432.686377	572.87396	202.5927348	314.784167	952.1879642	322.5576719
GNGT2	2.93015605	1.01573397	0	0	0	0
PHB	5261.58355	5583.48965	5833.066067	5064.70629	8302.676005	7547.65019
SNRPD1	3415.58524	3315.33537	1422.160881	1142.47544	2095.811051	1648.115673
TTC16	0	3.04720192	2.005868661	0	2.015212623	0.906060876
SUN5	0	0	0	0	0	0
SAMD14	8.79046815	3.04720192	4.011737323	13.0741028	7.053244179	3.624243505
PIP5KL1	36.1385913	52.8181666	5.014671654	28.159606	92.69978064	31.71213067
BPIFB6	0	0	5.014671654	0	0	0
TMEM92	406.314972	429.65547	6.017605984	21.1197045	10.07606311	15.4030349
FAM102A	846.815099	1284.90347	3434.047148	3142.81317	12286.75136	5548.716806
ACSF2	582.124336	566.779557	1687.938479	842.776781	221.6733885	1157.9458
GOLGA2	1833.30097	2204.14272	4307.011219	3501.84815	3902.459244	3408.601016
TRUB2	1032.39165	1046.20599	1075.145603	1089.17333	1971.885551	1416.173149
COQ4	1006.99696	912.129107	1075.145603	821.657076	1480.173671	1244.927644
SLC27A4	1746.37301	1783.62886	1102.224829	1639.29135	1801.600085	1735.106578
LINC00483	0	0	0	0	0	0
URM1	1216.01476	1152.85806	459.3439235	524.975513	1471.105214	733.9093097
CERCAM	1391.82412	1354.98912	427.2500249	522.964112	1400.572773	755.6547707
DOLPP1	437.56997	509.898454	682.9982792	672.813444	1295.781716	687.700205
CCDC103	33.5209852	30.9595715	20.63035918	28.5417721	24.13217116	45.45707416

ENDOG	305.712948	290.499916	411.2030756	380.154681	943.1195074	398.6667855
TBC1D21	0	0	0	0	0	0
PRRX2	0	1.01573397	0	0	22.16733885	0
UGT1A6	0	0	4751.521744	2333.79775	1957.023358	4058.282907
C15orf39	1108.57571	1305.21815	280.8216126	284.613161	995.5150356	390.5122376
ISLR2	0	1.01573397	0	0	2.015212623	0
SP2	886.860565	873.531216	675.9777389	792.49177	1066.047477	745.6881011
PRR15L	0	0	2407.042394	2131.07876	808.1002617	1902.72784
COQ7	326.22404	342.302349	568.6637655	812.605774	540.0769829	501.9577254
GPRC5B	412.175285	201.115327	68.19953449	115.655525	84.63893015	166.7152012
CRK	3685.15959	3709.46047	1168.418495	1197.78896	3098.389407	1691.615656
C16orf92	0	0	0	0	0	0
GOLGA6C	0.97671868	0	0	0	0.221673388	0
FBXO22	970.858372	1019.79691	755.209551	651.69374	1960.801882	859.8517715
TBC1D2B	1685.81645	1656.66211	242.710108	241.368052	1373.367402	377.8273854
NOD2	24.4179671	33.5192211	0	0	33.25100827	5.436365257
SNX20	1.95343737	0	0	1.00570022	0	2.718182629
LOXHD1	0	0	0	0	0	0
KATNAL2	0.97671868	1.01573397	94.27582709	99.5643213	51.38792188	116.881853
HDHD2	324.270603	340.230251	824.2214623	700.731682	534.031345	950.1951015
ZNF91	508.870434	534.276069	948.7758769	695.944549	816.1611121	984.8881724
IGF2	642.514852	359.569826	0	0	91.69217433	13.59091314
RNF214	896.627752	963.93154	662.9395926	765.337864	313.3655628	614.309274
CDK12	4111.98566	4089.34497	2017.903873	2118.00465	3072.191643	3549.946513
DPEP2	0	0	0	0	5.038031556	0.906060876
DUS2	297.899199	298.625788	456.3351205	360.040677	588.4420858	536.3880387
POP5	378.966849	563.732355	237.6954364	222.259748	665.0201655	368.7667766
ENGASE	384.827161	378.868772	838.4531005	605.43153	543.0998018	704.0093008
RBFOX3	0	0	0	0	1.007606311	0
ATP5L	3639.25382	3984.72437	3210.392793	2942.67883	3278.750937	2549.655306
CD3D	0	0	0	0	0	1.812121752
TBC1D16	1424.05584	1562.19885	18207.26984	7692.60095	2764.871718	11286.80033
ENTHD2	591.891522	541.386207	444.2999085	591.351727	422.1870444	515.5486385

MYO5B	199.250612	197.834506	4883.277227	7019.77745	1983.976827	8329.915968
ART5	0	0	0	0	0	0.906060876
ACAA2	900.534627	789.580803	17.04988362	183.037439	1343.139213	115.4140344
STIM1	2286.49844	2464.17062	505.4789027	1146.68933	658.9745276	976.8242306
RRM1	6136.72349	5757.18015	2437.130424	3443.51754	3467.173317	4415.23465
OR51E2	0	1.01573397	0	0	0	0
TRIM68	208.04108	225.492942	368.0768994	252.430754	73.55526072	267.2879585
MMP26	0	0	0	0	0	0
AC104389.28	0	0	0	0	0	0
OR51I1	0	0	0	0	0	0
OR51Q1	0	0	0	0	0	0
FN3K	87.9046815	79.2272498	947.7729425	929.266999	121.9203637	388.7001159
PRRT2	28.3248418	31.4877531	258.7570573	377.137581	81.61611121	258.2273497
ZNF23	282.906567	275.832718	200.9679812	137.801044	320.0359166	245.4337701
IRGQ	1273.64116	1443.35797	955.7964172	1126.38424	1472.112821	1128.951852
ZNF226	482.49903	420.513865	727.1273898	580.289024	313.3655628	596.1880565
ZNF180	475.661999	521.071528	224.6572901	358.029277	395.9892803	422.2243683
POM121L3P	0	0	0	0	0	0
PPP2R3B	407.291691	360.077693	361.0563591	276.567559	216.6353569	287.6743282
ZNF668	321.340447	386.994643	364.065162	465.6392	593.4801173	510.1122733
ZNF646	698.353859	732.344194	1326.88212	1453.23681	1286.71326	1518.558028
VKORC1	1038.15429	1033.00145	804.3533332	1091.87867	1135.804062	793.5552972
GNG8	0	0	0	0	0	0
LPO	0	0	0	0	0	0
CA4	0	0	0	0	242.833121	39.86667855
SMG8	848.768536	899.940299	306.8979052	348.977975	2373.920469	527.3274299
LINC00905	0	0	0	0	0	0
TPM4	21826.9278	19810.4285	7209.091969	6777.38358	22213.24539	9514.52714
RAB8A	3190.93994	3219.87669	1686.935544	1836.40859	3315.024764	2247.030973
GPX4	5238.1423	4780.04407	3996.693308	5006.37567	2616.75359	5034.980289
MIDN	1001.13665	1095.97696	2556.479609	2361.38411	3405.709332	4140.698204
JSRP1	31.2549979	51.8024326	51.14965087	63.3591136	44.3346777	41.6788003
FAM129C	1.95343737	1.01573397	9.026408976	5.02850108	3.022818934	12.68485227

KLHL26	359.432476	403.246387	347.0152784	612.471431	392.9664614	493.8031775
GATAD2A	5733.33867	5429.09808	2574.532427	2471.00543	4414.32325	3577.128339
NOS2P2	0	0	0	0	0	0
MVD	485.429186	957.837136	757.2154197	12975.5442	1921.505236	669.5789875
CDT1	1426.00928	1280.84054	615.8016791	1299.36468	3630.40554	1419.797393
TRAPPC2L	531.334964	545.449143	546.5992102	790.480369	1999.090922	730.2850662
ANKRD11	7673.88336	7880.63297	7553.981027	2658.91046	9948.762132	5028.637863
SPATA33	254.923576	256.980695	155.4548213	164.934835	525.9704945	227.4212799
SGK494	204.085369	206.011164	228.7492621	190.881901	213.3908646	307.8341827
PROCA1	23.4412484	20.3146794	73.21420614	119.678326	93.70738695	46.20910469
RPL13	19391.4993	18388.6548	26637.10339	19347.5702	45002.08589	19577.40232
ZNF641	139.670772	147.281426	598.7517954	728.126956	534.031345	708.5396052
LALBA	0	0	0	0	0	0
CACNB3	162.135302	181.816381	1508.413233	1389.8777	1063.024658	1610.070177
DHRS13	543.055588	508.88272	752.200748	701.97875	482.6434231	973.109381
TP53I13	969.881653	891.814428	804.3533332	828.696978	715.400481	663.2365614
KMT2D	4681.41265	4555.8208	6858.385892	7234.8565	10215.97933	7877.420106
CORO6	36.1385913	21.3304134	43.12617622	35.1995075	7.053244179	25.36970453
RHEBL1	41.9989034	38.5978909	63.18486283	25.1425054	14.10648836	21.74546103
TUBA1A	1921.65494	2673.43213	5.536197506	22.5276848	894.9156214	278.6680831
TUBA1C	17197.6231	17658.1491	4711.163666	11660.8325	19944.34773	8816.697174
ZNF610	38.0920287	39.6136249	103.3022361	52.2964112	81.61611121	93.32427025
ZNF528	477.615436	403.246387	944.7641395	428.428292	100.7606311	684.9820224
ZNF701	297.449908	323.826148	133.0191803	110.415827	456.5363436	255.3641973
SERTAD3	435.616533	474.347765	682.9982792	797.520271	738.5754262	727.5668836
NCKAP5L	950.347279	921.270713	1084.172011	1209.85736	954.2031768	1308.351905
RAB4B	117.372284	320.951621	370.2131495	873.54115	271.7514222	260.0394715
AQP2	0	0	0	0	0	0
GPD1	0.97671868	4.06293589	8.023474646	3.01710065	7.053244179	5.436365257
PROSER3	726.678701	720.155386	361.0563591	478.713303	580.3812353	519.1728821
CYP2S1	114.276086	182.832115	0	0	17.12930729	6.342426133
AXL	34598.3059	32141.8858	0	0	1175.876565	596.1880565
NFKBID	187.529987	146.265692	226.6631587	144.820831	137.0344583	233.7637061

TMC4	6.83703079	9.14160575	2105.15916	1344.62119	547.130227	1260.330679
ANKRD33	0	0	0	0	0	0
LAIR1	0	1.01573397	0	0	0	0
TTYH1	0	0	55.16138819	27.1539058	11.08366942	15.4030349
LENG8	5636.64352	5365.10684	5835.071936	4283.27722	1993.045284	3986.667855
CDC42EP5	7.81374947	3.04720192	285.8362843	200.134343	9.068456802	157.6545925
LAIR2	0	2.03146794	0	0	0	0
TMEM145	18.557655	35.550689	9.026408976	14.079803	0	17.21515665
ZNF526	614.356052	557.637951	613.7958104	498.857478	595.49533	641.4911003
TRAPPC9	386.780599	395.120515	2141.264796	1366.74659	700.2863863	1547.551977
KIR3DL1	0	0	0	0	0	0
NLRP7	0	2.03146794	0	0	0	0
ZNF146	3729.11193	3636.32762	3021.841138	2152.19846	2995.613563	3539.073782
ZNF283	245.15639	255.964961	367.073965	273.550459	330.4948701	427.6607336
PPP1R14A	0	0	0	0	0	0
SPINT2	4793.7353	4023.95202	15084.83439	17876.4521	9192.392378	13118.24837
C19orf33	1230.66554	1174.40178	7.231156524	8.28696978	556.1986838	117.0540046
YIF1B	1077.32071	1212.78636	911.6673066	758.297963	431.2555012	649.6456482
DNAAF3	67.3935892	143.21849	206.6044721	254.442155	83.63132384	242.8243148
PSCA	2.93015605	0	20.05868661	37.210908	454.4304464	139.5333749
ATCAY	0	0	0	0	0	0
LY6D	0	0	0	0	37.28143352	1.812121752
DAPK3	1736.60582	1548.99431	1507.410299	1929.02353	1086.199604	2225.285512
EEF2	50486.5888	47286.4793	86187.16171	78271.6364	73962.33367	65291.6528
TMIGD2	0	0	0	0	0	0
CHAF1A	2831.50746	2510.89438	1174.436101	1167.61795	1460.021545	2112.933963
UBXN6	2319.70687	2389.0063	2911.518362	2944.69023	903.8228612	2268.776434
CTB-50L17.10	2644.9542	2507.84718	2881.430332	2793.8352	1784.470777	2735.397785
PLIN4	2.93015605	5.07866986	3.008802992	3.01710065	30.22818934	2.718182629
SEMA6B	2151.71126	2570.82268	182.5340482	125.712527	695.2483548	792.8032667
ZNF444	836.071193	769.926351	1544.518869	1594.03484	1012.644343	1112.642756
NXN	2066.73674	1340.76884	5.014671654	4.02280086	203.5364749	57.98789608
FAM57A	932.766343	889.78296	63.18486283	92.5244198	538.0617702	116.881853

GLOD4	1803.02269	1681.03972	1073.139734	1033.85982	1256.48507	983.9821115
MFSD3	544.032307	456.064554	888.599817	984.580511	528.9933134	1288.418566
GPT	5.8603121	3.04720192	11.03227764	12.0684026	10.07606311	18.12121752
KIFC2	823.37385	888.767226	3624.604671	3649.68608	993.4998229	3199.300954
SLC43A2	173.855926	184.863583	141.4137406	532.015414	1157.739652	550.8850127
RILP	208.04108	329.097807	85.24941811	193.094441	43.32707139	101.4788181
SERPINF2	17.5809363	28.4405512	0	0	12.09127574	6.342426133
WDR81	620.216364	656.164146	258.7570573	645.659538	949.1651452	507.3940907
SRR	193.390299	166.580371	196.7355983	153.831905	250.7428306	106.4349711
TSR1	5332.88401	4743.47765	874.5587364	792.49177	3515.53842	1779.503561
TRPV3	2.93015605	1.01573397	0	0	99.75302482	5.436365257
HSD11B1L	117.821575	124.935279	257.754123	186.426649	41.31185876	129.0955536
CYB5D2	234.412484	281.35831	233.6836991	315.789868	302.2818934	231.0455234
GGT6	0	0	428.2529592	484.747504	44.3346777	550.8850127
C19orf48	2188.82657	1935.11542	1150.365677	1263.15947	2227.817554	2089.37638
KLK1	2.93015605	1.01573397	0	0	2.015212623	0
KLK4	0	0	0	0	0	0
KLK2	0	0	0	0	0	0
KLK5	0	0	0	0	7.053244179	0
KLK6	0	0	0	0	389.9436425	128.6606444
KLK11	0	0	0	0	152.148553	11.77879139
KLK13	0	0	0	0	2.015212623	1.812121752
ZNF83	745.236356	738.905835	1543.255172	1150.52105	890.0287308	1413.3553
KRT80	4325.88705	3887.39674	61.17899417	202.145743	16173.21989	2625.809722
KRT1	0	0	0	0	0	0
ACER1	0	0	0	3.01710065	0	0
OTUB1	2384.17031	2537.30346	2282.678537	1942.00712	2339.661855	2344.885548
RCOR2	32.2317166	34.5349551	22.06455528	6.03420129	73.55526072	23.55758278
ANGPTL4	42.9756221	27.4248173	0	0	169.2778603	130.4727662
CTD-2550O8.5	0	0	0	0	0	0
CD320	1298.05913	1134.57485	1188.477182	1862.5568	504.810762	1465.100437
SPRYD3	547.939182	635.849467	673.9718702	595.374528	2061.562513	566.2880476
IGFBP6	864.396035	545.449143	0	1.00570022	63.47919761	10.87273051

SOAT2	1.95343737	0	0	0	1.007606311	0
ZNF558	633.890426	609.440383	423.2382876	419.37699	126.9583952	414.9758813
CABP2	0	0	0	4.02280086	0	0
NDUFV1	3023.92105	2868.43274	4238.400482	3103.59087	3034.91021	4707.892313
CDK2AP2	1429.91615	1901.454	4130.083574	3584.31557	1084.184391	1761.382343
C3P1	0	0	0	0	0	0
NUDT8	97.6718684	134.076884	137.4020033	336.909572	143.0800962	256.415228
TBX10	0	0	0	0	0	0
CTD-2369P2.10	31.7921932	28.0647297	79.51263374	50.5565498	35.33675334	58.52247199
PRDX2	3333.54087	3386.45706	9488.761703	10670.4793	4855.654814	11237.87305
OR8J3	0	0	0	0	0	0
OR5I1	0	0	0	0	0	0
ZNF232	140.64749	167.596105	167.4900332	181.026039	192.4528055	128.6606444
MIS12	998.206495	976.120347	384.1238487	337.915272	999.5454608	432.1910379
CD300C	0.97671868	2.03146794	0	1.00570022	0	1.812121752
CD300A	0	0	1.002934331	2.01140043	0	1.812121752
TEKT1	0	0	0	0	0	0
HID1	184.599831	236.666016	33403.73082	27084.5125	3801.698612	26732.42009
ICT1	662.215268	608.424649	3363.841745	2780.7611	1009.621524	2741.740211
ATP5H	1595.95833	1639.39463	8526.94768	7924.9177	2371.905257	7811.150814
TMEM88	3.90687474	0	15.04401496	20.1140043	6.045637868	14.49697402
EVPL	2399.79781	2119.8368	3787.019857	4597.89042	3335.17689	5076.414453
SRP68	3703.71725	3792.75065	3993.684505	3338.92472	3531.660121	4825.680227
MGAT5B	982.578996	773.989287	0	0	98.74541851	12.68485227
TMC8	198.273893	288.468448	0	6.03420129	44.3346777	29.90000891
TK1	4815.22311	3995.89745	1646.818171	2767.68699	4763.96264	4227.680048
TMEM68	293.015605	314.877531	486.4231504	419.37699	390.9512488	343.3970721
CYP7A1	0	1.01573397	0	0	0	0.906060876
RP11-25K19.1	36.1385913	31.4877531	0	0	0	0
GSDMA	0	0	0	0	0	1.812121752
KRT24	0	0	0	0	5.038031556	0
TMEM99	311.57326	432.702672	191.5604572	182.031739	141.0648836	129.5667053
GHDC	723.748545	768.910617	309.9067082	467.6506	292.2058303	379.6395071

FAM234A	1127.81706	1235.78258	2173.529193	2337.46855	2928.779037	2773.044615
SOST	69.3470266	43.6765608	0	0	1.007606311	0
PRR25	0	0	5.014671654	1.00570022	1.007606311	2.718182629
ZNF598	1885.06706	1771.44005	3295.642211	2099.90205	2334.623823	3161.246397
RAB26	24.4179671	33.5192211	1250.65911	1688.57066	191.4451991	648.7395873
MLST8	1306.8496	1357.02059	1764.161488	2089.84505	1336.085969	1865.579344
E4F1	593.84496	567.79529	1629.768287	1950.05272	906.8456802	1489.56408
DNASE1L2	18.557655	24.3776153	133.390266	142.809431	32.24340196	77.92123535
ECI1	962.067904	933.45952	2164.332286	1398.929	1400.572773	1377.212532
CASKIN1	23.4412484	21.3304134	86.25235244	100.570022	331.5024764	223.7970364
ABCA3	839.978068	660.227082	6450.873615	7982.24261	2918.027877	9326.084599
KCTD5	1849.90519	1728.45419	1192.7898	832.719778	2466.257512	1260.973982
SRRM2	22438.1583	21549.812	27786.29563	34513.62	29651.83853	42110.99134
ZNF597	0	0	95.27876142	82.4674177	184.391955	203.8636971
NLRC3	1.95343737	0	20.05868661	13.0741028	2.015212623	22.6515219
SDHAF2	593.141722	614.498739	432.5655768	348.465068	479.1974095	503.1537258
DDB1	10519.2602	9806.9115	6826.973989	6938.32579	9291.137796	11574.02163
VPS37C	709.097764	678.510293	2177.370432	1749.91838	1155.724439	1307.445844
VWCE	6.83703079	11.1730737	22.06455528	28.159606	10.07606311	32.61819154
RAB3IL1	285.201856	402.230653	68.19953449	46.2622099	139.049671	48.92728731
BEST1	99.5178667	124.935279	706.9684097	248.407953	177.3387108	211.1121841
FTH1	29750.8902	49973.0551	120965.1332	67582.9137	50467.79594	61861.46035
BSCL2	175.955871	259.377827	294.270962	298.582337	420.1819079	461.4839861
POLR2G	1142.76086	1299.12375	1201.515328	1129.40134	1757.265407	1373.588288
SLC3A2	4921.68545	4974.04926	10186.804	5331.21684	10051.88056	4748.665052
HRASLS5	2.93015605	1.01573397	5.014671654	0	0	1.812121752
C11orf84	1585.21442	1639.39463	630.845694	657.727941	846.3893015	901.5305718
ATG16L2	603.612147	507.866986	1031.016492	790.480369	409.0881624	507.3940907
C2CD3	2147.80439	1437.26357	859.5147214	924.238498	1383.443465	1459.664072
TRANK1	308.643104	479.426435	79.23181213	85.4845183	193.4604118	383.2637506
TTC21A	45.9057781	77.1957819	215.6308811	303.721465	27.2053704	164.9030795
RPSA	20833.136	21057.506	16421.71576	19612.8036	23722.8714	18747.29653
ENTPD3	5.8603121	4.06293589	0	0	5.038031556	0

CTNNB1	10726.3246	11557.0211	5285.463923	5849.15245	6901.095626	7142.477887
ULK4	93.7649937	120.872343	53.15551953	98.5586211	75.57047335	119.6000357
FADD	2013.01721	1891.29666	2022.918545	1696.61626	1820.744604	2221.661268
LTBP3	1687.76989	1298.10802	1330.893857	1219.91436	1622.246161	2044.073337
NAALADL1	1.95343737	0	0	0	0	0
SAC3D1	1057.78633	1050.26893	299.8773649	483.741804	831.2752068	472.9637774
BATF2	29.3015605	39.6136249	1.002934331	0	4.030425245	15.4030349
SLC22A11	0	1.01573397	0	0	0	0
SF1	11073.0597	10440.7295	5626.461595	5391.55886	6454.72603	7275.668836
MAP4K2	865.372754	831.886123	485.4202161	702.984451	411.103375	960.4245287
C11orf85	0	0	0	0	0	0
CCDC88B	107.439055	157.438766	77.22594346	67.3819144	44.3346777	65.23638308
SCARA3	149.437959	89.3845896	13.0381463	14.079803	850.4197267	146.7818619
PBK	1253.13007	1233.10104	249.7306483	607.44293	1499.318191	694.0426312
SCARA5	0	0	0	0	44.3346777	0.906060876
PNOC	0	0	0	0	0	0
COPS6	2351.93859	2284.3857	2555.476675	1988.26933	3619.32187	2855.903882
PAFAH1B2	6102.53834	5658.65396	1842.390366	2221.59178	2122.018892	2737.209907
ANKS3	312.549979	233.618814	618.810482	372.10908	371.8067289	551.7910736
NUDT16L1	424.872627	405.277855	1340.9232	976.534909	1083.176785	1473.254985
KIAA1586	760.209453	691.826566	188.5516542	214.445457	324.731362	221.9849147
RAB4A	600.681991	631.786531	2152.297074	2664.09987	1054.963808	2066.724859
ZNF355P	0	0	0	0	0	0
OR1F1	0	0	0	0	0	0
OR2W6P	0.97671868	2.03146794	2.005868661	4.02280086	0	8.154547886
AC098817.5	0	0	0	0	0	0
OR2B2	0	0	0	0	0	0
KCNJ4	0	1.01573397	0	0	14.10648836	6.342426133
SETD5	5335.81417	5203.60514	6028.638262	4803.22423	6237.083067	5931.980556
VASN	421.942471	458.096021	758.218354	472.679101	739.5830325	1137.1064
FAM83B	3.90687474	3.04720192	89.26115543	184.043139	2039.395174	222.8909755
HIST3H3	0	0	0	0	0	1.812121752
THAP9	64.4634331	53.8339005	130.381463	121.689726	114.8671195	137.7212532

OR2C1	0	0	0	0	0	0
RNF187	1613.53927	1471.79853	29613.64198	14242.7265	2928.103941	7689.738656
HOOK3	1547.1224	1517.50655	1293.785287	1465.30521	1414.679261	1265.767044
MAPK1IP1L	5388.55698	5354.9495	2566.508952	2824.00621	3235.423866	2874.93116
DDIT4	4134.45019	4848.09825	14665.90872	23180.3843	1329.032725	3603.404105
RBPJ	4297.56221	3703.36606	1162.400889	1200.80606	1577.911483	1267.579166
LMBRD1	583.101054	754.690341	1523.457248	1875.6309	419.1642255	1218.651878
ZCCHC4	293.992324	329.097807	237.6954364	175.997538	164.2398287	168.527323
PTGDR	0	0	0	1.00570022	0	0
TTC39C	545.009026	421.529598	299.8773649	341.938073	273.0613104	504.675908
GLYCTK	218.784985	219.398538	44.12911055	132.752428	62.4715913	88.79396587
GNG4	0.97671868	1.01573397	0	0	105.7986627	5.436365257
UBTD2	1590.09802	1741.98376	728.1303241	795.508871	995.5150356	870.724502
POLR2J3	1014.77164	763.598328	588.9330683	546.980233	2050.438539	768.2852593
NKIRAS2	1017.74087	1119.33884	451.3204488	430.439692	1064.032265	536.3880387
DNAJC7	2619.55951	2606.37337	1074.142668	864.902185	1973.900764	1223.182183
KCNV2	0	0	0	0	0	0
IRF2BP2	2410.54171	2285.40144	12620.92562	9787.4745	1863.06407	9180.208797
PTF1A	0	0	0	0	0	0
NT5DC2	2192.73345	2440.80874	3478.176259	1963.12682	2077.684214	2354.852217
FOXI1	0	0	0	0	37.28143352	0
SMIM4	227.575453	290.499916	301.8832335	362.052078	255.9320031	236.4818887
COA6	504.96356	579.984098	984.8815128	979.55201	384.9056109	675.9214136
KIF5C	8.79046815	9.14160575	841.4619035	1837.41429	803.0622301	2265.15219
MGAT2	3015.13058	3523.58115	1824.337548	1988.26933	1048.91817	1826.618726
BMI1	2061.53082	2049.1925	2533.010946	2311.22984	2222.678762	3485.516524
THAP11	882.95369	975.104613	1095.204289	1156.55525	1382.435859	1631.815638
MMADHC	2724.06841	3053.29632	1740.091064	1999.33203	2939.18761	2178.170346
PDHB	2912.57512	2567.77548	1238.623898	1716.73027	990.477004	1320.130697
PXK	1021.64774	901.971767	535.5669326	396.245885	204.5440812	366.048594
HIST1H1E	99.6253058	119.856609	54.15845386	52.2964112	28.21297672	18.12121752
PCMTD1	328.177478	281.35831	1322.870382	808.582973	290.1906177	913.3093632
KCTD6	358.455757	333.160743	477.3967414	273.550459	98.74541851	279.0667499

MPLKIP	795.049009	877.594152	392.1473233	353.000776	635.7995824	315.3091849
ACOX2	99.6253058	189.942253	28.08216126	5.02850108	17.12930729	13.59091314
FAM107A	10.7439055	5.07866986	0	0	2.015212623	0
IRF2	439.523408	506.851252	594.7400581	545.089517	164.2398287	549.9789518
MOBP	0	0	1.002934331	2.01140043	0	6.342426133
CX3CR1	0	0	6.017605984	5.02850108	0	15.4030349
C8orf22	0	0	0	0	0	0
XIRP1	0	0	0	0	0	0.906060876
INSM2	2.93015605	2.03146794	1.002934331	2.01140043	0	7.248487009
DEGS2	0.97671868	1.01573397	1069.127997	1655.38255	373.8219415	1418.891332
SCN11A	0	0	0	0	0	0
LINC00917	0	0	0	0	0	0
ARF4	6040.02834	5959.31122	5752.831321	3718.0737	2726.582678	4119.858804
SEPT2	9469.28764	8315.81403	6102.855402	5087.83739	9552.107831	6864.317198
FILIP1L	406.314972	202.13106	0	0	37.28143352	6.342426133
MFSD2A	284.225137	367.695698	790.3122526	2421.72612	29.22058303	686.7941441
DTYMK	1909.48503	1691.19706	574.6813715	995.643213	1835.858699	1109.924573
TAP1	2944.80683	3187.3732	144.4225436	258.464955	864.5262151	648.7395873
ING5	969.881653	977.136081	637.8662343	590.346027	1019.697587	873.4426846
ATG4B	912.255251	858.295207	1612.718404	1497.48762	1745.174131	1495.000446
BDKRB2	4.19012315	15.2360096	7.020540315	10.0570022	135.0192457	359.6971072
MLKL	421.942471	584.047034	94.27582709	104.592822	332.5100827	233.7637061
CMAHP	5.8603121	6.09440383	26.0762926	15.0855032	35.2662209	46.20910469
RFWD3	3005.36339	2848.11806	843.4677721	927.255599	4419.361281	1774.067196
MTNR1A	0	0	0	0	0	0
KCNG4	0	0	0	0	0	0
RHOH	0	0	113.3315794	174.991838	10.07606311	118.6939748
KLHL30	10.7439055	6.09440383	0	0	5.038031556	0.906060876
COG7	318.410291	335.192211	875.5616707	765.337864	869.5642466	834.482067
CDC40	706.167608	688.667633	790.3122526	892.056091	1049.925776	1003.00939
STIP1	9122.55251	9247.24208	5354.666392	4021.79516	7957.06704	7305.568845
SCNN1B	0	0	0	0	163.2322224	28.08788716
HR	8.79046815	6.09440383	112.328645	380.154681	1123.481037	549.9789518

TXNDC2	9.76718684	11.1730737	0	0	3.022818934	0
RAB31	2104.82876	2062.9557	2.005868661	0	2735.651135	212.9243059
REEP4	614.356052	659.211348	727.1273898	1000.67171	1539.622444	906.0608762
TNXB	133.81046	145.249958	150.4401496	57.3249123	36.73732611	210.2061233
LGI3	0.97671868	0	0	0	4.030425245	0
SFTPC	0	0	0	3.01710065	0	0.906060876
BMP1	748.166512	639.912403	70.20540315	80.4560172	769.8112218	236.4818887
ATXN2L	4569.09	4580.96021	6454.885352	5773.72494	6297.539446	6058.829079
PHYHIP	16.6042176	9.14160575	0	0	0	2.718182629
CCDC110	6.83703079	5.07866986	3.008802992	9.05130194	0	4.530304381
POLR3D	499.103247	534.276069	249.7306483	289.641662	967.3020588	395.042542
FEN1	5109.21544	4820.66327	1155.380349	1462.28811	3448.028797	2037.730911
SDPR	1159.36508	569.826758	0	0	24.18255147	5.436365257
MTCL1	3397.02758	2309.77905	359.0504904	337.915272	649.9060708	426.7546727
GBX2	0	0	0	0	24.18255147	0.906060876
HFE2	0	0	0	0	0	0
SCGB1D1	0	0	0	0	0	0
HEXIM2	168.972332	179.784913	312.9155112	376.131881	159.2017972	275.4425064
FNTA	1029.46149	1082.77241	1388.061114	1369.76369	1289.736078	1425.233758
SERINC2	2933.08621	2059.9085	12701.16036	10540.744	236.7874832	8717.21169
MYL1	3.90687474	1.01573397	0	0	0	0
TRAPPC11	708.121046	633.817999	1530.477789	1390.8834	359.7154531	1486.845898
CHRM1	0	3.04720192	48.14084787	65.370514	8.06085049	48.02122644
COL3A1	0.97671868	1.01573397	0	0	28.21297672	10.87273051
GFRA2	0	0	0	0	0	0
ING2	310.596541	236.666016	250.7335827	354.006476	123.9355763	353.3637417
CDKN2AIP	480.545592	504.819784	532.5581296	580.289024	130.9888205	657.8001961
SNRNP48	581.147617	557.637951	513.5023773	590.346027	613.6322436	602.5304827
TMEM223	368.222944	463.174691	257.754123	263.493456	444.3543833	303.5303935
SLC20A2	1064.62337	909.081905	473.3850041	645.659538	755.7047335	810.0184233
CRYGA	0	0	0	0	0	0
DYNLRB2	0	2.03146794	22.06455528	14.079803	0	6.342426133
TMUB2	955.230873	883.688556	423.2382876	419.37699	494.7346988	436.7213423

ADAM29	0	1.01573397	0	0	1.007606311	2.718182629
STAT3	4834.75749	4471.26095	1592.659717	1536.70993	2821.297672	2274.212799
ZSWIM1	443.430282	414.419461	497.455428	433.456793	462.4912969	449.4061946
ADAM9	8205.41366	6032.44406	1110.248304	1209.85736	3207.210889	1400.770115
ADAM18	0	0	0	0	5.038031556	0
GDNF	0	0	0	0	18.1369136	1.812121752
SPINT5P	0	0	0	0	0	0
DPCR1	0	1.01573397	0	0	1.007606311	0
WFDC13	0	0	0	0	1.007606311	0
AXIN2	127.62783	77.1957819	152.4460183	138.78663	103.7834501	183.024297
NDUFS5	1529.54146	1576.41912	1065.116259	992.626113	1812.683754	1102.676086
VWA3B	0	2.03146794	0	0	4.030425245	0
ZNF30	122.089835	117.825141	223.6543557	232.31675	141.0648836	349.7394982
UGT3A2	0	0	0	0	0	0
FAM84B	143.577647	163.53317	10084.5047	11509.2333	8551.554764	18052.3569
LDLRAD4	37.11531	34.5349551	1536.495395	1007.71162	325.4568385	838.1063105
KCTD19	1.95343737	1.01573397	0	0	0	1.812121752
SLC16A4	6.83703079	7.11013781	19.05575228	23.131105	20.15212623	16.30909577
IL7R	1207.22429	1124.41751	0	0	28.21297672	15.4030349
TMEM208	293.992324	328.082073	739.1626017	612.471431	843.3664825	749.3123446
LRP1B	1.95343737	3.04720192	1052.679874	979.280471	158.1941909	1200.050449
WFDC12	0	0	0	0	0	0.906060876
AHCYL1	5290.88511	4872.47586	8139.815028	5200.47581	2921.050696	7106.235452
DNAJC21	1966.13471	2013.18473	2182.385104	2367.41831	1430.800962	1934.439971
PKIG	651.471362	572.87396	0	0	169.2778603	38.96061768
NPNT	0	0	6155.007988	4020.78946	1868.102101	8940.102665
LINC01620	0	0	0	0	0	0
CA7	0	0	0	0	0	0
FAM178B	0	1.01573397	0	0	113.8595132	3.624243505
TSPY2	0	0	0	0	0	0
SEMA4C	1130.06352	1012.68677	567.6608312	451.559397	6810.411058	905.1548153
CNNM3	883.930409	830.870389	445.3028428	848.810982	1566.827814	828.1396408
GSTM4	526.900661	740.22629	416.2177472	643.416827	249.7553764	708.4399385

TET2	677.842767	594.204374	1183.46251	1430.10571	6547.425811	1696.14596
CXXC4	7.81374947	17.2674775	128.3755943	152.866433	76.57807966	338.8667677
TCTN2	465.894812	464.190425	211.6191438	220.248347	302.2818934	236.4818887
SHOX2	507.893716	566.779557	1.002934331	2.01140043	87.66174908	27.18182629
PPIP5K1	698.041309	788.971363	2148.556129	2377.4552	532.6206961	2081.258075
TSPAN5	1272.66445	1323.50137	345.0094098	790.480369	89.6769617	492.8971166
OR12D2	0	0	0	0	0	1.812121752
ABHD15	511.80059	666.321486	722.1127181	778.411967	727.4917568	895.1881457
ZBTB5	428.779502	407.309323	1262.694322	1473.35082	778.8796786	1203.248844
CHTF8	2565.83998	2845.07086	1237.620964	1277.23927	3774.493242	2037.730911
ADAL	247.109827	280.342576	463.3556608	445.525195	184.391955	452.1243772
LCMT2	395.571067	400.199185	454.3292518	550.118018	467.5293284	704.0093008
SNTB2	2097.01501	1767.37711	383.1209143	594.368827	2219.968301	839.9184322
IL12A	66.4168705	59.9283044	0	0	3.022818934	1.812121752
ZNF507	1574.47052	1151.84232	920.6937156	961.449406	1152.70162	1076.400321
STX18	773.561198	651.075319	580.6989775	535.032515	406.0653434	588.0335086
NSG1	0	0	0	0	0	0
ZBTB49	108.415774	84.3059197	78.2288778	93.53012	88.66935539	105.1030616
GFM1	1354.70881	1332.64297	1509.416168	1393.9005	2187.513302	1967.058162
OR13J1	0	0	0	0	0	0
HTR1E	0	0	0	0	0	1.812121752
FSTL5	6.83703079	10.1573397	0	0	6.045637868	6.342426133
TPTE2P5	3.01806073	4.38797076	13.0381463	12.0684026	4.030425245	4.919910558
DDX19A	944.662777	1041.08669	1008.059325	945.720255	1838.196346	1533.571457
ATOH8	35.1618726	10.1573397	24.07042394	113.644124	27.2053704	74.29699185
SOX14	0	0	0	0	0	0
ANKRD49	437.56997	429.65547	774.2653033	625.545534	228.7266327	506.4880298
SFTPB	0	0	0	0	0	0.906060876
USP39	3254.42665	3064.46939	2519.371039	2196.44927	2584.510188	3164.87064
TNIP2	931.789624	1123.40177	423.2382876	521.958412	1023.728012	453.0304381
C2orf68	931.789624	1028.93851	1638.794696	1929.93871	1137.587525	1564.767133
TMEM150A	179.716238	265.106567	584.7107148	786.457569	182.3767423	845.3547975
RNF181	899.557908	1045.19026	1302.811696	1205.83456	1178.899384	1093.615478

VAMP5	9.76718684	7.11013781	53.15551953	68.3876147	44.3346777	48.02122644
BTNL3	0	0	0	0	0	0
LRRC28	214.87811	254.949227	165.4841646	243.379452	229.734239	191.1788449
MAT2A	7417.20169	7388.44891	14071.16866	8556.49743	6816.456696	12071.44905
PLA2G4F	0	0	146.4284123	190.077341	169.2778603	318.9334284
ENHO	6.83703079	15.2360096	17.04988362	55.3135119	21.15973254	34.43031329
ZNF608	246.133108	247.839089	1.002934331	0	23.17494516	11.77879139
SLC35G2	310.596541	413.403727	1.002934331	0	24.18255147	9.060608762
INPP5D	0	0	0	4.02280086	10.07606311	6.342426133
LETM1	2060.87642	2057.87703	1492.366284	1382.8378	2219.756704	3057.049396
CTRB1	0	0	0	0	0	0
CTRB2	0	0	0	0	0	0
TRIM49	0	0	0	0	0	0
TMEM129	772.584479	779.067957	1439.210765	990.614712	632.7767635	1080.024564
PPIC	465.894812	570.842492	1189.480116	1085.15053	1439.869419	1076.400321
SPRY3	0.48835934	5.07866986	44.12911055	68.8904648	68.01342601	38.50758724
CEP120	1038.25196	944.632594	578.6931088	413.342789	685.1722917	463.9031686
STXBP6	236.365921	123.919545	191.5604572	57.3249123	3.022818934	126.8485227
TM4SF20	0	0	0	0	0	0
MFF	1042.15884	1157.93673	1651.832843	1602.08044	1184.945022	1492.282263
GRM5	0.97671868	0	0	0	0	0.906060876
LGALS9	18.557655	10.1573397	0	0	7.053244179	19.32627849
PMCHL1	0	0	0	0	0	0
JMJD7-PLA2G4B	160.738594	151.344362	744.8492394	721.9419	248.1835105	594.3668742
OR7E102P	0	0	1.002934331	2.01140043	2.015212623	5.436365257
CPLX1	91.8115563	59.9283044	140.4108063	62.3534134	148.1181278	122.3182183
PXDC1	1094.90164	887.751492	0	0	47.35749663	9.966669638
SIGLEC7	0	0	0	0	0	0
NTSR2	0	0	0	0	0	0.906060876
E2F6	648.257958	723.009599	556.5182308	521.867899	1186.436279	641.0924335
FEM1B	1461.17115	1535.78977	4433.972676	4567.89038	3023.82654	4631.783199
COMMD8	386.780599	450.985884	218.6396841	265.504857	298.2514681	216.5485494
ATP5I	583.101054	631.786531	1014.969543	947.369603	944.1271137	877.972989

UQCRFS1	3131.63358	3100.92409	2539.008493	2691.11298	2800.682046	3025.681569
MFSD7	143.577647	94.4632594	0	0	4.030425245	1.812121752
COL4A3	2.93015605	1.01573397	0	0	21.15973254	10.87273051
MAP2K1	2390.03062	2299.62171	2463.206716	2544.42155	2184.490483	2589.521984
KLK7	0	0	0	0	7.053244179	2.718182629
PMCHL2	0	0	0	0	0	0
HNRNPH1	17866.3042	15146.6352	8488.00374	9048.37535	11225.42956	12389.92945
IRS1	5001.77638	3466.70005	1163.403824	1199.80036	6558.50948	1502.248933
MECP2	2790.48528	2608.40484	4196.27724	3836.74632	4916.111193	3898.77995
VCX3A	0	0	0	0	0	0
UPF3A	2008.81732	1966.08515	638.7889339	571.237722	1226.146044	651.2674972
ZBBX	0	1.01573397	0	1.00570022	3.022818934	3.624243505
ACTBL2	3.90687474	1.01573397	0	0	3.022818934	0
ROR2	0.97671868	0	773.262369	1002.68311	0	491.0849949
RP3-509L4.3	0	0	0	0	0	0
AR	0	3.04720192	19092.86085	15558.1823	2430.346423	22002.78232
DHRSX	298.905219	313.414874	107.4242962	108.545224	174.9607599	74.4510222
C8orf46	138.694053	107.667801	125.3667913	21.1197045	23.17494516	13.59091314
HSPBAP1	250.039983	232.60308	88.2582211	113.644124	91.69217433	97.85457463
ASMTL	367.734584	329.605674	1633.278558	769.360665	642.8528266	806.8472102
SLC25A6	4977.35841	4611.9401	4686.21066	6404.80182	6376.132738	2713.199294
CHST14	964.021341	908.066171	296.8685619	297.687264	716.4080873	403.1970899
PARM1	29.3015605	56.8811024	210.6162094	220.248347	60.45637868	627.9001872
CSNK1G1	840.808279	821.586581	1649.506035	1305.99224	1639.083263	1941.969337
FAM110B	0.97671868	0	1229.597489	939.324001	74.56286704	1602.82169
ARMC4	126.973429	142.974714	0	0	9.824161535	4.530304381
AFAP1L2	1267.78085	1025.89131	0	0	232.7570579	14.49697402
ZNF354A	405.103841	393.160149	628.6091505	692.2838	606.0651202	733.76434
ATF5	874.163222	1329.59577	757.2154197	2410.08011	1592.017972	1724.233847
UBE2V2	1583.26099	1700.33867	1506.407365	1241.03407	3083.275313	1725.139908
GOT1L1	0	0	0	0	0	0
ZBTB43	919.092282	1128.48044	853.4971154	727.121256	708.3472368	931.4305807
RP11-167P23.2	0	0	0	0	0	0

CPT1C	138.694053	262.059365	5.014671654	1.00570022	37.28143352	18.12121752
PCSK9	218.784985	768.910617	0	0	3.022818934	0
XPO6	4234.07549	4276.24002	2956.650407	3020.11775	4841.548326	4748.665052
GSG1L	0	0	0	0	4.030425245	0
MN1	14.6507803	8.12587178	64.18779717	12.0684026	8.06085049	82.45153973
APEX2	1120.29633	1133.55911	903.643832	1425.07721	1213.157999	1618.224725
NSMCE1	360.409194	404.262121	567.6608312	553.135119	556.1986838	520.0789429
CCDC126	216.831548	194.005189	266.780532	315.789868	123.9355763	182.1182361
IL13	0	1.01573397	2.005868661	1.00570022	0	3.624243505
RP11-231C14.4	225.319233	209.789695	598.8320302	638.358155	550.5460124	491.7192375
OR10G3	0	0	0	0	0	0
RAB3B	4.88359342	3.04720192	30.08802992	141.80373	504.810762	65.23638308
OR6F1	0	0	0	0	0	0
CD2BP2	1422.1024	1535.78977	2432.115752	2419.71472	2852.533467	2892.146317
RSPO1	0	0	0	0	1.007606311	0.906060876
RGS14	136.740616	179.784913	287.8421529	351.995075	68.51722917	229.2334017
TBC1D10B	1657.49161	1741.98376	4047.842959	4740.87082	3375.481143	4905.413584
LMAN2	3377.49321	3621.09161	4155.156932	3547.10466	6053.698718	3792.770828
GCSAML	0.97671868	0	0	0	4.030425245	2.718182629
RAB24	684.787236	670.110174	1538.140207	948.033365	690.9357998	1003.018451
PRELID1	4900.63716	4959.7071	2813.832558	2753.44628	7876.257014	4481.295548
THBS3	137.717334	80.2429838	1108.242435	937.312601	217.6429632	736.6274923
CA5B	259.836471	218.433591	233.7338458	180.724329	303.2794236	134.9124645
SLC50A1	667.098861	816.650114	2627.687946	5816.97005	1477.150852	3305.310076
EFNA1	114.276086	151.344362	2161.323483	2918.54203	1199.05151	1666.245951
CXCL10	3.90687474	1.01573397	0	0	1.007606311	5.436365257
NPIPB3	272.387307	358.82834	1140.025424	859.95414	679.0661974	977.7665339
SH3TC2	751.096668	642.959604	8.023474646	7.03990151	37.28143352	34.43031329
CXCL11	2.93015605	16.2517436	0	0	1.007606311	0
ZRSR2	208.959195	222.364481	509.0092315	346.876061	303.2894997	304.9710303
NMD3	1417.21881	1373.27233	1641.803499	1714.71887	12700.87755	2042.261215
ADRB2	1831.34753	1560.16738	11.03227764	4.02280086	42.31946507	28.99394804
RP11-220D10.1	0	0	0	0	0	0

B3GALNT1	725.701982	622.644925	979.8668411	1107.27594	3193.1044	788.2729623
GPRIN1	643.657613	634.833733	307.9008395	353.000776	1121.465824	640.5850395
HSPB3	0	0	0	0	0	0
KCNAB1	0	0	0	1.00570022	1.007606311	2.718182629
MRPL1	579.19418	623.660659	405.1854696	261.482056	581.3888416	407.7273943
SHE	2.93015605	2.03146794	0	0	1.007606311	0
NR0B1	0	0	0	0	0	0
PGM2	2019.85424	2152.34029	144.4225436	305.732866	1516.447498	496.5213601
STK32A	0	1.01573397	10.02934331	0	2.015212623	0.906060876
IL1RAPL1	138.694053	185.879317	0	0	27.2053704	11.77879139
P2RY12	0	0	0	0	0	0
C22orf15	0	0	0	0	0	0
GUSBP12	0	0	0	0	0	0
OR5AU1	0	0	0	0	0	0
KIAA1024	76.1840573	96.4947274	111.3257107	138.78663	24.18255147	143.1576184
PDILT	0	0	0	0	1.007606311	0
UMOD	0	0	0	0	0	0
GP2	0	0	0	0	8.06085049	0
SLC33A1	613.379333	820.71305	3165.260748	1905.80191	1174.868959	1651.676492
SNUPN	545.27274	574.651495	229.4212281	275.441175	157.8012244	241.4833447
CRADD	156.274989	134.076884	229.6719617	315.789868	184.391955	182.1182361
SIN3A	3256.38009	2976.10054	1301.808761	1475.36222	3372.458324	1996.05211
ARL13B	842.908224	895.877364	289.8480216	279.58466	474.5825726	389.6061768
RNASE2	0	0	0	0	0	0
ELSPBP1	0	0	0	0	0	0
RNASE3	0	0	0	0	0	0
PTK2	4645.27406	3839.47442	5268.414039	4000.67546	3137.686053	4667.119573
RSPH10B2	0	1.70643307	21.92414447	7.4622956	4.030425245	11.6881853
PTAFR	10.7439055	13.2045416	2.005868661	10.0570022	102.7758438	25.36970453
PTPN9	1619.39958	1594.70234	485.4202161	573.249123	974.355303	547.2607692
RNASE6	0	0	0	0	0	0.906060876
NPR1	13.6740616	21.3304134	1.002934331	1.00570022	89.6769617	57.98789608
KCNK9	0	0	8.023474646	4.02280086	0	7.248487009

CXCL8	3382.3768	4044.65268	0	23.131105	148.1181278	84.26366148
SCN9A	53.7195276	62.9755063	0	0	0	2.718182629
RASSF6	3.90687474	4.06293589	12.03521197	29.1653063	0	19.0272784
COL22A1	0	0	0	0	0	0
SDC2	1077.32071	932.443787	244.7159767	297.687264	86.65414277	289.0334195
CD52	0	0	0	0	3.022818934	0
MMGT1	1249.2232	1275.76187	1094.201355	1637.27995	1920.497629	1488.65802
SPRR1B	0	0	0	0	6.045637868	0.906060876
SPRR1A	0	0	0	0	1.007606311	0.906060876
OR4K14	0	0	0	0	0	0
OR4K15	0	0	0	0	0	0
TM2D2	905.41822	917.207777	464.3585951	561.18072	640.837614	638.7729177
HTRA4	0.97671868	1.01573397	0	0	2.015212623	1.812121752
PLEKHA2	1762.00051	1151.84232	58.17019118	54.3078116	677.1114412	170.3394447
CLIC4	10238.1118	8968.92082	1112.254173	663.762142	3079.234811	457.5607425
SLC38A11	0	0	0	0	1.007606311	1.812121752
GPR183	0	0	0	0	0	0
CRCT1	0	0	0	0	0	0
CCDC8	573.333867	514.977124	0	0	15.11409467	2.718182629
METTL15	435.255147	410.376839	766.9137947	592.226686	416.1615587	575.0768381
ZNF280A	0	0	0	0	10.07606311	0
MUC15	0	0	0	0	0	0
CT55	0	0	0	0	2.015212623	0
ZEB2	431.709658	419.498131	0	0	20.15212623	4.530304381
GJB1	0	0	1.002934331	0	0	7.248487009
PCBP1	10888.4599	10050.6877	7119.830814	7815.29638	10202.0139	8008.672085
HINT1	4200.86706	4430.63159	5054.789027	4927.93106	3315.024764	3547.22833
DTWD2	116.229523	189.942253	532.5581296	389.205983	637.814795	371.4849592
VPREB1	0	0	0	0	0	0
CLIC3	196.320455	120.872343	167.4900332	863.896485	1410.648836	325.2758545
INO80E	1201.36398	1093.94549	3359.830008	2659.07137	2329.585792	2790.667499
BNC1	0.97671868	0	0	0	6.045637868	0
DFFB	126.973429	134.076884	221.6484871	235.33385	208.5745064	307.154637

NFU1	701.284015	723.202588	648.898512	530.004014	502.7955493	386.8879941
ANTXR1	3850.22505	3319.41862	128.3755943	197.117242	284.1449798	387.794055
GKN1	0.97671868	1.01573397	0	0	0	0
CKAP2L	4395.23408	3935.96914	376.100374	851.828083	738.5754262	825.4214582
C15orf40	393.61763	401.214919	580.3278918	322.829769	491.7118799	362.8955021
FAM103A1	581.714114	611.360121	278.8157439	270.533358	710.3624495	292.9929055
PROKR1	10.7439055	12.1888077	0	0	0	0
APLF	252.970139	271.200971	4.011737323	0	9.068456802	9.966669638
BOLA2B	315.763383	335.872753	411.8349242	449.588224	1280.022754	707.8691201
RGPD8	381.643059	346.568431	95.5997004	85.7158294	362.254621	125.8156133
HIC2	211.977256	199.744086	202.1715024	203.684465	231.3464091	358.7910464
LUZP1	2231.80219	2331.10947	522.5287863	742.206759	1074.108328	1130.763973
HEXDC	565.520118	438.797076	648.898512	560.17502	358.7078468	467.5274121
BCRP6	0	0	0	0	1.007606311	0
BCRP2	29.6238777	25.5660241	28.18245469	6.5471084	37.07991226	35.03737408
DRD5	0	0	0	0	0	0
BUB1	7375.20278	7176.16051	545.5962759	1300.37038	2519.015778	1931.721788
SPNS1	493.291771	632.599118	1789.906812	3644.1849	1519.510622	2108.86575
LRRC45	829.234163	732.344194	953.7905485	1078.11063	1413.671655	1496.812567
CHRNA5	371.1531	505.835518	15.04401496	55.3135119	619.6778814	83.35760061
MT1B	0	0	0	0	0	0
STRA13	1049.97259	965.963008	1860.443183	1718.74167	2179.452451	1978.836954
AGPAT2	1215.03804	1326.54857	1422.160881	2091.85645	1400.572773	1603.727751
ASPSCR1	906.394939	685.620431	1426.172618	1374.79219	935.0586569	1259.424618
GP9	0	0	0	0	0	0
FASN	17300.618	21654.4326	19813.97064	130052.123	38579.23045	33263.30689
CNBP	6412.15816	5700.29905	5259.38763	4066.04597	10131.48146	6153.05941
MT1E	1773.72113	1983.72845	6.017605984	3.01710065	133.0040331	86.98184411
ACTRT2	0	0	0	0	0	0
DUS1L	1823.53378	1790.73899	2599.605785	1594.03484	3859.132172	3053.425153
GPS1	2814.90325	3021.80857	2484.268337	1964.13252	4066.699072	3238.261571
RFNG	841.931505	816.650114	995.9137904	775.394866	705.3244179	772.8699274
DCXR	861.465879	848.137867	2958.656276	2574.59255	1808.653329	3076.982736

ZNF32	245.15639	352.459688	855.5029841	595.374528	448.3848085	487.4607514
LDB2	0	0	0	0	5.038031556	0
RAC3	259.80717	207.20973	1172.430233	899.095993	394.981674	1391.709506
NRG4	0	0	0	1.00570022	0	0
LIMS1	2314.80375	2142.70097	715.0921778	1051.80151	1764.933291	898.6583588
TMEM266	0.97671868	0	2.005868661	1.00570022	4.030425245	2.718182629
NLGN1	44.9290595	75.1643139	12.03521197	4.02280086	9.068456802	5.436365257
TAPT1	562.589962	501.772582	968.8345635	894.067492	468.5369347	847.1669192
PRYP3	0	0	0	0	0	0
UGP2	2866.66934	3014.69843	2627.687946	1871.6081	2820.290065	1822.994483
TAS2R1	0	0	0	0	0	0
LINGO1	0	0	0	0	410.0957687	86.07578324
PRY	0	0	0	0	0	0
RBMY1F	0	0	0	0	0	0
PRY2	0	0	0	0	0	0
RBMY1HP	0	0	0	0	0	0
HNRNPF	9377.47608	10136.0093	10477.65495	12572.2584	11803.10033	10570.10618
BTD	560.636525	643.975338	186.5457855	255.447855	233.7646642	297.1879674
CSGALNACT2	1855.7655	2005.05886	379.109177	359.034977	402.0349182	445.7819511
TACR3	0	0	0	1.00570022	3.022818934	2.718182629
GSX1	0	0	0	0	0	0
TSPY14P	0	0	0	0	0	0
PCDH7	242.226234	280.342576	28.08216126	74.421816	246.8635463	76.1091136
ROBO1	6.83703079	8.12587178	972.8463008	1675.49656	2555.289605	1683.461108
ONECUT1	19.5343737	19.2989455	8.023474646	5.02850108	18.1369136	1.812121752
AVEN	391.664192	360.58556	123.3609227	234.32815	510.8563998	223.7970364
P2RY1	13.6740616	12.1888077	6.017605984	9.05130194	14.10648836	8.154547886
CTNND2	0	0	1277.738337	2027.49163	1066.047477	1162.476104
TRIM56	3330.61071	3033.99738	1091.192552	899.095993	3798.675794	1464.194376
MUC17	0	0	0	3.01710065	0	0
AHSP	0	0	0	0	0	0
WNT10B	104.508899	118.840875	29.08509559	23.131105	73.55526072	86.98184411
CALML6	7.81374947	4.06293589	19.05575228	60.3420129	11.08366942	19.93333928

REPS2	484.452467	554.590749	292.8568246	723.098455	267.0156725	454.8425598
MUC3A	1.95343737	0	0	0	32.24340196	13.59091314
SYAP1	2743.60278	2874.52714	3248.504297	2562.52415	4402.231974	2676.503828
ITGAM	0	0	0	0	66.50201655	8.154547886
PYDC1	0	0	0	0	0	0
TPST1	331.107634	485.520839	10.02934331	19.1083041	169.2778603	36.24243505
TM4SF4	0	0	2.005868661	0	0	2.718182629
TOR1AIP2	4189.14644	4133.02153	2395.007182	2600.74076	2225.802342	3887.001159
S100G	0	0	0	0	0	0
TM4SF1	2806.11278	3763.29437	4265.479709	5257.80073	105.7986627	2661.100793
OTUD3	489.336061	470.284829	765.2388943	757.292262	577.3584164	986.7002942
OTUD7A	3.98501223	11.8536155	66.81548511	20.3453154	30.56069942	34.10413138
GUSB	2086.08553	2076.01804	1297.797024	2095.87925	1562.797389	2231.627938
BRD3	1597.91177	1339.75311	2079.082868	1760.98108	1684.717752	1948.030884
KLF13	1031.41493	1505.31775	745.1802077	660.745042	543.0998018	661.4244396
FRMPD4	0.97671868	7.11013781	0	0	0	0
ZFPM2	14.6507803	12.1888077	14.04108063	33.1881071	4.030425245	44.39698293
ZNF764	190.723857	181.816381	673.9718702	592.357427	424.4944629	860.7578324
HSFY2	0	0	0	0	0	0
ZNF747	189.932715	147.281426	1168.558906	1227.21575	258.954822	1022.036668
ZNF768	950.347279	1051.28466	2962.577749	3479.65235	2880.746444	3153.091849
TAS1R3	1.95343737	4.06293589	41.12030756	29.1653063	4.030425245	32.61819154
TMEM42	224.645297	182.832115	740.1655361	449.547996	117.8899384	324.3969755
MAP3K2	2502.35327	2488.54823	1500.389759	1849.4827	2988.560319	2198.103686
PUSL1	471.755124	464.190425	644.8867746	639.625337	565.2671406	935.9608851
SF3B5	1277.54804	1437.26357	1559.562884	1296.34758	1860.041251	1215.027635
ZNF35	259.80717	250.886291	280.8216126	273.550459	169.2778603	396.8546638
TIGD4	0.97671868	4.06293589	7.020540315	3.01710065	7.053244179	5.436365257
IFFO2	1681.43098	1397.15224	1193.441707	920.125184	726.4841504	1705.152205
NLGN2	2711.37107	2726.22998	650.9043806	940.329702	1564.812601	1455.133767
MYO7B	104.508899	106.652067	0	0	3.022818934	3.624243505
CHD3	6500.06284	5601.77286	1992.830515	2491.11943	8396.383392	3020.806961
TMEM154	305.712948	504.819784	465.3615294	110.627024	271.0460977	267.2879585

MYRIP	0	0	201.5898005	239.356651	23.17494516	248.2606801
ALCAM	11131.6628	9725.65278	12288.95435	7627.23043	1944.680181	12159.33696
YWHAG	5989.23897	5910.55598	4603.468578	3757.29601	8709.748955	6666.795927
UBE2E3	1921.20565	2037.56235	266.780532	225.276848	1124.488643	202.0515754
CNTROB	1917.29878	1738.93656	288.8450872	523.969812	882.6631287	588.0335086
TRAPPC1	1566.65677	1702.37014	538.5757356	592.357427	1253.462251	623.3698828
ZPLD1	4.88359342	5.07866986	0	1.00570022	7.053244179	5.436365257
KCNAB3	62.5099958	44.6922948	16.04694929	17.0767897	30.22818934	18.12121752
SERPINA9	0.97671868	1.01573397	0	0	0	0
FAM153A	0	0	0	0	1.007606311	2.790667499
GPR37L1	0.97671868	0	1.002934331	9.05130194	810.1154743	107.8212443
SIMC1	681.651969	642.238433	4.011737323	1.00570022	597.9135851	58.41374469
TMEM192	607.519021	702.887909	2003.862793	2037.54864	1395.534741	1215.027635
RP11-423H2.1	637.553121	609.552114	1445.438987	1324.21553	1384.43092	1324.325758
HMP19	1.95343737	0	2.005868661	0	0	6.342426133
SPDYE5	1.90460143	0	1.494372153	1.00570022	0	0
SERPINA6	0.97671868	1.01573397	0	0	0	0
ZNF778	210.971236	203.146794	482.4114131	622.528433	2133.102561	891.5639022
NIPA1	1183.78304	1206.69196	633.854497	536.038215	1320.971874	582.5971434
FOXD4	24.7598186	20.6701863	61.80081346	21.2303316	11.32549494	39.25055716
GPR25	0	0	0	1.00570022	0	1.812121752
UBE2E1	2425.19249	2173.6707	1668.882726	867.919286	2131.087348	1421.609515
HNRNPA3	13936.4864	12795.211	13221.66322	9658.66441	16915.56377	11829.37677
SIK2	1765.90738	1780.58165	903.643832	1226.95426	754.6971272	1178.7852
RP11-374M1.7	0	0	0	0	0	0
RNF150	23.4412484	20.3146794	5.014671654	2.01140043	191.4451991	24.46364366
CCDC144A	20.179008	10.3300145	3.921473233	4.686563	23.64852013	2.038636971
RP11-262H14.4	123.066554	125.747866	48.7726965	48.6054914	121.7289185	48.80043879
VGLL2	14.6507803	7.11013781	0	0	0	0
CR848007.2	0.97671868	2.61043631	0	0	0	0
HOXD4	125.23487	78.6787535	0	0	0	0
CHRNB1	1765.6632	2087.9326	100.2934331	89.5073192	117.8899384	88.79396587
HOXD12	0	3.04720192	0	0	1.007606311	0

GYPA	0	0	0	2.01140043	0	0
USP38	911.278532	1022.84411	1955.721945	2284.95089	1433.823781	2317.703721
SLC16A5	343.804977	364.648496	1302.811696	1769.02668	11.08366942	897.9063283
NANP	829.234163	808.524242	465.3615294	503.855808	494.7346988	437.6274032
ANKK1	0	1.01573397	52.1525852	99.5643213	0	63.42426133
ADRA1B	45.9057781	35.550689	0	0	7.053244179	0.906060876
ADPRM	153.344833	156.423032	36.10563591	39.2223084	39.29664614	33.52425242
FABP6	4.88359342	0	1.002934331	2.01140043	1.007606311	11.77879139
PWWP2A	674.912611	776.020755	465.3615294	509.890009	734.5450009	531.8577343
USP50	0	1.01573397	1.002934331	2.01140043	2.015212623	0.906060876
USP47	2442.77343	2367.67589	1396.084588	1454.24251	1721.999186	1749.603552
PDCD6IP	6870.35643	6289.65837	6181.024104	6236.02521	4127.155451	5351.621383
MRGPRX1	0	0	0	0	0	0
ZNF212	551.846056	561.700887	342.0006068	299.698664	494.7346988	394.1364811
MRAP	0.97671868	2.03146794	0	0	0	0
FAM161A	424.872627	518.024326	138.4049376	151.860733	238.8026958	178.4939926
ZNF282	1015.78743	950.726998	1138.330465	1210.86306	1683.710146	1196.000357
GLB1	1819.62691	2055.84556	2827.271878	2717.40198	1579.926696	2307.737052
C14orf142	355.525601	403.246387	561.6432252	360.040677	484.6586357	497.427421
FAXDC2	41.0221847	65.0069742	18.05281795	71.4047153	16.12170098	49.83334819
CRTAP	7237.48545	8613.42408	2187.399775	3191.08678	4138.23912	2237.064303
HSPB2	0	0	2.005868661	1.00570022	0	0
C7orf33	0	0	0	0	0	0
CNGB3	0	0	0	0	63.47919761	0
SLN	0	1.01573397	0	0	0	0
ELP5	787.235259	765.863415	279.8186783	235.33385	584.4116605	342.4910112
CMTM8	104.508899	119.856609	543.5904072	504.861508	92.69978064	472.9637774
GABARAP	2786.57841	3088.84701	2866.386317	2737.51599	1768.349076	1726.045969
LGALS9B	24.779353	27.059153	0	0	0	3.678607157
STX8	268.597638	298.625788	154.4518869	160.912034	223.6886011	144.9697402
CDK1	4083.66082	3214.79802	1330.893857	2518.27334	2310.441272	2902.112986
UBB	10173.2967	10623.4194	8305.299193	10052.9794	8685.566403	10226.70005
NFRKB	1560.79646	1357.02059	1396.084588	1289.30768	1022.720406	1764.100526

FABP4	3.90687474	23.3618814	0	0	0	0
FRMPD2	0	0	0	0	10.07606311	1.812121752
PRDM10	638.774019	500.756848	397.161995	610.460031	549.1454397	581.6910825
B3GNT2	771.60776	1002.52943	807.3621362	982.569111	831.2752068	858.0396497
FOS	266.644201	606.393181	338.9918038	4635.27229	62.4715913	457.5607425
TMED10	7169.11514	7625.11493	12735.26013	9132.76366	7623.549351	9814.451411
OR2A20P	78.3914416	48.1457903	73.01361928	33.9926673	2.730613104	61.72086689
SETMAR	140.64749	111.730737	150.4401496	144.820831	303.2894997	218.3606712
SMAD1	131.857022	137.124086	306.8979052	404.291487	278.0993419	482.930447
CST5	2.93015605	1.01573397	0	0	0	54.36365257
CST2	19.0948503	10.2081264	0	0	1.007606311	69.76668747
EMX2	0	0	0	0	0	0
CST1	170.076024	111.761209	1.002934331	0	2.015212623	13.78118593
SP7	0	0	0	0	0	0
TCAF2	128.282232	107.881105	153.5893634	320.385918	144.3093759	178.6299017
SEMA3E	64.7955175	56.8811024	0	0	49.37270925	22.6515219
LRRN2	0	0	3.008802992	2.01140043	312.3579565	34.43031329
SLC30A1	998.206495	1242.24265	809.3680049	1357.69529	926.9978064	1460.570132
DCLK2	210.971236	262.059365	4.011737323	23.131105	30.22818934	9.966669638
ZNF804A	347.711851	332.145009	0	0	24.18255147	18.12121752
CTA-313A17.2	0	0	0	0	0	0
GPRC5C	65.4401518	111.730737	2592.585245	2676.16827	1200.059117	1475.067106
TMEM182	68.3703079	78.2115159	45.13204488	27.1539058	131.9964268	100.5727573
VSTM2A	0	0	0	0	0	0
KRT8	10976.8822	8816.0427	16881.72162	21075.8862	285019.9448	25288.34933
KRT78	0.97671868	0	0	0	0	0
ADORA2B	1929.0194	2462.13915	0	0	129.9812142	65.23638308
SDR9C7	0.97671868	0	0	0	0	0.906060876
MGMT	0	0	426.2470906	394.234484	589.4496921	309.8728197
METTL7B	15.6274989	29.4562852	26.0762926	13.0741028	163.2322224	40.77273943
KRT86	64.3559941	66.6524633	6.408750373	2.01140043	60.73850844	18.56518735
HARS	2849.0884	2712.00971	2152.297074	1465.30521	2830.184759	2317.703721
NFXL1	476.638718	443.875746	480.4055444	395.240185	616.6550625	569.9122911

KRT75	0	0	0	0	0	0.906060876
DENND5B	1350.80194	1153.87379	498.4583624	816.628575	228.7266327	869.8184411
CD14	350.642008	278.311108	4.011737323	3.01710065	23.17494516	10.87273051
DNAJC18	416.082159	512.945656	12.03521197	36.2052078	95.72259957	64.33032221
KRT6C	0	0	0	0	0	0
C14orf169	1173.03914	1261.54159	540.5816043	659.739341	947.1499326	797.333571
SPATA24	28.3248418	49.7709646	6.017605984	12.0684026	31.23579565	5.436365257
RALGAPB	3571.86023	3542.8801	4870.24911	4637.28369	2547.228755	5207.131855
PYM1	249.063264	355.50689	736.1537987	981.56341	977.3781219	642.3971612
MZB1	0.97671868	0	652.9102493	343.949474	4.030425245	232.8576452
KRT4	0	0	5.014671654	19.1083041	14.10648836	1.812121752
SLC23A1	0.97671868	0	39.1144389	18.1026039	0	21.74546103
KRT74	0	0	0	0	0	1.812121752
NPAS2	2383.19359	3020.79283	14.04108063	10.0570022	357.7002405	111.4454878
KRT72	0	0	0	0	1.007606311	2.718182629
KISS1	2177.10595	1280.84054	21.06162094	22.1254047	4.030425245	66.14244396
LONRF2	42.9756221	30.4720192	0	0	3275.728118	103.2909399
NUDT9	395.571067	447.938682	615.8016791	582.300425	566.2747469	540.9183431
HSD17B13	4.88359342	2.03146794	0	0	3.022818934	0
PA2G4	5945.28663	5484.95329	3539.325165	2958.75998	9597.429963	5183.565212
COX7B2	0	0	0	0	2.015212623	6.342426133
ELOVL6	2727.97528	3185.34174	642.880906	3933.29354	889.7163729	708.5396052
KRT83	0.98648587	0	2.005868661	1.00570022	73.12199001	21.74546103
PFKFB3	3055.17604	2874.52714	3683.777797	1260.14237	1865.079282	2766.203855
TMC7	127.950148	143.21849	124.363857	358.029277	142.0724899	121.4121574
ARL6IP1	5237.86882	5609.61432	6312.468677	9456.58907	7498.47518	7497.572205
SERPINB9	1697.53707	1211.77063	0	0	36.27382721	16.30909577
SMAGP	725.701982	699.840707	41.12030756	61.3477131	520.9324629	147.6879228
IRX1	39.0687474	51.8024326	0	0	0	0
CDH2	14.6507803	6.09440383	22.06455528	265.504857	1.007606311	171.2455056
IRX2	0	0	467.4576622	361.046377	821.1991437	320.7455502
EMB	984.483597	1124.48861	499.4612967	808.582973	1643.476426	492.8155712
SIX2	210.971236	204.162528	2126.220781	3958.43605	28.21297672	2047.69758

DLGAP1	0	0	0	1.00570022	1.007606311	0.906060876
STAT2	1555.91286	1782.61312	1346.940806	1612.13745	750.6667019	2123.806694
NUDCD2	1122.24977	1094.96122	594.7400581	712.035753	741.5982451	645.1153438
LINC00266-3	0	0	0	0	0	0
IRF2BP1	1196.48039	1286.93494	1431.18729	1507.54462	1098.290879	1483.221654
OR9K2	0	0	0	0	0	0
HSPA4	8233.7385	9019.71767	4118.048362	3932.28784	6001.30319	5436.365257
FOXA3	50.7893716	62.9755063	0	5.02850108	5.038031556	2.718182629
FAM71B	0	0	0	0	0	0
SLC26A5	0	0	0	0	0	0
COMMD5	671.005736	769.926351	735.1508644	1037.88262	694.2407485	838.1063105
SGCD	0	0	5.014671654	0	0	0
GTSF1	0	0	0	0	0	0
DPY19L2P2	124.043273	137.124086	0	1.00570022	12.22226456	9.558942244
ZNF16	190.460143	157.438766	466.3644638	432.451093	248.8787589	379.6395071
ARMC10	959.05961	966.694336	363.9849273	398.156715	1771.18045	630.5730668
RNF34	1354.70881	1224.97517	596.7459268	644.653838	1282.682834	902.4366327
ACYP2	155.298271	192.989455	207.6074065	299.698664	128.9736078	180.3061144
TRABD	1201.36398	1123.40177	2027.933217	1584.98354	923.9749875	1793.094474
TMEM133	132.833741	165.564637	13.0381463	17.0969037	18.1369136	6.342426133
ATF7	752.659418	719.139652	802.3474646	981.56341	1335.662774	892.7508419
RASA4B	7.74537916	14.3320063	5.255375893	12.9936468	80.03416931	30.94197892
SOCS6	803.839477	673.431624	948.7758769	872.947787	499.7727304	752.0305272
MURC	42.9756221	33.5192211	7.020540315	10.0570022	14.10648836	7.248487009
OR10A3	0	0	0	0	0	0
ZNF296	157.251708	169.627573	691.0217539	711.030052	247.8711526	516.4546994
OR5E1P	0	0	0	0	0	0
HOXB9	484.452467	406.293589	0	0	34.25861458	12.68485227
TTLL6	5.8603121	2.03146794	1.002934331	2.01140043	2.015212623	1.812121752
POLH	1470.93834	1176.21994	720.1068494	1200.80606	780.8948912	1023.84879
SYT9	0	0	1.002934331	0	2.015212623	0
KCNS3	607.519021	577.95263	465.3615294	418.37129	230.7418453	423.1304292
RBMXL2	1.95343737	0	0	0	0	0

KIF5B	12543.9981	12167.4773	6939.302634	6350.99686	10844.86673	6333.365524
GPR37	6.83703079	15.2360096	78.2288778	72.4104155	96.73020588	55.26971345
AKAP13	2808.06622	2564.72828	528.5463923	723.098455	2782.001025	1015.694242
TPD52L3	0	0	0	0	0	0
CDCA4	2470.12155	2450.96608	459.3439235	577.271924	2048.463631	890.6578413
OR10A4	0	0	0	0	0	0
SDR16C5	0	0	12.03521197	5.02850108	4.030425245	3.624243505
DYDC1	0	0	0	0	0	0
OR10A2	0	0	0	0	0	0
CHCHD7	224.645297	237.68175	258.7570573	172.980437	268.0232788	203.8636971
HTRA3	161.158583	136.108352	0	0	3.022818934	0
FOXN2	1009.92712	1049.25319	323.9477888	625.545534	624.715913	456.6546816
OR2AG1	0	0	0	0	0	0
LMOD2	0	0	0	0	0	0
BFSP2	0	0	1.002934331	0	0	0
FSHR	0	0	0	0	0	0
CELP	1.95343737	0	1.002934331	1.00570022	0	2.881273586
USP32	3376.25278	3329.71816	1737.694051	3250.25213	18002.06565	3684.75025
CEL	35.1618726	31.4877531	12.03521197	14.079803	18.1369136	39.70358759
PPM1D	522.544496	504.819784	518.517049	810.594374	8084.025435	1198.718539
GPR27	0	0	0	0	506.8259746	16.30909577
AC093323.3	237.34264	207.20973	650.9043806	473.684802	721.4461189	704.0093008
PSG6	4.88359342	0	0	0	0	0
KBTBD2	1503.17005	1570.32472	1181.456642	1242.03977	1828.805455	1239.491279
MINA	1569.58692	1543.91564	900.635029	612.471431	910.8761054	1007.539694
TRIAP1	690.540109	766.879149	433.2676309	390.211684	1125.49625	671.3911092
LILRP2	0	0	0	0	0	0
LSM3	1229.68882	1323.50137	1795.252452	1558.83533	1892.284653	1757.7581
KIAA0232	995.276339	940.569658	1654.841646	1540.73273	1591.010366	1758.664161
MTSS1	2.93015605	19.2989455	89.26115543	58.3306125	1823.767423	271.8182629
TMEM43	4142.26394	3638.34893	1082.015703	1096.92728	2811.221608	1387.179201
RNF139	383.850443	394.104781	1907.581097	1545.76123	2827.343309	1696.14596
RPS9	7477.74848	7417.88488	8648.292704	9302.70688	8672.820183	6085.965602

PLA2G1B	0	1.01573397	0	0	0	0
CYTL1	0	0	0	0	0	0
TSEN34	1675.07254	1831.36835	1181.456642	1874.6252	2194.566546	1931.721788
TRH	0	0	0	0	1.007606311	0
GSTA4	263.714045	261.043631	45.13204488	12.0684026	27.2053704	20.83940015
MSANTD4	770.631042	946.664062	754.2066167	619.511333	170.2854666	585.315326
NDUFA3	503.986841	497.709646	879.573408	657.727941	654.9441023	698.5729355
OSCAR	14.6507803	16.2517436	23.06748961	40.2280086	8.06085049	22.6515219
PAQR8	238.319359	145.249958	6.017605984	22.1254047	89.6769617	22.6515219
NUDT6	180.692957	201.115327	321.9419202	199.128643	101.7682374	286.3152369
TPT1-AS1	173.855926	165.564637	452.3233831	247.402253	108.8214816	259.1334106
OR7G3	0	0	0	0	0	0
TANC2	5831.98726	3995.89745	499.4612967	625.545534	9089.616534	2082.127893
OR7G2	0	0	0	0	0	0
TEX13B	0	0	0	0	0	0
PKHD1	0	1.01573397	8.023474646	32.1824069	1.007606311	111.4454878
OR1M1	0	0	0	0	0	0
NCBP2L	0	0	1.002934331	7.03990151	2.015212623	0
DNAJC24	405.338254	404.262121	167.4900332	159.906334	191.4451991	194.8030884
MBD3L1	0	0	0	0	0	0
ZNF160	826.304007	773.989287	713.0863091	698.96165	769.8112218	707.6335443
PGK2	0	0	0	0	2.015212623	0
OR8B12	0	0	0	0	0	0
ZNF415	81.0676508	76.1800479	0	1.00570022	2.015212623	0.906060876
PRKCDBP	1744.41957	2221.4102	0	0	41.31185876	18.12121752
CEACAM3	0	0	0	0	0	0
DCDC1	2.93015605	0	1.002934331	0	1.007606311	1.812121752
HAS2	1457.26428	1113.24443	0	2.01140043	3.022818934	0
PDGFD	12.6973429	34.5349551	7.020540315	34.1938073	14.10648836	45.30304381
PLAC1	20.5110924	21.3304134	742.1714047	208.179945	2.015212623	146.7818619
DDI1	0	0	0	0	0	0
LINC00208	0	0	0	0	0	0
S1PR1	45.9057781	47.7394967	0	0	1.007606311	0.906060876

HS6ST2	0	0	1168.418495	1792.15778	414.1261939	1778.5975
OR4D5	0	0	0	0	0	0
PYGO1	687.609953	656.164146	0	0	81.61611121	8.154547886
LRRC8E	567.473555	772.973553	228.6690274	248.407953	382.8903983	370.5788984
PKIA	28.3248418	37.582157	0	0	491.7118799	12.68485227
XKR6	60.5565584	39.6136249	0	0	67.50962286	11.77879139
TSNARE1	272.504513	290.499916	575.6843058	671.807744	110.8366942	376.9213245
FPR2	0	4.06293589	0	0	0	0.906060876
FPR1	65.4401518	107.667801	0	0	0	0.906060876
PATE1	0	0	0	0	0	0
OR13H1	0	0	0	0	0	0
FEZ2	1954.41409	1902.46973	533.5610639	535.032515	965.2868462	705.8214225
SOX7	19.8859924	12.6052586	4.011737323	1.90077341	15.11409467	7.067274834
C8orf74	0	0	0	0	0	0
C11orf24	1995.43627	2115.77386	1339.920266	1028.83132	1184.945022	1473.254985
FAM86JP	49.0019764	66.0227082	57.16725685	54.4385527	145.6998726	103.7711521
ALK	0	0	0	0	0	0.906060876
CCBL1	319.38701	293.547118	122.3579883	105.598523	54.41074081	104.1970008
MTM1	338.921383	351.443954	934.7347962	925.244198	648.8984645	810.9244842
SIGLEC17P	0	0	0	0	0	0
OBP2B	0	0	0	0	0	0
TRMT61B	274.45795	376.837304	330.9683291	235.33385	392.9664614	300.8122109
INSR	1488.51927	1510.39642	448.3116458	418.37129	464.5065095	553.6031953
MFN1	1589.1213	1783.62886	2207.458462	2643.98587	1546.675688	2882.179647
GIMAP8	1.95343737	9.14160575	0	0	0	0
HSFX1	13.6740616	5.99283044	150.4401496	71.5857413	34.25861458	72.48487009
NRTN	51.7660902	43.6765608	8.023474646	11.0627024	38.28903983	12.68485227
KCNMB3	35.9627819	48.5927132	63.66627131	53.4429095	287.9839598	99.5851509
FUT3	0	2.03146794	549.9389816	56.8321192	10.07606311	276.747234
KCNG3	7.81374947	2.03146794	3.008802992	0	37.28143352	6.342426133
ATP6V0E2	1118.34289	1219.8965	1977.7865	2506.20494	1700.839453	2488.043166
PRKCE	1170.10898	1125.43324	113.3315794	140.79803	524.9628882	194.8030884
OR2K2	0	2.03146794	0	0	0	0

JAGN1	718.864951	728.281258	789.3093183	711.030052	1093.252848	549.9789518
RLN3	0.97671868	0	0	0	0	0
TADA3	3391.16727	3451.46404	1801.270058	2379.48671	2605.669921	2285.991591
SOCS5	1034.34509	951.742732	674.9748046	801.543072	620.6854878	849.8851019
C1GALT1C1	801.886039	792.272498	588.7224521	534.026814	770.8188281	301.7182718
C9orf16	545.009026	635.849467	367.073965	574.254823	965.2868462	626.0880654
MORN4	210.971236	292.531384	171.5017706	78.4446168	172.3006792	103.2909399
ZNF672	853.65213	901.971767	1810.296467	2785.7896	897.7772234	1637.252003
ZNF692	529.381527	497.709646	1811.299401	1657.39396	448.3848085	2014.173328
NAIF1	276.411388	238.697483	346.0123441	281.59606	275.076523	296.2819065
RBKS	129.903585	80.2429838	113.3315794	70.3990151	83.63132384	115.9757922
OR2M4	0.97671868	0	0	0	2.015212623	0
GRIK1	0	0	0	0	0	0
MUC7	0	0	0	0	0	0
PROL1	0	0	0	0	0	0
SMR3B	0	0	0	0	0	0
TMEM126A	451.244032	481.457903	355.0387531	275.561859	344.6013585	298.0940283
TMEM126B	958.161029	1128.48044	780.2829093	949.381003	867.549034	674.1092919
TRIM8	6205.0938	6438.73765	2077.076999	2116.99895	1776.409927	2372.973435
NETO2	1.95343737	2.03146794	2877.418595	2709.35638	1150.686407	3114.131231
CSN3	0	0	0	0	0	0
CLDN20	0	0	0	0	0	0
CDC42BPG	145.531084	107.667801	1909.586966	2577.60965	1757.265407	3067.016066
SCAND1	1208.20101	1266.62026	2382.97197	1784.11218	1158.747258	1382.648897
JUNB	934.71978	1090.89829	4900.33714	12181.041	2515.992959	6217.389732
C10orf35	115.252805	115.793673	235.6895677	209.185645	292.2058303	238.2940104
TMEM37	1.95343737	7.11013781	25.07335827	25.1425054	59.44877237	40.77273943
UGT2B7	0	0	0	0	0	0
LRG1	9.76718684	12.1888077	0	0	106.806269	42.58486118
SHCBP1	2020.83096	1891.29666	728.1303241	1170.63505	2322.532548	1749.603552
SOSTDC1	0	0	0	0	0	0
NPTX1	0	1.01573397	2.005868661	0	43.32707139	11.77879139
FAM98B	1070.48368	1023.85984	788.3063839	869.930686	1245.401401	892.469963

ZNF439	421.522482	443.042844	2.005868661	6.03420129	5.038031556	7.248487009
ZNF440	460.454489	464.545932	298.8744306	313.778467	305.3047123	395.9486029
GAA	1418.19553	1373.27233	5845.101279	4108.28538	3094.358982	4934.407532
CANT1	3729.11193	3874.00937	4312.617622	3568.22436	3817.820313	4378.086154
KCNK3	0.97671868	4.06293589	1.002934331	0	33.25100827	4.530304381
ZDHHC16	1612.56255	1934.97322	1538.501263	1342.60979	1023.728012	992.1366594
CHST11	3497.62961	3500.21927	893.6144887	1106.27024	281.1221608	1129.857913
EXOSC1	781.374947	762.816213	791.3151869	474.690502	536.0465576	564.4759259
PGAM1	18526.7809	16609.028	5628.52764	5658.69295	12739.05576	7916.127027
CHD7	992.346183	826.807453	551.6138819	604.42583	2194.566546	877.972989
ESCO2	621.193083	597.251576	144.4225436	278.57896	1026.750831	530.0456126
KRT19	4927.54576	5480.90051	12410.30941	20708.3731	54887.33859	19193.08754
KRT15	51.7660902	177.753445	31.09096425	155.883533	214.6201443	77.92123535
LURAP1	3.90687474	3.04720192	3.008802992	1.00570022	5.038031556	5.436365257
KRT38	0	0	0	0	0	0
CLCN5	268.597638	574.905428	810.3709392	790.480369	484.6586357	323.4637328
TPPP	42.9756221	41.6450929	377.1033083	226.282548	23.17494516	308.9667588
KCND3	0.97671868	0	15.04401496	21.1197045	64.48680392	109.633366
APLN	341.851539	367.695698	3.008802992	1.00570022	17.12930729	6.342426133
KRTAP4-4	0	0	0	0	0	0
KRT13	0	0	1.002934331	1.00570022	156.1789782	6.342426133
XAGE3	0	0	0	0	0	0
KRT9	0	0	0	0	3.022818934	0.906060876
XAGE5	0	0	2.005868661	0	0	0.906060876
PDE7B	481.522311	336.207945	0	1.00570022	0	0.906060876
MRPL36	965.974778	910.097639	488.4290191	523.969812	756.7022637	503.7698472
ZNF581	352.19499	317.945048	449.9464288	476.701902	386.1852709	370.3252013
NAT1	82.0443694	89.3845896	86.25235244	152.866433	319.4112007	125.9424618
KRT20	0	0	0	1.00570022	7.053244179	1.812121752
GLOD5	0	0	7.020540315	10.0570022	10.07606311	3.624243505
KSR2	0	0	245.718911	322.829769	538.0617702	384.1698115
ZNF524	195.343737	230.571612	635.8603657	724.104155	143.0800962	372.3910201
MCC	227.575453	239.713217	249.7306483	501.844408	341.5785395	1349.124645

KRT27	1.95343737	0	0	0	0	0.906060876
ZBTB26	276.411388	252.917759	442.2940398	356.017876	572.3203848	324.3697937
CDK5R2	53.7195276	62.9755063	37.10857024	14.079803	10.07606311	27.18182629
DSEL	222.69186	250.886291	63.18486283	123.701127	24.18255147	66.14244396
POLR1C	874.163222	994.403559	404.1825353	256.453555	593.4801173	597.0941174
ASXL1	8892.0469	7525.573	1720.032377	2588.67235	1823.767423	3123.19184
OR1L6	0	0	0	0	0	0
DLK2	417.058878	269.169503	0	2.01140043	31.23579565	6.342426133
ZNF562	1270.28125	1262.55733	875.5616707	753.651628	941.1042947	1142.533704
ZNF318	1955.39081	2192.96965	1048.066376	1786.12358	1285.705653	1676.212621
ZNF561	518.090659	556.622217	631.8486283	757.915796	557.2062901	893.3760239
WIPF2	2213.24454	2232.58327	959.8081545	865.907886	1506.371435	906.9669371
HOPX	0	0	9.026408976	7.03990151	2.015212623	29.90000891
SPACA5B	0	0	0	0	0	0
OR1L3	0	0	0	0	0	0
SSX6	0	0	0	0	0	0
OR1B1	0	0	0	0	0	0.453030438
NLRP5	0	0	0	0	0	0
LRRC8C	886.997306	731.32846	36.10563591	38.2166082	103.1587341	102.384879
SPACA5	0	1.01573397	1.002934331	2.01140043	0	1.812121752
RSL1D1	4279.98127	4003.00758	4809.070116	3329.87341	6892.027169	5582.241058
LRRC8D	1250.84455	1132.54338	2232.53182	809.588674	774.5268194	1265.767044
MROH2B	0	0	0	0	0	0
OR1L8	0.97671868	0	0	0	0	0
PPID	806.769633	955.805668	925.7083872	627.556935	990.477004	954.0821026
OR1N2	0	0	0	0	0	0
COL24A1	0.97671868	1.01573397	8.023474646	2.01140043	2.015212623	19.93333928
ETFDH	255.900295	202.13106	505.4789027	465.6392	287.1677987	549.072891
OR1N1	0	0	0	0	0	0
RXFP1	0	0	0	0	0	0
LPAR3	1.95343737	1.01573397	15.04401496	6.03420129	5.038031556	10.87273051
PTGER4	379.943568	431.686938	147.4313466	245.390853	65.49441023	153.1242881
TBCA	2738.71919	2813.5831	2752.051803	2448.88002	2304.395634	1883.700562

NEUROD2	0	2.03146794	2.005868661	1.00570022	0	0.906060876
MAP6	34.1851539	49.7709646	0	0	22.16733885	1.812121752
OTP	0	0	4.011737323	1.00570022	11.08366942	5.436365257
ECEL1	0	0	0	0	31.23579565	3.624243505
BCL2L1	2796.9682	10318.8414	3926.487905	15160.2478	2305.40324	5673.753207
FGG	0	0	0	0	1.007606311	0
FGA	0	0	0	0	0	0
OR2AT4	0	1.01573397	0	0	0	0
FGB	0	0	0	0	1.007606311	2.718182629
PLRG1	1714.14129	2397.13217	1318.858645	1532.68713	1113.404974	1260.330679
RAB4B-EGLN2	43.7862986	17.2877922	23.54889809	37.130452	11.38595132	18.9276117
ZNF584	650.494643	716.09245	248.727714	447.536596	320.418807	327.0879763
DSCAM	0	1.01573397	0	0	2181.467664	181.2121752
DNAI2	0	0	0	0	0	0
NMUR1	0	0	0	0	0	0.906060876
CLSTN1	6325.2302	6670.325	6284.386516	7670.47554	4525.159944	12222.76122
CXXC5	399.477942	439.81281	1905.575228	2042.57714	5015.864218	1778.5975
ZNF274	1273.64116	1299.12375	1518.442577	1143.48115	746.6362767	1676.212621
PIK3CD	1425.03256	1494.14467	8.023474646	9.05130194	129.9812142	59.80001783
PTCRA	0	0	0	0	0	0
SLC25A33	450.413821	547.419667	611.0277116	670.560676	325.7893486	657.8183173
ENC1	6974.74812	6638.83724	259.7599917	322.829769	553.1758649	1111.736695
SPSB1	562.589962	591.157172	15.04401496	51.290711	359.7154531	85.16972236
P2RY6	97.6718684	136.108352	563.6490939	1041.90542	989.4693977	905.1548153
BPTF	3055.83044	3471.88029	10719.14148	12320.5719	11083.04471	13268.98971
S100Z	0	0	0	0	0	0
ZIK1	0.97671868	0	0	0	0	0
GPR82	0	0	0	0	0	0
RP11-443P15.2	17.5809363	66.0227082	0	0	8.06085049	6.342426133
GPR34	0	0	0	0	0	0
SHANK2-AS3	0	0	0	0	0	0
PLEKHG5	252.970139	298.625788	55.16138819	143.815131	303.2894997	155.8424707
ATF7IP	2397.84437	2277.27557	3608.557722	2866.24561	2195.574152	3425.816173

LKAAEAR1	0	0	0	0	3.022818934	0
RGS19	464.918094	370.7429	319.9360515	365.069178	955.2107831	379.6395071
TCEA2	921.045719	604.361713	980.8697754	790.480369	803.0622301	627.9001872
DEFB4A	0	0	0	0	9.068456802	1.812121752
ANO5	0	0	0	0	0	0
HDAC3	2570.72358	2572.85415	1061.104522	1069.05933	1243.386188	1734.200517
C1orf111	0	0	0	0	0	0
GPHN	835.094475	776.020755	918.6878469	1004.69452	608.594212	1125.327608
VAT1L	0	0	0	0	56.42595343	4.530304381
TMEM51	361.385913	486.536573	132.3873317	109.621323	275.076523	197.521271
CAMTA1	428.779502	383.947442	464.3585951	541.066716	525.9704945	491.9910558
LGALS4	1.95343737	0	1.002934331	1.00570022	1.007606311	2.718182629
LRRC34	71.3004639	100.557663	0	1.00570022	3.022818934	0.906060876
PAH	0	0	15.04401496	7.03990151	15.11409467	1.812121752
SPATA5L1	351.618726	338.239413	223.6543557	281.59606	287.1677987	355.1758635
GATM	5.8603121	9.14160575	566.6578969	753.269461	7.053244179	702.197179
SYCE1	0	0	0	0	0	0
NXNL1	0	0	0	0	0	0
RASGRP4	0	0	0	0	0	0
NHLH1	0	2.03146794	14.04108063	17.0969037	2.015212623	13.59091314
SLFNL1	4.88359342	2.03146794	12.03521197	8.04560172	9.068456802	9.060608762
BCL2	543.055588	8598.26731	65.1907315	856.319212	181.369136	148.0212264
RHNO1	1026.53134	930.412319	769.2506317	1034.86552	1169.830927	1090.897295
CTPS1	2581.46748	2108.66373	752.200748	947.369603	2864.624743	1877.358135
UTF1	0	0	0	0	1.007606311	0
KNDC1	44.9290595	21.3304134	0	0	359.7154531	85.16972236
WDR87	0	1.01573397	1.002934331	0	3.022818934	0.906060876
METTL18	116.229523	118.840875	289.8480216	252.430754	287.1677987	246.4485583
CFAP46	17.5809363	10.1573397	0	0	19.14451991	4.530304381
COL8A2	58.603121	50.7866986	0	0	1.007606311	1.812121752
PWWP2B	338.921383	390.041845	1074.142668	1367.75229	1187.967841	945.0214939
PCDHB1	0	0	0	0	4.030425245	1.812121752
ZNF540	2.93015605	2.03146794	181.5311139	94.5358203	7.053244179	131.378827

ANGPTL7	0	0	0	0	0	0
FBXL14	229.528891	254.949227	590.7283208	503.855808	363.7458784	385.0758724
EXOSC10	2687.92982	2307.74758	2710.931496	2615.82626	1413.671655	2857.716003
ZNF570	150.414677	151.344362	246.7218454	310.761367	133.0040331	302.6243326
NINJ2	64.4634331	59.9283044	0	0	4.030425245	0.906060876
MLLT3	498.126529	530.213134	319.9360515	503.855808	416.1414066	317.1213067
FAM90A1	0	0	1.002934331	1.00570022	0	1.812121752
RRM2	8103.83492	7910.53618	959.8081545	3569.23006	9851.366905	6486.489813
TRAPPC12	1238.47929	1264.5888	1122.283516	887.02759	1124.488643	1176.973078
IFNB1	0	0	0	0	0	1.812121752
RPS21	6618.2458	6691.65541	4978.566018	5808.92445	12192.03637	4230.398231
C3AR1	6.83703079	20.3146794	0	0	0	0
RNMTL1	676.866048	579.984098	232.6807647	172.980437	750.6667019	358.800107
PTEN	3567.60173	3622.81836	3114.722887	3727.48705	3042.799767	3632.298386
RPS7	15945.1181	15822.0576	15281.92101	11551.4425	17889.25405	10542.41696
PRND	0	0	0	0	0	0
RNASEH1	725.701982	658.195614	567.6608312	355.012176	1258.500283	630.5096425
PRNP	10911.9011	13513.3248	1080.160274	732.149757	1242.378582	841.730554
KLF17	9.76718684	12.1888077	26.0762926	88.501619	0	96.04245287
ADRA1D	0	0	0	0	2.015212623	0
FRMD5	611.425896	643.975338	2.005868661	2.01140043	181.369136	37.14849592
AQP4	0	0	0	0	0	0
MIR31HG	0	0	0	0	0	0
CYP4F11	0	0	1.002934331	0	202.5288686	35.33637417
TLN2	1024.5779	859.310941	1667.879792	2572.58115	511.8640061	3829.919324
LGALS9C	4.52220751	6.46006806	0	1.00570022	0	5.382001604
TVP23B	1240.11041	1357.45735	1165.90113	856.454304	851.9311362	919.5340014
FBXW10	11.2029633	3.61601294	142.5370271	62.7758075	4.735749663	56.81001694
OR10H3	0	0	0	0	0	0
ZNF217	3131.3601	3018.76137	2730.990183	2673.15117	13998.67448	5738.98959
OR10H2	0	0	0	0	0	0
SRGAP2C	1880.62299	1869.66152	786.3105446	1358.73116	674.8140988	890.5128715
OR52A5	0	0	0	0	0	0

SCG2	130.880304	98.5261953	3.008802992	0	4.030425245	2.718182629
ATPAF2	269.476685	232.60308	552.7070803	346.111729	421.1794381	474.0691716
CYP4F22	0	0	1.002934331	0	13.09888205	6.342426133
FOXB1	0	0	0	0	0	0
PPIH	677.842767	563.732355	436.2764339	470.667701	622.7007004	627.9001872
DRC3	14.6507803	12.1888077	33.09683291	18.1026039	28.21297672	29.90000891
ZNF57	86.9279629	103.604865	137.8834118	113.644124	120.691084	157.2196832
C20orf196	15.6274989	30.4720192	76.22300913	93.53012	45.34228401	30.80606979
C11orf40	0	0	0	0	0	0
JMJD1C	4198.91362	3560.14757	1982.801172	1671.47376	1378.405434	2084.846076
LDHAL6B	0	0	0	0	0	2.718182629
SYNPO	740.352762	627.723595	2.005868661	2.01140043	539.0693765	141.3454967
OR52P2P	0	0	0	0	0	0
ZNF556	0.97671868	0	0	0	0	0
MAL	0	0	0	0	0	0.906060876
ZNF554	76.1840573	66.0227082	258.7570573	226.31272	53.4031345	267.2879585
RAB33B	196.320455	195.020923	366.0710307	378.143281	299.2590745	394.1364811
THOP1	2997.54964	2829.83485	1851.416774	1837.41429	1654.489563	2193.573381
ANKRD20A4	14.4847381	8.56263739	17.01979559	33.5401022	0	6.759214136
REG3A	0	0	0	0	0	0
GAP43	0	0	0	0	0	0
REG1B	0	0	0	0	0	0
EPHX4	276.411388	297.610054	5.014671654	8.04560172	68.51722917	26.27576541
LAMB2	8244.48241	6863.31445	7391.626017	10307.4215	1938.634543	8237.905486
USP19	1885.06706	1840.50996	1927.639784	2041.57144	1325.002299	2330.388574
QARS	4512.44032	4697.76962	3257.530706	4045.93197	2054.509269	4482.283154
ORMDL3	662.215268	796.335434	2869.39512	2461.95413	616.6550625	3006.309987
SERF1A	24.3007609	23.8799057	12.04524131	93.8117161	21.07912403	23.05018869
KLF11	700.307296	747.580204	353.0328844	1189.74335	227.7190264	581.6910825
LRRC15	103.53218	88.3688556	2.005868661	23.131105	151.1409467	24.46364366
SMN1	1238.80161	1111.28407	710.0975648	631.740647	1104.669027	826.862095
EIF2AK3	671.005736	856.263739	848.4824438	1228.96566	1036.826894	873.4426846
TEX37	0	0	0	0	0	0

MOB3A	3438.04977	3399.66161	612.7928761	823.668477	548.1378333	583.5032043
KRCC1	248.086546	247.839089	864.5293931	668.790643	893.7467981	435.8152814
NME6	493.242935	507.866986	366.0710307	443.513795	380.8751857	375.1092027
CYCS	3759.39021	4225.45332	2214.479002	2272.88249	4681.338922	2690.094741
CD8B	0	0	0	0	0	0
SLFN12	783.328384	722.186854	146.4284123	311.767067	26.19776409	266.3818976
CALB2	988.439308	1135.59058	0	1.00570022	83.63132384	47.11516556
SLC9C1	0	0	0	0	0	1.812121752
OR1A1	0	0	0	0	0	0
AC005255.6	0	0	0	0	0	0
OR1A2	0	0	0	0	0	0
OR8I2	0	0	0	0	0	0
LCE1D	0	0	0	0	0	0
CCL11	0	0	0	0	0	0
FRMD3	83.9978068	56.8811024	3.008802992	0	248.8787589	13.59091314
SNTB1	56.6496837	70.0856441	0	1.00570022	66.50201655	17.21515665
MTBP	929.836187	915.176309	269.789335	226.282548	307.3199249	356.9879852
TEFM	346.735133	368.711432	301.8832335	219.242647	217.6429632	279.0667499
MRPL13	1536.37849	1558.13591	1449.240108	1261.14807	1009.621524	1242.209461
MALT1	2260.12703	1950.20923	621.819285	860.879385	575.3432037	861.6638932
PRL	0	0	0	0	0	0
ISG20	162.135302	182.832115	509.49064	107.609923	238.8026958	275.4425064
HMGN1P35	0	0	0	0	0	0
OR4C11	0	0	0	0	0	0.906060876
MBOAT1	132.833741	204.162528	35.10270157	140.79803	596.5029363	137.7212532
OR8U1	0	0	0	0	0	0
ID4	0	0	2.005868661	0	268.0232788	8.154547886
OR4X2	0	0	0	0	0	0
GPR22	0	0	0	0	0	0
CXCR6	2.93015605	4.06293589	0	0	0	0.906060876
CEBPB	2538.49186	13199.562	2530.254372	16761.3151	2850.518255	2552.748888
AZU1	0.97671868	0	0	0	3.022818934	0
TPSAB1	0	1.01573397	1.002934331	0	1.007606311	0

ATOH1	0	3.04720192	1.002934331	1.00570022	0	0.906060876
PAIP1	3114.3945	3179.79583	2500.937106	3033.75504	2240.483166	2464.621492
CLEC7A	0	0	0	0	1.007606311	1.812121752
C5orf34	291.062168	280.342576	177.5193765	171.974737	151.1409467	242.8243148
C1QTNF4	0.97671868	0	13.0381463	10.0570022	0	21.74546103
SERHL	2.93015605	1.37124086	0.501467165	0	0	4.947092384
NEGR1	340.874821	270.185237	0	0	37.28143352	8.154547886
ZNF131	1768.83754	1650.5677	881.5792767	896.078892	875.6098845	1307.445844
MACROD2	2.93015605	5.07866986	118.346251	135.769529	114.8671195	173.0576274
DPAGT1	1518.79755	1791.75473	1355.967215	1197.78896	938.0814758	1658.997464
BSG	12845.8041	13161.8808	10338.24708	9428.43952	13777.00109	11794.19443
HINFP	645.61105	603.34598	477.3967414	606.43723	418.1566192	563.569865
PRYP4	0	0	0	0	0	0
CDY1	0	0	0	0	0	0
OR10V1	0	0	0	0	0	0
CERS6	2111.66579	2129.99414	8868.948286	15664.7866	2214.718672	9424.845234
CSPG4P4Y	0	0	0	0	0	0
SPTLC3	620.216364	810.55571	11.03227764	14.079803	21.15973254	41.6788003
GOLGA2P3Y	0	0	0	0	0	0
COPRS	1567.63349	1819.17954	909.661438	1459.27101	682.1494727	814.5487277
TP53RK	745.236356	875.562684	988.8932501	978.54631	904.8304675	1002.103329
B3GALT1	21.487811	9.14160575	0	0	0	0
OR5A1	0	0	0	0	0	0
CLEC12A	0	0	0	0	0	0.906060876
OR5A2	0	0	0	0	0	0
BPGM	340.874821	339.255147	236.692502	183.037439	136.026852	210.2061233
POP7	865.372754	853.216537	385.126783	280.59036	1274.621984	462.9971077
ALG14	193.390299	213.304134	228.6690274	194.100142	87.66174908	169.4333838
SUCLG2	2563.87678	1913.62249	1851.406745	1180.682	1146.645906	1859.218797
CSPG4P3Y	0	0	0	0	0	0
STARD5	28.3248418	39.6136249	434.2705652	184.043139	97.73781219	521.8910647
CSDC2	0.97671868	0	0	0	1.007606311	0
RCAN2	13.6740616	16.2517436	10.02934331	24.1368052	2.015212623	3.624243505

IL16	0.97671868	3.04720192	7.020540315	24.1368052	8.06085049	18.12121752
ABCG4	43.9523408	66.0227082	0	2.01140043	1.007606311	3.624243505
CDY1B	0	0	0	0	0	0
GNB2	4452.86048	4352.42007	4366.776076	4692.59721	10537.5468	4739.604443
CFAP53	2.93015605	2.03146794	133.390266	84.4788181	21.15973254	108.7273051
OR5B12	0	0	0	0	0	0
OR5B2	0	0	0	0	0	0
FAM195A	320.363728	386.994643	1506.407365	1388.872	1123.481037	1389.897384
PDZD3	0	1.01573397	1.002934331	0	0	0
C2CD2L	1098.80852	1306.23389	1577.615702	1364.73519	602.5485742	1273.015531
OR9I1	0	0	0	0	0	0
ARNT2	2185.89641	1892.31239	0	0	1221.218849	164.9030795
GNG12	6754.98642	5900.39864	2197.429119	2213.54617	1426.770537	2673.785646
OR6Q1	0	0	0	0	0	0
PRSS27	27.3481231	23.3618814	192.5633915	92.5244198	125.9507889	95.136392
MYOZ2	0	0	0	0	1.007606311	0.906060876
SYNPO2	1.95343737	5.07866986	0	0	210.5897191	13.59091314
DNAJB7	0	0	0	0	0	0
CLP1	576.264023	597.251576	374.0945054	437.479594	288.175405	521.8910647
INSL5	0	0	0	2.01140043	0	0
EFCAB3	0	0	0	1.00570022	0	0
TTC36	3.90687474	9.60884338	56.16432252	46.66449	7.053244179	62.82626115
RSPH9	4.88359342	3.04720192	6.017605984	3.01710065	3.022818934	0.906060876
MYEOV2	514.730746	552.559281	577.6901745	570.232022	970.3248778	475.68196
GTPBP2	2724.06841	2536.28773	4493.145802	1245.05687	875.6098845	1531.242881
FGGY	32.2317166	34.5349551	57.16725685	84.4788181	147.1105214	82.45153973
OR9G4	0	0	0	0	0	0
IL17D	11.7206242	5.07866986	0	10.0570022	69.52483548	4.530304381
OR5AR1	0	0	0	0	0	0
PRSS30P	0	0	4.011737323	0	0	0
FUT9	0	0	732.1420614	911.164395	1312.911024	1242.209461
OR5AP2	0	0	0	0	0	0
TCEAL1	465.787373	472.214724	1037.776269	999.655957	513.6071651	496.4307541

ZNF24	2936.01636	2684.58489	4494.148736	3569.23006	2957.324524	3033.491813
HSFY1	0	0	0	0	0	0
MANEA	374.083256	389.026111	587.7195178	425.411191	419.1642255	454.8425598
RAB40A	2.93015605	1.19856609	6.328515627	1.00570022	3.466165711	0
C2orf54	0	0	1720.032377	2020.45173	65.49441023	1359.091314
AGXT	1.95343737	1.01573397	0	0	2.015212623	1.812121752
OR8J1	0	0	0	0	0	0
OR5T3	0	0	0	0	0	0
AFF1	2433.00624	2149.29309	2040.971363	2362.38981	1352.20767	3242.791876
ACOT12	0	0	0	2.01140043	0	0
FIBP	2488.67921	2874.52714	1258.682585	1341.60409	1718.976367	1871.92177
CARNS1	2.93015605	10.1573397	3.008802992	11.0627024	15.11409467	28.99394804
OR10H5	0	0	0	0	0	0
BANP	417.029576	356.50231	343.8560353	324.84117	506.6849097	278.994265
PPP1CA	6666.10502	6964.88785	5092.900531	7008.7248	8250.280477	6242.759437
HCFC1	6002.91303	5988.7675	3953.567132	5235.67532	8312.752068	7664.368952
FAM170B	0	0	0	0	0	0
CTSW	138.694053	270.185237	0	0	11.08366942	2.718182629
NIPAL4	0	0	0	0	0	0
MUCL1	0	0	2417.071737	786.457569	4.030425245	3682.231401
SNTG2	0	0	0	0	0	0
FNDC9	0.97671868	0	0	0	0	0
PDE3A	0	0	0	0	0	0
RASGRP1	9.76718684	2.03146794	9.026408976	7.03990151	1018.689981	118.6939748
KLHL6	0	0	0	0	0	0
CHCHD1	948.393842	906.034703	1063.110391	868.924986	732.5297883	767.4335621
MRPL52	1325.40725	1458.59398	736.1537987	589.340326	968.3096651	634.2426133
SMPDL3A	84.9745255	137.124086	1446.231305	1922.89881	129.9812142	2613.985628
RND1	0.97671868	18.2832115	309.9067082	394.234484	80.6085049	418.6001248
RAD9A	465.894812	518.024326	553.6197506	1063.02513	620.6854878	1119.673788
EFEMP2	223.668579	163.53317	32.09389858	33.1881071	37.28143352	37.14849592
OR10AD1	0	0	0	0	0	0
AGAP5	179.550196	172.705247	395.2463904	249.534337	122.9078179	330.9568562

FAM21C	1650.03924	1999.21839	936.0486402	1788.2959	886.7237821	1078.737958
TMEM134	364.316069	350.42822	2257.605178	1831.38009	699.27878	1835.679335
ZMAT3	520.591058	680.541761	408.1942726	572.243423	1070.077903	620.6517002
ZFAND4	190.460143	256.980695	65.1907315	181.026039	36.27382721	117.7879139
THEMIS	0	0	0	0	0	0
MOS	0	0	0	0	0	0
ZNF738	113.299367	119.856609	201.5898005	185.04884	145.0953088	241.9182539
MS4A10	0	0	0	0	0	0
SLFN11	8.18490257	1.05636333	8.494853781	13.5970669	195.3647877	30.18994839
FAM71D	0.97671868	1.01573397	1.002934331	8.04560172	0	7.484062837
CCL19	0	0	0	0	0	0
CORO1B	3676.36913	4262.55809	10101.55458	11125.0558	3665.832977	7769.472013
FUT10	260.783889	281.35831	106.3110391	109.621323	122.92797	154.030349
LRRC20	1587.16786	1573.37192	207.6074065	244.385152	325.4568385	309.8728197
MUS81	1432.84631	1295.06081	1207.532934	994.637513	848.4045141	1036.533642
PURG	2.93015605	3.04720192	0	0	1.007606311	0
TMEM217	49.8126529	76.1800479	0	11.0627024	12.09127574	13.59091314
OR4D9	0	0	0	0	0	0
RPL12P13	0	0	1.002934331	4.02280086	1.007606311	0
ZNF596	21.3901392	20.8834905	30.55940906	66.8589503	111.3908777	73.39093097
COL6A5	0	0	0	0	0	0
CFL1	27231.8936	27363.8732	14394.11351	16924.9289	27373.64066	20133.57873
TMCC1	314.503416	257.996429	1227.591621	1070.06503	1117.435399	1099.957904
NAA16	630.96027	575.921162	806.3592019	868.924986	658.9745276	1140.730643
OR5B3	0	0	0	0	0	0
EFCAB12	3.90687474	2.03146794	8.023474646	7.03990151	6.045637868	20.83940015
OR10W1	0	0	0	0	0	0
OR1S1	0	0	0	0	0	0
FAM192A	1143.73758	1233.10104	2072.062327	1873.6195	1598.06361	1940.764276
RAB43	685.9886	740.114559	854.770842	1157.46038	600.3217642	1241.819855
FADS6	0	0	1.002934331	0	0	1.812121752
CBWD1	719.978411	717.788726	457.6489644	470.245307	1142.000841	584.690144
HOXC5	15.0805365	7.63831947	40.9899261	40.4894907	9.491651452	54.34553135

RAB37	6.83703079	12.1888077	75.2200748	42.2394091	257.9472157	110.5394269
DCP2	1928.04268	1860.82464	1738.085195	2681.19677	2254.015318	2966.443309
ZBTB8OSP2	0	0	19.105899	17.1170177	0	7.257547618
SNX32	9.76718684	13.2045416	5.014671654	13.0741028	10.07606311	11.77879139
RPL38	4994.93935	5116.25202	12708.1809	10451.2366	7199.347094	10123.41817
CYP7B1	0	0	0	0	0	0
OVOL1	0	0	144.4225436	504.861508	422.1870444	402.291029
RARG	932.766343	1137.62205	222.6514214	583.306125	3774.493242	606.1547262
CES4A	0.97671868	4.06293589	883.5851454	942.341102	95.72259957	506.4880298
CES3	15.6274989	13.2045416	427.2500249	222.259748	10.07606311	549.9789518
SSH3	490.312779	432.702672	1601.686126	1707.67897	2153.254687	1660.809586
CES2	951.323998	920.254979	1717.023574	1757.96398	1071.085509	2027.764241
PDP2	873.186503	1220.91223	804.3533332	2555.48425	555.1910775	707.6335443
SP3	4064.12644	3661.72097	2696.890415	3039.22605	3753.33351	3719.379897
KRT2	0	0	0	10.0570022	0	0.906060876
DMXL1	718.864951	725.234056	1665.873923	1802.21479	2170.383995	1825.712665
METAP1D	394.594348	363.632762	194.5692602	115.655525	185.3995613	275.4425064
ZNF621	1510.00709	1262.55733	1059.098653	1195.77756	1171.84614	1438.824671
EGFL7	653.4248	564.748089	74.21714047	113.644124	934.0510506	345.2091938
NADSYN1	1216.01476	1338.73738	3187.325303	3059.34006	2783.008632	2662.006854
DHCR7	2291.38203	5368.15404	1374.020033	26931.6461	6187.710358	2786.137194
FLJ42102	0	0	0	0	1.007606311	0
LVRN	3.90687474	3.04720192	2.005868661	0	4.030425245	4.530304381
COX6B1P3	0	0	0	0	0	0
NBEA	175.809363	208.225464	594.7400581	1103.25314	940.0966884	604.3426044
RNASEH2C	2064.7833	2344.31401	915.6790439	1008.71732	1194.013479	1148.885191
MYEOV	4992.00919	4966.93912	0	0	572.3203848	289.0334195
ANKRD13D	1295.12897	1324.5171	1765.164422	1618.17165	1193.005873	1697.052021
MRGPRF	0.97671868	3.04720192	0	0	0	0
MYD88	1035.3218	1092.92975	522.5287863	851.828083	1069.070296	1152.509434
MRGPRD	0	0	0	0	0	0
OXSR1	4013.33707	3723.68074	784.2946466	987.597612	2027.303898	1286.606444
SLC22A13	2.93015605	7.11013781	0	5.02850108	1.007606311	6.342426133

PHF8	1599.8652	1533.7583	2752.051803	2332.2188	1691.770997	2160.95519
LCLAT1	1037.27524	1048.23746	455.3321861	400.268686	879.6403098	781.0244753
ADH6	0	0	0	0	0	0
MIR4435-2HG	1922.51445	1530.66031	344.7987936	288.545449	315.9752632	376.4320516
XKR3	0	0	0	0	0	0
FRG2C	0	0	0	0	0	2.718182629
UNC93B3	0	0	2.015898005	0	0	0
AC007318.5	29.7801527	53.9050019	27.54057672	23.7948671	29.34149578	25.7502501
KAT5	1513.91396	1600.79674	711.0804405	1364.73519	1162.777683	861.6638932
SH3RF3	920.069	929.396585	1.002934331	0	16.12170098	4.530304381
GXYLT2	255.900295	259.012163	0	2.01140043	136.026852	27.18182629
HPSE2	0	0	0	0	1.007606311	0
DCAKD	422.91919	433.718406	835.4442975	700.97305	593.4801173	398.6667855
ARPP21	0	0	0	0	3.022818934	0
TADA2B	729.608857	773.989287	1059.098653	977.54061	838.328451	913.3093632
CCDC96	33.2084353	42.6608268	59.17312551	63.3591136	38.28903983	71.57880922
ADRBK1	2503.32999	2523.08319	5446.93635	4055.98897	4428.429738	4432.449806
RELA	3824.83037	3699.30313	1616.730141	1890.71641	2646.98178	2455.424974
EVC2	0	0	0	0	0	0
ZNF680	232.459047	262.059365	895.6203573	943.346802	441.6439223	1114.454878
HECTD4	3315.95993	3167.05853	2563.500149	2776.7383	3451.051616	4001.164829
FAM222B	503.010122	585.062768	584.7107148	587.328926	764.7731903	749.3123446
BNC2	0	0	0	0	0	0
DEC1	0	0	0	0	0	0
RXFP4	0	0	0	0	0	0
HPSE	335.991227	449.97015	5.014671654	10.0570022	184.391955	59.80001783
COQ2	733.515732	620.613457	161.4724272	162.923435	758.7275524	322.5576719
C10orf131	0	0	1.002934331	1.00570022	3.022818934	6.061547262
CCDC63	0	0	0	0	58.44116605	1.812121752
HSPA6	8.79046815	32.351127	7.020540315	14.079803	13.75382615	24.20994661
TRMT112	3316.90735	3501.235	2358.901546	2383.50951	2993.598351	2116.558207
LRRN3	0	0	0	0	0	0
KDM2A	5984.35538	5764.29029	5269.416974	6283.61495	7015.962746	8286.832774

ACSM6	0	0	0	0	3.022818934	0
ADCK5	293.992324	295.578586	473.3850041	458.599298	281.1221608	437.6274032
MRPL57	786.258541	797.351168	1028.007689	982.569111	1300.819748	1131.670034
NOC3L	1484.6124	1297.09228	426.2470906	358.029277	830.2676005	688.6062659
ESRRA	1347.87178	1555.08871	985.8844471	1360.71239	1315.933843	1447.88528
RHOD	430.73294	374.805836	1127.298188	1767.01528	3333.161678	1331.003427
ADAMTS20	0.97671868	0	0	0	1.007606311	0
COMMD1	269.720865	279.164325	381.2554565	371.80737	363.7962587	328.1571281
RAPH1	7711.19401	6261.99994	4184.242028	3677.84569	1816.714179	3925.961776
MTX1	564.543399	557.637951	1117.268844	762.320763	760.1382012	974.8761997
ADCY5	2.93015605	1.01573397	0	1.00570022	729.5069694	221.9849147
PARP14	1636.98051	1414.91742	1195.497722	1220.92006	547.130227	3855.289028
CYSLTR1	0	0	16.04694929	3.01710065	1.007606311	17.21515665
PARP15	0	0	0	1.00570022	0	0
CKS1B	2595.94245	2373.37416	1977.565855	1911.50423	1783.201193	2187.955804
ABCD2	0.97671868	1.01573397	0	0	0	0.906060876
AHSA2	772.584479	721.17112	1344.934937	982.569111	922.9673811	1117.17306
ABLIM3	1521.72771	1503.28628	796.3298586	1106.27024	242.833121	706.7274834
MAB21L3	0	2.03146794	3.008802992	0	7.053244179	5.436365257
TUBB8P12	0	0	0	0	2.922058303	1.938970275
KIAA1919	332.084353	423.561066	358.0475561	366.074878	421.1794381	504.675908
VANGL1	2256.22016	2632.78246	289.8480216	464.6335	3414.777789	649.6456482
GLRX	694.446984	1054.33186	226.6631587	251.425054	51.38792188	260.9455323
IQCB1	377.013412	293.547118	758.218354	424.405491	746.6362767	840.8244931
SYT12	66.4168705	42.6608268	2509.341695	1611.13175	1231.294912	4270.264909
GOLGB1	3410.70164	3385.44133	8835.851454	9410.33692	4553.372921	8854.932943
TERF1P1	0	0	0	0	0	0
C11orf86	16.6042176	7.11013781	0	0	1.007606311	0
LIPM	0	0	0	0	0	0
GPR151	0	0	0	0	0	0
DMRT2	0	0	0	0	12.09127574	0.906060876
ZNF483	0	0	25.07335827	40.2280086	140.0572773	80.63941798
PLAC8L1	0	3.04720192	2.005868661	0	3.022818934	0

SLC2A14	14.0940506	17.8058165	0	0	1.007606311	1.812121752
GPR137	1038.25196	1499.22334	669.9601329	1139.45834	700.2863863	1293.854931
SNCG	89.8581189	114.777939	0	0	24.18255147	11.77879139
MMRN2	15.1684412	16.7189812	5.385757356	36.9192549	29.06944208	13.89897384
MZT2A	1181.6245	1098.48582	1711.728081	1229.43834	2140.568924	992.2544473
TNKS	881.000253	901.971767	948.7758769	756.286562	1641.390681	964.0487723
ZNF449	131.857022	159.470234	529.5493266	536.038215	245.85594	361.5182896
ZBTB21	855.605567	776.020755	747.1860764	793.49747	846.3893015	954.0821026
PPP1R3B	407.291691	336.207945	1176.44197	1502.51612	752.6819145	1176.973078
OR10K1	0	0	0	0	0	0
FAM86B3P	51.2679637	35.550689	66.19366583	60.3420129	109.8290879	91.54839093
GPR148	0	0	0	0	0	0
STOX2	125.019992	127.982481	0	0	122.92797	5.436365257
MAP3K11	3154.80135	3531.70702	2284.684405	2527.32464	4019.341576	3021.713022
TRIB1	252.970139	353.475422	471.3791354	534.026814	1039.849713	851.6972236
CST9	0	0	0	0	0	0
KCNK7	1.95343737	4.06293589	0	5.02850108	5.038031556	6.342426133
SFT2D3	199.250612	210.256932	441.2911055	368.086279	734.5450009	347.9273765
AC007967.3	0	0	0	0	0	0
RP11-330H6.5	4.50267313	0	18.71475461	0	0	5.083001515
C1QB	0	0	0	0	0	0
C1QA	0	0	0	0	0	0
NDNF	20.5110924	26.4090833	2.005868661	3.01710065	0	18.12121752
IQCF1	0	2.03146794	1.002934331	0	0	0
OLR1	4.88359342	0	0	0	0	0.906060876
GLIPR1L1	0	0	0	0	0	0
DAG1	9496.63576	9772.37655	4085.954463	7019.7875	7224.537252	7524.835577
INSM1	0	0	0	0	0	0
DAB1	0	0	0	0	0	1.812121752
ARV1	334.03779	378.868772	833.4384288	759.303663	261.9776409	668.6729266
NAA20	1869.43956	1691.19706	3262.545378	1943.01282	2032.34193	2001.488475
CCDC36	0	0	0	0	1.007606311	1.812121752
RNASE8	0	0	0	0	0	0

SAA1	8025.8537	5711.04552	0	0	99.14846103	46.34501382
MINOS1	968.904934	1058.34401	944.7641395	912.884143	809.107868	976.7336245
EHBP1L1	5352.41839	5125.39362	749.191945	860.879385	2116.98086	1319.224636
THAP2	46.8824968	48.9786921	196.5751288	232.095496	78.67390079	105.1030616
TMEM196	0	0	0	0	0	0
RNF26	3416.56196	3621.09161	1016.975411	1209.85736	1767.34147	1467.818619
PPP1R14B	6417.66685	6439.55024	2836.960224	2351.04551	4869.186967	3954.674846
RNASE11	0	0	0	0	0	0.906060876
SSSCA1	615.049522	560.167128	429.2558935	307.744266	778.1844303	521.1752766
AGR3	0	0	0	0	103.7834501	10.87273051
SMARCC1	4931.45263	4747.54059	2843.318828	2509.22204	3287.819394	3875.222367
ZNF417	299.471716	282.079481	439.4256477	320.868654	384.9862194	382.7563565
PTPRM	7387.90012	5686.07878	189.5545885	152.866433	113.8595132	223.7970364
FKBP2	1301.96601	1378.351	1291.779418	1087.16193	524.9628882	989.4184768
VEGFB	1786.41847	2039.59382	969.8374978	1524.64153	381.882792	980.357868
PEAK1	2492.58608	1916.69001	22.06455528	48.2736103	1346.162032	172.1515665
TNFRSF10D	827.280725	828.838921	0	0	31.23579565	11.77879139
MST1	34.1851539	36.566423	407.6627174	383.584119	27.2053704	138.8810111
TNFRSF10C	41.0221847	55.8653685	19.05575228	7.03990151	321.4264133	71.57880922
GMPPB	1216.99148	1272.71467	2874.409792	2950.72443	1065.755272	2192.66732
MOB1B	1447.49709	1497.19188	497.455428	660.745042	1111.389761	829.0457017
ZNF622	1112.48258	1273.7304	833.4384288	623.534134	1010.62913	581.6910825
CSPG4	979.64884	791.256764	1.002934331	0	17.12930729	7.248487009
SNX33	2060.87642	1685.10266	474.3879384	559.16932	753.6895208	610.6850305
C2orf70	1.95343737	6.09440383	0	0	6.045637868	2.718182629
NABP1	4052.25931	2696.7737	477.3967414	258.464955	413.1185876	810.0184233
ADGRF3	1.95343737	5.07866986	0	3.01710065	5.038031556	2.718182629
NLRP13	0	0	0	0	0	0
CHD2	3544.25816	3268.12406	4127.455886	3730.76563	3754.159747	3952.926148
XCR1	0	0	0	0	0	0
CCDC106	395.571067	310.814596	1379.034705	1150.52105	65.49441023	1341.876158
CCR9	0	0	2.005868661	0	0	0
CEP83	484.452467	504.819784	230.6748961	262.487756	416.1414066	338.8667677

SULT1B1	0	1.01573397	0	0	0	0.906060876
NUDT4	3024.39964	3223.25909	8557.33659	9439.91456	2048.534163	13340.39637
PC	2458.40093	2367.67589	897.626226	1629.23435	837.3208447	1457.85195
UGT2A1	0	0	0	0	0	0
SCAI	443.967478	390.874747	351.5986883	286.644675	651.5988494	473.5708382
GPRC6A	0	0	0	0	0	0
NMNAT1	224.645297	244.791887	292.8568246	200.134343	300.2666808	249.1667409
LRFN4	1285.36179	1252.39999	77.22594346	156.889234	1145.648376	221.0788538
TRAPPC3L	0	0	0	0	0	0
APOBEC4	0	0	0	0	0	0
SLC19A1	21.487811	17.2674775	503.473034	473.684802	2079.699426	906.0608762
HSPB7	0	0	0	0	0	0
RCE1	525.718832	552.325662	371.4668174	410.013921	757.5184248	545.620799
UQCRH	1523.68115	1616.03275	3399.877176	3407.21176	2182.465194	3028.870903
TAS1R1	0	0	5.014671654	1.00570022	0	0
HES3	0	0	0	0	0	0
EIF1AX	6007.59151	5843.17219	3108.855721	2624.72671	4532.767372	2989.883103
SPDYE2B	0	0	0	0	0	0
OR1L1	0	0	0	0	0	0
CXorf23	380.920287	330.113541	994.9108561	804.560172	254.9243968	584.4092651
PSMD1	4485.0922	4545.40953	3038.891022	2676.16827	4619.874937	3534.543478
ADGRG2	0.97671868	3.04720192	0	0	6.045637868	0
SPATA3	0	0	0	0	0	0
MUC13	1.95343737	0	0	0	0	0
SUSD5	521.567777	429.65547	0	0	17.12930729	8.154547886
HEG1	15647.0333	7023.80042	63.18486283	149.849332	348.6317837	304.4364544
WFIKKN2	0	0	2.005868661	0	0	0.906060876
C11orf80	486.161725	481.691522	670.5819522	955.726972	721.6476401	637.6947053
TOMM20	6722.6668	6154.25088	9438.56484	7124.30993	4247.745774	6225.526159
CMB9-22P13.1	103.53218	148.29716	15.04401496	26.1482056	50.38031556	34.43031329
C1orf100	0	0	0	0	0	0
AGFG1	1803.02269	1737.92083	1915.604572	2443.85152	2454.528974	3291.719163
STAT5B	1786.38917	1601.10146	206.6044721	159.906334	1307.993905	326.7074307

CD7	0.97671868	0	4.011737323	0	0	1.812121752
TOPAZ1	0	0	0	0	0	0
CNP	4428.44251	4101.53378	664.9454613	612.471431	1316.941449	1248.551887
JUP	1298.05913	1193.48742	5132.01497	8398.6025	16703.08982	8132.802425
HAP1	0	0	26.0762926	42.2394091	63.47919761	15.4030349
TDRD12	0.97671868	7.11013781	4.011737323	5.02850108	12.09127574	9.060608762
PPIAP7	0	0	0	0	0	0
CCDC13-AS1	0	0	0	0	0	0
EIF1	10309.2657	10580.9008	7431.743391	5783.78194	8038.683151	4756.8196
ENDOV	335.991227	360.58556	413.2089443	302.715765	491.7118799	317.1213067
RNF213	8383.47925	8097.16714	8812.282497	9043.88993	9946.918212	17498.88961
TIGD3	57.6264023	51.8024326	32.09389858	76.4332164	51.38792188	53.45759169
KCNH6	0	0	0	0	0	2.718182629
MARCH10	4.88359342	0	0	0	27.2053704	20.83940015
PLK3	245.15639	285.421246	75.2200748	74.421816	178.3463171	108.7273051
NET1	3332.56415	3527.64409	1968.760091	1441.16841	4483.848085	2165.485494
DPY19L1	1769.57984	2028.09571	68.19953449	149.849332	1123.430657	247.3546192
RP11-89N17.1	0	0	2.005868661	0	0	0
RP11-97O12.7	4.56127625	0	3.008802992	0	0	1.812121752
PHOSPHO1	14.6507803	15.2360096	0	20.1140043	7.053244179	16.30909577
ZNF791	768.677604	743.517268	1044.054638	935.3012	880.6479161	1224.994305
PHC3	1817.67347	1503.28628	2895.471413	3111.63647	3677.763036	3746.561723
GPR160	174.832644	190.846256	5057.727624	4970.90463	743.6134577	4421.776409
CBX2	642.680894	610.456117	1485.345744	1179.68635	3465.158105	1896.385414
SPTBN2	1024.5779	1074.64654	433.2676309	532.015414	3885.329936	1192.376113
GOLIM4	2209.33766	2464.17062	824.4120198	1350.65539	1037.834501	1048.312434
KRT28	0	0	0	0	0	1.812121752
RBM4B	564.455495	660.318498	568.3729146	435.971043	691.4597551	564.5574713
USMG5	1607.67895	1614.00128	1380.338519	1405.9689	1252.767003	1238.811733
HOXB2	2.93015605	0	688.0129509	556.152219	15.11409467	466.6213512
C1QTNF1	111.34593	143.21849	0	2.01140043	6.045637868	3.624243505
MARCH3	396.547786	428.639736	27.07922693	58.3306125	65.49441023	48.02122644
SWSAP1	50.7893716	73.132846	159.4665586	273.550459	143.0800962	112.3515486

SLCO4C1	41.9989034	201.115327	0	0	39.29664614	2.718182629
RBM4	2080.62567	1846.17775	2523.292512	2633.52658	3236.189646	3317.741232
PIFO	0	0	14.04108063	23.131105	24.18255147	11.77879139
XXYLT1	987.462589	1026.90705	250.7335827	424.405491	647.8908582	428.5667944
SNURFL	0	0	0	0	0	0
UBXN2A	1424.05584	1376.31953	401.1737323	593.363127	1208.119967	645.1153438
RP11-18M17.1	0	0	0	0	0	0
RAX2	0	0	0	0	1.007606311	0
LRRC63	0.97671868	2.03146794	2.005868661	14.079803	0	4.530304381
TCAP	23.4412484	35.550689	59.17312551	57.3249123	17.12930729	45.30304381
CCS	558.683087	545.449143	934.7347962	679.853346	455.4380527	906.9669371
NRROS	211.713542	343.013362	7.020540315	6.03420129	1.017682374	2.718182629
CEP19	138.694053	190.957987	133.390266	208.179945	54.41074081	147.6879228
KLHL15	1405.49819	1374.28806	108.3169077	107.609923	221.6733885	184.8364187
FBXO45	1554.93614	1485.00307	1715.017706	2007.37763	1474.128033	2776.170525
SPERT	1.95343737	0	0	0	14.10648836	0
FAM46D	0	0	0	0	0	0.906060876
GNG5	1830.37081	2128.97841	2141.264796	2535.37024	1422.740112	2385.649226
FAM3C2	113.123558	100.131055	63.44562576	64.5257258	59.90219521	47.79471122
SLC25A30	765.747448	955.805668	1061.104522	1324.50718	1229.2797	847.1669192
C9orf131	0	0	0	0	0	0
CD34	102.555462	96.4947274	0	0	1.007606311	0.906060876
CTSF	707.144327	666.321486	1635.785893	1320.48438	92.69978064	598.9062392
MSRB3	1466.05474	1158.95246	43.12617622	83.4731179	74.56286704	70.67274834
LEMD3	819.466976	828.838921	929.7201246	1348.64399	1314.926236	1336.439792
C16orf91	185.57655	246.823355	519.5199833	365.069178	607.5866057	516.4546994
TLR10	0	1.01573397	0	0	0	0
TLR1	16.7702598	14.7382999	0	0	9.068456802	0.906060876
TLR6	141.458167	333.658453	0	0	38.28903983	15.4030349
FAM174A	255.900295	278.311108	402.1766666	424.405491	268.0232788	304.4364544
RGMB	2418.35546	2717.08838	2029.939085	1238.01697	776.864466	1722.421726
FAM53A	101.578743	55.8653685	40.11737323	53.3021114	48.36510294	99.66669638
NWD2	0	0	0	0	1.007606311	0

CYB561D1	372.129819	474.347765	452.3233831	500.838707	1085.191997	540.9183431
GSTA3	0	0	0	0	0	0
ZDHHC24	383.850443	441.844278	750.1948794	962.455106	767.7960092	823.6093364
RP11-23P13.6	2.93015605	0	0	3.01710065	1.007606311	0.906060876
TRMT10C	1132.99367	1091.91402	565.6549625	526.986913	1019.697587	761.9971969
SELP	0	0	0	1.00570022	0	0
CTU2	477.615436	550.527813	243.7130424	227.288249	1040.85732	393.2304203
MGA	2307.00953	2066.0029	410.2001413	449.547996	2685.27082	694.0426312
C12orf66	56.6496837	70.0856441	209.6132751	288.635962	780.8948912	310.7788805
ARL13A	0	0	0	0	0	0
SNX31	0	0	1.002934331	0	2.015212623	0.906060876
PIGG	970.858372	852.200803	1091.192552	1082.13343	1194.013479	1379.024654
PRPF8	21789.6171	20138.9575	4377.808354	5374.46195	20702.27927	9124.939084
ADCY6	1134.94711	1126.44898	6410.756242	6059.3438	1669.603658	6193.83215
REP15	15.6274989	19.2989455	2.005868661	5.02850108	9.068456802	1.812121752
PITPNA	1799.11582	1738.93656	1103.227764	631.579735	2717.514222	1506.779237
DDX23	2298.21906	2288.44864	2687.864006	3196.11529	5655.694225	4253.049753
ZNF80	0	0	0	0	0	1.812121752
ZNHIT2	324.270603	351.443954	454.3292518	271.539058	440.323958	502.8637863
EVX2	0	0	0	0	0	0
ZBTB4	3067.87339	3260.50605	1417.146209	1220.92006	2757.818474	1770.442952
TNK1	236.365921	273.232439	269.789335	394.234484	592.472511	441.2516467
ZHX3	3150.89447	2841.00792	1316.852776	1696.61626	3297.895457	2239.782486
PHLDA3	320.363728	367.695698	4.011737323	1.00570022	2209.680641	331.6182807
DIRC1	0	0	0	0	0	0
SLC16A11	0	0	1.002934331	0	1.007606311	0
SLC16A13	147.484521	303.704458	94.27582709	281.59606	233.7646642	56.17577432
GLIS1	0	0	0	17.0969037	0	0
OR2Y1	0	0	0	0	0	0
CHRNA9	0	0	2.005868661	1.00570022	0	0
PODN	0	0	0	0	0	1.812121752
STAG3L3	549.570302	482.585368	996.8064019	1098.58669	713.647246	984.4260814
SLC6A19	0	0	0	0	17.12930729	0

SNHG11	120.136398	124.935279	281.8245469	255.447855	158.1941909	318.0273675
C11orf45	140.64749	137.124086	24.07042394	22.1254047	78.59329228	60.7060787
EXO1	1338.1046	1215.83356	442.2940398	1006.70592	1012.644343	1675.30656
RALGAPA1	945.571125	880.935917	2130.473223	2659.33285	1742.070704	2597.060411
RP11-313P13.4	31.7921932	10.7972521	39.74628753	59.5575668	25.77456944	69.66702077
C20orf166-AS1	0	0	0	0	0	0
LIG4	560.636525	743.517268	351.0270157	254.442155	612.6246373	411.3516378
MIR1-1HG	0	0	0	0	0	0
PPIAP24	0	0	0	0	0	0
TRHR	0	0	0	0	0	0
RPL35P9	0	0	0	0	0	0
GTF2IRD2B	164.547797	152.563243	576.6671815	807.275563	445.4123699	593.0621465
ABRA	0	0	0	0	0	0
ATP2A2	16128.5556	16862.1997	6223.207522	7219.92185	10671.55844	10343.59096
ZWILCH	1370.33631	1297.09228	976.8580381	1080.12203	1694.793816	1780.409622
RPL4	21206.5747	19308.2902	61493.83532	39876.9488	35368.72469	36616.96437
SNAPC5	153.247161	171.984076	576.7173282	401.69678	434.0364947	555.1525594
STARD6	0	0	0	0	1.007606311	0.906060876
GOLGA6L2	0	0	0	0	0	0
VWC2L	0	0	0	0	0	0.906060876
C12orf76	72.2771826	127.982481	171.5017706	159.906334	186.4071676	139.5333749
ZCCHC12	0.97671868	1.01573397	0	0	12.09127574	0.906060876
CNTNAP2	0	0	4788.008495	5038.55808	21.15973254	2884.89783
GALNTL6	0	0	36.10563591	20.1140043	21.15973254	23.55758278
LINGO2	0	0	0	0	0	0
BBS1	406.314972	434.73414	1257.679651	686.893247	346.6165711	972.2033201
DENND4A	808.72307	702.887909	838.4531005	1000.67171	852.4349394	1118.079121
AC005017.2	0	0	0	0	0	0
IGDCC3	0	0	0	1.00570022	129.9812142	19.93333928
GCSAM	0.97671868	0	0	0	0	0
ANKRD36C	477.449394	365.217307	280.2900574	405.347472	272.7791806	283.8507513
SLC26A9	0	0	0	0	0	1.812121752
MFSD4	0.97671868	0	9.026408976	0	19.14451991	7.248487009

PELI3	616.30949	691.714835	691.0217539	708.012952	517.909644	615.2153349
TTC9B	4.88359342	3.04720192	7.020540315	11.0627024	0	0
MYO1H	0	0	0	0	1.007606311	0
TMEM81	35.1618726	35.550689	112.328645	84.4788181	46.34989032	77.01517448
MRPL11	1666.28207	1700.33867	1045.057573	943.346802	1634.337437	1295.667053
KLK15	0	0	0	0	0	0
IL20RB	20.5110924	13.2045416	5.014671654	20.1140043	24.18255147	20.83940015
GOLT1A	0	1.01573397	502.4700997	453.570797	152.148553	523.7031864
RP11-209A2.1	0	0	0	0	0	0
AKIRIN1	1636.0038	1659.70931	1409.122735	1205.83456	1629.299405	1954.37331
NPAS4	0.97671868	0	0	0	0	0.906060876
MSL2	803.839477	787.193828	979.8668411	1086.15623	1016.674768	1199.6246
ZNF497	72.6581029	58.0695112	160.8405786	119.557642	162.7485714	104.9037282
TRAM1L1	31.2549979	46.7237627	123.3609227	142.809431	7.053244179	137.7212532
CMKLR1	24.4179671	18.2832115	1.002934331	0	0	0
ANGEL2	768.677604	729.296992	1792.243649	1575.93224	456.445659	1463.288315
UGT8	713.981358	740.470066	0	0	50.38031556	22.6515219
KY	0	0	0	0	4.030425245	0
IQCK	50.7893716	55.8653685	258.7570573	213.208446	177.3387108	311.6849414
SLCO2A1	0	0	176.5164422	81.4617175	6.045637868	215.6424885
ZNF266	876.116659	851.185069	630.845694	680.859046	209.5821127	697.6668747
OR7D4	0	0	0	0	0	0
SLC29A2	498.126529	869.46828	785.2975809	3787.46701	812.1306869	982.1699898
BRSK2	78.1374947	59.9283044	80.23474646	21.1197045	251.9015778	86.98184411
VN1R6P	0	0	0	0	0	0
FAM47DP	0	0	0	0	0	0
GRIK1-AS1	0	0	0	0	0	0
B4GAT1	925.929312	987.293421	934.7347962	887.02759	477.6053916	1009.351816
TMEM167A	2582.20002	3053.2049	2460.197913	2140.13006	2413.741071	2237.689485
LEP	0	0	1.002934331	0	0	0
SH3PXD2B	352.595445	468.253361	127.37266	79.450317	736.5602136	430.3789162
RP11-79L9.2	1.95343737	1.01573397	1.002934331	0	1.007606311	2.718182629
KIAA1551	765.747448	905.018969	618.810482	679.853346	800.0394112	658.706257

LARP7	933.743062	958.85287	1344.934937	1046.93392	910.8761054	906.0608762
FGFBP3	58.603121	124.935279	41.12030756	33.1881071	61.46398499	55.26971345
NR1D2	2055.01611	1583.52926	1439.210765	1918.87601	1138.595132	1013.88212
PABPC5	0	0	0	0	0	0
BRMS1	1216.99148	1441.32651	1035.028229	1232.98846	1634.337437	1493.188324
RPL15	23919.0494	23730.928	29666.52671	20887.0056	26177.23915	16849.62451
C4orf32	454.174188	523.102996	3791.09177	3783.44421	634.7919761	2736.303846
HRAS	1845.02159	1765.34564	1724.044114	2984.91824	1130.534281	2045.885458
WDR49	0.97671868	0	0	1.00570022	0	0.906060876
SRP72	4335.65424	3904.48139	4367.77901	3033.19185	4115.064175	4011.131499
PCP2	0	0	90.26408976	51.290711	3.022818934	33.52425242
RIN1	1033.36837	1382.41394	43.12617622	90.5130194	224.6962074	196.6152101
C4orf26	56.6496837	109.699269	0	1.00570022	2.015212623	2.718182629
THAP6	314.503416	306.75166	454.3292518	371.10338	409.0881624	385.9819333
CEP135	793.095571	648.038274	361.0563591	399.262986	573.3279911	558.1334997
FZD4	352.595445	587.094236	5921.324289	3872.95153	399.0120993	4342.74978
CD248	0.97671868	0	0	0	0	0
BTC	10.7439055	11.1730737	17.04988362	34.1938073	1.007606311	29.90000891
PDZK1	9.76718684	2.71200971	0	8.00537372	232.4043957	29.90000891
ADGRE1	16.6042176	12.1888077	0	0	3.022818934	0
DENND6A	1242.38617	1191.45595	960.8110888	1034.86552	646.8832518	982.1699898
PDE12	3251.4965	2665.28594	1089.186683	970.500708	1341.124	1527.618637
GLMN	468.824968	513.96139	177.5193765	198.122942	170.2854666	237.3879496
DNAH12	28.3248418	16.2517436	11.03227764	8.04560172	2.015212623	9.060608762
YIF1A	1507.07693	1468.75132	2209.464331	1751.92978	1521.48553	1717.891421
CNIH2	31.2549979	34.5349551	30.08802992	116.661225	250.8939715	231.9515843
AMY1B	0	0	0	0	0	0
NLRP6	0	0	0	0	4.030425245	1.812121752
NDUFA11	2535.69844	2506.05949	2161.995449	1892.15456	1117.334639	1549.599674
RSRC1	799.932602	744.533002	527.543458	676.836245	946.1423263	703.1032399
CATSPERD	0	0	1.002934331	0	1.007606311	0
PQLC2L	37.11531	55.8653685	0	0	5.038031556	2.718182629
RAB1B	7666.25518	7922.72498	11312.09632	12938.3232	7794.832348	12319.70067

METTL15P1	10.1285728	5.05835518	10.36031164	2.14214146	7.033092053	3.896061768
OR9G1	0	0	0	0	0	0
PTDSS2	1388.89397	1639.39463	1099.216026	1065.03653	581.3888416	762.9032577
C19orf70	1060.71649	1110.19723	666.9513299	606.43723	347.6241774	447.5940728
C3orf33	81.0676508	116.809407	116.3403824	144.820831	159.2017972	124.13034
VN2R1P	0	0	0	0	0	0
OR5M3	0	0	0	0	0	0
SEZ6L2	889.790721	943.61686	342.0006068	544.083817	1575.896271	1622.755029
ASPHD1	213.901392	263.075099	2.005868661	7.03990151	267.0156725	47.11516556
OR5R1	0	0	0	0	0	0
KCTD13	317.433572	326.050605	882.582211	709.018652	909.8684991	821.7972147
P2RY14	0	0	0	0	0	0
AMZ1	0.97671868	1.01573397	0	0	1051.940989	90.60608762
GPR171	0	0	0	0	0	0
GPR149	0	0	0	0	0	0
CD164L2	0	0	0	0	5.038031556	0
FUT1	66.4168705	83.2901857	349.0211471	140.79803	418.1566192	84.26366148
DHX36	1502.19334	1640.41037	2480.2566	2806.9093	2963.370161	3165.776701
OR5J2	0	0	0	0	0	0
ZIC4	0	0	0	0	128.9736078	7.248487009
OR10AG1	0	0	0	0	0	0
AC026271.5	19.8957596	25.8605869	18.433933	21.5622126	19.57779063	22.51561277
OR4S2	0	0	0	0	0	0
FBXW8	913.231969	830.870389	944.7641395	448.542296	1027.758438	691.3244485
CA5A	0	0	0	0	0	0
ZG16	0	0	0	0	6.045637868	2.718182629
KLC2	3571.86023	3815.0968	2646.743699	2630.91176	2486.772376	2517.943175
SLC22A1	1.95343737	4.06293589	5.014671654	6.03420129	1.007606311	20.83940015
TEX36	0	0	0	0	0	1.812121752
CTBP2	2701.59411	2446.8422	4395.851142	3627.54056	2882.761657	4544.783234
CHST2	779.42151	1012.68677	0	0	41.31185876	27.18182629
ZDHHC14	281.294981	325.034871	151.4430839	295.675863	187.4147739	319.8394893
ATR	1392.80084	1260.52586	1098.213092	951.392404	2039.395174	1519.464089

LRRC75A-AS1	5652.33939	4890.75908	9416.550431	6765.34535	7964.120284	5608.516824
UBE2C	4114.91582	3854.71042	881.5792767	2194.43787	2175.422026	1797.624778
DSG4	0	0	0	0	0	0
GK5	478.592155	490.599509	769.2506317	892.056091	1035.819288	1009.351816
VCPIP1	1005.04353	1006.59237	1263.697257	1383.8435	841.3512699	1626.379273
RTP1	0	0	0	0	0	0
DES	0.97671868	1.01573397	0	0	0	1.812121752
PDIK1L	231.482328	269.169503	780.2829093	821.657076	375.8371541	743.8759793
SPSB4	0	0	0	0	55.41834712	0.906060876
RAG2	0	0	0	0	0	0
TRAF6	347.711851	325.034871	213.6250124	358.029277	329.4872638	283.5970542
ZNF654	658.308393	706.950845	501.4671654	749.246661	592.472511	639.6789786
TVP23C	199.660833	165.909987	73.51508644	52.1958412	84.98151629	47.06986252
MRPS22	1000.15993	1077.69374	973.8492351	869.930686	974.355303	1062.809408
PACS1	5139.49371	4499.7015	2109.170897	2332.2188	4661.186796	4074.55576
WFDC5	0	0	0	0	0	1.812121752
MARCKSL1	919.092282	897.908831	3957.578869	2974.86124	3056.069942	5945.571469
SH3BP5L	1628.19005	1693.22853	2339.845794	2508.21634	722.4537252	2243.406729
OR2T1	0	0	0	0	0	0
TMEM51-AS1	1.95343737	2.03146794	13.0381463	4.02280086	43.32707139	27.18182629
YPEL2	132.833741	180.800647	229.6719617	367.080579	242.833121	408.6334552
CADM2	0	0	6.017605984	4.02280086	1.007606311	1.812121752
ABO	0	0	0	0	1.007606311	1.812121752
PSMD2	19344.8903	18740.2918	7895.099051	8555.49173	12633.35785	13183.17669
FAM182B	4.88359342	3.04720192	101.2963674	115.223074	1.007606311	152.2182272
PPM1E	235.389203	206.193996	0	0	606.5789994	28.08788716
FAM131A	671.005736	632.802265	669.9601329	406.302887	162.2246161	517.3607603
CSRP2	597.751835	616.550521	28.08216126	20.1140043	90.68456802	48.92728731
INHBC	0	0	3.008802992	1.00570022	1.007606311	0.906060876
PARL	1395.731	1277.79334	1238.623898	1046.93392	1520.477924	1486.845898
DDIT3	220.738423	2281.35831	258.6028509	2531.00971	195.4756244	189.9334284
PCCA	212.924673	214.319868	616.8046134	486.758904	223.6886011	574.4425955
HIGD2B	0	0	0	0	0	0

DCTN2	2372.44968	2179.7651	3228.445611	3080.45976	3047.001485	2619.421993
NPPA	0	0	0	0	0	0
ZNF408	297.899199	271.200971	321.9419202	343.949474	597.5105426	391.4182985
CTDSP2	4555.41594	4443.83613	5323.575427	7792.16527	4384.09506	6627.835309
CKAP5	11575.0931	10637.7819	3260.539509	4688.5744	9979.332907	6066.983627
ARHGAP1	2183.94298	2068.03437	2038.965494	2170.30107	3330.138859	2842.312969
MED16	1712.18785	1380.38247	1331.896791	1808.24899	1443.899844	1611.882299
ATG13	1942.69346	1756.20404	1382.043508	1289.30768	1940.649756	1766.818709
GAL3ST3	0	1.01573397	0	0	1.007606311	0
C1orf127	0	0	0	0	5.038031556	3.624243505
CHST1	0	1.01573397	159.4665586	234.32815	33.25100827	1109.018512
GOLGA8A	201.848683	187.392761	1247.15887	995.562757	1172.773138	1597.684325
VWA3A	0	0	1.002934331	2.01140043	1.007606311	0.906060876
TP53I11	854.628848	650.069742	3865.308911	2776.7383	2361.829194	2499.821957
APITD1	284.830703	370.367078	293.6491427	299.07513	267.2877262	391.7716623
DOLK	499.103247	647.02254	410.2001413	440.496694	969.3172715	452.1243772
PHYHD1	822.397132	635.849467	0	0	20.15212623	12.68485227
CATSPER1	87.9046815	140.171288	0	0	9.068456802	1.812121752
ANKRD30BP1	0.97671868	0	1.103227764	0	0	0
CCNE2	988.439308	943.61686	398.1649293	512.90711	1333.06315	1020.224547
PHYKPL	391.664192	302.688724	1650.829908	889.038991	674.0886223	877.0669281
ANKS4B	0	0	0	2.01140043	0	5.436365257
CST6	165.065458	153.37583	0	0	31.23579565	18.12121752
GRAMD2	15.6274989	15.2360096	34.09976724	81.4617175	8.06085049	146.7818619
NF1P5	0	0	0	0	0	0
ZNF519	171.902488	157.438766	399.3985385	288.525335	237.5431879	418.6001248
LSM1	418.035597	425.592534	738.1596674	698.96165	631.7691572	716.6941531
PROP1	0	0	0	0	0	0
ISX	0	0	7.020540315	7.03990151	0	80.63941798
BANF1	6264.78108	6156.03857	3332.29946	3595.53918	4788.618766	4474.355122
APOF	0	0	12.03521197	1.00570022	0	4.530304381
CHRNA7	61.6993193	37.5923143	0	0	3.798675794	0
TMEM9B	884.907128	902.987501	633.854497	614.482832	845.3816952	714.8820313

NRIP3	26.3714045	25.3933493	0	0	8.06085049	3.624243505
PTPN2	1572.51708	1445.37929	582.7048461	540.050959	912.891318	905.1366941
SCUBE2	6.83703079	13.2045416	1.002934331	3.01710065	596.5029363	67.04850484
EIF1AD	1122.24977	1044.17452	770.253566	838.75398	1186.960235	1081.836686
SMAD2	2212.26782	2240.70914	1918.613375	2783.7782	1483.19649	2201.727929
EIF3F	5604.50948	5017.54299	1853.181939	1754.66528	2438.215828	1447.269159
OR10A6	0	0	0	0	0	0
ZNF25	138.694053	128.998214	327.9595261	174.991838	160.2094035	176.6818709
OR10P1	0	0	0	0	0	0
ARL10	149.437959	117.825141	6.017605984	2.01140043	3.022818934	4.530304381
CLTB	2728.952	2798.34709	1443.222502	1580.96074	3766.432392	1576.545925
PCSK1	0.97671868	0	0	0	0	0
LPL	17.5809363	18.2832115	0	0	0	0
RFESD	78.1374947	93.4475254	20.05868661	24.1368052	38.28903983	23.55758278
CCDC14	2223.98844	1816.13234	1850.41384	1622.19445	1529.546381	1638.158064
TBC1D10C	4.88359342	0	0	6.03420129	0	4.530304381
SART1	4091.47457	3861.82056	1890.531213	1599.06334	3465.158105	2169.109738
PPP2R2D	910.301813	779.067957	1452.248911	1400.9404	1331.047937	1523.088333
MCTP1	927.88275	1504.30201	88.2582211	60.3420129	73.55526072	104.1970008
POLD4	575.287305	636.875358	915.9297775	1107.63799	749.0948361	642.8320704
OR52W1	0	0	0	0	0	0
LRRC25	0.97671868	1.01573397	0	0	0	0
DPP10	0	0	0	0	0	0
CLCF1	532.311683	461.143223	25.07335827	17.0969037	315.3807754	69.76668747
RP11-395L14.13	0.97671868	0	0	0	0	0
TSGA10IP	0	1.01573397	0	0	0	0
GPR152	0	0	0	0	1.007606311	0
UBQLNL	0.97671868	0	7.020540315	1.00570022	0	2.718182629
UBQLN3	0	0	0	0	0	0
PNLIP	0	0	0	0	0	0
LIPT2	20.5110924	36.566423	33.09683291	65.370514	73.55526072	28.99394804
KCNE3	1.95343737	2.03146794	0	0	1.007606311	0
CABP4	0	0	2.005868661	7.03990151	0	2.718182629

ALG10B	215.405539	178.342571	465.3715588	372.591816	409.1284666	563.4158346
DRAP1	4189.14644	4779.02834	1998.848121	1635.26855	2232.855586	2273.306738
LONRF3	297.899199	393.089047	79.23181213	52.2964112	32.24340196	105.1030616
UCP3	36.1385913	42.6608268	31.09096425	27.1539058	15.11409467	38.0545568
UCP2	2330.45078	1333.65871	5394.783765	7903.79799	2194.566546	4793.968096
C11orf68	1957.34424	1946.14629	548.6050789	538.049615	581.3888416	489.2728731
PAAF1	323.293884	327.066339	667.9542643	393.228784	965.2868462	639.6789786
MRPL48	650.494643	650.069742	633.854497	519.947011	664.0125591	395.042542
RAB6A	3508.36375	3666.03784	4488.381864	4698.2593	4751.800832	3544.899754
P2RY2	313.526698	341.286615	202.5927348	510.895709	1591.010366	540.9183431
FOSL1	11272.3103	14782.9922	1.002934331	0	1233.279897	613.4032132
ERCC4	343.804977	377.853038	748.1890107	814.617175	899.792436	732.097188
SUGCT	7.81374947	5.07866986	0	0	3.022818934	0.906060876
CCDC85B	3866.82927	5216.80968	984.8815128	968.489308	1642.398287	1542.115611
RP11-276H1.3	0	0	0	0	0	0
TMEM70	524.497933	601.314512	1116.26591	1019.78002	583.4040542	978.5457463
LINC00476	41.9989034	41.6450929	43.12617622	29.1653063	122.92797	33.52425242
OR4B1	0	0	0	0	0	0
RPS6KB2	1385.96381	1209.73916	1666.876858	1584.98354	2200.612184	2256.997643
RMI2	419.012315	343.318083	721.1097838	806.571573	1041.864926	1178.7852
PRM1	0	0	0	0	0	0
DRD5P2	0	0	0	0	0	0
TOM1L2	1496.33302	1219.8965	2762.081147	2640.96877	3100.40462	4215.901257
TEX26	0	0	0	0	0	0
GOLGA8EP	0	0	0	0	0	0
ZNF77	94.7417123	65.0069742	183.5369825	142.809431	89.6769617	161.278836
GPR156	18.557655	24.3776153	69.20246882	56.3192121	7.053244179	104.1970008
LINC00521	0	0	0	0	0	0
LINC00116	422.91919	455.04882	147.4313466	115.655525	387.9284298	152.2182272
KDF1	0	0	813.3797422	761.315063	356.6926342	771.0578056
B3GNTL1	606.542303	515.992858	165.4841646	78.4446168	137.0344583	154.9364098
RBMXL3	0	0	0	0	0	0
MLXIP	3751.57646	4098.48658	631.8486283	2469.99973	3273.712905	1788.56417

C11orf44	3.90687474	15.2360096	0	0	0	0
BAK1P1	1.97297174	6.12487585	3.008802992	7.07007252	1.027758438	0.915121485
RWDD4P2	6.94446984	4.11372259	14.04108063	0	4.604760843	8.317638843
NR2F1	281.294981	301.67299	7084.728112	8228.63916	15.11409467	11055.75481
C15orf54	2.93015605	3.04720192	0	0	1.007606311	0
EIF3KP1	0	0	0	0	0	0
AURKAIP1	1754.18676	1693.22853	1967.757157	1889.71071	2368.882438	1876.452075
TTLL11	135.763897	142.202756	149.4372153	120.684026	135.0192457	86.98184411
EIF4E1B	0	0	0	0	3.022818934	4.530304381
TOMM5	2014.52135	2009.99533	1830.25486	1466.33103	1646.07605	1704.590448
LINC01106	82.2397132	134.117514	109.2195486	79.098322	146.5865662	136.1628285
RP11-121M22.1	0	0	3.008802992	1.00570022	0	4.530304381
C15orf53	0	0	0	0	0	0
SLC35E3	400.45466	475.363499	263.771729	365.069178	659.9821339	378.7334462
PRIMA1	0	0	155.4548213	145.826531	47.35749663	146.7818619
ZNF169	54.6962463	31.4877531	76.22300913	62.3534134	144.0877025	55.26971345
RUVBL1	2009.11033	1829.33688	1546.524738	1036.87692	2938.180004	1909.976327
SFN	1897.7644	1948.17776	1566.583425	8500.17822	1892.284653	1240.397339
OR52B3P	0	0	0	0	0	0
MSRA	269.574357	278.311108	214.6279468	229.299649	332.5100827	176.6818709
ZNF645	0	0	0	0	0	0
CCDC168	16.6042176	23.3618814	7.020540315	8.04560172	3.022818934	10.87273051
CTDNEP1	3737.54102	3690.16152	1319.861579	1223.93716	2160.297855	1368.151923
ETV4	1873.34644	2144.21442	89.26115543	62.3534134	82.62371753	115.0697313
FAM172BP	0.97671868	4.06293589	0	0	0	0
SWI5	197.297174	303.704458	295.8656276	334.898172	518.9172503	334.3364633
GAPT	0	0	0	0	0	0
BAIAP2	1102.71539	1405.77582	1384.049376	2216.56328	1195.021085	1031.097277
CALCB	0	0	0	0	0	0
CREG2	1.95343737	4.06293589	0	0	62.4715913	9.966669638
WBSCR28	1.95343737	0	0	5.02850108	4.030425245	5.436365257
HOXD8	0	0	57.16725685	31.1767067	12.09127574	20.83940015
RPL7AP66	8.94674314	16.5463064	17.17023574	28.9641662	14.90249734	14.42448915

ZDHHC21	224.645297	188.926519	937.7435992	834.731179	1032.796469	844.4487366
TSPEAR	0	0	2.005868661	6.03420129	6.045637868	7.248487009
PLEKHF2	540.125432	477.394967	4675.67985	4691.59151	1674.641689	3902.404194
A2M	0	0	0	0	13.09888205	2.718182629
ARL4D	271.527794	235.650282	220.6455528	98.5586211	15.11409467	79.7333571
DOK7	0.97671868	3.04720192	8.023474646	22.1254047	439.3163517	35.33637417
LRRN1	0	0	554.6226849	837.74828	0	656.8941352
UBE2O	2315.8	2374.78603	3235.466151	2835.06891	2182.47527	3742.937479
ORAI3	383.92858	609.806048	654.1639172	1086.65908	242.8835013	474.8302628
KLHL38	0.97671868	8.12587178	89.26115543	131.746728	1.007606311	20.83940015
RP11-544L8__B.4	0	0	0	1.00570022	0	0
UNC119B	1585.21442	1594.70234	924.7054529	774.389166	2568.388487	1598.291386
DENND2C	153.344833	213.304134	0	0	219.6581759	35.33637417
PLEKHD1	0	2.03146794	131.3843973	279.58466	24.18255147	110.5394269
FAM220BP	0	0	3.008802992	2.01140043	0	0
ASCL3	0	0	0	0	0	0
TUBB6	8725.028	7110.13781	10.02934331	1.00570022	2401.12584	264.5697758
LYSMD3	1176.94601	1428.12196	1058.095719	951.392404	899.792436	1224.994305
AMIGO3	0	0	0	0	12.38348157	0
B3GALT6	939.603374	857.279473	615.8016791	628.562635	809.107868	904.2487544
ZNF613	151.391396	141.187022	444.2999085	279.58466	109.8290879	712.1638487
C11orf16	0	0	0	0	0	0
TMPRSS7	0.97671868	0	0	4.02280086	1.007606311	0.906060876
RP1-146I3.1	0	0	1.002934331	0	0	0.906060876
NUPR1	15.6274989	11.1730737	20311.42607	9644.66507	158.1941909	2458.143157
JAKMIP2	0	0	0	0	13.09888205	0.906060876
RPL23P2	1.95343737	4.06293589	10.02934331	7.03990151	4.030425245	3.624243505
MBLAC2	372.129819	316.908999	278.8157439	247.402253	280.1145545	394.1364811
TPRN	540.125432	611.471851	2064.038853	2165.27256	1562.797389	2494.385592
LINC00302	0	0	0	0	0	0
KCNE5	2.93015605	7.11013781	0	0	0	0
ZNF683	0	0	0	0	0	0
SLC35A4	2596.11826	2767.87507	1401.09926	1651.35975	2460.574612	1829.336909

AIM1L	13.6740616	16.2517436	146.4284123	277.573259	81.61611121	144.9697402
IP6K1	2504.30671	2653.09714	1269.714863	1995.30923	1198.043904	1813.933874
SSNA1	1308.80304	1289.98214	2099.141554	2436.81162	1632.322224	1738.730821
CSTF3	1211.13117	1285.91921	1036.031164	1151.52675	939.0890821	1877.358135
YES1	4195.98347	4153.33621	1047.063441	1246.06257	2886.792082	1542.115611
CHMP6	630.96027	523.102996	678.9865419	560.17502	598.5181489	539.1062213
AQP7P4	0	0	0	0	0	0
DLEU1	280.523373	371.078092	366.3518523	437.137656	242.2789375	416.3168514
UFSP1	26.3714045	32.5034871	8.023474646	15.0855032	115.8747258	19.93333928
RP11-574E24.3	0	0	0	0	0	0
MC5R	0	1.01573397	0	3.01710065	1.007606311	4.530304381
TMEM39A	697.37714	705.935111	1029.010623	628.562635	659.9821339	689.5123268
TCP11L1	587.984648	566.779557	149.4372153	248.407953	405.0577371	155.8424707
GPX2	1.95343737	0	0	2.01140043	158.1941909	283.5970542
CCDC57	1515.8674	1275.76187	1567.586359	986.591911	948.1575389	1208.685209
HSF5	0	1.01573397	0	0	0	0
FOXG1	0	0	0	0	0	0
SPHK1	333.061071	437.781342	740.1655361	1180.69205	420.1718318	1423.421636
BNIP3	5697.13171	4503.3683	3141.260529	1910.3879	2252.292312	1929.574424
ENTHD1	0	0	0	1.00570022	0	0
MYPOP	243.202952	287.452714	339.9947381	337.915272	363.7458784	388.7001159
CH17-12M21.1	0	0	0	0	0	0
CIDEA	0	0	0	0	0	0
OR11H4	0	0	0	0	0	0
OR4D11	0	0	0	0	0	0
LRRTM4	0	0	6.017605984	0	0	0
ATAD5	1462.14787	1185.36155	544.5933416	461.616399	616.6550625	1083.648808
SMIM19	145.531084	140.171288	790.3122526	633.591136	234.7722705	328.9000981
OR11H6	0	0	0	0	0	0
ZNF404	17.7274441	26.4090833	179.5252452	92.0215697	11.08366942	67.71898989
RTTN	622.169802	557.637951	322.9448545	407.308587	290.1906177	569.0062302
OR4K17	0	0	0	0	0	0
OR10H4	0	0	0	0	0	0

RP5-1193P9.4	0	0	0	0	0	0
C10orf111	0.97671868	9.14160575	1.002934331	5.02850108	3.022818934	6.342426133
OR51B6	0	0	0	0	0	0
CDV3P1	0	0	0	0	0	0
ACBD7	88.8814002	81.2587178	36.10563591	112.638424	94.71499326	81.54547886
OR4L1	0	0	0	0	0	0
ANAPC2	1059.73977	959.868604	2739.013657	2393.56651	1769.356683	2844.12509
OR4K13	0	0	0	0	0	0
HMGB4	0	0	0	0	0	0
ZBTB8OS	303.759511	262.059365	470.3260544	292.638649	263.9928536	410.4365163
CYCSP34	0	0	6.017605984	1.00570022	4.030425245	2.718182629
OR4F21	0	0	0	0	2.015212623	0
SLC35G1	384.827161	355.50689	34.09976724	20.1140043	197.490837	77.92123535
OR4K5	0	0	0	0	0	0
IDSP1	5.89938085	4.33718406	3.159243142	1.74991838	0	7.103517269
OR4K3	0	0	0	0	0	0
ZNF135	22.4645297	21.3304134	0	0	10.07606311	0.906060876
OR4N2	0	2.03146794	0	0	0	0
OR4M1	0	0	0	0	0	0
FOXR1	0	0	0	0	0	0.906060876
OR4H12P	0	0	0	0	0	0
FOXN3P1	0	0	0	0	0	0
RP11-404O13.5	0	0	0	0	3.022818934	0
COX8A	3690.88317	3931.90621	2872.403923	3467.65434	3273.712905	2858.622064
RPL37AP8	0	0	0	0	0	0
AC110781.3	0	0	0	0	0	0
TAC4	0	0	0	3.01710065	0	0
ZSCAN2	149.437959	136.108352	51.14965087	49.2793106	182.3767423	88.79396587
PFN1P10	0	0	0	0	0	0
PRR18	2.93015605	2.03146794	2.005868661	1.00570022	1.007606311	2.718182629
B3GNT4	63.4867145	55.8653685	11.03227764	10.0570022	79.60089859	23.55758278
CDC26	489.961161	708.982313	307.9008395	275.561859	497.3242471	299.0000891
HSD11B2	9.76718684	23.3618814	135.3961346	128.729628	52.39552819	458.4668033

CRLF3	1085.13446	1107.15003	278.8157439	301.710065	328.4796575	259.1334106
RNPEP	1880.18347	1944.11482	7749.673573	8896.42411	2147.209049	7393.45675
EID2	879.046815	669.368688	575.6843058	637.613937	405.0577371	541.824404
DMRTA1	0	0	0	0	141.0648836	19.0272784
EID2B	110.369211	125.951013	74.21714047	36.2052078	94.71499326	54.36365257
GJC3	0	0	99.29049874	58.3306125	2.015212623	78.82729623
RIMS2	944.486967	886.735758	1.002934331	2.01140043	106.806269	37.14849592
KCMF1	2187.84985	2072.0973	1702.982494	1763.99818	2486.772376	1466.006498
DNAJC30	377.990131	326.050605	377.1033083	348.977975	719.4309063	590.7516913
SPRYD4	249.063264	248.854823	401.1737323	469.662001	495.7423052	446.688012
VPS37D	52.7428089	43.6765608	133.390266	114.649825	84.63893015	82.45153973
CLEC14A	0	0	0	0	0	0
SYNE3	646.587769	669.368688	148.4342809	89.5073192	121.9203637	93.32427025
CLK2	778.444791	673.431624	1746.09864	1458.26531	925.980124	1523.088333
LPCAT4	940.580093	1022.84411	564.6520282	722.092755	523.9552819	620.6517002
SLCO3A1	769.654323	951.742732	9.026408976	12.0684026	376.8447604	35.33637417
ZNF575	27.3481231	36.566423	42.12324189	86.4902185	14.10648836	23.55758278
WDR25	145.531084	140.171288	539.5786699	313.778467	240.8179084	334.3364633
SGF29	203.098883	209.241198	446.3057772	452.534926	432.2127272	380.5274468
PLA2G16	2862.76246	2799.36283	24.07042394	34.1938073	362.7382721	107.8212443
DIRAS1	16.6042176	10.1573397	4.011737323	0	288.175405	26.27576541
OR5AN1	0	0	0	0	0	0
OR10AC1	0	0	0	0	0	0
RP5-856G1.2	0	0	0	0	0	0
PHLDB3	195.343737	209.241198	277.8128096	353.000776	286.1601924	322.5576719
PRR15	0	0	1773.187897	2627.89466	268.0232788	684.0759615
GNG7	129.903585	115.793673	0	0	92.69978064	9.060608762
OR4C5	0	0	0	0	0	0
USF3	815.560101	771.957819	985.8844471	1464.29951	1834.851093	1630.003516
OR4C3	0	0	0	0	0	0
OR4S1	0	0	0	0	0	0
CNTD1	4.88359342	3.04720192	426.2470906	330.875371	0	189.3667231
DCAF4L2	0	0	0	0	0	0.906060876

OR4X1	0	0	0	0	0	0
CNBD1	0	0	0	0	0	0
DMBT1P1	0	0	0	0	0	0
CTD-2368P22.1	250.039983	271.200971	94.27582709	69.3933149	142.0724899	90.60608762
KBTBD11	136.740616	109.699269	7.020540315	4.02280086	200.5136559	16.30909577
B3GNT5	1318.57022	1418.98036	2221.499543	409.319988	201.5212623	978.5457463
MAP3K19	0	0	0	0	0	1.812121752
C14orf177	0	0	0	0	0	0
LMNB2	16776.3155	14068.6265	3225.346543	3825.89482	6607.448919	6119.12743
RMDN1	818.490257	818.681582	1629.768287	1387.8663	2141.163411	1353.654949
MEX3C	882.95369	799.382636	854.5000498	1228.96566	1140.610344	1113.548817
HORMAD2	0	0	0	0	0	0
RNF152	244.179671	232.60308	3369.859351	1762.99248	160.2094035	1723.327786
NANOGP1	0	0	0	0	0	0
MYO1D	88.8814002	92.4317915	1472.307597	2567.55265	2151.239475	2120.18245
C20orf197	7.81374947	5.07866986	0	0	6.045637868	0
FOXL1	0	0	0	0	1.007606311	0
TGIF2LY	0	0	0	0	0	0
LRRC37A	58.495682	28.8570022	58.2203379	43.2752803	41.90634649	27.76170525
FOXC2	0.97671868	2.03146794	1.002934331	0	0	0
OR4F17	0	0	0	0	0	0
BDNF	1576.42396	809.539976	0	0	3.022818934	8.154547886
SCAND2P	137.873609	153.37583	213.3341615	164.824208	344.3293048	225.6091582
CCDC121	31.2549979	43.6765608	121.355054	97.5529209	78.59329228	80.63941798
ACSF3	461.011219	425.592534	1011.96074	700.97305	1475.13564	997.5730247
OR10AB1P	0.97671868	0	7.020540315	1.00570022	0	0.906060876
BOK	1195.50367	1026.90705	395.1561263	569.226322	857.4729709	693.1365703
ZNF843	0	1.01573397	18.05281795	8.04560172	12.09127574	11.77879139
TTTY14	0	0	0	0	0	0
C8orf59	753.050105	768.910617	817.3914795	835.736879	719.4309063	629.7123089
PFN4	0	3.04720192	4.011737323	8.04560172	8.06085049	1.812121752
OR51V1	0	0	0	0	0	0
MAGEB6	0	0	0	0	1.007606311	0.906060876

OR52Z1	0	0	0	0	0	0
CDK5R1	429.756221	338.239413	87.25528677	85.4845183	169.2778603	223.7970364
OR51A1P	0	0	0	0	0	0
C15orf56	9.76718684	18.2832115	0	2.01140043	3.022818934	3.624243505
LINC00303	0	0	0	0	0	0.906060876
ZNF285B	0	0	0	0	1.047910564	0
TCERG1L	0	0	3.008802992	2.01140043	0	0.906060876
NCKAP5	0	0	358.0475561	362.052078	140.0572773	290.8455413
MAGEB18	0	0	0	0	0	0
DEFB104A	0	0	0	0	0	0
RUFY1	1976.87862	1885.20225	1119.274713	1269.19367	1336.085969	1272.10947
OR52E2	0	0	0	0	0	0
BASP1	3555.25601	3525.61262	4.011737323	1.00570022	5098.487935	547.2607692
DEFB103A	1.95343737	5.07866986	0	0	0	0.453030438
OR51L1	0	0	0	0	0	0
LRRC37A3	42.6728393	42.6303548	430.5597082	515.431417	203.2039648	556.6203781
RP11-629O4.1	0	0	0	0	0	0
FKBP9P1	12.3750257	15.1547509	6.33854497	9.91620413	6.509136771	2.038636971
VSIG10	1129.0868	1112.2287	537.5728013	389.205983	1514.432286	434.9092206
MIR7-3HG	0	0	0	0	0	0
IRX5	5.88961366	3.04720192	4923.504923	3291.47578	459.004979	4454.856692
METRNL	95.718431	88.3688556	525.5375893	484.747504	2146.201443	483.8365079
FAM91A1	5111.09074	4940.43862	2406.02943	3010.04063	7543.918224	3717.540593
KRT18P28	0	1.01573397	1.002934331	0	3.516546026	0
GJA1P1	0	0	0	0	0	0
RP11-334J6.7	2.93015605	5.07866986	31.09096425	11.0627024	8.06085049	32.61819154
WSB2	2944.80683	2513.94158	4146.130523	4042.91487	2217.741491	3835.355689
OR51G1	0	0	0	0	0	0
RP11-432M24.4	0	0	0	0	0	0
GRIN1	4.88359342	4.06293589	19.05575228	28.159606	5.038031556	17.21515665
SOX11	0	0	112.328645	99.5643213	0	124.13034
TYMS	8603.91489	7901.39457	229.6719617	735.166858	3027.856965	1109.018512
OR51G2	0	0	0	0	0	0

PXMP2	410.221847	374.805836	400.170798	501.844408	442.3391707	394.1364811
OR51A7	0	0	0	0	0	0
TCEANC	14.6507803	20.3146794	148.4342809	168.957636	64.48680392	96.94851375
OR51T1	0	0	0	0	0	0
PNMA1	3061.03636	3176.20013	835.4442975	1324.50718	2342.684674	1224.088244
OR51H1	0	0	0	0	0	0
C8orf4	0	0	0	0	24.18255147	19.0272784
MAMSTR	51.7660902	73.132846	20.05868661	12.0684026	27.2053704	29.90000891
TYMSOS	91.8115563	99.5419293	16.04694929	42.2394091	63.47919761	38.0545568
ANKLE2	2719.18482	2401.19511	2445.153898	2190.41507	3281.773756	2748.082637
C8G	2.93015605	2.03146794	2.005868661	1.00570022	0	4.530304381
FUT2	0	0	11.03227764	10.0570022	1.007606311	18.12121752
OR51S1	0	0	0	0	0	0
OR7A15P	0	0	0	0	0	0
OR51F2	0	0	0	0	0	0
EFCAB5	41.0221847	25.3933493	2.005868661	7.03990151	3.022818934	9.060608762
GCNT4	12.6973429	11.1730737	0	3.01710065	29.22058303	10.87273051
TOB2P1	19.5343737	29.4562852	14.04108063	6.03420129	26.19776409	4.530304381
OR52R1	0	0	0	0	0	0
MUC20	0	2.03146794	943.3199141	531.552792	200.8159378	1087.961658
THAP4	1348.8485	1316.39123	975.8551038	997.654614	1936.61933	1211.403391
OR51N1P	0	0	0	0	0	0
NFATC2IP	1942.69346	1653.61491	2953.641604	2939.66173	2556.297212	4215.901257
LY6H	0	0	0	0	0	0.906060876
RPL7L1P11	0	2.03146794	0	0	0	0
FIBIN	0	0	8.023474646	0	0	0
FAM89B	683.009608	688.169924	660.9337239	911.164395	831.9704552	762.712985
SHMT1	1126.15664	1087.85108	1562.571687	1899.76771	2151.239475	1673.494438
DPP7	1953.43737	1912.62707	6058.726292	5209.52712	1565.820208	4104.455769
TRIM60	0	0	0	0	0	0
AP000679.2	0	0	0	0	0	0
SEC24C	3909.80489	4488.52842	4859.216832	3713.0452	4316.585438	4639.031686
FMR1NB	0	0	3.008802992	0	0	0

SMCR8	2227.89532	2601.2947	2634.708487	3637.61768	1923.520448	3878.846611
HCG4	0	1.12746471	9.026408976	8.04560172	5.038031556	4.530304381
MTHFR	389.710755	626.707861	1355.967215	2642.98017	261.9776409	1387.179201
DEFB104B	0	0	0	0	0	0
C19orf18	13.6740616	14.2202756	6.017605984	1.00570022	0	4.530304381
DEAF1	941.556811	943.61686	1566.583425	1986.25793	519.9248566	1215.933696
MTX3	1449.45053	1456.56252	1079.15734	625.545534	1384.451072	1022.942729
TMEM80	243.202952	232.60308	1738.085195	1853.5055	231.7494516	1371.776167
SIX5	626.076676	548.496345	697.0393598	852.833783	506.8259746	620.6517002
IFNW1	0	0	0	0	0	0
FBXO46	703.569537	599.790911	846.4765751	933.360199	426.2174697	1047.406373
ZDHHC13	478.592155	484.505105	400.170798	546.095217	513.8792188	413.1637595
SLC38A9	411.198566	485.520839	149.4372153	194.100142	253.9167904	268.1940193
ACER2	28.3248418	44.6922948	62.1819285	84.4788181	234.7722705	172.1515665
WDR73	739.307673	593.18864	492.4407564	322.487831	1613.510214	670.1860483
POLE	4133.47347	3031.96591	3827.197406	4849.48644	4655.141158	6094.165453
FAM109B	4.88359342	8.12587178	320.9389858	285.618861	20.15212623	318.9334284
SCN4B	0.97671868	2.03146794	0	0	0	2.718182629
DSCAML1	0	0	0	0	2.015212623	1.812121752
RHOG	1521.72771	1482.9716	394.153192	645.659538	521.9400692	565.3819867
EPS8L2	5894.49726	3837.44295	5716.725685	5748.58243	2807.191183	6266.31702
ZDHHC22	0	0	0	0	10.07606311	0.906060876
MRVI1-AS1	0	0	0	0	1.007606311	0
ANO6	3317.91337	2879.60581	2520.373973	3090.51676	2925.081122	3559.007122
ZBTB34	501.056685	522.087262	352.0299501	306.738566	569.2975659	411.3516378
LINC00982	0	0	0	0	19.14451991	2.718182629
FAM9B	9.76718684	10.1573397	0	0	0	0
CETN1	0	0	0	0	0	0
NUDT4P1	3.66269506	1.35092618	1.855428512	7.83440468	2.478711526	7.02197179
FAM210A	1461.17115	1397.64995	290.8509559	400.268686	628.7463382	529.1395517
OR2T35	0	0	0	0	0	0
TALDO1	4766.38718	5304.1628	7722.594346	6299.70615	4284.342036	7736.853822
ULK1	792.118853	662.25855	3928.493773	2859.20571	7498.606169	3498.301043

NAP1L4P1	104.508899	83.2901857	1.002934331	4.02280086	31.23579565	2.718182629
OR14C36	0	0	0	0	0	0
RIMKLA	74.23062	87.3531216	33.09683291	31.1767067	154.1637656	81.54547886
CLVS1	0	1.01573397	2.005868661	3.01710065	0	7.248487009
OR2M7	0	0	0	0	0	0
RPS6KA3	2959.45761	2816.63031	457.3380548	406.302887	1693.786209	398.6667855
B3GNT8	61.5332771	59.9283044	47.13791354	95.5415205	4.030425245	74.29699185
PUS1	657.331674	679.526027	423.2382876	394.234484	2124.034104	864.3820759
PCNPP5	1.95343737	3.04720192	0	4.02280086	1.007606311	0
CHD9	1370.33631	1225.9909	2861.371646	3169.96708	2230.840373	3832.637506
OR2T12	0	0	0	0	0	0
SPACA4	0	0	0	1.00570022	15.11409467	0
OR2T33	0	0	0	0	0	0
PDDC1	2167.33876	1924.81588	3154.22847	2228.63168	928.0054127	2373.879496
OR2M1P	0	0	0	0	0	0
LINC01561	6.83703079	4.06293589	192.5633915	64.3648138	1.007606311	77.92123535
TRIM72	0	0	4.011737323	2.01140043	0	1.812121752
MAN1B1	1737.58254	1779.56592	4255.450365	3885.01993	2311.448878	4728.731713
DEFB103B	1.95343737	5.07866986	0	0	0	0.453030438
DEFB4B	0	0	0	0	0	0
ENSAP3	0	0	0	0	0	0
KCNA3	0	0	0	0	0	0
OR2AJ1	0	0	0	0	0	0
FZD8	29.3015605	46.7237627	45.13204488	154.877833	284.1449798	106.9151834
GJD4	0.97671868	0	0	2.01140043	0	0
FBXO39	1.95343737	3.04720192	0	0	0	0.906060876
CLDN22	0	1.01573397	1.002934331	0	0	0
KCNA2	0	0	0	0	0	0
TOP3A	2354.86875	2054.82983	1076.148537	1113.31014	1209.127574	1853.800553
CASKIN2	758.910417	809.539976	2711.93443	3670.80579	3780.53888	4032.87696
OR7E125P	0	0	0	0	0	0
ZBTB38	5845.66132	5467.69597	349.0211471	646.665239	1158.747258	663.2365614
BEND2	0	0	0	0	0	0

C8orf31	0	0	0	0	2.015212623	0
DLGAP1-AS1	61.5332771	99.5419293	8.023474646	1.00570022	25.19015778	9.060608762
LINC00469	0	0	0	0	0	0
FLJ13224	0.97671868	2.03146794	2.005868661	5.02850108	2.015212623	1.812121752
RPL13AP3	0	2.03146794	0	2.01140043	0	0
CCDC71	645.61105	589.125704	519.5199833	432.451093	437.3011391	574.4425955
C10orf71	0	0	0	0	0	0
OVOS2	6.8272636	1.01573397	0	4.666449	1.864071676	6.578001961
LRRN4CL	0	8.12587178	3.008802992	2.01140043	1.007606311	6.342426133
FLJ40194	0	0	0	0	0	0
TIMM22	801.886039	765.863415	314.9213798	298.692964	569.2975659	358.800107
HIC1	212.924673	216.351336	10.02934331	10.0570022	21.15973254	39.86667855
PPFIA3	120.136398	168.611839	892.6115543	1282.26777	745.6286704	1288.418566
MAGEF1	1450.42725	1421.01183	3075.999592	2895.41092	2036.372355	2644.791698
UMODL1	0	0	10.02934331	8.04560172	0	39.86667855
OR7E8P	0	0	0	0	0	0
RP11-218M22.1	43.9523408	55.8653685	43.12617622	51.290711	82.62371753	66.14244396
SAMD9L	511.80059	452.001618	0	1.00570022	27.2053704	28.99394804
ZFAS1	1386.94053	1114.26017	2470.277403	2041.57144	2669.985432	1695.457354
UBE2U	2.93015605	0	0	0	0	0
RP4-756H11.4	0	2.03146794	2.005868661	0	0	5.436365257
PAWR	5022.28747	3384.4256	4366.776076	3481.73415	5789.705865	3159.434275
TGIF1	916.162125	928.380851	498.4583624	610.460031	679.1266538	490.178934
MIEF2	567.473555	469.269095	749.191945	1002.68311	296.2362555	652.3638308
NAP1L5	28.3248418	52.8181666	244.7159767	250.419354	16.12170098	228.3273408
CBX3P1	0	0	0	0	0	0
RP4-597J3.1	0	0	0	0	0	0
NIM1K	20.5110924	9.14160575	3.008802992	2.01140043	17.12930729	0.906060876
CD19	0	0	0	0	0	0
ERICH5	0	0	0	0	2.015212623	0
OR2T8	0	0	0	0	0	0
NR2C2	2636.16373	2456.04474	1783.21724	1959.10402	2034.357142	1832.961153
GPR4	5.8603121	8.12587178	2.005868661	0	5.038031556	2.718182629

ACOT4	117.206242	67.0384422	255.7482543	319.812669	219.6581759	221.9849147
OLIG3	0	0	0	0	0	0
PTRF	26274.7093	21403.5463	0	7.03990151	1403.595592	221.9849147
OR2G3	0	0	0	0	0	0
ARIH2	3553.30257	3365.12665	3167.266616	2167.28396	1736.105674	3244.603998
RBM44	3.90687474	6.09440383	5.014671654	12.0684026	7.053244179	10.87273051
ZBTB33	960.114466	956.821402	1044.054638	1014.75152	1742.151312	1401.676175
OR2G2	0	0	0	0	0	0
ZBED2	0	0	0	0	0	0
VCX2	0	0	0	0	0	0
IRX3	248.057244	201.115327	4525.039113	3333.05143	489.1525599	4610.282374
ST8SIA3	0	0	0	0	0	0
RPRM	0.97671868	7.11013781	3.008802992	1.00570022	95.72259957	5.436365257
OR2B11	0	0	0	0	0	0
SLC25A22	1508.05365	1703.38587	2354.889809	2184.38087	920.9521685	2789.761438
RABEP2	258.830451	288.468448	2564.503084	1701.64476	983.4237598	1673.494438
NHLH2	0	0	0	0	23.17494516	0.906060876
RP11-56N19.5	0	0	0	0	0	0
ATOX1	1093.92493	1243.25838	1394.07872	1489.44202	836.3132384	1524.900455
FAM187B	0	0	0	0	0	0
TBL1XR1	3315.95993	3403.72454	8599.158951	8057.67013	2511.962534	8307.672174
SAMD12	523.452844	667.33722	1220.711491	1191.94584	571.3127785	1327.379184
CD163	0	4.06293589	0	0	2.015212623	0
C18orf32	283.873518	345.54254	1457.925519	1090.89308	437.6336492	657.3743475
OR7E149P	0	0	0	0	0	0
GIMAP3P	0	0	0	0	0	0
PIDD1	553.799494	603.34598	509.49064	584.311825	312.3579565	790.085084
RP11-512C24.3	0	0	0	0	0	0
ZNF491	30.2782792	50.7866986	0	0	4.030425245	1.812121752
RPLP2	10043.0122	10600.3318	9965.15551	10575.8328	5720.181029	8336.077182
GSG2	975.741965	1016.74971	72.21127181	198.122942	569.2975659	350.6455591
JUN	5910.12476	4857.23986	1017.978346	2261.81978	1416.694474	879.7851108
CSTF2T	965.974778	1003.54516	808.3650706	811.600074	733.5373946	863.476015

PGBD5	3.90687474	5.07866986	0	0	138.0420646	10.87273051
C12orf54	0	0	0	1.00570022	0	0
GBA	1767.89012	2281.55181	2648.569039	4582.95577	1564.51032	3100.612803
CASC2	18.557655	20.3146794	26.0762926	11.0627024	50.38031556	30.80606979
ACAD9	1099.78524	918.223511	1108.242435	959.438006	1651.466744	1389.897384
IL17RA	669.052298	655.148412	1203.521197	1406.9746	1280.667622	1784.033865
PNPLA2	3008.29355	2405.25805	5249.358287	5001.34717	773.8416471	4795.780218
MBOAT4	1.95343737	1.01573397	0	0	0	0
C2orf57	0	0	0	0	0	0
AGTRAP	372.129819	502.788316	673.9718702	1083.13913	287.1677987	748.4062837
CD163L1	958.161029	979.167549	70.20540315	10.0570022	111.8443006	83.35760061
SRRM3	54.6962463	46.7237627	0	0	354.6774216	59.80001783
THAP5	754.378443	734.284246	631.4173666	413.543929	947.9660937	599.8123
DEFB114	0	0	0	0	0	0
CRACR2B	158.228427	254.949227	8807.769292	9086.50145	145.0953088	5966.41087
SUMO4	2.93015605	6.09440383	8.023474646	8.04560172	3.022818934	4.530304381
MAGEB10	0	0	0	0	0	0
DNAJC28	34.1851539	32.5034871	42.12324189	38.2166082	36.27382721	39.86667855
OR4F4	0	0	0	0	0	0
NAALADL2	3.90687474	5.07866986	646.8926433	654.71084	62.4715913	767.4335621
CD151	5245.95605	4732.30458	9797.665477	7163.60264	2428.33121	6863.411137
RP11-16K12.1	0	1.01573397	0	0	0	0
POLR2L	3257.35681	3403.72454	2204.449659	2840.09741	1607.132067	3452.997999
FAM20C	4199.89034	2783.11108	938.7465335	1151.52675	457.4532653	507.3940907
PVRL3	1247.26976	1198.56609	36.10563591	115.655525	157.1865846	59.80001783
SLC35G5	1.95343737	0	0	2.01140043	6.045637868	0.906060876
ANXA2R	18.557655	15.2360096	187.5487198	314.784167	14.10648836	275.4425064
CTD-2530N21.5	2.93015605	7.11013781	0	0	2.015212623	2.718182629
TMEM94	1468.00818	1277.79334	2642.731961	5675.16632	1199.05151	2889.428134
FLII	17022.2532	13670.7635	8491.844978	11184.3921	7084.479975	10269.29397
SOX12	1498.28646	1510.39642	3956.575935	4747.91072	1969.870339	3770.119306
HNRNPA0	5337.76761	4940.53004	4300.58241	3623.53788	5394.724191	4781.283244
FXNP2	3.90687474	0	0	1.00570022	2.015212623	0.906060876

CTD-2201E18.3	49.8126529	29.4562852	140.4108063	411.331388	83.63132384	136.8151923
YIPF7	0	0	1.002934331	0	0	1.812121752
FAM87B	1.95343737	0	0	1.00570022	2.015212623	0
CDKN2AIPNLP1	0	0	0	0	0	0
RPS2P2	0	0	0	0	0	0
RP5-1061H20.4	4.88359342	12.1888077	27.07922693	27.1539058	21.15973254	47.11516556
MYOZ1	0.97671868	0	0	0	0	0.906060876
TMEM78	0	0	0	0	0	0
RP11-606P2.1	0	3.04720192	0	0	0	0
KCNJ10	0	0	0	0	2.015212623	0
AC108142.1	1.95343737	2.03146794	0	0	0	0.906060876
CHID1	1533.44833	1471.79853	1403.105129	1047.93962	1187.967841	1587.418655
PCDHB9	35.6599991	39.8269291	23.85980773	9.84580511	104.1361123	55.1156831
ZNF620	187.529987	221.430006	399.1678636	488.770305	242.833121	427.6607336
ZNF518A	829.234163	717.108184	1376.025902	898.090292	900.8000423	1025.660912
TMEM187	69.3470266	73.132846	523.5317206	542.072416	468.5369347	385.9819333
CACYBPP2	12.912221	15.4797857	9.176849126	3.05732866	9.572259957	6.378668568
SVBP	196.320455	227.52441	175.5135079	118.672625	137.0344583	104.1970008
ZNF619	183.623113	138.13982	293.8597589	362.052078	231.7494516	302.6243326
CCDC184	0	1.01573397	0	1.00570022	5.038031556	0
AP3S1	1880.18347	1945.13056	1007.949002	1436.13991	1131.531812	1427.951941
GRB2	3319.86681	3474.82592	19583.29574	15876.9893	8330.888982	20662.71828
ZBTB41	792.118853	785.162361	2241.558229	3273.5542	1445.915057	2724.525055
UBE2N	3516.18726	3262.53752	2465.212585	2155.21556	3212.24892	2773.452342
SPATA31C2	0	0	0	0	0	0
ARL6IP6	1540.28536	1235.13251	255.7482543	395.240185	439.3163517	374.2031419
ZNF354C	498.126529	449.97015	534.5639983	468.686471	246.8635463	509.2062124
CAPZA3	0	0	0	2.01140043	0	0.906060876
MAMDC4	222.69186	179.784913	270.7922693	276.567559	177.3387108	188.4606622
CENPBD1	183.623113	182.832115	246.7218454	132.752428	734.5450009	197.521271
ODF3	0	0	0	0	0	0
BET1L	1098.94526	1096.2512	1680.687263	1589.79079	820.1613092	2137.370425
RPS27	6746.18618	7324.31547	16333.89883	14329.7799	12312.90882	10153.01012

RIC8A	5433.48604	5019.75729	2491.288877	3369.09572	2228.825161	4098.113343
IMP3	1606.70223	1601.81247	697.0393598	701.97875	1566.827814	844.4487366
ASB8	392.640911	375.82157	1157.386218	1089.17333	906.8456802	1052.842738
LCN15	0	0	0	0	0	0
ODF3B	42.9756221	65.2609077	393.8422823	297.516295	7.375678199	157.1018953
DPY19L2	3.90687474	5.21071528	0	0	3.476241774	0
SPATA31E1	0	0	0	0	0	0
ZNRF3-AS1	0	0	0	0	0	0
C2orf73	0	0	0	0	0	0
GPR150	1.95343737	0	0	0	0	0.906060876
TSPYL6	0	0	0	0	0	0
LRRC75B	77.160776	62.9755063	876.564605	716.058553	65.49441023	626.9941263
DMAP1	623.14652	643.975338	1282.753009	1402.9518	715.400481	1218.651878
ADAMTSL1	949.370561	844.074931	12.03521197	9.05130194	36.27382721	33.52425242
FAM26E	5.8603121	13.2045416	0	0	0	0
IMPDH2	8011.04665	8035.47145	3718.880498	5943.68827	4764.970246	10232.14547
ALS2CL	881.000253	708.982313	266.780532	284.613161	269.0308851	257.3212888
MLF1	349.665289	366.679964	270.7922693	335.903872	242.833121	373.297081
PRSS42	0	0	0	0	0	0
NDUFAF3	971.83509	1016.74971	1069.127997	1084.14483	709.3548431	925.0881546
C2orf69	1106.62227	1018.78117	349.0211471	498.766965	1067.145768	766.5275012
GRAMD1C	251.016702	132.045416	381.1150457	480.724703	51.38792188	366.048594
STAP2	409.245129	525.134464	493.4436907	956.420905	757.7199461	639.6789786
ULK4P3	0	0	0	0	0	0
TWF1P1	16.7604926	7.52658873	10.50072244	14.19043	31.24587171	14.52415585
HTR3C	0	0	0	0	0	0
TSSK6	50.7893716	39.6136249	54.15845386	43.2451093	54.41074081	40.77273943
BOLA1	2.93015605	6.09440383	569.6666998	539.055316	704.3168116	331.6182807
PDE4DIP	1142.09669	1449.3508	2214.097887	5250.16746	3140.960774	3034.995874
DDX10	1774.69785	1928.87881	659.9307896	445.525195	561.2367154	852.6032845
RP11-998D10.4	0	0	0	0	0	0
GOLGA8Q	2.02180768	0	38.99408678	36.8689699	9.29013019	8.56227528
PPP1R42	0.97671868	0	0	0	0	1.014788181

NDUFV2	2193.7004	1967.83221	3405.433432	2358.7894	1909.716242	2514.255507
FAM27L	0	0	0	0	0	0
RP1-232L22__B.1	0.98648587	2.08225464	10.1898128	5.05867208	1.037834501	2.772546281
DALRD3	652.448081	711.013781	1294.788221	1639.29135	338.5557206	1337.345853
ZNF114	542.07887	806.492774	71.20833748	64.3648138	245.85594	69.76668747
FAR2P2	0	0	2.005868661	0	79.83264804	8.154547886
ZNF518B	792.118853	798.366902	0	0	47.35749663	19.0272784
AMER3	0	0	0	0	0	0
SPINK6	0	0	0	0	2.015212623	0.906060876
ZNF366	0	0	0	0	0	0
LCORL	442.453564	424.5768	463.3556608	622.528433	341.5785395	356.9879852
PARD6G	166.042176	144.234224	83.24354945	80.4560172	177.3387108	156.7485316
ZNF454	0	0	1.002934331	0	1.007606311	0.906060876
SH2B1	782.351666	732.344194	2457.18911	2200.47207	947.1499326	2223.47339
RP4-682C21.5	0	0	0	0	0	0
ZC3H12D	17.5809363	6.09440383	2.005868661	0	7.053244179	0.906060876
VN1R1	21.487811	45.3829939	98.80909026	41.2337088	22.16733885	53.63880387
KDELC2	5383.67339	4448.9148	167.4900332	296.681564	828.2523879	246.4485583
PLEC	58065.9258	65225.357	13608.81593	17044.6073	24003.85249	20587.51523
SH2D4B	1.95343737	0	0	0	0	8.154547886
RNF212	0	0	0	0	0	0
PRSS36	5.8603121	4.06293589	16.04694929	28.159606	16.12170098	27.18182629
ZNF543	203.157486	151.344362	143.4196093	146.832231	152.148553	192.0849057
TMEM151B	0	0	0	0	0	2.718182629
GALNT11	1024.5779	1156.92099	1271.720731	1308.41598	660.9897402	884.3154151
SLITRK1	0	1.01573397	0	0	2.015212623	0
C9orf62	0	0	0	0	0	0
AP000345.1	0	0	1.002934331	8.04560172	0	0.906060876
WDR6	5176.60902	4879.586	4412.911055	5975.87068	4018.333969	5058.537872
PRM3	0	0	0	0	0	0
TNP2	0	0	0	0	0	0
SPAG11A	0	0	0	0	0	0
GEN1	1202.3407	975.104613	626.8339567	761.315063	2078.69182	1572.01562

TMPRSS9	12.6973429	13.2045416	4.011737323	125.712527	3.022818934	1.812121752
AQP11	30.2782792	38.5978909	84.24648378	82.4674177	92.69978064	119.6000357
TMEM11	778.444791	813.602912	839.4560348	865.907886	868.5566403	649.6456482
ZNF354B	236.600334	204.091427	461.580467	342.581721	327.9859304	334.481433
KCNG2	0.97671868	0	0	0	1.007606311	1.812121752
SHISA3	1.95343737	11.1730737	0	0	3.022818934	0
OR2D3	0	0	0	0	0	0
CALML3	0	0	0	0	0	0
CALML5	0	0	13872.58766	3601.41247	239.8103021	5130.116681
ZFAND2A	206.087642	198.068125	295.8656276	240.362352	753.6895208	302.6243326
PLEKHM3	102.555462	79.2272498	47.13791354	61.3477131	123.9355763	105.1030616
ZNF223	37.3497225	71.1927941	138.6456419	125.873439	93.68723482	167.4128681
HTR1A	0	0	0	0	0	0
CCDC185	0	0	0	0	3.022818934	0
FAM220A	577.240742	602.330246	527.543458	426.416891	780.8948912	466.6213512
DNAJC22	106.462337	131.029682	375.0974397	358.029277	247.8711526	489.2728731
NEUROG2	0.97671868	3.04720192	0	0	1.007606311	0.906060876
CEP295NL	23.6756609	25.7082268	0	0	1.007606311	0
BEND3	644.634331	465.206159	254.74532	463.627799	471.5597537	828.1396408
RP11-567M16.3	0.97671868	2.03146794	0	0	1.007606311	0
NT5DC1	480.545592	483.489371	752.200748	846.799581	905.8380738	834.482067
RPS3AP5	10.1383399	5.34276069	20.17903873	17.1471887	10.85191997	8.951881457
LINC00843	1.51391396	2.68153769	9.588052202	6.90916048	0	15.86512594
GLDC	0	2.03146794	0	0	78.59329228	13.59091314
COX14	294.969043	309.798862	597.7288024	527.892043	817.1586424	417.6306397
LINC00314	0	0	0	0	0	0
H3F3AP6	6.42680894	11.8840875	30.72990789	15.2162443	11.76884172	28.77649343
MCMDC2	4.88359342	4.06293589	0	0	15.11409467	2.718182629
TUBAL3	6.83703079	30.4720192	14.04108063	3.01710065	3.022818934	1.812121752
CTD-2192J16.15	37.2032147	39.6034676	282.0451925	182.806128	114.151719	120.8322784
P4HTM	542.07887	631.786531	2053.006575	1902.78481	993.4998229	1479.597411
UCN3	0	0	0	0	0	0
DTX3	300.829355	310.814596	1134.318728	1141.46974	389.9436425	839.9184322

KLHL11	291.062168	238.697483	92.26995843	96.5472207	277.0917356	182.1182361
NECAP1P1	0	0	0	0	0	0
AMBN	0	0	0	0	0	0
CTXN1	249.063264	195.020923	462.3527265	462.622099	889.7163729	1155.227617
SLC25A20	149.437959	148.29716	334.9800665	265.504857	249.8863652	324.3697937
CA8	0.97671868	0	1848.407971	658.733641	42.31946507	1089.085173
CKS1BP6	0	0	0	0	0	0
CD28	0	3.04720192	0	0	0	0
EPM2AIP1	1248.24648	1273.7304	1627.762419	1655.38255	1300.819748	1549.364098
ERBB4	0	1.01573397	276.8098753	408.314288	1973.900764	393.2304203
MAF	0	0	39.1144389	277.573259	11.08366942	34.43031329
CTNNBIP1	1121.27305	1024.87558	860.5176557	963.460806	644.8680392	824.5153973
OR6B3	0	0	0	0	0	0
DEFB125	0	0	0	0	0	0
GAPDHP29	0	0	0	0	0	0
PSAPL1	2.93015605	5.07866986	0	1.00570022	13.09888205	2.718182629
OTOS	0	0	0	0	0	0
GTPBP6	1027.99641	1088.35895	667.9542643	890.547541	807.5964585	578.066839
ERN1	648.541206	681.557495	3514.281895	1023.80282	988.4617914	1665.33989
GPR35	35.8358085	39.6136249	56.76608312	39.0513394	16.79679721	34.96488921
ACTG1P1	0	1.01573397	8.023474646	5.02850108	2.015212623	0.906060876
RP11-455G16.1	0.97671868	0	0	1.00570022	0	1.812121752
C10orf53	0	0	0	0	0	0
PPIAP33	0	0	0	0	0	0
ARMC10P1	1.05485618	2.31587346	1.083169077	1.10627024	2.206657822	0.95136392
CSRNP3	16.6042176	34.5349551	0	0	2.015212623	2.718182629
ZNF713	34.5856086	44.6618228	131.725395	147.77759	96.44807612	185.3800553
PARP10	961.091185	936.506722	1665.873923	1515.59022	48.36510294	2718.182629
DYNAP	6.83703079	2.03146794	0	0	0	0
SUZ12	3329.7805	3174.50386	2453.137256	2941.09988	1268.89878	3432.611629
NSUN3	223.668579	264.090833	208.6103408	277.573259	346.6165711	231.0455234
KCTD12	1472.89178	1395.61848	5.014671654	11.0627024	140.0572773	53.45759169
DHFRL1	98.6485871	155.407298	357.0446217	220.248347	224.7465877	131.378827

RP11-169K16.8	0.97671868	0	2.005868661	0	0	0
RPP25	1093.92493	1307.24962	768.2476973	1055.98523	889.7163729	690.4183876
GRINA	2847.13496	3314.33995	16158.275	18790.5028	4743.810514	15667.60467
C5orf64	0	0	1.002934331	0	2.015212623	0.906060876
GLULP4	0	0	0	1.00570022	0	0
THBD	1681.90957	1371.24086	647.8955776	2252.76848	215.6277506	910.5911806
GP5	3.90687474	0	2.005868661	0	1.007606311	1.812121752
LMO7DN	0	0	0	0	0	0.906060876
COX5A	2701.60388	2773.96948	973.8492351	1078.11063	2457.551793	1193.282174
STX19	0	0	33.09683291	13.0741028	0	15.4030349
FAM132B	378.966849	225.492942	15.04401496	9.05130194	133.0040331	25.36970453
FAM219B	1018.71759	825.791719	745.1802077	581.294725	762.7579776	584.4092651
HIST1H2BPS1	0	0	0	0	0	0
ZHX2	371.1531	310.814596	751.1978137	736.172558	1630.307012	961.3305896
CPN2	0	0	0	0	0	1.812121752
CPNE7	51.7660902	71.1013781	358.0475561	191.083041	62.4715913	249.1667409
C5orf46	0	0	0	0	0	0.906060876
CD300LB	0	0	0	0	0	0.906060876
GDPD4	0	0	0	0	0	0
RIIAD1	0.97671868	0	3.008802992	4.02280086	0	5.436365257
MPI	1246.29304	1159.9682	654.916118	528.998313	729.5069694	641.4911003
ADORA2A-AS1	2.93015605	1.01573397	0	1.00570022	0	0
H1FOO	0	0	0	0	0	0
TRIM73	44.3723298	78.9530017	138.5052311	150.543265	13.08880598	90.73293614
OPLAH	288.132012	206.193996	1167.415561	648.676639	201.5212623	525.5153082
TMEM52	47.8592155	50.7866986	46.13497921	113.644124	106.806269	82.45153973
TMEM139	29.3015605	35.550689	1.002934331	3.01710065	6.045637868	6.342426133
RNF186	0	0	0	0	0	0
AC114812.5	0	0	2.005868661	0	0	0.906060876
EFCAB13	34.8102539	46.7237627	4.011737323	1.00570022	9.975302482	11.77879139
MSC	0	0	0	0	0	0
APOLD1	107.546494	86.2053422	57.16725685	123.01725	130.6563104	139.2253142
FAM101A	4.88359342	5.07866986	103.1919133	42.138839	0	4.530304381

EXOSC4	516.684184	603.34598	677.9836076	582.300425	802.0546238	983.0760507
DPY19L3	1527.58802	1281.85627	1980.795303	1405.9689	892.7391918	1202.342783
TAF7	3708.60084	3774.46744	3355.818271	2387.53231	3723.10532	2700.061411
ZNF852	75.2073387	87.3531216	103.3022361	112.638424	61.46398499	124.1212794
FOXE1	0	0	0	0	0	0
PFAS	2705.51075	2535.27199	211.6191438	259.470656	1511.409467	582.5971434
HYI	2023.76111	1326.54857	1305.820499	1443.17981	298.2514681	725.7547618
C17orf62	1123.22649	1140.66925	1146.35394	1236.00556	1694.793816	1761.382343
TPRX1	0	0	0	0	0	0
LGALS7B	0	0	0	0	0	0
ZNF552	158.619114	147.443943	224.4266152	246.999973	281.777105	299.0906952
SMIM10L2A	0	2.03146794	0	0	22.16733885	1.812121752
GAK	1132.99367	1115.2759	3349.800665	2792.8295	2282.228295	3215.61005
ZBTB7A	3095.22151	2921.2509	3037.888088	2706.33928	3602.192563	3435.782842
TUFM	4334.67752	4181.77676	7426.728719	6867.92677	7210.430764	8707.24502
ERICH3	0	0	0	0	0	0
RMI1	1106.62227	983.230485	377.1033083	542.072416	1048.91817	426.7546727
CTC1	1058.76305	832.901857	464.3585951	438.485294	474.5825726	475.68196
FBXO34	1531.4949	1386.47687	1050.072244	1170.63505	861.5033962	1061.903347
LINC00324	17.5809363	14.2202756	2.005868661	11.0627024	8.06085049	8.154547886
SEPW1	618.262927	590.141438	2512.350498	2211.53477	1660.535201	2918.422082
EIF3K	2756.30013	3038.06031	2249.63185	2773.72119	2719.529434	2806.070534
MRFAP1L1	869.279629	846.106399	1885.516542	1571.90944	1272.606771	1386.273141
SNX18	881.000253	836.964793	852.4941811	671.807744	760.742765	925.0881546
EXD1	0.97671868	2.03146794	3.008802992	8.04560172	5.038031556	2.718182629
AURKB	2226.9186	2188.90671	156.4577556	385.183183	1278.652409	472.9637774
TAS1R2	0	0	0	0	0	0
C14orf39	0	0	4.011737323	2.01140043	3.677763036	1.812121752
MRFAP1	6082.02724	5828.28153	9667.284014	7887.70679	9015.053667	7979.678137
C3orf38	654.401518	675.463092	490.4348877	459.604999	714.3928747	547.2607692
KLHDC7A	0.97671868	2.03146794	463.3556608	156.889234	65.49441023	227.4212799
RP11-363G10.2	0	0	0	0.50285011	0	0
TMEM107	258.830451	283.389778	81.77926533	70.3990151	224.6962074	102.384879

LL0XNC01-131B10.2	0	0	0	1.00570022	0	0
RP11-157K17.5	0	0	0	0	0	0
RRS1	932.766343	1061.30996	983.8785784	691.921748	1834.851093	1818.464178
EXOC3L1	8.79046815	10.1573397	0	0	2.015212623	3.624243505
TRIML2	125.99671	182.832115	0	0	5.038031556	6.342426133
RCC2	4023.10426	3822.20694	11733.32873	4206.844	8192.846917	8629.323785
OR13D1	0	0	0	0	0	0
IGSF22	0.97671868	1.01573397	1.002934331	6.03420129	0	0.906060876
C9orf50	0.97671868	0	0	0	4.030425245	0
ZFP42	0	0	0	0	0	2.718182629
CTD-2527I21.15	0	0	2.005868661	0	0	1.812121752
CCDC89	0	0	0	4.02280086	18.1369136	4.530304381
TAAR3	0	0	0	0	0	0
C9orf106	0.97671868	3.04720192	29.08509559	24.1368052	103.7834501	28.99394804
FAM133A	58.603121	58.9125704	0	0	163.2322224	1.812121752
DPM3	185.57655	215.335602	502.4700997	774.389166	347.6241774	337.9607068
C12orf42	0	0	0	0	9.068456802	0.906060876
CYC1	3917.61864	3751.10556	3502.246683	3117.67067	5588.184602	4537.552868
PER1	243.202952	263.075099	296.8685619	610.460031	496.7499115	525.5153082
HTR1F	0	0	0	0	1.007606311	0
RP11-349N19.2	0	0	3.028861679	1.31746728	0	0
TMTC2	254.923576	345.349551	348.0182128	570.232022	442.3391707	902.4366327
HES7	22.4645297	22.3461474	11.03227764	14.079803	2.015212623	29.90000891
FARSA	2908.66824	2936.48691	2091.11808	1906.80761	3219.302165	3091.47971
SPTY2D1	1407.45162	1364.13072	2603.617523	2919.54773	1641.390681	3064.297883
RP11-458F8.3	0	0	0	0	0	0
C10orf67	0	0	0	0	0	0
SAMD4B	2843.22809	2838.97645	2868.392186	2517.26764	3158.845786	3319.80705
LINC00670	0	0	0	0	0	0
MTUS2-AS1	0	0	1.002934331	1.00570022	2.015212623	3.624243505
CYP11B2	0	0	0	0	0	0
GIMAP7	0	0	0	0	0	0
ALOXE3	12.6973429	47.7394967	18.05281795	50.2850108	153.1561593	65.23638308

EDC3	1242.38617	1213.8021	619.8134164	562.18642	1688.748178	805.4881189
TCAIM	719.84167	747.580204	647.8955776	1007.71162	562.2443217	710.3517269
RPS2P28	0	0	0	0	0	0
FUCA1	941.556811	884.70429	451.3204488	375.12618	504.810762	428.5667944
PXT1	0.97671868	1.01573397	0	1.00570022	0	3.624243505
GGN	24.4179671	45.7080288	13.0381463	19.1083041	17.12930729	12.68485227
OR7E97P	0	0	0	0	0	0
HNRNPCL1	0	0	0	0	0	0
TMEM125	0	0	600.7576641	913.175796	220.6657822	409.539516
ZNF664	4348.35158	4471.26095	5921.434611	4705.77188	7924.753106	6084.15348
SIGLECL1	0	0	0	0	0	0
CALR	2281.52922	34361.2645	5666.092457	29723.4699	22384.48917	21985.56716
LINC00311	0.97671868	0	0	0	1.007606311	0
MAGED1	1398.66116	1984.74418	9281.154296	8270.87857	2951.278886	5989.062392
RP11-111M22.2	47.8592155	47.7394967	206.6044721	137.78093	182.3767423	258.2273497
LDLRAD3	1371.31303	1143.71645	933.7318619	1712.70747	377.8523667	2006.01878
CDH4	2504.30671	1690.18133	1.002934331	1.00570022	242.833121	119.6000357
RP11-429E11.3	0	0	0	0	1.007606311	0
SMCO3	0	0	0	0	1.007606311	0
RAD23A	4437.23298	4033.4796	3252.516034	2972.84984	3807.74425	3364.204033
C2orf71	0	0	0	0	0	0
GADD45GIP1	2092.13142	2282.35424	1315.849842	1091.18473	1760.288226	1668.058073
MEIS3P1	112.850077	96.5455141	95.7601699	50.9487729	170.2552384	131.6234635
DAND5	3.90687474	5.07866986	1.002934331	0	1.007606311	1.812121752
TMEM151A	0	0	0	0	7.053244179	0.906060876
PTPN11	12764.7365	12274.1293	4994.612967	5447.87807	9852.364436	6918.67179
NSUN7	85.9512442	66.0227082	379.109177	429.433992	121.9203637	356.0819243
ZCCHC5	19.5343737	11.1730737	0	0	0	0
FAM156B	265.394001	476.490964	133.3802366	34.1334653	103.1889623	57.1815019
WSCD1	0.97671868	0	0	0	65.49441023	8.154547886
RAB39A	15.6274989	11.1730737	0	0	6.045637868	0
CLK3	1208.20101	1168.09407	939.7494679	890.044691	1387.473891	862.5699541
GS1-124K5.9	0	0	0	2.01140043	1.007606311	1.812121752

HLA-DQB1	12.6973429	10.1573397	27.07922693	77.4389166	602.5485742	138.6273141
GATA2	476.638718	385.978909	1333.90266	1795.17488	1604.109248	1975.21271
ARID3B	225.622016	218.382804	83.24354945	131.746728	249.8863652	168.527323
HMGN2P46	10.7536727	11.1730737	1.002934331	1.00570022	1.007606311	3.624243505
TMEM31	0	0	2.005868661	2.01140043	2.015212623	0.906060876
PACS2	1451.40396	1490.08174	3105.084688	3351.99882	3565.918736	3277.222189
OR4K11P	0	0	0	0	0	0
ELMOD2	472.731843	587.094236	1296.79409	1146.49825	519.9248566	897.0002674
EGR3	0.97671868	10.1573397	2.005868661	5.02850108	246.8635463	34.43031329
C1orf101	11.7206242	9.14160575	12.03521197	6.03420129	1.007606311	4.530304381
GPC5	1.95343737	2.03146794	0	0	0	0
VWA1	723.748545	574.905428	216.6338154	209.185645	894.7544044	541.824404
LINC00174	48.8359342	49.7709646	519.5199833	334.898172	143.0800962	467.5274121
DNAJB8	0	0	0	0	0	0
GEMIN4	2860.80903	2748.57613	439.2852369	448.542296	2017.227835	1000.291207
HNRNPCL4	0	0	0	0	0	0
OR6W1P	0	0	0	0	0	1.812121752
AC073072.5	9.76718684	14.2202756	0	0	1.007606311	0
FJX1	2169.2922	1928.87881	244.7159767	375.12618	324.4492322	1028.379094
OR13C6P	0	0	0	0	0	0
RP5-1027G4.3	0	0	0	0	0	0
GM140	0	0	0	0	0	0
KLHL28	298.875917	291.51565	371.0857024	482.736103	712.3776621	668.6729266
MKRN3	25.3946858	22.3461474	53.15551953	41.2337088	8.06085049	47.11516556
ZBTB18	1975.9019	1651.58344	291.8538902	311.767067	59.44877237	135.9091314
EEF1A1P27	0	0	0	0	0	0
OR9A2	0	0	6.017605984	1.00570022	0	1.812121752
C14orf28	130.880304	103.604865	242.710108	183.037439	127.9660015	150.4061054
ALOX12B	9.76718684	17.2674775	13.0381463	17.0969037	4.030425245	19.93333928
SLC17A8	0.97671868	0	1.002934331	1.00570022	0	0.906060876
EIF3J-AS1	126.973429	134.076884	281.8245469	283.607461	134.0116394	212.9243059
SHARPIN	1126.15664	1134.57485	2104.156226	1771.03808	853.4425457	2072.161224
LBX2	91.8115563	113.762205	40.11737323	77.4389166	45.34228401	40.77273943

DNHD1	700.307296	954.789934	733.7567857	1481.39642	84.34672432	502.8637863
SLITRK4	42.9756221	34.5349551	563.6490939	273.550459	80.6085049	263.663715
HTR1D	48.8359342	48.7552307	0	0	29.22058303	8.154547886
GCC1	531.334964	612.487585	1136.324597	1308.41598	1197.036298	1275.733714
LSMEM2	4.88359342	2.03146794	8.023474646	4.02280086	3.022818934	1.812121752
NBPF17P	0	0	0	0	0	0
KB-1552D7.2	0	0	0	0	0	0
RNF151	0	0	0	1.00570022	0	0
CIITA	671.982454	592.172906	0	2.01140043	18.1369136	17.21515665
ZFPM1	175.809363	172.674775	462.3527265	464.6335	694.2407485	588.0335086
ALOX15B	0.97671868	0	0	0	1.007606311	2.718182629
PLD6	333.41269	462.575408	469.644059	531.029828	253.5943564	307.2180613
GPHB5	0	0	0	0	0	0
GRM8	0	0	1.002934331	0	0	5.436365257
CDC42EP4	1781.53488	1403.74435	1148.359809	1348.64399	1399.565166	1616.412603
DGKZP1	21.4292079	16.983072	16.84929676	13.7579789	41.33201089	25.32440149
OR2AP1	0	0	0	0	0	0
OR6C4	0	0	0	0	0	0
ZBTB42	134.787178	177.753445	1617.733075	1954.07552	1213.157999	2098.436989
LACC1	268.597638	504.545536	234.5261639	344.63335	267.8519857	286.3152369
MAF1	3438.04977	3461.62138	3165.260748	3687.90269	2817.267246	2848.655395
TPPP2	0	0	0	0	0	0
FCER1A	0	0	0	0	0	0
RPRML	13.6740616	2.03146794	0	0	1.007606311	2.718182629
ARL14	1.95343737	1.01573397	0	0	6.045637868	0
LINC00305	0	0	0	0	0	0
OR6C2	0	0	0	0	0	0
WDR97	43.9523408	53.8339005	76.22300913	92.5244198	72.54765441	49.83334819
NLRP8	0	0	1.002934331	0	0	0.906060876
PCED1B	20.5110924	16.2517436	429.2558935	261.482056	142.0724899	85.16972236
FLJ37453	35.1618726	35.550689	85.24941811	138.78663	102.7758438	83.35760061
APOBEC3B	926.173492	741.414698	581.1302392	1425.83148	181.2381472	1612.471238
SYCN	0	0	0	0	0	0.906060876

CTD-2144E22.5	0	0	0	0	0	0
PIPOX	3.90687474	6.09440383	2.005868661	2.01140043	3.022818934	8.154547886
ATP8B5P	0.97671868	4.07309323	1.002934331	5.02850108	1.007606311	1.812121752
FOXS1	0.97671868	0	0	0	3.022818934	2.718182629
ATOH7	3.90687474	6.09440383	0	0	1.007606311	0.906060876
CDH5	85.9512442	52.8181666	0	0	51.38792188	27.18182629
LRRC3B	0	0	0	0	0	0
OR7E22P	5.8603121	9.14160575	5.014671654	2.01140043	0	0
FAM216B	0	0	0	0	0	0
MRGPRX4	0	2.03146794	0	0	0	0
PCBP1-AS1	125.99671	151.344362	407.1913383	503.855808	200.5136559	441.2516467
MYADM	6358.43863	4905.99509	1603.691995	2037.54864	1466.067183	2145.552155
MRGPRX3	9.76718684	11.1730737	0	0	0	1.812121752
MROH1	1162.29523	1321.4699	2351.881006	2909.49072	1284.698047	1861.955101
SERTAD2	4484.11548	4162.47782	575.6843058	606.43723	468.5369347	725.7547618
PIK3CD-AS1	0	0	0	0	0	0
AKAP5	145.531084	135.092618	2233.534754	1018.77432	220.6657822	1672.588377
NKPD1	1.95343737	1.01573397	29.54644538	27.1539058	26.19776409	25.36970453
GIPC3	0	0	0	0	0	0
AC025335.1	89.8581189	87.3531216	97.28463008	77.4389166	23.17494516	66.14244396
CITED4	101.578743	112.746471	0	0	733.5373946	105.1030616
ABCA13	0.97671868	1.01573397	0	0	0	0.906060876
NLRP11	0.97671868	4.06293589	0	1.00570022	0	0
TIGD5	665.145424	648.038274	751.1978137	720.081354	616.6550625	748.4062837
PDXDC1	1760.99449	1934.10984	6161.196092	6492.74025	5755.44725	6579.877507
PHC1P1	53.1530308	62.2644925	58.11001512	83.804999	58.19934054	73.97080993
C1orf194	0	0	3.008802992	6.03420129	1.007606311	5.436365257
ZNF154	0	0	0	0	0	0
R3HDM2	1002.11337	897.360335	1999.851055	1680.46472	1740.579446	1448.274886
B3GNT3	61.5332771	94.4632594	0	1.00570022	405.0577371	50.73940907
ITLN1	0	0	0	0	0	0
NRXN1	0	0	0	0	0	0
SEPHS2	929.836187	1024.87558	7323.426483	6316.80305	3290.842213	4332.78311

OR10A7	0	0	0	0	0	0
GPBAR1	0	0	3.008802992	0	3.022818934	7.248487009
ZNF784	99.6253058	125.951013	129.3785287	151.860733	142.0724899	107.8212443
ZNF648	0	0	1.002934331	0	0	1.812121752
C14orf119	1571.54036	1679.00826	942.7582709	852.833783	1299.812142	889.7517804
CCR8	0	0	0	0	0	1.812121752
LINC00652	0.97671868	5.07866986	3.008802992	1.00570022	6.045637868	1.812121752
GOLGA8J	1.45531084	0	0	0	0	0
BBS10	557.706368	567.79529	278.8157439	198.122942	417.1490129	202.9576363
FIZ1	492.266217	459.111755	380.1121113	394.234484	393.9740677	327.0879763
PUF60	3563.06976	3497.17207	3675.754322	3623.53788	4801.244073	4360.870997
SSC5D	11.7206242	10.1573397	6.017605984	2.01140043	9.068456802	10.87273051
DCTPP1	917.138844	923.302181	1075.145603	2087.83365	3037.933029	2574.118949
ZNF771	184.599831	324.019137	349.0211471	315.789868	223.6886011	339.7728286
PPP1R14BP3	19.8762252	13.3975311	20.27933217	12.3499986	7.60742765	14.72348924
RP11-1319K7.1	98.3555715	72.2897868	65.09043806	169.611341	95.62183894	188.397238
CRIPAK	134.787178	184.863583	284.8333499	201.140043	93.70738695	169.4333838
TSHZ1	321.340447	261.043631	1062.107456	1131.41274	335.5329017	1025.660912
PSTK	151.391396	124.935279	91.26702409	115.655525	78.59329228	130.4727662
SPDYE7P	0.75207339	0	0	0	0	0
AC010894.4	0	0	0	0	0	0
SOCS4	1955.39081	1961.3823	1591.656783	1850.4884	1841.904337	2157.330946
ZADH2	718.864951	559.669419	1223.579883	1259.13667	871.5794593	1210.497331
RP11-756P10.3	28.4420481	31.9448334	14.69298794	35.4609896	12.01066723	28.06976594
OR1E1	0	0	0	0	0	0
AC079741.2	0	0	2.005868661	0	0	0
ZNF48	257.853733	238.697483	1293.785287	1123.36714	971.3324841	1192.376113
OR1R1P	0	0	0	0	0	0
FAM71E2	1.95343737	1.01573397	0	0	0	0
C3orf80	9.76718684	13.2045416	0	0	17.12930729	6.342426133
NKX2-6	0	0	0	0	0	0
TMEM150B	0	0	0	0	0	0
C10orf91	20.5110924	30.4720192	0	0	12.09127574	2.718182629

OR3A4P	0	0	0	0	0	0
ANKRD18A	238.358428	227.879917	741.47938	836.712408	417.3203059	486.8536906
WFDC11	0	0	0	0	0	0
TMEM86B	135.646691	151.00917	36.55695635	21.1901035	49.61453477	39.9119816
OR3A1	0	0	0	0	0	0
SEPT1	28.3248418	37.582157	162.4753616	237.586619	25.19015778	93.32427025
TRNAU1AP	410.221847	312.846063	563.6490939	452.565097	301.2742871	390.5122376
EXOC3	1456.28756	1544.93137	1469.298794	1671.47376	1602.094035	1675.30656
AC017081.2	0	0	0	0	0	1.812121752
TDRD6	13.6740616	9.14160575	1.002934331	0	0	0.906060876
C12orf40	0	0	0	0	0	0
CSNK1A1L	0	0	0	0	0	0
ACTA2-AS1	22.4645297	27.4248173	0	3.01710065	2.015212623	0
HMGN2P9	0	0	0	0	0	0
AC079753.4	3.90687474	7.11013781	0	2.01140043	0	0
LYNX1	18.557655	25.3933493	86.25235244	162.923435	9.068456802	67.95456571
RPS12P23	0	0	0	0	0	0
TH	0.97671868	5.07866986	1.002934331	0	94.71499326	4.530304381
FAR2P1	0	0	43.12617622	33.1881071	84.50794133	20.75785467
MED14	5249.86293	4653.07733	1479.328138	2034.53154	2196.581759	2062.194554
FAHD1	400.45466	485.520839	800.3415959	1554.81253	1091.237635	1110.830634
HMGB1P14	0	3.04720192	1.002934331	0	0	0
TDRP	290.085449	248.854823	1.002934331	0	5.038031556	1.812121752
RCC1	2561.64986	2312.69421	1634.62249	2166.16764	3362.724847	3039.970149
WFDC9	0	0	0	0	0	0
MYLPF	0	2.03146794	1.002934331	10.0570022	0	6.342426133
F2	0	1.01573397	0	0	2.015212623	0.906060876
RP1-278E11.3	3.90687474	0	2.005868661	0	3.022818934	1.812121752
FAM71C	0	0	0	0	0	0
TPT1P10	0.97671868	0	4.011737323	0	0	2.718182629
PRKRA	1081.22758	1027.92278	585.7136491	652.69944	1045.895351	708.5396052
HERC2P3	105.09493	59.7353149	132.397361	139.158739	44.223841	197.2403921
NACAP2	0	0	0	0	0	0

ZNRF2	348.68857	308.783128	2893.465544	3207.80152	689.0918802	2086.812228
RRH	0.97671868	3.04720192	5.014671654	8.04560172	0	3.624243505
SLC9A4	0	2.03146794	0	0	10.07606311	4.530304381
ZNF816	106.462337	160.587541	209.6132751	195.628806	227.7190264	236.7537069
PRNT	0	0	0	0	0	0
FGD6	1474.84521	1393.58701	535.5669326	481.730403	418.1566192	706.7274834
ADGRD2	0	0	0	0	1.007606311	0
GPR139	0	0	0	0	3.022818934	0
MGC45922	0	0	2.005868661	1.00570022	2.015212623	1.812121752
RP5-1055C14.6	0	1.01573397	0	0	0	0
PLD5	0	0	0	0	0	0.906060876
OAZ2	1433.82303	1654.63064	3493.220274	2314.1162	1410.648836	2821.473568
WFDC10A	0	0	0	0	0	0
PNPLA1	2.93015605	3.04720192	0	1.00570022	0	0
ALX1	0	0	0	0	0	0
CCDC43	921.045719	917.207777	1218.565212	1000.67171	921.9597748	1067.339712
KCTD4	30.2782792	31.4877531	0	0	0	0.906060876
C17orf104	11.290868	1.50328628	3.008802992	3.86188883	30.01659201	9.613305896
FZD2	2273.52761	2538.01448	378.1062427	329.869671	251.9015778	401.1493923
TIGD2	343.804977	392.073313	412.2060099	370.097679	282.1297672	502.8637863
CCDC129	0	0	0	0	7.053244179	0.906060876
HCLS1	55.672965	60.9440383	2.005868661	3.01710065	3.022818934	8.154547886
MTURN	420.692272	423.855629	877.5675394	995.713612	323.4416259	664.1426222
ZNF609	2247.42969	2383.92763	4414.916924	5006.37567	3533.675334	5981.813905
PAK2	7757.09979	6700.78686	3891.385203	8903.45395	6516.190015	7533.896185
CCDC66	496.173091	455.04882	574.6813715	396.245885	187.4147739	494.7092384
DEFB124	0	0	0	0	0	0
EMC3-AS1	175.809363	157.438766	371.105761	263.493456	552.1682586	531.8577343
KRTAP9-7	0	0	0	0	0	0
ATP5EP2	0.97671868	0	0	0	1.007606311	0
MCFD2	10536.8412	9014.639	4184.242028	2390.54941	4466.718778	2912.985717
OR10AA1P	0	0	0	0	0	0
LINC00304	0.97671868	1.01573397	52.1525852	8.04560172	18.1369136	29.90000891

HARBI1	85.9512442	107.667801	187.5487198	193.094441	151.1409467	191.1788449
DEFB123	0	0	0	0	0	0
C11orf71	89.8288174	66.794666	246.7017867	291.301067	116.5800502	232.2324632
CYP8B1	0	0	0	0	0	0
OR6K6	0	0	0	0	0	0
OR6K4P	0	0	0	0	0	0
TPRXL	0	0	0	2.01140043	2.015212623	0
SERTM1	0	0	0	0	0	0
GAS1	0	0	18.05281795	45.2565097	6.045637868	9.060608762
HMHA1	2165.38532	1720.65335	33.09683291	190.077341	238.8026958	136.8151923
CTD-3064H18.4	0	0	1.002934331	4.02280086	0	0.906060876
OR10Q1	0	0	1.002934331	0	0	0.906060876
ZNF571	52.7428089	48.7552307	250.0515873	240.362352	61.46398499	255.5091671
GLIPR1L2	0	1.01573397	6.017605984	10.0570022	2.015212623	1.812121752
DEFB119	0	0	0	0	0	0
FAM73A	691.516828	714.060982	720.1068494	836.742579	456.445659	791.8972058
KCNE1	22.1910485	33.9864587	1.002934331	4.02280086	1.803615297	3.624243505
PRR26	0	0	0	0	0	0
NRIP1	1893.85753	1255.44719	3832.212078	4380.83014	5744.363581	7455.929647
ZSCAN4	0	3.04720192	0	0	0	0
BHLHA15	0.97671868	6.09440383	0	0	14.10648836	4.530304381
RNF182	347.711851	348.396752	0	0	11.08366942	3.624243505
C9orf139	0	2.03146794	4.011737323	7.03990151	3.022818934	7.248487009
TSPYL5	273.481231	220.414272	0	0	2946.240854	220.1727929
FUT7	0	0	1.002934331	0	0	0
HIST1H2AC	348.68857	260.027897	978.8639068	930.272699	488.5580722	1236.773096
EIF2S3L	7.87235259	9.18223511	7.050628345	23.231675	9.12891318	10.02103329
SRP9P1	5.3426512	2.2244574	7.76271172	4.41502395	2.206657822	10.97239721
SKIDA1	14.6507803	20.3146794	2.005868661	7.03990151	109.8290879	7.248487009
HIST1H2BC	28.3248418	22.3461474	44.12911055	102.581422	116.8823321	231.9515843
ZBTB12P1	0	4.06293589	0	1.00570022	0	0
MB21D2	301.806073	194.005189	137.4020033	392.223084	638.8224014	223.7970364
GSX2	1.95343737	1.01573397	0	0	0	0

SSTR2	29.3015605	57.8968364	0	0	7.053244179	0.906060876
ZNF594	241.249515	225.492942	370.082768	254.442155	477.6053916	357.8940461
PCGF5	3356.98212	2974.06907	131.3843973	135.769529	920.9521685	132.2848879
SLC47A2	0	0	0	0	1.007606311	0.906060876
PRF1	0	0	0	0	0	0
OR2A4	0	0	0	0	0	0
MAB21L1	0	0	0	0	0	0
RPL21P8	0	0	0	0	0	0
VN1R3	0	0	0	0	0	0
YOD1	832.164319	692.730569	1233.609227	1218.90866	62.4715913	1676.212621
EXOC5P1	0	3.0675166	2.005868661	1.00570022	0	1.812121752
TMEM64	1044.11227	1179.26714	374.0945054	609.454331	21669.58133	2095.718807
C3orf22	2.93015605	1.01573397	0	0	0	0.906060876
OR10K2	0	0	0	0	8.06085049	0
RP11-290F5.2	1.95343737	1.01573397	3.008802992	10.0570022	2.015212623	9.966669638
OR5AZ1P	0	0	0	0	0	0
CHRM4	1.95343737	2.03146794	0	1.00570022	5.038031556	6.342426133
OR51A9P	0	0	0	0	0	0
SHISA2	632.913707	849.153601	181.5311139	190.077341	92.69978064	522.7971256
S1PR5	44.9290595	41.6450929	0	6.03420129	47.35749663	18.12121752
CLRN3	0.97671868	0	0	0	0	0.906060876
SMG1P3	1169.08343	1077.32808	1386.305979	1642.66045	1666.530459	1834.302123
GPR157	448.313876	500.756848	686.0070822	981.56341	746.6362767	641.4911003
PIPSL	14.9242615	9.20254979	7.040599002	11.1632724	15.14432286	11.84221565
CHST13	0.97671868	2.03146794	0	0	0	0
WDFY3-AS2	44.9290595	42.6608268	88.2582211	117.666925	67.50962286	94.23033112
OR7E129P	1.95343737	0	0	0	0	0
AGTR2	0	0	0	0	1.007606311	0
SLC36A4	1346.89507	1220.91223	227.6660931	402.280086	172.3006792	533.6698561
ZDHHC20	3626.55647	3434.19656	2794.175045	3928.25498	3565.918736	3872.495124
ANKRD30B	2.93015605	5.07866986	160.3691995	122.695426	0	113.2576095
OR51E1	0	0	0	0	0	0
ZFP3	66.4168705	69.0699101	312.9155112	306.738566	462.4912969	389.6061768

ARSJ	839.978068	733.359928	250.7335827	301.710065	58.44116605	263.663715
RP11-467D10.2	0	0	0	0	0	0
HOXC9	1.95343737	5.07866986	296.8685619	220.248347	120.9127574	325.2758545
MAP3K15	308.643104	178.769179	2.005868661	4.02280086	8.06085049	6.342426133
PPA1	4353.22541	4241.70507	2784.145702	3203.14513	3467.173317	3248.21918
HOXC10	0	0	2344.860465	2853.26202	28.21297672	2662.53237
PSMG4	480.545592	512.945656	291.8538902	192.088741	411.103375	298.0940283
BHLHE22	0	0	0	0	0	0
MAP6D1	271.527794	320.971935	49.1437822	100.570022	314.3731691	163.0909577
CSNK1G2-AS1	0	0	0	0	0	0
ZNF443	115.653259	78.9733163	289.8480216	241.006	220.000762	291.1173595
LINC01559	0	0	0	0	0	1.812121752
PDIA3P1	71.8083576	90.0752887	277.0806675	154.737035	138.8884539	152.5262879
LINC01555	0	0	1.002934331	0	0	6.342426133
CXCR2	0.97671868	0	0	0	1.007606311	2.718182629
DEFB112	0	0	0	0	0	0
GREM2	2.93015605	1.01573397	0	0	0	0.906060876
C11orf42	0	0	0	0	0	0
SSR4	1188.66664	1392.57128	6538.128902	4409.99545	4048.562159	4417.952832
CAPS2	86.9279629	58.9125704	94.27582709	56.3192121	42.31946507	67.04850484
ZNF792	323.293884	370.7429	163.4782959	169.963336	171.2930729	212.018245
CUEDC1	1440.66006	1250.36852	784.2946466	569.226322	1389.489103	1155.227617
SCRIB	4673.5989	4686.59655	3052.932103	3740.1991	6710.658033	5277.804604
KCTD2	1031.41493	1155.90526	4265.479709	4490.45146	1208.119967	5970.035113
D2HGDH	254.923576	183.847849	1238.623898	1045.92822	1026.750831	1237.679157
OR52B1P	0	0	0	0	0	0
TTTY11	0	0	0	0	0	0
OR56B3P	0	0	0	0	0	0
OXTR	462.964656	203.146794	30.08802992	20.1140043	71.5400481	61.61213958
CMTR2	623.14652	610.456117	1329.890923	1178.68065	995.5150356	1144.354887
OR56B4	0	0	0	0	0	0
FAM83H	975.741965	864.38961	5683.628852	9924.24973	8046.744002	7361.744619
OR7E18P	0	0	0	0	0	0

GPR62	0.97671868	4.06293589	0	1.00570022	0	0.906060876
OR56A1	0	0	0	0	0	0
ZNF572	2.93015605	6.09440383	0	0	386.9208235	28.08788716
ST20	46.4039047	79.1053618	48.13081853	85.3437203	151.0401861	25.04352262
PITPNB	2871.55293	2804.4415	2192.414447	2277.91099	2174.41442	3210.173684
TCEAL8	605.565584	771.957819	0	0	27.2053704	25.36970453
OR52E4	0	0	0	0	0	0
LRRC57	641.704175	618.581989	475.3908728	497.821607	309.3351376	518.2668212
OR52N2	0	0	0	0	0	0
MRPL14	2519.9342	2304.70038	949.7788112	875.964888	1116.427793	1019.318486
GPR137C	239.296078	275.263906	125.3667913	416.359889	115.8747258	156.7485316
C1orf105	0.97671868	0	0	0	0	0
OR52N1	0	0	0	0	0	0
BBS12	91.8115563	118.840875	68.19953449	105.598523	51.38792188	89.70002674
ZFP82	352.595445	393.089047	0	0	20.15212623	2.718182629
OR52N5	0	0	0	0	0	0
C17orf47	0	0	0	0	0	0
LSMEM1	20.5110924	22.3461474	62.1819285	51.290711	36.27382721	50.73940907
OR56B2P	0	0	0	0	0	0
NQO1	3900.0377	6026.34966	19863.11442	17805.9223	18024.0617	14034.88297
OR56B1	0	0	0	0	0	0
AEN	1384.01038	1605.87541	304.8920365	255.447855	2026.296292	910.5911806
FKRP	974.765246	990.340623	355.0387531	342.943774	337.5481143	462.9971077
TRAPPC5	896.627752	949.711264	1580.624505	1232.98846	855.4577583	985.7942333
RPH3AL	865.372754	1001.5137	2.005868661	0	235.7798768	19.93333928
SLC25A42	142.600928	207.20973	151.4430839	155.883533	277.0917356	252.7909845
FCRL6	1.95343737	0	1.002934331	0	0	2.718182629
METTL23	670.029017	832.901857	629.8427597	693.933149	435.2859265	552.6971345
SLC26A11	350.642008	358.554092	865.5323274	797.520271	513.8792188	766.5275012
HIGD1A	2363.68852	2497.99456	1964.116505	1449.99846	1402.708898	2140.786275
CHRM2	0	0	0	0	0	0
OR52N4	0	0	0	0	0	0
MAPK15	16.6042176	17.2674775	8.023474646	1.00570022	21.15973254	9.966669638

EHMT1	1867.48612	1768.39285	5561.270864	5202.48722	3346.26056	5379.283422
ADIPOQ	0	0	0	0	1.007606311	0
RP11-429J17.2	33.2084353	38.5978909	20.05868661	26.1482056	19.14451991	31.71213067
SDCCAG3P2	0	1.01573397	0	0	0	0
F2R	1879.20675	1711.51174	1.002934331	2.01140043	52.39552819	26.27576541
OR52P1P	0	0	0	0	0	0
RP4-539M6.14	0	0	0	0	0	0.906060876
HLA-V	0.97671868	0	0	5.02850108	4.030425245	6.342426133
ZNF707	349.665289	338.239413	509.49064	575.260523	465.5141158	641.4911003
MUC16	0.97671868	1.01573397	0	0	101.7682374	6.342426133
NPM1	33288.7704	32211.7277	15361.09265	16334.3114	35448.9503	16987.6357
FER1L6-AS1	0	0	0	0	0	0
PJA1	431.709658	485.520839	545.5962759	480.724703	363.7458784	451.2183163
DHTKD1	1251.17663	1114.26017	3144.199127	2900.43942	4466.718778	3573.504096
PENK	0	0	0	0	0	0
HIST3H2BA	0	0	0	0	0	1.857424796
HECW1-IT1	0	0	0	0	0	0
OR8G2P	0	0	0	0	0	0
C4orf50	0	0	0	0	0	0
HIST3H2A	0	0	1540.507132	2730.47609	426.2174697	933.2427025
ZNF746	770.523603	770.373274	836.6678774	912.79363	1226.377794	928.8392466
POLR2A	6286.16145	5942.04374	2479.253666	3592.36117	9766.727975	5474.419814
RP4-682C21.2	4.89336061	2.03146794	0	2.01140043	0	2.718182629
TMEM132C	0	0	0	0	0	0
SLC25A41	419.012315	424.5768	12.03521197	17.0969037	3.022818934	9.060608762
MTHFD2P7	0	0	1.002934331	0	0	0
TMEM136	612.402615	528.181666	260.762926	280.59036	128.9736078	237.3879496
OR5AK2	0	0	0	0	0	0
FRAT2	318.410291	389.026111	1242.635636	1362.57294	560.2291091	920.5578502
OR5AK3P	0	0	0	0	0	0
TMEM102	163.11202	172.674775	160.4694929	255.447855	295.2286492	245.5424974
TMEM132E	0	0	6.017605984	11.0627024	0	7.248487009
OR5G1P	0	0	0	0	0	0

ZNF322	1034.5795	1080.74095	811.6647245	911.164395	800.1502479	937.7730068
NME9	2.93015605	0	0	0	2.015212623	0
SPEM1	0.97671868	0	0	2.01140043	0	0
OR9G3P	0	0	0	0	0	0
HEPHL1	0	0	1.002934331	0	0	0.906060876
LRRC75A	135.763897	164.548904	55.16138819	101.575722	117.8899384	50.73940907
OFCC1	0	1.01573397	1.002934331	0	1.007606311	1.812121752
CTAGE10P	0	0	0	1.00570022	0	0
HSP90AA6P	0	0	0	0.9252442	1.007606311	0
OR5M8	0	0	0	0	0	0
CCL13	0	0	0	0	0	0
CCDC108	0	0	17.04988362	24.1368052	0	16.30909577
DDX60L	886.860565	742.501534	64.18779717	84.4788181	70.53244179	332.5243416
SYNE4	8.79046815	15.2360096	663.9425269	1024.80852	359.7154531	630.6183698
OR5AL1	0	0	0	0	0	0
OGFOD3	1250.19992	1239.19545	622.8222194	541.066716	989.4693977	663.2365614
WASH1	225.807593	191.120504	511.8174476	435.920758	87.50053207	373.0796264
UTS2R	0	0	0	0	0	0
AATK	4.88359342	2.03146794	15.04401496	34.1938073	52.39552819	19.93333928
DDN	57.6264023	63.9912403	36.10563591	41.2337088	20.15212623	90.60608762
SAGE1	0	0	0	0	0	0
ZNF467	29.3015605	22.3461474	1939.674996	2819.9834	1997.075709	2063.100615
SOX2	0	3.04720192	7.020540315	6.03420129	1041.864926	58.89395695
ZNF678	318.410291	283.389778	836.4472318	874.959188	468.5470108	1097.239721
TMEM45A	102.555462	179.784913	85.24941811	42.2394091	24.18255147	36.24243505
RAP2B	1761.02379	1798.86486	592.7341894	858.867984	2335.63143	1015.694242
ZBTB2	891.744158	795.3197	507.4847713	553.135119	1221.218849	723.9426401
RNF135	636.820582	615.534787	554.6226849	775.394866	399.0120993	657.8001961
OR6T1	0	0	0	0	0	0
RP4-655L22.4	0	0	0	0	0	0
ACBD4	302.782792	223.461474	413.2089443	470.667701	109.8290879	347.9273765
RP3-474G15.1	0	0	0	0	0	0
OR8D4	0	0	0	0	0	0

SGSH	1241.40945	1190.44022	1426.172618	1221.92576	1191.998266	1292.94887
RPL24P4	39.1273505	26.4801847	31.171199	22.1555757	42.42022571	21.80888529
MAB21L2	0	0	0	1.00570022	23.17494516	0
FANCB	302.782792	275.263906	91.26702409	124.706827	258.954822	167.6212621
EDDM3B	0	0	0	0	1.007606311	0
SETD2	4040.6852	3725.71221	2687.864006	2917.53633	2588.540614	4130.731534
EDDM3A	0	0	0	0	0	0
C6orf223	0.97671868	0	20.05868661	15.0855032	0	13.59091314
TMIE	4.88359342	6.09440383	1.002934331	0	2.015212623	0
MEX3D	2045.24892	1995.91726	1727.052917	1945.02422	1555.744145	2146.458216
OR52D1	0	0	0	0	0	0
MRPS23	1017.74087	1089.88255	534.5639983	374.12048	3182.020731	684.9820224
OR52H1	0	0	0	0	0	0
FDCSP	0	0	0	0	0	0
GPR135	290.085449	242.506486	199.9650469	144.58952	127.9660015	214.2471548
SLX1B	647.427747	442.453718	793.4012903	1195.27471	2027.334127	1238.404006
ANKRD62	0	0	0	0	0	0.906060876
P2RY13	0	0	0	0	0	0
TNFSF15	896.627752	1006.59237	25.07335827	8.04560172	86.65414277	86.07578324
ZFP41	587.59396	590.375057	728.4612924	577.774774	520.8317023	724.8487009
PHLDA2	2336.31109	2690.67929	253.7423857	790.480369	1901.353109	994.854842
ATG9B	8.79046815	2.03146794	50.14671654	67.3819144	12.09127574	117.7879139
GPR88	0	0	0	0	0	0
HKR1	459.057781	364.648496	1354.964281	985.586211	530.0009197	976.7336245
OR8K3	0	0	0	0	0	0
PLAG1	231.482328	199.083859	10.02934331	4.02280086	19.14451991	7.248487009
OR8H1	0	0	0	0	0	0
OR5T1	0	0	0	0	0	0
YIPF6	1659.44504	1844.57289	3018.832335	2247.73998	1857.018432	1417.985271
RP11-11L12.2	0	0	0	0	0	0
OR5T2	0	0	0	0	0	0
ZBTB20	41.0221847	34.5349551	4.011737323	2.01140043	8.06085049	1.812121752
OR2Z1	0	0	0	0	0	0

FDX1P1	0	0	0	0	0	0
C3orf58	443.430282	458.096021	167.4900332	349.983675	969.3172715	353.3637417
C5orf30	727.655419	627.723595	1569.592228	1850.4884	773.8416471	1259.424618
OR8K5	0	0	0	0	0	0
AMIGO1	374.083256	393.089047	55.16138819	25.1425054	507.8335809	137.7212532
OR8H3	0	0	0	0	0	0
OR8H2	0	0	0	0	0	0
GPR3	189.483425	214.319868	24.07042394	21.1197045	20.15212623	21.74546103
TMEM252	0	0	0	0	0	0
OR5J1P	0	0	10.02934331	8.04560172	0	7.248487009
ODF3L2	0	0	3.008802992	4.02280086	0	0
OR5AS1	0	0	0	0	0	0
ACTL9	0	0	0	0	0	0
SIAH2	613.379333	587.094236	687.0100165	636.608236	2750.76523	787.3669014
COPG1	3608.97554	3854.71042	9247.054529	6065.378	6140.352861	5676.471389
ADGRB1	1.95343737	6.09440383	108.3169077	80.4560172	98.74541851	86.98184411
LINC00471	7.81374947	5.07866986	26.0762926	17.0969037	7.053244179	19.93333928
CELF2-AS1	0	0	0	0	0	0
OR6S1	0	0	0	0	0	0
SLC9A9	4.88359342	18.2832115	6.017605984	1.00570022	2.015212623	5.436365257
LSM10	434.639814	478.410701	796.3298586	505.867208	548.1378333	650.5517091
KCTD9P2	1.37717334	3.17924733	2.035956691	0	1.047910564	4.648092295
RELL1	294.969043	271.200971	57.16725685	171.974737	176.3311045	115.9757922
RFX7	1162.29523	1199.58182	3113.108163	6655.72403	1280.667622	6857.974772
SLC35C1	583.101054	756.721809	399.1678636	603.420129	1887.246621	1009.351816
OR5D17P	0	0	0	0	0	0
TIGIT	0	0	0	0	0	0
RNF41	876.116659	794.303966	2252.590507	1163.59515	1098.290879	1487.751959
SLC2A4	19.5343737	19.2989455	3.008802992	1.00570022	33.25100827	9.966669638
FTMT	0	0	0	0	0	0.906060876
IBA57	281.294981	300.657256	438.2823025	730.138356	717.4156936	781.9305361
CLDN7	598.728553	436.765608	2544.444397	2013.41183	2701.392521	2684.658376
ZNF329	167.995614	183.847849	445.3028428	441.502395	422.1870444	540.0122822

ZNF101	335.014509	332.145009	395.1561263	512.90711	519.9248566	437.6274032
OR4C6	0	0	0	0	0	0
C5orf24	2137.06048	1930.36178	1249.656176	1064.57391	1390.49671	1366.140468
AP003774.4	0.97671868	2.03146794	0	1.00570022	5.038031556	1.812121752
ADO	1231.64226	1201.61329	1059.098653	760.309363	1089.222423	910.5911806
COA4	1286.33851	1486.0188	1136.324597	1010.72872	2205.650215	1286.606444
OR4P4	0	0	0	0	0	0
PRKAG1	974.765246	1051.03073	1437.886891	1369.91455	1242.519647	1255.673526
OR4C16	0	0	0	0	0	0
GINS3	454.174188	421.529598	240.7042394	359.034977	634.7919761	561.7577432
OR4C15	0	0	0	0	0	0
OR4A21P	0	0	0	0	0	0
OR4A13P	0	0	0	0	0	0
OR4A15	0	0	0	0	0	0
OR4A16	0	0	0	0	0	0
OR52K2	0	0	0	0	0	0
NEUROG1	0	0	0	0	0	0
PYY3	0	0	0	0	0	0
CCDC149	110.369211	119.856609	1359.978952	2503.18784	277.0917356	1118.985182
GOLGA8CP	0	2.48854823	0	0	0	0
MRPS11	706.167608	719.139652	847.4795094	689.910348	1154.716833	771.0578056
LINC00301	0	0	0	0	0	0
AQP7P2	0	0	0	0	0	0
RP11-651P23.2	0	0	0	0	0	0
SNRPE	1202.3407	1196.3721	1907.510892	1729.80437	2372.912863	2035.819122
RTKN2	314.503416	246.823355	292.8568246	452.565097	292.2058303	663.2365614
PNMAL1	974.765246	777.036489	0	0	13.09888205	6.342426133
RP11-381O7.3	5.05940278	5.76936896	0	0	0	0
CHST15	3290.56525	2755.68627	2340.848728	3157.89868	1533.576806	2931.106934
ADIG	0	0	0	0	0	0
USH1G	0.97671868	0	4.011737323	5.02850108	6.045637868	26.27576541
TRPC2	0	0	1.002934331	0	0	0
MGAT4C	0	5.07866986	0	0	0	0

TRIM49B	0	0	0	0	0	0
IDH2	1310.75647	900.956033	886.5939483	1010.72872	6062.767175	1321.036757
OGFRP1	9.76718684	12.1888077	16.04694929	20.1140043	18.1369136	31.71213067
OR52A1	0	0	0	0	0	0
PTCHD3	0	0	0	0	0	0
OR6B2	0	0	0	0	0	0
TMEM259	3979.15192	4236.6264	4270.49438	3593.36687	2187.513302	4079.086065
WRB	417.058878	365.66423	744.1772734	544.083817	1357.245701	675.9214136
TNRC18	5732.36196	5403.70473	11597.86239	8499.14235	7801.895668	14277.6801
FAM181B	0	0	0	0	0	0
TMEM30B	14.6507803	7.11013781	3286.615802	4855.52064	2209.680641	2965.537248
DEXI	449.290595	447.938682	593.7371238	572.243423	757.7199461	618.6221238
RP11-69E11.4	2.93015605	3.04720192	38.79349991	33.3188481	10.4891817	25.57809853
ZNF716	0.97671868	0	0	0	16.12170098	0
NOP10	1287.31523	1319.43843	1958.730748	1733.82717	1307.872992	1107.206391
FAM89A	242.226234	230.571612	0	0	112.8519069	21.74546103
KCNIP1	0	0	0	0	0	0
TDRKH	249.063264	351.443954	469.3732668	1156.55525	851.427333	694.948692
ZNF708	159.205145	161.501702	353.0328844	535.032515	329.4872638	629.9207029
IST1	1929.98635	1844.56274	2166.338154	1889.71071	3402.666361	2428.234088
ERCC6L2	937.649937	983.230485	317.9301828	375.12618	1038.842107	371.4849592
MRPL41	983.555715	1126.44898	4854.202161	4798.19573	2326.562973	3285.376737
ENPP7	0	0	0	0	0	0
CREB3L2	3612.88241	3450.4483	3659.707373	2161.24976	2728.597891	2232.533999
P2RY8	0	1.01573397	0	0	0	0
TP53TG1	7.81374947	9.14160575	668.9571986	416.359889	236.7874832	490.178934
UNC5C	0	1.01573397	3.008802992	1.00570022	54.41074081	29.90000891
MRGPRG	0	0	0	0	0	0
TSEN54	635.843863	611.471851	1481.334006	1832.38579	3209.226101	2467.765524
RGMA	0	0	0	0	13.09888205	0.906060876
ASB18	0	0	0	0	0	0
UBA7	487.382623	488.568041	12.03521197	9.05130194	6.045637868	117.7879139
MRPS16	3180.19603	3039.07604	2209.464331	2239.69438	2918.027877	2501.634079

FAM159A	0	0	0	0	0	0
RAD51B	152.368115	133.06115	408.1942726	319.812669	145.0953088	443.0637685
CRYGB	0	0	0	0	0	0
LDOC1	435.616533	476.379233	1566.583425	2358.36701	2426.315998	1638.158064
ARL6IP4	2602.95529	2596.44965	1830.55574	1832.94898	2207.977786	1811.967722
EXT1	11171.7083	9692.13356	597.7488611	634.596836	802.0546238	1068.245773
SHMT2	4611.08891	3962.37823	8749.599101	3375.12992	5191.187716	4213.183074
MOB2	462.964656	429.65547	483.4143474	445.525195	185.3995613	333.4304024
HHIPL1	0.97671868	0	8.023474646	0	7.053244179	1.812121752
ATP6AP2	2475.98186	2679.50622	3363.841745	3288.6397	2779.985813	2869.494795
ZAR1	0	0	0	0	0	1.812121752
CYB5D1	223.268124	236.676173	384.2843182	271.408317	780.0384259	467.1559271
FAM153B	0	0	0	0	1.249431826	0
BACE2	3417.53867	2795.29989	15.04401496	23.131105	1459.013939	215.6424885
UBE2E2	616.30949	622.644925	527.543458	474.690502	669.0505907	593.4698739
SYNM	1200.38726	1477.89293	368.0768994	597.385928	313.3655628	732.097188
KCNA4	0	0	0	0	0	0
GABRG3	0	0	0	0	0	0
PRR34	1.95343737	1.01573397	5.014671654	12.0684026	1.007606311	5.436365257
NLRP10	2.93015605	0	0	0	0	0
FIGN	820.443694	818.681582	26.0762926	33.1881071	1629.299405	84.26366148
IZUMO1	0.97671868	2.03146794	2.005868661	1.00570022	25.19015778	0.906060876
TMIGD1	0	0	0	0	0	0
B4GALNT4	497.14981	421.529598	1097.210158	955.415205	1031.788863	2155.518824
AP1S2	2447.23703	2872.98323	266.7303853	242.243011	474.4515838	251.7490144
C8orf33	1368.38288	1306.23389	1640.800565	1148.50965	1917.47481	1770.442952
DCAF4L1	9.76718684	8.12587178	32.09389858	27.1539058	8.06085049	36.24243505
SPACA6P	101.578743	86.3373876	268.7864006	346.966574	113.8595132	241.0121931
MBD3L3	0	0	0	0	0	0
ZSCAN22	202.180768	219.398538	112.328645	162.923435	218.6505695	200.2394536
KCNJ14	139.729375	155.671389	35.10270157	42.2394091	62.4715913	49.83334819
FBXL6	927.999956	766.503327	715.653821	915.086626	694.1399878	1537.512822
C1S	24.4179671	28.4405512	43.20641097	24.1368052	47.35749663	67.06662605

GLTPD2	0	0	3.008802992	6.03420129	9.018076486	7.248487009
KIAA2012	0	0	0	0	0	0.906060876
PRAMEF8	0	0	0	0	0	0.906060876
LIPF	0	0	1.002934331	0	0	0
OR5P3	0	0	0	0	0	0
DAOA	0	0	0	0	0	0
PDSS1P1	0	0	0	0	0	0
ZNF804B	0	0	0	0	8.06085049	0
CRIP1P4	0	0	0	0	0	0
C17orf77	0	0	0	0	0	0
KBTBD3	80.0909321	97.5104613	168.4929676	119.678326	23.17494516	115.9757922
YBEY	197.297174	226.508676	228.6690274	253.436454	153.1561593	162.1848968
OR5F2P	0	0	0	0	0	0
FAM87A	0	2.03146794	0	0	0	0
CLN8	306.689667	323.003403	337.9888694	368.086279	1462.036758	462.0910469
RP5-1142A6.8	0	0	0	3.01710065	0	2.718182629
PLCXD1	171.902488	146.773559	663.4410598	574.757673	227.7190264	721.6774879
NXPH4	96.6951497	184.863583	416.2177472	197.117242	89.6769617	260.0394715
RPL27AP5	0	0	0	0	0	0
CACNB4	172.879207	168.611839	20.05868661	2.01140043	29.22058303	24.46364366
IFNL1	0	5.07866986	1.002934331	2.01140043	2.015212623	17.21515665
DNM1P46	0	0	0	0	0	0
TRAPPC6B	1033.36837	1088.86682	1411.128603	1306.40458	966.2944525	1466.912559
PGBD4	86.9279629	76.1800479	181.5311139	150.855032	185.3995613	237.3879496
CDY2A	0	0	0	0	0	0
NPLOC4	6387.74019	6015.17658	3411.982593	3241.37179	6779.175262	4840.177201
OTOL1	0	0	0	0	0	0
KCNK4	1.95343737	0.87353122	0	1.00570022	2.015212623	0.906060876
TEX19	0.97671868	3.04720192	72.21127181	22.1254047	54.41074081	125.9424618
TSHZ2	0	0	0	0	1.007606311	0
CAPN12	13.6740616	12.1888077	1.002934331	0	29.22058303	8.154547886
EXOC7	4469.4647	4458.0564	8413.6161	7668.46414	4307.516981	8946.445091
OR2B8P	0	0	1.002934331	0	0	0

KPNA2	9606.48731	10279.9185	7328.691888	16326.9798	15649.50643	17025.9802
WASH6P	288.141779	253.120906	445.9647795	413.041079	169.7212071	388.2289642
NCF1B	0	0	0	0	0	0
XKRX	1.95343737	4.06293589	5.014671654	17.0969037	52.39552819	14.49697402
BGN	0	0	66.19366583	8.04560172	0	103.2909399
CEP97	1490.47271	1463.67265	360.0534247	542.072416	412.1109813	524.6092473
LHFPL1	0	0	5.014671654	4.02280086	0	3.624243505
FES	4.88359342	4.06293589	3.008802992	0	6.045637868	5.436365257
GLRX5	1434.79975	1335.69017	1591.656783	1771.03808	2257.038137	1711.548995
FAM104B	108.415774	126.966747	231.6778304	246.396553	149.1257341	197.521271
TBPL2	0	0	0	0	0	0
OR7E115P	0	0	0	0	0	0.906060876
CAV3	0.97671868	2.03146794	0	0	0	0
MXRA7	3950.42662	3273.27383	12.03521197	5.02850108	281.2733018	96.04245287
LIMK2	408.278177	354.501314	6220.319071	7515.59771	914.9367588	6697.6473
MFSD5	545.009026	672.41589	662.9395926	701.97875	1058.994233	608.8729088
RNASE10	6.83703079	3.04720192	1.002934331	2.01140043	0	0.906060876
ADI1	3424.37571	3727.74368	2666.802385	2444.85722	3371.450718	2355.758278
RWDD4	768.570165	690.556898	747.1860764	655.716541	297.1733294	725.5916709
SPNS3	3.90687474	5.07866986	0	0	0	0
OR4C2P	0	0	0	0	0	0
CLEC4G	0	0	0	0	0	0
SATB1	269.574357	366.679964	59.17312551	47.2679101	20.15212623	8.154547886
RP3-341D10.1	0	0	0	0	0	0
NXPH3	6.83703079	9.14160575	34.09976724	173.986137	80.6085049	115.0697313
CSF1R	51.7660902	121.888077	0	0	3.022818934	0
EPHB3	323.293884	370.7429	2008.877464	2072.74814	688.1951106	1530.33682
VCX	0	0	3.008802992	7.03990151	1.007606311	1.812121752
ACTL10	13.6740616	18.2832115	93.27289276	213.208446	122.92797	144.9697402
EPGN	0.97671868	0	0	0	0	2.718182629
LINC00334	0	0	1.002934331	1.00570022	0	9.060608762
KRTAP11-1	0	0	0	0	1.007606311	0
C2orf82	50.0959013	36.9422446	40.95983807	32.453946	28.52533467	49.83334819

HS3ST4	0	0	0	0	0	0
TRAK1	2831.50746	2638.87686	779.279975	671.807744	972.3400904	835.3881278
TSPAN10	28.3248418	55.8653685	24.07042394	39.2223084	25.19015778	43.49092206
OR2V2	0	0	0	0	0	0
PLCB1	0	0	358.0475561	241.368052	1081.161572	335.2425242
RP11-123C21.1	0	0	0	0	0	0
SKA2	2233.74586	2005.05886	1280.737111	1660.401	4385.102667	1503.154994
RXFP3	0.97671868	1.01573397	0	0	0	0
CCNYL2	0	0	0	0	0	0
OR10G7	0	0	0	0	0	0
NDN	0	0	0	0	0	0
CCDC172	0	0	0	0	0	0
LINC01006	184.599831	179.784913	93.27289276	71.4047153	1584.964728	118.6939748
OR4Q3	0	0	0	0	0	0
NTM	11.7206242	4.06293589	0	0	0	0
TTC3	6204.06824	6287.3425	11006.18129	10608.0957	5615.722483	14214.21054
KCNB2	0	0	0	0	0	0
PPP1R27	0.97671868	0	0	1.00570022	0	0.906060876
BRICD5	41.9989034	48.7552307	327.9595261	1007.71162	184.391955	214.7364277
GALR2	3.90687474	7.11013781	10.02934331	20.1140043	9.068456802	9.966669638
RESP18	0	0	0	0	0	0.906060876
IGIP	234.412484	232.60308	172.5047049	143.815131	79.60089859	105.1030616
TSKU	829.234163	911.113373	7112.810273	3623.53788	3673.732611	6983.917234
FXYD6P3	0	0	0	0	0	0
CMC4	121.542873	118.404109	556.0267929	824.985944	329.5880244	590.642964
ANXA2	27294.345	22154.8644	13923.55679	18548.9537	13336.29425	15391.65477
SEPHS1P1	0	0	0	0	0	0
RGS6	0.97671868	4.06293589	3.008802992	0	35.2662209	20.83940015
HOXB4	11.1150586	21.9093818	0	0	3.022818934	1.812121752
SLC35D3	0	0	0	1.00570022	0	1.812121752
PAQR7	522.544496	505.835518	9.026408976	16.0912034	126.9583952	64.33032221
PAPPA	15.6274989	11.1730737	0	1.00570022	5.038031556	1.812121752
MAFA	9.76718684	3.04720192	4.011737323	19.1083041	142.0724899	25.36970453

NGRN	3246.93522	3538.78669	1901.73399	1357.01141	5067.58465	2737.463604
GRID1	0	0	0	0	10.07606311	1.812121752
RPS17	13829.3598	13731.7076	6960.334167	4862.52031	19186.52702	5983.544481
RP11-114H20.1	0	0	0	0.50285011	0	0
HCAR2	0	0	16.51832843	23.0506489	17.11923123	13.87179201
OR2T29	0	0	0	0	0	0
CCDC87	7.81374947	5.07866986	10.02934331	19.1083041	32.24340196	35.33637417
GSTA5	0	0	0	0	0	0
C1orf116	23.4412484	22.3461474	1457.263583	1317.46728	42.31946507	1304.727662
TMEM198B	181.669675	138.13982	434.2705652	327.85827	321.4264133	438.5334641
MAGEB17	0.97671868	1.01573397	0	1.00570022	0	1.812121752
CRIP2	50.7893716	50.7866986	319.9360515	802.548772	4429.437344	1243.115522
DDX28	317.433572	297.610054	413.2089443	337.915272	545.1150144	464.8092295
FUNDC2P2	0	0	0	0	0	0
KRTAP13-2	0	0	0	0	0	0
AC008132.15	0	0	0	0	0	0
ACBD3	1709.2577	1965.44524	3497.232011	3114.65357	1705.877485	3135.876692
C16orf72	2961.41105	2774.98521	4987.522221	4371.77884	4222.878051	5555.965293
PLCXD3	0	0	2.005868661	1.00570022	65.49441023	3.624243505
RRP7BP	229.694933	218.392961	620.4252063	257.177659	213.0281263	642.4605855
VMO1	1.95343737	1.01573397	39.1144389	100.570022	12.09127574	50.73940907
OR4F15	0	0	0	0	0	0
ALG12	571.38043	567.79529	1442.219568	1504.52752	536.0465576	1392.615567
LCK	1.95343737	0	0	0	0	1.812121752
GALNT9	0	0	0	0	0	1.812121752
COL18A1	5270.37402	4276.24002	100.2934331	44.2508095	4770.008278	733.0032488
RBM10	2448.63374	2309.77905	2236.543557	1989.27503	2767.894537	2221.661268
RP11-181G12.2	21.487811	12.1888077	87.25528677	23.131105	8.06085049	45.30304381
ADGRG3	0	1.01573397	0	1.00570022	0	1.812121752
RP11-558O12.1	0	0	0	0	0	0
GLUD2	24.8574905	19.5935083	22.44567032	24.4888002	121.4367126	42.41270961
TMEM95	0	0	0	0	0	0
TCHHL1	0	0	0	0	5.038031556	4.530304381

RPL35A	7729.75166	7449.39295	11282.00829	9184.05437	7531.857177	7933.469032
RGS7	0.97671868	1.01573397	0	0	0	0
SLC25A18	0.97671868	1.01573397	0	0	3.022818934	4.530304381
ZNF721	661.248316	620.725188	1021.177706	918.39538	858.611566	1372.86344
TSPEAR-AS2	1.95343737	7.11013781	3.008802992	0	1.007606311	1.812121752
TCEAL7	0	0	0	0	0	0
C11orf54	535.241839	591.157172	1632.77709	1332.55279	155.1713719	1027.473034
CCDC75P1	0	0	0	0	0	0
CEP63	547.939182	500.756848	765.2388943	620.517033	566.2747469	617.9335176
WFDC10B	0	2.03146794	0	0	0	0
SRPR	4686.29625	5177.19606	4390.8465	2686.22528	3296.887851	3572.598035
OTOP3	0	0	0	0	0	0
EWSR1	7002.07671	6816.57037	11137.56568	7465.30264	8786.256502	9693.909072
ODF3L1	0	0	1.002934331	1.00570022	0	0
HMGN4	1938.78659	1964.4295	504.4759683	716.058553	652.9288897	608.8729088
SPATA13	895.651033	657.17988	1793.306759	2212.54047	2741.061981	2761.36549
GJC1	1.95343737	7.11013781	0	1.00570022	3.022818934	3.624243505
NPM1P14	0	0	0	0	0	0
SOX1	0	0	0	0	0	0
CNOT10	826.304007	954.789934	848.4824438	867.919286	1046.902957	1138.01246
RP11-294C11.1	0	0	0	0	0	0
MTA1	2495.51624	2369.70736	4107.016084	2783.79831	4530.812615	3992.10422
ZNF662	40.045466	36.566423	165.4841646	155.883533	8.06085049	145.8758011
CADM1	0	1.01573397	236.692502	368.086279	915.914137	434.0031597
ZNF320	514.379128	670.841502	970.0982607	1467.56804	179.1725543	895.1881457
C12orf60	15.6274989	14.2202756	23.06748961	15.0855032	35.2662209	23.55758278
PYCR1	2799.27575	2390.02204	6366.627131	4457.26336	2159.300325	4850.14387
NAA38	949.370561	1008.62383	302.8861679	303.721465	702.301599	294.4697848
SPNS2	178.739519	248.854823	8.023474646	20.1140043	593.4801173	158.5606533
MCEMP1	0	0	0	0	0	0
AP2A2	3025.87448	2551.52374	1249.656176	1537.71563	1272.606771	1535.773185
TPM3P8	1.95343737	3.04720192	0	1.00570022	3.022818934	0
SLC8A1	0	3.04720192	0	0	117.8899384	7.248487009

OR1G1	0	0	0	0	0	0
SLC25A21	5.8603121	8.12587178	31.09096425	97.5529209	32.24340196	69.76668747
OTOP2	0	0	28.08216126	30.1710065	0	21.74546103
CYLC1	0	0	0	0	0	1.812121752
PCP4	0	0	2.005868661	0	67.50962286	0.906060876
ABAT	1.95343737	9.14160575	722.1127181	1162.58945	4431.452557	1091.803356
SLC25A10	1215.03804	1203.64476	1526.466051	1687.56496	3170.937062	3105.976684
CAMK1D	173.855926	232.60308	157.4606899	211.197045	175.3234982	145.8758011
RGPD6	763.159144	1033.72262	192.6436262	38.7194583	827.9299539	237.9134649
FAM133CP	1.95343737	1.01573397	0	0	0	0
LYSMD4	174.832644	186.895051	121.355054	95.5415205	148.1181278	126.8485227
WBP2NL	2.93015605	3.04720192	54.15845386	82.4674177	13.09888205	67.95456571
IGSF5	0	0	14.04108063	12.0684026	13.09888205	15.4030349
NKX2-5	1.95343737	4.06293589	0	0	2.015212623	0
AFMID	526.451371	402.230653	337.9888694	409.319988	288.175405	391.4182985
GAS6	9949.83323	7284.84405	0	0	452.4152338	139.5333749
FREM3	0	0	0	0	0	0.906060876
NEB	20.5110924	14.2202756	7.020540315	24.1368052	148.1181278	69.76668747
BEGAIN	14.6507803	4.06293589	3.008802992	8.04560172	75.57047335	8.154547886
GPC6	87.9046815	74.14858	0	1.00570022	2603.654708	129.5667053
ARHGEF37	17.5809363	22.3461474	2224.508346	1274.22217	126.9583952	1803.967204
FAM43B	0	0	0	0	0	0
CSMD1	0	0	0	0	0	0.906060876
OR2A3P	0	0	0	0	0	0
CALHM3	35.1618726	57.8968364	0	0	12.09127574	0
OR2T11	0	0	0	0	0	0
PTGDR2	1.95343737	2.13304134	5.014671654	2.01140043	1.188975447	5.436365257
CEP57L1	420.965753	377.84288	292.8568246	353.000776	259.9624283	384.1698115
RIPPLY3	7.81374947	0	0	0	292.2058303	7.248487009
PRORY	0	0	0	0	0	0
ANKRD20A2	12.1308461	1.34076884	0	0.53302111	0	0.906060876
GPR19	13.6740616	26.4090833	58.17019118	50.2850108	41.31185876	75.20305272
GJD3	3.90687474	2.03146794	0	6.03420129	27.2053704	3.624243505

RP11-863K10.7	2.93015605	3.04720192	2.005868661	5.02850108	6.045637868	1.812121752
RABIF	540.125432	633.817999	825.4149542	1263.15947	1342.131607	892.469963
TMEM119	0.97671868	0	0	0	0	0
FANCF	139.670772	162.517436	813.3797422	656.722241	458.4608716	621.5577611
CALN1	0	0	0	0	0	0.906060876
POM121L1P	0	2.03146794	0	0	0	2.328576452
RP11-49C9.2	0.97671868	3.04720192	7.030569658	7.03990151	3.022818934	1.812121752
SMDT1	314.503416	334.176477	2099.141554	1631.24575	380.8751857	956.8002852
GABRR3	0	0	0	0	0	0
C2CD4C	23.4412484	28.4405512	22.06455528	22.1254047	48.36510294	26.27576541
CHST6	4.88359342	14.2202756	2.005868661	2.01140043	4.030425245	1.812121752
HSP90AB3P	66.0847862	42.996019	29.35588786	18.2534589	61.08109459	44.79564972
POTEC	0	0	0	0	1.007606311	0
RUVBL2	4327.84049	3920.73313	3001.782452	2066.71394	3372.458324	3294.437346
GDPGP1	51.7660902	74.14858	108.3169077	84.4788181	73.55526072	140.4394358
CTNNA3	0	0	5.014671654	0	6.045637868	5.436365257
RP5-1120P11.4	0	0	0	1.00570022	1.007606311	0
WT1-AS	0	0	0	0	0	0
RIMBP3C	31.1084901	0	0	51.6427061	52.24438724	49.07225705
PRR36	48.8359342	62.9755063	760.2242227	1217.90296	528.9933134	1169.724591
NF1P3	0	0	0	0	0	0
LINC01547	61.5332771	62.9755063	169.4959019	180.020339	212.6049317	231.9515843
OR51B4	0	0	0	0	0	0
PTTG1IP	17294.7577	14458.9731	5179.152884	4308.41972	7713.226313	4157.913361
DDX41	1813.7666	1708.46454	1737.082261	1402.9518	3113.503502	2125.618816
ABHD16B	4.88359342	10.1573397	20.05868661	13.0741028	8.06085049	12.68485227
OR52E8	0	0	0	0	0	0
CCDC60	0	0	0	0	0	0
PLGLB1	10.2653134	10.5331613	40.54863499	37.190794	8.06085049	15.46645916
DAZAP2	2590.25795	2895.85755	4283.532526	5925.58567	7001.856257	4365.401301
CCBE1	1493.40287	1024.87558	0	0	20.15212623	4.530304381
SEP15	2749.4631	2811.55164	2234.537689	1656.38826	1814.698967	1820.2763
TISP43	0	0	0	0	0	0

RPSAP19	21.1557267	21.4827735	6.589278553	15.5179543	17.13938336	15.53894403
OR5P2	0.97671868	0	0	0	0	0
FAM9A	0	0	0	0	0	0
MAGEA2B	0	0	0	0	0	0
CECR6	66.4168705	57.8968364	8.023474646	13.0741028	15.11409467	38.96061768
AC005037.3	9.76718684	7.11013781	3.008802992	2.01140043	10.07606311	0
ZNF623	1053.87946	964.947274	1568.589293	1426.08291	1392.511922	1698.864143
OR2T34	0	0	0	0	0	0
OR52L1	0	0	0	0	0	0
EPHA10	37.11531	33.5192211	31.09096425	63.3591136	287.1677987	68.86062659
SPDYE4	0	0	0	0	0	0
CCDC125	379.943568	396.136249	980.0473693	1056.27688	659.9821339	982.3783838
REC114	0	0	0	0	0	0
BOLA2	180.058089	257.844069	195.6825173	302.494511	732.0461373	341.3493745
BCOR	1304.89616	1301.15522	1440.213699	1097.21894	1514.432286	2252.467338
JRKL	579.19418	669.368688	246.7218454	217.231247	164.2398287	188.4606622
C10orf107	0	1.01573397	0	0	37.28143352	0
GBP6	0	0	0	0	0	0
KIAA2026	392.640911	415.435195	1152.371546	979.55201	769.8112218	857.1335889
OVCH2	5.8603121	2.03146794	0	0	0	0
SYNDIG1L	0	0	0	0	0	0
TTTY8	0	0	0	0	0	0
FHL3	450.267313	382.931708	83.24354945	71.4047153	108.8214816	150.4061054
OR56A4	0	0	0	0	0	0
PMCH	0	0	0	0	0	0
TMEM89	0	2.03146794	0	0	0	0.906060876
C19orf71	3.90687474	1.01573397	16.04694929	35.1995075	1.007606311	7.248487009
CCDC159	33.2084353	44.6922948	212.6220781	242.373752	123.9355763	191.1788449
RIPK4	362.362632	307.767394	220.6455528	214.214146	430.2478949	362.4243505
LRIT3	4.88359342	5.07866986	8.023474646	11.0627024	0	28.08788716
NPIPA1	989.357424	775.116752	1421.398651	1151.55692	905.3443468	1171.056501
SF3A3	2769.97419	2701.85237	2535.417988	1555.81823	2856.563893	2290.521895
ZBTB8OSP1	0	0	0	0	0	0

TFDP3	0	0	0	0	0	0
TRIM61	34.292593	26.7341181	0	0	0	0
OR7E38P	77.160776	75.1643139	34.09976724	30.1710065	65.49441023	40.77273943
GRIN2A	0	0	0	0	0	0.906060876
RP11-958N24.1	935.100701	831.449358	1612.397465	1352.79753	1002.28615	1188.824354
URAD	0	0	0	0	0	0
FLJ40288	0	0	0	0	0	0
GTF2H2C	1048.10705	985.607303	454.0384009	444.147386	896.9812144	666.4077744
ASB7	552.822775	566.779557	491.437822	482.736103	1108.366942	601.6244218
SH2D7	0	0	0	0	0	0
TREX2	14.7777537	12.1888077	36.21595868	16.0912034	50.51130438	52.96831882
GPR132	2.93015605	1.01573397	0	0	0	0.906060876
MX2	131.857022	150.328628	18.05281795	18.1026039	7.053244179	27.18182629
EP400	3966.78666	3477.09101	3160.49681	2854.65995	7621.775964	4118.236955
MEX3B	50.7893716	46.7237627	90.26408976	141.80373	237.7950895	156.7485316
PI4KAP2	470.837009	374.237025	715.8243199	825.207198	346.4755062	822.6760937
FAM46C	232.459047	346.365285	57.16725685	36.2052078	271.0460977	144.0636793
COA5	338.921383	339.255147	844.4707065	576.266224	533.0237387	594.3759348
TDGF1P2	0	0	0	0	0	0
UTP11L	995.276339	1106.1343	1039.039967	676.836245	1006.598705	916.0275458
PSMG1	1599.8652	1639.39463	708.0716375	676.836245	1510.401861	789.1790232
PRR14L	1497.30974	1459.60972	3390.920972	3392.22683	3588.086074	4564.734694
COL18A1-AS1	0	1.01573397	0	0	0	0
KLRC4	6.83703079	2.45807621	0	0	0	0
ACSM5	0	0	0	0	0	0
C10orf120	0	0	0	0	0	0
IZUMO1R	0	0	0	0	0	0
CTC-343N3.1	0	0	0	0	0	0
BPIFA4P	0	0	50.14671654	15.0855032	3.022818934	28.08788716
SERHL2	7.81374947	10.8175668	9.527876142	7.03990151	59.44877237	49.41656019
PCBP3	120.136398	134.076884	0	0	90.68456802	19.0272784
PGPEP1L	0	0	0	0	1.007606311	0
SETD3	2384.17031	2221.4102	3665.724979	3198.12669	3666.679367	3307.122198

TNFAIP8L3	3.90687474	9.14160575	0	0	26.19776409	2.718182629
ZNRF3	464.918094	419.498131	507.4847713	786.457569	666.0277718	845.3547975
FBXL7	0	0	9.026408976	9.05130194	0	19.93333928
HMGN3P1	0	0	0	0	0	0
TANGO2	335.014509	432.702672	1533.486592	2254.77988	719.4309063	1155.227617
HIST2H3D	3.23293884	1.16809407	12.70717797	6.02414429	12.00059117	14.67818619
SMG1P5	147.406384	125.707236	526.580641	718.95497	555.3724467	827.6413073
SFXN4	565.520118	597.251576	165.4841646	134.763829	667.0353781	190.272784
GKN2	0	0	0	0	0	0
FAM167B	0	2.03146794	41.12030756	45.2565097	4.030425245	16.30909577
MRPL54	677.842767	671.400156	449.3145802	584.311825	372.8143352	318.0273675
ZNF438	350.642008	414.419461	46.13497921	37.210908	144.0877025	54.36365257
HMCES	1332.24428	1130.51191	934.7347962	1066.04223	2667.133906	1416.173149
CCR3	0	0	0	0	4.030425245	0
DGCR6	7.99932602	14.3726357	235.5892743	327.65713	20.35364749	203.7549698
GOLGA8G	12.9317554	20.5889276	0	0	0	0
PRR32	0	0	1.002934331	0	0	0.906060876
TP53TG3	0	0	0	0	0	0
RP1L1	39.0687474	22.3461474	1.002934331	2.01140043	6.045637868	5.436365257
KRTAP8-1	0	0	0	0	0	0
C15orf32	0	0	0	0	2.015212623	0
C11orf88	0	0	0	0	0	0
ZNF530	243.202952	245.807621	119.3491854	140.79803	208.5745064	243.7303757
NDUFB1	671.005736	773.989287	1603.691995	1457.25961	781.9024976	1067.339712
MARCH11	0	0	0	0	0	0
KLHL25	215.854829	240.728951	129.3785287	151.860733	604.5637868	196.6152101
FAM19A1	0	0	0	0	0	0
RP11-44N17.1	0	0	0	0	0	0
TRMT12	65.4401518	69.0699101	405.1854696	334.898172	1449.945482	519.1728821
GUSBP1	131.788652	109.557066	129.1077364	146.510407	271.570053	194.5765732
PSG9	11.8378304	8.12587178	0	0	141.296633	87.88790499
GPR1	212.924673	197.052391	0	0	7.053244179	3.624243505
LINC00518	19.5343737	32.5034871	0	0	0	0.906060876

BMP8A	0	0	0	0	19.1747481	1.458758011
ALYREF	2808.06622	2568.79122	1430.184356	1298.35898	4998.73491	2102.967294
FAM101B	76.1840573	50.7866986	0	0	78.59329228	8.154547886
EFHC2	0	0	0	0	4.030425245	0
NOG	1449.45053	1423.0433	1.002934331	2.01140043	28.21297672	30.80606979
MRGPRX2	0	0	0	0	0	0
UPP1	1043.13555	1277.79334	1.002934331	2.01140043	138.0420646	36.24243505
SLC9B1P1	0	0	0	0	0	0
OR4N4	0	0	0	0	0	0
IFNL2	0	0	0	2.01140043	0	6.342426133
OPCML	0	0	0	0	0	0
TRIM52	230.505609	284.405512	710.0775061	686.893247	560.2291091	640.5850395
LHFP	525.474652	652.10121	5.014671654	6.03420129	28.21297672	10.87273051
CMTM4	1582.28427	1558.13591	8309.31093	5732.49123	4383.087454	5990.874513
TMEM50A	1753.21004	1927.86308	1937.669127	2024.47453	982.4161535	1716.98536
NPBWR1	0	0	0	0	0	0
FIGLA	0	0	0	0	0	0
ASCL2	0	0	1.002934331	10.0570022	411.103375	9.966669638
TBK1	1037.27524	1037.06439	1070.130931	1258.13097	1970.877945	1154.321556
CBX6	3180.19603	3430.13362	4453.028428	4304.39692	2894.852932	5242.46823
MACC1	0.97671868	0	339.8142099	676.836245	95.72259957	649.5097391
ACSM2A	0	0	0	0	0	0
TBL3	1109.55242	1059.41053	1480.331072	1480.39072	1923.520448	1817.558118
BPY2	0	0	0	0	0	0
PAPL	18.557655	28.4405512	0	1.00570022	18.1369136	1.812121752
KREMEN1	369.199662	323.003403	734.1479301	843.782481	632.7767635	565.3819867
TRAIP	429.756221	393.089047	209.6132751	216.225546	249.8863652	447.5940728
CHEK2	275.434669	231.587346	657.9249209	552.129418	590.4572984	1071.870017
FOXL2	0	0	0	0	0	0
AIFM3	7.81374947	6.09440383	0	1.00570022	44.3346777	7.248487009
KCTD16	0.97671868	1.01573397	1.002934331	7.03990151	0	5.436365257
B3GALT5	250.039983	582.015566	1.002934331	1.00570022	91.69217433	23.55758278
ZNF703	1815.72003	1633.30023	1125.292319	1171.64075	3197.134826	1709.736873

SLC35F3	386.780599	336.207945	3.008802992	0	12.09127574	2.718182629
KCTD8	0	0	0	0	0	0
C9orf66	0	0	0	0	96.73020588	8.154547886
TUBA8	10.7439055	0	31.09096425	23.131105	18.1369136	42.58486118
TCEB3C	0	0	0	0	0	0
NPIPA5	0	5.7287396	362.0893814	249.946675	31.09473077	257.6927738
BPY2B	0	0	0	0	0	0
EMILIN3	1.95343737	1.01573397	1.002934331	2.01140043	3.022818934	1.812121752
OLFML1	0.97671868	0	0	0	1.007606311	0
FAM162B	0	0	0	0	0	0
RBM12B	1109.55242	959.868604	1838.378628	1497.48762	2239.90883	1724.233847
CCR4	0	1.01573397	0	6.03420129	0	14.49697402
LIN9	556.72965	562.716621	692.0246882	641.636738	311.3503502	820.8911538
CTA-833B7.2	0	0	1.002934331	1.00570022	0	0
BTBD9	615.332771	751.643139	417.2206816	499.833007	431.2555012	470.2455947
NUDT14	351.618726	415.435195	692.0246882	1010.72872	514.8868251	388.7001159
ANKRD45	0	2.03146794	0	3.01710065	2.015212623	2.718182629
MAATS1	0.97671868	2.03146794	0	0	17.12930729	5.436365257
PNMA3	0	0	0	0	1.007606311	0
GPR39	357.479038	503.80405	362.0592934	332.886771	118.8975447	290.8455413
FAM3B	0	1.01573397	0	0	10.07606311	0.906060876
ZNF730	0	0	0	0	111.0986719	0
KIRREL	4034.82488	4074.10896	0	0	254.9243968	110.5394269
IQGAP3	3979.15192	3007.58829	864.5293931	2420.72042	1771.371895	1834.773274
CNGA2	0	0	58.17019118	1.00570022	0	14.49697402
TOB2	1343.64259	1259.43902	4512.121319	4447.03538	2374.474653	4539.111293
SCN5A	3514.23382	2544.4136	0	0	107.8138753	40.77273943
ARSI	268.597638	298.625788	0	0	3.022818934	4.530304381
UTY	0	0	0	0	0	0
C1orf64	0	0	5.014671654	4.02280086	0	2.718182629
AC138969.4	1211.07256	1309.07794	2289.287874	1634.19245	1528.911589	1668.284588
TTC32	152.368115	165.564637	81.23768079	100.570022	97.73781219	83.35760061
LRRC55	0	1.01573397	0	0	0	0

OR4G2P	0	0	0	0	0	0
RPL21P132	0	0	0	0	0	0
DNAH2	2184.9197	2118.82107	1.002934331	2.01140043	34.25861458	10.87273051
SH2D1A	0	0	0	0	0	0
SDR42E2	0	0	0	1.00570022	0	0
DUSP5P1	14.6507803	11.1730737	378.1062427	198.122942	0	152.2182272
HTR7P1	92.788275	102.589131	139.407872	190.077341	171.2930729	119.6000357
TRBV21OR9-2	0	0	0	0	0	0
PRKX	898.512819	860.834541	456.3150618	504.730767	1612.442152	531.4681281
SETD8	1829.31596	1915.62348	2330.658915	2129.93226	2460.544384	2115.479994
KCNH8	0	0	0	1.00570022	0	0
SMTN	2448.63374	2880.62155	1805.281795	2561.51845	1083.176785	2857.716003
NPW	0	0	4.011737323	5.02850108	11.08366942	0.906060876
PP2D1	0	0	0	0	0	0
COA3	922.999156	969.01021	491.437822	431.445392	732.5297883	394.1364811
NPB	5.8603121	9.90340623	33.54815336	23.131105	16.66580839	18.67391466
MAGEA13P	0	0	0	0	0	0
ST6GALNAC3	0.97671868	1.01573397	0	0	4.030425245	0.906060876
PTP4A2	5993.14584	5852.65915	7617.286242	7038.89581	3160.860999	13874.5102
ACTG1	82592.9435	78538.247	32584.72461	53130.9714	85574.83519	60698.27752
TMPRSS2	19.5343737	27.4248173	1020.987149	1148.50965	408.0805561	798.2396319
DENND5A	1803.99941	1455.54678	338.9918038	319.812669	902.8152549	409.539516
OR2T10	0	0	0	0	0	0
DSCR4	0	0	0	0	0	0
KRTAP20-2	0	0	0	0	0	0
CTAG1B	0	0	0	0.50285011	1.007606311	0
DIABLO	1337.12788	1436.43067	844.4707065	877.33264	1627.526018	1098.490085
OR7E87P	0	0	0	0	0	0
VPS33B	350.642008	411.372259	248.727714	214.214146	598.5181489	269.1000802
TBX1	286.178574	256.980695	0	0	11.08366942	2.718182629
ADAP2	102.555462	102.589131	15.04401496	5.02850108	66.50201655	11.77879139
RP5-821D11.7	2.93015605	4.06293589	20.05868661	40.2280086	6.045637868	16.30909577
UQCR10	1223.82851	1160.98393	2687.864006	2545.42725	1742.151312	2301.394625

FAM120C	482.49903	480.442169	552.6168162	658.733641	1100.306092	819.9850929
CTD-2372A4.1	0	0	0	0	0	0
BRD7P2	8.96627752	7.24218322	7.191039151	7.17064254	2.065592938	5.572274388
TREML3P	0	0	0	0	1.007606311	0
TRIML1	0	0	0	0	0	0
EIF3C	8059.03283	8137.08548	8335.858602	7262.42274	25402.78287	12403.93715
RP11-366I13.3	0	0	0	0	0	0
CLDN5	0.97671868	0	0	0	1.007606311	0
RP13-1039J1.2	0	0	0	0	0	0
NIPSNAP1	718.864951	820.692735	4290.553067	5234.66962	2801.145545	4317.380075
RPL7AP28	0	0	0	0	0	0
OR4F6	0	0	0	0	0	0
CNTN2	0	0	0	0	2.015212623	0.906060876
SPRR4	0	0	0	0	0	0
LRTOMT	65.4401518	69.0699101	248.727714	222.259748	231.7494516	175.77581
OR10J5	0	0	0	0	0	0
KCNQ3	36.1385913	38.5978909	0	0	4.030425245	3.624243505
ADRA2C	9.76718684	3.04720192	186.5457855	556.152219	981.4085472	230.1394625
NR2C2AP	595.798397	551.543547	382.11798	431.445392	944.1271137	569.0062302
FAM132A	2.93015605	6.09440383	28.08216126	19.1083041	6.045637868	6.342426133
CRELD2	450.267313	545.449143	2412.057065	1591.01774	979.3933346	1487.751959
OR1D2	0	0	0	0	0	0
SCFD2	233.435765	255.964961	293.8597589	215.219846	509.8487935	398.6667855
UBE2F	762.817292	827.823187	527.3930178	414.821168	846.4094536	477.4940817
KCNJ12	5.8603121	10.8378815	2.005868661	0	2.015212623	0.009060609
CTC-512J14.7	0	0	0	0	0	0
GPR173	210.971236	220.414272	0	0	78.59329228	12.68485227
PPP1R2	1324.30356	1344.83178	1167.375444	987.497042	1132.438657	966.7669549
TSPYL2	361.385913	381.915974	1016.975411	3373.11852	361.7306658	951.36392
GOLGA6L4	57.0501383	47.0386403	15.23457248	9.75529209	34.64150498	21.51894581
PGP	936.673218	1013.7025	1311.838105	4578.95308	2238.901224	1854.706614
C22orf46	731.562294	801.414104	1579.621571	1734.83287	616.6550625	1707.018691
SNRNP35	415.105441	421.529598	523.5317206	513.91281	369.7915162	517.3607603

DGAT2L6	0	0	0	0	0	0
IRAK1	6651.45424	7674.88589	6112.884746	6067.3894	15473.81012	7728.699274
CMSS1	1423.07912	1441.32651	361.0563591	258.464955	986.4465788	459.3728642
OLIG1	0.97671868	4.06293589	0	0	0	0.906060876
C11orf72	0	1.01573397	1.002934331	0	1.007606311	0.906060876
PCDH9	47.8592155	107.667801	0	0	371.8067289	9.966669638
ACOT1	46.159725	76.9520057	369.6314476	1590.39421	97.76804038	303.3944844
OAF	1879.20675	2095.45918	280.8216126	241.368052	182.3767423	455.7486207
ALDH1A3	561.613243	421.529598	3.008802992	2.01140043	1350.192457	419.5061857
CDR1	0	0	0	0	0	0.906060876
HIST2H2AC	16.3307364	16.3939463	19.67757157	34.9380255	39.63923229	15.28524698
KCNK12	4.88359342	2.03146794	103.3022361	64.3648138	26.19776409	30.80606979
HIST2H2AB	0	1.01573397	1.002934331	1.00570022	2.015212623	3.624243505
POU6F1	67.3935892	72.117112	6.017605984	3.01710065	7.053244179	4.530304381
LINC00315	0	0	0	0	0	0
DEFB108B	0	0	0	0	0	0
TM2D3	465.894812	496.693912	550.6109476	478.713303	656.959315	531.8577343
TSSC4	846.815099	950.726998	811.3738735	760.309363	665.0201655	685.8880833
TACSTD2	3087.40776	2349.39268	3182.310631	3581.29847	4875.80694	5536.938014
CLECL1	0	0	0	0	0	0
SIX6	0	0	0	0	0	0
DRD5P1	0	0	0	0	0	0
PRKD1	0	0	80.23474646	119.678326	23.17494516	78.82729623
CCSER1	2.93015605	0	66.19366583	92.5244198	94.71499326	109.633366
ZDHHC23	193.390299	199.083859	378.1062427	446.530896	799.0318049	517.3607603
MROH7	0	0	0	0	2.015212623	0
RPL23AP82	320.617675	314.582969	336.6649961	329.779158	142.9793356	363.3938356
OR51J1	0	0	0	0	0	0
S100A7A	0	0	0	0	5.038031556	1.812121752
SRPK3	8.11653226	2.41744685	20.3796256	13.2953568	2.700384914	14.45167098
GDF3	0	0	0	0	0	0
IQCF2	0	0	0	0	0	0
SLIT3	0.97671868	0	40.11737323	87.4959188	0	52.55153082

EFNA5	1288.29194	997.450761	239.701305	322.829769	255.9320031	241.9182539
MRGPRE	0	0	0	0	0	0.906060876
KRTAP19-1	0	0	0	0	0	0
HIST1H1B	0	0	1.002934331	10.0570022	11.08366942	4.530304381
SPATA32	0	2.03146794	2.005868661	0	2.015212623	0
PKP3	1010.90384	941.585392	3931.502576	5004.36427	4185.596617	5607.610763
MAP7D2	3.90687474	2.03146794	7.020540315	13.0741028	16.12170098	18.12121752
CSF1	18302.7314	19416.7706	18.05281795	13.0741028	143.0800962	93.32427025
COLEC10	1.95343737	4.06293589	0	0	0	0.906060876
ACTRT3	52.7428089	53.8339005	40.11737323	44.2508095	60.45637868	68.86062659
PLA2G6	93.7649937	182.832115	1126.295253	1511.56742	110.8366942	708.5396052
MAML2	2012.04049	1684.08693	2.005868661	1.00570022	31.23579565	20.83940015
UMODL1-AS1	0	0	0	0	0	0
PABPC1L2B	0	0	0	0	8.06085049	0
A3GALT2	0.97671868	0	1.002934331	0	0	0
OR4N5	0	0	0	0	0	0
SS18L1	1006.99696	920.254979	1179.450773	957.426605	1526.523562	1094.521538
KCND2	0	0	1.002934331	0	0	0
RP11-44M6.3	2.93015605	0	0	0	1.007606311	4.530304381
RPL23AP38	0	0	0	0	0	0
TOP1MT	735.469169	785.162361	565.6549625	717.064254	985.4389724	718.5062748
COPB2	3951.80379	3999.96038	7561.121919	6364.07096	5822.956873	6221.920037
LRRC19	0	0	0	1.00570022	0	0
THAP7	496.173091	497.709646	1488.354547	1199.80036	714.3928747	1501.342872
AP001062.7	90.8348376	146.265692	92.26995843	83.4731179	228.7266327	176.6818709
KNTC1	3514.23382	2606.37337	1202.518263	1404.9632	2669.149119	2541.500758
CCR10	25.3946858	30.4720192	9.026408976	0	7.053244179	3.624243505
NCMAP	4.88359342	1.01573397	0	0	3.022818934	0.906060876
BPIFC	0	0	0	0	0	0
WDR27	548.9159	446.922948	327.9595261	302.715765	379.8675794	428.5667944
TXNRD2	484.452467	351.443954	1385.052311	845.793881	761.7503713	873.4426846
C1QTNF8	0	0	0	0	0	0
OR56A3	0	0	0	0	0	0

FOXO4	176.786082	183.847849	661.9366583	1109.28734	38.28903983	792.8032667
POU3F2	0.97671868	1.01573397	7.020540315	34.1938073	6.045637868	10.87273051
PTP4A3	114.276086	93.4475254	442.2940398	384.177482	198.4984433	362.4243505
FOXD4L1	15.8033083	8.12587178	30.55940906	16.9762196	12.51447039	19.62527858
TMEM255B	271.527794	277.295374	1.002934331	0	16.12170098	5.436365257
PROS1	695.423703	898.924565	6.017605984	19.1083041	47.35749663	41.6788003
GAST	0	0	0	0	0	0
NUTM1	0	0	0	0	0	0
HDDC3	280.318262	308.783128	270.7922693	300.704364	462.4912969	237.3879496
BEX5	1.95343737	2.03146794	0	0	4.030425245	0.906060876
ZFP1	269.574357	236.666016	188.5516542	221.254047	837.3208447	246.4485583
PTGER4P2	0	0	0	0.62353413	0	0
CEND1	0	0	0	0	1.007606311	0
C6orf58	0.97671868	6.09440383	0	0	1.007606311	0.906060876
DHRS7C	0	0	0	0	0	0
DUSP8	134.787178	74.14858	77.22594346	228.293949	452.4152338	224.7030973
SOCS3	1200.38726	1107.15003	20.05868661	85.4845183	123.9355763	46.20910469
C17orf74	0.97671868	0	0	1.00570022	0	0.906060876
SLITRK6	0	0	449.3145802	223.265448	881.6555224	471.1516556
RP13-786C16.1	0	0	0	0	0	0
PIWIL3	0	0	0	0	2.015212623	0
LPAR5	4.88359342	13.2045416	0	0	16.12170098	1.812121752
XPOT	5860.2535	5013.65273	9221.941053	2569.55399	11922.92487	4511.249921
TMEM173	806.769633	990.340623	29.08509559	57.3249123	19.14451991	149.5000446
PDE4B	83.0210881	61.9597723	509.49064	335.903872	1.007606311	288.1273586
FAM19A3	1.95343737	3.04720192	0	1.00570022	0	0
C14orf180	0	0	0	0	0	0
SNN	293.015605	399.183451	626.8339567	533.021114	668.0429844	603.4365435
FAM167A-AS1	0	0	0	0	0	0
KCNH7	0	0	0	0	0	0
RPL7P26	0	0	0	0	0	0
NELL2	38.0920287	44.6922948	9.026408976	2.01140043	41.31185876	31.71213067
SPDYE12P	0	0	0	0	1.007606311	0

ZNF840P	0	0	0	0	0	0.906060876
KRBA2	50.3986841	70.7052418	103.9742021	130.730971	74.3814979	96.92133192
ZNF72P	0	0	0	0	0	0
MED12	1436.75318	1509.38068	1899.557622	2573.58685	1475.13564	2295.052199
ZNF93	363.251446	327.066339	464.7597689	535.555479	279.7820445	854.9228003
SEPT9	8667.4016	7864.82815	8020.465843	8528.33783	9093.646959	10743.16381
PRSS55	0	0	0	0	0	0
ODF4	0	0	0	2.01140043	0	0
FOXD4L4	1.46507803	1.01573397	16.11715469	5.81294725	2.317494516	7.737759883
CDCA2	974.765246	1006.59237	190.5575228	450.553697	1277.644803	692.2305094
OR7E14P	4.88359342	3.04720192	44.12911055	92.5244198	130.9888205	60.7060787
RALYL	0	0	0	1.00570022	0	0.906060876
AMER1	383.850443	399.183451	421.2324189	394.234484	630.7615509	524.6092473
ZBTB40	1741.48941	1845.58863	1091.192552	1505.53322	1131.541888	1598.291386
HIST2H2BE	43.6886267	40.751247	821.523569	1107.96987	1223.475887	1223.227486
PRR33	0	0	0	0	1.007606311	0.906060876
CLDN6	0.97671868	1.01573397	3.008802992	5.02850108	6.045637868	6.342426133
OR51M1	0	0	0	0	0	0
SEPT5	122.050767	110.755632	409.9393783	341.897845	384.8451545	585.8136595
EIF4ENIF1	521.567777	493.646711	1292.782352	1262.15377	833.2904194	1521.276211
LRRC26	38.0920287	43.6765608	2922.55064	1394.77546	210.9222291	2697.343228
SERINC4	2.93015605	0	8.575088528	10.0570022	1.007606311	6.505517091
RNLS	130.880304	123.919545	156.4577556	153.872133	113.8595132	75.20305272
KRTAP6-1	0	0	0	0	0	0
APOBR	2.93015605	4.06293589	50.14671654	202.145743	17.12930729	58.89395695
FAM110C	1.95343737	0	759.2212883	644.653838	474.5825726	605.2486653
DDX53	0	0	0	0	4.030425245	1.812121752
OR5AQ1P	0	0	0	0	0	0
ATL3	7238.46217	6487.49288	2610.638063	3367.08432	2590.555826	4044.655751
NDUFA12	1514.89068	1340.76884	835.4442975	672.813444	1084.184391	732.097188
AC013269.5	0	0	0	0	0	0
MGAT4EP	0	0	2.005868661	1.00570022	8.06085049	17.21515665
SMIM10	0	0	0	0	0	0

TCTE3	30.2782792	40.6293589	49.1437822	33.1881071	75.57047335	48.92728731
UBE2G2	2869.59949	2570.82268	2797.183848	1554.81253	4015.31115	3267.255519
SATL1	0	0	4.011737323	3.01710065	0	0
OR4C10P	0	0	0	0	0	0
OSBP2	318.410291	442.860012	355.0387531	300.704364	466.5217221	204.769758
UNC93B5	0	0	0	0	0	0
B3GALT5-AS1	0	0	0	0	12.09127574	2.718182629
TUSC5	0	0	0	0	0	0
PRR23B	0	0	0	0	0	0
ZBTB7C	0.97671868	0	1707.997165	1626.21725	10.07606311	2351.227974
APOO	451.244032	514.977124	394.153192	487.764605	286.1601924	311.6849414
PRR16	44.9290595	27.4248173	0	0	1.007606311	0.906060876
TMED9	4304.39924	4765.8238	3603.54305	2840.09741	5370.541639	3414.037381
CYCSP45	0	0	0	0	0	0
DRD1	0	0	0	0	80.6085049	4.530304381
LINC00308	0	0	0	0	0	0
TMEM186	158.228427	188.926519	358.0475561	234.32815	408.0805561	348.8334373
SDR42E1	231.482328	268.153769	588.7224521	585.317525	793.9937733	1024.754851
RBM33	2518.95749	2105.61652	1746.10867	1801.20909	2352.760737	2174.546103
ARMCX2	1237.50257	1477.89293	1815.311139	1158.56665	236.7874832	1514.933785
OR51B2	0	0	0	0	0	0
BTBD6	1415.26537	1428.12196	1472.307597	1816.29459	1737.113281	2065.818798
SRY	0	0	0	0	0	0
H1FX	2929.17933	2884.68448	5993.53556	8378.4885	5474.325089	6086.916966
RBM43	78.1374947	71.1013781	141.4137406	95.5415205	138.0420646	140.4394358
SUMO3	5921.84538	5546.92322	3049.9233	3360.04442	7604.404831	3781.898097
IMMP2L	60.5565584	79.2272498	96.28169575	81.4617175	93.70738695	67.04850484
TCEAL2	0	0	0	0	2.015212623	0
AC129778.2	3.73106537	0	0	0	0	0
CLCNKB	0	0	0	0	0	0
DMRTC1B	0	0	1.002934331	1.74991838	0	1.812121752
JAG2	195.988371	145.341374	3643.660423	3123.70487	1787.876487	4728.731713
FMNL1	4941.21982	5541.84455	46.13497921	34.1938073	442.3391707	327.9940372

NUTM2A	38.5608536	92.6450956	33.47794796	24.9413653	5.47130227	24.45458305
PTRHD1	743.282918	760.784745	194.5692602	370.097679	371.8067289	202.9576363
LCN12	3.90687474	2.03146794	25.07335827	62.3534134	8.06085049	16.30909577
OR6A2	1.95343737	0	0	0	0	0
WT1	186.553269	169.627573	8.023474646	0	0	8.154547886
ZFP90	489.336061	475.363499	1545.521804	1088.16763	1147.663589	1351.842827
AQP12A	0	0	0	0	0	0
FAM227A	29.3015605	33.5192211	176.5164422	57.3249123	87.66174908	258.2273497
OR6C70	0	0	0	0	0	0
MUC6	0	2.03146794	0	0	1.007606311	1.812121752
AL772307.1	5.32311683	0	0	0	3.859132172	0
NOC4L	479.568874	440.828544	760.2242227	594.368827	715.400481	827.23358
USP18	40.9733488	66.0227082	1058.07566	1190.5278	205.5516875	1447.323522
NDUFA6	937.649937	1008.62383	2378.960232	3154.88158	1200.059117	2396.531017
CHRM5	0	0	0	0	0	0
SORCS2	1508.05365	974.088879	0	0	86.65414277	15.4030349
TMEM121	75.2073387	70.0856441	94.27582709	46.2622099	255.9320031	112.3515486
TMEM106A	182.646394	222.44574	89.26115543	88.501619	61.46398499	36.24243505
SIVA1	1949.53049	1828.32115	1386.055245	1559.84103	2913.997452	1784.939926
TTTY13	0	0	0	0	0	0
BRI3BP	1706.32754	1751.12537	2131.235453	3172.98418	6506.113952	3703.070801
IFNE	0	0	0	0	0	0
SLC22A10	0	0	1.002934331	0	0	0.906060876
DGAT1	856.513915	833.744916	1349.608611	1472.76751	406.0653434	1486.275079
RFX6	0.97671868	0	0	0	0	0
ROBO2	3.90687474	0	67.19660016	93.53012	0	148.5939837
AP3M1	1972.97174	2020.29487	1187.474248	927.255599	1780.440352	1247.645826
F8	104.508899	143.21849	324.9507231	307.744266	112.8519069	318.9334284
NT5C1B	0	0	0	0	1.007606311	0
CA13	503.986841	458.096021	3.008802992	16.0912034	55.41834712	19.93333928
UBOX5	298.875917	290.499916	252.7394513	219.393502	187.5759909	289.5136318
MAFF	1648.70114	1601.81247	68.19953449	257.459255	124.9431826	86.98184411
BRF1	1191.59679	1174.18847	2622.673275	2522.29614	1952.741031	2767.109916

LRRC14B	0	0	0	0	0	0
SLC2A3P2	1.95343737	0	3.008802992	2.01140043	0	0
SEMA4B	2491.60936	2048.73542	331.9712635	427.422592	1435.838994	826.3275191
ZNF733P	0	0	0	2.01140043	0	0
MROH2A	0	0	1.002934331	4.02280086	14.10648836	5.436365257
SPDYE16	2.16831548	0	3.008802992	2.01140043	4.030425245	1.132576095
RP11-321E2.6	0	0	0	0	0	0
CIB1	1055.8329	1269.66747	527.543458	694.938849	1464.05197	709.445666
ANKS1B	2.93015605	7.11013781	459.3439235	545.089517	25.19015778	508.3001515
NELFA	932.766343	815.63438	798.3357272	609.454331	993.4998229	774.6820491
SLC24A3	1.95343737	3.04720192	2107.165029	1364.73519	473.5749663	1705.206569
SGCZ	0	0	0	0	0	0
EFCAB10	0	0	0	0	8.06085049	0.906060876
C5orf47	0	0	1.002934331	0	0	0
AC000068.5	9.76718684	5.07866986	42.12324189	75.4275162	43.32707139	59.80001783
KRT76	0	0	0	0	0	0
FLRT2	77.160776	101.573397	0	0	13.09888205	10.87273051
OR7E31P	0	0	0	0	0	0
AC093698.1	0	0	0	0	0	0
INTS5	781.374947	900.956033	740.1655361	741.613396	1012.644343	1042.876068
FAM169B	3.90687474	0	0	0	2.015212623	0
RPS27L	810.676508	818.681582	1074.142668	1224.94286	1384.451072	1052.842738
MANEAL	706.167608	828.838921	783.2917123	969.495008	1533.576806	1317.412514
ADSSL1	11.7206242	4.06293589	271.7952036	74.421816	313.3655628	234.6697669
ANO9	6.83703079	8.12587178	705.0628345	1075.09353	307.3199249	653.2698917
FAF1	1892.88081	1867.93477	1323.873317	1367.75229	1822.759817	1316.506453
MYADML2	0	0	1.002934331	1.00570022	0	0.906060876
FAM43A	204.134205	301.67299	110.3227764	261.482056	70.53244179	111.4454878
NDNL2	621.193083	709.998047	632.8515627	564.197821	485.666242	417.6940639
HSF1	2638.11717	2817.64604	3107.090557	3292.66251	2324.54776	3320.713111
C6orf120	1073.41383	1166.0626	874.5587364	778.411967	1265.553527	921.4639111
PURA	1923.15909	1985.75992	1369.005361	1170.63505	1271.599165	1178.7852
HIST1H2BL	0.97671868	2.03146794	17.04988362	16.0912034	7.053244179	15.4030349

INPP5J	57.6264023	61.9597723	11150.62389	10691.599	92.69978064	6564.411048
NPY2R	0	0	0	0	0	0
MIXL1	0	0	1.002934331	0	0	0
MFSD6L	0	0	0	0	0	0
LRRC37B	264.397748	237.265299	143.0084062	170.85841	94.66461295	158.0713805
DDX51	826.304007	683.588963	851.4912468	622.528433	1073.100722	828.1396408
NOMO2	3491.11489	3973.78492	6123.305233	6105.8876	7399.487936	6980.229566
LINC00482	10.7439055	3.04720192	8.023474646	6.03420129	52.39552819	13.59091314
AQP12B	0	0	0	0	0	0
ZNF479	0	0	2.005868661	0	0	0
GOLGA8DP	1.24043273	0	0	0	0	0
LINC00313	0	21.4827735	1.002934331	0	2.015212623	2.718182629
SIGIRR	60.5565584	96.4947274	1168.418495	1769.02668	731.522182	1304.727662
NRBP2	811.653226	772.973553	1959.733682	1181.69775	192.4528055	829.0457017
PRSS57	0	0	0	0	0	0.906060876
IFITM2	1101.84611	880.946074	366.0710307	399.262986	484.7291682	752.6828911
WASIR1	0	0	0	0	0	1.350030706
TNFAIP2	10120.759	5525.59281	7.020540315	13.0741028	2570.4037	877.0669281
ZNF445	1367.40616	1163.0154	1454.25478	1313.44448	836.3132384	1884.606622
PGBD2	144.554365	181.816381	195.5721945	310.761367	286.1601924	254.6031062
GAPDHP24	0	0	0	0	0	0
WBP5	1482.65896	1728.77922	0	0	29.22058303	18.12121752
MC2R	0	0	0	0	0	0
RAB11B	2061.85314	2062.9557	1239.626833	1780.08938	879.6403098	1641.782308
PRMT3	1135.92383	1054.33186	207.6074065	233.32245	908.8608928	401.3849681
GP1BA	0	1.01573397	3.008802992	0	4.030425245	0.906060876
PRPF39	1148.62117	932.443787	1865.457855	1693.59916	789.9633481	1437.91861
MAGEA11	0	0	0	0	0	1.812121752
PPIL6	9.76718684	7.11013781	69.20246882	30.1710065	26.19776409	34.43031329
ZNF74	216.831548	245.807621	1092.195486	1193.76616	530.0009197	1300.197357
KIAA0825	5.8603121	9.14160575	56.16432252	57.3249123	30.22818934	67.04850484
UBALD2	1145.69102	1043.15879	1652.835777	2107.94765	461.4836906	1485.033776
TEX33	0	0	0	0	0	0

CDNF	6.83703079	7.11013781	32.09389858	10.0570022	15.11409467	14.49697402
NOTUM	3.90687474	2.03146794	1.002934331	1.00570022	3.022818934	0.906060876
KLHL33	0	0	0	0	0	0
RBM11	5.8603121	5.07866986	48.14084787	75.4275162	2.015212623	87.88790499
WBSCR17	0	0	1.002934331	0	0	0
CD24P4	0	0	0	0	0	0.924182094
ZBTB37	468.824968	441.844278	1602.68906	1299.36468	1207.112361	1523.994394
NUPR1L	0	0	830.4296258	1038.88832	0	242.8243148
IL3RA	0	1.01573397	0	0.50285011	0	0.453030438
SPPL2C	0	0	0	0	0	2.718182629
CTD-2139B15.1	0	0	0	0	3.022818934	0
CCDC137	1187.68992	1102.07136	830.4296258	809.588674	1808.653329	1383.554958
SFTPA2	0	0	3.008802992	0	5.038031556	11.77879139
RGPD2	3.13526698	3.92073313	142.8178487	105.970632	72.55773048	82.29750938
ARL15	295.945761	332.145009	208.6103408	665.773543	245.85594	375.1092027
C12orf56	0	0	0	0	0	6.342426133
SCN10A	0	0	0	0	0	0.906060876
SSXP4	0	0	0	0	0	0
CDK10	1174.01586	977.136081	925.7083872	781.429067	1438.861813	1050.124555
TMEM105	0	0	10.02934331	1.00570022	53.4031345	6.342426133
SOCS1	106.462337	99.5419293	43.12617622	79.450317	59.44877237	64.33032221
TCN2	94.7417123	121.888077	91.26702409	221.254047	6.045637868	148.5939837
GAS2L1	2106.7822	2401.19511	1211.544671	2560.51275	1104.336517	1729.670213
ATP6V0A2	829.234163	980.183283	559.6373565	747.23526	1280.667622	812.7366059
PARK2	0	0	5.014671654	0	3.022818934	6.342426133
C14orf80	835.094475	764.756265	813.0186858	1336.91752	1677.442835	1407.502147
HS6ST3	4.88359342	6.09440383	361.0563591	618.505633	1074.108328	547.2607692
HGS	5082.84403	4988.26954	2176.367498	2134.09586	3143.731691	2481.70074
TNFAIP8L1	837.047912	785.162361	91.26702409	231.31105	259.9624283	244.6364366
OR2V1	0	0	0	0	0	0
RAD51D	504.96356	466.993851	518.517049	604.42583	380.8147293	632.4304916
OR7A17	0	0	0	0	0	0
MAPK11	154.321552	163.53317	36.10563591	35.1995075	197.490837	123.2242792

FGF7P2	0	0	0	0	0	0
SP140L	377.003645	386.761025	0	0	326.4140645	83.35760061
MRPL30	1385.8173	1292.52148	1492.286049	1258.35222	1977.18556	1684.711472
TARSL2	140.64749	142.202756	132.3873317	192.088741	418.1566192	149.5000446
SMYD3	757.933699	769.926351	329.9653948	236.339551	347.6241774	313.4970632
METTL7A	127.950148	183.847849	1035.028229	2725.44758	46.34989032	1291.136749
LINC00158	0	0	2.005868661	0	0	0.906060876
IFNLR1	89.8581189	114.777939	3.008802992	8.04560172	6.045637868	25.36970453
SH3BGR	10.7439055	9.14160575	34.09976724	31.1767067	72.54765441	35.33637417
FAM174B	14.6507803	20.3146794	1320.864514	1399.9347	892.7391918	1216.839757
FAM47A	0	0	1.002934331	0	0	0.906060876
C19orf68	642.680894	591.157172	313.9184455	231.31105	247.8711526	306.2485761
KPNA7	0.97671868	1.01573397	12.03521197	10.0570022	21.15973254	6.342426133
TMEM179B	697.37714	621.629191	817.3914795	808.582973	426.2174697	891.5639022
GPRIN3	0	3.04720192	0	0	23.17494516	32.61819154
KRT6B	0	0	0	0	7.053244179	0.906060876
PARPBP	896.627752	910.097639	358.0475561	562.18642	733.5373946	773.7759883
STAC3	2.93015605	7.11013781	40.11737323	52.3869242	32.60614023	40.16567864
ROR1	2162.45517	1777.53445	87.25528677	78.4446168	39.29664614	46.20910469
SDHAP1	345.084478	342.962576	1014.85922	776.269825	564.5618162	917.4863038
RP11-504P24.3	134.210914	163.68553	502.9414788	219.182305	94.34217893	270.8668989
MUC1	209.017798	144.234224	6.017605984	32.1824069	1154.716833	356.9879852
FAAP100	1377.17334	1135.59058	969.8374978	1104.25884	1273.614377	1906.352083
IRF7	330.130915	388.010377	1123.28645	1526.65293	150.1333404	3001.779683
L3MBTL1	381.897005	380.90024	372.0886367	317.801268	89.6769617	250.9788627
BRCC3	1236.52585	1134.57485	2650.755436	3527.99636	1954.756244	4826.586287
SV2B	0	0	0	0	0	0
FAM131C	9.76718684	5.07866986	9.026408976	3.33892472	106.1110206	13.59091314
LMNTD2	143.577647	101.573397	293.8597589	404.291487	53.4031345	256.415228
SPATA45	0	1.01573397	0	7.03990151	0	0
PDE6G	4.88359342	4.06293589	2.005868661	0	1.007606311	2.718182629
PRKG1	0	2.03146794	0	0	9.068456802	1.812121752
NR2F2	6091.79443	4176.69809	2362.913283	2401.61211	10628.23137	3404.976773

DLK1	0	0	0	0	2.015212623	0
TLCD2	180.692957	213.304134	23.06748961	7.03990151	278.0993419	85.16972236
LSAMP	0	0	0	0	0	0
AHNAK2	1504.14677	2878.59008	29.08509559	11.0627024	606.5789994	102.384879
OLFML2A	2197.61704	1968.49244	0	0	172.3006792	6.342426133
SP1	3485.90898	3314.33995	3764.012543	4820.32113	5495.484822	4324.628562
SPATA8	0	0	0	0	0	0
WASH3P	318.224714	240.891469	366.6126152	393.40981	377.5097806	329.5162194
ACTBP7	3.90687474	8.12587178	0	0	5.038031556	0.906060876
MRPL40	457.104344	656.164146	1200.512394	956.420905	537.0541639	963.1427114
DBX2	0	0	0	0	0	0
FAM212A	22.4645297	19.2989455	0	2.01140043	4.030425245	0
PDIA2	0	1.01573397	1.002934331	4.02280086	31.23579565	3.624243505
PCGF3	1293.17554	1249.35279	1389.064048	1402.9518	1665.573233	1350.936766
LMLN	985.509152	1057.37907	380.1121113	384.177482	283.1373735	538.2001604
P4HB	18746.1617	19448.2584	27868.53625	19966.1664	22836.38944	20669.96677
PSMD13	3827.76052	3713.5234	4871.252044	3867.92303	2255.022925	4341.843719
PBX1	137.717334	136.108352	10300.13558	11710.3733	1511.409467	11350.2246
AC017079.3	1.95343737	0	0	1.00570022	0	2.718182629
NDUFA4L2	0	2.03146794	85.24941811	14.079803	12.09127574	7.248487009
SHC4	13.6740616	22.3461474	4.011737323	2.01140043	25.19015778	15.4030349
FTH1P14	0	0	0	0	0	0
KRT79	0	0	0	0	1.007606311	0.906060876
CTD-2287O16.1	13.4103475	14.1694889	23.72942626	18.1830599	23.64852013	10.86366991
ZFP36L1	4623.78625	6370.68347	16318.74449	20061.7079	1948.710606	17305.76274
UBE2L3	2941.75947	2747.50961	4744.872289	4020.56821	3195.835014	4086.071794
NTF3	0	0	0	0	0	0
BRWD1	2592.21139	1943.09909	6917.238079	6583.31361	5514.629342	6469.274656
SMIM23	0	0	0	0	0	0
PMEL	15.6274989	18.2832115	21.06162094	49.2793106	63.47919761	44.39698293
SYN3	0	0	2.005868661	4.02280086	1.007606311	19.0272784
POU3F1	0	0	0	0	6.045637868	0.906060876
SNAI3	3.90687474	4.06293589	8.023474646	10.0570022	20.15212623	19.0272784

ZBTB3	120.136398	112.746471	121.355054	137.78093	161.2170098	129.5667053
LYG2	0	0	1.002934331	0	0	1.812121752
MORN5	0.97671868	0	1.002934331	0	0	0
EP400NL	242.870868	285.187627	738.9118681	571.760687	857.2311454	913.1190904
PRAME	0	0	0	1.00570022	15.11409467	10.87273051
C6orf201	0	0	0	1.00570022	0	4.530304381
MYBL1	2459.37765	1982.71271	109.319842	294.670163	674.0886223	345.2091938
TTTY8B	0	0	0	0	0	0
OR5M5P	0	0	0	0	0	0
SMG1P4	136.906658	119.92771	163.4782959	205.182958	402.7301666	236.5815554
C16orf52	376.036693	447.938682	359.0504904	251.425054	382.8903983	284.5031151
DRG1	1213.07484	1169.07933	2198.401965	1770.00221	1455.890359	2197.152322
ANKFY1	2297.24234	2253.91368	905.6497006	1151.52675	2927.096334	2070.349102
YTHDF3	1839.16128	1914.65854	3097.061213	3561.18446	2035.364749	3618.807139
ZNF696	327.200759	347.381019	348.0182128	324.84117	340.5709332	465.7152904
ADARB2	0	3.04720192	1.002934331	0	2.015212623	0
NRG3	0	0	180.5281795	242.373752	139.049671	210.2061233
SRL	0	0	0	1.00570022	7.053244179	0
C11orf87	0	0	0	0	0	0
IFIT1	849.745255	1288.96641	54.15845386	252.430754	60.45637868	2406.497687
CXorf38	1295.12897	861.342408	732.1420614	630.574035	990.477004	835.3881278
CLDN24	0	0	0	0	0	0
KCNQ5	745.236356	590.141438	3.008802992	1.00570022	33.25100827	48.92728731
ADAMTSL5	303.759511	331.129275	21.06162094	44.2508095	182.3767423	100.5727573
KCNIP4	0	1.01573397	0	0	0	0
SPATA31A6	0	0	0	0	0	0
MORF4L1	6431.49719	6425.83783	2832.226374	2496.77147	7311.554133	3030.429328
NLRP9	0	0	0	0	0	0
WDR53	261.760607	272.216705	495.4495594	534.026814	214.6201443	610.6850305
DMWD	1281.45491	1236.14824	769.2506317	906.135894	1239.355763	962.2004081
SLC52A2	2105.80548	2672.39608	2054.009509	2384.51521	2296.334783	2673.785646
PIGP	166.042176	172.674775	509.49064	398.257285	257.9472157	264.5697758
IKZF1	0	0	0	0	0	0

PCYT2	1400.61459	1940.05189	1248.653242	4036.88067	1252.454645	1379.024654
NAT8L	276.411388	262.059365	61.17899417	70.3990151	417.1490129	104.1970008
OR6C76	0	0	0	0	0	0
NPAP1	0	0	0	0	0	0
BCAP31	3866.82927	3791.73492	8207.011628	7151.53423	7809.956519	9934.957507
ARL17A	585.982374	585.763624	567.4100976	550.369443	356.8538512	597.6921176
RPL12P4	14.4066006	3.05735926	20.01856924	9.49381003	26.09700346	17.74067196
CECR5-AS1	0	0	13.0381463	1.00570022	7.053244179	6.342426133
GNB1L	309.199834	299.235228	600.9983683	498.827307	285.1525861	517.0073966
RP4-592A1.2	5.47939182	6.61242816	6.42880906	7.85451868	6.791266538	4.077273943
DNAH14	338.921383	410.356525	1012.963674	1356.68959	192.4528055	856.227528
LINC01405	0	0	0	0	0	0
CCDC190	197.297174	54.8496345	0	0	0	0
EVI2B	1091.97149	1445.20661	0	0	7.819024976	0
TMEM210	0	0	4.011737323	8.04560172	0	2.718182629
NPIPB4	641.284186	367.411292	710.5890027	1090.41034	716.1763379	734.1177037
ZNF829	192.413581	180.800647	337.9888694	254.442155	212.6049317	324.3697937
TMPRSS11B	0	0	0	0	0	0
THNSL1	251.99342	176.737711	212.6220781	209.185645	670.058197	338.8667677
TRIM69	155.298271	216.351336	585.7136491	823.668477	140.0572773	445.7819511
ATP6V0C	2437.88983	2745.52893	5737.787306	6395.56949	4250.82905	5350.289474
IFITM1	317.765657	282.394359	1.002934331	1.00570022	61.26246373	96.75824097
PRSS38	0.97671868	1.01573397	0	0	0	0.906060876
BPY2C	0	0	0	0	0	0
LAMP1	8139.97351	9099.96066	3263.548312	5012.40987	5893.489315	4215.901257
FFAR3	0	0	0	0	0	0
TAS2R60	0	0	0	0	0	0
POMK	316.456854	311.830329	158.4636243	317.801268	339.5633269	350.6455591
OR11N1P	0	0	0	0	0	0
LINC00839	0	0	0	0	0	0
C16orf54	0	0	0	0	0	0.906060876
KLHDC8B	212.924673	208.225464	90.26408976	54.3078116	138.0420646	123.2242792
KLHL34	0	0	0	0	0	0

SETD4	503.010122	444.89148	434.2705652	360.040677	392.9664614	470.2455947
PTCH1	594.821678	494.662444	299.8773649	340.932373	67.50962286	179.4000535
RTN4RL1	14.6507803	6.09440383	1338.917331	1121.35574	1044.887745	1072.776077
OR4C46	0	0	0	0	0	0
CALHM1	0	0	0	0	0	0.906060876
KRTAP5-5	0	0	0	0	0	0
NKAIN3	0	0	0	1.00570022	0	0
RNPC3	348.288115	378.868772	778.989124	525.397907	194.4680181	523.1867317
ZNF267	371.1531	348.396752	484.4172817	524.975513	645.8756455	691.3244485
IRS2	2523.84108	2597.23177	16.04694929	11.0627024	404.0501308	66.14244396
C7orf61	3.64316069	14.2202756	0	0.98558621	8.06085049	2.718182629
FAM186A	0	0	0	0	0	0.906060876
SHOX	0	0	0	0	0	0
LCE3A	0	0	0	0	0	0
BICD2	2836.39106	2729.27718	885.591014	871.942087	2147.209049	743.8759793
LCE3E	0	0	0	0	0	0
CCIN	1.95343737	1.01573397	0	0	0	0
TMLHE	0	2.03146794	1709.571772	1533.43135	554.788035	1427.218032
GRK1	0	0	0	0	0	0.906060876
DEFB128	0	0	0	0	0	0
SLITRK2	0	0	0	0	0	0.906060876
SDHAP3	8.91744158	2.05178262	41.31086508	24.7603393	64.66817306	30.80606979
PLK5	0	0	2.005868661	5.02850108	0	3.624243505
RASA3	3435.11961	3397.63014	7.020540315	7.03990151	779.8872849	129.5667053
LRCH3	1552.98271	1463.67265	1060.101588	1095.20753	915.914137	1115.360939
LEMD1	0	0	0	0	0	0.906060876
RPS4XP21	0	0	0	0	0	0
ATP4B	0	0	0	0	0	0
NDUFA13	1621.12837	1452.23549	3221.88642	2095.72839	2099.650031	2237.227394
ZNF566	349.665289	343.318083	315.9243142	235.33385	315.3807754	268.1940193
AC084219.4	16.6042176	31.4877531	31.09096425	27.1539058	43.32707139	22.6515219
ZNF529	257.853733	292.531384	548.6050789	432.451093	190.4375928	530.0456126
ZNF284	171.267621	176.879914	187.6389839	113.211673	56.08336729	204.8603641

HTR3E	0	0	0	0	2.015212623	0
DLEU7	0.97671868	1.01573397	0	0	0	0.906060876
KRT73	0	0	0	0	0	0
TAL2	0.97671868	1.01573397	1.002934331	2.01140043	1.007606311	0.906060876
MATN1-AS1	18.557655	15.2360096	63.18486283	41.2337088	50.38031556	51.64546994
AIDA	3987.53216	3579.01991	2556.961017	2671.22023	1835.667254	2639.346272
C15orf41	276.411388	243.776153	130.381463	108.615623	281.1221608	184.8364187
CD300LF	0	0	0	0	0	0
ZPBP2	0	0	0	0	0	0
RP11-887P2.3	148.285431	135.955992	37.45959725	31.5488158	121.1545829	81.48205459
KRT5	0	0	0	0	0	0
KRT18P14	0	0	0	0	0	0
NBPF6	0	0	0	0	183.1324471	3.66048594
GSAP	218.784985	164.548904	400.170798	273.550459	104.7910564	140.4394358
HTR3D	0	0	0	0	1.007606311	0
OR4F5	0	0	0	0	0	0
AGBL4	0	0	11.03227764	6.03420129	4.030425245	18.12121752
ARGFX	0	0	0	0	0	0
CYP2R1	222.69186	283.389778	383.1209143	507.878609	206.5592938	363.3304113
LRRC70	2.93015605	2.79326842	20.05868661	3.01710065	0	8.553214671
ANKRD46	194.034934	217.488958	341.1782006	333.812016	568.4713287	429.8080978
PIP5K1C	2126.31657	2405.25805	2111.176766	2092.86215	1182.929809	2589.521984
OR5D14	0	0	0	0	0	0
CYP4F2	0	0	0	0	0.231749452	0.906060876
OR5L1	0	0	0	0	0	0
TEX38	0	0	0	4.02280086	1.007606311	0
OR5D18	0	0	0	0	0	0
OR9M1P	0	0	0	0	0	0
ZBTB6	367.246225	418.482397	630.845694	513.91281	688.1951106	676.8274745
C2orf76	88.8814002	84.3059197	145.425478	174.991838	139.049671	133.1909488
TAS2R42	0	0	0	0	0	0
POLR3C	747.189793	757.737543	611.7899417	544.083817	1496.295372	737.5335532
PRR30	0	0	0	0	0	0

DEFB131	0	0	0	0	0	0
AC013271.3	0	0	0	1.00570022	0	0.906060876
UBL4B	0	0	1.002934331	0	0	0
LILRP1	0	0	0	0	0	0
WWOX	62.5099958	101.573397	43.12617622	77.4389166	890.7239792	141.3454967
CYP4Z1	0	1.01573397	0	1.23701127	0	3.189334284
CIDECP	187.529987	228.540144	188.5516542	157.894934	322.4340196	202.9576363
TRY2P	0	0	0	0	0	0
CCDC84	1188.66664	919.239245	519.5199833	521.958412	459.4684779	769.2456839
BCL9L	8672.28519	10098.4272	5568.291404	6651.70123	12742.18941	7682.490169
POLR1D	1191.59679	1294.04508	2580.550033	3320.82211	2139.148199	2453.612853
KIF18B	2704.53404	2539.33493	399.1678636	1136.44124	1297.796929	1466.912559
ZNRF1	1341.03475	978.151815	591.7312551	589.340326	3899.436425	901.5305718
FFAR4	0.97671868	6.09440383	3.008802992	4.02280086	1.007606311	7.248487009
BPIFB3	0	0	0	0	0	0
BPIFB4	0	0	0	0	0	0
SAPCD2	1477.77537	1506.33348	6397.718096	8523.30933	5250.636488	8381.969166
EDARADD	4.2291919	4.37781342	467.8788946	171.984794	39.84075355	477.4306575
SLC51B	0	0	0	1.00570022	0	0
CYP4F12	0	0	2.005868661	1.00570022	2.015212623	6.342426133
MARC1	294.969043	282.374044	462.3527265	428.428292	748.6514893	736.6274923
LCE5A	0	0	0	0	0	0.906060876
SOWAHB	15.6274989	5.07866986	61.17899417	93.53012	199.5060496	106.0091225
BLOC1S4	279.341544	288.468448	240.7042394	305.732866	398.004493	344.3031329
SSU72P4	0	0	0	0	0	0
LCE1E	0	0	0	0	1.007606311	0
ZNF749	297.899199	327.066339	205.6015378	182.031739	216.6353569	192.9909666
KLHL32	0	0	9.026408976	4.02280086	0	4.530304381
SSU72P3	0	0	0	0	0	0
FAM86MP	0	0	0	0	0	0
AC016757.3	0	0	0	0	0	0
RP11-1079K10.1	0	1.01573397	0	0	0	2.718182629
MKL2	1043.13555	914.160575	2117.194372	2684.21388	3093.351376	3144.03124

BTLA	0.97671868	1.01573397	0	0	0	0.906060876
OR10D4P	0	0	0	0	0	0
ZNF17	166.042176	183.847849	196.5751288	199.128643	161.2170098	246.4485583
KDM4D	26.3714045	20.3146794	49.1437822	35.1995075	19.14451991	96.04245287
GPAT2	394.76039	358.554092	1.002934331	0	79.60089859	55.26971345
TOR3A	700.307296	830.870389	2045.986035	1549.78403	2692.324064	1653.561099
PABPC1L2A	0	0	0	0	0	0
GABRA5	0	0	0	0	1.007606311	0
PPP1CC	9111.8086	8477.31573	4050.851762	5631.92121	5755.44725	6008.08967
ZNF555	134.787178	119.856609	320.4575773	328.86397	77.80735936	250.507711
MST1P2	0.97671868	3.04720192	53.85757356	65.360457	15.01333404	32.31013084
OR10T2	0	0	0	0	8.06085049	0
NAP1L3	0.97671868	4.06293589	1.002934331	4.02280086	0	0
CA5BP1	460.005199	507.816199	238.648224	290.949072	360.7331355	269.1906863
PRELID2	206.087642	217.36707	70.20540315	140.79803	135.0192457	98.7606355
BACE1	1641.86411	1838.47849	1146.35394	1599.06334	2502.894077	1608.258055
RGS9BP	41.9989034	32.5034871	32.09389858	28.159606	76.57807966	18.12121752
RP11-140K17.2	0	0	0	0	0	0
TMEM212	0	0	0	0	0	0
SLC36A3	0	0	0	0	0	0
SLC36A2	0	0	0	0	0	0
THBS2	62.5099958	61.9597723	0	0	0	0
RXRA	1684.83973	1560.16738	4123.063034	3763.33021	5883.413252	3891.531463
ANKRD37	77.160776	73.132846	106.3110391	49.2793106	9.068456802	20.83940015
C9orf47	2.98875917	2.06193996	74.48793274	118.803366	128.0869143	83.61129765
NUDT17	61.5332771	35.550689	128.3755943	88.501619	187.4147739	70.67274834
KIAA1024L	0	0	0	0	8.06085049	0.906060876
LINC00643	2.93015605	3.04720192	0	0	0	0
ZNF75D	169.949051	157.438766	1437.204896	1463.29381	896.769617	1653.561099
CYP4X1	1.95343737	2.03146794	7.020540315	1.00570022	0	12.68485227
KRT26	0	0	0	0	0	0
KRT10	626.076676	816.650114	382.11798	344.955174	482.6434231	327.0879763
GOLGA8R	4.40500126	3.04720192	68.15941711	18.555169	19.02360716	35.42698026

OR10X1	0	0	0	0	0	0
CD300E	0	0	0	0	0	0
CCDC30	10.9001805	8.59310941	113.7126944	92.0919687	14.10648836	63.1796249
NKRF	739.376044	773.989287	449.3145802	446.530896	1155.724439	591.6577521
GLDN	1.95343737	0	0	0	123.9355763	77.01517448
FCAR	0	0	0	0	0	0
KPNA4	2829.55403	2988.28935	1363.99069	1311.43308	3751.308221	2181.79459
TRDN	0	0	0	0	2.015212623	1.812121752
OR6P1	0	0	0	0	0	0
KRT3	0	0	0	4.02280086	0	2.718182629
ZNF501	117.206242	131.029682	49.1437822	45.2565097	2.015212623	29.90000891
ZNF197	515.707465	407.309323	787.3034496	730.138356	285.1525861	713.9759704
SPATA12	0.97671868	2.03146794	0	0	4.030425245	2.718182629
TMPRSS12	0	0	0	0	0	0.906060876
FAM228A	2.93015605	2.03146794	0	1.00570022	0	1.812121752
DEFB132	0	0	67.19660016	62.3534134	0	30.80606979
NAP1L2	0	0	126.3697257	71.4047153	205.5516875	122.3182183
AQP7P1	0	0	0	0	0	0
RPS23	13975.0277	13888.4861	13206.25815	9811.57107	10689.27216	7390.765749
GNG2	16.6042176	24.3776153	0	0	0	0
BTN3A2	833.668465	1120.28347	29.14527165	27.7372119	18.73140133	65.01892847
AKAP14	1.95343737	0	0	1.00570022	0	0.906060876
PCLO	242.226234	437.781342	347.0152784	493.798806	250.8939715	592.563813
KLK12	0	0	0	0	2.015212623	0.906060876
RGS7BP	0	0	0	0	0	0.906060876
INSIG1	1860.64909	6178.70975	1068.125062	28520.6524	2187.513302	923.2760328
ANKRD20A5P	155.298271	136.057566	501.4370773	642.039018	54.07823073	293.1469359
MYT1L	0	0	0	1.00570022	0	0
C5orf38	0	0	255.7482543	274.556159	304.297106	212.9243059
ZNF396	10.7439055	20.3146794	76.22300913	48.2736103	35.2662209	62.51820046
TMEM222	475.661999	434.73414	944.7641395	750.252361	632.7767635	740.2517358
OR9I2P	0	0	0	0	0	0
OR9Q1	0	0	0	0	0	0

CLCNKA	0	0	1.002934331	4.02280086	1.007606311	3.624243505
OR9Q2	0	0	0	0	0	0
ARHGAP30	17.5809363	41.6450929	67.19660016	90.5130194	25.19015778	59.80001783
SEPT10	3984.03551	3232.0655	478.3996757	756.286562	228.7266327	501.9577254
FAM86B1	64.0629785	64.1131283	39.69614081	30.2615195	198.4178348	78.18399301
CYP4F8	0	0	1335.908529	48.2736103	17.12930729	4129.825474
CYP4F3	0	1.01573397	0	1.00570022	3.798675794	5.436365257
SMYD4	782.351666	735.391396	269.789335	251.425054	705.3244179	424.9425509
CROCCP5	0	0	1.002934331	0	0	0
DEFB105A	0	0	0	0	0	0
FOXD2	15.6274989	8.12587178	14.04108063	19.1083041	46.34989032	28.08788716
GPATCH8	3580.6507	3317.38715	929.7201246	898.090292	1799.584872	1109.018512
CEACAM19	135.851802	117.886085	112.328645	40.2280086	51.41815007	86.13014689
DEFB107A	0	0	0	0	0	0
NF2	552.822775	427.624002	3059.952643	3571.24147	2385.004139	3539.073782
C6orf1	865.372754	1269.66747	721.1097838	1165.60655	224.6962074	416.788003
DEFB106A	0	0	0	0	0	0
SPATC1	0	0	0	2.01140043	0	0
UBE2H	2771.92762	2476.35942	2502.321155	3024.14055	2946.240854	2444.552244
MIR22HG	434.639814	488.568041	109.319842	126.718227	314.3026367	244.6364366
DEFB105B	0	0	0	0	0	0
HPDL	0	0	0	0	365.761091	7.248487009
KTN1-AS1	153.344833	169.627573	75.2200748	86.4902185	64.48680392	135.0030706
KATNA1	400.45466	392.073313	458.3409891	469.662001	387.9284298	530.0456126
FSD2	0	0	2.005868661	0	1.007606311	0
ARAP1	3345.26149	4019.25933	2541.435594	2692.25948	1886.239015	2693.718985
KIF24	616.30949	663.274284	463.3556608	558.16362	512.8716124	921.4639111
PDE2A	231.482328	278.311108	0	0	20.15212623	13.59091314
SPDYE17	0	0	0	0	0	0
LRRC16B	2.93015605	12.1888077	43.12617622	24.1368052	34.25861458	44.39698293
PRG2	0	1.47281426	8.484824438	0	0	5.010516645
PRR5	18.557655	18.2832115	454.2490171	560.17502	425.0486464	625.3722773
ZFP91	5548.73884	5626.15047	3704.638831	4103.25688	3122.571959	4135.261839

C17orf58	209.017798	237.68175	5216.261454	3424.40923	405.0577371	1558.424707
BCDIN3D	51.7660902	44.6922948	462.3527265	369.091979	275.076523	276.3485672
MAGEE2	0	0	0	0	0	0
EEF1GP1	0	0	0	1.00570022	1.007606311	0
RP11-266I3.7	0	0	0	0	0	0
CYP27C1	13.6740616	19.2989455	0	0	0	0.906060876
LYRM7	641.704175	618.581989	508.4877057	360.040677	443.346777	542.7304648
DTX2P1	180.370639	136.128667	76.99526857	101.193556	44.66718778	128.7603111
CFAP73	7.81374947	6.09440383	6.017605984	9.05130194	33.25100827	14.49697402
CCDC73	1.95343737	3.04720192	12.03521197	9.05130194	2.015212623	7.248487009
MST1L	2.93015605	0	4.683703324	11.6661225	1.108366942	7.302850662
BCR	1936.80385	1840.29665	2176.427674	2369.7817	2890.006346	2965.220126
OR10H1	0	0	0	0	0	0
MPPED1	0	0	1.002934331	5.02850108	1.007606311	11.77879139
TPI1P3	0	0	0	0	0	0
FSCN2	25.3946858	6.09440383	72.21127181	37.210908	63.47919761	69.76668747
FOXI2	0	0	0	0	0	0
SPIN4	563.566681	716.09245	337.9888694	536.038215	590.4572984	327.9940372
ZNF732	0	0	37.10857024	24.1368052	84.63893015	90.71481492
SPIN2B	153.042051	193.375434	183.2962783	172.940209	262.6728893	226.9048252
SPATA31D3	0	0	0	0	0	0
FOXE3	0	0	1.002934331	4.02280086	0	4.530304381
HYAL3	170.710892	216.25992	64.03735702	182.242936	89.35452769	168.1467774
KCNK18	0	0	0	0	0	0
IFNA10	0	0	0	0	0	0
VSIG10L	15.6274989	23.3618814	85.24941811	146.832231	36.27382721	106.9151834
CXCR3	0	0	0	0	0	0
ZNF397	202.180768	230.571612	1129.304056	922.227098	587.4344795	975.8275636
ZSCAN30	844.861662	747.580204	778.2770406	492.793106	170.2854666	666.8608049
TPCN1	2322.63703	2117.80533	2042.977232	2439.82872	1329.032725	1511.309541
LILRB4	0	0	0	0	0	0
C2orf27B	0	0	0	0	0	1.730576274
TNFRSF4	0	0	2.005868661	1.00570022	1.007606311	1.812121752

KRT17P2	0	0	0	0	0	0
KRT16	3.90687474	5.07866986	0	0	126.9583952	9.966669638
HEXIM1	3910.78161	3795.79785	3566.43448	3362.05582	1784.470777	3609.746531
SELV	0	2.03146794	0	0	0	0
LINC00846	0	0	14.36201962	3.29869671	0	0
LCE1A	0	0	0	0	0	0
KRT14	0	0	0	0	0	0
TRABD2A	504.96356	400.199185	1.002934331	4.02280086	28.21297672	3.624243505
KRTAP17-1	0	0	0	0	0	0
PDZD7	38.0920287	66.0227082	239.701305	83.4731179	33.25100827	96.94851375
POFUT2	1304.89616	1410.85449	928.7171902	657.727941	670.058197	889.7517804
QRFPR	0	0	0	0	0	0
MAPT	58.603121	61.9597723	427.2500249	206.168544	962.2640273	571.7244129
ERCC6L	1368.38288	1379.36673	222.6514214	416.359889	687.1875043	768.339623
OR13F1	0	0	0	0	0	0
OR5D3P	0	0	0	0	0	0
TMEM17	29.3015605	47.7394967	32.09389858	30.1710065	44.3346777	29.90000891
TNFRSF18	0.97671868	8.12587178	0	0	177.3387108	17.21515665
FGF3	0	0	0	0	0	0
C1QL4	2.93015605	5.07866986	2.005868661	1.00570022	11.08366942	0
RTN4RL2	251.99342	352.459688	818.3944139	869.930686	35.2662209	460.2789251
ZDHHC17	1364.476	1137.62205	796.3298586	684.881847	743.6134577	650.5517091
SERPINA11	0	0	0	0	0	1.812121752
P2RY4	0.97671868	0	7.020540315	9.05130194	1.007606311	5.436365257
ZNF395	2396.86765	1900.43826	1107.239501	1377.8093	1531.984788	1566.579255
ZACN	25.3946858	29.4562852	54.15845386	50.2850108	43.32707139	43.49092206
KRTAP22-1	0	0	0	0	0	0
KRTAP19-6	0	0	0	0	0	0
KRTAP6-2	0	0	0	0	0	0
CHCHD2P9	0	0	1.002934331	1.94100142	0	1.621848968
OR13C8	0	0	0	0	0	0
PPARA	1287.31523	1628.22156	529.5493266	427.422592	244.8483336	658.706257
TMEM232	2.93015605	3.04720192	24.07042394	13.0741028	0	9.060608762

LINC01551	0	0	0	0	0	0
KRTAP19-2	0	0	0	0	0	0
KRTAP19-4	0	0	0	0	0	0
KRTAP15-1	0	0	0	0	0	0
KRTAP13-4	0	0	0	0	0	0
FAM183A	0	0	0	0	2.015212623	0.906060876
EFCAB6	2.93015605	1.01573397	28.08216126	23.131105	18.1369136	15.4030349
KRTAP19-5	0	0	0	0	0	0
KRTAP23-1	0	0	0	0	0	0
KANK3	0.97671868	2.03146794	1.002934331	14.079803	17.12930729	5.436365257
EMID1	1.95343737	0	4.011737323	12.0684026	124.9431826	27.18182629
ACTL7A	0	0	0	0	0	0
KRTAP21-1	0	0	0	0	0	0
RHD	0	0	1.002934331	0	1.007606311	0
LINC00207	0	0	0	0	0	0
C17orf82	10.7439055	10.1573397	0	0	355.6850279	49.83334819
ESPN	33.2084353	48.7552307	2813.230798	3034.19755	1527.531168	3216.51611
PNLIPRP1	0	0	0	0	0	0
PTRH1	171.902488	192.989455	92.26995843	89.5073192	278.0993419	172.1515665
KRTAP21-2	0	0	0	0	0	0
SAMD7	0	0	0	0	0	0
GPR141	0	0	0	0	0	0
TMPRSS6	0.97671868	0	0	0	19.14451991	12.68485227
CYP4A11	0	0	0	0	0	0
TMEM216	337.944665	388.010377	166.4870989	159.906334	309.3351376	169.4333838
RPS19BP1	1254.10679	1388.50834	3042.902759	2183.37517	1308.880598	2486.231044
TMPRSS11A	0	0	0	0	1.007606311	0.906060876
TMEM262	13.6740616	6.09440383	11.03227764	8.04560172	6.045637868	6.342426133
C3orf70	6.83703079	18.2832115	4.011737323	1.00570022	50.38031556	8.154547886
TEAD1	11524.3038	8383.86821	21.06162094	52.2964112	3864.170204	356.9879852
OR2AK2	0	0	0	0	0	0
DEFB106B	0	0	0	0	0	0
PLCD1	305.712948	346.365285	183.5369825	251.425054	94.71499326	155.8424707

CCK	0	0	0	0	1.007606311	0
ENTPD5	795.049009	736.40713	999.9255277	1021.79142	653.936496	1162.476104
MITF	624.123239	385.978909	124.363857	175.997538	153.1561593	248.2606801
FUNDC2P3	0	0	0	0	0	0
HEATR4	0.97671868	8.12587178	15.04401496	23.131105	2.015212623	6.342426133
NAP1L1	17717.6769	18207.0315	7445.784471	7198.79209	9915.853709	6071.504871
LILRA5	0	0	0	0	0	0
CMC1	369.199662	399.183451	406.1884039	351.995075	527.9857071	323.4637328
SLIT1	0.97671868	1.01573397	1.002934331	6.03420129	26.19776409	12.68485227
LYPD6	401.431379	372.774368	34.09976724	157.894934	889.7163729	113.2576095
AKR1C1	13.6740616	35.6725771	0	0	239.961443	98.5522415
VSTM2B	0	0	0	0	0	0
FOXD3	22.4645297	19.2989455	1.002934331	0	13.09888205	1.812121752
SPATA21	3.90687474	3.04720192	8.023474646	2.01140043	3.022818934	0
RNF220	1408.42834	1375.3038	1831.358088	1619.17735	1424.755324	1584.700472
ANGPTL5	0	0	0	0	0	0
SHTN1	916.162125	866.421078	3163.254879	2345.2929	2260.060956	1968.870284
H1FNT	0	0	0	0	0	0
LCE4A	0	0	0	0	0	0
BAGE2	0	1.03604865	1.033022361	1.03587122	236.5053534	15.33055002
LCE2A	0	0	0	0	0	0
KRTAP12-1	0	0	0	0	1.007606311	0
LCE2C	0	0	0	0	0	0
CTD-2600O9.1	13.6740616	11.1730737	0	0	2.015212623	1.812121752
RP11-195F19.5	30.6396651	34.5349551	101.25625	102.410453	15.63804995	46.6349533
ZNF546	150.414677	147.281426	104.3051704	108.615623	14.10648836	60.7060787
TSPYL4	453.197469	369.727166	709.0745718	726.115556	1169.830927	1035.627581
DAZ3	0	0	0	0	0	0
MT1X	669.022997	962.001645	150.4401496	50.2850108	289.1830113	147.6879228
GCNT1	465.894812	448.954416	110.3227764	507.878609	413.1185876	130.4727662
LCE2D	0	0	0	0	0	0
RP11-161I2.1	26.3714045	14.2202756	0	0	4.030425245	1.812121752
SESTD1	752.073387	816.650114	255.7482543	301.710065	1154.716833	256.415228

LCE3B	0	0	0	0	0	0
FNBP1	2821.74028	2197.03258	976.8580381	1366.74659	1715.953548	1362.715558
DYNC2H1	726.678701	823.760251	1093.19842	1121.35574	442.3391707	1046.500312
KRT12	0	0	0	0	0	0
MAGED4B	0.89858119	0	0	0	71.85240606	23.45791608
BCAM	1371.31303	820.71305	7016.528578	9156.90046	2616.75359	6553.538317
RSBN1L	920.069	830.870389	988.8932501	1021.79142	1003.575886	978.5457463
NPSR1	0	0	0	0	0	0
WDR86	2.93015605	0	2.005868661	0	0	0
EPOR	222.69186	264.090833	409.1972069	426.416891	89.6769617	267.2879585
FAM9C	0	0	0	0	0	0
KRTAP9-8	0	0	0	0	0	0
CIDEC	0	0	0	0	1.007606311	1.812121752
DCC	0	0	0	0	0	0
TAF9B	662.215268	757.737543	721.1097838	1085.15053	1134.564707	752.0305272
PCDHB13	277.593217	313.059368	40.57872302	22.1254047	135.3517558	35.20952565
MAGI2	14.6507803	30.4720192	65.1907315	34.1938073	8.06085049	18.12121752
LUZP2	0	0	5.014671654	0	10.07606311	15.4030349
LHFPL3	0	0	0	0	0	0
CHP1	2433.00624	2696.7737	4218.341795	7521.63191	2233.863192	4525.774077
RP11-67P15.1	0	0	0	0	0	0
FPR3	0	2.03146794	35.10270157	21.1197045	0	9.060608762
HIST1H1T	0	0	0	1.00570022	0	3.624243505
C11orf96	4.88359342	4.06293589	0	0	4.030425245	7.248487009
HSD3BP1	0	0	0	0	0	1.812121752
SERPINA13P	0	0	0	0	0	0
KCNJ11	13.6740616	17.2674775	389.1385203	527.992613	146.1029151	216.5485494
CDHR4	0	1.01573397	0	0	0	0
COL4A1	3706.64741	3101.03582	0	0	173.3082855	85.16972236
RPL7P48	0	0	0	0	0	0
PLEKHG7	1.95343737	0	0	0	0	0.906060876
GJA4	0	0	0	0	1.007606311	0
PTMA	21482.8884	21218.4491	22293.17416	30977.5177	37531.25943	34669.43182

HYPM	0	0	0	0	2.015212623	0
HSPA14	1843.06816	1691.19706	813.3797422	915.187196	1586.97994	1216.839757
ATP13A5	0	0	42.12324189	18.1026039	0	32.61819154
SIRT7	707.144327	737.422864	692.0246882	722.092755	1243.386188	868.0063194
PRR27	0	0	0	0	0	0
PRR13P5	15.3540177	11.660626	16.69885661	8.71942087	26.59073055	11.23515486
IFT140	342.828258	347.381019	687.0100165	820.349666	660.9897402	832.6699452
TPM3P7	0	0	1.002934331	3.01710065	0	0
POTEG	0	0	0	0	0	0
PRAMEF10	0	0	0	0	0	0
AGMO	4.88359342	3.04720192	0	0	2.015212623	1.812121752
SBK2	5.8603121	0	0	3.01710065	3.022818934	12.68485227
CYP26C1	0	0	1.002934331	1.00570022	0	1.812121752
TLR5	21.487811	30.4720192	278.8157439	150.855032	23.17494516	141.3454967
USP7	3942.03661	3574.36785	5364.695735	4986.26167	6311.645934	6141.280619
NANOS3	4.88359342	2.03146794	0	1.00570022	0	0
FOXD4L3	1.17206242	9.80183283	15.91656783	4.63627799	5.038031556	11.54321556
NHLRC1	28.3248418	37.582157	105.3081047	34.1938073	47.35749663	55.26971345
DPPA3	0	0	0	0	0	0
COX8C	0	0	0	0	0	0
PLEKHN1	177.7628	131.029682	52.1525852	119.678326	94.71499326	159.4667142
TERF1P2	0	0	0	0	1.007606311	0
ZNF385C	7.81374947	5.07866986	21.06162094	8.04560172	10.07606311	15.4030349
TMEM247	0	0	0	0	0	0
MAGEH1	57.6264023	49.7709646	654.916118	463.627799	150.1333404	240.1061322
TET3	1686.79317	1705.41734	1782.214306	2433.79452	5815.903629	2728.149298
ZNF286A	888.735865	849.884929	556.6285535	597.627296	949.4371989	716.8753652
ISG15	969.881653	1387.49261	520.5229176	527.992613	515.8944314	2725.431116
EXD3	204.134205	174.706243	666.9513299	419.37699	311.3503502	410.4455769
OR5W2	0	0	0	0	0	0
TMEM8C	0	0	0	0	0	0
TCL6	0	0	0	0	0	0
C17orf97	29.3015605	40.6293589	169.4959019	122.695426	69.52483548	105.1030616

ZKSCAN4	140.627956	158.26151	180.1871819	181.639516	129.9812142	183.5407517
RGPD1	9.01511345	27.7396948	116.6914094	117.697096	83.11744462	116.1751255
DHRS4L2	120.136398	168.469637	355.3496627	309.001391	347.876079	267.2607766
SAMD11	13.6740616	14.2202756	1128.301122	1392.8948	421.1794381	1835.679335
PERM1	0.97671868	2.03146794	52.1525852	77.4389166	15.11409467	33.52425242
VMAC	39.0687474	44.6922948	258.7570573	230.295292	79.60089859	204.0449093
TMSB4XP8	0	0	0	0	0	0
TSPY13P	0	0	0	0	0	0
C5orf52	0	0	0	0	0	0
HAPLN4	0	0	1.002934331	1.00570022	1.007606311	0
ERC2	1.95343737	0	3.008802992	0	41.31185876	9.060608762
B3GLCT	717.888233	546.464877	206.6044721	497.821607	269.0308851	337.9607068
SPRY4	2109.71236	2421.50979	840.4589691	722.092755	190.4375928	978.5457463
ERAS	2.93015605	1.01573397	1.002934331	1.00570022	0	0
KRT18P59	0.97671868	4.06293589	0	0	5.038031556	2.718182629
TRPV2	40.045466	69.0699101	1.002934331	1.00570022	1.007606311	4.530304381
AMTN	0	0	3.008802992	0	45.34228401	5.436365257
CXorf67	0	0	0	0	0	0
RP11-723O4.6	0	0	113.8731639	52.1556132	6.045637868	101.6872121
C2orf88	52.7428089	54.8496345	0	1.00570022	6.045637868	3.624243505
OR2T27	0	0	0	0	0	0
TMEM203	819.466976	913.144841	1369.005361	1259.13667	1276.637196	1579.264107
SLC18A3	0	0	0	0	0	0
KBTBD12	0	0	0	0	0	0
THSD4	1930.97284	1705.41734	1298.799958	743.212459	1877.170558	1080.930625
GTF2IP3	0	0	0	0	0	0
DNAJB13	2.93015605	0	11.03227764	1.00570022	1.007606311	7.248487009
GABRD	0	0	0	0	61.46398499	2.718182629
AMY1C	0	0	0	0	0	1.141636704
TCEA1	2877.2472	2829.33714	8571.397729	2928.25709	2676.756546	3187.34095
NHEJ1	213.696281	219.713416	154.3315348	182.655273	271.3483796	193.1268758
FANCA	1784.46504	1541.88417	650.9043806	554.140819	2718.521828	1197.812478
SECISBP2	1223.82851	1127.46471	1183.46251	1041.90542	1847.949975	709.445666

OR52B6	0	0	0	0	0	0
C9orf153	0	0	0	1.00570022	0	2.718182629
SSX7	0	0	0	0	0	0
ADH1A	0	0	0	0	0	0
HSPE1P11	3.90687474	0	2.005868661	1.00570022	5.038031556	1.812121752
OR2B7P	0	0	0	1.00570022	0	0.906060876
SEMA4D	1309.77976	1296.10702	427.2500249	394.234484	685.1722917	349.7394982
KRTAP10-8	0	0	0	0	0	0
LIN28B	0	0	0	0	0	0
FAM69C	0	0	0	0	1.007606311	0
DNAH17	2.93015605	8.12587178	0	0	19.14451991	9.060608762
MCRS1	1307.82632	1354.98912	1337.914397	1280.25637	2479.719132	1187.845809
TMEM72	0	0	0	0	0	0
FANCM	642.680894	615.534787	693.0276225	692.927449	602.5485742	1128.951852
FAM205C	0	0	0	0	0	0
ZNF70	169.949051	204.162528	452.3233831	536.038215	190.4375928	449.4061946
CARD9	23.5389203	33.6614238	16.01686126	18.836765	17.95554447	28.49561456
PEAR1	226.598735	144.234224	75.2200748	27.1539058	32.24340196	101.4788181
ZFP69B	82.0443694	116.809407	68.19953449	73.4161157	83.63132384	87.88790499
TMEM202	0	0	0	0	0	0
SOWAHD	5.8603121	5.07866986	4.011737323	2.01140043	19.14451991	6.342426133
RP11-24M17.5	0	0	0	0	0	0
ZFP69	94.7417123	101.573397	97.28463008	71.4047153	148.1181278	110.5394269
HELT	0	0	0	0	0	0
ZCCHC16	0	0	1.002934331	0	0	0
TMEM220	0	0	0	1.00570022	0	0
C2orf78	0	0	0	0	2.015212623	0
HIST1H1C	865.372754	821.728784	1083.169077	1090.17903	367.7763036	1582.888351
TMEM256-PLSCR3	906.082389	816.964991	148.4342809	142.266352	708.2364001	293.8717846
EIF4EBP1	1788.37191	1512.42788	5737.787306	1862.5568	1389.489103	1783.127804
OR7E25P	0	0	0	0	0	0
P2RX2	0	0	0	0	91.69217433	23.55758278
ASCL4	0	0	0	1.00570022	1.007606311	2.718182629

OR6C75	0	0	0	0	0	0
CCDC157	48.8359342	74.14858	151.4430839	118.672625	61.46398499	219.266732
TTC24	0	0	0	0	0	0.906060876
FAM122A	351.618726	400.199185	583.7077805	445.525195	439.3163517	300.8122109
PALM3	0	0	12.03521197	8.04560172	428.2326823	58.89395695
RNFT1P3	0	0	3.008802992	0	4.090881624	1.83024297
GFRAL	0	0	0	0	0	0
C1orf168	0	0	17.04988362	19.1083041	2.015212623	38.0545568
CXXC1P1	0	0	0	0	0	0
OR5H7P	0	0	0	0	0	0
SHISA7	0	0	0	0	0	2.718182629
AC097382.5	0.97671868	0	0	1.00570022	0	0.906060876
LRRC74B	1.95343737	2.03146794	1.002934331	0	0	0
DMBT1	253.946858	151.344362	115.337448	129.735328	4.030425245	199.3333928
CLEC17A	0.97671868	0	0	0	0	0
OR51I2	0	0	0	0	0	0
LCN10	0.97671868	0	0	0	0	0
LDLRAD2	9.76718684	5.07866986	0	16.0912034	5.038031556	7.248487009
C2orf66	1.95343737	1.01573397	0	1.00570022	1.007606311	0.906060876
OVCH1	0	0	3.008802992	12.0684026	0	11.77879139
ARHGAP11B	840.739909	706.788327	1169.401371	1171.40944	673.4739824	1538.572913
HS6ST1P1	8.13606664	4.20513865	3.91144389	11.0325314	20.80707033	10.50124555
PMS2CL	403.179706	332.52083	614.9692436	377.368892	531.9355239	536.079978
CYHR1	1583.26099	1511.41215	2763.084081	2916.53063	1307.872992	2756.237185
COL14A1	0.97671868	0	0	0	7.053244179	0
DNER	58.603121	124.935279	0	5.02850108	84.63893015	8.154547886
CPSF4L	0	1.01573397	1.002934331	0	1.007606311	0.906060876
KLHL17	441.476845	343.318083	476.3938071	552.129418	177.3387108	512.8304559
ZCCHC13	0	0	0	0	0	0
AC008079.9	0	0	0	0	0	0
PLA2G2C	0	0	0	0	0	0.906060876
ANKRD19P	0.97671868	0	0	0	7.224537252	0
ZSCAN23	12.6973429	15.2360096	90.26408976	54.3078116	0	78.82729623

KCTD9P3	0	0	0	1.00570022	0	0
RINL	4.88359342	4.06293589	11.03227764	4.02280086	90.68456802	14.49697402
C17orf99	0	0	0	0	0	0.906060876
HNRNPA1P61	0	0	0	0	0	0
OR7D2	0	1.01573397	0	0	0	3.624243505
TPRG1	1.95343737	3.04720192	749.191945	112.638424	10.07606311	385.9819333
RP11-43F13.1	36.2948663	39.6237823	212.5819607	283.004041	441.2207277	256.1162279
C1orf204	28.3248418	37.582157	7.020540315	3.01710065	1.007606311	4.530304381
MORN2	163.11202	207.20973	124.363857	102.581422	278.0993419	186.6485405
RTP5	0	0	0	0	0	0
MEIS3P2	41.4714753	30.4212325	12.55673782	5.37043915	47.75046309	35.99779861
S100A3	335.014509	310.814596	1.002934331	0	10.07606311	4.530304381
UBQLN2	1192.57351	1356.00485	1135.321662	1354.67819	1343.139213	670.4850484
RILPL1	433.077064	491.137848	472.8033022	424.747429	412.2318941	349.304589
CTSL3P	0	0	0	0	0	0
C19orf67	0	0	0	0	0	0
ZNF490	73.449245	83.9300981	127.1620438	178.391104	161.0054125	202.7945453
CLCN1	0	1.01573397	0	0	1.007606311	0
NRN1L	1.39670772	1.01573397	1.103227764	1.21689726	2.780993419	0
NWD1	11.7206242	32.5034871	2.005868661	47.2679101	0	40.77273943
ARL4C	1748.32644	1639.39463	3.008802992	2.01140043	419.1642255	35.33637417
RNF133	0	0	3.008802992	5.02850108	0	2.718182629
TMEM221	2.93015605	13.2045416	0	0	6.881951106	7.248487009
TREML4	0	0	0	0	2.015212623	1.812121752
RAB42	108.415774	87.3531216	0	0	7.053244179	2.718182629
WNT7B	436.593252	319.956201	1001.931396	939.324001	1259.507889	1310.164027
OR51F1	0	0	0	0	0	0
C11orf95	988.439308	824.775985	226.6631587	247.402253	246.8635463	388.7001159
SCGB1C1	0	0	0	3.01710065	0	0
RP5-1119A7.11	0	0	0	0	0	0
PRSS45	0.97671868	0	0	0	0	0.906060876
PLA2G4E	0	0	0	0	0	3.624243505
GPR89B	217.505484	195.467846	602.8136795	510.563828	787.0312137	579.2265969

MESP2	2.93015605	3.04720192	0	0	6.045637868	1.812121752
FAM25A	0.97671868	1.90957987	0	0	11.01313698	0
ALOX15P2	0	0	0	0	0	0
EYS	0.97671868	0	0	0	1.007606311	1.812121752
C6orf132	2055.01611	1175.20421	2087.106342	2873.28552	567.2823533	1495.906507
DAZ1	0	0	0	0	0	0
OR2AG2	2.93015605	6.09440383	0	0	0	0
MAPK12	1014.07817	993.337038	79.24184147	66.3460432	327.0085523	154.7733189
TMEM215	0	0	0	0	0	0
NUTM2G	0.97671868	0	0	0	1.511409467	3.624243505
COL4A5	16.6042176	14.2202756	1619.738944	2080.79375	553.1758649	2782.512951
KRTAP10-6	0	0	0	0	0	0
AGRN	12085.9854	10528.4889	7633.082457	7670.72697	11048.4032	15245.335
NHS	922.022438	780.083691	227.6660931	118.672625	288.175405	308.0606979
OTOG	0	0	0	0	1.007606311	0.906060876
FAM166A	2.93015605	4.06293589	3.008802992	20.1140043	2.015212623	5.436365257
TMPPE	162.135302	173.690509	104.3051704	72.4104155	164.2398287	121.4121574
ZNF626	209.144772	222.364481	0	0	0	1.812121752
HEPACAM2	0	0	0	1.00570022	0	0
SMTNL2	0	1.01573397	0	0	107.8138753	2.718182629
ZC3H6	70.3237452	74.14858	621.819285	593.363127	276.0841293	356.0819243
LINC00265	42.4970299	80.3039278	92.25992908	129.232478	147.0903693	130.3549783
LAMTOR4	404.361535	535.291803	545.5962759	1056.99093	921.9597748	440.3455858
PRKAR1B	607.519021	514.977124	623.8251537	668.790643	1794.54684	1325.567062
NUTM2B	28.3150746	37.7548317	59.57429924	45.4978778	45.95692386	50.44040898
HNRNPU-AS1	638.471236	498.390188	2528.297154	1337.07844	352.4707638	1999.649172
NCR3LG1	41.0221847	55.8653685	258.7570573	1251.09107	37.28143352	171.2455056
DCUN1D3	497.970254	467.004008	392.4181156	384.177482	304.9520501	469.3395339
POTEE	1.95343737	5.07866986	2.647746633	0	73.73662986	10.12069999
AC002398.9	0	30.675166	65.73231603	0	20.85745064	41.21670926
ZNF793	3.90687474	8.12587178	340.9976724	227.288249	318.4035944	311.6849414
TUBB4B	17313.6768	17070.74	8752.66808	14347.7417	22648.93436	17946.81893
AGAP4	352.380567	325.684941	408.2143313	312.551513	143.5939754	386.5074486

PP7080	326.22404	306.75166	657.9249209	618.505633	553.1758649	514.6425777
COMMD6	537.195276	620.613457	480.4055444	439.490994	498.7651241	334.3364633
PLA2G2A	0	0	174.5105735	189.071641	0	28.08788716
IL17REL	0.97671868	0	4.011737323	12.0684026	9.068456802	6.342426133
HYKK	52.7428089	44.6922948	34.09976724	70.3990151	188.4223802	68.86062659
OR7A5	0	0	0	0	0	0
C15orf62	12.6973429	17.2674775	54.15845386	28.159606	5.038031556	28.99394804
AC007731.1	0	0	0	0	0	0
RUFY4	0	0	0	1.00570022	0	2.718182629
ZNF383	208.04108	194.005189	292.8568246	241.368052	115.8747258	278.160689
HES4	213.901392	152.360096	1378.03177	1146.49825	663.0049528	877.0669281
IGFL1	0	0	0	2.01140043	188.4223802	0
ZNF669	182.646394	172.674775	374.0945054	451.559397	242.833121	420.4122465
C19orf35	0	0	0	1.00570022	0	0
LRRIQ4	0	0	0	0	3.022818934	0.906060876
CENPP	450.267313	437.781342	234.6866334	357.023577	637.814795	318.9334284
PLSCR1	553.799494	570.842492	250.7335827	286.624561	624.715913	726.6608227
OR7D1P	0	0	0	0	0	0
C3orf62	317.433572	339.255147	412.2060099	317.801268	124.9431826	397.7607246
ENO4	10.7439055	10.1573397	10.02934331	9.05130194	1.007606311	2.718182629
ZNF559	388.265211	427.93888	363.8344872	302.937019	101.7682374	260.7552596
SBK1	10.7439055	8.12587178	1314.846908	1283.27348	687.1875043	1910.882388
OR6C6	0	0	0	0	0	0
BSPH1	0	0	0	0	0	0
SLC38A3	0	0	23.06748961	21.1197045	6.045637868	96.04245287
OR6N2	0	0	0	0	0	0
GTF2F2	1427.96272	1432.1849	574.6813715	561.18072	807.0926553	704.0093008
FAM92A1	678.799951	814.578016	3.008802992	1.00570022	438.2583651	49.83334819
FOCAD	69.3470266	43.6765608	809.3680049	1092.19043	1317.949055	1066.433651
AC092171.2	0	0	1.002934331	2.01140043	0	0
PRR19	95.718431	97.5104613	56.16432252	153.872133	78.59329228	104.1970008
ZP3	5.91891522	0	0	3.88200283	411.7583191	40.67307273
C10orf99	0	0	0	0	0	0

H3F3C	0	0	1.012963674	2.03151444	5.088411872	2.745364455
IFNA2	0	0	0	0	0	0
AC018892.9	0	0	0	0	0	0
JAKMIP3	2.93015605	2.03146794	149.4372153	220.248347	42.31946507	168.527323
PPP3R2	0	0	0	0	0	0
GOLGA6L3	0	0	0	2.42373752	1.400572773	0
PDCD1	0.97671868	0	0	0	0	0.906060876
CLEC2A	0	0	0	0	0	0
GPR21	0	2.03146794	0	2.01140043	0	0.906060876
TCTEX1D4	0.97671868	1.01573397	2.005868661	0	0	1.812121752
ANKRD36P1	0	0	0	0	0	0
IGHV1OR15-9	0	0	0	0	0	0
SELL	1.95343737	8.12587178	101.2963674	101.575722	58.44116605	174.8697491
MAGEB5	0	0	0	0	0	0
CHM	859.512442	878.609886	842.4648378	1025.81422	629.7539446	690.4183876
NANOS2	0	0	0	0	0	0
BLOC1S5	525.992313	545.449143	363.8244578	315.085878	458.8840663	383.2637506
RP11-1396O13.14	0	0	0	0	0	0
OR4P1P	0	0	0	0	0	0
SRP72P2	0	1.01573397	13.0381463	0	0	0
CERKL	36.1385913	42.6608268	0	0	20.15212623	1.812121752
WASF4P	5.88961366	4.08325057	4.041825353	6.0543153	0	3.633304113
ACTBP11	0	0	0	0	0	0
SLC24A5	1.95343737	1.01573397	2.005868661	0	0	2.718182629
AC003003.5	0	1.01573397	0	1.00570022	1.007606311	0.906060876
IER5L	562.589962	834.933325	517.5141146	1951.05842	2345.707493	701.2911182
H2AFX	6904.42438	5990.79897	2204.449659	3521.96215	7622.541745	4658.965025
INSC	2.93015605	5.07866986	0	0	0	0
SERPINA5	28.3248418	27.4248173	0	0	80.6085049	130.4727662
C19orf54	495.196373	545.449143	449.3145802	645.659538	235.7798768	403.1970899
LCTL	1.95343737	1.01573397	1.002934331	3.01710065	3.022818934	0.906060876
NCCRP1	0.97671868	1.01573397	0	0	156.1789782	17.21515665
KRTDAP	0	0	0	0	0	0

C22orf34	0	0	0	0	0	0
RP1-273G13.2	0.97671868	1.01573397	1.002934331	1.00570022	3.022818934	0
COL25A1	0	1.01573397	11.03227764	2.01140043	0	3.624243505
FAM83G	2433.00624	2481.43809	979.8668411	1827.35729	1497.302979	1171.536713
CFAP77	0	0	0	0	1.007606311	0
AC010969.1	0	0	0	0	0	0
SRSF10	5017.40388	4194.98131	3369.859351	2714.38488	3765.424785	4527.586198
HBA2	0	1.01573397	0	0	1.622246161	0
DUSP28	66.4168705	74.14858	100.2934331	179.014638	140.0572773	172.1515665
C15orf52	3666.60194	3633.28042	209.6132751	73.4161157	339.5633269	263.663715
NBR1	4793.7353	3949.17368	2348.872203	2130.07306	2271.144626	2384.752226
OR2G6	0	0	0	0	0	0
RALGAPA2	694.446984	904.003235	1146.35394	1377.8093	1591.010366	2174.546103
NDOR1	727.655419	698.824973	876.564605	956.420905	758.7275524	1650.842916
FBLL1	1.95343737	1.01573397	5.014671654	2.01140043	1.007606311	6.342426133
NKAIN2	0	0	228.6690274	259.470656	0	353.3637417
KRTAP1-1	0	0	0	0	0	0
PAQR9	0	0	0	0	0	0
LINC00083	0	0	0	0	0	0
CFAP54	9.76718684	9.14160575	6.017605984	2.01140043	3.022818934	20.83940015
NPIPP1	69.2102859	85.900622	256.9718342	139.430278	135.0293218	151.8376816
CLN3	448.313876	580.35992	2700.149952	6340.26604	1334.070756	2522.600328
FAM72B	533.366539	564.859819	83.15328536	285.498177	394.2763496	230.2028868
ASAH2	78.4598119	94.5140461	77.56694114	78.5250728	58.92481708	73.19159758
SUMO2	5564.00496	5023.57645	17593.23333	17752.0771	5218.151261	15988.10558
NANOS1	790.165415	316.908999	1730.06172	398.257285	110.8366942	424.0364901
HMX3	0	0	0	0	1.007606311	0
IGFL3	0	0	0	0	0	0
GOLGA8M	5.36218557	2.40728951	0	0	0	0
ZNF177	30.2392105	19.6036657	224.215999	126.939481	2.468635463	97.46496845
LDOC1L	1221.87507	1239.19545	1606.700798	1429.10001	1296.789323	1824.806605
DPYD	1681.90957	1436.24784	75.2200748	99.5643213	180.3615297	143.1576184
S100A16	3299.35571	3858.77336	7015.525643	13713.7281	4525.159944	8040.384215

RP11-361A23.2	0	0	0	0	0	0
PTAR1	2268.9175	1774.48725	1028.007689	865.907886	1100.306092	796.4275102
CC2D2B	0	0	0	2.01140043	0	0.906060876
RNASE9	0	0	0	0	0	0
TSPY7P	0	0	0	0	0	0
SAXO2	2.93015605	3.04720192	19.05575228	8.04560172	2.015212623	9.966669638
LINC00319	0	1.01573397	0	0	0	0
HILS1	0	0	1.002934331	0	0	0
OR7E90P	0	0	0	0	0	0
RHCE	2.93015605	1.01573397	4.011737323	4.02280086	5.038031556	3.624243505
C2orf80	0	0	0	0	0	0
IDO2	0	0	0	0	0	0
PARVB	0.97671868	5.07866986	5.014671654	1.00570022	15.11409467	2.718182629
TEKT4P2	1.46507803	1.01573397	123.2907173	92.5244198	54.23944774	100.3643633
SLC4A5	22.9040531	25.1597305	76.77462302	55.4341959	13.09888205	151.203439
UROS	1456.28756	1433.20063	994.9108561	725.109855	811.1230806	849.8851019
OR56A5	0	0	1.002934331	0	0	0
CYP51A1-AS1	0	0	2.005868661	3.01710065	5.038031556	4.530304381
KRTAP24-1	0	0	0	0	0	0
ZDHHC9	949.370561	1050.26893	1613.721338	1402.9518	2083.729852	1637.252003
ZBED6CL	350.642008	232.60308	75.2200748	124.706827	225.7038137	125.0364009
QRFP	0	0	0	0	0	3.624243505
OR13D3P	0	0	0	0	0	0
DUPD1	0	0	0	0	0	0
SMIM15	2354.86875	2248.83501	1218.565212	1140.46404	1818.729392	1315.600392
OSTN	0	0	0	0	0	0
VWC2	0	0	0	0	0	0
FAM221A	127.950148	157.438766	306.8979052	355.012176	78.59329228	236.4818887
TMEM120B	860.48916	978.151815	481.4084787	314.784167	979.3933346	642.3971612
FSIP2	16.6042176	18.2832115	502.4700997	265.504857	5.038031556	241.9182539
RBM34	941.537277	1058.01898	940.3512285	992.696512	638.3085221	991.4933562
NOXA1	111.34593	86.3373876	710.0775061	272.544758	148.1181278	231.9515843
TBC1D3P2	0	0	0	0	0	3.624243505

TMEM198	65.4401518	81.2587178	79.23181213	134.763829	84.63893015	112.3515486
BCL2L15	4.88359342	3.04720192	12.03521197	8.04560172	14.10648836	6.342426133
FZD9	0	1.01573397	0	0	46.34989032	1.812121752
TMSB4XP2	0	0	0	0	0	0
SPRED3	193.390299	281.35831	96.28169575	100.570022	66.50201655	142.2515576
OPTC	0	0	0	0	0	0
PLET1	0	0	0	0	0	0
ADRB3	0	0	0	0	0	0
SKOR1	15.6274989	11.1730737	16.04694929	27.1539058	6.045637868	25.36970453
CATSPER4	0	0	0	0	0	0
PRELP	4.88359342	3.04720192	1.002934331	7.03990151	3.022818934	2.718182629
PLA2G2E	0	0	0	0	0	0
ZNF548	249.60046	214.248767	412.1057165	332.625289	273.7565587	399.0926341
MTF1	663.191986	745.548736	891.60862	956.420905	1200.059117	1431.576184
TMCO2	0.97671868	1.01573397	1.002934331	2.01140043	0	0
ZNF322P1	0	0	7.73262369	0	6.337843698	0
SHISA6	0	0	0	0	0	0
TMEM201	954.254154	1040.11159	413.2089443	545.089517	912.891318	927.8063372
NHLRC3	389.710755	446.922948	1320.864514	1368.75799	547.130227	1110.830634
HMX2	0	0	0	0	0	0
SNTN	0	0	0	0	0	0
ZDHHC11	67.3935892	77.1957819	289.7778162	252.641951	117.8597102	345.9974668
FAM26F	0.97671868	4.06293589	0	0	11.08366942	0.906060876
CNR2	0	3.04720192	0	0	0	0
LINC00910	21.487811	17.2674775	41.12030756	43.2451093	61.46398499	34.47561634
SLX4	696.400422	711.013781	886.5939483	1074.08783	798.0241985	1201.436722
GLRA4	0	0	0	0	0	0
DPPA3P2	0	0	0	0	0	0
ENTPD8	5.8603121	7.11013781	6.017605984	16.0912034	236.7874832	89.70002674
RPL14	9709.70694	9557.24409	11229.88579	10005.983	9255.06549	7941.460489
BEND4	0.97671868	5.07866986	0	0	1.007606311	0.906060876
RP11-159F24.2	0	0	0	0	0	0
RPSAP47	1.04508899	0	0	0	1.098290879	0

FAM78B	56.6496837	45.7080288	0	0	13.09888205	4.530304381
ZNF563	61.5332771	55.8653685	2.005868661	2.01140043	47.35749663	14.49697402
TMC3	0	0	5.014671654	0	0	4.530304381
RPL10AP2	4.88359342	1.01573397	3.008802992	2.01140043	3.022818934	0
POTEA	0	0	0	0	0	0
FBF1	443.537722	358.574407	342.7427782	248.639264	352.8133499	535.9984325
KLRG2	0	0	629.8427597	816.628575	180.3615297	615.2153349
ASTL	0	0	2.005868661	3.01710065	1.007606311	7.248487009
MSL1	2364.63593	2329.078	1753.12921	1756.95828	2428.33121	2191.761259
CTD-3088G3.8	0	2.03146794	34.09976724	103.587122	2.015212623	113.2576095
LRRK2	61.5332771	362.975506	127.37266	659.906334	84.03042525	90.60608762
BSX	0	1.01573397	0	0	0	0
GJB3	76.1840573	83.2901857	0	0	160.2094035	9.060608762
FAM196A	10.7439055	11.1730737	0	0	25.19015778	0
TRMT2B	708.121046	897.908831	624.828088	932.2841	1726.029611	574.4425955
HACD4	0	0	0	0	1.007606311	0
CFAP126	0	0	0	0	0	0.906060876
USP32P1	18.3916128	13.1131256	7.150921778	3.96245885	64.83946613	13.40970097
NYX	0	0	0	0	0	0
FAM120AOS	656.354956	802.429838	1144.348071	1200.80606	2567.380881	864.3820759
UTS2B	9.76718684	7.11013781	1.002934331	2.01140043	2.015212623	0
C9orf152	0	1.01573397	1731.064655	1597.05194	36.27382721	636.0547351
NOC2L	2969.21503	2929.37678	3085.026001	2515.24618	4384.760081	5342.977563
MSANTD1	7.81374947	12.1888077	13.0381463	2.01140043	15.11409467	19.0272784
AADACL3	0	0	0	0	0	0
DHFRP1	5.63566681	10.8277241	5.084877057	7.24104155	12.16180818	6.451153438
NELFB	2822.717	2669.34888	3397.941512	4055.98897	3044.986273	4257.580057
SLC15A5	0	0	2.005868661	0	0	0.906060876
LIPI	0	0	0	0	0	0
LRRC66	4.88359342	1.01573397	0	0	3.022818934	0
ZNF292	1697.53707	1515.47509	2205.452593	2052.63414	1446.922663	1694.333838
HUS1B	3.90687474	9.14160575	12.03521197	5.02850108	2.015212623	4.530304381
KCTD21	348.68857	354.491156	262.7687946	340.932373	615.6474562	340.6788894

SBSN	6.83703079	28.4405512	1.002934331	0	12.09127574	3.624243505
PROS2P	0	0	0	0	0	0
ADAT2	178.739519	203.146794	127.37266	52.2964112	173.3082855	227.4212799
KIR2DL4	0	0	0	0	0	0
FAM35DP	19.1436862	4.12387993	1.384049376	0	8.977772234	4.928971166
MAGEB16	0	0	0	0	0	0
VHLL	0	0	3.008802992	0	0	2.718182629
DUSP21	0	0	0	0	0	0
ZNF567	265.667482	298.625788	261.7658603	213.208446	204.5440812	324.3697937
NDUFA4	1165.22539	1422.02756	2204.449659	2559.50705	2203.594699	2143.740033
ANKDD1B	0.97671868	0	5.014671654	10.0570022	0	13.59091314
ALKBH2	355.525601	384.963175	385.126783	325.84687	500.7803367	572.6304737
RNFT1	204.134205	231.587346	344.4878839	561.18072	696.2559611	652.4634975
RNF222	0	0	1.002934331	6.03420129	0	4.530304381
CGB5	0	0	0	0	0	0
RELN	409.245129	384.963175	0	0	0	0
FAM111B	1658.46833	1379.36673	1122.283516	1365.74089	1010.62913	2629.388663
APOD	0	0	17.04988362	6.03420129	105.7986627	501.9577254
H1F0	8021.79055	7070.52418	19614.38671	27007.0736	7123.776621	22401.4491
GAGE2A	0	0	0	0	0	0
LITAF	2127.29329	1835.43129	6677.536774	8706.34677	2072.646182	8773.387464
VSTM1	70.3237452	97.5104613	0	0	0	2.718182629
TMEM120A	361.385913	405.277855	591.7312551	1022.79712	334.5252953	495.6152993
ARID2	1028.48477	853.216537	2056.015378	2100.90775	2878.731231	2720.900811
RIMKLBP1	0	0	0	0	0	0
FAM25G	0	0	0	0	0	0
SF3B3	6506.89987	6245.7482	2644.73783	4122.36518	15189.66514	8090.217563
PRSS48	0	0	0	0	0	0
IL1RAPL2	0	0	4.011737323	14.079803	2.015212623	18.12121752
BLOC1S3	233.435765	328.082073	731.1391271	1158.55659	757.6695658	801.8638754
SP6	236.365921	245.807621	976.8580381	1045.92822	207.5669001	3368.734338
ANKRD34B	0	0	0	0	34.25861458	5.436365257
PLAC9	0	0	0	0	0	0

FAM47B	0	0	0	0	0	0
NKAPL	0.97671868	0	0	1.00570022	0	0
UBE2Q2P1	41.0221847	32.5034871	6.017605984	11.0627024	21.15973254	20.83940015
FSCB	0	0	0	0	0	0
CLDN4	1549.07583	2224.4574	2843.318828	5023.47258	4998.73491	3402.25859
ZNF573	87.9046815	76.1800479	88.2582211	98.5586211	73.55526072	79.7333571
RP11-77G22.2	0	0	0	0	0	0
CRYM-AS1	9.76718684	9.14160575	6.017605984	5.02850108	16.12170098	7.248487009
GRAPL	0	0	0	0	0	0
FAM47E	2.93015605	6.09440383	59.4940645	98.3574811	50.30978312	32.31013084
HN1	3536.69835	3656.6423	8223.058577	10061.025	4802.25168	13975.08295
ZNF527	179.716238	170.643307	178.5223109	190.077341	97.73781219	190.272784
TNRC18P3	0	0	2.076074065	1.03587122	0	0.933242702
ZAR1L	0	0	0	0	2.015212623	0
KRTAP10-12	0	0	0	0	0	0
S100A13	670.029017	841.29182	847.028189	1761.75547	1974.283654	1007.530634
ZNF33A	1001.35153	893.734165	1510.268662	1463.89723	1570.374588	1256.697375
OR14I1	0	0	0	0	0	0
KRT77	0	0	0	0	0	0
PCDH18	10.7439055	25.3933493	0	0	2.015212623	0
DCAF8L2	0	0	0	0	2.015212623	0.906060876
ZNF600	314.083427	335.42583	222.7517149	359.52777	277.2932569	233.4556454
BTBD8	39.0687474	29.4562852	26.0762926	21.1197045	13.09888205	28.08788716
LINC00994	0	0	0	2.01140043	1.007606311	0
DPY19L2P1	5.8603121	2.03146794	0	0	150.5666111	6.750153528
MAOA	921.045719	971.041677	16.04694929	10.0570022	53.4031345	38.0545568
PAX8-AS1	1595.47974	1738.03256	1.002934331	8.04560172	727.8847232	140.2763448
C15orf61	153.344833	196.036657	559.6373565	453.570797	217.6429632	241.9182539
AC069277.2	0	0	0	0	0	0
NUGGC	0	1.01573397	0	0	1.007606311	1.812121752
LINC00943	0	0	0	0	0	0
TSPYL1	1754.18676	1861.84037	3573.45502	5299.03444	3690.861918	4018.379986
SPANXN3	0	0	0	0	0	0

TRIM64B	0	0	0	0	0	0
PNRC2	1633.94292	1757.14867	5214.255585	6367.98314	1847.415944	5416.359433
DRICH1	0.97671868	0	12.03521197	10.0570022	4.030425245	10.87273051
LINC01164	0	0	3.008802992	0	10.07606311	0.906060876
GJB5	0	0	0	0	6.045637868	0.906060876
FHIT	55.672965	65.0069742	114.3345137	96.5472207	22.16733885	51.64546994
FAM150B	0	0	0	0	0	0
ANKRD62P1-PARP4P3	0	0	0	1.00570022	4.11103375	0
ZKSCAN3	316.476388	328.275062	115.6784457	137.167452	77.58568597	146.2654072
FOXR2	0	0	0	0	0	0
RRP7A	2923.15298	2952.7285	2327.198792	1632.53305	1589.579565	2892.988953
LIN54	936.673218	947.679796	1279.744206	1607.10894	1146.655982	1352.748888
RP11-797H7.5	0	0	1.002934331	0	0	0
FAM53B	487.275184	656.783744	851.9626259	986.964021	1083.297697	1411.57036
FAM180A	0	0	0	0	0	0
C6orf222	0	0	0	0	0	1.812121752
SPANXN4	0	0	0	0	0	0
RP11-113D6.10	0.97671868	0	0	0	0	0
S100A14	2.93015605	0	8888.004039	13219.9293	1546.675688	5256.965204
KAZN	469.801687	417.466663	718.1009808	547.100917	459.4684779	460.2789251
SLC35E2B	2926.56173	2264.1726	2681.896547	2271.28342	1608.01876	2411.870628
RPS2P46	63.0178895	64.8342994	84.62759883	138.092697	154.2947544	106.9061228
FAM90A27P	0	0	0	0	0	0
FAM179A	65.4401518	33.5192211	0	1.00570022	10.07606311	7.248487009
SPATA31D4	0	0	0	0	0	0
NEMP2	498.126529	508.88272	34.09976724	59.3363127	209.5821127	118.6939748
ALG1L	0	0	1.002934331	0	208.5745064	18.12121752
KIAA0408	62.9495192	94.4023154	0	0	10.07606311	4.530304381
GSPT2	288.132012	342.302349	0	0	8.06085049	3.624243505
RP6-149D17.1	0	0	0	0	0	0
TBC1D28	0	0	1.002934331	0	0	0.453030438
C8orf76	686.965319	778.925754	644.8265986	584.86496	1085.091237	532.954068
CXCL17	0	1.01573397	0	0	0	1.812121752

FAM90A14P	0	0	0	0	0	0
OR7E12P	0	0	0	0	0	0
OTUD6A	0	0	0	0	0	0
HMGB1	10305.4174	9622.52531	9239.70302	12135.1409	18574.88984	16379.36985
MMP23B	11.2224977	16.0485968	23.06748961	24.1368052	1.007606311	4.086334552
SH2D5	548.9159	674.447358	1.002934331	16.0912034	71.5400481	25.36970453
SPATA41	2.93015605	4.06293589	13.0381463	5.02850108	7.053244179	4.530304381
ZFP92	0.97671868	2.03146794	143.4196093	181.026039	1.007606311	185.7424796
USP32P3	46.7652906	39.9996038	5.0949064	8.94067492	32.18294558	10.99051843
NCR1	0	0	0	0	0	0
RASSF10	13.6740616	17.2674775	0	0	19.14451991	0
GJB4	0	0	0	0	1.007606311	1.812121752
RNU1-75P	0	0	0	0	0	0
MIR494	0	0	0	0	0	0
RNU1-15P	0	0	0	0	0	0
Y_RNA	0	0	0	0	0	0
OR2L13	0	0	0	0	0	0
BLOC1S2	685.656516	665.305752	1005.943134	888.03329	1171.84614	898.8123892
SYCP2	25.3946858	24.3776153	9658.257605	4779.08742	289.1830113	4467.78618
ZNF724P	244.179671	282.374044	145.425478	208.179945	398.4982201	462.9971077
IL1RAP	526.451371	587.094236	213.6250124	256.453555	192.4528055	293.5637239
AC013442.1	0	0	0	0	0	0
PTPRT	0	0	0	0	0	6.342426133
MYBPC1	0	0	557.6314879	17.0969037	3.022818934	578.9728999
PAX5	6.83703079	1.01573397	4.011737323	8.04560172	71.5400481	7.248487009
AC079610.2	0	0	2.005868661	1.00570022	0	1.812121752
OR5K4	0	0	0	0	0	0
OR6M1	0	0	0	0	0	0
SPOCK3	0	0	0	0	0	0
ZNF676	0	0	0	0	0	0
ZNF699	345.758414	205.178262	58.17019118	96.5472207	52.39552819	57.0818352
RP3-391O22.3	0	1.01573397	1.002934331	0	0	0.906060876
ADAM5	0	0	0	0	0	0

TDRD7	591.891522	594.204374	374.0945054	621.522733	675.0962286	419.5061857
CCDC189	87.9046815	132.045416	74.21714047	152.866433	152.148553	217.4546103
OR8A1	0	0	0	0	1.007606311	0
KIAA0895L	261.760607	330.113541	1038.037032	975.529209	369.7915162	1067.339712
HLA-DRB1	410.690672	325.715413	0	0	1174.475993	108.4192444
VN1R2	0	0	0	0	0	0
MYT1	0	0	295.8656276	290.647362	12.09127574	212.9243059
SERPINA3	13.6740616	6.97809239	1.002934331	2.01140043	1832.765348	1223.689577
AKR1C3	41.8133269	115.539739	0	1.00570022	191.1429173	108.9991234
SPATS2L	4966.61451	4835.90944	145.425478	132.752428	3438.96034	562.6638041
OR11H13P	0	0	0	0	0	0.906060876
ZNF250	251.016702	207.20973	542.5874729	485.753204	206.5592938	482.930447
WDSUB1	284.225137	347.381019	410.2001413	485.753204	187.4147739	159.4667142
ZNF79	113.299367	136.108352	91.26702409	105.598523	191.4451991	135.9091314
S100A4	4380.5833	4274.20856	0	0	79.60089859	38.0545568
PLEKHG4	832.164319	659.211348	3881.35586	3451.56314	104.7910564	3427.628295
KRTAP4-3	0	0	0	0	0	0
FAT4	1404.52147	928.380851	18.05281795	40.2280086	168.270254	121.4121574
C8orf86	0	0	0	0	0	0
COLCA1	0.97671868	0	5.014671654	2.01140043	4.030425245	6.342426133
KIF19	0	0	0	0	0	0
OR6K2	0	0	0	0	0	0
ZNF681	136.740616	100.557663	198.5809975	202.145743	214.6201443	282.6909934
ACADSB	829.234163	764.847681	2932.579983	1605.09754	612.6246373	1633.62776
STK40	3661.71835	3440.29096	1346.940806	1041.90542	1395.534741	1536.679246
RPS2P4	0	0	27.40016591	20.4760564	29.15005059	27.0912202
OR10J1	0	0	0	0	0	0
TMEM63A	1864.55597	2033.49941	5996.544363	5306.07434	1152.70162	6388.635238
CTSE	7.81374947	10.1573397	0	0	16.12170098	4.530304381
SEMA4A	2.93015605	0	1965.751288	1710.69607	304.297106	2228.003695
HRCT1	1.95343737	5.07866986	5.014671654	1.00570022	4.030425245	3.624243505
MPHOSPH8	1762.00051	1801.91207	1776.18667	1688.57066	1390.49671	1339.946248
RNF216P1	456.723424	446.780745	339.8643566	324.328263	467.2068944	371.0591106

EEF1A1P5	1673.52933	1662.93934	1235.915976	1062.69325	1262.057133	858.6648317
GREB1	27.3481231	59.9283044	2.005868661	12.0684026	12611.20059	882.5032934
SIRPB2	0	0	0	0	0	0
ZNF766	258.830451	276.27964	631.8486283	499.833007	456.445659	662.3305005
RYR1	0	0	2.005868661	1.00570022	16.12170098	5.436365257
SRGAP3	6.83703079	9.14160575	55.05106541	76.1113923	28.51525861	129.4036143
KRTAP5-3	0	0	0	0	0	0
FAM217B	540.125432	805.47704	720.1068494	756.286562	1472.112821	593.4698739
SULT1C3	0	0	0	0	0	0
TUBB	82679.4222	74417.1908	11162.53875	20658.6815	38975.88475	33319.22896
LCOR	1215.03804	1208.72343	758.218354	806.571573	1086.199604	919.6517893
SUPT5H	4440.16314	4803.40595	3335.759584	3377.14132	4247.060602	4176.940639
XPNPEP3	516.684184	530.213134	1359.978952	1091.18473	893.7467981	1560.236829
OR2T2	0	0	0	0	0	0
OR2C3	0	0	0	0	0	0
LINC00615	2.93015605	1.01573397	0	0	0	0
ZNF107	305.712948	291.51565	943.7612052	970.691791	1069.070296	1832.961153
OR10S1	0	0	1.002934331	1.00570022	0	0
SFTA2	0	1.01573397	0	0	0	0
PPIA	23923.8451	23604.3371	18504.27881	29818.9209	27247.71002	26614.13384
ZNF471	0	0	0	0	0	0
OR10J3	0	0	0	0	0	0
ZNF836	104.704243	117.479791	251.8668985	377.067182	80.12485387	220.4808536
ZNF493	152.368115	175.721977	578.6931088	541.066716	171.2930729	648.1234659
LINC00523	0	0	0	0	0	0
Metazoa_SRP	0.97671868	0	2.005868661	0	0	0
GTF2IRD2	68.8879688	47.4347765	335.521651	487.362324	130.3540285	346.6498306
GRM7	0	0	0	0	0	0
SUPT3H	611.425896	528.181666	59.17312551	56.3192121	108.8214816	42.58486118
BECN2	0	0	0	0	0	0.906060876
NIF3L1	838.024631	947.679796	608.7811387	552.129418	811.1230806	683.1699006
AC005154.6	236.746842	228.540144	713.7181578	441.200685	400.0297816	438.1529185
ATP2A1	28.3248418	33.5192211	37.10857024	85.4845183	37.28143352	103.2909399

HLA-DRB9	0	0	0	0	1.007606311	0
RP11-497H16.5	0	26.9880516	0	2.17231247	10.22720406	0
IARS	9205.5736	8427.54477	8753.610838	2586.66095	8514.27333	4299.258857
HIATL2	147.777537	111.781524	94.23570971	61.5488532	198.1457811	69.56735407
POM121	3871.77147	3629.44094	1942.623623	2325.58118	3325.090751	3732.662749
ZBTB44	3450.74711	2888.74742	1539.504198	2100.90775	936.0662632	2214.412781
GIMAP5	0	0	0	0	0	0.906060876
STK31	1.95343737	1.01573397	9.026408976	8.04560172	19.14451991	7.248487009
CGB7	4.2291919	17.2674775	5.536197506	2.01140043	4.937270925	7.39345675
NLGN3	9.76718684	22.3461474	271.7952036	199.128643	11.08366942	115.0697313
OR8D1	0	0	0	0	1.007606311	0
ADH7	0	0	0	0	0	0
ZKSCAN7	70.3237452	49.7709646	0	0	0	2.727243237
ZNF729	0	0	0	0	0	0
CD55	3026.8512	1997.94872	1745.105735	741.201059	1228.272093	569.0062302
CPNE4	0	1.01573397	0	0	24.18255147	14.49697402
ZNF565	62.5099958	81.2587178	61.17899417	57.3249123	72.54765441	46.20910469
NTNG2	133.81046	143.21849	0	0	16.12170098	0.906060876
ELAVL3	0.97671868	2.03146794	4.011737323	1.00570022	3.022818934	0
WDR5	2490.63264	2382.9119	1082.166143	934.2955	3453.066829	2009.643023
PRSS29P	0	0	0	0	1.007606311	0.906060876
LONP1	6252.95301	5629.19767	6810.92704	2158.23266	2811.221608	3677.701096
C9orf163	8.79046815	12.1888077	54.15845386	83.4731179	47.35749663	59.80001783
TRRAP	5232.28199	5021.78876	2038.965494	2112.97615	9327.411624	3481.991947
NUDT11	0	0	0	0	0	0
SRGAP2B	1357.44363	1282.36414	843.5279482	1422.89484	837.8347239	762.4321061
FUT4	471.618384	500.299768	1.002934331	0	277.1924962	27.18182629
ASB13	573.333867	636.865201	313.9184455	333.892472	836.3132384	406.8213334
SLC35F1	0	0	0	0	3.022818934	0
ZNF34	72.2771826	53.8339005	207.6074065	269.527658	93.70738695	250.9788627
ZNF781	0.97671868	4.06293589	25.07335827	30.1710065	2.015212623	9.060608762
OR4Q2	0	0	0	0	0	0
ZNF140	339.898102	338.239413	611.7899417	493.798806	583.4040542	569.0062302

INCA1	3.90687474	6.09440383	2.005868661	10.0570022	11.08366942	8.154547886
CTD-2503O16.3	0	0	0	0	0	0
ZNF774	90.8348376	95.4789934	73.21420614	48.2736103	105.7986627	92.41820937
RP13-77O11.11	0	0	0	0	0	0
PTPN1	4991.03247	4069.03029	1267.708994	1644.31985	5409.838285	1964.33998
OR10D1P	0	0	0	0	0	0
EVL	468.824968	413.403727	585.7136491	513.91281	3800.691006	1413.454967
SPANXD	4.11198566	7.86178095	0	0	0	0
THEM5	0	0	0	0	0	0
NOXO1	2.93015605	4.06293589	9.026408976	68.3876147	8.06085049	21.74546103
EPHB4	1819.62691	1720.65335	1175.439036	1537.71563	4900.997098	2573.212888
PRTN3	2.93015605	1.01573397	0	0	1.007606311	0
ZNF765	347.643481	361.936486	479.0014363	601.700382	485.7266984	547.3604359
ZNF124	132.833741	135.092618	201.5898005	257.459255	161.2170098	289.0334195
XRCC6	12250.9629	11328.4708	12327.05583	13348.6288	10387.40339	18888.61484
S100A5	52.7428089	62.9755063	0	0	0	0
LINC00176	42.9756221	52.8181666	247.7247797	368.086279	34.25861458	192.0849057
PPP1R26	1300.98929	1232.08531	1824.337548	1402.9518	2153.254687	1652.655038
NBPF4	0	0	0	0	72.79955599	11.74254896
TSC22D2	1090.99477	957.837136	696.0364255	800.537372	2716.506615	1283.888262
CRYBA4	0	0	0	0	0	0
ASMT	0.97671868	0.50786699	2.005868661	0	0	0.453030438
NPIPB15	73.29297	88.9376666	171.9932084	193.16484	32.92857425	134.6497068
ZNF569	268.597638	349.412486	338.9918038	388.200283	88.66935539	313.4970632
ARMCX4	157.251708	149.312894	384.1238487	452.565097	152.148553	282.6909934
YRDC	659.285112	578.968364	338.9918038	311.767067	768.8036155	425.8486118
ZNF777	795.049009	730.312726	655.9190523	608.44863	895.7620107	718.5062748
PIK3R4	726.678701	708.982313	769.2506317	835.736879	1294.77411	1059.185164
ZNF775	102.106171	119.887081	107.1434745	117.666925	80.64880916	85.86738924
ZNF605	516.684184	529.1974	866.5352617	687.898947	383.8980046	760.1850751
TRAPPC2	407.291691	476.379233	465.3615294	414.348489	410.0957687	410.4455769
RFX8	0.97671868	0	0	0	1.007606311	0
MYL6B	730.585576	682.573229	1100.218961	868.924986	503.8031556	648.7395873

ZNF799	129.835215	116.017134	341.7197852	308.025862	175.9885183	304.5542423
FGF16	0	0	0	0	0	0
SIAH1	381.897005	414.419461	1803.275927	911.164395	606.5789994	898.8123892
RP13-644M16.4	0	0	0	0	0	0
GK2	0	0	0	0	0	0
C20orf96	123.066554	133.06115	233.6836991	321.824069	64.48680392	261.8515932
ESRRG	0	0	252.7394513	130.741028	3.022818934	222.8909755
IPO4	2574.18116	2550.20328	1312.469953	1311.58394	5400.759752	3268.360914
NCOR2	7301.94888	7958.27567	5924.333091	6836.75007	12495.32587	6862.505076
SULT1A1	32.0363728	28.0647297	2758.229879	1200.04173	372.9050197	1619.574756
ARL9	0	0	1.002934331	1.00570022	1.007606311	0
PRPF40A	6444.38988	6073.07342	4050.851762	3322.83351	4404.247187	5034.980289
GDAP2	598.728553	577.95263	826.4178885	650.688039	876.6174908	830.8578235
TCEAL3	961.169322	859.402357	952.0454428	758.30802	1067.306985	637.0242202
ANAPC7	1981.29338	1944.79536	1498.424007	1336.71638	2026.65903	2103.66496
TPK1	141.624209	177.753445	34.09976724	54.3078116	93.70738695	46.20910469
SLC6A9	221.715141	171.659041	2803.201454	1768.02098	543.0998018	1422.515576
AFAP1	6024.40084	5129.45656	182.5340482	121.689726	1089.222423	385.0758724
NACA	8321.64319	8364.56926	19275.3949	13399.9497	11980.43904	9731.999871
OR9K1P	0	0	0	0	0	0
MYO18A	5403.05148	3860.0024	3829.313597	3898.65723	1664.696615	3807.902044
OR2T3	0	0	0	0	0	0
SPTSSB	0	1.01573397	2263.622784	2526.31894	25119.62534	2780.700829
BORCS6	197.297174	272.216705	248.727714	883.004789	291.198224	231.9515843
MAN2A2	1845.99831	1749.0939	2043.980166	1640.29705	2008.159378	2021.421815
MME	0	0	5230.302535	5123.0369	42.31946507	924.1820937
FAM72A	322.004616	416.36967	198.8618191	488.096486	598.8103547	422.0340955
LINC00238	0	0	0	0	0	0
CACNA1H	0	1.01573397	0	0	5508.583704	855.3214671
SULF2	0	0	0	0	22394.05027	2271.494617
RP3-499B10.3	0	0	0	0	0	0
HBG2	0	0	0	0	0	0
RP11-57C13.3	0	0	0	0	0	0

LAMA2	12.6973429	32.5034871	1.002934331	4.02280086	0	1.812121752
PFN3	0	0	0	0	0	0
PLXNB2	8342.15428	6534.21664	16445.11422	15629.587	9779.826857	19963.23928
OR5AC2	0	0	0	0	0	0
AJAP1	0	0	0	0	12.09127574	2.718182629
XRCC2	1050.9493	930.412319	374.0945054	287.630262	1627.284193	1046.500312
MYO6	2912.57512	2958.83306	5388.766159	5012.40987	3713.029257	5553.24711
MKL1	813.606664	713.045249	982.8756441	1223.93716	821.1991437	1182.409443
CTB-25J19.1	0	0	0	0	0	0
HDAC2	3675.39241	3426.07069	2908.509559	2748.57869	3052.039517	3217.422171
ANKRD20A19P	0	0	2.005868661	0	8.06085049	1.812121752
ZNF782	113.299367	98.5261953	258.7570573	209.185645	369.7915162	156.7485316
SLC22A25	0	0	2.005868661	0	0	0.906060876
POTEF	0	0	2.39701305	1.05598523	45.87631535	4.865546905
ZNF846	43.9523408	56.8811024	135.3961346	122.695426	34.25861458	80.63941798
MMP1	594.821678	1827.30542	0	0	82.62371753	36.24243505
ADH1B	0	0	0	0	0	0
UGT2B15	0	0	4.011737323	6.5269944	54.21929561	23.91094652
TCF4	1.95343737	4.06293589	3.008802992	20.1140043	0	19.93333928
WNK3	365.292788	328.082073	0	0	11.08366942	3.624243505
LUADT1	0	0	1.002934331	0	0	0
SDHAF3	299.852636	348.396752	133.390266	106.604223	25.19015778	141.3454967
HRH1	1113.4593	1069.56787	0	0	74.56286704	27.18182629
RABL6	4384.76365	4101.51347	9580.269431	9387.84946	6583.115226	10381.85391
ZNF136	142.600928	137.124086	234.6866334	210.191345	205.5516875	249.1667409
ZKSCAN5	623.14652	628.739329	235.6895677	281.59606	625.7235193	299.0000891
ZNF502	163.11202	127.982481	0	0	3.022818934	3.624243505
TRAPPC4	1457.26428	1519.53802	1095.204289	887.02759	998.5378545	1201.436722
AC004057.1	177.049796	179.002798	223.0927125	224.703599	363.8063348	267.9222011
TTC30B	96.8318903	123.28979	169.616254	152.926775	240.4753223	107.8484261
SLC30A10	0	0	0	0	0	0
OR8B3	0	0	0	0	0	0
TECPR2	1064.62337	1145.74792	775.2682376	784.446168	1084.184391	1012.97606

TLR7	0.97671868	0	0	0	0	0.906060876
FAM180B	0	0	0	0	3.022818934	0.906060876
LINC00173	12.6973429	14.2202756	0	0	23.17494516	11.77879139
ZFP62	277.388106	293.547118	1006.946068	895.073192	966.2944525	880.6911716
ERI2	401.587654	376.055189	1066.851336	797.520271	987.8068473	998.4790855
TOMM7	1201.36398	1305.21815	1947.69847	1779.08368	1801.600085	1025.660912
HSH2D	3.90687474	1.01573397	2.005868661	6.03420129	450.4000211	225.6091582
TRPV1	200.461743	233.090632	267.0914416	277.945369	139.4527135	321.1260957
ZNF33B	400.239782	358.665823	1101.372335	816.025155	826.7208263	714.8910919
PDXDC2P	322.043684	324.852039	657.9449796	680.456766	554.9593281	537.2940996
ZNF512B	1190.62008	1071.59934	2610.638063	1923.90451	5212.347448	3056.143335
AMZ2	1932.76023	2123.70675	5956.015787	5024.19668	3098.238266	4677.349
ZNF431	527.525761	538.745299	939.4887049	997.383075	577.9529041	1656.388009
FAM150A	0.97671868	1.01573397	0	0	0	0
NF1	1653.58473	1174.18847	4519.222094	5309.09144	3833.942014	7980.584197
VKORC1L1	2719.18482	2816.11228	1129.304056	1353.67249	2424.129492	1486.845898
ZNF418	104.508899	82.2744518	204.5986035	146.832231	169.2778603	238.2940104
DAPK1	7.81374947	7.11013781	44.12911055	12.0684026	5.038031556	19.93333928
LCE1B	0	0	0	0	1.007606311	0
HLA-DQA1	0	0	0	0	1.007606311	0
COL27A1	825.327288	832.901857	14.04108063	0	191.4451991	126.8485227
LINC01560	30.2782792	29.4562852	29.08509559	33.1881071	48.36510294	27.18182629
GM2A	1559.81974	1943.09909	2603.617523	2759.64139	704.3168116	2130.14912
HIST1H2AI	13.1857022	0	105.6190144	129.001167	24.60574612	199.6414535
CLPSL2	4.88359342	12.1888077	0	0	3.022818934	0
S100A2	636.820582	612.487585	10.02934331	4.02280086	22.16733885	13.59091314
SNHG17	881.976972	702.319098	322.3029765	284.995327	486.6738484	794.8781461
ZNF700	700.307296	625.153788	246.6917573	271.388203	369.1869525	363.8196842
AC079612.1	0	0	0	0	0	0
POU3F4	0	0	0	0	0	0
OR14A16	0	0	0	0	0	0
CD47	2265.98735	2064.98717	539.5786699	528.998313	3018.788509	933.2427025
OR52K1	0	0	0	0	0	0

TLE1	1958.32096	1793.75572	2120.203175	2241.67561	1882.178361	2305.834324
MAML3	204.134205	211.272666	552.6168162	596.380228	197.5109891	706.7274834
HIST1H2AG	55.672965	31.4877531	308.9037739	530.004014	187.4147739	443.9698293
STRN3	1331.26757	1362.09926	1685.93261	1588.00064	1182.929809	1880.076318
ZNF239	292.038886	219.398538	398.1649293	260.476356	305.3047123	545.4486475
CTB-134H23.2	0	0	1.133315794	0	0	1.603727751
SPINK14	0	0	0	0	0	0
SPRR2B	0	0	0	0	0	0
CTBP1-AS2	535.241839	448.954416	455.3321861	403.285786	660.9897402	460.2789251
CHRNG	0	0	0	0	1.007606311	0
ZSCAN16	110.369211	158.4545	231.6778304	138.78663	127.9660015	207.4879406
MVB12B	399.477942	466.221893	186.5457855	212.202745	518.9172503	219.266732
C6orf106	4846.47811	5271.65932	3125.143374	3787.46701	4501.984999	3904.216315
CTD-2192J16.17	8.47791818	8.08524242	0	0	2.901906177	46.80710486
OR11G2	0	0	0	0	0	0
POTEI	0	0	17.47111604	22.4472288	202.9319111	25.75931071
ADA	125.99671	133.06115	0	0	221.6733885	26.27576541
ARID5A	313.526698	291.51565	201.5898005	130.741028	195.4756244	256.415228
PATE2	0	0	0	1.00570022	8.06085049	0
PPTC7	2077.48064	1907.5484	494.446625	501.844408	897.7772234	561.7577432
KRT39	1.95343737	1.01573397	0	0	0	0
TOMM20L	0	0	0	2.01140043	1.007606311	0.906060876
RGPD4	6.32913707	4.74347765	0	0	8.796403098	0
NHLRC2	2345.10156	2152.34029	1093.19842	1345.62689	1601.086429	1550.270159
HIST1H2AD	0	0	11.1626591	24.9715364	15.77911483	34.40313147
ZFP28	449.290595	532.244601	282.8274813	955.415205	7.053244179	248.2606801
KIAA1211L	7.81374947	4.06293589	130.381463	106.604223	693.2331422	173.0576274
CBWD3	234.021797	214.888679	368.5583078	563.141836	814.0149107	386.7702062
SCN8A	46.8824968	15.2360096	7.020540315	6.03420129	242.833121	17.21515665
LAMB3	8387.08334	8195.95742	31.09096425	33.1881071	2205.650215	682.2638398
HIST3H2BB	0	0	300.8802992	444.519495	56.42595343	361.4729866
AC090286.4	0	0	0	0	0	0
TEX43	0	0	0	0	0	0

KPNA5	182.646394	166.580371	379.109177	412.337088	126.9583952	436.7213423
ANKRD36B	396.274304	290.784322	582.1331736	483.108213	181.7721786	406.785091
ARHGEF12	14246.4187	12388.9073	8029.492252	9504.87274	4351.851658	10736.82138
HCAR1	84.9745255	100.557663	903.643832	1387.8663	413.1185876	858.9457106
ZNF252P	804.816196	905.018969	1147.356874	967.483607	971.3324841	1289.324627
PDLIM7	3396.05086	3269.64766	737.1567331	790.480369	2714.491403	1607.351994
FLNA	78846.5925	76603.609	32231.30059	42344.0019	126729.6686	109485.6781
TMEM26	0	0	0	0	135.0192457	8.154547886
RPS26P11	0	0	2.51736517	0	0	0
SRGAP1	1705.35082	1351.94192	145.425478	158.900634	1927.550873	173.0576274
OR2L8	0	0	0	0	0	0
FAM3C	3202.83637	3289.37321	2929.310417	1711.54085	1365.860735	1473.4815
NOP9	1424.81768	1299.50973	1796.987528	1562.63688	3352.245741	2493.506713
OR2T4	0	0	0	0	0	0
ZNF705A	0	0	0	0	0	0
SLC39A10	2280.63813	1668.85092	437.2793682	466.6449	589.4496921	629.7123089
SCOC-AS1	0.97671868	6.09440383	23.06748961	6.03420129	3.022818934	4.530304381
CASP4	779.42151	840.011995	833.4384288	465.6392	51.38792188	427.6607336
AP2A1	4954.89388	4864.34999	2138.255993	3459.60874	3946.793921	3379.607068
ZNF585A	292.732357	319.08267	320.7283696	346.051387	209.9045468	381.4788107
FUT11	1243.36288	1245.28985	375.0974397	435.468193	339.5633269	453.0304381
NXF4	0	0	0	0	0	0
SMIM10L2B	11.7206242	8.12587178	0	0	1.007606311	3.624243505
ANXA4	1006.02024	1223.95944	1148.359809	1001.67741	1225.249275	938.6790677
LAGE3	368.222944	480.442169	2767.095818	3318.81071	871.5794593	3559.913183
RP11-88G17.6	0	0	0	0	0	0
WDR5B	117.206242	153.37583	320.9389858	240.362352	429.2402886	257.3212888
FAM163B	0	0	0	0	0	0
NPIPB9	6.07519021	13.0623389	57.3879024	52.2260122	39.71984079	42.63922483
WDR45	1022.62446	964.947274	1087.080521	1088.16763	592.472511	716.6941531
METTL9	1882.1369	1773.47152	1787.228977	2553.47285	3735.196596	2167.297616
ZNF138	109.392493	132.045416	487.4260847	413.342789	485.666242	695.8547529
ZNF429	35.1618726	52.8181666	356.0416874	504.861508	306.3123186	294.4697848

ZNF470	501.056685	553.575015	253.7423857	242.373752	10.07606311	242.8243148
SERTAD1	457.104344	638.896669	305.8949709	481.730403	338.5557206	393.2304203
ZNF100	411.198566	434.73414	380.1121113	486.869531	425.8245032	831.9360359
CXorf40B	394.467375	420.910001	1389.194429	1158.43591	884.6481131	1119.302303
OR51A6P	0	0	0	0	0	0
ZNF398	574.310586	515.992858	987.8903157	1099.23034	1067.055084	1234.054913
ZSCAN25	406.314972	384.963175	211.6191438	203.151444	623.7083067	298.0940283
RBMY1A3P	0	0	0	0	0	0
ANXA6	8486.70864	8139.07632	1893.540016	5737.51973	1104.336517	2293.240078
ZNF441	123.066554	101.573397	0	0	12.09127574	4.530304381
GMFB	6083.98068	5635.29208	3631.625211	3810.59812	2733.635923	3688.573827
SIGLEC15	0	3.04720192	1045.057573	1774.05518	67.50962286	717.6002139
ZNF420	231.482328	217.36707	308.9037739	305.732866	193.4604118	279.0667499
ZNF763	38.0920287	29.4562852	10.35028229	10.0570022	6.56959315	18.12121752
ZMYM1	925.929312	770.942085	408.1942726	386.188883	442.3391707	540.0122822
DTHD1	0	0	0	0	0	0
HIST1H4C	22.4645297	35.550689	9.026408976	17.0969037	11.08366942	9.966669638
ZSCAN26	471.755124	474.347765	279.1065949	266.158562	143.0800962	212.1360329
MAFG	2720.16153	3392.55147	1282.753009	1169.62935	1392.511922	971.2972593
OR2T32P	0	0	0	0	0	0
ARRDC1	792.118853	802.429838	7526.019218	9975.54044	2536.145086	5057.631811
KIAA1671	1929.99612	1846.60436	5352.660523	5125.0483	2406.163871	5047.665141
KRT35	0	0	0	0	0	0
IGF2R	8296.2485	9500.15984	5146.056051	9376.14311	4743.810514	6953.111164
ZNF300P1	0	1.01573397	0	0	0	0
LCE1C	0	0	0	0	0	0
NPSR1-AS1	0	0	0	0	0	0
GOLGA6L16P	0	0	0	0	0	0
GAL3ST4	28.3248418	34.5349551	31.09096425	22.1254047	6.045637868	23.55758278
RP11-573D15.8	0	0	0	0	0	0
DYNC1H1	55018.5635	50810.0605	15010.91813	21562.2126	24345.78369	29761.3816
SLC6A17	0	0	4.011737323	0	3.022818934	0.906060876
IFNL3	0	0	0	0	0	4.530304381

PCBP2	9668.12803	8932.41534	17465.46952	17533.2267	19629.09794	16510.41344
ZGPAT	969.19795	953.012399	822.5265033	798.717054	1063.32694	903.6869967
SLC25A29	1298.05913	1192.44121	11211.75274	8823.95335	2941.182671	6594.95436
PGAP1	642.612524	583.0313	238.6983707	188.06594	304.297106	258.2273497
SRC	1576.42396	1550.01004	5.014671654	4.02280086	1258.500283	247.3546192
ZNF679	0	0	0	0	0	0
ZNF682	70.3237452	73.132846	406.1884039	208.179945	142.0724899	402.291029
OR8B8	0	0	0	0	0	0
ZNF772	360.409194	344.333817	514.7660746	379.148981	427.225076	415.0664874
ZNF257	0	0	56.16432252	98.5586211	92.8206934	107.8212443
PCNXL3	2609.79232	2977.11627	2162.326417	2365.40691	5490.44679	3562.631365
ADAM32	5.8603121	4.06293589	59.17312551	26.1482056	69.52483548	28.99394804
ACSL5	2838.3445	3065.48513	0	0	91.69217433	50.73940907
LRRC8B	115.252805	157.438766	1124.289385	1123.36714	667.0353781	1464.194376
RP11-452G18.2	2.93015605	2.03146794	1.113257107	4.12337088	1.007606311	0.942303311
ABCB8	907.371657	992.372091	891.60862	1046.93392	853.4425457	961.3305896
HIST1H3J	0.97671868	5.07866986	14.04108063	29.7988974	15.83957121	27.04591715
SND1	5396.16562	5521.52987	10707.38709	6917.20608	6812.909922	6021.608098
OR4C4P	0	0	0	0	0	0
ZNF785	100.602024	88.3688556	1156.383283	836.742579	500.7803367	978.5457463
SULT1A2	0	0	98.87929566	31.5287018	14.368466	53.80189483
NEK5	0.97671868	1.01573397	43.12617622	21.1197045	5.038031556	35.33637417
PSMD12	4067.0566	3852.67896	6542.140639	5506.20868	7986.287623	7668.899256
OR2B4P	0	0	0	0	0	0.906060876
MAGEA6	0	0	2.005868661	0	0	0
RP11-76E12.1	0	0	0	0	0	0
ADGRA1	0	0	0	0	0	0
CH17-340M24.3	13.6740616	20.3146794	381.1150457	597.385928	83.63132384	272.7243237
PIWIL2	2.93015605	11.1730737	2.005868661	2.01140043	2.015212623	1.812121752
MIRLET7BHG	78.1374947	83.2901857	714.0892435	656.722241	50.38031556	815.4547886
NOL4L	299.852636	250.886291	129.3785287	141.80373	1076.12354	421.3183074
SSXP1	0	0	0	0	0	0
CYSRT1	37.11531	37.582157	127.37266	93.53012	159.2017972	140.4394358

SLC22A4	130.880304	214.39097	8.023474646	74.451987	34.25861458	121.4121574
KB-1592A4.15	0	0	0	4.27422592	0	1.920849057
ZSCAN5B	0	0	0	1.00570022	0	0
TCEA1P1	0	0	0	0	0	0
ENTPD4	1545.16896	1401.71288	657.9249209	662.756442	1221.218849	626.9941263
C1D	407.26239	441.844278	296.8384739	372.048738	353.6597392	326.1637942
TBC1D9B	5246.93277	5268.61211	5135.023773	5037.55238	6849.707704	6722.06564
OR1J2	0	0	0	0	0	0
HIST1H4J	16.6042176	17.2674775	142.416675	140.79803	46.34989032	163.9970186
SLC2A7	0	0	0	0	0	0
FAM110D	0.97671868	0	2.005868661	4.02280086	2.015212623	9.060608762
SERPINA1	800.909321	1208.72343	2.005868661	3.01710065	173.3082855	179.4000535
LINC00336	0	2.03146794	0	0	0	0
TPSB2	0	0	0	0	0	1.812121752
AP000445.1	0	0	0	0	0	0
KANK2	4282.91143	4113.72259	806.3592019	2086.82795	2839.434585	1515.839846
EIF4BP6	32.6517056	23.5142415	45.44295452	63.0372895	96.13571816	53.24013708
C6orf141	130.880304	170.643307	0	0	733.5373946	110.5394269
OR8D2	0	0	0	0	0	0
GTF2E2	945.463686	952.758466	346.0123441	298.692964	734.5450009	366.9546549
IL27	0	0	1.002934331	1.00570022	4.030425245	2.718182629
GUCA2A	0	0	1.002934331	0	0	0
RAD54B	624.123239	556.622217	342.0006068	363.057778	1322.866174	676.954323
ZNF165	20.5110924	44.6922948	197.5780631	482.736103	109.8290879	215.6424885
SYNGAP1	511.478273	676.641343	1020.987149	1383.05905	411.6676345	1367.028408
RAMP2-AS1	0.97671868	3.04720192	0	0	5.038031556	0
FITM2	779.42151	896.893097	640.8750373	552.129418	898.7848297	476.5880209
BLM	1132.01695	1028.93851	143.4196093	271.539058	950.1727515	499.2395428
RP11-366L20.2	0.97671868	2.03146794	0	0	0	0
ZNF720	157.251708	173.690509	652.9102493	582.803275	891.7315855	600.7183609
GATA3-AS1	0	0	932.8492797	1341.20181	670.380631	797.1886013
OR10D3	0	0	0	0	1.007606311	0
DDI2	1271.23844	1302.9937	2757.918969	2344.24697	2159.239869	3834.159689

AC060834.2	0	0	2.216484871	9.7754061	0	6.550820135
SVIL	4277.79342	4369.53519	2164.231992	2735.18276	2771.834278	10870.22979
C17orf102	0	0	0	0	0	0
TRIM33	2879.36668	2923.28237	1622.747747	1811.26609	12302.87306	2876.743282
LRP10	7635.98667	8103.52563	4611.492053	6555.154	5491.454397	7698.799265
PELI1	394.594348	314.877531	6.017605984	2.01140043	150.1333404	30.80606979
CTC-499B15.5	26.088156	27.9631563	62.43266209	53.9155886	20.82722245	61.67556384
Metazoa_SRP	0	0	0	0	0	0
ZNF655	1885.36984	1454.53105	801.5551464	797.520271	152.148553	932.7081265
MRPL21	787.235259	851.185069	1255.673782	896.078892	1137.587525	1236.773096
LYPD2	0	0	0	0	0	0
UAP1L1	1090.99477	1899.42253	2434.121621	4891.72585	959.2412083	1197.812478
BNIP3P1	21.5561814	11.5692099	13.97087523	9.49381003	5.753432037	4.865546905
ZNF98	0	0	0	0	35.44759003	1.812121752
FBXL22	0.97671868	1.01573397	53.15551953	35.1995075	50.38031556	51.64546994
ZNF786	220.738423	245.807621	353.0328844	359.034977	587.4344795	381.4516289
ZNF517	67.3935892	67.0384422	251.736517	175.997538	150.1333404	139.5333749
S100A7L2	0	0	0	0	0	0
ZNF675	275.434669	271.200971	475.3908728	602.414429	344.6013585	668.6729266
SLC22A5	618.262927	623.589558	1018.98128	1018.74415	1233.310125	1306.539783
OR8S1	0	0	0	0	0	0
DACT3	9.76718684	23.3618814	13.0381463	27.1539058	19.14451991	16.30909577
ADARB1	1013.83399	938.53819	254.74532	282.601761	1631.314618	500.1456037
ZNF860	122.089835	102.589131	225.6602244	234.32815	186.4071676	231.0455234
HTT	2329.47406	2639.89259	2559.488412	2832.05181	5160.959526	4263.922483
OR6N1	0	0	0	0	0	0
C5AR1	0	2.03146794	2.005868661	3.01710065	25.19015778	8.154547886
DIO3	0	0	0	3.01710065	30.22818934	6.342426133
CYP2B6	0	0	1.002934331	0	0	0.906060876
HIST1H3D	0	0	16.6286512	17.6198678	2.025288686	2.518849236
DCHS2	0	0	0	0	0	1.812121752
VEPH1	132.833741	149.312894	234.6866334	302.715765	43.32707139	93.32427025
FABP12	0	0	0	0	0	0

SHPK	437.335558	375.902828	372.7806614	510.453201	1007.324182	593.5695406
GGT3P	0	0	4.162177472	0	2.055516875	2.835970542
OR51D1	0	0	0	0	0	0
IPP	234.412484	200.099593	262.7687946	275.561859	443.346777	270.912202
OPALIN	0	0	0	0	0	0.906060876
OR13G1	0	0	0	0	0	0
MAP3K5	677.842767	630.770797	342.0006068	440.496694	204.5440812	686.7941441
OGDHL	5.8603121	7.11013781	1.002934331	0	67.50962286	8.154547886
C16orf47	1.95343737	0	0	0	1.007606311	0
CYP2F1	0	0	0	2.01140043	0	0
GSTK1	1263.87398	1253.41572	1414.137406	1172.64645	974.355303	1037.439703
HNRNPAB	8908.65112	8369.64793	4593.439235	4570.90748	13655.08073	8327.605513
OR2L5	0	0	0	0	0	0
STMN3	1043.13555	589.125704	17.04988362	31.1767067	674.0886223	111.4454878
PDGFA	335.991227	271.200971	439.2852369	400.268686	601.5409678	372.3910201
AC005276.1	1.95343737	0	0	0	0	0
GYPE	0	0	0	0	0	0
COL13A1	4020.1741	3332.62316	1.002934331	4.02280086	63.47919761	25.36970453
RP11-747H12.1	0	0	0	0	0	0
SPN	0.97671868	0	1.002934331	0	0	0
ZNF695	90.698097	108.439759	316.9372778	312.098948	184.9058342	477.3309908
ADAM3A	0	0	0	0	0	0
RP11-170L3.7	0	0	0	0	0	0
PCDHB11	78.5281822	72.624979	5.014671654	10.0570022	23.36639036	2.718182629
ZNF628	390.687474	398.167717	179.5252452	348.977975	313.3655628	332.5243416
GALP	0	0	0	0	0	0
SLC2A10	58.603121	48.7552307	7547.080839	4049.95477	887.7011602	2125.618816
ZNF665	44.9290595	39.6136249	0	0	14.10648836	0.906060876
RPF2	1037.27524	1174.18847	668.9571986	484.747504	1027.758438	695.8547529
LINC00477	0	0	0	0	0	0
SLC28A3	36.1385913	34.5349551	0	0	6.045637868	3.624243505
FAM177B	0	3.04720192	8.023474646	0	2.015212623	3.624243505
MIB2	883.930409	860.326674	1356.970149	1055.98523	401.0273119	875.2548064

OR6Y1	0	0	0	0	0	0
MYO5A	2252.31328	2273.21263	165.4841646	116.661225	3305.956307	427.6607336
C5orf56	143.577647	78.2115159	36.10563591	20.1140043	42.31946507	65.23638308
GZMM	2.93015605	4.06293589	3.008802992	0	2.015212623	0.906060876
ATG7	746.213074	693.746303	486.4231504	366.074878	870.571853	419.5061857
PRAMENP	0	0	0	0	0	0
RP11-460N11.2	0	0	2.005868661	0	1.007606311	4.530304381
SIPA1L1	2832.48418	2503.78424	1712.008903	1969.16102	2371.905257	2539.688636
TTC30A	170.789029	168.22586	210.4958573	111.572382	289.5255975	120.4789147
SSPO	0.97671868	6.09440383	369.0798337	677.841945	182.3767423	283.5970542
ELANE	0	0	0	0	0	0
RAB40C	365.292788	378.868772	1233.609227	1503.52182	1101.313698	1342.782218
PIGN	519.61434	538.339005	841.4619035	1063.02513	347.6241774	1483.221654
COL4A6	0	0	66.19366583	58.3306125	32.24340196	234.6697669
ZNF624	141.624209	141.187022	87.25528677	108.615623	106.806269	97.85457463
HHLA3	5.8603121	7.11013781	196.5751288	199.128643	66.50201655	107.8212443
RPS17P2	0	0	0	0	0	0
HOXA4	0	0	0	0	74.56286704	7.248487009
TOPORS	1108.57571	1223.95944	1900.560557	1841.43709	756.7123398	1604.633812
BCO2	8.79046815	2.03146794	26.0762926	31.2873337	5.038031556	20.35918789
GPX1P1	6.04588865	5.15992858	14.13134472	19.4200712	2.015212623	17.00676265
KCNMB2	0	0	0	0	0	0
AC107218.3	0	0	0	0	0	0
ENTPD6	2327.52062	2159.45042	2839.30709	2217.56898	1803.615297	2236.158242
DMBX1	5.8603121	4.06293589	10.02934331	43.2451093	16.12170098	31.71213067
KLKP1	0	0	0	0	0	0
OR11L1	0	0	0	0	0	0
ENPP1	1222.85179	1118.3231	3887.373466	1876.6366	844.3740889	1562.048951
ATP11AUN	0	0	0	0	0	0
CCDC154	11.7206242	3.04720192	15.04401496	7.03990151	5.038031556	9.966669638
FAR1	2903.78465	2862.33833	1409.122735	1301.37608	2581.48737	1290.230688
C5orf42	1929.0194	1417.96463	638.8691687	765.337864	883.670735	908.7790588
ZNF841	497.77491	596.997642	1040.303664	718.804115	452.8888087	1159.250527

MFAP5	0	0	0	0	0	0
MYH6	0	0	0	0	1.007606311	0
VN1R5	0	0	0	0	0	0
ZNF615	235.389203	201.115327	750.1948794	481.730403	102.7758438	837.2002496
CXorf40A	206.214616	193.609052	1299.672511	1268.31871	573.3582193	855.0043458
CDC42SE1	1652.60801	1911.61134	7490.916516	10066.0535	5317.138505	8561.369219
UBE2Q2P12	0	0	0	0	0	0
MPEG1	0	0	0	0	0	0
SERPINB2	2.93015605	7.61800479	0	0	39.29664614	19.93333928
DPP4	1.95343737	5.07866986	0	0	30.22818934	4.530304381
SERPINB13	0	0	0	0	0	0
AC106870.2	0	0	0	0	0	0
PDCD1LG2	185.57655	180.800647	0	0	4.030425245	0.906060876
ZNF433	68.3703079	77.1957819	0	0	185.3995613	14.49697402
CCER1	0	0	0	0	0	0
DNAH10	0.97671868	8.12587178	26.0762926	9.05130194	7.053244179	19.93333928
SLC22A24	0	0	0	0	0	0
AC007952.6	4.44407001	1.45249958	0	0	6.972635674	0
RP4-724E16.2	0	0	0	0	0	0.906060876
OR51C1P	0	0	0	0	0	0
KRTAP26-1	0	0	0	0	0	0
CBX3P7	0	0	0	0	0	0
SPTAN1	14422.2281	12004.9598	4310.611753	7050.96421	8364.13999	10935.24871
NMB	282.2717	179.784913	4.011737323	2.01140043	356.6926342	37.14849592
PARVA	4457.74407	3961.36249	257.754123	555.146519	1034.811682	443.9698293
KLHL14	0	0	0	0	1.007606311	0
OR6C74	0	0	0	0	0	0
FAM114A1	1510.00709	1573.37192	6.017605984	4.02280086	470.5521474	104.1970008
RPE	1350.80194	1240.21118	1603.691995	1302.38178	2665.108617	1770.442952
ZNF460	156.274989	123.919545	206.6044721	153.872133	213.612538	201.1455145
CR1L	0	0	0	0	0	0
PHF2	1535.40177	1464.68839	1355.967215	1476.36792	2714.491403	1125.327608
RPS26	3226.34599	3641.06094	4039.468457	4743.29455	6347.476414	4914.655405

C14orf178	1.95343737	0	0	1.61917735	1.007606311	0.906060876
PTMAP2	7.5695698	8.13602912	8.865939483	14.039575	7.204385126	15.46645916
SCGB1D4	0	0	0	0	0	0
PSAP	13183.7488	13608.8038	11123.54466	14316.1426	10017.62195	14995.3075
S100A10	5828.08039	4504.78017	72.21127181	261.482056	7774.690298	728.4729444
CFAP43	2.93015605	2.03146794	2.005868661	0	1.007606311	4.530304381
LHFPL5	2.93015605	1.01573397	3.008802992	2.01140043	1.007606311	0
RPL37A	9118.64563	8758.67404	13331.00312	11380.5036	14325.13893	9446.590695
HOXC6	9.76718684	17.2674775	481.5589189	337.744303	118.7363277	476.2980814
TXNRD3	350.202484	225.492942	223.6543557	215.219846	309.3351376	340.6788894
CFD	64.4634331	53.8339005	74.21714047	63.3591136	97.73781219	84.26366148
C9orf173	0	0	1.002934331	0	0	0.906060876
MAP1LC3C	2.93015605	1.01573397	0	0	2.015212623	0
MCMBP	4978.33513	4273.19282	3519.296566	3062.35716	2654.035024	5343.040987
EME2	192.413581	240.728951	1608.706666	1898.76201	770.8188281	1955.279371
KLHDC1	32.2317166	25.3933493	44.12911055	22.1254047	5.038031556	26.27576541
ZNF81	251.99342	250.886291	490.4348877	481.730403	293.2134366	446.688012
TAF13	846.815099	841.027729	409.1972069	445.525195	930.0206253	537.2940996
ZNF780A	480.106069	452.082876	575.192868	451.95162	355.5842673	575.7110807
ATAD3A	1879.00164	1725.75233	1640.228892	1425.58006	1843.325062	2872.729432
OR5B17	0	0	0	0	0	0
OR52M1	0	0	0	0	0	0
IGKV7-3	0	0	0	0	0	0
FAM118B	548.9159	792.272498	235.6895677	219.242647	205.5516875	310.7788805
ZNF461	115.252805	101.573397	191.5604572	179.014638	105.7986627	224.7030973
CTC-301O7.4	1.95343737	2.03146794	10.02934331	18.1026039	6.045637868	16.30909577
RP1-253P7.4	0.97671868	0	4.011737323	0	1.007606311	1.812121752
CCDC180	94.1068452	90.0752887	266.9911482	106.775192	64.18452203	135.039313
SLC9A8	667.098861	719.139652	1052.078113	954.409505	776.864466	735.7214315
OCLN	544.032307	513.96139	875.1203796	1058.73079	1563.794919	1353.165676
C4orf22	2.93015605	6.09440383	0	0	0	0
HIST4H4	12.6973429	6.09440383	55.16138819	67.3819144	87.66174908	168.527323
CYP2A13	0	0	0	0	0	0

ZNF181	159.546997	167.545319	291.7535968	332.715802	210.5897191	266.3275339
SLC22A20	13.9475428	23.4634548	2.948626932	12.4002837	60.62767175	8.181729712
OR8G1	0	0	0	0	0	0
FAM212B	81.0676508	100.557663	628.0073899	845.793881	168.270254	519.1728821
ZNF44	133.81046	109.699269	326.9565918	290.647362	151.1409467	321.651611
GPAA1	2428.12265	2360.56575	4253.444497	4642.31219	2711.468584	4857.392357
ADAMTSL2	1.95343737	5.07866986	0	0	0	0
SGTB	746.213074	703.903643	79.23181213	71.4047153	159.2017972	75.20305272
ZNF790	176.786082	188.926519	158.4636243	193.094441	79.60089859	166.7152012
OR5M12P	0	0	0	0	0	0
PRB3	0	0	0	0	3.022818934	0
FAM49A	183.623113	253.933493	2.005868661	1.00570022	37.28143352	5.436365257
MYO1C	12123.0323	10460.0284	4821.105328	4734.83661	7658.815572	6153.965471
MDS2	0	0	0	0	0	0
OR7E13P	0	0	1.002934331	2.01140043	0	7.248487009
NKIRAS1	520.591058	476.379233	280.8216126	284.613161	170.2854666	233.7637061
OR1S2	0	0	0	0	0	0
UGT2B17	0	0	7.020540315	2.52430754	6.237083067	19.57997553
MEIG1	0.97671868	5.07866986	5.014671654	10.0570022	1.007606311	1.812121752
SLC22A12	0	0	0	0	0	0
KIF13B	767.700886	769.926351	524.534655	579.283324	569.2975659	629.7123089
NRAP	0	0	0	0	0	0
ADH5	2060.87642	2164.51894	2333.828188	2218.55456	1919.479947	1689.776352
SLC22A6	0	0	0	0	0	0
HIST1H2BK	607.450651	442.138841	628.4286223	534.227955	461.9874937	1110.223573
TEAD4	685.656516	585.062768	640.8750373	913.175796	550.153046	1069.151834
SPG7	1441.63678	1423.0433	1381.040573	1244.05117	2671.164331	2140.11579
HRNR	11.7206242	5.07866986	0	0	0	1.812121752
IFNA1	0	0	0	0	0	0
HES5	0.97671868	0	0	0	0	0
C2orf27A	58.603121	45.7080288	45.97450972	67.3819144	62.4715913	66.22398944
ZNF677	0	0	0	0	0	0
ERO1A	8746.51581	9249.27355	1863.451986	2307.07629	3072.191643	2362.100704

ZNF823	169.538829	164.548904	198.5809975	254.442155	210.5897191	289.0334195
CYYR1-AS1	0	0	1.002934331	0	0	4.530304381
ZNF311	90.8348376	112.746471	218.6396841	204.157144	34.25861458	246.4485583
ZNF347	966.951497	747.580204	20.05868661	13.0741028	27.2053704	48.92728731
OR5H2	0	0	0	0	0	0
PLCG2	228.552172	277.295374	8.063592019	4.02280086	232.7570579	38.0545568
FCHSD1	1022.62446	975.104613	175.5135079	240.362352	338.5557206	279.9728107
ZNF71	393.207408	350.42822	437.2793682	371.10338	11.08366942	306.2485761
AADACL2	0	0	0	0	0	0
S100A6	26104.7603	24899.7026	148.4342809	254.442155	964.2792399	361.5182896
RPL12	13704.3008	13424.834	16234.37766	14289.8737	23370.13865	13983.17408
DNM3	33.2084353	60.9440383	7.020540315	1.00570022	26.19776409	21.74546103
ZNF121	1535.40177	1369.20939	1289.773549	825.679877	1213.157999	2057.66425
MPZL1	3316.93665	3651.56363	3068.979052	4532.69087	1826.790242	3792.770828
VPS13A	2852.99528	2303.68465	1727.052917	1415.0202	2564.358062	1398.957993
MBP	2715.27794	2772.95374	410.2001413	324.84117	410.0957687	289.0334195
AKAP17A	817.513538	707.966579	1274.729534	984.580511	676.1038349	829.4987321
ELOVL2	0	1.01573397	0	0	1016.674768	19.93333928
GOLGA6L9	214.966015	180.252151	157.6612768	120.633741	448.2235915	137.123253
LEKR1	2.93015605	3.04720192	52.1525852	38.2166082	15.11409467	47.11516556
C1orf122	618.262927	679.526027	1458.266517	1295.34188	1433.823781	1592.85502
OR51A8P	0	0	0	0	0	0
SNHG12	414.128722	368.711432	306.6170836	379.581432	281.7670289	687.700205
ZNF734P	0	0	0	0	0	1.812121752
RP11-310K10.1	0	0	0	0	0	0
CLEC9A	0	2.03146794	0	0	0	0
KEL	0.97671868	0	47.13791354	24.1368052	1.007606311	35.33637417
NOL8	1604.7488	1726.74775	518.517049	648.676639	1477.150852	625.1820046
IRAK4	377.990131	432.702672	355.0387531	545.089517	587.4344795	364.2364722
CCDC151	7.81374947	24.3776153	34.09976724	53.3021114	20.15212623	32.61819154
DLGAP2	0	0	0	0	0	0.906060876
MRPL42	2037.67935	1842.54143	1816.153603	1236.00556	1925.253531	1879.904167
ENTPD7	360.409194	393.089047	327.9595261	360.040677	545.1150144	439.4395249

FCGR1B	0	0	0	0	0	0
SPANXA1	33.6870274	52.2595129	0	0	419.1642255	8.154547886
SAGE2P	0	0	0	0	0	0
ZNF335	1238.47929	1200.59756	1061.104522	1399.9347	1269.583952	1348.218584
ZNF560	0	0	0	0	0	0
TUBA3C	0	0	0	0	0	0
RPS4X	14687.8956	15863.723	23084.53949	17285.9753	16370.57974	12058.7642
ZNF273	139.670772	109.699269	199.5839318	283.607461	258.954822	372.3910201
ZNF84	681.749641	588.10997	1382.043508	1207.84596	1016.674768	1267.579166
MAK16	909.325095	858.295207	383.1209143	318.806968	904.8304675	634.2426133
ZNF667	0	0	0	1.00570022	0	0
AVPR1B	0	0	0	0	0	0
SIRPA	52.7428089	55.8653685	2557.372221	2594.57582	52.39552819	2833.25236
DSCR8	0	0	0	0	4.030425245	7.248487009
GRK6	1644.79426	1534.77403	1046.060507	1145.49255	2606.677527	1723.327786
PRIM1	712.027921	654.132678	450.3175145	584.311825	910.8761054	592.563813
MARCH5	1552.00599	1429.1377	2226.514214	1471.33942	1422.740112	1589.230777
POTEH	0	0	0	0	0	0
RP11-347C12.1	47.7420093	23.7478603	64.64914696	61.7801642	72.960773	71.74190018
AKR1B10	0	11.1730737	0	0	24.18255147	17.21515665
SULT1C4	0	0	0	0	0	0.906060876
CYP2A7	0	0	0	0	0	0.906060876
ZBTB14	328.177478	310.814596	311.9125769	383.171782	481.6358168	258.2273497
KRTAP9-9	0	0	0	0	0	0
CD2AP	4566.15985	4142.16314	2096.132751	2302.04779	1971.885551	3678.607157
NUP62CL	42.9756221	56.8811024	50.14671654	64.3648138	26.19776409	36.24243505
SFI1	528.404808	526.150198	1489.357481	2133.09016	694.2407485	1843.833883
KRTAP4-6	0	0	0	0	0	0
TMPRSS11F	0	0	0	0	0	0
ZNF649	160.181864	184.863583	475.3908728	335.903872	9.068456802	595.7259655
ADH4	0	0	0	0	0	0
OR2T6	0	0	0	0	0	0
ZNF248	366.269506	386.994643	629.8427597	510.895709	450.4000211	405.0092117

RP11-231C14.7	1.04508899	0	44.01878777	23.241732	3.748295478	28.86709952
CHSY3	12.6973429	32.5034871	6.017605984	8.04560172	18.1369136	16.30909577
TOR4A	2577.56061	2594.18457	49.1437822	43.2451093	262.9852472	125.0364009
LPAR1	2675.23248	2157.41896	31.09096425	42.2394091	1.007606311	27.18182629
MB	32.2317166	39.6136249	1061.104522	552.129418	252.9091841	261.8515932
OR2L3	0	0	0	0	0	0
DEFB107B	0	0	0	0	0	0
HIBCH	464.918094	625.692127	463.3556608	358.029277	449.3924148	522.7971256
ZNF544	1157.41164	1148.79512	451.3204488	275.561859	1189.983054	726.6608227
TMEM229B	26.3714045	54.8496345	23.06748961	12.0684026	304.297106	214.7364277
RP11-267J23.4	0	0	1.002934331	0	0	0
SOWAHC	1338.1046	1557.12018	462.3527265	765.337864	325.4568385	540.0122822
ZNF770	1588.14458	1608.92261	1644.812302	1392.8948	1955.76385	1926.285423
ZNF849P	0	0	0	0	1.007606311	0
ZNF876P	0.97671868	1.01573397	20.05868661	18.1026039	69.52483548	59.80001783
NPIPB6	23.3435765	21.7874937	42.41409285	43.2048813	22.30840373	47.27825652
HMGN5	2.93015605	2.03146794	453.3263175	626.551234	50.38031556	483.8365079
MIER1	1684.83973	1611.96981	1387.058179	1414.0145	788.9557417	1832.055092
MAN1A2	2229.84876	2321.96786	1184.465445	1418.0373	4017.326363	1813.933874
SVIP	533.288401	680.541761	942.7582709	1171.64075	863.5186088	1041.970008
ZNF251	450.267313	356.522624	1112.254173	693.933149	418.1566192	813.6426668
DDRGK1	1186.7132	1183.33008	2575.535361	1967.14962	1072.093115	2359.382522
FAM47C	0	0	0	0	0	0
TFDP1	6999.16609	6255.90554	1469.298794	1659.40536	5502.538066	2607.643202
CLEC4C	0	0	0	0	0	0
ZNF607	207.064361	183.847849	469.3732668	562.18642	243.8407273	441.2516467
BPIFA1	0	0	0	0	1.007606311	9.966669638
ZNF334	139.670772	92.4317915	0	0	0	0
HSD17B11	1584.23771	1534.77403	1.002934331	5.02850108	124.9431826	35.33637417
SZT2	772.584479	709.998047	1773.187897	1365.74089	1084.184391	1590.136838
SULT1C2	3.90687474	7.11013781	4.011737323	0	0	3.624243505
ZXDA	108.992038	90.1768621	247.4640168	245.974159	135.6036574	204.8875459
RPS6KL1	305.712948	302.688724	430.2588279	403.285786	201.5212623	357.8940461

RP11-566K11.2	0	8.53216537	2.226514214	2.3432815	0	0
CACNA1E	0	0	0	1.00570022	1.007606311	0
OR51H2P	0	0	0	0	0	0
QRICH1	3460.5143	3193.46761	2372.942626	2156.22126	1847.949975	2616.70381
MLLT4-AS1	4.88359342	7.11013781	13.0381463	22.1254047	10.07606311	11.77879139
CSF2RA	439.035048	487.552307	0	0	3.52662209	0.453030438
FKBP1C	51.3949371	46.0127489	6.669513299	10.7006503	18.29813061	19.6433998
DDX42	4212.58768	3570.30491	9193.89901	5127.0597	6871.875043	8738.05109
RP11-98J23.2	28.9987777	39.9081878	33.02662751	55.806305	61.34307223	45.37552868
RPL23A	12231.7313	12194.6786	14206.11347	13036.6807	12848.55233	10759.74472
SLC29A3	208.04108	183.847849	116.3403824	228.293949	421.1794381	253.6970453
CTC-490E21.13	0	0	0	0	0	0
STYX	1216.99148	1211.77063	627.836891	905.130194	1383.443465	1246.739766
UBL5	1511.96052	1686.11839	1654.841646	1943.01282	1081.161572	1559.330768
OR5BB1P	0	0	0	0	0	0
HELZ	1974.92518	1912.62707	5263.399368	6474.69799	5713.127785	8288.644895
TMEM116	456.127625	429.65547	280.8216126	390.211684	334.5252953	271.8182629
KRTAP4-5	0	0	0	0	0	0
UCKL1	1667.25879	1560.16738	908.6585036	988.603312	1044.887745	761.091136
RP11-468N14.09	0	0	0	0	0	0
OR5B21	0	0	0	0	0	0
NUP210P1	0	0	0	0	0	0
CARD11	855.605567	1048.23746	0	0	72.54765441	34.43031329
ZNF485	156.274989	149.312894	153.4489526	86.4902185	237.7950895	240.1061322
PEG3	0	0	2.005868661	0	0	0
SDAD1	1475.82193	1596.7338	1081.163209	846.799581	1471.105214	1268.485227
BMS1P9	0	0	0	0	1.027758438	0
ZKSCAN8	2219.10485	1988.80712	1143.345137	978.54631	766.7884029	1273.921592
FAM109A	528.404808	513.96139	233.6836991	202.145743	503.8031556	344.3031329
TMEM239	0	1.01573397	0	0	0	0
HYLS1	1079.27415	1049.25319	482.4114131	577.271924	304.297106	441.2516467
MYL4	0	1.01573397	0	0	0	0
ZNF442	23.5193859	53.8339005	85.24941811	51.290711	60.45637868	51.9082276

ZNF813	120.995911	145.9305	0	0	365.6704064	12.9294887
HOXC4	1.95343737	1.01573397	311.3509336	239.416993	4.504000211	348.679407
DCAF12L2	0	0	4.011737323	15.0855032	5.038031556	13.59091314
PIM3	1839.16128	1577.43486	2951.635735	3039.22605	2032.34193	2062.194554
ASNA1	1834.27769	2150.30882	3815.162194	6158.90812	2088.767883	3492.864678
RP11-544M22.1	0	0	0	0	0	0
ASPH	6027.331	5233.06142	45731.79961	27508.918	1253.462251	38274.72959
OR7A11P	0	0	0	0	0	0
SPRED2	1850.88191	2080.22318	5480.033183	5072.75189	1262.530708	5343.040987
WWP2	1208.20101	1208.72343	1528.47192	1682.53646	2425.308391	1681.648986
GFPT1	3239.77587	3587.57239	7371.567331	4610.12979	2755.803261	3426.722234
UVRAG	779.42151	943.61686	849.4853781	1049.95103	1226.256881	882.5032934
TPTE2P3	0	0	0	0	0	0.906060876
KRTAP13-1	0	0	0	0	0	0
ZNF26	324.270603	335.192211	576.6872402	527.992613	507.8335809	639.6789786
TMEM207	0	0	0	0	0	0
ITSN2	1753.21004	2003.02739	1362.987755	1652.36545	1932.588905	1310.164027
NTRK1	0	0	0	0	3.022818934	0
BZW1P2	149.965387	119.12528	32.49507231	57.9283324	128.0768382	88.38623847
MGEA5	3697.85694	3760.24717	4901.340074	3377.14132	3189.073975	3932.304203
TATDN2P1	0	0	0	0	0	0
ZNF658B	21.5171126	41.2692713	97.92650805	130.167779	56.94990871	103.2909399
MT1F	47.8592155	86.3373876	153.4489526	141.80373	51.38792188	54.36365257
TCAF1	2755.07923	2396.14691	2788.087234	4083.42447	2777.486949	2933.471753
ZNF69	210.971236	184.863583	0	0	21.15973254	5.436365257
TXNRD1	15221.184	17419.8376	4825.117065	4643.3179	11678.15715	7637.187125
NRARP	73.2539013	47.7394967	777.2741063	566.209221	618.6702751	1161.570043
ZNF583	125.019992	125.951013	168.4929676	166.946236	20.15212623	130.4727662
KRTAP4-1	0	0	0	0	21.15973254	0.906060876
CCT8L2	0	0	0	0	0	0
OR14L1P	0	0	0	0	0	0
ZNF568	90.8348376	115.793673	268.7864006	230.305349	85.64653646	202.9576363
PRR31	0	0	0	0	0	0

ZXDB	551.269792	423.784528	702.3147944	685.304241	526.3936891	567.0763206
ZNF480	553.799494	669.368688	701.0510972	836.742579	908.8608928	928.7123981
ZNF587	1318.03303	1219.85587	1139.704485	1005.91141	1244.756533	1383.790534
TPM2	893.697596	900.956033	31.09096425	23.131105	251.9015778	146.7818619
FLVCR1-AS1	13.6740616	33.5192211	45.13204488	202.145743	18.1369136	36.24243505
RTP2	0	0	1.002934331	0	0	0
SH3BGRL2	108.415774	128.998214	96.28169575	57.3249123	405.0577371	97.85457463
ZNF808	249.825105	249.911187	478.3996757	477.255037	376.3711855	572.023413
ANKRD35	0	0	4.011737323	6.03420129	38.28903983	4.530304381
B3GNT6	0	0	2.005868661	0	8.06085049	0
RP11-208N14.4	0	0	0	0	0	0
YTHDF2	2366.56984	2187.88082	2860.358682	2575.59825	1988.007252	3388.613313
NBR2	64.9322581	67.0384422	121.7863158	56.7717772	65.56494268	63.00747333
TMA16	580.170898	615.534787	428.2529592	323.835469	798.0241985	456.6546816
HLA-DRB5	57.9682539	13.5194192	0	0	289.5759778	56.48383502
ATL1	253.946858	257.996429	20.05868661	25.1425054	15.11409467	43.49092206
CNGA1	19.5343737	15.2360096	1.002934331	0	2.015212623	1.812121752
MAFK	1044.11227	899.940299	590.7283208	729.132656	1264.545921	1070.963956
C1orf228	0.97671868	4.06293589	13.0381463	20.1140043	7.053244179	10.87273051
ZNF43	486.405905	470.284829	684.0012135	637.613937	582.7894144	1043.782129
GPN1	1503.17005	1456.56252	754.2066167	745.435057	1290.743685	894.4995394
PLN	0	0	0	0	0	0
PABPC1P2	0	0	0	0	0	0
C2CD4A	0	0	165.4841646	226.282548	7.053244179	179.4000535
ZNF28	379.816595	446.689329	878.7710606	1057.90611	735.7944328	929.500671
ITGBL1	135.763897	78.2115159	0	0	2.015212623	1.812121752
ZNF511	1058.76305	946.664062	535.1055828	640.661208	915.914137	601.0354822
C20orf203	0.97671868	0	13.0381463	5.02850108	1.007606311	1.812121752
ZNF627	191.436862	217.36707	438.2823025	382.166082	386.9208235	364.2364722
KCNRG	22.4645297	13.2553283	3.028861679	17.3483287	2.942210429	5.56321378
WDHD1	2278.68469	2423.54126	671.9660016	808.582973	1455.99112	1463.288315
RP11-598D12.4	0	0	0	0	0	0
ZNF789	217.808267	228.540144	209.6132751	249.413653	622.7007004	298.0940283

CTNND1	5261.58355	4580.96021	6911.220473	7998.33381	4821.3962	7898.132658
DDX39B	10627.3048	8686.31315	8506.156851	6703.02211	7388.484875	10293.07807
SLC34A3	5.8603121	8.12587178	3.008802992	1.00570022	20.15212623	1.812121752
RD3	0	0	0	0	0	0
SPANXC	3.61385913	0	0	0	0	0
SH2D1B	23.4412484	23.3618814	0	0	0	0
ARC	3.90687474	14.2202756	117.3433167	114.649825	57.43355974	110.5394269
RP11-611L7.2	0	0	1.002934331	2.01140043	1.007606311	3.624243505
NUDT16	749.143231	896.893097	787.3034496	1130.40704	1039.849713	933.2427025
TLK1	2009.11033	2196.01685	1747.111604	2048.61134	1966.84752	2314.985539
LRBA	1863.57925	1912.62707	3655.695635	6876.97807	2616.75359	6303.465516
C3orf35	10.7439055	6.09440383	34.09976724	21.1197045	8.06085049	28.08788716
ZNF536	0	0	0	0	0	0
MMP17	50.7893716	73.132846	1623.750681	1038.88832	750.6667019	1131.670034
OR2M2	0	0	0	0	0	0
BAZ1A	3322.79696	3265.58472	3085.026001	3252.4345	3905.482063	3824.482958
AKR1C4	0	0	0	0	0	0
COPS8	1067.55352	1034.01718	860.5176557	830.708378	1234.317731	868.9123803
PPIAP22	283.394926	371.067935	317.5189798	459.253003	461.2922454	400.7144831
CCDC69	1748.32644	2171.63923	18.05281795	55.3135119	299.2590745	125.0364009
MDM4	1222.85179	957.837136	5188.179293	3754.2789	2784.016238	4038.313325
RYR2	4.88359342	4.06293589	120.3521197	61.3477131	5.038031556	57.98789608
ZNF534	0.97671868	0	0	0	0	0.906060876
KLHL9	0	0	1087.180814	900.101693	867.549034	596.1880565
FAM3D	2.93015605	0	0	0	0	0
NCOA6	3875.61974	3463.65285	2277.663865	3237.34899	2317.494516	2835.064482
STK39	1678.0027	1281.85627	421.2324189	616.494232	1505.363829	630.6183698
TAT	0	0	503.473034	54.3078116	0	329.8061589
OR8B4	0	0	0	0	0	0
ABHD17AP1	5.09847153	4.28639736	6.388691687	10.5799663	2.156277506	2.890334195
C6orf89	3971.33817	3826.26987	1346.940806	1090.17903	2505.916896	1588.324716
CALM1	8801.21206	8815.55515	5918.315486	7672.48694	14654.62619	7302.850662
LPA	0	0	0	0	0	0

RP11-168J19.2	0	0	0	0	0	0
FAM19A2	4.88359342	6.09440383	19.05575228	9.05130194	2.015212623	10.87273051
OR10G6	0	0	0	0	0	0
TTC37	3057.12948	3170.10573	4006.722651	3583.30987	1760.288226	3107.788805
OR5BS1P	0	0	0	0	0	0
TUSC1	287.155293	292.531384	2636.714355	3103.59087	297.2438618	1823.900544
MAGEA1	0	0	0	0	0	0
PAPSS2	3405.81805	2678.49048	0	3.01710065	7800.888062	449.4061946
LINC01565	0	0	0	0	0	0
SLC9A6	893.697596	1053.31613	201.5898005	374.12048	744.621064	337.0546459
FAN1	645.61105	539.354739	1612.718404	903.118794	611.617031	1482.315593
ABCA4	5.8603121	4.06293589	2.005868661	20.1140043	9.068456802	22.6515219
EIF1AY	0	0	0	0	0	0
MT-ND6	6417.03199	8185.80008	3823.145551	7554.79991	5722.115633	6830.774824
IPO9	4508.53344	4083.25057	7439.766865	7441.17589	2968.408193	9861.566576
OR10R3P	0	0	0	0	0	0
GPX6	0	0	0	0	0	0
CEP290	1430.89287	1272.71467	1442.219568	851.828083	664.0125591	1010.257877
SSBP3-AS1	0	1.01573397	13.0381463	2.01140043	1.007606311	2.718182629
MT-CO2	154252.752	128770.477	195268.165	240187.762	173881.6841	229355.0585
GLMP	759.887136	970.025943	548.6050789	1697.62196	459.4684779	988.5124159
FAM179B	593.84496	599.283044	859.5147214	956.420905	1087.20721	1163.382165
DLL1	14.6507803	8.12587178	468.3703324	257.459255	274.0689167	395.9486029
ANKRD13B	644.634331	590.141438	219.6426184	248.407953	356.6926342	397.7607246
ECI2	2306.03281	2189.92244	2433.118686	2012.40613	344.6013585	2663.818976
UNC13B	1236.52585	1201.61329	1499.386824	2010.39473	1715.953548	1886.418744
C19orf45	1.95343737	4.06293589	17.04988362	20.1140043	8.06085049	18.12121752
MT-CYB	70537.6466	83528.8832	139018.7334	149158.416	128672.3336	131764.809
LDB1	1067.55352	1126.44898	1232.606292	1460.27671	1181.922203	1488.65802
PPP1R14C	20.5110924	24.3776153	0	0	49.37270925	19.0272784
CTR9	2550.21248	2671.38035	892.631613	892.056091	1383.443465	1201.445782
SMOC1	0.97671868	0	6.017605984	1.00570022	2.015212623	13.59091314
F5	0	0	4.011737323	0	4.030425245	3.624243505

MSRB1	392.640911	454.033086	614.7987447	1102.24744	1528.538774	1099.051843
SMIM11P1	0	0	0	0	0	0
LRRTM3	0	0	0	0	4.030425245	1.812121752
ZNF652	645.61105	534.276069	3410.979659	4399.93844	2547.228755	3944.082994
SMURF1	1791.30207	1686.11839	352.0299501	435.468193	2487.779983	787.3669014
SLC5A3	3943.71656	3920.12369	1199.429225	1563.46156	1251.326126	1231.662913
MTCO3P12	84.3787271	99.145793	101.9382454	152.444039	88.21593255	143.7193762
GPATCH3	252.970139	278.311108	265.7775976	415.354189	211.5973254	363.3304113
CDC42BPB	5732.36196	5147.73977	6885.14418	6586.33071	8915.300642	8172.669103
PLXNB3	490.009997	367.309719	1830.034215	1076.88368	970.6473118	1466.957862
OXCT2	22.4645297	9.14160575	0	0	21.77437239	0
RPL10A	13133.8287	13103.923	12845.48261	14590.6082	10514.28117	9844.278935
COLGALT2	126.973429	87.3531216	8.023474646	0	13.09888205	14.49697402
EPS8L3	0	1.01573397	0	0	0	0
EGFL6	0	0	0	0	0	0
MT-ND2	135012.898	169878.155	119350.4992	106879.463	108844.6666	120250.4696
SYCP1	0	0	0	0	0	0
APCDD1L	388.734036	476.379233	0	0	60.45637868	28.99394804
RCSD1	414.128722	180.800647	32.09389858	2.01140043	1.007606311	11.77879139
RASSF9	3.90687474	0	0	0	0	0
FAM169A	626.076676	518.024326	6.017605984	1.00570022	222.6809948	28.08788716
ZNF830	288.132012	279.326842	540.5816043	481.730403	228.7266327	535.4819778
GRIN3A	0.97671868	1.01573397	0	0	0	0.906060876
MT-ND5	90894.4174	83633.5038	127159.035	163743.091	123771.3264	185810.4251
OR4D12P	0	0	0	0	0	0
MUC2	13.6740616	3.04720192	3.008802992	12.0684026	44.3346777	167.6212621
CNOT7	1587.16786	1656.66211	1022.993017	1140.46404	3401.678907	1505.873176
TMEM184B	3238.79916	3501.235	2514.356367	3594.37257	1823.767423	2898.488743
MTOR	2809.04293	2916.17223	3505.255486	3359.03872	3228.370621	4855.580235
SCAMP5	535.241839	552.559281	7.020540315	2.01140043	668.0429844	90.60608762
ZNF521	164.088739	130.013948	18.05281795	30.1710065	0	21.74546103
ALPK2	756.95698	500.756848	0	0	0	0
BRINP2	0	0	0	0	17.12930729	2.718182629

MAGEB3	0	0	0	0	0	0
LRIG2	952.300717	927.365117	618.810482	693.933149	508.8411872	827.23358
MT-CO1	399390.652	333581.176	564989.9469	755107.379	296936.4109	713651.0661
PNP	1718.04816	1993.88579	1080.160274	2989.94674	1784.470777	2180.888529
PAX9	7.81374947	9.14160575	892.6115543	1001.67741	200.5136559	496.5213601
LRRC10	0	0	0	0	0	0
GK	217.798499	235.548708	760.0637532	460.570471	195.4453962	398.6124219
FOXJ3	1757.11691	1822.22675	1937.669127	2111.97045	1912.436779	2489.855288
ZNF358	1132.01695	1160.97377	1666.876858	1983.24083	554.1834712	980.357868
SFT2D1	1277.54804	1290.99788	1039.039967	1030.84272	497.7575178	839.9184322
CD247	0	0	1.002934331	0	0	0.906060876
GRM3	0	0	0	0	0	2.718182629
CHAMP1	1874.32315	1940.05189	505.4789027	585.317525	1098.290879	727.5668836
INPP5F	1046.81778	1122.3251	296.8685619	388.200283	829.7738734	444.8758902
ARHGAP11A	4080.23253	3990.69689	1829.763422	3570.84924	3697.743869	4081.25155
SUCNR1	0	0	0	0	0	0
HMGN2	8838.24923	9525.46178	9598.813687	16818.5258	11662.95237	13539.05022
SELM	322.317166	343.318083	94.27582709	26.1482056	120.9127574	110.5394269
UBE2J1	2196.64032	1925.83161	2580.550033	2309.08769	1938.634543	1736.9187
GJC2	24.4179671	18.2832115	12.03521197	3.01710065	15.11409467	22.6515219
OPA1	4049.47566	3931.90621	3289.624605	3747.239	3502.439538	4433.355867
DENND4B	1361.54585	1293.02935	3205.378121	2574.59255	2269.129413	2636.63715
RYR3	0	0	0	0	0	1.812121752
ZNF277	296.92248	271.200971	654.916118	928.261299	378.859973	412.2576987
MT-ND3	20947.6856	23081.5388	21764.67791	30535.0699	17131.3225	23882.85864
KTI12	321.526024	331.494939	307.389343	325.203222	371.4641427	323.1284903
DUSP27	0.97671868	1.01573397	0	0	0	2.718182629
SELT	1748.30691	1683.04072	2616.625581	2136.05697	3610.203033	2274.049708
ARHGEF15	0.97671868	0	0	0	0	0
TOX	68.3703079	74.14858	0	0	0	0.906060876
CES1	0	0	0	0	1.007606311	1.812121752
CD3E	0	0	0	0	0	0
RUSC2	1866.5094	1865.90331	34.09976724	64.3648138	364.7534847	153.1242881

C1orf68	0	0	0	0	0	0
FICD	291.062168	329.097807	452.3233831	312.772767	295.2286492	250.0728018
OSTC	1310.75647	1576.41912	2376.954364	1443.17981	1512.417073	1340.064036
HSD3BP5	0	0	0	0	0	0
R3HDM4	1279.50148	1237.16398	1080.160274	947.369603	1540.63005	1594.667142
TSEN15	1526.6113	1233.10104	1689.944347	620.517033	1200.059117	862.5699541
LTN1	1861.62581	1566.26179	1429.181421	1330.54139	1559.77457	1887.324805
RUNDC1	791.142134	824.775985	1049.06931	753.269461	955.2107831	1051.936677
CCDC152	0	0	28.08216126	27.1539058	1.007606311	11.77879139
MTND4LP30	0	0	1.002934331	0	0	0
STKLD1	13.6740616	8.12587178	31.09096425	52.2964112	15.11409467	35.33637417
GRK5	635.843863	856.263739	30.08802992	62.3534134	97.73781219	34.43031329
TYW1	548.759625	559.801464	685.5958791	590.879048	806.8306777	866.7106523
DCAF12	1330.29085	1371.24086	3118.122834	3073.41986	1796.562053	3395.916164
OR5D13	0	0	0	0	0	0
SFMBT2	0	0	2082.091671	1451.22541	247.8711526	1900.915718
ASB12	0	0	0	1.00570022	0	0.906060876
PNMA5	0	0	0	0	0	0.906060876
ITPRIPL1	0	0	0	0	0	0
MT-ND4	216829.467	213845.409	236986.3518	388984.709	213963.1749	367772.8006
SMC5	1925.11253	1590.6394	636.8633	648.676639	1689.755784	740.2517358
MT-ND1	111295.756	117240.982	66724.36853	67491.4955	77523.36552	79766.83605
DCAF12L1	0	0	0	0	0	0
PRMT6	534.26512	579.984098	573.6784372	425.411191	6998.833438	924.1820937
SHISA4	120.136398	144.234224	74.21714047	128.729628	102.7758438	69.76668747
CIPC	690.540109	739.454332	1279.543619	1398.07415	1229.2797	1714.267178
CAPZA2	2083.34095	2203.12699	1613.721338	1789.14068	3129.625203	1727.858091
MT-ATP6	110374.349	113789.904	145128.3787	178851.695	86924.92689	148885.9165
TOP1	6371.13597	5982.6731	5594.367697	5234.66962	9348.571356	6629.647431
PRC1	6502.01628	5656.62249	1374.020033	2336.2416	5768.546132	2334.012817
BHLHB9	288.132012	388.010377	119.3491854	117.666925	217.6429632	91.51214849
MAP3K3	2349.98515	2387.99057	1380.037639	1755.95258	2286.25872	1220.464
L1CAM	3210.47431	2321.96786	1.002934331	0	4065.691466	320.7455502

SREBF2	3644.13741	4666.28187	8504.883124	21409.3462	2810.214002	4890.010549
C1orf174	567.473555	641.94387	430.2588279	544.083817	425.2098634	508.3001515
POU3F3	0	0	0	0	0	0
RASGEF1A	15.6274989	13.2045416	22.06455528	9.05130194	20.15212623	36.24243505
C9orf114	920.069	969.01021	693.0276225	669.796344	1796.562053	1128.045791
RPL39	6355.53778	6345.91988	10520.68084	8694.46945	10939.03761	6110.157428
DZIP3	571.38043	542.401941	502.4700997	455.582198	450.4000211	568.1001694
KIAA0753	918.115563	867.436812	276.8098753	209.185645	623.7083067	334.3364633
RP11-290L1.7	0	0	0	0	0	0
DCLRE1A	878.070097	812.587178	619.8134164	757.292262	659.9821339	737.5335532
ATG9A	2152.25822	2219.44983	1208.957101	1277.23927	1885.835972	1681.014744
NOS1AP	84.8084833	95.3875773	19.05575228	38.5585463	352.0072648	46.96113521
CSAG1	0	0	0	0	0	0.906060876
APRT	1409.40506	1391.55554	1470.301729	1761.98678	5668.793107	2670.161402
GPRASP1	19.5343737	37.582157	137.4020033	80.4560172	102.7758438	48.92728731
TBKBP1	156.274989	145.249958	558.6344222	334.898172	546.1226207	719.4123357
MAGEE1	167.995614	185.879317	269.789335	264.499157	19.14451991	215.6424885
CCDC167	464.918094	395.120515	212.6220781	138.78663	204.5440812	117.7879139
MT-CO3	153661.886	153235.039	141546.489	234468.365	127262.1381	226293.5784
ZFP2	3.90687474	5.07866986	31.09096425	27.1539058	4.030425245	36.24243505
SOWAHA	3.90687474	10.1573397	5.014671654	11.0627024	18.1369136	21.74546103
L3MBTL3	159.205145	174.706243	70.20540315	19.1083041	96.73020588	38.0545568
DMD	0	0	0	0	0	4.530304381
MFAP3L	211.947954	211.272666	105.3081047	61.3477131	131.9964268	156.7485316
NAGA	671.982454	652.10121	1764.161488	2624.87756	510.8563998	2339.449182
SMG5	3599.20835	3508.34514	4752.905793	4209.8611	5145.845432	4764.974148
KIF1BP	2368.54281	2320.95213	1372.014164	1383.8435	1275.62959	1416.173149
TGM2	25394.6858	22144.0163	0	0	1916.467204	533.6698561
ARMCX6	544.032307	649.978326	1199.409166	1199.80036	928.0054127	692.6472974
PJA2	4369.83939	4058.87295	3387.912169	3779.42141	1716.961154	3619.7132
RORB	2.93015605	0	0	0	2.015212623	0
SGMS1	1574.47052	1465.70412	419.2265502	749.246661	918.9369559	680.451718
OR10R2	0	0	0	0	0	0

OR10Z1	0	0	0	0	0	0
MIRLET7E	0	0	0	0	0	0
MIR375	0	0	0	0	0	0
MIR30E	0	0	0	0	0	0
MIRLET7A2	0	0	0	0	0	0
MIR429	0	0	0	0	0	0
AL132709.2	0	0	0	0	0	0
MIR449A	0	0	0	0	0	0
MIR345	0	0	0	0	0	0
MIRLET7A3	0	0	0	0	0	0
MIR16-2	0	0	0	0	0	0
MIR340	0	0	0	0	0	0
MIR107	0	0	0	0	0	0
MIR448	0	0	0	0	0	0
MIR21	0	0	0	0	0	0
MIR370	0	0	0	0	0	0
MIR412	0	0	0	0	0	0
MIR377	0	0	0	0	0	0
MIR1-1	0	0	0	0	0	0
MIR381	0	0	0	0	0	0
MIR339	0	0	0	0	0	0
MIR103A2	0	0	0	0	0	0
MIR369	0	0	0	0	0	0
MIRLET7C	0	0	0	0	0	0
MIR371A	0	0	0	0	0	0
MIR425	0	0	0	0	0	0
MIR103A1	0	0	0	0	0	0
MIR219A1	0	0	0	0	0	0
MIR210	0.97671868	0	0	0	0	0
MIR335	0	0	0	0	0	0
MIR378A	0	0	0	0	0	0
MIR361	0	0	0	0	0	0
MIR324	0	0	0	0	0	0

MIR135B	0	0	0	0	0	0
MIR101-2	0	0	0	0	0	0
MIR330	0	0	0	0	0	0
MIR323A	0	0	0	0	0	0
MIR423	0	0	0	0	0	0
MIRLET7F1	0	0	0	0	0	0
MIR26A1	0	0	0	0	0	0
MIR129-2	0	0	0	0	0	0
MIR133B	0	0	0	0	0	0
MIR342	0	0	0	0	0	0
MIR148A	0	0	0	0	0	0
MIR379	0	0	0	0	0	0
MIR326	0	0	0	0	0	0
MIR410	0	0	0	0	0	0
MIR30C2	0	2.03146794	1.002934331	0	0	0
MIR372	0	0	0	0	0	0
MIR302C	0	0	0	0	0	0
MIR346	0	0	0	0	0	0
MIR409	0	0	0	0	0	0
MIR411	0	0	0	0	0	0
MIR26B	0	0	0	0	0	0
MIR148B	0	0	0	0	0	0
MIR383	0	0	0	0	0	0
MIR365A	0	0	0	0	0	0
MIR450A1	0	0	0	0	0	0
MIRLET7D	0	0	0	0	0	0
MIR101-1	0	0	0	0	0	0
MIR373	0	0	0	0	0	0
MIR302D	0	0	0	0	0	0
MIRLET7G	0	0	0	0	0	0
MIR337	0	0	0	0	0	0
MIR30D	0	0	0	0	0	0
MIR422A	0	0	0	0	0	0

MIR208A	0	0	0	0	0	0
MIR96	0	0	0	0	0	0
MIR126	0	0	0	0	0	0
MIRLET7A1	0	0	0	0	0	0
MIR374A	0	0	0	0	0	0
MIR367	0	0	0	0	0	0
MIR331	0	0	0	0	0	0
MIR31	0	0	0	0	0	0
MIRLET7I	0	0	0	0	0	0
MIR365B	0	0	0	0	0	0
SNORA25	0	0	0	0	0	0
RN7SKP72	0	0	0	0	0	0
Y_RNA	0	0	0	0	0	0
Y_RNA	0	0	0	0	0	0
RNA5SP289	0	0	0	0	0	0
Y_RNA	0	0	0	0	0	0
Y_RNA	0	0	0	0	0	0
RNU105C	0	0	0	0	0	0
RNU6-1123P	0	0	0	0	0	0
RN7SKP184	0	0	0	0	0	0
RNU6-500P	0	0	0	0	0	0
Y_RNA	0	0	0	0	0	0
Y_RNA	0	0	0	0	0	0
Y_RNA	0	0	0	0	0	0
Y_RNA	0	0	0	0	0	0
RNU6-50P	0	0	0	0	0	0
snoU2_19	0	0	0	0	0	0
RNU6-834P	0	0	0	0	0	0
RNA5SP46	0	0	0	0	0	0
Y_RNA	0	0	0	0	0	0
Y_RNA	0	0	0	0	0	0
RNU6-896P	0	0	0	0	0	0
RNU6-28P	0	0	0	0	0	0

RNU6-664P	0	0	0	0	0	0
RNU6-874P	0	0	0	0	0	0
Y_RNA	0	0	0	0	0	0
SNORA60	0	0	0	1.00570022	0	0
RNA5S12	0	0	0	0	0	0
RNU6-349P	0	0	0	0	0	0
Y_RNA	0	0	0	0	0	0
RNA5SP26	0	0	0	0	0	0
RNU6-661P	0	0	0	0	0	0
SNORA9	0	0	0	0	0	0
RNU1-58P	0	0	0	0	0	0
Y_RNA	0	0	0	0	0	0
RNU6-1094P	0	0	0	0	0	0
RNU6-502P	0	0	0	0	0	0
Y_RNA	0	0	0	0	0	0
Y_RNA	0	0	0	0	0	0
SNORA21	0	0	0	0	0	0
RNU6-1312P	0	0	0	0	0	0
RNA5SP430	0	0	0	0	0	0
RNU6-208P	0	0	0	0	0	0
Y_RNA	0	0	0	0	0	0
RNU6-858P	0	0	0	0	0	0
RNU6-222P	0	0	0	0	0	0
SNORD115-34	0	0	0	0	0	0
RNU4-82P	0	0	0	0	0	0
RNA5SP347	0	0	0	0	0	0
RNA5SP52	0	0	0	0	0	0
RN7SKP25	0	0	0	0	0	0
SNORD60	0	0	0	0	0	0
RNA5SP183	0	0	0	0	0	0
RNU4-39P	0	0	0	0	0	0
RNU6-1298P	0	0	0	0	0	0
RNU6-230P	0	0	0	0	0	0

Y_RNA	0	1.01573397	0	0	0	0
Y_RNA	0	0	0	0	0	0
RNA5S11	0	0	0	0	0	0
RNU6-204P	0	0	0	0	0	0
RNA5S3	0	0	0	0	0	0
RNU5E-1	0	0	0	1.00570022	0	0.906060876
RNU6-766P	0	0	0	0	0	0
Y_RNA	0	0	0	0	0	0
RNA5SP432	0	0	0	0	0	0
RNA5S1	0	0	0	0	0	0
RNA5SP273	0	0	0	0	0	0
Y_RNA	0	0	0	0	0	0
RNU6-1115P	0	0	0	0	0	0
RNU1-150P	0	0	0	0	0	0
Y_RNA	0	0	0	0	0	0
SNORA63	0	0	0	0	0	0
RNA5SP327	0	0	0	0	0	0
Y_RNA	0	0	0	0	0	0
RNU6-1084P	0	0	0	0	0	0
U3	0	0	0	0	0	0
RNA5SP453	0	0	0	0	0	0
RNU5F-1	0	0	0	0	0	0
Y_RNA	0	0	0	0	0	0
RNU6-79P	0	0	0	0	0	0
RNU6-369P	0	0	0	0	0	0
SNORD114-7	0	0	0	0	0	0
SNORA25	0	0	0	0	0	0
RNU6-600P	0	0	0	0	0	0
RNA5SP93	0	0	0	0	0	0
RNA5S5	0	0	0	0	0	0
Y_RNA	0	0	0	0	0	0
RNY4P19	0	0	0	0	0	0
RNA5SP308	0	0	0	0	0	0

RNA5SP250	0	0	0	0	0	0
SNORA1	0	0	0	0	0	0
RNA5SP301	0	0	0	0	0	0
Y_RNA	0	0	0	0	0	0
Y_RNA	0	0	0	0	0	0
SNORD62	0	0	0	0	0	0
RNA5SP370	0	0	0	0	0	0
RN7SKP170	0	0	0	0	0	0
VTRNA3-1P	0	0	0	0	0	0
Y_RNA	0	0	0	0	0	0
RNU1-108P	0	0	0	0	0	0
SNORD9	0	0	0	0	0	0
Y_RNA	0	0	0	0	0	0
Y_RNA	0	0	0	0	0	0
Y_RNA	0	0	0	0	0	0
RNU6-543P	0	0	0	0	0	0
RNU6-845P	0	0	0	0	0	0
RNA5SP276	0	0	0	0	0	0
SNORD115-12	0	0	0	0	0	0
RNA5SP388	0	0	0	0	0	0
RNA5SP191	0	0	0	0	0	0
RNU4-35P	0	0	0	0	0	0
Y_RNA	0	0	0	0	0	0
RNU6-1216P	0	0	0	0	0	0
Y_RNA	0	0	0	0	0	0
Y_RNA	0	0	0	0	0	0
RNU6-556P	0	0	0	0	0	0
RNU1-62P	0	0	0	0	0	0
SNORA64	0	0	0	0	0	0
Y_RNA	0	0	0	0	0	0
Y_RNA	0	0	0	0	0	0
SNORA63	0	0	0	0	0	0
SNORA27	0	0	0	0	0	0

RN7SKP104	0	0	0	0	0	0
Y_RNA	0	0	0	0	0	0
SNORA31	0	0	0	2.01140043	1.007606311	0
RNA5SP389	0	0	0	0	0	0
RNU6-633P	0	0	0	0	0	0
RNU4-15P	0	0	0	0	0	0
Y_RNA	0	0	0	0	0	0
RNU1-70P	0	0	0	0	0	0
SNORD115-25	0	0	0	0	0	0
Y_RNA	0	0	0	0	0	0
RNU6-1313P	0	0	0	0	0	0
RNU1-94P	0	0	0	0	0	0
RNU6-247P	0	0	0	0	0	0
RNA5SP110	0	0	0	0	0	0
RNA5SP477	0	0	0	0	0	0
RNU6-212P	0	0	0	0	0	0
RNU6-235P	0	0	0	0	0	0
Y_RNA	0	0	0	0	0	0
Y_RNA	0	0	0	0	0	0
RNU6-386P	0	0	0	0	0	0
RNA5SP226	0	0	0	0	0	0
RNA5SP295	0	0	0	0	0	0
RNU6-462P	0	0	0	0	0	0
Y_RNA	0	0	0	0	0	0
RNA5SP305	0	0	0	0	0	0
RNU6-315P	0	0	0	0	0	0
Y_RNA	0	0	0	0	0	0
RNA5SP195	0	0	0	0	0	0
Y_RNA	0	0	0	0	0	0
Y_RNA	0	0	0	0	0	0
RNU6-545P	0	0	0	0	0	0
SNORA63	0	0	0	0	0	0
RNA5SP361	0	0	0	0	0	0

RNU6-37P	0	0	0	0	0	0
RNA5SP502	0	0	0	0	0	0
Y_RNA	0	0	0	0	0	0
SNORA64	0	0	0	0	0	0
Y_RNA	0	0	0	0	0	0
RNU5A-1	0	3.04720192	1.002934331	0	3.022818934	0
RNU6-228P	0	0	0	0	0	0
SNORA22	0	0	0	0	0	0
RNA5SP174	0	0	0	0	0	0
SNORD18C	0	0	0	0	0	0
SNORD114-1	0	0	0	0	0	0
RNU6-822P	0	0	0	0	0	0
Y_RNA	0	0	0	0	0	0
Y_RNA	0	0	0	0	0	0
RNA5SP119	0	0	0	0	0	0
Y_RNA	0	0	0	0	0	0
RNA5SP321	0	0	0	0	0	0
SNORD114-14	0	0	0	0	0	0
RNU6-1301P	0	0	0	0	0	0
Y_RNA	0	0	0	0	0	0
RNU6-859P	0	0	0	0	0	0
RNU6-562P	0	0	0	0	0	0
RNU6-951P	0	0	0	0	0	0
RNY4P24	0	0	0	0	0	0
RNA5SP124	0	0	0	0	0	0
RNA5SP258	0	0	0	0	0	0
RN7SKP20	0	0	0	0	0	0
RNU6-989P	0	0	0	0	0	0
RNU6-1010P	0	0	0	0	0	0
RNU1-14P	0	0	0	0	0	0
Y_RNA	0	0	0	0	0	0
SNORD33	0	0	0	0	0	0
SNORA63	0	0	0	0	0	0

RNU4-79P	0	0	0	0	0	0
Y_RNA	0	0	0	0	0	0
Y_RNA	0	0	0	0	0	0
RNA5SP319	0	0	0	0	0	0
RN7SKP294	0	0	0	0	0	0
RNU4-90P	0	0	0	0	0	0
RNU6-1330P	0	0	0	0	0	0
RNU6-1272P	0	0	0	0	0	0
RNU1-35P	0	0	0	0	0	0
RNU6-1266P	0	0	0	0	0	0
U3	0	0	0	0	0	0
Y_RNA	0	0	0	0	0	0
Y_RNA	0	0	0	0	0	0
RNU4-21P	0	0	0	0	0	0
SNORD16	0	0	0	0.50285011	0.221673388	0.670485048
RNU6-862P	0	0	0	0	0	0
Y_RNA	0	0	0	0	0	0
Y_RNA	0	0	0	0	0	0
RN7SKP185	1.95343737	0	0	0	0	0
RNU1-38P	0	0	0	0	0	0
RN7SKP173	0.97671868	3.04720192	0	5.02850108	1.007606311	0
RNU6-1128P	0	0	0	0	0	0
RNU6-446P	0	0	0	0	0	0
Y_RNA	0	0	0	0	0	0
RNU6-223P	0	0	0	0	0	0
Y_RNA	0	0	0	0	0	0
RNU6-170P	0	0	0	0	0	0
SNORD115-29	0	0	0	0	0	0
Y_RNA	0	0	0	0	0	0
RNU4-23P	0.97671868	0	0	0	0	0
Y_RNA	0	0	0	0	0	0
Y_RNA	0	0	0	0	0	0
SNORD115-2	0	0	0	0	0	0

U8	0	0	0	0	0	0
Y_RNA	0	0	0	0	0	0
Y_RNA	0	0	0	0	0	0
RN7SKP74	0	0	0	0	0	0
U3	0	0	0	0	0	0
RNU6-655P	0	0	0	0	0	0
RN7SKP95	0	0	0	1.00570022	0	0
RNU6-1079P	0	0	0	0	0	0
Y_RNA	0	0	0	0	0	0
RNA5SP307	0	0	0	0	0	0
RNU6-227P	0	0	0	0	0	0
RNU6-552P	0	0	0	0	0	0
Y_RNA	0	0	0	0	0	0
SNORD36A	0	0	0	0	0.816161112	0
Y_RNA	0	0	0	0	0	0
SNORD104	0	0	0	0	0	0
Y_RNA	0	0	0	0	0	0
Y_RNA	0	0	0	0	0	0
Y_RNA	0	0	0	0	0	0
Y_RNA	0	0	0	0	0	0
RNU6-363P	0	0	0	0	0	0
RNU6-78P	0	0	0	0	0	0
RNA5SP426	0	0	0	0	0	0
RNU1-76P	0	0	0	0	0	0
Y_RNA	0	0	0	0	0	0
Y_RNA	0	0	0	0	0	0
SNORD115-9	0	0	0	0	0	0
SNORD56	0	0	0	0	0	0
RNU6-1287P	0	0	0	0	0	0
SNORA52	1.56274989	1.89942253	0	0.11062702	0	0.58893957
RNA5SP188	0	0	0	0	0	0
SNORA42	0	0	0	0	0	0
RNY3P2	0	0	0	0	0	0

RNU6-836P	0	0	0	0	0	0
RNU6-451P	0	0	0	0	0	0
RNU6-1233P	0	0	0	0	0	0
RNU6-924P	0	0	0	0	0	0
Y_RNA	0	0	0	0	0	0
SNORD114-5	0	0	0	0	0	0
Y_RNA	0	0	0	0	0	0
RNU6-1159P	0	0	0	0	0	0
RNA5SP383	0	1.01573397	0	0	0	0.906060876
RNU1-134P	0	0	1.002934331	0	0	0.906060876
RNA5SP491	0	0	0	0	0	0
RNA5SP374	0	0	0	0	0	0
RNU6-428P	0	0	0	0	0	0
RNU6-1260P	0	0	0	0	0	0
SNORA17	0	0	0	0	0	0
RNU6-199P	0	0	0	0	0	0
RNU6-1290P	0	0	0	0	0	0
RN7SKP291	0	0	0	0	0	0
Y_RNA	0	0	0	0	0	0
SNORD115-21	0	0	0	0	0	0
RNU1-47P	0	0	0	0	0	0
RNA5SP71	0	0	0	0	0	0
RNA5SP150	0	0	0	0	0	0
Y_RNA	0	0	0	0	0	0
RNA5SP358	0	0	0	0	0	0
RNA5SP375	0	0	0	0	0	0
RNU1-72P	0	0	0	0	0	0
RNU5F-6P	0	0	0	0	0	0
U3	0	0	0	0	0	0
RNU6-490P	0	0	0	0	0	0
U3	0.97671868	0	0	0	0	0
SNORD50	0	0	0	0	0	0
RNU6-1120P	0	0	0	0	0	0

RNU6-582P	0	0	0	0	0	0
Y_RNA	0	0	0	0	0	0
RNU6-495P	0	0	0	0	0	0
Y_RNA	0	0	0	0	0	0
Y_RNA	0	0	0	0	0	0
Y_RNA	0	0	0	0	0	0
RNU6-942P	0	0	0	0	0	0
RNA5SP452	0	0	0	0	0	0
Y_RNA	0	0	0	0	0	0
RN7SKP149	0	0	0	0	0	0
RNU1-120P	0	0	1.002934331	0	1.007606311	0
RNU6-888P	0	0	0	0	0	0
Y_RNA	0	0	0	0	0	0
RN7SKP90	0	0	0	1.00570022	0	0
Y_RNA	0	0	0	0	0	0
RNU6-330P	0	0	0	0	0	0
RNU6-249P	0	0	0	0	0	0
Y_RNA	0	0	0	0	0	0
RNU1-17P	0	0	0	0	0	0
snoU2_19	0	0	0	0	0	0
Y_RNA	0	0	0	0	0	0
Y_RNA	0	0	0	0	0	0
RNA5SP463	0	0	0	0	0	0
Y_RNA	0	0	0	0	0	0
RNU6-1273P	0	0	0	0	0	0
RNU6-492P	0	0	0	0	0	0
RNU5B-2P	0	0	0	0	0	0
RNA5S10	0	0	0	0	0	0
Y_RNA	0	0	0	0	0	0
Y_RNA	0	0	0	0	0	0
Y_RNA	0	0	0	0	0	0
SNORD114-16	0	0	0	0	0	0
RNU4-44P	0	0	0	0	0	0

RNU6-161P	0	0	0	0	0	0
RNU6-1252P	0	0	0	0	0	0
ACEA_U3	0	0	0	0	0	0
RNA5SP405	0	0	0	0	0	0
RNU1-18P	0	0	0	0	0	0
RNY1P16	0	0	0	0	0	0
SNORD81	0	0	0	0	0	0
Y_RNA	0	0	0	0	0	0
Y_RNA	0	0	0	0	0	0
RN7SKP75	0	0	0	0	0	0
SNORD114-19	0	0	0	0	0	0
RNU6-653P	0	0	0	0	0	0
Y_RNA	0	0	0	0	0	0
RNA5SP443	0	0	0	0	0	0
SNORA2	0	0	0	0	0	0
SNORD115-14	0	0	0	0	0	0
SNORD1B	0	0	0	0	0	0
Y_RNA	0	0	0	0	0	0
RNU6-605P	0	0	0	0	0	0
Y_RNA	0	0	0	0	0	0
SNORD115-19	0	0	0	0	0	0
SNORD115-3	0	0	0	0	0	0
RNU6-797P	0	0	0	0	0	0
RN7SKP243	0	0	0	0	1.007606311	0
SNORA73	1.95343737	0	0	0	0	0
Y_RNA	0	0	0	0	0	0
RNA5SP264	0	0	0	0	0	0
RNU6-486P	0	0	0	0	0	0
VTRNA1-1	0	0	0	0	0	0
RNA5SP145	0	0	0	0	0	0
RNU6-986P	0	0	0	0	0	0
Y_RNA	0	0	0	0	0	0
Y_RNA	0	0	0	0	0	0

RNU6-623P	0	0	0	0	0	0
RNA5SP448	0	0	0	0	0	0
Y_RNA	0	0	0	0	0	0
U8	0	0	0	0	0	0
RNA5SP98	0	0	0	0	0	0
RNU6-642P	0	0	0	0	0	0
RNU6-403P	0	0	0	0	0	0
RNU4-73P	0	0	0	0	0	0
RNA5SP65	0	0	0	0	0	0
RNA5SP32	0	0	0	0	0	0
Y_RNA	0	0	0	0	0	0
Y_RNA	0	0	0	0	0	0
RN7SKP115	0.97671868	0	0	0	0	0
RNU6-812P	0	0	0	0	0	0
Y_RNA	0	0	0	0	0	0
SNORD45	0	0	0	0	0	0
Y_RNA	0	0	0	0	0	0
RN7SKP63	0	0	0	0	0	0
RNA5SP218	0	0	0	0	0	0
Y_RNA	0	0	0	0	0.201521262	0
Y_RNA	0	0	0	0	0	0
RNU4-58P	0	0	0	0	0	0
SNORA30	0	0	0	1.00570022	0	0
RNY4P9	0	0	0	0	0	0
Y_RNA	0	0	0	0	0	0
RNU4-80P	0	0	0	0	0	0
SNORD44	0	0	0	0	0	0
SNORA25	0	0	0	0	0	0
RNA5SP326	0	0	0	0	0	0
SNORD68	0	0	0	0	0	0
Y_RNA	0	0	0	0	0	0
RNU6-433P	0	0	0	0	0	0
SNORA73B	10.7439055	10.1573397	3.008802992	6.03420129	0	21.74546103

SNORD114-31	0	0	0	0	0	0
Y_RNA	0.97671868	0	0	1.00570022	0	0
RN7SKP163	0	0	0	0	0	0
RNU6-181P	0	0	0	0	0	0
RNU6-1167P	0	0	0	0	0	0
RNU6-508P	0	0	0	0	0	0
RNU6-252P	0	0	0	0	0	0
RNU6-251P	0	0	0	0	0	0
RNU6-984P	0	0	0	0	0	0
RNU6-1249P	0	0	0	0	0	0
RNU6-774P	0	0	0	0	0	0
SNORD56	0	0	0	0	0	0
SNORA51	0	0	0	0	0	0
RNA5SP123	0	0	0	0	0	0
Y_RNA	0	0	0	0	0	0
Y_RNA	0	0	0	0	0	0
Y_RNA	0	0	0	0	0	0
SNORA63	0	0	0	0	0	0
RN7SKP77	0	0	0	0	0	0
RNU6-1021P	0	0	0	0	0	0
Y_RNA	0	0	0	0	0	0
RNY1P10	0	0	0	0	0	0
RNU6-778P	0	0	0	0	0	0
Y_RNA	0	0	0	0	0	0
RNU6-544P	0	0	0	0	0	0
SNORD113	0	0	0	0	0	0
RN7SKP231	0	0	0	0	0	0
RNU6-216P	0	0	0	0	0	0
RNU6-23P	0	0	0	0	0	0
RNU5B-1	0	1.01573397	0	1.00570022	2.015212623	0
RN7SKP145	0	0	0	0	0	0
Y_RNA	0	0	0	0	0	0
SNORD115-18	0	0	0	0	0	0

Y_RNA	0	0	0	0	0	0
RNA5SP350	0	0	0	0	0	0
RNU5D-1	0	0	0	0	1.007606311	0
Y_RNA	0	0	0	0	0	0
Y_RNA	0	0	0	0	0	0
Y_RNA	0	0	0	0	0	0
RNU6-1309P	0	0	0	0	0	0
RNU1-19P	0	0	0	0	0	0
Y_RNA	0	0	0	0	0	0
RNU6-238P	0	0	0	0	0	0
RNU1-20P	0	0	0	0	0	0
RNU6-832P	0	0	0	0	0	0
U8	0	0	0	0	0	0
RNU1-21P	0	0	0	0	0	0
RN7SKP211	0	0	0	0	0	0
Y_RNA	0	0	0	0	0	0
RNU1-22P	0	0	0	0	0	0
SNORD74	0	0	0	0	0	0
Y_RNA	0	0	0	0	0	0
Y_RNA	0	0	0	0	0	0
RNY4P27	0	0	0	0	0	0
RNU6-992P	0	0	0	0	0	0
SNORD113-6	0	0	0	0	0	0
RNU6-745P	0	0	0	0	0	0
RNU6-839P	0	0	0	0	0	0
RNU6-697P	0	0	0	0	0	0
RNU6-179P	0	0	0	0	0	0
U3	0	0	0	0	0	0
RNU6-626P	0	0	0	0	0	0
RNA5SP382	0	0	0	0	0	0
RNA5SP197	0	0	0	0	0	0
RNU1-23P	0	0	0	0	0	0
SNORA27	0	0	0	0	0	0

SNORA70	0	0	0	0	0	0
RNA5SP133	0	0	0	0	0	0
Y_RNA	0	0	0	0	0	0
RN7SKP79	0	0	0	0	0	0
RNA5SP263	0	0	0	0	0	0
RNU6-254P	0	0	0	0	0	0
RNA5SP214	0	0	0	0	0	0
RNU6-1147P	0	0	0	0	0	0
Y_RNA	0	0	0	0	0	0
RNU6-529P	0	0	0	0	0	0
RNU6-536P	0	0	0	0	0	0
Y_RNA	0	0	0	0	0	0
RNU6-97P	0	0	0	0	0	0
SNORD35A	0	0	0	0	0	0.815454789
Y_RNA	0	0	0	0	0	0
Y_RNA	0	0	0	0	0	0
RNU6-66P	0	0	0	0	0	0
RNU6-838P	0	0	0	0	0	0
RNU4-32P	0	0	0	0	0	0
RNA5SP199	0	0	0	0	0	0
RNA5SP352	0	0	0	0	0	0
SNORD114-10	0	0	0	0	0	0
RNU6-624P	0	0	0	0	0	0
Y_RNA	0	0	0	0	0	0
Y_RNA	0	0	0	0	0	0
SNORA18	0	0	0	0	0	0
Y_RNA	0	0	0	0	0	0
RNA5SP393	0	0	0	0	0	0
SNORA36	0	0	0	0	0	0
RNU6-527P	0	0	0	0	0	0
RNU1-83P	0	0	0	0	0	0
Y_RNA	0	0	0	0	0	0
RNA5SP483	0	0	0	0	0	0

RNU6-940P	0	0	0	0	0	0
RNU6-1255P	0	0	0	0	0	0
Y_RNA	0	0	0	0	0	0
Y_RNA	0	0	0	0	0	0
RNA5SP215	0	0	0	0	0	0
RN7SKP255	0	0	0	0	0	0
RNA5-8SP7	0	0	0	0	0	0
Y_RNA	0	0	0	0	0	0
U3	0	0	0	0	0	0
SNORA63	0	0	6.509043806	0	0	1.368151923
Y_RNA	0	0	0	0	0	0
RNA5SP391	0	0	0	0	0	0
RNA5SP62	0	0	0	0	0	0
RNU6-183P	0	0	0	0	0	0
Y_RNA	0	0	0	0	0	0
Y_RNA	0	0	0	0	0	0
RNA5SP333	0.97671868	0	0	0	0	0
RNU1-49P	0	0	0	0	0	0
RNU1-105P	0	0	0	0	0	0
RNA5S8	0	0	0	0	0	0
Y_RNA	0	0	0	0	0	0
RNU6-485P	0	0	0	0	0	0
RNU6-1285P	0	0	0	0	0	0
Y_RNA	0	0	0	0	0	0
SNORA71D	0	0	0	0	0	0.906060876
SNORA72	0	0	0	0	0	0
RNU6-833P	0	0	0	0	0	0
RNU6-792P	0	0	0	0	0	0
Y_RNA	0	0	0	0	0	0
RNU6-511P	0	0	0	0	0	0
SNORD113-8	0	0	0	0	0	0
RNU6-666P	0	0	0	0	0	0
RNA5S17	0	0	0	0	0	0

RNU5E-8P	0	0	0	0	0	0
RNU5E-10P	0	0	0	0	0	0
SNORD56	0	0	0	0	0	0
RNU5B-4P	0	0	0	0	0	0
RN7SKP45	0	0	0	0	0	0
RNA5S4	0	0	0	0	0	0
SNORA42	0	0	0	0	0	0
RNU6-618P	0	0	0	0	0	0
RNU6-509P	0	0	0	0	0	0
Y_RNA	0	0	0	0	0	0
Y_RNA	0	0	0	0	0	0
RNU6-698P	0	0	0	0	0	0
SNORA38B	0	0	0	0	0	0
Y_RNA	0	0	0	0	0	0
SNORD115-24	0	0	0	0	0	0
RNU6-1099P	0	0	0	0	0	0
SNORD114-23	0	0	0	0	0	0
RNU6-1169P	0	0	0	0	0	0
RNA5SP74	0	0	0	0	0	0
RNA5SP346	0	0	0	0	0	0
SNORD114-26	0	0	0	0	0	0
SNORA63	0	0	0	1.3878663	0	0
Y_RNA	0	0	0	0	0	0
Y_RNA	0	0	0	0	0	0
SNORD45	0	0	0	0	0	0
RNU6-1155P	0	0	0	0	0	0
Y_RNA	0	0	0	0	0	0
Y_RNA	0	0	0	0	0	0
RNU4-81P	0	0	0	0	0	0
Y_RNA	0	0	0	0	0	0
RNA5-8SP2	0	0	0	0	0	0
Y_RNA	0	0	0	0	0	0
RNU6-799P	0	0	0	0	0	0

RNU6-1066P	0	0	0	0	0	0
RNU6-1339P	0	0	0	0	0	0
RNU6-1085P	0	0	0	0	0	0
Y_RNA	0	0	0	0	0	0
RNU6-1112P	0	0	0	0	0	0
RNU6-1097P	0	0	0	0	0	0
RNU6-727P	0	0	0	0	0	0
SNORD118	0	0	1.464284123	0	0	0
RNA5SP403	0	0	0	0	0	0
RNU6-112P	0	0	0	0	0	0
RNU6-1125P	0	0	0	0	0	0
RN7SKP44	0	0	0	0	0	0
RNA5SP507	0	0	0	0	0	0
RN7SKP162	0	0	0	0	0	0
SNORD115-41	0	0	0	0	0	0
SNORD114-28	0	0	0	0	0	0
RNU6-1017P	0	0	0	0	0	0
RNU6-1244P	0	0	0	0	0	0
Y_RNA	0	0	0	0	0	0
SNORD115-11	0	0	0	0	0	0
RNA5SP322	0	0	0	0	0	0
RN7SKP203	0	0	1.043051704	0	0	0
U3	0	0	0	0	0	0
Y_RNA	0.32231717	0	0	0	0.332510083	0
RNU6-1205P	0	0	0	0	0	0
U8	0	0	0	0	0	0
Y_RNA	0	0	0	0	0	0
SNORD115-5	0	0	0	0	0	0
Y_RNA	0	0	0	0	0	0
Y_RNA	0	0	0	0	0	0
RNA5SP378	0	0	0	0	0	0
RNU6-1214P	0	0	0	0	0	0
Y_RNA	0	0	0	0	0	0

RNU6-957P	0	0	0	0	0	0
RNU6-1209P	0	0	0	0	0	0
RNY4P14	0	0	0	0	0	0
RNA5SP457	0	0	0	0	0	0
RNU6-1331P	0	0	0	0	0	0
SNORD35B	0	0	0	0	0	0.851697224
SNORA33	0	0	0	0	0	1.812121752
SNORA25	0	0	0	0	0	0
RNY4P6	0	0	0	0	0	0
U3	0	0	0	0	0	0
Y_RNA	0	0	0	0	0	0
U3	0	0	0	0	0	0
Y_RNA	0	0	0	0	0	0
RNU6-137P	0	0	0	0	0	0
Y_RNA	0	0	0	0	0	0
Y_RNA	0	0	0	0	0	0
RNU6-1020P	0	0	0	0	0	0
RNU6-525P	0	0	0	0	0	0
RNU6-103P	0	0	0	0	0	0
RNA5SP429	0	0	0	0	0	0
RNU6-288P	0	0	0	0	0	0
RNU6-640P	0	0	0	0	0	0
SNORD115-39	0	0	0	0	0	0
RNU5F-7P	0	0	0	0	0	0
RNU6-829P	0	0	0	0	0	0
RNU6-1284P	0	0	0	0	0	0
Y_RNA	0	0	0	0	0	0
RNU6-414P	0	0	0	0	0	0
Y_RNA	0	0	0	0	0	0
RNA5SP514	0	0	0	0	0	0
Y_RNA	0	0	0	0	0	0
SNORD115-33	0	0	0	0	0	0
RNU6-824P	0	0	0	0	0	0

RNU1-87P	0	0	0	0	0	0
Y_RNA	0	0	0	0	0	0
RNA5SP50	0	0	0	0	0	0
Y_RNA	0	0	0	0	0	0
SNORD114-11	0	0	0	0	0	0
Y_RNA	0	0	0	0	0	0
SNORD114-25	0	0	0	0	0	0
RNA5SP349	0	0	0	0	0	0
Y_RNA	0	0	0	0	0	0
Y_RNA	0	0	0	0	0	0
RNA5SP447	0	0	0	0	0	0
SNORA7	0	0	0	0	0	0
RNU6-1059P	0	0	0	0	0	0
SNORD18A	0	0	0	0	0	0
RNA5S6	0	0	0	0	0	0
RNA5SP325	0	0	0	0	0	0
RNY1P11	0	0	0	0	0	0
Y_RNA	0	0	0	0	0	0
SNORD113-7	0	0	0	0	0	0
Y_RNA	0	0	0	0	0	0
SNORD114-27	0	0	0	0	0	0
RNU5F-3P	0	0	0	0	0	0
SNORD115-37	0	0	0	0	0	0
RNU6-1210P	0	0	0	0	0	0
Y_RNA	0	0	0	0	0	0
RNU6-226P	0	0	0	0	0	0
RNA5SP343	0	0	0	0	0	0
RNA5SP114	0	0	0	0	0	0
Y_RNA	0	0	0	0	0	0
SNORA25	0	0	0	0	0	0
RNU4-92P	0	0	0	0	0	0
SNORA5B	0	0	0	0	0	0
SNORD116-10	0	0	0	0	0	0

Y_RNA	0	0	0	0	0	0
RNU6-1188P	0	0	0	0	0	0
RNU6-912P	0	0	0	0	0	0
RN7SKP174	0	0	0	0	0	0
RN7SKP160	0	0	0	0	0	1.812121752
SNORD18	0	0	0	0	0	0
SNORD115-4	0	0	0	0	0	0
RNU6-263P	0	0	0	0	0	0
RNU6-379P	0	0	0	0	0	0
Y_RNA	0	0	0	0	0	0
RNY3P3	0	0	0	0	0	0
RNA5SP335	0	0	0	0	0	0
Y_RNA	0	0	0	0	0	0
U3	0	0	0	0	0	0
RNU6-674P	0	0	0	0	0	0
Y_RNA	0	0	0	0	0	0
RNU6-873P	0	0	0	0	0	0
SNORD45	0	0	0	0	0	0
RN7SKP93	0	0	0	0	0	0
RNA5SP27	0	0	0	0	0	0
RNA5SP28	0	0	0	0	0	0
RNU6-682P	0	0	0	0	0	0
Y_RNA	0	0	0	0	0	0
RN7SKP103	0	0	1.002934331	0	0	0
RNA5SP260	0	0	0	0	0	0
RNU6-931P	0	0	0	0	0	0
SNORD115-8	0	0	0	0	0	0
RNU6-271P	0	0	0	0	0	0
RNU1-124P	0	0	0	0	0	0
RNU6-194P	0	0	0	0	0	0
SNORD38	0	0	0	0	0	0
Y_RNA	0	0	0	0	0	0
RNA5SP472	0	0	0	0	0	0

RNA5SP161	0	0	0	0	0	0
Y_RNA	0	0	0	0	0	0
RNU1-31P	0	0	0	0	0	0
Y_RNA	0	0	0	0	0	0
Y_RNA	0	0	0	0	0	0
SNORD56	0	0	0	0	0	0
Y_RNA	0	0	0	0	0	0
RNA5SP68	0	0	0	0	0	0
RNU6-236P	0	0	0	0	0	0
SNORD115-16	0	0	0	0	0	0
RNU6-884P	0	0	0	0	0	0
RN7SKP113	0	0	0	0	0	0
RNU6-961P	0	0	0	0	0	0
Y_RNA	0	0	0	0	0	0
Y_RNA	0	0	0	0	0	0
RNU6-478P	0	0	0	0	0	0
RNU6-105P	0	0	0	0	0	0
RN7SKP180	0	0	0	0	0	0
SNORD8	0	0	0	0	0	0
RNA5SP234	0	0	0	0	0	0
Y_RNA	0	0	0	0	0	0
RNU1-65P	0	0	0	0	0	0
RNU6-631P	0	0	0	0	0	0
SNORA80A	0	0	1.002934331	0	0	0
RN7SKP250	0	0	0	0	0	0
RNU4-1	0	1.01573397	0	1.00570022	0	0
RNU6-753P	0	0	0	0	0	0
RNU6-770P	0	0	0	0	0	0
RNU1-130P	0	0	0	0	0	0
SNORD115-28	0	0	0	0	0	0
RNA5SP275	0	0	0	0	0	0
RNU1-32P	0	0	0	0	0	0
SNORD115-6	0	0	0	0	0	0

RNU6-595P	0	0	0	0	0	0
RNU6-1091P	0	0	0	0	0	0
SNORA38	0	0	0	3.01710065	0	0
RNU6-899P	0	0	0	0	0	0
RNU6-1204P	0	0	0	0	0	0
Y_RNA	0	0	0	0	0	0
SNORD114-2	0	0	0	0	0	0
RNU6-324P	0	0	0	0	0	0
Y_RNA	0	0	0	0	0	0
RN7SKP134	0	0	0	0	0	0
SNORD36B	0	0	0	0	0	0
SNORD114-4	0	0	0	0	0	0
Y_RNA	0	0	0	0	0	0
Y_RNA	0	0	0	0	0	0
RNA5SP48	0	0	0	0	0	0
RNU6-82P	0	0	0	0	0	0
Y_RNA	0	0	0	0	0	0
Y_RNA	0	0	0	0	0	0
Y_RNA	0	0	0	0	0	0
RNA5SP499	0	0	0	0	0	0
Y_RNA	0	0	0	0	0	0
Y_RNA	0	0	0	0	0	0
Y_RNA	0	0	0	0	0	0
RN7SKP36	0	0	0	0	0	0
RNU6-457P	0	0	0	0	0	0
RNU6-810P	0	0	0	0	0	0
RNA5SP456	0	0	0	0	0	0
RNA5SP399	0	0	0	0	0	0
Y_RNA	0	0	0	0	0	0
RNU6-340P	0	0	0	0	0	0
RNU6-560P	0	0	0	0	0	0
SNORD14E	0	0	0	0	0	0
RNU6-681P	0	0	0	0	0	0

Y_RNA	0	0	0	0	0	0
Y_RNA	0	0	0	0	0	0
RNU1-146P	0	0	0	0	0	0
RNU6-598P	0	0	0	0	0	0
Y_RNA	0	0	0	0	0	0
RNU4-13P	0	0	0	0	0	0
RNA5SP99	0	0	0	0	0	0
SNORD74	0	0	0	0	0	0
RNU6-944P	0	0	0	0	0	0
RN7SKP245	0	0	0	0	0	0
SNORD74	0	0	0	0	0	0
RNU6-1243P	0	0	0	0	0	0
RNU6-627P	0	0	0	0	0	0
RNU1-42P	0	0	0	0	0	0
RNU6-854P	0	0	0	0	0	0
SNORD46	0	0	0	0	0	0
RNA5SP435	0	0	0	0	0	0
RNU6-553P	0	0	0	0	0	0
Y_RNA	0	0	0	0	0	0
RNU6-1048P	0	0	0	0	0	0
RNU6-960P	0	0	0	0	0	0
RNU6-1090P	0	0	0	0	0	0
RNU6-694P	0	0	0	0	0	0
RNU6-501P	0	0	0	0	0	0
SNORD115-32	0	0	0	0	0	0
Y_RNA	0	0	0	0	0	0
RNU6-801P	0	0	0	0	0	0
SNORA74A	1.95343737	2.03146794	0	1.00570022	1.007606311	0.906060876
RNU6-289P	0	0	0	0	0	0
RN7SKP87	0	0	0	0	0	0
SNORD95	0	0	0	0	0	0
RNU5A-8P	0	0	0	0	0	0
RNU4-87P	0	0	0	0	0	0

RNU1-7P	0	0	0	0	0	0
Y_RNA	0	0	0	0	0	0
Y_RNA	0	0	0	0	0	0
SNORA45A	0	0	0	0	0	0
RNA5SP493	0	0	0	0	0	0
RNU6-819P	0	0	0	0	0	0
SNORD115-30	0	0	0	0	0	0
SNORA25	0	0	0	0	0	0
RNU1-85P	0	0	0	0	0	0
Y_RNA	0	0	0	0	0	0
SNORD74	0	0	0	0	0	0
RNA5SP516	0	0	0	0	0	0
RNU6-270P	0	0	0	0	0	0
SNORA58	0	0	0	0	0	0
Y_RNA	0	0	0	0	0	0
SNORD46	0	0	0	0	0	0
RN7SKP224	0	0	0	0	0	0
Y_RNA	0	0	0	0	0	0
Y_RNA	0	0	0	0	0	0
RNA5SP232	0	0	0	0	0	0
RNU6-280P	0	0	0	0	0	0
RNU6-374P	0	0	0	0	0	0
RNU6-743P	0	0	0	0	0	0
RNY3P11	0	0	0	0	0	0
SNORD74	0	0	0	0	0	0
Y_RNA	0	0	0	0	0	0
RN7SKP107	0	0	0	0	0	0
RNU6-151P	0	0	0	0	0	0
Y_RNA	0	0	0	0	0	0
RNU6-704P	0	0	0	0	0	0
RNU1-96P	0	0	0	0	0	0
Y_RNA	0	0	0	0	0	0
RNA5SP469	0	0	0	0	0	0

SNORD113	0	0	0	0	0	0
RNA5SP242	0	0	0	0	0	0
SNORA38	0	0	0	0	0	0
RNU6-268P	0	0	0	0	0	0
Y_RNA	0	0	0	0	0	0
Y_RNA	0	0	0	0	0	0
RNU6-668P	0	0	0	0	0	0
RNA5SP336	0	0	0	0	0	0
RNU6-461P	0	0	0	0	0	0
RN7SKP280	0	0	0	0	0	0
RNU4-84P	0	0	0	0	0	0
Y_RNA	0	0	0	0	0	0
Y_RNA	0	0	0	0	0	0
Y_RNA	0	0	0	0	0	0
RNU4-51P	0	0	0	0	0	0
RNU6-188P	0	0	0	0	0	0
RN7SKP214	0	0	0	0	0	0
Y_RNA	0	0	0	0	0	0
RNU6-173P	0	0	0	0	0	0
RNA5SP394	0	0	0	0	0	0
Y_RNA	0	0	0	0	0	0
RNU6-266P	0	0	0	0	0	0
RNA5SP387	0	0	0	0	0	0
RNU6-366P	0	0	0	0	0	0
RNY1	0	0	0	2.01140043	0	0
Y_RNA	0	0	0	0	0	0
RNU6-11P	0	0	0	0	0	0
RNA5SP245	0	0	0	0	0	0
RNU6-647P	0	0	0	0	0	0
Y_RNA	0	0	0	0	0	0
Y_RNA	0	0	0	0	0	0
RNU1-33P	0	0	0	0	0	0
RNY1P12	0	0	0	0	0	0

RNU6-773P	0	0	0	0	0	0
SNORA58	0	0	0	0	0	0
SNORA4	0	0	0	0	0	0
Y_RNA	0	0	0	0	0	0
RNU6-777P	0	0	0	0	0	0
RNU6-353P	0	0	0	0	0	0
RN7SKP128	0	0	0	0	0	0
RNVU1-8	0	0	0	0	0	0
SNORD115-42	0	0	0	0	0	0
RNA5SP175	0	0	0	0	0	0
RNA5SP42	0	0	0	0	0	0
SNORD56	0	0	0	0	0	0
RNU6-371P	0	0	0	0	0	0
RNU1-24P	0	0	0	0	0	0
SNORA62	0	0	0	0	0	0
Y_RNA	0	0	0	0	0	0
RN7SKP10	0	0	0	0	0	0
RNU6-454P	0	0	0	0	0	0
RNU4-36P	0	0	0	0	0	0
RNU6-1229P	0	0	0	0	0	0
RNA5SP368	0	0	0	0	0	0
RNU1-132P	0	0	0	0	0	0
RNU6-853P	0	0	0	0	0	0
Y_RNA	0	0	0	0	0	0
RNU6-1322P	0	0	0	0	0	0
RNU6-115P	0	0	0	0	0	0
RNU6-670P	0	0	0	0	0	0
RNVU1-3	0	0	0	0	0	0
RNU4-68P	0	0	0	0	0	0
RNA5SP202	0	0	0	0	0	0
RNU4-33P	0	0	0	0	0	0
Y_RNA	0	0	0	0	0	0
RNU6-879P	0	0	0	0	0	0

RN7SKP118	0	0	0	0	0	0
Y_RNA	0	0	0	0	0	0
Y_RNA	0	0	0	0	0	0
Y_RNA	0	0	0	0	0	0
SNORD42	0	0	0	0	0	0
RNA5SP139	0	0	0	0	0	0
Y_RNA	0	0	0	0	0	0
Y_RNA	0	1.01573397	0	0	1.007606311	0
Y_RNA	0	0	0	0	0	0
RNU4-40P	0	0	1.002934331	0	0	0
RNU6-1305P	0	0	0	0	0	0
RNA5-8SP3	0	0	0	0	0	0
Y_RNA	0	0	0	0	0	0
SNORA63	0.97671868	0	0	0	0	0
RNU4-50P	0	0	0	0	0	0
Y_RNA	0	0	0	0	0	0
SNORD114-9	0	0	0	0	0	0
RNU6-978P	0	0	0	0	0	0
RNY1P1	0	0	0	0	0	0
RNU6-654P	0	0	0	0	0	0
SNORA25	0	0	0	0	0	0
SNORD114-13	0	0	0	0	0	0
RNU4-10P	0	0	0	0	0	0
RNU6-990P	0	0	0	0	0	0
RNU6-1075P	0	0	0	0	0	0
SNORD114-6	0	0	0	0	0	0
SNORD73	0	0	0	0	0	0
RNU6-755P	0	0	0	0	0	0
RNU1-112P	0	0	0	0	0	0
RNU6-776P	0	0	0	0	0	0
RNA5SP192	0	0	0	0	0	0
Y_RNA	0	0	0	0	0	0
Y_RNA	0	0	0	0	0	0

Y_RNA	0	0	0	0	0	0
5S_rRNA	0	0	0	0	0	0
RN7SKP171	0	0	0	0	0	0
RNU6-1047P	0	0	0	0	0	0
RN7SKP76	0	0	0	0	0	0
RNU1-34P	0	0	0	0	0	0
RNU6-153P	0	0	0	0	0	0
RNU6-1019P	0	0	0	0	0	0
RNU4-41P	0	0	0	0	0	0
Y_RNA	0	0	0	0	0	0
RNU6-1164P	0	0	0	0	0	0
SNORD115-27	0	0	0	0	0	0
RNA5SP130	0	0	0	0	0	0
SNORA65	0	0	0	0	0	0.906060876
RNU6-512P	0	0	0	0	0	0
Y_RNA	0	0	0	0	0	0
Y_RNA	0	0	0	0	0	0
RNA5SP72	0	0	0	0	0	0
Y_RNA	0	0	0	0	0	0
RN7SKP227	0	1.01573397	0	0	0	0
SNORA7	0	0	0	0	0	0
RNU4-59P	0	0	0	0	0	0
SNORD114-30	0	0	0	0	0	0
RNA5S9	0	0	0	0	0	0
RNU6-361P	0	0	0	0	0	0
RNA5SP142	0	0	0	0	0	0
SNORD115-22	0	0	0	0	0	0
U3	0	0	0	0	0	0
SNORD32B	0	0	0	0	0	0
SNORD115-23	0	0	0	0	0	0
Y_RNA	0	0	0	0	0	0
Y_RNA	0	0	0	0	0	0
RNU6-421P	0	0	0	0	0	0

RNU4-38P	0	0	1.002934331	0	0	0.906060876
Y_RNA	0	0	0	0	0	0
U3	0	0	0	0	0	0
RNA5SP69	0	0	0	0	0	0
RNU105B	0	0	0	0	0	0
Y_RNA	0	0	0	0	0	0
Y_RNA	0	0	0	0	0	0
RNA5S14	0	0	0	0	0	0
RNA5SP500	0	0	0	0	0	0
RN7SKP193	0	0	0	0	0	0
RNA5SP433	0	0	0	0	0	0
Y_RNA	0	0	0	0	0	0
RN7SKP37	0	0	0	0	0	0
Y_RNA	0	0	0	0	0	0
RNU6-522P	0	0	0	0	1.007606311	0
U3	0	0	0	0	0	0
Y_RNA	0	0	0	0	0	0
Y_RNA	0	0	0	0	0	0
RNU6-1251P	0	0	0	0	0	0
SNORA70	0	0	0	0	0	0
RNY4P23	0	0	0	0	0	0
RNU4-76P	0	0	0	0	0	0
RNU6-558P	0	0	0	0	0	0
SNORA32	0	0	0	0	0	0
RNU6-1163P	0	0	0	0	0	0
SNORA68	0.97671868	0	0	0	0	0
RNU6-1141P	0	0	0	0	0	0
RNU6-1078P	0	0	0	0	0	0
SNORA71	0	0	0	0	0	0
RNA5SP140	0	0	0	0	0	0
RN7SKP257	0	0	0	0	0	0
U8	0	0	0	0	0	0
SNORD14B	0	0	0	0	0	0

Y_RNA	0	0	0	0	0	0
SNORA68	0	0	0	0	0	0
snoZ40	0	0	0	0	0	0
Y_RNA	0	0	0	0	0	0
RNA5SP141	0	0	0	0	1.007606311	0
RNA5SP204	0	0	0	0	0	0
RNA5SP512	0	0	0	0	0	0
Y_RNA	0	0	0	0	0	0
RN7SKP246	0	0	0	0	0	0
Y_RNA	0	0	0	0	0	0
RN7SKP71	0	0	0	0	0	0
RNU6-1277P	0	0	0	0	0	0
Y_RNA	0	0	0	0	0	0
RNU6-335P	0	0	0	0	0	0
RNU4-24P	0	0	0	0	0	0
RNU6-660P	0	0	0	0	0	0
RNU4-67P	0	0	0	0	0	0
RNA5SP515	0	0	0	0	0	0
RNU6-646P	0	0	0	0	0	0
Y_RNA	0	0	0	0	0	0
RNU6-309P	0	0	0	0	0	0
RNU6-1082P	0	0	0	0	0	0
RNA5SP509	0	0	0	0	0	0
SNORA63	0	0	0	0	0	0
Y_RNA	0	0	0	0	0	0
RNU6-1317P	0	0	0	0	0	0
SNORA55	0	0	0	0	0	0
RNU4-4P	0	0	0	0	0	0
Y_RNA	0	0	0	0	0	0
SNORA51	0	0	0	0	0	0
Y_RNA	0	0	0	0	0	0
SNORA16	0	0	0	0	0	0
RNA5SP379	0	0	0	0	0	0

RNY4P7	0	0	0	0	0	0
RNU6-164P	0	0	0	0	0	0
RNA5SP353	0	0	0	0	0	0
SNORD115-17	0	0	0	0	0	0
Y_RNA	0	0	0	0	0	0
SNORD45B	0	0	0	0	0	0
Y_RNA	0	0	0	0	0	0
RNU4-75P	0	0	0	0	0	0
RNA5SP78	0	0	0	0	0	0
RNU1-141P	0	0	0	0	0	0
RN7SKP275	0	0	0	0	0	0
Y_RNA	0	0	0	0	0	0
RNU6-312P	0	0	0	0	0	0
SNORD113	0	0	0	0	0	0
Y_RNA	0	0	0	0	0	0
SNORD74	0	0	0	0	0	0
RN7SKP217	0	0	0	0	0	0
Y_RNA	0	0	0	0	0	0
SNORA71C	0.97671868	0	0	0	1.007606311	0.906060876
SNORA51	0	0	0	0	0	0
RNU6-707P	0	0	0	0	0	0
RNA5SP513	0	0	0	0	0	0
RNU6-645P	0	0	0	0	0	0
RNA5SP205	0	0	0	0	0	0
RNU6-450P	0	0	0	0	0	0
RNA5SP478	0	0	0	0	0	0
Y_RNA	0	0	0	0	0	0
RNA5SP194	0	0	0	0	0	0
RN7SKP237	0	0	0	0	0	0
Y_RNA	0	0	0	0	0	0
SNORA1	0	0	0	0	0	0
SNORA62	0	0	0	0	0	0.906060876
SNORA16B	0	0	0	0	0	0

RNU4-85P	0	0	0	0	0	0
Y_RNA	0	0	0	0	0	0
Y_RNA	0	0	0	0	0	0
Y_RNA	0	0	0	0	0	0
RNU6-726P	0	0	0	0	0	0
Y_RNA	0	0	0	0	0	0
Y_RNA	0	0	0	0	0	0
SNORD114-15	0	0	0	0	0	0
RNVU1-6	0	0	0	0	0	0
RNU6-973P	0	0	0	0	0	0
Y_RNA	0	0	0	0	0	0
RN7SKP50	1.95343737	0	0	0	0	0
Y_RNA	0	0	0	0	0	0
Y_RNA	0	0	0	0	0	0
RNA5SP510	0	0	0	0	0	0
SNORD114-17	0	0	0	0	0	0
RNU4-56P	0	0	0	0	0	0
Y_RNA	0	0	0	0	0	0
RNU1-93P	0	0	0	0	0	0
RNU6-343P	0	0	0	0	0	0
RN7SKP78	1.95343737	1.01573397	0	1.00570022	0	0
RN7SKP191	0	0	0	0	0	0
Y_RNA	0	0	0	0	0	0
RNU6-593P	0	0	0	0	0	0
RNA5S2	0	0	0	0	0	0
RNU6-99P	0	0	0	0	0	0
snoU2_19	0	0	0	0	0	0
RNA5SP517	0	0	0	0	0	0
RNA5SP132	0	0	0	0	0	0
Y_RNA	0	0	0	0	0	0
RNU6-219P	0	0	0	0	0	0
RN7SKP124	0	0	0	0	0	0
Y_RNA	0	0	0	0	0	0

RNU6-740P	0	0	0	0	0	0
RNU4-16P	0	0	0	0	0	0
RNU4-42P	0	0	0	0	0	0
RNU4-28P	0	0	0	0	0	0
RNA5SP84	0	0	0	0	0	0
RN7SKP15	0	0	0	0	0	0
RNU6-200P	0	0	0	0	0	0
RNU1-91P	0	1.01573397	0	0	0	0
RNA5SP505	0	0	0	0	0	0
SNORA67	0	0	0	0	0	0
RNA5SP51	0	0	0	0	0	0
RNU6-621P	0	0	0	0	0	0
RNU6-923P	0	0	0	0	0	0
Y_RNA	0	0	0	0	0	0
Y_RNA	0	0	0	0	0	0
RNU4-7P	0	0	0	0	0	0
Y_RNA	0	0	0	0	0	0
SNORD115-48	0	0	0	0	0	0
Y_RNA	0	0	0	0	0	0
RNY4P16	0	0	0	0	0	0
RN7SKP28	0	0	0	0	0	0
RNU6-1187P	0	0	0	0	0	0
RNU6-865P	0	0	0	0	0	0
SNORA14A	0	0	0	0	0	0
Y_RNA	0	0	0	0	0	0
RNU4-91P	0	0	0	0	0	0
RNY4P34	0	0	0	0	0	0
RNU6-7	0	0	0	0	0	0
RNU6-283P	0	0	0	0	0	0
RNU12-2P	0	0	0	0	1.007606311	0
SNORA20	0	0	0	0	0	0
RNU6-60P	0	0	0	0	0	0
Y_RNA	0	0	0	0	0	0

RN7SKP6	0	0	0	0	0	0
SNORD74	0	0	0	0	0	0
Y_RNA	0	0	0	0	0	0
RNA5SP90	0	0	0	0	0	0
SNORD113-4	0	0	0	0	0	0
U3	0	0	0	0	0	0
SNORD32A	0	0	0	0	0	0
Y_RNA	0	0	0	0	0	0
SNORD115-15	0	0	0	0	0	0
Y_RNA	0	0	0	0	0	0
Y_RNA	0	0	0	0	0	0
RN7SKP239	0.97671868	1.01573397	11.03227764	1.00570022	1.007606311	7.248487009
RNU6-1107P	0	0	0	0	0	0
SNORD114-29	0	0	0	0	0	0
RNY1P2	0	0	0	0	0	0
RNA5SP334	0.97671868	0	0	0	0	0
RNU1-59P	0	0	0	0	0	0
SNORD113-3	0	0	0	0	0	0
SNORA25	0	0	0	0	0	0
RNA5SP396	0	0	0	0	0	0
RNU6-818P	0	0	0	0	0	0
RNA5SP359	0	0	0	0	0	0
RNU6-686P	0	0	0	0	0	0
SNORD113	0	0	0	0	0	0
RNU6-1303P	0	0	0	0	0	0
RNA5SP125	0	0	0	0	0	0
RN7SKP133	0	0	0	0	0	0
Y_RNA	0	0	0	0	0	0
Y_RNA	0	0	0	0	0	0
RNU6-304P	0	0	0	0	0	0
RNA5SP173	0	0	0	0	0	0
RNA5SP479	0	0	0	0	0	0
SNORA43	0	0	0	0	0	0

RNA5SP160	0	0	0	0	0	0
RNU1-133P	0	0	0	0	0	0
Y_RNA	0	0	0	0	0	0
RNU6-563P	0	0	0	0	0	0
RNU6-34P	0	0	0	0	0	0
RNU6-828P	0	0	0	0	0	0
RNU6-534P	0	0	0	0	0	0
Y_RNA	0	0	0	0	0	0
RNU6-119P	0	0	0	0	0	0
SNORD52	0	0	0	0	0	0
Y_RNA	0	0	0	0	0	0
RN7SKP55	0	0	0	0	0	0
RNU6-336P	0	0	0	0	0	0
RNA5SP267	0	0	0	0	0	0
RNA5SP302	0	0	0	0	0	0
RNU6-384P	0	0	0	0	0	0
SNORA5C	0.97671868	0	0	0	0	0
Y_RNA	0	0	0	0	0	0
RNU6-1325P	0	0	0	0	0	0
Y_RNA	0	0	0	0	0	0
RNU6-275P	0	0	0	0	0	0
RN7SKP226	0	0	0	0	0	0
SNORD117	0	0	0	0	0	0
Y_RNA	0	0	0	0	0	0
Y_RNA	0	0	0	0	0	0
RNU6-1071P	0	0	0	0	0	0
RNA5SP189	0	0	0	0	0	0
SNORA63	0	0	0	0	0	0
RN7SKP9	0.97671868	0	2.005868661	0	0	0
RN7SKP130	0	0	0	0	0	0
RNU6-392P	0	0	0	0	0	0
Y_RNA	0	0	0	0	0	0
RNU5E-4P	0	0	0	0	0	0

RNU6-1180P	0	0	0	0	0	0
RNU4-8P	0	0	0	0	0	0
SNORA27	0	0	0	0	0	0
U8	0	0	0	0	0	0
U8	0	0	0	0	0	0
SNORA71	0	0	0	0	0	0
RNA5SP488	0	0	0	0	0	0
RNU6-915P	0	0	0	0	0	0
RNU6-526P	0	0	0	0	0	0
SNORA73	0	0	0	0	0	0
RNY4P17	0	0	0	0	0	0
Y_RNA	0	0	0	0	0	0
RNU4-9P	0	0	0	0	0	0
RNA5SP149	0	0	0	0	0	0
SNORD48	0	0	0	0	0	0
RNU6-1131P	0	0	0	0	0	0
RNU6-867P	0	0	0	0	0	0
SNORA33	0	0	0	0	0	0
Y_RNA	0	0	0	0	0	0
SNORD115-1	0	0	0	0	0	0
SNORD114-3	0	0	0	0	0	0
Y_RNA	0	0	0	0	0	0
RNA5SP166	0	0	0	0	0	0
SNORD31	0	0	0	0	0	0
Y_RNA	0	0	0	0	0	0
RNU6-702P	0	0	0	0	0	0
RNA5SP331	0	0	0	0	0	0
Y_RNA	0	0	0	0	0	0
RNA5SP298	0	0	0	0	0	0
SNORA51	0	0	0	0	0	0
Y_RNA	0	0	0	0	0	0
Y_RNA	0	0	0	0	0	0
RNU6-294P	0	0	0	0	0	0

RN7SKP178	0	0	0	0	0	0
RNA5SP97	0	0	0	0	0	0
Y_RNA	0	0	0	0	0	0
snoU2-30	0	0	0	0	0	0
Y_RNA	0	0	0	0	0	0
Y_RNA	0	0	0	0	0	0
Y_RNA	0	0	0	0	0	0
RNU6-967P	0	0	0	0	0	0
RN7SKP153	0	0	0	0	0	0
SNORA72	0	0	0	0	0	0
SNORD114-24	0	0	0	0	0	0
RNY1P13	0	0	0	0	0	0
RN7SKP48	0	0	0	0	0	0
SNORD115-38	0	0	0	0	0	0
RNU6-590P	0	0	0	0	0	0
RNU1-140P	0	0	0	0	0	0
RN7SKP189	0	0	0	0	0	0
Y_RNA	0	0	0	0	0	0
Y_RNA	0	0	0	0	0	0
RNU6-1022P	0	0	0	0	0	0
RNA5SP442	0	0	0	0	0	0
RNU1-43P	0	0	0	0	0	0
RNA5SP485	0	0	0	0	0	0
RNA5S15	0	0	0	0	0	0
RNA5SP172	0	0	0	0	0	0
Y_RNA	0	0	0	0	0	0
Y_RNA	0	0	0	0	0	0
RNA5SP224	0	0	0	0	0	0
RNA5SP363	0	0	0	0	0	0
SNORD115-10	0	0	0	0	0	0
SNORA72	0	0	0	0	0	0
SNORA70	0	0	0	0	0	0
SNORD113-9	0	0	0	0	0	0

RNU6-673P	0	0	0	0	0	0
RNY3P1	0	0	0	0	0	0
SNORA25	0	0	0	0	0	0
Y_RNA	0	0	0	0	0	0
RNA5SP126	0	0	0	0	0	0
Y_RNA	0	0	0	0	0	0
RNA5-8SP4	0	0	0	0	0	0
RN7SKP22	0	0	0	0	0	0
RNA5SP138	0	0	0	0	0	0
SNORD115-20	0	0	0	0	0	0
SNORA62	0	0	0	0	0	0
Y_RNA	0	0	0	0	0	0
Y_RNA	0	0	0	0	0	0
Y_RNA	0	0	0	1.00570022	0	0
Y_RNA	0	0	0	0	0	0
SNORD115-35	0	0	0	0	0	0
SNORA23	0.97671868	0	1.002934331	0	1.007606311	0
RNA5SP428	0	0	0	0	0	0
RNU1-36P	0	0	0	0	0	0
Y_RNA	0	0	0	0	0	0
Y_RNA	0	0	0	0	0	0
Y_RNA	0	0	0	0	0	0
RNU6-619P	0	0	0	0	0	0
RNU6-806P	0	0	0	0	0	0
Y_RNA	0	0	0	0	0	0
Y_RNA	0	0	0	0	0	0
SNORD81	0	0	0	0	0	0
RNU6-934P	0	0	0	0	0	0
RNU6-1240P	0	0	0	0	0	0
RNU6-1134P	0	0	0	0	0	0
Y_RNA	0	0	0	0	0	0
RNU6-580P	0	0	0	0	0	0
SNORD38A	0	0	0	0	0	0

RNU6-399P	0	0	0	0	0	0
Y_RNA	0	0	0	0	0	0
RNU6-215P	0	0	0	0	0	0
Y_RNA	0	0	0	0	0	0
RNA5SP223	0	0	0	0	0	0
Y_RNA	0	0	0	0	0	0
RNA5SP324	0	0	0	0	0	0
SNORD114-20	0	0	0	0	0	0
RNU6-391P	0	0	0	0	0	0
RNU6-382P	0	0	0	0	0	0
RNA5SP152	0	0	0	0	0	0
RNA5SP19	0	0	0	0	0	0
RN7SKP80	0.99625306	0	11.52371546	0	2.105897191	2.745364455
SNORA1	0	0	0	0	0	0
RNA5SP455	0	0	0	0	0	0
RNA5SP79	0	0	0	0	0	0
RNU4-66P	0	0	0	0	0	0
RNU6-1175P	0	0	0	0	0	0
Y_RNA	0	0	0	0	0	0
RNU6-715P	0	0	0	0	0	0
Y_RNA	0	0	0	0	0	0
Y_RNA	0	0	0	0	0	0
RNU6-1280P	0	0	0	0	0	0
RNU6-290P	0	0	0	0	0	0
RNU6-1306P	0	0	0	0	0	0
RNA5SP190	0	0	0	0	0	0
SNORD58C	0	0	0	0	0	0
RNU6-1172P	0	0	0	0	0	0
RNU6-234P	0	0	0	0	0	0
Y_RNA	0	0	0	0	0	0
RNU4-12P	0	0	0	0	0	0
VTRNA1-2	0	0	0	0	0	0
RNU6-690P	0	0	0	0	0	0

RNU6-302P	0	0	0	0	0	0
RNY4P3	0	0	0	0	0	0
RNU1-100P	0	0	0	0	0	0
Y_RNA	0	0	0	0	0	0
Y_RNA	0	0	0	0	0	0
SNORD114-18	0	0	0	0	0	0
Y_RNA	0	0	0	0	0	0
Y_RNA	0	0	0	0	0	0
RNA5SP329	0	0	0	0	0	0
RNU6-407P	0	0	0	0	0	0
RNY4P28	0	0	0	0	0	0
RNU4-11P	0	0	0	0	0	0
RNU6-742P	0	0	0	0	0	0
RNU5E-7P	0	0	0	0	0	0
RNA5SP117	0	0	0	0	0	0
RNU1-114P	0	0	0	0	0	0
Y_RNA	0	0	0	0	0	0
RNU6-1327P	0	0	0	0	0	0
RNA5-8SP5	0	0	0	0	0	0
RNA5SP128	0	0	0	0	0	0
Y_RNA	0	0	0	0	0	0
Y_RNA	0	0	0	0	0	0
SNORA4	0	0	0	0	0	0
RNU6-1283P	0	0	0	0	0	0
RNU6-497P	0	0	0	0	0	0
RNA5SP355	0	0	0	0	0	0
SNORD115-31	0	0	0	0	0	0
SNORA30	0	0	0	0	0	0
Y_RNA	0	0	0	0	0	0
SNORD113-1	0	0	0	0	0	0
RNA5SP427	0	0	0	0	0	0
Y_RNA	0	0	0	0	0	0
RN7SK	136.721081	199.083859	391.6157681	263.493456	152.0578684	126.8213408

RNU1-115P	0	0	0	0	0	0
Y_RNA	0	0	0	0	0	0
RNU6-1034P	0	0	0	0	0	0
RNU6-169P	0	0	0	0	0	0
Y_RNA	0	0	0	0	0	0
RNU1-51P	0	0	0	1.00570022	0	0
SNORA17	0	0	0	0	0	0
RN7SKP47	0	0	0	0	0	0
Y_RNA	0	0	0	0	0	0
Y_RNA	0	0	0	0	0	0
RNA5SP240	0	0	0	0	0	0
RNU6-282P	0	0	0	0	0	0
RNU6-1138P	0	0	0	0	0	0
SNORA9	0	0	0	0	0	0
U3	0	0	0	0	0	0
RNU6-53P	0	0	0	0	0	0
RNU6-396P	0	0	0	0	0	0
RNU6-737P	0	0	0	0	0	0
RN7SKP186	0	0	0	0	0	0
RNU6-1276P	0	0	0	0	0	0
RNU6-728P	0	0	0	0	0	0
RNA5SP85	0	0	0	0	0	0
RNU5E-5P	0	0	0	0	0	0
RNU6-443P	0	0	0	0	0	0
Y_RNA	0	0	0	0	0	0
SNORD14C	0	0	0	0	0	0
Y_RNA	0	0	0	0	0	0
Y_RNA	0	0	0	0	0	0
RNA5S16	0	0	0	0	0	0
RNU6-1318P	0	0	0	0	0	0
RN7SKP69	0	0	0	0	0	0
SNORD115-44	0	0	0	0	0	0
RNA5SP22	0	0	0	0	0	0

RNA5SP77	0	0	0	0	0	0
RNU6-596P	0	0	0	0	0	0
U3	0	0	0	0	0	0
U8	0	0	0	0	0	0
SNORD114-12	0	0	0	0	0	0
Y_RNA	0	0	0	0	0	0
Y_RNA	0	0	0	0	0	0
SNORD51	0	0	0	0	0	0
Y_RNA	0	0	0	0	0	0
Y_RNA	0	0	0	0	0	0
RNA5SP235	0	0	0	0	0	0
SNORA33	0	0	0	0	0	0
RNU6-117P	0	0	0	0	0	0
RNA5SP37	0	0	0	0	0	0
SNORD114-22	0	0	0	0	0	0
RNU6-1335P	0	0	0	0	0	0
RNY3P12	0	0	0	0	0	0
RNU6-487P	0	0	0	0	0	0
RNU6-1130P	0	0	0	0	0	0
Y_RNA	0	0	0	0	0	0
RNU6-996P	0	0	0	0	0	0
Y_RNA	0	0	0	0	0	0
Y_RNA	0	0	0	0	0	0
RNU1-64P	0	0	0	0	0	0
SNORD6	0	0	0	0	0	0
RNU1-84P	0	0	0	0	0	0
Y_RNA	0	0	0	0	0	0
RNA5SP131	0	0	0	0	0	0
RNA5SP366	0	0	0	0	0	0
RNU6-609P	0	0	0	0	0	0
RNA5SP167	0	0	0	0	0	0
Y_RNA	0	0	0	0	0	0
RNA5SP238	0	0	0	0	0	0

SNORD50	0	0	0	0	0	0
RNU6-359P	0	0	0	0	0	0
RNU6-8	0	0	0	0	0	0
RNU4-45P	0	0	0	0	0	0
RNU6-1051P	0	0	0	0	0	0
SNORA2	0	0	0	0	0	0.906060876
RN7SKP208	0	0	0	0	0	0
Y_RNA	0	0	0	0	0	0
RNU1-16P	0	0	0	0	0	0
RNU6-326P	0	0	0	0	0	0
RN7SKP204	0	0	0	0	0	0
RNY3	0.32231717	0	0	0.50285011	0.332510083	0
RNA5SP277	0	0	0	0	0	0
Y_RNA	0	0	0	0	0	0
RNU6-652P	0	0	0	0	0	0
RN7SKP249	0	0	0	0	0	0
Y_RNA	0	0	0	0	0	0
SNORA62	0	0	0	0	0	0
Y_RNA	0	0	0	0	0	0
SNORD115-43	0	0	0	0	0	0
SNORA62	0	0	0	0	0	0.906060876
SNORA25	0	0	0	0	0	0
SNORA70	0	0	0	0	0	0
RNU1-55P	0	0	0	0	0	0
Y_RNA	0	0	0	0	0	0
RNA5SP279	0	0	0	0	0	0
Y_RNA	0	0	0	0	0	0
RNA5SP211	0	0	0	0	0	0
Y_RNA	0	0	0	0	0	0
SNORA70	0	0	0	0	0	0
RN7SKP292	0	0	0	1.00570022	0	0.906060876
RN7SKP1	0	0	0	0	0	0
RNU6-61P	0	0	0	0	0	0

Y_RNA	0	0	0	0	0	0
SNORD82	0	0	0	0	0	0
Y_RNA	0	0	0	0	0	0
RN7SKP187	0	0	0	0	0	0
Y_RNA	0	0	0	0	0	0
RNU1-122P	0	0	0	0	0	0
Y_RNA	0	0	0	0	0	0
RNA5SP259	0	0	0	0	0	0
RNY3P13	0	0	0	0	0	0
Y_RNA	0.97671868	0	0	0	0	0
RN7SKP269	0	2.03146794	2.005868661	0	0	0.906060876
Y_RNA	0	0	0	0	0	0
RNA5SP30	0	0	0	0	0	0
RNU6-827P	0	0	0	0	0	0
RNU6-744P	0	0	0	0	0	0
RNU6-108P	0	0	0	0	0	0
RNU4-48P	0	0	0	0	0	0
RNA5SP88	0	0	0	0	0	0
RNU6-438P	0	0	0	0	0	0
RNU6-54P	0	0	0	0	0	0
SNORA4	0	0	0	0	0	0
Y_RNA	0	0	0	0	0	0
SNORD42	0	0	0	0	0	0
RNY4P10	0	0	0	0	0	0
RNU5E-6P	1.94367018	0	0	0	0	0.869818441
RNU6-669P	0	0	0	0	0	0
SNORA63	0	0	0	0	0	0
Y_RNA	0	0	0	0	0	0
Y_RNA	0	0	0	0	0	0
RNU4-17P	0	0	0	0	0	0
Y_RNA	0	0	0	0	0	0
Y_RNA	0	0	0	0	0	0
RNU4-20P	0	0	0	0	0	0

RNA5SP248	0.97671868	0	0	0	0	0
RN7SKP81	0	0	0	0	0	0
RNA5SP283	0	0	0	0	0	0
Y_RNA	0	0	0	0	0	0
RNU6-81P	0	0	0	0	0	0
SNORD14	0	0	0	0	0	0
RNU6-499P	0	0	0	0	0	0
RNU6-597P	0	0	0	0	0	0
RNU6-716P	0	0	0	0	0	0
Y_RNA	0	0	0	0	0	0
RNU6-898P	0	0	0	0	0	0
SNORD116	0	0	0	0	0	0
SNORD115-36	0	0	0	0	0	0
RNA5SP151	0	0	0	0	0	0
SNORD34	0	0	0	0	0	0
Y_RNA	0	0	0	0	0	0
RN7SKP230	0	0	0	0	0	0
RNU6-805P	0	0	0	0	0	0
Y_RNA	0	0	0	0	0	0
VTRNA1-3	0	0	0	0	0	0
SNORA64	0	0	0	0	0	0
RNA5S7	0	0	0	0	0	0
Y_RNA	0	0	0	0	0	0
Y_RNA	0	0	0	0	0	0
RNA5S13	0	0	0	0	0	0
RNU6-650P	0	0	0	0	0	0
SNORD18B	0	0	0	0	0	0
RNU6-395P	0	0	0	0	0	0
Y_RNA	0	0	0	0	0	0
RNU6-1329P	0	0	0	0	0	0
Y_RNA	0	0	0	0	0	0
U8	0	0	0	0	0	0
RNU4-2	2.93015605	0	2.005868661	2.01140043	0	4.530304381

Y_RNA	0	0	0	0	0	0
MIR539	0	0	0	0	0	0
MIR421	0	0	0	0	0	0
MIR146B	0	0	0	0	0	0
MIR582	0	0	0	0	0	0
MIR488	0	0	0	0	0	0
RP11-47J17.2	0	0	0	0	0	0
RP5-961K14.2	0	0	0	0	0	0
RP11-560A15.3	0	0	0	0	0	0
RP11-498P14.5	3.90687474	22.3461474	24.07042394	30.1710065	6.045637868	16.30909577
CTA-221G9.12	0	0	17.04988362	12.0684026	0	4.530304381
Metazoa_SRP	0.97671868	0	1.002934331	0	1.007606311	1.812121752
RP11-98D18.9	5.8603121	6.09440383	60.17605984	184.043139	98.74541851	57.98789608
RP11-375H19.2	0	0	0	0	0	0
C9orf41-AS1	1.95343737	0	0	0	1.007606311	0
RP11-277A4.4	0	0	0	0	3.022818934	0
ZNF525	163.834792	148.29716	427.3603477	498.042861	596.2812629	590.7426307
AC012358.7	7.81374947	15.2360096	12.03521197	2.01140043	7.053244179	7.248487009
RP11-25I9.3	1.95343737	1.01573397	0	1.00570022	2.015212623	0.906060876
AC006019.3	0	0	0	0	0	0
LINC01562	0	1.01573397	0	1.00570022	0	0
RP3-337H4.8	0	0	0	0	0	0.906060876
AC012454.4	0	0	0	0	0	0
RP11-370F5.4	0	0	0	4.02280086	0	0.906060876
LINC01317	0	0	0	0	0	0
AC074019.2	0	0	0	0	0	0
CTD-2026K11.6	45.9057781	26.4090833	7.020540315	17.0969037	21.15973254	11.77879139
AC015969.3	0	0	0	0	0	0
RP11-217O12.1	0	0	0	0	0	0
RP13-36G14.3	0	0	0	0	0	0
RP11-571E6.3	0	0	0	0	0	0
OR6C71P	0	0	0	0	0	0
Metazoa_SRP	0	0	0	0	0	0

ACTBP6	0	0	0	0	0	0
BTBD7P1	0	0	0	0	38.40995259	0
FAM32B	0	0	0	0	0	0
RP11-163F15.1	0	0	0	0	0	0
E2F3P2	0	1.01573397	2.005868661	0	2.015212623	1.812121752
Metazoa_SRP	0	0	0	0	0	0
RP11-820K3.3	0	0	0	0	0	0
LINC00449	2.93015605	3.04720192	3.008802992	1.00570022	1.007606311	6.342426133
AC004988.1	0	0	0	0	0	0
RP11-814E24.1	0	0	0	0	0	0
RP5-1113E3.3	0	0	0	1.00570022	0	0.906060876
RP11-77K12.4	0	0	0	0	0	0
INF2	5134.61012	4815.59476	2422.086409	2275.89959	6698.566757	3473.837399
HMGB1P39	0	0	0	0	0	0
OR2N1P	0	0	0	0	0	0
RP11-291L22.4	0	0	0	1.81026039	0	0
PDCD4-AS1	5.8603121	7.11013781	24.07042394	71.4047153	19.14451991	41.6788003
RP11-556O15.1	0	0	0	0	0	0
FAM83H-AS1	12.6973429	10.1573397	1465.287057	1422.0601	2339.661855	1889.136927
RBMS3-AS2	0	0	0	0	0	0
RP11-697H9.3	0	0	0	0	0	0
TAS2R2P	0	0	0	0	0	0
Z99756.1	0	0	0	0	0	0
AC016712.1	0	0	0	0	0	0
RP11-176H8.1	8.25327288	2.82374044	4.753908728	14.7033372	48.18373381	22.71494617
OR52J1P	0	0	0	0	0	0
RP13-259N13.2	0	0	0	0	0	0
Metazoa_SRP	0	0	0	0	0	0
OR1F2P	0	0	0	0	3.022818934	0
RP11-542B15.1	0	0	0	0	0	0
IGBP1-AS1	0	0	0	0	0	0
RP5-1096D14.6	0	0	2.005868661	0	0	0
LINC00970	0	0	0	0	0	0

RP11-335E6.2	0	0	0	0	0	0
RHOT1P2	0	0	2.056015378	6.37613937	8.665414277	5.164546994
GP1BB	8.8295369	8.08524242	84.50724671	78.4848448	93.76784333	90.10775414
RP11-84A19.2	0	0	1.002934331	1.00570022	0	0
AC144450.2	2.93015605	0	79.23181213	98.5586211	45.34228401	152.2182272
AC012456.3	0	0	0	0	0	0
RP11-332M2.1	123.066554	117.825141	53.15551953	75.4275162	167.2626477	75.20305272
LINC00501	0	0	0	0	0	0.906060876
RP11-320P11.1	0.97671868	0	0	3.01710065	1.007606311	2.718182629
AC007618.3	0	0	0	0	0	0
LINC01285	0	0	1.002934331	3.01710065	2.015212623	0
OR2T5	0	0	0	0	0	0
OR2L2	0	0	0	0	0	0
OR2W5	0	0	0	0	0	0
EFCAB2	105.485618	133.06115	37.10857024	75.4275162	146.1029151	78.82729623
COX20	1137.60379	1408.93475	2064.861259	1712.70747	951.1602057	1733.94682
CHML	2350.96187	1635.3317	1661.862186	1715.72457	2768.902143	2152.800642
IBA57-AS1	0	0	5.014671654	0	0	1.812121752
C1orf95	0	0	0	0	268.0232788	154.030349
RP11-351J23.1	0.97671868	4.06293589	0	0	0	0
TCP10	0	0	0	0	0	0
CAPN8	0.97671868	2.03146794	103.3022361	172.980437	67.50962286	288.1273586
TATDN3	111.34593	93.4475254	469.3732668	425.411191	108.8214816	386.8879941
SERTAD4-AS1	218.784985	163.53317	55.16138819	161.917735	17.12930729	46.20910469
C1orf132	150.414677	112.746471	1336.911463	1274.79542	137.0344583	959.5184679
CR1	0	0	0	0	0	1.812121752
C6orf99	0.97671868	3.04720192	49.1437822	89.5073192	8.06085049	55.26971345
LINC00862	4.88359342	4.06293589	7.020540315	9.05130194	2.015212623	4.530304381
RAET1G	59.3454272	62.2137058	1.002934331	0	8.363132384	5.472607692
C1orf53	45.9057781	28.4405512	12.03521197	12.0684026	40.30425245	20.83940015
SAMD5	311.57326	337.223679	0	0	4.030425245	4.530304381
LINC00272	0	0	0	0	0	0
TEDDM1	0	0	0	0	0	0

GJE1	0	0	0	0	0	0
ECT2L	0	0	0	0	2.015212623	2.718182629
GPR52	0.97671868	0	0	0	0	0
RP11-296O14.3	14.6507803	17.2674775	14.04108063	13.0741028	12.09127574	8.154547886
METTL11B	0	0	0	0	0	0
FCGR3A	0	0	0	0	0	0
TMEM244	0	0	0	0	0	0
OR6K3	0	0	0	0	0	0
OR10T1P	0	0	0	0	0	0
CENPW	404.361535	424.5768	245.718911	363.057778	284.1449798	384.1698115
MSTO2P	87.3870206	59.2579199	163.7892055	135.316964	39.33695039	101.8684243
SPRN	1.95343737	0	3.008802992	1.00570022	46.34989032	11.77879139
FAM229B	27.3481231	32.5034871	101.2963674	85.4845183	11.08366942	85.16972236
FANK1	5.8603121	7.11013781	8.023474646	14.079803	39.29664614	17.21515665
AL591704.9	0	0	0	0	0	0
LOR	0	0	0	0	0	0
PRR9	0	0	0	0	0	0
LELP1	0	0	0	0	0	0
SPRR2E	0	0	0	0	0	0
KPRP	0	0	0	0	0	0
METTL10	708.228485	801.271901	463.8470986	378.284079	407.7782742	380.6814771
FAM24A	0	0	0	0	0	0
DDO	18.557655	15.2360096	8.023474646	1.00570022	1.007606311	29.90000891
CCDC162P	0	0	148.4342809	68.3876147	26.19776409	31.71213067
LINC00222	0	0	2.005868661	0	0	3.624243505
ADAMTSL4-AS1	17.5809363	22.3461474	15.04401496	34.1938073	22.16733885	26.27576541
PLPP4	188.506706	191.973721	1589.650914	932.2841	18.1369136	1647.218673
BVES-AS1	0	0	0	0	0	0
LINC00577	0	0	0	0	0	0
HIST2H3C	18.40138	24.8854823	132.7183	0	92.56879182	0
HIST2H2AA3	2.68597638	0	336.3641158	225.105879	0	0
HIST2H2BF	66.8759283	81.3501338	139.2975492	154.1839	179.8778787	264.497291
HIST2H3PS2	0	0	0	0	2.13612538	0

HIST2H2BC	4.32686377	0	46.56624097	61.4784542	148.8133761	81.02902416
RP11-744H18.1	0	0	0	0	0	0
PNLIPRP3	0	0	94.27582709	4.02280086	0	13.59091314
HIST2H3A	18.40138	24.8854823	0	100.580079	92.56879182	362.1887746
HSD3BP4	0	0	0	0	0	0
HSD3B1	0	0	0	0	0	0.906060876
HSD3BP2	0	0	0	0	0	5.436365257
HSD3B2	0	0	0	0	0	0
C1orf137	0	0	0	0	0	0
ATP1A1-AS1	15.6274989	4.06293589	16.04694929	9.05130194	40.30425245	13.59091314
RBM20	5.8603121	8.12587178	2.005868661	8.04560172	5.038031556	5.436365257
SMIM9	0	0	0	0	0	0
C6orf163	18.557655	15.2360096	35.10270157	11.0627024	15.11409467	29.90000891
SNHG5	1707.30426	1602.82821	490.4348877	602.414429	1144.64077	628.8062481
ADD3-AS1	0	0	0	0	0	0
RIPPLY2	0	0	0	0	0	0
CHIAP2	0	0	0	0	0	0
GDI1	2698.67372	2541.3664	8921.100872	8999.00553	5744.363581	9187.457284
PCMTD2	1377.17334	1293.02935	1511.422036	1182.70345	1200.059117	1274.827653
SOX18	19.5343737	8.12587178	13.0381463	15.0855032	123.9355763	24.46364366
CYP17A1-AS1	0	0	2.005868661	0	0	0
LIME1	304.482283	267.310709	231.7580651	200.536623	242.7928168	181.5927208
SPATA42	0	2.03146794	0	0	0	0
RP11-261N11.8	0	0	0	0	0	0
OOEP	2.93015605	2.03146794	0	0	19.14451991	3.624243505
KHDC3L	0	0	0	0	0	0
DPPA5	0	0	0	0	0	0
C1orf146	0	0	0	0	0	0
HSP90B3P	0	1.01573397	0	0	0	0
SPANXN1	0	1.01573397	0	0	0	0
SPANXA2	33.6870274	51.0711041	0	0	0	8.154547886
LINC00632	7.81374947	9.14160575	2.005868661	5.02850108	3.022818934	0.906060876
CXorf66	0	0	0	0	0	0

C10orf62	0	0	0	0	0	0
SAMD13	1.95343737	9.14160575	520.5229176	547.734508	8.06085049	323.7989753
FAM127B	1176.94601	1036.72919	1963.334216	1811.62814	1820.744604	1475.583561
CCDC160	0	0	409.1972069	337.915272	3.022818934	298.0940283
C1orf141	0	0	0	0	0	0
EFCAB7	126.973429	140.171288	223.1027419	203.151444	72.54765441	170.3394447
DEFB110	0	0	0	0	0	0
RP13-379L11.2	0	0	0	0	0	0
GLYATL3	0	0	0	0	0	0
LDLRAD1	0	0	11.03227764	1.00570022	1.007606311	9.060608762
RP11-188C12.3	0	0	0	0	1.007606311	0
RHOXF2B	0	0	0	0	0	0
ARRDC1-AS1	233.435765	237.68175	493.4436907	236.339551	279.1069482	349.7394982
ZYG11A	251.99342	215.335602	742.1714047	756.286562	431.2555012	684.9820224
LINC01270	71.3004639	45.7080288	43.12617622	18.1026039	86.65414277	38.96061768
LCN8	0	0	0	0	0	0
RP11-216L13.16	0	0	0	0	0	0
C1orf185	0	0	0	0	0	0
GLT6D1	0	0	0	0	0	0
IFIT1B	0.97671868	1.01573397	0	0	0	0
COL5A1-AS1	0	0	0	0	0	0
CT83	79.1142134	106.652067	0	0	6.045637868	0.906060876
LIPN	0	0	0	0	0	0
LIPK	0	0	0	0	0	0
LIPJ	0	0	0	0	2.015212623	0
TRPC5OS	0	0	1.002934331	0	0	0.906060876
LCN1P2	0	0	0	0	0	0
LRIT2	0	0	0	0	0	0
RP11-465L10.10	0	1.01573397	0	0	55.41834712	3.624243505
RP11-126H7.4	0	0	0	0	15.11409467	1.812121752
LRRC73	17.5809363	13.2045416	6.017605984	7.03990151	76.57807966	38.96061768
MYCLP1	0	0	0	0	0	0
LINC00963	311.57326	415.435195	2089.523414	1444.18551	1379.08053	1321.036757

RP11-247A12.2	7.81374947	5.07866986	8.023474646	21.1197045	18.1369136	5.436365257
FOXO6	73.2539013	45.7080288	391.144389	543.078116	460.4760843	410.4455769
TCEAL5	0.97671868	2.03146794	0	1.00570022	0	0.906060876
SYS1	1109.22034	1083.60532	477.0958611	524.341921	691.2380817	506.8957572
TCEAL6	1.98273893	1.02589131	0	0	1.027758438	0.924182094
ARMCX7P	0	6.18581989	6.117899417	0	0	16.79836864
INPP5B	297.899199	294.562852	891.60862	635.602536	455.4380527	737.5335532
RPA4	0.97671868	1.01573397	2.005868661	0	8.06085049	0
TDRG1	0	0	0	0	0	0.906060876
LINC00951	0	0	0	0	0	0
NEU4	1.95343737	3.04720192	1.002934331	3.01710065	8.06085049	5.436365257
MAFB	5.8603121	6.09440383	2126.220781	2073.75384	283.1373735	947.7396765
TRAF3IP1	471.755124	465.206159	480.4055444	422.394091	668.0429844	552.6971345
RP1-153P14.8	0	0	0	1.00570022	0	0
BMP2KL	0	0	0	0	0	0
CHIC1	380.920287	432.702672	452.3233831	523.969812	318.4035944	321.651611
RP4-640H8.2	0	0	0	0	0	0
NAP1L6	0	0	0	0	23.17494516	8.154547886
GIGYF2	1963.89802	1973.19529	2848.493969	2453.63699	2567.068523	2739.92809
ECEL1P1	0	0	0	0	0	0
C2orf72	11.7206242	23.3618814	11.03227764	6.03420129	40.30425245	11.77879139
RUFY2	896.627752	901.971767	358.0475561	385.183183	570.3051722	476.5880209
NHSL2	18.557655	15.2360096	3.008802992	33.1881071	59.44877237	20.83940015
GGTA1P	0	0	0	0	0	0
PHACTR4	1309.77976	1318.4227	1555.551147	2131.07876	945.13472	1535.773185
CLPSL1	0	0	0	0	2.015212623	0
ASAH2B	329.808598	397.101196	376.7623107	269.447202	240.3342574	417.8933973
LINC00474	0	0	0	0	0	0.906060876
AGAP6	531.217758	425.562062	798.0348469	511.529301	330.958369	930.7872775
TIMM23B	438.7518	316.482391	422.6465563	239.889672	288.8807294	459.6718643
ZDHHC18	825.327288	889.78296	694.0305569	706.001551	841.3512699	802.7699363
C10orf128	0	0	0	0	0	0
CXorf65	0	0	0	0	0	0

AGAP9	621.378659	429.564054	574.490814	392.695763	241.4023201	549.2722244
LRRC37A5P	0	0	0	0	0	0
NPY4R	3.87757317	3.04720192	0	0	0	0
GPRIN2	9.76718684	7.11013781	0	0	2.015212623	0
SYT15	56.0050493	26.3481392	30.08802992	13.1947868	11.42625557	17.80409622
BMS1P1	203.450502	190.114928	186.9670179	135.09571	115.0887929	260.2750473
TMEM57	569.426993	691.714835	1699.973691	1867.5853	612.6246373	1624.567151
PTPN20	6.83703079	5.07866986	27.07922693	41.2337088	0	18.50176309
GDF5OS	1.95343737	1.01573397	0	0	0	0
ZDBF2	451.244032	347.381019	74.21714047	37.210908	310.3427439	67.04850484
LINC00619	0	0	0	0	0	0
GGNBP1	1.95343737	0	0	1.00570022	0	0.906060876
TXNDC8	0	0	0	0	0	0
RPL12P1	4.88359342	2.03146794	11.11251238	2.04157144	9.249825938	6.342426133
AWAT1	0	0	0	0	0	0
RPL12P16	40.2212754	41.1270685	42.21350598	38.1964942	59.80143457	33.70546459
DAXX	2787.55512	2818.66177	1081.163209	1354.67819	1667.588445	1621.848968
BMPR2	3429.2593	3265.58472	2287.693208	2918.54203	3122.571959	3411.319199
TCEA3	254.923576	222.44574	1239.626833	672.813444	46.34989032	931.4305807
PFDN6	1166.20211	1115.2759	718.1009808	486.758904	572.3203848	740.2517358
RING1	1272.66445	1339.75311	1163.403824	1026.81992	818.1763248	1074.588199
HSD17B8	43.9523408	39.6136249	258.7570573	229.299649	135.0192457	255.5091671
RXRB	1946.60034	1861.84037	1024.998886	1103.25314	524.9628882	1056.466982
OXLD1	277.388106	261.043631	807.3621362	545.089517	628.7463382	744.7820402
RP11-713P17.3	0	0	58.17019118	29.1653063	1.007606311	23.55758278
OR13C3	0	0	0	0	0	0
COL11A2	20.5110924	24.3776153	95.27876142	178.008938	50.38031556	96.04245287
LINC00587	0	0	0	0	0	0
HLA-DOA	24.4179671	14.2202756	0	0	0	0
HNRNPCP2	35.8748773	30.4313898	40.71913383	22.4874568	42.79304004	31.28628205
BRD2	8069.64977	7904.44177	4838.155211	6882.00658	7778.720723	7250.299131
HLA-DMA	467.84825	344.333817	162.4051562	232.236294	45.33220794	144.0636793
PSMB8-AS1	106.462337	168.611839	2.005868661	0	3.022818934	10.87273051

COL5A2	30.2782792	41.6450929	62.1819285	84.4788181	44.3346777	79.7333571
PSMB8	1589.1213	1698.3072	46.13497921	57.3249123	44.3346777	302.6243326
TAP2	6188.22587	5625.44962	184.399506	146.077956	884.7387977	771.4292906
SPIN3	92.788275	96.4947274	191.5604572	125.712527	146.1029151	180.3061144
LINC01420	355.525601	444.89148	858.5117871	802.548772	531.008526	557.2274388
RP11-219G17.4	0	0	0	0	0	0
TMEM235	0	0	0	0	1.007606311	0
PAGE3	0	0	0	0	0	0
TNRC6C-AS1	315.695013	339.275461	103.4225882	75.7191692	110.1313698	104.0248492
FLJ45079	0	0	0	0	0	0
HLA-DRA	1413.31194	1176.21994	0	0	38.28903983	39.86667855
BTNL2	0	0	0	0	0	0
COL15A1	20.5110924	28.4405512	0	0	1.007606311	0.906060876
OR8B2	0	0	0	0	0	0
C6orf10	0	0	1.002934331	0	0	0
TMEM225	0	0	0	0	0	0
NOTCH4	20.5110924	29.4562852	4.011737323	10.0570022	6.045637868	2.718182629
PBX2	2679.13935	2588.53708	1381.040573	1301.95939	1779.745104	1677.109621
AGER	39.0687474	47.7394967	58.17019118	68.3876147	39.29664614	61.61213958
RNF5	1489.42762	1492.05226	822.897589	1019.26711	722.4537252	605.8376049
AGPAT1	4085.61425	4535.25219	1389.064048	1375.79789	1043.880138	1714.267178
DFNB59	12.6973429	23.3618814	5.014671654	8.04560172	5.038031556	5.436365257
PRRT1	95.718431	121.888077	85.24941811	99.5643213	39.29664614	145.8758011
FKBPL	318.410291	339.255147	231.6778304	209.185645	192.4528055	226.515219
MRPL38	1382.90668	1284.24325	1426.413323	1285.83801	2405.226797	1703.784053
SMIM5	0.97671868	1.01573397	4.011737323	4.02280086	59.44877237	25.36970453
ERICH2	13.6740616	4.06293589	1.002934331	0	1.007606311	0
SP5	217.808267	167.596105	1.002934331	6.03420129	6.045637868	3.624243505
CYP21A1P	3.90687474	2.03146794	7.140892435	0	0	1.812121752
STK19	359.539915	446.922948	427.2500249	339.805989	319.4112007	353.3637417
CD300LD	0	0	0	0	0	0
BTBD17	0	0	0	0	0	0
DXO	547.939182	453.017352	501.4671654	400.268686	330.4948701	345.2091938

SKIV2L	2056.96955	2206.17419	1370.008296	1463.29381	1260.515495	1709.736873
C9orf129	3.32084353	1.14777939	0	2.3332245	4.584608716	3.134970632
NELFE	2450.58718	2512.92585	1633.780025	1393.9005	1552.721326	1704.300508
NXPE2	0	0	0	0	0	1.812121752
RP11-380J14.1	122.089835	158.4545	0	0	2.015212623	0
SPANXN5	0	0	0	0	0	3.624243505
C10orf126	0	0	0	0	2.015212623	0
ZBTB12	307.666385	415.435195	140.4108063	195.105842	268.0232788	198.4273319
RP11-552E4.3	0	0	0	0	0	0
SDHD	3094.32293	3092.89979	1933.617272	1563.00899	1276.617044	1886.753987
EHMT2	2955.55074	3218.86096	2070.056459	2424.74322	2869.662775	3323.431294
XAGE1A	957.848479	35.7233638	1.544518869	0.57324912	40.92896836	23.55758278
AC005042.4	0.97671868	0	5.014671654	2.01140043	2.015212623	1.812121752
LAYN	2099.94517	1707.44881	0	0	30.22818934	9.966669638
XAGE1B	3097.4875	4108.47124	3.470152784	3.44955174	42.70235547	23.55758278
SLC44A4	0	1.01573397	2.005868661	5.02850108	6.045637868	8.154547886
NEU1	1371.31303	3473.81019	1980.795303	8633.93635	1254.469858	1858.330857
C6orf48	2014.97064	1689.1656	2541.435594	1387.8663	2172.399207	1750.509613
HSPA1B	5548.05514	7082.79425	5296.335731	5366.44652	8082.151288	4414.464498
HSPA1A	2468.85182	12704.8082	1345.273384	10972.1491	10638.16637	12147.40414
HSPA1L	29.3015605	45.7181861	111.4059455	93.540177	33.26108434	58.00601729
LSM2	1302.94272	1336.70591	429.2558935	489.776005	715.400481	706.7274834
RP11-410N8.4	0	0	0	0	0	0
VARS	6997.21265	7050.2095	1891.534148	1629.23435	4961.453477	5036.792411
VWA7	35.1618726	34.5349551	12.03521197	4.02280086	22.16733885	28.08788716
CARD16	25.3165483	27.4248173	0	0	0	1.812121752
RP11-65D24.2	0	0	0	0	0	0
AC012306.2	0	0	0	0	0	0
CASP12	0	0	0	0	0	0
MBD5	287.155293	314.877531	234.6866334	290.647362	672.0734096	544.5425866
MSH5	575.472881	577.068942	335.6018857	461.173891	284.6084787	491.592389
CSHL1	0	0	0	0	0	0
C6orf25	0	0	48.14084787	70.3990151	7.053244179	19.93333928

LY6G6C	0	1.01573397	0	2.01140043	9.068456802	0.906060876
XXbac-BPG32J3.20	0	0	0	0	0	0
LY6G6F	0	0	0	0	1.007606311	0
ABHD16A	1086.11118	1311.31256	642.880906	902.113093	640.837614	925.9942155
LY6G5C	16.6042176	21.3304134	52.1525852	76.4332164	48.36510294	37.14849592
RP11-1E11.1	0	0	0	0	0	0.906060876
POTEKP	0	0	217.5966324	250.540038	612.6750176	321.234823
CSNK2B	4777.09201	4390.08349	2677.603988	2236.67728	3079.758767	3092.322346
CTSLP6	0	0	0	0	0	0
GPANK1	649.517925	793.288232	386.1297173	331.881071	230.7418453	343.3970721
C6orf47	999.183214	945.648328	812.3768079	766.343564	778.8796786	893.3760239
FAM155A	13.6740616	6.09440383	2.005868661	0	23.17494516	6.342426133
APOM	16.6042176	9.14160575	26.0762926	18.1026039	16.12170098	19.0272784
C9orf170	0	0	0	0	0	0
TRIM49C	0	0	0	0	0	0
TRIM64	0	0	0	0	0	0
TRIM51BP	0	0	0	0	0	0
RP11-313I2.10	0	0	0	0	0	0
AC079586.1	0	0	0	0	0	0
BAG6	14374.3689	14149.1742	5563.276732	4967.15336	6581.684425	5791.541121
C1orf195	0	0	0	0	0	0
PRRC2A	23956.9559	23616.8306	13269.82413	15539.074	14047.03959	21959.2914
TBC1D3P4	0	0	0	0	0	0
AIF1	0	0	0	0	0	0
NCR3	0	0	0	0	0	0
PRAMEF20	0	0	0	0	0	0
PRAMEF17	0	0	0	0	0	0
PRAMEF19	0	0	0	0	0	0
PRAMEF14	0	0	0	0	0	0
LST1	1.95343737	7.11013781	0	1.00570022	4.030425245	0
NFKBIL1	509.847153	574.905428	687.0100165	583.306125	427.225076	524.6092473
PRAMEF15	0	0	0	0	0	0
PRAMEF9	0	0	0	0	0	0

PRAMEF7	0	0	0	0	0	0
MCCD1	0	0	0	0	0	0
ZNF814	95.5914576	99.3387825	369.731741	341.94813	193.7526176	488.7020548
MICB	1422.57123	1824.3801	119.3491854	476.701902	509.3147622	260.2659867
AADACL4	0	0	0	0	0	0
ZNF551	288.59107	280.261318	193.4760617	157.894934	387.6966804	347.0031944
MICA	982.110171	1527.54201	313.9184455	1057.99663	619.2043065	502.6372711
ZNF805	121.113117	87.3531216	81.23768079	94.5358203	98.74541851	166.7152012
HLA-C	3229.22731	3191.91354	3108.354254	5162.23909	645.4322988	6966.520865
PSORS1C3	7.81374947	2.03146794	0	0	0	0
GUCY2EP	0.97671868	0	0	0	0	0
POU5F1	7.81374947	8.12587178	0	1.00570022	8.06085049	1.812121752
ZSCAN5CP	0	0	0	0	0	0
CCHCR1	1687.76989	1431.16917	587.7195178	539.055316	564.2595343	832.6699452
PSORS1C2	0.97671868	0	0	1.00570022	0	0.906060876
CDSN	3.90687474	0	133.390266	247.402253	110.8366942	93.32427025
PSORS1C1	176.786082	166.580371	3.008802992	2.01140043	22.16733885	5.436365257
C6orf15	0	0	1.002934331	1.00570022	0	0
MUC21	0	0	0	0	0	0
DEFB122	0	0	0	0	0	0
DEFB121	0	0	0	0	0	0
CFTRP3	0	0	0	0	0	0
CTD-2514C3.1	1.52368115	4.17466663	0	0	1.380420646	0
DHX16	2701.60388	2830.85058	1147.356874	1201.81176	1871.12492	1697.052021
C6orf136	606.542303	625.692127	327.9595261	378.143281	376.8447604	403.1970899
LINC01552	0	0	0	0	0	0
MRPS18B	1823.53378	1602.82821	1599.680257	1126.38424	1551.713719	1635.439882
PPP1R10	2500.39983	3025.8715	3009.805926	4881.66885	2488.787589	3869.786002
KRTAP5-11	0	0	0	0	0	0
KRTAP5-10	0	0	1.002934331	0	0	0
ABCF1	6511.78347	6923.24275	1979.792369	1859.5397	3575.994799	2760.76749
PRR3	678.819485	582.015566	401.1737323	276.567559	460.4760843	590.7516913
LILRB3	0	0	0	0	0	0

DDR1	2035.48174	2162.49763	7308.382468	9085.49575	9496.689484	9055.172397
AC096670.3	0.97671868	0	1.002934331	5.02850108	2.015212623	0.906060876
LRCOL1	0	0	0	0	0	0.906060876
RP11-304F15.3	7.81374947	9.14160575	24.07042394	26.1482056	1.007606311	38.96061768
LINC01123	346.539789	376.796674	214.7081815	157.241229	752.1781114	261.5978962
GNL1	1525.63458	1744.01523	1238.623898	1511.56742	1359.260914	1956.185432
HLA-E	8140.10048	7977.90981	1194.895962	1346.63259	2460.232026	1569.297438
DPRX	0	0	0	0	64.48680392	9.060608762
TRIM39	772.584479	830.870389	306.4164967	315.196505	435.2859265	434.9092206
LINC01257	0	0	0	0	4.030425245	0
ZNF468	480.672566	558.887304	622.6216325	1025.90473	630.5197254	754.5856189
TRIM15	0	1.01573397	0	0	2.015212623	0
ZNF616	180.692957	179.784913	487.4260847	445.525195	340.5709332	665.954744
FOXB2	0	0	0	0	0	0
TRIM10	0	0	0	0	0	0
TRIM40	1.95343737	0	0	0	0	0
TRIM31	0	0	0	1.00570022	10.07606311	18.12121752
RNF39	0	1.01573397	400.170798	488.770305	15.11409467	242.8243148
PPP1R11	1458.241	1569.30899	1082.166143	927.255599	991.4846103	963.1427114
RP11-1148L6.9	5.8603121	3.04720192	4.011737323	4.02280086	9.068456802	2.718182629
HLA-J	6.83703079	1.01573397	0	0	1.007606311	0
ZNRD1-AS1	100.602024	114.777939	375.0974397	186.05454	144.0877025	241.9182539
PTCHD2	5.8603121	7.11013781	87.25528677	36.2052078	28.21297672	85.16972236
HCG9	0	0	0	0	0	0
GNB2L1	27652.8594	25608.6849	28460.2675	20658.0881	27872.40578	19218.45724
HLA-G	0	0	0	0	0	0
TBC1D8	759.887136	567.79529	1344.934937	1220.92006	400.0197056	1548.458037
AC068538.2	0	0	0	0	0	0
NMS	0	0	0	0	0	0
HLA-F	131.896091	138.170292	22.06455528	22.1254047	60.45637868	104.1970008
ZFP57	0	0	0	0	0	0
SSX9	0	0	0	0	0	0
CRHR1-IT1	305.830154	410.742504	845.6240809	556.353359	421.1794381	715.9058801

RPS26P8	0	0	0	2.99698664	0	0
ASPDH	0	0	1.002934331	0	1.007606311	0
MOG	0	0	0	0	0	0
OR2H2	0	0	0	0	0	0
CST9LP2	0	0	0	0	0	0
CBY3	0	0	1.002934331	9.05130194	0	9.060608762
C5orf60	0	0	1.002934331	3.01710065	7.053244179	0.906060876
CST9LP1	0	0	0	0	0	0
CST13P	0	0	0	0	0	0
FLJ26850	0	0	8.023474646	2.01140043	3.022818934	4.530304381
C9orf57	0	0	0	0	0	0
IGKV1OR2-3	0	0	0	0	0	0
IL31	0	0	0	0	0	0
AKT1S1	2174.17579	2432.68286	1282.753009	2603.75786	2562.34285	2137.397607
FAM153C	0	0	7.020540315	3.01710065	10.84184391	6.269941263
GABBR1	933.743062	984.246219	1394.07872	1036.87692	201.5212623	794.6153884
CASC10	136.740616	223.461474	1.002934331	4.02280086	229.734239	16.30909577
C10orf113	0	0	0	1.00570022	0	0.906060876
RP5-1004I9.1	0	0	0	0	0	0
STARD7-AS1	87.9046815	86.3373876	50.14671654	51.290711	112.8519069	45.30304381
MAS1L	0	0	0	0	0	0
OR2H1	0	0	0	0	0	0
OR11A1	0	0	0	0	0	0
OR14J1	0	0	0	0	0	0
OR2U1P	0	0	0	0	0	0
UBTFL3	0	0	0	0	0	0
OR2J2	0	0	0	1.00570022	0	0
OR2J3	0	0	0	0	0	1.812121752
OR2J1	0	0	0	0	0	0
OR2B3	0	0	0	0	0	0
OR2W1	0	0	0	0	0	0
UBTFL5	0	0	0	0	0	0
MAMDC2-AS1	10.7439055	5.07866986	1.002934331	5.02850108	1.007606311	4.530304381

LINC01556	0	1.01573397	0	1.00570022	0	2.718182629
SPDYC	0	0	0	0	0	0
C9orf135	0.97671868	0	0	0	0	0
TRIM27	2648.86107	2910.07783	2177.370432	1889.71071	1763.311045	2313.173417
AC073464.11	0	0	0	0	0	0
CNN2P12	0	0	0	0	0	0
MALRD1	0	0	0	2.01140043	890.7239792	23.55758278
AC083899.3	268.734379	177.022117	121.0942911	98.6692481	166.1039004	173.8821427
CTC-281M20.1	0	0	0	0	0	0
CTC-308K20.1	52.7428089	44.6922948	6.017605984	6.03420129	16.12170098	3.624243505
RANBP17	251.016702	216.351336	88.2582211	112.638424	395.9892803	152.2182272
FAM196B	399.477942	383.947442	0	0	61.46398499	20.83940015
IGKV1OR-3	0	0	0	0	0	0
RP11-15J10.1	12.6973429	8.62358142	0	0	0	0
FOXD4L5	3.68222944	4.06293589	10.45057573	13.828378	7.093548431	7.012911182
IGKV1OR9-1	0	0	0	0	0	0
REG1P	0	0	0	0	0	0
ZNF204P	89.8581189	120.872343	408.1942726	355.012176	26.19776409	184.8364187
BX255923.2	0	0	0	0	0	0
CTD-3065J16.6	1.95343737	0	1.002934331	5.02850108	0	0.906060876
LINC01291	110.369211	123.919545	4.011737323	0	76.57807966	25.36970453
RP11-96J15.2	0	0	0	0	0	0
RP11-111F5.4	94.1947499	92.6552529	31.23137506	41.76673	151.5943695	20.35012728
FAM27E4	0	0	0	0	0	0
RP11-160N1.10	0	0	0	0	0	0
TTC25	5.8603121	8.12587178	14.04108063	4.02280086	23.17494516	8.154547886
RP11-475I24.1	0.43952341	0.56881102	0	1.82031739	0	4.358152814
ADGRF5P2	1.24043273	0	0	0	0	0
MRPL53	693.470266	674.701291	989.4147759	1151.0943	699.27878	920.5578502
ST8SIA6-AS1	0	0	418.2236159	295.675863	0	220.1727929
RP11-204M4.2	0	1.01573397	0	0	10.07606311	0
MROH6	255.900295	247.839089	174.5105735	227.288249	252.9091841	165.8091403
ATXN2	2308.96297	2119.8368	2172.35576	1772.04378	1662.550414	2001.488475

DCTN1	6586.23873	6548.99557	6514.780591	5676.75532	4679.32371	5877.11857
SPATA31A1	0	0	0	0	0	0
CTB-158D10.3	0	0	0	0	0	0
PNMAL2	0	0	0	0	0	0
TCTN1	282.2717	297.610054	716.0951121	399.262986	532.0161324	564.4759259
FAM216A	604.588865	521.071528	106.3110391	139.79233	295.2286492	251.8849236
ZBTB48	245.15639	229.555878	584.7107148	392.223084	244.8483336	548.1668301
FAM201A	96.6951497	109.699269	330.9683291	414.348489	164.2398287	159.4667142
IGFL2	0	0	0	0	0	0
IGFL4	0	0	0	2.01140043	0	0
NAT8B	0	0	0	0	0	0
KRTAP9-3	0	0	0	0	0	0
AC021218.2	123.066554	135.092618	0	0	13.09888205	9.060608762
KRTAP4-8	1.95343737	1.01573397	0	0	0	0
GPR20	0	0	0	0	4.030425245	3.624243505
KRTAP1-4	0	0	0	0	0	0
KRT40	0	0	0	0	0	0
RP11-208G20.2	0	0	0	0	0	0
KRT25	0	0	0	0	0	0
MZT1	1109.55242	1161.99966	488.4290191	613.477131	930.0206253	513.7365168
LINC01545	0	1.01573397	5.014671654	2.01140043	0	0
SPINK9	0	0	0	0	0	0
LRRC3C	0	0	0	0	0	0
RP6-99M1.1	3.90687474	2.03146794	0	0	0	0
PRR20B	0	0	0	0	0	0
PRR20A	0	0	0	0	0	0
ZNF155	240.272796	235.650282	316.9272485	191.083041	177.3387108	331.6182807
UQCC3	731.562294	749.611672	860.5176557	565.203521	681.1418664	677.7335354
FBXO48	225.622016	171.659041	104.3051704	66.3762142	49.37270925	83.35760061
GRXCR2	2.93015605	0	0	0	0	0
AC074391.1	5.8603121	3.04720192	1.002934331	1.00570022	0	0
FAM221B	0	0	24.07042394	14.079803	0	11.77879139
CD177P1	0	0	0	0	0	0

ATP6V0E2-AS1	188.506706	187.910785	135.3961346	193.094441	79.60089859	103.2909399
CD177	17.5809363	27.4248173	0	0	11.08366942	3.624243505
PSG5	75.2073387	48.6333426	0	0	9.562183894	7.248487009
ZNF783	424.677284	405.135652	623.805095	584.090571	592.9863903	705.1509375
ZNF425	61.5332771	51.8024326	100.2934331	168.957636	214.6201443	121.4121574
FAM83A-AS1	0	0	0	0	1.007606311	0.906060876
LRRC10B	0	0	0	0	88.66935539	5.436365257
FBXO47	0	0	0	0	1.007606311	0
C12orf73	188.506706	242.760419	360.0534247	206.168544	321.4264133	203.8636971
PCDHGA1	25.0235327	30.2079283	34.35050083	12.0684026	130.1525072	31.96582771
ARHGEF34P	142.981848	120.994231	1161.087045	1089.64601	417.0281001	1137.151703
BLACE	0	0	0	0	0	0
PCDHA9	0	0	4.733850041	11.3543554	0	8.172669103
PCDHA8	0	0	4.673673981	3.15789868	4.584608716	5.63569865
PCDHA7	1.95343737	5.07866986	26.01611654	10.7710493	56.42595343	31.41313058
PCDHA5	0	0	39.58581803	102.299826	2.015212623	60.15338157
PCDHA4	0	1.01573397	115.7185631	288.615848	1.007606311	192.7463302
PCDHA2	0	0	0	6.5269944	0	2.718182629
PCDHA1	0	5.07866986	5.014671654	0	7.63765584	1.812121752
RP11-807H22.7	0	0	0	0	0	0
TRIM13	549.892619	574.854642	759.2012297	572.997698	468.6175433	294.3429362
ERICH4	0	0	0	2.01140043	0	0
MS4A13	0	0	0	0	0	0
PRSS3P4	0	0	0	0	0	0
PRSS1	0	0	0	0	0	0
OR4D8P	0	0	0	0	0	0
RP5-842K16.1	0	0	0	0	0	0
SPIRE2	332.084353	428.639736	517.5141146	361.046377	371.8067289	528.2334908
AC139712.2	0	1.01573397	0	0	0	0
AARD	5.8603121	14.2202756	0	1.00570022	53.4031345	2.718182629
RP11-46C24.3	0	0	0	0	0	0.906060876
RP11-830F9.6	0	0	0	0	19.14451991	0.906060876
PABPN1L	0	0	0	0	13.09888205	0

OR5G5P	0	0	0	0	0	0
OR5D16	0	0	0	0	0	0
OR5L2	0	0	0	0	0	0
RP11-707M1.1	0	0	0	0	0	0
RP11-863P13.4	0	0	0	0	0	0
PKHD1L1	0	1.01573397	3.008802992	0	2.015212623	0.906060876
CTC-425O23.2	80.0909321	78.2115159	35.10270157	22.1254047	37.28143352	37.14849592
RP11-56P9.11	0	0	0	0	0	0
SLFN12L	53.7195276	45.7080288	2.005868661	2.01140043	1.007606311	10.87273051
LINC01121	0	1.01573397	15.04401496	13.0741028	0	2.718182629
RP11-693J15.5	0	0	0	0	0	0
CLLU1OS	0	0	0	0	0	0
SLC35B4	1602.79536	1637.36316	514.5053117	571.237722	1296.789323	732.097188
LGALS7	0	3.04720192	0	0	0	0
SYCE1L	11.8475976	3.05735926	1.002934331	2.01140043	8.101154743	2.718182629
TMEM231	289.098963	292.368867	146.2077667	182.413905	362.1437843	201.1455145
FAM71F2	9.76718684	6.09440383	31.09096425	37.210908	12.09127574	130.4727662
C2orf91	0	0	0	0	0	0
CCNI2	8.79046815	1.01573397	1.002934331	0	38.28903983	2.718182629
TMEM240	10.7439055	16.2517436	13.0381463	7.03990151	10.07606311	2.718182629
FRG2	0.97671868	0	0	0	0	23.28576452
HSP90AA4P	0	0	0	1.03587122	0	0
COX17P1	0	0	0	0	0	0
DKFZp779M0652	0	0	2.005868661	1.00570022	1.007606311	4.530304381
FAM205A	0	0	1.002934331	0	0	0
CDKL4	0.97671868	3.04720192	0	4.02280086	0	3.624243505
TMEM88B	0	0	0	0	0	0
ACCSL	0	0	0	0	0	0
C4orf47	6.83703079	7.11013781	0	2.01140043	2.015212623	2.718182629
TRIQK	428.779502	474.347765	986.8873814	1178.68065	1788.501203	644.209283
SDHAF1	219.761704	219.398538	530.5522609	421.38839	395.9892803	491.0849949
ARID3C	0.97671868	0	29.08509559	23.131105	0	25.36970453
PSENEN	571.38043	572.955219	1214.192418	979.55201	539.0089202	766.6905922

C7orf66	0	0	0	0	0	0
REXO1L1P	0	0	0	0	0	0
C11orf91	6.71005736	4.54033086	1.313843973	1.00570022	0	0.906060876
LINC00654	53.7195276	55.8653685	0	1.00570022	1.007606311	0
SLC10A5P1	0	0	1.002934331	0	0	0.906060876
FABP9	0	0	0	0	0	0
ZBTB10	949.370561	804.461306	63.18486283	82.4674177	2279.205476	176.6818709
C4orf46	1080.25086	998.466495	457.3380548	549.112318	502.7955493	646.9274656
SCGB2B2	13.6740616	7.11013781	6.017605984	7.03990151	4.030425245	12.68485227
CCDC144NL	0	0	0	0	0	4.530304381
LGR4	1094.90164	1272.71467	92.26995843	413.342789	803.0622301	227.4212799
KRT17P7	0	0	0	0	0	0
PSMB10	517.612067	419.802851	190.5474935	247.764305	452.7074396	502.9453318
VIT	0	0	0	0	0	0
TTLL10-AS1	0	0	0	0	3.022818934	0
RP11-514P8.6	31.8703307	0	26.42731961	22.1958038	107.4813652	12.06873087
SPDYE2	0	0	0	0	0	4.303789162
OR7E36P	0	0	0	0	0	0
E2F4	1798.1391	1876.06065	1384.049376	1215.89156	2294.319571	1937.158153
KRT17P5	0	0	0	0	0	0
DGAT2L7P	0	0	0	0	0	0
PDE7A	345.758414	338.239413	3308.680357	2160.24406	722.4537252	2371.161313
TMEM170B	320.363728	480.442169	285.8362843	496.815906	335.5329017	331.6182807
TRBV20OR9-2	0	0	1.002934331	0	0	0
MUC12	1.95343737	1.01573397	0	0	9.068456802	2.718182629
CTXN3	0	0	0	0	0	0
LINC01602	0	0	0	0	0	0
RP11-352D3.2	0	0	0	0	0	0
MGAT4D	0	0	0	0	0	0
SNX2	1708.28098	2128.97841	1814.308204	2305.06489	1760.288226	1887.324805
SAP25	10.4997259	8.18681582	7.572154197	3.77137581	5.934801173	0
NT5M	40.045466	28.4405512	138.4049376	110.627024	33.25100827	65.23638308
KRT17P4	0.97671868	1.01573397	1.002934331	0	0	0

GCNT6	11.7206242	8.12587178	2.005868661	6.03420129	0	0.906060876
SARNP	858.936178	759.769011	967.8316291	859.873684	944.1271137	820.3928203
AC005863.1	1.95343737	2.03146794	0	0	0	0
OR6C68	0	0	0	0	0	0
OR6C65	0	0	0	0	0	0
OR6C3	0	0	0	0	0	0
OR6C1	0	0	0	0	0	0
OR6C72P	0	0	0	0	0	0
GOLGA2P1	0	0	0	0	0	0
LINC01460	4.88359342	8.12587178	0	0	0	1.812121752
ADGRG1	2665.46529	2774.98521	9142.749359	8178.35415	4092.896836	5766.171416
IPO7	9852.09299	9280.75115	2784.145702	2997.99234	6878.928287	4294.728553
PRR13	1202.18442	1447.72563	1388.321877	1974.51135	4215.81473	2026.205816
TECPR1	682.72636	748.595938	760.2242227	664.767842	412.1109813	629.7123089
MT1H	0	0	0	0	0	0
SLCO6A1	0	0	0	0	0	0
MT1CP	0	0	0	0	0	0
MT1DP	0	0	0	0	0	0
MT1A	13.7033631	21.3304134	0	0	0	0.906060876
C15orf59	10.7439055	17.2674775	3.008802992	0	46.34989032	2.718182629
MT1M	0	0	0	0	0	0
LINC00661	0	0	0	0	4.030425245	0
CFI	0	0	0	0	0	2.718182629
OR52E6	0	0	0	0	0	0
HNRNPA1P20	0	0	0	0	0	0
SAMD9	1076.34399	1247.32132	52.1525852	89.5073192	90.68456802	370.5788984
RP11-401P9.6	0	0	1.002934331	0	0	0
KRT6A	0	0	0	0	18.1369136	0
CNEP1R1	357.479038	419.498131	371.0857024	317.801268	236.7874832	356.0819243
AL592528.1	0	0	0	0	0	0
KRT81	347.819291	310.002008	2.617658603	0	339.7144679	51.20150011
SNRPGP6	0	0	0	0	0	0
EXOC3L4	6.83703079	5.07866986	18.05281795	22.1254047	55.41834712	34.43031329

KRTAP12-3	0	0	0	0	0	0
IZUMO3	0	0	0	0	0	0
RP4-529N6.1	0	0	0	0	0	0
KRTAP10-2	0	0	0	0	1.007606311	0
NBPF22P	0	0	0	0	0	0
RP11-812E19.3	0	0	0	0	0	0
TP53TG3D	1.95343737	3.04720192	0	0	0	0
TP53TG3C	0	0	0	0	0	0
ATP6AP1L	69.3470266	57.8968364	116.3403824	95.5415205	14.10648836	59.80001783
CCDC85C	3961.57098	3028.91871	1737.082261	2356.35561	4428.429738	3151.279727
SPDYE18	1.04508899	0	0	0	0	0
AC004980.7	123.066554	110.715003	82.66184754	50.6168918	77.05165462	94.64711913
CALML3-AS1	0	0	11.03227764	6.03420129	19.14451991	7.248487009
OR52A4P	0	0	0	0	0	0
OR52J3	0	0	0	0	0	0
OR51A2	0	0	0	0	0	0
OR51A4	0	0	0	0	0	0
AC013472.3	0	0	1.002934331	0	1.007606311	0
C2CD4B	0	0	11.03227764	9.05130194	0	0.906060876
RGL3	209.994517	240.728951	1658.853383	1751.92978	3.022818934	1190.563991
NAP1L4	3789.66849	3687.11432	4120.054231	3682.87419	2825.328097	4582.855912
SMG1P2	82.1518085	75.1135272	161.8936597	267.445858	294.6039333	239.1910107
RP11-89H19.1	0	0	0	0	0	0
TMSB4X	32677.5691	30298.095	5282.415002	5736.42352	25992.81697	5386.504727
TMEM256	549.736344	640.57263	295.8656276	377.087296	306.050341	283.4249027
C9orf92	0	0	0	0	0	0
CHKB-AS1	7.81374947	11.1730737	22.06455528	36.2052078	9.068456802	48.92728731
CPT1B	372.168887	236.280037	1090.600821	1171.75138	107.612354	704.2176948
RP11-497E19.1	0	0	0	0	0	0
SMN2	1523.35883	1383.35857	988.8731914	699.806438	1184.612512	820.3565779
SERF1B	652.565287	682.055205	301.8732042	154.596237	440.4045665	222.4923088
POM121B	22.4547625	27.1911984	16.44812302	32.856226	9.461423263	27.88855377
DYNLL1P1	0	0	0	0	0	0

HMGN1	6495.17925	6150.22857	8374.461544	7459.21816	14075.20218	9090.318498
STAG3L1	79.2607212	104.031473	133.6209409	112.095346	47.21643175	111.8441546
AC005488.11	0	0	0	0	0	0
MUC19	0	4.06293589	3.008802992	3.01710065	3.022818934	62.51820046
DENND6B	66.4168705	47.7394967	464.3585951	191.083041	72.54765441	290.8455413
RP5-1132H15.2	0	0	0	0	0	0
EIF3CL	2997.26639	1811.35839	2310.008497	2224.61893	3828.541245	2397.699836
LINC01597	1.95343737	6.09440383	0	0	0	0
AF064858.6	0	0	1.002934331	0	0	0.906060876
RP11-15G8.1	0	2.03146794	0	0	0	0
LINC01446	0	0	0	0	2.015212623	0
LCMT1	364.316069	405.277855	630.845694	626.551234	803.0622301	549.072891
LINC01310	0	0	0	0	0	0
LINC00898	0	0	4.011737323	2.01140043	17.12930729	10.87273051
LINC00583	0	0	0	0	0	0
MFSD2B	16.6139848	10.1573397	1.002934331	0	7.053244179	5.436365257
VCX3B	0	0	0	0	2.015212623	0
CDPF1	94.7417123	97.5104613	276.8098753	209.185645	78.59329228	148.5939837
CTD-2375G15.1	0	0	0	0	0	0
HTN3	0	0	0	0	0	0
RP3-416J7.5	0	0	0	0	0	0
LIN52	314.503416	311.830329	346.0123441	354.006476	309.3351376	358.800107
RP11-706O15.7	0	0	0	0	26.52019811	4.530304381
RP11-706O15.5	1.19159679	0	35.78469692	27.5561859	244.0624007	33.23431294
RP11-706O15.1	401.597421	391.47403	293.5588786	290.144512	351.7452872	351.1620138
ARSH	0	0	3.008802992	2.01140043	0	0.906060876
ACOT6	0	0	5.014671654	2.01140043	1.007606311	1.812121752
SMIM11A	134.044872	315.334612	54.0982778	124.5962	59.17671866	60.189624
TECRL	0	0	0	0	0	0
RP11-798L4.1	0	1.01573397	0	0	1.007606311	0
DPF3	264.690763	188.926519	39.1144389	58.3306125	24.18255147	58.89395695
MANSC4	0	0	0	4.02280086	1.007606311	0.906060876
RP11-15H7.2	0.97671868	0	0	0	0	0

ADARB2-AS1	0	0	0	0	0	0
CYP2D7	5.45985744	17.3995229	32.47501363	23.131105	5.320161324	28.80367525
LINC00634	2.93015605	4.06293589	58.17019118	46.2622099	18.1369136	39.86667855
LYRM5	78.1374947	54.8496345	321.9419202	246.396553	184.391955	208.3940015
C17orf107	22.4645297	20.3146794	10.02934331	6.03420129	40.30425245	9.060608762
FAM183DP	0	0	0	0	0	0
MBD3L4	0	0	0	0	0	0
ITSN1	2212.26782	2221.4102	572.6755028	1061.01373	1133.5571	659.6123179
ITPRIPL2	2219.28066	2062.9557	5061.809567	4134.43359	648.8984645	4179.658822
RP11-363N22.3	22.4645297	7.11013781	9.026408976	8.04560172	45.34228401	30.80606979
DENND1C	2.93015605	4.06293589	3.008802992	3.01710065	0	1.812121752
AC083864.4	0	0	0	0	0	0
RP11-1212A22.1	652.379711	462.321475	1044.245196	624.861658	617.219322	764.7244401
SLCO1B7	2.69574357	6.09440383	1.002934331	0	1.007606311	1.812121752
CRLF2	12.2089835	31.9956201	0	0	0	0
CRYZL1	577.240742	522.087262	408.1942726	336.909572	325.4568385	322.5576719
RP9P	125.557187	110.989251	1.163403824	0	125.0540193	2.990000891
C5orf51	1891.90409	2088.34905	905.6497006	1345.62689	890.7239792	1083.648808
CTD-2302E22.1	0	0	0	0	0	0
CATSPER2P1	22.2008157	16.5361491	74.13690573	43.2953943	5.300009197	35.86188948
GAGE1	0	0	0	0	1.007606311	0
ARRDC5	0	0	0	0	0	0
LINC01531	0	0	0	0	0	0
DPP9-AS1	0.97671868	3.04720192	0	4.02280086	1.007606311	0.906060876
LOH12CR2	7.81374947	1.01573397	28.08216126	24.1368052	2.015212623	14.49697402
RP11-333E13.4	3.90687474	3.04720192	6.017605984	11.0627024	1.007606311	0.906060876
CYS1	53.7195276	67.0384422	0	0	19.14451991	4.530304381
PLPP6	78.1374947	96.4947274	331.9712635	374.12048	640.837614	244.6364366
KLRC2	30.2099089	28.2475618	0	0	1.440877025	0
KLRC3	14.0354475	1.40171288	0	0	0	0
TPTE2P6	0.97671868	3.04720192	0	0	1.007606311	0
AC024132.1	0	0	0	0	0	0
C16orf96	0	0	2.005868661	3.01710065	0	2.718182629

GMNC	4.88359342	3.04720192	0	0	0	0
LINC00487	0	0	0	0	0	0
TTC23L	0	1.01573397	86.25235244	117.666925	4.030425245	19.0272784
CLEC6A	0	0	0	0	0	0
OR7E91P	0	0	0	0	5.038031556	0.906060876
RFPL3S	0	0	35.10270157	18.1026039	1.007606311	34.43031329
C22orf42	0	0	0	0	0	0
NANOGNB	0	0	0	0	0	0
LRRC72	0	0	0	0	0	0
C1QTNF9B-AS1	14.6507803	28.4405512	6.017605984	31.1767067	11.32549494	12.68485227
C1QTNF9B	0	0	1.002934331	0	0	0.906060876
KRTAP5-6	0	0	0	0	0	0
FAM99B	0	0	0	0	0	0
FAM99A	0	0	0	0	0	0
KRTAP5-2	0	0	0	0	1.007606311	0.906060876
KRTAP5-1	0	0	0	0	0	0.906060876
RPS3AP47	8.86860565	9.19239245	24.24092277	26.3392886	22.83235901	11.92376113
FAM90A2P	0	0	0	0	5.038031556	0.906060876
DEFB134	0	0	0	0	0	0
DEFB135	0	0	0	0	0	0
DEFB136	0	0	0	0	0	0
C1RL-AS1	65.4401518	86.3373876	0	0	5.038031556	2.718182629
RP11-473M20.5	0	0	14.04108063	4.02280086	1.007606311	4.530304381
OR7E136P	0	0	0	0	2.015212623	0
RP11-3B12.4	0	0	0	0	1.007606311	0
BHLHA9	0	1.01573397	0	0	0	0
ZNF316	1409.40506	1559.15165	2219.493674	2282.93949	2763.864112	2370.255252
SRRM2-AS1	18.557655	27.4248173	60.17605984	35.1995075	85.64653646	71.57880922
DAZ4	0	0	0	0	0	0
PDPK2P	31.0889557	28.67417	71.48915909	49.0781705	45.92669567	88.5221476
ONECUT3	0	0	0	0	0	0
CEMP1	5.8603121	5.07866986	13.0381463	16.0912034	3.022818934	12.68485227
OLIG2	0	0	0	0	0	0

C21orf62	0	0	0	0	0	0
C21orf62-AS1	2.93015605	3.04720192	12.03521197	6.03420129	0	10.87273051
PPP1R12BP2	0	0	0	0	0	0
RNPS1	4028.93527	3813.04502	2641.708968	2790.80804	6573.502662	4771.25315
HSP90AB2P	0	3.05735926	0	0	1.007606311	0
DAZ2	0	0	0	0	0	0
USP17L6P	0	0	0	0	1.007606311	0
HSP90AA5P	0	0	0	0	0	2.718182629
RP11-689P11.2	8.79046815	6.09440383	12.03521197	16.0912034	1.007606311	19.0272784
AC074389.6	0	0	0	0	0	0
RP11-404K5.2	0	0	0	0	0	0
NYNRIN	22.4645297	47.7394967	0	19.1083041	117.8899384	16.30909577
DNAJC19	417.058878	408.325057	1016.965382	827.691277	481.6358168	842.6275542
DEFB109P1B	0	0	0	0	0	0
IFITM5	1.95343737	0	0	0	0	0
OR7E161P	0	0	0	0	0	0
SMIM21	0	0	0	0	0	0
CTA-373H7.7	0	0	7.020540315	2.01140043	0	0.906060876
DEFB109P1B	0	0	0	0	0	0
DEFA7P	0	0	0	0	0	0
C18orf63	0	0	0	0	0	1.812121752
DEFA1	0	0	0	0	0	0
DOK6	5.8603121	4.06293589	0	0	0	0
HN1L	4313.23854	4041.52422	9106.282666	14472.6396	7892.086509	10254.32584
RP13-212L9.1	0	0	0	0	0	0
IGLL3P	0	0	1.012963674	0	0	0
TMEM211	0	0	0	0	0	0
SERPINB11	0	0	0	0	0	0
SERPINB4	0	2.03146794	0	0	0	0
SERPINB5	0	0	0	0	82.62371753	15.4030349
ZDHHC11B	0	0	192.3427459	88.1496239	10.75115934	166.1896859
AP000350.7	0	0	0	0	0	0
KRTAP19-8	0	0	0	0	0	0

KRTAP20-3	0	0	0	0	0	0
KRTAP20-4	0	0	0	0	0	0
KRTAP22-2	0	0	0	0	0	0
KRTAP27-1	0	0	0	0	0	0
CFAP99	0	0	4.011737323	12.0684026	4.030425245	20.83940015
EGFEM1P	5.8603121	1.01573397	3.008802992	3.01710065	0	4.530304381
GOLGA8O	0	12.7880907	0	0	0.060456379	0
CTD-2008L17.2	0	0	0	0	0	0
TMEM191C	24.7109827	24.2557273	31.40187389	72.2595605	29.90575532	43.54528571
KB-1183D5.13	0	0	0	0	0	0
RP11-400K9.2	0	0	0	0	0	0
P2RX6P	0.97671868	3.04720192	0	0	0	0
RPL23AP57	0	0	1.002934331	0	0	0
HERC2P9	545.565755	542.848864	962.2252262	847.121406	600.4930573	1274.302137
RNASE13	0	0	0	0	0	0.906060876
GYG2P1	0	0	0	0	0	0
Z69890.1	0	0	0	0	0	0
HBA1	0	1.01573397	0	5.02850108	12.4842422	4.530304381
HBM	0	0	0	0	0	0
HBZP1	0	0	0	0	0	0
TCEB3B	0	0	0	0	0	0
RP11-1084I9.1	0	0	0	0	0	0
ATP10A	0.97671868	0	0	0	0	0
ANKRD20A9P	0	0	2.587570573	7.13041453	0	0.960424529
DUXAP8	962.106972	909.701503	294.1706685	271.981566	793.631035	385.3295694
ANKUB1	0	0	0	0	0	0
TSSK2	0	0	1.002934331	0	0	0
HNRNPA1P4	12.8731523	13.5397338	18.79498936	17.6098108	22.1270346	17.94000535
CTD-2308N23.2	0	0	0	0	0	0
PRR23A	0	0	0	0	0	0
FOXL2NB	0	0	2.005868661	5.02850108	6.045637868	2.718182629
HCP5	99.6253058	95.4789934	4.011737323	3.01710065	12.09127574	41.6788003
HLA-H	51.1019215	28.4710232	1.133315794	0	11.48671195	2.065818798

HCG27	18.557655	27.4248173	26.0762926	19.1083041	15.11409467	34.43031329
RP11-93O17.2	0	0	0	0	0	0
COL6A6	0	0	0	0	7.053244179	4.530304381
H1FX-AS1	65.4401518	83.2901857	106.3110391	146.832231	88.66935539	103.2909399
RAB12	1230.66554	1234.11678	648.898512	368.086279	1289.736078	617.0274567
LRRC30	0	0	0	0	0	0
TMEM200C	0	0	0	0	0	0
PPIAP30	3.90687474	1.01573397	0	0	0	0
OR10C1	0	0	0	0	0	0
TXNRD3NB	2.39296078	0	1.002934331	0	0	0
HLA-A	11918.4781	11730.0413	1079.367956	797.520271	1002.457443	1791.010534
HACD2	1961.25112	1789.72326	6871.1031	4840.43514	6360.011037	5477.137996
CFAP44	223.668579	228.540144	764.23596	443.513795	345.6089648	808.2063016
CD200R1L	0	0	0	0	1.007606311	0
RP11-553A10.1	0.97671868	2.03146794	0	0	9.068456802	0.906060876
LNP1	14.6507803	20.3146794	58.17019118	55.3135119	29.22058303	37.14849592
OR5K3	0	0	0	0	0	0
VGLL3	196.320455	136.108352	0	0	22.16733885	14.49697402
PRSS50	0	1.01573397	1.002934331	1.00570022	0	1.812121752
KRBOX1-AS1	0	0	0	0	0	0
TRIM71	0.97671868	0	0	1.00570022	12.09127574	1.812121752
ZCWPW2	16.6042176	9.14160575	13.0381463	5.02850108	8.06085049	3.624243505
ANKRD28	5908.17132	5659.66969	1070.130931	1199.80036	2555.289605	1719.703543
COLQ	8.79046815	13.2045416	52.1525852	22.6383119	6.045637868	49.83334819
METTL6	372.129819	438.797076	432.2646965	320.818369	574.3355974	464.8092295
AC022007.5	6.83703079	6.09440383	45.13204488	59.3363127	36.27382721	55.26971345
THUMPD3-AS1	236.375689	252.917759	525.5375893	377.137581	499.38984	501.0516645
XKR4	0	0	0	1.00570022	0	0
Y_RNA	0	0	0	0	0	0
RNU6-1292P	0	0	0	0	0	0
RNVU1-7	2.83248418	3.04720192	0	71.4047153	0	8.063941798
RNU6-46P	0	0	0	0	0	0
RNU1-28P	2.83248418	3.04720192	9.698374978	0	2.126049317	8.063941798

RNU6-354P	0	0	0	0	0	0
RNU6-132P	0	0	0	0	0	0
SNORA18	0	0	0	0	0	0
RNU6-47P	0	0	0	0	0	0
RNU6-877P	0	0	0	0	0	0
RNU1-27P	2.83248418	3.04720192	9.698374978	0	2.126049317	8.063941798
SNORA57	0	0.90400324	0	0	0.372814335	2.845031151
RNU6-1286P	0	0	0	0	0	0
RNU6-841P	0	0	0	0	0	0
RNU6-25P	0	0	0	0	0	0
RNU6-431P	0	0	0	0	0	0
SNORD58A	0	0	0	0	0	0
SNORA22	0	0	0	0	0	1.812121752
RNU6-425P	0	0	0	0	0	0
RNU6-946P	0	0	0	0	0	0
RNU6-1296P	0	0	0	0	0	0
SNORD116-11	0	0	0	0	0	0
SNORD24	0	0	0	0	0	0
SNORA2A	0	0	0	0	2.015212623	0
RNU6-1336P	0	0	0	0	0	0
RNU6-477P	0	0	0	0	0	0
RNU6-338P	0	0	0	0	0	0
RNU6-741P	0	0	0	0	0	0
RNY1P5	0	0	0	0	0	0
RNU6-1264P	0	0	0	0	0	0
SNORD45C	0	0	0	0	0	0
SNORD116-14	0	0	0	0	0	0
SNORA69	0	0	0	0	0	0.906060876
RNU6-979P	0	0	0	0	0	0
RNU1-39P	0	0	0	0	0	0
RNU6-1	0	0	0	0	0.110836694	0
RNU6-969P	0	0	0	0	0	0
RNU1-63P	0	0	0	0	0	0

SNORD60	0	0	0	0	0	0
RNU6-657P	0	0	0	0	0	0
SNORA80B	0	0	0	0	0	0
SNORA22	0	0	0	2.01140043	0	2.718182629
RNU6-1062P	0	0	0	0	0	0
Y_RNA	0	0	0	0	0	0
SNORA70	0	0	0	0	0	0
RNU6-672P	0	0	0	0	0	0
Y_RNA	0	0	0	0	0	0
Y_RNA	0	0	0	0	0	0
RNU6-449P	0	0	0	0	0	0
RNU6-1279P	0	0	0	1.00570022	0	0
Y_RNA	0	0	0	0	0	0
Y_RNA	0	0	0	0	0	0
SNORA2	0	0	0	0	0	0
SNORA20	0	0	0	0	0	0
SNORA70G	0	0	0	0	0	0
Y_RNA	0	0	0	0	0	0
RNU1-1	2.83248418	3.04720192	9.698374978	0	2.126049317	8.063941798
RNU6-608P	0	0	0	0	0	0
SNORD116-17	0	0	0	0	0	0
RNU6-1039P	0	0	0	0	0	0
Y_RNA	0	0	0	0	0	0
Y_RNA	0	0	0	0	0	0
SNORA70	0	0	0	0	0	0
Y_RNA	0	0	0	0	0	0
Y_RNA	0	0	0	0	0	0
RNU6-573P	0	0	0	0	0	0
Y_RNA	0	0	0	0	0	0
RNU6-471P	0	0	0	0	0	0
Y_RNA	0	0	0	0	0	0
RNU6-945P	0	0	0	0	0	0
RNU6-32P	0	0	0	0	0	0.299000089

Y_RNA	0	0	0	0	0	0
Y_RNA	0	0	0	0	0	0
RNU6-144P	0	0	0	0	0	0
Y_RNA	0	0	0	0	0	0
SNORD21	0	0	0	0	0	0
RNU6-730P	0	0	0	0	0	0
Y_RNA	0	0	0	0	0	0
RNU6-157P	0	0	0	0	0	0
RNU6-1189P	0	0	0	0	0	0
RNU6-1036P	0	0	0	0	0	0
RNU1-109P	0	0	0	0	0	0
SNORD116-18	0	0	0	0	0	0
Y_RNA	0	0	0	0	0	0
Y_RNA	0	0	0	0	0	0
SNORA56	0	0	0	0	0	0
RNU6-442P	0	0	0	0	0	0
RNY1P8	0	0	0	0	0	0
RNU1-73P	0	0	1.002934331	0	0	0
Y_RNA	0	0	0	0	0	0
RNU6-723P	0	0	0	0	0	0
RNU6-1040P	0	0	0	0	0	0
RNU1-11P	0	0	0	0	0	0
RNU6-128P	0	0	0	0	0	0
Y_RNA	0	0	0	0	0	0
Y_RNA	0	0	0	0	0	0
Y_RNA	0	0	0	0	0.201521262	0
RNU6-1227P	0	0	0	0	0	0
RNU6-1080P	0	0	0	0	0	0
RNU6-147P	0	0	0	0	0	0
Y_RNA	0	0	0	0	0	0
RNU6-26P	0	0	0	0	0	0
Y_RNA	0	0	0	0	0	0
Y_RNA	0	0	0	0	0	0

RNU6-444P	0	0	0	0	0	0
RNU6-133P	0	0	0	0	0	0
Y_RNA	0	0	0	0	0	0
RNU6-546P	0	0	0	0	0	0
Y_RNA	0	0	0	0	0	0
Y_RNA	0	0	0	0	0	0
RNU6-1074P	0	0	0	0	0	0
RNU6-1056P	0	0	0	0	0	0
RNU6-756P	0	0	0	0	0	0
RNU6-1027P	0	0	0	0	0	0
RNU6-259P	0	0	0	0	0	0
SNORD116-9	0	0	0	0.33188107	0	0
Y_RNA	0	0	0	0	0	0
RNU6-1126P	0	0	0	0	0	0
RNU6-468P	0	0	0	0	0	0
SNORA36	0	0	0	0	0	0
RNU6-936P	0	0	0	0	0	0
RNU6-641P	0	0	0	0	0	0
Y_RNA	0	0	0	0	0	0
RNVU1-18	2.83248418	3.04720192	9.698374978	0	2.126049317	8.063941798
Y_RNA	0	0	0	0	0	0
Y_RNA	0	0	0	0	0	0
Y_RNA	0	0	0	0	0	0
RNU6-484P	0	0	0	0	0	0
RNU6-620P	0	0	0	0	0	0
RNU6-643P	0	0	0	0	0	0
RNU6-142P	0	0	0	0	0	0
RNU6-245P	0	0	0	0	0	0
RNU6-1083P	0	0	0	0	0	0
Y_RNA	0	0	0	0	0	0
RNU6-1323P	0	0	0	0	0	0
SNORD101	0	0	0	0	0	0
SNORA30	0	0	0	0	0	0

Y_RNA	0	0	0	0	0	0
RNU6-317P	0	0	0	0	0	0
RNU6-787P	0	0	0	0	0	0
SNORA6	0	0	0	0	0	0
RNU6-418P	0	0	0	0	0	0
RNU6-10P	0	0	0	0	0	0
RNU6-152P	0	0	0	0	0	0
RNU6-203P	0	0	0	0	0	0
RNU6-435P	0	0	0	0	0	0
RNU6-949P	0	0	0	0	0	0
Y_RNA	0	0	0	0	0	0
RNU6-116P	0	0	0	0	0	0
Y_RNA	0	0	0	0	0	0
RNU6-44P	0	0	0	0	0	0
RNU6-211P	0	0	0	0	0	0
SNORD37	0	0	0	0	0	0
SNORA32	0	0	0	0	0	0
RNU6-637P	0	0	0	0	0	0
RNU6-213P	0	0	0	0	0	0
Y_RNA	0	0	0	0	0	0
SNORA75	0	0	0	0	0	0
Y_RNA	0	0	0	0	0	0
RNU6-224P	0	0	0	0	0	0
Y_RNA	0	0	0	0	0	0
SNORA15	0	0	0	0	0	0
RNU6-701P	0	0	0	0	0	0
RNU6-76P	0	0	0	0	0	0
RNU6-629P	0	0	0	0	0	0
Y_RNA	0	0	0	0	0	0
RNU1-129P	0	0	0	0	0	0
Y_RNA	0	0	0	0	0	0
Y_RNA	0	0	0	0	0	0
RNU6-811P	0	0	0	0	0	0

Y_RNA	0	0	0	0	0	0
SNORA32	0	0	0	0	0	0
RNY3P7	0	0	0	0	0	0
RNU6-639P	0	0	0	0	0	0
RNU6-926P	0	0	0	0	0	0
RNU6-968P	0	0	0	0	0	0
RNU6-1293P	0	0	0	0	0	0
Y_RNA	0	0	0	0	0	0
Y_RNA	0	0	0	0	0	0
RNU6-815P	0	0	0	0	0	0
Y_RNA	0	0	0	0	0	0
SNORA10	0	0	0	0	0	0
RNU6-38P	0	0	0	0	0	0
Y_RNA	0	0	0	0	0	0
Y_RNA	0	0	0	0	0	0
RNU6-483P	0	0	0	0	0	0
Y_RNA	0	0	0	0	0	0
Y_RNA	0	0	0	0	0	0
RNU6-849P	0	0	0	0	0	0
RNU6-260P	0	0	0	0	0	0
RNU1-138P	0	0	0	0	0	0
Y_RNA	0	0	0	0	0	0
Y_RNA	0	0	0	0	0	0
RNU6-974P	0	0	0	0	0	0
RNU6-1032P	0	0	0	0	0	0
U1	1.95343737	3.04720192	0	0	1.007606311	0
RNU6V	0	0	0	0	0	0
RNU6-20P	0	0	0	0	0	0
SNORA1	0	0	0	0	0	0
RNU1-74P	0	0	0	0	0	0
RNU6-1029P	0	0	0	0	0	0
SNORA5A	0	0	0	0	0	0
RNU6-746P	0	0	0	0	0	0

Y_RNA	0	0	0	0	0	0
RNU6-409P	0	0	0	0	0	0
RNU6-413P	0	0	0	0	0	0
RNU6-206P	0	0	0	0	0	0
Y_RNA	0	0	0	0	0	0
RNU6-209P	0	0	0	0	0	0
Y_RNA	0	0	0	0	0	0
Y_RNA	0	0	0	0	0	0
RNU6-890P	0	0	0	0	0	0
SNORA18	0	0	0	0	0	0
Y_RNA	0	0	0	0	0	0
RNU6-895P	0	0	0	0	0	0
SNORA51	0	0	0	0	0	0
RNY3P5	0	0	0	0	0	0
RNU6-571P	0	0	0	0	0	0
RNU6-427P	0	0	0	0	0	0
RNU6-214P	0	0	0	0	0	0
Y_RNA	0	0	0	0	0	0
RNU6-767P	0	0	0	0	0	0
Y_RNA	0	0	0	0	0	0
RNU5A-6P	0	0	0	0	0	0
RNU6-207P	0	0	0	0	0	0
Y_RNA	0	0	0	0	0	0
RNU6-187P	0	0	0	0	0	0
RNU6-356P	0	0	0	0	0	0
SNORA70F	0	0	0	0	1.007606311	0
RNU6-398P	0	0	0	0	0	0
RNU6-533P	0	0	0	0	0	0
RNU6-761P	0	0	0	0	0	0
RNU6-1103P	0	0	0	0	0	0
SNORA51	0	0	3.008802992	3.01710065	0	0
RNU6-1310P	0	0	0	0	0	0
RNU6-190P	0	0	0	0	0	0

RNA5SP87	0	0	0	0	0	0
SNORA75	0	0	0	0	0	0
SNORA70	0	0	0	0	0	0
RNU6-1008P	0	0	0	0	0	0
RNU6-48P	0	0	0	0	0	0
RNU6-1200P	0	0	0	0	0	0
RNU6-217P	0	0	0	0	0	0
RNU6-42P	0	0	0	0	0	0
Y_RNA	0	0	0	0	0	0
RNU6-1124P	0	0	0	0	0	0
SNORA9	0	0	0	0	0	0
SNORA51	0	0	0	0	0	0
RNU6-36P	0	0	0	0	0.110836694	0
RNU6-98P	0	0	0	0	0	0
SNORA72	0	0	0	0	0	0
SNORA24	0	0	0	0	0	0
Y_RNA	0	0	0	0	0	0
RNU6-458P	0	0	0	0	0	0
RNU6-1013P	0	0	0	0	0	0
RNU1-136P	0	0	0	0	0	0
SNORA70	0	0	0	0	0	0
SNORA29	0	1.84863583	0	0	0	0
Y_RNA	0	0	0	0	0	0
Y_RNA	0	0	0	0	0	0
SNORA7	0	0	0	0	0	0
Y_RNA	0	0	0	0	0	0
RNU6-439P	0	0	0	0	0	0
RNU1-52P	0	0	0	0	0	0
RNU6-1181P	0	0	0	0	0	0
RNY1P6	0	0	0	0	0	0
RNU6-481P	0	0	0	0	0	0
RNU6-80P	0	0	0	0	0	0
RNU6-432P	0	0	0	0	0	0

RNU6-689P	0	0	0	0	0	0
Y_RNA	0	0	0	0	0	0
RNU6-1024P	0	0	0	0	0	0
Y_RNA	0	0	0	0	0.201521262	0
Y_RNA	0	0	0	0	0	0
RNU6-1042P	0	0	0	0	0	0
RNU6-4P	0	0	0	0	0.110836694	0
RNU6-514P	0	0	0	0	0	0
RNU4-52P	0	0	0	0	0	0
SNORA70B	0	0	0	0	0	0
RNU4-31P	0	0	0	0	0	0
RNU6-281P	0	0	0	0	0	0
SNORD15A	0	0	0.581701912	0	0	1.467818619
RNU6-708P	0	0	0	0	0	0
SNORA20	0	0	0	0	0	0
SNORA36A	0	0	3.008802992	1.00570022	0	0.906060876
RNU6-1324P	0	0	0	0	0	0
Y_RNA	0	0	0	0	0	0
Y_RNA	0	0	0	0	0	0
SNORA76A	0	0	0	0	1.007606311	0
RNU6-1201P	1.95343737	0	0	0	0	0
Y_RNA	0	0	0	0	0	0
SNORA70	0	0	0	0	0	0
Y_RNA	0	0	0	0	0	0
RNU6-793P	0	0	0	0	0	0
SNORA51	0	0	0	0	0	0
RNU6-74P	0	0	0	0	0	0
RNU6-675P	0	0	0	0	0	0
Y_RNA	0	0	0	0	0	0
RNU6-5P	0	0	0	0	0.110836694	0
Y_RNA	0	0	0	0	0	0
RNU6-1316P	0	0	0	0	0	0
RNU6-474P	0	0	0	0	0	0

RNU6-17P	0	0	0	0	0	0
RNU6-1145P	0	0	0	0	0	0
RNU6-1144P	0	0	0	0	0	0
RNU6-13P	0	0	0	0	0	0.299000089
SNORA7	0	0	0	0	0	0
SNORA8	0	0	0	0	0	0
Y_RNA	0	0	0	0	0	0
SNORD61	0	0	0	0	0	0
RNU6-662P	0	0	0	0	0	0
RNU6-40P	0	0	0	0	0	0
Y_RNA	0	0	0	0	0	0
RNU6-49P	0	0	0	0	0	0
RNU6-198P	0	0	0	0	0	0
U8	0	0	0	0	0	0
SNORD63	0	0	0	0	0	0
Y_RNA	0	0	0.972846301	0	0	0
RNU6-610P	0	0	0	0	0	0
RNU6-574P	0	0	0	0	0	0
Y_RNA	0	0	0	0	0	0
RNU6-842P	0	0	0	0	0	0
RNU6-524P	0	0	0	0	0	0
Y_RNA	0	0	0	0	0	0
RNU6-622P	0	0	0	0	0	0
RNU6-820P	0	0	0	0	0	0
SNORD116-2	0	0	0	0	0	0
SNORA5	0	0	0	0	0	0
RNU6-611P	0	0	0	0	0	0
RNU6-301P	0	0	0	0	0	0
RNU1-2	2.83248418	3.04720192	9.698374978	0	2.126049317	8.063941798
RNU6-891P	0	0	0	0	0	0
SNORA54	0	0	0	0	0	0
Y_RNA	0	0	0	0	0	0
RNU6-318P	0	0	0	0	0	0

RNY4P13	0	0	0	0	0	0
RNU6-72P	0	0	0	0	0	0
RNU6-804P	0	0	0	0	0	0
SNORD116-3	0	0	0	0.33188107	0	0
SNORA36C	0	0	0	0	0	0
RNU6-167P	0	0	0	0	0	0
Y_RNA	0	0	0	0	0	0
Y_RNA	0	0	0	0	0	0
SNORA51	0	0	0	0	0	0
RNU6-975P	0	0	0	0	0	0
Y_RNA	0	0	0	0	0	0
Y_RNA	0	0	0	0	0	0
RNU6-472P	0	0	0	0	0	0
SNORA67	0	0	0	0	0	0
RNU6-43P	0	0	0	0	0	0
SNORD59A	0	0	0	0	0	0
Y_RNA	0	0	0	0	0	0
RNU6-154P	0	0	0	0	0	0
Y_RNA	0	0	0	0	0	0
RNY3P14	0	0	0	0	0	0
RNU6-339P	0	0	0	0	0	0
Y_RNA	0	0	0	0	0	0
RNU6-3P	0	0	0	0	0.110836694	0
RNU6-196P	0	0	0	0	0	0
RNU6-1226P	0	0	0	0	0	0
RNU6-532P	0	0	0	0	0	0
RNU6-886P	0	0	0	0	0	0
SNORD51	0	0	0	0	0	0
Y_RNA	0	0	0	0	0	0
SNORA27	0	0	0.250733583	0	0	0
RNU6-378P	0	0	0	0	0	0
RNU6-937P	0	0	0	0	0	0
RNU1-8P	0	0	0	0	0	0

RNU6-784P	0	0	0	0	0	0
RNU6-480P	0	0	0	0	0	0
Y_RNA	0	0	0	0	0	0
SNORA15	0	0	0	0	0	0
SNORD116-1	0	0	0	0	0	0
RNU5A-2P	0	0	0	0	0	0
SNORA72	0	0	0	0	0	0
RNU6-463P	0	0	0	0	0	0
Y_RNA	0	0	0	0	0	0
RNU6-1207P	0	0	0	0	0	0
RNU6-39P	0	0	0	0	0	0
Y_RNA	0	0	0	0	0	0
Y_RNA	0	0	0	0	0	0
RNU6-1113P	0	0	0	0	0	0
RNU6-1119P	0	0	0	0	0	0
Y_RNA	0	0	0	0	0	0
RNU6-616P	0	0	0	0	0	0
RNU6-171P	0	0	0	0	0	0
RNU6-22P	0	0	0	0	0	0
SNORA72	0	0	0	0	0	0
Y_RNA	0	0	0	0	0	0
RNU6-242P	0	0	0	0	0	0
SNORA7B	0	0	0	1.00570022	0	0.906060876
RNU6-921P	0	0	0	0	0	0
RNU6-517P	0	0	0	0	0	0
Y_RNA	0	0	0	0	0	0
Y_RNA	0	0	0	0	0	0
SNORD116-8	0	0	0	0.33188107	0	0
SNORA67	0	0	0	0	0	0
RNU6-424P	0	0	0	0	0	0
RNU6-614P	0	0	0	0	0	0
SNORA70	0	0	0	0	0	0
RNU6-166P	0	0	0	0	0	0

SNORA8	0	0	0	0	0	0
Y_RNA	0	0	0	0	0	0
RNU6-434P	0	0	0	0	0	0
Y_RNA	0	0	0	0	0	0
Y_RNA	0	0	0	0	0	0
SNORD38	0	0	0	0	0	0
RNU1-106P	0.97671868	1.01573397	1.002934331	0	5.038031556	9.060608762
SNORA25	0	0	0	0	0	0
RNU6-16P	0	0	0	0	0	0
RNU6-348P	0	0	0	0	0	0
RNU6-364P	0	0	0	0	0	0
RNU6-31P	0	0	0	0	0	0
Y_RNA	0	0	0	0	0	0
SNORD14D	0	0	0.802347465	0	0	0
U3	0	0	0	0	0	0
RNU6-1230P	0	0	0	0	0	0
RNU6-591P	0	0	0	0	0	0
Y_RNA	0	0	0	0	0	0
RNU6-163P	0	0	0	0	0	0
Y_RNA	0	0	0	0	0	0
RNU6-729P	0	0	0	0	0	0
RNA5SP187	0	0	0	0	0	0
SNORA24	0	0	0	0	0	0
Y_RNA	0	0	0	0	0	0
Y_RNA	0	0	0	0	0	0
SNORD116-7	0	0	0	0	0	0
RNU6-106P	0	0	0	0	0	0.906060876
RNU6-452P	0	0	0	0	0	0
RNU6-769P	0	0	0	0	0	0
SNORD116-13	0	0	0	0	0	0
RNU6-869P	0	0	0	0	0	0
Y_RNA	0	0	0	0	0	0
Y_RNA	0.32231717	0	0	0.50285011	0.332510083	0

RNU6-1297P	0	0	0	0	0	0
SNORA18	2.04134205	0	0	0	0	0
Y_RNA	0	0	0	0	0	0
SNORA51	0	0	0	0	0	0
Y_RNA	0	0	0	0	0	0
RNU6-465P	0	0	0	0	0	0
RNU6-185P	0	0	0	0	0	0
Y_RNA	0	0	0	0	0	0
RNU6-933P	0	0	0	0	0	0
RNU1-46P	0	0	0	0	0	0
RNY1P14	0	0	0	0	0	0
RNU6-505P	0	0	0	0	0	0
RNY3P4	0	0	0	0	0	0
RNU6-929P	0	0	0	0	0	0
RNU6-1289P	0	0	0	0	0	0
Y_RNA	0	0	0	0	0	0
RNU6-549P	0	0	0	0	0	0
RNU6-905P	0	0	0	0	0	0
Y_RNA	0	0	0	0	0	0
SNORA70	0	0	0	0	0	0.262757654
SNORA68	0	0	0	0	0	0.199333393
RNU6-1340P	0	0	0	0	0	0
SNORA15	0	0	0	1.00570022	0	0
RNU6-24P	0	0	0	0	0	0
RNU6-1215P	0	0	0	0	0	0
SNORA51	0	0	0	0	0	0
RNU6-1162P	0	0	0	0	0	0
Y_RNA	0	0	0	0	0	0
SNORD116-15	0	0	0	0	0	0
RNU1-67P	0	0	0	0	0	0
Y_RNA	0	0	0	0	0	0
SNORA51	0	0	0	0	0	0
RNU6-1122P	0	0	0	0	0	0

RNU6-411P	0	0	0	0	0	0
SNORA14B	0	0	0	3.01710065	0	0
RNU1-44P	0	0	0	0	0	0
RNU6-843P	0	0	0	0	0	0
RNU6-1157P	0	0	0	0	0	0
RNA5SP122	0	0	0	0	0	0
SNORA64	0	0	0	0	0	0
Y_RNA	0	0	0	0	0	0
RNU6-809P	0	0	0	0	0	0
SNORD116-5	0	0	0	0	0	0
RNU6-649P	0	0	0	0	0	0
Y_RNA	0	0	0	0	0	0
RNU6-1026P	0	0	0	0	0	0
Y_RNA	0	0	0	0	0	0
Y_RNA	0	0	0	0	0	0
SNORD116-12	0	0	0	0	0	0
RNU6-1195P	0	0	0	0	0	0
SNORD38	0	0	0	0	0	0
RNU6-45P	0	0	0	0	0	0
RNU1-148P	0	0	0	0	0	0
RNU6-800P	0	0	0	0	0	0
RNU6-71P	0	0	0	0	0	0
RNU6-393P	0	0	0	0	0	0
RNVU1-15	1.95343737	0	0	0	0	0
RNU6-1035P	0	0	0	0	0	0
Y_RNA	0	0	0	0	0	0
RNU6-790P	0	0	0	0	0	0
Y_RNA	0	0	0	0	0	0
Y_RNA	0	0	0	0	0	0
RNU6-102P	0	0	0	0	0	0
U3	0	0	0	0	0	0
SNORA42	0	0	1.002934331	0	0	0
Y_RNA	0	0	0	0	0	0

RNU1-57P	0	0	0	0	0	0
SNORA70E	0	0	0	0	0	0
RNU6-456P	0	0	0	0	0	0
Y_RNA	0	0	0	0	0	0
RNU6-692P	0	0	0	0	0	0
RNU6-900P	0	0	0	0	0	0
Y_RNA	0	0	0	0	0	0
Y_RNA	0	0	0	0	0	0.906060876
SNORA37	0	0	0	0	0	0
RNU6-125P	0	0	1.002934331	0	0	0
Y_RNA	0	0	0	0	0	0
RNU6-110P	0	0	0	0	0	0
Y_RNA	0	0	0	0	0	0
SNORD45A	0	0	0	0	0	0
RNU6-1065P	0	0	0	0	0	0
Y_RNA	0	0	0	0	0	0
SNORA70	0	0	0	0	0	0
SNORD116-29	0	0	0	0	0	0
Y_RNA	0	0	0	0	0	0
RNU6-1005P	0	0	0	0	0	0
SNORA72	0	0	0	0	0	0
RNU6-342P	0	0	0	0	0	0
Y_RNA	0	0	0	0	0	0
RNU6-1117P	0	0	0	0	0	0
RNU6-880P	0	0	0	0	0	0
RNU6-18P	0	0	0	0	0	0
Y_RNA	0	0	0	0	0	0
RNU6-35P	0	0	0	0	0	0
RNU6-738P	0	0	0	0	0	0
SNORD116-16	0	0	0	0	0	0
RNU6-15P	0	0	0	0	0	0
Y_RNA	0	0	0	0	0	0
RNU6-1241P	0	0	0	0	0	0

RNU6-1081P	0	0	0	0	0	0
SNORA70C	0	0	0	0	0	0
RN7SKP62	0	0	0	0	0	0
RNU6-601P	0	0	0	0	0	0
Y_RNA	0	0	0	0	0	0
SNORA70	0	0	0	0	0	0
RNU6-441P	0	0	0	0	0	0
RNU6-1160P	0	0	0	0	0	0
RNA5SP284	0	0	0	0	0	0
RNU6-831P	0	0	0	0	0	0
SNORD116-24	0	0	0	0	0	0
SNORD20	0	0	0	0	0	0
Y_RNA	0	0	0	0	0	0
Y_RNA	0	0	0	0	0	0
Y_RNA	0	0	0	0	0	0
Y_RNA	0	0	0	0	0	0
RNU6-1274P	0	0	0	0	0	0
RNU6-1235P	0	0	0	0	0	0
Y_RNA	0	0	0	0	0	0
RNU6-30P	0	0	0	0	0	0
Y_RNA	0	0	0	0	0	0
Y_RNA	0	0	0	0	0	0
Y_RNA	0	0	0	0	0.201521262	0
RNU6-851P	0	0	0	0	0	0
RNU6-140P	0	0	0	0	0	0
SNORD7	0	0	0	0	0	0
RNU6-83P	0	0	0	0	0	0
SNORD56	0	0	0	0	0	0
Y_RNA	0	0	0	0	0	0
Y_RNA	0	0	0	0	0	0
Y_RNA	0	0	0	0	0	0
SNORA8	0	0	0	0	0	0
Y_RNA	0	0	0	0	0	0

RNU6-1152P	0	0	0	0	0	0
RNU6-145P	0	0	0	0	0	0
RNU6-878P	0	0	0	0	0	0
RNU4-70P	0	0	0	0	0	0
RNU6-1127P	0	0	0	0	0	0
RNU6-429P	0	0	0	0	0	0
SNORA2B	0	0	0	0	0	0
Y_RNA	0	0	0	0	0	0
Y_RNA	0	0	0	0	0	0
RNU6-1184P	0	0	0	0	0	0
Y_RNA	0	0	0	0	0	0
Y_RNA	0	0	0	0	0	0
RNU6-160P	0	0	0	0	0	0
RNU1-89P	0	0	0	0	0	0
RNU6-901P	0	0	0	0	0	0
RNY1P4	0	0	0	0	0	0
Y_RNA	0	0	0	0	0	0
RNU6-883P	0	0	0	0	0	0
RNU6-636P	0	0	0	0	0	0
Y_RNA	0	0	0	0	0	0
RNU6-73P	0	0	0	0	0	0
RNU6-1263P	0	0	0	0	0	0
RNU6-146P	0	0	0	0	0	0
RNU6-680P	0	0	0	0	0	0
RNU6-12P	0	0	0	0	0	0.299000089
RNU6-658P	0	0	0	0	0	0
RNU6-296P	0	0	0	0	0	0
RNU1-69P	0	0	0	0	0	0
RNVU1-1	0	0	0	0	0	0
RNA5SP64	0	0	0	0	0	0
Y_RNA	0	0	0	0	0	0
RNU6-225P	0	0	0	0	0	0
SNORA22	0	0	0	2.01140043	0	0

RNU6-1222P	0	0	0	0	0	0
RNU6-306P	0	0	0	0	0	0
RNVU1-17	0	0	0	0	0	0
Y_RNA	0	0	0	0	0	0
RNU6-540P	0	0	0	0	0	0
RNU6-1257P	0	0	0	0	0	0
Y_RNA	0	0	0	0	0	0
RNU6-2	0	0	0	0	0.110836694	0
RNU6-925P	0	0	0	0	0	0
RNU6-14P	0	0	0	0	0	0
RNU6-178P	0	0	0	0	0	0
RNU6-422P	0	0	0	0	0	0
Y_RNA	0	0	0	0	0	0
RNU6-939P	0	0	0	0	0	0
Y_RNA	0	0	0	0	0	0
RNU6-297P	0	0	0	0	0	0
RNU6-29P	0	0	0	0	0	0
Y_RNA	0	0	0	0	0	0
RNU6-665P	0	0	0	0	0	0
Y_RNA	0	0	0	0	0	0
Y_RNA	0	0	0	0	0	0
SNORD116-23	0	0	0	0	0	0
RNU6-748P	0	0	0	0	0	0
Y_RNA	0	0	0	0	0	0
Y_RNA	0	0	0	0	0	0
RNU6-950P	0	0	0	0	0	0
Y_RNA	0	0	0	0	0	0
Y_RNA	0	0	0	0	0	0.906060876
Y_RNA	0	0	0	0	0	0
RNU6-310P	0	0	0	0	0	0
Y_RNA	0	0	0	0	0	0
Y_RNA	0	0	0	0	0	0
RNU1-4	2.83248418	3.04720192	9.698374978	0	2.126049317	8.063941798

Y_RNA	0	0	0	0	0	0
SNORA20	0.97671868	0	0	3.01710065	0	0
RNU6-136P	0	0	0	0	0	0
RNU6-1060P	0	0	0	0	0	0
Y_RNA	0	0	0	0	0	0
RNU6-1179P	0	0	0	0	0	0
RNU6-1011P	0	0	0	0	0	0
Y_RNA	0	0	0	0	0	0
RNU6-959P	0	0	0	0	0	0
Y_RNA	0	0	0	0	0	0
Y_RNA	0	0	0	0	0	0
SNORA64	0	0	0	0	0	0
SNORA41	0	0	0	0	0	0
SNORA1	0	0	0	0	0	0
Y_RNA	0	0	0	0	0	0
SNORA8	0	0	0	0	0	0
Y_RNA	0	0	0	0	0	0
RNU6-455P	0	0	0	0	0	0
RNU6-768P	0	0	0	0	0	0
RNU6-1151P	0	0	0	0	0	0
Y_RNA	0	0	0	0	0	0
Y_RNA	0	0	0	0	0	0
SNORA67	0	0	0	0	0	0
Y_RNA	0	0	0	0	0	0
SNORD38B	0	0	0	0	0	0
RNU6-813P	0	0	0	0	9.068456802	1.812121752
Y_RNA	0	0	0	0	0.201521262	0
Y_RNA	0	0	0	0	0	0
RNU6-95P	0	0	0	0	0	0
U8	0	0	0	0	0	0
RNU6-906P	0	0	0	0	0	0
U8	0	0	0	0	0	0
RNU6-246P	0	0	0	0	0	0

RNU6-1072P	0	0	0	0	0	0
RNU6-114P	0	0	0	0	0	0
Y_RNA	0	0	0	0	0	0
Y_RNA	0	0	0	0	0	0
RNU6-541P	0	0	0	0	0	0
RNU6-21P	0	0	0	0	0	0
SNORD116-6	0	0	0	0	0	0
RNU6-417P	0	0	0	0	0	0
SNORD56B	0	0	0	0	0	0
SNORD15B	0.97671868	1.01573397	0	1.00570022	0	0.906060876
RNU6-520P	0	0	0	0	0	0
RNU6-19P	0	0	0	0	0	0
Y_RNA	0	0	0	0	0	0
RNU6-291P	0	0	0	0	0	0
RNU6-606P	0	0	0	0	0	0
RNU6-535P	0	0	0	0	0	0
RNU6-1104P	0	0	0	0	0	0
RNU6-331P	0	0	0	0	0	0
RNU6-997P	0	0	0	0	0	0
RNU6-476P	0	0	0	0	0	0
RNY3P9	0	0	0	0	0	0
RNU6-1311P	0	0	0	0	0	0
SNORD116-19	0	0	0	0	0	0
RNU6-253P	0	0	0	0	0	0
RNU6-376P	0	0	0	0	0	0
Y_RNA	0	0	0	0	0	0
Y_RNA	0	0	0	0	0	0
SNORA19	0	0	0	0	0	0
RNU6-41P	0	0	0	0	0	0
Y_RNA	0	0	0	0	0	0
RNY1P7	0	0	0	0	0	0
SNORA80E	0.97671868	0	0	0	0	0
RNU6-286P	0	0	0	0	0	0

Y_RNA	0	0	0	0	0	0
Y_RNA	0	0	0	0	0	0
RNU6-1067P	0	0	0	0	0	0
Y_RNA	0	0	0	0	0	0
RNU6-1044P	0	0	0	0	0	0
Y_RNA	0	0	0	0	0	0
RNU6-987P	0	0	0	0	0	0
RNU6-423P	0	0	0	0	0	0
RNU6-713P	0	0	0	0	0	0
SNORA46	0	0	0	0	0	0
Y_RNA	0	0	0	0	0	0
RNY3P10	0	0	0	0	0	0
SNORA7A	0	0	0	0.61347713	0	0
Y_RNA	0	0	0	0	0	0
Y_RNA	0	0	0	0	0	0
SNORD102	0	0	0	0	0	0
RNVU1-14	0	0	1.865457855	0.69393315	3.254568385	0.906060876
SNORA42	0	0	0	0	0	0
SNORA32	0	0	0	0	0	0
RNU6-1031P	0	0	0	0	0	0
RNU6-1105P	0	0	0	0	0	0
RNU6-9	0	0	0	0	0.110836694	0
RNU6-1237P	0	0	0	0	0	0
RNU6-771P	0	0	0	0	0	0
Y_RNA	0	0	0	0	0	0
RNU1-3	2.83248418	3.04720192	9.698374978	0	2.126049317	8.063941798
RNU6-555P	0	0	0	0	0	0
SNORA41	0	0	0	0	0	0
RNU6-59P	0	0	0	0	0	0
SNORA66	0	0	0	0	0	0.906060876
RNU6-33P	0	0	0	0	0.110836694	0
Y_RNA	0	0	0	0	0	0
MIR548C	0	0	0	0	0	0

MIR25	0	0	0	0	0	0
MIR217	0	0	0	0	0	0
MIR521-2	0	0	0	0	0	0
MIR99B	0	0	0	0	0	0
MIR608	0	0	0	0	0	0
MIR633	0	0	0	0	0	0
MIR647	0	0	0	0	0	0
MIR636	0	0	0	0	0	0
MIR487A	0	0	0	0	0	0
MIR578	0	0	0	0	0	0
MIR635	0	0	0	0	0	0
MIR34C	0	0	0	0	0	0
MIR23B	0	0	0	0	0	0
MIR203A	0	0	0	0	0	0
MIR433	0	0	0	0	0	0
MIR615	0	0	0	0	0	0
MIR363	0	0	0	0	0	0
MIR550A2	0	0	0	0	0	0
MIR661	0	0	0	0	0	0.906060876
MIR649	0	0	0	0	0	0
MIR587	0	0	0	0	0	0
MIR583	0	0	0	0	0	0
MIR662	0	0	0	0	0	0
MIR526B	0	0	0	0	0	0
MIR199B	0	0	0	0	0	0
MIR30B	0	0	0	0	0	0
MIR606	0	0	0	0	0	0
MIR196B	0	0	0	0	0	0
MIR181D	0	0	0	0	0	0
MIR135A2	0	0	0	0	0	0
MIR544A	0	0	0	0	0	0
MIR593	0	0	0	0	0	0
MIR508	0	0	0	0	0	0

MIR215	0	0	0	0	0	0
MIR520A	0	0	0	0	0	0
MIR181A2	0	0	0	0	0	0
MIR490	0	0	0	0	0	0
MIR124-3	0	0	0	0	0	0
MIR520E	0	0	0	0	0	0
MIR598	0	0	0	0	0	0
MIR611	0	0	0	0	0	0
MIR106A	0	0	0	0	0	0
MIR7-1	0	0	0	0	0	0.724848701
MIR206	0	0	0	0	0	0
MIR191	0	0	0	0	0	1.812121752
MIR554	0	0	0	0	0	0
MIR200A	0	0	0	0	0	0
MIR127	0	0	0	0	0	0
MIR491	0	0	0	0	0	0
MIR149	0	0	0	0	0	0
MIR604	0	0	0	0	0	0
MIR181C	0	0	0	0	0	0
MIR193A	0	0	0	0	0	0
MIR515-2	0	0	0	0	0	0
MIR515-1	0	0	0	0	0	0
MIR3074	0	0	0	0	0	0
MIR585	0	0	0	0	0	0
MIR516A2	0	0	0	0	0	0
MIR224	0	0	0	0	0	0
MIR619	0	0	0	0	0	0
MIR194-1	0	0	0	0	0	0
MIR128-2	0	0	0	0	0	0
MIR562	0	0	0	0	0	0
MIR581	0	0	0	0	0	0
MIR651	0	0	0	0	0	0
MIR526A1	0	0	0	0	0	0

MIR7-3	0	0	0	0	0	0
MIR641	0	0	0	0	0	0
MIR588	0	0	0	0	0	0
MIR505	0	0	0	0	0	0
MIR521-1	0	0	0	0	0	0
MIR499A	0	0	0	0	0	0
MIR631	0	0	0	0	0	0
MIR595	0	0	0	0	0	0
MIR99A	0	0	0	0	0	0
MIR193B	0	0	0	0	0	0
MIR510	0	0	0	0	0	0
MIR571	0	0	0	0	0	0
AC253576.1	0	0	0	0	0	0
MIR512-2	0	0	0	0	0	0
MIR512-1	0	0	0	0	0	0
MIR655	0	0	0	0	0	0
MIR153-1	0	0	0	0	0	0
MIR192	0	0	0	0	0	0
MIR138-2	0	0	0	0	0	0
MIR570	0	0	0	0	0	0
MIR28	0	0	0	0	0	0
MIR621	0	1.01573397	0	1.00570022	0	0
MIR558	0	0	0	0	0	0
MIR128-1	0	0	0	0	0	0
MIR489	0	0	0	0	0	0
MIR548AA2	0	0	0	0	0	0
MIR548A2	0	0	0	0	0	0
MIR630	0	0	0	0	0	0
MIR183	0	0	0	0	0	0
MIR592	0	0	0	0	0	0
MIR602	0	0	0	0	0	0
MIR184	0	0	0	0	0	0
MIR659	0	0	0	0	0	0.906060876

MIR573	0	0	0	0	0	0
MIR32	0	0	0	0	0	0
MIR518A2	0	0	0	0	0	0
MIR597	0	0	0	0	0	0
MIR211	0	0	0	0	0	0
MIR7-2	0	0	0	0	0	0
MIR548AA1	0	0	0	0	0	0
MIR129-1	0	0	0	0	0	0
MIR518F	0	0	0	0	0	0
MIR639	0	0	0	0	0	0
MIR141	0	0	0	0	0	0
MIR197	0	0	0	0	0	0
MIR20B	0	0	0	0	0	0
MIR525	0	0	0	0	0	0
MIR627	0	0	0	0	0	0
MIR200C	0	0	0	0	0	0
MIR584	0	0	0	0	0	0
AC009065.1	0	0	0	0	0	0
MIR572	0	0	0	0	0	0
MIR551B	0	0	0	0	0	0
MIR623	0	0	0	0	0	0
MIR555	0	0	0	0	0	0
MIR186	0	0	0	0	0	0
MIR520B	0	0	0	0	0	0
MIR222	0	0	0	0	0	0
MIR455	0	0	0	0	0	0
MIR449B	0	0	0	0	0	0
MIR556	0	0	0	0	0	0
MIR200B	0	0	0	0	0	0
MIR506	0	0	0	0	0	0
MIR218-1	0	0	0	0	0	0
MIR637	0	0	0	0.9151872	0	0
MIR517A	0	0	0	0	0	0

MIR520D	0	0	0	0	0	0
MIR657	0	0	0	0	0	0
MIR181B2	0	0	0	0	0	0
MIR520C	0	0	0	0	0	0
MIR218-2	0	0	0	0	0	0
MIR590	0	0	0	0	0	0
MIR382	0	0	0	0	0	0
MIR495	0	0	0	0	0	0
MIR10B	0	0	0	0	0	0
MIR575	0	0	0	0	0	0
MIR299	0	0	0	0	0	0
MIR553	0	0	0	0	0	0
MIR130B	0	0	0	0	0	0
MIR199A1	0	0	0	0	0	0
MIR452	0	0	0	0	0	0
MIR487B	0	0	0	0	0	0
MIR450A2	0	0	0	0	0	0
MIR580	0	0	0	0	0	0
MIR93	0	0	0	0	0	0
MIR532	0	0	0	0	0	0
MIR181A1	0	0	0	0	0	0
MIR329-1	0	0	0	0	0	0
MIR329-2	0	0	0	0	0	0
MIR617	0	0	0	0	0	0
MIR133A2	0	0	0	0	0	0
AL132780.1	7.66724167	0.50786699	1.594665586	4.686563	4.765977852	2.020515754
MIR626	0	0	0	0	0	0
MIR516A1	0	0	0	0	0	0
MIR188	0	0	0	0	0	0
MIR586	0	0	0	0	0	0
MIR568	0	0	0	0	0	0
MIR550A1	0	0	0	0	0	0
MIR642A	0	0	0	0	0	0

MIR548A1	0	0	0	0	0	0
MIR551A	0	0	0	0	0	0
MIR122	0	0	0	0	0	0
MIR15B	0	0	0	0	0	0
MIR648	0	0	0	0	0	0
MIR625	0	0	0	0	0	0
MIR150	0	0	0	0	0	0
MIR564	0	0	0	0	0	0.87887905
MIR542	0	0	0	0	0	0
MIR500A	0	0	0	0	0	0
MIR519C	0	0	0	0	0	0
MIR26A2	0	0	0	0	0	0
MIR187	0	0	0	0	0	0
MIR216A	0	0	0	0	0	0
MIR520G	0	0	0	0	0	0
MIR504	0	0	0	0	0	0
MIR646	0	0	0	0	0	0
MIR518A1	0	0	0	0	0	0
MIR599	0	0	0	0	0	0
MIR483	0	0	0	0	0	0
MIR95	0	0	0	0	0	0
MIR27A	0	0	0	0	0	0
MIR519E	0	0	0	0	0	0
MIR34B	0	0	0	0	0	0
MIR19B2	0	0	0	0	0	0
MIR605	0	0	0	0	0	0
MIR147A	0	0	0	0	0	0
MIR563	0	0	0	0	0	0
MIR124-2	0	0	0	0	0	0
MIR614	0	0	0	0	0	0
MIR105-2	0	0	0	0	0	0
MIR9-3	0	0	0	0	0	0
MIR545	0	0	0	0	0	0

MIR640	0	0	0	0	0	0
MIR519B	0	0	0	0	0	0
MIR596	0	0	0	0	0	0
MIR30A	0	0	0	0	0	0
AC244250.1	0	0	0	0	0	0
AC245028.1	0	0	0	0	0	0
AC244157.1	0	0	0	0	0	0
AC244250.2	0	0	0	0	0	0
AC244250.3	0	0	0	0	0	0
MIR650	0	0	0	0	0	0
MIR517B	0	0	0	0	0	0
MIR517C	0	0	0	0	0	0
MIR33B	0	0	0	0	0	0
AC061975.1	0	0	0	0	0	0
AL035088.1	0	0	0	0	0	0
AC108867.1	0	0	0	0	0	0
AC097639.1	0	0	0	0	0	0
MIR622	0	0	0	0	0	0
MIR520H	0	0	0	0	0	0
MIR518B	0	0	0	0	0	0
MIR125B2	0	0	0	0	0	0
MIR27B	0	0	0	0	0	0
MIR34A	0	0	0	0	0	0
MIR514A2	0	0	0	0	0	0
MIR514A3	0	0	0	0	0	0
MIR514A1	0	0	0	0	0	0
MIR498	0	0	0	0	0	0
MIR221	0	0	0	0	0	0
MIR513B	0	0	0	0	0	0
MIR513A1	0	0	0	0	0	0
MIR610	0	0	0	0	0	0
MIRLET7B	0	0	0	0	0	0
AL161652.1	0	0	0	0	0	0.906060876

MIR566	0	0	0	0	0	0
MIR559	0	0	0	0	0	0
MIR196A2	0	0	0	0	0	0
MIR516B2	0	0	0	0	0	0
MIR135A1	0	0	0	0	0	0
MIR302A	0	0	0	0	0	0
MIR632	0	0	0	0	0	0
MIR603	0	0	0	0	0	0
MIR577	0	0	0	0	0	0
MIR33A	0	0	0	0	0	0
MIR9-1	0	0	0	0	0	0
MIR654	0	0	0	0	0	0
MIR204	0	0	0	0	0	0
MIR511	0	0	0	0	0	0
MIR223	0	0	0	0	0	0
MIR567	0	0	0	0	0	0
MIR552	0	0	0	0	0	0
MIR136	0	0	0	0	0	0
MIR634	0	0	0	0	0	0
MIR574	0	0	0	0	0	0
MIR658	0	0	0	0	0.201521262	0.389606177
MIR516B1	0	0	0	0	0	0
MIR152	0	0	0	0	0	0
MIR328	0	0	0	0	0	0
MIR214	0	0	0	0	0	0
MIR561	0	0	0	0	0	0
MIR624	0	0	0	0	0	0
MIR138-1	0	0	0	0	0	0
MIR219A2	0	0	0	0	0	0
MIR579	0	0	0	0	0	0
MIR105-1	0	0	0	0	0	0
MIR656	0	0	0	0	0	0
MIR153-2	0	0	0	0	0	0

MIR496	0	0	0	0	0	0
MIR30C1	0	0	0	0	0	0
MIR569	0	0	0	0	0	0
MIR628	0	0	0	0	0	0
MIR629	0	0	0	0	0	0
MIR620	0	0	0	0	0	0
MIR507	0	0	0	0	0	0
MIR660	0	0	0	0	0	0
MIR125B1	0	0	0	0	0	0
MIR638	0	0	0	0	0	0
MIR589	0	0	0	0	0	0
MIR557	0	0	0	0	0	0
MIR181B1	0	0	0	0	0	0
MIR607	0	0	0	0	0	0
MIR154	0	0	0	0	0	0
MIR527	0	0	0	0	0	0
MIR23A	0	0	0	0	0	0
MIR519D	0	0	0	0	0	0
MIR548B	0	0	0	0	0	0
MIR613	0	0	0	0	0	0
MIR513A2	0	0	0	0	0	0
AC136932.1	0	0	0	0	0	0
MIR518E	0	0	0	0	0	0
MIR576	0	0	0	0	0	0
MIR493	0	0	0	0	0	0
MIR182	0	0	0	0	0	0
MIR601	0	0	0	0	0	0
MIR519A1	0	0	0	0	0	0
MIR134	0	0	0	0	0	0
MIR100	0	0	0	0	0	0
MIR325	0	0	0	0	0	0
MIR301A	0	0	0	0	0	0
MIR644A	0	0	0	0	0	0

MIR509-1	0	0	0	0	0	0
MIR431	0	0	0	0	0	0
MIR643	0	0	0	0	0	0
MIR549A	0	0	0	0	0	0
MIR323B	0	0	0	0	0	0
MIR503	0	0	0	0	0	0
MIR16-1	0	0	0	0	0	0
MIR125A	0	0	0	0	0	0
MIR130A	0	0	0	0	0	0
MIRLET7F2	0	0	0	0	0	0
MIR652	0	0	0	0	0	0
MIR653	0	0	0	0	0	0
MIR362	0	0	0	0	0	0
MIR140	0	0	0	0	0	0
MIR645	0	0	0	0	0	0
MIR618	0	0	0	0	0	0
MIR185	0	0	0	0	0	0
MIR199A2	0	0	0	0	0	0
MIR591	0	0	0	0	0	0
MIR485	0	0	0	0	0	0
MIR616	0	0	0	0	0	0
MIR548A3	0	0	0	0	0	0
MIR609	0	0	0	0	0	0
MIR92A2	0	0	0	0	0	0
MIR143	0	0	0	0	0	0
MIR106B	0	0	0	0	0	0
MIR320A	0	0	0	0	0	0
MIR492	0	0	0	0	0	0
SNORA40	0	0	0	0	0	0
SNORD94	0.97671868	0	0	0	0	0
SNORD73A	0	0	0	0	0	0
SNORA35	0	0	0	0	0	0
SNORD96B	0	0	0	0	0	0

SNORA49	0	0	0	0	0	1.812121752
SNORD12C	0	0	0	0	0	0
MT-TL1	0	0	0	1.00570022	0	0
SNORD83B	0	0	0	0	0	0
SNORD83A	0	0	0	0	0	0
SNORA48	0	0.47739497	0	0	0	1.721515665
SNORD105	0	0	0	0	0	0
SNORD41	0	0	0	0	0	0
MT-TF	0.97671868	0	0	0	1.007606311	0
MT-TV	0.97671868	0	0	0	0	0.906060876
MT-RNR2	313690.62	218678.281	271548.3414	300541.25	329406.4941	272788.4728
MT-TI	0	0	0	0	0	0
MT-TQ	0	1.01573397	1.002934331	1.00570022	1.007606311	0
MT-TM	0	0	0	0	0	0
MT-TW	0	0	0	0	0	0
MT-TA	5.8603121	2.03146794	3.008802992	7.03990151	4.030425245	1.812121752
MT-TN	2.93015605	0	0	4.02280086	0	0.906060876
MT-TC	0	1.01573397	0	1.00570022	0	0
MT-TY	0	0	1.002934331	0	0	0
MT-TS1	5.8603121	2.03146794	0	6.03420129	3.022818934	0.906060876
MT-TD	0	0	0	0	0	0
MT-TK	0	0	0	0	0	0
MT-TG	0	0	0	0	0	0
MT-TR	0	0	0	0	0	0
MT-TH	0	0	0	0	0	0
RNU6ATAC4P	0	0	0	0	0	0
MT-TS2	0	0	0	0	0	0
MT-TL2	0	0	0	0	0	0
MT-TE	0.97671868	1.01573397	2.005868661	0	2.015212623	0
MT-TT	0	0	1.002934331	1.00570022	0	0.906060876
MT-TP	0	0	0	0	0	0
RNU6ATAC2P	0	0	0	0	0	0
RNU6ATAC3P	0	0	0	0	0	0

MIR196A1	0	0	0	0	0	0
SNORA40	1.06462337	0	0	0	4.080805561	0
RNU6ATAC5P	0	0	0	0	0	0
RNU6ATAC26P	0	0	0	0	0	0
MIR190A	0	0	0	0	0	0
GPX3	1159.36508	1522.58522	1.002934331	0	673.0810159	162.1848968
DIO2	15.6274989	1.01573397	2.005868661	0	160.2094035	21.74546103
C11orf31	1017.74087	1020.81264	1909.586966	1721.75877	1027.758438	1413.454967
GNRHR2	12.7852476	25.3933493	23.42854597	37.210908	58.44116605	38.0545568
DIO1	0	0	10.02934331	18.1026039	8.06085049	21.74546103
AKR7L	9.18115563	2.03146794	225.4696669	140.49632	4.030425245	167.6393833
STK38L	561.613243	731.32846	884.5880797	881.999089	563.251928	1081.836686
SACM1L	1639.91067	1895.35959	1973.774763	1620.18305	747.643883	1772.255074
MT-RNR1	68908.4799	54211.7536	83335.81941	68958.8524	78789.77551	48472.44475
TSN	3325.72712	3236.12844	2618.661537	2329.2017	3829.911589	2854.09176
AC092574.1	0	0	0	0	0	0
AC018450.1	0	0	0	0	0	0
MIR320D1	0	0	0	0	0	0
AL009174.1	0	0	0	0	0	0
AC110056.1	0	0	0	0	0	0
AL359538.1	0	0	0	0	0	0
AL590726.1	0	0	0	0	0	0
MIR320E	0	0	0	0	0	0
MIR454	0	0	0	0	0	0
AC079005.1	0	0	0	0	0	0
MIR671	0	0.55865368	0	1.02581422	0	0
AC006988.1	0	0	0	0	0	0
MIR147B	4.16082159	0.76180048	0	0	0	0
MIR216B	0	0	0	0	0	0
MIR718	0	0	0	0	0	0
BX323860.1	0	0	0	0	0	0
AL121869.1	0	0	0	0	0	0
AP001525.1	0	0	0	0	0	0

AL354933.1	0	0	0	0	0	0
MIR526A2	0	0	0	0	0	0
AL121588.1	0	0	0	0	0	0
MIR711	0	0	0	0	0	0
MIR501	0	0	0	0	0	0
AC007436.1	0	0	0	0	0	0
MIR320B1	0	0	0	0	0	0
AC069286.1	0	0	0	0	0	0
AC253576.2	0	0	0	0	0	0
AL157702.1	0	0	0	0	0	0
AC103686.1	0	0	0	0	0	0
AC115099.1	0	0	0	0	0	0
AC114788.1	0	0	0	0	0	0
MIR670	0	0	0	0	0	0
AC010525.1	0	0	0	0	0	0
AC125238.1	0	0	0	0	0	0
MIR770	0	0	0	0	0	0
MIR760	0	0	0	0	0	0
AC090673.1	0	0	0	0	0	0
MIR766	0	0	0	0	0	0
MIR759	0	0	0	0	0	0
MIR769	0	0	0	0	0	0
MIR765	0	0	0	0	0	0
MIR758	0	0	0	0	0	0
MIR767	0	0	0	0	0	0
SLC48A1	253.946858	280.342576	3623.601737	1636.27425	643.8604329	1939.876336
MIR802	0	0	0	0	0	0
MIR762	0	0	0	0	0	0
IGKC	0	15.2360096	0	0	0	0
IGKJ5	0	0	0	0	0	0
IGKJ4	0	0	0	0	0	0
IGKJ3	0	0	0	0	0	0
IGKJ2	0	0	0	0	0	0

IGKJ1	0	0	0	0	0	0
IGKV4-1	0	0	0	0	0	0
IGKV5-2	0	0	0	0	0	0
IGKV6-21	0	0	0	0	0	0
IGKV2D-26	0	0	0	0	0	0
IGKV3D-20	0	0	0	0	0	0
IGKV6D-41	0	0	0	0	0	0
IGKV3D-11	0	0	0	0	0	0
IGKV1D-42	0	0	0	0	0	0
IGLV4-69	0	0	0	0	0	0
IGLV8-61	0	0	0	0	0	0
IGLV4-60	0	0	0	0	0	0
IGLV6-57	0	0	0	0	0	0
IGLV11-55	0	0	0	0	0	0
IGLV10-54	0	0	0	0	0	0
IGLV5-52	1.39670772	4.245768	8.76564605	5.26986913	13.64298945	0
IGLV1-51	0	0	1.053081047	2.12202745	3.022818934	2.953758456
IGLV1-50	0	0	0	0	0	0
IGLV5-48	0	0	0	0	0	0
IGLV1-47	0	0	0	0	0	0
IGLV7-46	0	0	0	0	0	0
IGLV5-45	0	0	0	0	0	0
IGLV1-44	0	0	0	0	0	0
IGLV7-43	0	0	0	0	0	0
IGLV1-40	0	0	0	0	0	0
IGLV5-37	0	0	0	0	0	0
IGLV1-36	0	0	0	0	0	0
IGLV2-33	0	0	0	0	0	0
IGLV3-32	0	0	0	0	0	0
IGLV3-27	0	0	0	0	0	0
IGLV3-25	0	0	0	0	0	0
IGLV2-23	0	0	0	0	0	0
IGLV3-22	0	0	0	0	0	0

IGLV3-21	0	0	0	0	0	0
IGLV3-19	0	0	0	0	0	0
IGLV2-18	0	0	0	0	0	0
IGLV3-16	0	0	0	0	0	0
IGLV2-14	0	0	0	0	0	0
IGLV3-12	0	0	0	0	0	0
IGLV2-11	0	0	0	0	0	0
IGLV3-10	0	0	0	0	0	0
IGLV3-9	0	0	0	0	0	0
IGLV4-3	0	0	0	0	0	0
IGLV3-1	0	0	0	0	0	0
IGLJ1	0	0	0	0	0	0
IGLC1	0	0	0	0	0	0
IGLJ2	0	0	0	0	0	0
IGLC2	0	0	0	0	0	0
IGLJ3	0	0	0	0	0	0
IGLC3	0	0	0	0	0	0
IGLJ4	0	0	0	0	0	0
IGLJ5	0	0	0	0	0	0
IGLJ6	0	0	0	0	0	0
KB-1572G7.3	0	0	3.139184455	4.17365589	2.095821127	1.884606622
IGLJ7	0	0	0	0	0	0
IGLC7	0	0	0	0	0	0
TRGJ2	0	0	0	0	0	0
TRGJP2	0	0	0	0	0	0
TRGC1	0	0	0	0	181.8729392	19.0272784
TRGJ1	0	0	0	0	0	0
TRGJP	0	0	0	0	0	0
TRGJP1	0	0	0	0	0	0
TRGV11	0	0	0	0	0	0
TRGV10	0	0	0	0	2.015212623	0
TRGV9	0	0	0	0	52.39552819	0
TRGV8	0	0	0	0	0	0

TRGV5	5.0496356	10.8683535	0	0	4.030425245	0
TRGV4	0	0	0	0	0	0
TRGV3	0	0	0	0	0	0
TRGV1	0	0	0	0	0	0
TRBV6-1	0	0	0	0	0	0
TRBV7-1	0	0	0	0	0	0
TRBV4-1	0	0	0	0	0	0
TRBV6-4	0	0	0	0	0	0
TRBV7-3	5.8603121	2.03146794	0	0	0	0
TRBV5-3	0	0	0	0	0	0
TRBV9	0	0	0	0	0	0
TRBV10-1	0	0	0	0	0	0
TRBV11-1	0	0	0	0	0	0
TRBV6-5	0	0	0	0	0	0
TRBV6-6	0	0	0	0	0	0
TRBV5-5	0	1.01573397	0	0	0	0
TRBV7-6	0	0	0	0	0	0
TRBV5-6	0	0	0	0	0	0
TRBV5-7	0	0	0	0	0	0
TRBV5-1	0	0	0	0	0	0
TRBV12-2	0	0	0	0	0	0
TRBV4-2	0	0	0	0	0	0
TRBV19	0	0	0	0	0	0
TRBV20-1	0	0	0	0	0	0
TRBV23-1	0	0	0	0	0	0
TRBV24-1	0	0	0	0	0	0
TRBC1	0	0	0	0	0	0
TRBV27	0	0	0	0	0	0
TRBV28	0	0	0	0	0	0
TRBJ2-1	0	0	0	0	0	0
TRBJ2-2	0	0	0	0	0	0
TRBJ2-2P	0	0	0	0	0	0
TRBJ2-3	0	0	0	0	0	0

TRBJ2-4	0	0	0	0	0	0
TRBJ2-5	0	0	0	0	0	0
TRBJ2-6	0	0	0	0	0	0
TRBJ2-7	0	0	0	0	0	0
TRBC2	1.95343737	1.01573397	0	0	0	0
TRAV2	0	0	0	0	0	0
TRAV3	0	0	0	0	0	0
TRAV4	0	0	0	0	0	0
TRAV5	0	0	0	0	0	0
TRAV6	0	0	0	0	0	0
TRAV7	0	0	0	0	0	0
TRAV8-1	0	0	0	0	0	0
TRAV9-1	0	0	0	0	0	0
TRAV10	0	0	0	0	0	0
TRAV12-1	0	0	0	0	0	0
TRAV8-2	0	0	0	0	0	0
TRAV8-3	0	0	0	0	0	0
TRAV13-1	0	0	0	0	0	0
TRAV12-2	0	0	0	0	0	0
TRAV8-4	0	0	0	0	0	0
TRAV13-2	0	0	0	0	0	0
TRAV14DV4	0	0	0	0	0	0
TRAV9-2	0	0	0	0	0	0
TRAV12-3	0	0	0	0	0	0
TRAV8-6	0	0	0	0	0	0
TRAV16	0	0	0	0	0	0
TRAV17	0	0	0	0	0	0
TRAV18	0	0	0	0	0	0
TRAV19	0	0	0	0	0	0
TRAV20	0	0	0	0	0	0
TRAV21	0	0	0	0	0	0
TRAV22	0	0	0	0	0	0
TRAV23DV6	0	0	0	0	0	0

TRDV1	0	0	0	0	0	0
TRAV24	0	0	0	0	0	0
TRAV25	0	0	0	0	0	0
TRAV26-1	0	0	0	0	0	0
TRAV8-7	0	0	0	0	0	0.906060876
TRAV27	0	0	0	0	1.007606311	0
TRAV29DV5	0	0	0	0	0	0
TRAV26-2	0	0	0	0	0	0
TRAV34	0	0	0	0	0	0
TRAV35	0	0	0	0	0	0
TRAV36DV7	0	0	0	0	0	0
TRAV38-1	0	0	0	0	0	0
TRAV38-2DV8	0	0	0	0	0	0
TRAV39	0	0	0	0	0	0
TRAV40	0	0	0	0	0	0
TRAV41	0	0	0	0	0	0
TRDV2	0	0	0	0	0	0
TRDJ1	0	0	0	0	0	0
TRDJ4	0	0	0	0	0	0
TRDJ2	0	0	0	0	0	0
TRDJ3	0	0	0	0	0	0
TRDC	0.97671868	2.03146794	0	0	0	0
TRAJ61	0	0	0	0	0	0
TRAJ59	0	0	0	0	0	0
TRAJ58	0	0	0	0	0	0
TRAJ57	0	0	0	0	0	0
TRAJ56	0	0	0	0	0	0
TRAJ54	0	0	0	0	0	0
TRAJ53	0	0	0	0	0	0
TRAJ52	0	0	0	0	0	0
TRAJ50	0	0	0	0	0	0
TRAJ49	0	0	0	0	0	0
TRAJ48	0	0	0	0	0	0

TRAJ47	0	0	0	0	0	0
TRAJ46	0	0	0	0	0	0
TRAJ45	0	0	0	0	0	0
TRAJ44	0	0	0	0	0	0
TRAJ43	0	0	0	0	0	0
TRAJ42	0	0	0	0	0	0
TRAJ41	0	0	0	0	0	0
TRAJ40	0	0	0	0	0	0
TRAJ39	0	0	0	0	0	0
TRAJ38	0	0	0	0	0	0
TRAJ35	0	0	0	0	0	0
TRAJ34	0	0	0	0	0	0
TRAJ33	0	0	0	0	0	0
TRAJ32	0	0	0	0	0	0
TRAJ31	0	0	0	0	0	0
TRAJ30	0	0	0	0	0	0
TRAJ29	0	0	0	0	0	0
TRAJ28	0	0	0	0	0	0
TRAJ27	0	0	0	0	0	0
TRAJ26	0	0	0	0	0	0
TRAJ25	0	0	0	0	0	0
TRAJ24	0	0	0	0	0	0
TRAJ23	0	0	0	0	0	0
TRAJ22	0	0	0	0	0	0
TRAJ21	0	0	0	0	0	0
TRAJ20	0	0	0	0	0	0
TRAJ19	0	0	0	0	0	0
TRAJ18	0	0	0	0	0	0
TRAJ17	0	0	0	0	0	0
TRAJ16	0	0	0	0	0	0
TRAJ14	0	0	0	0	0	0
TRAJ13	0	0	0	0	0	0
TRAJ12	0	0	0	0	0	0

TRAJ11	0	0	0	0	0	0
TRAJ10	0	0	0	0	0	0
TRAJ9	0	0	0	0	0	0
TRAJ8	0	0	0	0	0	0
TRAJ7	0	0	0	0	0	0
TRAJ6	0	0	0	0	0	0
TRAJ5	0	0	0	0	0	0
TRAJ4	0	0	0	0	0	0
TRAJ3	0	0	0	0	0	0
TRAJ2	0	0	0	0	0	0
TRAJ1	0	0	0	0	0	0
IGHA2	0	0	0	0	5.038031556	15.4030349
IGHE	0	0	27.07922693	0	249.8359849	214.7364277
IGHG4	0	0	0	0	4.030425245	0.906060876
IGHG2	0	0	0	0	2.015212623	0
IGHA1	0	0	1.002934331	0	8.06085049	61.61213958
IGHG1	0	0	0	0	3.022818934	3.578940461
IGHG3	0	0	0	1.00570022	1.007606311	2.763485672
IGHD	0	0	0	0	0	0
IGHM	0	0	0	0	2.015212623	47.11516556
IGHJ6	0	0	0	0	0	0
IGHJ2	0	0	0	0	0	0
IGHJ1	0	0	0	0	0	0
IGHD1-26	0	0	0	0	0	0
IGHD5-24	0	0	0	0	0	0
IGHD3-22	0	0	0	0	0	0
IGHD2-21	0	0	0	0	0	0
IGHD6-19	0	0	0	0	0	0
IGHD5-18	0	0	0	0	0	0
IGHD3-16	0	0	0	0	0	0
IGHD2-15	0	0	0	0	0	0
IGHD6-13	0	0	0	0	0	0
IGHD5-12	0	0	0	0	0	0

IGHD3-10	0	0	0	0	0	0
IGHD3-9	0	0	0	0	0	0
IGHD2-8	0	0	0	0	0	0
IGHD5-5	0	0	0	0	0	0
IGHD3-3	0	0	0	0	0	0
IGHD2-2	0	0	0	0	0	0
IGHV6-1	0	0	0	0	0	0
IGHV1-2	0	0	0	0	0	0
IGHV1-3	0	1.01573397	0	0	0	0
IGHV2-5	0	0	0	0	0	0
IGHV3-7	0	0	0	0	0	0
IGHV3-11	0	0	0	0	0	0
IGHV3-13	0	0	0	0	0	0
IGHV3-15	0	0	0	0	0	0
IGHV3-16	0	0	0	0	0	0
IGHV1-18	0	0	0	0	0	0
IGHV3-20	0	0	0	0	0	0
IGHV3-21	0	0	0	0	0	0
IGHV3-23	0	0	0	0	0	0
IGHV1-24	0	0	0	0	0	0
IGHV2-26	0	0	0	0	0	0
IGHV4-28	0	0	0	0	0	0
IGHV3-33	0	0	0	0	0	0
IGHV4-34	0	0	0	0	0	0
IGHV3-35	0	0	0	0	0	0
IGHV3-38	0	0	0	0	0	0
IGHV4-39	0	0	0	0	0	0
IGHV1-45	0	0	0	0	0	0
IGHV1-46	0	0	0	0	0	0
IGHV3-48	0	0	0	0	0	0
IGHV3-49	0	0	0	0	0	0
IGHV5-51	0	0	0	0	0	0
IGHV3-53	0	0	0	0	0	0

IGHV1-58	0	0	0	0	0	0
IGHV4-61	0	0	0	0	0	0
IGHV3-66	0	0	0	0	0	0
IGHV1-69	0	0	0	0	0	0
IGHV2-70	0	0	0	0	0	0
IGHV3-73	0	0	0	0	0	0
IGHV5-78	0	0	0	0	0	0
IGHV7-81	0	0	0	0	0	0
AL158035.1	0	0	0	0	0	0
AC079117.2	0	0	0	0	0	0
AC104802.1	0	0	0	0	0	0
AL117209.1	0	0	0	0	0	0
AL158141.1	0	0	0	0	0	0
AP000428.1	0	0	0	0	0	0
MIR708	0	0	0	0	0	0
AC009297.1	0	0	0	0	0	0
AL596329.1	0	0	0	0	0	0
MIR509-2	0	0	0	0	0	0
MIR509-3	0	0	0	0	0	0
MIR548U	0	0	0	0	0	0
AC080032.1	0	0	0	0	0	0
MIR550A3	0	0	0	0	0	0
AC011748.1	0	0	0	0	0	0
MIR374B	0	0	0	0	0	0
AP002498.1	0	0	0	0	0	0
AC117444.1	0	0	0	0	0	0
AC007486.1	0	0	0	0	0	0
AC084262.1	0	0	0	0	0	0
AC016575.1	0	0	0	0	0	0
AL049831.1	0	0	0	0	0	0
AL592205.1	0	0	0	0	0	0
MIR543	0	0	0	0	0	0
AL360091.1	0	0	0	0	0	0

AC109351.1	0	0	0	0	0	0
AL590007.1	0	0	0	0	0	0
MIR320C2	0	0	0	0	0	0
AL354764.1	0	0	0	0	0	0
AC122136.1	0	0	0	0	0	0
AL139318.1	0	0	0	0	0	0
AC016543.1	0	0	0	0	0	0
AC112693.1	0	0	0	0	0	0
AC244258.1	0	0	0	0	0	0
AL645941.1	0	0	0	0	0	0
AP005227.1	0	0	0	0	0	0
AC112219.1	0	0	0	0	0	0
AL162511.1	0	0	0	0	0	0
AL353147.1	0	0	0	0	0	0
AC121332.1	0	0	0	0	0	0
AC112778.1	0	0	0	0	0	0
MIR761	0	0	0	0	0	0
AL121914.1	0	0	0	0	0	0
AC105245.1	0	0	0	0	0	0
AL390119.1	0	0	0	0	0	0
AP002490.1	0	0	0	0	0	0
AC013268.1	0	0	0	0	0	0
AP000807.1	0	0	0	0	0	0
AC241377.1	0	0	0	0	0	0
AC096922.1	0	0	0	0	0	0
AL390966.1	0	0	0	0	0	0
AL035467.1	0	0	0	0	0	0
MIR764	0	0	0	0	0	0
AL080276.1	0	0	0	0	0	0
MIR301B	0	0	0	0	0	0
AC026877.1	0	0	0	0	0	0
AL121584.1	0	0	0	0	0	0
AC093267.1	0	0	0	0	0	0

TSSK1B	0	0	1.002934331	1.00570022	5.038031556	0.906060876
PRR22	67.3935892	100.557663	72.21127181	127.723927	47.80084341	113.2757307
TAS2R19	8.79046815	8.12587178	2.005868661	3.01710065	6.045637868	4.530304381
TAS2R15P	8.79046815	7.11013781	9.026408976	8.04560172	13.09888205	6.342426133
TAS2R50	0	1.01573397	2.005868661	4.02280086	2.015212623	0.906060876
TAS2R14	20.5110924	16.2517436	48.60219767	29.1653063	22.4796968	19.93333928
TAS2R13	0	4.06293589	5.014671654	2.01140043	4.030425245	1.812121752
RNU6-1168P	0	0	0	0	0	0
SNORA40	0	0	0	0	0	0
SNORD67	0	0	0	0	0	0
RNU6-696P	0	0	0	0	0	0
RNA5SP372	0	0	0	0	0	0
RNU6-1320P	0	0	0	0	0	0
U8	0	0	0	0	0	0
U8	0	0	0	0	0	0
RNU6-910P	0	0	0	0	0	0
RNU6-1106P	0	0	0	0	0	0
SNORA40	0	0	0	0	0	0
RNU1-82P	0	0	0	0	0	0
RNA5SP377	0	0	0	0	0	0
RNU6-576P	0	0	0	0	0	0
RNU6-1319P	0	0	0	0	0	0
SNORD66	0	0	0	0	0	0
RNU6-205P	0	0	0	0	0	0
SNORD64	0	0	0	0	0	0
SNORD91A	0	1.01573397	0	0	1.007606311	0
U3	0	0	0	0	0	0
RNU6-763P	0	0	0	0	0	0
SNORD78	0	0	0	0	0	0
RNU1-77P	0	0	0	0	0	0
RNA5SP86	0	0	0	0	0	0
RNU1-149P	0	0	0	0	0	0
SNORA12	0	0	0	0	0	0

RNA5SP207	0	0	0	0	0	0
SNORA48	0	0	0	0	0	0
U3	0	0	0	0	0	0
RNU6-440P	0	0	0	0	0	0
RNU6-258P	0	0	0	0	0	0
SNORA26	0	0	0	0	0	0
RNU6-328P	0	0	0	0	0	0
RNU6-298P	0	0	0	0	0	0
SNORD65	0	0	0	0	0	0
U3	0	0	1.002934331	0	2.015212623	0
RNU6-127P	0	0	0	0	0	0
RNA5SP91	0	0	0	0	0	0
Y_RNA	0	0	0	0	0	0
SNORA69	0	0	0	0	0	0
RNU6-1321P	0	0	0	0	0	0
U3	0	0	0	0	0	0
SNORA48	0	0	0	0	0	0
RNU6-913P	0	0	0	0	0	0
RNU6-816P	0	0	0	0	0	0
RNU6-604P	0	0	0	0	0	0
RNU6-220P	0	0	0	0	0	0
SNORA26	0	0	0	0	0	0
RNU6-907P	0	0	0	0	0	0
SNORA48	0	0	0	0	0	0
SNORD65	0	0	0	0	0	0
RNU6-747P	0	0	0	0	0	0
SNORD17	10.7439055	12.1888077	2.005868661	6.03420129	14.10648836	13.59091314
RNA5SP18	0	0	0	0	0	0
RNA5SP244	0	0	0.130381463	0	0.77585686	0
RNU6-930P	0	0	0	0	0	0
Y_RNA	0	0	0	0	0	0
RNA5SP219	0	0	0	0	0	0
RNU6-360P	0	0	0	0	0	0

RNU6-278P	0	0	0	0	0	0
RNU6-750P	0	0	0	0	0	0
U8	0	0	0	0	0	0
RNA5SP376	0	0	0	0	0	0
RNU6-124P	0	0	0	0	0	0
RNU6-1176P	0	0	0	0	0	0
RNA5SP176	0	0	0	0	0	0
RNU6-308P	0	0	0	0	0	0
RNU6-724P	0	0	0	0	0	0
SNORD65	0	0	0	0	0	0
RNA5SP185	0	0	0	0	0	0
SNORA40	0	0	0	0	0	0
RNU6-788P	0	0	0	0	0	0
SNORD37	0	0	0	0	0	0
SNORA40	0	0	0	0	0	0
RNA5SP292	0	0	0	0	0	0
SNORD81	0	0	0	0	0	0
SNORD77	0	0	0	0	0	0
RNA5SP256	0	0	0	0	0	0
RNU6-578P	0	0	0	0	0	0
SNORD89	0	0	1.002934331	0	0	0
RNA5SP339	0	0	0	0	0	0
RNA5SP424	0	0	0	0	0	0
RNU6-239P	0	0	0	0	0	0
SNORA16	0	0	0	0	0	0
SNORD28	0	0	0	0	0	0
SNORD72	0	0	0	0	0	0
RNU6-821P	0	0	0	0	0	0
RNU6-1009P	0	0	0	0	0	0
SNORD41	0	0	0	0	0	0
RNU6-1154P	0	0	0	0	0	0
SNORD12	0	0	0.952787614	0	0	0
RNU6-679P	0	0	0	0	0	0

Y_RNA	0	0	0	0	0	0
RNA5SP23	0	0	0	0	0	0
SNORD70	0	0	0	0	0	0
RNA5SP109	0	0	0	0	0	0
RNU6-1307P	0	0	0	0	0	0
RNU6-1228P	0	0	0	0	0	0
Y_RNA	0	0	0	0	0	0
U3	0	0	0	0	0	0
RNU6-129P	0	0	0	0	0	0
Y_RNA	0	0	0	0	0	0
RNU6-882P	0	0	0	0	0	0
RNU6-316P	0	0	0	0	0	0
RNU6-244P	0	0	0	0	0	0
RNA5SP297	0	0	0	0	0	0
RNU6-780P	0	0	0	0	0	0
RNA5SP213	0	0	0	0	0	0
Y_RNA	0	0	0	0	0	0
RNA5SP210	0	0	0	0	0	0
SNORA67	0	0	0	0	0	0
RNU6-739P	0	0	0	0	0	0
SNORA12	0	0	0	0	0	0
RNU6-823P	0	0	0	0	0	0
RNU6-700P	0	0	0	0	0	0
SNORD77	0	0	0	0	0	0
RNU6-1300P	0	0	0	0	0	0
RNU6-1242P	0	0	0	0	0	0
RNU6-837P	0	0	0	0	0	0
RNU6-550P	0	0	0	0	0	0
RNU6-1177P	0	0	0	0	0	0
SNORA40	0	0	0	0	0	0
RNA5SP332	0	0	0	0	0	0
RNU6-1246P	0	0	0	0	0	0
RNU6-1000P	0	0	0	0	0	0

RNU6-482P	0	0	0	0	0	0
SNORA46	0.97671868	0	0	1.00570022	1.007606311	0.906060876
RNA5SP171	0	0	0	0	0	0
RNU6-401P	0	0	0	0	0	0
SNORD37	0	0	0	0	0	0
SNORD78	0	0	0	0	0	0
RNU6-269P	0	0	0	0	0	0
SNORD115-45	0	0	0	0	0	0
RNU6-159P	0	0	0	0	0	0
SNORA48	0	0	0	0	0	0
SNORD113-2	0	0	0	0	0	0
RNU6-817P	0	0	0	0	0	0
RNU6-1055P	0	0	0	0	0	0
RNU6-796P	0	0	0	0	0	0
RNU6-1275P	0	0	0	0	0	0
SNORA48	0	0	0	0	0	0
Y_RNA	0	0	0	0	0	0
SNORA67	0	0	0	0	0	0
RNA5SP323	0	0	0	0	0	0
snosnR66	0	0	0	0	0	0
RNU6-1248P	0	0	0	0	0	0
SNORA74B	0.97671868	0	0	0	0	0
Y_RNA	0	0	0	0	0	0
RNU6-663P	0	0	0	0	0	0
RNY4P18	0	0	0	0	0	0
RNU6-932P	0	0	0	0	0	0
SNORD115	0	0	0	0	0	0
RNU11-3P	0	0	0	0	0	0
SNORD77	0	0	0	0	0	0
SNORD77	0	0	0	0	0	0
RNY4P36	0.97671868	0	0	0	0	0
RNU6-1111P	0	0	0	0	0	0
SNORA26	0	0	0	0	0	0

U3	0	0	0	0	0	0
RNU1-119P	0	0	0	0	0	0
RNA5SP105	0	0	0	0	0	0
SNORD115	0	0	0	0	0	0
RNU11-6P	0	0	0	0	0	0
SNORA75	0	0	0	0	0	0
RNA5SP252	0	0	0	0	0	0
U3	0	0	0	0	0	0
SNORA75	0	0	0	0	0	0
RNU6-1269P	0	0	0	0	0	0
RNU6-243P	0	0	0	0	0	0
SNORA53	1.95343737	1.01573397	3.008802992	0	2.015212623	0
SNORA48	0	0	0	0	0	0
RNU6-131P	0	0	0	0	0	0
SNORD90	0	0	0	0	0	0
Y_RNA	0	0	0	0	0	0
RNU6-241P	0	0	0	0	0	0
RNU11-4P	0	0	0	0	0	0
SNORD69	0	0	0	0	0	0
RNA5SP286	0	0	0	0	0	0
SNORA40	0	0	0	0	0	0
RNU6-644P	0	0	0	0	0	0
SNORA48	0	0	0	0	0	0
RNU6-695P	0	0	0	0	0	0
RNU6-460P	0	0	0	0	0	0
SNORA17	0	0	0	0	0	0
SNORA12	0.97671868	1.01573397	0	2.01140043	1.007606311	1.812121752
RNU6-952P	0	0	0	0	0	0
RNU6-754P	0	0	0	0	0	0
RNU6-1158P	0	0	0	0	0	0
RNU1-101P	0	0	0	0	0	0
RNU6-400P	0	0	0	0	0	0
U3	0	0	0	0	0	0

RNU6-530P	0	0	0	0	0	0
RNU6-1197P	0	0	0	0	0	0
RNU6-1109P	0	0	0	0	0	0
SNORA26	0	0	0	0	0	0
SNORD19	0	0	0	0	0	0
RNU6-332P	0	0	0	0	0	0
RNU6-1093P	0	0	0	0	0	0
RNA5SP465	0	0	0	0	0	0
SNORD86	0	0	0	0	0	0
RNA5SP300	0	0	0	0	0	0
RNA5SP299	0	0	0	0	0	0
RNU6-972P	0	0	0	0	0	0
U3	0	0	0	0	0	0
Y_RNA	0	0	0	0	2.015212623	0
RNU6-1268P	0	0	0	0	0	0
SNORA26	0	0	0	0	0	0
RNU11-5P	0	0	0	0	0	0
RNU6-1250P	0	0	0	0	0	0
RNU6-918P	0	1.01573397	0	0	0	0
RNA5SP212	0	0	0	0	0	0
RNU6-466P	0	0	0	0	0	0
RNA5SP63	0	0	0	0	0	0
SNORD115-47	0	0	0	0	0	0
SNORA57	0	0	0	0	0	0
SNORD66	0	0	0	0	0	0
SNORA75	0	0	0	0	0	0
SNORD70	0	0	0	0	0	0
RNU6-808P	0	0	0	0	0	0
RNA5SP474	0	0	0	0	0	0
U3	0	0	0	0	0	0
U3	0	0	0	0	0	0
RNU6-510P	0	0	0	0	0	0
RNA5SP496	0	0	0	0	0	0

RNU6-337P	0	0	0	0	0	0
RNU6-995P	0	0	0	0	0	0
RNA5SP354	0	0	0	0	0	0
RNU1-78P	0	0	0	0	0	0
U3	0	0	0	0	0	0
SNORD116	0	0	0	0	0	0
RNU6-192P	0	0	0	0	0	0
Y_RNA	0	0	0	0	0	0
SNORA26	0	0	0	0	0	0
RNA5SP35	0	0	0	0	0	0
RNU6-630P	0	0	0	0	0	0
RNU6-381P	0	0	0	0	0	0
RNU6-1326P	0	0	0	0	0	0
SNORA68	0	0	0	0	0	0
SNORA57	0	0	0	0	0	0
RNU6-1254P	0	0	0	0	0	0
Y_RNA	0	0	0	0	0	0
RNA5SP482	0	0	0	0	0	0
RNU6-903P	0	0	0	0	0	0
RNA5SP467	0	0	0	0	0	0
SNORA40	0	0	0	0	0	0
SNORA75	0	0	0	0	0	0
U8	0	0	0	0	0	0
RNU6-587P	0	0	0	0	0	0
SNORA8	0	0	0	0	0	0
SNORA40	0	0	0	0	0	0
SNORA26	0	0	0	0	0	0
SNORA17	0	0	0	0	0	0
SNORA26	0	0	0	0	0	0
SNORA75	0	0	0	0	0	0
U8	0	0	0	0	0	0
5S_rRNA	0	0	0	0	0	0
RNU6-876P	0	0	0	0	0	0

U3	0	0	0	0	0	0
RNU6-279P	0	0	0	0	0	0
RNA5SP53	0	0	0	0	0	0
SNORA48	0	0	0	0	0	0
RNU1-56P	0	0	0	0	0	0
SNORA45B	0	1.01573397	1.002934331	0	0	0
SNORA26	0	0	0	0	0	0
RNU1-139P	0	0	0	0	0	0
U3	0	0	0	0	0	0
SNORD30	0	0	0	0	0	0
RNU6-1239P	0	0	0	0	0	0
SNORD58	0	0	0	0	0	0
snoMBII-202	0	0	0	0	0	0
SNORA75	0	0	0	0	0	0
RNU6-493P	0	0	0	0	0	0
SNORA26	0	0	0	0	0	0
RNA5SP397	0	0	0	0	0	0
SNORA48	0	0	0	0	0	0
RNA5SP241	0	0	0	0	0	0
ZRSR1	5.00079966	0	13.23873317	0	0	0
KRTAP16-1	0	0	0	0	0	0
KRTAP29-1	0	0	0	0	0	0
KRTAP9-6	0	0	0	0	0	0
RP1-122K4.3	0	0	0	0	0	0
RP11-592N21.1	34.3511961	12.5646292	58.2203379	20.0335483	16.77664508	20.44979398
LINC01089	76.8872948	79.9992077	314.6907049	417.013594	145.8107093	260.2750473
RP11-134K1.2	0	1.01573397	0	0	0	0
CTAGE1	4.88359342	2.03146794	0	0	0	0.906060876
AP002414.1	0	0	0	0	0	0
DEFB117	0	0	0	0	0	0
C17orf51	1403.62289	1362.45476	0	0	50.38031556	12.03248844
KRTAP4-11	0	0	0	0	0	0
KRTAP4-9	0	0	0	0	0	0

KRTAP2-3	181.669675	212.2884	0	0	2.015212623	0.906060876
KRTAP2-1	0	0	0	0	0	0
RP11-563J2.3	3.90687474	3.04720192	62.1819285	6.03420129	4.030425245	14.49697402
FAM127C	659.490223	635.372072	1381.451776	1944.66216	1094.542584	1334.111216
EWSAT1	1.95343737	2.03146794	1.002934331	16.0912034	2.015212623	10.87273051
HMGN2P8	0	0	1.002934331	2.01140043	0	0
ST13P5	5.87984648	1.32045416	14.71304663	17.1170177	3.264644449	15.58424707
RPL15P3	54.002776	44.3164732	65.30105427	40.4995477	67.72122018	48.32022653
OR2A42	0.48835934	0	0	0	0	0.906060876
RPS26P3	2.5199342	0	0	5.47100917	0	3.642364722
TTTY2	0	0	0	0	0	0
TTTY2B	0	0	0	0	0	0
RNF208	108.415774	91.4160575	924.7054529	650.688039	545.1150144	1041.970008
KRTAP3-3	0	0	0	0	0	0
KRTAP3-2	0	0	0	0	0	0
KRTAP3-1	0	0	0	0	2.015212623	0
MT-ND4L	23918.8638	23642.2239	25094.41989	41312.1535	24703.48393	39225.18745
MAP10	201.204049	208.225464	14.04108063	7.03990151	182.3767423	22.6515219
RP11-414H23.2	0	0	0	0	0	0
RPL23AP4	0	0	0	0	0	0
KRTAP12-4	0	0	0	0	0	0
KRTAP10-3	0	0	0	0	0	0
KRTAP6-3	0	0	0	0	0	0
RP1-29C18.10	0	0	0	0	1.007606311	0.906060876
RP11-211N8.3	0	0	5.014671654	0	0	0
RP11-475I24.9	0.97671868	1.34076884	0	0	0	1.812121752
HNRNPA1P40	0	0	0	0	0	0
AC016747.3	111.34593	96.4947274	0	0	179.3539234	9.060608762
RP11-351E7.2	0	0	0	0	0	0
POU5F1B	0	0	60.17605984	13.0741028	2.015212623	39.86667855
RPS26P6	6.49517925	6.00298778	15.96671454	5.91351727	5.551910775	1.866485405
BTF3P12	0	0	0	0	0	0
PTTG3P	0.97671868	0	0	0	0	0

RPS15AP36	0.97671868	1.01573397	6.017605984	6.03420129	0	9.966669638
VN2R17P	0	0	0	0	0	0
ZNF580	737.823061	603.325665	1304.185716	1208.85166	385.6411635	914.4691211
AC008746.9	0	0	0	0	0	0
AL590762.11	0	0	0	1.00570022	0	0
ZNF611	442.609839	427.319282	353.8552906	480.774988	332.9937338	518.919185
KLK9	0	0	0	0	0	0
SYT3	0	0	0	0	98.19123504	11.77879139
NUP62	3826.70567	3621.01035	2427.081022	2557.48559	3586.070862	3059.713215
COX20P1	2.2269186	1.91973721	1.18346251	0	1.027758438	2.065818798
CFL1P4	0	1.01573397	0	0	0	0
RP11-108F13.2	0	0	0	0	0	0
SPHAR	0	0	0	0	0	0
CGB8	0	0	0	0	1.108366942	0
NDUFA3P3	0	0	0	0	0	0
AURKAPS1	5.85054492	2.2244574	17.69176159	11.9879466	0	7.629032577
RPL23AP80	0	0	0	0	0	0
RP11-365D23.4	0.97671868	1.01573397	0	0	4.040501308	1.812121752
RP11-383G10.3	0	0	0	0	0	0
RP11-532L16.1	0	0	0	0	0	0
EEF1A1P32	0	0	0	0	0	0
DENND1B	377.013412	367.695698	3538.352319	4856.52634	677.1114412	6904.183876
OR5S1P	0	0	0	0	0	0
HNRNPA1P34	0	0	0	0	0	0
TPM3P1	0	0	0	0	0	0
RPL5P5	0	0	0	0	0	0
EEF1B2P7	0	0	0	0	0	0
C1orf220	18.557655	15.2360096	12.03521197	8.04560172	45.34228401	19.0272784
RP4-765C7.2	0.97671868	0	3.008802992	0	0	1.812121752
RP4-612B18.3	0	2.03146794	4.011737323	0	0	0
PFN1P11	0	0	0	1.00570022	0	0
RP1-206D15.6	1.95343737	3.04720192	19.05575228	14.079803	48.36510294	28.99394804
RPL29P7	0	0	0	0	0	0

SFT2D2	1808.883	1869.96624	1246.647373	2033.52584	1800.592478	1995.146049
RP3-431P23.2	0	0	0	0	0	0
FGFR1OP	1589.092	1535.55615	654.7355898	572.082511	653.3722365	1016.428152
RP11-760D2.10	0	0	0	0	0	0.906060876
RP11-277B15.1	0	0	0	0	0	0
KRT8P40	0	0	0	0	0	0
HMGB3P6	0	0	1.002934331	0	1.007606311	0
LPAL2	89.8581189	48.7552307	3.008802992	1.00570022	8.06085049	2.718182629
RP11-288H12.3	25.3946858	28.4405512	25.07335827	64.3648138	40.30425245	55.72274388
RPL31P11	0	0	0	0	0	0
yR211F11.2	0	0	2.005868661	0	0	0
FAM106A	88.9888393	66.0227082	1.002934331	0	28.60594318	7.248487009
RP5-933K21.2	0	1.01573397	0	0	0	0
SCAF8	1782.57997	1712.52748	1523.758129	1563.89401	2062.237609	1929.529121
RP11-312J18.5	12.6875757	16.4244183	8.063592019	3.06738566	11.17435399	3.651425331
AC012362.3	0	0	0	0	0	0
PPP1R14BP2	0	1.01573397	1.002934331	3.01710065	0	0.906060876
AC010731.6	0	0	0	0	0	0
CFAP45	49.8126529	34.5349551	16.04694929	25.1425054	41.31185876	54.36365257
RP11-613F7.1	0	0	0	0	0	0
ACKR1	0	0	0	0	0	0
PDCL3P5	0	3.0675166	3.038891022	0	1.017682374	0
AC007256.5	2.93992324	1.01573397	2.005868661	3.01710065	3.022818934	1.812121752
PHBP1	0	0	0	0	0	0
ZNF254	251.016702	239.631959	358.2581723	387.818117	440.3944905	438.2707064
KRT18P68	0	0	0	0	0	0
NPM1P46	3.90687474	3.15893265	0	4.07308587	3.022818934	1.812121752
AHCYP5	0	0	0	0	0	0
BTF3L4P3	0	0	1.103227764	0	0	0
RP11-55K22.2	0	0	0	0	0	0
AC019178.2	0	0	0	0	0	0
COX5BP2	0.05860312	0.97510461	0	0	0	0
TUBB8P8	0	0	0	0	0	0

AC104131.1	0	0	0	0	0	0
RP3-352A20.1	0	0	0	0	0	0
CHCHD2P4	0	0	0	0	0	0
LIN28AP1	0	0	0	0	0	0
RP11-306O13.1	0.97671868	0	0	0	1.007606311	0
RPL23AP46	0	0	0	0	0	0
TCTEX1D2	155.298271	177.753445	873.525714	972.552336	259.9624283	575.9013535
AC092642.1	0	0	0	0	0	0
RPL32P31	0	0	0	0	0	0
EEF1DP5	0	0	2.005868661	6.03420129	0	1.812121752
YWHAZP4	4.89336061	5.07866986	0	1.00570022	1.027758438	2.718182629
PPP1R14BP5	0	0	0	0	0	0
ARF1P2	0	0	1.002934331	2.01140043	0	1.812121752
CRYGS	17.5809363	23.3618814	119.0583344	43.2451093	11.08366942	78.33802335
ELK2AP	0	0	0	3.01710065	0	0
RP11-64B16.2	0.98648587	1.02589131	1.012963674	0	20.46448418	3.669546549
CRIP1	0	0	212.5919901	197.680434	32.51545567	393.4388143
RPL23AP60	1.45531084	0	0	0	0	0
AC073465.1	0	0	0	0	0	0.906060876
CNN2P9	1.95343737	2.03146794	1.002934331	1.00570022	0	1.812121752
RP11-346C16.2	0	0	0	0	0	0
RPL7AP60	5.8603121	6.09440383	10.02934331	4.02280086	6.045637868	3.624243505
RP11-259P6.3	0	0	0	0	0	0
RP11-252O18.3	0	0	0	0	0	0
RP11-259P15.1	0	0	0	0	0	0
GAPDHP36	0	0	0	0	0	0
CEND1P1	0	0	0	0	0	0
KLHL23	985.079396	900.803673	39.23479102	61.6896512	267.2776501	117.7697927
Metazoa_SRP	0	0	0	0	0	0
RP11-393N4.2	0	0	0	0	0	0
RP11-81B10.2	0	0	0	0	0	0
LINGO4	0	0	2.005868661	11.0627024	0	9.060608762
RP1-228H13.2	0	0	0	0	0	0

RP11-373E16.1	0	0	0	0	0	0
RPL13P6	0	0	0	0	0	0
PRDX2P4	0	0	0	0	0	0
RP11-641D5.1	13.8791725	22.813385	31.52222601	25.7258115	10.69070296	11.76067017
RPL17P41	0	0	0	0	0	0
RPL36AP48	0	0	0	0	0	0
OR10N1P	0	0	0	0	0	0
OR10D5P	0	0	0	0	0	0
RPS7P8	0	0	0	0	0	0
RP11-335F8.2	0	0	0	0	0	0
FAM24B	108.376705	78.1099425	78.82060905	45.9001578	71.52997204	49.45280262
TRIM59	906.853996	892.830162	634.8574313	907.141594	764.1686265	883.1465966
COPS5P1	0	0	0	0	0	0
YBX1P4	0.97671868	0	0	2.01140043	2.015212623	0
BTF3L4P2	12.0429414	9.71041677	24.15065868	20.2246313	12.81675228	17.65912648
MLLT11	81.0676508	115.793673	1052.078113	1166.61225	84.63893015	1292.042809
AC016732.2	0	0	0	0	0	0
AC012066.1	2.93015605	1.01573397	0	0	2.015212623	0
ASIC3	127.950148	197.052391	240.7042394	229.299649	46.34989032	135.9091314
FABP5P10	0	0	0	0	0	0
GIMAP1	0	0	0	0	1.007606311	0
RP3-382I10.7	8.40954787	0	20.29939085	45.3671367	2.91198224	22.82367347
STRADBP1	0	1.01573397	0	1.00570022	1.007606311	2.718182629
AC005229.7	0	0	0	0	0	0
AC005229.5	0	0	0	0	0	0
KRT18P64	0	0	0	0	0	0
NCLP1	0	4.06293589	0	0	2.015212623	3.624243505
CCDC183	6.1630949	4.65206159	262.6685012	190.821559	15.34584412	111.0377604
ARHGEF35	217.710595	182.994632	1005.000375	967.383037	500.9415537	792.0149937
OR2F1	0	0	0	0	0	0
RP11-355O1.7	0	0	0	0	0	0
CSH2	0	0	1.002934331	2.01140043	0	1.812121752
DNLZ	163.991067	145.107755	143.4496973	210.462884	515.0681942	219.7650655

AC093724.2	5.87007929	4.06293589	7.030569658	4.02280086	7.053244179	8.082063016
AC018804.7	4.55150907	1.01573397	4.081942726	3.12772767	694.4624219	50.94780307
RP11-337C18.4	0	0	0	0	1.007606311	0.906060876
RPL12P38	0	0	0	0	15.12417073	2.718182629
TCL1B	0	0	0	0	2.015212623	0
PPP1R2P10	0	0	0	0	0	0
RPL23AP62	0	0	0	0	0	0
ST13P10	0	0	2.005868661	0	1.007606311	0.906060876
EEF1A1P16	2.822717	0	0.932728928	0	0	2.672879585
YWHAZP2	5.87007929	4.10356525	3.018832335	7.06001551	15.15439892	4.53936499
AC008155.1	0	0	0	0	0	0
AC008155.2	0	0	0	0	0	0
NPM1P32	0	0	0	0	0	0.906060876
RP11-458D21.5	265.169355	131.578179	54.34901138	103.496609	95.02735122	89.51881457
HIST2H3DP1	32.7005415	18.6590331	0	0	4.262174697	0.960424529
SUPT4H1	1234.57242	1239.19545	519.5199833	475.696202	2532.11466	680.451718
RP11-479A21.1	0	0	0	0	0	1.812121752
RBMS2P1	16.4381754	14.7484573	0	1.08615623	5.430998018	4.657152904
RP11-819M15.1	0	0	3.018832335	3.07744266	0	0.924182094
RPL12P42	0	0	0	1.00570022	3.022818934	0.906060876
YWHAZP5	1.99250612	0	0	3.06738566	0	0
EEF1B2P6	36.6855538	35.3780143	11.2328645	33.8518693	24.65612644	32.62725215
VDAC2P3	0	0	0	0	0	0
NIP7P2	0	0	0	0	0	0
TSGA13	0	0	0	0	0	0
AC004386.4	0	0	1.002934331	0	0	0
RPL6P25	0	1.01573397	0	0	0	0
RP11-613M5.1	0	0	1.002934331	1.00570022	0	0
IFITM9P	0	0	0	0	0	0
MARCKSL1P1	0	0	0	0	0	0
RP1-29C18.9	0	0	0	0	0	0
RP11-212P7.1	0	1.01573397	0	0	0	0.906060876
NRAS	4266.30721	4193.96557	1845.399169	2298.02499	13015.25072	2529.721966

RPL23AP45	0	0	0	0	0	0
RP11-680L20.1	0	0	0	0	0	0
PGK1P2	0	2.03146794	3.008802992	0	1.007606311	4.530304381
RP11-420H19.3	0	0	0	0	0	0
CTD-2666L21.3	0	0	0	0	0	0
AC092106.2	0	0	0	0	0	0
RP5-1106E3.1	0	0	0	0	0	0
ZNF625-ZNF20	68.1358954	74.8189644	0	0	115.6732045	17.84939926
HNRNPA3P6	43.5714205	51.7110165	39.55573	41.907528	94.73514539	78.00278083
HMGB3P11	0	0	0	0	0	0
RP11-560I19.2	0	0	0	0	0	0
CTC-398G3.2	0	0	0	0	1.007606311	2.718182629
CTC-398G3.1	0	0	2.005868661	2.01140043	1.007606311	0
HNRNPCP6	0.97671868	5.0888272	2.005868661	2.01140043	3.022818934	2.718182629
RPL18P11	0	0	0	0	0	0
RPL9P18	0	0	2.005868661	0	3.022818934	0
CYCSP19	0	0	0	0	0	0
RPL7AP34	0	0	0	0	0	0
CTD-3035D6.1	0.97671868	0	0	0	2.015212623	0
LTC4S	2.73481231	3.85978909	18.76490133	34.0932373	16.96809028	17.90376291
RP11-331F4.1	0	0	0	0	0	0
RPS7P11	46.0425188	42.6303548	51.83164621	30.1609495	59.2472511	35.75316217
RP11-281O15.2	0	0	0	0	0	0
RP11-713C19.2	0	0	0	0	0	0
SLC25A5P6	0	0	0	0	0	0
NPM1P50	0	0	0	0	0	0
CLNS1AP1	0	0	0	0	0	0
ANKRD39	362.391933	345.268292	420.9515973	281.787143	970.526399	368.9932918
ATF4P1	0	0.50786699	3.510270157	2.01140043	1.511409467	1.812121752
QTRT1	1058.76305	860.326674	504.4759683	411.331388	734.5450009	888.8457195
CHUK	1536.37849	1548.99431	640.8750373	893.061791	1188.975447	1143.448826
RPL21P18	0	0	0	0	0	0
PCNPP3	0	2.03146794	0	0	0	0

MXD3	531.227525	473.606279	502.831156	1018.11056	498.0396475	553.5941347
GAPDHP44	0	0	0	0	0	1.812121752
CNN2P8	0	0	0	2.01140043	0	0
AC092933.4	0	0	0	0	0	0
RP4-778K6.1	0	0	0	0	0	0
FTH1P12	0.97671868	7.12029515	12.05527066	4.06302887	3.022818934	8.163608494
RPS3P6	0.9669515	0	0	1.00570022	0	0
AP000593.6	0	0	0	0	0	0
GSTM2	123.857696	179.023113	123.3609227	61.2672571	4.171490129	38.84282976
ST13P11	0	0	0	0	0	0.906060876
RANP6	0	0	0	0	0	0
NAP1L1P3	0	1.01573397	0	0	1.007606311	0
LINC00671	0	0	0	0	0	0
GAPDHP71	0.97671868	2.03146794	0	0	0	0
COG8	1018.34643	1077.81563	1143.335108	963.350179	1591.473864	1150.325828
AC104297.1	0	0	2.005868661	0	0	0
EIF4E2P1	0	0	0	0	0	0
RP11-577H5.1	8.46815099	6.80541761	1.002934331	0	24.76696313	2.328576452
RP11-779O18.2	0	0	0	0	0	1.812121752
ARHGAP19	1332.24428	1042.14306	586.7165835	713.041453	770.8188281	737.5335532
RP11-66N11.6	0	0	7.020540315	6.03420129	0	2.718182629
RP11-546B8.1	0	0	0	0	0	0
RPSAP46	0	0	0	0	2.015212623	0
HAUS7	398.37425	398.167717	562.5358368	494.804506	902.6842661	747.0834348
LCAT	187.656961	143.797458	3.008802992	17.1874167	20.15212623	18.12121752
AC022210.2	2.99852636	0	1.002934331	9.71506408	1.118443006	3.134970632
RPL12P18	0	0	0	0	0	0
MAGEA12	0	0	2.005868661	0	0	0
PTPRCAP	0	9.61900072	0	0	2.861601924	0
CISD1P1	0	0	0	0	0	0
ANXA2P1	2.95945761	0	3.008802992	1.00570022	0	0
C1QBPP2	0.97671868	0	4.011737323	0	2.015212623	0
RBM22P2	4.88359342	1.01573397	2.005868661	1.00570022	5.038031556	7.248487009

HNRNPA1P33	59.3063585	27.4248173	0	2.01140043	6.045637868	1.821182361
PVRIG	0.97671868	0	7.110804405	6.77841945	0	10.55560921
CTB-47B8.5	0	0	0	0	0	0
KRTAP4-12	0	0	0	0	0	0
KRTAP2-4	0	0	0	0	0	0
GPC2	3.90687474	10.1573397	71.20833748	79.450317	20.15212623	76.1091136
RP11-84O12.2	0	0	0	0	0	0
RBMX2P2	0	0	0	0	0	0
KRT222	0	1.01573397	0	0	0	0
HSPD1P1	9.86485871	14.2202756	24.35124555	7.12035753	18.53995613	24.76264375
RP11-301G23.1	0	0	0	0	0	0
RP11-624L12.1	0	0	0	0	0	0
RPLP1P6	0	1.01573397	6.017605984	3.01710065	5.038031556	1.812121752
VTI1BP2	0	0	0	0	1.007606311	0
ATP6V0CP3	0	0	0	0	0	0
YBX2P1	0	0	0	0	0	0
OR5AC1	0	0	0	0	0	0
H2AFZP1	0	0	0	0	0	0
RPL18AP3	244.492221	312.602287	307.3793137	255.367399	367.9375206	247.5358314
RP11-75L1.2	21.7515251	22.0109552	64.43853075	46.3326089	36.36451177	56.53819867
SIPA1	2291.38203	2354.47135	330.9683291	428.428292	439.3163517	468.433473
RPS23P2	0	0	0	0	0	0
GAPDHP28	0	0	0	0	0	0
VDAC1P7	0	0	0	0	0	0
OR6C69P	0	0	0	0	0	0
AKR1B1P2	0	0	0	0	0	0
FTH1P3	2.93015605	2.03146794	4.031796009	4.03285786	3.184035944	12.03248844
AC091654.7	0	0	0	0	0	0
RP13-235E23.1	0	0	0	0	0	0
ERV3-1	41.0221847	36.566423	130.030436	143.422908	486.1498931	260.3565928
SYNJ2BP	1037.15804	1152.83774	2886.34471	2956.52732	1911.368716	2985.597436
ARL2	3322.79696	3110.17742	394.153192	412.337088	726.4841504	440.3455858
HMGB1P37	0	0	0	0	0	0

FIRRE	6.83703079	12.7271467	139.4580187	58.8736906	59.87196702	180.2426901
RP11-972K6.1	0	0	0	0	0	0
TTLL13P	4.88359342	4.06293589	9.858844471	13.5166109	0	3.424910112
CFL1P2	0	0	0	0	0	0
RP11-364P2.2	0	0	0	0	0	0
NDUFAF4P2	0	0	0	0	0	0
EIF4A1P8	0	0	0	0	0	0
AC108039.1	0	0	0	0	0	0
ASS1P4	0	0	0	0	0	0
CTB-85P21.1	0	0	0	0	0	0
RP13-147D17.1	0	0	0	0	0	0
NT5C3AP1	2.99852636	9.3345952	0	2.04157144	1.027758438	3.66048594
ACTN4P1	0	0	0	0	0	0
RP11-416L21.1	0	0	0	0	0	0
TPT1P13	0	0	0	0	0	0
LAP3P2	0	0	0	0	0	0
PPIAP16	1.95343737	3.04720192	1.002934331	1.00570022	0	0.906060876
GBP7	0	0	0	0	0	0
RP13-39P12.2	0.97671868	11.1730737	12.03521197	15.0955602	27.2053704	13.59091314
RP11-428P16.2	0	1.02589131	0	0	0	0
RBMXL1	671.914084	712.994462	878.6105911	818.076783	400.8358667	671.9619276
AC132008.1	0	0	21.06162094	7.03990151	0	9.060608762
RAC1P5	0	0	0	0	0	0
SRA1	789.188697	837.980527	709.0745718	448.542296	645.8756455	613.4032132
RP11-383B24.3	0	0	0	0	0	0
SETP8	0	2.03146794	0	0	1.007606311	0
RP11-591L14.1	0	0	0	0	0	0
RP11-432F4.2	0	0	0	0	0	0
MTHFD2P5	0	0	0	0	0	0
TMEM110	734.453382	1046.57166	710.4887092	571.609832	302.4229583	547.9221937
GNG5P1	0	0	0	0	0	0
KRT8P41	0	0	0	0	0	0
YBX1P6	0	0	0	0	0	0.906060876

RP11-486G15.1	0	0	1.002934331	1.00570022	0	0
RP11-467H10.1	4.2291919	5.44433409	0	3.01710065	4.645065095	5.825971434
RARRES2P2	0	0	0	0	0	0
VN1R64P	0	0	0	0	0	0
AC005522.6	0	0	0	0	0	0
AC005077.8	0	0	0	0	0	0
DNAJC9	2939.37628	2658.37895	884.537933	1167.61795	2080.707033	1551.864826
RPLP0P6	185.371439	180.272465	138.1542041	137.529504	221.0587486	107.1145168
RP11-3J11.1	0	0	0	0	0	0
RP11-240E2.2	0	0	0	0	0	0
HMGN2P32	0	0	0	0	0	0
HNRNPA1P64	0	0	0	1.00570022	1.007606311	2.718182629
RP11-386I14.3	0	0	0	0	0	0
RP11-386I14.2	0	0	0	0	0	0
C8orf82	1084.15774	1088.86682	1210.541737	1934.96721	973.3476967	1547.551977
HNRNPA1P13	0	0	0	0	0	0
RP13-383K5.4	0	4.06293589	1.002934331	3.01710065	3.04297106	2.718182629
LDHAP5	0	0	0	0	0	0
CPLX3	4.11198566	0	0	1.54877833	4.252098634	6.061547262
RP4-595K12.1	0	0	0	0	0	0
VDAC1	9394.06077	9166.96863	8261.129965	6870.87347	7746.437017	9332.390782
RP11-646D13.1	0	0	0	0	0	0
ZBTB9	667.421178	718.977135	241.7071737	183.821885	665.4635122	395.2599966
RP11-229P13.2	2.93015605	4.06293589	2.005868661	1.00570022	3.022818934	4.530304381
AP000662.9	0	0	0	0	0	0
TMX2	2762.16044	2890.77888	2483.225285	2200.46201	1686.642281	3232.825206
GAPDHP25	0	0	0	0	0	0
RP11-112J1.1	4.90312779	2.05178262	11.05233632	4.16359889	1.027758438	6.36960796
SLX1A-SULT1A3	232.283237	268.113139	644.9068333	1220.29653	1155.613602	1284.61311
XXcos-LUCA16.1	0	0	0	1.00570022	0	3.624243505
KRT18P19	0	0	0	0	0	0
RP11-444K7.8	0	0	0	0	0	0
AC111200.2	0	0	0	0	0	0

AKR1B10P1	0	0	0	0	0	0
OR4A45P	0	0	0	0	0	0
SLC25A14P1	0	0	3.008802992	3.01710065	0	0.906060876
RP11-170M17.2	0	3.04720192	3.048920365	2.01140043	2.015212623	0.924182094
FAM220CP	0	0	0	0	0	0
RP11-380G5.3	10.7439055	2.03146794	14.04108063	10.0570022	7.053244179	19.93333928
HEXA	1798.52002	2079.11603	1457.123172	1895.52365	2068.877735	1468.253529
NDUFS3	1165.84072	1242.1106	1555.671499	1462.70045	2475.305816	1620.870423
AL121657.4	0	0	0	0	0	0
RPSAP54	12.7364116	17.1659041	13.68002427	16.1314315	11.20458218	13.59091314
LEPROT	1993.26796	1650.38487	1281.679869	1551.79543	1011.19339	1189.911627
LBH	17.5809363	13.2045416	0	0	2.015212623	0.906060876
BOLA3P3	0	0	0	0	0	0
ADAT3	137.883377	153.37583	230.6748961	257.50954	169.2778603	221.0788538
PPP1CB	6073.59816	5538.39106	4983.530543	3824.36615	5945.572485	4644.096566
EEF1DP4	0	0	1.002934331	2.01140043	0	11.77879139
RPL7AP4	0	2.03146794	0	1.00570022	0	0
RP11-561N12.5	0	0	0	0	0	0
VN1R37P	0	0	0	0	0	0
SAPCD2P1	0	0	0	0	0	0
SLC25A1P3	0	0	0	0	0	0.906060876
SULT1A4	64.8052847	56.6068543	112.7599068	25.6352985	588.159956	196.1440585
RP11-760D2.7	0	0	0	0	0	0
HMGB3P30	0	0	0	0	0	0
RPL22P22	0	0	0	0	0	0
GPSM3	1259.9671	1337.72164	7.020540315	3.01710065	20.15212623	13.59091314
CTD-2347I17.1	0	0	0	0	0	0
RP11-346D6.4	0	0	0	0	0	0
LAT	9.60114466	8.16650114	43.28664571	96.4466507	35.06469963	55.48716806
RSU1P3	0	0	0	0	0	0
RP11-510I6.2	0	0	0	0	0	0
CTD-2158P22.1	0	0	0	0	0	0
RPS16P8	0	0	0	0	0	0

PGGT1BP1	0	0	0	0	0	0
RP11-476B13.2	0	0	1.002934331	0	0	0
OLA1P2	0	0	0	0	0	0
NCKIPSD	1508.05365	1267.636	754.2066167	794.50317	801.0470175	1260.330679
SLC25A5P3	0	0	0	0	0	0
ATF6B	3141.12729	3319.41862	2105.15916	1998.32633	1555.744145	2238.876425
AC002056.3	2.93015605	5.09898454	0	2.01140043	8.292599942	0.906060876
LDHBP2	3.90687474	0	0	0	0	0
TREX1	118.73969	117.916557	30.31870482	13.908834	35.14530814	32.21952476
RP11-513D4.1	0	0	0	0	0	1.812121752
SEC14L1P1	30.2782792	19.2989455	55.16138819	17.0969037	41.31185876	28.99394804
S1PR3	99.5667026	109.668797	3078.737603	4455.12121	2724.446553	2529.468269
RPS7P14	0	0	0	0	0	0
CTBP2P6	0	0	0	0	0	0
RP1-172N19.4	0	0	0	0	0	0
SLC35F6	1500.2399	1421.01183	2511.347564	1583.97784	2563.350456	1842.927822
RPL17P50	6.83703079	8.18681582	5.014671654	5.05867208	1.007606311	0.906060876
SETP22	0	0	0	0	0	0
RP5-849H19.2	0	0	0	0	0	0
EEF1A1P15	0	0	0	0	0	0
AL590762.7	0	0	2.005868661	4.02280086	0	0.906060876
HMGB1P10	2.18784985	9.11113373	6.920246882	10.4190542	11.57739652	8.154547886
SLC16A14P1	0	0	0	0	0	0
PHBP7	0	0	1.002934331	0	0	0
PIGCP1	0.97671868	2.03146794	0	3.01710065	3.022818934	0
FAM209B	1.95343737	1.01573397	4.011737323	10.0570022	2.015212623	4.530304381
FABP5P5	0	0	0	0	0	0
AC004692.5	0	0	0	0	0	0
CLIC1	19133.9092	18843.8967	4609.486184	5791.82754	7339.404371	4614.568042
HMGN2P30	0	0	0	0	0	0
DDAH2	1282.43163	1489.066	1196.500657	2037.54864	922.9673811	1314.694331
PRDX2P2	0	0	0	0	0	0
RPS2P52	0	0	0	0	0	0

LA16c-60G3.7	0	0	0	0	0	0
AC098828.3	0	2.03146794	0	0	0	0
POLD2P1	0	0	0	0	0	0
RAB5CP1	0	0	0	0	0	0
ANAPC10P1	0.97671868	1.01573397	0	1.00570022	3.022818934	0.906060876
RP11-662I13.1	0	0	0	0	0	0
AC007679.1	0	0	0	0	0	0
SERBP1P1	17.6395394	17.3081069	3.048920365	4.03285786	16.01086429	10.43782129
RPS29	5767.91452	5942.64302	9383.303158	8194.34479	6043.793948	5562.796992
ZNF337-AS1	84.9745255	83.2901857	222.6514214	119.678326	142.0724899	164.9030795
RPS10P14	0	0	0	0	0	0
RP13-278H16.2	0	0	0	0	0	0
KB-1683C8.1	0	0	0	0	0	0
CTA-268H5.9	0	0	0	0	0	0
CENPBD1P1	985.763099	1159.47049	663.5313239	880.591109	1016.039976	650.9050728
RP11-284B18.3	3.90687474	3.04720192	2.005868661	2.01140043	0	0.906060876
RP11-342F17.1	0	0	0	0	0	0
CTC-451P13.1	3.01806073	2.0213106	3.129155112	3.09755666	2.13612538	3.624243505
UGT2B11	0	0	86.2423231	9.93631813	0	70.74523321
ATP6V1G2	15.6470333	13.2248563	77.71738129	73.1646907	10.07606311	143.7374974
MT1P1	0	0	0	0	0	0
ZNF134	740.352762	798.366902	115.337448	64.3648138	614.6398499	144.0636793
ACTBP2	0.97671868	1.01573397	0	0	0	0
FAM210CP	1.95343737	1.01573397	0	0	0	0
KRT8P37	0	0	0	8.04560172	22.36886011	6.433032221
EIF1P7	0	0	0	0	0	0
AC010904.1	0	0	0	0	0	0
CTD-2224J9.8	2.3734264	1.01573397	0	0	2.015212623	1.97521271
HNRNPA1P32	0	0	0	0	0	0
RP11-452G18.1	0	0	0	0	0	0
GTF2H4	877.230119	856.375469	644.3652488	556.373473	498.744972	641.6541913
PSMC1P2	0	0	0	0	0	0
DDX47	2014.86321	1713.67526	1038.037032	1110.97691	1308.205502	1324.063001

RPL35P4	0	0	0	0	0	0
RP11-449L13.2	0	0	0	0	0	0
NHP2P2	0	0	0	0	0	0
RPL7AP38	0	0	0	0	0	0
OLA1P1	9.7964884	6.09440383	1.002934331	3.02715765	2.015212623	4.53936499
RP11-941H19.2	0	0	0	0	0	0
ZNF888	3.96547786	4.17466663	2.066044721	3.11767067	0	5.499789518
AC004129.9	0	0	0	0	0	0
ZNF845	279.59549	336.207945	289.3365251	427.311965	489.807504	484.4888717
ZNF321P	60.5565584	43.2804246	53.15551953	69.5542269	85.64653646	110.5394269
KLRK1	4.29756221	9.51742732	0	0	0	0
CNN2P4	0	0	1.002934331	2.01140043	0	0
RPL13P2	0	0	0	0	0	0
CEACAM18	0	0	0	0	0	0
CFL1P5	1.95343737	1.01573397	21.8840271	20.8280515	19.14451991	24.25524966
TMX2P1	0.97671868	2.03146794	2.045986035	5.03855808	1.098290879	3.624243505
SUGT1P2	9.76718684	6.09440383	9.026408976	3.01710065	4.030425245	7.248487009
AC098614.2	64.1118144	75.5198208	4.011737323	10.4995103	55.68032476	7.003850573
AC104306.1	0	0	0	0	0	0
RP11-324J13.1	0	0	0	0	0	0
EMP2	928.859468	953.7742	9359.383174	11167.2952	8458.854983	7647.153795
CNN2P6	0	0	0	0	0	0
VDAC1P2	6.83703079	4.06293589	7.020540315	1.00570022	6.055713931	8.734426846
RP11-12M9.4	1.95343737	0	1.002934331	0	0	0
KCTD11	402.408098	415.435195	163.4782959	242.373752	579.373629	232.8576452
RPL21P75	25.199342	45.0782737	11.25292319	12.1388016	14.45915057	36.76795036
CTD-2270N23.1	4.61011219	1.30013948	0	5.02850108	5.289933134	0
XX-C2158C12.2	0	2.43776153	1.002934331	0	0	0.906060876
EEF1B2P2	0	0	0	0	0	0
C8orf44	26.3714045	28.4405512	92.26995843	56.3192121	35.35690546	67.04850484
YBX1P10	41.7547237	46.5003012	30.01782452	29.2256483	45.98715205	36.80419279
RP11-829H16.2	0	0	0	0	0	0
TAF9BP1	0	0	0	0	0	0

AC092798.2	0	0	0	0	0	0
RP11-678D18.1	0	0	0	0	0	0
AC005019.2	0	0	0	0	0	0
RPL7AP64	0	0	0	1.00570022	0	0
CFL1P7	0	0	0	0	0	0
RPL7AP7	0	0	0	0	0	0
NPM1P6	21.4292079	19.3802042	7.020540315	12.3298846	23.17494516	3.624243505
CYB5P4	0	0	0	0	0	0
RPL13AP7	11.9745711	11.1121297	11.5337448	11.8974335	7.627579776	8.2723358
UBD	0	0	0	0	20.15212623	69.76668747
LINC01521	54.6962463	50.7866986	104.3051704	196.111542	178.3463171	158.5606533
PPM1N	14.6507803	22.3461474	12.03521197	9.05130194	30.22818934	10.87273051
RPL3P6	0	0	0	0	0	0
CEACAM16	0	0	0	0	0	0
RP11-546M4.1	0	0	0	1.00570022	0	0
RPS17P1	0	0	0	0	0	0
SLC23A3	18.9581097	21.9195391	35.47378728	44.9045146	10.46902957	55.41468319
LTB4R	749.143231	802.429838	773.262369	553.135119	552.1682586	701.2911182
LIPE-AS1	18.557655	21.3304134	37.10857024	58.3306125	19.14451991	37.14849592
LTB4R2	379.181727	353.089443	371.3564946	359.256231	388.9964925	407.7002125
CYP2A7P1	0	0	0	0	0	0
OR2G1P	0	0	0	0	0	0
RPL13P	0	0	0	0	0	0
RPL5P8	0	0	1.002934331	0	0	0
DNASE1	420.965753	410.356525	2235.540623	2119.01035	1311.903417	1742.355065
MDP1	214.946481	281.561457	245.9897033	361.91128	243.8709555	193.7248759
LEUTX	0	0	0	0	0	0
CSNK1E	3086.95847	3254.41165	4572.377614	3213.21219	1653.481957	3968.546638
HNRNPH1P2	0	0	0	0	0	0
NPM1P33	0	0	0	0	0	0
MSRB1P1	0	0	0	0	0	0
CCL27	0	2.03146794	0	5.97385928	0.332510083	2.111121841
IRF9	414.177558	339.021528	421.0819787	359.749024	259.1261151	2076.854619

GALT	544.032307	545.34757	1114.470658	1080.27289	174.3158919	730.9011876
HBE1	0	0	0	0	0	0
HBG1	0	0	0	0	0	0
AC092610.12	0	0	0	0	0	1.812121752
CLDN9	16.6042176	6.09440383	68.19953449	71.4047153	385.9132172	65.23638308
SEPHS1P6	0	0	0	0	0	1.812121752
RP11-314A20.1	0	0	0	1.00570022	0	0
RP11-94D20.1	0	0	0	0	0	0
RP1-102E24.1	0	0	0	0	0	0
KRT18P17	0	0	0	0	15.2148553	3.823576897
DNAJB1P1	0	0	0	0	0	0
ITGA1	973.866665	998.496967	3.008802992	4.02280086	22.16733885	8.154547886
RPS10P2	0	0	0	1.00570022	2.015212623	0
ATP5HP3	0	0	0	0	0	0
RP11-269M20.1	0	0	0	0	0	0
KRT18P29	0	0	0	1.00570022	3.476241774	0
RP5-1100H13.3	0	0	0	0	0	0
API5P2	0	1.01573397	0	0	0	0.906060876
AC074286.1	77.6491354	39.0752859	1.073139734	0	0	0
CHCHD4P4	0	0	0	0	0	0
NUDT19	1266.80413	1258.49439	658.9278553	576.266224	854.450152	850.7911627
ZNF726	34.1851539	26.4090833	163.4782959	125.712527	166.2550414	251.8849236
RP11-264F23.1	1.95343737	0	0	0	3.022818934	0
RP11-15H20.6	66.2605955	91.4160575	134.3932003	210.191345	86.65414277	212.9243059
RP3-522P13.2	0	0	0	0	0	0.906060876
ZNF99	0	0	0	0	0	0
CTD-2561J22.2	15.6274989	9.14160575	40.11737323	69.3933149	16.12170098	57.98789608
TAX1BP3	3416.09313	2910.75837	650.4731189	1069.89406	4152.688195	1393.095779
RPL7AP14	0	0	0	0	0	0.906060876
AC007277.3	0	0	0	0	0	0
AP1G2	1649.67786	1604.85968	2666.802385	2854.17721	1785.478384	2755.331124
AC078899.1	2.93992324	2.04162528	2.015898005	5.04861508	6.08594212	3.642364722
RP11-399E6.2	0	0	0	0	0	0

ZNF90	315.128516	350.793885	190.5274348	143.855359	25.90555826	296.6533915
RP11-414H17.5	0	0	0	0	0	0
CARKD	940.580093	836.964793	650.9043806	480.724703	1090.230029	737.5335532
TM6SF2	0	0	2.005868661	0	0	0
PGAM1P7	9.76718684	9.14160575	1.002934331	2.01140043	25.19015778	7.248487009
MEF2B	30.9619823	28.9281035	55.11124147	27.6969839	5.622443217	26.52040185
ATP5F1P3	0	0	0	0	0	0
PCNAP3	0	0	0	0	0	0
KRT18P38	0	0	2.005868661	0	9.068456802	1.812121752
GANC	388.255444	311.231046	341.2283473	409.531185	85.64653646	431.3302801
RP11-414H17.2	0	0	0	0	0	0
RPSAP61	0.99625306	1.03604865	1.002934331	2.03151444	2.065592938	0.924182094
RRM2P3	0	0	0	0	0	2.718182629
RP6-218J18.2	0	4.06293589	0	1.00570022	1.007606311	3.624243505
FTLP4	0	0	0	0	0	0
TTLL3	466.676187	476.206558	547.67235	719.306965	292.5987967	714.8457889
REPIN1	3331.53859	3491.04719	4284.535461	4267.18601	4932.132133	5236.125803
RPL23AP29	0	0	0	0	0	0
ATP5F1P4	0	0	0	0	0	0
MRPL23	845.83838	804.258159	1221.403516	861.885085	763.0904877	781.0878995
ARPC3P5	0	0	0	0	0	0
ZNF891	282.2717	273.232439	451.3204488	340.932373	405.0577371	376.9213245
RP11-169L17.3	0	0	0	0	0	0
AC073310.4	0	0	0	0	0	0
RP11-474D1.3	0	0	0	0	0	0
PGAM1P4	0	0	0	0	0	0
IFNA7	0	0	0	0	0	0
RP5-944M2.3	0	0	0	0	0	0
RP6-186E3.1	0	0	0.611789942	0	0	0
SMIM7	1834.23862	1852.59719	1451.245977	1557.66872	1464.05197	1299.291296
RPS11P7	0	0	0	0	0	0
UCA1	486.405905	407.309323	0	0	82.62371753	120.5060965
FBXO16	6.83703079	6.09440383	71.20833748	26.1482056	87.23855443	63.42426133

ARF4P3	0	0	0	0	0	0
RPL7P17	0	0	0	0	0	0
TSPAN4	1959.29768	1720.65335	1651.832843	1436.13991	302.2818934	1309.257966
RPL6P5	0	0	0	0	0	0
RP11-549L6.2	0	0	4.011737323	0	0	0
AC011999.1	0	0	0	0	2.015212623	0
RP11-31H15.2	0	0	0	0	0	0
RPL21P17	0	0	0	0	0	0
RPL23AP39	0	0	0	0	0	0
CPSF1P1	0	0	106.9830051	52.2964112	7.567123398	113.384458
GNAQP1	0	0	0	0	37.55348722	0
CPNE1	6979.3094	6266.70279	1205.527066	1562.94865	2091.548877	2416.409993
CYP4F30P	0	0	0	0	12.52454645	3.687667766
ARL16	622.169802	596.235842	321.9419202	225.276848	371.8067289	374.2031419
RP11-256K7.1	0	0	0	0	0	0
RP11-247I13.3	2.93015605	2.03146794	4.011737323	4.02280086	3.022818934	3.624243505
SMCO1	0	1.01573397	0	0	0	0.906060876
AC079776.2	0	0	0	0	3.022818934	0
WEE2	0	0	7.020540315	10.0570022	0	3.624243505
PAXIP1-AS2	95.718431	115.793673	116.3403824	103.587122	47.35749663	84.26366148
MAGEB1	0	0	0	0	0	0
TPT1P5	0	0	0	0	0	0.906060876
LDHAP4	194.46469	174.14759	88.6293068	69.5240559	92.09521685	113.3210338
RP1-258N20.3	0	0	0	0	1.007606311	0
LYRM4	774.537916	840.011995	213.6250124	181.026039	182.3767423	262.7576541
MYCBP	563.18576	453.352544	529.5493266	380.255251	1312.659122	939.8388256
PRDX1P1	0	0	0	0	1.007606311	0
SNRPEP9	0	0	0	0	0	0
RP3-412A9.15	0	0	0	0	0	0
TMEM213	0.97671868	2.03146794	2.005868661	0	1.007606311	0
RP11-420O16.2	0	1.03604865	1.012963674	1.02581422	0	0
AC024560.3	350.134114	338.798066	793.1906741	659.125864	343.1705575	682.8527793
PRCD	5.8603121	8.12587178	0	2.01140043	1.007606311	0

ACTN4P2	0	0	0	0	0	0
RPL7P60	0	0	0	0	0	0
RP11-488L18.1	0	0	0	0	0	1.812121752
LINC00887	0.97671868	0	121.355054	107.609923	5.038031556	27.18182629
RP11-699L21.1	0	0	1.002934331	0	2.015212623	0
RP11-332P22.2	0	0	0	0	0	0
ALG3	1112.48258	1294.04508	1234.612161	1024.80852	1313.91863	1450.603463
SDC4P	0	0	0	0	0	0
AMZ2P1	49.9786951	49.9639541	372.4998398	285.900457	140.2084182	240.7494354
PLEKHM1P	440.285248	519.507297	964.3614763	1058.87159	701.4652857	891.4732961
PPIAP23	0	0	0	0	0	0
PTMAP5	18.8409034	24.6010768	39.32505511	34.2943774	32.15271739	47.12422617
GCC2-AS1	15.6274989	11.1730737	7.020540315	7.03990151	9.068456802	4.530304381
XPOTP1	5.91891522	1.02589131	5.044759683	1.01575722	8.131382932	1.821182361
ST7-OT4	3.12549979	1.06652067	0	1.00570022	2.015212623	1.984273319
ZNF788	713.004639	613.503319	0	0	16.12170098	6.342426133
RNF152P1	0	1.01573397	0	0	0	0
UBE2V1P2	10.7439055	11.1730737	3.008802992	5.02850108	6.065789994	0.906060876
SH3D21	416.082159	338.818381	147.7021389	186.738416	397.7828196	350.1109832
LINC00998	511.80059	649.054008	1407.116866	1139.45834	354.6774216	739.345675
HMGN2P31	0	0	0	0	0	0
TTC41P	15.6274989	14.2202756	2.005868661	5.02850108	14.10648836	9.966669638
EEF1A1P12	55.7315681	45.7283434	51.30009102	21.9544357	44.89893723	33.74170703
TPM3P2	0	0	0	0	0	0
RPS4XP1	1.95343737	0	1.002934331	0	1.007606311	2.718182629
HNRNPA1P43	0	0	0	0	0	0
KRT18P10	0	0	0	0	8.111230806	1.812121752
RP11-208P4.1	0	0	0	0	0	0
CTAGE14P	0	0	0	0	0	0
C19orf38	0	3.04720192	100.2934331	36.2052078	15.11409467	40.77273943
C12orf74	0	0	2.005868661	0	0	0.906060876
IQCJ	0	0	0	0	0	0
TUBBP2	10.7634399	10.1878117	1.002934331	3.02715765	2.025288686	4.53936499

HNRNPA1P10	81.3802007	73.9555905	38.97402809	48.1428693	89.71726596	58.25971434
C17orf67	73.2539013	93.4475254	71.20833748	48.2736103	146.1029151	68.86062659
RP11-166N6.1	0	0	0	0	0	0
AC004980.10	0	0	0	0	0	0
SETP21	0	0	0	0	0	0.933242702
PUDPP3	0	0	0	0	0	0
CTD-3193O13.12	41.0221847	46.7237627	38.11150457	31.1767067	42.31946507	30.80606979
CTAGE11P	0.97671868	1.01573397	0	0	0	0
AZGP1P2	0	0	0	0	0	0
FIS1	829.234163	778.052223	824.4120198	641.636738	2172.399207	651.45777
AC136896.2	0	0	0	0	0	0
RPS2P6	0	0	0	0	0	0.906060876
RP11-568J23.1	0	0	0	0	0	0
ANKRD36BP1	0	0	0	0	2.015212623	0
RPSAP53	2.93015605	1.02589131	0	2.08179945	4.43346777	3.687667766
KCTD9P4	0	0	0	1.00570022	0	0
RP11-701H24.9	0	0	6.51907315	1.70969037	0	3.116849414
STARP1	0	0	0	0	0	0
RPS3AP33	0	0	0	0	0	0
LGMNP1	0.97671868	8.12587178	0	0	0	0.906060876
AGGF1P1	4.88359342	1.24935279	16.69885661	4.83741804	1.007606311	10.41970008
ANG	56.1515571	68.7651899	165.7850449	82.7288997	13.99565166	30.52519092
CTD-2228K2.2	0	0	0	0	0	0
SCART1	43.9523408	24.3776153	656.9219866	292.658763	31.23579565	429.4728553
RP11-553K23.2	0	1.03604865	2.106162094	2.09185645	0	0
HMGN2P39	0	0	0	0	0	0
KRT8P14	0	0	0	0	0	0
RAD51AP1P1	0	0	0	0	0	0
NPS	0	0	0	0	0	0
PDCL3P3	0	0	0	0	0	0
HMGB3P13	0	0	0	0	0	0
RPL39P5	1.13299367	0	1.002934331	0	3.315024764	4.90178934
COLCA2	0	1.01573397	13.0381463	14.079803	1.007606311	2.718182629

APTR	68.3703079	68.0541761	62.1819285	74.421816	85.64653646	86.07578324
FOXO1B	0	0	0	0	0	0
ALDOAP2	0	0	0	0	0	0
MRPS21P6	0	0	0	1.00570022	0	0
SPDYE3	0.97671868	0	0	0	0	1.812121752
RP11-91K8.4	0	0	0	0	0	0
Metazoa_SRP	0	0	0	0	0	0
MBLAC1	67.3935892	58.9125704	50.14671654	70.3990151	38.28903983	69.76668747
AZGP1P1	0	0	247.6144569	106.201943	0	290.6552685
ATP5G1P6	0	0	0	0	0	0
CXADRP1	0	0	0	0	0	0
CBX1P4	0	0	0	0	0	0
CBX1P2	0	0	0	0	0	0
C3orf56	0	0	0	0	0	0
RPL31P1	0	0	0	0	0	0
SLC9B1P2	0	0	0	0	0	0
ABCD1P5	0	0	0	0	0	0
RP11-252A24.2	104.381926	57.0436199	165.2234016	191.988171	322.5045521	245.2888004
CTAGE16P	0	0	0	0	0	0
FOXI3	0	1.01573397	0	0	41.31185876	0
SOGA3	76.5649776	88.561845	0	0	5.038031556	3.624243505
ATP5F1P2	0	0	0	0	0	0
OR4F13P	0	0	0	0	0	0
CTB-180A7.8	0	0	0	0	0	0
OR1X5P	0	0	0	0	0	0
VAC14-AS1	6.83703079	2.03146794	0	0	0	0
AC133644.3	0	0	0	0	0	0
NEURL1B	347.711851	367.695698	658.9278553	972.512108	1304.850173	980.357868
RPL18P10	1.95343737	3.04720192	0	7.03990151	3.022818934	6.342426133
EFCAB9	0	0	0	0	0	0
RP11-7D5.2	0	0	0	0	0	0
EIF4EP5	0	0	0	0	0	0
HAUS3	662.938039	670.049229	502.450041	422.906998	557.8410821	606.6077566

AC009967.3	0	0	0	0	0	1.812121752
RPLP2P1	0	2.03146794	0	0	0	0
VSTM5	34.1851539	13.2045416	0	1.00570022	2.015212623	0
RP11-457K10.2	0.97671868	0	0	0	0	0
LINC00488	0	0	0	0	0	0
RPS3AP26	105.954443	90.8980332	169.295315	167.187604	120.6810079	101.7506364
TUBAP2	40.3775504	55.103568	8.364472318	32.47406	73.59556498	40.07507255
KANSL1-AS1	4.88359342	7.11013781	13.0381463	9.05130194	12.09127574	1.812121752
LCNL1	0	0	0	0	0	0
RAP1BP2	0	0	0	0	0	0
RP11-221J22.1	55.5069228	103.889271	0	0	22.94319571	10.38345764
BBIP1	264.690763	289.484182	332.9741978	237.345251	296.2362555	154.030349
TRIM77	0	0	0	0	0	0
GNAT3	0	0	0	0	0	0.906060876
KRT18P13	0	0	0	0	0	0.906060876
FAM149B1P1	0	0	0	0	0	0
LRRC37A4P	893.150633	786.015577	0	0	74.28073727	14.30670123
NPM1P10	0	0	0	0	0	0
CYCSP6	0	0	0	0	5.038031556	0.906060876
AC068831.10	1.95343737	0	4.011737323	3.01710065	11.08366942	2.718182629
GOLGA2P8	0	0	0	0	0	0
NIFKP1	0	0	0	0	0	0
AS3MT	135.763897	93.5084695	5.014671654	0	0	0
FAM185BP	4.06314972	2.09241198	0	3.78143281	13.55230489	2.92657663
FAM187A	51.4535403	39.1260726	37.539832	38.8401423	40.35463277	80.48538763
RCN1P2	70.4995546	63.564632	24.92291812	9.19209997	5.612367154	14.81409533
PLIN5	3.90687474	0	5.15508246	2.41368052	40.30425245	10.87273051
RPL7P29	0	0	0	0	0	0
TPT1P6	0.97671868	0	0	0	0	0
SMARCE1P6	0	0	3.008802992	5.02850108	1.007606311	0
RPSAP28	0	0	0	0	0	0
RPL7P1	126.123684	117.469634	264.9752502	190.75116	139.5635502	183.5316911
OR7E148P	0	0	0	0	0	0

SEC14L6	31.2549979	40.5176282	0	0	7.053244179	2.718182629
SPINK13	0	0	0	0	0	0
HIGD1C	0	0	0	0	0	0
NOTO	0	0	0	0	0	0
KRT42P	0	0	1.002934331	1.00570022	0	0.906060876
PPME1	5857.38195	3140.64944	1470.301729	1437.14561	1549.698507	1920.849057
KRTAP2-2	0	0	0	0	0	0
AC130709.1	0	0	0	0	0	0
AP000343.1	0	0	0	0	0	0
STARD10	690.540109	703.903643	10710.33572	10471.3506	12863.10217	6332.459464
KRT18P33	0	1.01573397	1.002934331	0	0	0
ZNF705E	1.95343737	0	15.04401496	23.131105	3.022818934	10.87273051
RPS15AP1	2.0804108	0	0	0	0	0
RPS4XP3	0	2.04162528	2.015898005	0	0	0
GTF2IRD2P1	0	7.2117112	4.493145802	18.806594	1.592017972	8.933760239
AC087491.2	0	0	0	0	0	0.906060876
MEG3	0	0	0	0	1.007606311	0.906060876
SDHCP2	0	0	0	0	0	0
COPS8P2	0.97671868	0	1.002934331	0	0	0
LRRC37A11P	4.88359342	1.01573397	4.011737323	1.00570022	0	9.060608762
RP11-74E24.2	2.38319359	6.53116944	14.81334006	9.12170096	8.201915374	17.28764152
RP11-323J4.1	0.97671868	1.01573397	0	4.02280086	0	0
RPL21P41	0	0	0	0	0	0
RBBP4P4	0	0	1.002934331	0	0	0.915121485
NUTM2D	230.466541	272.80583	101.4267489	41.6058179	48.89913429	54.15525857
GAPDHP15	0	0	0	0	0	0
CPEB1	0.97671868	5.07866986	18.05281795	0	8.06085049	7.248487009
HMGN2P15	0	0	1.022993017	0	0	0
ZNF971P	0	0	0	0	0	0.906060876
PGGT1BP2	0	0	0	0	0	0
RP11-65N13.6	0	0	0	0	1.007606311	0
EML6	301.806073	284.405512	421.2324189	635.602536	51.38792188	624.2759437
CTBP2P5	0	0	0	0	0	0

NPM1P36	0	0	0	0	0	0
ADAM24P	0	0	0	0	0	0
RPS19P1	0	0	0	5.02850108	1.007606311	0.906060876
RP11-989E6.3	0	0	0	0	0	0
SLC6A10P	0	0	1.002934331	0	0	0
POLR3DP1	0	1.01573397	5.014671654	1.00570022	1.007606311	1.812121752
RP11-392M9.2	0	0	0	0	0	0
RPSAP6	0	2.03146794	1.002934331	0	3.022818934	0.906060876
RP11-793K1.1	0	0	1.002934331	0	0	0
DEFB113	0	0	0	0	0	0
DEFB133	0	0	0	0	0	0
RP11-114H24.4	0	0	0	0	0	0
RP11-83B20.1	3.90687474	1.01573397	0	0	0	0.906060876
RP11-477J21.2	0	0	0	0	0	0
ZNF727	9.83555715	7.11013781	0	0	2.025288686	0
HNRNPA3P3	5.58683087	2.88468448	0	2.85618861	3.919588551	5.309516734
RP11-27I1.4	76.1840573	118.840875	57.16725685	37.210908	102.7758438	42.58486118
ZSWIM8	1276.36621	1425.07476	3737.805869	4159.57609	1231.234456	3110.506988
RP11-120J4.1	0	0	0	0	0	0
KRT8P26	0	0	0	0	0	0
SLC29A4P2	0	0	0	0	0	0
SLC29A4P1	0	0	0	0	0	0
RP11-18B16.1	0	0	0	0	0	0
RPL6P12	0	0	0	0	0	0
RPL9P16	0	0	1.002934331	0	0	0
IQCF5	0	0	0	0	0	0
AC003045.1	0	0	0	0	0	0
IQCF6	0	0	0	0	0	0
C10orf105	0	0	0	0	0	0
AC104654.1	0	0	0	0	0	0
ARHGEF33	1.95343737	5.07866986	22.06455528	8.04560172	0	9.966669638
NPAP1P2	0	0	0	0	0	0
C12orf71	0	0	0	0	0	0

RP11-247C2.1	0	0	0	0	0	0
IFRD2	3343.30805	3047.20192	1234.612161	1364.73519	2035.364749	1641.782308
AC090616.2	15.6274989	11.1730737	146.4284123	98.5586211	7.053244179	68.86062659
CAPN14	0.97671868	2.03146794	12.03521197	3.01710065	8.06085049	10.87273051
ZBED1	448.284574	426.547324	161.8635716	131.314277	450.7627594	142.5505576
AC005562.1	222.096062	271.068925	87.67651919	79.359804	54.20921955	78.53735675
KRT18P49	0	0	0	0	0	0
CDIPT-AS1	0.97671868	1.01573397	0	1.00570022	0	0.906060876
RPL5P35	0	0	0	0	0	0
RP1-139D8.6	0	0	0	0	0	0
RP11-429J17.8	0	0	16.04694929	23.131105	4.030425245	19.93333928
TOMM6	4228.13704	4559.21335	1222.41648	1445.684	1856.615389	1438.407883
MRPS5P3	0	0	0	0	0	0
RP11-54I5.1	0	0	1.002934331	4.02280086	1.007606311	0
RP11-332E4.1	0	0	0	0	0	0
HNRNPUL2	4001.65552	3539.59927	3812.133332	3251.33828	3188.076445	3702.989255
AC004870.5	0	0	0	0	0	0
METTL12	102.555462	98.637926	100.2934331	74.421816	151.7757387	104.0701522
CTD-2509G16.1	0	0	0	0	0	0
RPL21P1	0	0	0	0	0	0
HNRNPA1P15	0	1.01573397	0	0	0	0
SEPT7P2	150.414677	136.138824	356.3124797	240.724404	231.0743554	267.0161402
RP11-544I20.2	7.81374947	13.2045416	17.04988362	11.9980036	38.02706219	11.90563991
RP11-174G6.1	0	0	0	0	0	0
RP11-717D12.1	3.90687474	5.07866986	3.008802992	2.01140043	1.007606311	5.436365257
RP11-726G1.1	55.6827322	45.7080288	39.1144389	38.5786603	7.204385126	27.95197803
MS4A18	0	0	0	0	0	0
POLR2J4	24.3398296	31.9042041	124.2334755	84.2676211	57.70561345	127.210947
AC010468.1	22.9528891	33.8036266	34.54105835	31.8605828	46.63202009	28.18755386
MS4A4E	0.97671868	0	0	0	0	0
OOSP1	0	0	0	0	0	0
KRT18P42	0	0	0	0	0	0
RP11-480I12.5	15.6274989	31.4877531	55.16138819	124.706827	400.0197056	181.2121752

RP11-1036E20.9	0	0	2.005868661	2.01140043	0	0.906060876
RP11-37N22.1	0	0	0	1.00570022	3.022818934	0
NPM1P31	0	0	0	0	0	0
CYCSP55	0	0	0	0	0	0
RP5-1053E7.3	0	0	0	0	0	0
FER1L6	0	3.04720192	9.026408976	0	11.08366942	3.624243505
AC008265.2	0	0	0	0	1.007606311	0
CDRT15L2	0	0	0	0	0	0
MPRIPP1	3.90687474	7.11013781	3.008802992	5.02850108	2.025288686	6.351486742
HMGB1P4	0	0	0	0	0	0
KRT16P3	0	0	0	0	0	0
NXT1P1	0	0	0	0	0	0
EI24P3	0	0	0	0	0	0
DDX12P	171.101579	155.163522	88.31839716	128.6894	147.5538682	186.5126314
MTCP1	45.4760219	81.6954834	197.1768894	222.953681	149.0249734	165.9178676
UPF3AP2	5.49892619	7.89225296	2.056015378	0	3.133655628	3.361485851
RPL23AP6	0	1.01573397	0	0	0	0
LINC01347	44.538372	43.4937287	57.5483719	40.0369256	73.917999	75.84635594
RAD51AP2	0	0	0	0	0	0
ZFYVE9P2	0	0	0	0	0	0
RP11-115L11.1	0	0	0	0	0	0
LINC00612	0	0	3.008802992	1.00570022	2.015212623	0
RP11-509M23.1	0	0	0	0	0	0
APOC1P1	0	0	0	0	0	0
KRT16P1	0	0	0	0	0	0
SHFM1P1	0	0	0	0	0	0
EVPLL	0	0	58.12004446	71.5756843	21.15973254	111.2370938
DCDC2C	0	0	0	0	0	0
SRSF9P1	0	0	0	0	0	0
TPM3P4	0	0	0	0	0	0
AC004540.5	3.90687474	7.11013781	0	2.01140043	7.053244179	0
SMTNL1	0	0	0	0	0	0
MED28P1	0.97671868	4.06293589	3.008802992	1.00570022	1.007606311	2.718182629

RPL5P31	0	0	0	0	0	0
OR7E5P	0	0	0	0	0	0
TMEM14DP	0	0	0	0	1.06806269	0.95136392
RP11-574M7.2	0	0	0	0	0	0
FAM177A1P1	0	0	0	0	2.015212623	0
XXyac-YM21GA2.7	0	0	0	0	0	0
RPS9P1	0	0	2.005868661	1.00570022	1.007606311	2.718182629
CTD-2299I21.1	0	0	0	0	0	0
TRIM64C	0	0	0	0	0	0
USP8P1	0	0	0	0	0	0
LINC00243	6.83703079	5.07866986	66.19366583	45.2565097	26.19776409	35.33637417
RPSAP55	0	0	0	0	0	0
U82695.5	0	0	0	0	0	0
LINC01588	181.669675	255.964961	7.020540315	32.1824069	106.806269	59.80001783
RPS15AP3	0	0	0	0	0	0
DUTP8	0	0	0	0	0	0
RP11-229M1.2	0	0	0	0	0	0
RPL23AP3	4.88359342	0	1.002934331	0	0	0
XX-FW80269A6.1	0	0	0	0	0	0
RP11-53I6.1	0	0	0	0	0	0
RP11-674E16.4	3.90687474	0	3.008802992	4.02280086	4.030425245	8.154547886
HLA-F-AS1	28.3443762	31.7010573	0	0	9.068456802	3.116849414
RP3-507I15.1	0	0	0	0	0	0
SPATA31D1	0	0	0	0	0	0
KRT8P6	0	0	0	0	0	0
NPIPA8	0	1.87910785	2.256602244	0	3.768447604	0
ZSWIM7	184.599831	219.398538	389.1385203	396.245885	184.391955	378.7334462
AC113167.1	0	0	0	0	0	0
GPR33	0	0	0	0	0	0
ARHGEF28	4857.22201	3997.92891	1.002934331	4.02280086	543.0998018	97.85457463
TBC1D26	0	0	2.958656276	0	0	1.549364098
LRRC69	10.7439055	13.2045416	12.03521197	12.0684026	6.045637868	13.59091314
AP000318.2	1.95343737	0	0	0	0	0

ISPD	65.4401518	28.4405512	2.005868661	8.04560172	40.30425245	9.060608762
RP11-355K23B.1	0	0	0	0	0	0
NPIPA7	37.0567069	62.010559	115.8088272	125.360532	76.34633021	91.17690597
CTC-297N7.7	0	0	0	0	0	0
CHCHD3P3	0	0	0	0	1.007606311	0.906060876
PPIAP29	1.02555462	2.14319868	2.116191438	4.28428292	3.04297106	2.020515754
VDAC2P1	0	0	0	0	0	0
GSG1L2	0	0	0	0	0	0
RP11-274J2.1	0	0	0	0	2.055516875	0.906060876
RPL7AP26	0	0	0	0	3.022818934	0.906060876
AKAP17BP	0	0	0	0	0	0
CCNB2P1	0	0	0	0	0	0
AC129492.6	0	1.01573397	50.14671654	113.644124	0	24.46364366
RP1-31B8.1	0	0	0	0	0	0
RPL23AP59	0	0	0	0	0	0
RPL15P20	0	0	0	0	0	0
MESTP4	0	0	0	0	0	0
HSPD1P7	0	0	0	0	0	0
CHCHD2P2	0	0	0	0	0	0
DNAJA1P3	0.97671868	0	1.002934331	1.00570022	1.007606311	0
ACSM4	0	0	0	0	0	0
C22orf29	652.86807	589.531997	1426.934848	1334.56419	915.914137	1911.235752
RP5-832C2.5	3.90687474	5.07866986	3.008802992	7.03990151	12.09127574	21.74546103
RPL24P7	0	0	0	0	0	0
COL28A1	0	0	8.023474646	20.1140043	6.045637868	7.248487009
PHB2	3991.15579	3725.87473	5213.082152	4356.59276	4990.573299	5231.957923
RP1-257A7.4	3.90687474	4.06293589	32.09389858	27.1539058	9.068456802	18.12121752
AC114730.5	0	0	0	0	0	0
TCP11X2	0	0	0	0	0	0
RPL13P12	51.0628528	49.9639541	70.03490431	45.3470227	1328.65991	146.6368922
GNL3LP1	46.0229844	20.6092423	27.00902153	20.1140043	107.1488551	34.43031329
DSTNP4	0	0	0	0	0	0
FDPSP5	0	0	0	0	0	0

VENTXP2	0	0	0	0	0	0
CD27-AS1	75.2073387	120.872343	578.6931088	509.890009	74.56286704	459.3728642
NEURL4	1143.85479	1023.12852	582.4942299	591.130473	1099.298486	815.4547886
GLULP6	0	0	0	0	0	0
GRID2IP	0	0	9.026408976	21.1197045	20.15212623	10.87273051
PRDX2P1	0	0	0	0	0	0
HNRNPCP10	0	0	0	0	0	0
PKMP5	0	0	2.005868661	2.01140043	0	0
RPL21P2	0	0	0	0	0	0
DUSP8P4	0	0	0	0	0	0
ALOX12-AS1	58.603121	61.9597723	65.1907315	74.421816	58.44116605	67.95456571
AC025171.1	37.11531	32.5034871	176.5164422	195.105842	65.49441023	187.5546014
XRCC6P5	0	0	0	0	0	0
RP11-471G13.2	0	0	0	0	0	0
NPM1P24	1.95343737	1.03604865	0	3.01710065	6.11617031	1.866485405
RPS5P3	0	0	0	0	0	0
KRT18P11	0.97671868	1.01573397	5.014671654	3.01710065	41.00957687	0
EEF1A1P29	0	0	0	0	0	0
RP11-244K5.7	0	0	0	0	0	0
IFITM8P	0	0	0	0	0	0
DUSP8P3	0	0	11.03227764	5.02850108	1.007606311	0.906060876
TERF1P4	0.99625306	2.11272666	1.012963674	1.02581422	0	0
TTC3P1	48.8847701	46.7745494	22.08461396	33.2182781	445.0294795	67.12098971
UGT2B25P	0	0	4.713791354	1.28729628	0	6.58706257
FAM74A1	0	0	0	0	0	0
CXorf49B	0	0	0	0.50285011	0	0
UBXN2B	485.429186	446.922948	1233.609227	1095.20753	931.0181556	1070.048834
CXorf49	0	0	0	0.50285011	0	0
LINC00588	0	0	0	0	0	0
AL590763.5	0	0	0	1.00570022	0	0.906060876
AMD1P4	0	0	0	0	0	0
CBWD7	222.789532	206.427615	425.2742443	271.428431	333.6587539	406.9210001
SYT14P1	0	0	0	0	0	0

C16orf90	0	0	0	0	0	0
RP11-313J2.1	122.089835	143.21849	4.011737323	2.01140043	11.08366942	4.530304381
PRSS41	0	0	0	0	0	0
KRT18P32	0	0	0	0	0	0
ABCD1P2	0	0	0	0	0	0
AC141586.5	201.204049	132.370451	407.8933923	343.949474	350.0021283	563.8326226
RP11-1023L17.2	0.97671868	3.04720192	0	0	4.030425245	6.342426133
RP11-1023L17.1	126.279959	115.946033	80.97691786	162.350186	147.4430315	134.1966764
RP11-629N8.3	0	0	0	0	0	0
OR7E122P	0	0	0	0	0	0
LINC00269	0	0	0	0	0	0
TCEA1P3	10.7439055	8.12587178	18.05281795	12.0684026	0	7.248487009
RP11-30M20.1	0	0	0	0	0	0
NLRP2P	0	0	0	2.01140043	0	0
IGLV8OR8-1	0	0	0	0	0	0
MAPK6PS4	1.95343737	1.01573397	0	0	2.015212623	0
MUC5AC	28.3248418	10.1573397	0	0	224.6962074	58.89395695
MSMP	0.97671868	1.01573397	2.005868661	2.01140043	1.007606311	4.530304381
RP11-8L18.2	0	1.01573397	2.005868661	1.00570022	3.022818934	0.906060876
GOLGA6B	0	0	5.014671654	0	0	0
FAM166B	0	4.06293589	10.02934331	3.01710065	7.053244179	5.436365257
LINC00680	196.633005	160.770373	64.44856009	62.3534134	198.8410295	99.56702968
PEX26	1106.62227	1041.12732	3100.070016	2505.19924	2387.019351	3692.19807
AC091878.1	0	4.06293589	0	0	8.06085049	0.906060876
PGAM4P1	0	0	0	0	0	0
RP11-182N22.7	0	0	0	0	0	0
YWHAZP6	0	0	0	0.96547221	0	0
GRXCR1	0	0	0	0	0	0
TRBV24OR9-2	0	0	0	0	0	0
KRT18P60	0	0	0	0	0	0
RBMXP2	0.98648587	0	3.018832335	1.00570022	9.098684991	2.727243237
C5orf49	0	0	10.02934331	2.01140043	3.022818934	0
UBE2QL1	0	0	0	0	499.7727304	46.20910469

UBA52P6	0	0	1.002934331	4.02280086	1.007606311	2.718182629
CYP51A1P3	1.95343737	0	5.014671654	1.00570022	0	10.87273051
NUTF2P5	0	0	0	0	0	0
LINC01020	0	0	0	0	0	0
RPL7P33	0	0	0	0	0	0
RP11-54D18.2	0	0	1.002934331	2.01140043	0	1.812121752
RP11-266K4.9	0	0	2.005868661	5.02850108	2.015212623	5.436365257
RP11-563J2.2	3.90687474	6.09440383	64.18779717	5.02850108	10.07606311	21.74546103
RP11-43F13.3	0	0	0	0	2.015212623	0
FASTKD5	761.840573	838.996261	806.3592019	759.152808	627.5775149	841.2503417
GOLGA8B	2843.55041	2189.42473	2512.500939	1929.16432	735.6332157	1929.610666
DHRS4-AS1	112.322649	128.998214	1332.899726	829.702678	665.0201655	787.3669014
KCNU1	1.95343737	0	0	0	0	0
AC025750.7	0	0	0	0	0	0
RPL10AP3	0	0	0	0	0	0
AKR1C7P	0.97671868	4.06293589	0	0	1.007606311	0
LA16c-60G3.8	0	0	0	0	1.007606311	0
GAGE12G	0	0	0	0	0	0
LA16c-60H5.7	0	4.26608268	0	0	0	2.355758278
HOMEZ	16.6042176	22.3461474	550.6109476	830.708378	553.1758649	501.9577254
GAGE10	0	0	0	0	0	0
RNF212B	0	0	2.005868661	2.01140043	0	3.624243505
RPS7P6	0	0	0	0	0	0
HMGB3P24	0	2.03146794	0	0	0	0
RP11-198M15.1	0.97671868	4.06293589	0	0	0	0
RP5-1158E12.2	0	0	0	0	0	0
RP11-613H2.1	0	0	0	0	0	0
TMCO5B	0	0	0	0	0	0
RP11-62E14.2	0	0	0	0	0	0
DDX3X	16691.1456	15089.7134	9133.682832	10691.599	14314.05526	12015.26422
CTD-3092A11.1	91.4111016	46.7237627	58.16016184	43.8988144	25.36145086	37.40219297
RP13-395E19.3	56.5813134	19.0957987	0	0	1.370344583	0
VPS16	717.888233	743.517268	1223.549795	1543.74983	627.7387319	1479.597411

RP6-60B16.1	0	0	0	0	0	0
NPM1P12	0	0	1.002934331	1.00570022	0	0
RPL6P30	0	0	0	0	0	0
THUMPD1P1	0	0	0	0	0	0
EIF5P1	2.93015605	0	1.002934331	0	0	0
ASS1P10	0	0	0	0	0	0
GPX1P2	0	0	0	0	0	0
KRT18P23	0	0	0	0	0	0
ZNF705CP	0	0	0	0	0	0
ZNF705D	0	0	0	0	0	0
SLC25A5P1	0	0	0	0	0	0
MRPL3P1	0.97671868	1.01573397	1.002934331	0	1.007606311	0
PPIAP1	0	0	0	0	0	0
C1QBPP1	0	0	0	0	0	0
FAM90A24P	0	0	0	0	0	0
ZNF705B	0	0	0	0	0	0
HSPD1P8	0	0	0	0	0	0
FAM90A22P	0	0	0	0	0	0
TMED11P	0	0	0	0	0	0
BOD1P2	0	0	0	0	0	0
RPL37P3	0	0	0	0	0	0
DEFB108P2	0	0	0	0	0	0
ZNF705G	0	0	0	0	0	0
FAM90A5P	0	0	0	0	0	0
FAM66B	17.5809363	12.0262902	11.44348071	5.23969812	12.44393794	9.124033023
MYL5	65.4401518	68.0541761	190.5575228	194.100142	81.61611121	166.7152012
DEFT1P	0	0	0	0	0	0
AC090625.1	0	0	0	0	0	0
RP5-1057J7.1	0	0	0	0	0	0
MIR99AHG	2.93015605	3.04720192	87.25528677	105.598523	81.61611121	57.98789608
ACTG1P3	13.6740616	11.1730737	4.011737323	15.0855032	3.022818934	19.0272784
SCRT2	0	0	0	0	0	0
RP11-754I20.1	0	0	0	0	0	0

HMGB3P7	0	0	0	0	0	0
LL22NC01-81G9.3	0	0	0	0	0	0
RP11-748L13.2	0	0	0	0	0	0
PSMA6P1	2.93992324	6.70384422	4.563351205	2.01140043	2.015212623	3.624243505
MIR17HG	62.5099958	48.7552307	14.04108063	12.0684026	44.3346777	38.96061768
PEX12P1	0	0	0	0	0	0
ZNF407	431.709658	403.246387	253.7423857	338.920973	429.2402886	440.3455858
MCM3AP-AS1	161.158583	125.951013	120.3521197	226.282548	375.8371541	168.527323
NPEPL1	688.645275	648.038274	1132.312859	940.329702	2942.734384	943.490251
CTAGE12P	0	0	0	0	0	0
RP5-827A12.2	0	0	0	0	0	0
BX322557.10	227.575453	249.870557	238.6983707	133.758129	372.8143352	184.8364187
SRMP1	0	0	0	0	0	0
RP5-890O15.3	0	0	0	0	0	0
ZNF663P	0	0	0	0	0	0
KRTAP10-4	0	0	0	0	0	0
KRTAP10-1	0	0	0	0	0	0
BCRP4	0	0	0	0	0	0
RPS8P3	0	0	0	0	0	0
AATBC	1.95343737	1.01573397	4.011737323	0	0	0.906060876
AP000354.2	0	0	0	1.00570022	0	0
RPL27AP	0	0	0	0	0	0
RPL17-C18orf32	234.305045	67.2314316	369.3205379	486.587935	119.1998266	315.8437608
SKOR2	0	0	0	0	0	0
SIAH3	0	0	0	0	0	0
RCN1P1	0	0	0	0	0	0
CES5AP1	0	0	2.005868661	5.02850108	0	0
OR7E37P	0	0	0	0	0	0
BCRP3	9.47417123	7.17108184	44.96154605	11.1934434	0	42.30398231
CALM2P3	3.90687474	6.09440383	3.008802992	1.00570022	0	2.718182629
LINC00598	1.95343737	1.01573397	4.011737323	6.03420129	1.007606311	0
ARL2BPP3	0	0	0	0	0	0
HNRNPA1P7	187.432315	167.585948	137.2214751	116.61094	210.7811643	138.7994656

XXbac-B562F10.11	0	0	1.002934331	2.01140043	2.015212623	1.821182361
FAM230B	0	0	0	1.00570022	0	0
TPTE2P4	0	0	0	0	0	0
RBMY2DP	0	0	0	0	0	0
AP005901.1	0	0	0	0	0	0.906060876
IFIT1P1	0.97671868	0	0	6.03420129	7.053244179	5.436365257
RPS4XP4	0	0	0	0	0	0
EFCAB8	5.8603121	4.06293589	6.017605984	4.02280086	1.007606311	9.966669638
LINC00189	0	0	0	0	0	0
ZNF736P11Y	0	0	0	0	0	0
RBMY2CP	0	0	0	0	0	0
BCRP7	0	0	0	0	0	0
DEFB116	0	0	0	0	0	0
DKKL1P1	0	0	0	0	0	0
DEFB115	0	0	0	0	0	0
FRG1JP	16.2330645	10.523004	0	0	1.027758438	0
KRT18P3	0.97671868	0	0	0	0	0.906060876
ANKRD20A11P	0	0	18.51416774	77.9115957	141.3973937	30.5070697
TTTY5	0	0	0	0	0	0
CNN2P7	0	0	0	0	0	0
RP11-79C23.1	0	0	0	0	0	0
GAB4	0	0	0	0	0	0
GRK6P1	0	0	0	1.00570022	0	1.812121752
ESRRAP1	0	0	0	0	0	0
BCORP1	0	0	0	0	0	0
ASS1P6	0	0	0	0	0	0
RP5-836E8.1	0	0	0	0	0	0
RP5-967N21.2	0	0	0	0	0	0
C20orf202	0	0	1.002934331	0	0	0
TSPY24P	0	0	0	0	0	0
ZNF92P1Y	0	0	0	0	0	0
ZNF962P	0	0	0	0	0	0
KRT18P35	0	0	0	0	0	0

HMX1	0	0	0	0	0	0
GUSBP9	60.5663256	24.6417062	13.32899726	16.7348516	36.90861918	8.136426668
GCGR	0	0	2.005868661	3.01710065	1.007606311	1.812121752
AC114730.8	0	1.01573397	2.005868661	5.02850108	5.038031556	5.436365257
RSC1A1	58.0756929	76.3730374	40.26781338	127.764155	152.2090094	316.5051853
CELA2B	0	0	0	0	0	0
TMEM242	115.252805	150.328628	305.8949709	270.533358	129.9812142	257.3212888
TMEM167B	881.976972	763.831947	1169.42143	626.551234	1156.732045	1069.151834
RP1-21O18.3	0	0	0	0	0	0
MRPL20P1	0	0	0	0	0	0
TAF9BP2	0	0	0	0	0	0
CTB-61M7.1	0	0	0	0	0	0
RP13-104F24.2	259.836471	290.154567	366.0710307	473.373034	223.4165474	384.9943269
LRRC37A14P	0	0	6.017605984	1.00570022	0	0
FAM72D	228.434966	252.958388	78.08846699	109.178815	550.2336545	129.0683718
CFL1P6	0	0	0	1.00570022	0	0
TNFRSF25	94.7417123	87.3531216	19.05575228	28.159606	108.8214816	34.43031329
SLC35E2	146.107348	108.896839	214.517624	232.145781	190.2360716	273.1048693
RP11-488L18.3	0.97671868	2.03146794	25.07335827	16.0912034	3.022818934	26.27576541
RP11-551G24.2	0	0	0	0	0	0
RSL24D1P4	0	0	0	0	0	0
RFKP1	0	0	0	0	0	0
RP11-438O11.1	0	0	0	0	0	0
KRT18P65	0	0	0	0	0	0
LINC01139	6.83703079	3.04720192	0	0	2.015212623	0.906060876
BTNL10	0	0	0	0	0	0
ZNF847P	0	0	1.002934331	0	0	0
ZC3H11B	3.54548882	4.84505105	22.63622784	16.3325715	8.131382932	14.08924662
KRT18P12	0	0	0	0	0	0
QRSL1P1	0	0	0	0	0	0
FMO9P	0	0	0	1.00570022	0	0.906060876
RP11-9L18.2	5.87984648	7.13045249	6.037664671	2.02145743	9.108761054	8.190790321
SDHAP2	12.3066554	0	8.334384288	0	9.058380738	162.9006849

RP11-466F5.6	0	0	0	0	2.015212623	0
RP11-122G18.7	5.8603121	5.07866986	6.017605984	6.03420129	6.24715913	8.154547886
RP11-800A3.2	0	0	0	0	0	0
TSTD1	66.4168705	69.0699101	2140.261862	2023.23752	673.0810159	1686.161169
MPTX1	0	0	0	0	0	0
RPTN	0	0	0	0	0	0
RP6-74O6.2	0	0.33519221	0	0	1.36026852	0
WI2-1896O14.1	3.2622404	6.51085476	1.022993017	12.5611957	9.662944525	5.028637863
NBPF7	0	0	0	0	0	0
LINC01356	26.3714045	24.3776153	0	0	1.007606311	0.906060876
KRT18P57	0	0	0	1.00570022	0	0
RP11-364B6.2	0	0	0	0	0	0
RP5-837M10.1	0	0	0	0	0	0
FEN1P1	0	0	0	0	0	0
CAPNS1P1	0	0	0	0	0	0
ST13P20	0	0	0	0	0	0
MARCKSL1P2	0	0	1.002934331	0	1.007606311	0.906060876
RP11-65D24.1	0	0	0	0	0	0
CYB5RL	156.636375	229.555878	305.7044133	328.129809	200.5136559	289.368662
ZNF859P	0	0	0	0	0	0
RPL23AP17	0	1.01573397	0	0	0	0
RP11-334L9.1	4.90312779	3.05735926	0	2.02145743	5.058183683	1.821182361
RP11-439L8.2	0	0	0	0	0	0
SEPW1P	0	0	0	0	0	0
RP11-4M23.7	0	0	0	0	0	0
LACTBL1	0	0	0	0	0	0
CROCCP2	270.551075	248.854823	525.5275599	450.17153	207.5669001	367.5617156
BRWD1P1	0	0	0	0	0	0
C1orf167	0.97671868	3.04720192	0	4.02280086	0	1.812121752
TTC34	3.90687474	2.03146794	36.10563591	55.3135119	20.15212623	29.90000891
MMP23A	1.47484521	0.20314679	0	0	0	4.068213334
ATAD3C	5.95798397	11.3457485	3.068979052	5.02850108	12.26256881	18.36585396
AC092965.1	0	0	0	0	0	0

AC007528.1	0	0	0	0	0	0
AL157788.1	0	0	0	0	0	0
MIR942	0	0	0	0	0	0
AC008166.1	0	0	0	0	0	0
MIR190B	0	0	0	0	0	0
MIR873	0	0	0	0	0	0
MIR892A	0	0	0	0	0	0
AC021613.1	0	0	0	0	0	0
MIR941-1	0	0	0	0	0	0
AC055873.1	0	0	0	0	0	0
MIR921	0	0	0	0	0	0
AC026700.1	0	0	0	0	0	0
AP002884.1	0	0	0	0	0	0
MIR300	0	0	0	0	0	0
AC068292.1	0	0	0	0	0	0
MIR297	0	0	0	0	0	0
MIR876	0	0	0	0	0	0
AC007560.2	0	0	0	0	0	0
AL360176.1	0	0	0	0	0	0
MIR933	0	0	0	0	0	0
AC078819.1	0	0	0	0	0	0
AC024651.1	0	0	0	0	0	0
AC110769.1	0	0	0	0	0	0
MIR208B	0	0	0	0	0	0
AC079603.1	0	0	0	0	0	0
AC017084.1	0	0	0	0	0	0
MIR935	0	0	0	0	0	0
AC004052.1	0	0	0	0	0	0
MIR450B	0	0	0	0	0	0
MIR888	0	0	0	0	0	0
MIR874	0	0	0	0	0	0
AC010888.1	0	0	0	0	0	0
AL512505.1	0	0	0	0	0	0

AL354936.1	0	0	0	0	0	0
AL139429.1	0	0	0	0	0	0
AC008449.1	0	0	0	0	0	0
AC068062.1	0	0	0	0	0	0
AC095061.1	0	0	0	0	0	0
MIR298	0	0	0	0	0	0
MIR938	0	0	0	0	0	0
AL356479.1	0	0	0	0	0	0
MIR922	0	0	0	0	0	0
AL356693.1	0	0	0	0	0	0
AL135784.1	0	0	0	0	0	0
AC019196.1	0	0	0	0	0	0
AC097372.1	0	0	0	0	0	0
MIR891A	0	0	0	0	0	0
MIR944	0	0	0	0	0	0
MIR934	0	0	0	0	0	0
MIR891B	0	0	0	0	0	0
AC104457.1	0	0	0	0	0	0
MIR875	0	0	0	0	0	0
AL158155.1	0	0	0	0	0	0
AL591034.1	0	0	0	0	0	0
AP000870.1	0	0	0	0	0	0
MIR890	0	0	0	0	0	0
MIR887	0	0	0	0	0	0
AC010146.1	0	0	0	0	0	0
MIR936	0	0	0	0	0	0
AC131213.1	0	0	0	0	0	0
AC005048.1	0	0	0	0	0	0
AL157830.1	0	0	0	0	0	0
MIR937	0	0	0	0	0	0
AL356585.1	0	0	0	0	0	0
MIR892B	0	0	0	0	0	0
MIR889	0	0	0	0	0	0

AC233702.1	0	0	0	0	0	0
MIR877	0	0	0	0	0	0
AC010086.2	0	0	0	0	0	0
MIR943	0	0	0	0	0	0
AL713999.1	0	0	0	0	0	0
AL033403.1	0	0	0	0	0	0
AC117479.1	0	0	0	0	0	0
AC012119.1	0	0	0	0	0	0
AC109496.1	0	0	0	0	0	0
AC025284.1	0	0	0	0	0	0
MIR939	0	0	0	0	0	0
MIR885	0	0	0	0	0	0
AC092896.1	0	0	0	0	0	0
MIR941-3	0	0	0	0	0	0
AC109357.1	0	0	0	0	0	0
AL611946.1	0	0	0	0	0	0
AL121988.1	0	0	0	0	0	0
AC131160.1	0	0	0	0	0	0
AC100832.1	0	0	0	0	0	0
AC098650.1	0	0	0	0	0	0
MIR513C	0	0	0	0	0	0
MIR541	0	0	0	0	0	0
AL157882.1	0	0	0	0	0	0
AL137024.1	0	0	0	0	0	0
AC048346.1	0	0	0	0	0	0
MIR8485	0	0	0	0	0	0
MIR920	0	0	0	0	0	0
MIR941-4	0	0	0	0	0	0
AC036178.1	0	0	0	0	0	0
AF228730.1	0	0	0	0	0	0
FABP12P1	0	0	0	0	0	0
RP11-490H24.5	19.7883205	14.7179853	7.913151869	3.02715765	32.34416259	22.30721877
KRT19P2	0	0	0	0	1.007606311	0

RP3-400B16.2	0	0	0	0	0	0
RP3-354N19.3	0	0	0	0	7.053244179	2.718182629
RP3-382I10.2	0	0	0	0	0	0
HIST1H1PS1	0	0	0	0	0	0
RP3-334F4.2	0	0	0	0	0	0
RP11-486E2.1	0	0	0	0	0	0
RP11-360O19.1	0	0	0	0	0	0
RP1-182O16.2	0	0	0	0	0	0
MRPL42P2	0	0	0	0	0	2.718182629
RPL37P15	0	0	0	0	0	0
RP3-486D24.1	0	0	0	0	0	0
RP3-344J20.1	0	0	0	0	0	0
RPL12P2	0	0	0	0	0	0
AC004066.2	0	0	4.011737323	4.02280086	0	2.718182629
HIST1H2APS1	0	0	0	0	0	0
DUTP5	0	0	0	0	0	0
RAET1M	0	0	0	0	0	0
RPSAP43	0	0	0	0	1.007606311	0
RP1-91J24.1	0	0	0	1.00570022	0	0.906060876
RP3-393E18.1	0	0	0	0	0	0
IFI30	555.752931	852.200803	1382.234065	3402.32406	514.9976618	1454.227706
RP11-317M22.1	0	0	0	0	0	0
EEF1GP6	0	0	0	0	0	0
RP3-406A7.1	0	0	0	0	0	0
RP3-435K13.1	0	0	0	0	0	0
RP11-471B18.1	0	0	0	0	0	0
LINC00955	0	0	0	0	0	0
IGSF23	1.95343737	0	0	0	0	0
RP3-406A7.5	0	0	0	0	0	0
RP11-550C4.4	0	0	0	0	0	0
RP11-244K5.6	0	0	0	0	0	0
GAPDHP72	0.97671868	1.01573397	0	1.00570022	0	0
OR2W4P	0	0	0	0	0	1.812121752

RPL7P25	0	0	0	0	0	0
RP3-355L5.3	0	0	0	0	0	0
RP1-95L4.3	0	0	0	0	0	0.906060876
GAGE12E	0	0	0	0	0	0
GLRX3P2	0	1.01573397	0	1.00570022	0	0.906060876
RP11-506B6.5	0	0	0	0	0	0
CCNYL3	0	0	0	0	0	0
RP1-15D7.1	0	0	0	0	0	0
RP11-278J20.2	0	0	0	0	0	0
CIR1P3	0	0	0	0	0	0
COX6A1P3	0	0	0	0	0	0
MTND4P13	0	0	0	0	0	0
RP3-522P13.1	0	0	0	0	0	0
AC093850.1	0	0	0	0	0	0
NUDT19P3	0	0	0	0	0	0
ANXA2P3	0	0	0	0	1.007606311	0
HMGA1P7	0	0	0	0	0	0
RP1-190J20.2	0	0	0	0	0	0
VN1R13P	0	0	0	0	0	0
RP1-152L7.5	719.84167	475.363499	183.5369825	256.453555	137.0344583	220.1727929
PRRC2CP1	0	0	0	0	0	0
RP1-290I10.2	0	0	0	0	0	0
RP11-390P2.2	0	0	1.002934331	0	0	0
RP11-57K17.1	4.88359342	4.06293589	1.002934331	0	1.007606311	2.718182629
RP1-69B13.2	0	0	0	0	0	0
RP11-812I20.2	1.95343737	1.01573397	3.008802992	0	0	0.906060876
R3HDM2P2	0	0	0	0	0	0
TUBB2BP1	4.55150907	1.08683535	0	0	1.188975447	0
ZNF736P10Y	0	0	0	0	0	0
RP11-175I6.1	0	0	0	0	0	0
RBMXP1	0	0	0	0	0	0
AC009494.3	0	0	0	0	0	0
RP3-399J4.1	0	0	0	0	0	0

RPL17P26	0	0	0	0	0	0
RP11-448N11.1	0	0	0	0	0	0
LY86-AS1	0	0	0	0	0	0.906060876
RPS2P55	39.8696567	23.5853428	97.06398453	175.645543	81.47504633	137.2229197
AC114776.1	0	0	0	0	0	0
AC009403.2	60.5565584	57.8968364	24.07042394	33.1881071	96.73020588	48.92728731
AL022393.7	1.95343737	0	3.008802992	1.00570022	1.007606311	0
RP1-161C16.1	0	0	0	0	0	0
SOCS5P5	0	0	0	0	0	0
RP11-350J20.9	14.6507803	16.2517436	2.005868661	2.01140043	7.053244179	2.718182629
RP4-753D5.3	0	0	0	0	0	0
RP1-97D16.1	1.95343737	0	0	0	5.038031556	0.906060876
RP5-988G15.1	0	0	0	0	0	0
AC131097.4	0	0	0	0	0	0
CCDC7	25.3946858	82.2744518	93.00210049	82.0148526	41.31185876	52.55153082
RPL7P58	0	0	0	0	0	0
RP11-568A7.1	0	0	0	0	0	0
RPL21P65	0.97671868	0	0	3.01710065	0	1.812121752
HSPD1P10	0.97671868	0	1.002934331	1.00570022	0	1.812121752
CYP2AC1P	0	0	0	0	0	0
RP3-329A5.1	0	0	0	0	0	0
RPL10P1	0	0	0	0	0	0
TPT1P4	0	0	0	2.56453555	1.007606311	0.960424529
RP11-428J1.2	0	0	0	0	0	0
RP11-346C16.1	0	0	0	0	0	0
RP11-331O9.4	0	0	0	0	0	0
RP11-30P6.1	0	0	1.002934331	0	1.017682374	0
RP11-448N11.2	0	0	0	0	0	0
RP1-13D10.3	0	0	0	0	0	0
RP1-245M18.2	0	0	0	0	0	0
HMGB3P19	0	0	0	0	0	0
RPS29P13	0	0	0	0	0	0
PPIAP31	26.0588545	36.1093427	31.2414044	45.3872507	26.30860079	40.79992125

RP1-76C18.1	0	0	0	0	0	0
FNIP1	2158.54829	2584.02723	1214.553474	1923.90451	934.0510506	1224.994305
RP3-375P9.2	3.77013412	1.01573397	0	0	0	0
RP1-140K8.3	0	0	0	0	0	0
RP11-129H15.4	0	0	0	0	0	0
LARP1P1	0	0	4.011737323	5.02850108	0	3.624243505
RP11-331O9.1	0	0	0	0	0	0
ANKRD18EP	0	0	0	0	0	0
MTHFD2P2	0	0	0	0	0	0
RP11-9G10.1	0	0	0	0	0	0
MTCYBP2	0	0	0	0	0	0
RP11-360O19.5	0	0	0	0.99564321	0	0
RP11-277I20.2	0	0	0	0	0	0
RP1-319M7.2	0	0	0	0	0	0
GSTA12P	0	0	0	0	0	0
RP3-340H11.2	0	0	0	0	0	0
SP9	0	0	0	0	5.038031556	0.906060876
RP1-182O16.1	0	0	0	0	0	0
CBX3P9	7.82351666	4.07309323	5.024700997	5.04861508	19.27550873	6.360547351
AC007249.3	0	0	0	0	0	0
POM121L4P	0	0	1.09319842	8.3975968	0	15.87418655
TXNP7	0	0	0	0	0	0
RP1-263J7.2	0	0	0	0	0	0
RP1-34B20.4	0	0	19.05575228	25.1425054	8.06085049	29.90000891
AC018865.5	0	0	8.043533332	2.01140043	2.246962074	9.060608762
UQCRFS1P3	0	0	0	0	0	0
OR2W2P	0	0	3.008802992	1.00570022	1.007606311	0.906060876
PRELID1P1	13.1564007	12.2903811	5.325581296	7.18069954	9.249825938	3.705788984
RPS7P5	0	0	0	0	0	0
SSXP10	0	0	0	0	1.007606311	0
RP11-304C16.3	0	0	0	0	0	0
RP11-33E24.3	0	0	0	0	1.007606311	0
TUBB4BP7	0	0	0	0	0	0

AK4P5	0	0	0	0	0	0
RP11-254A17.1	0	0	0	0	0	0
PSMC1P11	0	0	0	0	0	0
RPS4XP9	0	0	1.002934331	0	0	0
PRELID1P2	0	0	0	0	0	0
RP11-528A10.2	0	0	0	0	0	0
ISCA1P1	2.16831548	2.33618814	0	0	3.274720512	0.969485138
SYCE3	0	0	0	0	3.022818934	0.906060876
RP3-366N23.4	0	0	0	0	0	0
AC091801.1	0.97671868	0	0	0	0	0
RP11-184C23.1	0	0	0	0	0	0
HMGB1P17	0	0	0	0	0	0
RP11-135M8__A.1	0	0	0	0	0	0
RP11-474L11.5	0	0	0	0	0	0
RP1-95L4.2	0	0	0	0	0	0
RP11-477E3.2	0.97671868	3.04720192	3.008802992	1.00570022	1.007606311	3.624243505
RP3-495O10.1	0	0	0	0	0	0
RPS16P5	0	4.06293589	3.008802992	0	0	0
IQCB2P	0	0	0	2.01140043	0	2.718182629
CKLF	517.006501	522.828748	741.3891159	633.41011	149.2164186	725.72758
RP11-157L10.1	0	0	0	0	0	0
TDGF1P4	0	0	0	0	0	0
RP11-248G5.8	0	0	0	15.1257312	4.705521474	4.258486118
RP1-13D10.5	0	0	0	0	0	0
RP1-28C20.1	0	0	0	2.01140043	0	0.906060876
RP11-204C16.4	38.8734036	34.413067	13.88061114	24.6396553	70.36114872	28.38688725
RP1-142O9.2	0	0	0	0	0	0
PTGES3P2	0	0	0	0	0	0
RP4-803A2.1	0	0	0	0	0	0
HIST1H2BPS2	0	0	0	0	1.007606311	0.906060876
RP1-95L4.4	7.81374947	11.1730737	34.09976724	20.1140043	15.11409467	29.90000891
RP11-63K6.1	0	0	0	0	0	0
RP11-415D17.1	0	0	0	0	0	0

RPS3AP24	0	0	0	0	0	0
NUS1P4	0	0	0	0	0	0
RP11-287D1.4	0	1.01573397	1.002934331	0	1.007606311	0
SERPINB8P1	0	0	0	0	0	0
RPS10P3	2.1487811	0	0	0	2.186505695	0
AC064847.4	0	0	0	0	0	0
CCT7P1	0	2.03146794	1.002934331	0	3.022818934	0
RP1-303A1.1	0	0	0	0	0	0
RP11-160A9.2	0	0	0	0	0	0
RP11-76H14.2	0.78137495	0	0.431261762	2.08179945	0	0.887939659
FEM1AP3	0	0	0	0	0	0
RP11-156F23.2	0	0	0	0	0	0
LDHAL6FP	0.97671868	0	1.002934331	1.00570022	0	6.342426133
RP3-508D13.1	0	0	0	0	0	0
ASS1P9	0	0	0	0	0	0
RP11-465B22.3	136.740616	197.052391	4.011737323	12.0684026	28.21297672	9.966669638
RP11-191A15.2	0	0	0	0	0	0
FAT1P1	0	0	0	0	0	0
HNRNPDP2	0	0	0	0	0	0
SNRPEP6	0	0	0	0	0	0
AC099552.4	0	0	0	0	0	0
RP6-159A1.2	2.93992324	1.01573397	2.005868661	3.01710065	3.032894997	1.812121752
HIST1H4PS1	0	0	0	0	0	0
OR4F7P	0	0	0	0	0	0.906060876
RP11-534P19.1	0	0	0	0	0	0
KRT18P48	0	0	0	0	0	0
ZNF839P1	0	0	0	0	0	0
HSPE1P8	0	0	0	0	0	0
CICP18	21.8589641	18.6082464	26.99899218	22.3768298	50.47100013	40.73649699
PAM16	318.410291	299.316487	425.2441562	242.373752	628.3029915	399.5728464
AC073869.20	1.30880304	3.04720192	5.957429924	5.93363127	6.166550625	4.385334641
KRT19P1	0	0	1.002934331	0	7.053244179	0
ZNF192P2	0	0	0	0	0	0

RP4-800J21.3	0	0	0	0	3.022818934	2.718182629
PRKRIRP5	0	0	0	0	0	0.906060876
RP11-157J24.1	0	1.01573397	0	0	1.007606311	0
RP1-232L24.2	0	0	0	0	0	0
RP3-407E4.4	0	0	0	0	0	0
RPL31P33	0	0	0	0	0	0
ADAMTS7P4	0.97671868	2.03146794	0	0	0	0
RSL24D1P1	0	0	1.002934331	1.00570022	0	0
RP1-13D10.2	1.95343737	0	7.020540315	0	0	9.060608762
DNAJA1P4	0	0	0	0	0	0
RP1-261G23.4	0	0	0	0	0	0
KIRREL3-AS3	0	0	0	0	0	0
AP000567.27	0	0	0	0	0	0
ERHP2	0	0	0	0	0	0
KRT18P22	0	0	0	0	0	0
RP11-427E4.1	0	0	0	0	0	0
AC016739.2	27.3481231	23.3618814	50.14671654	61.3577702	59.44877237	52.56059143
SLC25A5P7	0	0	1.002934331	0	0	0
KRT8P43	0	0	0	0	0	0
RP11-325O24.1	0	0	0	0	0	0
POM121L14P	0	0	0	0	1.007606311	0
HLFP1	0	0	0	0	0	0
RPS20P32	0	0	0	0	0	0
RP11-367G18.2	0	0	1.002934331	0	0	0
FTH1P26	0	0	0	0	1.007606311	0
TATDN2P2	0.97671868	0	2.005868661	0	0	1.812121752
RP11-889L3.1	25.404453	15.2461669	16.04694929	16.0912034	17.02854666	8.163608494
NEPNP	0	0	0	0	0	0
RP11-96J19.1	0	0	0	0	0	0
RPS4XP7	0	0	0	0	0	0
RP3-407E4.3	0	0	0	0	0	0
HIST1H2APS3	0	0	0	0	0	0
MORF4L1P1	295.164386	318.422443	107.3741494	117.043391	327.0992368	133.5352519

RP11-63K6.4	0	0	0	0	1.007606311	0
CDC14C	0	0	0	0	0	0
RP11-393I2.2	0	0	0	0	0	0
TENM3	77.160776	68.0541761	0	1.00570022	22.16733885	9.060608762
RP4-724P12.1	0	0	0	0	0	0
HNRNPA1P1	0	0	0	1.00570022	0	0
LYPLA1P3	0	1.02589131	0	2.01140043	2.045440812	0.906060876
RPS3AP23	0	0	0	0	0	0
LL22NC03-75H12.2	0	0	0	0	0	0
RAET1K	19.5343737	19.8575992	26.15652734	35.1995075	8.101154743	33.78701007
SUMO2P13	0	0	0	0	0	0
SLC25A20P1	0	0	0	0	0	0
AC012078.2	0	0	0	0	0	0
PP14571	0.97671868	4.06293589	8.023474646	2.01140043	730.5145757	93.32427025
RP11-296E7.1	15.4126208	10.675364	4.232382876	9.45358203	5.279857071	10.41063947
NDUFS5P1	0	0	0	2.01140043	0	0
RP11-475C16.1	97.1639747	75.0525832	52.74431645	48.3238954	64.50695605	33.8141919
NIP7P3	0	0	0	0	0	0
HNRNPA1P19	0	0	0	0	0	0
RPS27P15	0	0	0	0	0	0
RP1-140K8.2	0	0	0	0	0	0
ACTG1P18	0	0	0	0	0	0
RP11-108N13.1	0	0	0	0	0	0
RP11-554D15.2	0	0	0	0	0	0
FCF1P10	0	0	0	0	0	0
RP3-422G23.3	0	0	0	0	0	0
H2AFZP3	0	0	0	3.01710065	0	0
LINC00339	252.970139	229.555878	193.5663258	166.946236	89.6769617	168.527323
SPTLC1P2	0	0	0	0	0	0
RP11-184C23.4	0	0	0	0	0	0
RP11-420A21.1	0	0	0	0	0	0
AP002530.2	0	0	0	0	0	0
MIF-AS1	29.3015605	44.6922948	252.7394513	213.208446	129.9812142	165.8091403

OR4K12P	0	0	0	0	0	0
RP1-253B10.1	0	0	0	0	0	0
RP11-12A2.1	0	0	0	0	0	0
HNRNPA1P37	0	0	0	0	0	0
RP3-416J7.1	0	0	0	0	0	0
GAPDHP63	0	0	0	0	0	0
RP4-791C19.1	0.98648587	0	0	1.00570022	0	0.915121485
RP11-2J18.1	0	0	0	0	0	0
RP3-407E4.2	0	0	0	0	0	0
RP3-395C13.1	0	0	3.008802992	2.01140043	2.015212623	1.812121752
RPL7P28	0	0	0	0	0	0
RPL5P20	0	0	0	0	0	0
AC008060.7	0	0	0	0	0	0
BRD7P4	0.97671868	2.03146794	1.002934331	0	0	0.906060876
AC010150.1	3.90687474	0	0	0	0	4.530304381
RPL5P21	0	0	0	0	0	0
HIST1H2APS4	0	0	3.008802992	14.079803	18.1369136	13.59091314
ST13P16	0	0	0	0	0	0
RP1-34L19.1	0	0	0	0	0	0
RPL12P23	0	0	0	0	0	0
B3GALNT2P1	0	0	0	0	0	0
KRT18P44	0	0	0	0	0	0
RP3-453I5.2	0	0	0	0	0	0
RP5-1046G13.2	0	0	0	0	0	0
CEBPZOS	1171.0857	1311.31256	1444.225436	816.628575	1144.64077	983.9821115
DBIP1	0	0	0	0	0	0
RP3-340B19.5	0	0	0	0	0	0
RPL18AP8	0	0	0	0	0	0
RP3-472M2.2	0	0	0	0	0	0
RP11-207F8.1	0	0	0	0	0	0
FAM8A6P	0	0	0	0	0	0
RP11-331O9.3	0	0	0	0	0	0
HSPD1P16	0	0	0	0	0	0

RP3-382I10.3	0	0	1.002934331	0	0	0
GSTM2P1	0	0	0	0	0	0
RP3-369A17.2	0	0	0	0	0	0
RP1-229K20.5	0	0	0	0	0	0
RP11-59D5__B.3	0	0	0	0	0	0
TDRD15	0	0	0	0	0	0
PAPOLB	0	0	0	0	0	0
RP11-361F19.1	0	0	0	0	0	0
FAM138C	0	0	0	0	0	0
RP1-209B5.2	0	0	0	0	0	0
CNN3P1	0	0	0	0	0	0
SLC25A6P6	0	0	0	0	0	0
RP11-249L21.2	0	0	0	0	0	0
NUFIP1P	0.97671868	1.02589131	2.015898005	0	1.017682374	3.624243505
ZNF579	475.661999	444.89148	578.6931088	466.6449	467.5293284	522.7971256
SUMO2P12	0	0	0	0	0	0
TUBB8P2	0	0	0	0	0	0
PTMAP3	0	0	0	0	0	0
NACAP7	0	0	0	0	0	0
RP11-528A10.1	0	0	0	0	0	0
FTH1P15	0	1.01573397	0	0	0	0
RP1-137F1.3	0	0	0	0	0	0
CCNG1P1	0	0	0	0	0	0
RP1-99E18.2	4.06314972	2.16351336	2.166338154	0	2.287266327	0
RP3-340B19.2	1.12322649	0	0	2.14214146	0	1.150697313
RPS3AP2	0	0	3.008802992	0	0	0
AC005102.1	0	0	0	0	0	0
TRIM51FP	0	0	0	0	0	0
CELA3B	0	0	0	0	0	0
SOD1P1	0	0	0	0	0	0
NPM1P37	0	0	12.25585752	4.05297187	0	0.924182094
MTND4LP19	0	0	0	0	0	0
RP3-359N14.1	0	0	0	0	0	0

RP11-323I14__A.1	0	0	0	0	0	0
HNRNPA3P12	4.95196373	3.0675166	4.152148129	3.09755666	8.121306869	3.651425331
RP11-203F10.6	2.12924673	0	2.116191438	3.33892472	5.47130227	1.322848879
RPL23AP48	0	0	0	0	0	0
RP11-304C16.2	0	0	0	0	0	0
RPS4XP8	0	0	0	0	0	0
RP11-214N16.2	0	0	0	0	0	0
RP11-569A2.2	0	0	0	0	0	0
AC011298.2	0	0	0	0	0	0
HMGB1P20	0	0	0	0	0	0
FTH1P19	0	0	0	0	0	0.906060876
CACYBPP3	0	0	0	0	0	0.906060876
RP1-253B10.2	0	0	0	0	0	0
RNASEK	2135.63447	2578.45085	1344.212825	1752.23149	1362.938677	1095.762842
RP11-181C21.4	14.0452147	15.8251353	7.100775061	10.8213343	6.609897402	8.036759972
RPL12P47	0	0	0	0	0	0
RP11-63K6.5	0	0	0	0	0	0
AMZ2P2	0	0	0	0	0	3.624243505
RPS6P7	0	0	0	0	0	0
NPM1P38	0	0	0	1.00570022	0	0
OR2E1P	0	0	0	0	0	0
RP1-217P22.2	0	0	0	0	0	0
RPS20P2	0	0	0	0	0	0
VN1R8P	0	0	0	0	0	0
RP11-151M7.1	0	0	0	0	0	0
PIP5K1P1	0	0	0	0	0	0
MRPL42P3	0	0	0	0	0	0
RP11-472G23.3	0	0	0	0	0	0
RP11-174C7.3	0	0	0	0	0	0
RP1-273P12.1	0	0	0	0	0	0
RP11-397G5.2	0	0	0	0	1.007606311	0
RPL31P52	0	0	0	1.00570022	0	0
RPSAP72	0	0	0	0	0	0

ZNF299P	0	0	0	0	0	0
RP11-69L16.3	0	0	0	0	0	0
RP11-491H9.3	0	0	0	0	0	0
ATF1P1	0	0	0	0	0	0
AC019129.1	0	0	0	0	0	0
RP1-265C24.5	0	0	0	0	0	0
HSPA8P15	4.88359342	2.03146794	2.005868661	2.01140043	23.17494516	3.624243505
RP11-524C21.1	0	0	0	0	0	0
RP11-106E7.1	0.97671868	0	0	1.00570022	0	0
RP4-761J14.8	10.7439055	7.11013781	5.014671654	7.03990151	6.045637868	5.436365257
MBL3P	0	0	0	0	0	0
BTBD10P2	0.97671868	0	4.011737323	3.01710065	1.007606311	0.906060876
TEX40	4.50267313	3.707429	0	1.80020339	2.88175405	6.5145777
FAM19A5	0	0	0	0	175.3234982	19.0272784
RP11-3B12.3	0	0	0	0	0	0
AC005251.3	0	1.01573397	0	2.01140043	0	0.906060876
RP4-810F7.1	0	0	0	0	0	0
RPSAP42	0	0	0	0	0	0
RP3-337H4.6	13.8791725	3.05735926	11.80453707	7.31144057	24.51506155	13.62715558
NBPF1	1045.52852	930.879556	871.6101094	1256.73305	3773.344571	1214.918908
RP11-365H23.1	1.95343737	0	0	2.01140043	1.007606311	0
TPT1P8	0	0	0	0	0	0
RP11-1396O13.13	0	0	0	0	0	0
RP11-15J23.1	0	0	0	0	0	0
FTH1P8	14.9047271	42.1834319	86.23229375	55.3336259	43.91148305	40.12943621
AP000580.1	3.90687474	2.03146794	33.83900432	20.7375384	19.5576385	18.14839935
RP3-323K23.3	0	0	0	0	0	0
UMAD1	217.485949	238.94126	282.8274813	436.091727	278.6031451	280.199326
RPL17P25	0	0	0	0	0	0
RP11-374I15.1	0	0	0	0	0	0
RP1-281H8.3	0	1.01573397	1.002934331	0	1.007606311	0.906060876
RP11-346C16.4	0	0	0	0	0	0
ZNF259P1	0	0	0	0	1.007606311	0

RP1-288M22.1	0	0	0	0	0	0
HNRNPA1P58	0	0	0	0	0	0
NCSTNP1	0	0	0	0	0	0
RP11-132M7.2	0	0	0	0	0	0
PPP1R3G	36.1385913	61.9597723	5.014671654	0	34.25861458	5.436365257
HIGD1AP16	0	0	0	0	1.007606311	0
RP11-126M14.1	0	0	0	0	0	0
RP11-291C6.1	0	0	0	0	0	0
FAM228B	78.7528275	74.14858	124.2234462	63.3591136	32.24340196	58.89395695
CYCSP17	0	0	0	0	0	0
BMPR1APS1	0.98648587	0	0	0	0	0
GCNT1P4	0	0	0	0	0	0
CTD-2006C1.2	137.717334	150.328628	0	0	48.36510294	10.87273051
RP11-174C7.1	0	0	0	0	0	0
BECN1P2	0	0	0	0	0	0
RP11-191A15.4	0	0	0	0	0	0
RP3-425P12.4	5.29381527	1.17825141	5.435904072	5.72243423	7.325297883	3.107788805
RP11-262H14.11	1.95343737	0	0	0	0	0
RP11-325O24.2	0	0	0	0	0	0
PTCHD3P3	0	0	0	0	0	0
RP5-991C6.2	0	0	0	0	0	0
RAP1BP3	0	0	0	0	0	0
RP11-532F6.2	4.88359342	4.06293589	4.011737323	1.00570022	3.022818934	1.812121752
RPL26P20	0	0	0	0	0	0
RP11-560O20.1	0	0	0	0	0	0
XXbac-BPG34I8.3	0	0	0	0	0	0
RP1-292B18.1	0.97671868	0	0	0	2.025288686	0
RP1-199J3.5	2.93015605	3.04720192	1.002934331	8.04560172	3.022818934	3.624243505
RP11-793L10.1	0	0	0	0	0	0
RP1-249H1.3	0	0	0	0	0	0
VN1R11P	0	0	0	0	0	0
RPSAP45	0	0	0	0	1.017682374	0
RPL21P67	0	0	0	0	0	0

RP1-172I22.1	0	0	0	0	0	0
OSTCP6	0	0	0	0	0	0
PPIAP9	3.89710755	1.00557663	0.992904987	1.00570022	4.010273119	1.794000535
ATP5F1P6	0	0	0	0	0	0
ARF1P1	0	0	0	0	0	0
RPL23AP47	0	0	51.14965087	45.0151416	0	27.45364455
RPL31P28	0	0	0	0	0	0
RP11-40G16.1	0	0	0	0	0	0
RP11-430A19.2	0	0	0	0	0	0
GAPDHP42	0	0	0	0	0	0
ZSCAN12P1	0	1.01573397	83.24354945	119.678326	21.15973254	58.89395695
RPL35P3	0	0	1.002934331	0	2.015212623	0
RP11-394A14.2	0	0	0	0	0	0.906060876
RP11-40C6.2	2.9692248	3.09798862	2.025927348	4.07308587	4.080805561	3.66048594
GAPDHP67	0	0	0	0	0	0
RPL12P8	1.97297174	0	0	0	1.078138753	0.933242702
SPTLC1P3	0	0	0	0	0	0
KRT18P50	0	0	0	0	0	0
RP11-378G13.2	0	0	1.002934331	0	0	0
BTF3P7	0	0	0	0	0	0
RP11-420L9.2	0	0	0	0	0	0
RP11-288G3.3	0	0	0	0	0	0
LINGO3	0	0	11.03227764	3.01710065	24.18255147	28.08788716
RP1-91N13.1	0	0	0	0	0	0
RPL7P27	0	0	0	0	0	0
RP1-207H1.2	0	0	0	0	0	0
LAP3P1	0.97671868	0	0	0	0	0
RP11-307I14.2	0	0	0	0	0	0
RP1-6P5.2	0	0	0	0	0	0
MTCYBP4	0	0	0	0	0	0
MRPL32P1	0	0	0	0	0	0
RP1-72A23.3	0	0	0	0	0	0
RP4-570O12.2	0	0	0	0	0	0

AF238380.8	0	0	0	0	0	0
RP3-354N19.1	0	0	0	0	0	0
RP11-46B11.2	0	0	0	0	0	0
HNRNPA1P12	1.00602024	2.122884	5.12499443	1.80020339	4.332707139	8.217972147
FLJ35934	0	0	0	1.44820831	2.015212623	3.87794055
RP11-486M3.2	0	0	0	0	0	0
HMGB3P18	0	0	0	0	0	0
ZGLP1	28.5104184	35.9468253	191.4601637	131.626044	32.96887851	70.0113239
RP1-53C18.3	0	0	0	0	0	0
VAMP2	988.204896	891.448763	521.1948836	347.499595	501.8282473	377.1659609
OR4F1P	0	0	0	0	0	0
RPS24P12	0	0	0	0	0	0
RP11-76H14.5	0	0	0	0	0	0
ZNF402P	0	0	0	0	0	0
AC093802.1	0	0	0	0	0	0
ACTBP8	0	0	0	0	0	0
RP3-455E7.1	0	0	0	0	0	0
HNRNPH1P1	1.95343737	2.03146794	19.05575228	18.1026039	20.15212623	7.248487009
RPL35AP18	0	0	0	0	0	0
HIST2H2BD	17.1609473	17.2674775	78.16870174	192.9637	185.7119192	246.0589521
RP11-129H15.1	0	0	0	0	0	0
RP3-433F14.1	0	0	0	0	0	0
RP11-440K22.1	0	0	0	0	0	0
RP11-230C9.1	0	0	0	0	0	0
RP3-431A14.4	0	0	0	0	0	0
RP3-399J4.2	0	0	0	0	0	0
GSTA8P	0	0	0	0	0	0
KRT8P42	0	0	0	0	0	0
FCF1P5	0	0	0	0	1.007606311	0
RP11-95M15.2	0.97671868	1.01573397	0	0	1.007606311	0
TUBB3P1	0	0	0	0	0	0
RP3-520B18.1	0	0	0	0	0	0
RP1-276J11.2	0	0	0	0	0	0

RP11-69L16.5	3.90687474	2.36666016	5.937371238	2.01140043	9.602488147	3.624243505
SLC25A51P1	0	0	0	0	0	0
YAP1P1	0	0	0	0	0	0.906060876
EIF4EBP2P3	0	0	0	0	0	0
RP3-415N12.1	0	0	0	0	0	0
MTND4LP20	0	0	0	0	0	0
PGAM1P10	0.97671868	5.07866986	0	0	0	0
ASS1P1	0	0	2.005868661	0	2.408179084	0
RP1-177A13.1	0	0	0	0	0	0
RP3-492P14.2	0	0	0	0	0	0
RP1-298M8.1	0	0	0	0	0	0
RP5-1170D6.1	0	0	0	0	0	0
VIM2P	0	0	0	0	0	0
RPL5P19	0	0	0	0	0	0
RP1-202I21.3	0	0	0	0	0	0
HMGB1P13	0	0	0	0	0	0
PKMP3	0	0	11.03227764	3.01710065	2.015212623	8.154547886
HTR5A-AS1	0	0	0	0	0	0
VN1R12P	0	0	0	0	0	0
RPL35P2	27.3969591	25.444136	15.06407365	22.1555757	23.22532548	9.984790855
DDX18P6	0	0	0	0	0	0
TUBBP9	0	0	0	0	0	0
SSR1P1	0	0	0	0	0	0
RP3-509I19.6	0	0	1.002934331	1.00570022	0	0
RP11-480N24.4	0	0	0	0	0	0
KRASP1	0	0	0	0	0	0
RP3-391O22.1	0	0	0	0	0	0
RP3-406A7.3	0	0	0	0	0	0
RCC2P7	0.97671868	0	0	0	5.038031556	0
RP11-530A18.1	8.79046815	4.06293589	6.017605984	1.00570022	2.015212623	3.624243505
RP3-403A15.1	0	0	0	0	0	0
RP1-121G13.3	0	0	1.002934331	1.00570022	0	0
RP1-32I10.10	0	0	0	0	0	0

OR1F12	0	0	0	1.00570022	0	0
RP11-228O6.2	0	0	0	0	0	0
RP11-474L11.3	0	0	0	0	0	0
RP11-331O9.5	0	0	0	0	0	0
RP3-510O8.3	0	0	0	0	0	0
RP11-277I20.3	0	0	0	0	0	0
RPL5P18	0.97671868	2.03146794	2.005868661	3.01710065	2.015212623	3.624243505
RP1-101K10.4	0	0	0	0	0	0
RP1-59B16.1	0	0	0	0	0	0
RPL21P28	46.9899359	45.7486581	57.76901745	70.157647	108.5494279	53.02268247
VN1R10P	0	0	0	0	0	0
BOLA2P3	0	0	0	0	1.007606311	0
RPSAP44	0	0	0	0	0	0
MTMR9LP	34.1851539	26.4090833	275.8069409	310.761367	14.10648836	145.8758011
RPL21P119	46.9215656	54.057362	73.95637755	112.658538	70.59289817	92.10108806
AC093642.5	49.3633623	33.5090637	342.0006068	488.076372	62.62273225	241.4561629
NDUFA5P9	0	0	0	0	0	0
RP11-572P18.1	210.346136	159.825741	369.029687	273.439832	346.3948977	247.3455586
RPS18P9	9.76718684	16.2517436	28.36298287	20.4659994	6.045637868	20.83940015
HSPE1P26	0	0	0	0	0	0
MRPL35P1	0	0	0	0	0	0
DNAJC19P6	0	0	0	0	0	0
HIST1H3PS1	0	0	0	0	0	0
MESTP1	0	0	0	0	0	0
LL22NC03-63E9.3	0	0	0	0	0	0
RP11-127B16.1	0	0	0	0	0	0
RP11-235G24.2	0	0	0	0	0	0
RP1-92C4.1	0	0	0	0	0	0
RP3-525L6.2	0	0	0	0	0	0
OSTCP4	0	0	0	0	0	0
IGBP1-AS2	0	0	0	0	0	0
HNRNPA1P41	0	0	0	0	0	0
TRIM51GP	0	0	0	0	0	0

CTC-499B15.1	0	0	0	0	0	0
RP1-72A23.1	0	0	0	0	0	0
AL391384.1	0	0	0	0	0	0
AC009784.1	0	0	0	0	0	0
AC025674.1	0	0	0	0	0	0
SNORA50	0	0	0	0	0	0
AC084368.1	0	0	0	0	0	0
SNORD88C	0	0	0	0	0	0
AC063944.1	0	0	0	0	0	0
AL450423.1	0	0	0	0	0	0
AL158151.1	0	0	0	0	0	0
AC007611.1	0	0	0	0	0	0
AC034236.1	0	0	0	0	0	0
AL161651.1	0	0	0	0	0	0
AC017076.1	0	0	0	0	0	0
AC100757.1	0	0	0	0	0	0
AC022120.1	0	0	0	0	0	0
AC131254.1	0	0	0	0	0	0
AL161723.1	0	0	0	0	0	0
AL354984.1	0	0	0	0	0	0
AC136698.1	0	0	0	0	0	0
AL008708.1	0	0	0	0	0	0
RNU6ATAC29P	0	0	0	0	0	0
AC002558.1	0	0	0	0	0	0
MIR1323	0	0	0	0	0	0
AC136365.1	0	0	0	0	0	0
AC127526.1	0	0	0	0	0	0
AC107993.1	0	0	0	0	0	0
AL035697.1	0	0	0	0	0	0
RNU6ATAC19P	0	0	0	0	0	0
MIR1250	0	0	0	0	0	0
AL592063.1	0	0	0	0	0	0
MIR1231	0	0	0	0	0	0

AC016194.1	0	0	0	0	0	0
AC010930.1	0	0	0	0	0	0
CoTC_ribozyme	0	0	0	0	0	0
AL590683.1	0	0	0	0	0	0
MIR1272	0	0	0	0	0	0
MIR1193	0	0	0	0	0	0
RNU6ATAC7P	0	0	0	0	0	0
MIR1286	0	0	0	0	0	0
U3	0	0	0	0	0	0
AP001537.1	0	0	0	0	0	0
CPEB3_ribozyme	0	0	0	0	0	0
U3	0	0	0	0	0	0
U3	1.95343737	0	0	0	0	0
RNU6ATAC38P	0	0	0	0	0	0
AC079781.1	0	0	0	0	0	0
AC091304.1	0	0	0	0	0	0
AC118344.1	0	0	0	0	0	0
MIR1266	0	0	0	0	0	0
AC115286.1	0	0	0	0	0	0
MIR1302-3	0	0	0	0	0	0
AC090666.1	0	0	0	0	0	0
RNU6ATAC6P	0	0	0	0	0	0
SNORA11	0	0	0	0	0	0
MIR1292	0	0	0	0	0	0
MIR1296	0	0	0	0	0	0
MIR1233-2	0	0	0	0	0	0
SNORD111	0	0	0	0	0	0
AC000029.1	0	0	0	0	0	0
AC008625.1	0	0	0	0	0	0
AC124853.1	0	0	0	0	0	0
AC112198.2	0	0	0	0	0	0
AC078851.2	0	0	0	0	0	0
AC119751.1	0	0	0	0	0	0

AL117190.1	0	0	0	0	0	0
AC083829.1	0	0	0	0	0	0
MIR320D2	0	0	0	0	0	0
AC119751.2	0	0	0	0	0	0
SNORA77	0	0	1.002934331	0	0	0
AP000547.1	0	0	0	0	0	0
AC096664.3	0	0	0	0	0	0
AC096546.1	0	0	0	0	0	0
MIR1302-5	0	0	0	0	0	0
SNORA3	0	0	0	0	0	0
AL671972.1	0	0	0	0	0	0
AC245033.1	0	0	0	0	0	0
AL138706.1	0	0	0	0	0	0
SNORA11B	0	0	0	0	0	0
AC092865.1	0	0	0	0	0	0
AL159997.1	0	0	0	0	0	0
AL845259.1	0	0	0	0	0	0
AL449083.1	0	0	0	0	0	0
AL590726.2	0	0	0	0	0	0
AP000908.1	0	0	0	0	0	0
AC104133.1	0	0	0	0	0	0
RNU6ATAC30P	0	0	0	0	0	0
SNORD110	2.92038886	0	0	3.01710065	0	0
AC112249.1	0	0	0	0	0	0
MIR1224	0	0	0	0	0	0
AC083811.1	0	0	0	0	0	0
AC104417.1	0	0	0	0	0	0
U3	0	0	0	0	0	0
AC092265.1	0	0	0	0	0	0
AC007450.1	0	0	0	0	0	0
AP001482.1	0	0	0	0	0	0
AC008410.1	0	0	0	0	0	0
AL035422.1	0	0	0	0	0	0

AC007339.1	0	0	0	0	0	0
AC021078.1	0	0	0	0	0	0
AC013751.1	0	0	0	0	0	0
SNORD23	0	0	0	0	0	0
AC126323.1	0	0	0	0	0	0
AC104405.1	0	0	0	0	0	0
SNORA3	0	0	0	0	0	0
AC007551.1	0	0	0	0	0	0
AC009196.1	0	0	0	0	0	0
MIR1281	0	0	0	0	0	0
AL138963.1	0	0	0	0	0	0
SNORA11	0	1.01573397	0	0	2.015212623	0
AL359983.1	0	0	0	0	0	0
AC013444.2	0	0	0	0	0	0
MIR1304	1.16229523	0	0	0	0	0
AC091096.1	0	0	0	0	0	0
AL161908.1	0	0	0	0	0	0
AL050327.1	0	0	0	0	0	0
AL023876.1	0	0	0	0	0	0
MIR1207	0	0	0	0	0	0
AC008045.1	0	0	0	0	0	0
AC016670.1	0	0	0	0	0	0
AC093897.1	0	0	0	0	0	0
MIR1254-1	1.95343737	0	0	0	0	0.906060876
AC103816.1	0	0	0	0	0	0
AC114798.1	0	0	0	0	0	0
MIR548M	0	0	0	0	0	0
AC108211.1	0	0	0	0	0	0
MIR1184-2	0	0.33519221	0	0	0	0
AC004745.1	0	0	0	0	0	0
AL138696.1	0	0	0	0	0	0
AC114765.3	0	0	0	0	0	0
MIR1253	0	0	0	0	0	0

AL049691.1	0	0	0	0	0	0
MIR1262	0	0	0	0	0	0
AC106795.1	0	0	0	0	0	0
RNU6ATAC15P	0	0	0	0	0	0
AP001137.1	0	0	0	0	0	0
AC078794.1	0	0	0	0	0	0
MIR548E	0	0	0	0	0	0
RNU6ATAC27P	0	0	0	0	0	0
AL359851.1	0	0	0	0	0	0
AL035045.1	0	0	0	0	0	0
AL139815.1	0	0	0	0	0	0
AL139139.1	0	0	0	0	0	0
AL138837.1	0	0	0	0	0	0
MIR1305	0	0	0	0	0	0
MIR548L	0	0	0	0	0	0
AC012047.1	0	0	0	0	0	0
AC007842.1	0	0	0	0	0	0
AC009495.1	0	0	0	0	0	0
AC110813.1	0	0	0	0	0	0
AL137190.1	0	0	0	0	0	0
AC106736.1	0	0	0	0	0	0
MIR1285-2	0	0	0	0	0	0
MIR1258	0	0	0	0	0	0
SNORD88A	0	0	1.002934331	0	0	0
SNORA11	0	0	0	1.00570022	0	0
AC144522.1	0	0	0	0	0	0
AC243562.1	0	0	0	0	0	0
AC004980.1	0	0	0	0	0	0
AC120045.1	0	0	0	0	0	0
MIR1263	0	0	0	0	0	0
AL163952.1	0	0	0	0	0	0
RNU6ATAC37P	0	0	0	0	0	0
AC012175.1	0	0	0	0	0	0

AC011465.1	0	0	0	0	0	0
AC002978.1	0	0	0	0	0	0
AC083810.1	0	0	0	0	0	0
MIR1208	0	0	0	0	0	0
AC005159.1	0	0	0	0	0	0
MIR548P	0	0	0	0	0	0
MIR1284	0	0	0	0	0	0
MIR1255A	0	0	0	0	0	0
AC044860.1	0	0	0	0	0	0
MIR1236	0	0	0	0	0	0
MIR1302-8	0	0	0	0	0	0
AC067742.1	0	0	0	0	0	0
MIR1237	0	0	0	0	0	0
MIR548I2	0	0	0	0	0	0
AC003658.1	0	0	0	0	0	0
AC026366.1	0	0	0	0	0	0
AC092289.1	0	0	0	0	0	0
MIR1289-2	0	0	0	0	0	0
MIR663B	0	0	0	0	0	0
AC013406.1	0	0	0	0	0	0
MIR1182	0	0	0	0	0	0
AC090660.1	0	0	0	0	0	0
AC009987.1	0	0	0	0	0	0
AL021394.1	0	0	0	0	0	0
MIR548T	0	0	0	0	0	0
AL590708.1	0	0	0	0	0	0
Z83826.1	0	0	0	0	0	0
SNORD75	0	0	0	0	0	0
AC116562.1	0	0	0	0	0	0
AC018628.1	0	0	0	0	0	0
SNORA79	1.95343737	5.07866986	0	0	0	0
AC068706.1	0	0	0	0	0	0
MIR548I3	0	0	0	0	0	0

AC091167.1	0	0	0	0	0	0
AC008967.1	0	0	0	0	0	0
AL109922.1	0	0	0	0	0	0
AC010207.1	0	0	0	0	0	0
AC093700.1	0	0	0	0	0	0
AL445204.1	0	0	0	0	0	0
AL513487.1	0	0	0	0	0	0
AC135983.1	0	0	0	0	0	0
AL441988.1	0	0	0	0	0	0
AC023385.1	0	0	0	0	0	0
AC016764.1	0	0	0	0	0	0
AC116533.1	0	0	0	0	0	0
MIR1178	0	0	0	0	0	0
AL590787.1	0	0	0	0	0	0
MIR1200	0	0	0	0	0	0
AL121652.1	0	0	0	0	0	0
AL117333.1	0	0	0	0	0	0
AC009159.1	0	0	0	0	0	0
MIR548Q	0	0	0	0	0	0
U3	0	0	0	0	0	0
MIR548K	0	0	0	0	0	0
AC119751.3	0	0	0	0	0	0
AP000745.1	0	0	0	0	0	0
AL356137.1	0	0	0	0	0	0
AC099537.1	0	0	0	0	0	0
AC108456.1	0	0	0	0	0	0
RNU6ATAC18P	0	0	0	0	0	0
AC068946.1	0	0	0	0	0	0
U3	0	0	0	0	0	0
AL356458.1	0	0	0	0	0	0
AC096649.4	0	0	0	0	0	0
MIR548F5	0	0	0	0	0	0
AC068570.1	0	0	0	0	0	0

AL356214.1	0	0	0	0	0	0
AC023310.1	0	0	0	0	0	0
MIR1288	0	0	0	0	0	0
AC004492.1	0	0	0	0	0	0
AC104387.1	0	0	0	0	0	0
AC026150.1	0	0	0	0	0	0
AL355512.1	0	0	0	0	0	0
AC106860.1	0	0	0	0	0	0
RNU6ATAC20P	0	0	0	0	0	0
MIR1228	0	0	0	0	0	0
MIR1306	0	0	0	0.34193807	0	0
MIR548G	0	0	0	0	0	0
MIR1265	0	0	0	0	0	0
AC010739.1	0	0	0	0	0	0
AC010297.1	0	0	0	0	0	0
AC007242.1	0	0	0	0	0	0
RNU6ATAC23P	0	0	0	0	0	0
SNORA77	0	0	0	0	0	0
AC073255.1	0	0	0	0	0	0
AL096864.1	0	0	0	0	0	0
AC004460.1	0	0	0	0	0	0
AC108696.1	0	0	0	0	0	0
SNORD88B	0	1.88926519	0	0	0	0
AC096971.1	0	0	0	0	0	0
AC016907.1	0	0	0	0	0	0
AC023798.1	0	0	0	0	0	0
AL354984.2	0	0	0	0	0	0
AC002979.1	0	0	0	0	0	0
RNU6ATAC8P	0	0	0	0	0	0
AC093865.1	0	0	0	0	0	0
AC010754.1	0	0	0	0	0	0
MIR1295A	0	0	0	0	0	0
Z73964.1	0	0	0	0	0	0

MIR1302-6	0	0	0	0	0	0
MIR1229	0	0	0	0	0	0
AC099506.1	0	0	0	0	0	0
SNORA11	0	0	0	0	0	0
U3	0	0	0	0	0	0
AC025594.1	0	0	0	0	0	0
AL353664.1	0	0	0	0	0	0
AC096633.1	0	0	0	0	0	0
AC123768.1	0	0	0	0	0	0
MIR320B2	0	0	0	0	2.015212623	0
U85056.1	0	0	0	0	0	0
AC068506.1	0	0	0	0	0	0
MIR1238	0	0	0	0	0	0
MIR1227	0	0	0	0	0	0
AC124861.2	0	0	0	0	0	0
AC023593.1	0	0	0	0	0	0
AC118282.1	0	0	0	0	0	0
AL133232.1	0	0	0	0	0	0
MIR1257	0	2.03146794	0	0	0	0.887939659
AC003968.1	0	0	0	0	0	0
MIR1287	0	0	0	0	0	0
SNORA81	0	0	0	0	0	0
MIR1283-1	0	0	0	0	0	0
AC090954.1	0	0	0	0	0	0
AL157904.1	0	0	0	0	0	0
AL121819.1	0	0	0	0	0	0
AC006150.2	0	0	0	0	0	0
AC018635.1	0	0	0	0	0	0
MIR1294	0	0	1.002934331	0	0	0
AC092925.1	0	0	0	0	0	0
AC079834.2	0	0	0	0	0	0
MIR548F3	0	0	0	0	0	0
AC068058.1	0	0	0	0	0	0

AC114877.1	0	0	0	0	0	0
RNU4ATAC16P	0	0	0	0	0	0
RNU6ATAC31P	0	0	0	0	0	0
MIR548F4	0	0	0	0	0	0
AC017083.1	0	0	0	0	0	0
AC139426.1	0	0	0	0	0	0
MIR1301	0	0	0	0	0	0
AC099805.1	0	0	0	0	0	0
AL591415.1	0	0	0	0	0	0
AL118509.1	0	0	0	0	0	0
AL109618.1	0	0	0	0	0	0
AL589675.1	0	0	0	0	0	0
AL354984.3	0	0	0	0	0	0
AC005631.1	0	0	0	0	0	0
U3	0	0	0	0	0	0
MIR1202	0	0	0	0	0	0
AC092958.1	0	0	0	0	0	0
AP003498.1	0	0	0	0	0	0
SNORA11C	0	0	0	0	0	0
U3	0	0	0	0	0	0
MIR1277	0	0	0	0	0	0
MIR1271	0	0	0	0	0	0
MIR548AJ2	0	0	0	0	0	0
AC132803.1	0	0	0	0	0	0
RNU6ATAC11P	0	0	0	0	0	0
AL133260.1	0	0	0	0	0	0
AL132875.1	0	0	0	0	0	0
AC098847.1	0	0	0	0	0	0
AL138710.1	0	0	0	0	0	0
AC068754.1	0	0	0	0	0	0
SNORA11D	0	0	0	0	0	0
MIR1827	0	0	0	0	0	0
AC108739.1	0	0	0	0	0	0

MIR1251	0	0	0	0	0	0
AC000003.1	0	0	0	0	0	0
AC069294.1	0	0	0	0	0	0
AC092017.1	0	0	0	0	0	0
AC007358.1	0	0	0	0	0	0
SNORA34	0	1.01573397	0	0	0	0
AL591610.1	0	0	0	0	0	0
MIR320C1	0	0	0	0	0	0
MIR548I4	0	0	0	0	0	0
AC098680.1	0	0	0	0	0	0
U3	0	0	0	0	0	0
AL034449.1	0	0	0	0	0	0
SNORA77	0	0	0	0	0	0
AC078881.1	0	0	0	0	0	0
SNORD100	0	0	0	0	0	0
MIR1245A	0	0	0	0	0	0
AC011456.1	0	0	0	0	0	0
AC239395.1	0	0	0	0	0	0
AC092038.1	0	0	0	0	0	0
RNU6ATAC40P	0	0	0	0	0	0
MIR548O	0	0	0	0	0	0
AC090825.1	0	0	0	0	0	0
SNORD111B	0	0	0	0	0	0
AC007333.1	0	0	0	0	0	0
AP002806.1	0	0	0	0	0	0
RNU6ATAC16P	0	0	0	0	0	0
AL390071.1	0	0	0	0	0	0
MIR1285-1	0	0	0	0	0	0
AL158151.2	0	0	0	0	0	0
AC079080.1	0	0	0	0	0	0
AC074391.2	0	0	0	0	0	0
MIR1185-1	0	0	0	0	0	0
AC040162.1	0	0	0	0	0	0

RNU6ATAC41P	0	0	0	0	0	0
AC016597.1	0	0	0	0	0	0
AC005037.1	0	0	0	0	0	0
AC074091.1	0	0	0	0	0	0
MIR1184-1	0	0.33519221	0	0	0	0
AC090079.1	0	0	0	0	0	0
AC027319.1	0	0	0	0	0	0
AC091643.1	0	0	0	0	0	0
MIR548H1	0	0	0	0	0	0
SNORD99	0	0	0	0	0	0.906060876
MIR1180	0	0	0	0	0	0
AC079753.1	0	0	0	0	0	0
AC016405.1	0	0	0	0	0	0
MIR1255B2	0	0	0	0	0	0
AL359853.1	0	0	0	0	0	0
MIR1283-2	0	0	0	0	0	0
AL645859.1	0	0	0	0	0	0
AP000478.1	0	0	0	0	0	0
MIR1303	0	0	0	0	0	0
AC004924.1	0	0	0	0	0	0
AC068669.1	0	0	0	0	0	0
AC103794.1	0	0	0	0	0	0
AC024082.1	0	0	0	0	0	0
AL357519.1	0	0	0	0	0	0
AC241929.1	0	0	0	0	0	0
RNU6ATAC10P	0	0	0	0	0	0
MIR1269A	0	0	0	0	0	0
RNU6ATAC42P	0	0	0	0	0	0
AP004242.1	0	0	0	0	0	0
MIR1181	0	0	0	0	0	0
AF228730.2	0	0	0	0	0	0
AC026797.1	0	0	0	0	0	0
RNU6ATAC35P	0	0	0	0	1.007606311	0

AC099535.1	0	0	0	0	0	0
RNU6ATAC33P	0	0	0	0	0	0
AC004543.1	0	0	0	0	0	0
AL049798.1	0	0	0	0	0	0
AC093162.1	0	0	0	0	0	0
AL353707.1	0	0	0	0	0	0
RNU6ATAC21P	0	0	0	0	0	0
MIR1226	0	0	0	0	0	0
MIR1261	0	0	0	0	0	0
AC100817.1	0	0	0	0	0	0
AC009299.1	0	0	0	0	0	0
AL161740.1	0	0	0	0	0	0
AC026150.2	0	0	0	0	0	0
MIR548F1	0	0	0	0	0	0
AC010458.1	0	0	0	0	0	0
MIR1249	0	0	0	0	0	0
AL136446.1	0	0	0	0	0	0
RNU4ATAC2P	0	0	0	0	0	0
AC133041.1	0	0	0	0	0	0
MIR1184-3	0	0.33519221	0	0	0	0
MIR1293	0	0	0	0	0	0
AL356131.1	0	0	0	0	0	0
AP005718.1	0	0	0	0	0	0
AC079235.1	0	0	0	0	0	0
AC107890.1	0	0	0	0	0	0
SNORD88	0	0	0	0	0	0
AC106775.1	0	0	0	0	0	0
AL161452.1	0	0	0	0	0	0
MIR1185-2	0	0	0	0	0	0
AC140132.1	0	0	0	0	0	0
MIR548H4	0	0	0	0	0	0
AC138123.1	0	0	0	0	0	0
AC093817.1	0	0	0	0	0	0

AC012671.1	0	0	0	0	0	0
AC009835.1	0	0	0	0	0	0
AC090150.1	0	0	0	0	0	0
MIR1302-4	0	0	0	0	0	0
AL162614.1	0	0	0	0	0	0
MIR1179	0	0	0	0	0	0
AP000897.1	0	0	0	0	0	0
U3	0	0	0	0	0	0
MIR1276	0	0	0	0	0	0
AL157372.1	0	0	0	0	0	0
AC105252.1	0	0	0	0	0	0
U3	0	0	0	0	0	0
SNORA3	0	0	0	1.00570022	0	0
MIR1268A	0	0	0	0	0	0
SNORA77	0	0	0	1.00570022	0	0
AC027238.1	0	0	0	0	0	0
MIR1233-1	0	0	0	0	0	0
MIR1267	0	0	0	0	0	0
AC007131.3	0	0	0	0	0	0
AC013402.1	0	0	0	0	0	0
AL354978.1	0	0	0	0	0	0
MIR1225	0	0	0	0	0	0
AC020703.1	0	0	0	0	0	0
AC104441.1	0	0	0	0	0	0
MIR1290	0	0	0	0	0	0
AC095030.1	0	0	0	0	0	0
AL591708.1	0	0	0	0	0	0
MIR1825	0	0	0	0	0	0
MIR548N	0	0	0	0	0	0
AL596092.1	0	0	0	0	0	0
U3	0	0	0	0	0	0
RNU6ATAC	2.93015605	0	0	2.01140043	0	0
AL161898.1	0	0	0	0	0	0

AC087237.1	0	0	0	0	0	0
MIR1278	0	0	0	0	0	0
AC104837.1	0	0	0	0	0	0
AL354775.1	0	0	0	0	0	0
AC244205.1	0	0	0	0	0	0
AC004875.1	0	0	0	0	0	0
AC090825.2	0	0	0	0	0	0
AL590226.1	0	0	0	0	0	0
AL390335.1	0	0	0	0	0	0
AL157884.1	0	0	0	0	0	0
MIR1275	0	0	0	0	0	0
MIR548H3	0	0	0	0	0	0
AC024610.1	0	0	0	0	0	0
AC005284.1	0	0	0	0	0	0
AL356270.1	0	0	0	0	0	0
MIR302E	0	0	0	0	0	0
AC105399.1	0	0	0	0	0	0
SNORA11E	0	0	0	0	0	0
AC090573.1	0	0	0	0	0	0
AL592294.1	0	0	0	0	0	0
AL353733.1	0	0	0	0	0	0
MIR1298	0	0	0	0	0	0
SNORD75	0	0	0	0	0	0
SNORA11	0	0	2.005868661	0	0	0
AL583860.1	0	0	0	0	0	0
SNORA3	0	0	0	0	0	0
AC096951.1	0	0	0	0	0	0
AC010455.1	0	0	0	0	0	0
AC019322.1	0	0	0	0	0	0
AC078855.1	0	0	0	0	0	0
RNU6ATAC25P	0	0	0	0	0	0
AL031653.1	0	0	0	0	0	0
AC233287.1	0	0	0	0	0	0

AL162419.1	0	0	0	0	0	0
AC091321.1	0	0	0	0	0	0
AC006452.1	0	0	0	0	0	0
AL109843.1	0	0	0	0	0	0
AC003681.1	0	0	0	0	0	0
MIR548I1	0	0	0	0	0	0
MIR1203	0	0	0	0	0	0
SNORD93	0	0	0	0	0	0
AC093304.1	0	0	0	0	0	0
MIR1197	0	0	0	0	0	0
AL645730.1	0	0	0	0	0	0
AC118282.2	0	0	0	0	0	0
AC107020.1	0	0	0	0	0	0
SNORA11	0	0	0	0	0	0
AC006380.1	0	0	0	0	0	0
MIR1273A	0	0	0	0	0	0
MIR1260A	0	0	0	0	0	0
AL159977.1	0	0	0	0	0	0
MIR548J	0	0	0	0	0	0
AC092786.1	0	0	0	0	0	0
MIR1289-1	0	0	0	0	0	0
AC107068.1	0	0	0	0	0	0
AC084872.1	0	0	0	0	0	0
MIR1307	0	0	1.704988362	0	2.710460977	0.679545657
AL356309.1	0	0	0	0	0	0
AC010203.1	0	0	0	0	0	0
MIR1205	0	0	0	0	0	0
AL353141.1	0	0	0	0	0	0
AC016821.1	0	0	0	0	0	0
AL160056.1	0	0	0	0	0	0
AC034236.2	0	0	0	0	0	0
AL359815.1	0	0	0	0	0	0
MIR548F2	0	0	0	0	0	0

MIR1183	0	0	0	0	0	0
AC120045.2	0	0	0	0	0	0
AC004673.1	0	0	0	0	0	0
MIR1252	0	0	0	0	0	0
AC130462.1	0	0	0	0	0	0
AC011467.1	0	0	0	0	0	0
MIR1282	0	0	3.008802992	1.00570022	0	0.906060876
AC103863.1	0	0	0	0	0	0
MIR1324	0	0	0	0	0	0
AL356739.1	0	0	0	0	0	0
AC092812.1	0	0	0	0	0	0
AC132068.1	0	0	0	0	0	0
MIR548H2	0	0	0	0	0	0
SNORD23	0	0	0	0	0	0
AC021749.1	0	0	0	0	0	0
RNU6ATAC34P	0	0	0	0	0	0
AC078927.1	0	0	0	0	0	0
MIR1256	0	0	0	0	0	0
AC074051.1	0	0	0	0	0	0
OR6B1	0	0	0	0	0	0
PPP3CB-AS1	82.0443694	89.3845896	36.10563591	48.2736103	79.60089859	38.96061768
EBF2	0	0	0	0	2.015212623	3.624243505
GAS8-AS1	0	0	2.005868661	0	0	1.812121752
C6orf226	280.318262	231.587346	168.4929676	274.556159	106.806269	117.7879139
PPP3R1	3255.49128	3146.65243	1284.72879	1601.14514	3008.027273	1722.303938
PSG3	1.95343737	0	0	0	0	1.812121752
FANCG	1238.47929	1116.29164	889.6027513	1369.76369	1357.245701	1645.406551
OR2A5	0	0	0	0	0	0
KRTAP10-9	0	0	0	0	0	0
AP4M1	536.218557	439.81281	428.2529592	519.947011	431.2555012	503.7698472
OR4A5	0	0	0	0	0	0
C2orf16	0	0	1.002934331	0	0	0
DPP3P2	0	0	0	0	0	0

C7orf65	2.93015605	2.03146794	0	0	1.007606311	0
AC018880.1	0	0	0	0	0	0
KRTAP1-5	0	0	0	0	0	0
TAS2R41	0	0	0	0	0	0
CTD-2527I21.4	0	0	0	0	0	0
OR2A12	0	0	0	0	0	0
KRTAP10-10	0	0	0	0	0	0
KRTAP12-2	0	0	0	0	0	0
PLXNA4	16.6042176	35.550689	0	3.01710065	117.8899384	12.68485227
MAGEA3	1.95343737	0	0	0	0	0.906060876
CEBPD	1180.85289	754.690341	175.5135079	113.644124	417.1490129	182.1182361
TMEM257	0	0	0	0	0	0
ZNF816-ZNF321P	0	20.6193996	0	3.34898172	0	0
PSG7	3.24270603	0	0	0	0	0
MRPS21P3	0	0	0	0	0	0
KRTAP1-3	0	0	0	0	0	0
OR3A2	0	0	0	0	0	0
ARIH2OS	17.5809363	12.1888077	50.14671654	42.2394091	17.12930729	29.90000891
ZBED8	165.065458	207.20973	215.6308811	144.820831	176.3311045	94.23033112
HMSD	105.96421	132.45171	0	2.47402253	7.859329228	1.812121752
OR1C1	0	0	0	0	0	0
NPTXR	220.738423	235.650282	46.13497921	83.4731179	1045.895351	179.4000535
FAM218BP	0	0	0	0	0	0
POM121L12	0	0	0	0	0	0
FAM200A	142.600928	137.124086	95.27876142	55.3135119	215.6277506	78.82729623
OR2F2	0	0	0	0	0	0
PPP2R2A	1155.4582	1216.8493	719.1039151	888.03329	2193.55894	1395.333749
C19orf73	17.5809363	25.3933493	25.07335827	36.2052078	26.19776409	15.4030349
ZNF880	107.439055	139.155554	25.07335827	22.1254047	131.9964268	48.02122644
TRIM16	1137.99447	1781.98337	3508.013555	2809.56435	3326.733149	3156.244941
FAM45B	8.55605567	13.3772164	5.636490939	12.3097706	6.942407485	3.814516289
OR6X1	0	0	0	0	0	0
HEPN1	0.97671868	0	0	0	0	0

OR2A25	0	0	0	0	0	0
TAS2R40	0	0	0	0	0	0
OR2A14	0	0	0	0	0	0
TIGD1	84.9745255	62.9755063	270.7922693	162.923435	260.9700346	189.3667231
FXYD7	1.95343737	0	1.002934331	4.02280086	0	1.812121752
XKR9	50.7893716	48.7552307	47.13791354	65.370514	13.09888205	47.11516556
LINC01465	4.88359342	4.06293589	2.005868661	6.03420129	16.12170098	5.436365257
C1orf229	22.4645297	13.2045416	78.2288778	85.0118392	8.06085049	52.55153082
OR4C12	0	0	0	0	0	0
SLC12A8	190.460143	183.847849	295.8656276	189.071641	179.3539234	358.800107
KIR2DS4	0	0	0	0	0	0
PRR21	0	0	0	0	0	0
TMEM14EP	0	0	0	0	0	0
APOL6	1150.57461	1211.77063	51.14965087	20.1140043	51.38792188	258.2273497
FADS3	3380.42336	2777.01668	108.3169077	149.849332	675.0962286	134.0970097
OR2A1	0.48835934	0	0	0	0	0.906060876
TTC4P1	0	0	0	0	0	0
C3orf36	0	0	8.023474646	2.01140043	0	2.718182629
OR4E2	0	0	0	0	0	0
CCNL2	1845.99831	1441.32651	5941.382975	3643.65188	1861.048857	4658.965025
UBA52	6444.38988	6721.11169	5256.378827	5498.16308	6913.186902	5400.122822
MYBPHL	0	0	1.002934331	1.00570022	1.007606311	3.624243505
PPT2	1032.29398	1161.25818	173.297023	206.419969	203.5364749	213.1417605
OR2A2	0	0	0	0	0	0
EXOC3-AS1	78.1374947	136.108352	85.24941811	128.729628	190.4375928	108.7273051
ZNF630	36.1385913	28.4405512	147.4313466	182.031739	159.2017972	95.136392
TIAF1	136.896891	109.485965	122.2476656	65.8130221	50.24932674	87.25366238
OR52B4	0	0	0	0	0	0
AC092675.3	10.7439055	14.2202756	0	0	0	0
AC106876.2	0	0	0	0	0	0
C7orf71	0	0	0	0	0	0
LINC01118	2.93015605	1.01573397	2.005868661	0	3.022818934	3.624243505
AC064874.1	0	0	0	0	0	0

BTBD19	35.1618726	37.582157	13.0381463	31.1767067	9.068456802	16.30909577
FAM185A	104.352624	89.2627015	61.17899417	43.4864773	302.6547077	75.9007196
AC005481.5	0	0	0	0	0	3.624243505
RAB6C	0.98648587	2.79326842	81.23768079	89.8794283	101.9093023	100.1831511
AC011997.1	0	0	0	0	11.08366942	0
C21orf140	0	0	0	0	0	0
URAHP	0.97671868	1.01573397	64.18779717	47.2679101	50.38031556	46.20910469
AC062017.1	2.93015605	3.04720192	5.014671654	3.01710065	2.015212623	2.718182629
AC112721.1	0	1.01573397	0	0	0	0
AC004945.1	0	0	0	0	0	0
PSMB11	0	0	0	0	0	0
AC007131.1	0	0	0	0	0	0
AC023469.1	0	0	0	0	0	0
AC112721.2	1.95343737	2.03146794	0	0	0	0
LINC01124	81.0676508	66.0227082	2.005868661	1.00570022	3.022818934	5.436365257
AC079354.3	0.97671868	0	0	0	0	0
POTEM	0	0	0	0	1.017682374	0
IGLC6	0	0	0	0	0	0
POTEJ	0	0	0	2.3231675	41.45292365	0
LINC00152	1733.34358	1466.77064	189.7652047	243.469965	169.6909789	266.8711705
AP000233.4	0	0	0	0	0	0
AC079305.10	1.95343737	7.11013781	5.014671654	16.0912034	22.16733885	12.68485227
RP5-1039K5.16	0	0	0	0	0	0
DCDC2B	0.97671868	0	1.002934331	1.00570022	0	0
C10orf55	45.9057781	46.7237627	0	0	4.030425245	0
RNU6-1165P	0	0	0	0	0	0
RNA5SP177	0	0	0	0	0	0
AC092117.1	0	0	0	0	0	0
RNU4-62P	0	0	0	0	0	0
AC108137.1	0	0	0	0	0	0
AL163760.1	0	0	0	0	0	0
RNU4-86P	0	0	0	0	0	0
RN7SKP154	0	0	0	0	0	0

RN7SKP285	0	0	0	0	0	0
AC010150.2	0	0	0	0	0	0
MIR1915	0	0	0	0	0	0
Y_RNA	0	0	0	0	0	0
RNU2-3P	0	0	0	0	0	0
RN7SKP110	0	0	0	0	0	0
AL356310.1	0	0	0	0	0	0
AL445074.1	0	0	0	0	0	0
AC024568.1	0	0	0	0	0	0
AC010609.1	0	0	0	0	0	0
RNU6-721P	0	0	0	0	0	0
RNU6-908P	0	0	0	0	0	0
RNU2-65P	0	0	0	0	0	0
SNORD113	0	0	0	0	0	0
AC019051.1	0	0	0	0	0	0
RN7SKP32	0	0	0	0	0	0
AC091705.2	0	0	0	0	0	0
RN7SKP232	0	0	0	0	0	0
RN7SKP117	0	0	0	0	0	0
RNA5SP317	0	0	0	0	0	0
AC093515.1	0	0	0	0	0	0
RN7SKP148	0	0	0	0	0	0
RN7SKP16	4.88359342	1.01573397	0	0	0	0.906060876
RNU6-985P	0	0	0	0	0	0
AL391867.1	0	0	0	0	0	0
RNA5SP487	0	0	0	0	0	0
RNA5SP81	0	0	0	0	0	0
AL512290.1	0	0	0	0	0	0
RNU6-648P	0	0	0	0	0	0
RNA5SP390	0	0	0	0	0	0
RNU2-9P	0	0	0	0	0	0
RNA5SP36	0	0	0	0	0	0
AC003666.1	0	0	0	0	0	0

RNU6-380P	0	0	0	0	0	0
SNORA73	0	0	0	0	0	0
RNU4-37P	0	0	0	0	0	0
RNY4P30	0	0	0	0	0	0
RNA5SP239	0	0	0	0	0	0
RN7SKP218	0	0	0	0	0	0
AP003471.1	0	0	0	0	0	0
Y_RNA	0	0	0	0	0	0
Z97356.1	0	0	0	0	0	0
RN7SKP151	0	0	0	0	0	0
RN7SKP266	0	0	0	0	0	0
AL162590.1	0	0	0	0	0	0
RNU6-257P	0	0	0	0	0	0
RN7SKP146	0	0	0	0	0	0
RNU4-30P	0	0	0	0	0	0
RNA5SP181	0	0	0	0	0	0
RN7SKP26	0	0	0	0	0	1.812121752
RNA5SP156	0	0	0	0	0	0
SNORD113	0	0	0	0	0	0
AC121157.1	0	0	0	0	0	0
AL592466.1	0	0	0	0	0	0
RNU4-26P	0	0	0	0	0	0
RNA5SP266	0	0	0	0	0	0
Y_RNA	0	0	0	0	0	0
RNA5SP407	0	0	0	0	0	0
RNA5SP129	0	0	0	0	0	0
RNA5SP56	0	0	0	0	0	0
RN7SKP65	0	0	0	0	0	0
RNU6-588P	0	0	0	0	0	0
AC091736.1	0	0	0	0	0	0
RN7SKP129	0	0	0	0	0	0
RNU2-17P	0	0	0	0	0	0
Y_RNA	0	0	0	0	0	0

RNU6-447P	0	0	0	0	0	0
AC006926.1	0	0	0	0	0	0
RNA5SP158	0	0	0	0	0	0
RNU2-54P	0	0	0	0	0	0
RNA5SP89	0	0	0	0	0	0
AL157392.1	0	0	0	0	0	0
RNA5SP163	0	0	0	0	0	0
RNU2-43P	0	0	0	0	0	0
RN7SKP156	0	0	0	0	0	0
RNA5SP431	0	0	0	0	0	0
MIR1471	0	0	0	0	0	0
RNA5SP201	0	0	0	0	0	0
RNU6-262P	0	0	0	0	0	0
RNA5SP95	0	0	0	0	0	0
RNU6-101P	0	0	0	0	0	0
RN7SKP199	0	0	0	0	0	0
RN7SKP114	0	1.01573397	0	0	0	0
AC092846.1	0	0	0	0	0	0
RNU6-757P	0	0	0	0	0	0
RNU6-892P	0	0	0	0	0	0
RNA5SP425	0	0	0	0	0	0
AC018690.1	0	0	0	0	0	0
RNU2-33P	0	0	1.002934331	0	0	0
RN7SKP111	0	0	0	0	0	0
RNU6-584P	0	0	0	0	0	0
RNA5SP193	0	0	0	0	0	0
RNU6-1043P	0	0	0	0	0	0
RNU2-36P	0	0	0	0	0	0
RNU6-1156P	0	0	0	0	0	0
AL031655.1	0	0	0	0	0	0
AC092116.1	0	0	0	0	0	0
RNU2-21P	0	0	0	0	0	0
RNA5SP371	0	0	0	0	0	0

RNU6-935P	0	0	0	0	0	0
Y_RNA	0	0	0	0	0	0
RNA5SP198	0	0	0	0	0	0
AL391839.1	0	0	0	0	0	0
AC084882.1	0	0	0	0	0	0
RNA5SP178	0	0	0	0	0	0
RN7SKP267	0	0	0	0	0	0
RNU6-1118P	0	0	0	0	0	0
AC008581.1	0	0	0	0	0	0
RNA5SP118	0	0	0	0	0	0
RNU6-1050P	0	0	0	0	0	0
MIR1912	0	0	0	0	0	0
MIR1908	0	0	0	0	0	0
RNU6-855P	0	0	0	0	0	0
RNU2-2P	0	1.01573397	1.825340482	0	2.992590745	3.5155162
RNU6-1076P	0	0	0	0	0	0
AC104434.1	0	0	0	0	0	0
AC093680.1	0	0	0	0	0	0
AC008598.1	0	0	0	0	0	0
Y_RNA	0	0	0	0	0	0
RN7SKP225	0	0	0	0	0	0
RNU6-174P	0	0	0	0	0	0
AP000807.2	0	0	0	0	0	0
RN7SKP139	0	0	0	0	0	0
RNU6-613P	0	0	0	0	0	0
SNORD19	0	0	0	0	0	0
RNA5SP237	0	0	0	0	0	0
RNY1P15	0	0	0	0	0	0
RN7SKP221	0	0	0	0	0	0
RNU2-29P	0	0	0	0	0	0
RNU6-710P	0	0	0	0	0	0
RNU2-20P	0	0	0	0	0	0
RNU6-355P	0	0	0	0	0	0

RNU6-1186P	0	0	0	0	0	0
RNU4-34P	0	0	0	0	0	0
RNU6-96P	0	0	0	0	0	0
SNORD12B	0	0	0	0	0	0
SNORA36B	0	0	0	0	0	0
RN7SKP202	0	0	0	0	0	0
AC022021.1	0	0	0	0	0	0
RN7SKP127	0	0	0	0	0	0
RN7SKP152	0	0	0	0	0	0
RNA5SP44	0	0	0	0	0	0
RNA5SP203	0	0	0	0	0	0
RN7SKP158	0	0	0	0	0	0
RN7SKP86	0	2.03146794	0	0	0	0
RNU2-28P	0	0	0	0	0	0
AL137118.1	0	0	0	0	0	0
Y_RNA	0	0	0	0	0	0
Y_RNA	0	0	0	0	0	0
RN7SKP229	0	0	0	0	0	0
RNU6-554P	0	0	0	0	0	0
RNU6-791P	0	0	0	0	0	0
RN7SKP281	0	0	0	0	0	0
RNU4-65P	0	0	0	0	0	0
RNA5SP80	0	0	0	0	0	0
AL160279.1	0	0	0	0	0	0
AC103996.1	0	0	0	0	0	0
Y_RNA	0	0	0	0	0	0
RN7SKP125	0	0	0	0	0	0
RNU2-59P	0	0	0	0	0	0
RNA5SP274	0	0	0	0	0	0
RNA5SP113	0	0	0	0	0	0
RNA5SP511	0	0	0	0	0	0
Y_RNA	0	0	0	0	0	0
RNU2-50P	0	0	0	0	0	0

RNA5SP338	0	0	0	0	0	0
RNA5SP231	0	0	0	0	0	0
RNU6-955P	0	0	0	0	0	0
Y_RNA	0	0	0	0	0	0
RNU6-141P	0	0	0	0	0	0
Y_RNA	0	0	0	0	0	0
RN7SKP278	0	0	0	0	0	0
AC118282.3	0	0	0	0	0	0
RNU6-1077P	0	0	0	0	0	0
RNU6-994P	0	0	0	0	0	0
RNU2-26P	0	0	0	0	0	0
RN7SKP56	0	0	1.002934331	0	0	0
RN7SKP150	0	0	0	0	0	0
RNU6-1140P	0	0	0	0	0	0
RN7SKP143	0	0	0	0	0	0
RN7SKP59	0	0	0	0	0	0
RNA5SP296	0	0	0	0	0	0
RNU6-121P	0	0	0	0	0	0
RNA5SP293	0	0	0	0	0	0
RN7SKP271	0	0	0	0	0	0
AP005138.1	0	0	0	0	0	0
RNU2-5P	0	0	0	0	0	0
Y_RNA	0	0	0	0	0	0
RNA5SP261	0	0	0	0	0	0
RN7SKP7	0	0	0	0	0	0
RNU2-23P	0	0	0	0	0	0
AC012150.1	0	0	0	0	0	0
AC005071.1	0	0	0	0	0	0
RNU6-77P	0	0	0	0	0	0
RNU6-285P	0	0	0	0	0	0
SNORA79	0	0	0	0	0	0
RNU6-712P	0	0	0	0	0	0
AC091178.1	0	0	0	0	0	0

RN7SKP200	0	0	0	0	0	0
RN7SKP177	0	0	0	0	0	0
RNA5SP475	0	0	0	0	0	0
RNU4-25P	0	0	0	0	0	0
Y_RNA	0	0	0	0	0	0
Y_RNA	0	0	0	0	0	0
Y_RNA	0	0	0	0	0	0
Y_RNA	0	0	0	0	0	0
AL137019.1	0	0	0	0	0	0
RN7SKP223	0	0	0	0	0	0
RN7SKP240	0	0	0	0	0	0
FO393407.1	0	0	0	0	0	0
RNA5SP451	0	0	0	0	0	0
AC026956.1	0	0	0	0	0	0
RNU6-519P	0	0	0	0	0	0
RN7SKP109	0	0	0	0	0	0
AC060835.1	0	0	0	0	0	0
Y_RNA	0	0	0	0	0	0
MIR1468	0	0	0	0	0	0
RNU6-705P	0	0	0	0	0	0
RNU2-39P	0	0	0	0	0	0
RN7SKP220	0	0	0	0	0	0
RNU6-735P	0	0	0	0	0	0
AC080037.1	0	0	0	0	0	0
RNA5SP251	0	0	0	0	0	0
AC006534.1	0	0	0	0	0	0
RNA5SP60	0	0	0	0	0	0
RNU6-1064P	0	0	0	0	0	0
RNU4-43P	0	0	0	0	0	0
RNU6-1025P	0	0	0	0	0	0
AF216667.1	0	0	0	0	0	0
RN7SKP61	0	0	0	0	0	0
RNA5SP345	0	0	0	0	0	0

Y_RNA	0	0	0	0	0	0
RNU2-47P	0	0	0	0	0	0
RNU2-66P	0	0	0	0	0	0
RN7SKP222	0	0	0	0	0	0
RNA5SP494	0	0	0	0	0	0
AC010999.1	0	0	0	0	0	0
SNORA19	0	0	0	0	0	0
RN7SKP159	0	0	0	0	0	0
RNU6-887P	0	0	0	0	0	0
RN7SKP235	0	0	0	0	0	0
RNU2-49P	0	0	0	0	0	0
Y_RNA	0	0	0	0	0	0
AL136160.1	0	0	0	0	0	0
SNORA7	0	0	0	0	0	0
RNU6-628P	0	0	0	0	0	0
RNA5SP504	0	0	0	0	0	0
RNU4-69P	0	0	0	0	0	0
RNU6-402P	0	0	0	0	0	0
AL355388.1	0	0	0	0	0	0
RNU2-52P	0	0	0	0	0	0
Y_RNA	0	0	0	0	0	0
Y_RNA	0	0	0	0	0	0
AC134025.1	0	0	0	0	0	0
RN7SKP27	0	0	0	0	0	0
RN7SKP254	0	0	0	0	0	0
RNU6-1100P	0	0	0	0	0	0
RNU2-15P	0	0	0	0	0	0
RNU2-48P	0	0	0	0	0	0
RNU2-37P	0	0	0	0	0	0
RNU2-42P	0	0	0	0	0	0
RN7SKP131	0	0	0	0	0	0
AL356476.1	0	0	0	0	0	0
RNU6-1203P	0	0	0	0	0	0

RN7SKP54	0	0	0	0	0	0
RNU2-51P	0	0	0	0	0	0
AL603632.1	0	0	0	0	0	0
RNU2-16P	0	0	0	0	0	0
AC002070.1	0	0	0	0	0	0
Y_RNA	0	0	0	0	0	0
RNU2-70P	0	0	0	0	0	0
MIR1469	0	0	0	0	0	0
RNU6-248P	0	0	0	0	0	0
Y_RNA	0	0	0	0	0	0
RNU2-8P	0	0	0	0	0	0
RNU4-55P	0	0	0	0	0	0
RN7SKP123	0	0	0	0	0	0
U3	0	0	0	0	0	0
RNU6-394P	0	0	0	0	0	0
RNA5SP146	0	0	0	0	0	0
AL513123.1	0	0	0	0	0	0
RN7SKP213	0	0	0	0	0	0
RNU6-1267P	0	0	0	0	0	0
RNA5SP38	0	0	0	0	0	0
AC093004.1	0	0	0	0	0	0
RN7SKP119	0	0	0	0	0	0
RNU4-72P	0	0	0	0	0	0
RNU6-733P	0	0	0	0	0	0
AC007402.1	0	0	0	0	0	0
RN7SKP120	0	0	0	0	0	0
Y_RNA	0	0	0	0	0	0
Y_RNA	0	0	0	0	0	0
RN7SKP182	0	0	0	0	0	0
RN7SKP39	0	0	0	0	0	0
RN7SKP89	0	0	0	0	0	0
RN7SKP51	0	0	0	0	0	0
RN7SKP38	0	0	0	1.00570022	0	0.906060876

MIR1911	0	0	0	0	0	0
AC116038.1	0	0	0	0	0	0
AC027307.1	0	0	0	1.00570022	0	0.906060876
RN7SKP188	0	0	0	0	0	0
AL161790.1	0	0	0	0	0	0
RNU2-63P	1.95343737	0	0	1.00570022	0	0
RNU2-7P	0	0	0	0	0	0.906060876
RNU4-64P	0	0	0	0	0	0
AC008671.1	0	0	0	0	0	0
RNY4P29	0	0	0	0	0	0
RNU4-6P	0	0	0	0	0	0
RNA5SP328	0	0	0	0	0	0
RNA5SP229	0	0	0	0	0	0
RNU6-1190P	0	0	0	0	0	0
RN7SKP166	0	0	0	0	0	0
RNA5SP25	0	0	0	0	0	0
AL021398.1	0	0	0	0	0	0
RNU4-46P	0	0	0	0	0	0.534575917
RN7SKP206	0	0	0	0	0	0
RNU4-53P	0	0	0	0	0	0
RNU6-684P	0	0	0	0	0	0
RN7SKP106	0	0	0	0	0	0
RNU2-40P	0	0	0	0	0	0
Y_RNA	0	0	0	0	0	0
AC138761.1	0	0	0	0	0	0
RN7SKP121	0	0	0	0	0	0
AC243980.1	0	0	0	0	0	0
RNU6-233P	0	0	0	0	0	0
RNA5SP144	0	0	0	0	0	0
RN7SKP212	0	0	0	0	0	0
RN7SKP91	0	0	0	0	0	0
AL645944.1	0	0	0	0	0	0
RNU2-38P	0	0	0	0	0	0

RNU4-14P	0	0	0	0	0	0
RNU6-67P	0	0	0	0	0	0
RNU6-860P	0	0	0	0	0	0
RNU4-83P	0	0	0	0	0	0
RNU6-383P	0	0	0	0	0	0
RNU2-62P	0	0	0	0	0	0.906060876
AC010319.1	0	0	0	0	0	0
RNA5SP225	0	0	0	0	0	0
RNU4-47P	0	0	0	0	0	0
RNU2-68P	0	0	0	0	0	0
RNU4-27P	0	0	0	0	0	0
RN7SKP286	0	0	0	0	0	0
MIR1537	0	0	0	0	0	0
RNA5SP120	0	0	0	0	0	0
AC069067.1	0	0	0	0	0	0
AC074035.1	0	0	0	0	0	0
RNA5SP100	0	0	0	0	0	0
RNA5SP136	0	0	0	0	0	0
RN7SKP168	0	0	0	0	0	0
RNU6-321P	0	0	0	0	0	0
RN7SKP42	0	0	0	0	0	0
RNA5SP21	0	0	0	0	0	0
Y_RNA	0	0	0	0	0	0
RNA5SP59	0	0	0	0	0	0
RNU6-920P	0	0	0	0	0	0
RN7SKP136	0	0	0	0	0	0
RNU6-1086P	0	0	0	0	0	0
AL596268.1	0	0	0	0	0	0
RNU6-565P	0	0	0	0	0	0
RNU6-1328P	0	0	0	0	0	0
RNU4-78P	0	0	0	0	0	0
RN7SKP33	0	0	0	0	0	0
RN7SKP261	0	0	0	0	0	0

Y_RNA	0	0	0	1.00570022	0	0.906060876
Y_RNA	0	0	0	0	0	0
AC023513.1	0	0	0	0	0	0
RN7SKP29	0	0	0	0	0	0
RNU6-1068P	0	0	0	0	0	0
AL662800.1	0	0	0	0	0	0
RNU6-1133P	0	0	0	0	0	0
RN7SKP97	0	1.01573397	18.05281795	5.02850108	0	10.87273051
AL110292.1	0	0	0	0	0	0
RNU6-193P	0	0	0	0	0	0
RNU6-564P	0	0	0	0	0	0
RNA5SP29	0	0	0	0	0	2.718182629
RNA5SP104	0	0	0	0	0	0
RNA5SP501	0	0	0	0	0	0
RNU2-41P	0	0	0	0	0	0
RNU6-1148P	0	0	0	0	0	0
RN7SKP205	0	0	0	0	0	0
RNU6-172P	0	0	0	0	0	0
RN7SKP284	0	0	0	0	0	0
SNORD63	0	0	0	0	0	0
AC079467.1	0	0	0	0	0	0
RNU6-370P	0	0	0	0	0	0
Y_RNA	0	0	0	0	0	0
RN7SKP58	0	0	0	0	0	0
AC091866.1	0	0	0	0	0	0
RN7SKP262	0	0	0	0	0	0
RNA5SP41	0	0	0	0	0	0
AC110611.1	0	0	0	0	0	0
RNA5SP265	0	0	0	0	0	0
MIR1913	0	0	0	0	0	0
RNU6-272P	0	0	0	0	0	0
AC008949.1	0	0	0	0	0	0
SNORA40	0	0	0	0	0	0

RN7SKP8	0	0	0	0	0	0
RNA5SP222	0	0	0	0	0	0
RNU6-651P	0	0	0	0	0	0
RNU2-25P	0	0	0	0	0	0
RN7SKP228	0	0	0	0	0	0
RN7SKP94	0	0	0	0	0	0
RN7SKP167	0	0	0	0	0	0
RN7SKP216	0	0	0	0	0	0
RNA5SP127	0	0	0	0	0	0
RNU2-14P	0	0	0	0	0	0
RNU5A-5P	0	0	0	0	0	0
RN7SKP68	0	0	0	0	0	0
RNU4-22P	0	0	0	0	0	0
AC006322.1	0	0	0	0	0	0
Y_RNA	0	0	0	0	0	0
RN7SKP259	0	0	0	0	0	0
AC105247.1	0	0	0	0	0	0
RNU2-61P	0	0	0	0	0	0
RNA5SP184	0	0	0	0	0	0
SNORD29	0	0	0	0	0	0
RN7SKP137	0	0	0	0	0	0
RN7SKP73	0	0	0	0	0	0
AL136234.1	0	0	0	0	0	0
RN7SKP264	0	0	0	0	0	0.906060876
RNA5SP344	0	0	0	0	0	0
RNU6-1135P	0	0	0	0	0	0
AL096700.1	0	0	0	0	0	0
RNA5SP306	0	0	0	0	0	0
Y_RNA	0	0	0	0	0	0
RNU6-55P	0	0	0	0	0	0
RN7SKP247	0	0	0	0	0	0
SNORA57	0	0	0	0	0	0
AC004969.1	0	0	0	0	0	0

AC091697.1	0	0	0	0	0	0
AC024158.1	0	0	0	0	0	0
RNU6-284P	0	0	0	0	0	0
RN7SKP268	0	0	0	0	0	0
RN7SKP144	0	0	0	0	0	0
RN7SKP161	0	0	0	0	0	0
RNU6-130P	0	0	0	0	0	0
RNA5SP369	0	0	0	0	0	0
RNU6-847P	0	0	0	0	0	0
AC007096.1	0	0	0	0	0	0
AC022142.1	0	0	0	0	0	0
RN7SKP169	0	0	0	0	0	0
AL354896.1	0	0	0	0	0	0
Y_RNA	0	0	0	0	0	0
RNU6-1245P	0	0	0	0	0	0
AL157823.1	0	0	0	0	0	0
RNU6-325P	0	0	0	0	0	0
AC006377.1	0	0	0	0	0	0
RN7SKP126	0	0	0	0	0	0
RNA5SP31	0	0	0	0	0	0
RNU2-55P	0	0	0	0	0	0
Y_RNA	0	0	0	0	0	0
AC121336.1	0	0	0	0	0	0
RNA5SP155	0	0	0	0	0	0
RNU6-602P	0	0	0	0	0	0
RMRPP5	0	0	0	0	0	0
RNU6-981P	0	0	0	0	0	0
RNA5SP115	0	0	0	0	0	0
RNU5E-9P	0	0	0	0	0	0
AC119751.4	0	0	0	0	0	0
AP002364.1	0	0	0	0	0	0
RNU2-72P	0	0	0	0	0	0
MIR1538	0	0	0	0	0	0

SNORA74	0	0	1.002934331	0	0	0
RNA5SP247	0	0	0	0	0	0
AL157931.1	0	0	0	0	0	0
RN7SKP296	0.97671868	0	0	0	0	0
RN7SKP102	0	0	0	0	0	0
RNU6-1139P	0	0	0	0	0	0
RN7SKP181	0	0	0	0	0	0
AC010546.1	0	0	0	0	0	0
RNU2-32P	0	0	0	0	0	0
RN7SKP263	0	0	0	0	0	0
RN7SKP140	0	0	0	0	0	0
RNA5SP304	0	0	0	0	0	0
AL122015.1	0.94741712	0	0	0	0	0
RN7SKP207	0	0	0	0	0	0
RNA5SP450	0	0	0	0	0	0
RN7SKP252	0	0	0	0	0	0
RN7SKP112	0	0	0	0	0	0
AC010376.1	0	0	0	0	0	0
RNU4-88P	0	0	0	0	0	0
RNU2-18P	0	0	0	0	0	0
RNY1P3	0	0	0	0	0	0
RNA5SP280	0	0	0	0	0	0
AL445384.1	0	0	0	0	0	0
RN7SKP142	0	0	0	0	0	0
RNA5SP209	0	0	0	0	0	0
RN7SKP17	0	0	0	0	0	0
RNU4-61P	0	0	0	0	0	0
RNA5SP39	0	0	0	0	0	0
RNU6-373P	0	0	0	0	0	0
AC087738.1	0	0	0	0	0	0
RNU6-1199P	0	0	0	0	0	0
Y_RNA	0	0	0	0	0	0
RNU6-177P	0	0	0	0	0	0

RN7SKP100	0	0	0	0	0	0
RNU6-293P	0	0	0	0	0	0
RNU6-1001P	0	0	0	0	0	0
RNU2-22P	0	0	0	2.01140043	2.015212623	1.812121752
AC008836.1	0	0	0	0	0	0
AC004699.1	0	0	0	0	0	0
RN7SKP297	0	0	0	0	0	0
RNA5SP221	0	0	0	0	0	0
RNU6-1217P	0	0	0	0	0	0
RNU4-74P	0	0	0	0	0	0
AC091559.1	0	0	0	0	0	0
RNU6-607P	0	0	0	0	0	0
RNU6-938P	0	0	0	0	0	0
Y_RNA	0	0	0	0	0	0
RN7SKP192	0	0	0	0	0	0
SNORD71	0	0	0	0	0	0
RNU6-1054P	0	0	0	0	0	0
RN7SKP270	0	0	0	0	0	0
RNA5SP294	0	0	0	0	0	0
AL161935.1	0	0	0	0	0	0
MIR1909	0	0	0	0	0	0
RNU4-63P	0	0	0	0	0	0
RNU2-64P	0	0	0	0	0	0
Y_RNA	0	0	0	0	0	0
AC022872.1	0	0	0	0	0	0
Y_RNA	0	0	0	0	0	0
RNU6-785P	0	0	0	0	0	0
RNU6-575P	0	0	0	0	0	0
RNA5SP508	0	0	0	0	0	0
RN7SKP194	0	0	0	0	0	0
RNA5SP492	0	0	0	0	0	0
RNU6-387P	0	0	0	0	0	0
RNU6-592P	0	0	0	0	0	0

RN7SKP53	0	0	0	0	0	0
Y_RNA	0	0	0	0	0	0
RN7SKP172	0	0	0	0	0	0
RNA5SP498	0	0	0	0	0	0
AC006026.1	0	0	0	0	0	0
RNU6-57P	0	0	0	0	0	0
RN7SKP85	0	0	0	0	0	0
RN7SKP165	0	0	0	0	0	0
RNU6-195P	0	0	0	0	0	0
AL121952.1	0	0	0	0	0	0
RNU6-954P	0	0	0	0	0	0
RNA5SP107	0	0	0	0	0	0
AL354680.1	0	0	0	0	0	0
RNA5SP270	0	0	0	0	0	0
SNORD83	0	0	0	0	0	0
AC116154.1	0	0	0	0	0	0
RNY3P8	0	0	0	0	0	0
RN7SKP108	0	0	0	0	0	0
Y_RNA	0	0	0	0	0	0
Y_RNA	0	0	0	0	0	0
RN7SKP30	2.93015605	5.07866986	2.005868661	0	0	0
RNU6-1304P	0	0	0	0	0	0
AC119674.1	0	0	0	0	0	0
RN7SKP101	0	0	0	0	0	0
RNU6-722P	0	0	0	0	0	0
RNU6-516P	0	0	0	0	0	0
RN7SKP279	0	0	0	0	0	0
AC027281.1	0	0	0	0	0	0
RNA5SP111	0	0	0	0	0	0
RN7SKP293	0	0	0	0	0	0
AL033378.1	0	0	0	0	0	0
RN7SKP273	0	0	1.002934331	1.00570022	0	0
RNU2-71P	0	0	0	0	0	0

RNU6-1052P	0	0	0	0	0	0
RNU6-603P	0	0	0	0	0	0
RNU2-6P	0	0	0	0	0	0
RN7SKP3	0	0	0	0	0	0
RP3-429O6.1	0	0	0	0	0	0
RP13-131K19.2	1.95343737	1.01573397	19.05575228	9.05130194	12.09127574	8.154547886
RP11-541F9.1	0	0	0	0	0	0
HIST2H2BA	37.7306428	20.2740501	0	1.00570022	6.166550625	6.360547351
KLF2P3	0	0	0	0	0	0
IGLV9-49	0	0	0	0	0	0
ZNF385D-AS2	0	0	0	0	0	0
RP11-290P14.2	0	0	0	0	0	0
RP11-66D17.5	1.95343737	0	9.026408976	4.02280086	0	2.718182629
EHHADH-AS1	0	0	0	0	0	0
AC096559.2	0	0	0	0	0	0
FTH1P10	32.5344994	35.4491156	99.9524354	66.4667272	42.2590087	46.02789251
CDY15P	0	0	0	0	0	0
RP11-401G5.1	0	0	0	0	0	0
AC108066.1	0	0	0	0	0	0
AC005104.3	0.97671868	0	0	0	0	0
RP11-224O19.5	0	0	0	0	0	0
RP5-1014O16.1	0	0	0	0	0	0
RP11-374M1.4	0	0	11.03227764	9.05130194	0	1.812121752
RP11-655H13.2	0	0	0	0	0	0
RP5-1125N11.2	0	1.01573397	3.008802992	2.01140043	1.007606311	0
HIST1H2APS6	0	0	0	0	0	0
RP11-408H1.3	1.95343737	2.03146794	0	0	0	0
SDCBP2P1	0	0	0	0	0	0
RP11-91A18.4	0.97671868	2.03146794	1.002934331	2.01140043	3.022818934	0.906060876
GS1-309P15.3	0	0	0	0	0	0
CLDN10-AS1	0	0	0	0	0	0
RP5-858B6.3	2.93015605	2.03146794	2.005868661	6.03420129	1.007606311	3.624243505
TRBV29OR9-2	0	0	0	0	0	0

RBM22P7	0	0	0	0	0	0
RP11-134G8.5	20.3450502	27.5873347	460.6878555	188.981128	70.05886682	134.4413128
AC027124.2	0	0	0	0	0	0
AP006748.1	0	0	0	0	0	0
RP11-211G3.2	0	0	0	0	0	0
AC074121.4	0	0	0	0	0	0
MEG9	0	0	0	0	0	0
LINC00397	1.95343737	0	0	0	0	0.906060876
XKRYP5	0	0	0	0	0	0
USP9YP18	0	0	0	0	0	0
RPL17P17	0	0	0	0	0	0
RP1-191L6.2	0	0	0	0	0	0
LINC00473	6.83703079	1.01573397	8.023474646	0	0	28.99394804
RPS26P15	0	0	0	0	0	0
TRIM49D1	0	0	0	0	0	0
GS1-590J6.2	1.95343737	1.01573397	0	0	0	0
AC006022.4	0	0	0	0	0	0
RP11-572H4.1	0	0	0	0	0	0
AC007274.2	0	0	0	0	0	0
AC016716.1	2.93015605	0	0	1.00570022	0	0
RP11-399K21.5	0	0	0	0	0	0
RP4-800M22.2	0	0	0	0	0	0
AC011243.1	0	0	0	0	0	0
MTND6P21	0	0	0	0	1.007606311	0
RP11-62L18.3	0	0	0	0	0	0
AC079767.3	0	0	0	0	0	0
AC011625.1	0	0	0	0	0	0
TMSB4XP3	0	0	0	0	0	0
RP1-171K16.5	0	0	0	0	0	0
RP11-555J4.4	0	0	0	0	0	0
TH2LCRR	0	2.03146794	0	0	0	0
USP17L2	0	0	0	0	0	0
RPL6P21	0	0	0	0	0	0

RP11-274J16.5	0	0	0	0	0	0
RP11-52I18.1	0	0	0	0	0	0
HMGN2P18	0	0	0	0	0	0
RP11-781P14.3	0	0	0	0	0	0
HTATSF1P1	0	0	0	0	0	0
LMO7DN-IT1	0	0	0	0	0	0
TCAF1P1	248.330725	242.729947	121.4252594	265.223261	299.7427255	332.8777053
GAPDHP69	0	0	0	0	0	0
AC004471.9	0	0	0	0	0	0
RP11-285G1.9	0	0	0	0	0	0
AC064834.2	0	0	0	0	0	0
CALM1P1	0	0	0	0	0	0
RP11-344J7.2	0	0	0	0	0	0
RP11-490O24.2	0	0	0	0	0	0
GS1-124K5.3	5.8603121	5.07866986	29.08509559	26.1482056	35.2662209	36.24243505
RP11-310H4.1	0	0	0	0	0	0
VN1R42P	0.97671868	0	1.002934331	2.01140043	0	1.812121752
RP11-545E17.3	140.64749	147.281426	28.08216126	116.661225	77.58568597	57.98789608
RP4-788P17.1	0	0	0	0	0	0
NUTM2A-AS1	645.2985	539.730561	369.8621225	364.676955	338.0821456	368.5855644
TRPC6P	0	0	0	0	0	0
LINC01615	56.6496837	37.582157	0	0	2.015212623	0
AC092198.1	0	0	1.002934331	2.01140043	0	0
FRMPD4-AS1	0	0	0	0	0	0
MAPK6PS2	0	0	0	1.00570022	0	0
NEFHP1	0	0	0	0	0	0
CHCHD2P5	0	0	0	0	0	0
RP11-15M15.1	0	0	0	0	0	0
RP11-763B22.7	0	0	0	0	1.007606311	0
EXOSC6	1103.69211	1117.30737	890.6056857	793.49747	2139.148199	1026.566973
AC063979.1	0	0	0	0	0	0
RP11-716A19.3	0	0	0	0	0	0
AC083862.6	0	0	3.008802992	2.01140043	0	0.906060876

VPS52	1709.2577	1675.96105	1729.058786	1730.81007	1703.862272	2008.736962
RP11-96B5.3	0	0	0	0	0	0
RP11-29H23.6	0	0	0	0	1.007606311	0.906060876
RP11-542F9.1	0	0	0	0	0	0
RP11-397P13.7	0	0	0	0	0	0
SLC20A1P1	0	0	0	0	0	0
RPL23AP53	14.6996162	12.2395944	2.076074065	7.34161157	41.53353215	18.25712665
RP11-632K20.7	222.232802	145.382003	70.54640082	83.925683	77.54538172	69.15962668
CDRT15	0	0	0	0	0	0
RP13-297E16.4	4.88359342	0.50786699	0	0	0	0
ATP6V0E1P2	0	0	0	0	0	0
AC004866.1	0	0	0	0	0	0
AFF2-IT1	0	0	0	0	0	0
AC010723.1	0	0	0	0	0	0
CSNK1A1P1	0.97671868	0	0	0	0	0
KIF28P	0	0	38.11150457	8.04560172	0	9.966669638
AC093690.1	0	0	0	0	0	1.812121752
AC079613.1	0	0	0	0	12.09127574	0
RABGAP1L-IT1	0	0	1.002934331	0	0	0
RP11-223P11.3	0	0	0	0	0	0
EEF1A1P8	0	0	0	0	2.015212623	0
AC012506.1	0	0	0	0	0	0
HLA-DQB1-AS1	0	0	0	0	0	0
FAM90A17P	0	0	0	0	0	0
AC008069.1	0	0	0	0	0	0
RP5-919F19.5	0	0	0	0	0	0
RP11-216P16.6	0	0	0	0	0	0
AC011431.1	0	0	0	0	0	0
RPS15AP5	0	0	0	0	0	0
RP1-283K11.3	2.93015605	0	0	0	0	0
TCEAL4P1	0	0	0	0	0	0
AC005838.2	0	0	1.002934331	0	0	0
LINC00630	125.99671	79.2272498	107.3139734	101.575722	139.049671	89.70002674

ZNF844	291.062168	244.791887	0	0	16.12170098	11.77879139
AC034228.3	0	0	0	0	0	0
MTND5P28	0	0	0	0	0	0
SNRPBP1	0	0	0	0	0	0
TMSB4XP4	10.7732071	6.10456117	6.037664671	2.01140043	8.081002617	2.727243237
RP11-24F11.2	47.8592155	46.7237627	0	0	1.007606311	1.812121752
SMPD4P1	0	0	0	0	1.007606311	2.718182629
AC078851.1	0	0	0	0	0	0
USP9YP23	0	0	0	0	0	0
TRIM60P17	0	0	0	0	0	0
RP11-700P18.1	0.97671868	2.03146794	13.04817564	6.03420129	6.055713931	3.633304113
AC003090.1	0	0	0	0	0	0
RP11-543B16.3	0	0	0	0	0	0
AP001601.2	0	0	0	0	0	0
CYP4F32P	0	0	0	0	0	0
RP11-344L13.1	0	0	0	0	0	0
TNRC18P2	0	0	2.005868661	3.01710065	0	0
RPL3P11	0	0	0	1.00570022	0	0
USP17L15	0	0	0	0	0	0
RP11-275I14.2	0	0	0	0	0	0
DHRSX-IT1	1.95343737	2.03146794	1.504401496	0	1.511409467	0.453030438
CKMT1A	0.97671868	0	1197.403297	2698.77641	292.4678079	3164.553519
TINCR	0	1.01573397	122.3579883	644.653838	929.013019	178.4939926
TPMTP4	0	0	0	0	0	0
RBMX2P3	0	0	1.002934331	0	0	0
RP11-61K9.2	0	0	0	0	0	0
RP11-393K12.2	0	0	0	0	0	0
RP3-476K8.4	0.97671868	0	1.002934331	0	0	5.436365257
TVP23CP1	0	0	0	0	0	0
LINC01312	0	0	0	0	0	0
AY269186.1	0	0	0	0	0	0
RP5-884C9.3	0	0	0	0	0	0
CENPVP1	0	0	0	0	0	0

FNDC3CP	0	0	0	0	0	0
AL356806.3	0	0	0	0	0	0.906060876
LYPLA1P1	0	0	0	0	0	0
CTD-2296D1.5	0	0	0	0	0	0
RP1-292B18.4	0	0	0	0	0	0
RP11-216N14.7	2.93015605	3.04720192	14.04108063	23.131105	5.038031556	7.248487009
EEF1A1P41	0	0	0	0	0	0
EBLN1	0	1.01573397	1.002934331	0	0	0
AC125634.1	0	0	0	0	0	0
CRPP1	0	0	0	0	0	0
AC007394.3	0	0	0	0	0	0
AC107399.1	0	0	0	0	0	0
DSCR4-IT1	0	0	0	0	0	0
HBD	0	1.01573397	0	0	0	0
SUPT20HL2	0	0	0	0	0	0
RP11-289I10.2	3.07666385	2.21430006	0	0	2.156277506	0.960424529
ZNF735	0	0	0	0	0	0
RP11-196I18.2	0	0	0	0	0	0
LINC00370	0	0	0	0	0	0
MTCO1P17	0	0	0	0	0	0
RBM48P1	0	1.01573397	0	0	0	0
AK4P4	0	0	4.011737323	2.01140043	0	0.906060876
GSTA6P	0	0	0	0	0	0
RP3-425P12.1	0	0	0	0	0	0
RP1-125I3.4	0	0	0	0	0	0
CYCSP32	0	0	0	0	0	0
LINC01044	0	0	0	0	0	0
AC023449.2	0	0	0	0	0	0
DEFA8P	0	0	0	0	0	0
LINC01120	0	0	0	0	0	2.718182629
RP11-143A22.1	0	0	0	0	0	0
AC012506.3	0	0	0	0	0	0
RP4-613A2.1	0	0	0	0	0	1.812121752

UBE2Q2P5Y	0	0	0	0	0	0
RBMY2EP	0	0	0	0	0	0
RFPL4A	6.83703079	1.01573397	0	0	0	0
RPL30P3	0	0	0	0	0	0
TTTY17C	0	0	0	0	0	0
AC008277.1	0	0	0	0	0	0
RP11-108M9.1	0	0	0	0	0	0
AC002463.3	0	0	0	0	0	0
AL133249.1	0	0	0	0	0	0
IGHV3-64	0	0	0	0	0	0
RP11-359E8.3	0	0	0	0	0	0
UHRF2P1	0	0	0	0	0	0
RP5-1121H13.1	0	0	0	0	0	0
AC106786.1	1.95343737	0	12.03521197	24.1368052	37.28143352	16.30909577
RP11-131L23.1	2.93015605	0	0	0	0	0
RAB9AP3	0	0	0	0	0	0
HMGB3P10	0	0	0	0	0	0
KRT8P51	0	0	0	0	0	0
C1GALT1C1L	32.2317166	20.3146794	0	0	100.7606311	5.436365257
AC012314.20	0	0	0	0	0	0
SAMSN1-AS1	0	0	0	0	0	0
RP5-890O3.3	0	0	0	1.00570022	0	0.906060876
CTB-111F10.1	0	0	0	0	0	0
EEF1A1P24	0	0	0	0	0	0
RP11-93B14.4	0.97671868	0	0	0	0	0
RPL34P3	0	0	0	0	0	0
RP11-272P10.3	0	0	0	0	0	0
RP11-86H7.6	0	0	0	0	0	0
RPL34P26	0	0	0	0	0	0
OR2AD1P	0	0	0	0	0	0
RP11-311H10.4	0	0	0	0	0	0
AP000362.1	0	0	0	0	0	0
RPSAP65	0	0	0	0	0	0

IFNWP18	0	0	0	0	0	0
LINC00571	0.97671868	3.04720192	7.020540315	5.02850108	0	2.718182629
RPS24P14	0	0	0	0	0	0
AC068044.1	0	0	0	0	0	0
DIP2A-IT1	0	3.04720192	2.005868661	0	0	0.906060876
ADH5P3	0	0	0	0	0	0
RP4-633O19__A.1	0.97671868	0	0	0	1.007606311	2.718182629
AF230666.2	4.88359342	3.04720192	3.008802992	1.00570022	0	0
GOLGA6L11P	0	0	0	0	0	0
RAET1E-AS1	1.43577647	1.01573397	0	0.55313512	0	0
ZDHHC20P2	0	0	0	0	0	0
AC027612.4	2.94969043	0	0	0	0	1.812121752
LINC01422	0	0	4.011737323	0	0	0
NSUN5P1	829.439274	734.162358	1332.51861	1047.55746	754.7978878	1241.113128
SFR1P2	0	0	0	0	0	0
TRIM64EP	0	0	0	0	0	0
CRYGGP	0	0	0	0	0	0
AC091633.3	0	0	0	0	2.015212623	0.906060876
RP5-1172N10.2	0	1.01573397	0	1.00570022	4.030425245	0
LINC01208	0	0	34.09976724	6.03420129	0	25.36970453
RP11-113O24.3	0	0	0	0	0	0
DNAJA1P1	0	0	0	0	0	0
AC093107.7	0.97671868	2.03146794	0	1.00570022	0	1.812121752
AC024162.2	0	0	0	0	0	0
RP5-1024N4.2	0	0	0	0	0	0
UQCRFS1P2	0	0	0	0	0	0
RP11-467L13.5	0	2.03146794	0	0	0	0
BX842568.2	4.88359342	0	0	0	2.015212623	0.906060876
RAD17P2	0	0	1.002934331	0	0	0
AC007879.5	0.97671868	0	0	0	0	0
CTA-929C8.7	0	0	0	0	0	0
AC026188.1	0	0	0	0	0	0
RP11-314N2.2	0	0	0	0	0	0

RP11-145E17.2	0	0	0	0	0	0
RP1-127B20.4	0	0	1.002934331	0	0	0
SUPT20HL1	0	0	0	0	0	0
RP11-321C24.1	0	0	0	0	0	0
RPL18AP14	0	0	0	0	0	0
RP11-644K8.1	0	0	0	0	0	0
OR51B3P	0	0	0	0	0	0
RP11-781E19.1	0	0	0	0	0	0
AC007389.2	0	0	0	0	0	0
RP11-760D2.4	0	0	0	0	0	0
PSMD4P1	0	0	0	0	0	0
RP11-149B9.2	0	0	0	0	0	0
RBMY2GP	0	0	0	0	0	0
RP4-717I23.3	164.088739	141.187022	346.0123441	186.05454	71.5400481	123.2242792
TCEB1P30	0	0	0	0	0	0
MIR503HG	119.159679	43.6765608	0	0	6.045637868	4.530304381
SIRPB3P	0	0	0	0	0	0
AC116609.2	0	0	0	3.01710065	0	0
GHc-602D8.2	0	0	0	0	0	0
AC008073.9	0	0	0	0	0	0
TSSC2	0	0	0	0	0	0.906060876
MED15P9	0	0	5.014671654	4.02280086	0	4.530304381
RP11-57C13.4	0	0	0	0	0	0
RP11-54O7.3	0	0	523.5317206	921.221397	230.7418453	1119.891243
RP1-288M22.2	0	0	0	0	0	0
LINC00205	117.206242	128.998214	97.28463008	47.2679101	123.9355763	75.20305272
AC006145.4	0	0	0	0	0	0
GEMIN8P3	0	0	0	0	0	0
CD99P1	32.7200759	28.9484182	36.60710307	30.1710065	40.80805561	19.0272784
RP11-307B6.3	0	0	1.002934331	1.00570022	0	2.718182629
LGALS8-AS1	3.90687474	3.04720192	122.3579883	50.2850108	19.14451991	40.77273943
AC092162.2	0	0	0	0	0	0
RP11-403I13.4	0	0	0	0	0	0

RPS21P8	0	0	0	0	0	0
AC124944.5	0	0	0	0	0	0
RP11-554I8.2	149.437959	148.29716	0	0	4.030425245	0.906060876
RP11-554D15.1	0	0	11.03227764	0	0	0
RP4-593M8.1	0	0	0	0	0	0
DIS3L2P1	0	0	0	0	0	0
UBE2E1-AS1	0.97671868	0	0	0	0	0
COX5BP8	0	0	0	0	0	0
RP11-473E2.2	0	0	0	0	1.007606311	0
ENTPD3-AS1	38.0920287	37.582157	64.18779717	62.3534134	38.28903983	42.58486118
IL10RB-AS1	2.93015605	3.04720192	0	0	0	0
RP11-598C10.2	0	0	0	0	0	0
CERS1	14.5628756	10.523004	30.78005461	25.1425054	245.2614522	65.23638308
RPS20P14	0.98648587	0	0	5.31009714	3.194112007	0
CH17-472G23.2	40.6901004	74.747863	0	0	9.068456802	6.07060787
LINC00114	0	0	0	0	0	0
BOLA3P4	0	0	0	0	0	0
RP11-428L9.2	0	0	1.002934331	3.01710065	66.50201655	9.060608762
RP11-85L21.6	0	0	0	0	0	0
KRT8P28	0	0	0	0	0	0
RP11-437J19.1	0	0	0	0	0	0
RP11-197K6.1	2.93015605	0	0	0	0	0
AC007255.8	0.97671868	1.01573397	121.355054	121.689726	29.22058303	44.39698293
RP11-543D5.2	0	0	0	0	0	0
DIAPH3-AS2	0	0	0	0	0	0
IGKV1OR2-2	0	0	0	0	0	0
CDH23-AS1	0	0	0	0	0	0
RP3-347M6.1	0	0	0	0	0	0
CFL1P1	4.88359342	6.09440383	12.03521197	1.00570022	0	17.21515665
RP11-505P4.6	0	0	0	0	0	0
EEF1A1P1	0	0	0	2.01140043	0	0
LINC01342	0	0	0	0	5.038031556	0.906060876
AC019178.3	0	0	0	0	0	0

DAZAP2P1	0.97671868	0	1.002934331	0	3.022818934	0
AC092570.2	0	0	0	0	0	0
BANF1P4	0	0	0	0	0	0
AC004870.4	0.97671868	0	0	0	0	0
RP1-56J10.8	0	0	0	0	0	0
RP11-472N13.2	0	0	0	0	0	0
RP11-548K12.2	0	0	0	0	0	0
BRD2-IT1	6.83703079	8.12587178	4.011737323	7.03990151	0	0.906060876
AC007091.1	0	0	0	0	0	0
FAM95B1	42.5263315	41.6552502	28.04204389	53.4931945	3.022818934	1.929909666
RP11-448K10.1	0	0	0	0	0	0
RP11-135J2.3	0	0	0	0	0	0
EFCAB6-AS1	0	0	0	0	0	0.906060876
VN1R40P	0	0	0	0	0	0
RP5-925F19.1	0	0	0	0	0	0
RP11-80I15.1	0	0	0	0	0	0
MYCNUT	0	0	0	0	0	0
HRAT92	1.95343737	12.1888077	18.05281795	37.210908	137.0344583	32.61819154
RAB9AP2	0	0	0	0	0	0
AC007403.3	0	0	0	0	0	0
RP11-74C1.2	0.97671868	2.03146794	5.014671654	8.04560172	4.030425245	9.069669371
AC008074.4	0	0	0	0	0	0
NPM1P19	0	0	0	0	0	0.906060876
HLA-DPB1	247.109827	192.989455	0	0	4.030425245	9.966669638
AC002486.3	0	0	0	0	0	0
AC098592.7	0	0	0	0	0	0
RP11-323K10.1	0	0	0	0	0	0
AP000233.3	0	0	0	0	0	0
AC006372.5	0	0	0	0	0	0.906060876
SAP18P2	0	0	0	0	0	0
AC007557.1	0	0	0	0	0	0
NBEAP3	37.11531	11.1730737	1.002934331	2.01140043	5.038031556	7.248487009
RPS27P1	0	0	0	0	0	0

RPS8P10	0	0	0	0.50285011	0	0
AC005517.3	0	0	0	0	0	0.906060876
LINC01078	0	0	0	0	0	0
RP11-553K8.2	0	0	0	0	0	0
ABCC5-AS1	0	0	0	0	0	0
RP11-424D14.1	0	0	0	0	0	0
AC019048.1	0	0	0	0	0	0
FAM90A13P	0	0	0	0	0	0
RP11-251G23.2	0	0	0	0	0	0
RP11-73B2.3	0	0	0	0	0	0
AC008171.1	0	0	0	0	0	0
OSER1-AS1	117.206242	118.840875	123.3609227	146.832231	33.25100827	56.17577432
GNL2P1	0	0	0	0	0	0.906060876
CCNJP2	0	0	0	0	0	0
AC069157.1	0	0	0	0	0	0
SEC13P1	0	0	2.005868661	0	0	0
AP001469.5	0	0	0	3.01710065	0	0
RPL23P4	0	0	0	0	0	0
HMGB1P12	0	0	0	0	0	0
RP11-335E14.1	0	0	0	0	0	0
RP5-837M10.2	0	0	0	0	0	0
LINC01226	1.95343737	0	0	1.00570022	0	0.906060876
AC068657.2	0	0	0	0	0	0
ZNF32-AS3	0	0	0	0	0	0
AC009480.3	0	0	0	0	0	0
EEF1A1P36	0	0	0	0	0	0
AC079630.2	0	0	0	0	0	0
DPPA2P1	0	0	0	0	0	0
RP11-353H3.1	0	1.01573397	0	0	0	1.812121752
AC009237.2	0	0	0	0	0	0
RP13-461N9.2	0	0	0	0	0	0
RP11-430G17.1	0	0	0	0	0	0
MTND1P27	0	0	0	0	0	0

ASS1P2	3.90687474	3.04720192	4.011737323	4.02280086	13.09888205	9.966669638
AC010136.2	0	0	0	0	0	0
RP11-183E9.2	0	0	0	0	0	0
MIR4432HG	0	0	0	0	0	0
RP11-33A14.1	0	0	0	2.01140043	0	2.718182629
RP11-1166P10.8	0	0	0	0	0	0
AC008074.3	0	1.01573397	2.005868661	3.01710065	0	0.906060876
KRT8P8	1.96320455	3.04720192	4.011737323	4.06302887	2.015212623	2.491667409
LINC01014	0	0	0	0	0	0
RP11-252P19.2	0	0	0	0	0	0
RP5-1180C18.1	0	0	0	0	0	0
RP11-458I7.1	0	0	0	0	0	0
RP11-533O20.2	0	0	0	0	0	0
AC016738.4	6.83703079	2.03146794	2.005868661	1.00570022	1.007606311	0.906060876
RP11-421N10.1	0	0	0	0	0	0
ROR1-AS1	0	1.01573397	0	0	0	0
AL133244.1	0	0	0	0	0	0
C1QTNF5	15.6274989	20.3146794	0	0	0	0
MTND6P1	0	0	0	0	0	0
RP4-710M16.2	0	0	0	0	0	0.906060876
RP11-552J9.12	0	0	0	0	0	0
AFG3L1P	595.798397	554.590749	423.2382876	430.439692	1337.093575	623.3698828
AC009948.5	112.322649	140.171288	33.09683291	13.0741028	24.18255147	24.46364366
UBBP3	0	0	0	0	0	0
PRKRIRP8	0	0	0	0	0	2.718182629
ZNF587P1	0	0	0	0	0	0
RP11-141J10.2	0	0	0	0	0	0
RP11-505P4.5	0	0	0	0	0	0
AC098614.1	0	0	0	0	0	0
AC002456.2	0.97671868	1.01573397	0	0	0	0
RP11-598O8.1	0	0	0	0	0	0
DDX11L1	0	0	5.817019118	0	0	4.330970988
AC068491.3	0	0	0	0	0	0

BNIP3P42	0	0	0	0	0	0
AP001048.4	0	0	0	0	0	0
EXTL2P1	0	0	0	0	0	0
AC138623.1	0	0	0	0	0	0
ZNF736P1Y	0	0	0	0	0	0
SMCR2	0	0	1.002934331	0	0	4.530304381
SNRPD2P2	0	0	0	0	0	0
HNRNPRP1	0	0	0	0	1.007606311	1.812121752
AC093326.1	0	0	0	0	0	0
RP11-528A4.3	0	0	0	0	0	0
RP11-445P17.5	0	0	0	0	0	0
RP11-84A14.5	0	0	0	0	0	0
AC104809.2	0	0	0	0	0	1.812121752
RP11-475D12.1	0	0	0	0	0	0
RPL32P35	0	0	0	0	0	0
TRDD1	0	0	0	0	0	0
LL22NC03-102D1.18	0	0	0	0	0	0
RP3-471M13.2	3.90687474	0	0	0	0	0
RP11-270F18.2	0	0	0	0	0	0
YES1P1	0	0	14.04108063	1.00570022	1.007606311	4.530304381
ATP5C1P1	2.93992324	1.01573397	0	2.02145743	2.015212623	0
AC019070.1	0	0	0	0	0	0
LINC01441	0	0	0	0	35.2662209	9.966669638
AC111155.1	0	0	0	0	1.007606311	0
RP11-756G12.1	0	0	0	0	0	0
RP11-488L18.6	0	0	2.005868661	0	0	2.718182629
AC063976.3	0	0	0	0	0	0
RP11-728K20.1	0	0	1.343932003	1.4079803	0	2.373879496
LINC01449	1.95343737	0	0	0	0	0
AP000470.2	0	0	0	0	0	0
RPL21P32	0	0	3.008802992	2.01140043	0	3.624243505
MIR181A2HG	7.81374947	13.2045416	0	0	0	0
FLJ37035	8.42908224	24.3776153	39.1144389	26.1482056	12.09127574	27.18182629

RP11-274B18.3	0	0	0	0	0	0
RP1-231P7P.1	0	0	0	0	0	0
AC073928.2	0	0	0	0	0	0
RP11-136K14.2	0	0	0	0	0	0
TCEAL3-AS1	0	0	0	0	0	0
EPB41L4A-AS1	304.736229	279.326842	597.7488611	320.818369	404.0501308	381.4516289
CDY8P	0	0	0	0	0	0
RP11-445P17.8	0	4.06293589	0	0	0	0
SFPQP1	0	0	0	0	0	0
RP11-347J14.8	0	0	0	0	0	0
AC091493.1	0	0	0	0	0	0
HMGN1P4	0	0	0	0	0	0.906060876
IGKV3D-15	0	0	0	0	0	0.906060876
MTND4P6	0	0	0	0	0	0
CCNT2-AS1	16.6042176	16.2517436	9.026408976	16.0912034	37.28143352	8.154547886
RP11-98L5.4	0	0	0	0	0	0
AC005076.5	3.90687474	4.06293589	4.011737323	1.00570022	14.10648836	6.342426133
AC104777.4	0	0	0	0	0	0
RP11-30O15.1	0	0	0	0	0	0
RP1-90G24.6	0	0	0	1.00570022	1.007606311	0
CPTP	732.539013	833.917591	1229.597489	1231.98276	634.7919761	1521.276211
RP4-551E13.2	0	0	0	0	0	0
GAPDHP55	0	0	0	0	0	0
EGFR-AS1	0	0	0	0	0	0
AC006509.7	0	0	0	0	0	0
HSPA8P16	0	0	0	0	0	0
ARSEP1	0	0	0	0	0	0
AC092106.1	0	0	0	0	0	0
TUBB4BP5	0	0	0	0	0	0
AC007319.1	3.90687474	0	0	0	1.007606311	1.812121752
RP11-344H11.5	0	0	0	0	0	0
SRIP2	0	0	0	0	0	0
RP4-622L5.7	4.88359342	7.11013781	25.07335827	23.131105	2.015212623	9.060608762

RP11-430K21.2	0	0	0	0	1.007606311	0
CTD-2183H9.7	0	0	0	0	0	0
HMGN1P6	0	0	0	0	0	0
AC009474.1	0	0	0	0	0	0
RP11-75A9.3	0	0	1.002934331	0	0	0
LINC00691	0	0	0	3.01710065	0	0
TTTY22	0	0	0	0	0	0
AC009487.4	0	0	0	0	0	0
AP000936.4	0	0	0	0	0	0
SNHG14	0	0	885.7414542	773.836031	0	1227.621881
AC091729.7	0	0	0	0	0	0
UBE2FP1	10.7439055	5.07866986	2.005868661	2.01140043	6.045637868	1.812121752
LINC01057	2.93015605	1.01573397	3.008802992	4.02280086	0	5.436365257
UBTFL8	0	0	0	0	0	0
RP11-39K24.4	0.97671868	0	5.014671654	1.00570022	1.007606311	0.906060876
RPL23AP41	0	0	0	0	0	0
LL22NC03-86G7.1	61.5332771	70.0856441	44.12911055	61.3477131	45.34228401	41.6788003
AC018804.3	0	0	0	0	2.015212623	0
CT47A10	0	0	0	0	0	0
AC097468.4	0	0	9.026408976	2.01140043	0	5.436365257
AC104389.16	0	0	0	0	0	0
RP11-443B9.1	0	0	0	0	0	0
RP5-1033H22.2	0	3.04720192	5.014671654	5.02850108	0	0
RPS24P8	16.3893395	13.0115522	9.618140231	17.3684427	22.37893617	12.31336731
AC108051.1	0	0	0	0	0	0
RP11-472B18.1	0	0	10.02934331	3.01710065	1.007606311	8.154547886
AC064834.1	0	0	0	0	0	0
AP001630.5	0	0	0	0	0	0.906060876
ELMO1-AS1	0	0	0	0	0	0
AC079809.2	0	0	0	0	0	0
CYP4F25P	0	0	0	0	0	0.670485048
LINC00633	0	0	0	0	0	0
CENPVP3	0	0	0	0	0	0

MTRF1LP2	0	0	0	0	0	0
RP11-343H5.4	0	0	1.002934331	0	0	0.906060876
INHBA-AS1	0	0	0	0	1.007606311	0.906060876
PTPN2P2	0	0	0	0	0	0
ATG12P2	0	0	0	0	0	0
POU6F2-AS1	0	0	0	0	0	0
POM121L10P	0	0	0	0	0	0
UBE2SP2	12.9610569	7.67894883	3.219419202	8.64902185	1.108366942	6.007183609
RP11-510C10.2	0	0	0	0	0	0
LINC01248	0	0	0	0	0	0
DPPA2P2	0	0	0	0	0	0
AC112715.2	0	0	0	0	0	1.812121752
AC004866.3	0	0	0	0	0	0
AC007568.1	0	0	0	0	0	0
AC079767.4	0	0	0	0	0	0
AC000123.4	0	0	0	0	0	0
MIR548XHG	0	0	0	0	0	0
AMMECR1-IT1	0	0	0	0	0	0
ASH2LP1	0	0	0	0	0	0
RP11-510C10.3	0	0	0	0	0	0
USP9YP28	0	0	0	0	0	0
AC009506.1	10.7439055	6.09440383	196.5751288	289.641662	103.7834501	340.6788894
RP11-433C9.2	0	0	0	0	0	0
RP11-700P18.2	0	2.03146794	1.012963674	0	0	0.906060876
HCG14	0.97671868	1.01573397	0	1.00570022	0	0
HMGB1P9	0	0	0	0	0	0
CICP10	0	0	0	0	0	0
RPS26P54	0	0	0	0	0	0
UQCRHP3	0	0	0	0	0	0
RP11-309L24.6	2.72504513	0	0	0	0	0
RP3-369A17.4	0	0	0	0	0	0
DNAJC27-AS1	1.95343737	1.01573397	8.023474646	10.0570022	14.10648836	9.060608762
PRYP1	0	0	0	0	0	0

RP3-522D1.1	132.814207	127.982481	0	0	2.015212623	3.624243505
HSFY6P	0	0	0	0	0	0
RP11-416N13.1	0	0	0	0	0	0
AC007422.1	0	0	0	0	0	0
RP4-798A10.4	0	0	0	0	0	0
LINC00570	0	0	0	0	1.007606311	0
SDHDP6	1.96320455	0	0	2.01140043	0	0
MIR3681HG	27.3481231	17.2674775	0	0	2.015212623	1.812121752
SNX18P9	0	3.8090024	0	7.03990151	0	1.812121752
C5orf66	27.3481231	29.4562852	26.0762926	23.131105	15.11409467	20.83940015
RP11-132N15.3	0	0	0	0	0	0
UBE2D3P4	0	0	0	0	0	0
HAGLR	632.913707	580.999832	58.17019118	25.1425054	34.25861458	22.6515219
RP11-442O18.2	0	0	0	0	0	0
RP11-259P1.1	0	0	0	0	0	0
AC008278.3	0	0	0	0	0	0
RP11-574K11.5	0	0	0	0	0	0
OR5AK4P	0	0	0	0	0	0
RPS23P10	0	0	0	0	0	0
PHEX-AS1	0	0	0	0	0	0
AP000351.4	0	0	0	0	0	0
RP11-10L12.1	0	0	0	0	0	0
AL590762.6	0	0	2.005868661	0	0	0
LINC00466	0.97671868	2.03146794	0	0	1.007606311	0
TRIM60P5Y	0	0	0	0	0	0
RP11-371A19.2	0	0	0	0	0	0
RP13-228J13.1	3.90687474	1.01573397	3.008802992	3.01710065	0	9.078729979
RP11-232D9.1	0	0	1.012963674	0	0	0
RP11-359D14.3	0	0	0	0	0	0
AC074290.1	0	0	0	0	0	0
AC104699.1	0	0	0	0	0	0
RPS6P8	0	0	0	0	3.022818934	1.812121752
RP11-262I2.2	0.97671868	2.03146794	0	0	0	0

VSTM2A-OT1	0	0	0	0	0	0
HAUS1P2	0	0	0	0	0	0
OR2L1P	0	0	0	0	0	0
RP1-15D23.2	0	0	0	0	0	0
AC069394.1	0	0	0	0	0	0
AC110299.5	0	1.01573397	1.002934331	1.00570022	1.007606311	0.906060876
OR2J4P	0	0	0	0	0	0.906060876
RP5-1027O11.1	0	0	0	0	0	0
MRRFP1	0	0	0	0	0	0
MINOS1P3	0	0	0	0	0	0
WARS2-IT1	0	0	0	0	0	0
AC090044.2	0	0	0	0	0	0
CYCSP49	0	0	0	0	0	0
LINC00403	0	0	0	0	0	0
AC090283.3	0	0	0	0	0	0
RP11-533K9.3	0	0	0	0	0	0
AJ009632.3	0	0	0	0	0	0
RP11-78A18.2	0	0	0	0	0	0
RP11-499O7.7	0.97671868	0	0	0	0	0
AC113618.2	0	0	0	0	0	0
MTCO1P39	0	0	0	0	0	0
PDCL3P6	0.97671868	0	1.002934331	0	0	0
CTA-292E10.7	0	0	0	0	0	0
VWC2L-IT1	0	0	0	0	0	0
LINC01133	0	0	92.26995843	12.0684026	1.007606311	19.0272784
RP1-272L16.1	0	0	0	0	0	0
RPSAP18	2.5980717	1.04620599	6.027635328	2.47402253	0	2.754425064
CYCSP12	0	0	0	0	0	0
RP11-168L22.2	0	0	0	0	0	0
RP11-149F8.5	0	0	0	0	0	0
AP000697.6	0	0	0	0	0	0
RP11-191L9.4	0	0	53.15551953	35.1995075	19.14451991	57.98789608
AC114730.3	0	0	0	0	0	0

AC005077.7	0	0	0	0	0	0
ENSAP1	0	0	0	0	0	0
RP11-336K24.5	0	0	0	0	0	0
AP000345.2	0	0	0	0	0	0
RP11-405P11.1	0	0	0	0	0	0
RP1-199H16.6	0	0	0	0	0	0
AC005014.5	0	0	0	0	0	0.906060876
SLC25A5-AS1	20.5110924	12.1888077	51.14965087	81.4617175	67.50962286	39.86667855
CTD-2290C23.1	0	0	0	0	0	0
LINC01142	0	0	0	0	0	0
MSL3P1	373.106537	354.044233	0	0	329.9205345	50.73940907
MTCYBP11	0	0	0	0	0	0
IFIT6P	0	0	0	0	0	0
AC012671.2	0	0	0	0	0	0
AF196972.9	0.97671868	7.11013781	1.002934331	2.01140043	0	3.624243505
RP3-326I13.1	0	0	0	0	0	0
OLFM5P	0	0	1.002934331	0	0	0
MRPL49P1	0	0	0	0	0	0
RP11-197P3.1	0	0	0	0	0	0
MTATP6P16	0	0	0	0	0	0
OR51P1P	0	0	0	0	0	0
RP11-144G16.1	0	0	0	0	0	0
RP11-302K17.3	0	0	0	0	0	0
RP11-344B5.2	0	0	0	0	0	0
LINC01527	0	0	1.002934331	0	0	0
ANKRD30BP2	0	0	0	0	0	0
LINC01567	0	0	0	0	0	0
RP11-40H20.4	0	0	0	0	0	0
MCCD1P2	0	0	0	0	0	0
RP3-378P9.2	0	0	0	0	0	0
RPL7P7	0	1.01573397	1.002934331	5.02850108	3.022818934	0.906060876
GTF2IP5	0	1.01573397	0	0	0	0
CHL1-AS2	0	0	0	0	0	0

RPL12P14	0	2.03146794	0	0	2.015212623	1.812121752
AC004009.3	0	0	0	0	0	0
DPRXP1	0	0	0	0	0	0
THAP5P1	0.97671868	0	0	0	0	0
RP5-933B4.1	0	0	0	0	0	0
MDC1-AS1	1.95343737	4.06293589	0	0	9.068456802	1.812121752
LINC00297	0	0	0	0	0	0
AC005019.3	0	0	0	0	0	0
AC019181.3	0	0	0	2.01140043	3.022818934	7.248487009
GAPDHP20	0	0	0	0	0	0
AP000357.4	0	0	0	0	1.007606311	0
RP11-301M17.2	0	0	0	0	0	0
FAM197Y1	0	0	0	0	0	0
FAM8A3P	0	0	0	0	0	0
RP11-149P14.1	0	0	0	0	0	0
AL121578.5	0	0	0	0	0	0
RP11-496H15.2	0	0	0	0	0	0
RP4-562J12.1	0	0	0	0	0	0
AC007879.1	0	0	0	0	0	0
KNOP1P3	0	0	0	0	0	0
BICD1P1	0	0	0	0	0	0
SCEL-AS1	0	0	0	0	0	0
RP11-306K13.1	0	0	0	0	0	0
RP11-462G2.2	0	2.03146794	0	0	0	0
CDKN2AIPNLP3	0	0	0	0	0	0
AC132479.4	0	0	0	0	0	0
ACE3P	0	0	0	0	0	0
MTND2P5	0	0	0	0	0	0
RP11-151A6.4	0.97671868	0	3.008802992	0	1.007606311	8.154547886
ATG3P1	0	0	0	0	0	0
RP11-466F5.8	14.6507803	2.03146794	30.08802992	21.1197045	8.06085049	24.46364366
RP11-467I20.2	0	0	0	0	0	0
AC011239.1	0	0	0	0	0	0

RP11-289H16.1	0	2.97610054	0	0	0	0
RP11-252P18.1	0	2.54949227	0	0	0	0
AC138472.4	0	0	0	0	0	0
OACYLP	0	0	0	2.01140043	0	0
ARAFP2	0	0	0	0	0	0
RP11-814E24.3	0	1.01573397	0	0	0	0
RP11-235G24.1	0	0	0	0	0	0
HLA-N	0	0	0	0	0	0
IGHV4-59	0	0	0	0	0	0
RP1-32B1.4	0	0	0	0	0	0
AC009276.4	0	0	0	0	0	0
AC017104.6	6.83703079	14.2202756	0	0	3.022818934	0.906060876
LINC00703	0	0	1.002934331	0	0	0
PRR29	8.79046815	2.03146794	31.09096425	12.0684026	11.08366942	63.42426133
RP3-417O22.3	0	0	0	0	0	0
RP3-395M20.2	0	0	0	0	0	0
BACE2-IT1	0.97671868	1.01573397	0	0	0	0
C4B	76.3403323	22.8032277	53.09534347	60.0805309	20.19243048	54.20056161
LINC01280	0	0	0	0	0	0
GPC6-AS2	0	0	0	0	0	0
METTL15P3	0	0	0	1.00570022	0	0
LINC01272	3.90687474	5.07866986	0	0	0	0
AC010880.1	0	0	0	0	0	0
RPL7P57	0	2.03146794	0	0	0	0.906060876
OR6D1P	0	0	0	0	0	0
DPPA2P3	0	0	0	0	0	0
LL22NC03-13G6.2	0	0	0	0	0	0
LINC00572	0	0	0	1.00570022	0	0
RP11-48F14.1	0	0	0	0	0	0
RP5-956O18.3	4.88359342	6.09440383	1.002934331	0	0	0
USP9YP22	0	0	0	0	0	0
RP11-114B7.6	0	0	0	0	3.022818934	0
AC097499.2	0	0	0	0	0	0

HSP90AA2P	60.9081771	80.039837	52.55375893	48.6758904	99.64218812	63.82292812
RP11-351O1.3	0	0	0	0	0	0
AP001476.2	0	0	0	0	0	0
AC010886.2	0	0	0	0	0	0
RP11-401L13.4	0	1.01573397	1.002934331	1.00570022	2.015212623	0.906060876
IFNA22P	0	0	0	0	0	0
RP11-503C24.6	0	0	0	0	0	0
STK24-AS1	3.90687474	5.07866986	2.005868661	2.01140043	15.11409467	3.624243505
KRT18P27	0	0	0	0	0	0
ADM5	74.6994449	95.5907241	70.20540315	49.2692536	94.57392838	67.0122624
ATP5J2LP	0	0	0	0	0	0
PRKAR2A-AS1	43.9523408	67.0384422	119.3491854	71.4047153	38.28903983	69.76668747
AC073869.2	0	0	0	0	14.88234522	0
SLC31A1P1	0	0	0	0	0	0
AP000281.1	0	0	0	0	0	0
LINC00539	0	0	1.002934331	1.00570022	2.015212623	0.906060876
MKRN5P	0	1.01573397	6.017605984	1.00570022	0	3.624243505
AC063976.7	0	0	0	0	0	0
NF1P6	0	0	0	0	0	0
RP11-284L19.1	0	0	0	0	0	0.906060876
PIGUP1	0	2.03146794	2.025927348	1.00570022	0	1.812121752
RPL23AP14	0	0	0	0	0	0
RPSAP10	0	0	0	0	0	0
CXorf51A	0	0	0	0	0	0
AC068831.3	0.97671868	0	2.005868661	0	0	0
AC017035.2	0.97671868	0	0	0	0	0
AC006509.4	0	0	0	0	0	0
RP11-413P11.1	0	0	2.005868661	0	0	0
HIST1H1PS2	0	0	0	0	0	0
GS1-259H13.7	0	0	0	0	0	0
ATP5F1P1	0	0	1.002934331	0	0	1.812121752
RSL24D1P6	0	1.01573397	1.002934331	1.00570022	0	0.906060876
GUSBP6	0	0	0	0	0	0

RP11-169K16.4	0	1.01573397	0	0	0	0
RP11-439L18.2	0	0	0	0	0	0
C4BPAP1	0	0	4.011737323	2.01140043	0	0
AC079354.6	0	0	0	0	0	0
PGAM1P6	0.97671868	0	56.16432252	21.1197045	0	36.24243505
SOCS2P2	0	0	0	0	0	0
CDCA4P2	0	0	0	0	0	0
AC009313.1	0	0	0	0	0	0
RP11-181K3.4	0	0	0	0	0	0
AC009517.2	0	0	0	0	0	0
ATXN1L	1188.66664	1203.64476	1058.095719	1013.74582	3687.839099	1681.648986
MTCO3P19	0	0	0	0	0	0
CCND3P1	0	0	0	0	0	0
AC073900.4	0	0	0	0	0	0
RPL36P16	0	0	0	0	0	0
RP1-81D8.3	0	0	0	0	0	0
RP11-13P5.1	0	0	0	0	0	0
AC136289.1	3.90687474	9.14160575	2.005868661	0	7.053244179	1.812121752
RP11-495P10.3	0	0	0	0	0	0
HSFY4P	0	0	0	0	0	0
RP11-548K12.4	0	0	0	0	0	0
USP9YP7	0	0	0	0	0	0
HCG19P	0	0	0	0	0	0
SAMM50P1	0	0	0	0	0	0
TTC21B-AS1	0	0	0	0	0	0
RP11-87O11.1	0	0	0	0	0	0
HNRNPA3P14	0	0	0	0	0	0
RPL36P4	0	0	0	0	0	0
MAPKAPK5P1	0	0	0	0	0	0
RP11-328K22.1	0	0	0	0	0	0
RP11-475D12.2	0	0	0	0	0	0
AC002117.1	1.95343737	0	7.020540315	4.02280086	4.030425245	6.342426133
RP1-293L8.2	0	0	0	0	1.007606311	0.906060876

AC010884.1	0	0	0	0	0	0
POLR2KP2	0	0	0	0	0	0
LINC00365	0	0	134.3932003	5.02850108	16.12170098	38.96061768
AC109309.4	0	0	0	0	0	0
LINC00620	0	0	0	0	0	0
RP11-5K23.5	0	0	0	0	0	0
AC068134.8	0	0	0	0	0	0
HTR2A-AS1	0	0	0	0	0	0
AC006989.2	0	0	0	0	0	0
HMGN1P19	0	0	0	0	0	0
KRT8P45	0.97671868	1.01573397	5.024700997	6.03420129	47.73031097	5.445425866
RP11-438F14.3	0	0	0	0	0	0
RP13-52K8.1	0	0	0	0	0	0
CYCSP42	0	0	0	0	0	0
RP11-561I11.3	0	0	53.15551953	27.1539058	0	16.30909577
AC093698.4	0	0	0	0	0	0
PPIAP10	0	0	0	0	0	0
SMIM13	2438.86655	2253.91368	375.0974397	314.784167	638.8224014	380.545568
RP3-470L22.1	0	0	0	0	0	0
TMLHE-AS1	0	5.07866986	51.14965087	38.2166082	17.12930729	38.94249646
RP11-61J19.2	0	0	0	0	0	0
RP11-134G8.7	14.6507803	10.1573397	287.8421529	119.678326	25.19015778	77.01517448
MEP1AP4	0	0	0	0	0	0
SAGE4P	0	0	0	0	0	0
RP11-385M4.3	0	0	0	0	0	0
AP001439.2	0	0	0	0	0	0
SNRPGP15	12.3261898	6.82573229	6.679542643	4.62622099	1.168823321	8.435426757
AC008264.4	0	0	0	0	0	0
EIF4BP3	9.37649937	21.9906405	4.904348877	20.3352584	37.81546486	7.049153617
ZNF619P1	0	0	0	0	0	0
RP1-107N3.2	0	0	0	0	0	0
RP11-370B11.3	0	0	0	0	0	0
RP11-270C12.3	0.97671868	9.14160575	10.02934331	10.0570022	5.078335809	12.68485227

HMGB3P21	0	0	0	0	0	0
AC008065.1	0	0	0	0	0	0
RP11-438N5.2	0	0	0	0	0	0
RP11-485B17.3	0	0	0	0	0	0
HLA-DPB2	0	0	0	0	0	0
LINC01087	0	0	122.3579883	154.877833	675.0962286	271.8182629
RP11-270L13.1	0	0	0	0	0	0
LPP-AS1	0	0	0	0	0	0
RP1-148H17.1	0	0	0	0	3.022818934	0
FAM96AP2	0	2.03146794	0	5.02850108	2.015212623	1.812121752
RP11-295P22.4	0	0	0	0	0	0
AC096669.3	0	0	0	0	0	0
RP11-341G5.2	0	0	0	0	0	0
RP11-430H12.2	0	0	0	0	0	0
USP9YP13	0	0	0	0	0	0
AC083863.5	0	0	0	0	0	0
COL18A1-AS2	0	0	0	0	0	0
LINC01117	4.88359342	10.1573397	0	0	6.045637868	3.624243505
HNRNPA1P48	218.423599	206.285412	123.4511868	185.199695	230.4496395	152.5172273
AC012314.19	0	0	0	0	0	0
LINC01015	0	0	0	0	0	0
RP11-554D15.4	0	0	1.002934331	0	0	0
RP11-365D23.2	0	0	0	0	0	0
AC103563.5	0	0	0	0	0	0
GPX5	0	2.03146794	0	0	0	0
RP11-12D5.6	0	0	0	0	0	0
MTND3P16	0	0	0	0	0	0
RP5-884C9.2	0	0	0	0	0	0
RP11-30B1.1	0	2.03146794	1.002934331	2.01140043	7.053244179	0.906060876
RPL29P19	0	0	0	0	0	0
AC073626.2	0	0	0	0	0	0
ZMIZ1-AS1	64.4634331	95.4789934	10.02934331	7.03990151	5.038031556	3.624243505
SVIL-AS1	708.121046	688.667633	351.0270157	479.719003	470.5521474	437.6274032

RPS5P2	0	0	0	0	0	0
BMS1P12	9.69881653	5.10914188	0	0	0	3.80545568
RP4-612B18.1	0	0	0	0	0	0
RPS26P5	0	0	0	0	0	0
RP11-96J15.1	0	0	0	0	0	0
AC008069.2	0	0	0	0	0	0
RP1-177I10.1	0	0	0	0	0	0
TGFA-IT1	0.97671868	0	0	0	0	0
IGKV1D-27	0	0	0	0	0	0
HSD52	0	0	0	0	0	0
RP11-265P11.1	0	0	0	0	0	0
AC007919.18	0	0	0	0	0	0
AC011901.2	0	0	0	0	0	0
RP11-153F1.1	0	0	0	0	0	0
TNK2-AS1	1.95343737	1.01573397	16.04694929	101.575722	5.038031556	54.36365257
RTCA-AS1	0.97671868	1.01573397	11.03227764	6.03420129	1.007606311	6.342426133
RP11-402P6.5	0	0	0	0	0	0
AC096921.2	0	0	0	0	0	0
MEF2AP1	0	0	0	0	0	0
RP11-276H7.3	4.88359342	4.06293589	0	0	0	1.812121752
OR9R1P	0	0	0	0	0	0
TUBB8P6	0	0	0	0	0	0
AC106053.1	0	0	0	0	0	0
RPL37P10	0	0	0	0	0	0
RP5-854E16.2	1.95343737	0	0	0	0	0.915121485
RP5-1142J19.2	0	0	0	0	0	0
RP1-169P22.2	0	0	0	0	0	0
RP11-51O6.1	66.7391877	75.4995062	65.36123033	52.0751572	93.79807152	56.8734412
LL0XNC01-73E8.1	0	0	0	0	0	0
ZNF736P6Y	0	0	0	0	0	0
RP4-564F22.5	40.045466	33.5192211	12.03521197	13.0741028	16.12170098	22.6515219
PDSS1P2	0	0	0	0	0	0
AC106900.6	0	0	0	0	0	0

SRSF1P1	0	0	0	0	0	0
AC064871.3	0	0	0	0	0	0
RP11-16L21.7	0	0	0	0	0	0
RP11-126K1.8	0	0	0	0	0	0
AC007387.2	0	0	0	0	0	0
AC026954.6	0	0	0	0	0	0
RP11-397D12.4	0	0	0	0	0	0
AF124730.4	0	0	0	0	0	0
IGHV3-74	0	0	0	0	0	0
LINC00885	0	3.04720192	1.002934331	4.02280086	15.11409467	17.21515665
RP11-548K12.10	0	0	0	0	0	0
AC018737.3	0	0	0	0	0	0
RP1-212G6.4	0	0	0	0	0	0
RBMY2BP	0	0	0	0	0	0
RP11-631F7.1	0	0	0	0	0	0
GAGE12J	0	0	0	0	0	0
SH3BP5-AS1	105.485618	78.2115159	869.5440647	449.547996	242.833121	724.8487009
AC010907.5	0	0	0	0	0	0
ATP6V1G1P3	0	0	0	0	0	0
RPL36AP53	0	0	0	0	0	0
RP1-50J22.4	0	0	0	0	0	0
AC013470.8	0	0	0	0	0	0
IPO8P1	3.90687474	3.04720192	0	0	1.007606311	0.906060876
RP11-561N12.1	0	2.03146794	2.005868661	1.00570022	0	0.906060876
AC068135.1	0	0	0	0	0	0
RP11-335O13.8	0	0	0	0	0	0
AC007272.3	0.97671868	4.06293589	3.008802992	0	3.022818934	7.248487009
EIF3EP2	0	0	0	0	0	0
AC009227.2	0	0	0	0	0	0
PDIA3P2	0	0	0	0	0	0
GAPDHP46	0	0	0	0	0	0
MED15P4	0	0	0	0	1.007606311	0
PLA2G12AP1	0.97671868	1.01573397	0	5.02850108	2.055516875	1.812121752

RP11-146D12.5	0	0	0	0	0	0
SOCS5P2	0	0	0	0	0	0
RPL36AP29	0	0	0	0	0	0
RP11-84O12.3	0	0	0	0	0	0
TCEB1P23	0	0	0	0	0	0
RASAL2-AS1	11.7206242	22.3461474	6.017605984	7.03990151	6.045637868	6.342426133
E2F6P2	0	2.9151565	0	1.01575722	0	0
ZNF812	0	0	0	0	0	0.915121485
UBE2D3P3	0	0	0	0	0	0
GS1-174L6.4	0	0	0	0	0	0
LL0XNC01-177E8.2	0	0	0	0	0	0
MTND6P7	0	0	0	0	0	0
NEFMP1	0	0	0	0	0	0
RP11-131J3.1	0	0	0.501467165	0	25.69396094	5.436365257
LAMTOR5-AS1	25.3946858	13.2045416	57.16725685	36.2052078	49.37270925	52.55153082
RP11-697E14.2	0	0	0	0	0	0
MED28P4	0	0	0	0	0	0
RP11-38C18.2	0	0	0	0	0	0
RPS17P13	0	0	0	0	0	0
E2F3-IT1	0	3.04720192	0	0	0	0
OR11M1P	0	0	0	0	0	0
FAM90A8P	0	0	0	0	0	0
RP5-988G17.1	0	0	0	0	0	0
NPIPA3	11.3592383	9.24317915	28.16239601	24.4083442	18.59033644	35.74410157
AC025165.8	0	1.01573397	1.002934331	1.00570022	0	0.906060876
RP11-179K3.2	0	0	0	0	0	0.906060876
CITF22-49D8.1	0	0	0	0	0	0
RP11-576D8.4	0	0	0	0	0	0
RP11-222A5.1	0	0	0	0	0	0
AC009237.6	0	0	0	0	0	0
AC007182.6	0	0	0	0	2.015212623	0
RP11-255B23.1	4.0045466	5.21071528	3.079008395	5.08884309	5.158944314	5.590395606
GUSBP10	0	0	0	0	0	0

CEP57L1P1	0.97671868	1.02589131	0	2.01140043	1.007606311	0.906060876
FCF1P7	0	0	0	0	0	0
AC090945.1	10.7439055	12.1888077	2.005868661	5.02850108	26.19776409	9.060608762
PCOLCE-AS1	0.97671868	5.07866986	9.026408976	8.04560172	1.007606311	6.342426133
AC009892.10	0	0	0	0	0	0
AC016716.2	0	0	0	0	0	0
MAGEA7P	0	0	0	0	0	0
RP1-86D1.5	0	0	0	0	0	0
RP11-272G22.1	0	0	0	0	0	0
AC099850.1	42.9756221	42.6608268	10.02934331	7.03990151	1039.849713	57.98789608
AC016735.1	0	0	0	0	0	0
TEX26-AS1	0.97671868	0	0	0	0	0
RP11-380G5.2	0	0	1.002934331	0	0	4.530304381
AC015987.1	1.95343737	3.04720192	3.008802992	10.0570022	2.015212623	0
MTCYBP21	0	0	0	0	0	0
RP11-94M14.2	0	0	0	0	0	0
SHMT1P1	0	0	0	0	0	0
VN1R21P	0	0	0	0	0	0
HMGN1P34	0	0	0	0	0	0
LINC01167	0.97671868	0	0	0	5.038031556	0
C1DP3	0	0	0	0	0	0
RP11-508N22.9	0	0	0	0	0	0
CR769776.3	0	0	0	0	0	0
FDPSP8	1.95343737	1.01573397	2.005868661	1.00570022	0	2.718182629
RP11-54O15.3	0	0	0	0	0	0
RP1-137H15.2	0	0	0	0	0	0
MUC20P1	1.95343737	0	109.1994899	50.4258088	42.01718318	148.4852564
ATP2B2-IT2	0	0	0	0	0	0
HSPA8P7	0.97671868	1.20872343	0	1.00570022	1.007606311	0.906060876
BRAFP1	0	0	1.002934331	1.00570022	0	0
RPSAP50	0	0	0	0	0	0
OR4F2P	0	0	0	0	0	0
CENPIP1	0	0	0	0	0	0.906060876

EIF4A2P4	1.95343737	5.07866986	2.005868661	5.02850108	2.015212623	2.718182629
MIPEPP2	0	0	0	0	0	0
AL591704.7	0	0	0	0	0	0
AC006026.9	0	0	0	0	1.007606311	0
CETN4P	0	0	0	0	0	0
RP5-933E2.1	0	0	0	0	0	0
AC012363.4	0	4.06293589	1.002934331	2.01140043	0	0
AP000704.5	6.83703079	6.09440383	3.008802992	4.02280086	4.030425245	4.530304381
KRT18P39	0	0	0	0	0	0
IQCF4	0	0	0	0	0	0
RP3-333H23.8	0	1.01573397	1.002934331	2.01140043	12.09127574	0
NTM-AS1	0	0	0	0	0	0
RPL32P1	0	0	0	0	0	0
RP11-57C19.6	0	0	0	0	0	0
RP11-445O16.3	0	0	0	0	0	0
RP11-235D19.2	0	0	0	0	0	0.906060876
TUBB4BP2	0	2.03146794	2.005868661	2.01140043	1.007606311	0.906060876
LINC00853	1.95343737	2.03146794	0	0	7.053244179	1.812121752
ARL5AP4	0	0	0	0	0	0
DUX4L9	0.97671868	0	0	0	0	0
SRGAP3-AS1	0	0	0	0	0	0
BEND3P2	0	0	0	0	0	0
RP11-538D16.2	0	0	2.005868661	0	0	1.812121752
TMEM72-AS1	1.95343737	0	1.002934331	0	0	0
SEPT14P13	0	0	0	0	0	0
AC114812.8	0	0	1.002934331	0	0	0
RP11-307E17.9	0	0	0	0	0	0
NAP1L4P2	0	0	0	0	0	0
RP11-190J1.3	0	0	0	0	0	0
RP11-134G8.10	0	0	0	0	0	0
AC093843.1	0	0	0	0	0	0
BTF3P4	0	0	0	0	0	0
COL4A2-AS2	0	0	0	0	0	0

THRB-IT1	0	0	17.04988362	2.01140043	1.007606311	17.21515665
RORB-AS1	0	0	0	0	0	0
AC019109.1	0	0	0	0	0	0
LINC00265-2P	0	0	0	0	0	0
TOMM20P1	0	0	0	0	0	0
OR2X1P	0	0	0	0	0	0
RP11-651P23.4	6.12402615	19.8575992	53.65698669	83.0507238	48.55654814	68.02705058
AP000469.2	0	0	0	0	0	0
BTF3P9	0	0	0	0	0	0
RP11-341G2.3	0	0	0	0	0	0
GCSHP5	1.01578743	2.23461474	0	1.08615623	5.300009197	1.984273319
RPL12P17	0	4.14419461	5.937371238	4.02280086	4.85666242	1.812121752
RP11-123K19.1	0	1.01573397	3.008802992	5.02850108	10.07606311	0.906060876
LINC00240	16.6042176	21.3304134	4.011737323	1.00570022	2.015212623	8.154547886
AC107079.1	0	0	0	0	0	0
RP1-90J20.8	0	0	0	0	0	0
RP11-535M15.1	0	10.1573397	31.09096425	23.131105	1.007606311	18.12121752
AC108051.3	0	0	0	0	0	0
LINC00502	0	0	0	0	0	0
MTND5P24	0	0	0	0	0	0
LINC00393	0	0	0	0	0	0
CDKN2A-AS1	0	0	0	0	0	0
OPA1-AS1	0	0	0	0	0	0
RP4-796I17.5	0	0	1.002934331	0	2.015212623	0
RP11-344P13.1	0	0	0	0	0	0
RPL29P11	11.2811008	11.8739301	15.02395627	11.6158375	8.685566403	3.796395071
GXYLT1P2	0	0	0	0	0	0
YBX1P1	93.4036077	88.0641354	52.13252651	80.0637942	133.2357825	77.54975039
LINC01398	0	0	0	0	0	0
CTC-260E6.2	3.99477942	2.69169503	9.06652635	2.98692964	7.345450009	8.716305629
AC009518.4	0	0	0	0	0	0
USP9YP25	0	0	0	0	0	0
AC062016.1	0	0	0	0	0	0

RP4-758J18.2	227.575453	291.51565	585.7136491	427.422592	419.1642255	595.2819956
XX-FW81066F1.2	0	0	1.002934331	0	1.007606311	0.906060876
CDY13P	0	0	0	0	0	0
AC083949.1	0	2.03146794	0	0	2.015212623	0
RP11-49G10.3	0	0	0	0	0	0
C17orf89	651.471362	621.629191	496.4524937	517.935611	989.4693977	858.0396497
AC011754.1	0	0	0	0	0	0
MTCYBP29	0	0	0	0	0	0
AC068279.3	0	0	0	0	1.007606311	0
CTA-250D10.19	0	0	0	0	0	0
AC034187.2	0	0	0	0	0	0
TSSC1-IT1	2.93015605	3.04720192	3.008802992	1.00570022	5.038031556	1.812121752
RP11-119F19.4	0	8.12587178	12.03521197	15.0855032	10.07606311	14.49697402
RP11-276H19.4	0	0	0	0	0	0
RP5-1142A6.2	2.93015605	1.01573397	1.002934331	7.03990151	12.09127574	10.87273051
AC007899.3	0	0	0	0	0	0
RPS4XP16	2.93015605	5.96235842	53.15551953	22.9299649	2.015212623	10.87273051
RP1-200K18.1	0	0	0	0	0	0
VPS26BP1	0	1.01573397	4.011737323	2.01140043	2.015212623	0
SIGLEC30P	0	0	0	0	0	0
POT1-AS1	3.90687474	3.04720192	2.005868661	2.01140043	3.022818934	1.812121752
RP11-3B12.5	0	0	0	0	13.09888205	3.624243505
RP11-448G4.4	0	0	0	0	0	0
GAGE12H	0	0	0	0	0	0
AC005534.8	2.93015605	2.03146794	4.011737323	0	12.09127574	5.436365257
RP5-934G17.6	0	0	0	0	0	0
AP001347.6	13.6740616	4.06293589	18.05281795	14.029518	7.053244179	43.29158866
RP1-102E24.9	0	0	0	0	0	0
RP11-451M19.2	0	0	0	0	0	0
TIMM8BP2	0.98648587	0	0	1.00570022	2.015212623	0
AC002115.5	0	0	0	0	0	0
AC015936.3	0	0	0	0	0	0
LINC00863	237.127762	266.589538	108.3670544	88.9139561	88.79026815	107.2504259

APOC4-APOC2	0	0	0	0	0	0
AC016694.2	0	0	0	0	0	0
AY269186.2	0	0	0	0	0	0
RP11-144G6.4	0	0	0.501467165	0	0	0.453030438
TACC1P1	0	0	0	3.01710065	0	13.59091314
AL050303.7	0	0	0	0	0	0
LINC00320	0	0	0	0	0	0
NDUFA5P10	0.97671868	0	0	0	0	0
KRT8P30	0.97671868	0	0	0	2.005136559	0.906060876
AC152010.1	0	0	0	0	0	0
AC107399.2	0	0	0	0	0	0
LINC01034	0	0	0	0	0	0
RP11-441O15.3	7.81374947	9.14160575	25.07335827	24.1368052	14.10648836	15.4030349
RP11-74C3.1	0	0	0	0	0	0
SUCLA2P1	0	2.03146794	0	0	1.007606311	0
LINC00184	0.97671868	0	0	1.00570022	1.007606311	1.812121752
PRRT4	1.95343737	4.06293589	7.020540315	6.03420129	25.19015778	11.77879139
RP11-51C14.1	0	0	0	0	0	0
RP11-180D21.3	0	0	0	0	0	0
CASC6	0	0	0	0	0	0
RP11-82L18.2	16.6042176	17.2674775	13.0381463	4.02280086	4.030425245	6.342426133
AC007312.3	0	0	0	0	0	0
VN1R25P	0	0	0	0	0	0
ATP5G1P8	0	0	0	0	0	0
RP11-161N3.3	0	0	0	0	0	0
RP5-1086K13.1	0.97671868	3.04720192	5.014671654	6.03420129	25.19015778	4.530304381
SRIP3	0	0	0	0	0	0
KCTD10P1	0	0	0	0	0	0
LINC01266	0	0	0	0	0	0
PGM5-AS1	0	0	0	0	0	0
AC017002.2	20.5110924	19.2989455	6.017605984	6.03420129	6.045637868	2.718182629
PPP4R3CP	0	0	0	0	0	0
RP1-278O22.1	0	0	0	0	0	0

PSAT1P4	0	0	0	0	0	0
U82695.9	0	0	0	0	0	0
TRAPPC2P3	0	0	0	0	0	0
KCNC4-AS1	0	1.01573397	1.002934331	0	0	2.718182629
TBC1D3P6	0	0	0	0	0	0
AC009303.3	0	0	0	0	0	1.812121752
RP1-35C21.1	0	0	0	0	0	0
RP11-54O7.11	0	0	1.002934331	0	0	1.812121752
RP11-114L10.2	0	0	0	0	0	0
SUMO2P3	0	0	0	0	0	0
RP11-403H13.1	0	0	0	0	0	0
LARGE-AS1	0	0	0	0	0	0
INE1	28.3248418	28.4405512	50.14671654	58.3306125	51.38792188	38.0545568
PARP4P2	0.99625306	0	0	0	0	0.915121485
RP11-160H22.3	0	0	0	0	0	0
RP13-228J13.9	0	0	0	0	0	0
RPL23AP85	0	0	0	0	0	0
AC000367.1	0	0	0	0	0	0
TMEM233	0	0	0	0	0	0
RP11-524H19.2	0	0	0	0	75.57047335	14.49697402
RP11-297K8.2	0.97671868	2.03146794	15.04401496	7.03990151	3.022818934	3.624243505
PPP1R8P1	0	0	3.008802992	0	0	1.812121752
RP1-191J18.65	0	0	0	0	0	0
RP11-409K20.7	0	0	0	0	0	0
FAM41AY1	0	0	0	0	0	0
RP11-295G24.5	0	2.03146794	3.008802992	0	0	0.906060876
RPL29P12	0	0	0	0	0	0
LINC01526	0	0	0	0	0	0
VTA1P1	0.97671868	0	0	0	0	0
AC010082.2	0	0	0	0	0	0
RP11-6F6.1	0	0	0	0	0	0
OR51A5P	0	0	0	0	0	0
RP11-483G21.3	0	0	0	0	0	0

RP11-669M2.1	0	0	0	0	0	0
AC000067.1	0	0	0	0	0	0
RP13-228J13.6	0	0	3.008802992	1.00570022	1.007606311	1.812121752
RP11-810H18.1	0	0	0	0	0	0
RP13-348B13.2	0	0	0	0	0	0
KCTD9P1	0	0	2.005868661	0	0	0
LINC00328-2P	0	0	0	0	0	0
AP004289.1	0	0	0	0	0	0
RP11-419M24.5	0	0	0	0	0	0
UBE2D3P1	0	1.11730737	0	0	2.015212623	0
AC015977.5	0	0	0	0	0	0
KIF4CP	0	0	0	0	0	0
AC091492.2	0	0	0	0	0	0
IFNWP4	0	0	0	0	0	0
RP11-330M19.1	0	0	0	0	0	3.624243505
RP4-784A16.3	0	0	0	0	0	0
EIF4BP7	3.97524504	12.3817971	33.51806533	30.7241416	66.94536332	25.49655306
RP11-228B15.4	36.1385913	45.7080288	12.03521197	36.2052078	10.07606311	29.90000891
RP11-414B7.1	0	0	0	0	0	0
EIF1AX-AS1	0	0	0	0	0	0
LINC01058	0.97671868	0	1.002934331	0	0	0
RPL18AP2	0	0	1.002934331	0	3.022818934	0
RP11-204C23.1	0	0	0	0	0	0
MTND5P27	0	0	0	0	0	0
RP1-20I3.3	0	0	0	0	0	0
RP11-349E4.1	0	0	0	0	0	0
HMGB3P22	0	0	27.07922693	25.1425054	8.06085049	10.87273051
PRPF38AP1	0	0	0	0	0	0
RP11-344N17.9	0	0	0	0	0	0
RP11-218C14.5	0	0	0	0	0	0
AC096574.4	0	1.01573397	7.020540315	1.00570022	3.022818934	0.906060876
RP11-132N15.1	0	0	0	0	0	0
CATIP-AS1	0	1.01573397	3.008802992	0	2.015212623	0.906060876

RP11-383G10.5	0	0	0	0	0	0
AC007879.6	0	0	0	0	0	0
RP1-272E8.1	0	0	0	0	0	0
RPL23AP2	2.06087642	3.27066339	7.61227157	2.23265448	3.284796575	3.053425153
RP5-1025A1.2	0	0	0	0	0	0
GS1-184P14.2	0	1.14777939	1.15337448	1.14649825	1.118443006	0
OR7E116P	0	0	0	0	0	0
RP11-426L16.3	0	0	1.002934331	4.02280086	0	0
WWC3-AS1	0	0	0	0	0	0
LINC00337	10.7439055	26.4090833	1.002934331	3.01710065	13.09888205	0
RP11-123N4.4	0	0	0	0	0	0
FTH1P22	0	0	0	0	0	0
PFN1P4	0	0	0	0	0	0
DAP3P1	0	0	0	0	0	0
GRTP1-AS1	0	0	2.005868661	0	2.015212623	0
AL450226.2	0	0	0	0	0	0
RP11-15B24.1	0	0	0	0	0	0
RP4-660H19.1	0	0	0	0	0	0
SNORA71A	3.90687474	0	0	1.00570022	1.007606311	0.906060876
RP11-405O10.2	3.90687474	1.01573397	12.03521197	11.0627024	3.022818934	14.49697402
RPL3P7	0	0	1.002934331	1.00570022	0	0.906060876
SETP20	0.97671868	1.01573397	3.008802992	5.02850108	6.045637868	4.530304381
XXbac-BPG55C20.7	0	0	0	0	0	0
BCRP1	0	0	0	1.00570022	0	0
ATP6V1E1P1	0	0	0	0	0	1.812121752
DPY19L2P5	0	0	0	0	0	0
OR52K3P	0	0	0	0	0	0.906060876
RP11-456H18.1	0	0	0	0	0	0
LINC01076	0	0	0	0	0	0
RP11-429E11.2	0	0	0	0	0	0
AC092484.1	0	0	0	0	0	0
ZBTB45P1	2.93015605	0	3.008802992	6.03420129	19.14451991	2.718182629
LL0XNC01-16G2.1	0	0	0	0	0	0

AC083900.1	0	0	0	0	0	0
RP11-295P9.6	0	0	0	0	0	0
RP5-1160K1.3	0	0	0	0	0	0
RP11-137F15.1	0	0	0	0	0	0
ARSDP1	0	0	0	0	0	0
RP11-477L16.2	0	0	0	0	0	0
EEF1B2P5	0	0	0	0	0	0
RP11-408E1.1	0	0	0	0	0	0
RP11-255A11.4	0	0	0	0	0	0
RPL23AP36	0	0	0	0	0	0
RANP4	0	0	15.04401496	3.01710065	0	4.530304381
RP4-549F15.1	0	1.01573397	0	0	0	0
LINC00237	0	0	0	0	0	0
LINC00972	0	0	0	0	0	0
RP4-614C15.2	0	0	0	0	0	0
PSME2P2	23.2361375	21.1882107	24.93294746	6.66779243	14.97302979	23.27670391
MORF4L1P4	0	0	0	0	0	0
PGBD4P5	0	0	0	0	0	0
DYNC1I2P1	34.6246773	38.120496	13.19861579	17.2980437	13.28025118	7.375335532
CTD-2228K2.7	146.507803	134.076884	1131.309925	1247.06827	240.8179084	841.730554
RP11-809C18.3	1.95343737	2.03146794	0	2.01140043	0	1.812121752
RP11-490K7.1	0	0	0	0	0	0
AC018643.4	0	0	0	0	0	0
AC073957.15	0	0	0	0	1.007606311	3.624243505
RPS12P10	0	0	0	0	0	0
RP5-899B16.2	0	0	0	0	0	0
GOLGA2P7	1099.32618	1131.45654	333.5458704	175.736056	1407.444648	432.9702503
RP11-338O1.2	0	0	0	0	0	0
RP11-184J23.2	0	0	0	0	0	1.812121752
TOMM22P5	0	0	0	0	0	0
AC012354.6	0	0	1.002934331	0	0	0
HSPE1P24	0	0	0	0	0	0
NPM1P39	11.8378304	12.2395944	0	8.27691277	12.66561133	2.727243237

LINC00618	1.95343737	1.01573397	3.008802992	0	4.030425245	1.812121752
CTD-2538A21.1	0	0	0	0	0	0
AC012462.2	0	0	0	0	0	0
TXNP2	0	0	0	0	0	0
BRI3P1	0	0	0	0	0	0
RP5-828K20.1	0	1.02589131	1.012963674	0	0	0.915121485
DUTP6	1.95343737	1.01573397	11.03227764	2.01140043	0	0
RP11-16L9.2	0	0	0	0	0	0
XXbac-BPG308K3.5	3.90687474	2.03146794	9.026408976	15.0855032	0	16.30909577
OSTM1-AS1	0	0	0	0	0	0.906060876
RP11-795A2.2	0	0	0	0	0	0
ATP5LP4	0	0	0	0	0	0
RP11-390P2.4	12.6973429	10.1573397	10.02934331	21.1197045	22.16733885	12.68485227
RPSAP58	577.758403	603.935105	677.4821404	772.981186	923.269663	717.645517
LINC00457	0	0	0	0	0	0
AATK-AS1	0	0	1.002934331	0	0	0
RP11-388K2.1	0	0	0	0	0	0
AC092601.1	0	0	0	0	0	0
RP4-758J24.4	0	0	0	1.00570022	0	0
RP11-481K9.4	0	0	0	0	0	0
AC073283.7	1.95343737	2.03146794	1.002934331	1.00570022	2.015212623	1.812121752
REREP1Y	0	0	0	0	0	0
PLEKHM1	1369.19355	1744.46215	555.6657366	850.993351	684.900238	829.988005
RP11-97C18.1	0	0	0	0	0	0
ZNF33BP1	0.97671868	0	7.020540315	3.01710065	0	4.530304381
RPS12P26	0	0	2.005868661	4.02280086	3.022818934	7.248487009
LINC00092	0	0	0	0	0	0
RP11-338E21.2	0	0	0	0	0	0
RP5-1118D24.2	0	0	0	0	0	0
SSXP3	0	0	0	0	0	0
AB019441.29	2.93015605	3.25034871	2.005868661	5.03855808	7.063320242	1.812121752
RP11-182M20.2	0	0	0	0	0	0
AC093818.1	96.6951497	77.1957819	1.002934331	4.02280086	4.030425245	3.624243505

MIR137HG	0.97671868	4.06293589	0	0	0	0
RP11-107I14.2	0	0	0	0	0	0
RP11-358M3.1	0	0	0	0	0	0
DUXAP9	1299.50467	1098.17094	265.5970695	207.646924	584.2202153	288.0911162
AKR1B1P6	0	0	0	0	0	0
RP11-197M22.2	0	1.01573397	2.005868661	5.02850108	2.015212623	2.718182629
AC079790.2	0	0	0	0	0	0
SMARCE1P1	0	1.01573397	1.002934331	0	1.007606311	1.812121752
AC007362.3	0	0	0	1.00570022	0	0
HSPA7	0	2.18382804	0	0	2.367874832	1.159757922
AP001628.6	1.95343737	5.07866986	0	0	1.007606311	0.906060876
PLA2G12AP2	0	0	0	0	0	0
CHCHD4P5	0	0	0	0	0	0
RP4-814D15.2	3.90687474	3.04720192	2.005868661	2.01140043	4.030425245	2.718182629
ARL2BPP10	0	0	0	0	0	0
AC007250.4	0	0	0	0	0	0
AC008937.3	0	0	0	0	0	0
AC091814.2	0	0	0	0	0	0
RP11-554K11.2	0	0	0	0	0	0
TRAPPC12-AS1	0	0	1.002934331	3.01710065	0	0
DDX26B-AS1	0	0	2.005868661	1.00570022	1.007606311	2.718182629
AP000936.5	0	0	0	0	0	0
RP1-169I5.4	0	0	0	0	0	0
RPL21P107	0	0	0	0	0	0
AC117490.2	0	0	0	0	0	0
COX6B1P7	0	0	0	0	0	0
RP1-127D3.4	0	0	0	0	0	0
RP11-220H4.2	0	0	0	0	0	0
RPS2P1	0	0	0	1.00570022	0	0
LINC00378	0	0	0	0	0	0
RPL5P25	0	0	0	0	1.007606311	0
AC011225.1	0	0	0	0	0	0
ARMC8P1	0	0	0	0	0	0

LINC01297	0	0	0	1.00570022	0	1.812121752
TRAPPC2P5	0	0	0	0	0	0
AC009478.1	0	0	0	0	0	0
ST13P6	1.36740616	0.92431791	14.52248911	6.34596836	6.781190475	8.109244842
RP5-937E21.8	0	0	0	0	0	0
PCDH9-AS3	0	0	0	0	0	0
ZNRF2P2	36.1385913	34.5349551	14.04108063	20.1140043	28.21297672	11.77879139
TAF1A-AS1	18.557655	26.4090833	75.2200748	42.2394091	18.1369136	46.20910469
RPL8P2	0	0	0	0	0	0
LINC00705	0	0	0	0	0	0
CICP12	0	0	0	0	0	0
RP11-66N5.2	0	0	0	0	0	0
RP11-466F5.4	0	0	0	0	0	0
NUP210P2	0	0	0	0	0	0
MTND1P18	0	0	0	0	0	0
RP11-536C5.2	0	0	0	0	0	0
RP11-227D2.3	0	0	0	0	0	0
AP000350.6	0	0	1.002934331	0	0	0.906060876
AC018693.6	0	0	0	0	0	0
RP4-758J18.10	3.90687474	1.01573397	0	0	1.007606311	1.812121752
AC005105.2	0	0	0	0	0	0
OFD1P13Y	0	0	0	0	0	0
RPL29P29	0	0	0	0	0	0
RP11-57H14.3	0	0	0	0	0	0
ABCD1P4	0	0	0	0	0	0
OSTCP2	0	0	0	0	1.007606311	0
MTND4P4	0	0	0	0	0	0
RP4-612C19.2	0	0	0	0	0	0
LINC00113	0	0	0	0	0	0
RP11-344A5.1	0	0	0	0	0	0
RP11-1086F11.1	2.93015605	3.04720192	9.026408976	10.0570022	1.007606311	4.530304381
RP11-539I5.1	123.066554	141.187022	131.3843973	107.609923	143.0800962	131.378827
RP11-512N4.2	0	0	0	0	0	0

AC106901.1	0	0	0	0	0	0
ASS1P11	0	0	1.002934331	0	0	0
DNAJC19P4	0	0	0	0	0	0
RP1-91J24.3	0	0	0	0	0	0
RP11-415J8.3	0	0	0	0	0	0
AC018634.9	0	0	0	0	0	0
RP11-293P20.2	0	0	0	0	0	0
LINC00350	0	0	0	0	0	0
LINC01427	0	0	0	0	0	0
Z84721.4	0	0	0	0	0	0
RP11-479J7.1	0	0	0	0	0	0
USP9YP19	0	0	0	0	0	0
USP17L3	0	0	0	0	0	0
LINC01594	0	0	0	0	0	0
LHFPL3-AS2	0	0	0	0	1.007606311	3.624243505
AF064860.5	0	0	0	0	0	0
AP001055.6	0	0	2.005868661	0	0	1.812121752
RP1-144F13.4	0	0	0	0	0	0
RP11-449J1.1	0	0	0	0	0	0
XXbac-B476C20.9	2.93015605	1.01573397	19.05575228	29.1653063	32.24340196	41.6788003
HMGB3P1	0	0	0	0	0	0
RP11-561O23.7	0	0	0	0	0	0
RPL23AP18	0	0	0	0	0	0
RP11-513I15.6	100.602024	221.430006	214.6279468	329.869671	100.7606311	115.0697313
AC013269.4	0	0	0	0	0	0
AC079630.4	1.95343737	4.06293589	2.005868661	5.02850108	0	1.812121752
RPL21P66	0	0	0	0	0	0
RP4-738P15.4	0	0	0	0	0	0
SNX18P3	65.7722362	50.288989	354.0859655	419.417218	101.9899108	291.153602
SLC25A5P8	0	0	1.002934331	0	0	0
RP11-820K3.2	0	0	0	0	0	0
FREM2-AS1	0	0	0	0	0	0
RPL31P53	0	0	0	0	0	0

RP11-400G3.3	0	0	0	0	0	0
ARL6IP1P2	0	0	3.008802992	1.00570022	0	0.906060876
RP11-433O3.1	0	0	1.002934331	1.00570022	0	2.718182629
RPF2P1	0	1.01573397	0	0	1.007606311	0.906060876
MIPEPP1	0	0	0	0	0	0
RP11-540K16.2	0	0	0	0	0	0
RP11-149F8.4	0	0	0	0	0	0
PPP1R26-AS1	67.3935892	63.9912403	72.21127181	53.3021114	176.3311045	82.45153973
CT62	5.8603121	4.06293589	0	2.01140043	8.06085049	0
ATP6V0E1P1	0	0	0	0	0	0
AC078942.1	0	0	0	0	0	0
TDGF1P3	0	0	0	0	0	0
AC009506.2	0.24417967	1.42202756	0.712083375	2.37345251	1.430800962	1.304727662
AC011515.2	0	0	0	0	0	0
CICP8	0	0	0	0	0	0
TMEM246-AS1	0	0	0	0	0	0
NRSN2-AS1	21.487811	47.7394967	117.3433167	102.581422	62.4715913	48.92728731
AC015977.6	0	0	0	0	0	0
IGKV1OR9-2	0	0	0	0	0	0
RPS5P7	0	0	0	0	0	0
SFTA1P	160.181864	147.281426	0	0	15.11409467	7.248487009
RP13-643D4.1	0	0	0	0	0	0
RP11-350E12.4	0	0	0	0	0	0
AC099754.1	0	0	0	0	0	0
RP11-422P22.1	0	0	0	0	0	0
NHEG1	0	0	0	0	0	0
AC016700.4	1.95343737	0	0	0	2.025288686	0
RP11-218L14.4	0	0	3.008802992	2.01140043	0	3.624243505
AC106883.1	0	0	0	0	0	0
RP11-69A21.2	0	0	0	0	0	0
RP11-552J9.13	0	0	0	0	0	0
PGM5P4	0	0	0	0	0	0
RP11-3B7.1	0	0	0	0	0	4.530304381

RAB28P5	0	0	0	0	0	0
TGIF2P1	1.95343737	1.01573397	0	2.01140043	0	7.248487009
AC010878.3	0.97671868	0	0	0	0	0
RPL26P34	0	0	0	0	0	0
RPS15AP17	0	0	0	0	0	0
AC080125.1	0	0	0	0	1.027758438	0
CTD-2384B11.2	0.97671868	0	0	0	0	0
RP11-207C16.4	0	0	7.020540315	4.02280086	0	0.906060876
RP11-764K9.1	0	1.04620599	0	0	2.529091841	0
AP000345.4	0	0	0	0	0	0
RP3-509I19.1	0	0	0	1.00570022	0	0
AC104843.3	0.97671868	5.07866986	0	0	0	0
RP5-1178H5.2	0	0	0	0	0	0
AKR1C5P	0	0	0	0	0	0
RPL21P3	0	0	2.45718911	0	0	0
AC104134.2	4.88359342	0	1.002934331	2.01140043	0	2.718182629
AC019330.1	0	0	0	0	0	1.812121752
RBMS1P1	5.91891522	4.08325057	0	0	1.027758438	2.727243237
TNPO1P1	0.97671868	3.04720192	1.002934331	1.00570022	3.022818934	0.906060876
RP11-168C9.1	0	0	0	0	0	0
LINC00331	0	0	0	0	0	0
AC108479.1	0	0	0	0	0	0
AP001626.1	0	0	5.014671654	3.01710065	0	1.812121752
MTND1P12	0	0	0	0	0	0
LINC01504	0	0	0	0	1.007606311	0
PACRG-AS2	0	0	0	0	0	0
KRT41P	0	0	0	0	0	0
BOLA3-AS1	50.7893716	57.8968364	10.02934331	9.05130194	14.10648836	4.530304381
MPRIP-AS1	0	0	0	0	0	0
AC004938.5	0	0	0	0	0	0
AC087073.1	0	0	0	0	0	0
RP4-665J23.2	0.97671868	1.01573397	0	0	0	0
RPS15AP10	8.79046815	7.11013781	12.03521197	15.0855032	3.022818934	10.87273051

AC009477.7	0	0	0	0	0	0
RAB6C-AS1	0	1.01573397	420.2294846	233.32245	51.43830219	251.8849236
RP3-508I15.14	0	1.01573397	5.014671654	7.03990151	3.022818934	3.624243505
RP11-340I6.5	0	0	0	0	0	0
TPI1P4	0	0	0	0	0	0
RP11-736E3.1	0	0	0	0	0	0
RP5-1121H13.4	0	0	0	0	0	0
RP13-93L13.1	0	0	0	0	0	0
FDPSP1	0	0	0	0	0	0
ZNF70P1	0	0	0	0	0	0
RFPL1S	0	1.01573397	3.008802992	1.00570022	3.022818934	0
OFD1P10Y	0	0	0	0	0	0
RP3-406C18.2	0	1.01573397	0	0	0	0
JPX	377.335729	371.626588	861.0492109	607.181448	457.6648627	550.6403763
RP11-262D11.2	0	0	0	0	0	0
RP11-120J1.1	7.81374947	8.12587178	14.04108063	21.1197045	1.007606311	14.49697402
ATP13A4-AS1	0	0	0	0	0	0
RP11-377K22.2	0	0	0	0	0	0
AC013437.6	0	0	0	0	0	0
CTD-2302A16.2	0	0	0	0	0	0
RP1-8B22.2	0	0	0	0	0	0
PLCB1-IT1	0	0	0	0	2.015212623	0
AC006548.19	0	0	0	0	0	0
RP11-542E6.3	0	0	0	0	0	0
RP11-38J22.2	0	0	0	0	0	0
NUTM2B-AS1	308.506363	281.896649	474.3377917	502.477999	393.5307209	529.0217638
RP11-301G21.1	0	0	0	0	0	0
RP11-403P14.1	0	0	0	0	0	0
RP11-760D2.1	0	0	0	0	0	0
RP11-390F4.3	0	0	113.3315794	76.4332164	114.8671195	37.14849592
UBE2Q2P4Y	0	0	0	0	0	0
GBP1P1	0	0	0	0	0	0
LINC01107	0	0	0	0	0	0

AC104651.2	0	0	0	0	0	0
KCNMA1-AS2	0	0	0	0	0	0
AC002064.5	0	0	0	0	0	0
RPL15P4	0	0	0	0	0	0
SNRPFP3	0	0	0	0	0	0
GAPDHP16	0	0	0	0	0	0
RP11-330C7.3	0	0	0	0	0	0
CYP4A22-AS1	1.95343737	6.09440383	0	0	2.015212623	0.906060876
AC069282.6	0	0	0	0	0	0
SSXP5	0	0	0	0	0	0
AIFM1P1	0	0	0	0	0	0
PCDH8P1	0	0	0	0	0	0
LINC00475	0	0	0	0	25.19015778	0.906060876
RP11-165N19.2	1.95343737	2.03146794	0	0	4.030425245	0
MTND1P34	0	0	0	0	0	0
FAM197Y5	0	0	0	0	0	0
RP11-396C23.2	25.3946858	18.2832115	25.07335827	10.0570022	20.15212623	9.060608762
RP11-236B18.2	0	0	0	0	0	0
TTTY16	0	0	0	0	0	0
AC005237.4	0	0	1.002934331	0	0	0
RP11-203F10.1	1.95343737	1.01573397	0	0	0	0.906060876
IGKV6D-21	0	0	0	0	0	0
MKRN2OS	0	0	76.22300913	96.5472207	57.43355974	47.11516556
RP11-383B4.4	0.97671868	1.01573397	2.005868661	1.00570022	1.007606311	1.812121752
RP3-370M22.8	0	0	0	0	0	0
SP3P	3.90687474	2.03146794	0	0	0	0
RP11-196I18.3	0	0	0	0	1.007606311	0
XX-C2158C6.3	0	0	0	0	2.015212623	0
PAWRP1	0	0	0	0	0	0
LINC01393	0	2.03146794	0	0	0	0
STIP1P3	1.95343737	0	2.005868661	0	4.030425245	0.906060876
AC073325.1	0	0	0	0	0	0
OR5BE1P	0	0	0	0	0	0

AC018799.1	0	0	0	0	0	0
AC002480.5	1.95343737	10.1573397	0	0	1.007606311	0
ZNF385D-AS1	0	0	0	0	0	0
KB-1027C11.4	0	0	1.694959019	0	0	4.013849681
RP11-545I10.2	0	0	0	0	0	0
RP11-328J2.1	0.97671868	0	0	0	0	0.906060876
AC098973.2	0	0	0	0	0	0
RP11-210H10__A.1	0	0	0	0	0	0
RP11-19J3.5	0	0	0	0	0	0
NAALADL2-AS1	0	0	0	0	0	0
RP11-527D7.1	0	0	1.002934331	0	0	0
AP000320.6	0.97671868	0	5.014671654	3.01710065	0	3.624243505
C2CD4D	2.93015605	2.03146794	32.09389858	19.1083041	32.24340196	25.36970453
Z95114.6	0	0	0	0	0	0
UBE2D3P2	0	0	0	0	0	0
AC083867.4	0	0	0	0	0	0
FAM197Y8	0	0	0	0	0	0
RP11-412H9.2	0	0	0	0	0	0
AC006076.1	0	0	0	0	0	0
LINC01492	0	0	0	0	0	0
RP11-384B12.3	1.95343737	0	1.002934331	1.00570022	0	2.718182629
CCT4P2	0.97671868	1.01573397	0	0	1.007606311	0
AC013733.4	0	0	0	0	0	0
DOCK4-AS1	0	0	0	0	0	0
RPL35P5	3.90687474	6.09440383	14.06113932	3.01710065	7.053244179	1.812121752
DRAXINP1	0	0	0	0	0	0
NCBP2-AS1	0	0	0	3.01710065	0	0.906060876
EDNRB-AS1	0	0	0	0	0	0
RP1-159M24.1	4.88359342	1.01573397	4.021766666	4.03285786	5.038031556	1.812121752
TRIM53AP	0	0	0	0	0	1.812121752
AC011193.1	0	0	0	0	0	0
RP4-630C24.1	0	0	0	0	0	0
IPPKP1	0	0	0	0	0	0

AC096669.1	0	0	0	0	0	0
RP11-360D2.2	0	0	0	1.00570022	0	0
AC098826.4	0	0	0	0	0	0
LINC01623	0	0	24.07042394	16.0912034	0	41.6788003
RP11-339D23.1	0	0	0	0	0	2.718182629
MTOR-AS1	0	0	0	2.01140043	0	0.906060876
RP11-325P15.1	0	0	0	0	0	0
RP11-550H2.1	0	0	0	0	0	0
AC005281.2	0	0	0	0	5.038031556	0
CDY20P	0	0	0	0	0	0
AC007679.3	0	0	0	0	0	0
RP11-70C1.1	0	1.01573397	0	0	1.007606311	4.530304381
AC099552.2	0	0	0	0	0	0
LINCMD1	0	0	0	0	0	0
ZNF469	0	1.01573397	0	0	884.6783413	97.85457463
RBMY2UP	0	0	0	0	0	0
RP4-604A21.1	0.97671868	0	0	0	0	0
MYT1L-AS1	0	0	0	0	0	0
RP11-569A11.2	0	0	0	0	0	0
PPIAP17	0	0	0	0	0	0
AGBL4-IT1	0	0	1.002934331	0	0	0
TBL1YP1	0	0	0	0	0	0
RP11-31H5.1	0	0	0	0	0	0
C9orf135-AS1	0	0	0	0	0	0
MTND2P28	668.954627	727.570244	607.4672948	552.451242	548.1277573	576.3725052
RP5-997D24.3	0	0	0	0	0	0
HNRNPA3P15	0	0	0	0	0	0
AP001046.6	0	0	0	0	0	0
PABPC1P12	0	0	0	0	0	0
PGAM1P2	0	0	1.002934331	0	0	0
RP5-1022P6.4	0	0	0	0	0	0
SNRPEP5	0	0	0	0	0	0
RP11-70P17.1	0.97671868	3.04720192	2.005868661	0	0	0.906060876

RPL35AP4	0	0	0	0	0	0
AC005487.2	0	0	0	0	0	0.906060876
SBDSP1	418.221173	357.528201	457.1174093	394.023287	528.5398906	618.9483057
AC064875.2	0	1.01573397	0	0	0	0
EIF2S2P5	0	0	0	0	0	0
KCNMA1-AS3	0	0	3.008802992	0	0	0.906060876
RNF19BPY	0	0	0	0	0	0
PGM5-AS1	2.12924673	1.01573397	0	0	0	0
RP5-858B6.1	0	0	1.002934331	1.00570022	0	0.906060876
RP5-843L14.1	0	0	0	0	0	0
TAF13P2	0	0	0	0	0	0
RP1-73A14.1	0	0	0	0	0	0
RPL14P5	0	0	0	0	0	0
RP11-1036F1.1	0	0	0	0	0	0
FAM195B	814.583382	980.183283	984.8815128	1068.05363	444.3543833	582.5971434
YWHAZP8	0	0	0	0	0	0
SAR1P3	0	0	0	0	0	0
RPL23AP27	0	0	0	0	0	0
CADM3-AS1	0	0	0	0	0	0
FCF1P6	0	0	1.002934331	0	0	0
CCNT2P1	0	0	0	0	0	0
RP11-641C17.3	0	1.01573397	1.002934331	2.01140043	0	0
IPO7P2	11.7792273	4.06293589	0	2.01140043	0	0.906060876
RP4-784A16.5	0	2.03146794	1.002934331	0	13.09888205	0
RP3-438O4.4	0	0	0	0	0	0
AP000619.5	0.97671868	0	0	0	0	0
AL163953.2	0	0	0	0	0	0
AC005730.2	0	0	0	0	0	0
PACRG-AS3	0	0	0	0	0	0
FAM225B	287.223663	385.643717	0	0	42.40007358	0
TSPY5P	0	0	0	0	0	0
RP1-30E17.2	0	0	0	0	0	0
TREML5P	0	0	0	0	0	0

LAGE3P1	0	2.03146794	20.05868661	9.05130194	3.022818934	9.060608762
HNRNPA1P35	2.17808267	2.16351336	0	1.07609923	4.252098634	0
SLC26A6	877.093378	1289.98214	651.907315	1695.61056	367.7763036	1135.294278
IGHV3-72	0	0	0	0	0	0
EIF4A1P13	0	0	0	0	0	0
AC005522.7	0	0	0	0	0	0
RP4-798A17.5	0	0	0	2.01140043	0	0
AC004899.3	0	0	0	0	0	0
PTPRD-AS1	0	0	0	1.00570022	0	0
RP13-379L11.1	0	0	18.05281795	18.1026039	0	17.21515665
RP5-875O13.6	0	0	0	0	0	0
RP11-345I18.4	0	0	0	0	0	0
ATP5G2P1	0	0	0	0	0	0
RPL30P1	0	0	0	0	0	0
RP11-325E14.5	0	0	0	0	0	0
TCEB1P13	0	0	0	0	0	0
RP11-3P22.2	0	0	0	0	0	0
NMNAT1P2	0	0	0	0	0	0
RP4-742C19.12	6.83703079	6.09440383	0	0	0	0
RP11-269F19.2	0	0	0	1.00570022	2.015212623	0.906060876
RP11-706O15.8	0	0	0	0	0	0
MTND6P15	0	0	0	0	0	0
LL22NC03-80A10.11	0	0	0	0	2.015212623	0
FAM66E	0	0	6.057723357	2.08179945	1.027758438	0
AC007000.10	0.97671868	0	3.008802992	5.02850108	1.007606311	0
RP11-71C5.2	0	0	0	0	0	0
RP6-191P20.3	0	0	0	0	0	0
RP3-365O12.2	0	0	0	0	0	0
AP001627.1	0	0	0	0	0	0
FGD5-AS1	4536.85829	4420.47425	2700.902153	2656.05427	5829.002511	2725.431116
NEK4P1	0	0	0	0	0	0
NRG3-AS1	0	0	0	0	0	0
NPM1P18	0	1.03604865	0	0	1.017682374	0

TCEB1P6	0	0	0	0	0	0
LL22NC03-88E1.17	0	0	0	0	0	0
RP11-513G11.4	0	0	1.002934331	0	1.007606311	0.906060876
AC017002.4	0	0	0	0	0	0
AL773572.7	0	0	0	0	0	0
SNHG23	0	0	0	0	0	0
RP11-242O24.3	0	0	0	0	0	1.812121752
AC087501.1	0	0	0	0	0	0
RP1-45I4.2	0	0	0	0	0	0
RP11-267N12.2	0	0	0	0	0	0
DBH-AS1	167.018895	211.272666	0	0	1.007606311	0.906060876
RPS17P17	0	0	0	0	0	0.906060876
LINC00489	0	0	0	0	0	0
LINC00431	0	0	18.26343416	19.5407552	0	11.31670034
RP11-417O11.5	0	2.03146794	0	0	0	0
LINC01389	2.93015605	1.01573397	9.026408976	11.0627024	9.068456802	5.436365257
P3H2-AS1	0	0	0	0	0	0
AC068535.3	0	0	0	0	0	0
DHRS4L1	0	0	7.481890107	3.15789868	4.262174697	0
RP5-850O15.3	0	0	0	0	0	0
RP11-127O4.3	0	0	0	0	6.045637868	6.342426133
CROCCP1	0	0	0	0	0	0
AC092933.3	0	0	0	0	1.007606311	0
SIRPAP1	0.97671868	0	0	0	0	0
RP11-354I10.1	0	0	0	0	0	0
DDX39AP1	0	0	0	0	0	0
PROSER2-AS1	0	1.01573397	0	0	0	0
RP4-603I14.3	0	0	2.005868661	3.01710065	0	1.812121752
OR6V1	0	0	0	0	0	0
MIAT	26.9672029	13.9257128	25.00315286	12.8427918	13.22987087	18.12121752
RP4-583K8.1	0	0	0	0	0	0
LSM3P3	0	0	0	0	0	0
RP11-2L8.1	0	0	0	0	0	0.906060876

TRAM2-AS1	262.737326	198.068125	97.28463008	78.4446168	107.8138753	67.04850484
AC004540.4	0	0	0	0	5.038031556	1.812121752
RP1-234P15.4	11.7206242	24.3776153	4.011737323	4.02280086	3.022818934	6.342426133
AC073321.4	0	0	1.002934331	0	0	2.718182629
RP11-395G17.1	0	0	0	0	0	0
MTND4P23	0	0	0	0	0	0
UBDP1	0	0	0	0	0	0
AC025918.2	0	0	0	1.00570022	0	0
RABEPKP1	0	0	0	0	0	0
WARSP1	0	0	0	0	0	0
MRPS11P1	0	0	0	0	0	0
CTD-2313N18.7	0	0	0	0	0	0
RP1-309F20.3	3.90687474	3.04720192	21.06162094	16.0912034	5.038031556	18.12121752
RP11-44M6.1	0	0	0	0	0	0
DNAJC19P5	0.97671868	2.03146794	6.017605984	4.02280086	4.030425245	4.530304381
RBMY2KP	0	0	0	0	0	0
RP11-547I7.1	0	0	0	0	0	0
AC009299.4	0	0	0	0	3.022818934	0
GRPEL2P2	0	0	0	0	0	0
AC092669.6	0	0	0	0	0	0
GTF3AP5	0	0	0	0	0	0
AC118653.2	0	0	0	0	0	0
AC013269.3	0	0	0	0	0	0
UBXN7-AS1	0.97671868	0	6.017605984	4.02280086	1.007606311	7.248487009
RPL7P45	0	0	0	0	0	0
IGHD6-25	0	0	0	0	0	0
LINC00626	0	0	0	0	0	0
SFTPA3P	0	0	0	0	0	0
FAM229A	231.482328	178.769179	321.9419202	255.447855	35.2662209	210.2061233
ERCC6	883.735065	843.262344	544.7939284	669.192923	442.1981058	718.0441838
RPS18P1	0	0	0	0	0	0
RP3-348I23.3	0	0	0	0	0	0
GS1-594A7.3	0	0	0	0	0	0

RP11-80H5.6	0	0	0	0	0	0
TRMT2B-AS1	0	0	0	0	0	0
AC010970.2	0	0	0	0	0	0
AC139712.1	0	0	0	0	0	0
NIPA2P1	0	0	0	0	0	0
RP11-630D6.1	0	0	0	0	0	0
RP5-981L23.2	0	0	0	0	0	0
RP11-452K12.4	3.90687474	3.04720192	2.005868661	4.02280086	1.007606311	1.812121752
HLA-S	0	0	0	0	0	0
RP11-569G9.7	0	0	0	0	0	0
RUSC1-AS1	20.5110924	23.3618814	105.3081047	143.815131	43.32707139	75.20305272
PCNPP2	0	0	0	0	0	0
RP11-46A10.2	0	0	0	0	1.007606311	1.812121752
RP1-172N19.3	0	0	0	0	0	0
RP11-1018N14.2	0	0	0	1.00570022	0	1.812121752
HCG4P11	0	2.03146794	0	0	1.007606311	1.812121752
RP5-1177I5.3	0	0	1.002934331	0	0	0
AC016582.2	0	0	0	0	0	0
CTB-70D19.1	0	0	0	0	0	0
LINC00368	0	0	0	0	0	0
RP11-495P10.10	1.43577647	2.19398538	1.133315794	3.01710065	4.191642255	0.625182005
LINC01529	0	0	0	0	0	0
LINC00694	0	0	0	0	0	0
AC024067.1	0	0	0	0	0	0
AC004603.4	4.88359342	3.04720192	0	0	0	0
SERBP1P2	0	0	0	0	0	0
RP3-495K2.3	0	0	0	0	0	0
LINC00115	26.4690763	11.6199966	32.62545378	46.56392	12.54469858	21.36491546
AC009365.4	0	0	0	0	0	0
LINC01456	0	0	0	0	3.022818934	0.906060876
BMS1P11	3.94594348	4.46922948	5.977488611	7.87463269	4.604760843	0
AC098872.3	0	0	0	0	0	0
AC023590.1	0	0	0	0	0	0

RP11-288L9.4	0	0	0	0	0	0
AC074289.1	4.88359342	5.07866986	0	0	0	0
RP3-476K8.3	0	0	2.005868661	0	0	3.624243505
RP11-6J24.3	0	0	0	0	0	0
TRAPPC2P8	0	0	0	0	0	0
AC007742.3	0	0	0	0	0	0
AC002075.3	0	0	0	0	1.007606311	0
FRG2B	0	0	0	0	0	2.083940015
HSPE1P13	0.97671868	1.01573397	1.002934331	0	0	0
MTND2P9	0	0	0	0	0	0
RP1-144F13.3	0	0	0	0	0	0
MORF4L1P7	0	0	0	0	0	0
RP4-758J18.7	0.97671868	1.01573397	0	0	0	0
AP000949.1	0	0	0	0	0	0
RPS27P16	0	0	0	1.00570022	0	0
AC009310.1	0	0	0	0	0	0
RP13-258O15.1	3.90687474	2.03146794	1.002934331	2.01140043	2.015212623	0
RP11-57C13.6	0	0	0	0	3.022818934	0
XXbac-BPG154L12.4	4.88359342	3.04720192	0	0	1.007606311	0
AC007879.4	0	0	0	0	0	0
RPL7P59	0	0	0	0	0	0
RP11-66A2.1	0	0	0	0	0	0
RIMKLBP2	0	0	0	0	0	0
NOL7	3105.96541	3439.27523	1009.954871	746.22956	1591.010366	794.6153884
RP11-57G10.1	0	0	0	0	0	0
RP4-736I12.1	0	0	0	0	0	0
RP1-111D6.2	0	0	0	0	0	0
RP13-313G19.2	0	0	0	0	0	0
CACYBPP1	0	0	0	0	0	0
AC000036.4	0	0	1.002934331	0	0	0
DKFZP434L187	0	0	0	0	0	0
RP3-395M20.7	0	3.04720192	0	0	0	0
CTAGE4	48.6405905	51.1929922	1130.056257	649.662225	128.6108696	590.6248427

AC133965.1	0	0	0	0	0	0
RP4-566D2.1	0	0	0	0	0	0
BANF1P5	0	0	0	0	0	0
RP11-501J20.5	0	0	0	0	0	0
PCA3	0	0	0	0	0	0.906060876
RP4-575N6.4	0	0	0	0	0	0
C5orf67	0	0	0	0	0	0
AC093326.2	0	0	0	5.02850108	0	1.812121752
AC016907.2	0	0	0	0	0	0
RIOK3P1	0	0	0	0	0	0
RP3-441A12.1	0	0	0	0	0	0
RP11-313E4.1	0	0	0	0	0	0
RP11-554I8.1	0	0	0	0	0	0
RP3-358H7.1	0	0	0	0	0	0
NTF4	0	1.01573397	18.53422643	5.02850108	3.022818934	6.197456393
ODF2-AS1	0	0	0	0	0	0
RP11-69E9.1	0	0	0	0	0	0
SATB2-AS1	1.95343737	2.03146794	1.002934331	0	4.030425245	0.906060876
RP5-1077I2.3	0	0	0	0	0	0
CTD-2267G17.2	0	0	0	0	0	0
MTND4P3	0	0	0	0	0	0
RP11-360A18.1	0	0	0	0	0	0
AC009950.2	0	2.03146794	12.03521197	2.01140043	1.007606311	3.624243505
NRIR	0	0	0	0	0	0
RP11-508N22.6	0	0	0	0	0	0
ELFN1	0	1.01573397	1.002934331	0	137.0344583	27.18182629
ABHD11-AS1	3.90687474	4.06293589	1.002934331	2.01140043	2.015212623	4.530304381
RP3-526F5.2	0	0	0	0	0	0
CTA-85E5.6	0	0	1.002934331	0	2.015212623	0.906060876
MTND1P23	87.2893488	91.5277882	63.03442269	65.410742	52.24438724	60.75138175
PIGBOS1	119.159679	97.5104613	158.4636243	149.778933	127.9660015	114.1636704
AC005550.5	0	0	0	0	0	0
LINC01534	11.7206242	20.3146794	29.08509559	22.1254047	36.27382721	29.90000891

RP11-192N10.2	0	0	0	14.4116841	0	3.107788805
HAR1A	0	0	0	0	4.030425245	0.906060876
AC010746.3	0	0	0	0	0	0
OR7E19P	0	0	0	0	0	0
AC102953.4	2.93015605	0	0	0	0	0
RP11-538D16.3	0	0	0	1.00570022	0	0
HMGB1P26	0	0	0	0	0	0
UBXN10-AS1	0	0	0	0	0	0
LAMP5-AS1	0	0	0	0	0	0
SNRPEP7	0	0	0	0	0	0
RPL23AP34	0.97671868	0	4.011737323	2.01140043	2.015212623	0.906060876
TRGVA	0	0	0	0	0	0
OR4A8	0	0	0	0	0	0
NDUFA12P1	0.97671868	0	0	0	0	0
GTF2IP14	0	0	0	0	0	2.718182629
RP11-312J18.3	0	0	0	0	0	0
RP11-10J5.1	0	0	0	0	0	0
RP11-464C19.3	0	0	0	0	0	0
RP11-211N8.2	48.3866436	25.952003	22.88696143	17.1572457	29.5833213	21.22900633
SEPT14P3	0	0	0	0	0	0
KCNIP2-AS1	0	4.06293589	0	3.01710065	1.007606311	0.906060876
RP11-403E24.1	1.95343737	0	0	0	2.015212623	0
OFD1P1Y	0	0	0	0	0	0
AP001434.2	0	0	0	0	0	0
MRPS18BP1	0	0	0	0	0	0
RP11-467I20.4	0	0	0	0	0	0
CCT8P1	2.93015605	7.11013781	29.11518362	12.0684026	12.09127574	40.78180004
RPL36AP55	0	0	0	0	0	0
PRICKLE2-AS3	0	0	0	0	0	0
CDK2AP2P3	0	0	0	0	0	0
AC026167.1	0	0	0	0	0	0
CT47A6	0	0	0	0	0	0
COX5BP7	0	0	0	0	0	0.906060876

LGALS17A	0	0	0	0	0	0
RP11-57H12.3	0	3.04720192	2.005868661	1.00570022	0	0
PFN1P12	0	0	0	0	0	0
RP4-798A10.2	5.8603121	1.01573397	29.08509559	22.1254047	5.038031556	31.71213067
HLA-DQB3	0	0	0	0	0	0
FGF13-AS1	0	0	0	1.00570022	0	1.812121752
RP1-111C20.3	0	0	0	0	0	0
NANOGNBP1	0	0	0	0	0	0
RP11-338C15.2	0	0	0	0	0	0
LINC01040	0.97671868	0	0	0	0	0
PPIAP21	0	0	0	0	2.015212623	0
AC005740.3	0	0	0	0	6.08594212	0
AC010745.1	0	0	0	0	0	0
CDY10P	0	0	0	0	0	0
AP000705.7	0	0	0	0	0	0
AC009274.6	1.95343737	0	0	0	1.007606311	0
AP1S2P1	0	0	0	0	0	0
RP11-196I18.4	0	0	0	0	0	0
YBX1P8	0	0	0	1.00570022	0	0
TLK2P1	15.1782083	16.6885092	6.54916118	20.8682795	23.38654249	8.444487366
ZNF503-AS1	0	0	1.002934331	0	4.030425245	3.624243505
RP5-1070A16.1	20.5110924	16.2517436	0	0	0	0
MEMO1P1	14.8363568	6.19597723	8.103709392	5.1391281	6.186702751	15.72921681
PAICSP1	0.97671868	0	2.005868661	6.03420129	2.015212623	1.812121752
MTND4P32	0	0	0	0	0	1.812121752
PHF2P2	0	0	0	0	2.015212623	0
AC092570.1	0	0	0	0	0	0
HMGB3P20	0	0	0	0	0	0
PCMTD1P1	0	0	0	0	0	0
AC009531.2	0	0	0	0	0	0
PGBD4P6	0	0	0	0	0	0
ZBTB45P2	0	20.3146794	0	0	0	0
AC009541.1	0	0	0	0	0	0

LINC00623	64.297391	136.362286	0	0	17.90516415	4.122576987
HNRNPA3P4	0.97671868	1.01573397	0	3.32886771	0	0
RP11-552E20.4	0	0	0	2.01140043	0	0
AC016772.4	0	0	0	0	0	0
PRSS44	0	0	0	0	0	0
PHKG1P1	0	0	0	0	0	0
RP11-205K6.2	0	0	0	0	0	0
RP3-399L15.2	0	0	0	0	0	0
RP11-345J13.1	0	0	0	0	0	0
USP12PX	0	0	0	0	0	0
RP11-24H1.1	0	0	0	0	0	0
SLC39A12-AS1	0	0	0	0	0	0
RP4-706A16.3	75.9203433	87.8406739	161.8435129	66.0141621	79.43968158	58.36844164
UQCRFS1P1	166.745414	175.833708	131.8056297	161.052833	135.4827446	145.5314979
EIF3LP3	0	1.01573397	1.002934331	0	1.007606311	0
AC106869.2	0.97671868	0	0	0	0	0
RP11-180O5.2	2.93015605	2.03146794	0	0	0	0
RP1-300G12.2	0	0	0	0	0	0
LINC00937	0.97671868	6.09440383	0	0	3.022818934	0
RBMY2AP	0	0	0	0	0	0
RPS28P8	0	0	0	0	0	0
RPL7P3	0	0	0	0	0	0
AC087499.9	0	0	0	0	0	0
AC099342.1	0	0	0	0	0	0
SEC11B	0	2.03146794	0	0	2.015212623	0
AC007461.2	0	0	0	0	0	0
SEPT7P3	0	0	0	0	0	0
AC004383.3	1.95343737	0	4.011737323	0	5.038031556	4.530304381
RAB28P1	0	0	0	0	0	0
RP11-272G22.2	0	0	0	0	0	0
FCF1P4	0	0	0	0	0	0
RP11-672F9.1	0	0	0	0	0	0
AC111200.8	0	0	0	0	0	0

AP001476.3	0	0	0	0	0	0
USP9YP6	0	0	0	0	0	0
AC079199.2	0	0	0	0	0	0
MTND3P1	0	0	0	0	0	0
RP1-197J16.2	0	0	0	0	0	0
AC009487.6	53.895337	46.7847068	44.35978545	38.9708834	34.54074435	80.7662665
DDC-AS1	0	0	1.002934331	0	0	0
FTCDNL1	11.7206242	11.1730737	17.04988362	8.04560172	92.69978064	15.4030349
AC098823.3	0	0	0	0	0	0
PSMC1P12	0	0	0	0	0	0
RPL26P9	0	0	0	0	0	0
AC013248.2	0	0	0	0	0	0
AC073465.3	0	0	0	0	0	0
RPS3AP46	0	0	0	0	0	0
RP4-683M8.2	0	0	0	0	0	0
RP11-120E13.1	0	0	0	0	0	0
BAIAP2-AS1	343.804977	382.931708	674.9748046	692.927449	635.7995824	658.706257
RP1-228P16.7	0	0	0	0	0	0
RP11-461K13.1	0	0	0	0	0	0
AF277315.13	0	0	0	0	0	0
RPL35P8	0	0	0	0	0	0
RP1-138B7.5	0	0	0	0	0	0
RPS27AP3	0	0	0	0	0	0
KRT16P6	0	0	0	0	0	0
TUBBP10	6.83703079	2.03146794	20.67047656	0	0	11.22609426
SLC25A39P1	0	0	0	0	0	0
RP1-69D17.4	0	0	0	0	0	0
RP3-389A20.5	0	0	0	0	0	0
AC124944.3	0	0	0	1.00570022	0	0.906060876
RPL23AP76	0	0	0	0	0	0
OR52E3P	0	0	0	0	0	0
CLCP2	0	0	0	0	0	0
LINC01375	0	0	0	0	0	0

LA16c-17H1.3	1.51391396	1.79784913	5.666578969	2.76567559	0	1.630909577
EIF4A1P5	0	0	0	1.00570022	0	1.812121752
RP11-167P22.3	0	0	0	0	0	0
FGFR3P6	0	0	0	0	0	0
RP11-474O21.2	0	0	0	0	0	0
AP4B1-AS1	2.93015605	2.03146794	0	0	356.6926342	23.55758278
RP11-564C4.4	0	0	0	0	0	0
RP11-375H17.1	0	0	0	0	0	0
RP4-712E4.1	0	0	0	0	0	0
TEX22	9.76718684	1.01573397	84.24648378	58.3104985	43.72003785	38.96061768
LINC00685	1.46507803	1.52360096	12.53667913	18.605454	3.52662209	7.248487009
RP11-278A23.1	6.83703079	12.1888077	10.02934331	12.0684026	78.59329228	25.36970453
RP1-92C8.3	0	0	0	0	0	0
RANP7	0	0	0	0	0	0
TRIM64FP	0	0	0	0	0	0
TCEB1P21	0.97671868	0	0	0	0	0
HNRNPA1P3	2.93015605	0	0	0	0	0.906060876
CLK3P2	0	0	0	0	0	0
RP3-398G3.5	0.97671868	0	0	0	0	0
RP1-137D17.1	0.97671868	1.01573397	3.008802992	0	0	0
GAPDHP75	0	0	0	0	0	0
RP11-536O18.1	0	0	0	0	0	0
RP11-12D5.2	0	0	0	0	0	0
SGMS1-AS1	41.0221847	31.4877531	150.4401496	90.5130194	73.55526072	131.378827
RP11-328D5.1	0	0	0	0	0	0
RP4-760C5.5	0	0	0	0	0	0
AL157359.3	0	0	0	0	0	0
AC007790.4	0	0	0	0	0	0
RP5-1050E16.2	0	0	1.002934331	0	0	0
RP1-104O17.3	0	0	0	0	0	0
RP11-99H8.1	0	0	0	0	0	0
C8orf59P1	0	0	0	0	0	0
WASH7P	330.873221	266.599696	910.2932866	869.357437	361.4384599	871.8661387

RP11-453O22.1	0	0	0	0	0	0
TRGV6	0	0	0	0	0	0
UBQLN4P2	0	0	0	0	0	0
RPS12P5	0	2.03146794	0	0	0	0
RPL19P1	0	0	0	0	0	0
AC079163.1	0	0	0	0	0	0
CICP22	0	0	0	0	0	0
RPL26P19	13.5177866	10.3808012	13.20864514	9.44352502	15.6783542	6.5145777
RP11-753E22.3	0	0	0	0	0	0
TSPY16P	0	0	0	0	0	0
PRELID3BP1	0	0	0	0	0	0
RPLP0P1	0	0	0	0	0	0
AC007237.2	0	0	0	0	0	0
RP11-419C5.2	23.421714	21.9601685	82.85240506	73.4664007	42.148172	61.18629097
RP1-151B14.9	0	0	0	0	0	0
KRT18P24	0	0	0	0	0	0
LEMD1-AS1	0	0	0	1.00570022	1.007606311	1.812121752
RP11-276H19.1	0	0	8.023474646	6.03420129	0	2.718182629
AC132807.1	0	0	0	0	0	0
RP1-310O13.7	0	0	0	0	1.007606311	0
LINC00381	0	0	0	0	0	0
LL0XNC01-221F2.2	0	0	0	0	0	0
RPS17P8	0	0	0	0	0	0
RPL37AP1	0	0	0	0	2.015212623	0.906060876
ZNF32-AS1	0	0	0	1.00570022	0	0
KRT18P36	0	0	0	0	0	0
SUPT4H1P1	0	0	0	0	0	0
RP11-307P5.2	0	0	0	0	0	0
LINC00408	0	0	0	0	0	0
RP11-15I11.3	0	0	0	0	0	0
RP1-18D14.7	0	0	0	0	0	0
MRPL35P3	5.38171995	1.03604865	1.053081047	1.02581422	1.037834501	0.915121485
PTP4A1P3	0	0	0	0	0	0

UBE2V2P1	0	0	0	0	0	0
GRM7-AS3	0	0	0	0	0	0
GTF2H2B	259.660662	251.556676	341.6194917	352.025246	141.8508165	454.8697417
AC064836.3	0	0	0	0	0	0
PHKBP1	0	0	0	0	0	0
ISM1-AS1	0	1.01573397	0	0	0	0
AC009961.3	9.76718684	15.2360096	10.02934331	13.0741028	9.068456802	19.0272784
RP11-61N20.3	3.76036693	7.17108184	2.768098753	8.28696978	1.68270254	14.4788528
ZNF736P2Y	0	0	0	0	0	0
ARHGAP26-AS1	0	0	0	0	0	0
AC079305.5	0	0	0	0	0	0
AC093382.1	0	0	4.011737323	2.01140043	1.007606311	0
AC105393.2	0	0	0	0	0	0
PSPHP1	1.2599671	2.03146794	0	0	0	0
RPL12P10	0.97671868	0	0	0	0	0
RP6-22P16.1	0	0	0	0	0	0
RP1-80N2.2	0	0	0	0	0	0
ARPC3P1	0	1.04620599	0	0	0	1.02384879
AC091813.2	0	0	0	0	0	0
RP1-163G9.2	0	0	0	0	2.015212623	0
TMEM191A	14.2796272	29.3547118	152.2755194	552.310444	251.7000566	194.186967
OR52I2	0	0	0	0	0	0
CDCA4P3	0	0	0	0	0	0
AC091729.8	0	0	0	0	0	0
RP11-7G12.1	0	0	0	0	0	0
RP11-71J2.1	0	0	0	0	0	0
AC007677.2	0	0	0	0	0	0
RAD23BLP	0	0	0	0	0	0
RPSAP62	0	0	0	0	0	0
PDCL3P1	0	0	0	0	0	0
RP11-528N21.1	0	0	0	0	0	0
RP11-435M3.2	0	0	0	0	0	0
NPY6R	0	0	0	0	0	0.906060876

HNRNPDLP1	0	0	0	0	0	0
RP4-813D12.3	0	0	0	0	0	0
SMARCE1P2	0	0	0	0	0	0
RP3-323P24.3	0	2.03146794	7.020540315	4.02280086	1.007606311	5.436365257
CFLAR-AS1	15.6274989	15.2360096	0	0	1.007606311	0.906060876
MTCYBP12	0	0	0	0	0	0
ZNF192P1	29.3015605	24.3776153	10.02934331	7.03990151	2.015212623	14.49697402
LINC00351	0	0	0	0	0	0
RP11-474D14.2	15.6274989	3.04720192	31.09096425	8.04560172	11.08366942	9.060608762
AC018359.1	0	0	0	0	0	0
CROCC2	0	0	1.002934331	0	0	0
AC006150.1	0	0	0	0	0	0
RP11-115M14.1	0	0	0	0	0	0
RPSAP49	0	0	0	0	0	0
NUP50-AS1	89.8581189	118.840875	335.9830008	185.04884	108.8214816	202.0515754
AC005682.6	0	0	0	0	0	0
RP11-739N20.2	0	0	0	0	0	0.906060876
RPL23AP28	0	0	0	0	0	0
RP11-157P1.4	33.2084353	36.566423	28.08216126	33.1881071	40.30425245	23.55758278
RP11-217B7.2	0.97671868	0	0	0	0	0
MRPS16P3	0	0	0	0	0	0
RP11-274B18.4	0	0	0	0	0	0
AC079150.2	0	0	0	0	0	0
RPS26P56	0	0	0	0	0	0
AC105402.3	0	0	0	0	0	0
AC062016.2	0	0	0	0	0	0
NMD3P1	0	0	0	0	0	0
CST12P	0	0	0	0	0	0
AC083822.2	0	0	1.002934331	0	0	0
VN2R10P	0	0	0	0	0	0
RP11-145A3.2	0	0	0	0	0	0
RP11-523H24.3	0	0	1.002934331	0	1.007606311	0
TAF9P1	0	0	0	0	0	0

RP11-65J3.2	0	0	0	0	0	0
RPS6P20	0	1.01573397	0	0	0	0
KRT8P38	0	0	0	0	0	0
ACTG1P24	1.95343737	4.06293589	2.005868661	0	0	6.342426133
RPL10AP6	22.5719688	15.2867963	18.76490133	22.1958038	12.1819603	20.91188502
TERF1P5	0	0	0	1.04592822	0	0
FAM41AY2	0	0	0	0	0	0
HAGLROS	0	0	10.02934331	22.1254047	14.10648836	14.49697402
AC102948.2	0	0	0	0	0	0
AC097463.1	0	0	0	0	0	0
ST7-AS2	0	0	0	0	0	0
USP9YP11	0	0	0	0	0	0
LINC00375	0	0	0	0	0	0
DCAF8L1	0	0	0	0	1.007606311	0
RP13-614K11.2	0	0	0	0	0	0
RP3-395P12.2	0	0	0	0	0	0
RP11-29B9.1	0	0	0	0	0	0
AC084809.2	0	0	1.002934331	4.02280086	3.022818934	0.906060876
AC058791.1	187.529987	195.020923	4.011737323	5.02850108	20.15212623	12.68485227
AC093375.1	2.93015605	0	0	0	2.015212623	0
PARD3-AS1	1.95343737	0	0	0	0	2.718182629
SORCS3-AS1	0	0	0	0	0	0
ELL2P4	0	0	0	0	0	0
RP11-507K13.4	0	0	0	0	0	0
RP11-358D14.2	0	0	0	0	0	0
RP4-736H5.1	0	0	0	0	0	0
IFNA20P	0	0	0	0	0	0
RP11-413E1.2	0	0	0	0	0	0
RP11-218D6.3	0	0	0	0	0	0
RP5-1056L3.3	0	1.01573397	11.03227764	6.03420129	4.030425245	5.436365257
C12orf77	0	0	0	0	0	0
AC013480.2	0	0	0	0	0	0
CTD-2269E23.2	0	0	0	0	0	0.906060876

RP5-965K10.3	0	0	0	0	0	0
RP11-31F19.1	1.95343737	0	1.995839318	2.01140043	1.007606311	5.436365257
RP5-984P4.1	0	0	0	0	0	0
RBMX2P1	0.97671868	0	0	1.00570022	0	0
RPL23P11	0	0	0	0	0	0
RP11-735G4.1	0	0	0	0	0	0
AC104058.1	0	0	0	0	0	0
CTD-2526L21.2	0	0	0	0	0	0
RP11-476F14.1	0	0	0	0	0	0
OR8T1P	0	0	0	0	0	0
RP11-375A20.1	0	0	0	0	0	0
TPI1P1	15.9302817	32.6050605	8.053562676	10.5699093	43.33714745	21.49176398
MRPL23-AS1	0	0	0	0	1.007606311	0
SLC16A1-AS1	5.87984648	6.09440383	30.08802992	8.04560172	30.22818934	17.21515665
IGLV3-4	0	0	0	0	0	0
SLC25A5P5	0	0	0	0	3.022818934	0
AC093642.4	0.97671868	0	0	0	0	0
RP11-348J12.2	0	0	0	0	0	0
RP11-174J11.1	0	0	0	0	0	0
HMGN2P7	0	0	0	0	0	0
RP11-106M7.4	0	0	0	0	0	0
RP11-111F5.6	0	0	0	0	0	0
USP17L7	0	0	0	0	2.015212623	0
RP11-416N2.3	0	0	0	0	2.015212623	0
CTD-2015B23.2	2.0804108	0	4.412911055	4.40496694	4.413315643	6.994789964
AP000290.7	0	0	0	0	0	0
RP11-1L9.1	0	1.01573397	0	0	0	0
ANKRD18DP	0	0	0	0	0	0
RP1-22N22.1	0	0	2.005868661	1.00570022	0	2.718182629
RP11-420K8.1	0	0	0	0	0	0
RP11-621K7.1	0	0	0	0	0	0
RP11-506B6.6	0	0	0	0	0	0
PLCL2-AS1	0	0	0	0	0	0

AC006037.2	0	0	0	0	0	0
GAPDHP32	1.95343737	0	0	0	0	0
ACTR3BP6	0	0	0	0	0	0
XXyac-YX65C7_A.2	0.97671868	0	6.017605984	4.02280086	17.12930729	2.718182629
NOTCH2P1	0	0	0	0	0	0
RP11-393J16.4	0	0	0	0	0	0
RP11-93L14.1	0	0	0	0	0	0
CDY5P	0	0	0	0	0	0
CYP2D8P	0	5.07866986	7.020540315	8.04560172	4.030425245	4.530304381
RP11-379B8.1	0	0	0	0	11.08366942	2.718182629
RP3-462C17.1	0	0	0	0	0	0
RP3-512E2.2	0	0	0	0	0	0
RP11-430L17.1	0	0	0	0	0	0
OR10G5P	0	0	0	0	0	0
RP13-401N8.1	5.9384496	5.07866986	0	1.00570022	6.045637868	0.906060876
RPA2P2	0	0	0	0	0	0
AC018641.7	0	0	0	0	0	0
ADAM1B	0	0	0	1.00570022	0	0.906060876
RP11-132E11.3	0	0	0	0	0	0
CTA-292E10.6	2.93015605	3.04720192	27.07922693	31.1767067	19.14451991	25.36970453
RP11-551L14.4	5.8603121	1.01573397	6.017605984	4.02280086	3.022818934	0.906060876
AC079807.1	0	0	0	0	0	0
LA16c-2F2.5	0	0	0	0	0	0
RP11-776H12.1	0	0	0	0	12.09127574	0.906060876
LL22NC03-84E4.11	0	0	0	0	0	0
UPF3AP1	5.53799494	5.68811024	1.043051704	0	0	2.26515219
TMEM185B	1442.6135	1502.27054	444.2999085	663.762142	1250.439432	784.6487188
OR7H1P	0	2.03146794	0	0	0	0
ACTR3BP2	0	0	0	0	0	0
ADIPOQ-AS1	0	0	0	0	0	0
RP5-964H19.2	0	0	0	0	0	0
RP11-87M18.2	0	0	0	0	0	0
RBM22P3	0	0	0	0	0	0

LINC01035	0	0	0	0	0	0
RP4-749H3.2	0	0	0	0	0	0
AC021021.2	0	0	0	0	0	0
AC104308.2	0	0	0	0	0	0
AC138647.1	0	0	0	0	0	0
FTOP1	0	0	0	0	0	0
RP11-524O24.2	0	0	0	0	0	0
LINC00323	3.90687474	1.01573397	0	0	0	0
RP11-406O16.1	0	0	0	0	0	0
RPSAP21	2.93015605	0	0	1.00570022	1.007606311	0.906060876
RP5-882O7.1	0	0	0	1.00570022	0	0
RP11-196G18.1	0	0	0	0	0	0
USF1P1	0	0	0	0	0	0
KRT8P27	0	0	0	0	0	0
TMEM167AP1	0	0	0	0	0	0
AC007463.2	0	0	0	0	0	0
IPMKP1	0	0	0	0	0	0
AC104655.3	0	0	0	0	18.1369136	2.718182629
AC005588.2	0	0	0	0	0	0
UPK1A-AS1	0	0	1.002934331	1.00570022	1.007606311	0.906060876
AC004386.3	0	0	0	0	0	0
FAM138B	2.93015605	1.81816381	0	0	0	0
LINC00390	0	0	0	0	0	0
KIRREL-IT1	0.97671868	0	0	0	0	0
AC126365.1	0	0	1.002934331	0	0	0
AC004744.3	0	0	0	0	0	0
AC074375.1	0	0	0	0	0	0
RPS7P10	3.9361763	7.1609245	16.05697863	5.07878609	17.58273013	6.451153438
RP1-37C10.3	3.90687474	3.04720192	1.002934331	1.00570022	1.007606311	0.906060876
AP000289.6	0	0	0	0	0	0
MTND1P1	0	0	0	0	0	0
RP11-348F1.2	0	0	0	0	0	0
RP5-1052M9.1	0	0	0	0	0	0

BTBD9-AS1	0	0	0	0	0	0
KB-1183D5.16	0	0	0	0	0	0
RP11-417L14.1	0	0	1.002934331	0	1.007606311	0
SETP15	0	0	0	0	0	0
OR7E33P	0	0	0	0	0	0
AC012512.1	0	0	0	0	0	0
GAPDHP73	0	0	0	0	0	0
RP11-795J1.2	0	0	0	0	0	0
AC114814.4	0	0	0	0	0	0
MYL6P1	0	0	0	0	0	0
RPL7P22	6.15332771	2.20414272	0	3.01710065	0	0
RP4-633I8.3	0	0	0	0	0	0
SSU72P1	0	0	0	0	0	0
AC016722.3	0	0	0	0	0	0
SCDP1	0.97671868	1.01573397	3.008802992	30.2212915	3.022818934	0
RP5-1092L12.2	0	0	0	0	0	0
AC018735.1	0.97671868	0	0	0	0	0
MTCL1P1	0	0	0	0	0	0
AGKP1	0	0	0	0	0	0
NPM1P49	0	0	0	1.00570022	0	0
TRAF6P1	0	0	0	0	0	0
RP11-213N20.1	0	0	0	0	0	0
CYP4F26P	0.97671868	1.01573397	0	0	13.09888205	0
FTH1P20	1.1134593	8.0039837	27.26978445	18.9976771	14.5599112	22.3344006
RP11-164O23.5	0	0	0	0	0	0
RP11-436F21.1	0	0	0	0	0	0
LINC00606	0	0	0	0	0	0
RP11-505O17.1	0	0	0	0	0	0
RP11-445F6.2	0	0	0	0	0	0
SNORD57	0	0	0	0	0	0
CTA-85E5.7	0	0	0	0	0	0
RP11-523O18.1	0	0	0	0	0	0
MICC	0	0	0	0	0	0

RP11-258F22.1	0	0	7.020540315	5.02850108	0	0.906060876
MTCYBP31	0	0	0	0	0	0
RPL39P40	0	0	0	0	0	0
RP11-340I6.8	0	0	0	0	2.015212623	0
UBE2V1P5	0	0	0	0	0	0
RP5-905H7.9	0	0	0	0	0	0
UBE2V1P10	0	0	0	0	0	0
AC004911.2	0	0	0	0	2.015212623	0
RP11-550C4.6	0	0	0	0	0	0
KB-288A10.19	0	0	0	0	0	0
IFNWP9	0	0	0	0	0	0
AC017060.1	0	0	0	0	0	0
RP11-136K14.3	0	0	0	0	0	0
CT47A9	0	0	0	0	0	0
RP11-239E10.3	0	0	0	0	0	0
PAPPA-AS2	0	0	0	0	0	0
AC007098.1	0	0	0	0	0	0
FTLP3	65.4206174	103.645495	6.027635328	13.0841598	10.08613918	12.69391288
RP11-276E15.4	0	3.04720192	0	1.00570022	1.007606311	0
OFD1P2Y	0	0	0	0	0	0
OR52M2P	0	0	0	0	0	0
RPL21P110	0	0	0	0	0	0
RP11-301J16.7	0	0	0	0	0	0
LINC00343	0	0	0	0	0	0
AC083855.5	0	0	0	0	0	0
AC092155.4	0	0	0	0	0	0
CTA-351J1.1	0	0	0	0	0	0
RBM17P1	0	0	0	0	0	0
NOP56P2	0	0	0	0	0	0
SHANK2-AS1	0	0	0	0	0	0
LINC00974	0	0	0	0	0	0
SLC9A3P3	0	0	0	0	1.007606311	0
UBE2V1P1	2.93015605	2.03146794	1.002934331	2.01140043	2.015212623	2.718182629

PPIAP28	0	0	0	0	0	0
RP5-1049N15.2	0	0	0	0	0	0
RP11-21J7.1	0	0	0	0	0	0
RP13-13A3.1	0	0	0	0	0	0
ACTG1P12	0	0	0	0	0	0
RP11-358H9.1	0	0	0	0	0	0
RP11-128M1.1	0	0	0	6.03420129	1.007606311	0.906060876
AP006216.10	0	0	0	0	0	0
RPL7P37	0	0	0	0	0	0
RP11-336A10.2	0	0	0	0	0	0
PLCG1-AS1	2.93015605	2.03146794	0	4.02280086	8.06085049	0.906060876
AC019118.4	0	0	0	0	0	0
KIF4B	1.95343737	1.01573397	3.038891022	1.01575722	2.015212623	0.906060876
PSMD10P2	9.76718684	9.14160575	0	0	1.007606311	0.906060876
OR13Z1P	0	0	0	0	0	0
AC096732.2	0	0	0	0	0	0
RPL23AP30	0	0	0	0	0	0
RP11-137H2.4	2.93015605	0	0	0	9.068456802	0
TRBV2	0	0	0	0	0	0
RP5-1139I1.2	0	0	0	0	0	0
CHMP1AP1	0	0	0	0	0	0
MTND4P10	0	0	0	0	0	0
RP4-745E8.2	0	0	0	0	0	0
SSR1P2	0	0	0	0	0	0
HSPA9P1	0	1.01573397	0	0	1.007606311	2.718182629
RP11-526D8.7	0	0	0	0	0	0
RP11-509J21.4	0	0	0	0	0	0
BCAS2P3	0	0	0	0	0	0
AC005008.3	0	0	0	0	0	0
LINC01108	1.95343737	0	0	0	0	0
TEX41	0	0	2.005868661	31.1767067	0	0
RP11-589B3.6	0	0	0	0	0	0
IGBP1P1	0.97671868	1.01573397	0	0	0	0

RP13-43E11.1	0	0	0	0	0	0
RP11-61D3.1	0	0	0	0	0	0
AC020595.1	0	0	0	0	0	0
PWWP2AP1	0	0	0	0	0	0
CT47A12	0	0	0	0	0	0
LINC01535	0	0	2.72798138	1.00570022	7.053244179	1.948030884
ENTPD1-AS1	106.462337	123.919545	80.23474646	148.843632	97.73781219	149.5000446
AC005281.1	0	0	0	0	0	0
NXNP1	0	0	0	0	0	0
RP11-69C17.2	0	0	0	0	0	0
ANKRD20A10P	0	0	0	0	0	0
LENG8-AS1	24.4179671	31.4877531	61.17899417	37.210908	26.19776409	45.30304381
RP1-50O24.6	0	0	0	0	0	0
RP11-122K13.7	0	0	0	0	0	0
MTND4P25	0	0	0	0	0	0
RP11-570G20.1	0	0.7414858	0	0.19108304	0	0
MIR217HG	0	0	0	0	0	0
NPM1P4	0	0	0	0	0	0
SDCBPP1	0	0	0	0	0	0
RP11-426A6.5	1.95343737	0	0	6.03420129	4.030425245	2.718182629
RP11-719J20.1	0	0	0	0	0	0
AC073409.1	0	0	0	0	0	0
FGF12-AS3	0	0	0	0	0	0
FAM66C	10.7439055	5.07866986	109.7711625	47.2477961	38.68200629	64.26689795
RP11-411H5.1	0.97671868	0	3.008802992	0	3.022818934	0.906060876
AL589986.2	0	0	0	0	0	0
PTPRD-AS2	0	0	0	0	0	0
SNRPGP8	0	0	0	0	0	0
EEF1DP2	0.97671868	5.07866986	11.04230698	11.0627024	10.08613918	4.530304381
LINC00424	0	0	0	0	0	0
RP11-142L4.3	6.89563391	3.07767394	1.002934331	3.01710065	4.080805561	0.906060876
RP11-795A2.3	0	0	0	0	0	0
PABPC1L2B-AS1	0	0	0	0	1.511409467	0

TMEM256P2	0	0	0	0	0	0
RPL35AP30	0	0	0	0	0	0
RP11-397G17.1	0	0	0	0	0	0.906060876
SNRPGP12	0	0	0	0	0	0
CTA-384D8.31	0	0	0	0	0	0
RP11-347L18.1	0	0	0	0	0	0
CTA-929C8.6	0	0	0	0	0	0
HSBP1L1	290.085449	215.335602	857.5088528	992.626113	163.2322224	529.1395517
AC079781.5	0	0	0	0	0	0
RP11-115D7.3	0	0	1146.35394	1341.60409	0	712.1638487
SMCR5	0	0	0	0	0	0
AC007966.1	0	0	2.005868661	2.01140043	0	0
RP11-513D4.3	0	0	0	0	0	0
AF127936.5	0	0	0	1.00570022	0	0
PSMD5-AS1	299.852636	332.145009	1783.21724	957.426605	1027.758438	822.7032756
RP11-212D3.2	0	0	0	0	0	0
RP5-1024G6.5	0	4.06293589	20.05868661	36.2052078	30.22818934	16.30909577
VPS25P1	0	0	0	0	0	0
AC007365.3	0	1.01573397	0	0	0	0
PP12613	0	0	0	0	0	0
DISC1-IT1	0	0	0	0	0	0
DAB1-AS1	0	0	0	0	0	0
TAS2R46	1.95343737	1.01573397	5.014671654	1.00570022	4.030425245	0
RP11-298E9.6	0	0	0	0	0	0
SRRM5	3.12549979	7.11013781	51.14965087	25.1425054	20.66600544	58.21441129
AC010145.4	0	0	0	0	0	0
RPSAP33	0	0	0	0	3.022818934	0
FABP7P1	1.95343737	1.01573397	0	0	0	0
RP11-328P23.3	0	0	0	0	0	0
GAPDHP54	0	0	0	0	0	0
AC000124.1	0	0	0	0	0	0
AP001136.2	0	0	0	0	0	0.906060876
CTA-747E2.10	0	0	0	0	0	0

RP4-775D17.1	0	0	0	0	0	0
RP11-654E17.2	0	0	3.008802992	1.00570022	0	1.812121752
KRTAP3-4P	0	0	0	0	0	0
KIAA0125	0	0	0	0	6.045637868	12.68485227
NAALADL2-AS2	0	0	0	0	0	0
RP11-38J22.3	0	0	0	0	0	0
TBCAP1	0	0	0	0	1.007606311	0
RP11-706D8.3	0	0	0	0	0	0.906060876
TLK1P1	0	0	0	0	0	0
PGAM4	13.2052366	17.0440161	2.707922693	0	12.8671326	6.813577789
AC073218.1	0	0	0	0	0	0
RP1-167F1.2	0	0	0	0	0	0
AC110926.4	0	0	0	0	0	0
HNRNPA3P1	0.97671868	0	3.008802992	0	8.06085049	5.816910825
AC109826.1	0	0	0	0	0	0
LINC00371	0	0	0	0	0	2.718182629
MTND1P20	0	0	0	0	0	0
AC015923.1	0	0	1.002934331	0	0	0
RP11-289F5.1	0	0	3.008802992	8.04560172	0	0
CACTIN-AS1	27.3481231	32.5034871	5.014671654	9.05130194	6.045637868	9.060608762
OSTCP8	2.93015605	3.04720192	1.002934331	3.01710065	0	1.812121752
RP11-72I18.1	0	0	0	0	0	0
RP11-203B9.4	0.97671868	2.03146794	2.005868661	5.02850108	0	1.812121752
RP4-675G8.3	0	0	0	0	0	0
AC011893.3	1.95343737	6.09440383	0	0	0	0.906060876
MROH5	0	0	0	0	0	3.624243505
LINC00840	0	0	0	0	0	0
RP11-45H22.1	0	0	0	0	0	0
RP5-881L22.5	0	0	0	0	0	0
AC114813.1	0	0	0	0	0	0
RP3-419C19.2	0	0	0	0	0	0
AC005082.12	0	1.01573397	0	0	0	0.906060876
SLC6A6P1	0	0	0	0	0	0

MEIS1-AS3	0	0	0	0	0	0
RP11-747I9.1	0	0	0	0	0	0
RP11-356N1.2	0	0	4.011737323	1.00570022	0	9.966669638
SUGT1P	0.97671868	1.01573397	0	0	2.015212623	0
RP4-756H11.3	4.88359342	3.04720192	6.017605984	11.0627024	12.09127574	7.248487009
LINC01509	0	0	0	0	0	0
NPM1P11	0	0	0	0	0	0
RP11-278H7.1	0	0	0	0	0	0
RP5-945F2.3	0	0	0	0	0	0
MED15P3	0	0	0	0	0	0
AC097724.3	5.8603121	8.12587178	2.005868661	0	4.030425245	2.718182629
RP11-148B18.1	0.97671868	4.06293589	0	0	1.007606311	0.906060876
RP11-77B22.1	0	0	0	0	0	0
HMGB1P32	0	0	0	0	0	0
AC096582.8	0	0	0	0	0	0
MTND6P11	0	0	0	0	0	0
PAICSP3	0	0	0	0	0	0
RP11-166N17.3	0	0	0	0	0	0
RP11-2P2.1	0	0	0	0	0	0
EEF1GP3	0	0	0	0	0	0
LINC00348	0	0	0	0	0	0
PRPS1P1	0	0	0	0	0	0
RP4-635E18.7	1.95343737	0	8.023474646	11.0627024	3.022818934	7.248487009
AC004112.4	0.97671868	0	0	0	0	0
AC010894.3	2.93015605	6.09440383	7.020540315	5.02850108	7.053244179	9.966669638
RP11-212D3.4	0	0	0	0	0	0
RPSAP17	2.05110924	1.06652067	0	1.03587122	0	0
AC093901.1	3.90687474	1.01573397	1.002934331	6.03420129	1.007606311	0.906060876
DUTP3	0	0	0	0	0	0
RP11-48F14.2	0	0	0	0	0	0
AC109638.1	0	0	0	0	0	0
RP5-1119O21.2	0	0	0	0	0	0
RP11-569A11.1	0	0	2.005868661	0	0	0

SHROOM2P1	0	0	0	0	0	0
ATE1-AS1	6.83703079	2.03146794	17.04988362	21.1197045	1.007606311	11.77879139
RP13-77O11.8	0	0	0	0	0	0
RP11-122M14.3	0	0	0	0	0	0
LHFPL3-AS1	0	0	0	0	0	0
RP11-402P6.13	0	0	3.008802992	0	0	0
AC135178.7	0	0	0	0	0	0
AC002472.11	0	0	0	0	0	0
CDY14P	0	0	0	0	0	0
AC005154.8	0	0	2.005868661	0	5.038031556	3.624243505
ZNF877P	0	0	10.02934331	7.03990151	0	14.49697402
RP11-36N20.1	0	0	0	0	0	0
RP11-575L7.2	0.97671868	0	0	0	1.007606311	0
GSTM3P2	0	0	0	0	0	0
H3F3AP5	0	0	0	0	0	0.906060876
RP4-782L23.2	0	0	0	0	0	0
AC016727.3	0	0	0	0	0	0
E2F6P3	0	0	0	0	2.468635463	0
AC093787.1	0	0	0	0	0	0
ERVMER34-1	30.2782792	30.4720192	233.6836991	407.308587	42.31946507	499.2395428
RP11-561G1.3	0	0	0	0	0	0
RP11-474I16.8	0.97671868	4.06293589	0	0	0	0
LA16c-395F10.1	0	0	2.005868661	1.00570022	1.007606311	0
LINC01359	2.93015605	4.06293589	6.017605984	9.05130194	0	12.68485227
RP11-298J20.3	0	0	0	0	0	0
RP11-432J24.5	0	0	0	1.00570022	1.007606311	0.906060876
CHCHD2P1	0	0	0	0	0	0
LINC00354	0	0	0	0	0	0
RP11-561O23.8	0	1.01573397	2.005868661	1.00570022	1.007606311	0.906060876
AC005040.3	0	0	0	0	0	0
TTTY4	0	0	0	0	0	0
HIST1H2BPS3	0.97671868	0	0	0	0	0
ISCA2P1	0	0	0	0	1.007606311	1.812121752

BSN-AS2	0	0	0	0	1.007606311	0
AC068137.13	0	0	0	0	0	0
LINC01276	0	0	0	0	0	0
AC010086.1	0	0	0	0	0	0
RP11-80B9.1	0	0	0	0	0	0
RP5-1068B5.5	0	0	0	0	0	0
LINC00454	0	0	0	0	0	0
AC007271.3	0	0	0	0	0	0
PDZPH1P	3.90687474	0	0	1.00570022	0	0
RP11-302I18.3	0	0	0	0	0	0
RPS14P4	4.88359342	1.01573397	2.005868661	5.02850108	1.007606311	0.906060876
CT47A11	0	0	0	0	0	0
GTF2IP2	0	0	0	0	0	0
NRBF2P2	0	0	1.002934331	2.01140043	0	2.718182629
LINC00161	0	0	8.023474646	8.04560172	0	5.436365257
CEP164P1	3.90687474	4.06293589	8.023474646	1.00570022	2.015212623	7.248487009
RP11-117D22.1	0	0	0	0	0	0
AC062021.1	0	0	0	0	0	0
RBMY1J	0	0	0	0	0	0
IL9RP3	0.97671868	2.03146794	11.11251238	15.6889234	13.97549954	22.77837043
ALG1L5P	14.6507803	13.2045416	0	0	0	0
RP1-120G22.11	2.93015605	0	0	7.03990151	0	1.812121752
RP11-564A8.4	0	0	0	0	0	0
AC062022.1	0	0	0	0	0	0
RP1-52J10.9	0	0	0	0	0	0
RPS4XP2	0	1.01573397	2.005868661	0	0	0
OR6K5P	0	0	0	0	0	0
DANCR	803.839477	1091.91402	987.8903157	1848.477	913.8989243	1581.076229
RP4-714D9.4	0	0	0	0	0	0
AC010890.1	0	0	0	0	1.007606311	0
RP5-983L19.2	0	0	0	0	3.022818934	0.906060876
AC104306.4	0	0	0	0	0	0
AP000432.2	0	0	0	0	0	0

RP4-533D7.4	0	1.01573397	0	0	0	0.906060876
MTCO1P21	0	0	0	0	0	0
AC018463.5	0	0	0	0	0	0
RAC1P6	0	0	0	0	0	0
AC078883.4	3.90687474	3.04720192	0	0	0	2.718182629
RHEBP2	53.8171995	24.4182447	2.66780532	9.00101693	6.065789994	1.458758011
AC003088.1	0	0	1.002934331	1.00570022	2.015212623	6.342426133
HAUS4P1	0	0	0	0	0	0
LINC00423	0	0	0	0	3.022818934	0
RP11-547D24.3	0	0	0	3.01710065	0	0
RP11-82H13.2	0.98648587	0	3.028861679	0	4.141261939	0
AF196972.4	0.97671868	0	0	0	0	0
RPL12P19	0	0	0	0	0	0
RP4-663N10.2	0	0	0	0	0	0
AC006987.6	0	0	0	0	0	0
COX6A1P2	11.554582	17.429995	28.06210257	26.8019107	37.01945588	16.78024743
HMGN1P24	0.97671868	0	3.008802992	0	1.007606311	0
MAGI2-AS2	0	0	0	0	0	0
LTA	15.6274989	13.2045416	3.008802992	3.01710065	1.007606311	0
ABHD17AP6	1.00602024	0	0	0	0	0
CENPCP1	0	0	0	2.01140043	0	2.718182629
AP000235.2	0	0	0	0	0	0
RP4-591B8.2	0	0	0	0	0	0
LINC01203	0.97671868	2.03146794	0	0	0	0
RP11-543B16.2	0	1.01573397	1.002934331	0	1.027758438	0
RP11-544A12.5	0	1.01573397	0	0	0	0
AL049758.2	0	1.01573397	3.008802992	3.01710065	0	0
RP11-543F8.2	0	0	0	0	0	0
AC112229.6	0	0	0	0	0	0
AC144525.1	0	0	0	0	0	0
AC012593.1	0	0	0	0	0	0
LINC00658	0	0	0	0	0	0
AP000477.3	0	0	0	0	0	0

RP11-291J9.1	0	0	0	0	0	0
AC073325.2	0	0	0	0	0	0
HSPD1P14	0	0	0	0	0	0
NBPF2P	4.95196373	0	6.107870074	7.25109855	0	6.73203231
SNRPEP10	0	0	0	0	0	0
AC108032.1	0	0	0	0	0	0
RP5-956O18.2	0	0	0	0	0	0
LINC01247	0	0	0	0	0	0
RP3-417G15.1	23.2166031	29.1617223	16.61862186	18.3942569	34.57097254	11.65194287
FUNDC2P4	0	0	0	0	0	0
C17orf112	0	0	0	0	0	0
RP1-97J1.2	0	0	0	0	0	0
OR7E96P	0	0	0	0	0	0.906060876
AC007285.6	2.93015605	3.04720192	4.011737323	7.03990151	1.007606311	8.154547886
RP11-548K12.6	0	0	0	0	0	0
RP11-262K1.1	0	0	0	0	0	0
AC007036.6	0	0	0	0	0	0
IL6STP1	0	0	0	0	0	0
OR7E101P	0	0	0	0	0	0
AC007557.3	0	0	0	0	0	0
OR51A3P	0	0	0	0	0	0
SLC8A1-AS1	0	1.01573397	10.02934331	20.1140043	0	11.77879139
RP11-168P8.3	0	0	0	0	0	0
RP11-34E5.4	0	1.01573397	0	0	0	0
AC105461.1	0	0	0	0	0	0
RP11-234N17.1	0	0	1.002934331	0	0	0
RP11-3N2.8	0	0	0	0	0	0
LINC00511	325.247322	314.877531	12.03521197	6.03420129	155.1713719	38.96061768
GTF2IP7	0.97671868	3.04720192	41.12030756	37.210908	3.022818934	38.96061768
ITGB2-AS1	40.045466	53.8339005	1.002934331	0	5.038031556	6.342426133
RP11-533F5.2	0	1.01573397	0	1.00570022	1.017682374	0
RP6-1O2.1	0	0	0	0	0	0
RP11-286M16.1	0	0	0	0	0	0

RP11-98D18.1	0	0	0	0	2.015212623	0
AC012065.4	0	0	0	0	1.007606311	1.812121752
RP11-134G8.2	0	0	0	0	0	0
RP11-460I13.2	0	0	0	0	0	0
C14orf132	0	0	404.1825353	1164.60085	1143.633163	910.5911806
RP11-395B7.4	3.90687474	5.07866986	0	0	4.030425245	0
FDX1P2	0	0	0	0	0	0
AC009961.5	0	0	0	0	0	0
RPL6P2	0	0	0	0	0	0
WDR46	2832.48418	2872.49567	995.9137904	833.725479	1793.539234	1885.512683
RP13-34L8.7	0	0	0	0	0	0
ANHX	0	0	0	0	0	1.812121752
LINC00629	3.90687474	2.03146794	1.002934331	0	1.007606311	4.530304381
AC079779.7	0	0	0	0	0	0
RP11-115A15.4	0	0	0	0	0	0
RPL41P1	4.9714981	0	1.855428512	4.16359889	4.735749663	2.899394804
RP3-363L9.1	0	0	0	0	0	0.906060876
RP3-340N1.2	1.95343737	0	0	0	0	0
DPPA3P1	0	0	0	0	0	0
RP11-12A16.3	0	0	2.005868661	0	0	0
CNN2P2	0	0	0	0	0	0
RP11-191G24.1	0.97671868	2.03146794	2.005868661	2.01140043	2.015212623	0.906060876
FOCAD-AS1	0	0	0	0	0	0
RP3-466I7.1	0	0	0	0	0	0
SDHDP2	6.87609953	1.01573397	4.041825353	1.02581422	1.017682374	1.232242792
AP000472.3	0	0	0	0	0	0
RP11-4C20.4	0	0	0	0	0	0
AC107983.4	2.04134205	0	0	0.51290711	0.534031345	0.625182005
AC004448.2	0	0	0	0	0	0
CTD-2021A8.3	0.97671868	0	1.002934331	0	3.022818934	1.812121752
RP11-543P15.1	50.2619435	44.8751269	92.40033989	105.789606	112.7007659	53.94686457
CH17-437K3.1	5.8603121	6.09440383	1.002934331	2.01140043	0	4.530304381
L29074.3	0	0	2.005868661	1.00570022	0	0.906060876

RBMX2P5	0	0	0	1.00570022	0	0
AC084149.2	0	0	1.002934331	0	0	0
RP1-135L22.1	0	0	0	0	0	0
AP000402.3	0	0	0	0	0	0
RP5-1028L10.1	0	0	0	0	0	0
RP11-565P22.2	0	0	0	0	0	0
HMGB3P8	0	0	0	0	0	0
RPS28P7	0	0	3.129155112	3.10761367	1.06806269	2.518849236
AC073636.1	0	0	0	0	0	0
RP11-433J20.2	0	0	0	0	0	0
OR2AS1P	0	0	0	0	0	0
PIGQP1	0	0	0	0	0	0
PARP1P1	0	0	0	0	0	0
AC096574.5	0	0	0	0	0	1.812121752
IGHD1-14	0	0	0	0	0	0
CRIP1P3	0	0	0	0	1.007606311	1.812121752
LMCD1-AS1	2.93015605	1.01573397	12.03521197	14.079803	11.08366942	11.77879139
RP11-460N20.4	0	0	0	0	1.007606311	0
RP11-267C16.1	0	0	0	0	0	0
RP3-471C18.1	0	0	0	0	0	0
HORMAD2-AS1	0	0	0	0	0	0
BTF3P13	0	0	0	0	0	0
AC009238.7	54.6962463	48.7552307	0	0	0.503803156	0.906060876
RP11-319F12.2	0	0	0	0	1.007606311	0
RPL12P44	0	0	0	0	0	0
ZNF717	164.088739	152.360096	195.5721945	217.231247	5.038031556	204.769758
AP002856.6	0	0	0	0	0	0
LBX1-AS1	0	0	0	0	0	0.906060876
RP11-552E20.1	0	0	0	0	0	0
AC011742.5	0	0	0	0	0	1.812121752
AC093084.1	0	0	0	0	0	0
GCSAML-AS1	0	0	0	0	0	0
RP11-421B23.2	0	0	0	0	0	0

RP11-533N14.3	0	0	0	0	0	0
USP17L5	0	0	0	0	0	0
RP11-545A16.3	0	0	0	1.00570022	10.07606311	4.530304381
LINC01153	0	0	0	0	0	0
IL21-AS1	0	0	0	0	3.022818934	0.906060876
RP11-196D18.1	0	0	0	0	0	0
MTCO1P44	0	0	0	0	0	0
RP11-473E2.3	0	0	0	0	0	0
PRR20D	0	0	0	0	0	0
OR2T7	0	0	0	0	0	0
MKRNP2	0	0	0	0	0	0
RP11-165F24.3	0	0	0	0	0	0
AC068535.2	0	0	0	0	0	0
AC073621.2	0	0	0	0	0	1.812121752
DDX11L16	0	0	26.26685012	13.2852998	0	9.857942333
THEM7P	0	0	0	0	0	0
AC092755.4	0	0	0	0	0	0
RP5-1198O20.5	0	0	0	0	0	0
WDR11-AS1	0.97671868	1.01573397	5.014671654	1.00570022	0	6.342426133
STSP1	0	0	0	0	0	0
RP11-570H19.2	0	0	0	0	0	0
RP1-37J18.1	0	0	0	0	0	0
AF178030.2	0	0	0	0	0	0.906060876
AC011290.4	0	0	0	0	0	0
MYL6P3	0	0	0	0	0	0
RP11-214D15.2	0	0	0	0	0	0
AC092641.2	0	0	0	0	0	0
AIMP1P2	0	0	0	0	0	0
PGCP1	0	0	0	0	0	0
AC013467.1	0	0	0	0	0	0
RP11-317P15.3	0	0	0	0	0	0
VN1R28P	0	0	0	0	0	0
HDGFP1	0	0	1.002934331	0	3.022818934	0

RP11-77G23.5	0	0	0	0	0	0
RP11-6C10.1	0	0	0	0	0	0
MGAT3-AS1	0	0	0	0	0	0
AC092535.3	0	0	0	0	0	0
TRGC2	0	0	0	0	1.511409467	0
RP1-45I4.3	0	1.01573397	6.017605984	1.00570022	0	0
RP11-439A17.4	0	0	0	0	0	0
MIR663AHG	0.97671868	0	0	0	1.007606311	0
IGHD4-23	0	0	0	0	0	0
AC009518.8	0	0	0	0	0	0
C6orf47-AS1	0.97671868	2.03146794	4.011737323	3.01710065	9.068456802	3.624243505
ST7-AS1	43.9523408	26.4090833	157.4606899	103.587122	10.07606311	67.95456571
RP11-121A14.3	1.95343737	1.01573397	1.002934331	0	0	1.812121752
CNN2P1	0	0	0	0	0	0
SUB1P1	1.95343737	0	0	0	1.007606311	1.812121752
RBMY2JP	0	0	0	0	0	0
PFN1P9	0	0	0	0	0	0
XXbac-BCX196D17.5	0	0	0	0	0	2.718182629
RPL31P12	0	0	0	0	0	0
WARS2P1	0	0	0	0	0	0
AC079145.4	0	1.01573397	0	2.01140043	1.007606311	1.812121752
RP5-1100E15.4	0	0	0	0	0	0
PFN1P6	0	0	0	1.00570022	0	0.906060876
SPATA13-AS1	0	0	0	0	0	0
HCG15	29.3015605	28.4405512	10.02934331	15.0855032	13.09888205	8.154547886
RP11-445L13__B.3	0	0	0	0	0	0
RP11-367J7.3	0	0	0	0	0	0
RP11-203J24.8	0	0	0	1.00570022	10.07606311	0.906060876
RP11-69I8.3	10.7439055	10.1573397	0	0	0	0.906060876
RP11-72H11.1	0	0	0	0	0	0
MTND1P14	0	0	0	0	0	0
AC017101.10	0	0	0	1.00570022	1.007606311	0
RP11-261C10.5	1.95343737	1.01573397	0	0	0	0

WASH7P	253.927323	157.621598	423.9403416	403.366242	160.6628263	370.9232015
CICP17	0	0	0	0	0	0
SPANXB1	288.229684	350.966559	0	0	0	0.906060876
RP11-365D9.1	0	0	0	0	0	0
AL672294.1	0	0	0	0	0	0
TTC39DP	0	0	0	0	0	0
RP11-563D10.1	0	0	0	0	0	0
AC011998.4	0	0	0	0	0	0
NBPF13P	0	0	3.008802992	0	0	1.821182361
SLAMF6P1	0	0	0	0	0	0
LINC00845	0	0	0	0	0	0.906060876
RP11-136C24.2	0	0	0	0	1.007606311	0
TRIM60P13	0	0	0	0	0	0
FAM155A-IT1	0	0	0	0	0	0
AC000374.1	0	0	0	0	0	0
TRIM60P9Y	0	0	0	0	0	0
AC105760.2	13.6740616	14.2202756	11.03227764	13.0741028	8.06085049	16.30909577
RP11-166N17.1	0	0	0	0	0	0.906060876
VDAC1P12	0	0	0	0	0	0
CDRT15P2	0	0	0	0	0	0
MIS18A-AS1	0	0	3.008802992	0	0	2.718182629
RP11-330M2.3	0	0	0	0	0	0
SMIM2-AS1	1.95343737	7.11013781	13.0381463	30.1710065	2.015212623	2.718182629
HMGN2P22	0	0	0	0	0	0
AC116035.1	0	1.01573397	0	0	0	0
YWHAZP7	0	0	0	0	0	0
HCG4B	7.81374947	7.11013781	2.005868661	0	0	0
ACTR3BP5	0	0	0	0	0	0
AC073464.4	0	0	0	0	0	0
RP11-305O4.2	0.97671868	0	0	1.00570022	2.015212623	2.718182629
KLLN	23.4412484	28.4405512	13.0381463	31.1767067	110.8366942	46.20910469
RP11-96L7.2	0	0	0	0	0	0
AC009963.4	0	0	0	0	0	0

RPL39P25	0	0	0	0	0	0
AC005358.3	0	0	0	0	0	0
MTND6P10	0	0	0	0	0	0
RP11-656D10.5	0	0	0	0	2.015212623	0
AC015933.2	1.95343737	0	0	0	0	0
RP11-164D18.2	0	0	0	0	0	0
RPL35AP12	0	0	0	0	0	0
MARK2P10	0	0	0	0	0	0
RP5-837I24.1	0	0	0	0	0	0
HSFY3P	0	0	0	0	0	0
LINC01364	0	0	0	0	0	0
AC092170.1	0	0	0	0	0	0
AC009229.5	0	0	0	0	0	0
AC118345.1	0	0	0	0	0	0
AC016994.2	0	0	0	0	0	0
ELL2P1	1.95343737	2.05178262	0	3.02715765	2.015212623	3.651425331
RP4-718J7.4	0	0	0	0	0	0
ARAFP3	0	0	0	0	0	0
KRT16P2	0	0	0	0	0	0
RP11-384P7.5	0	0	0	0	0	0
SSXP9	0	0	0	0	0	0
GS1-256O22.5	0	0	0	0	0	0
RP11-791G16.2	0	0	0	0	12.12150392	0.906060876
RP11-340I6.3	0	0	0	0	0	0
AP006285.6	0	0	0	0	0	0
RP11-95I16.2	0	0	0	0	0	0
AC009502.4	1.95343737	1.01573397	0	0	0	5.436365257
AC140076.1	0	0	0	0	0	0
RP1-228H13.1	0	0	0	0	1.007606311	0.906060876
RP11-496D1.2	0	0	0	0	0	0
RP11-309N24.1	0	0	0	0	0	0
MTND4P15	0	0	0	0	0	0
RP11-258C19.4	0	0	0	0	0	0

AP000998.2	0	0	0	0	0	0
AC005042.2	4.88359342	2.03146794	3.008802992	1.00570022	4.030425245	3.624243505
IGHJ1P	0	0	0	0	0	0
LINC00434	0	0	0	0	0	0
RPL23AP43	0.97671868	0	0	0	0	0.915121485
RP11-195B3.1	0	0	0	0	0	0
THRAP3P1	0	1.01573397	0	0	0	0
LINC00307	0	0	0	0	0	0
RPL15P1	0	0	0	0	0	0
HAUS6P1	3.13526698	3.24019137	3.239477888	2.21254047	4.373011391	1.993333928
PARG	1431.62541	1377.76187	900.665117	711.975411	1034.539628	953.402557
HNRNPKP2	0	5.07866986	0	2.3332245	4.030425245	2.718182629
MTND5P25	0	0	0	0	0	0
CEACAMP7	0	0	0	0	0	0
RP11-438P9.2	0	0	0	0	0	0
NANOGP6	0	0	0	0	0	0
ARHGEF7-AS1	0	0	0	0	0	0
RAB28P3	0	0	0	0	0	0
RBM26-AS1	29.3015605	38.5978909	9.026408976	11.0627024	29.22058303	13.59091314
RP11-162D16.2	0	0	0	0	0	0
LINC00866	0	0	0	0	0	0
RP11-180I4.1	0	0	0	0	0	0.906060876
AC017074.2	9.76718684	7.11013781	0	0	2.015212623	6.342426133
RP11-518I13.1	0	0	0	0	0	0
RPS24P7	0	0	0	0	0	0
AC019172.2	0	0	0	0	0	0
AC018685.2	0	0	0	0	0	0
AC008060.5	0	0	0	0	0	0
SLC9B1P4	0	0	0	0	0	0
AC079753.5	0	0	1.002934331	0	0	0
RP4-669P10.19	0	0	13.0381463	24.1368052	3.022818934	9.060608762
RP11-3L10.2	0	0	0	0	0	0
TP73-AS1	387.757317	379.884506	1003.937265	873.953487	19.14451991	1088.179112

RP11-160H22.5	1.95343737	0	0	0	0	0.906060876
RP11-109A6.3	0	0	0	0	0	0
DLG1-AS1	6.83703079	11.1730737	8.023474646	12.0684026	3.022818934	7.248487009
FTH1P16	0.97671868	0	0	1.20684026	3.577002405	4.97427421
PPIAP2	0	2.03146794	2.005868661	1.00570022	6.045637868	0
EIF4A2P2	1.95343737	0	3.008802992	1.00570022	2.015212623	0.906060876
RP11-571F15.2	0	2.03146794	5.014671654	0	0	2.718182629
AC091705.1	0	0	0	0	0	0
RP11-112J3.16	3.90687474	6.09440383	17.04988362	20.1140043	11.08366942	17.21515665
SALL1P1	0	0	0	0	0	0
HPN-AS1	0	0	0	0	0	0
RP6-127F18.2	0	0	0	0	0	0
AC007386.3	0	0	0	0	2.015212623	0
EIF2AP4	0	0	0	0	0	0
CTD-2526L21.3	0	0	0	0	0	0
KIF9-AS1	23.4412484	31.4877531	80.23474646	127.723927	30.22818934	132.2848879
RP11-118F2.2	0	0	0	0	0	0
AC012501.2	0.97671868	2.03146794	3.500240814	0.10057002	1.007606311	1.812121752
RPL37P1	0	0	0	0	0	0
AC009299.3	259.80717	227.52441	0	1.00570022	99.75302482	9.060608762
KRT8P20	0	0	0	0	0	0
RNF6P1	0	0	0	0	0	0
AC008746.3	0.97671868	2.03146794	2.005868661	4.02280086	0	3.624243505
AMYP1	0	0	1.033022361	0	0	0
ZMYM4-AS1	0	0	0	0	0	0
BAATP1	0	0	0	0	0	0
STK33P1	0	0	0	0	0	0
RP5-1042K10.12	0	0	0	0	0	0
RP11-109P14.2	0	0	0	0	0	0
RP11-397P13.6	0	0	0	0	0	0
PCGEM1	0	0	0	0	2.035364749	0
RP11-476B1.1	0	0	0	0	0	0
OR10U1P	0	0	0	0	0	0

MRPS21P2	0	0	0	0	0	0
VN1R33P	0	0	0	0	0	0
RP1-49C23.1	0	0	0	0	0	0
AC019185.2	0	0	0	0	0	0
CSE1L-AS1	0	0	0	0	2.015212623	0
AC053503.11	0	0	0	0	3.022818934	0
AC069213.4	0	0	0	0	0	0
RNF19BPX	0	0	0	0	0	0
FCF1P1	0	0	1.002934331	5.02850108	0	3.624243505
RP11-179H18.5	0	0	0	0	0	0
AP001471.1	0	0	0	0	0	0
TTTY17B	0	0	0	0	0	0
ATP5G1P4	0	0	0	0	0	0.906060876
RP11-344A7.1	0	0	0	0	0	0
AC007322.5	0	0	0	0	0	0
OR10R1P	0	0	0	0	0	0
XGY1	0	0	0	0	0	0
RP11-111F5.5	7.46213074	6.4702254	125.7880238	63.5803676	46.42042276	61.70274567
HNRNPA1P63	0	0	0	0	0	0
MTND4P30	0	0	0	0	0	0
RP11-300M24.1	0	0	0	0	0	0
LINC00310	11.7206242	1.01573397	6.017605984	4.02280086	4.030425245	1.812121752
AC079612.2	0	0	0	0	0	0
RP11-108B14.4	0	0	0	0	0	0
RP11-158D2.2	0.97671868	0	1.002934331	0	0	0
GPN3P1	0	0	0	0	0	0
RP11-397C12.1	0	0	0	0	0	0
LINC01537	6.83703079	18.2832115	0	0	1.007606311	0
AL928742.12	0	0	0	0	1.007606311	0.906060876
AC073415.2	1.95343737	2.03146794	0	0	0	0
AKR1B15	0	2.03146794	0	0	0	7.248487009
TSSK5P	0	0	0	0	1.007606311	0
RP11-295P9.2	0	0	0	0	1.007606311	0

RP11-810B23.1	0	0	0	0	0	0
STK4-AS1	11.7206242	12.1888077	3.008802992	26.1482056	17.12930729	9.966669638
AC124861.1	0	0	0	0	0	0
CCDC148-AS1	0	0	0	0	0	0
RP11-89N17.2	0	0	0	0	0	0
RP11-18B16.2	0.97671868	3.04720192	0	0	3.022818934	0
XX-C283C717.1	0	0	0	0	0	0
RP11-5P4.2	0	0	0	0	0	0
RP13-188A5.1	0	0	23.06748961	21.1197045	0	13.59091314
NCAM1-AS1	0	0	6.017605984	6.03420129	0	10.87273051
GAGE12D	0	0	0	0	0	0
AC006458.3	0	0	0	0	0	0
CCNJP1	0	0	0	0	0	0
RP11-316M21.6	0.97671868	0	0	0	1.007606311	0.906060876
RP11-104D21.1	0	0	0	0	0	0
USP9YP14	0	0	0	0	0	0
AC109333.10	0.97671868	2.03146794	0	1.00570022	1.007606311	0.906060876
RP11-145A3.1	1.95343737	2.03146794	0	0	0	0.906060876
PABPC1P6	0	0	0	0	0	0
AC018359.3	0	0	0	0	0	0
RP11-419M24.4	0	0	0	0	0	0
SCAMP4	3036.45235	3147.75958	2452.174439	1561.80215	1712.930729	2094.812746
LINC01268	0	0	0	0	0	0
RP11-434J24.2	0	0	0	0	0	0
LTB	327.200759	281.35831	0	5.02850108	62.4715913	23.55758278
LINC01624	0	0	0	0	0	0
EIF2B5-IT1	0	0	0	0	0	0
LINC01442	0	0	0	0	0	0
GS1-466O4.2	0	0	0	0	0	0
RP11-413M3.4	0	0	0	0	1.007606311	0
AC114755.3	0	0	0	0	0	0
RP11-709P2.1	0	0	0	0	0	0
C1DP4	0	0	0	0	0	0

RP5-973N23.4	0	0	0	0	0	0
LINC01483	0	0	0	0	0	0
XXyac-YRM2039.3	1.39670772	0	3.530328844	5.51123718	3.294872638	5.545092562
CTA-342B11.1	0	0	0	0	0	0
RP13-213K19.1	0	0	0	0	0	0
RPS20P15	0	0	0	0	0	0
RPL7P6	0	0	0	0	0	0
RP11-223A3.1	0	0	0	1.00570022	0	0
DIAPH3-AS1	0	0	0	0	0	0
RP11-202G18.1	0	0	0	0	0	0
AC002542.2	0	0	0	0	0	0
SLC2A1-AS1	8.79046815	2.03146794	15.04401496	13.0741028	19.14451991	11.77879139
RP3-323B6.1	0	0	0	0	0	0
RP11-809N15.2	0	0	0	0	0	0
SOCS5P4	0	2.03146794	17.04988362	10.0570022	4.030425245	9.966669638
RP11-215C7.3	0	0	0	0	0	0
HNRNPFP1	0	2.03146794	0	0	0	0
RP11-152N13.5	32.2317166	18.2832115	16.04694929	23.131105	28.21297672	11.77879139
SFR1P1	0	0	0	0	0	1.812121752
AC092614.2	14.6507803	21.3304134	0	0	11.08366942	0.906060876
SPAG5-AS1	5.8603121	3.04720192	4.011737323	6.03420129	9.068456802	13.59091314
AC018647.3	0	0	0	0	0	0
RP5-905H7.10	0	0	0	0	0	0
OR4A2P	0	0	0	0	0	0
AC116035.2	0	0	0	0	0	0
TRBV7-5	0	0	0	0	0	0
USP17L12	0	0	0	0	0	0
RP11-444D13.1	0	0	0	0	0	0
MIR4290HG	0	0	0	0	0	0
RP11-472F19.1	0	0	0	0	0	0
MTND3P9	0	0	0	0	0	0
RP11-139K1.2	0	2.03146794	1.002934331	1.00570022	1.007606311	0
RP11-796I2.1	0	0	0	0	0	0

RNF11B	0	0	0	0	0	0
LINC00376	0	0	0	0	0	0
SNX18P26	1.95343737	0	1.002934331	0	0	0
RP5-1166A24.1	0	0	0	0	0	0
RP13-1039J1.3	0	0	0	0	0	0
RPS3AP53	0	0	0	0	0	0
RP1-35C21.2	0	0	0	0	2.015212623	0
RP13-140E4.1	0	1.01573397	0	0	0	0
ADGRF5P1	5.65520118	2.06193996	2.005868661	9.05130194	6.045637868	1.812121752
RP11-119F7.3	0.97671868	0	0	0	0	0
MTND4P5	0	0	0	0	0	0
RP11-103C3.2	0	0	0	0	0	0
RP11-162A23.5	0	0	0	2.02145743	0	0
CNTN4-AS2	0	0	0	0	0	0
RP5-1092A11.5	0	0	0	1.00570022	0	0.906060876
ATP5G1P5	0	0	0	0	0	0
RP1-28O10.1	3.90687474	2.03146794	0	0	1.007606311	1.812121752
PIGFP3	0	0	0	0	0	0
PHBP16	0	0	0	0	0	0
WASF1P1	0	0	0	0	0	0
RP1-167A14.2	0.97671868	5.07866986	2.005868661	1.00570022	1.007606311	1.812121752
RP4-753D4.2	0	0	0	0	0	0
RP3-445N2.1	0	0	0	0	0	0
RP11-165J3.6	2.93015605	1.01573397	7.020540315	11.0627024	5.038031556	13.59091314
TOMM22P3	0	0	0	0	0	0
AC005392.13	0	0	0	0	0	0
SUMO2P2	0	0	0	0	0	0
TMEM183AP1	0	0	0	0	0	0
FRMPD3-AS1	0	0	0	0	0	0
LINC01074	0	0	0	0	0	0
QRSL1P2	0	0	0	0	0	0
RP11-864N7.2	45.9155453	41.6857222	62.25213391	56.3896111	60.49668293	38.12704167
AC063976.1	0	0	0	0	0	0

CERS6-AS1	1.95343737	0	20.05868661	32.1824069	6.045637868	10.87273051
RP11-492E3.2	3.90687474	7.11013781	0	0	10.07606311	8.154547886
ALG1L8P	0	0	0	0	0	0
PHBP11	0	0	0	0	0	0
AC073987.2	0	0	0	0	0	0
SNRPEP3	0	0	0	0	0	0
RP11-520H14.1	0	0	0	0	0	0
RP1-101K10.6	8.41931505	12.4630558	15.42513001	13.798207	10.57986627	22.0263399
SLC25A15P1	0	0	0	0	0	0
LINC01132	0.97671868	0	29.08509559	16.0912034	4.030425245	43.49092206
AC018804.6	0	0	0	0	4.634989032	0
RBMY2YP	0	0	0	0	0	0
RP11-431K24.3	1.95343737	1.01573397	0	0	0	0
USP9YP21	0	0	0	0	0	0
HNRNPA1P14	0	0	0	0	0	0
SOX21-AS1	0	0	0	0	0	0
HIGD1AP11	0	0	0	0	0	0
RPL7P54	0	0	0	0	0	0
STEAP2-AS1	0	0	0	0	0	0
RP11-522L3.9	0	0	0	0	0	0
ISCA1P6	0	0	0	0	0	0
SERPINA7P1	0	0	0	0	0	0
AC005160.3	0	0	0	0	0	0
CLYBL-AS2	0	0	0	0	0	0
UST-AS1	0	0	0	0	0	0
MTCO3P18	0	0	0	0	0	0
RPL7P2	0	0	0	0	0	0
CYCSP24	3.90687474	4.06293589	4.011737323	1.00570022	1.007606311	0
RP11-331H2.3	0	0	0	0	0	0
RP11-488L18.4	778.376421	679.526027	1670.718096	1406.85392	571.23217	1611.637662
RP11-243J18.2	0	0	0	0	0	0
LINC00355	7.81374947	8.12587178	0	0	0	0
LINC01068	0	0	0	0	0	0

RP11-73O6.3	0	0	0	0	0	0
RP11-453N3.1	0	0	0	0	0	0
AC108051.2	0	0	0	0	0	0
RP11-307P5.1	3.90687474	3.04720192	0	0	0	0
ATP5A1P2	0	2.03146794	0	0	0	0
RP11-445N18.5	0	0	0	0	0	0
CROCCP4	0	0	1.002934331	0	2.015212623	2.718182629
AC087499.10	0	0	0	0	0	0
RP4-681L3.2	0	0	0	0	0	0
HNRNPA3P2	1.95343737	0	2.005868661	2.01140043	0	5.436365257
SRP68P2	0	0	0	0	0	0
MED28P3	0	0	0	0	0	0
GSTM3P1	0	0	0	0	0	0
RPL23AP74	1.96320455	2.03146794	3.018832335	6.0543153	3.022818934	6.378668568
DNMBP-AS1	8.79046815	12.1888077	0	1.00570022	1.007606311	0
AP001619.2	0.97671868	0	0	0	0	0.906060876
RP11-301M17.1	1.95343737	0	0	0	0	0
LINC00111	0	0	0	0	0	0
RP1-266L20.4	0	0	0	0	0	0
RP11-15M15.2	0	0	0	0	0	0
RP11-301G19.1	0.97671868	0	0	0	0	0.906060876
SDAD1P3	0	0	0	0	0	0
AC079150.3	0	0	0	0	0	0
RP11-118D22.3	0	0	0	0	0	0
LL22NC03-30E12.13	0	0	0	0	0	0
FAM166AP4	0	0	0	0	0	0
RP11-418J17.3	0	0	0	0	0	0
AC116609.1	0	0	0	0	0	0
MTND6P18	0	0	0	0	0	0
AP000459.7	0	0	0	0	0	0
RP11-578F21.4	0	0	0	0	0	0
AC016730.1	0	0	0	0	0	0
AC006042.6	0	0	1.002934331	0	0	0

RPSAP64	0	0	0	0	0	0
RP5-1059H15.1	0	0	0	0	0	0
CTA-31J9.2	0	0	0	0	0	0
GCOM2	0	0	4.262470906	1.06604223	0	2.845031151
AP001271.3	0	0	2.005868661	2.01140043	3.022818934	5.436365257
RP11-690C23.4	0	0	0	0	0	0
RD3L	0	0	0	0	0	0
MTND6P5	0.97671868	0	0	1.00570022	0	0
RP4-565E6.1	0	0	0	0	2.015212623	0
RP11-49L2.1	0	0	0	0	0	0
CYCSP5	0	0	0	0	0	0
MTCO3P16	0	1.01573397	0	0	0	0
OR8B1P	0	0	0	0	0	0
AC092646.2	0	0	0	0	0	0
RP11-318C24.2	0.97671868	1.01573397	1.002934331	1.00570022	6.045637868	6.342426133
RP11-536C5.7	2.93015605	4.06293589	3.008802992	2.01140043	1.007606311	1.812121752
CALR4P	0.97671868	0	0	0	3.022818934	0
RP4-745K6.1	0	0	0	0	0	0
FLJ43879	0	0	0	0	0	0
AC098826.5	0.97671868	2.03146794	0	0	0	0
RP11-16L9.1	0	0	0	0	0	0
RP11-497D6.3	0	0	0	0	0	0
RP1-20B21.4	0.97671868	1.01573397	3.008802992	4.02280086	3.022818934	1.812121752
RP11-216N21.1	0	0	0	0	0	0
AP000344.4	0	0	54.59974496	159.906334	5.038031556	34.43031329
AP000282.2	0	0	0	0	0	0
HCG9P5	0	0	0	0	0	0
VTI1BP4	0	0	0	0	0	0
RP11-298E10.1	0	0	0	0	0	0
GUSBP8	0	0	0	0	0	0
RP11-354K1.1	2.93015605	2.03146794	0	0	0	0
MYL12BP2	0	0	0	0	0	2.718182629
HCG4P5	93.7649937	85.3216537	10.02934331	7.03990151	12.09127574	7.248487009

AC072062.3	1.95343737	0	0	0	0	0
RPL7P12	0	0	0	0	0	0
ASH1L-IT1	0	0	1.002934331	2.01140043	0	1.812121752
RP1-283E3.4	5.8603121	2.122884	5.014671654	5.02850108	4.030425245	2.718182629
AKR1D1P1	0	0	0	0	0	0
RP4-738P11.3	0	0	0	0	1.007606311	0
GS1-164F24.1	0	0	0	0	0	0
CTC-529I10.2	1.95343737	4.06293589	6.017605984	1.00570022	9.068456802	5.436365257
ZNF965P	0	0	0	0	0	0
AC092013.1	0	0	0	0	0	0
AC012363.8	0	0	0	0	0	0
MTCYBP7	0	0	0	0	0	0
AC015922.5	1.95343737	0	0	0	0	0
AC007365.4	0	0	2.005868661	0	0	0
MTND4LP9	0	0	0	0	0	0
AC012358.4	3.90687474	1.01573397	2.005868661	5.02850108	24.18255147	3.624243505
IGHD4-17	0	0	0	0	0	0
DNAJB3	0	0	0	0	0	0
RP11-239H6.2	0	0	0	0	0	0
RP11-248J23.5	0	0	0	0	0	0
OR5W1P	0	0	0	0	0	0
RP11-171A24.2	0	0	0	0	0	0
FAM212B-AS1	6.83703079	7.11013781	51.14965087	123.701127	4.030425245	36.24243505
RP1-180M12.1	0	0	0	0	0	0
MRPS17P9	0	0	0	0	0	0
RP11-195C7.3	0	0	0	0	0	0
ARHGAP42P1	0	0	1.113257107	2.01140043	0	0
RP11-227F8.2	0	0	0	0	0	0
AC122136.2	0	0	0	0	0	0
SLC9A7P1	13.6740616	11.1730737	0	1.00570022	27.2053704	0.906060876
RP11-958N24.2	2.8910873	9.46664062	30.70984921	11.1632724	10.77131147	25.65964401
TRIM60P3Y	0	0	0	0	0	0
ST13P21	0	2.03146794	0	0	0	0

AP003385.2	0	0	0	0	0	0
CARM1P1	0	0	0	0	0	0
AC008850.3	0	0	1.002934331	0	0	0.906060876
TRIM60P11Y	0	0	0	0	0	0
RP1-213J1P__B.1	0	0	0	0	0	0
KRTAP8-3P	0	0	0	0	0	0
MTND5P31	0	0	0	0	0	0
AC093166.2	0	0	0	0	0	0
RP11-184B22.2	0	0	0	0	0	0
UGT1A11P	0	0	0	0	0	0
RP11-420H19.2	0	0	0	0	0	0
SUCLA2-AS1	0	0	1.002934331	1.00570022	0	0
SEPT2P1	0	0	0	0	0	0
RP11-381K7.1	0	0	0	0	0	0
RP11-156G14.6	0	0	0	0	0	0
RP11-307O1.1	0	0	0	0	0	0
DPY19L2P3	17.5809363	15.2360096	2.005868661	7.03990151	6.045637868	4.530304381
RP4-533D7.5	0	3.04720192	3.008802992	3.01710065	5.038031556	1.812121752
MTND5P18	0	0	0	0	0	0
HNRNPA1P31	0	0	0	0	0	0
AC002383.2	0	0	0	0	0	0
ARL5AP1	0	0	0	0	0	0
TCEB1P11	0	0	0	0	0	0
C1orf234	0	0	0	0	1.007606311	0
RP11-807H17.1	0	0	0	0	0	0
USP9YP12	0	0	0	0	0	0
RP3-389A20.2	0	0	0	0	0	0
GRAMD4P1	0	0	0	1.00570022	0	0
RPL23AP89	0	0	0	0	0	0
MRLN	0	0	1.002934331	0	0	0.906060876
AC114760.1	0	0	0	0	0	0
PSPC1P1	0	0	2.51736517	2.38350951	0	1.069151834
RP11-46E17.6	0	0	0	0	0	0

ASS1P5	0	0	0	0	0	0
PRKRIRP6	0	0	1.002934331	0	0	0
ATP6V0E1P4	0	0	0	0	0	0
RP11-79N23.1	3.90687474	5.07866986	16.04694929	8.04560172	1.007606311	10.87273051
RPS26P13	0	1.01573397	1.002934331	1.00570022	3.022818934	0.906060876
FAM66A	0	1.17825141	2.116191438	7.77406267	1.098290879	4.530304381
PSMA2P3	0	0	0	0	0	0
OR5P4P	0	0	0	0	0	0
LINC00352	0	0	0	0	0	0
ISX-AS1	0	0	0	0	0	0
RP11-77P6.2	27.3481231	29.4562852	35.10270157	53.3021114	33.25100827	58.89395695
AC062032.1	0	0	0	0	0	0
MED6P1	0	0	0	0	0	0
SNAP25-AS1	4.88359342	2.03146794	0	3.01710065	4.030425245	1.812121752
RP11-102C16.3	0	0	0	0	0	0
FLJ31104	13.6740616	26.4090833	0	2.01140043	4.030425245	4.530304381
RP11-73B2.6	0.97671868	0	0	0	17.12930729	0
RP11-141M1.1	0	0	0	0	0	0
RP11-69C17.3	0	0	0	0	0	0
KRT8P44	0	0	0	0	0	0
RP11-130C19.3	0	0	0	0	0	0
TCEB1P16	0	0	0	0	0	0
RP11-143M1.3	0	0	1.002934331	0	0	0
AL353997.11	0	0	0	0	0	0
RP1-153P14.5	0	0	0	0	0	0.906060876
SPTLC1P5	0	0	0	0	0	0
RP5-905H7.7	0	0	0	0	0	0
RBPJP6	0	0	0	2.01140043	0	0
RP11-191N8.2	0	0	0	0	0	0
MACROD2-IT1	0	0	1.002934331	0	0	0
SRGAP3-AS3	0	0	0	0	0	0
AC009508.1	0	0	0	0	0	0
RP13-16H11.2	0	0	0	0	0	0

RP11-331F9.4	0	0	0	0	0	0
RP11-423F24.3	0	0	0	0	0	1.812121752
RP6-102O10.1	0	0	0	0	0	0
MTND2P30	0	0	0	0	0	0
AC104695.4	0	2.03146794	0	0	0	0
RPL3P2	2.93992324	5.10914188	17.57140947	12.1488586	10.09621524	5.436365257
RP11-372M18.1	0	0	0	0	0	0
UQCRBP2	0	0	0	0	0	0.906060876
FRMD8P1	0	0	0	0	0	0
RP11-103C16.2	0	0	0	0	0	0
AC007383.3	18.557655	30.4720192	40.11737323	61.3477131	45.34228401	53.45759169
RP11-543D5.1	0	0	0	0	0	0
AC003989.4	0	0	0	0	0	0
CYCSP46	0	0	0	0	0	0
RP11-312B8.2	0	0	0	0	0	0
RPL18AP17	0	0	0	0	0	0
LINC01341	12.6973429	16.2517436	41.12030756	15.0855032	29.22058303	63.42426133
TARID	3.90687474	2.03146794	0	0	0	0.906060876
RP11-399H11.3	0	0	0	0	0	0
RP11-276H7.2	21.487811	13.2045416	2.005868661	3.01710065	4.030425245	1.812121752
RP5-837I24.6	0	0	0	0	0	0
RP11-382D8.3	0.97671868	0	0	3.01710065	0	2.718182629
RP5-1074L1.1	6.83703079	7.11013781	8.023474646	9.05130194	1.007606311	3.624243505
LINC01429	0	1.01573397	0	0	4.030425245	0
AC098592.6	0	0	0	0	0	0
BUB3P1	0	0	0	0	0	0
NPM1P22	0	0	0	0	0	0
NR1H5P	0	0	0	0	0	0
RP11-600G3.1	0	0	0	0	0	0
PRKRIRP3	0	0	0	0	0	0
PIN4P1	2.97899199	1.02589131	0	0	0	0
AC002127.2	0	0	0	0	0	0
AC012668.3	0	0	0	0	0	0

RPL7P30	0	0	0	0	0	0
BRK1P2	0	0	0	0	0	0
TRIM60P18	7.81374947	8.12587178	36.10563591	38.2166082	15.11409467	19.0272784
AC092675.4	0	0	0	0	0	0
TDGF1P1	0	0	0	0	0	0
AC010141.8	0	0	0	0	0	0
AC108463.2	14.6507803	4.06293589	1.002934331	3.01710065	0	0.906060876
RP5-891H21.1	0	0	0	0	0	0
RAB11AP1	0	0	0	0	0	0
MTND5P1	0	0	0	0	0	0
RPL7AP65	0.97671868	0	0	0	0	0
DHX9P1	0	1.01573397	0	0	3.022818934	0
MYCLP2	0	0	0	0	0	0
AC020743.2	0	0	0	0	0	0
AC011933.2	0	0	0	0	0	0
CTD-2330K9.3	0	0	8.023474646	3.01710065	0	0
AC073343.13	0	0	5.014671654	8.04560172	7.053244179	3.624243505
RP11-350G8.5	0	0	0	1.00570022	0	0
ZNF680P1	0	0	0	0	0	0
AC074338.5	0	0	0	0	0	0
RAPGEF4-AS1	0	0	0	0	0	0
RP11-166O4.4	0	0	0	0	0	0
HNRNPA1P46	0	0	0	0	1.007606311	0
RP11-383C5.3	0	0	0	0	0	0
HCG20	4.88359342	8.12587178	1.002934331	0	0	0
CYP1D1P	0	0	0	0	0	0
RP1-251M9.3	0	0	0	0	0	0
AC069257.8	0	0	0	0	0	0.906060876
AC019084.7	0	0	0	0	0	0
AC078842.3	0	0	0	0	0	0
AC010967.2	0	0	0	0	0	0
HMGN2P21	0	0	0	0	0	0
RP4-663N10.1	0	0	0	0	0	0

HSPD1P9	0	0	1.002934331	1.00570022	0	4.530304381
RP3-395M20.9	0	0	0	0	0	0
VN1R51P	0	0	3.008802992	1.00570022	0	0
KB-1125A3.10	0	0	0	0	0	0
RP3-522D1.2	0	0	0	0	0	0
RP4-581F12.1	0	0	0	0	0	0
RP11-441A11.1	0	0	0	0	0	0
AC097721.2	6.83703079	8.12587178	7.020540315	10.0570022	13.09888205	9.060608762
RP4-781K5.4	5.8603121	0	0	0	0	0.906060876
DNAJA1P6	0	0	0	0	0	0
RP11-15B24.2	0	0	0	0	0	0
RP11-598C10.1	0	0	0	0	0	0
POLR2J2	64.6880784	58.2523433	50.48771421	52.2662402	126.0616256	92.10108806
TOP3BP1	0	0	1.504401496	0	6.710658033	5.164546994
RP11-325E14.2	0	0	0	0	0	0
RP11-151F5.2	1.99250612	0	0	1.01575722	0	0
RANP5	0	0	0	0	0	0
LINC00864	0	0	0	0	0	0
CFL1P3	0	0	0	0	0	0
SEC63P1	3.97524504	2.04162528	6.047694014	3.08749966	2.045440812	0.915121485
RP11-552D4.1	0	0	0	0	0	0.906060876
RP11-69E11.8	0	0	0	0	1.007606311	0
Z83001.1	0	0	0	0	0	0
LYPLAL1-AS1	0	0	3.008802992	2.01140043	0	0
AC023469.2	0	0	0	0	0	0
LINC01515	0	1.01573397	35.10270157	40.2280086	69.52483548	55.26971345
RP11-61J19.3	0	0	0	0	0	0
RP11-100G15.3	0	0	0	0	0	0
RPL7P47	0	0	0	0	0	0
RP11-255A11.2	0	0	0	0	0	0
AC133106.2	0	0	0	0	0	0
UBBP5	0	0	0	0	0	0
BOD1L2	0	0	0	0	0	0

RP11-475E11.2	0	0	0	0	0	0
HLA-U	0	1.01573397	0	0	0	0
AC012368.1	0	1.01573397	0	0	1.007606311	0
RP11-385M4.2	0	0	0	0	0	0
RP1-121G13.2	0	0	0	0	0	0
IFNA14	0	0	0	0	0	0
RP5-884G6.2	2.93015605	0	5.014671654	4.02280086	13.09888205	8.154547886
AC004920.2	0	0	0	0	0	0
RP5-837M10.4	0	0	0	0	0	0
PNKDP1	0	0	0	0	0	0
COX6CP15	0	0	0	0	0	0
RP11-361M4.1	0	0	0	0	0	0
MTATP6P11	0	0	1.002934331	1.00570022	0	0
AC110620.1	0	0	0	0	0	0
AC016912.3	0	0	2.005868661	1.00570022	0	0
RP3-340N1.6	0	0	0	0	0	0
RP11-452F19.3	90.8348376	128.998214	132.3873317	210.191345	34.25861458	100.5727573
AP000692.9	4.19989034	3.04720192	8.254149542	6.72813444	6.237083067	10.45594251
AC092839.3	0	0	0	0	0	0
MFI2-AS1	34.1851539	27.4248173	70.20540315	102.581422	45.34228401	66.14244396
ST13P19	3.3403779	1.17825141	7.080716375	6.57727941	1.007606311	5.436365257
TPT1P2	0	0	0	0	0	0
AC112220.2	0	0	0	0	0	0
AC003991.3	0.97671868	2.03146794	0	0	0	1.812121752
RP11-165F24.5	0	0	0	1.00570022	0	0
MYL6P5	0	0	0	0	0	0
AP001631.10	0	1.52360096	0	0	0	0.906060876
PHBP3	0	0	0	0	0	0.742969918
MTND1P17	0	0	0	0	0	0
RP11-15B24.3	0	0	0	0	0	0
RP1-122O8.7	0.97671868	0	0	0	0	0
AKIRIN1P2	0	0	0	0	0	0
FALEC	0.97671868	2.03146794	8.023474646	8.04560172	6.045637868	3.624243505

RP11-12L8.1	0	0	0	0	0	0
IGHD6-6	0	0	0	0	0	0
RP11-279E1.1	0	0	0	0	0	0
AC099684.1	0	0	0	3.01710065	0	1.812121752
AC092578.1	0	0	0	0	0	0
LINC01494	0	0	0	0	0	0
RP11-216M21.5	0	0	0	0	0	0
AP001469.7	4.88359342	3.04720192	1.002934331	5.02850108	10.07606311	7.248487009
LINC01402	0	0	0	0	0	0
RP3-467K16.4	7.81374947	13.2045416	0	5.02850108	0	3.624243505
AC105339.1	0	0	0	0	2.015212623	0
RP11-180I4.2	0	0	0	0	0	0
RP11-308N19.5	0	0	0	0	0	0
RP11-745O10.4	0	0	0	0	0	5.699122911
CASP16P	0	0	1.002934331	1.00570022	0	1.812121752
RPL3P1	0	1.01573397	2.005868661	0	0	0
RP11-84A14.4	0	1.01573397	1.002934331	0	0	0
AC004941.3	0	0	0	0	0	0
RP11-23I7.1	0	0	0	0	0	0
RP11-302F12.2	0	0	0	0	0	0
RP11-410N8.1	0	0	0	0	0	0
AC007952.5	2.93015605	5.63732355	0	0	1.007606311	0
TLE1P1	14.6507803	9.17207777	12.03521197	15.1156742	11.11389761	10.9633366
AP001465.5	0	0	0	0	0	0
RP13-57D9.3	0	0	0	0	1.007606311	0
LL22NC03-23C6.15	0	0	0	0	0	0
AC097713.3	0	0	0	0	0	0
RP11-125K10.5	0	0	0	0	0	0
MTND1P11	0.97671868	0	2.005868661	1.00570022	2.015212623	0
RP11-489C13.1	0	0	0	0	0	0
HNRNPA1P21	0	0	0	0	0	1.812121752
PPIAP19	0	1.3306115	0	4.13342789	4.100957687	0.933242702
RP1-40E16.11	0.97671868	2.03146794	0	0	0	0.906060876

AC121251.1	0	0	0	0	0	0
RP1-317E23.3	2.93015605	5.07866986	26.0762926	17.0969037	15.11409467	16.30909577
AC091770.3	0	0	0	0	0	0
RP11-244N9.6	0	0	0	0	0	0
GEMIN8P4	4.88359342	8.12587178	26.0762926	6.03420129	8.06085049	23.55758278
RP4-656G21.1	0	0	0	0	0	0
RP11-455B2.9	0	0	0	0	0	0
MTND2P25	0	0	0	0	0	0
SNX19P1	0	0	0	0	0	0
RP11-363H12.1	0	0	0	0	0	0
RP13-60M5.2	0	0	0	0	0	0
RPL23AP16	0	0	0	0	0	0
RP11-345I18.6	0	0	0	0	0	0
RP11-342M1.3	0	1.01573397	3.008802992	3.01710065	12.09127574	11.77879139
TCEB1P14	0	0	0	0	0	0
CTA-415G2.2	0	0	0	0	0	0
RPL5P27	0	0	0	0	0	0
PTPN2P1	1.95343737	4.07309323	2.005868661	1.01575722	1.007606311	2.736303846
OR2M3	0	0	0	0	0	0
AL022341.3	3.90687474	1.01573397	11.03227764	6.03420129	0	4.530304381
RNF144A-AS1	0	0	0	0	0	0
RP4-724E13.2	0	0	0	0	0	0
RP11-778D9.4	3.90687474	1.69627573	6.418779717	7.04995851	15.5977457	0.906060876
AC016717.1	0	0	0	0	0	0
AC007967.2	0	0	0	0	0	0
C1orf143	2.93015605	1.01573397	0	0	3.022818934	0
AC073091.2	0	0	0	0	0	0
RPL7AL2	0	0	0	0	0	0
HYALP1	0	0	0	0	0	0
OFD1P17	0	0	0	0	1.007606311	0
NLGN1-AS1	0	0	0	0	0	0
LINC00693	1.95343737	2.03146794	1.002934331	0	0	0
RP11-541F9.2	0	0	0	0	0	0

RP11-367F23.1	0	0	0	0	0	0
PNRC2P1	1.08415774	1.08683535	0	1.11632724	3.556850279	0.978545746
ATF4P3	10.9392493	13.4584751	44.01878777	19.4502422	5.118640061	14.76879228
NPM1P30	0	0	0	0	0	0
LINC00578	0	0	7.020540315	0	1.007606311	6.342426133
AC074363.1	0.97671868	0	0	1.00570022	0	0
HCG11	71.3004639	95.5399374	81.24771013	51.310825	152.1687051	73.4181128
NACAP1	0	0	0	0	1.007606311	2.718182629
AC016908.1	0	0	0	0	0	0
AC074019.1	0	0	0	0	0	0
RP11-219C24.10	0	0	0	0	0	0
RP11-124I4.2	0	0	0	0	0	0
GAPDHP1	22.4352282	8.12587178	11.18271779	18.1026039	32.25347802	27.19088689
GLRXP	0	0	0	0	0	0
AP001476.4	0	0	0	0	0	0
TXNP5	0	0	0	0	0	0
EFCAB14-AS1	0	0	0	0	0	0
GS1-304P7.2	0	0	0	1.00570022	1.007606311	0
RP11-85G21.1	0	0	0	0	0	0
TTTY17A	0	0	0	0	0	0
MOB1AP1	0	0	0	0	0	0
AC093495.4	1.95343737	4.67237627	21.75364563	14.079803	9.068456802	21.74546103
UBBP2	0	0	0	0	0	0
NUTF2P3	0	0	0	0	0	0
AC012442.6	0	0	1.002934331	0	1.007606311	0.906060876
COL6A4P2	0	0	1.002934331	7.03990151	0	3.624243505
MT-ATP8	25512.8687	27211.0865	29794.89228	38110.1045	19871.15521	32440.31367
RP11-323I1.1	0	0	0	0	0	0
AC010141.6	0	0	0	0	0	0
RP11-1379J22.7	0	0	0	0	0	0
RP11-127L20.3	0	0	6.017605984	2.01140043	0	6.342426133
LINC01320	0	0	0	0	0	0
PSMD8P1	0	0	0	0	0	0

RALY-AS1	9.76718684	17.2674775	18.05281795	7.03990151	11.08366942	16.30909577
RP11-513G11.2	0	0	2.005868661	0	0	0
AC106874.1	0	0	0	0	0	0
RP3-508I15.9	7.81374947	9.14160575	138.4049376	67.3819144	0	79.7333571
ARMCX3-AS1	0	1.01573397	6.017605984	2.01140043	2.015212623	0
AC112518.3	0	0	0	0	0	0
ORM2	0	0	0	0	0	0
RP11-367B6.2	0	0	0	0	0	0
KATNBL1P6	0	1.01573397	0	0	0	0.906060876
LYPLA2P1	0	0	2.005868661	1.00570022	1.007606311	0.906060876
AP000593.5	0	1.01573397	1.002934331	0	0	1.812121752
PCAT6	14.6507803	9.14160575	45.13204488	47.2679101	41.31185876	67.95456571
RP4-640E24.1	0	0	0	0	0	0
RP11-132M7.3	0	0	0	0	0	0
RP11-445P17.6	0	0	0	0	0	0
RP11-220I1.4	0	0	0	0	0	0
RP4-686C3.7	0	0	0	0	0	0
BMS1P17	0.48835934	0	0	0.50285011	0	0.453030438
LINC00392	0	0	0	0	0	0
TTTY4C	0	0	0	0	0	0
C19orf24	743.282918	775.005021	496.4524937	829.702678	507.8335809	805.4881189
RPL7P55	0	0	0	0	0	0
RP11-186N15.3	0	2.03146794	0	0	2.015212623	0
RP11-10F11.2	0	0	0	0	0	0
OR4K6P	0	0	0	0	0	0
AC016734.2	3.00829355	6.10456117	2.096132751	1.02581422	3.022818934	5.436365257
OR2S1P	0	3.04720192	0	0	0	0
LINC01209	0	0	4.011737323	0	0	3.624243505
LINC01350	0	0	0	0	0	0
GAPDHP45	0	0	0	0	0	0
CYP4F29P	0	0	21.68344023	91.5086626	56.12367154	22.09882477
GUSBP11	82.5815647	147.108751	602.5428872	547.613824	183.5052614	369.4735041
RP11-522L3.5	0	0	0	0	0	0

RP11-235C23.5	0	0	0	0	0	0
AP001610.5	0.97671868	0	0	0	0	0
SPATA2P1	0	0	0	0	0	0
RP11-358M14.2	0	1.01573397	0	0	0	0
AC008753.6	0	0	1.002934331	0	0	0
IGKV3D-7	0	0	0	0	0	0
RP11-206L10.2	754.534718	685.234452	1256.847215	2032.73133	623.6881546	1110.142028
RP11-516A11.1	4.88359342	4.06293589	0	2.01140043	0	2.718182629
AC097495.3	0	0	0	0	0	0
RP11-730A19.5	0	0	0	0	2.015212623	0
RPL17P43	0	0	0	0	0	0
AC024028.1	0	0	0	0	0	0
AC073063.10	3.91664192	1.01573397	2.005868661	1.00570022	4.030425245	6.342426133
OR9H1P	0	0	0	0	0	0
RP11-804H8.5	0	0	0	0	0	0
RP5-845O24.8	0	0	0	0	0	0
AMD1P1	0	0	0	0	0	0
MIR646HG	26.3714045	15.2360096	0	0	6.045637868	0.906060876
CTB-20D2.1	0	0	0	0	0	0
RP11-1148L6.5	0	0	0	0	0	0
RP11-12D5.4	0	0	0	0	0	0
FTLP17	0	0	0	0	0	0
EFCAB14P1	0.97671868	1.01573397	0	0	0	0
RPS26P4	0	0	0	0	0	0
CELSR3-AS1	0	0	0	0	0	0
AC018832.1	0	0	0	0	0	0
RP11-537H15.3	0	0	5.014671654	7.03990151	0	2.718182629
RP11-184A2.2	0	0	0	0	0	0
RP11-552J9.9	0	0	0	0	0	0
BX322559.3	0	0	0	0	0	0
AC113617.1	3.90687474	3.04720192	0	0	0	0
RP11-365F18.1	0	0	0	0	0	0
RP11-503C24.3	0	0	0	0	0	0

AC015971.2	0	0	4.011737323	7.03990151	2.015212623	1.812121752
SAR1P1	0	0	0	0	0	0
RP1-90J20.2	0	0	0	0	0	0
RP11-18B3.3	0	0	0	0	1.007606311	0
RP11-524L6.2	0	0	0	0	0	0
AC106873.4	0	0	0	0	0	0
TXNDC12-AS1	0	0	0	0	0	0
RP11-85L21.4	0	0	1.002934331	0	0	3.624243505
RP11-497K15.1	0	0	0	0	0	0
AC012497.2	0	0	0	0	0	0
RPS20P25	0	0	0	0	0	0
GAS2L1P2	0	0	0	0	0	3.624243505
AC010891.2	0	0	0	0	0	0
MYL6BP1	0	0	0	0	0	0
ITPKB-IT1	0	1.01573397	0	1.00570022	0	0.906060876
FAM197Y7	0	0	0	0	0	0
AC007040.6	0	0	0	0	0	0
RP5-1125A11.4	0	0	0	0	0	0
AC011995.3	0	0	0	0	0	0
RP11-343J18.1	0	0	0	0	0	0
LINC01004	2.93015605	7.11013781	14.04108063	12.0684026	49.37270925	6.342426133
RP11-216B9.6	0	8.12587178	3.008802992	14.079803	4.030425245	2.718182629
RP1-224A6.3	0	2.03146794	1.002934331	1.00570022	0	2.718182629
HMGN2P25	0	0	0	0	0	0
RP4-575N6.2	0	0	0	0	0	0
AC079154.1	0	0	0	0	0	0
RP11-251M1.1	0	0	0	0	0	0
RP11-563N6.6	0	0	0	0	0	0
AP001468.58	0	1.01573397	3.008802992	0	1.007606311	2.718182629
RP4-800M22.1	0.97671868	0	0	1.00570022	0	0
RP1-111D6.3	0	0	0	0	0	0
CCT6P1	47.673639	60.0095631	56.1141758	73.1043487	73.2328267	92.82593676
TCEB1P24	0	0	0	0	0	0

CDY4P	0	0	0	0	0	0
RP4-625H18.2	0	0	0	4.02280086	15.11409467	10.87273051
AC024937.2	0	0	0	0	0	0
LINC01185	0	0	0	0	0	0
PTMAP1	2.93015605	0	2.005868661	1.00570022	0	0
RP11-549L6.3	0	0	0	0	0	0
RP4-735C1.6	0	1.01573397	0	0	1.007606311	0
AC005013.5	0	0	38.11150457	22.1254047	1.007606311	9.966669638
LINC00687	0	0	0	1.00570022	0	0
RP4-633I8.4	0.97671868	0	2.005868661	1.00570022	0	0.906060876
RP11-402L1.11	0	0	0	0	0	0
RP5-1091N2.9	0	0	1.002934331	1.00570022	0	0
RP5-859M6.1	0	0	0	0	0	0
RP11-15B24.5	0	0	0	0	0	0
ARL5AP3	0	0	0	0	0	0
DHFRP2	0	0	0	0	0	0
OR7E23P	0	0	0	0	0	0
AC004951.6	50.7893716	56.8811024	74.21714047	70.3990151	169.2778603	120.5060965
RP5-864K19.4	2.93015605	2.03146794	11.03227764	14.079803	2.015212623	1.812121752
RP11-400N13.2	0	1.01573397	0	0	0	0
RP5-859I17.2	0	0	0	0	0	0
TSTD3	16.6042176	21.3304134	27.07922693	32.1824069	45.34228401	18.12121752
MTND5P6	0	0	0	0	0	0
RP11-173B14.4	2.93015605	1.01573397	2.005868661	0	0	0
UGT1A2P	0	0	7.020540315	7.03990151	7.053244179	12.68485227
AC073052.1	3.90687474	1.01573397	5.014671654	3.01710065	8.06085049	8.154547886
RP1-154J13.3	0	0	0	0	0	0
SDAD1P1	21.487811	23.3618814	7.020540315	26.1482056	19.14451991	24.46364366
RP5-994D16.9	0	0	0	0	0	0
RPS15AP9	0	0	0	0	0	0
LINC01546	2.93015605	5.07866986	0	0	0	0
CYP2C59P	0	0	0	0	0	0
RP11-445N18.3	0	0	0	0	0	0

AP006222.2	28.1685668	50.4718211	93.03218852	87.3148927	111.5621708	168.6451109
RP11-617O8.1	0	0	0	0	0	0
TRAPPC2P10	0	0	0	0	0	0
TUBB4AP1	0	0	0	0	0	0
RP11-402N8.1	0	0	0	0	0	0
RP11-176D17.3	0	0	0	0	0	0.915121485
AC018865.11	0	0	0	0	0	0
LIN28AP2	0	0	0	0	0	0
OST4	1455.31084	1581.49779	1516.436708	1797.18629	1691.770997	1401.676175
CSTP1	0	0	0	0	0	0
RP3-342P20.2	5.97751835	0	21.08167963	66.5874113	5.04810762	14.51509524
RP1-290I10.3	0	0	0	0	0	0
AC130464.1	0	0	0	0	0	0
U51244.2	0	0	0	0	0	0
RP5-859D4.3	0	0	0	0	0	0
RP11-106M7.1	0	0	0	0	0	0
GRK5-IT1	0	2.03146794	0	0	0	0
LINC01125	21.487811	29.4562852	62.1819285	113.644124	38.28903983	75.20305272
RP13-225O21.2	0	0	0	1.00570022	0	0
AC092168.4	0	0	0	0	0	0
RPL21P50	0	0	0	0	0	0
RAB11FIP1P1	0	1.01573397	2.005868661	1.00570022	0	0
LINC01013	0	0	0	0	0	0
AC106875.1	0	0	0	0	0	0
TMSB10P1	0	0	0	1.00570022	0	1.812121752
RPL15P18	0	0	0	0	0	0
EEF1A1P11	38.9613083	37.4501116	36.5669857	24.1669762	35.89093681	26.30294724
RP11-347D21.2	0	0	0	0	17.12930729	2.718182629
RP4-586O15.1	0	0	0	0	0	0
AC011897.2	0	0	0	0	0	0
RP11-98I9.4	25.3946858	18.2832115	39.1144389	47.2679101	50.38031556	67.04850484
DAP3P2	0.97671868	3.04720192	1.002934331	0	0	0
AC006460.2	0	0	0	0	0	0.906060876

RP11-339B21.8	0.97671868	4.06293589	1.002934331	0	2.015212623	1.812121752
RP11-281A20.2	0	0	0	0	0	0
AC023271.1	0	0	0	0	0	0
CT47A7	0	0	0	0	0	0
SURF6P1	0	0	0	0	0	0
RP11-298C3.2	4.9714981	6.19597723	7.120833748	3.06738566	8.211991437	13.79024654
AC099552.3	0	0	0	0	0	0
RP11-236F9.2	0	0	0	0	0	0
RP11-131O15.2	0	0	0	0	0	0
RP5-1087E8.2	0	0	0	0	0	0
MIR34AHG	16.6042176	17.2674775	601.7605984	535.032515	179.3539234	491.9910558
RP11-179B15.5	0	0	0	0	0	0.906060876
AC068057.1	0	0	0	0	0	0
RP11-567B20.1	0	0	0	0	0	0
RP1-241P17.4	15.5591286	8.68452546	38.02124048	50.5163218	19.23520448	34.32158599
RP11-392O17.1	0	0	0	0	2.015212623	0
AC009411.1	0	0	0	0	0	0
RP11-526K17.2	0	0	0	0	0	0
AC073326.3	0	2.03146794	0	0	0	0
AC093159.1	0	0	0	0	0	0
GS1-519E5.1	0	0	0	0	0	0
CCDC183-AS1	21.487811	52.8181666	137.4020033	374.12048	123.9355763	387.794055
CTA-313A17.3	0	0	0	0	0	0
OR7E26P	0	0	0	0	0	0
ITPKB-AS1	0	0	0	0	0	0
RP11-108M9.3	0.97671868	0	0	0	0	0
RP11-483M24.2	0	0	1.002934331	3.01710065	0	0
SNRPGP9	0	0	0	0	0	0
RP11-391J2.3	0	0	0	0	0	0
AC004837.5	0	0	0	0	0	0
HSPA8P17	0	0	0	0	0	0
RP3-340B19.3	0	1.01573397	0	0	2.015212623	0
RP11-550P17.5	0	0	0	0	0	0

RP11-114M1.1	0	0	6.017605984	1.00570022	2.015212623	6.342426133
AL133247.2	0	0	0	0	0	0
LINC01005	0	0	0	0	0	0
RP11-170M17.1	0	0	0	0	0	0
VN1R4	0	0	0	0	0	0
AC006461.2	0	0	0	0	0	0
AC073133.2	0	0	0	0	0	0
NUTM2E	0	5.15992858	6.077782044	0	2.408179084	2.065818798
HSFY7P	0	0	0	0	0	0
LL0YNC03-29C1.1	0	0	0	0	0	0
RP11-279N8.1	0	0	0	0	0	0
AC010731.2	0	0	0	0	0	0
TUBB1P2	0	0	0	0	0	0
AC073143.1	0	0	0	0	0	0
AC005042.5	0	0	0	0	0	0
RP1-41P2.7	0	0	0	0	0	0
SPCS2P4	24.3495968	17.0643307	27.18954971	16.4934835	23.88026958	27.48988698
AC007381.3	0	0	0	0	0	0
D21S2088E	0	0	0	0	0	0
CFTRP1	0	0	0	0	0	0
C1orf233	251.016702	286.43698	626.8339567	774.389166	1051.940989	1246.739766
PAICSP2	0	0	0	0	0	0
AC013436.6	0	0	0	0	0	0
MTND4P1	0	0	0	0	0	0
MACC1-AS1	0	0	0	0	0	0
RPL7P52	0	0	0	0	0	0
POLR2CP	0	0	0	0	0	0
RPL39P	0	0	0	0	0	0
RP5-872K7.7	0	0	0	0	0	0
RP11-574F21.2	5.8603121	5.07866986	3.008802992	5.02850108	3.022818934	5.436365257
CLDN25	0	0	0	0	0	0
PDCL2P1	0	0	0	0	0	0
HNF4GP1	0	0	0	0	0	0

HK2P1	0.97671868	6.10456117	7.030569658	7.03990151	1.007606311	0.906060876
AC144450.1	1.95343737	0	55.16138819	31.1767067	12.09127574	19.0272784
RP13-476E20.1	0	0	0	2.01140043	0	0
AC093166.3	0	0	0	0	0	0
RP11-66N11.7	0	0	0	0	0	0
AC012462.3	0	0	0	0	0	0
RP3-434P1.6	0	0	0	0	0	0
RP1-302D9.1	0	0	0	0	0	0
ZNF883	1.95343737	4.06293589	294.8626932	211.197045	39.29664614	170.3394447
RP3-399L15.3	40.045466	19.2989455	0	5.02850108	1.007606311	10.87273051
RP11-1B20.1	0	0	2.005868661	0	0	0
RP11-495P10.9	0	0	0	0	0	0
GS1-278J22.2	0	0	0	0	0	0
AC092295.4	0	0	0	0	2.015212623	0
HOTAIR	0	0	609.7840731	236.339551	75.57047335	323.4637328
RP4-534N18.2	0	0	0	0	0	0
RP5-1051H14.2	0	0	0	0	0	0
FCF1P2	40.045466	39.6136249	83.24354945	81.4617175	23.17494516	88.79396587
AC005152.3	0	0	0	0	0	0
AC079779.4	0	0	0	1.00570022	0	0.906060876
PHKG1P2	0	0	0	0	0	0
RPL31P13	0	0	0	0	0	0
RP11-568A7.2	0	0	0	0	0	0
AC005682.5	95.718431	90.4003235	1.002934331	0	34.25861458	9.060608762
AC008940.1	0	0	0	0	0	0
RP11-556E13.1	0	0	0	0	0	0
HNRNPCP7	0	0	0	0	0	0
AC096558.1	0	0	0	0	0	0
MYO5BP3	0	0	0	0	0	0
RPL23AP58	0	0	0	0	0	0
RP11-791O21.5	0	0	0	0	0	0
LINC01201	0	0	0	0	0	0
VN1R35P	0	0	0	0	0	0

AC090587.5	0	0	0	1.00570022	1.007606311	0.906060876
PSMD10P1	0	0	0	0	0	0
RP11-416K24.1	0	0	0	0	0	0
RP11-20O24.1	0	0	0	0	0	0
KRT18P1	0	0	0	0	1.047910564	0
RP11-129O7.2	0	0	0	0	0	0
TRGV5P	0.81067651	0.30472019	0	0	0	0
LINC00448	0	0	0	0	0	0
NANOGP2	0	0	0	0	0	0
PROB1	118.182961	137.124086	27.07922693	39.2223084	166.2550414	96.94851375
CTB-147C22.3	0	0	0	0	0	0
AC006482.1	0	0	0	0	0	0
TTC3-AS1	0	0	0	0	0	0
RP4-676J13.2	0	0	0	0	0	0
AC004692.4	0	0	0	0	0	0
AC008072.1	0	0	0	0	0	0
RP11-31E13.2	0	0	0	0	0	0
RP11-336A10.7	0	0	0	0	0	0
RP11-492I21.1	0	0	0	0	0	0
RP3-419C19.3	0	0	0	0	0	0
COL11A2P1	0	0	0	0	0	0
RP3-335N17.2	0	0	3.008802992	0	0	1.812121752
RP5-826L7.1	0	0	0	0	0	0
MTND4P18	0	0	0	0	0	0
CES1P1	0	0	0	0	0	0
ARL17B	129.805913	109.353919	261.6455082	270.533358	104.9421973	259.3508652
RP5-968D22.1	0	1.01573397	0	0	0	0
RP11-155G14.1	0	0	2.005868661	0	1.007606311	0
TNKS2-AS1	0	5.07866986	1.002934331	3.01710065	7.053244179	0
RP11-671E7.1	3.90687474	1.01573397	0	0	0	0
RP5-1160K1.6	0	0	1.002934331	0	0	0
RP11-138M12.1	0	0	0	0	0	0
LINC00659	0.97671868	6.09440383	5.014671654	1.00570022	18.1369136	0.906060876

RP11-787B4.2	0	0	0	0	0	0
AL109763.1	0	0	0	0	0	0
AP001065.15	13.6740616	18.2832115	0	0	5.038031556	0.906060876
AC004006.2	0	0	0	0	0	0
RP11-284G10.1	0	0	0	0	1.007606311	0
DHFR	5441.52443	5010.96103	1674.830127	2747.37185	4057.509703	3199.192226
AF013593.1	0	0	0	0	0	0
RP11-145H9.3	0	0	0	0	2.015212623	0
RP5-1119A7.14	0	0	2.005868661	0	0	1.812121752
AC016909.2	0	0	0	0	0	0
SRGAP3-AS2	0	1.01573397	0	0	0	0
MTND2P12	0	0	0	0	0	0
SAPCD1	94.3021889	65.3624811	49.6753374	42.9836272	10.35819288	29.4741603
RP11-316K19.3	0	0	0	0	0	0
RP11-211A18.2	0	0	0	0	0	0
RP11-95L3.2	0	0	0	0	0	0
RP11-335E6.3	0	0	0	0	0	0
GS1-18A18.2	0	0	0	0	0	0
AC008781.7	0	1.01573397	0	1.00570022	3.022818934	1.812121752
RP11-218I7.2	0	0	0	0	0	0
GABRG3-AS1	0	0	0	0	0	0
SPATA13	0	1.01573397	0	1.00570022	0	0
RP5-884M6.1	79.1142134	114.777939	0	0	18.1369136	11.77879139
RPS3AP30	0	0	0	0	0	0
RP13-39P12.3	2.93015605	5.07866986	3.008802992	10.0570022	7.053244179	3.624243505
RP11-242F24.1	0	0	0	0	0	0
AC004022.8	0	0	0	0	0	0
EIF4BP2	0	0	0	0	2.075669001	0
RP11-534L6.3	0	0	0	0	0	0
PABPC1P8	0	0	0	0	0	0
AB019438.66	0	0	0	0	0	0
FAF2P1	0	0	0	0	0	0.906060876
LIMS1-AS1	0	0	0	0	0	0

ZNF885P	0	0	0	0	0	0
RPL7L1P8	13.3419772	6.13503319	1.012963674	2.03151444	3.04297106	4.557486207
KRT8P18	0	0	0	0	1.007606311	2.718182629
AC003101.1	0	0	3.008802992	1.00570022	0	0.906060876
ABCF2P2	0	0	0	0	0	0
AC007327.6	0	0	0	0	0	0
RP11-552J9.1	0	0	0	0	0	0
RP1-309H15.2	0	0	0	0	0	0
WEE2-AS1	0.97671868	5.07866986	83.24354945	76.4332164	27.2053704	29.90000891
RP11-319C21.1	0	0	0	0	0	0
RP11-282C5.1	0	0	0	0	0	0
RP11-129J12.1	0	0	0	0	0	0
Z69666.2	0	0	1.002934331	0	0	0
RP1-50A13.1	0	0	0	0	0	0
RP11-336N8.3	0	0	0	0	0	0
CTD-2026D20.3	52.1860793	33.1027702	106.5517433	104.039687	41.09018537	133.5443125
RP11-147I11.1	0	0	0	0	0	0
LINC00954	0	0	0	0	2.015212623	0.906060876
LINC00266-4P	0	0	1.644812302	1.30741028	0	1.866485405
NLGN4Y-AS1	0	0	0	0	0	0
HCG22	0	0	0	0	0	0
AC098784.1	0	0	0	0	0	0
THRB-AS1	0	0	10.02934331	13.0741028	1.007606311	19.0272784
RP11-354K1.2	0	0	0	0	0	0
RP1-223B1.1	0	1.01573397	12.03521197	11.0627024	0	9.966669638
LINC01128	305.712948	325.034871	994.9108561	1018.77432	203.5364749	774.6820491
FAM207BP	2.95945761	0	0	1.00570022	1.007606311	7.266608227
AP000473.5	0	1.01573397	3.008802992	0	2.015212623	5.436365257
AC113607.2	0	0	0	0	0	0
RP11-253D19.1	0	0	0	0	1.007606311	0
RP11-110G21.1	9.76718684	11.1730737	44.12911055	43.2451093	45.34228401	49.83334819
AC073641.2	0	0	0	0	0	0
AC073072.6	0	0	0	0	0	0

RP11-211G3.3	0	0	0	0	0	0
AC092628.3	0	0	0	0	0	0
MRPS18CP6	0	0	0	0	0	0
HMGB3P4	0.97671868	0	6.017605984	1.00570022	2.015212623	0
RP4-705D16.3	0	0	0	0	0	0
PABPC1P11	0	0	0	0	0	0
LAMA5-AS1	0	0	0	0	0	0
RP11-119H12.5	0	0	0	0	0	0
MARK2P13	0	0	0	0	0	0
AK3P5	0	0	0	0	0	0
BACH1-IT2	9.76718684	8.12587178	0	1.00570022	0	7.248487009
RP11-567G24.1	8.79046815	8.12587178	2.005868661	4.02280086	5.04810762	4.53936499
RP1-305B16.3	0	0	0	0	0	0.906060876
RPSAP1	0	0	0	0	0	0
RP11-293F5.8	0	0	0	0	0	0
MIR4500HG	1.95343737	0	0	0	0	0
LAMTOR3P1	0	0	0	0	0	0
RP11-344P13.4	0	0	0	0	0	0
RP11-234P3.2	0	0	0	0	0	0
RP11-162G10.1	1.95343737	4.06293589	34.87202668	9.05130194	1.007606311	18.12121752
AC005077.5	0	0	0	0	0	0
RP4-781K5.2	8.79046815	6.09440383	43.12617622	49.2793106	5.038031556	31.71213067
RP5-1014O16.9	0	0	0	0	0	0
RP11-249L21.4	0	0	0	0	0	0
AC012123.1	0	0	0	0	0	0
CT45A5	0	0	0	0	0	0
CBX3P6	0	0	0	0	0	0
RP4-784A16.2	0	1.01573397	0	0	0	0
PIK3IP1-AS1	0	0	0	4.02280086	2.015212623	0
PCDH9-AS2	0	0	0	0	0	0
RP11-112J3.15	1.95343737	0	2.005868661	2.01140043	6.045637868	0.906060876
RP11-467I20.3	0	0	0	0	0	0
ATP5G2P4	0	0	0	0	0	0

AC105402.2	0	0	0	0	0	0
CDY12P	0	0	0	0	0	0
RP11-1217F2.10	0	0	0	0	0	0
RP11-57H12.5	0	0	0	0	0	0
NEGR1-IT1	0	0	0	0	0	0
RP3-433G13.1	0	0	0	0	0	0
USP17L30	0	0	0	0	0	0
AC104653.1	1.95343737	0	1.002934331	0	0	0.906060876
RP11-565H13.3	0	0	0	0	0	0
RPL23AP12	0	0	0	0	0	0
RP11-91I20.3	0	0	0	0	0	0
RP11-404F10.2	0	0	0	0	2.015212623	0
MYLKP1	0	0	0	0	0	0
COX4I1P2	0	0	0	0	0	0
AC096664.2	0	0	0	0	0	0
AC012307.2	0	0	0	0	0	0
AC090954.5	0	0	0	0	0	0
AC010745.2	0	0	0	0	0	0
RP11-473E2.4	0	0	0	0	0	0
SEPT7-AS1	8.79046815	23.3618814	42.12324189	43.2451093	9.068456802	12.68485227
RP11-439E19.6	0	0	1.002934331	0	0	0
CICP9	0	0	0	0	0	0
RPL23AP26	0	0	0	0	0	0
RP11-290D2.3	0.97671868	1.01573397	5.014671654	0	0	1.812121752
EEF1DP1	0	0	1.002934331	1.00570022	7.063320242	0
LINC01428	0	0	0	0	0	0
UBAC2-AS1	20.5110924	28.4405512	7.020540315	10.0570022	48.36510294	15.4030349
TTTY21	0	0	0	0	0	0
CTD-2021A8.2	0	0	0	2.01140043	0	0
MTCO1P43	0	0	0	0	0	0
HMGN2P36	0	0	0	0	0	0
ST6GALNAC2P1	0	0	0	0	0	0
RASA4CP	16.8776989	30.73611	43.91849434	142.920058	23.06410847	50.31356045

RP13-216E22.4	0	2.03146794	4.011737323	8.04560172	2.015212623	6.342426133
AC008281.1	0	0	0	0	0	0
OR1H1P	0	0	0	0	0	0
OR7E128P	0	0	2.005868661	0	0	1.812121752
RP11-544M22.8	0	0	0	6.03420129	0	0.906060876
LINC01344	0	0	0	0	0	0.906060876
AC097381.1	2.93015605	1.01573397	0	0	0	0
RP13-210D15.1	0	0	0	0	0	0
AP000355.2	0	0	0	0	0	0
AC016722.4	0.97671868	2.03146794	0	0	0	0
TSPY3	0	0	0	0	0	0
AC002069.5	0.97671868	0	0	0	1.007606311	0
RPS13P2	2.98875917	9.31428053	1.022993017	0	2.055516875	0.924182094
MTCO1P3	0	0	0	0	0	0
RP11-268G12.1	0	0	1.002934331	0	0	0.906060876
RP11-189G24.2	0	0	0	0	0	1.812121752
AC005539.2	0	0	0	0	0	0
SNRPD2P1	0	0	0	0	0	0
AKT3-IT1	1.95343737	2.03146794	0	0	0	0
RP11-348A7.1	0	0	0	0	0	0
UBE3AP2	0	0	0	0	0	0
RP5-1053E7.2	0	0	0	0	0	0
AC004485.3	0	0	0	0	0	0
CLUHP2	0	0	0	0	0	0
SLC25A6P5	0	1.01573397	0	0	0	0
UGT1A12P	0	0	0	0	2.015212623	0
AC023137.2	0	0	2.005868661	2.01140043	1.007606311	0
RP11-336A10.4	0	0	0	0	0	0
RP11-567G11.1	0	0	0	0	5.038031556	0
AC006026.12	0	0	0	0	0	0
RP11-452J6.2	0	0	0	0	0	0
SATB1-AS1	0.97671868	3.04720192	0	0	2.015212623	0
RP11-23J9.3	0	0	0	0	0	0

PIGPP3	0	0	0	0	0	0
RP5-1121H13.3	0	0	0	0	0	0
OR2A9P	61.6504833	52.4525023	82.50137804	87.6467738	35.37705759	73.61744619
AP000282.3	0	0	0	0	0	0
HCG23	0.97671868	3.04720192	0	0	0	0.906060876
OR7E93P	0	0	0	0	0	0
RPSAP60	0	0	0	0	0	0
HOMER2P1	0	0	0	0	0	0
AC096554.1	0	0	0	0	0	0
UBTFL6	0.97671868	0	0	1.00570022	0	0
RP11-286B14.1	0	0	0	0	0	0
AC009955.8	0	0	0	0	0	0
AC006483.5	0	0	0	0	0	0
SUMO2P8	0	0	0	0	0	0
CTD-2501B8.5	0	0	0	0	0	0
LINC01205	0	0	0	0	0	0
RP11-364L4.1	5.88961366	5.10914188	7.933210556	21.8639227	13.28025118	14.19797393
RP11-107G24.3	0	0	0	0	0	0
SLC47A1P1	0	0	0	0	0	0
RP11-101E19.8	0	0	0	0	0	0
TRDD3	0	0	0	0	0	0
RP13-228J13.8	0.98648587	0	0	0	0	0
RP4-677H15.4	0	0	0	0	0	0
AC133528.2	9.76718684	13.2045416	6.017605984	13.0741028	48.36510294	19.93333928
RPL5P32	0	0	0	0	0	0
MTND1P9	0	0	0	0	0	0
RP1-186E20.1	7.81374947	4.06293589	0	0	0	0
RP11-697E2.7	8.00909321	0	4.97455428	6.61750742	0.90684568	0
AC068490.1	0	0	0	0	0	0
SEPT7P8	0	0	0	0	0	0
ACTBP14	0	0	2.005868661	0	0	0.906060876
RP11-666A1.4	0	0	0	0	0	0
HNF4A-AS1	0	0	0	0	0	0

EXOSC3P1	3.91664192	0	4.011737323	5.02850108	0	5.472607692
TMPRSS11GP	0	0	0	0	0	0
RP5-979D14.1	0	0	0	0	0	0
LINC01038	0	0	0	0	0	0
GS1-542M4.4	0	0	0	0	0	0
AC083939.1	0	0	0	0	0	0
RPL30P13	0	0	0	0	0	0
RP11-265D19.6	0	0	0	0	0	0.906060876
RP11-224O19.4	0	0	0	0	0	0
LINC01277	0	3.04720192	23.06748961	1.00570022	0	3.624243505
PMS2P7	11.4373758	6.04361713	29.50632801	31.5387588	22.48977287	35.91625313
RP11-88I18.3	0	0	0	0	0	0
AKR7A2P1	0.97671868	2.03146794	0	0	0	0
AL591893.1	0	2.03146794	0	0	6.045637868	2.718182629
RP11-553N16.1	0	0	0	0	0	0
AC067945.3	0	1.01573397	3.008802992	0	3.022818934	1.812121752
AP001595.1	0	0	0	0	0	0
HSFY1P1	0	0	0	0	0	0
KRT223P	0	0	17.04988362	13.0741028	1.007606311	3.624243505
CDCA4P1	0	0	0	0	0	0
RP11-402P6.6	0	0	0	0	0	0
RP11-666G4.1	0	0	0	0	0	0
RP11-91A18.1	0	0	0	0	0	0
SPRR2C	0	0	0	0	0	0
VDAC1P8	10.7439055	2.03146794	2.005868661	7.03990151	2.015212623	11.77879139
RP11-272G22.3	0	0	0	0	0	0
RP1-232N11.2	0	0	0	0	0	0
AC091729.9	32.2317166	23.3618814	155.4548213	107.609923	151.1409467	109.633366
RP11-266K22.2	0	0	0	0	0	0
HMGN1P2	0	0	0	0	0	0
AF127577.10	0	0	1.002934331	1.00570022	3.022818934	1.812121752
DUTP1	0	0	0	0	0	0
RP5-952N6.1	0	0	0	0	0	0

RP11-386I23.1	0	0	0	0	0	0
AC074338.4	0	0	1.002934331	0	0	0
AC020571.3	0	2.03146794	12.03521197	16.0912034	2.015212623	21.74546103
RPS3AP54	0	0	0	0	3.022818934	0.906060876
RP11-20P5.2	0	0	0	0	0	0
TRBV23OR9-2	0	0	0	0	0	0
RP11-746P2.5	0	0	0	0	0	0
RP11-80I15.4	0	0	0	0	0	0.906060876
AC096649.3	0	0	0	0	0	0
RP11-91I11.1	0	0	0	0	0	0
TMPOP1	0	0	0	0	0	0
HMGN2P24	0	0	0	0	0	0
CR848007.5	0	0	0	0	0	0
RP11-291J9.2	0	0	0	0	0	0
LINC01165	0	0	0	0	3.022818934	1.812121752
OR5AW1P	0	0	0	0	0	0
PSMA6P2	0.97671868	0	0	0	0	0
LINC01548	0	0	0	0	0	0
RP11-32P22.1	0	0	0	0	0	0
MTND1P10	0	0	0	0	0	0
ANKRD20A8P	0	0	0	0	0	0
RP1-232L22__A.1	0	0	0	0	0	0
HSPA8P8	0	0	2.005868661	0	0	1.812121752
IGHV3-47	0	0	0	0	0	0
OR51AB1P	0	0	0	0	0	0
CALM2P2	2.93992324	4.07309323	2.005868661	1.00570022	3.022818934	0.906060876
TCEB1P20	0	0	0	0	0	0
RP11-360P21.2	0	0	0	0	0	0
YY1P2	0	0	0	0	0	0
ASTN2-AS1	0	0	0	0	0	0
BTBD6P1	0	0	0	0	0	0
ABHD17AP4	0	0	1.083169077	4.22394091	0	0.978545746
MEOX2-AS1	0	0	0	0	0	0

RP11-439K3.1	0	0	0	0	0	0.906060876
AC006355.3	0	0	0	0	0	0
MED4-AS1	0	0	3.008802992	1.00570022	1.007606311	1.812121752
RP11-690C23.3	0	0	0	0	0	0
RP11-417B4.3	0	0	0	0	0	0
RP11-20J15.3	0	0	0	0	0	0
RPL41	9408.52597	9261.65535	10567.45769	12149.9548	18589.62104	11422.92692
AC068491.2	0	0	0	0	0	0
CTB-63M22.1	42.0965753	27.8920549	39.1144389	35.5112746	65.8873767	28.78555404
CYCSP4	0	0	0	0	0	0
AGBL5-IT1	0	0	0	0	0	0
VIM-AS1	545.985744	632.802265	0	0	19.14451991	11.77879139
AC007038.7	19.5343737	20.3146794	22.06455528	11.0627024	6.045637868	28.08788716
ACTG1P2	0	0	0	0	0	0
AC016710.1	0	0	0	0	0	0
EIF4A1P10	77.9030822	69.6895078	15.68589293	22.8495089	91.04730629	31.55810032
RPS7P4	0	0	0	0	0	0
CDY6P	0	0	0	0	0	0
CCDC26	0	0	0	0	0	0
HCG4P8	0	0	0	0	0	0
AC009480.4	0	0	0	0	0	0
ACTBP1	2.93015605	0	0	0	0	0
SNX18P4	0.97671868	1.26966747	0	2.01140043	0	0
SMPD4P2	0	0	0	0	0	0
CRYGEP	0	0	0	0	0	0
RP11-348F1.3	0	0	0	0	0	0
ANKRD10-IT1	76.1840573	36.566423	43.12617622	14.079803	59.44877237	46.20910469
EPHA1-AS1	2.93015605	0	3.008802992	5.02850108	3.022818934	0
KCNQ5-AS1	0	0	0	0	0	0
RP11-528A4.2	0	0	0	0	0	0
RP11-250H24.2	0	0	0	2.62487756	0	0
TSPY23P	0	0	0	0	0	0
AC009229.6	0	0	0	0	0	0

TCP1P1	0	0	0	1.00570022	0	0
RP11-84D1.1	0.97671868	2.03146794	0	0	0	0
NAP1L1P2	0	0	0	0	0	0
GDI2P1	0	0	0	0	0	0
RP11-73M7.1	0	0	0	0	0	0
RPL19P20	0	0	0	0	0	0
RP11-247I13.6	0	0	0	0	0	0
AC073065.3	0	0	0	0	0	0
AC108059.4	0	0	0	0	0	0
LINC00382	0	0	0	0	0	0
AC008154.5	0	0	0	0	0	0
AC069513.4	7.81374947	5.07866986	10.02934331	24.1368052	9.068456802	9.966669638
GS1-124K5.11	264.690763	265.106567	271.7952036	217.231247	198.4984433	244.6364366
MRPS16P1	0	0	0	0	0	0
PGA4	0	0	0	0	0	0.906060876
ATP5HP2	0	0	0	0	0	0
ADAM1A	0	0	1.935663258	0	0	0
RP4-553F4.2	0	0	0	0	0	0
RP11-390B4.3	0	0	0	0	0	0
RP11-168O16.1	0	0	0	0	3.022818934	0.906060876
AC004870.3	0	0	0	0	0	0
AC009495.4	0.97671868	0	0	0	0	0.906060876
RP11-775D22.2	0	0	0	0	0	0
RP11-227H15.7	0	0	0	0	0	0
RP5-827O9.1	0	0	0	0	0	0
AC103564.7	0	0	0	0	0	0
PTGES3P3	2.95945761	4.10356525	8.093680049	2.04157144	9.149065306	4.575607425
LINC00200	0	0	0	0	0	0
RP11-397O4.1	0	0	0	0	0	0
SERPINH1P1	0	0	0	0	0	0
RBMY2NP	0	0	0	0	0	0
AC073987.1	0	0	0	0	0	0
KCTD9P6	0	0	0	0	0	0

RP11-561C5.4	41.2858988	43.5445154	31.2414044	24.5089143	77.86781574	16.11882299
RP1-118J21.24	0	0	0	0	0	0
LINC00242	11.7206242	11.1730737	0	6.03420129	3.022818934	8.154547886
CYCSP11	0	0	0	0	0	0
RP11-47A17.2	0	0	0	0	0	0
HNRNPA1P66	0	0	1.002934331	0	1.007606311	0
KRT18P4	0.97671868	3.04720192	14.04108063	11.0627024	6.267311256	15.4030349
AC105398.3	0	0	0	0	0	0
RP11-5P4.3	0	0	0	0	0	0
BTF3L4P4	0	0	0	0	0	0
RP11-38L15.2	1.95343737	0	2.005868661	1.00570022	1.007606311	1.812121752
LINC00582	0	0	0	0	0	0
AC022201.4	0	0	0	0	0	0
MT1P3	0	0	0	0	0	0
FEM1AP1	0	0	0	0	0	0
KRT18P53	0	0	0	0	0	0
CTD-2320J21.2	0	0	0	0	0	0
RBMY1KP	0	0	0	0	0	0
RP11-8L18.3	0	0	0	0	0	0
TTTY10	0	0	0	0	0	0
HMGN1P37	0	0	4.723820698	0	0	1.812121752
PPP1R12BP1	0.97671868	0	0	0	0	0
RP11-223J15.2	0	0	0	0	0	0
LINC00710	0	0	0	0	0	0
PNPT1P1	1.95343737	0	0	1.00570022	2.015212623	0.906060876
RP5-1111A8.3	0	0	0	0	0	0
AC098973.1	0	0	0	0	0	0
RP11-542K23.7	0	0	0	0	0	0
LINC00377	0	0	0	0	0	0
RP11-492M19.3	0	0	0	0	0	0
WBP2P1	0	0	0	0	0	0
LINC00446	0	0	0	0	0	0
USP9YP31	0	0	0	0	0	0

HNRNPA1P8	0	0	0	0	0	0
OR8C1P	0	0	0	0	0	0
RP11-407H12.8	0	0	0	0	0	0
ST13P13	0	0.71101378	1.735076392	0.78444617	0	0
RP11-229P13.22	0	1.01573397	2.005868661	2.01140043	0	1.812121752
RP11-552D8.1	0	0	0	0	0	0
LRRC37A12P	0	0	0	0	0	0
RP11-227H15.4	1.95343737	5.07866986	0	0	0	0
RP4-726N1.2	0	0	0	0	0	0
AC004691.5	0	0	0	0	0	0
POM121L8P	0	0	0	0	0	0
AC072062.1	7.81374947	8.12587178	0	4.02280086	14.10648836	6.342426133
PES1P2	0	0	0	0	0	0
RP11-298A8.2	0	0	0	0	0	0
AC091493.2	0	0	0	0	0	0
RP11-498J9.2	0	0	0	0	0	0
RP11-104G3.2	2.93015605	4.2965547	1.985809975	0	0	0
XXbac-BPG13B8.10	0	0	0	0	0	0
LL22NC03-121E8.3	0	0	0	1.00570022	0	0
REV3L-IT1	0	0	0	0	0	0
RP11-483F11.7	0	0	0	0	1.007606311	0.906060876
EEF1DP6	0	0	0	0	0	0
GPR53P	0	0	0	0	0	0
RP1-40E16.2	0	0	0	0	0	0
RP5-1125M8.2	0	0	0	0	0	0
LA16c-4G1.4	0	0	0	0	0	0
FABP5P4	0	0	0	0	0	0
RP11-1E11.2	0	0	0	0	0	0
AP000431.2	0	0	0	0	0	0
RP4-580N22.2	0	0	0	0	0	0
RFPL4AL1	0	0	0	0	1.007606311	0
RP5-1044H5.1	0	0	0	0	0	0
RP11-540H22.2	0	0	0	0	0	0

TUBB8P1	0	0	0	0	5.038031556	0
RP4-583P15.10	12.6973429	9.14160575	6.017605984	5.02850108	6.045637868	5.436365257
RP5-905H7.8	0	0	0	0	0	0
TAF9P2	0	0	0	0	0	0
PPIAP25	0	0	0	0	0	0
RP11-431K24.2	0	0	1.002934331	0	0	0
AP001464.4	0	0	0	0	0	0
LINC00459	0	0	0	0	0	0
AC010084.1	0	0	0	0	0	0
RP11-452B18.2	0	0	0	0	0	0
RPP40P1	0	0	0	0	0	0
RP11-475I24.8	0	0	0	0	0	0
RP11-470P21.2	0	1.01573397	0	0	1.007606311	0
RP3-425P12.5	0	0	0	0	0	0
ORM1	0	0	0	0	1.007606311	0
MCHR2-AS1	0	0	0	0	0	0
HMGB1P45	0.97671868	0	0	0	0	0
KRT8P12	48.8359342	51.8024326	155.4548213	201.170214	164.2398287	154.9364098
AC008269.2	0.97671868	1.01573397	0	0	0	1.812121752
RP5-937E21.1	0	0	0	0	0	0
DLEU1-AS1	0	0	3.008802992	0	0	1.812121752
NUTF2P7	0	0	0	0	0	0
ACAP2-IT1	11.7206242	6.09440383	4.011737323	4.02280086	9.068456802	7.248487009
AC069154.4	0	0	0	1.00570022	0	0
RP11-135A24.2	0	0	0	0	0	0
AC006947.1	0	0	0	0	0	0
GK-IT1	0	0	0	0	0	0
PGBD4P8	0	0	0	0	0	0
AC046143.3	8.79046815	13.2045416	2.005868661	7.03990151	5.038031556	0
DANT1	0	0	0	0	0	0
AP000568.2	0	0	0	0	1.007606311	0
AC079305.8	0	0	0	1.00570022	0	0
RP11-92C4.4	0	0	0	0	0	0

RP11-193I22.2	0	0	0	0	0	0
CDY22P	0	0	0	0	0	0
MTCO2P12	211.400992	173.903813	297.0089727	333.490191	238.7321633	325.0312181
RP11-332M4.1	0	0	0	0	0	0
RP11-504E21.1	0	0	0	0	0	0
HYI-AS1	0	1.01573397	2.005868661	1.00570022	0	0
ACTG1P9	0	0	0	0	0	0
KRTAP9-11P	0	0	0	0	0	0
AC007563.3	0	0	0	2.01140043	0	0.906060876
LRRC3-AS1	0	0	0	0	0	0
CYCSP51	0	0	0	0	0	0
DPY19L1P1	15.7056364	22.2547313	2.005868661	2.01140043	28.26335703	1.812121752
PIN1P1	0	0	0	0	0	0
PPP1R2P5	0	0	0	0	0	0
UBTFL7	0	0	0	0	0	1.812121752
RP4-639J15.2	0	0	0	0	0	0
HMGN2P19	0	0	0	0	0	0
AC090587.4	1.95343737	8.12587178	1.002934331	6.03420129	3.022818934	1.812121752
RP13-225O21.5	0	0	0	0	0	0
AC092635.1	0	0	0	0	0	0
SZT2-AS1	14.6507803	10.1573397	4.011737323	3.01710065	0	4.530304381
LINC00452	239.296078	138.13982	0	0	14.10648836	0
USP24P1	0	0	0	0	0	2.718182629
CICP3	0	0	0	0	0	0
AC006041.1	0	0	0	0	0	0
AC147651.5	0	1.01573397	0	0	0	0
BX322557.13	0	0	0	0	0	0
HMGB1P16	0	0	0	0	0	0
AC010975.1	0	0	0	0	0	0
OR8B9P	0	0	0	0	0	0
RP11-442N24__B.1	2.93015605	1.01573397	2.005868661	0	7.053244179	5.436365257
CTD-2022H16.1	0	0	0	0	0	0
MICD	0	1.01573397	0	0	1.007606311	0

HLA-DRB6	3.09619823	6.11471851	0	0	151.1409467	10.87273051
RP3-395M20.3	0	0	0	0	0	0.906060876
AC013401.1	0	1.01573397	0	0	0	0
RP4-620E11.5	0	0	0	0	0	0
RP11-378J18.6	0	0	0	0	0	0
RP11-3L21.2	0	0	0	0	0	1.812121752
MIR5689HG	3.90687474	1.01573397	0	0	0	0
AL162151.4	0	0	0	0	0	0
RP4-718N17.2	0	0	0	0	0	0
LINC00858	0	0	0	0	11.08366942	3.624243505
AC092580.1	0	0	0	0	0	0
OFD1P4Y	0	0	0	0	0	0
RP11-12M5.3	0	0	0	0	0	0
RP11-494O16.3	0	0	0	0	0	0
RP11-274B21.12	0.97671868	0	0	0	1.007606311	0.906060876
KCNQ1-AS1	0	0	0	0	0	0
SFTA3	0	0	0	0	0	0
USP9YP8	0	0	0	0	0	0
NPM1P25	0	0	8.023474646	15.0855032	0	5.436365257
RP11-35J23.1	0	0	0	0	0	0
RALGAPA1P	44.8216204	28.1459884	25.83558836	44.9950276	42.40007358	33.23431294
AC133141.1	0	0	0	0	0	0
RP11-262H14.5	1.89483425	4.11372259	25.21376907	60.442583	56.14382366	21.16558207
RPL27AP8	0	0	0	0	0	0
AC007349.4	0	0	0	0	0	0
AJ006998.2	0	0	35.10270157	5.02850108	1.007606311	18.12121752
ANKRD26P4	5.8603121	2.03146794	0	3.01710065	1.007606311	1.812121752
RP1-92O14.6	0	0	0	0	2.015212623	0
RP11-329B9.1	0	0	0	0	0	0
AC017048.1	0	0	0	0	0	0
PIP5K1P2	0	0	0	0	0	0
AC073850.6	0	0	0	0	0	0
LINC00366	0	0	0	0	0	0

RP5-1033K19.2	0	0	0	0	0	0
THEMIS3P	0	0	0	0	0	0
LINC00433	0	0	0	0	0	0
RP11-184I16.4	0	0	0	0	0	0
RP11-490K7.4	0.97671868	4.06293589	2.005868661	5.02850108	4.030425245	4.530304381
AC005162.4	0	0	0	0	0	0
SPINK8	0	0	127.37266	14.079803	0	26.27576541
RP11-202I11.2	0	0	0	0	0	0
RPS10P18	0	0	0	0	0	0
RLIMP1	0	0	0	0	0	0
AC006227.1	0	0	0	0	0	0
RP11-202D18.2	0	0	0	0	0	0
AC023669.1	0	0	0	0	0	0
AC127383.1	0	0	0	0	0	0
LYST-AS1	0	0	0	0	0	0
ACTG1P11	0	0	0	0	0	0
RP11-56I23.1	0	0	0	0	0	0
RPL39P18	0	0	0	0	0	0
RGS17P1	0	1.01573397	5.014671654	5.02850108	3.022818934	0.906060876
PATL2	17.5809363	54.8496345	5.014671654	5.02850108	5.038031556	10.87273051
FAM90A16P	0	0	0	0	0	0
ROBO2P1	0	0	0	0	0	0
CTD-2554C21.3	0	0	0	1.00570022	0	0
LINC00362	0	0	0	0	0	0
RP5-888M10.2	0	0	0	1.00570022	0	0
KSR1P1	0	0	0	0	0	0
RP4-552O12.1	0	0	0	0	0	0
ALG13-AS1	0	0	0	0	0	0
RP11-342D14.1	0	0	0	0	0	0
AC004019.10	0	0	0	0	0	0
AC012494.1	0	0	0	0	0	0
RP11-173D14.3	0	0	0	3.01710065	0	0
AC005189.6	0	0	0	0	0	0

AC105053.3	0	0	0	0	0	0
RPL17P1	0	0	0	0	0	0
RP11-52J3.2	0	0	0	0	0	0
AC092155.1	1.89483425	0	0.962816957	2.05162844	0	0
RP4-682E18.1	0	0	0	0	0	0
PHKG1P4	0	0	0	0	0	0
RP11-421E17.4	0	0	0	0	0	0
GAS2L1P1	0	0	0	0	0	0
AC068580.5	0	9.14160575	2.005868661	1.00570022	12.09127574	0.906060876
FLT1P1	0	0	0	0	0	0
UBE2V1P3	0	0	0	0	0	0
RP11-58A11.2	0.97671868	0	0	0	0	0
LINC00404	0	0	0	0	0	0
MYCBP2-AS2	0	0	0	0	0	0
LINC01523	0	0	0	0	0	0
AC053503.4	2.93015605	1.01573397	11.03227764	6.03420129	3.022818934	1.812121752
KRT16P4	0	0	0	0	0	0
RP5-1066H13.4	0	0	0	0	0	0
RP11-62C3.8	0	0	0	0	0	0
RP1-102G20.5	0	0	0	0	0	0
AC105443.2	0	0	0	0	0	0
AC003986.5	0	0	0	0	0	0
HNRNPA1P53	0	0	0	0	0	0
AC079776.1	0	0	0	1.00570022	1.007606311	0
RP11-5P4.1	0	0	0	0	0	0
RP11-119B16.2	2.93015605	2.03146794	3.008802992	5.02850108	7.053244179	4.530304381
GAPDHP26	0	0	0	0	0	0
SSU72P7	0	0	0	0	0	0
RP11-90J7.2	0	0	0	0	0	0
NKX1-2	4.88359342	13.2045416	1.002934331	0	7.053244179	0.906060876
LINC00428	0	0	0	0	0	0
UBE2DNL	0	0	0	0	0	0
TSPY8	0	0	0	0	0	0

AC012445.1	0	0	0	0	0	0
GAPDHP17	0	0	0	0	0	0
USP9YP17	0	0	0	0	0	0
RPL21P24	0	0	0	0	0	0
LINC00379	0	0	0	0	0	0
SACS-AS1	0	0	0	0	0	0
RP1-153P14.3	0	0	0	0	0	0
LYARP1	0	0	0	0	0	0
ZFYVE9P1	0	0	0	0	0	1.812121752
LINC01204	30.2782792	34.5349551	0	0	1.007606311	0
AC108056.1	0	0	0	0	0	0
RP4-717I23.2	0	0	0	0	0	1.812121752
SMC4P1	0	0	0	0	0	0
RP11-481G8.2	0	0	0	0	0	0
GAPDHP58	0	0	0	0	0	0
PRAMEF25	0	0	0	0	0	0
EIF4BP4	0	0	0	0	0	0
LINC00358	0	0	0	0	0	0
USP17L26	0	0	0	0	0	0
RP11-423C15.3	1.95343737	0	0	3.01710065	2.015212623	2.718182629
RPL21P44	1.95343737	0	4.011737323	2.01140043	0	0.906060876
TNPO1P3	0	0	0	0	0	0
RP11-197P3.5	0	2.03146794	3.008802992	1.00570022	1.007606311	0.906060876
RP11-479J7.2	0	0	0	0	0	0
ACVR2B-AS1	31.2549979	22.3461474	11.03227764	32.1824069	13.09888205	13.59091314
MSX2P1	0	0	0	0	0	0.906060876
RP5-981O7.2	6.83703079	1.01573397	4.011737323	8.04560172	3.022818934	3.624243505
SUCLA2P3	0	0	1.002934331	0	0	0
RP11-167P23.4	0	0	0	0	0	0
RP5-1097F14.1	0	0	0	0	0	0
MYL8P	0	0	0	0	0	0
PRDX3P1	0	0	0	0	0	0
LINC00411	0	0	0	0	0	0

RP11-524K14.1	0	0	0	0	0	0
AL590762.10	0	0	0	0	1.007606311	0
AC004014.3	0	0	0	0	0	0
MTATP8P2	0	0	0	0	0	0
RP11-492M23.2	0	1.20872343	1.2737266	0	0	4.213183074
RP5-827L5.1	0	0	0	0	0	0
RP5-1125N11.1	0	0	0	0	0	0
GOLGA2P4	0	0	0	0	0	0
LINC01079	0	0	0	0	0	0
RP11-390F4.10	0	0	0	1.00570022	0	0
SUMO1P2	0	0	0	0	0	0
LINC01501	0	0	0	0	0	0
RP11-369J21.11	0	0	0	0	0	0
AC011288.2	0	0	0	0	0	0
MBNL1-AS1	55.672965	41.6450929	8.023474646	34.1938073	74.56286704	53.45759169
AC018742.1	0.97671868	0	0	0	0	0.906060876
MTND5P2	0	0	0	0	0	0
METTL21AP1	0.97671868	0	9.026408976	1.00570022	0	0
RP11-44N12.2	0	0	0	0	0	0
PIGCP2	0	0	0	0	0	0
BSNDP4	0	0	0	0	0	0
AC073115.7	0	0	0	0	0	0
RP11-302K17.4	0	0	0	0	0	0
LINC01264	0	0	2.005868661	0	1.007606311	0.906060876
RP4-713B5.2	0	0	0	0	0	0
KRT8P21	0	0	0	0	0	0
PRAC2	6.83703079	6.09440383	1.002934331	3.01710065	0	1.812121752
RPL4P4	87.6605019	79.8570049	245.6487056	149.396767	145.2968301	149.7628022
RP3-380B4.1	0	0	0	0	0	0
CYP2C56P	0	0	0	0	0	0
AC027119.1	0	0	0	0	0	0
LINC00280	0	0	0	0	0	0
NAMPTP1	306.240376	334.735131	26.93881612	42.8026012	125.5275943	64.74711021

RP11-330A16.1	0	0	0	0	0	0
AC007879.7	10.7439055	14.2202756	0	0	0	0
RPSAP22	0	0	0	0	0	0
RP11-107I14.4	0	0	0	0	0	0
RP11-435P24.2	0	0	0	0	0	0
RP1-60O19.2	0	0	0	0	0	0
RP11-462L8.1	68.3703079	69.0699101	0	0	0	1.812121752
RP11-494K3.2	0	0	0	0	0	0
PABPC1P9	0	0	0	0	0	0
RP11-345K20.2	10.7439055	2.03146794	6.017605984	2.01140043	1.007606311	2.718182629
RP5-1142J19.1	0	0	0	0	0	0
BUD31P2	0	0	0	0	0	0
RP11-552E4.5	0	0	0	0	0	0
AL672296.1	0	0	0	0	0	0
RP11-536K7.5	0	0	0	0	0	0
PRR20C	0	0	0	0	0	0
MAST4-AS1	5.8603121	9.14160575	2.005868661	4.02280086	3.022818934	1.812121752
UBE2V1P9	0	0	0	0	0	0
RP11-317F20.2	0	0	0	0	0	0
PKP4P1	0	0	0	0	0	0
LINC01150	0	0	0	0	0	0
RP11-184A2.3	0	0	0	0	0	0
LL22NC01-116C6.1	0	0	0	0	0	0
AL121578.7	0	0	0	0	0	0
ZNF492	0.97671868	0	0	0	44.15330856	0
RP11-383F6.1	9.76718684	2.03146794	3.008802992	7.03990151	5.038031556	10.87273051
AC007327.5	0	0	0	0	0	0
AC114755.5	0	0	0	0	0	0
SNORD56	0	0	0	0	0	0
RP4-654H19.2	0	0	0	0	0	0
ISPD-AS1	0	0	0	0	0	0
AC009237.8	295.945761	384.963175	122.3579883	79.450317	285.1525861	110.5394269
MTCO2P1	0	0	0	0	0	0

SOS1-IT1	20.5110924	7.11013781	13.0381463	15.0855032	62.4715913	27.18182629
RP11-25C15.1	0	0	0	0	0	0
RP11-305L7.6	2.93015605	1.01573397	0	0	0	0
AC011242.5	0.97671868	0	1.002934331	0	1.007606311	1.812121752
KARSP1	0	0	1.002934331	0	0	0
RP11-374M1.3	0	0	0	0	0	0
RP1-140J1.1	0	0	0	0	0	0
RP1-130G2.1	0	0	0	0	0	0
MTND2P20	0	0	0	0	0	0
RP1-274L7.1	0	0	0	0	0	0
XX-CR54.1	0	0	0	0	0	0
EIF2S2P2	0.97671868	2.03146794	2.005868661	0	0	0
SKP1P3	0	0	0	0	0	0
MARK2P12	0	0	0	0	0	0
USP9YP36	0	0	0	0	0	0
RP11-449O16.2	0	0	0	0	0	0
RP1-43O17.1	0	0	0	0	0	0
EEF1DP3	0	0	0	1.00570022	0	0.906060876
RPL23AP19	0	0	0	0	0	0
RP11-556N4.1	0	0	0	0	1.007606311	0
MIR194-2HG	0	4.06293589	1.002934331	9.05130194	2.015212623	1.812121752
RP3-495K2.2	0	0	0	0	0	0
RP11-252I14.1	2.93015605	0	0	0	2.015212623	3.624243505
RP11-557H15.5	0	0	0	0	0	0
LINC01054	0	0	0	0	0	0
OR2AQ1P	0	0	0	0	0	0
AC007322.1	0	0	0	0	0	0
AC013460.1	0	0	0	0	0	0
RP11-314N13.3	2.93015605	0	5.014671654	2.01140043	3.022818934	4.530304381
RP11-159G9.5	148.46124	105.636333	82.24061512	173.986137	158.1941909	139.5333749
AC013268.4	0	0	0	0	0	0
RP11-501H19.2	0	0	0	0	0	0
AC019349.5	0	0	0	0	0	0

RP5-1189B24.1	0	0	0	0	0	0
FTLP16	0	0	0	0	0	0
AC010240.3	0	0	0	0	0	0
RP11-295K2.3	0	0	5.014671654	3.01710065	0	4.530304381
U91324.1	0	0	0	0	0	0
RP11-365O16.5	0	0	0	0	0	0
LINC01159	0	0	0	0	0	0
AC011998.2	0	0	0	0	0	0
BPY2DP	0	0	0	0	0	0
RP11-567B20.2	0	0	0	0	0	0
COTL1P1	0	0	0	0	0	0
AC096649.2	0.97671868	0	0	0	0	0
RP11-142M10.2	0	1.01573397	0	0	0	0
RPL7P10	0	0	0	0	0	0
RP11-28P17.3	0	0	0	0	0	0
CXCR2P1	0	0	1.002934331	0	0	0.906060876
RP5-827L5.2	0	0	0	0	0	0
RPL31P20	0	0	0	0	0	0
RP4-738P11.4	0	0	0	0	0	0
DYNLT3P2	0	0	2.005868661	0	0	0
MRPS18AP1	0	0	0	0	0	0
RP11-266I3.5	0	0	0	0	0	0
RPS20P1	0	0	0	0	0	0
RP11-419M24.1	0	0	0	0	0	0
RP11-339A7.1	0	0	0	0	0	0
RP5-971N18.3	0	0	0	0	0	0.906060876
TRBV10-2	0	0	0	0	0	0
CTA-796E4.4	0	0	0	0	0	0
RP4-644L1.2	18.557655	15.2360096	0	0	0	1.812121752
AC018866.1	0	0	0	0	0	0
RP11-298H24.1	0	0	0	0	0	0
C4B-AS1	0	0.50786699	0	0	0	0
AC016751.2	0	0	0	0	0	0

UBE2Q1-AS1	0	0	0	0	0	0
AC013444.1	0	0	0	0	0	0.906060876
AC118754.4	1.95343737	1.01573397	0	0	0	0.906060876
SLC25A38P1	0	0	0	1.00570022	0	0.915121485
SNRPFP2	0	0	0	0	0	0
LINC00388	0	0	0	0	0	0
RP11-385E5.5	0	0	0	0	0	0
LINC00399	0	0	0	0	0	0
RP11-522L3.11	0	0	4.011737323	1.00570022	0	0
RPS21P1	0	0	5.014671654	0	1.007606311	0.906060876
AC140481.8	0	0	0	0	0	0
KRT18P26	0	0	0	0	0	1.812121752
ATP8A2P2	0	0	0	0	0	0.906060876
AC011306.1	0	0	0	0	0	0
RPS15P5	0	0	0	0	0	0.906060876
XIST	0	0	17049.88362	7799.20517	230.7418453	11427.23977
RP11-456P18.2	0	4.06293589	17.04988362	4.02280086	3.022818934	8.154547886
ZNF688	120.136398	126.966747	738.1596674	664.767842	352.662209	384.1698115
RPL35AP21	0	0	0	0	0	0
CCNB1IP1P1	0	0	0	0	0	0
DDX50P1	9.82578996	3.05735926	14.16143275	15.1458452	16.23253767	16.38158064
RP11-78H18.2	0	2.03146794	0	0	0	0
RP1-308E4.1	0.97671868	3.04720192	2.005868661	1.00570022	2.015212623	0
RP11-567E21.3	0	0	0	0	0	0
RP5-1174J21.2	0	0	0	0	0	0
RPL12P34	0	0	0	0	0	0
RP11-552J9.14	0	0	0	0	0	0
AC093899.3	0	0	0	1.00570022	0	0
PDE4DIPP1	0	0	0	0	0	0
DUTP4	0	0	0	0	0	0
RP11-341A22.1	0	0	0	0	0	0
AC108039.3	0	0	0	0	0	0
RP11-384C4.2	0	0	2.005868661	3.01710065	1.007606311	2.718182629

PET100	423.895909	385.978909	519.5199833	418.37129	198.4984433	414.0698204
RP11-48L13.1	0	0	0	0	0	0
KHSRPP1	0.97671868	2.03146794	0	0	0	0.906060876
XXbac-BPG248L24.10	0	0	0	0	0	0
AC018462.2	0	0	0	0	0	0
ISCA1P2	0	0	0	0	0	0
RP5-837O21.4	0	0	0	0	0	0
RP11-141A19.1	0	0	0	0	0	0
EMX2OS	0	0	0	0	0	0
RP13-766D20.2	2.93015605	4.06293589	27.07922693	15.0855032	33.25100827	19.0272784
PYHIN5P	0	0	0	0	0	0
ARSD-AS1	0	0	0	0	4.030425245	0.906060876
RP11-398K22.12	30.2880464	12.2497517	62.01142967	56.7214922	40.30425245	81.12869085
RP5-1049G16.4	0	0	0	0	2.015212623	0
RP11-524G24.2	0	0	0	0	0	0
CTC-546K23.1	0	1.01573397	0	0	0	0
RP11-159H20.3	0	0	0	0	0	0
AC016831.5	0	0	0	0	0	0
PGA3	0	0	0	1.00570022	0	1.812121752
RP11-505P4.7	0	1.01573397	0	0	0	1.812121752
RP11-157F20.1	0	0	0	0	0	0
RPL31P54	0	0	0	0	0	0
STEAP3-AS1	4.88359342	3.04720192	0	0	2.015212623	2.718182629
RP11-363N22.2	0	0	0	0	1.007606311	0
RP11-507K13.6	0	0	0	0	0	0
RPSAP20	0	0	0	0	2.015212623	0
OGFR-AS1	1.95343737	2.03146794	0	1.00570022	0	0
EIF1P1	0	0	0	0	0	0
CASC20	0	0	0	0	0	0
PAICSP5	0	0	0	0	0	0
RP11-479G22.7	0	0	0	0	0	0
IMMTP1	0	0	0	0	1.007606311	0
RP11-321E8.4	0	0	0	0	0	0

RP13-30A9.2	0	0	0	0	0	0
RP13-77O11.12	0	0	0	0	0	0
RP5-1132H15.3	0	0	0	0	0	0
HNRNPA1P6	0	0	0	0	0	0
ACTP1	0	0	0	0	0	0
LINC01315	2.93015605	9.14160575	6.017605984	13.0741028	17.12930729	3.624243505
RP4-730D4.1	0	0	0	0	0	0
AC004549.6	0	1.01573397	0	0	1.007606311	0
RP11-668G10.2	1.95343737	3.09798862	0	2.02145743	0	0.906060876
RP11-456H18.2	4.0826841	1.01573397	3.008802992	7.03990151	0	1.812121752
SEPT7P7	0.97671868	2.03146794	0	1.00570022	1.007606311	0
AC084290.2	0	0	0	0	0	0
HSPD1P21	0	0	0	0	0	0
RP11-399E6.4	0.97671868	1.01573397	0	0	0	0
RP11-206L10.4	0	0	0	0	0	0
SNRPGP11	0	0	0	0	0	0
DEFB108P1	0	0	0	0	0	0
TRIM60P16	0	0	0	0	0	0
HNRNPA1P18	0	0	0	0	0	0
RP4-630J13.1	0	0	0	0	0	0
AC128709.4	1.95343737	0	0	0	0	0
RP11-378I13.1	0	0	0	0	0	0
RP11-404O13.4	0	0	0	0	3.022818934	0
AC016999.2	1.95343737	2.03146794	0	0	2.015212623	0
RPL7P46	0	2.03146794	6.017605984	0	0	0
DOCK9-AS1	0	0	0	0	0	0
TCEB1P3	0	0	0	0	0	0
RPS4XP5	0.97671868	1.01573397	0	0	1.007606311	0
KIF25-AS1	0	0	0	0	0	0
RP11-240M16.1	0	0	0	0	0	0
RP11-102N11.1	0	0	0	0	0	0
FAM90A26	0	0	0	0	0	0
AP001043.1	0	0	0	0	0	0

RP11-424E7.3	0	0	0	0	0	0
RHEBP1	4.600345	23.8088043	6.408750373	11.1129874	18.77170558	3.098728197
LINC00400	0	0	0	0	0	0
RP11-504P24.5	5.8603121	1.01573397	0	0	0	0
RP1-151F17.1	6.83703079	17.2674775	14.04108063	20.1140043	45.34228401	23.55758278
YWHAZP3	9.99183214	23.7478603	10.73139734	8.24674177	34.36945128	11.72442774
MTCYBP9	0	0	0	0	0	0
PRPS1L1	0	0	0	0	0	0
MYO16-AS2	0	0	0	0	0	0
RP11-111F16.2	0	0	1.002934331	2.01140043	2.015212623	1.812121752
TSPY22P	0	0	0	0	0	0
AC012499.1	0	0	0	0	0	0
RP11-93N20.1	0	0	0	0	0	0
EIF4EP2	23.2263703	23.9306924	16.47821105	29.0043942	34.42990766	21.5098852
B3GNT2P1	0	0	0	0	0	0
RP13-766D20.1	0	0	0	0	0	0
B3GALNT1P1	0	0	0	0	0	0
AC005094.2	0	0	0	0	0	0
TFAP2A-AS1	44.9290595	18.2832115	14.04108063	30.1710065	5.038031556	33.52425242
FLJ31356	0.97671868	2.03146794	3.008802992	8.04560172	19.14451991	3.624243505
RP11-284F21.7	1.95343737	2.03146794	65.1907315	45.2565097	49.37270925	43.49092206
MTND2P2	0	0	0	0	0	0
RP1-5O6.4	0	0	0	0	0	0
ZRANB2-AS2	0.97671868	9.14160575	8.023474646	5.02850108	1.007606311	5.436365257
RP5-998H6.2	0	0	0	0	0	0
RP11-278H7.4	0	0	0	0	0	0
RP11-71G12.1	0	0	0	0	0	0
AP000221.1	0	1.01573397	0	0	0	0
RP11-135A1.3	0	0	0	0	0	0
RP11-118H4.1	0	0	0	0	0	0
RP11-366F6.2	0	0	1.002934331	0	0	0
RP3-393P12.1	0	0	0	0	0	0
RP11-96C23.10	0	0	0.501467165	0	0	0.453030438

AC007128.1	0	0	0	0	0	0
RP5-1119A7.10	0	0	0	0	0	0
IQCF3	0	0	0	0	0	0
LIPT1P1	0	0	0	0	0	0
RP5-1031J8.1	0	0	0	0	0	0
AC073264.10	0	0	0	0	0	0
PRAMEF36P	0	0	0	0	0	0
U82670.9	0	0	0	0	0	0
TOB1-AS1	1.95343737	5.07866986	76.22300913	106.604223	51.38792188	69.76668747
LINC01435	0	1.01573397	0	0	0	0
GTF3AP6	0	0	0	0	0	0
RP11-15I11.2	0	0	0	0	0	0.906060876
RP11-40H20.5	0	0	0	0	0	0
AP001042.1	0	0	0	0	0	0
HBBP1	0	0	0	0	0	0
MIR181A1HG	8.79046815	6.09440383	21.06162094	11.0627024	8.06085049	11.77879139
RP11-574K11.8	0	0	0	0	0	0
AKR1B1P1	3.90687474	6.09440383	0	0	1.007606311	1.812121752
HMGB3P9	0	0	0	1.00570022	1.007606311	0
RPL5P4	4.41476845	4.06293589	17.05991297	7.03990151	1.007606311	6.867941441
AC093585.6	0	0	0	0	3.022818934	0
RP5-1042K10.10	0	0	0	0	0	0
RP5-905H7.11	0	0	0	0	0	0
RP11-70J12.1	0	0	0	0	0	0
ALMS1-IT1	53.7195276	36.566423	13.0381463	6.03420129	33.25100827	5.436365257
RP11-217F16.1	0	0	0	0	0	0
SNAP47-AS1	0	0	1.002934331	0	0	0
ANKRD36BP2	0.97671868	0	1.002934331	0	1.007606311	0
MTCO2P3	0	0	0	0	0	0
RP4-568F9.6	0	0	0	0	0	0
CTSLP4	0	0	0	0	0	0
RP11-217B7.3	0.97671868	0	0	0	4.030425245	0
LINC00709	0	0	0	0	0	0

RP11-80B9.4	0	0	0	0	0	0
RP11-481H12.1	0	0	0	1.00570022	0	0
YWHAQP9	0	0	0	0	0	0
NHS-AS1	0	0	0	0	0	0
RP5-857K21.4	10.5680962	12.8591921	25.28397448	56.9427462	4.594684779	24.68109827
FNTAP2	0	0	0	0	0	0
RP11-10N16.2	0	0	0	0	0	0
RP11-95P13.1	0	0	0	0	0	0
AC007967.4	0	0	0	0	0	0
RP11-382D8.5	0	0	2.005868661	2.01140043	0	9.060608762
RP11-550H2.2	0	0	0	0	0	0.906060876
CDY11P	0	0	0	0	0	0
RP11-505C13.1	0	0	0	0	0	0
POTEB2	0	0	0	0	0	0
RP5-1121A15.3	0	0	0	0	0	0
RP11-174G17	0	0	0	0	0	0
UBBP1	0	0	0	0	0	0
RP11-314A15.2	0	0	0	0	0	0
LINC00364	0	0	0	0	0	0
AK3P3	8.79046815	2.03146794	10.02934331	6.03420129	6.045637868	7.248487009
TMSB4XP6	20.0129658	11.3965352	1.022993017	4.11331388	17.51219769	0.924182094
AC110754.3	0	0	0	0	0	0
FAM90A15P	0	0	0	0	0	0
BIRC6-AS1	0	0	0	0	0	0
AC017104.4	0	0	0	0	0	1.812121752
CTA-929C8.8	0	0	0	0	0	0
MTND3P2	0	0	0	0	0	0
RP11-76N22.1	0	0	0	0	0	0
RP11-402G3.5	0	0	0	0	0	0
DDX6P1	0	0	0	0	0	0
RP11-172E9.2	0	0	0	0	0	0.906060876
TRPM2-AS	0	0	0	0	0	0
ANKRD66	0	0	0	0	0	0.906060876

RP11-384C4.6	0	1.01573397	0	0	0	1.812121752
RP11-244N20.7	0	0	0	0	0	0
AC010974.3	0	0	0	0	2.015212623	0
FAM197Y3	0	0	0	0	0	0
HSPD1P6	0.98648587	1.02589131	2.025927348	0	1.017682374	2.736303846
CDC42-IT1	0	1.01573397	1.002934331	0	0	0
LRRC37A15P	2.93015605	4.06293589	18.05281795	27.1539058	11.08366942	23.55758278
RPL4P6	1.95343737	3.04720192	8.023474646	9.05130194	13.09888205	9.060608762
AC009947.2	0	0	0	0	0	0
RP11-195F19.9	6.83703079	3.04720192	46.13497921	33.1881071	18.1369136	18.12121752
AC016708.2	0	0	1.494372153	0	1.098290879	0
MTAPP2	1.95343737	1.01573397	0	0	0	0
STK24P1	0	0	0	0	0	0
AC139143.2	0	0	0	0	0	0
HSPE1P28	0	0	0	0	0	0
PRRT3-AS1	36.1385913	39.6136249	25.07335827	12.0684026	1.007606311	32.61819154
AC009237.13	0	0	0	0	0	0
RP4-613B23.1	0.97671868	0	4.011737323	4.02280086	0	2.718182629
VN1R96P	0	0	0	0	0	0
AC104389.31	0	0	0	0	0	0
KRT16P5	0	0	0	0	0	0
AC108025.2	0	0	0	0	0	0
TMEM254-AS1	12.6973429	16.2517436	252.7394513	230.707629	94.71499326	182.5078423
RP11-206L10.8	3.4868857	3.04720192	1.053081047	7.77406267	7.143928747	4.90178934
RP11-34C15.2	0	0	0	0	0	0
ME2P1	0	0	0	0	0	0
TCERG1L-AS1	0	0	0	0	2.015212623	0.906060876
TRBV5-4	0	0	0	0	0	0
RP11-344N17.8	0	0	0	0	0	0
TUBB3P2	0	0	0	0	0	0
RP11-407B7.1	0	1.01573397	17.04988362	5.02850108	2.015212623	8.154547886
AC018712.2	0	0	0	0	0	0
RP11-431N15.2	0	0	0	0	2.015212623	0.906060876

SNRPCP15	0	0	0	0	0	0
CTA-126B4.7	1.95343737	0	0	0	0	0
RP11-275N1.1	0	0	0	0	0	0
AC115283.1	0	0	0	0	0	0
RP11-56H7.2	0	0	0	0	0	0
AC091177.1	0	1.01573397	0	0	1.007606311	0
RP11-141A19.2	0	0	0	0	0	0
TPRG1-AS2	0	0	0	0	0	0
RP11-163G10.2	0	0	2.005868661	2.01140043	0	0
AC092569.2	0	0	1.002934331	0	1.007606311	2.718182629
AC123900.2	0	0	0	0	0	0
RP1-90G24.8	0	0	0	0	0	0
RP11-385N23.1	0	0	0	0	0	0
ECEL1P3	0	0	0	0	0	0
DLEC1P1	0	0	0	0	0	0
ACBD6	1148.62117	1302.17095	1847.405037	1667.45096	1523.500743	1464.194376
EEF1A1P39	0	0	0	0	0	0
FGF12-AS2	0	0	0	0	1.007606311	0
MTND2P39	0	0	0	0	0	0
RP11-282I1.1	0	0	0	0	0	0
[truncated: 349,421 more chars]
